# Integrated analysis of the microbiome and transcriptome in stomach adenocarcinoma

**DOI:** 10.1515/biol-2022-0528

**Published:** 2023-07-15

**Authors:** Daxiang Zhou, Shu Xiong, Juan Xiong, Xuesong Deng, Quanzhou Long, Yanjie Li

**Affiliations:** Chongqing Engineering Laboratory of Green Planting and Deep Processing of Famous-region Drug in the Three Gorges Reservoir Region, College of Biology and Food Engineering, Chongqing Three Gorges University, Chongqing 404120, China; Department of Basic Medicine, Chongqing Three Gorges Medical College, No. 666 Tianxing Road, Baianba, Wanzhou District, Chongqing 404120, China; Department of Neonatology, Jiulongpo People’s Hospital of Chongqing, Chongqing, 400050, China

**Keywords:** stomach adenocarcinoma, microbiota, subtype, prognostic model, immune infiltration

## Abstract

We aimed to characterize the stomach adenocarcinoma (STAD) microbiota and its clinical value using an integrated analysis of the microbiome and transcriptome. Microbiome and transcriptome data were downloaded from the Cancer Microbiome Atlas and the Cancer Genome Atlas databases. We identified nine differentially abundant microbial genera, including *Helicobacter*, *Mycobacterium*, and *Streptococcus*, which clustered patients into three subtypes with different survival rates. In total, 74 prognostic genes were screened from 925 feature genes of the subtypes, among which five genes were identified for prognostic model construction, including *NTN5*, *MPV17L*, *MPLKIP*, *SIGLEC5*, and *SPAG16*. The prognostic model could stratify patients into different risk groups. The high-risk group was associated with poor overall survival. A nomogram established using the prognostic risk score could accurately predict the 1, 3, and 5 year overall survival probabilities. The high-risk group had a higher proportion of histological grade 3 and recurrence samples. Immune infiltration analysis showed that samples in the high-risk group had a higher abundance of infiltrating neutrophils. The Notch signaling pathway activity showed a significant difference between the high- and low-risk groups. In conclusion, a prognostic model based on five feature genes of microbial subtypes could predict the overall survival for patients with STAD.

## 1 Introduction

Gastric cancer (GC) is a malignant tumor originating from the epithelium of the stomach. It is a disease with high molecular and phenotypic heterogeneity, with adenocarcinoma being the most common type [1]. The occurrence of GC is due to complicated influencing factors, and previous studies have shown that it is related to *Helicobacter pylori* infection, diet, smoking, drinking, and genetic factors [2]. In China, the incidence of GC ranks second only to that of lung cancer, and the death rate ranks third. There are approximately 1.2 million new cases of GC worldwide each year, and approximately 40% of these cases develop in China [3]. Due to the atypical symptoms of early GC, most patients have advanced gastric cancer at the time of diagnosis, and the overall 5 year survival rate is less than 50% [4]. Therefore, in-depth studies on the pathogenesis of GC and the search for scientific and reasonable intervention methods are urgent problems that need to be solved.

Gut microbiota affects the morphological, immunological, and nutritional functions of the digestive tract and may be implicated in the development of many diseases [5]. Gut microbiota plays a key role in gastrointestinal cancers and may be used as a new tumor biomarker [6,7]. Recent studies have reported that the intestinal bacterial community plays an important role in the occurrence and development of GC [8]. *H. pylori* can trigger the development of GC, and chronic infection causes decreased acid secretion, resulting in the development of a different gastric bacterial community [9,10]. For example, Guo et al. indicated that after *H. pylori* eradication, the Shannon and richness indices of the gastrointestinal microbial community were significantly increased in *H. pylori*-positive GC patients, involving obvious changes in 18 gastric microbial genera (e.g., increase in probiotic *Bifidobacterium*) [11]. Homeostasis of the bacterial community plays an important role in human health, and abnormalities in this community is usually related to the occurrence and development of GC [12]. Based on 16S rRNA sequencing of a cohort of 276 GC patients, Liu et al. found that significant changes were observed in the bacterial community, and the abundance of pathogenic bacteria such as *Propionibacterium* acnes and *Prevotella melaninogenica* was elevated, while *Bacteroides uniformis*, *H. pylori*, and *Prevotella copri* were reduced [13]. Dang et al. suggested that gastrointestinal microbiota could be used as a promising diagnostic biomarker for GC patients [14].

Recent advances in transcriptome sequencing have provided an unprecedented global view of transcriptomes. Transcriptome sequencing is widely used to identify the key genes and pathways involved in gastric adenocarcinoma [15]. A previous transcriptome analysis revealed 148 differentially expressed genes (DEGs) in tumor samples, and the analysis suggested that *SALL4* might be a key prognostic gene in gastric adenocarcinoma [16]. Moreover, several studies have performed integrated analyses of the microbiome and transcriptome to elucidate the key mechanisms involved in disease development. For instance, integrative bioinformatics analysis of the microbiome and transcriptome revealed a microbiome-related gene map for predicting the risk of colon adenocarcinoma [17]. Huang et al. performed an integrated analysis of the microbiome and host transcriptome and demonstrated that the gut microbiota might affect the clinical outcomes of HBV-related hepatocellular carcinoma by modulating the microbe-associated transcripts of host tumors [18]. Despite these findings, few studies have focused on integrated analysis of microbiome and transcriptome data to explore the effect of microbiota in GC. Therefore, based on data from the Cancer Microbiome Atlas (TCMA) and the Cancer Genome Atlas (TCGA) databases, we performed an integrated analysis to explore the differential microbiota and their associations with prognosis, immune infiltration, and clinical characteristics. Only stomach adenocarcinoma (STAD) samples, the most common type of GC, were included in this analysis, which may have eliminated inter-individual differences. This will provide theoretical foundations and potential microbiota and molecular targets for investigating GC pathogenesis and search for scientific and reasonable intervention methods.

## 2 Methods

### 2.1 Data acquisition and preprocessing

The normalized log (FPKM + 1,2) expression data of 407 STAD samples (Table S1) were acquired from the TCGA database [19]. Data were generated on an Illumina HiSeq 2000 RNA sequencing platform. The normalized microbiome data of 166 STAD samples (Table S2) were acquired from the TCMA database [20], including microbial abundance data at the phylum, class, order, family, and genus levels. Only samples from the TCGA and TCMA databases were retained, and the corresponding clinical data were downloaded. Finally, 91 STAD and 32 histologically normal tissue samples with relevant clinical data were included in this study.

Additionally, the microarray data GSE62254 [21] that had been preprocessed, normalized, and log2 transformed were downloaded from the Gene Expression Omnibus database and used as a validation dataset, which included the expression and clinical data of 300 tumor tissue samples. The data were generated on the GP570 Affymetrix Human Genome U133 Plus 2.0 Array platform. The platform annotation files were also downloaded. For the same gene mapped by different probes, the mean value of different probes was considered the final expression value of this gene.

**Figure A1:**
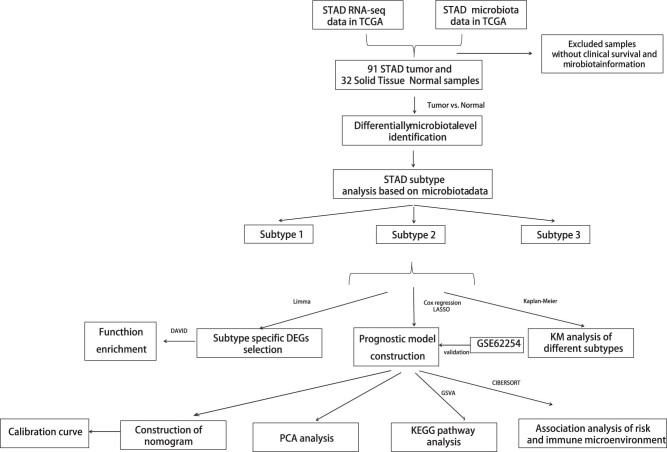
The workflow of this study.

These datasets were analyzed as per the workflow in Figure A1.

### 2.2 Identification of microbial subtypes

The differences in the microbial abundance between 91 STAD and 32 histologically normal samples were compared using unpaired t-tests. Based on the abundance of differential microbiota, microbial subtypes in the STAD samples were identified using ConsensusClusterPlus (version 1.54.0) in R 3.6.1 [22]. Survival differences among different microbial subtypes were assessed by Kaplan–Meier (KM) survival analysis using the survival package (version 2.41-1). Differences in clinical data among microbial subtypes were also compared using Fisher’s exact test.

### 2.3 Screening of feature genes for microbial subtypes

Using the Limma package (version 3.34.7) [23], the DEGs in subtype 1 vs subtypes 2 and 3, subtype 2 vs subtypes 1 and 3, and subtype 3 vs subtypes 1 and 2 groups were screened with a false discovery rate < 0.05, and |log2 fold change| > 0.263 as the cut-off value. The DEGs in the three groups were merged into a union set, and this set was used in the following analysis.

Functional enrichment analysis of the DEGs in the three groups was conducted using the Database for Annotation, Visualization, and Integrated Discovery (DAVID, version 6.8) [24]. *P* < 0.05 was used to select the significantly enriched Gene Ontology (GO) biological process and Kyoto Encyclopedia of Genes and Genomes (KEGG) pathways.

### 2.4 Construction of a prognostic model

The prognostic value of the DEGs was assessed by univariate Cox regression analysis using the Survival package (version2.41-1), in which genes with *P* < 0.05 were analyzed by multivariable Cox regression. Genes with *P* < 0.05 in the multivariable Cox regression analysis were defined as independent prognostic genes. In addition, optimal genes among the independent prognostic genes were further screened by LASSO regression analysis using the lars package (version 1.2) in R 3.6.1 [25]. These optimal genes were used to construct a prognostic model based on the following formula:

\text{Prognostic}\hspace{.25em}\text{risk}\hspace{.25em}\text{score}=\sum {\text{Coef}}_{\text{genes}}\times {\text{Exp}}_{\text{genes}},]

where Coef_genes_ refers to the LASSO prognostic coefficient, and Exp_genes_ refers to the expression level of each gene. KM survival curves were used to evaluate the differences in prognostic value of genes between different gene expression groups using the survival package (version 2.41-1). In addition, the prognostic risk score was calculated for samples in both the TCGA dataset and GSE62254 validation dataset. The tumor samples in each dataset were grouped into two risk groups based on the median value of the prognostic risk score. The survival differences of the different risk groups were evaluated using KM-survival analysis. The 1, 3, and 5 year prediction accuracy of the prognostic model was analyzed using the survival ROC package (version 1.0.3) [26] in R. Moreover, the heatmap of gene expression with the changes in risk score and clinical data distribution was visualized.

### 2.5 Analysis of the independent prognostic factors and creation of a nomogram

Based on the clinical data in the TCGA dataset, univariate and multivariate Cox regression analyses were utilized to determine the independent prognostic factors by analyzing the prognostic risk score and the various clinical variables, including age, sex, neoplasm histologic grade, pathologic stage, recurrence, and pathologic M, N, and T. Clinical variables (*P* < 0.05) in the univariate Cox regression analysis were included in the multivariate Cox regression analysis. Based on the independent prognostic factors elucidated by the multivariate Cox analysis, a nomogram was established to predict the 1, 3, and 5 year overall survival probabilities of patients with GC. Calibration of the nomogram was evaluated graphically using calibration curves.

### 2.6 Associations of risk groups with immune infiltration

Based on the expression data of TCGA-STAD samples, the abundances of the different infiltrating immune cells were evaluated using the CIBERSORT algorithm [27]. The differences in the abundance of immune cell infiltration among different risk groups were compared using an unpaired *t*-test.

### 2.7 Gene set variation analysis (GSVA)

The KEGG pathways and the involved genes were obtained from the “download window” of the gene set enrichment analysis database [28]. Based on the genome-wide expression data of the TCGA-STAD samples, using “c2.cp.kegg.v7.4. entrez.gmt” as the background gene set, GSVA (version 1.36.3) [29] in R3.6.1 language was used to quantitatively analyze each KEGG pathway based on gene expression level. Differential activities in these KEGG pathways between two different risk groups were analyzed using an unpaired *t*-test, and *P* < 0.05 was regarded as the cut-off value.

## 3 Results

### 3.1 Identification of microbial subtypes

**Figure 1:**
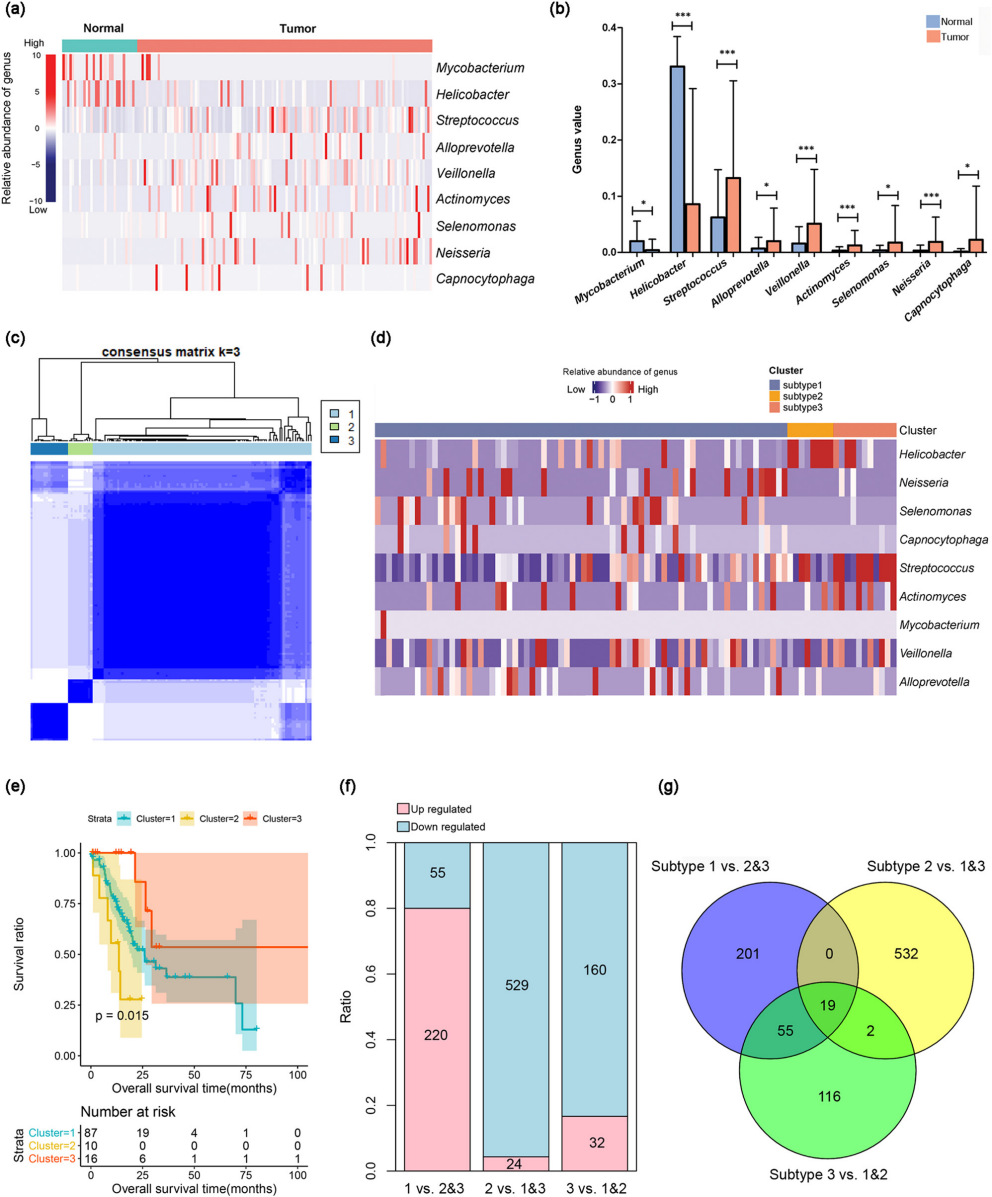
Identification of differential microbials and microbial subtypes. (a) Heatmaps showing the abundance of nine differential microbial genera between the STAD and histologically normal samples. The blue-to-red colors of the lateral bar on the left indicate the low-to-high relative abundance of differential microbial genera; (b) Bar graph of relative abundance of nine differential microbial genera between STAD (orange) and histologically normal samples (blue); (c) clustering heatmap reveals three microbial subtypes: subtype 1, subtype 2, and subtype 3; (d) heatmap showing the abundance of nine differential microbial genera among the three microbial subtypes; (e) KM survival curve showing the survival differences among three subtypes; (f) number of DEGs in subtype 1 vs subtypes 2 and 3, subtype 2 vs subtypes 1 and 3, and subtype 3 vs subtypes 1 and 2 groups; (g) Venn diagram for the DEGs of the three groups.

The differences in microbial distribution between STAD and histologically normal samples were compared, and two differential microbial phyla (*Firmicutes* and *Proteobacteria*), five differential microbial classes, eight differential microbial orders, eight differential microbial families, and nine differential microbial genera (e.g., *Streptococcus* and *Helicobacter*) were obtained (Table S3). There was more microbiota diversity at the genus level, and hence, the data at the genus level were used in the following analysis. Among the nine differential microbial genera, *Mycobacterium* and *Helicobacter* showed a higher abundance in the histologically normal samples, while the remaining seven microbial genera (e.g., *Streptococcus*, *Alloprevotella*, and *Veillonella*) showed a higher abundance in the STAD samples (Figure 1a and b). *Helicobacter* and *Streptococcus* were more abundant in both the tumor and normal samples than other microbial genera (Figure 1b).

Based on the abundance of nine different microbial genera, clustering analysis revealed that the STAD samples were categorized into three microbial subtypes: subtypes 1, 2, and 3 (Figure 1c). The majority of the STAD samples (*n* = 72) were clustered into subtype 1, while 8 and 11 samples were clustered into subtypes 2 and 3, respectively. As shown in Figure 1d, subtype 1 mainly contained a higher abundance of *Streptococcus*, subtype 2 mainly contained a higher abundance of *Helicobacter*, and subtype 3 mainly contained a higher abundance of *Neisseria*, *Selenomonas*, and *Capnocytophaga*. Survival analysis revealed that subtype 3 had a favorable overall survival, while subtype 2 had a worse overall survival than the other subtypes (Figure 1e). No significant differences were found in the clinical factors among the three subtypes (Table S4).

### 3.2 Feature genes of the microbial subtypes and their involved functions

A total of 275, 553, and 192 DEGs were obtained from subtype 1 vs subtypes 2 and 3, subtype 2 vs subtypes 1 and 3, and subtype 3 vs subtypes 1 and 2 groups, respectively (Table S5). In subtype 1 vs subtypes 2 and 3 group, most of the DEGs were upregulated, whereas most of the DEGs were downregulated in subtype 2 vs subtypes 1 and 3 and subtype 3 vs subtypes 1 and 2 groups (Figure 1f). The DEGs of the three groups were merged, and 925 overlapping DEGs were obtained (Figure 1g).

**Figure 2:**
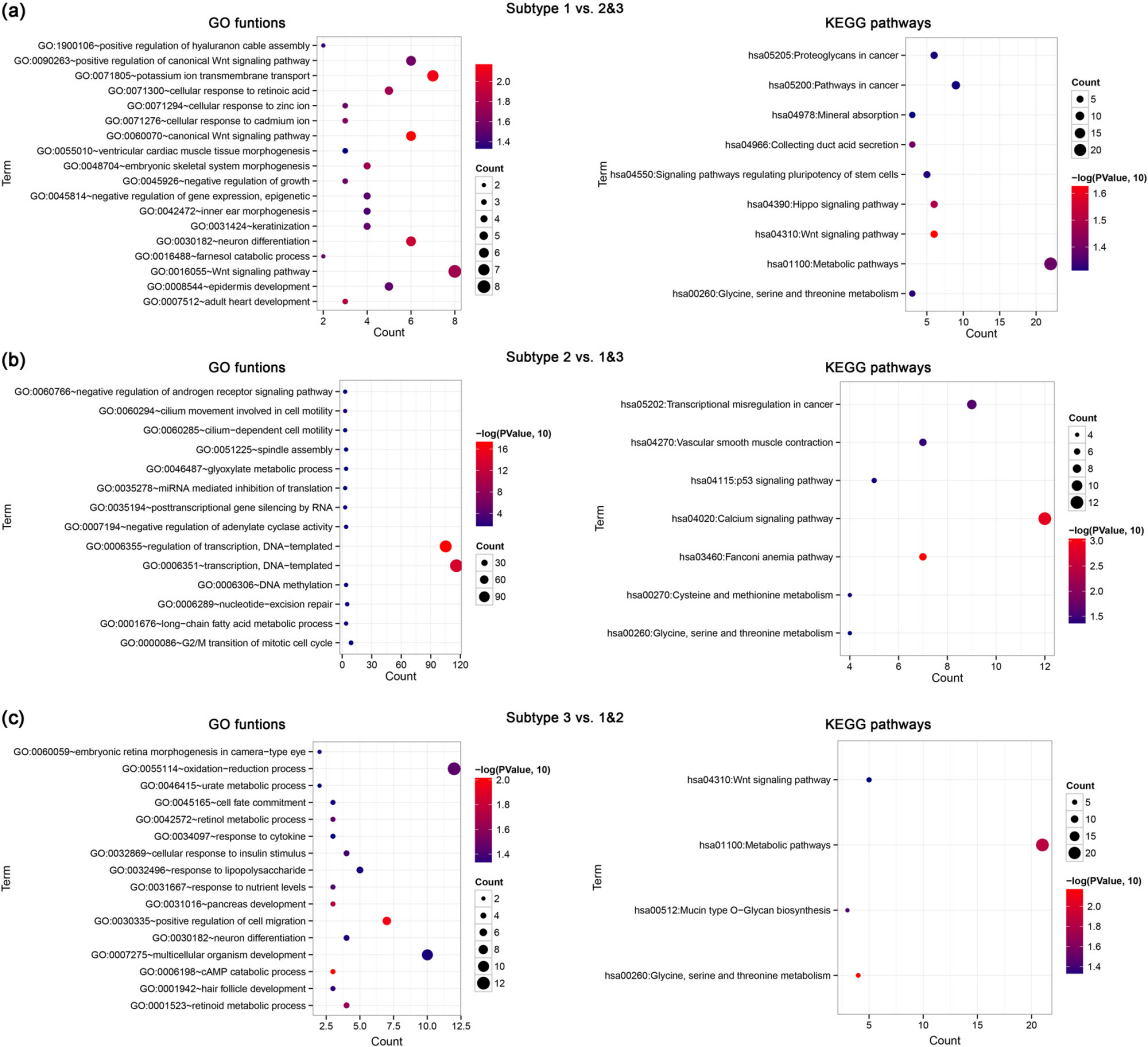
Results for functional enrichment analysis. The bubble diagram shows the significantly enriched biological processes (left) and KEGG pathways (right) for the DEGs in subtype 1 vs subtypes 2 and 3 (a), subtype 2 vs subtypes 1 and 3 (b), and subtype 3 vs subtypes 1 and 2 groups (c). The horizontal axis indicates the number of genes and the vertical axis indicates the terms of GO biological processes and KEGG pathways. The number of genes enriched in each functional and pathway term is proportional to dot size. *P* value is indicated by dot color from blue (small) to red (large).

Functional enrichment revealed that the DEGs in subtype 1 vs subtypes 2 and 3 group were mainly enriched in 18 GO biological processes, such as GO:0071805 ∼ potassium ion transmembrane transport and GO:0060070 ∼ canonical Wnt signaling pathway, and nine KEGG pathways, such as hsa04310:Wnt signaling pathway and hsa04390:Hippo signaling pathway (Figure 2a). The DEGs in subtype 2 vs subtypes 1 and 3 groups were mainly involved in 14 GO biological processes and 7 KEGG pathways, such as GO:0006355∼regulation of transcription, DNA-templated, hsa04020: calcium signaling pathway, and hsa03460: Fanconi anemia pathway (Figure 2b). In addition, the DEGs in subtype 3 vs subtypes 1 and 2 groups were mainly implicated in 16 GO biological processes, such as GO:0030335 ∼ positive regulation of cell migration, GO:0006198 ∼ cAMP catabolic process, and four KEGG pathways, such as hsa01100: metabolic pathways and hsa00260: glycine, serine, and threonine metabolism (Figure 2c).

### 3.3 Construction of a prognostic model

**Figure 3:**
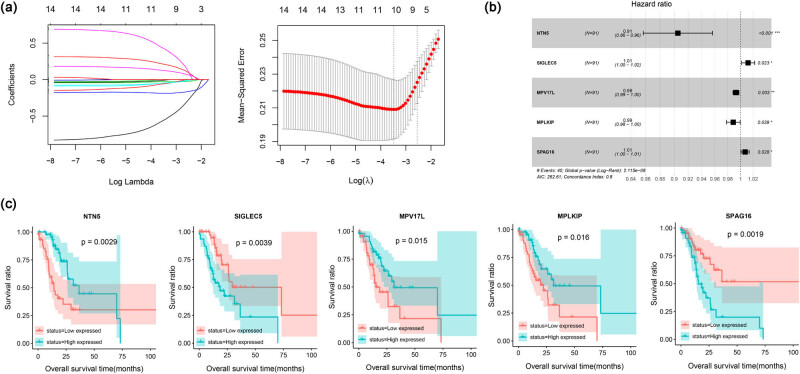
The optimal prognostic genes screened by LASSO. (a) The LASSO coefficient spectrum of the 14 independent prognostic genes (left) and optimized lambda determined in the LASSO regression model (right); (b) forest plot of the optimal prognostic genes screened by LASSO; (c) KM survival curves show the prognostic value of these five genes.

Among the 925 DEGs, the univariate Cox regression analysis revealed 74 prognostic genes. Further multivariate Cox regression analysis identified 14 independent prognostic genes. LASSO regression was then performed to screen the optimal prognostic genes, and the five most valuable prognostic genes were identified, including netrin 5 (*NTN5*), sialic acid binding Ig like lectin 5 (*SIGLEC5*), MPV17 mitochondrial inner membrane protein-like (*MPV17L*), M-phase-specific PLK1 interacting protein (*MPLKIP*), and sperm associated antigen 16 (*SPAG16*) (Figure 3a and Table 1). Forest plots for these five genes suggested that *NTN5*, *MPV17L*, and *MPLKIP* were protective factors (hazard ratio < 1), whereas *SIGLEC5* and *SPAG16* were risk factors (hazard ratio > 1) (Figure 3b). Consistently high expression levels of *NTN5*, *MPV17L*, and *MPLKIP* were associated with better overall survival, whereas higher expression levels of *SIGLEC5* and *SPAG16* were associated with worse overall survival (Figure 3c).

**Table 1:** The five optimal prognostic genes screened by LASSO

Gene name	Hazard ratio	95% Confidence interval	Standard error	*Z* score	*P*-value	LASSO coefficient
NTN5	0.905	0.857–0.956	0.028	−3.555	3.790 × 10^−4^	−0.63634118
SIGLEC5	1.012	1.002–1.023	0.005	2.278	2.273 × 10^−2^	0.241939939
MPV17L	0.993	0.988–0.998	0.002	−2.983	2.857 × 10^−3^	−0.05741236
MPLKIP	0.989	0.978–0.999	0.006	−2.069	3.852 × 10^−2^	−0.07923012
SPAG16	1.007	1.001–1.014	0.003	2.194	2.823 × 10^−2^	0.140763903

**Figure 4:**
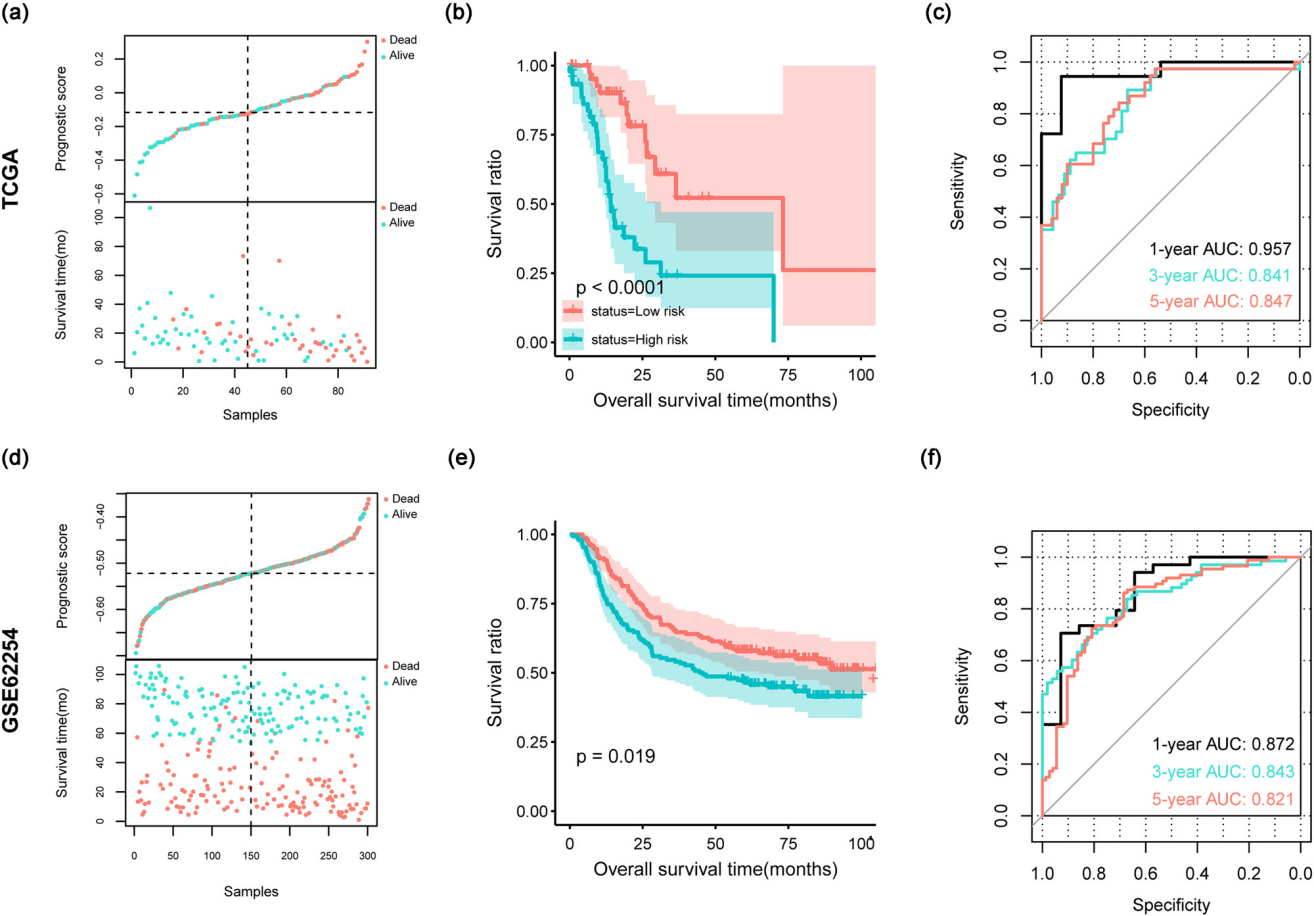
Construction and validation for prognostic model. (a–c) results for TCGA dataset; (d–f) results for GSE62254 dataset. (a and d) the scatterplots in the top panel show the distribution of the risk score, and the scatterplots in the bottom show the survival status of patients; (b and e) KM survival curves show the survival differences between the two risk groups; (c and f) ROC curves show the predictive performance for 1, 3, and 5 year survival.

A prognostic model was constructed based on these five aforementioned genes. The samples were then grouped into two risk groups based on the median risk score. In the TCGA dataset, the distribution of risk scores indicated that high-risk patients tended to have a worse prognosis (Figure 4a). KM curves confirmed that patients in the high-risk group had poor overall survival (Figure 4b). Receiver operating characteristic curves indicated that the prognostic model had a better predictive performance for 1, 3, and 5 year survival (Figure 4c). Similar results were obtained for the GSE62254 validation dataset (Figure 4d–f).

### 3.4 Associations of risk groups with clinical factors and microbial subtypes

**Figure 5:**
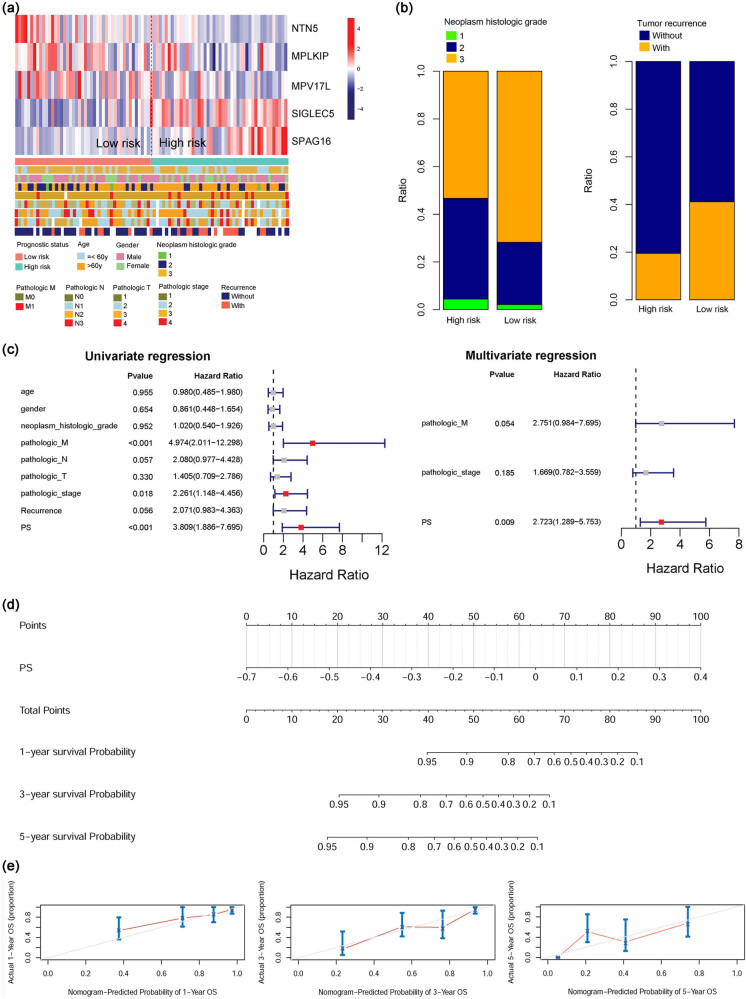
Associations of risk groups with clinical factors and microbial subtypes. (a) Heatmap showing the expression pattern of the five genes and clinical factor distribution in the high-risk and low-risk groups; (b) histograms showing the distribution of proportions for histologic grade and recurrence between two different risk groups; (c) univariate and multivariate Cox regression analysis of factors for overall survival; (d) a nomogram was constructed using the prognostic risk score to predict the 1, 3, and 5 year overall survival probabilities of STAD patients; (e) calibration curves showing the concordance between the predicted and actual 1, 3, and 5 year survival rates of patients.

The expression patterns of these five genes in the prognostic model are shown in Figure 5a. The expression of *NTN5*, *MPLKIP*, and *MPV17L* gradually decreased as the risk score increased, while the expression of *SIGLEC5* and *SPAG16* gradually increased as the risk score increased. The clinical factors of the two risk groups are shown in Table S6. There were significant differences in the neoplasm histologic grade (*P* = 0.0479) and reference (*P* = 0.0468) between the high- and low-risk groups. Specifically, the low-risk group had a higher proportion of samples without recurrence than the high-risk group. In addition, the high-risk group had a higher proportion of histological grade 3 samples than the low-risk group (Figure 5b). This information could be the reason for the poor prognosis of the high-risk group.

### 3.5 Construction of a nomogram

Univariate and multivariate Cox regression analyses showed that the prognostic risk score was an independent prognostic factor for patients with STAD (Figure 5c). Using the prognostic risk score, a nomogram was created to accurately estimate the 1, 3, and 5 year overall survival probabilities of patients with STAD (Figure 5d). Calibration curve analysis also showed that the predicted 1, 3, and 5 year overall survival times were consistent with the actual survival times (Figure 5e). These results demonstrated that the constructed nomogram was reliable for predicting the overall survival of patients with STAD.

### 3.6 Associations of risk groups with immune infiltration and pathways

**Figure 6:**
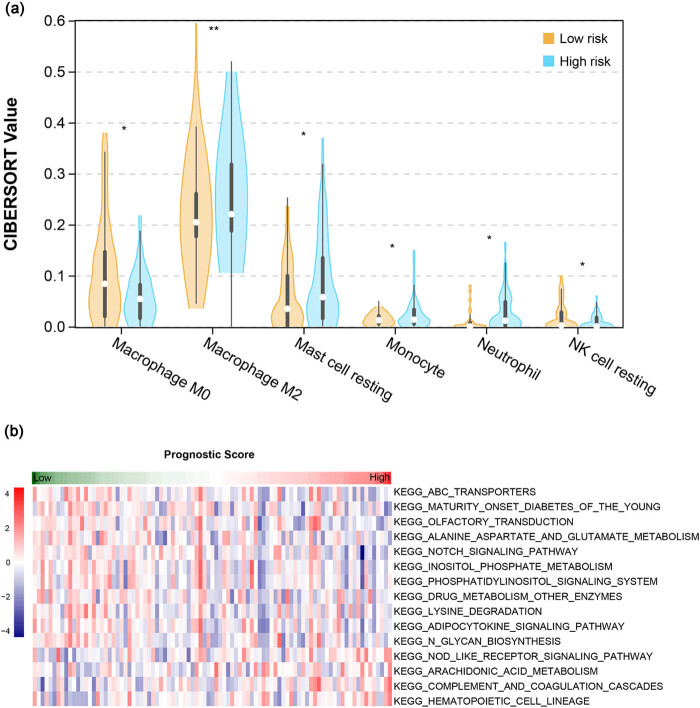
Associations of risk groups with immune infiltration and pathways. (a) Violin plot showing the difference in abundance of the six infiltrating immune cells between the two risk groups; (b) heatmap showing the 15 significant differential KEGG pathways between the two risk groups analyzed by GSVA.

The abundance of 22 infiltrating immune cells was evaluated using the CIBERSORT algorithm. The abundance of six infiltrating immune cells, including M0 macrophages, M2 macrophages, resting mast cells, resting NK cells, monocytes, and neutrophils, were significantly different between the two risk groups. The samples in the high-risk group had a higher abundance of infiltrating M2 macrophages, resting mast cells, and neutrophils (Figure 6a). KEGG pathways with significantly different activities between the high- and low-risk groups were screened using GSVA, and 15 pathways with *P* < 0.05 were screened, including Notch signaling pathway, complement and coagulation cascades, and adipocytokine signaling pathway (Figure 6b).

## 4 Discussion

The gastrointestinal tract is a repository of bacteria in the human body. Intestinal flora form a symbiotic relationship with the human body, which is not only involved in the metabolism of nutrients, the development of the body’s immune system, intestinal barrier function, and other normal physiological processes, but it is also closely related to the development of a variety of human diseases, especially gastrointestinal tumors [30,31,32]. Moreover, intestinal flora affects the efficacy and side effects of radiotherapy, chemotherapy, and immunotherapy [32]. In this study, we have identified nine different microbial genera in STAD samples, including *Helicobacter*, *Mycobacterium*, *Streptococcus,* and *Veillonella*. Of which, *Helicobacter* and *Streptococcus* were more abundant in both tumor and normal samples than other microbial genera.

*H. pylori* is a species of the Helicobacter genus, and its infection is a well-known risk factor for the development of GC [33]. There is a significantly decreased incidence of GC after the eradication of *H. pylori* infection [34]. However, not all people infected with *H. pylori* will develop stomach problems, nor will all people with stomach problems become infected with *H. pylori* [35]. Based on gastric biopsies, Chouhan et al. suggested that infection with *Mycobacterium abscessus* was highly prevalent in GC patients, and gastric *Mycobacterium abscessus* was primarily colonized in the epithelial cells, especially gastric gland-bearing cells and mucosa [35]. *Mycobacterium conceptionense* infection has been reported in patients with advanced STAD [36]. These two studies suggest the prevalence of *Mycobacterium* in GC. The *Streptococcus* genus can survive in low gastric pH and is acid-tolerant [37]. It has been reported that GC patients show a consistent increase in lactic acid bacterial abundance, such as *Streptococcus* [38]. *Veillonella* genus, *Streptococcus mitis*, and *Streptococcus salivarius* are all associated with GC risk, and they display a better diagnostic value in differentiating patients with GC from healthy individuals [39]. *Veillonella* and *Streptococcus* showed positive correlations with serum levels of l-threonine, l-alanine, and methionol in patients with GC [39]. These studies suggest the importance of these microbial genera in GC development. In our study, these nine differential microbial genera clustered the STAD samples into three subtypes. Subtypes 1 and 2 contained a higher abundance of *Streptococcus* and *Helicobacter*, respectively, which had a worse prognosis than subtype 3, further emphasizing the importance of *Streptococcus* and *Helicobacter* in GC development.

We then screened the feature genes of these three microbial subtypes, of which 74 showed prognostic value. Multivariable Cox and LASSO regression analyses identified the five genes with the most prognostic valuable: *NTN5*, *MPV17L*, *MPLKIP*, *SIGLEC5*, and *SPAG16*. *NTN5* encodes netrin-5 belonging to the netrin family, which is homologous to the C345C domain of netrin-1 and promotes tumorigenesis through cell adhesion, apoptosis, angiogenesis, and other processes [40]. *MPV17L*, a crucial paralog of *MPV17*, encodes a transmembrane protein involved in the metabolism of peroxisomal reactive oxygen species. Krick et al. indicated that MPV17L could be involved in protecting mitochondria from apoptosis and oxidative stress [41]. *MPLKIP*, also named *TTDN1*, encodes a protein that plays an important role in maintaining the integrity of the cell cycle by interacting with polo-like kinase 1, and its inhibition or overexpression results in multi-nuclei or multipolar spindles [42]. *SIGLEC5* encodes a siglec belonging to the sialic acid-binding immunoglobulin-like receptor family that regulates immune cell function in various disorders [43]. SIGLEC5 has been reported to regulate amnion signaling responses and neutrophils to group B *Streptococcus*, which means it has significance in regulating host immunity [44]. Soluble SIGLEC5 has also been identified as a prognostic marker in colorectal cancer patients [45]. Therefore, we can speculate that these five genes may be involved in GC development and that the microbiota may affect the clinical outcomes of GC by modulating the expression of these genes in tumors. Based on these five genes, a prognostic model was constructed to stratify patients into different risk groups, and the high-risk group was associated with poor overall survival. Moreover, a nomogram created by the prognostic risk score could accurately predict the 1, 3, and 5 year overall survival probabilities of patients with STAD. Therefore, our constructed prognostic model may guide doctors to assess disease risk and prognosis, and provide a new insight for developing effective personalized treatment plans for patients with GC. Furthermore, we found that the low-risk group had a lower proportion of recurrence than the high-risk group, while the high-risk group had a higher proportion of histologic grade 3 samples. These data suggest that the overall survival of GC patients can be multifactorial and can be determined by transcriptomic dysregulation as well as by risk factors such as disease recurrence and histologic grade.

Immune infiltration analysis showed that samples in the high-risk group had a higher abundance of infiltrating M2 macrophages, resting mast cells, and neutrophils. Tumor-associated macrophages are heterogeneous, with a tumor-promoting M2 phenotype and a tumor-inhibiting M1 phenotype. Macrophages in the tumor microenvironment are generally polarized to the M2 phenotype to promote tumor progression [46]. Elevated mast cell density has been observed in GC, promoting its progression by releasing lymphangiogenic and angiogenic factors [47]. The neutrophil-to-lymphocyte ratio was established as a prognostic marker in patients with GC [48]. The activation of neutrophils in the tumor microenvironment contributes to developing an immunosuppressive phenotype and promotes tumor growth and progression [49]. This might be the reason for the poor prognosis of the high-risk group.

Furthermore, the Notch signaling pathway activity showed a significant difference between the high-and low-risk groups. Notch signaling, a key regulator of multiple cellular functions, is highly expressed and activated in gastric cancer [50]. Activation of Notch signaling pathways has been reported to be involved in STAD progression through modulating immune cells and regulating other pathways such as PI3K-Akt signaling [51]. Notch signaling ligand Jagged1 has been shown to promote macrophage-mediated response to *H. pylori* [52]. Given the key role of the Notch signaling pathway, it can be speculated that the Notch signaling pathway may be a key mechanism affecting the role of microbiota in GC development and prognosis.

Our study had several limitations. First, the five prognostic genes used for the prognostic model construction were not validated in clinical samples. Second, our analysis was based on online public data, and the robustness of our constructed prognostic model needs to be validated in prospective clinical cohorts. Further studies are required to confirm our findings.

In conclusion, we have identified nine differential microbial genera in STAD that could cluster STAD patients into three microbial subtypes with significantly different survival rates. The prognostic model based on the key feature genes of these microbial subtypes could predict the overall survival of STAD patients, and the model showed the associations with the clinical characteristics and immune microenvironments of the patients. The Notch signaling pathway may be a key mechanism that affects the role of the microbiota in GC development and prognosis. These results deepened our understanding of the importance of microbiota and their clinical predictive value.

## Supplementary Material

Supplementary Table 1

Supplementary Table 2

Supplementary Table 3

Supplementary Table 4_6

Supplementary Table 5
