# Supplementary material for: Integrated analysis of the microbiome and transcriptome in stomach adenocarcinoma
Source: Open Life Sci. 2023 Jul 15;18(1):20220528. doi: 10.1515/biol-2022-0528 (PMC10350897; doi:10.1515/biol-2022-0528)
Supplement: Supplementary Table 4_6 [file j_biol-2022-0528-S4_6.pdf]

# Supplementary material

**Table S4:** Associations of the clinical factors among three subtypes

| Characteristics total cases | N of case 91 | Subtypes | | | *P* value |
|---|---|---|---|---|---|
| | | Subtype 1 ($N$ = 72) | Subtype 2 ($N$ = 8) | Subtype 3 ($N$ = 11) | |
| Age (years) | | | | | $4.82 \times 10^{-1}$ |
| ≤60 | 30 | 22 | 4 | 4 | |
| >60 | 61 | 50 | 4 | 7 | |
| Geneder | | | | | $3.27 \times 10^{-1}$ |
| Male | 59 | 46 | 7 | 6 | |
| Female | 32 | 26 | 1 | 5 | |
| Pathologic M | | | | | $5.89 \times 10^{-1}$ |
| M0 | 80 | 64 | 7 | 9 | |
| M1 | 10 | 7 | 1 | 2 | |
| Pathologic N | | | | | $6.27 \times 10^{-1}$ |
| N0 | 32 | 22 | 3 | 7 | |
| N1 | 29 | 25 | 2 | 2 | |
| N2 | 14 | 11 | 2 | 1 | |
| N3 | 13 | 11 | 1 | 1 | |
| Pathologic T | | | | | 1.35E- $\times 10^{-1}$ |
| T1 | 3 | 2 | 0 | 1 | |
| T2 | 36 | 27 | 6 | 3 | |
| T3 | 35 | 30 | 2 | 3 | |
| T4 | 13 | 9 | 0 | 4 | |
| Pathologic stage | | | | | $6.01 \times 10^{-1}$ |
| Stage I | 17 | 11 | 3 | 3 | |
| Stage II | 32 | 27 | 1 | 4 | |
| Stage III | 22 | 17 | 3 | 2 | |
| Stage IV | 13 | 10 | 1 | 2 | |
| Neoplasm histologic grade | | | | | $4.28 \times 10^{-1}$ |
| G1 | 3 | 2 | 0 | 1 | |
| G2 | 31 | 27 | 2 | 2 | |
| G3 | 57 | 43 | 6 | 8 | |
| Recurrence | | | | | $3.87 \times 10^{-1}$ |
| Yes | 21 | 20 | 1 | 0 | |
| No | 49 | 42 | 2 | 5 | |

**Table S6:** Comparison of the clinical factors between high- and low-risk groups

| Characteristics total cases | N of case 91 | Group | | *P* value |
|---|---|---|---|---|
| | | Low risk (*N* = 45) | High risk (*N* = 46) | |
| Age (years) | | | | $1.19 \times 10^{-1}$ |
| ≤60 | 30 | 11 | 19 | |
| >60 | 61 | 34 | 27 | |
| Gender | | | | |
| Male | 59 | 29 | 30 | $9.99 \times 10^{-1}$ |
| Female | 32 | 16 | 16 | |
| Pathologic M | | | | $9.05 \times 10^{-2}$ |
| M0 | 80 | 42 | 38 | |
| M1 | 10 | 2 | 8 | |
| Pathologic N | | | | $4.87 \times 10^{-1}$ |
| N0 | 32 | 18 | 14 | |
| N1 | 29 | 12 | 17 | |
| N2 | 14 | 9 | 5 | |
| N3 | 13 | 6 | 7 | |
| Pathologic T | | | | $1.77 \times 10^{-1}$ |
| T1 | 3 | 3 | 0 | |
| T2 | 36 | 17 | 19 | |
| T3 | 35 | 16 | 19 | |
| T4 | 13 | 9 | 4 | |
| Pathologic stage | | | | $4.44 \times 10^{-1}$ |
| Stage I | 17 | 9 | 8 | |
| Stage II | 32 | 17 | 15 | |
| Stage III | 22 | 13 | 9 | |
| Stage IV | 13 | 4 | 9 | |
| Neoplasm histologic grade | | | | $4.79 \times 10^{-2}$ |
| G1 | 3 | 2 | 1 | |
| G2 | 31 | 19 | 12 | |
| G3 | 57 | 24 | 33 | |
| Recurrence | | | | $4.68 \times 10^{-2}$ |
| Yes | 21 | 7 | 14 | |
| No | 49 | 29 | 20 | |
