# Supplementary material for: Integrated analysis of the microbiome and transcriptome in stomach adenocarcinoma
Source: Open Life Sci. 2023 Jul 15;18(1):20220528. doi: 10.1515/biol-2022-0528 (PMC10350897; doi:10.1515/biol-2022-0528)
Supplement: Supplementary Table 1 [file j_biol-2022-0528-S1.pdf]

Table S1: TCGA data

Symbol	TCGA-B7-5	TCGA-BR-4	TCGA-BR-4	TCGA-BR-4	TCGA-BR-4	TCGA-BR-4	TCGA-BR-4	TCGA-BR-4	TCGA-BR-4
A1BG	0.01808141	0.02069522	0.35378102	0.03770991	0.0710786	0.04281303	0.03231071	0.05165985	0.02371419
A2M	5.12695062	8.29540041	7.33036968	7.4683403	6.81809739	7.56914085	7.03729687	6.04060259	5.31189234
A4GALT	1.77062169	2.94144706	1.67874223	2.91264396	0.85100909	2.83984587	1.95700372	1.30386352	1.03968784
A4GNT	5.14413691	0.02338443	5.5054474	0.34919599	0.44390307	1.59598499	0.07208034	4.23234997	0.04263168
AAAS	3.41714175	2.96501421	3.2166327	3.27506744	3.38296628	3.38139402	3.10880653	3.51570473	3.852214
AACS	2.21655861	1.12703638	1.67652159	1.21429913	1.29985962	1.08287962	1.30148583	2.09684257	1.96075513
AADAT	1.00607184	0.87187565	0.58102145	2.5129708	2.19080529	1.34199682	1.20712132	2.00174847	0.49092777
AAK1	1.50668052	1.96926112	1.59714671	1.76841346	2.05642042	2.00191677	1.83326744	2.06483759	1.17518454
AAMDC	2.33068299	2.53875773	2.16504705	2.11979226	1.89032453	1.93298178	1.81303905	2.14813163	2.18703605
AAMP	5.4715556	3.7800149	4.34772156	3.99052572	4.56192941	4.23657473	4.10074444	4.74456913	4.82301047
AAR2	4.07560753	3.59425722	4.07965525	3.68337369	3.30197397	3.82353007	3.53318716	3.63085728	4.17229035
AARS	5.16520024	4.50697019	4.56147596	4.63434298	4.98888591	5.09287601	5.56602406	4.73022023	4.75499464
AARS2	2.51741495	1.92512268	2.41676637	2.05029543	2.37956007	2.32613367	2.24381366	2.88212672	2.76923288
AARSD1	0.75602588	0.59995148	1.22967834	1.12599579	0.81251303	0.60522907	0.71454225	0.84937955	0.99754851
AASDH	1.15843189	1.63598178	1.83376361	2.03913524	2.29640275	1.80691272	1.22769366	1.82449604	1.97144686
AASDHPPT	1.91616044	2.78312943	2.32691834	3.22100388	3.45013917	3.17300696	2.89995043	2.27568287	3.00484077
AATF	5.24233433	3.15745205	3.41408109	3.63309686	4.18793945	3.80218532	3.41788137	3.91929324	3.57983636
AATK	3.54808798	1.35073952	0.90135662	1.99631397	0.52606358	2.2766142	1.57639306	1.06577132	2.08412111
ABCA1	1.50396157	4.14315314	2.94132814	3.46313518	3.30822739	3.2326869	2.39453885	2.54551393	2.05086838
ABCA2	2.82455681	2.16991499	2.20055205	2.85006293	1.92978157	2.03999848	2.40917382	2.50105947	2.75564711
ABCA3	1.85161516	1.37961926	0.72870764	2.12361291	0.66383168	1.70477258	0.54712675	2.61944936	0.35890129
ABCA4	0.01327067	0.11022725	0.44415544	0.06260994	0.01903756	0.11754016	0.01587346	0.1151734	0.0300517
ABCA5	0.58969377	0.74907597	1.59961431	0.98145585	0.9465494	0.59926663	0.37206915	1.24242738	1.70197015
ABCA6	0.00770569	1.47201862	0.98374892	0.93546249	0.17883305	0.93585316	0.12966173	0.17988116	0.04792971
ABCA7	1.81476386	1.04030565	1.9727204	1.13217917	1.67150045	1.06075998	1.42460976	1.3811125	1.57636417
ABCA8	0.03205689	3.47855921	0.74720246	1.04610309	0.10213597	1.20644399	0.06821669	0.14263467	0.03368153
ABCA9	0.03545149	1.11984368	0.51132867	1.28950814	0.25183277	0.88758144	0.0834752	0.09227865	0.0735545
ABCB10	2.64590956	2.51919788	2.41906663	2.90119792	3.02525206	2.91338723	3.65013785	3.29624961	2.89279765
ABCB6	0.14002014	0.62489593	0.83059073	0.46313473	0.25259998	0.74795616	0.22107171	0.89566469	0.79893831
ABCB7	2.23785754	3.56859116	2.95138559	3.58827522	4.24145902	3.32469781	2.80900154	2.94499341	3.7715737
ABCB8	2.51370992	1.96971831	2.09638235	1.66395537	2.06193944	1.77561023	1.84005334	1.82903007	2.07483182
ABCC1	3.63288387	4.06573717	3.89414607	3.28915934	3.78306209	4.09845266	3.87052277	4.06268993	3.68918032
ABCC10	2.15795621	1.44189056	2.04910331	1.72766744	1.76263186	1.63184802	2.08287756	1.98490733	2.32811771
ABCC2	0.01164892	1.02870875	0.35081678	0.44353096	0.10086694	0.09961782	0.0242989	0.74005083	0.21122596
ABCC4	0.68181978	2.60097366	3.46058417	2.63735512	3.08940818	2.31518865	1.87990211	2.48891019	3.41177576
ABCC5	4.00268928	1.36930138	3.15913027	1.79241623	1.99876309	1.46278405	1.55271079	2.14244303	1.72412806
ABCC6	0.11130721	1.93056608	0.92460625	0.38378711	0.21740891	0.78203278	0.2388424	0.66310469	1.81373782
ABCC8	0.00536695	0.17660966	0.27824374	0.07541637	0.02300268	0.0273968	0.00642303	0.01648453	0.00845415
ABCC9	0.23505216	3.80319386	1.07327923	2.46115278	0.96529381	3.04936065	0.80686382	0.45953521	0.56689551
ABCD1	3.05701661	3.78820605	2.64517953	2.23112287	2.36373447	3.34934111	2.08532479	2.89536285	3.17480024
ABCD4	2.38653847	1.75679737	2.31536423	1.87453982	1.74020438	1.85038141	1.80113841	1.96229618	1.97789104
ABCE1	2.98021308	3.81599603	4.07594339	4.16131186	5.18850687	4.49819325	4.28957682	3.92611978	4.75690197
ABCF1	4.49490101	3.86057859	4.87133738	3.86103726	4.52355424	4.39217253	4.2800918	5.02100775	3.64480554
ABCF2	4.15422506	3.80998056	4.38345323	4.2344916	4.39726684	4.16845966	4.07898072	3.99472905	4.0084103
ABCF3	4.05205468	2.56731253	3.61813927	3.06419688	3.04241727	3.03431845	3.3210762	3.73699525	3.16045042
ABCG1	1.68166339	2.52514605	2.12443137	2.40825819	2.47999264	2.61550567	2.23062402	3.05991934	1.98828482
ABCG2	0.10857299	1.60338731	1.38056717	0.60900312	0.41559108	0.938902	1.30218309	0.43665818	0.5187933
ABCG4	0.02876956	0.17237052	0.10523342	0.21351588	0.0458081	0.18229749	0.0479005	0.03102309	0.01821351
ABCG8	0	1.74926686	0.82688766	0.0081814	0.07389653	0.08772198	0.2950989	0.06190206	0.0494605
ABHD10	3.38183972	3.18730489	3.41739829	3.86050965	3.95908805	3.2808792	4.0118379	3.37957025	3.78377388
ABHD11	4.29247954	3.69742919	4.28110438	4.24160778	2.93530273	3.81486702	5.00282905	3.65040703	5.47917072
ABHD12	4.95516666	4.04730218	4.91566075	4.38502172	4.34258145	4.08079336	5.03000335	4.50002816	4.48785096
ABHD13	2.41418301	2.73140786	3.24994857	2.93215323	2.82820442	2.86542626	2.72197075	2.80866185	3.30349761
ABHD14A	2.97499181	2.75233314	3.01366802	2.62350251	1.33243564	2.30058813	2.53473944	2.42236357	2.66534414
ABHD14B	4.74787561	4.53927423	4.00260521	4.19559168	3.85792964	4.49991282	4.54151818	4.22414291	5.27306532
ABHD15	2.81003614	2.47720235	2.89974042	2.34605859	2.18258008	3.17737217	2.74781472	3.26068467	2.33567534
ABHD16A	1.48568362	0.78051631	1.54617882	1.21706215	1.31261271	1.16375309	1.35935636	1.37659541	1.7331186
ABHD17A	3.8820519	2.64474451	2.36960805	1.9971753	2.35810556	2.98441616	2.85929841	2.90859919	2.70245707

ABHD2	4.41077705	5.48456795	4.91483897	5.14570906	4.69377397	5.72148617	5.12077594	6.28680551	4.57979775
ABHD3	2.69327064	2.02113358	4.47369355	2.87073671	3.42752814	2.23824253	3.92143864	3.38986202	2.77129638
ABHD4	3.76551643	4.08885606	4.75365223	3.61598204	2.66659911	3.77011246	3.06098337	4.22663399	3.42605552
ABHD5	2.31672113	3.29339821	2.99624424	3.33795439	3.6585064	3.42153297	3.37336145	2.8633583	2.07695474
ABHD6	1.77454308	2.47296243	2.37153551	2.30079083	1.90152855	2.17128612	2.29696498	3.11815246	1.85871371
ABHD8	2.58105248	2.66807898	2.63480907	2.09464437	1.40093832	2.47971352	1.23140951	2.77438052	1.24483135
ABI1	3.8744983	4.32105282	4.24724609	4.75200721	5.04257683	4.73040427	4.46702174	3.90420302	4.64879756
ABI2	1.37145665	1.78027047	1.48620886	1.80748944	1.38673528	1.81567452	1.3418729	1.1971363	1.10257553
ABI3	3.49774053	3.01677173	4.39057417	2.38431146	4.26433276	3.18807733	3.22671344	2.11965032	3.00221676
ABI3BP	0.29878997	4.57233987	2.059589	2.80763199	0.870166	3.24980017	0.46485691	1.05798111	0.40375994
ABL1	4.57741796	4.85541039	3.94843679	3.95971767	3.77853558	4.71154302	3.97469853	3.5386186	4.07387868
ABL2	1.46902096	2.6489023	2.03426923	2.14273918	2.0896278	2.48666496	2.02364977	2.36998914	1.68058136
ABLIM1	5.03598102	4.71755096	5.0144654	4.14196102	4.35314841	4.54900986	4.85954742	4.82265508	4.27916142
ABLIM2	0.30806067	0.94720956	0.65651691	0.55437566	0.28227081	0.50944351	1.00895593	0.37603796	2.34692302
ABLIM3	0.44745114	2.53370902	1.37934888	2.48170756	0.83862673	2.19586911	1.15748039	3.51480155	0.61287343
ABO	2.9023529	1.51399085	2.26994296	1.66040002	1.82866511	0.43981225	4.12415392	4.55753925	3.74244506
ABR	2.9809847	3.487524	3.33221307	3.27753105	3.92927539	3.35889339	3.57539287	3.0162191	4.16595432
ABTB1	1.27342727	1.77857279	2.25471861	2.52223052	1.45881804	2.35383024	2.60891483	2.49917827	2.06455149
ABTB2	2.38300293	1.67137367	0.95021915	2.21945297	2.60827228	2.46332987	2.47381117	1.44514651	1.7486264
ACAA1	2.75150457	2.02600829	2.57766856	2.24258985	2.19142058	2.29708218	3.47299579	2.48815362	2.4759148
ACAA2	3.31544468	4.96192531	4.72091857	4.60528492	2.98475388	4.37600809	6.32668674	2.07105724	5.20383292
ACACA	2.95752835	2.37459449	2.18487204	2.84113353	2.58544376	3.13147702	3.21420191	2.70407027	3.36277723
ACACB	0.3934005	1.5710539	1.4680912	1.12272195	1.3884226	1.09132812	0.54675448	1.70252585	1.55438116
ACAD10	1.22346739	1.95450592	1.56944521	1.50996631	1.64906888	1.42250288	1.76521223	2.15817281	2.19587388
ACAD11	0.07508935	0.10293499	0.14134022	0.14645276	0.11407678	0.1112396	0.0416862	0.13011103	0.05952729
ACAD8	1.65119792	1.53102954	1.54002637	1.92810701	1.77894334	1.57570429	1.75872703	1.30915464	1.68090782
ACAD9	4.16623498	3.04497754	3.25288661	2.96516148	3.42678414	3.24317362	3.2839793	3.1366009	3.38905912
ACADM	0.91632593	4.14465133	3.7409921	3.38004406	2.60305752	3.36064905	3.02706268	4.02370894	3.09753584
ACADSB	1.78213234	2.41115233	2.73309372	2.54924647	3.01848873	2.17672818	2.11644057	3.17652394	2.84647068
ACADVL	4.276616	4.98968111	4.87101405	5.26229915	5.0960877	4.87326201	4.63444526	4.6876321	5.8437379
ACAN	0.72110931	0.42607539	1.44271391	0.90822189	1.21126553	0.75927605	2.14042664	1.27080978	1.15671415
ACAP1	1.31079232	0.80632001	1.59059077	0.97226892	1.74901698	1.43282774	0.74019814	0.55294051	0.98589679
ACAP3	2.29534066	1.6778355	1.32192563	1.8354033	1.30235394	1.50520218	2.03389458	1.09804492	2.68099871
ACAT1	1.94827745	3.45226033	2.8275749	3.67146219	4.6403826	3.79497676	4.31721477	2.79032239	3.15873754
ACAT2	2.84078929	1.7116404	3.07323366	3.41075598	2.30830948	2.60002188	3.97589503	2.52224493	2.69724107
ACBD3	4.89753035	4.13527508	4.94289113	4.57478552	5.06041804	4.58133475	5.28332945	5.33705753	4.39023463
ACBD5	2.78191397	3.23091454	3.28316915	4.2863147	3.58257254	3.35962552	4.0907352	3.71489653	3.61139264
ACCS	1.68124249	0.55035615	1.32223076	1.24070986	0.85548635	0.77445845	0.35650671	0.77702984	1.60184252
ACD	3.48405291	2.15460928	2.49220294	2.3612444	2.4144552	2.7227485	2.28276005	2.79851099	2.73658077
ACE	1.90965676	1.94707583	2.38453494	2.00470438	2.64730425	2.30650019	2.2808206	3.39727885	1.12605034
ACE2	4.75050139	2.9627991	1.25417112	0.3362913	4.2403924	0.16727677	0.46201842	0.60952241	0.77136065
ACER3	2.94653053	2.3745318	3.34788224	2.6982239	3.46386313	2.37697936	2.87774741	2.53149011	3.03742009
ACHE	0.48488583	0.82519876	2.41256291	0.95180896	2.50905472	0.94145271	4.24665489	1.79220995	0.81398396
ACIN1	3.81700377	3.05395097	3.52024406	3.69951153	4.10858693	3.39550128	3.17430151	3.81036162	4.02513563
ACKR1	0.43066903	5.00344907	3.21889172	2.92037743	0.49959106	4.6050524	1.35509284	1.04173575	0.55864089
ACKR2	0.16362233	0.43780024	0.34138073	0.17309808	0.17018687	0.68641353	0.16207497	0.10163881	0.06840824
ACKR3	2.47045553	4.33576827	3.98400525	3.89926154	1.69288855	4.10294566	3.75309095	3.00589949	2.75264384
ACKR4	1.30277223	2.82883094	2.61227588	1.61683695	4.20356058	3.07795531	0.88354704	0.74938382	1.97333983
ACLY	5.39493685	4.59468958	4.68750722	4.5081944	4.91579239	5.09075256	5.21690053	5.19998152	5.2802286
ACMSD	0.90364077	0.16953463	0.79406851	0.00782983	0.13092347	0.01910555	0	0.43253225	0
ACO1	2.79669298	3.68668081	2.81393737	3.53106838	3.28575525	3.18467533	3.44064693	3.00308861	3.74299405
ACO2	5.74042478	4.65467022	4.63967856	4.40192914	4.98136529	4.89994039	5.14770348	4.67534998	5.40832502
ACOT1	1.72913475	1.53635055	1.21482134	0.42766607	0.62769074	1.15351992	1.02974702	1.04067434	0.97173593
ACOT11	0.90223076	1.35188918	1.13718863	1.59906897	1.09917387	0.78740456	1.02331282	1.72921677	2.30595392
ACOT13	2.30900401	2.73713035	3.03235908	2.93873002	3.1477328	2.85723702	2.35307317	3.11475847	2.62115972
ACOT4	0.70576414	1.161872	1.89223459	1.13322163	0.78638555	2.25234387	1.16066841	2.04567504	0.84210374
ACOT7	4.08068118	2.98214805	3.20100186	3.844972	4.54797577	3.6153646	3.45404217	1.89795464	5.25678008
ACOT8	2.56774601	2.5534184	3.00710043	2.23098577	1.98909514	2.0347728	2.60295611	2.42868484	2.95967822
ACOT9	1.98320675	3.00357971	3.16942178	3.32259785	4.01695448	3.09046786	2.92465931	2.90588461	1.67090231

ACOX1	3.61684974	3.43937481	4.05056928	3.56375441	3.11924196	3.44910536	3.27101875	5.07514093	3.11612497
ACOX2	0.18582832	2.45932059	0.99506309	1.13352126	0.34962098	1.06651874	0.59399039	0.28629394	3.46429645
ACP1	4.13370452	3.82714208	4.5842882	4.48615908	4.50041091	4.29419726	4.53984476	4.55581834	5.00489299
ACP2	4.73741731	3.91053619	4.13582905	4.10724854	4.42943245	4.57945837	4.18971325	3.94909248	4.13703723
ACP5	5.5693612	3.90658424	6.02998734	4.61716657	6.40948484	4.76497178	5.1029015	3.45206402	4.81683461
ACP6	1.4387555	0.95610041	1.28628542	1.34103642	1.36426329	1.15658172	1.79832308	1.53243203	1.31224917
ACR	0	0.04982332	0.09883817	0.09254211	0	0.08216093	0	0.01358	0.03674387
ACSBG1	0.03931419	0.10042026	0.19146761	0.05729636	0.08475769	0.04011363	0.02626752	0.0303991	0.1772003
ACSBG2	0.0105344	0.04178231	0.42669616	0.15045706	0.1717356	0.06085419	0.04363658	0.06377003	0.07577995
ACSL3	3.64992606	3.60100944	4.10655194	4.21340298	4.17339248	3.97782025	4.27261232	5.29921836	3.73047055
ACSL4	3.84332753	4.81511736	4.55150681	4.70414787	4.87239288	4.59326368	4.33458977	3.76089689	3.85900836
ACSL5	4.67536752	5.86116262	7.10740244	3.8575607	5.68218206	4.88629871	2.27534712	5.15059625	6.53676919
ACSS1	1.31173161	2.67077865	3.54860055	2.59615751	1.68160204	2.48315911	2.71808461	3.43836682	4.07623377
ACSS2	3.09242058	3.52740064	4.28425433	3.83379709	3.51107407	3.14614094	3.85673476	3.19017262	6.0498663
ACTA2	5.75047626	8.94153604	6.50577167	7.74651407	5.5232596	8.42814469	6.19715573	5.65431373	4.82711181
ACTB	10.9363079	10.3178033	10.3901706	10.7476735	10.8607227	10.9965182	10.9570886	9.52566513	10.3144597
ACTC1	0.16324267	2.62564574	1.20548395	0.86655225	0.36569437	1.35987512	0.30796372	0.48199602	0.20377149
ACTG1	10.5595273	9.53109702	10.0134568	10.6775866	10.3496059	10.7556794	10.6376621	10.8839605	11.032277
ACTG2	4.59021462	9.73418416	4.37301075	5.78047675	2.47330138	9.02178649	3.82795445	3.01925234	3.50793591
ACTL10	0.24713404	0.64493538	1.16472667	0.36900284	0.14721832	0.88137438	0.75360412	1.39634048	1.44120024
ACTL6A	4.24343897	3.27000512	4.92621934	4.31024547	4.00335212	3.94046909	4.51382962	4.62061163	4.54698883
ACTL6B	0	0.00871188	0.4180665	0.01818988	0.01867449	0.038712	0	0.0348538	0.01594904
ACTN1	4.50736618	6.00660881	4.4013131	5.36288731	4.09318705	6.17743318	5.11361967	5.32662962	4.49887241
ACTN2	0.01312097	1.12612088	0.33047019	0.18179324	0.02814354	0.04528948	0.00786862	0.04254765	0.01379053
ACTN4	7.56673014	6.52189228	6.03414259	6.5780975	7.01231047	6.67449175	6.8170746	6.67327289	6.84477398
ACTR10	3.35142341	4.02720903	3.82770119	4.09541917	4.2815663	3.68715013	3.15376242	3.56094112	3.69014685
ACTR1A	4.95321666	4.74053867	4.3377642	4.39159965	4.43950366	4.90313554	4.40025263	3.89584873	4.67888751
ACTR1B	4.77365893	3.84037318	3.83122933	3.88175692	3.58614668	3.96760409	4.34040779	4.19254238	4.39958116
ACTR2	5.40993159	6.08704356	6.00928686	6.03673609	6.4792159	6.35350827	6.10374131	6.13483705	5.95400782
ACTR3	3.89625821	4.23413766	4.98233666	4.93515762	5.44400157	4.82793331	4.64573072	4.67314016	4.5121143
ACTR3B	1.34427136	0.72539642	0.99327366	1.17180196	1.14003109	1.11220441	1.06831605	0.83502476	1.28129391
ACTR5	1.98711567	1.77879422	2.40534335	2.75345346	2.04849141	2.29090282	2.1127104	2.35250876	2.84835947
ACTR6	1.64462942	2.09847811	2.32785894	2.8449459	3.19127795	2.20932012	2.27469411	2.30301869	2.54180304
ACTR8	2.50072257	2.42687283	2.59471463	2.58012302	2.05310154	2.64183103	2.97689081	3.04996583	2.81255785
ACTRT3	0.12193782	0.83725086	2.01119271	0.92564104	0.20818644	0.42996072	0.8715486	1.43534679	0.91643142
ACVR1	4.51006703	3.72678411	4.03168073	3.64800148	3.44199253	3.49058868	3.51186575	3.53486307	2.88745459
ACVR1B	4.12360156	3.25083713	3.47087097	3.18164131	3.5587843	2.53379725	3.18466595	4.95839469	3.71739425
ACVRL1	2.15491948	3.50059925	3.13631167	2.34245473	2.4485645	2.91518653	2.52119378	2.0540598	2.18549772
ACY1	0.75881653	1.34078056	1.13022615	1.49927919	0.69230245	1.20323266	1.68651708	1.90935651	1.91258784
ACY3	4.39734802	3.94827348	2.54334734	1.99863156	3.01129828	1.13716585	0.38121282	3.00453108	4.34448119
ACYP1	1.83983347	1.70299531	2.02615049	2.33403216	2.62802767	1.60496316	1.25092371	1.12390764	2.4558261
ACYP2	1.37170572	1.75518538	1.47290229	1.72737437	1.28813187	1.28894456	0.97949022	0.90672331	1.65565748
ADA	1.5382265	2.44613487	2.50859206	2.82470373	4.47949891	2.90547701	1.88291882	2.06762925	3.31313582
ADAD2	0	0.01515412	0.05034945	0.01059847	0.00545106	0.02582576	0	0.01021426	0.00928981
ADAM10	2.63803658	2.92454752	3.55274665	3.74879107	4.28369072	3.56634015	3.64432961	2.60881676	3.34409538
ADAM11	0.20612156	0.12565849	0.37251977	0.26705607	0.14752008	0.03604064	0.04502621	0.15878496	0.07053708
ADAM15	3.82573769	4.1267935	3.72543053	4.12531233	4.1101167	4.74762938	6.21481601	4.74585832	4.49952149
ADAM17	2.05678837	2.33335595	2.8702219	2.84230853	2.54684795	3.10288936	2.63852668	2.25979152	2.22742187
ADAM19	2.78295313	2.63943416	2.67408264	2.62802974	2.85112615	2.78152309	2.51618165	1.70920128	1.9105253
ADAM22	0.8507766	0.54267326	0.65076933	0.74917353	0.51721321	0.8410024	0.97772909	0.72008774	0.53451805
ADAM23	0.22062449	1.60166503	0.70575551	2.92278063	0.15249279	1.42285094	0.15755827	0.17877316	0.06703775
ADAM33	0.0124402	2.65201674	0.95260843	1.66175746	0.10392134	1.58794848	0.32357235	0.245189	0.1014265
ADAM8	2.14726978	1.89233171	3.36095784	2.79664359	2.77621328	2.87691019	2.67882912	4.18534732	2.34898781
ADAM9	5.00488327	4.84090504	5.51324501	6.1743588	5.87145737	6.03515574	6.77547223	6.79417776	4.63832709
ADAMDEC1	3.43185921	3.80224402	5.48711447	2.94396052	6.16968259	4.00034387	3.83275288	3.22813561	3.96214663
ADAMTS1	1.32318772	4.72714111	2.81267018	3.6193132	2.60742807	4.54125305	2.67168853	2.43153441	1.93428721
ADAMTS13	0.66374392	0.18830729	0.8294137	0.46434877	0.27817455	0.15341665	0.0708501	0.16492476	0.530393
ADAMTS14	0.84082729	1.69640526	1.67940663	1.90018452	0.80020117	1.86417565	1.67345644	1.13580585	2.8847889
ADAMTS15	0.12308276	2.00627277	0.44394162	1.05611266	0.19584008	0.84542364	0.71814855	0.20722296	1.16384887

ADAMTS17	0.00881672	0.21819025	0.27807661	0.5475039	0.22642899	0.31411463	0.31713425	0.12872048	0.5967659
ADAMTS2	3.32256833	4.03759091	2.80336413	4.63899265	3.33861181	4.59102637	4.77484654	2.93539572	2.59673977
ADAMTS4	2.24789762	2.13235815	2.64967793	2.63069774	1.91203192	3.38414751	2.56657855	1.35839613	1.30823747
ADAMTS6	0.22048917	0.35657022	0.53767624	1.09116326	0.48505502	0.62250619	0.69717204	0.39859024	0.24232222
ADAMTS8	0.08601856	2.78443896	0.96217314	1.37200761	0.02460838	2.28704532	0.31117607	0.69211801	0.16018562
ADAMTSL2	0.95993453	1.1836886	1.59705307	0.78732931	0.5549968	0.85257933	0.9103017	0.99945351	0.62239641
ADAMTSL3	0.09051078	1.6053784	0.57008693	0.4943167	0.26043469	1.52956461	0.16867483	0.31563249	0.12592838
ADAMTSL4	1.04019057	2.92695514	2.59913156	2.34983453	1.5417211	3.24808505	1.97171035	2.35966386	0.70456675
ADAMTSL5	0.29862171	1.48555757	0.58829214	2.72665378	1.38636275	2.32398881	1.64114828	1.40112903	2.04680333
ADAP1	2.99759012	2.15758777	2.66521839	2.09305836	2.02220581	2.09400812	2.03381548	2.98698814	3.25335446
ADAP2	2.58782721	2.92932944	3.44549889	2.55735293	3.08446591	3.23237058	2.74544496	1.77501208	1.68395832
ADAR	5.26427595	5.94526441	5.47868007	5.48212215	6.3843029	6.2060665	6.03328925	5.90247676	5.78749279
ADARB1	1.07045185	1.94379066	1.15142192	1.99409102	1.31831453	1.55188224	1.44216837	0.86762336	1.16438588
ADAT1	1.87325193	2.60566441	3.12864762	2.58419031	3.17321132	2.80441368	3.18269625	2.81338995	2.431778
ADAT2	0.6925518	0.73596753	2.06685671	1.62816668	1.65310827	1.00927923	0.96073334	0.62300544	1.83241047
ADAT3	2.21295607	1.44392681	1.64210941	0.95734994	1.0261344	1.94814835	1.55253499	2.27206261	2.39070443
ADCK2	4.36813257	3.10434415	3.44031701	2.66287212	3.07116555	3.65987913	3.50146389	3.11282036	3.44695934
ADCK5	2.27827764	2.15762485	2.62566324	2.1061265	2.15587887	1.67666426	2.89450177	2.43281928	3.96888787
ADCY1	0.09385116	0.44744038	0.43660132	0.27036325	0.14060659	0.22751531	0.21251507	0.12901885	0.24905818
ADCY2	0.05704287	0.79983598	0.37007291	0.15807788	0.01020521	0.55213738	0.12503515	0.12864529	0.03453701
ADCY3	2.58792007	3.20162166	3.07436356	2.64184065	2.8569784	3.18472244	3.01286502	2.27056397	3.63303831
ADCY4	0.43373588	1.20415944	1.56190579	1.52072946	0.93253111	1.27558312	0.59442582	0.79696247	0.6876663
ADCY5	0.19559791	2.74301404	0.64864683	1.26341711	0.20487805	1.7074257	0.11116459	0.23201131	0.15927716
ADCY6	2.39653116	2.07165221	2.57552416	2.00538694	2.47159449	2.18636066	2.06935102	3.10479017	2.59676897
ADCY7	1.20143069	1.81835211	1.74487004	1.66317959	1.91243136	2.16223514	1.85303238	1.58402105	0.98109403
ADCY9	1.6580758	2.79463414	1.68710588	1.76477027	1.9307658	2.25232066	2.01991725	2.34015126	1.71621276
ADCYAP1	0.09273632	2.7589302	0.24739657	0.8129499	0.0747377	1.2032722	0.06172942	0.17369261	0.02495613
ADD1	3.73265453	4.37454626	4.0233311	4.00819601	3.82304025	4.2419795	3.67037875	4.01682845	4.11251009
ADD3	4.11670513	5.25583749	4.34339009	4.26880858	5.2763272	4.03316852	4.65126906	4.0694158	6.09890582
ADH1A	0.01605075	0.07212805	0.15465734	0.04762282	0	0.09189554	0.26412787	0.11934141	0.02523003
ADH1B	0.03858347	4.90857994	1.90792977	2.66360972	0.63100255	3.71776063	0.36966439	0.54857642	0.11563067
ADH1C	4.45835102	3.71374825	6.3217113	1.55147016	4.25024398	3.01721508	2.1867584	6.29362826	1.19220429
ADH4	0	2.48723035	0.88610122	0.07807241	0.39051984	0.52119171	0	0.17250465	0.75486376
ADH5	4.26079096	5.08753981	4.53978404	4.7551151	4.46795347	4.99699329	4.55034685	4.01621219	4.9283495
ADH6	2.01103683	3.2200661	1.85963311	0.62677688	0.43303692	0.1691955	0.55315931	0.29355349	3.1759558
ADH7	0.49200531	0	0.49676954	0.51020095	0	0	0.02090587	0.03129463	0
ADHFE1	0.12111353	0.84388677	0.86205745	0.64691911	0.51343508	0.52664257	0.15955432	0.1560601	0.09871419
ADI1	3.82677309	3.85218887	4.39345861	3.75469273	3.30427108	3.48949222	4.32625077	4.02770841	3.94270965
ADIPOQ	0	0.74364775	0.02826042	1.26871611	0	0.0028999	0	0.06548135	0.00414683
ADIPOR1	5.26058338	5.01955844	5.84837947	5.78589102	5.0936993	5.47805017	6.30811176	6.49560403	5.38802259
ADIPOR2	3.78823272	3.54613243	3.59471696	3.84074713	3.67898293	3.76730632	3.83356529	3.88068265	3.5709415
ADIRF	0	0	0	0	0	0	0	0	0
ADK	2.51419074	3.20351841	4.28179157	3.45077163	3.34058396	3.96067398	3.45595962	2.67950849	3.44411142
ADM	2.42446236	2.64612622	2.351261	3.55810281	2.00215017	3.35191338	4.16787879	2.73315916	3.67124809
ADM2	0.32657773	0.95661118	1.53996084	0.84313867	1.10682243	1.44431254	2.11833825	1.62985656	1.40203063
ADNP2	0.55732747	2.64152339	2.35061944	3.35188216	3.05205543	3.17188659	3.31066072	2.62172139	3.0868886
ADO	3.32374352	3.25662743	3.53381865	3.66392638	3.3085156	3.4992611	2.96424919	3.49070354	3.58820194
ADORA2A	0.14853754	0.08224802	0.16591072	0.06624695	0.13290446	0.08073168	0.07600715	0.08457237	0.03163202
ADPGK	3.32128964	2.88787328	4.20053234	3.57798526	3.87871025	3.67821875	3.36328703	2.87736994	3.41511865
ADPRH	1.0929399	2.12678252	1.97757064	1.75742439	1.76593634	2.23531952	1.55731851	1.22630742	0.73770537
ADPRHL1	2.36768971	1.35838172	0.64902977	0.98281753	0.64010279	0.71197386	1.30509134	1.32836478	2.08527473
ADPRHL2	4.25421849	2.86579417	4.12747601	3.26237609	3.08132943	4.06781406	3.39866859	2.69753429	3.86049423
ADPRM	1.37326005	2.52849734	2.53866426	3.084813	2.95659248	2.84785245	1.80417717	1.77487019	2.84896428
ADRA1B	0.08153018	0.12641995	0.68621336	0.39389004	0.07549828	0.27295264	0.42150854	0.13124035	0.01098045
ADRA1D	0.13697644	1.22211792	0.11774666	0.16286613	0.03183494	0.33664447	0.07614867	0.05434439	0.16831121
ADRA2A	0.32921228	4.33953163	2.48804569	2.45630497	0.06448337	1.83597849	0.49346767	5.83131936	4.11796292
ADRA2C	0.09701686	0.57667221	0.20259012	1.38607325	0.04063691	0.6078007	0.25618254	2.39213297	0.82197554
ADRB2	0.22497116	1.26525751	0.62852346	0.50404601	0.57790355	0.73627506	0.10388672	0.15826312	0.15794911
ADRM1	6.09268652	4.6203707	6.00248655	5.12920928	5.10671674	5.47027895	5.6538368	5.25051462	5.73712176

ADSL	3.61304888	3.44819379	3.6006509	3.70096246	3.5803379	3.72412981	3.30987806	2.8241107	3.97984139
ADSS	2.81199933	3.40910716	4.16881348	4.33385817	4.14705966	4.06067767	4.54653843	4.88368151	3.84961278
ADSSL1	0.61286507	1.13433611	0.32518226	0.89592567	0.33501783	0.60934167	0.56990109	1.35301113	0.11779392
ADTRP	0.15789236	2.39718566	3.88066178	0.67381011	2.63341684	0.16737191	1.3393658	2.28632674	3.24571482
AEBP1	5.44948428	6.73692644	5.21877205	7.01393772	3.51559193	6.40346732	6.01659687	5.1158431	4.16482254
AEBP2	2.99159138	2.32463303	2.62286502	3.16288081	2.89041507	2.60911185	2.22342517	2.86159719	2.46143909
AEN	2.56450005	2.09587051	3.02160784	2.69713804	2.76669759	2.71507231	2.64713976	3.38207756	2.82978832
AES	6.81045881	5.23959083	5.27913609	4.78951392	4.95198917	5.08073005	5.32060613	5.65730852	5.30230171
AFAP1	2.20462176	3.61050023	2.79516342	3.3178583	2.54471391	3.14251974	3.49307213	2.73064199	2.85011318
AFAP1L1	1.07196225	2.35769185	2.20256331	1.73303786	1.21936998	2.32105736	1.6163562	1.29511116	0.6999134
AFAP1L2	0.84334207	1.9153296	2.77039097	2.09687023	1.42376553	1.90863487	2.8175764	1.30153638	1.11756922
AFF1	2.71199256	3.67630554	3.00103517	3.31261833	3.50716589	3.67475209	3.22331901	3.58365562	3.35131025
AFF3	0.03672605	0.70431653	0.49988758	0.33137387	0.11514477	0.26351343	0.13714643	0.18985945	0.10161777
AFF4	2.64499587	4.13318914	3.98036463	4.10070342	4.48625228	4.24372909	3.75269294	4.55085402	3.67990801
AFG3L2	3.24802793	2.93330556	3.14247252	3.25590224	3.53288724	3.51714372	4.46109306	3.61353625	3.98075341
AFMID	3.1232213	1.89817061	3.1968439	2.6086664	1.78453217	1.80800318	2.34463081	3.44082417	3.64732827
AFTPH	3.02270486	2.65975531	3.15774353	2.95103602	3.33619489	2.98151048	3.11117869	3.32488757	3.0428811
AGA	2.01465893	3.24186334	3.26456764	2.83626478	2.85646371	2.22802862	2.82773842	3.05650828	2.48838292
AGAP1	2.93423658	2.15189807	1.67076144	1.84528283	1.77296028	2.18404577	2.56776397	1.86548726	2.48256619
AGAP2	0.99094764	0.59016008	0.99873806	0.65482868	1.46116435	1.48132902	1.22346648	0.46074975	0.79917568
AGAP3	2.90750585	2.04598329	2.34565245	2.41754891	1.99149053	2.48055713	2.34734435	1.95400582	2.6665187
AGBL5	3.54211521	2.26023979	2.46068888	2.31087064	2.20153586	2.48395946	2.93315688	2.95365353	2.77213456
AGER	0.95346662	0.46797838	1.40705879	1.71223074	1.2948893	0.83714525	0.5872322	0.7441869	1.10484696
AGFG1	3.92355969	3.46984838	3.74501441	3.78385779	4.18349742	3.70905899	3.99091449	4.06922759	3.8572033
AGFG2	4.14402569	1.93497503	3.35782237	1.43228928	2.55596922	1.5416355	1.59749767	2.70890431	2.22413666
AGGF1	2.08945289	3.39067814	3.78338379	3.8519573	3.47484145	3.54394215	3.01602916	3.61669807	3.7101302
AGK	0.73436517	2.24341409	2.83051515	2.7010196	2.62137624	2.04128533	2.44158327	2.11971705	2.51846398
AGL	1.50845819	2.56195567	2.09267423	2.78349465	2.86815669	3.11777535	2.96955877	2.77306542	2.75078229
AGMAT	3.36122158	3.1369093	2.2419715	2.21332222	3.53463429	2.8898277	3.34021014	2.33860093	4.16665913
AGO1	2.42695565	2.49279089	2.4296815	2.19467303	2.40713877	2.61383863	2.41006601	2.0664462	2.71852563
AGO2	2.59726591	2.21642465	2.71078419	3.04758923	3.17163369	3.01830278	3.51469	3.14432944	2.64675934
AGO3	0.79172219	1.27057258	1.4522421	1.34008414	1.32743156	1.32148186	0.73378538	0.82113932	0.95122644
AGO4	1.30882413	2.12666949	2.39489911	2.50907667	2.53955657	2.84178945	1.89034385	1.93064372	2.90784206
AGPAT1	4.37466636	4.37394066	4.67176472	4.28071171	3.86061375	4.21522764	3.98081537	5.10502071	4.40083627
AGPAT2	8.30242755	5.61438144	5.63965075	5.09178161	5.01722225	5.27194792	5.54358831	5.23725271	5.96790977
AGPAT3	2.80090976	2.97022075	2.66112414	2.90596701	3.20141364	3.4217485	3.1808274	3.36653534	3.60018954
AGPAT4	0.81601049	1.29499084	0.70910243	1.3968676	0.48288138	1.2278279	0.87820661	0.95720797	0.29885384
AGPAT5	2.38335263	2.96449907	2.89845987	4.15744299	4.12894673	3.87157238	4.30711752	2.88714836	4.08938344
AGPS	2.50621925	3.68768096	2.78711008	3.28802806	3.70261804	3.91071269	3.52108866	3.29668019	3.57451489
AGR2	8.85010463	6.45629956	9.27153764	8.57191358	8.11896049	5.48117542	10.9172402	8.84412256	8.47690694
AGR3	3.91134539	5.45527076	5.74194653	6.27730596	3.05977691	1.46483979	6.29676259	2.27625465	4.51137319
AGRN	5.95258006	3.50838261	3.7523044	4.15448936	4.10302295	4.22761279	5.62628109	4.2664501	3.89777467
AGT	0.7229589	8.07216962	4.26922048	2.46423121	0.4218456	3.78430277	4.95386169	2.58851774	3.22616103
AGTR1	0.05146461	2.31658244	0.17112848	1.03576055	0.02559301	1.70267947	0.0852477	0.0632139	0.02186546
AGTRAP	5.18329946	3.83306057	3.42943493	3.49758914	2.45253381	4.74816551	3.59887041	3.2637543	3.11374517
AGXT	0.04029864	0.2448512	0.2686134	0.06237919	0	0.02972245	0.07158356	0.05561712	0.07124708
AHCTF1	1.95557932	2.56221044	2.52932378	3.05166514	3.48771527	3.19660703	3.55691042	4.07227567	3.04210379
AHCY	5.97905147	5.09600737	6.02525754	5.78409448	5.7496835	5.38666609	5.70012218	5.60115236	6.75719075
AHCYL1	4.83413256	5.1390427	4.89681666	4.66379285	4.78206575	5.06529301	4.98475335	4.90998362	5.53138949
AHCYL2	3.39936544	4.04564421	3.66055144	3.78521948	4.91519106	4.06885906	5.06767398	5.65751041	2.92830259
AHDC1	2.45765932	3.04688569	2.18979495	2.25124036	1.57153015	2.63056511	2.29350943	2.02228125	2.01068374
AHI1	0.87181673	1.73572792	1.74607525	1.92387135	2.15739082	1.54349091	1.55770964	1.35882029	1.73454335
AHNAK	3.65814471	5.79712258	4.22237998	4.98252423	5.44193393	5.53313323	5.53797247	5.41051094	4.57174779
AHR	3.0780363	5.78800536	4.83527751	4.88545508	5.52019095	4.14696095	5.13957335	5.19922134	3.42305864
AHRR	0.1991936	0.57895109	0.70899072	0.13838325	0.23181442	0.0879535	0.23783832	0.11932947	0.18913905
AHSA1	5.05211114	4.55549195	4.99709814	5.19991713	5.45811994	5.22834844	5.08302572	5.16868309	5.31010025
AHSP	0	0.27020247	0.69333259	0.03839097	0.07776515	0.06976623	0.1119947	0.16513566	0
AIF1	5.06897312	5.73269023	5.99978468	5.10583131	6.22188646	5.64611195	4.72809737	3.82166131	3.65212598
AIF1L	0.18076319	2.14008898	2.65705931	2.20282018	0.33500155	1.873492	1.01755304	0.39417934	0.71280859

AIFM1	4.03535704	3.86562293	5.33212032	3.79037848	5.25858569	4.01717399	4.0509062	4.09821325	4.5785743
AIG1	2.11572482	3.0906438	3.04819064	2.89906004	2.24347702	3.1990013	2.29680233	2.06579777	2.88424909
AIM2	1.02225469	1.13935359	2.04122991	1.2189334	6.1035947	1.2005301	1.1354027	0.67016766	1.22349691
AIMP2	4.58459819	3.54441243	4.24206597	3.87658069	3.99955156	3.90089048	4.32170327	3.87188824	4.37565191
AIP	5.17779717	4.10130757	4.79224378	4.25160367	4.32720773	4.11691034	4.32418832	5.17812851	4.37881646
AIRE	0.03843697	0.03526555	0.06600859	0.00466653	0.01432688	0.0142419	0.0231314	0.00898008	0.01223325
AJUBA	2.85691812	1.62013669	2.55528261	2.24764033	3.39700379	2.27194618	1.88796859	2.39147618	2.28091119
AK2	4.88907032	3.80859465	4.04610473	3.99040317	4.77548643	4.30578032	3.6035813	3.34266014	4.74584413
AK3	3.69634036	4.95006349	4.20416828	4.40415047	4.81523426	4.76362419	4.12661783	5.19705432	5.32054665
AK4	2.43036541	2.82719024	1.21399268	2.19092449	0.40988384	3.07696389	1.80832045	3.58766201	3.12003667
AK9	0.35353087	0.51445141	0.65345495	0.50156096	0.6552061	0.51618596	0.42538289	0.18756895	0.97948238
AKAP1	4.71617429	3.86380498	4.47588973	4.4441004	4.19743674	4.01952227	4.14008137	5.06306797	5.12569958
AKAP10	1.56268535	2.73918367	3.06670206	2.73451061	3.09321939	2.97765682	2.00136143	2.86593472	2.62874081
AKAP11	2.06460035	3.59871954	3.04063016	3.33754365	4.05664893	3.40406101	3.07836723	3.32408695	3.68372533
AKAP12	0.82036857	3.75382116	2.00313955	3.77289057	1.36592657	5.47859483	1.4814366	1.62847253	0.58036579
AKAP13	3.04237384	3.46155323	3.78449841	3.37674191	3.56689402	3.43641647	2.87568774	4.17190526	2.41358124
AKAP14	0.03081803	0.06966052	0.24754603	0	0.48443074	0.13119071	0.01852683	0.01195597	0.69973191
AKAP17A	3.61926457	1.97824667	2.62908064	2.66297886	3.14098422	2.93349125	1.86037104	2.98495452	3.52179716
AKAP2	0.17854106	0.63255194	0.17335203	0.31594102	0.22435144	0.50738114	0.12485756	0.13466031	0.15466023
AKAP6	0.02922991	1.19278472	0.34571853	0.3946908	0.06773611	0.47909314	0.09614918	0.21380381	0.24477653
AKAP7	3.63099332	1.41662498	1.42786856	2.71676154	0.99398692	1.64874183	1.78817097	0.6214751	1.05996725
AKAP8	2.84402083	2.09555768	3.15272149	2.96771897	3.29318009	2.40820646	2.66123042	2.93964474	3.22854835
AKAP8L	2.70897884	1.94191273	2.53844018	2.60897277	2.65786249	2.55069436	2.31243494	2.57743775	2.70705576
AKAP9	2.04504071	2.37086422	2.1047899	2.00493454	2.61934452	2.16833653	2.39210357	2.75337081	2.57317602
AKIRIN1	4.2967273	4.32795912	5.21445154	4.56769869	4.26534609	4.77410232	4.5632403	4.13771857	4.1554056
AKIRIN2	4.21750928	3.9587975	3.96925128	4.64800805	4.68293247	4.61630047	4.2556747	3.34989328	4.57393377
AKNA	2.85760528	2.6774028	2.82423802	1.94138845	3.27154853	2.58481914	1.74879513	1.98219733	2.75767967
AKNAD1	0	0.16440667	0.39574998	0.13171028	0.0136393	0.02163266	0	0.04501681	0.06105179
AKR1A1	5.97303104	5.23119634	6.1843182	4.80985293	5.31536796	5.18641814	5.13977407	5.31329738	5.78623424
AKR1B1	4.68345785	3.82146044	4.13832922	4.19873349	4.10327598	3.81278059	3.67760322	3.37535105	2.71726382
AKR1B10	4.28734146	2.70431311	4.27231382	2.71026595	5.77513377	2.38269428	2.01558277	2.558394	1.08354104
AKR1C2	3.68145024	1.80281452	0.75138827	1.03501423	0.05584645	0.34170069	0.21994148	0.19749612	0.46916688
AKR1C3	5.75480552	5.05238081	4.21299375	2.79754519	2.63384937	2.28500872	3.32910213	3.59184124	3.26123381
AKR1C4	0.07422258	1.44955918	1.07457347	0.12048858	0.01168281	0.04816045	0.22146055	0.47684103	1.19121487
AKR1E2	0.05908768	0.77603577	1.20558883	0.3585928	0.32320741	0.42826759	0.22518099	0.36741207	0.45215699
AKR7A2	4.67779429	5.07404349	3.81408368	4.33127387	3.6855092	4.36643497	4.45255888	4.33741136	4.68915748
AKR7A3	2.47250459	4.34832751	3.26818981	2.89253735	2.92390159	3.87774925	3.96102493	6.4041695	5.47020621
AKT1	4.31763939	3.25893802	3.0268277	2.93965829	3.20481961	3.44366556	3.75882933	3.98336012	3.45034269
AKT1S1	4.37495553	3.09361898	3.38650147	3.04699837	3.72912351	3.34809643	3.208659	3.4269407	3.53410866
AKT2	3.69763434	2.575613	2.71172812	2.5260626	2.84198665	2.82328624	2.39215146	2.65352465	2.87218831
AKTIP	1.35274151	2.28780179	2.24486956	2.50638859	2.34243264	2.15057816	2.42780287	1.71437838	2.18201527
ALAD	3.4847733	3.26800743	4.08263558	3.32934975	2.71143232	3.1416151	2.52261852	3.36521014	3.18431383
ALB	0	0.01231075	0.82605286	0.1000461	0.59600401	0.2303953	0	1.31146524	0.00566161
ALCAM	1.25058792	2.15075611	3.68361946	3.06377294	2.21132068	3.29859357	4.13391454	4.63434517	3.76373427
ALDH16A1	3.69405532	2.08551708	2.47704861	2.03484027	3.40820333	2.63821199	2.7882739	2.72545067	2.53791373
ALDH18A1	4.87779291	5.20547387	4.4898312	5.42460005	4.87558234	4.97951645	5.52607997	5.2985125	5.80927013
ALDH1A1	6.19155638	5.51843593	7.45263008	3.02675028	5.65383746	4.56250547	2.02427951	2.69787509	2.80221214
ALDH1A2	0.04166233	0.24031582	0.24643753	0.23146198	0.13891593	0.3352473	0.10959308	0.07305979	0.01766623
ALDH1A3	0.79515886	2.96184948	1.93850636	2.59427207	0.61715027	2.19063763	1.32109587	1.74405386	0.53694654
ALDH1B1	4.11865109	6.21863933	3.39481459	4.88373726	3.52950278	5.70147717	4.03327784	3.65431136	6.40956986
ALDH1L2	0.51059906	2.54197649	1.2519411	2.47160268	0.98054917	1.89912012	3.21963128	0.689133	0.54950614
ALDH2	3.14971401	3.8003399	3.9171159	3.12223784	3.86831314	3.96335131	3.77916344	4.9406343	4.32131714
ALDH3A1	1.29699128	1.98754845	2.75050504	1.12677015	2.14530842	1.95455456	1.9686361	4.94941651	0.2465912
ALDH3A2	2.42808242	3.83115552	3.40812966	3.42378244	4.12030263	3.9816056	3.72900691	4.08619075	3.68868482
ALDH3B1	1.81247655	2.98399527	2.4988068	2.44353419	1.60119819	2.39463638	3.39242354	3.6998699	2.06223875
ALDH4A1	2.27957238	1.85450195	1.12592075	1.98927652	1.77283252	1.99149704	2.32687686	1.56993635	1.16929725
ALDH5A1	0.49255149	1.49311247	2.95332185	2.67773891	2.89620003	2.81717795	2.2209736	3.27617466	2.84571518
ALDH7A1	0.40634182	2.78882124	2.89650775	2.71064234	2.18193145	2.23391771	3.839181	2.77598381	2.92195549
ALDH9A1	4.35727921	4.92968713	4.3374353	4.5140985	4.59004674	4.80536211	4.73653077	5.33086799	4.75762212

ALDOB	0.05828435	6.44204096	5.50538397	0.93747051	5.21196394	0.5253495	0.49913576	5.83397082	3.17886564
ALDOC	4.97405557	2.42189854	1.91361321	4.29111599	1.1794917	3.11097713	1.65700847	2.41489795	4.50000184
ALG1	2.62508663	2.96296413	2.30497412	2.03489471	1.89151716	2.62463851	3.6024432	2.788196	2.64412166
ALG11	1.08607821	1.09900128	1.36473401	0.90011954	1.2701236	1.0864626	1.551099	1.49676872	1.31632145
ALG13	2.09116289	1.92007471	2.64878368	2.59161898	3.79063283	2.13749785	1.86199804	2.5076606	2.36182754
ALG14	1.22654183	0.97185039	1.60429881	1.1562496	1.23003642	1.13217703	0.94574091	1.34445527	1.50879842
ALG2	3.60526133	3.61001044	4.38010743	4.07615039	4.06069794	3.99866372	3.40410005	3.58411963	3.97050727
ALG3	5.53504345	3.75826682	5.60107606	4.11762123	4.07273847	4.15578774	4.6765467	5.39656349	4.46336529
ALG5	4.03511555	4.1466764	4.12616144	4.4082509	3.89954336	4.13024436	4.72237361	4.81278709	4.83723028
ALG8	2.94692937	3.52407604	3.72542282	3.41314245	3.82294855	3.35443691	3.99714034	4.12782833	3.65616837
ALG9	0.88846073	1.27785239	0.93951574	1.10051115	1.17039552	1.05306541	1.30236208	0.63605399	1.15998695
ALK	0.04655807	0.02685195	0.20172189	0.02811269	0.06646113	0.07710312	0.00628065	0.01210712	0.00551651
ALKBH1	2.25013741	2.49051409	2.79409342	2.26452617	2.75628132	2.28990318	1.74880029	2.53427786	2.78594515
ALKBH2	2.41557177	2.09954398	2.72827235	2.81608987	3.09823307	2.98822153	2.57351017	3.22370293	2.53777629
ALKBH3	2.68515153	2.58989266	3.45064516	2.84114303	2.4630623	3.11269891	3.69596593	2.5468467	3.45181513
ALKBH4	3.1852998	1.58628571	2.00839871	1.55600093	1.58130648	1.85819642	1.81748531	2.02800716	1.72631016
ALKBH5	4.2206094	4.04237156	4.18297906	4.41512187	4.02885822	4.0496934	4.23666672	3.5038255	4.70966064
ALKBH7	5.97289368	4.31940132	4.27246363	3.66885554	3.11761991	4.14052864	4.5204233	4.63317708	4.93354857
ALKBH8	0.95325949	1.22198125	1.1261051	1.662703	1.7479753	1.64421429	1.62167446	1.23956711	1.51574341
ALMS1	1.28781238	1.66231144	1.21616408	1.30380332	2.1402354	1.93558566	1.65131541	1.18084787	1.60937865
ALOX12B	0.37278154	0.01563458	0.05828719	1.05553538	1.12281873	0.240889	0.02443368	0.15528275	0.04263959
ALOX5	3.11207332	3.52955731	3.69144303	3.45778641	2.43452036	3.77828201	3.31019952	2.58590468	2.59049331
ALOX5AP	4.11932403	4.92217188	6.15625155	5.79440106	5.5424782	6.14136376	5.60381961	4.73880076	3.99836371
ALPK1	1.18057628	1.70018964	1.72748519	1.44378412	2.16143127	2.08730904	0.90956288	1.70875729	1.30800392
ALPK2	0.3903548	0.7846301	0.22209365	0.82748509	0.32096828	0.49308099	0.31279968	0.10620903	0.16670617
ALPL	0.7984948	2.54382161	1.60374532	3.15348281	1.16150828	1.61443696	1.97520163	0.72034877	0.99535361
ALS2	1.61951013	1.66178304	1.99116216	2.05554123	2.24419088	2.01753702	1.94209095	2.0142848	2.27693376
ALS2CL	0.97866369	0.74347794	1.37626178	2.27762845	1.42433013	1.94293878	1.95049851	2.47569305	1.59818466
ALYREF	6.34310039	3.44637987	4.75220076	4.44232554	4.26616607	4.79678158	4.74029198	4.66550866	4.95650109
AMACR	1.19187489	1.61104779	1.66063553	1.00627901	0.44729909	1.02301118	1.3629993	0.82929477	2.03998253
AMBRA1	2.82727367	2.40795377	2.79032172	2.35901478	2.55776348	2.58775532	2.59579094	2.95312975	2.78828745
AMD1	3.62446898	3.40233032	3.83896983	4.21076383	4.94007844	4.02375494	3.97585507	3.40840373	4.61629626
AMDHD1	0.0401418	0.26222219	0.47932806	0.5707156	0.06536675	0.36767304	0.17566751	0.69586092	0.31866947
AMDHD2	1.74128764	1.95484541	1.49486389	1.26240553	1.24194871	1.90724821	1.37667486	1.69453861	1.6173514
AMER1	1.50442077	1.34719122	1.08974941	1.35913617	1.95983342	1.24475193	1.18145992	1.16329102	1.50912657
AMFR	4.03516834	3.95723803	3.94778336	3.81035445	3.75692102	3.99747204	4.4140759	4.17578891	3.78116003
AMHR2	0	0.08077145	0.259587	0.01852638	0.0190199	0.01890727	0	0.0625105	0.01084985
AMIGO1	0.06239557	1.69309382	1.60241959	1.384957	0.23323691	1.35251623	1.19753071	1.8812484	1.43768485
AMIGO3	0.02524599	0.0072757	0.00917069	0.02273951	0	0.01396854	0.00760263	0.02433348	0.01332529
AMMECR1	1.49480203	1.84993782	2.27083509	2.706302	3.18529828	2.19388996	2.19415971	2.26880532	2.66983591
AMMECR1L	2.19749922	2.39534645	3.28959843	2.86029605	2.45368322	2.88416291	2.90964925	3.04038792	2.70104464
AMN	4.3182453	3.38963383	2.06550644	0.88608737	1.79855479	2.80804675	4.0158853	4.82827356	3.6353461
AMN1	1.67003962	1.1106323	1.46732396	1.53478579	1.26471672	1.44196264	0.950389	1.89580968	1.11349176
AMOT	3.29989477	1.78336574	1.80946215	0.70580169	2.81303326	1.12514304	1.31229802	1.5803061	1.48017874
AMPD1	0.01452795	0.13560438	0.91995667	0.29238952	0.32123091	0.22781195	0.02598592	0.19941164	0.28465196
AMPD2	2.44928026	2.84991141	2.9262992	2.75223735	2.33063915	3.16012245	2.89283207	3.23233084	3.30596439
AMPH	0.11791036	1.52316292	0.90885832	1.01184599	0.34551099	1.3437897	0.60456167	0.32249597	0.11745107
AMT	0.52361446	1.25866286	0.92320703	0.8352879	0.67237555	0.50715966	1.98454954	0.56589703	0.89665833
AMY2B	0.13017143	0.25011314	0.63962332	0.66605917	0.42075438	0.42436107	0.03453123	0.5903383	0.20025055
AMZ2	3.64775529	3.56103611	4.14144798	3.61259603	3.37062436	3.43404772	2.92107033	3.82967357	3.50996723
ANAPC1	1.18630494	1.84117483	1.78542679	1.50937541	2.34815309	2.13959678	2.16679921	1.91469441	2.53913949
ANAPC10	2.10060831	1.85281022	2.22618833	2.40388237	2.26357363	2.05500903	1.58695653	1.82644086	2.36648031
ANAPC11	5.17284076	3.24433929	4.06326241	2.85073171	3.28070357	3.66355927	3.33930198	4.12419865	3.47246655
ANAPC13	5.30954923	5.21565482	5.13828124	5.22233282	5.00620037	4.96774567	5.31660256	4.92399024	4.80773138
ANAPC15	2.6004153	1.55697771	1.67084008	1.68872132	2.10914212	1.72189808	2.30884303	1.6850076	2.48216912
ANAPC16	4.56698767	4.00349242	4.29967109	4.159742	3.84684287	3.67536818	3.94956822	3.84006868	4.01142145
ANAPC2	3.38181911	1.8116276	2.86440361	2.39503685	2.51381021	2.19216141	2.06879989	2.64216102	2.71809661
ANAPC4	1.35845808	1.8698465	2.34045607	2.36909159	2.63562316	2.00215258	1.77799785	1.84528815	2.28595915
ANAPC5	2.66312464	2.88568251	3.63183371	3.35348728	3.53027526	3.04126321	3.18340213	3.72416643	3.42981533

ANAPC7	2.46886888	2.48153164	3.20599012	2.93518166	2.86861903	2.64889101	2.5557051	3.15416069	3.18392358
ANG	4.09833857	2.63015991	4.1357587	3.66081422	3.036393	2.45892508	3.60488725	4.58780184	3.05446948
ANGEL1	2.80295553	2.4139286	2.81982812	2.4467284	2.69459994	2.71301003	2.2016638	2.00530027	2.95169048
ANGEL2	1.14709354	1.93688281	2.35458749	2.21270024	2.19704223	2.37256981	2.11718792	2.31285413	2.10311688
ANGPT2	1.29524127	1.70849412	2.85415696	1.85224668	2.57457886	1.78870023	1.79991062	1.66122025	1.92776533
ANGPTL2	3.44618018	5.91375083	5.61633317	5.86376899	3.50997665	6.273762	4.9550387	3.2802013	3.20665178
ANK2	0.0986105	2.54386861	0.63055506	1.44809039	0.23811042	1.58685621	0.24710399	0.25005561	0.14403518
ANKAR	0.1615078	0.67862043	0.73055715	0.62501111	0.61837061	0.45327146	0.52727671	0.61761218	0.4748048
ANKDD1A	0.22693854	0.84163855	1.0681726	0.98982642	0.94094683	1.00765807	0.38506138	0.42831233	0.3342527
ANKEF1	1.37052451	1.1947031	2.53263639	2.70607916	2.35653943	1.94215012	1.99470854	1.7005871	2.73914778
ANKFY1	1.57469043	3.12002769	2.87855224	2.77607017	3.44355964	3.50193371	2.68667636	2.4192578	3.13544988
ANKH	1.4302666	3.75048724	3.2419885	3.06920874	1.95034492	2.87704356	3.33959261	2.42983319	3.26562822
ANKMY1	1.03090735	0.4307919	0.85043715	0.91640584	0.99265852	0.87419589	1.12119943	1.23508163	0.94615425
ANKMY2	3.15735887	3.39318574	4.075447	3.4133677	3.98125621	3.57107685	3.75931178	3.44717499	3.37762135
ANKRA2	1.74679174	2.36517021	4.07218135	2.80422866	2.92078418	2.41843347	1.86570879	2.7093128	2.93390238
ANKRD1	0.17331798	0	1.21335831	0.14693823	0.44544219	0.27066635	0.05409231	1.04796127	0.05694167
ANKRD10	3.6889987	3.85951016	4.29972862	4.53878354	3.85175363	3.90207899	3.35092127	3.91911674	4.6975374
ANKRD11	3.08998525	2.57762675	2.60108043	2.43163028	3.14050777	2.83348646	2.68679667	2.69962868	2.91758705
ANKRD12	2.2138624	2.98671993	2.86692185	2.68159634	3.00343065	2.43678871	2.48351368	2.92565661	2.44597183
ANKRD13A	2.65452018	3.00685008	4.37437785	3.1158134	3.38186467	3.25176465	2.81060925	3.00135851	3.29569568
ANKRD13B	2.07252506	1.52983856	0.96448585	2.07505976	0.39343742	2.34844486	1.61719401	0.73505104	2.49773402
ANKRD13C	0.67739039	2.568376	2.7311883	2.93224643	2.46059451	2.70678225	2.44956104	3.08690038	2.55388989
ANKRD13D	2.18883819	1.37057791	2.62346156	2.22722325	1.89977344	2.11655126	2.01641103	1.77019826	1.83210155
ANKRD16	1.33785966	1.42182653	1.51975523	1.34531663	0.93789392	1.26047671	1.56200408	1.19073665	1.93414592
ANKRD17	2.97074326	3.50054108	3.4271518	3.61769412	4.05881094	3.76113496	3.37608659	4.08146301	4.00108696
ANKRD23	0.0263218	0.10276019	0.23011543	0.08258695	0.1792978	0.10657779	0.07739158	0.14901566	0.10758832
ANKRD26	0.73264668	1.28227923	0.91619836	1.0842872	1.6308362	0.96193914	0.49119606	0.83270245	1.07973527
ANKRD27	2.57742361	2.29542577	2.71164985	2.53857434	3.03765686	2.65147261	2.35661749	2.68794268	2.65176407
ANKRD29	0.17688339	1.28284178	1.6157248	1.13435831	2.10579334	1.01401493	0.33914244	0.81324121	0.30667795
ANKRD30A	0.00724895	0.01243418	0.18686814	0.00435342	0.01336858	0.01328926	0.00434364	0	0
ANKRD34A	0.43336562	0.56569432	0.51284123	0.3377638	0.84112587	0.31304804	0.25494404	0.17600056	0.07610546
ANKRD36	0.37608076	0.29037603	0.36175634	0.56520496	0.60389737	0.50435978	0.24431085	0.42149314	0.66320327
ANKRD37	1.04771903	0.79935252	0.43690224	1.46587817	0.47723573	1.62264712	1.05143746	1.40758039	1.31761924
ANKRD40	3.88248039	3.42292913	3.69110675	3.67725963	3.71918275	3.69786925	2.98152706	3.53001857	3.43853341
ANKRD44	0.5893654	1.62478485	1.6687716	1.44510375	2.45801291	1.73921496	1.17635039	0.94659092	1.07012605
ANKRD46	0.70046841	1.64074696	2.28937775	2.54815322	1.43329287	1.56637816	1.73804439	2.6758463	1.85930228
ANKRD49	1.49923386	1.82718509	2.71348535	2.45726744	2.72650406	1.87960808	1.71626918	2.14535826	2.93981753
ANKRD50	2.81991436	3.28959184	2.73451066	2.80008589	2.52349788	3.04309567	2.76482141	2.49657259	1.93036895
ANKRD54	3.28775001	1.95837431	2.16667377	2.01554849	1.98114577	1.97799639	1.98020881	1.62945435	2.33581449
ANKRD6	0.19225761	0.93906793	0.89958583	0.8130952	0.88618024	0.80273965	0.38607145	0.75676167	0.12125907
ANKRD9	2.41821326	2.28708744	1.53337708	1.83723664	1.43162886	2.24987956	2.12907723	3.17482974	2.55102404
ANKS1A	2.94176889	3.38427345	2.84840549	2.69330567	3.02775638	3.5653032	3.53118795	3.67898005	3.42885356
ANKS1B	0.01639297	0.33251785	0.28054674	0.07515874	0.0361073	0.22294026	0.06747923	0.0425742	0.01379923
ANKS3	0.88567012	0.63728791	0.6703861	0.93442367	0.68457014	0.72246973	0.7137008	0.76167247	0.94726391
ANKS4B	4.03252752	2.85084052	2.13921416	1.99314341	2.66177022	1.09686146	0.75233099	1.64505308	3.5943858
ANKS6	1.7662316	1.96319191	2.53991934	2.78577456	2.61664723	2.17739051	2.61842636	1.56489229	2.0188717
ANKZF1	1.96736304	1.39727748	2.23675564	2.26363002	2.14427754	1.59694566	1.9621234	2.05826012	3.23901165
ANLN	2.97714823	2.39640906	3.01422251	4.12966377	4.43206917	3.74403171	4.15844635	3.31365507	3.36157259
ANO1	0.79080109	2.2786951	3.09347124	4.62889023	4.78653721	3.54729135	4.46493911	4.26352833	2.10703385
ANO10	2.42800883	3.29747236	2.92996234	2.45733727	2.12579231	2.6879657	3.18224285	2.525721	3.01883705
ANO2	0.084853	0.30022116	0.30266583	0.10123353	0.11383828	0.09726399	0.08466151	0.10604289	0.23413034
ANO5	0.00527223	1.06238929	0.5566144	0.97237731	0.10053779	1.75686121	1.61459912	1.74330696	0.02749975
ANO6	3.1081894	4.09522495	3.86952655	4.44799364	4.32735762	4.51569368	4.19446786	3.67053454	3.86352618
ANO7	0.09892124	0.32663005	0.74793871	0.60307155	0.18476446	0.70840226	0.88935255	0.82181304	0.29415411
ANO8	2.01222719	1.0753656	1.38561331	1.85996475	1.96714085	1.14880831	1.89931872	2.14855512	2.1102378
ANO9	2.03606222	1.95223406	1.7489276	2.21128499	1.96193414	1.67320104	2.40598816	1.82101913	4.40520051
ANP32A	4.41362892	3.17272997	3.68715225	3.64843207	4.06520369	3.71452255	3.49940624	3.18625587	3.81715706
ANP32B	6.92956899	5.42527464	6.5173086	6.29205842	6.64205718	6.25280302	6.18651463	5.63800523	6.32069595
ANPEP	2.18666244	7.83110263	5.69902183	3.08617097	2.72260687	4.33872386	3.23323896	2.59337487	4.64840996

ANTXR1	3.43737303	5.43385988	4.59591429	6.08279748	3.40751311	5.2543249	5.5116815	4.6449611	2.84840572
ANTXR2	1.08697383	4.01848217	2.46807654	3.53646343	1.93926626	3.95019464	3.24546406	3.98823693	3.46391785
ANXA1	4.14875198	5.16071369	4.91449786	6.24480825	5.21584218	5.3567055	5.327648	4.01821914	2.82410246
ANXA10	0	2.13286967	5.17317189	5.09581788	6.03624546	2.7575916	4.20556255	8.00441086	0.5109915
ANXA11	6.14192082	4.73867993	4.67181742	5.11210992	4.62798336	5.69822725	5.09500081	5.79521525	5.08397175
ANXA13	0.05633943	2.63954773	1.33895365	2.75917262	0.9684965	0.16598974	0.65272405	0.89891198	2.43373597
ANXA2	6.87578493	6.24370787	6.63965759	8.19268533	7.40476147	7.31705537	7.12215975	6.68512672	7.012418
ANXA2R	0.48781989	0.69472747	1.00696214	0.79117397	1.02648342	1.1889302	0.65693848	0.53085786	0.50888804
ANXA3	3.63920196	3.3427987	3.87997167	4.9800406	5.38573918	4.61282068	3.64419892	5.05197767	4.05970498
ANXA4	6.58903217	4.45249559	5.51863155	5.15663745	5.8942555	4.16725628	4.70186211	5.87390888	5.46352708
ANXA5	6.32596008	7.33483101	7.7058603	8.01747715	7.13649683	7.31030369	7.46751771	7.87156472	6.02058522
ANXA6	3.78180181	5.47002743	4.60757367	4.33805779	3.93470338	5.02404043	3.90919439	4.94230491	4.54344832
ANXA8L1	0.10757324	0.04297176	0.12297468	0.10284635	0.04313249	0.01853306	0.00606501	0.02905483	0
ANXA9	1.59446785	2.85212389	2.74997664	2.09150507	0.91559977	0.86667957	1.45369751	2.1381146	3.15336583
AOAH	2.71284658	3.71370438	3.19178261	2.82103332	3.99472418	3.75006741	2.92193821	1.83424698	1.4077291
AOC1	4.28028946	6.8174919	3.71135001	3.91739218	2.51313118	1.60583956	3.37792504	0.84962171	4.83980431
AOC2	0.26262539	0.24508464	0.29475112	0.5591923	0.70195683	0.34367505	0.05636042	0.19271491	0.04960689
AOC3	2.14864786	5.34931597	2.76235238	4.01159523	1.60564977	4.36970454	2.25108232	1.6884851	1.34702778
AP1B1	5.47324797	4.50157203	4.58887397	3.95185833	4.94399886	4.66884937	4.77436498	4.01294191	4.8445136
AP1G1	2.33672287	3.22656714	3.71137854	3.69481678	4.12722696	3.80390135	4.05667242	4.11025739	3.71382395
AP1G2	2.62824566	1.86282147	2.90261883	2.87312435	2.77722603	2.35217211	1.83222742	3.27717369	3.26131604
AP1M1	2.42538255	2.25208914	2.12821151	2.03099518	2.18234523	2.45682075	1.71841106	1.96186039	1.95125436
AP1M2	6.15276412	4.30812409	4.93125612	5.09334956	5.8986296	4.92463126	5.49598931	5.58963969	6.05304414
AP1S1	7.20728125	4.74261301	5.02380779	4.22698746	4.01325899	4.27982392	5.30583222	4.87553638	5.24879164
AP1S2	2.08009479	3.52134129	2.46575965	3.03807198	2.85889631	3.1999332	2.00995273	1.54550786	2.13389616
AP2A1	3.64636149	3.62348816	3.34464491	3.41073531	3.72491756	3.71748952	3.49864639	3.76915022	3.41913143
AP2A2	3.41996268	3.205913	2.85248055	2.83288027	3.26784971	3.22771184	4.03447746	4.77539784	2.89760427
AP2B1	5.17701245	5.32447933	5.04368226	6.10515522	5.8644025	5.92055343	5.85662433	6.33800982	5.54876209
AP2M1	7.10246709	5.99098745	6.18992006	5.80173934	5.49448984	6.19954503	6.59279241	5.84394864	5.59022849
AP2S1	5.87160895	5.20300743	5.24158479	5.22170279	5.16833766	5.25520934	5.19531642	5.01169128	5.14901614
AP3B1	3.07315105	3.31629607	3.94796852	3.43081536	3.9078849	3.35199932	3.67265029	3.86542166	3.79160492
AP3B2	0.0041048	0.30833932	0.12508752	0.29555355	0.03502408	0.22342559	0.03404431	0.02045057	0.00861704
AP3D1	4.76733129	3.67084836	4.18924946	3.92038273	4.50976484	4.36370162	4.33054692	4.88238941	4.52092019
AP3M1	3.28618465	3.76111034	4.6001952	4.27408104	4.41726596	3.99386394	3.86763521	4.00842221	4.29883679
AP3M2	1.6752272	1.79079937	1.80891722	3.13950264	2.6351124	2.15630778	1.98067546	2.40271511	1.82416731
AP3S1	4.15457929	4.27407746	3.90953686	4.67599636	4.21279382	4.5360598	4.65511425	3.04687878	3.95141613
AP3S2	1.51447921	1.70854577	1.92139078	1.51964821	1.93814109	1.67380816	1.44130926	2.06789799	2.16912955
AP4B1	2.13963252	1.59517451	2.9301247	1.91858211	2.43295914	1.85837553	1.62226539	2.05120094	2.58989618
AP4E1	0.94366931	1.46251946	1.66788215	1.72177081	1.98726801	1.7038467	1.53643002	0.95460337	0.99582339
AP4M1	2.581594	1.67678338	1.92827811	1.58168178	1.41733786	1.60049194	1.92923053	1.90553296	2.28040177
AP4S1	0.94359409	1.11364821	0.91793753	0.96850963	0.86293062	0.65833573	0.42132557	0.843142	0.66244361
AP5B1	3.59383079	2.15276333	2.39925323	2.38790102	2.88719535	3.90640414	2.91414433	2.76727917	2.15202634
AP5Z1	3.08837474	1.59004338	2.76766304	1.76238065	2.23121294	2.13366576	1.99878794	1.98059259	2.35912007
APAF1	2.12873579	2.22641133	2.70343348	2.08239657	3.02983563	2.60235993	2.78682696	2.88748704	2.69352069
APBA1	0.12562632	0.49626436	0.73718855	0.46685476	0.33781105	0.54581697	0.72324076	0.27751639	0.29840528
APBA2	0.76134854	0.95726656	0.73486288	1.08764976	0.66404712	0.8630321	1.02948092	0.58666621	0.34554934
APBA3	2.42109751	1.6865788	1.99966455	1.75179388	1.94223581	2.19641485	1.80899138	2.24370877	1.81320263
APBB1	0.75193603	2.26953056	1.14024218	1.63145097	0.76079855	2.05268688	0.87449983	0.79667864	0.63648482
APBB1IP	2.36812447	3.60520362	3.90829606	3.05543278	4.2370271	3.76621982	2.71391555	1.70154838	1.88168494
APBB2	1.7279088	2.66109594	3.18181127	3.19671807	2.45490074	2.45635174	2.55293119	2.07960669	3.24425623
APBB3	1.324771	0.83706081	1.79896556	1.95478533	1.18391203	1.39172085	0.86037962	1.07389597	1.6571233
APC	1.57428916	2.46560494	2.09428696	2.390506	2.64105927	2.53896683	2.21923504	1.742287	2.50359235
APC2	0.88862146	0.362583	0.2868715	0.29249275	0.5989655	0.30256047	0.54170622	0.65838435	0.6801656
APCDD1	1.83830845	3.38668883	3.43123629	2.05913519	1.18270437	2.86609661	1.62952122	1.35317599	1.24466271
APCDD1L	0.17398069	1.28406554	0.42005093	0.4532213	0.12015562	1.04891729	0.39457814	0.16799409	0.09405949
APEH	4.43353869	3.75674899	4.36122804	4.10715308	3.86252881	4.45045356	4.83340167	4.70649548	4.90291741
APEX1	6.37014441	6.44431583	6.54482595	6.90221233	6.3947549	6.43173921	5.89731421	6.34035234	7.358941
APEX2	3.84072529	3.50060264	3.67040435	3.56620659	4.32786121	3.52502483	3.19896908	3.78626179	3.70833765
APH1A	6.43151895	4.94457216	5.84183964	5.19105265	5.19839974	5.61962377	6.3598389	5.74758132	5.49427458

APH1B	1.8618309	2.12106361	2.18833075	2.17103396	2.13256347	1.97309447	1.41425262	1.34081159	2.1337301
API5	3.57366206	4.72276811	4.65149673	5.27932339	4.675424	4.95904015	4.09513993	4.3303334	5.33521653
APIP	2.16317324	2.35032614	2.60525969	2.40196636	2.94282263	2.36054638	2.14031113	2.18532514	2.57798226
APLNR	2.76520314	5.18481268	4.37058853	4.08636745	2.34009354	4.83087969	3.4364748	2.56185169	1.65170024
APLP1	2.91918341	0.71472577	0.88255914	1.44600429	0.17338271	1.01658396	0.3760723	2.09254103	0.43153691
APMAP	5.70406403	5.53154539	5.9029149	5.7224154	5.0609237	5.75259295	5.81266083	4.8779256	5.58673863
APOA1	0.02536901	2.36910068	2.65965763	0.21368692	0.56643336	0.99330951	0.01523957	0.4168717	0.06574317
APOA4	0	3.4163078	3.39615922	0.02938687	1.20793402	0.32429183	0	0.1012585	0.07599473
APOB	0.00245576	1.88263841	1.73709823	0.09003122	0.35402711	0.15727217	0.01318114	0.1780123	0.04577573
APOBEC1	0.32854121	1.80335862	0.92849821	1.12189786	0.65780347	0.10176367	0.38535206	0.9574511	3.23178285
APOBEC3A	0.66832424	1.56440083	1.20666813	1.12182597	1.4273634	2.40555933	0.63367913	0.58459565	0.40774733
APOBEC3B	2.27020001	1.63877266	2.91930946	2.15744474	3.3952339	1.79177239	0.97456791	2.04341847	2.08256077
APOBEC3C	3.86247438	4.68216607	4.32766038	3.57374959	4.361034	3.83362504	3.33795731	3.66072556	2.87439716
APOBEC3F	1.86761429	1.30618978	1.34941935	1.11684141	2.25923454	1.37714268	0.66694544	0.97821989	1.17727895
APOBR	2.09681123	1.63523257	1.94638231	1.6573117	1.82890748	2.17391706	2.12753699	1.44103836	1.49615103
APOC1	7.76650236	4.01989599	5.22086062	4.07849256	7.44822385	4.34357694	5.26046224	3.12816497	3.93576532
APOC2	0.83192036	0.36336064	0.96622009	0.89839808	1.16638918	0.10230815	0.52906985	0.13778679	0.39550572
APOC3	0	1.06079249	0.8984485	0.02503029	0.14772615	0.17455368	0	0.06346385	0.06488205
APOD	0.76671823	7.86450626	5.66347561	5.19524675	0.98884681	4.86204345	1.69463729	1.04516319	0.58654438
APOE	9.48087659	6.42653944	6.19432115	5.61916867	7.89684209	7.80581819	7.12517257	4.95151022	5.58584227
APOL1	8.05134698	5.06753574	5.79219958	4.67885081	6.05354797	5.6476213	6.52459772	4.65399433	3.86782093
APOL2	5.14055145	3.77835313	3.97548562	3.81566702	5.07301505	4.10638008	3.96516863	3.03296553	4.05737359
APOL3	3.02285349	2.94699643	2.468336	2.10789567	4.21975273	3.08766622	1.6100531	1.26352103	2.70879882
APOL6	4.15646366	4.36354386	3.53239425	3.63855621	5.63109772	4.94642266	3.1129171	2.76285324	4.95582627
APOLD1	0.81869984	2.65674624	2.9931496	2.54388245	2.00057525	2.54060997	1.90030015	1.49643809	1.3562156
APOO	3.49469963	3.20077946	3.21926428	3.75887379	4.73249282	3.02421884	3.3160892	3.55322444	3.26959722
APOOL	2.04975436	2.32504667	2.65436574	2.88839484	2.72544698	2.17801338	1.91504475	2.22792413	2.70708389
APOPT1	2.06357253	2.01818804	1.45448512	1.93559261	1.78324758	1.84664107	1.64997553	2.27812505	1.93350604
APP	6.35904127	6.94891982	7.29236378	7.29542182	6.55639325	7.10452	7.47572518	7.9686749	7.33061566
APPBP2	2.2937649	2.89517934	3.15621752	2.75690193	2.58228894	2.72707191	2.09598125	3.24329395	2.52428567
APPL1	2.88976821	3.39219975	3.2742746	3.89552814	3.5952541	3.99953862	3.97529478	3.35468985	3.00659952
APPL2	3.29280977	3.25307537	4.16567254	3.54386362	3.23090789	3.01644642	3.38660578	4.28468895	3.68318406
APRT	5.22768232	4.79727283	5.03091649	4.57423074	4.48524033	5.26109005	5.70952844	5.51452562	5.716299
AQP1	8.40996432	6.3409487	5.82569126	5.07271424	3.13050114	5.5504185	3.51289693	2.80453905	3.8427221
AQP10	0.01581395	0.10535435	0.53198213	0.02833941	0.11300444	0.01742149	0.02827627	0.06001943	0.00833401
AQP11	1.97877329	1.26369013	1.11971321	0.95320936	1.53434851	1.26210699	0.80300419	1.24701273	1.69552422
AQP2	0.00844099	0.01447463	0.04219022	0.06456144	0	0.04892885	0.22478812	2.52625835	0
AQP5	0.03815926	0.82807072	3.42363822	0.71652725	0.30002338	2.91061163	3.00637967	8.34606736	0.05972321
AQP7	0.16996889	0.45614416	0.4001423	0.35599011	0.07495207	0.02777167	0.02470577	0.13518658	0.4099211
AQP8	0.0254221	0.07164524	0.62239415	0.42363343	0.01571402	0.0093928	0.01527157	0.09561085	0.20102103
AQP9	1.83556886	2.89115414	2.26715663	4.19574606	3.15676225	4.33241434	3.37400714	2.75328399	1.97479009
AQR	2.25856422	2.73260592	2.5249616	2.969759	3.59116268	2.96969034	2.60736292	2.22448793	3.16672443
AR	0.04770084	1.43967662	0.21413177	0.70378968	0.05517056	0.83331302	0.23289176	0.06765933	0.0602078
ARAP1	3.51016207	3.35121286	3.35900734	2.84677588	3.11451127	3.57786209	3.11454967	3.34697847	3.11212858
ARAP3	3.16072375	1.73238254	2.51837207	2.60497791	2.20793584	2.7055632	0.87274021	2.19060398	0.72469243
ARC	0.02416622	0.42597967	0.07712904	0.47526372	0.02971746	0.31272458	1.07879422	0.11287432	0.10496895
ARCN1	5.3934994	5.89422104	5.32980505	5.98842715	6.10161179	6.14646217	6.69465201	5.58772926	6.01121931
ARF1	7.67461804	7.02298787	7.60183573	7.09361292	7.25106188	7.55483515	8.32369642	7.97612483	7.69292883
ARF3	5.92494166	4.98779534	5.1578051	4.92339965	5.27733904	5.16629263	4.98131704	5.57362908	5.40451504
ARF4	6.52570711	6.92526126	6.9599147	7.16085614	6.29670837	7.03658678	7.61752755	6.90112117	6.61504235
ARF5	6.63498601	4.92874649	5.72156526	4.9871776	4.40623471	5.06152912	5.50422309	4.95153413	5.27132224
ARFGAP1	3.56294658	2.61408229	3.82895865	3.13472637	2.68565978	2.89412136	3.02971002	2.99376254	3.47846898
ARFGAP2	3.99999534	3.03617952	3.37933762	3.15970913	3.32249763	3.36421019	3.6063362	3.45851624	3.39816688
ARFGAP3	4.0706114	4.26574315	4.46037659	4.26368152	4.64328919	4.13462427	4.28331308	4.47812855	3.77540018
ARFGEF2	2.22906374	3.45947345	4.10810679	3.80270612	3.44869947	3.53638266	3.8195184	3.75046015	4.35806531
ARFIP1	3.18347524	3.64210565	3.84722628	3.68716877	3.95279241	3.46028746	3.47691094	3.84566345	3.96291617
ARFRP1	2.40486249	1.98467081	1.9385463	1.90237404	1.57097277	1.71822478	2.79742226	2.78905679	2.50671666
ARGLU1	3.87261003	2.54447342	4.29377519	4.06682796	4.26087732	3.15545832	2.99471254	3.24368786	4.86126394
ARHGAP1	4.83521145	5.01407436	4.77911166	4.94766457	4.58366196	5.01307977	5.00819451	4.6920099	4.28577921

ARHGAP10	1.41015717	1.5777732	2.05430918	1.45624754	0.8055506	1.73183966	1.33305134	1.36790202	0.46737814
ARHGAP11/	2.7225053	1.5360054	2.35446899	2.99792609	3.78709533	3.20370311	3.20654443	2.3882028	2.30008946
ARHGAP15	0.8103551	2.29061569	2.02379547	1.29647686	2.27465677	1.99277973	1.05386499	0.64462585	0.77252353
ARHGAP17	2.45486541	3.04316956	2.75233737	2.94938553	3.34911851	2.84856843	2.59469309	2.21341271	3.03096011
ARHGAP18	2.92675484	3.8914143	4.32064624	4.81640116	4.35636073	3.84644267	4.19419373	3.95903708	3.77976057
ARHGAP19	1.44817556	1.06098547	1.00233357	1.0978255	1.85171134	1.6264503	1.16870323	1.01990775	1.56423998
ARHGAP21	2.73942438	3.32570839	2.69185232	3.15379661	3.59658298	3.43570489	3.05917175	3.42780333	3.31579323
ARHGAP22	0.4877139	0.62266265	0.61104604	0.39020671	0.28446628	0.50598547	0.47059515	0.27106548	0.15515551
ARHGAP24	0.6107799	1.79044777	1.80996886	1.75060532	1.74148567	2.50340165	1.0233245	2.17868357	0.65313405
ARHGAP25	1.92899095	2.29164706	2.31134469	2.10610679	3.21689067	2.5886614	1.73785723	1.21173493	1.40934155
ARHGAP26	2.60720641	2.46827923	2.71034736	2.6876416	2.723273	2.80621974	1.96864794	2.76478097	2.12980425
ARHGAP27	2.69507558	1.79913548	2.50387419	2.4232832	2.1119085	2.17917496	2.53191834	2.88801417	3.19834101
ARHGAP29	1.2888827	2.23479364	1.93370892	1.82506654	1.86532834	1.86097631	0.93195109	1.01689607	1.07214006
ARHGAP30	3.03884838	2.23451718	2.79854958	1.91738865	3.23342831	2.98133149	2.26881574	1.55862804	1.62997997
ARHGAP32	2.90307484	3.063021	1.82830463	2.54195707	2.77889989	1.95137388	4.6213166	2.55338025	4.26750707
ARHGAP33	1.78430628	0.38747735	1.09161402	0.95340648	1.38212935	0.70014136	0.61256581	0.73670661	1.38576809
ARHGAP35	3.84712467	3.55551143	2.8715165	2.93495183	3.64534777	3.58121473	3.44787259	3.49227315	3.21761178
ARHGAP39	2.32345234	1.27380116	1.45217384	2.00386077	1.31430506	1.5533407	2.15962728	1.59919316	2.22008027
ARHGAP44	1.25754062	1.58286026	1.08466129	1.20155328	1.15645436	0.81971738	1.07136958	1.25003445	1.33391247
ARHGAP8	1.0483625	0.55842802	1.1960261	0.48324126	0.68539892	0.53803756	0.48544327	0.57695396	0.84022029
ARHGAP9	1.731491	1.63872094	2.57585378	1.64542062	2.65598883	2.54485111	1.62124815	1.17329655	1.70416668
ARHGDIA	7.23586325	5.60411503	6.49451112	5.90927365	5.79323787	6.47928879	5.77990893	6.84753819	6.7022607
ARHGDIB	5.8556154	6.24216123	7.03594262	5.92962782	6.98516451	7.08357145	5.99849399	5.00320439	4.95183343
ARHGDIG	0	0.1927892	0.17342417	0.59015179	0.02746641	0.19078543	1.03711143	0.00577132	0.29818837
ARHGEF1	3.24222164	2.35097829	3.35159142	3.36647913	3.50724563	3.21613511	2.71049961	3.6870146	2.95790409
ARHGEF10	0.91263066	2.18217269	1.87389806	1.60632681	0.83198633	1.44867566	2.54764025	2.37541212	1.89546509
ARHGEF11	3.66187333	2.98809012	3.30311179	2.94848951	3.54936956	3.26231187	3.62592336	3.69016487	3.29531455
ARHGEF12	2.79292948	3.41341752	2.94374221	3.17899832	3.38938817	3.45120191	3.69672648	3.02769526	2.91408622
ARHGEF15	1.16699214	1.97955828	2.03898828	1.67359183	1.39103453	2.0380032	1.08192238	0.82872425	0.84608238
ARHGEF16	4.09689915	1.58757808	1.83278528	2.35441405	2.03744887	1.90225445	2.88570835	2.59927776	3.83890429
ARHGEF17	1.58977643	2.96409935	2.69035444	2.57847002	1.62373516	3.06685679	2.27533196	2.16630448	1.36967372
ARHGEF18	1.56211831	1.02999534	1.03415342	0.94023058	1.20031576	1.144224	0.85216134	1.3814444	1.08420511
ARHGEF19	3.89942854	1.44737478	1.39111906	2.08599307	0.50980742	1.07238198	1.22437021	1.12282095	2.23099559
ARHGEF2	3.05992831	3.30519514	2.96251047	3.29203222	3.0905067	3.50959936	3.51794638	3.86388957	2.79056315
ARHGEF25	1.15441934	2.745133	1.18348055	2.07540554	0.42923597	2.16488026	0.91452458	0.72037386	0.50666029
ARHGEF26	0.15711633	2.55051781	0.57089265	1.4539232	0.11776879	2.00843419	0.38188402	0.97318625	0.96578771
ARHGEF28	1.03776425	0.9123226	2.02586245	1.03975412	1.04423132	0.68740302	0.86635268	2.00651571	0.83850031
ARHGEF3	3.02317048	3.12830096	3.57885132	2.809974	3.30189059	3.0154031	2.95488065	3.76154974	1.84869083
ARHGEF37	1.54913678	1.8153527	1.55761715	1.27021255	0.97209212	1.67341143	0.76203084	2.86357499	1.88989413
ARHGEF38	1.40774862	0.95129147	1.62206368	1.31602695	0.74109536	1.15517513	1.25741639	2.71510101	1.03400661
ARHGEF39	1.32255203	0.76127025	1.21965038	1.28384217	1.0418661	1.39914271	0.86346967	1.39299081	1.42346927
ARHGEF40	3.17272576	3.17826492	2.62799776	2.04795566	1.61622913	2.28446288	1.54984235	2.9684579	2.88690801
ARHGEF5	2.92679142	1.25868193	1.84568605	1.43213776	1.37082822	1.46366046	2.01099499	2.86671	2.14889558
ARHGEF6	1.3774005	3.39813009	3.07488219	2.42924705	3.21516275	3.26770279	2.32605076	1.67683872	1.60850407
ARHGEF7	2.59051823	2.61759311	3.07371096	2.78817641	2.58180204	2.69090799	2.55591654	2.4967104	3.29677054
ARHGEF9	1.81935758	2.36812703	1.83930902	2.30236111	1.90155702	2.21554197	1.70794262	1.69129844	1.34838064
ARID1A	5.18503319	3.8091653	2.50290672	3.65653697	3.74394699	4.17285412	3.77209957	3.85356579	4.31622332
ARID1B	2.89618843	2.8556578	2.9719472	2.56278216	2.72482042	3.00392885	2.97844819	2.41250077	2.44345927
ARID3A	2.53432817	1.18675455	1.73170362	1.08433817	1.4943655	1.386439	1.46449449	0.52087796	2.23320487
ARID4A	2.00390617	2.70103613	2.27548541	2.3855835	2.66127031	2.21948044	1.82440776	2.4527299	2.19125232
ARID4B	1.86737558	2.26306255	2.22817205	2.34137573	2.62638997	2.02548168	2.10551096	3.28706411	2.19746965
ARID5A	3.43011549	2.83941811	3.53760709	2.95518798	2.98532003	3.64625498	2.88602475	2.50408928	2.67067491
ARID5B	2.74741745	4.93550141	3.74588036	3.82490917	3.49596473	4.02855654	3.55419436	3.09013397	4.45500926
ARIH1	1.71804184	2.3558579	2.46393435	2.60925213	2.61280714	2.44211521	1.98028631	1.91434195	2.31633343
ARIH2	2.68887694	2.669562	3.30226947	3.23806628	3.47700627	3.38920761	3.02591408	2.9530653	3.2022011
ARL1	3.74252251	4.47139386	4.78145051	4.69197555	4.53786755	4.42353293	4.47971327	5.23389332	4.25128362
ARL10	0.29521369	0.91180794	0.71252432	1.20088964	0.4635243	0.73763113	0.52153619	0.52619816	0.20823878
ARL11	0.84713114	0.85848044	0.91628561	0.75977506	1.05512044	0.95880496	0.81787684	0.37317014	0.41486853
ARL13B	1.9682541	1.93151556	1.81634349	2.45427366	1.27492632	2.10815389	1.76354707	1.59137811	1.81642919

ARL14	1.62708152	2.44047084	4.25835356	2.81151231	2.23024644	3.17759762	3.32336781	5.41002793	2.13301274
ARL15	2.35377491	3.0970989	3.35590066	3.18061287	2.90538048	2.92787791	2.53382017	2.47952679	2.60105508
ARL16	3.31174066	2.5073784	3.12951819	2.84051233	2.11894787	2.08218761	2.26896064	2.77184855	2.55025153
ARL2	4.49210833	4.44548269	3.61717009	3.75348714	3.65672474	4.41672789	3.48943642	3.97164928	4.10566344
ARL2BP	3.14441684	2.77859039	2.96983419	2.57487639	2.31377424	2.74192772	2.44408765	2.68294276	2.38936331
ARL3	2.80328337	2.7480189	2.2263742	2.66861643	2.3588009	2.11186343	1.96465729	1.37331144	2.50780606
ARL4A	1.25726617	3.21810837	2.90663722	2.95343153	2.13607737	2.70095275	3.17339193	1.92280098	2.75805367
ARL4D	0.55594503	1.4643369	0.70842319	2.00985668	0.19510897	1.56446843	0.62675233	1.42823933	0.23018202
ARL5A	2.96150667	3.51313693	3.72352213	3.59377998	3.71968384	3.66584694	3.69354699	3.32755095	3.68269861
ARL5B	2.31234305	3.19881511	2.9383208	3.64284006	4.06594291	3.82128055	3.27107391	2.70135092	3.88597457
ARL6IP1	5.27090492	7.04265301	6.66961746	6.73493649	6.52271065	6.30763089	7.19610723	7.03895708	6.23276232
ARL6IP4	1.00596677	0.4370279	0.65163516	0.66277917	0.42368839	0.5726787	0.37747365	0.78342528	0.82660323
ARL6IP5	5.73297984	6.7206585	6.32313231	6.54212596	6.56812415	6.39241374	6.31970601	6.45794323	5.87632023
ARL8B	4.11540979	4.77773617	4.7462999	5.30980854	5.09318822	5.07195991	4.99983492	5.00002905	4.53313415
ARL9	0.16412355	0.49986647	1.06217329	2.02151425	1.89590345	1.15365958	0.23916315	1.15241116	2.52352696
ARMC1	2.77576131	3.60483918	3.72139566	4.19970954	3.99614385	3.97193684	3.83488447	3.73219158	4.00575692
ARMC10	3.95528504	3.10616551	3.49019709	3.31247775	3.15326799	3.36910276	3.50556744	3.21909935	3.15528207
ARMC12	0.05050877	0.23078759	0.34515164	0.17409803	0.16442997	0.16911344	0.04543257	0.03908936	0
ARMC2	0.53778215	1.1891348	0.96593384	0.83555341	0.94317401	0.52503776	0.67604753	0.33998749	0.56671806
ARMC5	2.1053195	0.94784983	1.20169068	1.01400182	1.07435498	1.40532991	1.71842562	1.2100149	1.63900158
ARMC6	3.54359439	2.00374397	2.78949798	2.27985902	2.76540363	2.80955361	2.64577118	2.58562231	3.26631411
ARMC7	4.39108617	1.67758989	3.77513019	2.66246648	2.51711761	2.9993944	2.21989341	3.13888158	2.53197932
ARMC8	2.59283955	2.24828604	2.56115535	2.67153209	2.65936421	2.55634232	2.44053871	2.62520677	2.080414
ARMC9	0.87920031	1.41035397	1.36168182	1.66644718	1.24440798	1.55332843	1.06201398	1.30296757	0.6823532
ARMCX2	1.05444133	3.42717616	2.10442682	2.53008988	1.01052981	2.51464512	1.71530087	2.67713192	0.6640866
ARMCX3	1.11241657	3.65368707	4.0693536	3.7054647	2.33400092	3.72227829	3.01709776	3.96315183	1.21140681
ARMCX5	1.29009266	1.49313352	1.99158561	2.12866441	2.77002814	1.74139576	1.41791493	2.22088553	1.72995753
ARMCX6	0.79139979	1.4440343	2.71003694	2.30974451	1.07924754	2.01140919	1.65567798	2.05139114	0.49649837
ARNT	2.99469244	3.47157083	3.44641076	3.48683492	2.87644562	3.40241425	3.7486097	3.03047476	2.89006732
ARNT2	0.24214339	1.25519002	2.32830981	2.34988423	2.05947693	2.01975218	0.97740551	0.33958063	0.41814456
ARNTL	1.34049349	1.95709399	1.71815272	1.79479114	2.15674075	1.64010532	1.12455177	1.41867209	1.92877534
ARNTL2	2.45189882	1.08899278	2.56911229	2.65339005	3.78507753	2.64821396	2.8881954	2.16265298	1.76300654
ARPC1A	5.90594016	5.2885288	6.33151508	5.32004398	6.15153895	5.51087701	6.3917326	6.17104823	5.74401833
ARPC1B	6.00611306	5.5079301	6.16629901	5.16091809	4.86765966	6.10534923	6.36598475	5.64731019	5.0398591
ARPC2	5.7288056	5.46013864	5.94435855	5.77983342	6.14901572	5.81386612	5.73374149	5.57850448	5.25736548
ARPC3	6.49874032	5.86981583	6.55518792	5.81449282	6.48757465	6.13327886	6.14751596	6.2919248	6.02412423
ARPC4	5.31174909	3.8288947	4.35220647	4.12963913	3.96431363	4.41695234	4.45295562	4.0575882	4.41406113
ARPC5	4.12354885	4.41540358	4.87156787	4.92187555	4.89439862	4.7299328	4.87790233	4.56756927	4.16977099
ARPC5L	4.44514335	2.88071448	3.93805141	3.33235806	4.04041405	3.58857546	3.52269805	2.42761094	3.29902832
ARPIN	2.20373926	2.67914021	2.2239751	2.32844829	2.31463349	2.31892716	2.18557142	2.9546135	2.7619809
ARPP19	4.32837965	4.80079378	4.73401088	5.28857599	5.16906911	5.2983669	4.60536732	3.89068849	4.94086625
ARRB1	3.48811069	3.01807722	3.10017117	1.84403698	2.02436813	2.79734155	2.80016009	2.40102896	3.31066055
ARRB2	3.86061149	3.69980188	4.27120758	3.84956779	3.59167173	3.91229959	3.75341413	2.40849777	3.9779759
ARRDC1	4.50198504	2.81483313	4.60595875	3.48309993	3.53066969	3.54758043	3.5348604	4.14009022	4.2700147
ARRDC2	2.52369572	1.89693871	2.83512057	2.45961228	3.07445939	2.66002296	2.59417375	3.23987852	2.07188791
ARRDC3	3.70226636	4.89164441	3.96379501	5.15243389	3.4159771	4.88777804	4.05310501	4.70426775	3.29954552
ARSA	4.63809793	4.02496278	3.26900285	2.35754428	2.62521108	3.5708948	3.60294779	3.12554038	3.6146613
ARSD	3.47729263	3.44958118	2.67746503	2.29214891	2.49623995	2.00085421	2.85001011	3.66442465	4.37001544
ARSE	2.34277447	3.17059674	2.88599648	1.82733355	2.92233656	0.4137906	3.03533578	4.54775296	4.38251296
ARSG	1.25366662	0.88420146	0.94931613	1.32997364	0.65453358	1.09663388	0.31954424	1.10332872	0.45514339
ARSI	1.05251664	1.11674306	0.39247655	1.72633185	0.28003675	1.5844237	1.09622978	0.31525122	0.31936204
ARSK	2.06136793	1.42787518	1.25045398	1.3179448	1.29075857	1.55615289	1.17753766	1.43628683	1.05470998
ARV1	3.64451584	3.74826081	3.43546705	3.19917703	4.17395953	3.87827248	3.4138061	3.91294685	4.17586509
ARVCF	1.86139283	1.41582216	0.98590389	1.72008279	0.76121655	1.11818897	1.07059984	0.88925065	2.43384899
ARX	0.01261677	0.39722145	0.51754499	0.1785486	0	0.93120862	0.13737332	0.03378975	0.1812846
AS3MT	0	0.23211251	0	0.04477499	0	0.03303983	0.08800957	0.01163896	0.04703718
ASAH1	4.99540414	5.66816568	5.76171135	6.11551481	6.28491262	5.95985398	6.44384757	5.12925108	5.07313034
ASAP1	2.39565319	3.29366636	3.47957632	3.66343413	3.46077565	3.44513664	2.79703491	3.29318107	2.01742391
ASAP2	2.04486526	2.19079369	2.81727408	3.05707752	3.03814086	2.94654767	3.46187164	3.46396063	2.59145675

ASAP3	0.64821133	2.75171189	1.45888516	2.18841816	0.61390092	2.09656208	2.12301204	0.58842593	0.49837727
ASB1	1.74368602	1.53800804	1.64524498	1.67102844	1.37869764	1.85216202	1.70537185	1.72278359	1.29155164
ASB10	0	0	0.01104398	0.00917722	0	0	0	0	0.00804357
ASB13	2.46616852	3.05729192	2.60569376	3.34341836	2.44392314	2.77238736	3.19576622	2.00082587	3.9545055
ASB2	0.60051849	2.58734385	1.28331658	0.95697135	1.03115746	2.18898264	0.26065654	0.42223322	0.4640259
ASB5	0	1.55430115	0.10613886	0.19590576	0	1.33664829	0.01069655	0.04759512	0.0600135
ASB6	2.47321395	1.83982256	3.00017098	2.38862302	2.08374527	2.87455044	2.64335129	2.03812987	2.36413917
ASB7	1.86575808	2.05414411	2.761115	2.11828363	2.75729156	2.29798126	1.91175535	2.21821332	2.43469055
ASB8	3.03903328	3.05994155	2.98660092	2.65686462	3.1440754	2.88127093	2.59528208	3.78883388	2.87105073
ASB9	1.41560872	0.68367754	0.76138342	1.89011291	1.26865448	0.76816473	1.19703604	0.80387343	0.47273149
ASCC1	2.53222288	2.82841759	2.65596608	3.04148503	3.04276398	2.7328766	2.87205248	2.29097007	2.33168925
ASCC2	4.50746853	3.22234248	3.9513793	3.16596405	3.57448421	3.25678772	3.31529775	3.21416035	3.51655911
ASCC3	1.95572835	2.53499504	2.19920225	2.95067019	3.66516504	2.93623443	3.65315201	2.34014338	3.20547024
ASCL2	2.08159074	3.88045465	0.98229167	0.80973184	1.87992136	1.23460433	1.66163957	2.72911365	6.71559904
ASF1B	5.10237966	2.85526645	3.6210171	3.89144803	4.497009	3.99656553	4.13037349	3.36531913	4.35774872
ASGR1	0.99333956	1.05191045	0.64153208	0.28494241	0.80520142	0.42411582	0.19056715	0.08372161	0.39540419
ASGR2	0.31439083	0.42530979	0.63881306	0.36978718	0.27798175	0.66469545	0.17317015	0.03895771	0.06763125
ASH1L	2.7404414	3.33040302	2.85188648	2.79752785	3.12882368	3.0783479	3.07301996	3.88957252	3.12974162
ASH2L	2.83033576	3.13236132	2.52730306	3.54386622	2.99793532	3.33846091	3.65488809	3.23725009	3.23311738
ASIC2	0.03219566	0.21126508	0.14319129	0.01940206	0.07807622	0.02137321	0.03846107	0.05900864	1.27706761
ASIC3	0.48056394	0.10261581	0.35494846	0.22089649	1.00578754	0.39780003	0.06215315	0.96051243	0.11389383
ASIP	0.17616997	0.43656458	0.40970881	0.2801091	0.35619077	0.26641375	0.18403581	0.13765685	0.32885047
ASL	5.08218395	3.39503303	4.37921081	2.97091288	2.98399022	3.55978201	4.70329847	3.940176	4.15470633
ASMTL	3.00193562	2.76437993	2.1776479	1.95086028	2.47587657	2.62596932	2.04961503	2.55836592	2.9038033
ASNS	3.28196184	1.82240245	3.23551949	3.26919513	3.75363424	3.76228506	3.65983502	2.37986706	3.04104936
ASNSD1	3.15859528	4.09344114	4.26355332	4.10813943	4.34455615	4.09485626	3.98366513	4.17395185	4.22300663
ASPA	0.01855709	1.67802578	0.79556914	0.64321859	0.30637685	0.4333906	0.07621203	0.41967856	0.07957534
ASPDH	0.43455514	0.07385078	0.10740189	0.18590584	0.29390029	0.02410013	0.07709068	0.02527033	0.02299403
ASPH	0.80508938	3.15779117	3.76261677	3.66428105	2.89878276	3.4890418	5.10748408	4.10773455	3.92409821
ASPHD1	2.23054321	2.57150594	2.83302099	2.15743579	2.06481829	1.82261611	2.02878556	1.54404554	2.88937963
ASPHD2	2.47708895	1.04288896	1.66659151	2.15063001	3.24964149	2.55853399	1.79805435	1.98277435	1.9065246
ASPM	1.28209182	1.08979261	1.66027918	2.03841729	3.36586302	2.35753246	2.22615087	2.27900269	2.42859188
ASPN	1.52186361	5.97172104	4.78853255	5.5995108	3.86693256	6.29732207	4.16265281	2.53340083	3.65795075
ASPRV1	0.51535403	0.39949062	0.21323454	0.31459702	0.38668527	0.23764784	0.27308036	0.3781225	0.34990327
ASPSCR1	2.35146937	0.97211086	2.24352275	1.08154632	1.01171315	1.1269107	1.10855339	2.48100504	1.36465909
ASS1	4.57650935	5.18253409	6.67666262	4.53061561	1.35113592	5.54439747	8.16431183	4.69467831	8.06628913
ASTE1	2.46624286	1.80370999	2.41548629	1.9688018	2.33257303	1.80058495	1.76008917	2.0865632	1.98559113
ASTN2	0.15412735	0.67846796	0.43535542	0.5840996	0.32155046	0.54059239	0.35922427	0.3947977	0.37085623
ASXL1	2.60967182	2.8903622	3.40022969	3.26001185	3.02166945	3.1323811	2.84697902	2.98112096	3.30648572
ASXL2	2.06927369	2.96777377	2.73255239	2.80216207	3.07335481	2.97294274	2.82962114	2.92402386	2.83554147
ATAD1	2.21084764	3.85360762	2.38642109	4.07850024	4.21425978	4.0063176	3.68111956	4.02195786	3.94167363
ATAD2	2.40540126	2.41082737	3.31891992	3.2300199	3.94866874	2.6097025	4.0475456	2.98972325	3.2168681
ATAD3A	3.54952912	2.19934859	1.93535262	2.2413775	2.30155182	2.95426133	3.24261214	1.93130408	3.35272626
ATAD3C	0.61102581	0.31900853	0.71196876	1.09743756	0.18522237	0.21255921	0.16079305	0.53905977	0.17663789
ATAT1	2.38222939	1.48553973	2.77384862	2.35627216	2.41446621	1.5998744	1.94278672	1.75255388	3.02930037
ATE1	2.15211214	3.37926437	3.52961591	3.36682827	2.89843752	3.33929204	3.25903665	3.22823923	3.25896944
ATF1	3.37200143	3.48228029	3.30020846	3.7687061	3.73689885	3.65044879	3.50765393	3.77086086	3.8486981
ATF2	2.82092475	3.4130016	3.75186492	3.55906338	3.36616991	3.79552211	3.64185732	3.90160411	3.45668489
ATF4	7.11721867	5.65374272	7.2359549	6.6186134	6.68784411	6.3301924	6.13704223	6.22819819	6.97503579
ATF5	6.23386824	2.5722565	3.38068769	3.42050575	3.98651159	2.93875859	3.07076982	2.80027894	3.37027579
ATF6	3.06087572	3.68463248	3.66857654	3.81078424	3.84587713	4.01310871	4.61939438	4.59719726	3.68781061
ATF7IP	2.21744227	3.43523008	2.87042233	3.32342071	3.47145841	2.81591868	2.26327636	2.88182698	3.03003859
ATF7IP2	0.64638064	1.19711266	2.3458561	2.11103967	2.98512465	1.25077791	1.02933898	1.90271547	1.15569299
ATG10	0.86071743	1.40259252	1.55874548	1.20444266	1.12212259	1.42289947	1.06010641	0.89349618	0.57040165
ATG101	3.52785513	1.8699306	2.52272103	1.98070232	2.12805164	2.71478976	2.5390673	2.83876861	2.32233149
ATG12	2.52268876	2.31535344	2.88801415	2.87744981	2.75040523	2.65051916	2.34431562	1.67643871	2.30309239
ATG13	3.60669428	3.5430732	4.29085856	3.65177562	3.69241852	3.97039087	3.74744291	4.18930502	3.76990099
ATG16L1	1.97089652	1.72541799	2.55367102	2.83755638	2.64327626	2.30992939	2.33946779	2.47713284	2.46214181
ATG16L2	0.97846249	0.60619511	1.51580678	1.60335655	1.68848829	1.42935715	0.8411291	0.95106511	1.49010459

ATG2A	2.67929563	2.29971397	2.6582939	2.42998012	2.60828771	2.80214053	2.90620258	3.21397043	3.09626874
ATG2B	1.13862981	2.36721229	1.86654296	2.09891941	2.06250919	2.08457739	1.78218401	2.49760538	2.44589342
ATG3	4.23330986	3.48398711	3.77879439	3.6722275	4.23837679	3.80890795	3.94060965	3.46475725	3.79750723
ATG4A	2.08579855	2.58566286	3.27307226	2.83194997	3.82906645	2.97435137	2.86847127	2.56958247	2.53710038
ATG4B	3.23309269	2.18064048	2.893001	2.81959173	2.83735617	2.44533083	2.95841513	2.72408991	3.07987353
ATG4C	1.57625542	2.8465415	2.89027765	2.47164902	3.01803217	2.76845228	1.71602092	2.42250671	1.96470817
ATG4D	4.26286575	1.90705304	3.43273252	2.42164728	2.82932659	2.49278679	2.79793928	3.3723916	3.4084234
ATG5	2.9891579	3.41265051	3.24578124	3.84375828	4.13408413	3.86995771	4.26054027	2.77032377	3.66175604
ATG7	2.01030049	1.94255204	2.31539579	1.85190905	2.33792545	2.45212398	2.17844522	2.15062183	1.87393948
ATG9A	3.83464775	3.18913478	3.20145816	3.23200613	3.23221104	3.36201116	3.92210696	3.63433029	3.6863088
ATG9B	0.14113228	0.40217945	0.44341086	0.91421971	1.210962	0.26702487	0.36587788	1.71997888	0.33547021
ATIC	4.297435	3.85404883	4.45493585	4.53181538	5.16974064	4.52336436	4.56787219	4.69618419	5.06608359
ATL1	0.62726454	1.978357	0.99426439	1.26565014	0.69057461	1.4638419	0.69672996	0.80578162	0.9178472
ATL2	2.47804382	3.99653324	4.03694033	4.17578683	3.97698376	3.97397535	3.50245569	3.840561	3.93986211
ATL3	3.0220377	4.43629876	4.17603182	4.83299134	4.11521579	4.52349433	4.54758901	4.59147026	3.57147892
ATM	0.9387595	2.12708262	1.75167989	1.64132987	2.31947844	1.69323962	1.35030056	1.48748682	1.32705889
ATMIN	2.50681139	3.64632138	3.58313085	3.94697069	3.84277805	3.87252349	3.92570766	3.62683589	3.48016951
ATOH1	0	0.41534613	0.21009465	0	0.11693009	0	2.10038662	0.07425793	3.56136139
ATOH8	0.15348868	0.97241255	0.39549195	0.42945017	0.12046262	0.61504585	0.15315032	0.08962998	0.11509353
ATOX1	3.77160902	2.85529155	3.07502921	2.54306367	3.04922338	2.71162989	2.25562239	2.65541416	2.75778345
ATP10D	1.0707617	2.38610016	2.02923088	2.53056176	2.9600983	2.51941802	1.61527757	1.84604909	1.92754464
ATP11A	5.13598697	2.86264604	3.89578709	3.10923938	3.91511433	2.86571604	3.22477596	4.12676775	3.55411928
ATP11B	4.48273135	3.20746541	4.2721789	3.22358114	4.47244483	3.40786901	3.798403	4.31967348	3.32235854
ATP11C	2.01391723	2.45455136	2.51252678	2.37702099	4.054583	2.65635516	1.74672432	1.52986912	2.21163588
ATP13A1	3.61572201	3.0317367	3.55629055	3.4156891	3.91648589	3.58767807	3.71284407	3.88765463	3.8705882
ATP13A2	4.6752508	3.75698435	3.12776535	3.44039631	4.37059643	3.94076616	4.50569528	3.25047274	4.24469261
ATP13A4	2.45266437	0.01382059	3.01401227	0.30382024	2.30727065	0.03953849	0.0216064	0.31369049	0.20460726
ATP1A1	7.82525692	7.35735021	7.47760057	6.98457798	6.53332941	5.98332824	7.23096206	7.8316368	7.7243589
ATP1A2	0.02296328	2.48593346	0.50608169	0.75140884	0.01771524	1.39181265	0.0476985	0.14016671	0.1664395
ATP1B1	7.80629406	6.27351912	7.85624407	8.16653972	7.5152712	6.21178081	9.28336211	9.25408677	7.7595961
ATP1B2	0.18751525	2.30448438	1.17571553	1.18117839	0.2963332	1.13463622	0.23881328	0.18863564	0.18111244
ATP1B3	7.4833849	5.97512144	6.18506327	5.8621786	5.95649698	5.73634263	6.92224515	6.05597861	6.49469816
ATP2A2	5.18861193	4.84116094	5.15954603	5.09586386	5.56820798	5.04082593	5.06533421	5.80743807	4.82439845
ATP2A3	2.0123846	3.45814103	4.59909574	2.84852686	3.11367639	3.6574096	3.55776682	2.76607655	2.67058639
ATP2B1	2.60722977	3.10433783	3.03695853	3.41508586	2.89135705	3.01205703	3.39192316	2.90026092	2.856806
ATP2B4	3.36320813	5.87505543	3.14775244	4.23768706	3.96111969	4.93960171	4.12517851	3.59053467	3.00182164
ATP2C1	5.221842	3.44331234	4.65123223	4.21584471	5.18628319	3.83847784	4.26290516	4.16039348	3.96272269
ATP2C2	0.02944045	0.94045186	2.3956904	1.65540282	0.74071901	0.4804257	3.86502457	2.55161138	3.27098617
ATP4A	0.01862189	0.00535791	2.04455602	0.57631663	0.11641014	0	0.00559883	0.25432145	0.01957026
ATP4B	0	0.06849463	3.28830365	1.23551979	0.54614226	0.0267479	0.05748499	0.56968666	0.14676556
ATP6AP1	6.10507622	4.585825	5.85252353	4.81265963	4.9470531	4.90087778	5.55383344	5.02527042	4.67143901
ATP6AP2	5.54422367	6.1328242	6.55608309	6.17926864	6.67628139	5.97528792	6.29356797	5.42407827	5.40244854
ATP6V0A1	3.81196213	2.75876002	2.89056896	2.85090889	3.11336993	3.07575766	2.88847215	2.65812477	2.79604225
ATP6V0A2	1.19293803	1.42847165	1.63305985	1.68260768	1.96583459	1.49948129	1.31502115	2.33246965	1.66376878
ATP6V0B	5.15010759	4.07916911	6.14465754	4.52750173	4.5808602	5.00831828	5.21683087	5.31268028	4.9507667
ATP6V0C	5.32893463	5.02046179	4.18432238	4.4540117	4.41177583	5.05877378	5.00935753	4.77867106	4.37003786
ATP6V0D1	3.92451234	3.73363388	4.2343257	3.63121237	3.68559582	4.15098284	4.18753376	3.96696122	3.86472452
ATP6V0E1	6.17473073	6.97467502	6.75648816	6.93299758	6.72380482	6.99630997	6.60219411	6.83277711	6.23590723
ATP6V0E2	1.4868614	1.83884916	3.27408894	2.56932805	1.57007646	2.63265567	2.66994523	3.29653095	1.82870439
ATP6V1A	4.38842827	3.92189307	4.28537801	4.26028632	4.55459713	4.27358422	4.19285628	4.12447277	3.84177513
ATP6V1B1	0.16836643	0.27173416	0.64533104	0.49704278	0.10602276	0.06898732	0.61898088	0.09724535	0.12444935
ATP6V1B2	3.63558468	3.80773062	4.06908697	4.58854305	4.85583104	4.7525255	4.58879642	3.71732243	4.00087118
ATP6V1C1	2.78159568	3.65434893	4.67216198	4.43681057	4.41831267	4.3049865	4.08157474	3.68235615	3.62955567
ATP6V1D	3.77539384	3.78715996	3.85478857	3.83490256	4.34964225	3.76396909	4.07263307	4.13285268	3.67254414
ATP6V1E1	5.44037774	5.13294413	5.64587164	5.25784873	5.47773837	5.21271476	5.01716232	5.25754028	4.80177194
ATP6V1E2	0.7434889	1.01551861	0.95579674	1.11991561	0.63579904	1.11542288	1.09321679	1.11665624	0.98567621
ATP6V1F	7.93798385	6.50213359	6.98073993	6.46693138	6.73983421	6.5182913	6.354082	6.07920891	6.39121648
ATP6V1G1	6.50123478	6.19300186	7.48022989	6.92802858	6.58794158	6.2839404	6.18028027	5.92949953	6.34105749
ATP6V1G2	0.05004633	0.66570556	0.19472883	0.48786643	0.16764413	0.25122476	0.02018066	0.06398429	0.11156659

ATP6V1H	2.3119231	3.17714355	3.27938084	3.25799358	3.36979995	3.41723214	3.51109806	3.05533412	2.85757064
ATP7A	1.41710464	2.14578757	2.29933685	1.85215571	2.50222689	1.89470602	1.69330406	2.33961411	1.43633441
ATP7B	5.09692521	2.99556712	1.84139759	1.76297521	1.50859792	0.90692543	2.99074042	1.10353721	3.88576551
ATP8A1	0.47298941	2.12497834	1.2541204	2.34721273	2.56507381	2.11633802	1.49025114	1.18391638	2.76057499
ATP8A2	0.06499975	0.22417506	0.30019298	0.12076602	0.06119889	0.18892831	0.17161371	0.08891389	0.04526611
ATP8B1	3.79351237	4.09438499	4.00513508	4.32173303	4.68187799	3.82950039	6.03661496	5.07780521	5.30247006
ATP8B4	0.07518478	1.47089181	1.29979157	0.69561707	0.68613419	1.74053202	0.80082588	0.44210054	0.37935035
ATP9A	5.05807715	3.53222528	4.78155344	4.20399367	3.29305663	3.84886624	4.35370798	4.07062321	3.72098534
ATP9B	0.77596632	1.44530601	1.59427511	1.38180284	1.79855367	1.41001943	2.09097419	1.14428255	1.22504852
ATPAF1	3.00713119	3.13575187	3.61796299	3.16065754	3.54563887	3.23574246	3.08219336	3.45927069	3.73496224
ATPAF2	1.83949153	1.41802971	2.17618826	1.66120042	1.76069293	1.88196333	1.77235883	1.18850675	2.10931099
ATR	1.59627031	1.82633806	1.94739465	1.84571494	2.9551196	2.10014577	1.72319679	1.97325155	2.37570966
ATRAID	5.84747715	5.67393359	6.00031484	5.71391768	4.7756806	5.51465519	5.88540377	5.509808	5.41026399
ATRIP	0.1553024	0.09099089	0.14988618	0.13751537	0.11016535	0.1844887	0.13125809	0.20029928	0.13693791
ATRN	3.63125526	2.76781766	4.88594411	3.7388615	3.61836012	3.36468694	3.5427784	2.94208336	3.77167821
ATRX	2.12339633	3.23351896	2.18360837	2.78945157	3.19097015	2.51349466	2.26835735	2.9756358	2.6563666
ATXN1	1.83263609	2.39870594	2.10057816	1.99950972	1.95811736	2.39027201	2.07199734	2.63537371	0.99147455
ATXN2	2.24169293	2.57053687	2.27752195	2.52117799	2.55771062	2.50787835	2.04692554	2.69240736	2.22580672
ATXN2L	5.10635271	4.29170689	4.50746771	4.32868834	4.84275586	4.51498665	4.27835836	4.72247344	4.58647524
ATXN3	0.52767184	1.06122593	0.98486101	1.12726484	0.86437469	0.88529685	0.75526378	1.08872037	0.88078957
ATXN7L2	1.06765701	0.4807699	0.92862609	1.3155321	0.82890051	0.86643357	0.57661249	0.59266804	1.3680063
AUH	2.33306391	2.85248566	3.46765869	2.85777443	2.5162861	2.61298806	2.50108464	1.81835944	3.53054022
AUNIP	2.07027884	0.57796811	1.30028752	1.74315135	2.44529653	1.8958085	1.85796758	0.57458062	1.72705041
AUP1	5.54888187	4.76778547	5.56330579	5.41671022	5.25521357	5.5051826	5.39002749	5.87649646	5.81627314
AURKA	3.15287918	2.10473603	4.2289716	3.45880754	3.86010426	3.26591727	3.67352143	3.2649013	4.17514367
AURKAIP1	6.32236515	4.3464119	4.05348941	4.41064127	4.55685351	4.87234077	4.94356927	4.22175898	5.04677771
AURKB	3.33100405	2.19443907	3.46167054	3.26443386	3.64944003	3.32552168	3.10739729	1.86970703	3.75259761
AURKC	0.28742089	0.39174476	0.84507445	0.31172113	0.90364503	0.34121829	0.12849516	0.32182054	0.26842761
AUTS2	1.10376061	2.28357234	1.45377585	2.06985702	1.42795308	2.00131452	2.03403021	1.04498062	2.08644005
AVEN	3.00252976	2.62154732	2.49914662	2.45982705	3.01659609	2.89683906	2.57322688	1.90551715	2.49045879
AVL9	3.10438131	2.49791593	2.34249265	2.41987608	2.9692204	2.55295019	2.8570141	2.70661959	3.14868036
AVPI1	4.27250493	4.5221778	4.16574446	4.59301629	5.49033241	4.46673388	4.13714245	5.61195964	4.17040236
AVPR1A	0.29222791	2.46042604	1.20211992	1.67051647	0.70395556	1.7463411	0.72398382	0.50385354	0.32380828
AXDND1	0.01192136	0.44237149	0.98340338	0.08709158	0.02558259	0.0174869	0.36522431	0.06759782	0.1464676
AXIN1	3.08182275	2.48536267	1.97096303	2.20961449	2.34016927	2.67755556	2.754932	2.55467165	2.93694348
AXIN2	0.19536729	2.25014239	2.83792296	1.66810728	0.96342862	1.04161896	0.84921457	2.52142681	4.38437572
AXL	3.25742018	4.48426512	4.72903286	3.76152937	3.94059243	4.27741775	3.65546864	2.80496531	2.55887627
AZGP1	4.54771554	2.52491312	1.97873383	2.58305545	2.54965314	0.09596361	0.17487066	1.96893061	4.57368001
AZI2	1.90358905	2.57292972	2.1703177	2.57311177	2.17157078	2.59750629	2.42058033	2.18347057	2.03912391
AZIN1	4.26136139	4.28843928	4.97024296	5.40957465	4.73993742	5.00376148	5.53094528	4.56561648	4.61000589
AZIN2	0.36177734	0.36625302	0.39443326	0.62575272	0.28765896	0.61015412	0.17518393	0.22921106	0.40481072
B2M	9.84899802	10.4279531	10.5540251	10.0360427	11.7778615	10.8028262	9.86043532	8.64185964	9.55039585
B3GALNT1	0.45247688	2.09738633	3.1944875	2.55859635	0.67379112	2.66065078	2.67501338	3.0574453	0.33012936
B3GALNT2	1.08164118	1.62454109	1.54302293	1.90626402	1.84839651	1.77970721	2.59300306	2.47086297	2.07746626
B3GALT4	1.38313414	2.06186897	2.87053391	1.33972483	1.00289715	2.34075637	2.37964039	2.93377348	1.5607483
B3GALT5	0.23440444	1.84195515	1.00955291	0.54324786	0.09701351	0.06937774	0.21083009	0.32532389	3.23490453
B3GALT6	3.2801699	2.49370888	2.04094896	2.72594383	2.06069264	3.08977312	2.65372678	2.70687969	2.6427971
B3GAT1	0.0926577	0.06955635	0.11937109	0.07459077	0.32579608	0.12025553	0.01330504	0.01589513	0.03478883
B3GAT3	4.52258044	2.60349513	3.26290632	2.60876852	2.33834635	2.89927472	3.34150418	2.88596453	3.36799741
B3GNT2	5.11698431	4.0258461	4.86799789	3.91916988	4.15621142	4.06083221	4.41253225	4.26839191	5.30932132
B3GNT3	6.41640633	3.74155447	5.69603922	4.67591023	4.33635207	4.57916862	5.63108574	6.76976942	5.46860402
B3GNT5	3.01359895	2.18527817	3.09766781	2.19724605	2.41628514	2.59916094	2.70594474	2.52620268	3.1588559
B3GNT6	0.02749337	0.08489784	0.66338838	0.27234979	0.17680447	0.01016268	0.45264168	0.5383763	0.09867785
B3GNT7	3.50853039	2.59303484	3.67440304	2.77100413	1.45300559	3.08066003	4.21113676	5.96266982	1.0911121
B3GNT8	1.90686831	2.99081928	1.92781834	1.73867193	0.70587098	2.07024227	1.5180324	0.49615001	4.43566088
B3GNT9	3.20453258	3.26476065	3.03035678	2.91880485	1.39351921	3.3004412	3.3804799	1.76689945	1.65679246
B4GALNT1	0.21534699	0.40648275	0.26659136	0.89071469	0.16672482	0.30095105	0.21253142	0.20118288	0.10388507
B4GALNT3	2.60655912	1.35697899	2.79851185	2.55530816	1.80005396	2.33008836	2.94548833	3.04930918	2.05380595
B4GALNT4	0.06628528	0.34841595	0.14633884	2.09087816	0.02368296	0.16469176	0.92036435	0.11136779	2.83514439

B4GALT2	3.97456403	3.60216736	3.7046581	3.56860191	2.91443211	3.95679321	4.12040646	4.3618035	3.56031156
B4GALT3	3.65664519	2.42611566	4.00373975	2.98683044	2.96956801	3.27400632	4.23535869	4.03676692	3.63912861
B4GALT4	4.72611113	2.01138464	3.01673793	2.88952049	2.28017337	2.15726233	2.67927397	3.64617666	2.72275345
B4GALT5	5.36732185	4.7858828	5.04703079	5.53955209	4.72498483	5.53263504	4.4908922	3.82827747	5.1392374
B4GALT7	3.53004341	2.15976699	2.76098018	2.35496368	1.77110543	2.4850348	2.24715592	2.47480493	2.80829562
B4GAT1	4.4820807	3.51958243	4.13562321	2.72472045	3.5515627	3.23803566	3.93862574	4.16774814	3.56989154
B9D2	3.04544765	1.9232711	2.13675749	1.69855063	2.34812242	1.98375114	2.05257695	2.05628566	2.27629032
BAALC	0.05369667	0.94889097	0.4051123	0.48351023	0.10818316	0.78424949	0.15018979	0.05853904	0.05178036
BABAM1	3.97888179	3.33507627	3.66414698	3.45857696	3.64283568	3.37345575	3.33558773	3.422928	3.72065251
BACE2	3.63811674	4.09926701	4.62465534	4.6542683	2.92555969	4.24814254	5.12821797	6.75152841	3.52178686
BACH2	0.12479066	0.55311234	0.35585766	0.43957942	0.15845235	0.64904857	0.24541046	0.13814311	0.12732732
BAD	5.09422377	3.84314039	3.83827665	3.67726727	3.26047181	3.71292412	3.74742444	4.34797957	3.38913557
BAG1	3.17354962	2.69114594	4.0791159	3.25614192	3.03697694	3.10088129	3.25258909	3.74811861	3.40030268
BAG3	4.33966938	4.83244492	3.93209259	4.59759758	3.92786441	5.15979882	4.56961436	4.88456001	4.29708419
BAG4	2.50600052	2.56769352	2.33501932	3.50298728	3.17710427	3.31550067	3.39526746	2.42794538	2.57909336
BAG5	3.25622065	3.74652542	3.09678349	4.02473442	4.25939346	3.901213	3.82106609	4.50041418	3.79367491
BAG6	5.5934633	4.98728538	6.05131449	5.16619822	4.86686944	5.48449141	5.1874853	6.0207724	5.87197098
BAIAP2	0.63457228	1.87337119	1.6464125	1.81158281	0.61442342	1.53336345	1.58589299	3.73511105	2.3222463
BAIAP2L1	4.45854606	3.32181228	4.8545599	4.31508853	4.65087442	3.59163952	4.59597156	4.82859333	4.19810411
BAIAP2L2	5.57584766	3.35389468	2.3741241	4.65793865	4.43987746	3.54794923	1.60906232	4.14128544	5.13338395
BAK1	5.07940145	3.90426556	4.69584003	3.67482046	4.29341798	4.71320269	5.03090628	4.80005329	5.03049089
BAMBI	3.64459697	0.89273098	1.91996143	3.87213361	2.76351197	1.67902041	3.56736409	0.84078744	0.46333983
BANF1	6.45784703	6.04314702	6.22263701	6.25744766	6.31395225	6.19377199	6.30208551	5.48195531	6.36397915
BANK1	0.22981868	1.55265139	1.12385235	1.46791411	0.62957824	1.13419619	1.12362025	1.54001411	0.26682146
BANP	1.1509182	0.9981935	1.16082953	1.07943541	1.22566519	1.44971866	1.36183308	1.17506477	1.23636402
BAP1	4.07994442	3.4254482	3.78497222	3.47066097	2.81624924	3.78157487	3.80497671	3.87587791	3.5964133
BARD1	1.31410109	1.46224679	2.12636347	2.54381922	2.69405708	2.26630976	2.5674513	1.6100146	2.08229888
BARX2	1.11992631	1.23395208	1.93446613	0.8605808	0.07762035	0.24195171	1.78316383	0.41704127	2.12177031
BASP1	4.09596683	4.93879286	3.55675187	4.59991323	3.61828532	3.79994456	3.39905955	2.69422091	2.48230107
BATF	3.94091266	2.88622401	3.64704959	2.13319619	4.47349889	2.34002804	1.93499483	1.83319303	3.58685292
BATF2	2.88488905	2.51511977	1.61953952	1.77149473	4.33323816	4.05567008	1.31544238	2.7323374	4.36818293
BATF3	0.92842872	0.72307065	1.17931736	0.92978908	1.19936239	0.93360333	0.54856322	0.48002329	0.44592305
BAX	4.5717645	3.66943212	4.78345509	4.02285679	3.92854615	3.80463317	3.75334576	3.93489948	3.7775663
BAZ1A	2.73710601	3.07592831	3.4513448	3.38057136	4.35344858	3.75229004	3.1330803	3.57732698	3.60369601
BAZ1B	4.27312174	4.12461944	4.09551374	3.99311613	4.34670387	4.21795671	4.2162566	4.07388487	4.03498527
BAZ2A	3.87636368	3.13242057	3.50915278	3.41247012	3.82133428	3.79955793	3.57526614	4.09600442	4.02365968
BAZ2B	0.9053979	1.78273256	1.69275042	1.68247104	1.82291305	1.53700364	1.47671447	1.58051684	2.02281789
BBS1	0.46708888	0.59997326	0.53452953	0.47476131	0.33149508	0.33664756	0.2769511	0.5810417	0.50068538
BBS10	0.74380177	2.26774662	2.71011249	1.68972812	2.44283726	1.63302253	1.150456	2.35060454	1.39849576
BBS12	0.84394311	1.80220942	1.41672332	1.55515348	1.57826232	1.56331901	1.01279355	1.15416646	1.5644891
BBS2	1.72371401	1.96291558	1.63604046	2.02260859	1.04804608	1.83528708	2.02015949	1.63597265	2.30276243
BBS4	1.8552365	2.30986921	3.09935279	2.39486817	2.27415103	1.99513326	1.66549954	1.72849747	1.82551112
BBX	2.22244995	3.14521215	2.45196644	2.78751352	3.12912728	3.41877767	3.22981648	2.43397079	2.52141328
BCAP29	2.41466443	2.9800128	3.68177033	3.00783453	2.53437166	3.27091351	3.06852827	2.20119506	2.45953212
BCAP31	6.67131797	5.81361841	6.13435869	5.58890039	5.91865951	5.47450504	6.24767474	5.89903644	5.76895044
BCAR1	3.39665752	2.85569939	3.14740773	2.94413626	3.02446606	3.63830378	3.83323616	4.47323183	2.95323991
BCAR3	1.55284907	1.99029641	1.6962645	1.69841051	1.94790268	1.53136223	1.8778462	1.90682118	1.35562413
BCAS1	1.34126019	1.29662429	4.06178462	0.9958282	1.72923799	0.37020265	2.06894263	3.4274678	0.80686807
BCAS2	4.63018992	4.75840183	4.89869731	5.45924341	5.42675643	5.17526052	4.76945486	4.83176067	4.80506848
BCAS3	1.74265813	1.3616577	1.84117449	1.24107265	1.1371611	1.41204555	1.08949651	2.2646419	0.85951614
BCAS4	0.56532052	0.73800718	1.82634585	0.7853568	0.54770579	0.70085987	0.23570429	1.04303784	0.46831037
BCAT1	1.3192262	2.34373362	1.64026418	2.97459982	2.20705874	2.67903917	2.47149349	1.102232	0.9121518
BCAT2	3.35664843	1.82475968	2.3335824	2.87930602	3.14308923	2.35958731	2.26489483	3.08593371	3.35603751
BCDIN3D	0.63218055	1.01352797	1.07620155	1.32128845	1.06747419	0.87038197	0.6683617	1.0479382	1.08441695
BCKDHA	1.68647768	1.12486208	1.08631529	1.14100494	1.54915426	0.99865698	0.88931894	1.3363378	1.65480338
BCKDK	4.26090958	4.28935044	4.07081591	3.71102938	3.22802093	4.00192838	3.71876384	4.3511966	3.83126201
BCL10	3.42503801	3.21085253	3.88021585	3.61439325	3.8182052	3.31266682	3.61393989	3.78152446	4.10582575
BCL11A	0.70769152	1.67702786	0.99906269	0.2757573	0.76307863	0.78032943	1.02955583	0.1203331	2.66262266
BCL11B	1.69811702	1.32269631	1.05524925	1.21636799	1.99266792	1.25748288	1.39748901	1.25670476	1.81715029

BCL2	1.0978268	2.00396937	1.22389268	1.2109478	1.39969558	1.79102094	1.99882493	0.70152868	1.55678236
BCL2A1	3.71757436	3.3251113	3.94605794	4.85667332	4.85488618	4.62909702	4.41568689	3.76240794	3.19492462
BCL2L1	4.96529321	4.68517246	6.41368932	5.75513096	5.14366831	5.51111882	4.47009695	6.36135986	5.83923684
BCL2L11	2.70667114	2.2962975	3.40368942	2.57038318	3.58689678	2.5692515	2.53356667	2.32179681	2.11190478
BCL2L12	3.73461967	2.07472078	2.8696068	2.68030081	3.4183699	3.36611932	2.57885192	2.41114859	3.18702572
BCL2L13	2.63748749	2.95060053	2.46865292	2.92201429	2.62126881	2.89878142	2.27578859	2.7932279	2.77771461
BCL2L15	3.24217139	2.12982629	4.28410503	1.7176034	2.74887422	1.41701179	1.77654022	3.10347618	4.09743108
BCL2L2	3.61291896	2.99782801	2.93927587	2.87696263	2.5239483	3.01699471	2.39100515	3.00890146	2.85184786
BCL3	5.58944897	4.34999255	5.39151619	4.23853361	5.00280276	5.15178793	4.840476	5.65705905	5.47895606
BCL6	3.60393983	3.48957425	3.48318565	3.5990893	3.06064992	3.64545349	2.80311504	3.73468725	2.09852992
BCL6B	1.59543783	2.74870069	2.3617949	2.36901513	1.86979668	2.80216463	1.65891494	1.39616236	0.93428405
BCL7A	2.50272537	1.21291124	1.59770431	2.35507877	2.17335345	1.91606209	1.73397604	3.31625043	2.44136661
BCL7B	5.10819444	3.86625459	4.38788297	3.87111628	3.72211382	4.09881351	3.97887879	3.6135295	3.91786107
BCL7C	4.52423102	3.36057785	3.21522321	2.94600012	2.42207953	3.01859013	2.97001027	2.62613342	2.72787557
BCL9	3.03506724	2.50450356	2.45423357	2.90216896	2.39213501	3.04067537	3.12255666	2.86335906	2.8514624
BCL9L	3.48340443	3.14859506	2.10600879	2.76038055	2.89394814	3.20346994	2.87927569	2.65697952	2.79871482
BCLAF1	3.38773375	3.83033298	3.77621422	4.50810787	4.78142785	4.00757088	4.1891271	4.0638093	4.63710605
BCO1	1.48642885	1.16640002	2.49263847	0.36700123	2.98369463	0.47471389	0.20121962	0.97355102	3.76491727
BCOR	3.0505393	2.59860565	2.56336321	2.90644281	3.74791544	2.66508434	3.03248076	2.08862787	2.73824464
BCORL1	2.00502891	1.63020238	1.86064072	1.89822145	0.58327184	2.21888925	1.6956145	1.80125548	1.32741386
BCR	3.82582469	2.82264319	2.39635643	2.63264658	2.25957906	3.09294631	2.78436115	2.51066471	2.71592225
BCS1L	2.12573125	1.91644984	2.55249436	2.59704872	2.7195318	2.60056928	2.289533	2.57575559	3.01248876
BDNF	0.0689553	0.46805818	0.27483864	0.12959977	0.08723157	0.16030145	0.54896165	0.08115516	0.1003774
BDP1	1.60766835	2.06429831	2.26377389	2.35352621	2.98439662	2.41322785	1.90942992	2.58187866	2.47794252
BECN1	3.55920178	3.09529496	3.18647	3.05131609	3.27230782	3.20831576	3.28527665	3.30054857	3.54396473
BEND5	0.09881503	1.40305672	0.36763348	0.82593396	0.09154442	1.07818386	0.19978912	0.07676093	0.00893245
BEND6	0.14270922	1.53122126	0.59094821	0.95690576	0.11576384	0.66720904	0.33936553	0.20580369	0.07676951
BEST4	0.10305459	0.2347719	0.95982549	0.13257886	0.07480339	0.51220163	0.0828367	1.55844954	0.31035402
BET1	1.68898716	2.50446227	2.79440706	2.75200862	2.80431747	2.46504989	2.64807022	2.30868076	2.46527771
BET1L	4.21375159	3.68802951	3.95634756	4.03758934	3.81102416	4.06577104	4.58914987	4.43768761	4.39609542
BEX1	0.08198884	1.26711312	0.14485186	0.45246146	0	2.11086689	0.09762213	0.06360757	0.84733064
BEX2	0.22878601	1.35763621	1.01740212	0.33084937	0.29274105	1.22758504	0.22300596	3.38267254	0.03221722
BEX4	2.07545818	4.30153857	3.52351358	3.32422635	2.90762696	4.1483058	2.68771974	3.73282264	1.53096285
BEX5	0.06699034	1.41180705	1.19388174	2.72006187	0.15978412	0.50499515	0.923121	1.85581786	0.80693602
BFAR	4.33096469	4.76349299	4.27427396	4.59429355	4.55806516	3.98386791	4.13997836	4.55032172	4.36432179
BFSP1	0.80232815	0.60861187	0.46957384	0.86936377	0.31733353	0.36803471	0.34695568	0.48064798	0.50990562
BGLAP	0.05998743	0.05169049	0.24371789	0.15655785	0.21030271	0.10835414	0.05397631	0.25865611	0.37992795
BGN	7.36830516	8.16369086	7.29923671	8.7141697	6.08303687	8.12128991	7.8939511	6.63695362	5.59120757
BHLHB9	0.24817092	0.94928758	1.08039745	1.24857281	0.59230225	0.83635435	0.43963249	1.27629795	0.4188868
BHLHE22	0.36349297	1.38417684	0.54476597	0.51812909	0.53451408	1.15100234	0.15819966	0.32963057	0.05756342
BHLHE40	6.47014003	4.64426691	5.28256831	6.04094045	5.209128	7.1711323	6.46437711	6.3925606	4.9866982
BHLHE41	1.96767356	3.32426224	2.20921637	3.26865733	2.19135715	4.25303626	3.62990026	1.11042446	1.44646675
BHMT	0	0.05242617	0.41351186	0.04887096	0.03778171	0.03384716	0.0123461	0.05866561	0
BICC1	0.84481824	3.76644273	1.97799025	3.16828743	1.00833344	2.70652992	2.16295659	1.31590241	0.72324137
BICD1	1.04545936	1.12330967	0.83077839	1.57354239	1.08398726	1.48970079	1.00003728	0.48491446	0.95302976
BICD2	2.59430505	2.75250767	2.79210928	2.85451937	2.56034448	3.09302448	3.01010304	2.11025217	1.76409864
BID	3.96160043	2.96986521	4.14324893	3.30950234	2.06290524	3.61007717	3.44728139	2.7810148	4.01542746
BIK	3.40901765	2.10259852	4.25530682	3.26120302	2.40900298	2.14039262	2.44858211	4.55564871	5.19650555
BIN1	2.96269049	3.45598614	3.46164791	2.20857835	1.97159135	2.16957236	2.96917749	2.95217669	3.15337468
BIN2	1.98351376	2.34720502	3.01223614	2.1686629	3.58150047	3.22622898	2.0748186	1.46102145	1.88445124
BIRC2	3.17241144	3.46193305	3.7626128	4.18102499	4.11561067	4.03483568	3.59627297	3.07903911	3.81129055
BIRC3	4.54650067	2.75879855	5.1648985	3.5066548	5.78981278	4.56308968	2.19291137	4.12172771	3.40783902
BIRC6	2.43374701	2.83734363	2.88773173	2.72778004	3.75465192	2.95986036	2.97233113	3.15386964	3.18465634
BIRC7	0.58626324	0.18771125	0.6953345	0.12078457	0.46320042	0.26547903	0.04130352	0.24731767	0.2473456
BIVM	1.39919737	2.09681881	1.92749237	2.01991732	2.08798189	1.57288039	1.95392832	1.93107883	2.32748415
BLCAP	2.97577634	2.80776652	3.19158385	3.47074621	2.76228415	2.96565356	4.08925818	2.50135587	3.49417216
BLID	0	0.11627435	0.17253182	0	0	0.03073911	0	0	0
BLK	0.0374495	0.47679439	0.53822142	0.41286664	0.1716742	0.27115791	0.02699896	0.2975638	0.06244506
BLM	1.17693882	0.94025197	1.43562337	1.40700361	2.34921555	1.59203787	1.11637193	1.2834615	1.47805863

BLMH	1.71426343	2.54323377	2.03078436	3.13769082	2.98229444	3.01828759	2.94948628	0.99783082	2.62756759
BLOC1S1	4.95943196	4.17512791	4.2518904	3.78699554	3.94721857	3.79144452	3.40437286	4.09177591	3.84707056
BLOC1S2	3.27983064	4.12037882	3.74111506	4.33617778	3.95660361	3.59673697	3.66847861	3.62266614	3.42568586
BLOC1S4	3.03047157	2.5440513	3.21657047	2.94866447	2.57212172	3.35069086	2.2704915	3.2509731	3.17359755
BLOC1S5	1.88362532	2.43293104	2.44145817	2.36365646	2.63916015	2.3557719	2.31945303	3.20489884	2.40216851
BLOC1S6	2.88911157	3.32286662	3.54090282	3.91404976	3.5047818	3.44918252	3.22069	2.55256287	2.40443041
BLVRA	4.74206718	4.14978175	3.6141255	3.64890213	4.69505413	4.48328945	2.91367415	3.33629916	3.03767415
BLVRB	6.22081745	4.88332449	5.51689934	4.21899289	5.67256782	5.30432849	4.60640343	5.88685776	4.96354843
BMF	3.8149364	2.33661013	3.56181349	2.10991564	1.98337958	2.34900587	1.90948127	1.14061986	2.17947343
BMI1	2.93047401	3.6395877	3.62565205	3.89789685	4.02106209	3.68512808	3.50216639	3.06442765	3.85104066
BMP1	2.84758215	3.24390253	2.53367716	3.82372285	2.49381592	3.35291769	3.55952962	1.99435444	2.11164781
BMP2	5.45492768	2.43832669	4.78224582	2.76547877	3.54422187	2.59863633	3.57256007	2.94029315	2.39276449
BMP3	0	2.10290092	0.38114345	0.16893569	0.01169904	1.4970639	0	0.04105525	1.51227665
BMP4	1.5888771	4.25507795	3.17941801	5.09128723	0.89797686	2.09545249	3.28587355	3.27471916	4.00133257
BMP7	0.1286248	0.44983794	0.99789341	1.55235372	0.14196123	0.2407315	0.48163648	3.39762433	0.25829574
BMP8B	0.34500182	0.84722504	1.49046987	2.14736965	0.57257286	1.15641062	1.30802076	0.83031747	1.87766791
BMPR1A	2.06578736	2.61094631	2.01364965	2.20441266	1.99955252	2.532845	2.0068504	2.48761269	2.09717721
BMPR1B	0.05105125	0.93364754	0.33368447	0.61187864	0.02817887	0.65666286	1.35727758	0.10304884	0.02108999
BMPR2	2.85567735	4.15161941	3.75822823	3.6056783	3.74735527	4.08259658	3.23822552	3.08520049	2.5161537
BMS1	2.63687329	2.70006157	2.60238355	3.39239262	2.69684946	3.26149481	3.10873314	3.21932745	3.46419429
BNC2	0.22423628	2.64983217	0.65779084	1.61878674	0.27286132	1.8063074	0.66826588	0.3201321	0.16631285
BNIP1	2.37081308	1.87757046	1.94758093	2.27461058	2.28812252	2.35713611	2.11989772	2.27223309	2.16267506
BNIP2	1.75866107	3.49557833	3.43690235	3.82828701	3.57157113	3.47980512	2.71367592	2.01776672	3.03032835
BNIP3	1.1468869	2.15414251	2.89505905	2.06135037	1.189713	1.76285017	1.55760811	3.04642124	0.95580273
BNIP3L	4.33983717	4.9303	4.16691288	5.62805298	4.14099785	4.8636694	5.15198548	4.1709838	4.97276176
BNIPL	0.29526567	0.11110439	0.66620952	0.35360967	0.16141759	0.06340948	0.05912092	0.18654227	0.08987833
BOC	0.56253989	2.93640771	0.77717464	1.50580814	0.35977426	2.56545263	0.70698882	0.41742521	0.30928111
BOD1	4.52556953	3.57711172	3.66707891	3.71636084	3.85984048	3.78181396	3.28353394	3.83423292	4.11314308
BOD1L1	1.93328803	2.53670354	1.9315677	2.32775498	2.49600723	2.30290211	1.87640272	2.73168418	2.48881013
BOK	3.78033099	3.7115941	3.76162552	3.38567522	0.70714204	3.45226062	3.95433243	4.76576188	3.98847733
BOLA2	0	0	0	0	0	0	0	0	0
BOLA3	3.05449599	2.0305571	2.37000428	2.24225235	2.88857798	2.27177073	2.49644057	2.13234096	2.99867746
BPHL	1.09809005	1.74231853	1.86257208	1.73371432	1.54316243	1.63620106	1.95331057	1.72143761	2.05935588
BPIFB1	0.44194857	0.30253553	7.50058993	6.20120856	3.96660543	0.29921166	1.13724267	8.77857765	0.17435549
BPIFB4	0	0.28534704	0.30867592	0.11992183	0	0.00545972	0.02649079	0.45823906	0
BPNT1	3.93760999	3.05028413	3.63670132	3.03230669	3.11747485	2.78484893	4.88737382	3.60400602	4.05856873
BPTF	2.9572374	2.80172458	2.89094881	3.01633527	3.335327	3.00945295	2.49372301	3.60142194	2.74680073
BRAF	2.04519628	2.14252449	1.86498849	1.87426818	2.16052853	2.16529033	2.13039418	1.95557363	1.88920518
BRAT1	3.67929692	2.22621376	2.97278792	2.16393761	2.31757262	2.65920026	3.14105788	2.76120875	3.05489617
BRCA1	1.94267141	1.51067002	1.75628221	1.79243824	2.87825034	2.12455947	1.79227593	1.62893398	2.19577686
BRCA2	1.15227443	1.0057927	0.85752929	1.34758546	2.38823395	1.55759138	1.24870402	0.82529446	1.64231354
BRCC3	3.77048438	3.94799426	3.41241001	3.59553938	4.77198748	3.88753341	3.99426072	3.74300213	4.29117727
BRD1	3.70048388	2.48878492	2.31367069	2.69587045	2.78945379	2.8146539	2.60269659	2.53272397	3.35365001
BRD2	5.07815565	3.89474894	5.48677325	4.86322879	4.87425872	4.94413338	5.13271418	5.31092166	5.01493809
BRD3	2.54378684	2.82324427	3.08129828	2.65002864	2.02788342	2.76045703	2.43782078	3.09163013	3.14474455
BRD4	3.77663245	2.90475287	3.09166988	3.27148611	3.45084988	3.33054611	2.80523146	3.09803775	3.19405369
BRD7	3.21069334	2.85731564	2.90712122	2.65386364	3.21007159	2.64150614	3.14702771	2.95498423	3.20722664
BRD9	1.77958543	1.49418538	1.7722024	1.78305423	1.52579018	1.62245412	1.55581151	1.92571735	2.02696768
BRF1	0.88204498	0.70313518	0.72065309	0.58245886	0.72371796	0.61651037	0.74004523	1.07133697	0.98969229
BRF2	1.95381599	1.04929168	1.43970319	1.66154972	1.29556915	1.5691621	1.9362599	1.66586789	1.76583773
BRI3	5.45999512	3.9576793	5.04291311	3.63963989	3.46410752	4.15941165	4.31470105	4.36653881	3.5579387
BRI3BP	4.29169945	2.17060246	3.99282379	3.3134287	3.34249252	3.25256574	3.99949817	4.24208712	4.04697398
BRICD5	0.77865139	0.26478032	0.39552516	1.03915274	0.61395457	0.38676692	0.66723841	0.26422513	0.81441854
BRINP1	0	0.34118236	0.03989914	0.10785271	0	0.32143492	0.01113041	0.52488842	0.0243224
BRIP1	1.11326577	0.67913254	1.52474832	1.42143141	2.29187113	1.30615207	1.31573086	1.39853312	1.5788766
BRIX1	2.38809396	2.75596359	3.01049683	3.43853466	3.71792796	3.27358762	2.86630561	1.98874029	3.33130473
BRK1	6.95359793	6.77950438	6.40989846	6.84696977	6.73748534	6.65399443	6.76979281	6.25168604	6.48765857
BRMS1	4.73734649	2.8385219	3.82095699	3.0331759	3.43119531	2.98326971	3.6411645	3.68162768	3.74206976
BROX	2.98902359	3.40323551	3.68391209	3.95717414	4.09906863	3.61754931	5.01848672	4.19150762	4.17638149

BRPF1	3.16141077	2.02031412	2.23228571	2.41954614	2.61892536	2.53925177	2.62750734	2.53289049	2.84004589
BRPF3	3.11294679	2.86086811	3.33299754	2.80547003	3.01033596	3.01477564	3.35749866	3.97643172	3.59428556
BRSK1	0.87598552	0.60752535	0.52320166	0.66714545	0.30450474	0.68395553	0.21588338	0.35768561	0.39461384
BRSK2	0.02096526	0.87025717	0.08156481	0.92613414	0.04798002	0.98565256	0.73064006	0.44325228	0.28831383
BRWD1	0.80665219	1.81221004	1.60778056	2.20266866	2.27637318	1.92489269	1.76049246	2.39363705	2.1835428
BRWD3	1.52537199	1.77255617	1.79972343	1.90077208	3.58192714	1.75265779	1.65381442	2.52914855	1.88746402
BSCL2	1.85541345	1.66012884	1.38969524	1.48758127	1.22066955	1.5572679	1.15825004	1.2330096	1.89244215
BSDC1	3.64163506	3.30940894	3.91735999	3.5330686	3.29349275	3.42896838	3.44800453	3.0980702	3.85869551
BSG	7.48871502	6.829162	6.75043493	6.36926857	6.5235649	7.14518034	8.68079046	7.61736404	7.33757459
BSN	0.11439239	0.12206417	0.12431191	0.12513337	0.18055879	0.11953093	0.02532388	0.16197371	0.08482816
BST1	1.06009394	2.7474133	2.29133914	1.9307336	1.28767438	3.07527571	1.46510145	0.85099186	1.40040729
BST2	7.87827236	7.20402904	6.68537656	6.79325673	8.07983676	8.04606454	5.50137115	7.11302947	6.57264109
BTAF1	1.90512167	2.47836428	2.64660565	3.13090783	3.40483405	2.8841823	2.37803664	3.04151908	3.35737105
BTBD1	3.65092234	4.24183207	4.59808706	4.87325866	4.22102536	4.57761992	4.22222386	4.22731868	4.64437276
BTBD10	2.71649881	2.99345036	3.6273376	3.68727236	3.78862614	3.50992633	3.75764156	3.80087437	3.27916814
BTBD16	1.20284658	0.56082521	0.63574441	1.51831759	0.24014037	0.24978441	1.24497201	0.28991555	0.17945227
BTBD17	0	0.18432029	0.02888356	0	0.03684137	0.05816112	0	0	0.06231386
BTBD3	1.75049391	3.41074484	3.55083497	3.90946251	2.87418185	3.194802	4.0141978	2.67271147	3.24379475
BTBD6	3.85556471	3.63752291	3.50799975	3.84617341	3.15141292	4.35745475	3.89556796	4.73835134	4.29334565
BTBD7	2.20160293	2.62819047	2.56042889	2.43254921	2.18968039	2.59340487	2.32547031	2.95132964	2.49384462
BTBD9	1.81951113	1.47965605	1.23916398	1.66195456	1.44614754	1.8598414	1.8160243	2.21904776	1.81846202
BTC	0.4231906	1.92226674	0.86125062	1.71693846	2.90670497	2.03386654	2.17031473	1.91964582	2.03989226
BTD	3.20555895	2.76081675	3.03713761	2.51339643	2.71060599	2.61636123	2.77960514	3.03881572	2.44690613
BTF3	7.63252015	7.19275793	7.63107242	7.65105399	7.81158038	7.41812346	7.0294731	7.67253385	8.03022006
BTF3L4	2.72525777	3.04512265	3.3316675	3.5814171	3.69482924	3.28608337	2.5537352	3.12302647	2.65654799
BTG1	4.93064422	5.07360606	4.90527402	4.73522645	4.5928679	4.87697981	4.61739663	4.32361722	4.01440681
BTG2	4.98994294	5.86612837	4.93093629	5.19442882	4.3750846	6.48822593	4.57525437	5.6338263	5.54272358
BTG3	2.27979296	3.70434445	4.50520737	4.85528067	3.79104531	4.44288077	3.88788967	4.65520123	3.52032627
BTK	1.29082676	2.02092681	2.58747267	1.54993086	2.4252054	2.11005495	1.63481886	0.83959718	0.82541077
BTLA	0.34859652	0.71879118	0.7705626	0.39578979	1.13453781	0.39652892	0.17223032	0.16157996	0.17083438
BTN2A1	2.71148929	2.14271331	3.96681561	2.81047211	3.51885538	2.66071267	2.80521578	3.17583433	3.30576115
BTN2A2	1.49377741	1.25755412	2.16720561	1.51099903	2.60031692	1.65201324	0.81061881	1.45865971	1.36586869
BTN3A1	2.89962059	3.13402982	3.71308535	3.12636674	4.32186306	3.78946094	2.25899753	3.65721032	4.30994351
BTN3A2	3.26553886	3.33520911	2.135047	2.80210122	3.17768805	3.43080825	2.59657236	3.36197738	3.94525288
BTN3A3	2.16861714	3.51335604	3.11290101	2.74074142	4.20483756	3.05312054	2.047891	2.80745403	3.20088309
BTNL2	0	0.12262217	0.37670775	0	0.03026836	0.00909302	0.01478513	0.11048199	0.07624685
BTNL3	0.29603989	2.20710328	0.84826569	0.34857619	1.18219751	0.19956936	0.37571045	0.17337065	2.56549361
BTNL8	0.62365229	2.66521965	2.82067774	0.57581589	2.37848984	0.25002773	0.28191272	0.0527199	2.62857863
BTNL9	0.59303056	0.63583241	1.3456142	2.12906392	0.24435924	0.81739352	0.61084898	0.157654	1.15473988
BTRC	1.82387419	2.17583141	1.82896718	2.36728246	2.34633676	2.34407188	2.07320744	2.55092211	2.29083523
BUB1	2.51173859	1.8529386	3.22227947	3.09003067	4.39779093	3.26489289	3.32984682	2.49749751	3.02406282
BUB3	3.73721452	3.16071833	3.99927037	4.4824227	4.50349138	4.02959681	3.86524538	3.76202506	4.43472618
BUD13	3.48144217	2.7384011	3.07693796	3.18844555	3.5229785	2.94075159	3.40681845	2.30496214	3.41284842
BUD31	4.98081081	4.35039285	4.74832319	4.29462322	4.54676303	4.16431117	4.36318807	3.856196	4.07891909
BYSL	3.41165773	2.32484401	3.54044656	3.2087374	3.27929366	3.34803933	3.24526403	3.77233018	3.54517735
BZW1	3.24535337	4.04868001	3.9844529	4.42137215	4.16654054	4.10112944	4.44738378	4.43456172	4.00647385
BZW2	4.72320283	4.16029258	4.94336707	4.92252224	5.24282897	4.39930557	5.5286684	4.61553697	5.03467671
C10orf88	1.33378979	1.42616686	2.22154681	1.93467741	2.11607598	1.9630654	1.23771477	1.75882727	1.73048852
C11orf1	2.13616665	2.39934165	1.9100875	2.59362792	2.53542259	2.15461038	2.89183397	1.76948109	2.59363916
C11orf24	3.88995738	4.24956	4.20572407	4.0845552	3.47870594	4.06923572	4.59865185	4.03379592	3.87731678
C11orf45	2.05169456	0.57161689	0.46934434	0.68004974	0.53838383	0.71244879	0.84997072	0.10473196	0.60119848
C11orf49	3.12845272	2.21367426	2.26993333	2.44255688	2.24099693	1.96799549	2.92419831	1.38344636	3.32065917
C11orf53	0.97405048	0.32443314	1.84395357	0.13831668	0.01851281	0.83646826	0.78898217	2.94756499	0.13642177
C11orf54	2.35338487	2.99093418	3.15115586	2.07401505	2.07204034	2.32250408	2.63689344	2.76108877	2.53596873
C11orf65	0.07063575	0.23960372	0.27161477	0.3507793	0.22380751	0.16498917	0.14141482	0.19018069	0.14961388
C11orf68	4.53822194	4.27229168	3.9169659	4.22788289	3.30600358	4.24147126	3.85957553	3.77317439	4.44150556
C11orf71	0.87742106	1.84313832	1.69825644	1.8399098	2.08008288	1.64509181	1.17824383	1.17323313	1.94748552
C11orf74	2.03633318	2.81099159	2.18699186	2.8906553	1.44968937	3.07128797	1.64321893	2.59925627	2.43192726
C11orf80	1.71720185	1.39691966	2.20973112	2.25698685	1.96714116	1.52903593	1.57730106	3.58973044	2.12000604

C11orf98	2.95414978	2.14593534	2.32546123	2.60282592	2.52473183	2.19495397	2.64011624	2.65826698	2.66206048
C12orf10	4.55043839	3.07027072	3.28658236	3.54892702	3.22093119	3.53543193	3.04869373	4.17898132	3.79399867
C12orf29	1.27782961	1.74977941	2.03437208	2.37946935	2.29603873	2.20602925	1.95502326	1.83800157	2.25498181
C12orf4	1.52205397	2.44709644	2.56428752	2.46629157	3.15267257	2.63225632	2.14302603	1.64480629	2.35504241
C12orf45	4.1364273	2.39998199	2.82011471	2.87015095	3.13620553	2.83461069	3.11731595	3.16328322	2.88871983
C12orf49	3.31825312	2.52057823	4.0336331	3.44893617	2.66450919	2.8968813	3.74123727	4.12820053	2.78253636
C12orf65	2.01662873	1.59867091	1.66799236	1.96613487	1.79921689	1.6659182	1.68048353	1.59523134	1.68087164
C12orf66	1.18645597	1.49995251	1.47336696	1.41544959	1.49359922	1.6690372	1.02753467	1.6732965	1.71460561
C12orf74	0.24034941	0.16627879	0.76643295	0.02742699	0.2908385	0.01126088	0	0.34625215	0.29843473
C12orf76	0.72104838	0.7979911	1.30903242	1.18552094	1.25317858	0.78236889	0.46867202	0.72753756	0.86021864
C14orf119	4.19786321	4.33750296	4.63003129	4.51578187	4.35662007	4.19663324	4.06371086	4.19154104	4.54057984
C14orf28	0.93755518	1.46273762	1.2098049	1.66583628	0.93373747	1.3031586	0.69799494	1.12049143	1.18364015
C14orf93	1.99379181	1.59075772	1.53236439	1.52370742	1.47449559	1.52451955	1.5108035	1.73215033	1.71980936
C15orf39	3.5555131	3.17657705	3.7512982	3.14511487	3.53413926	3.57881704	3.18537245	2.73818192	3.74772803
C15orf41	1.03076554	1.2142883	1.73714506	1.35586182	2.36726601	1.47631555	1.0063285	0.93110758	0.75558687
C15orf48	5.79993579	2.02103883	6.16056723	4.87468801	6.58665792	6.05773	4.98474347	4.3418936	5.23206173
C16orf46	0.55366492	0.36314973	0.55439824	0.71743312	0.50506464	0.59621503	0.569194	0.58094973	0.49932898
C16orf58	3.58106485	3.70424381	3.11138872	2.99956834	2.61022352	3.42415762	3.74880607	3.76592046	3.70307953
C16orf70	1.80154768	1.98972154	2.40815376	2.4331405	2.36187631	2.41719875	2.20871841	1.97807909	2.46322146
C16orf72	2.21241541	2.41919215	2.03583386	2.22603732	2.10193202	2.32253583	2.38741858	2.42455668	2.0449592
C16orf86	0.37465808	0.93046077	0.7688487	0.63288431	0.39304278	0.67972449	0.46505489	0.32232996	0.17143633
C16orf89	0.1974343	3.87291134	1.574254	1.91798577	0.14457522	1.34240054	0.78598313	0.42131525	0.09850645
C16orf91	4.10465786	3.4852644	2.70221378	3.11622469	3.29126316	3.0076483	3.7368296	2.88609336	3.86467498
C17orf49	0.65695545	0.85550242	0.69428043	0.82602177	0.36755551	0.92379014	0.55761837	0.31987866	0.72403768
C17orf53	2.31750987	0.65353447	1.37701848	1.2913108	2.05985014	1.52814365	1.35896998	1.22336277	1.48006586
C17orf58	2.73382423	2.4000454	3.03896334	3.15208281	3.02058445	2.87710695	2.71146063	3.734373	2.54296525
C17orf75	2.36337833	1.2655172	1.66667243	1.80863309	1.99851371	1.57885428	1.28282388	2.16589659	2.11090599
C17orf80	2.36451414	2.29843881	2.84792865	2.48603303	2.50649479	2.24129657	1.88299309	2.97090996	2.54620825
C17orf97	1.20478781	1.04442842	1.18020896	0.89193707	0.56231013	1.34164238	1.43695161	0.92735175	1.96734946
C18orf21	2.04047781	2.45922441	3.1090922	2.98509652	3.51810789	2.64124896	3.00258301	2.1875346	2.94500477
C18orf25	1.29021054	1.60904826	2.05060073	2.08291272	2.45516384	2.1209482	2.59356119	1.28731303	2.06908807
C18orf32	0.93984169	0.96619539	1.28139791	1.27244238	1.13609817	1.02240571	1.37393772	0.86044321	1.38388719
C19orf12	2.42197267	2.15270944	2.42334571	2.09771385	2.55124897	2.31159727	1.56413765	1.79618912	2.02468036
C19orf18	0.07753955	0.17186357	0.93924046	0.32014684	0.42240514	0.15261651	0.13640068	0.44550276	0.6063785
C19orf24	4.89491749	3.22344506	4.04985379	3.2605547	3.13615892	3.9915569	3.92710099	3.9909365	4.04603955
C19orf25	3.23151936	1.66386517	1.95136264	1.68753046	1.90318496	2.14391085	1.88455852	2.0407689	2.55622988
C19orf33	3.40892428	4.48164299	5.20708322	5.61029963	5.09455943	4.85551606	4.58135508	6.90592148	6.14025863
C19orf44	1.21408795	1.14042551	1.14913583	1.07069208	1.20315922	0.81385834	0.54093986	1.16484893	1.11660679
C19orf47	2.75971566	1.36019585	2.00627609	1.63879477	1.99796042	1.99126896	1.91219096	1.85930189	1.83202595
C19orf48	4.19266526	2.77670179	3.84032615	4.00815945	4.41690826	3.48243989	3.21496675	3.82985486	5.05085687
C19orf53	6.93269778	5.49859587	6.04091584	5.46203464	5.88509096	5.47339929	4.91464398	5.76797199	5.83684602
C19orf54	3.42464351	2.23266204	2.36087312	2.76021342	3.18612431	2.28620122	2.74303313	2.24187415	3.24646443
C19orf66	3.54434154	3.02501908	2.92532811	2.89153144	3.07703681	3.06637069	2.52655119	2.66097701	2.80007602
C19orf73	0.9128814	1.36476897	0.551962	1.02780056	0.79252069	1.06029896	1.43685569	1.06086434	1.53198232
C1D	2.60721429	2.96357861	3.2695865	3.10312332	3.41599579	3.0823561	2.94831319	3.08401071	3.07632385
C1GALT1	2.47518353	3.46744304	4.86536335	3.91940041	4.00215925	3.119057	4.3961942	5.4243966	3.26838991
C1orf109	1.93362994	1.61469335	2.92328352	2.51525736	2.21073205	1.92393525	1.90774109	1.54012049	2.3325233
C1orf112	0.84131132	1.03904623	1.50653818	1.47138982	1.9931369	1.47644124	1.95847166	1.37618211	1.47175037
C1orf115	0.31528033	2.99534099	2.40725706	2.68003243	0.52379564	2.27499684	2.20507385	3.30042001	4.0515212
C1orf116	3.62212657	3.02087507	3.85042642	4.33212416	4.20147711	3.63527543	4.81232818	4.36916117	4.47322376
C1orf122	4.21604314	3.94046221	4.35482935	3.35981619	2.83419636	3.77187825	3.27237259	3.17611909	3.31319156
C1orf131	1.69603552	1.88435795	2.55302503	2.34706152	2.58417621	1.74305788	2.53868661	2.65305548	2.71771354
C1orf159	2.1815229	0.70030345	0.90889059	1.52221347	1.33415441	1.23309688	1.3409784	0.94990605	1.8277546
C1orf162	4.20551541	4.22298873	4.20886004	3.91796518	4.65781311	4.44803456	3.47619463	1.56350759	2.02848693
C1orf198	3.70010601	3.58920715	3.6356607	4.25449311	3.36701982	3.86840738	4.75884773	4.84031537	3.79410266
C1orf21	1.4726424	3.05475534	2.32359819	2.34270312	1.78708153	2.1834239	2.37271254	0.4115745	2.52551667
C1orf210	5.03608309	2.79824626	4.22843501	2.40022272	3.54547355	2.99632822	3.13755323	4.03457565	4.30014282
C1orf216	2.04943649	2.09678655	2.48455223	2.15909851	1.50136927	2.69451961	1.81256525	1.63385929	1.25062314
C1orf35	2.43710093	1.34960641	1.76641229	1.51122169	1.1859839	1.69247036	2.0792405	2.18890913	1.62029558

C1orf43	6.236788	5.46146995	6.57813978	5.85627688	5.57716265	5.86455504	6.58020756	7.03018589	6.10781688
C1orf50	2.55981823	2.1827365	2.54973737	2.67155825	2.27410639	2.63707639	1.99061974	1.65782969	2.3452047
C1orf52	2.34826178	1.89549976	2.60287451	2.5653595	2.2697283	2.16152139	1.81931901	2.06937887	2.12510002
C1orf61	0.01179024	0.01349886	0.1365865	0.03334893	0.03066722	0.00759205	0.00353897	0.02151217	0.01393375
C1orf74	0.53369803	0.64930807	0.75340145	0.92808911	0.86181293	0.80982696	1.0776319	0.95571988	1.07228541
C1QA	8.57062945	8.033821	7.87854949	6.62136334	8.45886466	8.4662868	7.80803887	5.40138318	6.25946449
C1QB	8.93950998	8.70369342	8.94921865	7.34940471	9.75602951	8.94966577	8.79349565	6.21504396	7.26606057
C1QBP	4.84657691	5.31713396	5.89206096	6.06618594	6.63641865	5.99776916	6.07176245	4.88000193	6.90155247
C1QC	8.65846547	8.63283053	8.81857212	6.92451951	8.88131216	8.65826438	8.16945762	6.15063107	6.89487327
C1QL1	0.53472282	1.19921343	0.68347707	1.14870931	0.68963805	1.5916928	0.32986494	0.7062417	0.45326156
C1QTNF1	2.01225149	4.4061555	2.19280062	3.22628441	0.96273399	3.28768522	2.81250843	2.79228706	4.35421941
C1QTNF3	0.62000664	2.46567766	1.4703886	1.78646988	0.36913233	1.8750872	0.85076116	0.7443585	0.46335998
C1QTNF5	0	0	0	0	0	0	0	0	0
C1QTNF6	1.8660592	2.3698799	2.1749601	3.46054108	1.72941679	2.69995803	2.41566139	1.10279268	1.09855225
C1QTNF7	0.01945181	1.15942618	0.55010594	0.98153183	0.08964902	0.74985557	0.14840294	0.22332051	0.08656354
C1R	4.67066779	7.26745741	5.41162603	5.96053412	4.9423873	6.34452224	5.20558728	4.52579165	3.28206552
C1RL	3.44092972	3.97620573	3.06223754	3.61477591	3.21184487	3.34345013	2.45936615	2.59478936	2.93692762
C1S	4.37555845	7.53881534	6.09077405	6.64890755	5.40487084	6.8974908	5.42659094	4.44616657	3.36374963
C2	2.98093079	3.9163555	3.7026397	2.40922242	3.53858542	3.36295188	2.96375877	1.41346707	4.20645646
C20orf144	0.0868763	0.07495589	0.22884185	0.25386459	0.04525266	0.10059634	0.11170049	0.12130065	0.20445555
C20orf194	1.97817902	2.29177634	2.68570491	1.83713982	1.77002598	2.12150094	1.0614895	1.16073499	0.48976498
C20orf27	4.86846646	4.32965775	4.29487793	4.53861425	4.32031479	4.55665369	4.37149339	3.42497598	4.88415358
C21orf58	0.68560169	0.45730786	0.54609583	0.64842465	0.81902047	0.43404029	0.50409428	0.63356305	1.07159324
C21orf91	0.67408558	2.17798868	2.45686219	2.33896712	2.82123684	2.37630074	1.50383749	1.48728555	1.37935071
C22orf15	0.0848135	0.11535294	0.36593946	0.17731789	0.15303409	0.07504572	0.10096679	0.09762731	0.04520202
C22orf23	0.38699607	0.62413212	0.42369219	0.1702526	0.29883622	0.39938542	0.18666384	0.17255327	0.20184719
C22orf39	3.13704234	2.17899409	1.98448675	1.88165444	1.66639141	2.15739866	1.51601881	1.19101361	1.9766166
C22orf46	1.26871742	1.55704405	1.40995303	1.65578335	1.47221276	1.45394637	1.19601482	1.23203573	1.21575872
C2CD2	1.18184418	2.30930027	2.11753371	2.13187235	2.5911578	2.68012794	2.49863741	2.24410886	2.59933992
C2CD2L	1.72953369	0.8448133	1.33279742	1.23376356	1.57158329	1.27863853	1.61084609	1.2437425	1.69491447
C2CD3	1.84328707	1.62075004	1.7758993	1.56870084	2.23235545	1.91607942	1.65792328	2.07261103	1.99115136
C2CD4A	4.75697024	1.05621331	1.81410579	0.86954468	2.17745321	1.06617194	2.18343051	1.80319003	3.70671242
C2CD4B	6.25401214	2.64195836	2.60267464	0.72481872	0.59693689	2.18973264	1.76713969	3.14660476	2.92147036
C2CD5	2.35683569	2.82330595	3.07938607	3.09477673	3.04165018	2.89193322	2.94330665	3.18996457	3.34549553
C2orf40	0.04127072	4.94349487	1.12040436	2.39659611	0.01283856	2.22442336	0.08515261	0.29124523	0.15627243
C2orf42	1.18493079	2.00057878	1.92997948	2.22341818	2.03397425	1.80236398	1.29750034	1.88894895	1.66743951
C2orf49	2.42945345	2.56914399	3.30902571	2.87481582	2.96866152	2.37064341	2.29306781	2.52093444	2.91383868
C2orf50	0.10753314	0.06935958	0.23896517	0.13489522	0.17941257	0.16356263	0.00740647	0.35056221	0.01942966
C2orf69	1.90312113	2.54183603	2.45580472	2.87085082	3.18413288	2.90751769	2.68353533	2.23505245	2.41757227
C2orf76	1.96523822	2.03351593	2.36127041	2.73742326	1.91635295	1.7975639	1.54814908	2.02033051	1.66457111
C2orf88	0.76847628	2.45689086	1.42217847	0.85295169	3.02299109	1.44006766	1.07516725	0.4914786	1.15057192
C3	7.47311604	8.14252836	6.71666647	6.66242058	7.3976002	7.32900369	5.56395189	4.89961176	2.79080475
C3AR1	3.01189207	4.55111644	5.18094585	4.4360258	5.55139707	4.97658132	4.72902338	3.54994956	3.60276409
C3orf14	0.31638221	1.15795588	1.19242921	1.27896024	0.84875802	1.14334747	0.50574949	2.17871811	0.19770538
C3orf18	0.64153579	1.14519752	0.79584516	1.19695046	0.42758197	0.93477955	0.58319484	1.62590865	0.48187359
C3orf33	0.75410805	1.32888416	1.74497327	1.91449756	1.73708881	1.69984769	1.83110515	2.03329023	1.54114906
C3orf35	0.06948125	0.05989827	0.44614902	0.42268086	0.34886276	0.19918351	0.27699211	0.17220029	0.29758902
C3orf38	2.5947213	3.021944	3.20601879	3.14039061	3.76080115	3.18874559	3.15169589	2.98348785	3.01405222
C3orf52	0.85244292	0.60321604	2.54989703	1.73154643	2.22668551	1.26842569	1.51780082	2.16867481	2.11806965
C3orf70	0.1906407	2.64836879	1.05401208	2.20350898	0.34967359	2.18088759	1.58069	0.39159289	0.33201968
C4A	0.34539435	2.71364514	0.69307874	0.87049942	0.36494254	2.65056637	0.79227441	0.75168038	0.77477188
C4BPB	0.69102694	1.8415339	1.93173545	2.20528673	2.59123168	0.88876259	1.68114188	1.93839117	1.67598191
C4orf19	3.36997307	2.20954941	4.70650031	2.7397108	3.59510238	0.91170059	3.29199735	3.14606286	2.7253925
C4orf3	4.6192098	5.75900449	4.87214767	5.67658889	5.0812917	5.35407886	5.1468014	4.51008904	5.40054188
C4orf33	1.02397631	1.80990372	1.53695446	1.97360349	1.94428477	2.35620438	1.89630032	1.70713213	1.54003483
C4orf46	2.26285926	1.99813782	2.58862612	2.86590053	3.45789009	2.86802049	2.40306158	1.98266848	2.93946897
C5	0.09707431	0.51122436	1.6287326	1.01323828	1.37497303	0.67638824	0.27023789	1.22914717	1.74398935
C5AR1	3.82696201	4.82433343	4.21540848	4.47153934	3.89837155	5.21719436	5.10477484	3.76029671	2.76076693
C5orf15	5.35569697	4.81094711	6.33438867	5.92341721	6.0141482	5.44496728	4.34995868	5.61276924	5.48363798

C5orf24	3.05997548	3.71323291	3.95827892	4.04938556	3.4590619	3.87141228	3.37861696	3.76147669	3.51637498
C5orf30	2.07531218	2.20202596	2.16787364	2.56842593	4.33034672	2.68752898	3.19911003	1.78450717	2.9129036
C5orf51	2.31762645	3.29710877	2.96645787	3.63569589	3.47602328	3.35427717	3.03657149	3.1398391	3.47829289
C5orf56	0.75963394	1.04868993	0.90492706	0.6918099	1.90497438	0.94752317	0.4103008	0.49443991	1.46795559
C6	0.35297463	0.65166385	0.65779888	0.71898296	0.64902817	0.16029426	0	0.08628865	0.00806219
C6orf106	5.29871797	4.30002261	4.43914968	4.31366533	4.7960691	4.7649577	4.9824212	4.90016101	4.10768576
C6orf15	0	0	0.40074191	0.16305034	0	0.03481522	0.09264553	0.11823579	0.01670712
C6orf203	1.91336942	2.47946849	2.09788457	2.78723704	2.62279263	2.18772631	2.69628026	1.97680852	2.98425529
C6orf226	3.04101698	2.95175314	2.89670517	2.28432095	2.11327603	2.52991661	2.53457308	2.24468323	2.49637634
C6orf47	3.91018488	2.64926379	3.66116958	2.54689914	2.73803265	3.29358467	3.428894	3.63734088	2.97290747
C6orf48	5.09772878	4.78248376	4.93976673	5.43501252	4.33567661	4.4882806	4.59035925	4.47967099	5.50060932
C6orf58	0.02362573	0.26135788	4.69126519	3.88740986	0.82621698	0.04310992	0.08311291	1.0710142	0.41120146
C6orf89	3.85230764	4.06976069	4.08644048	3.74963894	3.5192071	4.04246755	4.21228618	4.30473531	3.71118463
C7	0.06147163	5.90366073	1.39798052	4.18898486	0.4346573	4.79564263	0.32743528	1.03133506	0.32680339
C7orf25	0.18903032	0.42549815	0.26577941	0.47227675	0.28168151	0.29703178	0.20035796	0.24592682	0.15635755
C7orf26	4.21175106	3.12129978	3.94208008	3.18941572	3.40378996	3.23430471	3.34838281	2.98036105	3.54828026
C7orf43	2.89258988	1.76952863	2.85497986	2.28095202	2.4490909	2.1722897	2.28388739	2.27610873	2.72001571
C7orf50	4.81641224	2.77876027	3.8367227	2.59034803	2.34426336	2.79653884	3.27517078	2.62743269	2.5184122
C8orf33	3.88435997	4.13775804	4.79297089	4.39026136	3.52750997	4.36317875	4.37880956	4.65186476	4.25325085
C8orf44	0.700653	0.90030491	1.16257505	1.32916166	0.95000187	0.7307835	1.02736945	0.67326375	0.90660716
C8orf48	0.04958104	0.9644412	0.32493039	0.98980828	0.13346872	0.56103511	0.26163138	0.24934002	0.11463815
C8orf58	0.60927516	1.11419733	1.0931838	1.66464326	0.96872005	1.37417319	0.85353449	1.39133742	0.65668471
C8orf76	1.70620253	1.72724921	2.45888801	2.06853285	2.03911725	1.76729248	1.99462802	1.756949	1.77714993
C9orf116	2.66748535	0.92046392	1.27483517	1.25723772	2.15047493	1.02201271	0.80434744	1.83165571	0.9773294
C9orf152	3.95774139	2.64585224	5.09385122	3.68359269	2.04481129	0.4723785	2.43776375	3.89652183	5.75744929
C9orf16	6.85741523	5.15869121	6.18380011	4.44388175	4.0924262	4.64466984	4.93234199	3.98539873	5.54015095
C9orf3	1.78580018	2.13482657	2.43578841	2.75807873	1.80816506	2.04902609	1.59727885	1.14346875	1.27960746
C9orf40	1.11931581	2.27771771	1.25118361	2.15020255	2.66527568	2.16931556	2.07389487	1.43004708	2.3768769
C9orf43	0.04697147	0.21944667	0.65426982	0.47558288	0.25130291	0.62205878	0.18735181	0.2474103	0.41825616
C9orf50	0.10831473	0.15848748	0.47954507	0.11663028	0.04098934	0.18587557	0.05288579	0.30380305	0.03504645
C9orf64	3.8381427	2.69419832	3.0778192	2.8685671	3.47313166	3.53960359	2.86136938	3.39537887	2.50856457
C9orf72	1.24803959	1.63029865	2.29685729	2.71925726	2.34971564	2.21063386	1.53770518	2.167787	2.05077807
C9orf78	3.63281313	3.74946806	4.0985979	3.9236289	4.0337924	3.82827466	3.57417442	2.86814138	3.6765301
C9orf85	1.24891071	1.53026499	1.64719398	2.0714434	1.59230579	1.40391481	1.30197245	1.28629983	1.69013938
CA1	0.01780752	0.06030205	0.66287161	0.04237138	0.15178193	0.03576197	1.78646029	0.12874577	0.27759054
CA11	1.98045913	1.15982224	1.75102935	1.91668336	1.76233674	1.41601543	1.20899908	1.47666475	1.20904886
CA12	5.14265683	3.86253649	4.93243622	3.98161325	0.80834067	3.63286355	3.31341474	2.43621188	1.57314375
CA2	7.77425695	3.08469704	6.33648127	4.71857351	7.25609249	5.06633593	6.67386401	8.5187937	2.64906842
CA3	0.42116688	0.41358663	1.49019141	0.85882784	0.93826538	0.46225979	0.1607038	0.9016991	0.52868796
CA4	1.40980852	0.23766501	0.62489462	0.18593462	0.00923264	0.47964132	0.05301432	0.15376165	0.22628136
CA5B	0.24707006	1.06958423	1.22312867	1.03654422	0.96198339	0.73417644	0.83263796	1.09870495	0.656861
CA8	5.18259249	0.24508005	3.28653456	0.54979368	0.11200795	1.0744005	2.77378819	2.01757786	0.63112683
CA9	4.58270365	2.53610033	2.37451803	2.85676139	0.52404494	3.40730484	3.13244067	5.10388929	5.09878201
CAAP1	3.21165399	2.93983589	2.91450928	3.68788776	3.51213826	3.11468232	2.77505649	3.2993596	3.96430549
CAB39	3.80671206	4.60354321	4.74632978	4.70397611	5.13676267	4.60593976	4.65570614	4.67862205	4.80724996
CAB39L	0.89756218	2.91811242	2.5941736	1.57971127	1.36520395	2.18665776	1.18762351	1.16029865	1.04890872
CABIN1	4.79664021	3.37964898	3.76772911	3.35238233	4.32314837	3.67303717	2.9039869	3.71365663	3.59258021
CABLES1	2.09766189	1.89800218	4.14127718	1.70162	2.13693398	1.77818039	2.52747619	3.33563652	2.32971291
CABLES2	2.54731295	1.58099451	2.24834849	2.38195422	2.33390433	1.69877513	2.06494178	2.55764963	3.0310861
CABP4	0.0380001	0.2933644	0.67604114	0.1790063	0.22976089	0.12850527	0.43027479	0.36804139	0.0787686
CABP7	0.05485801	0.25744401	0.18193167	0.0719758	0.05408641	0.16667084	0.02653106	0.07147171	0.08003233
CACFD1	5.03054809	2.26870376	3.89176176	2.28287875	1.60946947	2.4040312	3.00507789	3.63115622	2.64148265
CACHD1	2.52649589	1.9863721	2.97086	1.48718915	2.24050834	1.6182015	0.89373136	2.09838061	1.66741987
CACNA1A	0.20499299	0.3837941	0.71526143	0.18750227	0.08774158	0.14899505	0.03941305	0.0933853	0.08018153
CACNA1B	0.01829563	0.03335994	0.12480299	0.0240998	0.01579298	0.04002968	0.00220264	0.01412344	0.01156722
CACNA1C	0.2239714	1.91197492	0.63787136	0.82340486	0.23982932	1.55991825	0.39164449	1.68392986	0.18603121
CACNA1D	2.23854601	0.86078591	1.37729512	1.01311458	0.33835318	0.34284265	0.55131186	0.19912923	0.9379481
CACNA1E	0.05828034	0.35349992	0.08207364	0.18454088	0.08583251	0.13444365	0.02192978	0.01023908	0.01927436
CACNA1H	1.08174155	3.37367282	1.76675275	1.34374903	0.91250024	3.20792657	1.60108295	1.47922274	0.99782206

CACNA2D1	0.32138913	2.35867115	1.22208955	1.42698924	0.76952516	2.45800989	2.82865418	2.43303345	0.44474027
CACNA2D2	0.1215573	0.49772651	0.34637657	0.44826033	0.08950077	0.38284414	0.17525569	0.18336178	0.25994636
CACNA2D3	0.0980445	1.13080917	0.58142219	0.4195597	0.04263259	0.40269888	0.04597516	0.10999604	0.13687077
CACNB1	1.08523054	0.4511742	0.77187613	1.14772028	1.10253747	0.56704706	0.45512798	0.58456093	0.44096543
CACNB2	0.1632373	1.56077646	0.77091381	0.84603806	0.33011124	1.28421488	0.20208823	0.16240855	0.31940587
CACNB3	1.32043243	1.83637703	1.66042302	1.94001599	1.02236843	1.63626472	1.7280394	2.02192255	2.14324562
CACNG6	0.45216072	0.11888844	0.25940479	0.08032183	0.04766928	0.01438109	0.02335677	0.54832377	0.02052979
CACTIN	2.87261587	1.28785078	1.57274393	1.52184077	2.07766409	1.80404113	1.54003289	1.88397864	2.02573453
CACUL1	2.40448141	2.35865564	2.79194879	3.00836767	2.79799334	2.94271936	2.57081919	2.49263067	2.59407975
CACYBP	4.89402902	3.79181465	4.23402126	4.42567589	4.88850133	4.4531872	5.38252658	4.8512218	4.39505643
CAD	3.03276801	2.75682932	2.69415017	2.94515263	3.2164317	3.48383399	2.90165403	3.05260281	3.87490351
CADM1	0.39914237	1.35337011	2.25460997	0.76922195	0.71484606	1.80999576	1.47021932	0.62208023	0.9819011
CADM2	0.00364354	0.62859712	0.19219098	0.09117264	0	0.24588555	0.00653657	0.02922931	0.01715599
CADM4	3.5171321	2.36969184	1.77805144	2.47900175	2.04921005	2.00306386	2.07203769	1.73648379	2.34295663
CADPS2	2.66763023	2.77387959	3.26186997	2.83257757	3.07251343	3.01266553	2.29491266	3.8527851	2.413567
CALB2	0.19954559	1.26930433	0.57818601	0.84161809	0.62213612	1.23367665	0.07488503	0.39445409	0.08729579
CALCOCO1	2.24889992	2.8435653	2.4776355	3.04693643	2.19924196	2.89474544	1.7508858	2.94893597	3.63743177
CALCOCO2	4.15828012	3.66144829	4.05639041	3.97580962	5.27378479	4.08267536	3.58045244	3.82182443	3.8386676
CALCRL	1.26179971	4.25401163	3.93264643	3.23785902	3.12271174	3.62182894	2.30607452	1.69067721	1.49887676
CALD1	3.97723219	7.03447131	5.27671344	6.09553815	4.44282228	6.37727387	4.91649968	4.33682911	3.56289799
CALHM1	0	0	0.04248981	0.03537243	0.02912001	0	0.02127973	0.01828729	0.00626115
CALHM2	1.92757367	3.34252316	2.8933628	2.78974966	2.24804225	2.88866092	2.79305984	1.70566611	1.59494352
CALM1	5.67220779	6.43658533	6.04871075	6.49301824	5.97405764	6.23952436	6.04102809	5.88656074	5.77922835
CALM2	5.35914376	6.3003847	6.51773881	6.58735396	7.12468158	6.03120462	6.3950736	5.91856086	6.25343513
CALM3	7.4514441	6.25452574	6.3174304	5.97907628	7.35328109	6.4466894	6.44610131	6.31834698	7.06786674
CALML4	3.52290905	1.59412861	1.63278269	1.28390309	3.10829349	1.02400409	1.8037592	1.2308256	2.93939163
CALML6	0.01561743	0	0.05563482	0.04635071	0.01921798	0.01149284	0.00936898	0.04762106	0.03264269
CALR	8.73549779	8.12480457	9.15778759	8.18116396	8.75121528	8.4344454	9.3388716	8.24021426	8.74203132
CALU	5.6163827	6.32248783	6.72197482	6.48090913	5.83773447	6.36438933	6.5507574	5.75814077	5.17895055
CALY	0.03587414	0.683217	0.05996898	0	0.02220532	0.13980977	0	0	0.06228009
CAMK1	1.64469348	2.06209678	2.29625466	1.23169697	1.30475046	1.7059433	1.81721757	1.56637296	1.10574851
CAMK1D	0.86983171	2.2709412	1.72044905	1.35138514	1.4261621	1.84229153	1.07231998	0.85071983	2.50120179
CAMK2G	2.35323502	3.61990554	3.17210209	2.91513338	2.45490577	2.99498688	2.6810162	2.51608385	3.60284053
CAMK2N1	5.18031293	4.84854585	2.93041034	5.99292583	4.43672944	3.83432472	4.937177	5.15713776	6.26173024
CAMKK1	0.39292116	0.80767772	0.93881792	1.03082619	0.41952912	0.85909629	0.54831067	0.54888015	1.40451746
CAMKMT	1.75700483	1.08428177	1.83592295	1.67751538	1.15088281	1.2700378	1.62122459	1.13127059	2.04642513
CAMKV	0.0185697	0.0730568	0.05951706	0.04959618	0.01146798	0.00342944	0.02220381	0	0.09963133
CAMP	0.42124595	0.34700496	0.16669939	0.80958718	0.39323519	1.97828819	0.19166898	0.17720284	0.05053231
CAMSAP1	2.39559183	1.91177108	2.37453702	2.58579205	2.39721197	2.61299704	2.58013227	3.06083009	2.43638839
CAMSAP2	2.04773782	3.00962234	2.59025111	3.2997734	2.99887009	3.2796562	2.91551023	3.67548158	1.71942297
CAMSAP3	3.97778889	1.8035544	3.28993003	2.57939636	2.78938676	2.57388045	2.67514957	2.78198786	3.88985221
CAMTA1	2.19547412	1.84408199	1.43921693	2.1306761	1.89615676	1.94392873	1.64462703	1.13523656	1.98958156
CAMTA2	2.7105855	2.94994721	3.34487189	3.24467721	3.13547626	3.2786717	2.933494	2.54671628	3.37397237
CAND1	2.50083901	3.53802205	3.58558536	3.82615104	4.19478537	3.77410877	3.55496266	3.97076768	3.80458608
CAND2	0.31546048	1.5037614	0.41124272	0.50080524	0.15527648	1.26683017	0.1865299	0.16002391	0.12070078
CANT1	5.33151922	4.82539782	5.5426341	4.93717865	4.58899316	4.58076933	4.9651743	6.38762461	6.33085936
CANX	6.18519224	6.45467368	7.24329668	7.17056171	6.94286654	6.66981789	7.23952276	7.57690195	7.3937822
CAP1	6.2314312	6.7376933	7.15832487	7.04663896	7.08179474	7.11425464	6.93024845	6.15249898	6.55643075
CAP2	0.57835798	3.22633547	2.13823655	2.86060972	1.4481983	2.53550928	1.46618083	2.17299642	1.28597837
CAPG	6.52687608	5.22665944	6.13299239	5.94186937	5.6913814	5.85879212	5.99434085	5.5925006	4.37731628
CAPN1	5.48696	4.83138955	4.98628448	4.69286717	4.54390772	4.93864429	5.98466185	5.89151826	5.75398742
CAPN10	2.04726292	0.89499028	1.40238805	1.12042283	1.11505218	1.1750541	1.31797717	1.43714138	1.79620845
CAPN11	0.11824782	1.66590822	0.39362585	0.29435332	0.15007174	0.17729968	0.10661043	0.13227337	0.17275656
CAPN12	1.75688074	0.44507344	1.33713728	0.16272297	0.32841493	0.1369386	0.21373166	0.80659384	0.55762813
CAPN13	0.0139737	0.44907206	0.60772816	0.42081076	0.18257456	0.14235656	0.49645698	1.10668962	1.57525434
CAPN15	3.75872598	3.29939131	2.25062058	2.39024892	3.48336999	2.93271796	3.36021926	3.21368561	4.07834669
CAPN2	4.5500272	4.6025684	4.62944816	5.02318923	5.17017982	4.53278873	5.38022169	6.06808143	4.5649575
CAPN3	0.00778137	0.11588685	0.30384898	0.18842798	0.25084788	0.13926247	0.04596547	0.07614198	0.2166559
CAPN5	6.19555391	4.1983923	4.11738865	2.98311732	3.73433233	3.02657143	4.40905052	3.75147564	4.9036318

CAPN7	1.8331637	2.44311763	3.24336713	2.97462134	2.73009969	2.66007593	3.14390609	3.00947144	3.01866319
CAPN9	0.01398711	0.32963666	1.38115108	0.92609043	0.06765704	0.03570376	1.08782553	1.15807607	0.26269194
CAPNS1	6.98152903	6.29744752	6.42041332	6.62285364	6.79082049	6.59976981	6.59752543	6.59043604	6.62696978
CAPRIN1	5.18263051	5.42683718	5.44496807	5.86000832	6.13114256	5.78497488	6.18691084	5.52628483	5.88499442
CAPRIN2	0.6525469	1.42006141	1.71332413	2.21046523	1.59606457	1.95855149	0.91987889	2.57751872	1.18506469
CAPS	0.63541509	0.87700672	2.68071104	1.72996525	0.74397912	0.51148569	0.3561817	0.93214598	0.64502505
CAPS2	0.29021182	0.37206076	0.4977368	0.27316172	0.39405827	0.4296457	0.12489446	0.23938805	0.06714717
CAPZA2	4.01355488	4.39067975	4.77256714	4.42750558	4.56488639	3.86193139	3.96757248	3.69120866	4.03256222
CAPZA3	0.43146018	0.05649377	0.26481142	0	0.1930369	0	0.23982532	0.06320679	0
CAPZB	6.90123347	6.50227547	6.32314149	6.47748919	6.57027359	6.62072477	6.3221731	5.4739107	6.25770557
CARD10	5.00846472	2.82068186	2.67127623	2.52934472	2.57171022	2.66868823	2.43826455	2.225311	3.53520662
CARD11	1.27951874	1.94935075	3.67705608	1.03992194	3.42003995	1.63962486	1.20233135	4.1168075	0.95770491
CARD6	2.44335052	3.31665298	2.94025019	2.22212718	2.66411416	2.81314832	1.74471625	3.5008893	1.60145264
CARD9	0.79834916	0.32008123	1.95187676	0.34651981	0.6262997	0.58779788	0.45676254	0.27762458	0.38432931
CARF	0.22969216	0.94100616	1.00427897	0.95785405	0.86244805	0.80751221	0.50613981	0.52463046	0.96477493
CARHSP1	4.14610611	2.63697598	3.09530776	2.77171612	2.55935515	2.36896696	3.22725678	3.20437815	3.23630334
CARM1	4.35399662	4.03580582	3.79960287	3.8507692	4.46294647	4.32545134	4.00972679	3.86576698	4.82557166
CARS	2.76422119	2.09753144	2.07919995	2.37706606	3.04631061	2.65296328	3.11905998	2.28751107	2.79137013
CARS2	2.70239035	2.19451417	2.51901484	2.22123392	2.32255215	2.04075745	2.82532966	2.30401341	3.31795634
CARTPT	0	2.33849763	0.16358889	0.05296009	0	2.63714031	0	0	0.04650427
CASC10	0.11070116	0.65868521	0.77856931	0.41677202	0.26869001	0.54264304	0.14200535	0.15739144	0.13921964
CASC3	4.35005289	3.61356065	4.31925147	4.09523878	3.51938523	3.8176871	3.3015543	3.86189115	4.15549178
CASC4	3.4530246	4.33832633	4.45517426	4.76472529	4.46401792	4.51713897	4.49873391	3.83056536	3.32102698
CASD1	1.94185892	2.38311248	2.51234984	2.40012941	2.24511302	2.34583312	2.20894895	2.20745012	1.38669087
CASK	3.06985569	3.80275389	3.15391789	3.15887678	4.53891219	2.70335513	3.11233286	3.04710552	4.20941598
CASP10	2.10595865	2.70044086	3.76003087	2.27198448	3.78287585	2.63519131	3.0950781	3.24672621	3.49809618
CASP2	3.553091	2.38457145	3.10558874	3.24536704	3.19496997	2.65891624	3.03376861	2.29296366	3.05135976
CASP3	3.20978159	4.32818134	4.37628016	4.86296673	5.13686751	4.61568062	4.79027794	4.11345104	5.11715306
CASP4	4.21498751	3.45719332	3.79002076	3.984215	4.89046183	4.20038323	3.88473033	2.97787642	2.89738744
CASP5	0.63726438	1.2899354	0.9814416	0.75312815	3.90672357	0.85165678	1.69250644	0.3086132	2.18157478
CASP7	3.11582186	3.88004154	4.86960938	3.36104336	4.96523027	4.07205922	5.05321725	3.66909754	4.37806382
CASP8	2.65246778	2.3788784	2.82635229	2.76428422	3.64079767	2.42934605	2.75438895	2.78319726	3.08028453
CASP9	2.33390566	1.64665803	1.79335861	1.63618323	1.54441682	1.64604384	1.60296567	1.56136974	1.94117108
CASQ2	0.03895465	1.62773254	0.48074435	1.80791347	0.07118841	2.03583999	0.14954841	0.22933444	0.27047245
CAST	3.34549293	4.37452879	4.60601822	4.3642263	4.71245831	4.3796304	4.23148267	4.52669281	4.87943233
CASZ1	2.04102382	1.42898854	1.20721004	1.33273666	1.3728008	1.63501662	1.66828397	1.36612899	2.06196551
CAT	4.75173173	5.80323522	4.84956247	5.30522577	5.01372871	5.69143723	5.51402058	4.9609658	5.69789017
CATSPER1	0.15421704	0.26213295	0.65126502	0.15420624	0.12018682	0.137733	0.13181608	0.21792295	0.09651095
CATSPER3	0.15295988	0.13838987	0.258511	0.20586858	0.21100395	0.13724585	0.15675461	0.15964064	0.26492207
CATSPERB	0.82462657	0.2067327	0.92874948	0.42103745	1.19343329	0.2220167	0.35814074	1.10018987	0.69786122
CAV1	2.25737677	5.95268712	4.00645555	4.58592693	3.18245819	5.17859167	3.855692	3.16455368	3.46065015
CAV2	1.2143424	3.56469145	3.64319424	3.32796115	1.68301827	3.63357007	3.49391198	2.09322606	1.63195616
CBFA2T2	2.05567513	2.10189022	2.58595137	2.12083691	1.89024839	1.96609975	2.1446587	1.8424588	3.08112646
CBFA2T3	0.23073316	1.0502901	0.783435	0.6019029	0.53167388	0.7716022	0.23541869	0.27926218	0.38116204
CBL	2.31594286	2.55253155	2.37611228	2.48516673	2.93413863	3.09770897	3.04306101	1.65039643	2.12925917
CBLB	1.55449113	2.36842179	2.4556462	2.41217553	2.69533492	2.02164403	2.83941273	2.63102259	2.28699117
CBLC	4.73322919	3.4687383	3.95042167	4.01741491	4.47886787	3.68507389	3.08234968	4.45551041	4.91676182
CBLL1	2.84780742	3.1454731	3.30847991	3.34967678	3.40092467	3.24190318	2.82446014	2.82964464	3.40644845
CBR1	2.11106876	4.63647171	3.78870059	3.84914235	3.95197277	4.8455141	3.22129815	4.14785091	4.24167577
CBR3	1.27872183	2.14688576	1.33020355	1.40410553	1.76958681	2.75625891	1.12243903	2.40988496	1.57908322
CBR4	1.64419863	2.14056393	1.98319421	2.53651162	2.44448959	2.43858095	1.80025534	1.81355343	2.96354043
CBS	0	0.03944739	0.06078242	0.02554945	0.02622836	0.01179139	0.02233044	0.02666134	0.01962683
CBWD1	0.88781922	0.80021847	0.62261414	1.05305951	0.75301653	0.78833473	0.72238492	1.82020702	1.2494547
CBX1	4.74293688	3.76051452	3.35205858	3.86348128	3.57523136	3.77645907	3.78402349	3.62989265	3.68615682
CBX2	3.86099942	0.63188289	0.52893894	1.21857982	1.48995795	0.68449537	1.64610302	0.36086831	2.02350584
CBX4	5.74705253	3.08511151	3.8895764	3.29879759	4.22596439	4.09847254	3.95000039	4.25372586	4.05330568
CBX5	2.33088132	3.51791462	2.47293959	3.77452527	3.1817364	2.94467383	2.78025925	3.39641437	3.42206223
CBX6	4.37679335	3.30084464	2.05945659	2.19309989	1.09992324	3.30848095	1.00944951	1.074368	0.6603058
CBX7	1.7382174	2.18250519	1.92816971	1.49781863	1.07700853	1.94861276	0.7853923	1.60576957	1.47863016

CBX8	2.55374313	0.69470151	1.02147633	1.60324503	1.11755528	0.88346978	1.47480688	1.17949819	1.97357176
CBY1	3.59730568	2.61380448	2.48381605	2.60252517	1.93488878	2.40516214	1.51013403	1.76768265	2.48629316
CC2D1A	3.97350566	3.05552763	2.87259188	2.67577957	3.46753012	3.03923601	2.83044734	3.26712903	4.15884572
CC2D1B	2.9309941	1.78737776	2.41070179	2.16027048	2.20241268	2.28867354	1.95871915	2.28772535	2.20883542
CC2D2A	1.89732572	2.24660353	1.34036083	1.53080991	0.83999891	1.6428728	1.23966883	1.2832931	0.22252169
CCAR2	4.02255798	2.90513033	2.58558406	3.61932273	3.32373311	3.07274653	3.77059644	3.27521162	3.45228768
CCBE1	0.16229833	0.12750534	0.35956804	0.76402971	0.27288695	0.24129113	0.29856407	0.08097726	0.13344334
CCDC102A	1.94742831	1.93737851	1.15240099	1.44353329	1.10109484	2.04399767	0.99684201	1.87272226	0.92988069
CCDC102B	0.31453676	1.80294148	1.71168738	1.19655648	1.43532524	0.93919945	1.08211982	0.64310015	0.44011803
CCDC103	0.00932665	0	0.013457	0.00560281	0	0	0	0	0.00490995
CCDC106	4.08127893	1.74195466	1.44251624	1.09471881	0.9054973	1.69633756	0.78248108	0.54245467	0.54186273
CCDC107	3.16424375	2.2536011	2.33711668	1.52095928	1.24851597	2.17569189	1.86920566	2.58776368	1.89930279
CCDC112	1.69379911	1.65193416	1.92895413	2.59263718	2.3985303	2.07529906	2.12918652	1.15991192	2.71885694
CCDC114	0.10620145	0.01929412	0.06584599	0.1030403	0.03099976	0.02776469	0	0.131091	0.07383203
CCDC115	3.78637136	3.29556196	3.5571489	3.03795945	2.79702439	3.22445485	3.00164615	3.14388952	3.2090283
CCDC12	3.33512336	1.98938601	2.73523971	2.55290037	2.40726549	2.40842829	2.60855402	2.26215086	2.5083401
CCDC120	2.09842255	2.90537621	2.13557128	2.18437862	2.20968554	2.08681161	2.35059607	2.29914035	2.79184259
CCDC124	6.02453726	4.21987227	4.46729036	4.46951936	5.07184793	4.97630598	4.60894971	4.77577608	4.71309904
CCDC125	1.16277834	1.22813665	1.62508527	1.46255564	1.83916285	1.29955718	0.99374278	1.31552738	2.14570659
CCDC126	2.44181661	1.92298832	1.69207221	1.91158373	2.08093878	1.87557481	2.17719835	1.57407387	2.13751789
CCDC127	1.30373665	2.00736215	1.19808225	1.51479381	1.34757593	1.46041001	1.33866535	1.53450535	1.52436077
CCDC130	3.26667821	2.04561993	3.11529954	2.86367731	2.91027815	2.4599388	2.02116535	2.42785412	2.8789554
CCDC134	3.63891781	1.54306377	2.0915719	1.87233051	2.91054408	2.13698826	2.26252765	1.38825419	2.1707571
CCDC136	0.0746025	0.99890877	0.39893134	0.47703364	0.19643742	1.30185533	0.2839125	0.32937472	0.41888931
CCDC14	1.25227896	1.41317171	2.06991349	2.59085375	2.9525182	1.42809488	1.36845884	1.40002685	2.93266235
CCDC142	0.92024746	0.65102697	1.268296	0.83682449	0.80996509	0.70757835	0.52145738	1.2387565	0.91377436
CCDC144A	0.00233942	0.10598081	0.23947148	0.05105915	0.04961922	0.00343692	0.01948914	0.01168364	0.09062505
CCDC146	0.96090849	1.99993343	2.36058174	1.02413824	1.43319287	0.75998633	0.56071038	0.37285084	1.06993703
CCDC149	0.3010599	1.33140055	1.40588172	1.53054631	0.71372816	1.60013208	1.01751716	1.73350441	1.31454997
CCDC150	0.08051471	0.07764133	0.40510768	0.38946911	0.50208552	0.1570788	0.20153595	0.14873275	0.36049171
CCDC153	1.12175352	0.34036157	0.44800857	0.47648528	0.15792628	0.29295097	0.12266351	0.3366676	0.20895534
CCDC159	2.22262694	1.65560119	2.11808067	1.67146342	1.32255666	1.55533659	1.11908357	1.84967562	1.23430617
CCDC167	5.51501694	3.46620693	4.66748395	3.74826083	3.90239079	3.7887362	3.92769991	4.70444769	3.75096453
CCDC17	0.31413577	0.30975315	0.4863868	0.50084599	0.40396754	0.28104501	0.09526252	0.2119089	0.41322879
CCDC171	0.241014	0.42342619	0.23234798	0.3676262	0.41239616	0.25170254	0.2163154	0.73588732	0.44213953
CCDC174	2.45111828	2.5252755	2.76435121	2.89612593	2.9391445	2.41702795	2.51607864	2.79751886	2.93906149
CCDC178	0	0.28290726	0.12396253	0.1918727	0.00500843	0.2291606	0.03372849	0.01871158	0.01278574
CCDC18	0.60852313	0.72462226	1.01477252	1.21143774	1.84233185	0.85259481	0.65979036	0.53028268	0.92277816
CCDC184	0.39250622	0.50584429	0.46642578	0.57060331	0.07469227	0.54941515	0.40348806	0.3259463	0.14753962
CCDC22	3.71245921	2.66250504	2.61552423	2.11073778	3.01916024	2.81386306	2.57056837	2.90414751	2.94731759
CCDC24	1.94230344	0.88825068	1.39389429	1.50703324	0.68977718	1.07183647	0.97515762	2.40852603	1.2250059
CCDC25	3.1031319	3.8783571	2.59037272	3.79243089	3.54208987	3.90074708	3.72099033	3.07509233	4.015272
CCDC28A	3.71733295	3.81434825	3.95498451	3.50445049	3.80429287	3.3851584	3.53434482	3.18717506	3.45489741
CCDC28B	2.23061472	1.14523052	1.12347687	1.32533493	1.43933863	1.27280183	0.99086188	0.87666461	0.99924256
CCDC3	2.00646431	2.83528459	3.9139195	2.55392044	1.97077369	2.85847921	3.14238736	2.78227453	2.76191907
CCDC33	0.03885497	0.01793486	0.02817574	0.03270568	0	0.02011734	0.00470728	0.01509967	0.04483843
CCDC34	2.53721339	2.05223567	2.00066575	2.05047606	2.78462634	2.11081211	2.37533887	1.39226343	2.23573071
CCDC47	5.60156983	4.62549253	5.84482748	4.90120907	4.86291213	5.0797406	5.34776058	6.33104034	5.28758244
CCDC50	2.52681902	3.7667252	3.52378187	3.74491792	3.3908407	3.66475038	3.26739566	2.83399587	3.04911774
CCDC51	2.72876011	2.63774235	2.89324073	2.80983744	2.60147139	2.61316182	3.13739581	2.76326324	3.29992737
CCDC57	1.72806073	0.84387877	1.65304579	1.22946587	1.14249619	0.8240919	0.97083874	1.57946256	1.57968391
CCDC58	4.88347906	2.24164277	2.81593092	3.21563929	3.02176156	2.77335591	3.20733889	2.63031581	3.65596744
CCDC59	2.99814129	2.78187089	3.39186374	3.45695262	3.62894993	2.89174234	2.73263895	3.12758266	3.34415903
CCDC6	3.26896484	4.18651911	4.64430923	4.03629699	4.53330831	4.17762507	3.7565309	3.56485957	4.604237
CCDC61	2.43095275	1.59645589	1.79712113	1.53476753	1.67302556	2.32160799	1.76368811	1.72680611	2.12115121
CCDC66	0.82936764	1.00737736	1.54216358	1.78656972	1.52480922	0.92556457	0.78482541	0.97724248	1.06577043
CCDC68	1.16893452	1.66343112	2.07854485	2.01955592	3.94116192	3.07259313	4.04884195	1.8018711	2.85483939
CCDC69	3.59335711	4.9256136	3.03577043	2.95095157	2.84161086	4.56373347	2.20384837	1.7040329	1.97087075
CCDC71L	3.17545513	3.68953389	2.89168384	3.41686133	2.7800925	3.68295725	2.23760536	2.74438108	2.63860928

CCDC74A	1.39262034	0.98071079	0.70373753	1.73115438	0.23513505	0.89272696	0.79265183	1.39830254	0.69341141
CCDC74B	0.03212895	0.120446	0.1073953	0.25810744	0.0198773	0.2054865	0.06186163	0.07324133	0.04207452
CCDC77	1.11591462	0.9803426	1.67432173	1.75429568	2.19076238	1.43031667	1.09067916	1.16476462	1.46078738
CCDC78	0.46223776	0.05917654	0.14485851	0.70686895	0.68183566	0.2015812	0.13547454	0.58955184	0.65365586
CCDC8	0.92916227	2.24836866	0.63845791	1.42184181	0.36805624	1.64439182	0.98472864	0.5849628	0.23091023
CCDC80	2.44802887	6.72443526	3.28546451	5.18815244	1.99451849	5.37498661	2.80466237	1.94287453	1.08855216
CCDC82	1.32247737	1.97371268	2.3093291	2.21744623	2.02074688	2.04283984	1.87623316	1.72485045	1.82508088
CCDC84	1.06257357	0.8314516	1.65635042	2.27373601	1.89736066	1.13510949	0.92862283	1.02546946	1.32839178
CCDC85A	0.04505134	0.42783497	0.06465228	0.34941016	0.04987258	0.18589747	0.04848436	0.53107683	0.06122348
CCDC85B	4.27051629	3.94339972	3.07053523	3.11229494	2.89200802	4.38018695	3.30459774	3.86678457	3.44135493
CCDC87	0.24175047	0.14987012	0.12979756	0.29785957	0.14669569	0.1361199	0.14952155	0.4238573	0.12003036
CCDC88A	1.80976039	2.3061747	1.8882084	2.68896305	2.1963139	2.56518016	2.61426932	2.33112721	0.7473245
CCDC88B	1.79798951	0.87612274	1.16345401	0.75813536	1.28922343	1.03840607	0.65071824	0.96011984	1.36799455
CCDC9	3.66088526	2.00860851	2.67436804	2.08506078	2.11128254	2.29073153	2.34074016	2.6398222	2.15859669
CCDC90B	2.19316139	2.80710667	2.46109122	2.64453502	2.50464551	2.64937068	1.95128024	2.55928485	2.38229699
CCDC92	3.09456699	2.67817851	2.19744117	2.40598733	1.75494368	2.31515881	1.54318076	2.21146516	2.46081896
CCDC93	1.96596619	2.29767512	2.88347175	2.98947347	2.94116536	2.58812577	2.70403715	2.5745658	2.9551869
CCDC97	4.31529648	3.20736025	3.63241663	3.36309994	3.86781812	3.73057761	3.17217509	3.57330289	3.49518098
CCHCR1	2.4775635	0.99488381	2.83515124	1.25978851	1.25650625	1.14995592	2.24859806	2.70212042	2.38738937
CCKAR	0.02103484	0.10529528	0.19981581	0.17909528	0	1.55713445	0	0.0242942	0
CCKBR	0	0.06908409	0.63219848	0.24204776	0.02514865	0.04957422	0.0081937	0.01578521	0
CCL11	2.2323911	5.41366432	4.43194556	3.13217896	3.50899802	5.05936048	2.84969774	1.67885479	1.96505914
CCL13	1.31280313	3.57072675	1.99313418	2.52038109	2.05150429	4.25919716	1.63903135	0.98836399	0.90595987
CCL16	0.04120073	0.1038972	0.31114357	0.09696689	0.08741317	0.05277013	0	0.01601923	0.05390247
CCL18	6.66282612	4.3298553	5.1917045	7.76530769	8.27605424	6.69650758	6.73787113	3.4122079	4.38763867
CCL19	1.80876147	4.73268599	3.38113477	2.45583527	2.23429074	3.75946253	0.62754174	1.64047828	0.72194037
CCL2	4.29999586	4.41466254	4.68327993	4.80182974	3.91168826	5.99757172	2.52578813	2.5898024	2.37074915
CCL20	7.77916332	1.72965071	7.59878176	5.87641753	7.17483387	3.13908412	4.39830629	8.06519032	7.18115821
CCL21	6.53954421	5.6174167	6.24374932	5.7084806	4.41015967	7.19420438	4.22933237	3.14914322	2.90603296
CCL22	1.01272145	1.14749741	2.21378645	1.07541534	3.20551896	3.3454646	0.93645066	1.10666474	0.99481672
CCL23	0.59784068	2.24040903	0.69443144	0.89726499	0.89884533	2.19277212	1.25902416	0.24452272	0.3032291
CCL24	0.11354802	4.58675705	5.21399818	1.7892652	0.8934141	1.07943334	2.74114307	0.96111258	2.17933752
CCL26	1.10098254	0.84745831	0.94906981	1.18051119	0.33858446	0.76280557	1.15113347	0.38227948	0.44079636
CCL28	2.10998147	1.30899815	3.30263077	0.85389779	1.10593406	0.48338801	2.96015719	1.81917252	3.90355102
CCL3	2.06310643	1.66866384	3.31708868	4.32490173	3.56983992	3.54821702	2.76547798	3.20035449	2.18468203
CCL5	6.30330311	5.19077129	5.21346199	4.75121993	7.60949061	5.65828465	5.36193736	3.30544077	4.47419446
CCL8	2.96924863	2.10978019	2.4251613	3.55396919	3.49572849	4.99381207	1.53342575	0.51062092	0.93818916
CCM2	2.44807189	2.09173719	2.31010856	2.12859435	2.04850381	2.55826976	2.23073724	1.83091985	1.79454564
CCM2L	0.60173283	1.54799171	1.18260016	1.02225721	0.86721249	1.24303855	0.61534393	0.45690492	0.35295064
CCNA2	4.14000015	3.05169073	4.18900324	4.66503532	5.53230992	4.64100908	4.53884591	3.87430704	4.54373243
CCNB1	4.46236988	3.41818466	3.95730587	4.95602332	5.74907553	4.96582552	4.80847881	3.91340398	4.94686565
CCNB1IP1	3.17935797	2.41710088	3.22598598	3.68402351	3.18694137	2.89661422	3.03477738	3.03918383	3.86286005
CCNB2	3.97854464	2.78000446	3.6780762	4.07501224	4.86195291	3.99201978	4.374561	2.60865338	4.32639821
CCNC	2.62132013	2.99506009	3.18756055	3.7314514	4.52105111	3.91698598	3.90655701	2.83094885	3.73555771
CCND1	4.14609831	4.27556373	5.24663782	5.71842973	5.65802057	5.82287868	5.80877902	6.09551339	5.4645538
CCND2	1.98085149	5.07633269	2.92202732	3.04304907	2.97498182	4.96654376	5.17304262	1.85387003	5.72678038
CCND3	4.73931788	3.75458565	4.64915415	3.02394463	3.19482307	3.70022886	3.36703009	3.37208308	3.12424079
CCNDBP1	2.51959228	2.27641314	3.09191352	2.88715497	3.03294116	2.88613492	2.61891325	3.06477788	2.8599878
CCNE1	0.52194378	1.44326889	1.73381497	2.6665626	2.73759268	1.93666538	2.67055982	1.70334298	2.92158151
CCNF	2.57071337	1.27619255	1.48059583	2.38227425	2.85120854	2.29281754	2.60666473	1.77131275	2.52257794
CCNG1	4.92747992	5.05401235	5.10954228	5.31470796	5.59215413	4.98077232	5.50673946	6.12851626	6.14964935
CCNH	2.63070596	2.5870214	3.05994704	3.24520786	3.25589259	2.8279908	2.62359403	2.5979213	3.21332262
CCNI	6.35395215	6.78740814	6.25066293	7.09792673	6.34317503	6.73401173	6.79562359	6.40144989	6.71778671
CCNK	1.48905114	1.9915765	1.41784716	2.22770498	2.56387093	2.00535277	1.91404918	2.02298281	2.21144676
CCNL1	3.05187306	2.09723696	3.82007522	3.82755484	3.85225173	3.1698232	2.73544353	3.58094878	3.7716587
CCNL2	2.88145014	1.85489505	3.24415999	3.96822484	3.18898105	2.44045543	2.22514983	2.17201201	4.06625997
CCNO	3.13621981	0.517968	2.73110481	1.71686404	2.11983986	1.81526943	2.17874258	0.02310848	2.56331004
CCNT1	1.95306944	2.42012023	2.78251629	2.86750135	2.8662439	2.68899861	2.48053988	3.30244995	2.90439293
CCNT2	1.91801974	1.95639561	2.88342162	2.53853092	2.72742473	2.09896572	2.1774696	2.59640403	2.53544894

CCNY	3.98119704	3.808494	3.92799793	3.77655264	3.7063249	3.89684614	3.84691895	3.50463116	3.80268978
CCNYL1	2.38061967	2.11104834	2.86265687	2.63078883	3.11058668	2.56845057	3.30117805	2.19747446	2.16295331
CCP110	1.67779188	1.90057255	1.71605969	1.74508262	1.96634498	1.84028217	2.02449591	1.46249681	1.50097704
CCPG1	1.29187093	2.30622851	2.33593381	2.36993254	2.52003796	2.29297305	1.76767157	1.68247908	1.5369938
CCR1	2.30195216	4.23934817	4.03654399	4.0419649	4.68159832	4.56036444	3.3469529	2.21523211	2.71633472
CCR10	0.31166471	0.62428508	0.47533285	0.36663884	0.16200588	0.63163484	0.20901036	0.18120849	0.32596571
CCR2	0.52593246	1.98319297	1.1520212	1.20621456	1.56528485	1.84731055	0.59880128	0.3011531	0.80779947
CCR4	0.73923705	1.08863993	2.073089	0.77816468	1.07808703	1.34395241	0.66122091	0.61804363	0.39778401
CCR5	2.21603999	1.99529491	2.26071099	1.49579522	3.71659632	2.30471284	1.92768955	0.90892974	1.27999351
CCR7	0.92330034	2.14258987	2.10559227	1.54978051	1.80506049	2.0922579	1.03924412	1.17492313	1.04926857
CCS	3.53210038	2.90987756	2.74626534	2.68430353	2.32030256	2.7758756	2.68293388	3.33048204	2.64983982
CCSAP	1.56573081	1.34540842	2.26475786	2.22672044	2.76596172	2.44911306	2.62794509	1.63093472	1.80371316
CCSER2	1.65952347	3.42852184	2.55396741	3.2767381	3.48105646	3.16643867	2.47216312	2.6997591	3.12910883
CCT2	4.59475146	4.61250072	5.36798295	5.66152607	5.98437227	5.3778989	5.16986492	5.40334019	5.73838576
CCT3	5.73545603	5.51181257	5.72207925	6.21787793	6.42490204	6.14944012	6.91706449	6.68220714	6.56468324
CCT4	5.15736736	5.51099368	5.41744264	6.2586198	6.54450963	6.00039157	6.13936485	5.97079785	6.06655902
CCT5	5.14757632	4.84189273	5.0631443	5.19394237	5.82543218	5.47850284	5.31226511	5.23806381	5.36622113
CCT6A	5.25414304	5.20736977	6.01510775	5.98356004	6.25674385	5.75092405	6.24292425	5.63544395	5.74324493
CCT6B	0.16659124	0.54999253	0.77894569	0.68255321	0.56672812	0.40889233	0.35516181	0.26565989	0.44522678
CCT7	5.99132116	5.45634557	5.56221744	5.98315326	6.41760738	5.81837318	6.2625329	6.09468345	6.30997315
CCT8	4.44680008	5.0004507	5.07768321	6.14587606	6.54377267	5.91033923	5.57552647	5.90411051	6.29112824
CCZ1	0.86159686	1.22574558	1.2121292	1.19775615	0.52648959	1.54860175	1.36085859	1.039307	1.1359047
CD101	0.59906761	1.84522458	2.07987718	0.61403715	2.19457367	0.98872172	1.09338515	0.48472944	1.18462506
CD109	0.33818029	2.45625152	1.48329438	4.21499705	0.933336	2.47478657	1.95822748	0.91203655	0.7747779
CD151	6.58839402	5.84507032	5.32357236	5.39902616	4.78926791	5.48305553	6.27010641	5.95153389	5.55342868
CD163	2.58124306	5.31954686	4.14103632	4.42316371	4.99825526	6.01707114	4.20412819	2.51219631	2.19013164
CD163L1	0.04415304	1.53840696	2.17690611	1.44397207	1.93453536	1.64634408	0.30319029	0.80551276	1.25188507
CD164	5.25404076	5.98212131	5.80543128	5.91869809	6.60451253	5.62242061	6.49759566	5.19498728	6.27235595
CD180	0.39174921	2.02543626	1.91300668	1.72462394	1.91470063	1.49870393	1.45531114	0.79081869	1.3011169
CD19	0.3237646	0.85276466	1.14958746	0.53330621	0.80900013	0.45752758	0.08591058	0.31106813	0.49214509
CD1A	0.29747861	0.44253037	2.42755388	0.29178875	0.08324489	0.12828606	0.84865505	0.70230643	0.49752092
CD1C	0.35801788	1.95750391	3.31522181	1.08374503	0.9505837	0.58308006	1.44795337	1.10664146	0.38444686
CD1D	0.81758135	1.15073818	1.86745936	0.83214561	1.35284851	1.50247095	0.72338399	0.55684134	0.41147725
CD1E	0.35073162	0.51954746	1.72018742	0.36619201	0.31055068	0.28080714	0.45487746	0.35437829	0.11687636
CD2	3.62754849	3.98240014	4.74502865	3.74930783	6.42990627	4.40221093	3.70105957	2.65672574	3.98877841
CD200	1.50650487	3.05902979	2.86973616	3.17090782	2.4500142	2.98609838	1.60287315	1.4394885	1.49351888
CD207	0.26632787	1.02675369	0.75984564	0.05734008	0.14827699	0.1300832	1.05386499	0.47040217	0.10855873
CD209	1.34799705	4.27246391	3.80718579	3.14863515	3.33553584	3.88049383	3.30828349	1.36226249	2.16490364
CD22	0.18314046	1.22743384	1.43344226	1.60523042	0.75037087	0.61823091	0.36430677	0.41185438	0.26363948
CD226	0.25415817	0.70225836	0.70276833	0.39816636	1.22277077	0.44939834	0.41788375	0.1624985	0.28693151
CD24	9.77583478	7.38656355	6.71755823	7.30927545	8.65768076	6.62921287	9.2230512	8.3064623	7.62647537
CD244	0.76427953	0.79020391	0.91192157	0.56826565	2.51941984	1.31306412	0.81735934	0.20156811	0.66080395
CD247	1.47428826	1.06875112	1.4503641	0.79295821	2.28144467	1.40333629	1.08971904	0.80786797	1.28596391
CD248	3.75936692	6.03549375	4.18261484	4.69402284	2.89936453	4.31975186	4.4620068	3.15038469	2.86620664
CD27	1.78097846	2.081556	2.83235888	2.44341716	3.05616718	2.48577932	1.64810354	1.74914271	2.36806424
CD276	4.60703769	4.21948911	4.41783366	4.56604678	3.5730014	4.32039878	4.78878484	3.6937031	4.02218946
CD2AP	4.09636619	3.66219766	4.34606367	4.72808726	5.35380178	4.65679178	4.90777447	4.50367258	3.98390662
CD2BP2	4.09612886	3.88777664	3.70430501	3.25581266	3.56303173	3.4913485	3.44353759	3.71293083	3.50063987
CD300A	2.14822167	2.51851475	4.08874994	2.88946329	3.60943945	3.18201326	3.36385874	1.96611163	2.84487544
CD300C	1.31154118	1.80697742	2.26354981	1.42139302	1.73940039	1.97704877	2.23822201	0.93461514	1.04203407
CD300LB	0.36503104	1.26569516	1.22771565	0.75305624	0.29731772	0.76803995	0.59063908	0.39898482	0.11502075
CD300LF	2.545867	1.682418	2.87793773	1.7914792	3.72631346	3.05713148	2.19842162	1.28258609	1.92206547
CD300LG	0.00766034	0.39407369	0.17239473	0.41379382	0.01880352	0.17517722	0.00459041	0.02349011	0
CD302	0.42326165	3.85059206	2.9565796	2.55846461	1.97421811	2.99743391	2.09022617	1.2414048	2.23717246
CD320	4.43235964	3.28077689	3.94592152	3.75916344	3.4530083	3.92462408	4.727661	3.67004363	4.71588694
CD33	0.78843464	1.20041142	1.37032367	0.81428778	1.31150383	1.71115042	0.96652616	0.39808326	0.28689528
CD34	2.38779778	4.13194396	3.64362093	3.25530907	2.72047266	3.50717124	2.4622597	2.06240108	1.88665071
CD37	3.2413827	4.08302845	3.37233793	3.2695346	3.85552849	4.31504083	3.0463318	2.47884913	2.17628169
CD38	1.45579528	1.53908393	2.04088846	1.96129189	4.67666594	3.0175147	1.23809453	0.78606841	1.31591039

CD3D	3.73532168	3.2029552	4.24607376	2.71079789	5.66683495	3.66897666	3.64292433	2.17678315	3.58050495
CD3E	3.38310635	2.92096436	3.76862199	2.67168207	5.09816819	3.56023183	2.70938152	2.00578928	3.08321198
CD3EAP	1.32039732	0.75169714	1.0543572	1.53408239	1.70551149	0.87906584	0.98472208	1.00189253	1.39026891
CD3G	1.33789406	1.39275035	2.2748536	0.97382156	3.39249444	1.81708378	1.37659158	0.77028577	1.70810515
CD4	4.5939876	5.22303449	5.56454527	4.03247596	5.29532269	4.78085059	4.87170085	3.75559871	3.5962084
CD40	4.65661441	2.83212348	4.34684498	2.81302791	4.51800891	3.25228855	2.65441874	3.9676307	3.29532655
CD44	10.1731359	5.45526142	5.10889258	6.1351325	4.54233801	5.11245044	5.58174637	3.52843879	5.68797237
CD46	4.0164716	4.51093338	5.64491014	4.88111067	4.95737778	4.48795058	5.73759938	5.57623934	6.04173157
CD47	4.77900832	3.70361134	4.87666758	3.97507112	5.51536676	4.21225379	4.74920356	4.83637407	4.42934369
CD48	2.98007325	3.87479055	4.16201905	3.49646538	4.91554621	3.6802162	2.43340734	1.92086229	2.53352128
CD5	2.12064526	1.74781212	2.18311737	1.23869182	2.60243135	2.20046488	1.73491549	1.35770766	1.50309546
CD52	5.47394142	4.65204327	5.26885822	3.71599668	6.11112856	4.18692762	4.15017742	3.0888096	3.35488119
CD53	4.78774428	5.76515127	6.02100562	5.83796224	7.06743578	6.29064172	6.18311748	4.50586513	4.62850048
CD55	5.07673274	5.72996571	5.90915126	7.19896543	6.94876492	5.73950062	7.04931416	6.78665558	5.53903106
CD58	3.33570053	3.76932659	4.48167903	4.60333775	3.9741331	3.5558338	3.74500718	3.86274052	4.01724936
CD59	5.17644121	6.12668336	6.3874152	6.11618214	5.9337341	6.05250619	6.46844811	6.86512147	4.42379211
CD6	2.06349438	1.09532196	1.6314781	0.78445122	2.66780282	1.70093139	1.24985276	0.94481256	1.1280689
CD63	8.21577544	7.70642834	7.79669191	7.3273323	7.36801189	7.44095837	7.54967476	8.40785956	6.33695131
CD7	3.29584363	2.10833644	3.96721924	1.65285267	3.61328049	2.9798757	2.55089848	1.22998663	2.68217104
CD72	1.97800948	1.71270443	1.67524771	1.27168421	2.38636688	1.80282057	1.93788395	0.51734801	0.54868071
CD74	10.5528156	10.4172485	11.206013	9.41228279	11.5734171	10.4938616	9.8856304	9.44180981	10.0460206
CD79A	1.94758413	4.01204792	4.30721466	3.53581009	3.30809651	3.10156204	1.08871277	3.39808092	3.19585606
CD79B	0.82967406	2.23508644	2.02849676	1.5656302	1.19809533	1.25298163	0.5511829	0.66459057	0.97377139
CD80	0.42153124	0.64621193	1.91055874	1.08229349	2.22510473	0.95351256	1.36352004	0.73912769	0.7908465
CD81	5.90450202	6.3731892	5.65875737	5.34716501	4.70136757	5.79931135	6.12703004	4.88704832	5.00839248
CD82	5.89420163	3.64264779	4.89105461	3.88120266	3.85883452	4.53201255	4.72298096	4.17119329	3.90711793
CD83	3.70189604	1.99703444	3.25306605	3.07970576	3.72946774	3.45428004	3.05195407	2.86915682	2.08465921
CD8A	2.83133272	2.43505808	2.81592267	1.50125458	4.8570521	3.1442418	2.45240944	1.24106816	2.32329589
CD9	4.01859612	5.91168643	6.31571902	7.31305025	5.93783925	5.64239082	5.94275331	6.06983659	6.53320848
CD93	2.74693296	5.07899417	4.66919768	4.34818969	3.5168719	4.83348336	4.04719785	3.58956288	2.9115839
CD96	1.56332914	1.81405162	2.24221028	1.51450467	3.69532302	2.42368826	1.76275547	0.95065122	1.51238559
CD99	5.94666925	6.39472291	5.33398083	5.8362571	5.08711185	5.84800857	5.40868077	4.7366187	6.02878861
CD99L2	2.40623564	3.55775601	2.40894286	2.7643884	2.74564663	3.25944982	2.23198073	2.26514236	2.15897038
CDA	2.00601669	3.24406149	2.4720837	4.71565155	1.9547634	4.49575828	2.50574876	1.78099034	2.16180624
CDADC1	1.56966656	1.64332452	1.57738223	1.51584843	1.38037795	1.77481772	1.22333203	1.51350422	1.69803718
CDAN1	2.01733302	1.04891996	1.5425832	1.46125852	1.87547264	1.49276625	1.28045871	0.99026643	1.66219304
CDC123	4.07346583	4.4653794	4.3572487	5.02167771	4.63733478	4.42559867	4.81048182	3.63661293	4.93626188
CDC14A	1.09575136	1.38838301	1.64839794	1.65038087	1.69068978	1.50810335	1.31768121	1.49739461	1.50583026
CDC14B	2.08173915	1.7990378	3.43697266	2.17594063	2.46052663	1.83742818	2.31129892	1.86476686	2.33992083
CDC16	4.04010607	3.89639215	4.16139497	4.07778304	4.40639225	3.78094219	3.93473039	4.02345185	4.96453571
CDC20	5.10248539	2.91575803	4.79994511	4.60017683	4.35018771	5.08168328	4.58863307	3.75668435	4.59586118
CDC23	2.91293847	2.95464411	3.39513492	3.55034345	4.02137333	3.3292085	2.85950083	3.07405993	3.25092326
CDC25A	2.17305249	0.65985174	1.35887282	1.77185734	2.74503081	1.79531798	2.35127813	1.13840729	2.11386525
CDC25B	5.46111936	4.1601282	4.57331105	3.73370159	5.42472191	3.9386188	4.42006402	2.87926555	5.85118859
CDC25C	1.78481913	1.02857511	1.91580179	1.70848056	2.22568952	1.62461169	1.80992523	1.55090991	1.88486267
CDC26	3.1661136	3.0385191	2.887403	2.10502581	3.07329467	2.47864071	2.18242162	2.06130692	2.76256893
CDC34	5.53008972	3.64299557	3.76043475	3.56235256	4.0116771	3.88037587	4.22109574	4.14674477	4.47438446
CDC37	6.38049579	4.67566997	5.2845753	5.27919162	5.45934075	5.43681664	5.51047907	5.48643354	5.32920814
CDC37L1	1.50056744	2.18489487	1.67801528	2.74401019	2.40369247	2.32271602	1.84033544	3.21668787	1.7428783
CDC40	1.65718339	2.63238376	2.00067464	2.33416685	2.7902837	2.6570486	2.68075442	1.93778797	2.67248612
CDC42	6.05359154	6.37778738	6.06974466	6.84957998	6.55468467	6.66767537	6.31807138	5.74015519	6.44319224
CDC42BPA	2.07322582	3.12200163	3.1582463	2.85001863	3.01252457	2.80713843	3.74910997	4.42343068	2.35722697
CDC42BPB	4.43963228	4.3149039	3.38049175	4.35567797	4.36543162	4.41128927	4.34707509	5.38180385	4.38481319
CDC42BPG	2.05148207	1.17750572	1.47646578	1.86330364	1.15856719	1.13531347	2.55434682	2.98752279	2.92561429
CDC42EP1	9.28220132	5.33527671	6.99826941	5.61801887	6.83908519	6.86083636	6.91871024	6.82028088	6.66129025
CDC42EP2	2.76403421	3.62683597	4.02720757	3.46352472	2.87846621	3.51468107	4.01215871	5.23915665	4.24937815
CDC42EP3	1.75454712	3.12114359	2.20459094	2.67983654	1.75738932	3.17163727	1.4198189	1.69012473	1.23425919
CDC42EP4	5.33081803	3.80932626	4.47221602	3.74300717	3.8116392	4.35634955	3.69529166	5.13625947	4.22020345
CDC42EP5	7.78587918	4.64687602	5.79518494	3.19487561	5.82966377	4.35312254	4.53951216	3.50611188	7.12842664

CDC42SE1	4.14619141	3.46191427	4.77397578	4.55404691	4.4696527	4.50195562	4.32556282	3.97482607	3.85076542
CDC42SE2	3.78557304	3.88445074	4.69171097	4.24440148	4.93827877	4.4712698	3.89401105	4.21907046	4.97072266
CDC45	3.2389833	1.5889458	2.79855431	2.54351625	3.26979965	2.34942866	2.91572289	1.79066797	2.9526866
CDC5L	2.5790009	3.32809479	3.38238143	3.61284862	3.71575626	3.43880718	3.60429014	4.1409936	3.60792881
CDC6	2.59749412	1.63087298	2.34321237	2.87706477	3.53211651	2.57529408	2.81191176	2.45774209	2.85699446
CDCA2	1.86800836	1.07271265	1.31600248	2.58378177	2.91660419	2.77305104	3.08729251	1.51655564	2.27586581
CDCA3	2.27859265	1.26773098	2.11389063	2.14087084	2.38639739	1.97120766	1.74473431	1.43385077	2.70486854
CDCA4	3.90136912	2.1628031	2.7996064	3.39712089	3.81864573	3.62946583	3.13233159	2.98544216	3.34740694
CDCA5	3.65067835	1.72587595	2.49702327	2.86748333	3.17772486	3.00845474	3.54211442	2.29555596	3.05850434
CDCA7	4.83465523	3.78621214	5.5672796	4.40434329	5.0764981	4.11512854	4.34259592	3.75815008	5.88036587
CDCA7L	1.13128508	2.33193578	2.08063175	1.27344387	1.15705094	2.43522323	3.71154136	4.0525867	3.34557068
CDCA8	4.47380356	2.53601653	4.08524863	3.98911017	4.75051447	3.55847508	3.86636627	2.2491908	4.21742319
CDCP1	4.53910334	2.75453971	3.72346726	3.88323787	5.17715358	4.03194999	4.15716695	4.21961804	4.50696915
CDH1	6.23145243	5.43666204	6.36109463	6.16149237	6.40753769	5.4327517	6.85369653	6.78585182	6.97287931
CDH10	0	1.32782831	0.17587502	0.02370082	0	0.32315467	0.02949984	0.01906286	0.00522463
CDH11	1.98488388	4.17142235	2.96407371	4.10235804	2.90534215	3.90615051	3.37366298	2.32793559	1.47933215
CDH15	0.16360948	0.22120845	0.21621839	0.02343159	0.00967006	0.05116336	0.08689486	0.06527384	0.13051245
CDH16	0.38297165	0.27944008	0.18241665	0.64121422	0.16792677	0.0073068	0.02367359	0.04165558	0.41591186
CDH17	0.02775863	5.7956793	2.45099241	6.35051536	4.32918368	1.3240794	3.8130831	0.93996248	7.27198032
CDH23	0.06703383	0.48854787	0.33079473	0.31231942	0.13632406	0.35800675	0.07531465	0.12794023	0.06329908
CDH24	2.6579058	1.34944405	1.96247989	1.96473163	1.58161122	1.33514938	2.04785667	1.62573517	1.55653574
CDH3	0.96493324	2.5059433	0.99854961	2.7117177	3.65127301	2.94752057	4.87050132	3.53835358	3.66344199
CDH5	2.34418831	4.02065304	3.69341684	3.57103943	2.92167265	4.01224969	2.78444307	2.48941002	2.18754969
CDHR2	0.39668568	2.65109885	1.32478817	2.05259257	1.65555315	0.97323006	0.15091708	2.37143351	1.79203384
CDHR3	0.66085202	0.81686434	0.97505078	0.43373125	0.20078171	0.24128627	0.47999818	0.24545641	0.73076766
CDHR5	4.04417632	3.59201873	2.26670559	3.25200894	2.91024294	2.16341827	3.0675108	2.13628691	5.04640343
CDIP1	1.52717025	4.61560843	1.91819621	3.34305594	1.48231594	2.79657188	3.51708774	2.88330242	3.17471722
CDIPT	4.80115827	4.98393765	4.52077773	4.33425804	3.4719175	4.49900241	4.49899688	4.53507511	4.37017357
CDK1	3.32701981	2.85877003	4.23268318	4.13985499	5.08161974	3.85244436	4.03517863	3.18416696	3.83372316
CDK10	2.25907374	1.79739721	2.44708854	2.29901327	2.20454261	1.66778473	2.10956822	2.60467045	3.16067216
CDK11A	1.12223431	1.03535174	0.51695717	1.03633902	1.16653575	1.02334027	0.83061798	0.54984906	1.59030185
CDK12	3.0793165	2.60023122	2.75512293	3.10033473	3.61347479	3.2069362	3.03798593	3.07170431	3.17533938
CDK13	3.22769728	2.75211158	2.80278181	3.0675411	3.35102948	3.0963505	3.13456855	3.09707912	2.92483181
CDK14	0.85200475	2.6408904	1.54638286	2.71338166	1.4105434	2.21419944	1.53765064	1.58752918	0.6848532
CDK16	4.4850064	3.46591176	3.87930913	3.90411145	4.3535599	3.8775291	3.71600628	3.9576729	4.62759205
CDK17	1.65851406	3.06651969	2.78980415	3.09849114	3.06131869	3.21623572	2.24893386	2.74709291	2.20925337
CDK19	2.94482452	2.34511334	2.3268226	2.72280229	2.81524635	2.38223829	2.84607554	2.18146096	2.6817468
CDK2	3.45135792	2.52330536	3.52807925	3.70253186	2.89498039	3.23883579	3.03352958	3.43247549	3.57351345
CDK20	0.15663693	0.6678466	1.08525023	0.97132615	0.59277623	0.8755118	0.21872842	0.47084254	0.79299758
CDK2AP2	6.25592216	5.13133219	5.02888275	5.24079447	4.4051125	5.10176919	5.26120766	5.23198975	5.83208781
CDK3	0.15327032	0.05676005	0.31375312	0.18205035	0.11944045	0.1210352	0.07083194	0.14091185	0.13190644
CDK4	4.9522602	4.11320291	4.82542513	4.42399661	4.22224813	4.45083143	3.9587517	4.5649282	4.95391627
CDK5	3.63342038	2.79178758	3.25982533	2.29122913	2.84641013	2.73853517	3.0321064	3.02118712	2.88040257
CDK5RAP1	2.13081927	1.93590283	2.91337479	2.44894312	2.7755497	2.23044132	2.39089775	2.29443951	2.75820706
CDK5RAP2	2.39104533	2.40581188	2.37765759	2.24697922	2.71664165	2.27685664	2.19705717	1.69983488	2.36775873
CDK5RAP3	3.55302531	2.26870579	3.4712484	3.72488039	3.68138299	3.18388205	3.40988447	3.88244844	3.84634307
CDK6	3.09656393	3.02721189	3.34031966	2.98081755	2.9679179	2.52201235	2.55386101	4.53471115	5.10986202
CDK7	3.46684258	2.88762046	2.92630613	4.04662819	4.03631997	3.29422047	3.71176507	3.47648737	3.74241061
CDK8	2.58621706	3.51619002	2.54563172	3.28628812	3.21110678	3.26534335	3.40885187	2.47221729	3.7694245
CDK9	4.27163103	3.68811604	4.22895625	3.89263443	4.15985012	4.06511736	3.6406692	3.29827386	4.16680134
CDKAL1	1.53843109	2.04563156	2.72240436	1.95054266	2.28250401	2.11432913	1.91457703	2.25978073	2.12576617
CDKL1	0.10292518	0.4511196	0.68034005	0.32355138	0.17837072	0.46033158	0.5385368	0.78543923	0.3274757
CDKL2	0.01915028	0.5972385	0.44062604	0.42300524	0.13590547	0.93413427	0.18900823	0.28250688	0.13069546
CDKN1A	3.70426545	5.36116973	5.33619212	6.33902439	4.75143647	5.84998471	5.88720264	5.04407725	5.03862922
CDKN1B	4.16583071	4.13420552	4.08616627	4.06646699	3.98976541	4.16926289	3.71665557	1.44986971	5.30062324
CDKN1C	1.24343186	3.93670537	1.49250538	3.0535461	0.70653546	3.35696608	1.90850757	1.85917107	1.85548327
CDKN2A	0.52372231	0.72986326	1.50279764	1.10086562	0.57425285	3.66459181	0.77029191	3.04537452	1.20005436
CDKN2AIP	2.82295545	2.51310965	2.60439662	3.79763796	3.31164226	3.27587357	2.63997289	3.85971954	3.40006337
CDKN2AIPN	3.82330605	2.38766052	2.84825135	2.63662256	2.75832808	2.59574063	2.50858153	2.34283441	2.91113711

CDKN2B	1.28201754	1.49312273	2.10772829	1.79440273	1.11236414	2.22021463	2.03181962	2.05986699	0.93578989
CDKN2C	3.33275642	2.67783563	2.34365397	1.78207208	2.04394627	2.82576764	2.53468409	3.47746798	1.58735602
CDKN3	3.25257418	3.40052418	3.25381288	4.48361074	4.05278279	3.82109297	4.23444892	2.90207434	3.55690655
CDNF	0.23256558	0.59390515	0.39609064	0.49675632	0.63019961	0.28639723	0.13222651	0.30563801	0.45183937
CDO1	0.11207571	2.27123096	0.90079374	1.84097839	0.11810973	1.4973685	0.12407029	0.27917098	0.05157545
CDPF1	2.58845596	2.11576866	1.41455178	1.94760051	1.86046004	2.09678089	1.63773762	1.48123719	1.76443736
CDR2L	6.69563651	2.91442612	4.83131296	4.09871954	4.22540254	4.60109915	3.71882543	5.06393328	2.72061554
CDRT1	0.03219281	0.05488451	0.20773504	0.18317663	0.3381351	0.16110882	0.10338629	0.19345845	0.14632922
CDRT4	0.09769618	0.16400952	0.23875688	0.22996945	0.25897364	0.11115005	0.06571065	0.10837186	0.28318137
CDS1	2.61199921	2.91626912	3.3202342	3.09232492	4.30001389	3.17259981	3.86250763	2.99793099	4.66229612
CDS2	2.23921287	2.63765107	2.95487407	2.62915337	2.60957922	2.83270463	2.86492279	1.6772207	2.43656503
CDT1	3.78236591	1.82093379	2.51358465	2.42749535	3.34841818	3.27179864	3.58621933	2.8949882	3.83738022
CDV3	5.79930544	4.73370331	4.75259415	5.32350501	5.88119591	5.55825519	5.37913644	4.90020515	4.87369174
CDX1	0.1754623	4.87393432	1.14706761	0.25055472	0.19110342	0.17212013	5.40921015	0.19408247	7.21599223
CDX2	0.08581228	3.41943759	1.85291541	1.14623524	2.72964825	0.5274145	2.06499058	0.12087722	5.66316156
CDYL	2.3530416	2.47600723	2.66655592	2.78179112	2.77882168	2.68861631	3.36803217	2.99239256	2.71096731
CDYL2	0.62592446	1.74291524	1.40991227	1.79893808	1.70433274	1.63124355	1.64428337	0.7996876	1.08326541
CEACAM1	1.74833756	3.31858935	5.80632562	4.64615391	6.74814582	2.98230708	4.47775086	5.32396517	3.57157226
CEACAM3	0.23932579	0.42035535	0.42619052	0.86922885	0.80136303	1.07242062	0.49313848	0.67577894	0.58505802
CEACAM5	2.71037039	6.32818787	6.01108598	4.19039487	6.71322734	2.00844056	6.28669744	3.78837204	6.08814165
CEACAM6	1.50462913	7.83881742	4.082057	6.12222702	5.67275145	3.52524386	4.31090483	6.05639746	5.24042966
CEACAM7	6.24145663	1.20166549	3.52180732	1.06147254	6.48624071	0.21558871	5.46361312	1.57049302	1.19740159
CEBPA	4.12415298	2.8932124	2.69445915	2.05945327	3.69072682	2.71831401	2.43366098	1.88520807	3.4571802
CEBPB	6.23556633	5.61241914	6.18065814	5.29048837	4.31555702	6.15978246	5.1413784	4.55392701	4.59875731
CEBPD	6.77836039	6.41965678	5.82801556	5.10643938	3.97248516	6.33853157	5.27892768	5.24643293	4.89105627
CEBPG	3.42513678	3.21510636	3.28919974	3.63790455	4.66515995	3.69791175	3.85800827	3.03215803	4.46021819
CEBPZ	2.81031239	3.77940985	3.69814501	3.58149926	4.38172272	4.10756669	3.9550594	4.02781823	4.22146408
CEL	0.06641645	7.45818216	0.37798504	4.72551381	0.19549464	0.10589914	0	0.18189114	4.95139062
CELF1	3.39846297	3.01640837	3.3824727	3.38098301	3.65678111	3.40876995	3.41220588	3.52064665	3.55539762
CELF2	1.4150297	3.72730164	2.50754129	3.23406707	3.23176874	3.46816182	2.1506701	1.24840161	2.51373965
CELF6	0.02017322	0.00580648	0.01460261	0	0	0	0.00606754	0	0
CELSR1	2.50466472	0.60920429	1.72753476	2.0487458	1.25494745	2.14130594	2.3067121	2.19518397	0.38502733
CELSR2	1.65064929	1.00974855	2.592346	1.66386248	1.73010669	1.2659013	1.33392502	2.43912147	1.78502207
CELSR3	0.51391302	0.73862014	1.23573778	0.38382946	0.49856885	1.23356964	0.92957491	1.80122071	1.33777186
CEMIP	1.64985218	4.27870317	3.00344177	4.84261209	1.45944223	3.25730373	3.78288558	4.84927369	2.68307062
CENPA	2.8021226	1.523723	2.01662999	2.17740344	3.18819388	2.57453971	2.40000024	1.93478199	2.48376948
CENPB	5.29554187	4.57906944	5.01213439	4.50520792	3.3993211	4.68980341	4.59084394	4.28736359	4.84685987
CENPF	2.65653707	1.78435998	2.29137929	3.13595848	3.77667803	2.73662886	3.7392943	2.72906766	3.12028936
CENPH	2.83725077	1.45471781	1.93132254	2.45361865	3.13896154	2.48955518	2.52883485	1.38148987	2.98609807
CENPI	0.8534248	0.49077248	1.7687192	1.48328222	2.27478546	1.06137117	1.10525683	0.90459168	1.18800481
CENPJ	1.31914852	1.11447342	1.43568703	1.71101408	2.16637357	1.41363088	1.50430995	1.19951159	2.31213836
CENPK	1.96345292	0.89717961	1.5571542	1.96986058	2.96337984	1.49952809	1.52592162	1.21889693	1.77547658
CENPM	5.21785292	2.42935917	2.8925276	2.66553681	3.22362812	3.02249881	2.9968534	2.04693989	3.46918681
CENPN	1.82157487	1.74858101	2.69446695	2.44105485	3.46530145	2.64915577	2.97560207	1.69371655	2.35607576
CENPO	2.27666595	1.23456895	1.96371612	1.79689476	2.32087148	2.08853079	2.11150816	1.72361931	1.92730962
CENPP	0.56283744	0.76202271	1.0111956	0.73050686	1.05140748	0.71509375	0.61168589	0.3687973	0.66264754
CENPQ	1.3884833	2.23998754	2.53661131	2.62084135	2.28934281	2.78368837	2.8205737	2.53134464	2.33940462
CENPT	2.3165779	1.59548953	2.38427373	2.49094822	2.02832511	1.89192103	1.9367207	1.82732633	2.31251647
CENPU	2.87052098	2.55111323	2.25586546	4.28708473	4.43263656	3.21446797	3.66746309	2.59564216	3.7238645
CENPV	2.79655786	2.24681269	4.1518588	3.55394611	3.68379588	3.16791168	4.03484465	3.30850394	4.45730332
CENPW	4.23710268	3.32067996	3.00446079	3.7412042	5.10987807	3.89886209	4.55120584	2.11067679	3.62538825
CEP104	2.38432024	2.27931459	1.9133368	2.08359604	2.17792874	2.47832811	2.19559178	2.02791713	2.49043868
CEP112	0.14562439	1.20259585	0.64404346	1.00433419	0.49815315	0.89507313	0.34670094	0.4809958	0.24002926
CEP120	2.06857796	2.30153727	2.52097084	2.43800298	3.27334498	2.52597511	2.02619671	1.80983674	2.40213873
CEP131	3.25711698	1.30257683	1.7066734	1.59893483	1.76114684	1.65462739	2.12656265	2.16069848	2.20871274
CEP135	1.15195802	1.05440945	1.11644818	1.52277863	2.12466819	1.32422538	1.16857774	1.10016901	1.41963651
CEP152	0.65558512	0.71327679	1.02627952	1.19975763	1.62586896	0.86789029	0.70439263	0.65930238	0.73402644
CEP162	0.85038738	1.54159774	0.94625803	1.53434345	2.05013986	1.56151625	0.90530752	0.96201167	1.4576114
CEP164	1.33942062	1.14005277	1.15235648	1.62924646	1.22128877	1.1078374	0.92761463	0.92320827	1.25662594

CEP170	1.5647727	2.1796701	2.15226394	2.3841088	2.27619505	3.06786843	2.18497143	3.15007766	1.10076056
CEP170B	4.86158223	3.19258132	2.30440688	3.30021148	3.52513015	4.04732777	3.02349427	4.95886034	3.70128776
CEP192	1.55536851	1.77776353	2.14142355	1.90482211	3.14046698	1.87830577	2.55237305	2.24211914	2.28287545
CEP250	1.73353298	1.36573746	1.44853237	1.41205062	1.63232623	1.25450423	1.28208939	1.6593726	1.89659542
CEP290	0.83827327	1.25205177	0.91089	1.19334465	1.46872091	1.27327655	0.65134753	1.14283303	1.12590539
CEP41	1.56170442	1.3550132	1.25169836	1.09826811	1.52542894	1.00487107	0.58439243	1.05342286	1.0587446
CEP44	1.24965635	1.30623797	1.26135183	1.44654589	1.59690362	1.47120545	0.97899135	1.25024518	1.51223231
CEP55	3.24720284	2.49694142	3.1957965	4.20478161	4.91386801	4.17831176	3.95398326	2.93595757	3.35318739
CEP57L1	1.05662815	1.33772658	1.04548008	1.42859529	1.94945473	1.41896919	1.37648397	0.64999787	1.20975648
CEP63	1.63117506	1.50053534	1.32772285	1.55957787	1.32720269	1.38470308	1.51354035	1.13131172	1.49533502
CEP68	1.35861732	2.49693853	1.95547996	2.02895775	1.55231892	2.01195118	1.40991186	2.61312709	1.97524741
CEP70	2.44835947	1.94170659	2.77456187	2.22839093	2.44449689	2.42378605	2.20457665	2.1123742	2.14853842
CEP83	0.95962675	0.7476815	1.25474997	1.62560549	1.63595645	1.18819026	1.02041459	1.07292182	1.33921908
CEP85	2.04157526	1.0882819	1.71317024	2.18940225	2.384627	2.09238537	1.80805937	1.21179688	2.23804939
CEP89	1.52863209	1.25801653	1.46013123	1.58385515	1.65799091	1.37817701	1.21620222	1.50274187	1.37248576
CEP95	1.32576828	1.39448191	2.56251198	2.34507642	2.09288323	1.55404105	1.28200224	2.19679596	2.2262945
CERCAM	2.86538201	4.12670717	2.65704878	4.38860793	2.0128986	3.76014159	3.42538554	2.03157635	1.90365838
CERK	1.4592201	3.31074802	3.40527164	3.18533676	2.14282894	3.33968461	3.55406536	3.00419185	3.91042657
CERS2	5.04920552	4.38290107	5.16361317	4.75073283	4.54399704	4.83103856	5.53173228	5.23753811	4.63811592
CERS5	2.14685633	2.17441409	2.89819343	2.57764171	2.20291117	2.35894491	2.1224009	2.40298551	1.8941717
CERS6	2.85416117	3.50884813	4.49408248	3.86941892	3.97273753	4.26664641	4.30616822	4.28653906	5.06539216
CES1	0.65652416	3.55944293	0.94552689	1.08743148	0.49364813	2.6465911	0.35972363	0.31266556	0.18922921
CES2	3.00804315	5.52320264	3.22785178	4.71494299	3.16635582	3.98998672	6.72758482	4.30279745	5.58534703
CES3	2.80827345	2.39756487	0.90802464	1.53960928	1.31523707	1.54611319	2.47655136	0.87697373	4.37725352
CES4A	0.14374882	0.35996689	1.20126488	0.87273484	0.26400077	0.46090186	0.20577876	0.42089075	0.53276184
CETN2	4.93106605	4.75837319	4.17984326	4.86231149	4.79942971	4.3286225	4.11713049	4.40426874	3.92809017
CETP	0.40830994	1.20892859	1.6448036	1.0384421	1.13861421	1.01712654	0.64011699	0.58912102	0.64194868
CFAP20	4.04416696	3.7413634	4.09454143	3.835901	4.07360797	3.82479704	4.03523678	3.96256809	4.27464028
CFAP36	3.81571068	3.00994924	3.28584853	3.68006792	3.98820023	3.10125077	2.74585948	3.07233014	2.66573285
CFAP44	0.48853989	0.27433038	0.79903036	0.8580894	0.92771137	0.27944045	0.27520185	0.50707106	0.94315217
CFAP46	0	0.04092416	0.14909198	0.28467125	0.01690777	0.04124671	0.11318801	0.04839097	0.05166739
CFAP54	0.00286255	0.14360848	0.37839675	0.10122968	0.07899674	0.10553639	0.03222697	0.05525175	0.03863806
CFAP69	0.61681334	0.92428078	0.85023651	0.99280578	0.51168223	0.6127175	0.17934764	0.35275273	0.37612866
CFB	3.04958617	2.55350624	5.92789105	1.58290074	2.78237882	2.51830713	2.27055585	4.46862283	3.58797861
CFD	4.08574729	7.96452931	3.44848834	3.91307993	3.29277691	5.29438045	4.10834949	2.17939422	3.54615075
CFDP1	2.1587263	3.41684477	2.72512931	3.48756981	3.28331661	3.17174064	2.87516032	3.26881908	3.27543403
CFH	1.63213601	6.04850271	3.36128659	4.61829625	4.44216653	4.2670488	3.21590695	2.3786684	1.82173481
CFI	4.35379168	4.1914452	5.28187664	3.43315662	4.03683036	3.21643511	2.067696	2.80011087	1.49372725
CFL1	7.87352158	7.27367168	7.32673107	7.20204326	7.44807074	7.36348115	7.59375099	7.00997455	7.72299362
CFL2	1.11887288	4.21801942	2.67225905	2.95799703	3.0760858	3.95502684	1.64207334	1.33439047	1.33470904
CFLAR	2.60925994	2.38505062	3.14657293	2.62855212	3.67484249	3.17829254	2.23936453	3.29396888	2.33536659
CFP	0.23469187	1.31394192	1.04047853	0.43410956	0.44051623	1.12867932	0.38296878	0.4019397	0.34467468
CGB1	0	0	0	0	0	0	0	0.01684531	0
CGGBP1	3.0729694	4.26135469	4.15468324	4.5526015	4.43857306	4.51457148	3.93956412	4.34011418	4.32407519
CGN	3.40327561	2.02605037	3.54030657	3.42097943	3.87351928	3.00086324	2.99727897	4.74356977	4.02070998
CGNL1	0.45759863	1.82124452	0.75248197	0.93469708	0.19824993	1.27606616	0.39226888	0.31615826	0.37387325
CGREF1	0.35071877	2.79117699	0.85505682	1.69607328	0.09673981	2.83087376	0.55752061	1.38553272	2.58268429
CH25H	0.36141055	3.58010886	1.73905316	1.61707877	0.23312238	3.03866241	1.46358765	0.66289655	0.40370987
CHAC1	3.6240685	0.84358835	2.21561861	2.04866519	4.05705993	2.78830894	2.74615298	2.5679561	1.787354
CHAC2	1.7368335	1.98059412	2.20348849	3.00799554	3.03319953	2.72552444	2.63158181	1.99459197	3.15066584
CHAD	0.24152695	0.38420058	0.78164283	0.49786244	0.33237459	0.10045443	0.07087029	0.51282778	0.33945124
CHAF1A	3.15150839	1.70569787	2.49355669	2.40435804	3.52557483	2.65050325	2.50724554	2.39508648	2.86504553
CHAF1B	1.59159813	1.13297254	2.06625384	1.99885687	3.25602878	2.03092077	1.90604757	1.90721307	2.4659108
CHAMP1	3.28475208	3.40729675	3.60795657	3.86581138	3.76551792	3.43477195	3.42075131	3.40771326	4.56750256
CHCHD1	4.34822453	4.1531646	4.33439888	4.26697416	4.43641583	4.19814218	3.95512762	4.11721397	4.75498698
CHCHD10	7.87689143	5.31633649	4.86364291	4.8948983	4.89455159	5.17583309	5.52525816	5.50464292	6.26660134
CHCHD2	8.91708912	7.48142432	7.78267809	7.09762559	6.9720006	7.5730619	8.27398736	6.74701114	8.10803907
CHCHD3	4.86404652	3.89325239	3.8534782	3.86817513	4.29378742	4.35559152	4.42097851	4.07342171	4.6988459
CHCHD4	2.47558725	3.23432152	2.99320938	3.30172035	3.70784964	3.53235155	3.66284857	3.52081646	4.00759334

CHCHD5	3.16082837	2.34493141	2.89961081	1.94959074	2.05617813	2.44088534	2.27793943	2.50203982	2.42292784
CHCHD6	2.24749394	0.99262123	1.7419659	1.61728073	1.20231359	1.95315222	1.73141752	1.72293261	2.23575713
CHCHD7	2.11686717	2.04365636	2.39402038	2.60946752	2.51926421	2.48413262	2.69094482	2.56712623	2.60182688
CHD1	2.08437612	2.62362461	2.34023691	2.94188928	3.41946087	2.91847041	2.30646969	2.15946118	2.8982875
CHD1L	3.26155834	3.10421307	3.72733001	3.47202768	3.65775773	3.56667478	4.25282866	3.20467096	3.79914714
CHD2	2.18048983	2.10871939	2.54319	2.57003233	2.60443478	2.4606887	2.05203041	2.78183022	2.71748598
CHD4	4.84376101	4.96848484	4.40560973	4.77867299	4.80680834	4.99397584	5.00456842	4.94963505	4.89271717
CHD5	0.01830849	0.18961072	0.09777769	0.05849121	0.01804775	0.08287325	0.00879665	0.01834655	0.07363124
CHD6	2.11371792	2.48603968	2.4799573	2.15291578	2.20620818	2.1823629	1.63291129	2.13594651	2.67007071
CHD7	1.12211539	1.49440017	1.260653	1.91396738	1.85671571	1.81744881	2.31315206	1.73255234	2.34395082
CHD8	3.23172209	3.28723764	3.17517625	3.17640108	3.67441531	3.57342828	2.99561687	3.68343725	3.35533565
CHD9	1.42186596	2.58724069	2.01050733	2.20429953	2.22003844	2.49490911	2.13710635	1.97928541	1.66602329
CHDH	2.88531969	2.11909939	2.88085111	2.88484603	1.44526492	2.16861092	2.66997253	3.75004066	3.28838462
CHEK1	2.09566784	1.81004219	1.29600921	2.54695032	3.08160294	1.86152985	2.89981743	1.3869792	2.42042158
CHEK2	2.05420291	1.95163487	1.8879245	2.11672473	2.60489904	1.77689333	1.66384744	0.84925453	2.72957943
CHERP	3.84483809	2.81087241	2.76952377	3.08617599	3.6083732	3.38588722	3.14351209	3.41023923	3.75822547
CHFR	0.66365073	1.0392985	1.6289372	0.85997382	1.04482172	1.03237855	0.61246634	1.47592464	1.0501075
CHGA	0.04624623	3.25414948	2.35736575	0.57394184	0.72875584	0.13585241	0.08864798	0.53489802	0.87219148
CHIC1	0.13047506	1.85481358	1.3648314	1.15542556	0.79731081	2.05824854	0.52845904	1.84631437	1.42745194
CHIC2	3.83811436	3.12581385	3.46456843	3.23884728	3.02983563	3.33508139	3.22181819	3.1254929	3.23764816
CHID1	3.74265429	2.8928379	2.72837267	2.64335673	2.63921266	2.796366	3.63983258	2.95825867	3.09163733
CHKA	2.64725909	1.87374168	3.8215458	2.52053124	2.31436866	2.12632327	2.56615594	2.66140755	2.12088041
CHKB	0.88980689	0.52672854	0.84594841	1.02038127	0.90348799	0.64645965	0.36773207	0.56580337	1.01730042
CHM	1.31122093	2.276404	2.30924612	2.33069432	3.29900243	2.47642643	1.93280114	2.43752726	2.05640991
CHML	0.97615248	1.13215195	1.59282237	2.68967383	2.18823555	1.83443234	3.4530696	3.9373264	1.62580298
CHMP1A	4.91002052	3.81286495	4.37403053	3.98845289	3.98147295	4.4403074	4.96570786	4.68738704	4.47415892
CHMP1B	4.15512856	4.90010513	4.99502466	5.07696304	4.77265941	5.14607973	4.98050194	4.77797127	4.8657717
CHMP2A	6.19800997	5.19646455	5.54903687	4.77479417	5.28470339	5.23547877	5.096755	6.14149715	5.18070637
CHMP2B	4.15208556	4.38684924	4.74232713	5.21197899	4.9078646	4.67503017	5.44851288	4.74104267	4.71887207
CHMP3	3.78232756	4.21928879	4.33598482	4.32392551	3.69423165	4.2628671	4.18517273	3.98707982	4.12872361
CHMP4A	1.02604368	0.70455954	1.16862379	1.09123922	1.17892196	0.91434025	0.86964962	0.91353648	0.99712979
CHMP4B	6.76593801	6.20739475	6.79243315	6.3859507	5.8917318	6.07308033	6.18543238	6.43725396	6.8725181
CHMP5	4.67246496	5.08986817	5.75252328	5.06169606	5.59100161	5.14348671	5.15520892	5.25218366	4.76118341
CHMP6	4.32536288	2.63204687	3.39056177	2.83874196	2.52270352	3.10782764	2.80237968	3.29189702	3.04722169
CHMP7	3.41668047	3.24008051	2.88338398	3.63348785	3.27088109	3.96294925	3.74402435	3.42415582	3.43435967
CHN1	0.9897733	2.61905869	2.34973523	2.86889523	1.67278912	2.29348736	1.94271203	1.11592939	1.01654396
CHN2	1.13068272	1.59316112	2.0072267	0.76148823	0.83979419	0.79917058	0.83663552	1.04356785	1.20581416
CHODL	0.04637866	0.71038853	0.25667337	0.04865538	0.08457804	0.18274279	0.02100652	0.04030451	0.02456411
CHORDC1	1.50337971	1.37762913	1.62426513	2.17466418	2.78673869	2.3019803	2.00876048	1.66915781	1.83227713
CHP1	4.86174137	4.63865993	5.53030113	5.70502682	5.7533743	4.92246276	5.55708347	5.3580884	5.44688278
CHP2	0.01521959	4.5471489	1.2871393	0.03625819	0.14343621	0.13922313	2.54219876	0.14036891	3.53361727
CHPF	5.36236701	4.89020538	4.79781652	6.00031736	4.4165263	5.64430352	5.78240794	5.71896018	5.01734216
CHPF2	4.66554137	3.74138607	4.85770436	3.48728998	3.27148876	4.07681212	4.3612261	3.87368551	3.84816248
CHPT1	3.43846596	3.1534334	4.12222806	3.30973465	3.65703544	3.00026899	2.71726129	3.95351303	3.81257044
CHRAC1	4.29980619	3.42611363	4.24806735	4.54197328	3.65883816	4.01546524	4.07978292	3.75741451	3.93016161
CHRD	0.57010169	1.29486846	0.75400377	0.96185814	0.20808394	1.30986775	0.60700691	0.37691533	0.30413767
CHRDL1	0.16664093	4.0851057	1.15647729	3.74074504	0.1208025	3.24762259	0.1123795	0.29690565	0.14405752
CHRDL2	0.48841042	6.38129495	3.03072113	3.80782849	0.60993451	6.51363003	1.47502693	1.01783713	0.67172745
CHRFAM7A	0.01590367	0.07727942	0.05293264	0.0717424	0.15550254	0.09291251	0.10756959	0.04848409	0.04686344
CHRM2	0	1.96291859	0.06797912	0.59099941	0.02407877	1.79419253	0.05149988	0.04976829	0.04085123
CHRNA10	0.30292757	0.0898225	0.20826235	0.12394841	0.43424425	0.24540874	0.14573571	0.1896416	0.22889582
CHRNB1	0.53538683	0.94802384	1.02353636	1.51376382	0.69129083	1.33596464	0.55049488	0.43387834	1.78949406
CHST1	1.17411822	1.55520203	1.41896561	1.33887272	0.68172601	1.65212583	0.93587314	0.74868289	0.49417028
CHST10	0.35783239	1.16034999	0.89708469	1.64522921	0.50907156	1.08204486	0.45847175	0.40888123	0.23942118
CHST12	1.42931661	1.11454289	1.65446534	1.02940955	1.01086734	1.14986742	1.0766287	1.29451573	0.85681749
CHST13	0.70966669	0.73399371	0.84456544	0.48354085	0.53838153	1.82042515	0.52560639	0.07645447	0.1618965
CHST14	2.64587128	2.73902992	2.90896471	2.68245876	1.93088351	3.04681405	2.14775971	1.93649377	1.80445403
CHST15	1.29461072	3.89833576	2.77973695	3.14769593	2.33313275	3.5099524	2.60092485	3.32357408	1.60512572
CHST3	1.01324248	2.07081155	1.20413515	2.26200887	1.14560375	2.14786348	1.82926876	0.92357338	0.5375516

CHST5	0.0217764	0.59002234	0.45216245	0.03254019	0.30907807	0.01204235	0.07048858	0.05801478	2.98968984
CHST6	0.0916966	0.57731376	0.16714545	0.52271506	0.07490176	0.28462914	0.48906412	0.08220124	0.50608979
CHST7	0.79860743	2.14402211	1.51488171	1.47908697	0.93435128	1.95115287	1.34065402	0.82366137	0.54999473
CHSY1	2.69392637	4.19408353	3.62466009	4.18560578	3.52917556	4.17789953	3.37280172	2.92247767	2.67857532
CHSY3	0.5853079	1.64670045	1.007425	1.6489214	0.66795841	1.58434045	1.13875661	0.57486506	0.35218777
CHTF18	1.63068664	1.18542829	0.97042915	1.43626483	1.5330618	1.30730303	2.02769539	1.5704559	1.99789622
CHTF8	5.02123447	4.64234963	4.93730109	4.75092197	4.95643749	5.3935743	4.69050597	4.75934602	5.11591002
CHTOP	3.81901831	2.73593516	3.48679468	3.76112859	3.7619289	3.4091598	3.68491745	3.46547858	3.79488929
CHUK	1.97914088	2.25397134	2.63964419	2.88430683	3.27897053	2.85635957	2.76495712	2.9885724	2.69592275
CHURC1	2.36748971	3.85189923	3.46694542	3.48364946	4.14190283	3.44467121	2.8961557	3.79740241	3.59710852
CIAO1	3.81160881	3.85697711	4.27191508	4.08538721	4.20042166	4.26266215	3.9731899	4.41319077	4.23679855
CIAPIN1	3.72666876	3.55260599	3.97873615	4.12850236	4.25040033	4.08231941	4.19396021	3.60667679	4.07320192
CIART	0.25914782	0.74986076	1.29995812	0.899741	0.67315325	0.5198338	0.22437307	1.40228174	0.68771661
CIB1	6.78102752	5.62506377	6.36410926	5.93864796	6.12670269	6.26153928	6.29280252	7.06965917	6.26495137
CIB2	0.35897788	2.93895485	2.7345442	1.55687595	0.3755376	1.74725684	0.96962071	1.88556227	3.10197707
CIC	4.08153264	3.50890439	3.07780307	2.95883975	2.94472784	3.36036407	2.79383002	3.42732534	3.28557471
CIDEB	0.0113514	0.28890038	0.1410718	0.18578584	0.0818784	0.30461703	0.08606468	0.2859411	0.07014956
CIDEC	0.0187996	2.22691911	1.29267404	2.08744391	1.32360222	0.12001365	0.28669131	0.39333289	0.63370759
CIITA	2.26848706	1.51232905	2.224295	1.19767236	3.7098648	2.04628836	1.30468347	1.45811053	2.00258675
CINP	2.64318705	2.54496312	2.28576074	2.58017553	2.66532947	2.59130744	2.10887785	3.21053944	2.58539521
CIPC	2.38619396	2.75890955	2.56662739	2.50109733	2.51566488	2.58240374	2.37053775	2.6143647	2.99864822
CIR1	2.79497675	4.01569028	3.97156372	3.90991605	4.36925226	3.60762791	3.30521748	3.73945983	3.98661263
CIRBP	4.19938978	3.54733688	3.87487397	4.00075303	3.6371484	3.42972876	3.324659	4.00870892	4.23834824
CISD1	2.18729336	4.10408135	3.53894973	4.13130646	2.84884368	3.60478487	3.44945409	3.72807284	4.28799346
CISD2	4.00784726	3.56220289	3.99452011	3.99517936	4.17000105	4.02321028	3.58468403	4.12310512	4.08082602
CISH	1.9980658	1.8665463	2.42488749	1.8119485	2.54906993	2.16370766	2.2766551	1.9944077	3.11351349
CIT	1.56421375	0.9102332	1.29836464	1.48290554	1.94931578	1.33198561	1.54608227	1.52953491	1.37898154
CITED1	0.19349739	1.10501684	0.20857371	0.25029333	0.05014489	0.20531443	0.77663553	0.0927275	2.27506734
CITED2	3.673433	4.80583429	4.02287041	4.91074339	4.32326638	5.17049694	4.35210412	3.59232997	4.10004914
CITED4	2.21039304	2.64010946	5.30827066	3.0493736	3.95291106	3.67245652	2.78068149	3.15764971	3.23652131
CIZ1	4.85903482	3.84725478	4.64350255	4.1429503	4.03319184	3.89173964	3.76417182	3.48259425	4.66653708
CKAP2	3.42256371	3.58625263	3.34873281	3.65108147	4.64813098	3.80913501	4.15390893	2.89507161	4.76309282
CKAP2L	1.41940776	0.96829463	2.39295053	2.16129754	2.75020957	1.93447878	2.2902125	1.77069039	2.27631162
CKAP5	3.79640975	3.52450659	3.76087364	3.99708934	4.70631147	4.00927505	4.69843657	3.83414021	4.33389421
CKB	4.74320744	5.34662159	2.63154314	3.53202851	0.85636946	4.63251326	3.09058498	5.40253132	1.94699212
CKLF	4.96552757	3.21538244	3.83624685	3.5586057	4.36811225	3.61357964	4.03177119	3.21498865	3.02039376
CKMT1A	1.06267251	2.3373694	2.46928978	2.67617399	0.58549806	2.20457769	2.82272834	1.86765726	3.46759847
CKS1B	4.29162786	2.85815868	3.17636631	3.06754998	3.6540383	3.64442152	4.46331476	3.30672312	3.64001489
CKS2	7.19852714	5.20752098	6.29020403	5.92911352	7.29045197	6.44526168	5.70357739	4.07479496	6.15697394
CLASP1	2.30066803	2.81199994	2.82110461	2.67194145	2.76880908	2.90337738	2.53054588	2.62473521	2.56734435
CLASP2	1.39415024	2.11018552	1.79927754	2.18705364	2.27102645	2.26241342	1.90481701	2.23837952	2.13865324
CLASRP	2.90019631	1.37974434	2.58770839	2.22338444	2.46451537	1.78061448	1.75429444	2.50880007	2.8887563
CLCA1	0	2.32155722	3.49687229	0.02595125	0.57005006	0.03172222	1.63472461	1.82573088	1.32352931
CLCC1	2.27262222	2.49883421	2.71590388	2.60345769	2.8817413	2.76199117	2.39471234	3.01551963	2.78679721
CLCF1	2.43978216	1.8888847	3.88086896	2.41776246	2.70218713	2.70843908	2.20599376	3.62970579	0.99155266
CLCN2	2.32822029	1.27299809	2.38410351	1.7902973	0.87240774	1.41932632	2.31807397	1.80028397	2.10831927
CLCN3	3.84805319	3.73144326	3.02149097	4.63964038	4.02458034	3.93397324	4.52300591	4.92399236	3.46661559
CLCN4	0.60496038	1.48044766	1.51886578	1.59790743	2.36622727	2.09663669	2.25765166	1.98343193	0.83219967
CLCN5	1.81820029	1.81370363	2.06334892	1.4933684	3.04412174	1.26528898	1.32492796	2.35343109	1.15813697
CLCN6	1.51814548	1.67821049	1.4782636	1.97670807	1.75217741	1.92194476	1.21463204	1.04034165	1.67158817
CLCN7	3.08457386	3.27969686	3.05331202	2.71677832	2.92626158	3.22673129	2.9492929	3.23548813	3.03206154
CLCNKA	0.01044683	0	0.00755501	0.03111397	0.01286062	0.01913421	0.01249816	0	0.01643949
CLDN1	1.28768479	3.91558416	5.10359317	4.83635947	5.4004047	1.82230138	4.64061552	3.6540938	4.41671749
CLDN12	3.34519397	3.75917086	4.25194611	3.93560621	3.31643682	2.82243177	3.92043726	4.3636507	5.03478523
CLDN14	1.00039808	0.05033133	0.23770563	0.20671312	0.58672556	0.41693944	0.10326866	0.18460671	0.12224895
CLDN15	2.07497871	4.09680522	1.86523905	1.65439766	1.48371841	1.41594103	0.6977892	1.28713582	5.84460164
CLDN18	5.97866472	0.57751301	9.72620515	2.98936657	8.85083912	7.03888412	7.54094593	10.7968745	1.41873051
CLDN2	2.57255763	5.52425638	5.89101011	3.04888619	4.78192098	2.39276563	1.70558893	6.47903787	3.91388248
CLDN23	1.73523743	2.63928799	2.8330151	2.81929483	2.0972468	2.86856042	4.43073919	5.04797406	2.35788724

CLDN3	0.28465075	6.21160449	4.09849285	3.82710981	0.31534174	5.12422832	6.96880951	0.81278067	7.30821842
CLDN4	6.25739583	4.75427613	6.56236949	5.5867662	4.95416489	5.66462406	7.84862144	5.77791724	6.88197183
CLDN5	1.35301508	3.24325055	2.11852281	2.12988332	0.65661722	3.20402144	1.17148472	1.00575491	0.45601916
CLDN6	0	0.04723578	0.15761802	0.03723589	0.03822125	0.06768904	0.04932796	0.31770767	0.0751218
CLDND1	3.31446065	4.03016185	4.05423372	4.12792589	3.75507519	3.87032435	3.77229024	3.34319349	3.93753705
CLDND2	0.31078184	0.29314643	0.73386233	0.46488666	0.74253275	0.13270863	0.36489222	0.10541283	0.66522551
CLEC10A	1.61190117	3.6018216	3.03817439	1.76559704	1.99554312	1.81967389	0.7271275	0.70571384	0.51082923
CLEC11A	3.3035494	4.38116066	3.22094605	3.56474672	1.65074194	5.58996895	3.36825437	2.18035956	1.82006033
CLEC12A	0.75190047	1.52326443	1.19280526	1.33623662	2.98884797	2.39691024	0.94554807	0.39457959	0.43877743
CLEC14A	2.2796379	3.97148886	3.46979635	2.91808585	2.65052746	3.77701372	2.45308133	2.06731359	1.83173137
CLEC16A	2.139389	2.4088417	1.78360251	1.45539286	1.97122033	2.23039073	2.38445355	2.21608984	2.28227781
CLEC18A	0.0235494	0.01690033	0.11533649	0.00355675	0.01816824	0.0065279	0.01414294	0.03614691	0.03086746
CLEC2D	0.67401238	1.34551476	1.80845709	1.57121769	2.97012754	1.51995437	0.88233885	0.48569368	0.79638184
CLEC3B	0.60957496	3.10789617	2.35870676	0.90905003	0.76907532	1.92809302	1.27693116	1.07135031	0.73198325
CLEC4A	1.50283428	2.71563369	3.55540163	3.47300014	3.39567665	3.21102587	2.58621015	2.40652035	2.21361819
CLEC4F	0	0.19680908	0.22601755	0.09404634	0	0.05311404	0.0688477	0.06111639	0
CLEC4G	0.10032072	1.02233688	0.61415651	0.51807039	0.03789864	0.91763345	0.4366678	0.03949605	0.13542366
CLEC5A	0.82944482	0.67424108	2.33847062	1.98896338	0.80427456	2.44828207	2.42568431	1.29844138	0.51370927
CLEC7A	1.61728175	2.07321708	3.33865372	2.94331227	3.70460429	3.11992461	1.87604681	1.73984029	1.6806343
CLECL1	0.06217083	1.01246025	1.26292413	0.44473573	1.02849775	0.24290056	0.43452499	0.13990562	0.20703252
CLHC1	0.34478742	0.50369834	0.89452681	0.67149981	1.08522336	0.3663986	0.17276726	0.46084632	0.73746706
CLIC1	8.53412038	6.86037728	8.51915413	7.54025178	7.91407512	7.6786538	8.21536175	8.48761675	7.88230463
CLIC2	1.34416397	3.84133885	2.92107174	2.89140309	3.53508719	3.11557134	2.05385963	1.59583613	1.50223226
CLIC3	2.67517557	1.66721799	2.30860744	2.91974692	2.14119755	1.64579339	2.65086335	3.8000649	1.1273513
CLIC4	4.78037595	6.54273247	5.61900081	6.43744162	5.83087569	6.4006759	5.8243372	4.22937504	3.46130104
CLIC6	0.44355025	3.14073578	4.22695658	1.78004533	0.22070043	1.12952643	1.64308144	0.44434871	0.15957865
CLINT1	5.15525113	4.2102487	5.24443989	4.76851723	5.19538028	5.05078223	5.09842988	5.18355548	4.89868514
CLIP1	2.10465185	2.6137229	2.32032943	2.7480322	2.93144952	2.76876702	2.16453668	2.71957219	2.09468775
CLIP2	4.23018876	3.04089228	3.17093924	3.170855	3.57017932	2.67096671	2.52368824	3.78203537	3.14197643
CLIP3	1.65218551	4.3821126	2.34036264	3.45953838	1.38579923	3.63460159	1.88988284	1.45811283	1.10273305
CLIP4	0.55372406	2.25696576	1.80344914	1.58777006	1.65887754	1.96733984	0.98850916	0.52058454	0.37536214
CLK1	2.00883323	2.74140186	3.78919386	4.14526642	3.86994921	3.60636741	2.92239975	3.36159171	4.0783626
CLK2	2.78782405	2.20031197	3.16776243	3.86330412	3.27565754	2.94997517	2.77456715	3.72633638	3.56293568
CLK3	1.5558518	1.13455069	1.81567954	1.47318049	1.55752352	1.34306137	1.35073731	1.14106464	1.81539373
CLMN	2.76522999	1.96424623	2.80716518	2.42772844	2.63817422	2.04599382	1.91342357	2.69859785	2.06666578
CLMP	1.5102837	4.79815195	1.90549279	3.6989037	1.46715799	4.0690651	2.36306716	1.17068866	0.93690063
CLN3	2.51904032	2.35536553	3.1657914	1.49434445	1.82706065	1.89909796	2.42943931	2.62281741	2.4254616
CLN5	2.34730869	2.25290514	2.24224227	2.20858384	1.84054145	2.02675585	2.04148227	2.6149981	1.85243119
CLN6	4.6752632	2.72835347	3.62011217	3.42240906	3.78657274	3.6841738	3.8865872	3.1089899	3.75579592
CLN8	1.48712432	1.24059721	1.60660785	1.56474593	1.09610668	1.60369701	1.70139185	1.54746267	0.97036719
CLNK	0.13447432	0.43517102	0.43605164	0.12480341	0.373954	0.09598904	0.1501034	0.07712025	0.08975397
CLNS1A	3.97161965	4.06439419	4.44403493	4.47307504	4.69934967	4.16773614	4.21581408	4.40425975	4.67950091
CLOCK	1.40218812	2.94505094	2.54779269	2.49109001	3.1424318	2.77373524	3.13265394	2.62573355	2.39057295
CLP1	2.43422715	2.3703092	2.59875634	2.38901952	2.9506279	2.49038124	2.73739484	2.54302462	2.8073155
CLPB	2.54603022	2.19646139	2.79244589	2.43209863	2.4613656	2.52403363	2.99626209	2.29681809	2.68643531
CLPP	5.20794468	3.84435222	4.21108494	3.72965092	4.50129613	4.27649268	4.28959692	4.51932165	4.64563342
CLPTM1	5.494995	4.47732294	5.11941482	4.68632315	5.1313504	4.88837002	4.73754073	5.24621828	4.77901919
CLPTM1L	4.42703118	3.98072599	4.35306632	3.55605219	3.64155588	3.70007856	3.89160089	4.44400912	4.07210441
CLPX	2.73495987	2.97014832	3.36327596	3.47312033	3.97180115	3.2427402	3.07589995	2.62410937	3.55306145
CLRN3	4.28921312	3.72207339	2.32428347	4.31794747	3.54832462	0.29348539	1.68633976	0.26644599	5.60267109
CLSPN	1.75145236	0.83905476	1.56048168	1.68380723	2.35085069	1.55920606	1.85653778	0.90926668	1.43066095
CLSTN1	6.20642855	5.21286201	4.68361824	5.45083653	4.92574081	5.10707355	5.64015341	4.33048269	5.48383165
CLSTN2	0.10560294	0.30601425	0.69798509	0.48299365	0.06742954	0.75483759	0.6232676	0.66727191	0.1526269
CLSTN3	3.71342434	2.99579957	2.89405771	2.7593579	2.89886956	3.0532846	1.82723821	2.39012343	1.80695382
CLTB	5.84194274	4.62451227	4.99418312	4.59329225	5.5494266	4.7841058	5.50088706	4.90959583	4.67935942
CLTC	5.36926538	5.51872842	6.02125112	5.60413123	5.88218636	5.92366117	5.72545147	6.56260525	5.6791285
CLUAP1	1.45226806	1.87366632	1.04443134	1.72043145	2.08914375	1.66874795	1.50603964	1.36566302	1.46859113
CLUH	3.89523505	3.52751612	3.87422389	3.43042659	4.2227367	3.77919901	4.18547318	3.83445343	4.53904843
CLYBL	0.97434446	1.78772215	0.65837426	0.72987631	0.59487885	0.5482913	1.26920159	1.05971057	2.49316355

CMAS	3.40277963	3.60798255	3.95479692	4.07505149	3.84210699	3.95096598	3.88342576	3.71473474	4.70540576
CMBL	0.57916736	5.12237142	3.77027634	3.45451771	4.67928896	3.68471304	4.82945252	3.86179238	0.30997253
CMC1	1.293125	1.27668572	1.46553818	1.51527195	1.84429127	1.3261416	1.43337274	1.35816304	1.56302244
CMC2	1.16047895	1.19646378	1.73570224	1.41920026	1.86143833	1.43802971	1.50998829	1.23165287	1.46572199
CMC4	0.09174383	0.02687692	0	0.04200506	0	0.02586941	0	0	0.01239419
CMIP	3.31475139	3.87601404	3.61018261	3.00115494	3.02054235	3.54817725	3.46575336	3.77131519	3.78169618
CMKLR1	2.20181339	2.89953544	3.13708	2.28477533	3.77164417	3.30147648	2.45856061	1.46327597	1.99025673
CMPK1	6.38543191	5.92607303	7.25798492	6.20960584	6.40632415	5.98570923	5.78590079	6.45405137	6.45774993
CMPK2	1.26156509	3.81185671	2.53979908	2.72434561	2.88809155	4.52399419	3.38958525	1.96818783	1.89861208
CMSS1	3.11182436	2.06976968	2.45797774	2.87493455	2.78883297	2.2152285	2.75822474	2.09454176	2.75057009
CMTM4	3.12661207	1.65611933	2.949974	3.34143365	3.28516694	2.82304011	4.65621175	3.73345283	4.14574068
CMTM7	3.38711466	2.82209288	4.20118716	3.91930455	2.78999045	3.67200668	3.51821725	3.39945507	2.04219968
CMTM8	4.23558409	2.92486559	3.49911772	3.68959948	3.06298028	3.57223112	4.15663399	4.46964411	4.23892919
CMTR2	1.41462321	2.27438051	2.29050759	2.59486152	2.72141828	2.47478518	2.15586513	2.23962092	2.64960906
CMYA5	0.01279483	0.72875814	0.34224665	0.36939315	0.06046923	0.46090122	0.06174336	0.1004407	0.05565741
CNBP	7.30028334	7.12050768	7.13041831	7.46397674	7.28451779	7.32759202	7.44016257	6.89694236	7.26687438
CNDP2	3.89677819	3.27624117	4.05842294	3.30248101	4.56944276	4.3925476	5.29043846	3.65122318	4.10172413
CNEP1R1	1.63980106	2.01341245	2.66403401	2.39571383	2.52428327	2.11219054	2.07147472	1.68640251	1.91752436
CNFN	3.71689075	1.54582998	0.88554249	4.05601673	0.36052123	1.00037374	2.25146026	1.48143692	2.45739165
CNIH1	4.17864102	4.75900965	4.6266838	5.36831244	4.75968554	4.6619737	4.59745367	4.503457	4.80155438
CNIH2	0.41105738	0.7166986	0.21260409	0.57607786	0.66601984	0.21040547	0.1017678	0.37981318	0.46325857
CNIH4	2.63641558	2.87427572	3.45316068	3.16181859	2.96821685	3.24775225	3.77874534	3.75487784	2.90912441
CNKSR1	2.9928706	1.47629925	2.40529926	3.0774362	2.9458523	1.90612734	2.09767684	2.5923347	3.35874696
CNN1	3.14692352	8.19992547	3.39858431	4.75694664	1.56400942	7.49655162	2.69806698	2.17552582	2.53535264
CNN2	4.9833526	4.87481365	5.64584379	5.69077382	4.97870682	5.51701326	5.57055154	4.836919	5.48034153
CNN3	6.23421118	6.62236436	5.96603119	6.01675842	5.60654103	6.21831302	4.67632123	3.84562637	5.71256765
CNNM1	0.0225547	0.18603973	0.31254894	0.14888506	0.13683971	0.14490145	0.16383063	3.17057826	0.10093674
CNNM2	1.24931981	1.25427443	1.14968025	0.97903908	1.26273532	1.22275621	1.27341015	1.03460343	1.06574841
CNNM3	4.12438717	2.76895394	3.29868076	2.93505851	3.10339384	3.01792533	3.28932439	3.94439924	3.79853409
CNNM4	3.62548045	2.27689991	2.46810508	3.67853361	4.15133116	3.57045041	4.25540888	3.7585446	4.13195542
CNOT1	4.21476289	4.82271561	4.56018325	4.54766663	5.46983263	4.99236274	5.15067921	4.95592746	5.25017145
CNOT10	2.32594315	2.3248463	2.38767239	3.09641992	3.30724133	2.90517413	3.0866781	2.83226179	3.03627632
CNOT11	5.38648043	3.66634837	4.47281704	4.20226767	4.60678666	4.12305347	4.14622585	4.03451314	4.96317685
CNOT2	2.30269152	2.65905869	2.91797884	2.85118939	3.00857557	2.58863788	2.3410069	3.00846744	3.11164918
CNOT3	3.52978134	2.31409724	2.91754192	2.82505899	3.27027787	2.95444953	2.69835302	2.54788583	3.31360869
CNOT4	2.34646484	2.50395278	2.55040108	2.37034213	2.62362808	2.1436602	2.19196001	2.19338009	2.27215631
CNOT6	2.36891852	2.46622506	2.89047737	3.14934308	3.49886054	3.07792438	3.09776378	2.97281087	3.50244185
CNOT6L	2.05497194	3.35386522	2.76064642	3.05908637	3.49493849	3.71125909	2.9681943	3.22893436	3.23904758
CNOT7	2.56431939	2.91533821	2.78393911	3.83948261	3.46432312	3.51819771	3.79799241	2.96811967	3.55470386
CNOT8	3.41168099	3.58426143	4.32515255	3.71167029	3.67901298	3.79258886	3.68814361	3.92536317	4.39362975
CNP	4.09167144	3.51703549	3.56059157	3.37177094	3.38880187	3.68695034	3.21091408	3.06157264	3.27892201
CNPPD1	4.6792564	3.57284131	4.00689173	3.82848051	3.19784244	4.12222031	4.01583692	3.94650516	4.49620275
CNPY2	3.23275803	2.61935211	3.40693105	3.21498062	3.05072461	2.73104707	2.82112699	3.01875011	3.14967611
CNPY3	6.4256473	4.8908996	6.18413435	5.03051289	4.79783816	5.3970802	5.60157737	5.30696173	4.84906088
CNPY4	1.9052036	2.55768135	2.36500922	2.25092868	1.42836718	1.81562887	1.79679662	1.57470463	1.17339722
CNST	1.6277065	2.60693292	2.73113147	2.62280392	2.35998715	2.4797924	2.42260296	3.29315266	2.36844015
CNTD2	0.08671089	1.1230233	0.14718861	0.16211748	0.02185585	0.38906315	2.09693655	0.12528919	2.23234269
CNTF	0.07498633	0.20513866	0.62257208	0.49375533	0.40997803	0.45674871	0.66251233	0.43797773	0.40326539
CNTFR	0	2.0228897	0.66711426	1.09864844	0.01059655	0.4673057	0.07058634	0.15146007	0.53892601
CNTLN	0.25313225	1.16507161	0.47777396	1.27205016	1.23457642	0.93627116	0.2224887	1.78177617	0.16351851
CNTN4	0.02377989	1.62295215	0.92943294	0.83811168	0.28904504	0.70124399	0.23968182	0.48468113	0.15329967
CNTNAP1	0.82627784	2.91905645	1.20619505	2.14479795	0.82564183	2.68844962	1.06396666	0.89411975	0.7091317
CNTNAP2	0.38104518	1.6168879	0.24258563	1.35831774	0.03874079	0.27724737	1.74636785	0.50712612	2.64064618
CNTRL	1.22564535	1.37571268	1.76359542	1.71958845	2.61890084	1.82494695	1.20106415	1.30040991	2.07309281
CNTROB	2.27304673	1.32562224	1.95742541	2.19952139	2.11358362	2.11259598	1.89028383	1.50897021	2.15331588
COA1	2.55405333	1.76943314	2.38997914	1.92078021	2.3821548	1.86177084	1.62900802	1.68073931	2.02988124
COA3	5.74734451	4.91122893	4.80862285	4.50817955	4.39005196	4.54770832	4.87361795	5.48977544	5.66826368
COA4	5.15231915	4.04276372	4.78042116	4.30396891	4.50514474	4.28612244	4.26091111	4.29160856	4.83214024
COA5	2.60402457	2.76631607	3.31710405	2.98631911	3.1157636	2.56641396	2.59730781	2.8567818	3.1013501

COA6	4.51635903	3.80461128	4.34312504	3.70277271	4.55734448	3.65030648	4.65698761	4.62926198	4.46361616
COA7	2.50956802	2.35330489	2.30047163	3.04160807	3.35143235	3.05553561	2.71975927	2.93998442	3.04936356
COASY	5.06222706	3.61056798	3.81293726	3.60810262	4.1334319	3.75512806	4.06592903	4.18475223	4.681268
COBL	0.02775799	1.84141755	1.22238885	2.02674222	0.04633611	2.13735188	2.03102381	2.28743899	2.05753377
COBLL1	1.76927222	1.76904333	2.31765779	1.69132758	2.18742773	1.75385013	2.71678632	2.98755939	2.31506869
COG2	1.85559736	1.99534341	2.39134672	2.12161735	2.62035732	2.20205998	2.17627556	2.7136573	2.75968182
COG3	2.85512223	2.60973297	2.77204173	2.65200997	2.97267465	2.75558268	3.17286876	3.43850131	4.28382222
COG4	3.43600594	3.1325904	3.47714031	2.81924559	3.42735816	3.41726867	3.37365318	3.33855202	3.49879883
COG5	2.33163655	2.85894528	3.13798193	2.70613803	2.87092798	2.74045836	3.19845434	2.69123678	3.0857702
COG6	2.34276827	2.22232484	2.15973383	2.55011759	2.39513375	2.33067948	2.77071548	2.72652133	2.93452066
COG7	2.66441166	2.72625769	2.95242818	2.88959837	3.06166713	2.77598496	3.23879955	3.50726365	3.05331766
COG8	1.79432357	1.43060593	1.51569246	1.54316138	1.45969356	1.87671959	1.744367	1.90929004	1.8855398
COIL	3.22684972	2.60887491	3.55108497	2.9245888	2.89443078	2.63971321	2.45329533	3.37345669	3.36570602
COL10A1	3.1694276	0.86017494	2.11075327	5.63979807	1.60170091	4.62519028	5.1387386	3.60196076	1.4300761
COL11A1	2.84524813	0.16453047	0.55615288	4.21041056	1.45507015	3.60440143	2.7943367	1.55012521	0.9463601
COL12A1	3.97328873	5.34138889	3.99973665	6.06900287	3.37819783	5.49912741	6.08652742	4.75437635	3.7453904
COL13A1	0.31679157	0.24594052	1.00988342	0.68500702	0.31082595	0.36376705	0.69083464	0.34529164	0.28838207
COL15A1	4.51736013	6.31855857	4.43361431	5.37271661	4.19158069	5.08248832	4.35158478	3.32217548	3.09901325
COL16A1	2.22796879	2.7743459	2.37464693	2.71787372	1.28819901	2.33300547	1.49103119	1.91696102	0.82200237
COL17A1	0.79830827	4.00861969	0.82579687	3.18011831	2.58402885	1.18612486	2.83821092	0.6336781	1.82861858
COL18A1	4.88773342	4.42059678	5.00738255	3.94405741	3.30038857	4.50414836	3.6828005	3.95004943	3.13066431
COL1A1	8.7677202	9.56430768	7.42044616	9.85506086	6.86125073	9.17622881	8.80584607	7.38901578	6.97800341
COL1A2	6.97883229	8.90110705	6.7269859	9.31056905	6.46445843	8.59163131	8.24722065	6.78244719	6.04014467
COL20A1	0.00331721	0.08136238	0.02379584	0.0019911	0.004086	0.00608824	0.00397051	0.01654464	0.00870198
COL21A1	0.03056815	1.54579145	0.36601972	0.56106908	0.25251966	0.53845424	0.29701586	0.08477946	0.18992171
COL22A1	0.18333215	0.05236042	0.50306786	0.31485691	1.6861989	0.29304927	0.40959761	0.80720023	0.11063707
COL24A1	0.21543513	0.35418932	0.15879646	0.82876996	0.07320036	0.60311529	0.38931309	0.18570878	0.16087277
COL27A1	1.96761214	0.71942998	2.99456098	1.65965223	2.0576141	0.85402736	0.96125682	1.90799971	0.52081875
COL2A1	0.01648144	0.02198587	0.16929859	0.09306597	0.01017553	0.01615027	0.00659985	0.04197472	0.00579696
COL3A1	7.56082414	10.3778017	7.65676248	10.4128367	7.08733983	9.55700733	8.78763935	7.17419354	6.49065977
COL4A1	5.69997194	6.63283563	6.54350732	6.13429739	6.11669911	6.42072295	6.11722823	5.44474669	5.39559616
COL4A2	5.72318403	6.29174965	5.85106101	5.41017003	5.23772204	6.18621795	5.49603024	4.94022237	4.88711732
COL4A3	0.02376955	1.27085799	0.4158109	0.32861825	0.08597289	0.53670775	0.04935852	0.08808713	0.0676747
COL4A3BP	2.20491613	2.72905876	2.51271523	2.84702605	2.46980255	3.14141468	2.55725258	2.42206287	2.19159737
COL4A4	0.07883596	3.82400953	0.83948899	1.11170501	0.22826982	1.88151572	1.06429983	0.26036017	0.25488352
COL4A5	0.0492017	1.80483617	2.00125502	1.99019401	0.08239299	1.42132672	0.60657054	1.8207729	0.2248782
COL4A6	0	1.47685016	0.68840473	0.87785497	0.03347244	0.5489165	0.15580212	0.22900713	0.10159466
COL5A1	5.06968223	5.95296447	4.39013191	6.03447437	3.73494377	5.8765609	5.5930862	4.39918027	3.61754361
COL5A2	4.30470995	6.13897923	4.85255514	6.95430605	4.40176231	6.24459306	5.97820567	4.69329477	4.21954212
COL5A3	3.43466504	1.87196815	2.0101547	2.91854402	1.95419394	2.98312721	2.64309653	1.43636918	1.96551919
COL6A1	5.80932474	7.48644532	5.92552427	7.31595199	5.13417328	7.19923822	7.26145856	6.01859743	5.52144878
COL6A2	6.39334338	7.83894239	5.87078289	7.14124348	4.9375919	7.36339793	6.80889719	5.16757401	5.32422863
COL6A3	4.38942623	7.38408803	4.60283938	6.3926176	4.25085462	6.74607814	5.97925364	4.24433519	4.06630752
COL7A1	1.77055612	0.75151972	0.94348241	2.03443401	0.73420654	1.66626815	2.01939576	1.15328115	1.47067013
COL8A1	1.78947715	4.46399004	2.36984576	3.67883001	1.97374316	3.74472489	2.42198933	2.11447317	0.96755321
COL9A1	0.01028904	0.13808394	1.87404591	0.02152129	0.14799135	0.07934711	0.50793579	1.54935399	0.10213655
COL9A2	4.29307407	0.20149943	3.00782145	0.58866872	0.32329684	1.29873035	1.26236479	2.2530665	2.79964459
COL9A3	0.13665427	0.40434003	0.27235768	0.45767759	0.12458971	0.92682144	0.15404633	0.82036265	1.39200088
COLEC11	0.26310274	1.01872135	0.72281637	0.50598068	0.24472332	0.73252867	0.14908811	0.40320529	0.06736168
COLEC12	0.35863868	3.97201748	1.3681484	2.46896123	0.5078414	2.99757938	1.45679739	0.70376689	0.25528245
COLGALT1	5.01501789	3.92470774	4.80695927	4.63789166	4.81399889	4.61011903	5.00665052	4.10384721	4.02512746
COLGALT2	0.05011286	0.46108583	3.17408533	0.63943342	0.14057016	0.68390999	1.33657547	4.97405802	0.05263603
COMMD1	2.67731217	3.61704186	3.40680887	3.32609442	3.16926043	3.13550307	2.83817321	2.82066486	3.19975676
COMMD10	2.81318925	3.69035789	3.59905345	3.60672737	3.5506593	3.52996929	2.96118491	2.40576809	3.90591377
COMMD2	3.78858101	2.64167871	3.18813048	3.24761615	2.89522093	3.05324504	3.15859872	2.67304753	2.67556459
COMMD3	2.21186977	2.37998071	2.60501745	2.86535836	3.06695346	2.36294091	2.35777417	2.1817479	2.93229645
COMMD4	3.24283631	2.38992049	2.82958042	2.26995271	2.62630588	2.83110016	2.36306262	2.22064349	2.83805149
COMMD5	3.54036107	2.50784747	3.42597695	2.81398237	2.4333358	3.13993669	3.16726256	3.64794132	2.71127896
COMMD7	3.86389894	3.99359038	4.9706935	4.55274184	4.35168194	4.06585936	4.62170369	4.13454015	4.51508985

COMMD9	2.44603375	2.49298081	2.79810602	2.46349325	2.63629788	2.80145937	2.73981714	2.52134301	2.86885856
COMP	0.70245033	1.58806882	1.22039272	4.4694164	0.26994014	2.45830267	1.89759881	2.42675148	0.31009846
COMT	5.66340721	4.4911456	4.96390346	4.15934101	4.12952571	4.19899651	4.17266652	4.45858394	5.18275482
COMTD1	3.62477919	2.31724575	3.61886862	1.90065237	2.62550955	2.96505659	3.30475544	4.09557181	4.12280831
COPA	5.16937142	5.59065201	5.38402802	5.31749067	5.39688753	5.60403774	6.22368143	6.11354454	5.74147657
COPB1	4.07452876	4.92624109	5.24749379	5.02482388	5.32776196	5.13089137	5.56591467	5.3647635	5.26518256
COPB2	4.75158655	4.71435349	4.5840365	4.52721284	4.92484132	4.74059845	5.18467388	4.92292678	4.76509042
COPE	6.44981277	5.1103658	5.8652999	5.25685003	5.96015778	5.71561328	5.47435587	5.57594234	5.9535128
COPG1	6.95867654	4.93670786	5.77966099	5.33402744	5.49444497	5.66914921	6.34671841	6.26159949	5.96227521
COPG2	3.29293128	2.7728995	3.01147952	3.44449215	3.23779375	3.150222	3.43765499	2.71545598	3.42577279
COPRS	5.35602284	4.2689845	3.99315174	4.17395919	3.95899428	4.64057664	4.36821128	4.21798868	3.71668699
COPS3	3.18880365	4.11761783	4.47140271	4.68775599	4.97007899	4.34122044	4.30262091	3.53200807	4.87166352
COPS4	2.94514161	3.79822869	3.29771905	3.85527896	4.02413541	3.74247156	3.54987112	3.64670853	3.82519918
COPS5	2.71930872	2.67637882	2.57468236	2.93538181	2.96197207	3.04934929	3.4652269	2.62303307	2.77964777
COPS6	5.75412112	4.92959647	5.41537789	4.44435006	4.29036708	4.80626485	4.6310469	5.04505887	4.86828874
COPS7A	4.63986174	3.84528033	4.30627288	3.80425168	3.61271367	4.29754618	3.58176047	3.77822395	3.96551938
COPS7B	2.89416165	1.83362456	2.64527738	2.60631168	2.79741917	2.60080246	2.50377074	2.42724081	2.89743312
COPS8	3.52795092	4.81657272	3.92880353	4.63965841	4.25341193	4.32149418	3.62048289	3.72870335	3.76890328
COPZ1	5.60316279	4.89897826	5.79840489	5.28052646	5.35982629	5.21053205	5.36464557	5.8361012	5.43844499
COPZ2	1.8561255	4.13012478	2.05490464	3.42431542	1.30036278	3.02423979	2.27247519	1.42113381	0.93437198
COQ10A	1.23118875	1.05951985	1.26381438	1.35138906	1.19286481	1.40208623	1.03174222	1.2857505	1.3116269
COQ10B	2.61639475	3.64549954	4.24863074	4.16581459	4.03531734	3.77809321	3.81644988	3.53229095	2.99828234
COQ5	2.72181635	2.68509393	2.99648378	2.70764393	2.37523227	3.03488903	2.44552549	2.85600467	3.16903777
COQ7	2.40215153	2.42844044	2.46771022	2.25638956	2.36596881	2.46446214	2.70978	2.26516969	2.65648578
COQ9	3.46579218	3.51273719	3.79819116	3.57213194	3.34630352	3.54692258	3.72570254	3.83028166	4.21955116
CORO1A	3.57492979	3.63379401	4.29271481	2.85195997	4.42851535	4.64900803	3.44215691	2.49807502	3.50367479
CORO1B	5.57121372	3.79840642	4.71330845	3.59814653	3.92913546	4.38144794	4.83468595	4.835487	4.61006268
CORO1C	4.59095272	5.02111487	4.4711086	4.96585362	4.89628526	5.33401466	4.84038646	4.47676329	4.18585417
CORO2A	4.67277181	2.57612217	4.50248644	3.20862496	4.1416911	2.52163771	3.97862876	3.03811025	3.68839148
CORO2B	0.08839388	1.92924666	0.76807936	1.00330115	0.23763243	0.94236253	0.3215065	0.23308101	0.26615495
CORO6	0.09782006	0.41307939	0.23559927	0.77865462	0.16201829	0.28072492	0.1440311	0.10951351	0.12722446
CORO7	1.13431375	1.32902011	1.10285949	0.94917063	0.97681046	1.32678256	1.10237415	0.85022548	1.3972288
COTL1	6.14804272	4.36106446	5.4920838	4.08696833	5.99030737	5.06732066	5.34111153	5.11086693	5.01018287
COX10	2.21150187	2.49084182	2.80639074	2.88895547	3.39545245	2.99034988	2.632525	2.14083915	3.70896755
COX11	3.2690203	2.33887008	2.95963865	2.94040439	2.52938991	2.71196994	2.37721397	3.47787823	2.88708612
COX14	5.06462011	4.78836027	4.80349674	4.31973434	4.88686754	4.5460255	4.30538025	4.88802363	4.74427499
COX15	3.0893941	3.72935752	2.9824956	3.88777024	3.79762312	3.90938173	3.89144294	3.75746957	4.07931979
COX16	3.83951888	3.45381674	3.75595208	3.38361511	3.21773368	3.36759296	3.31630157	3.44880659	3.93793179
COX17	5.37172611	2.5401846	3.18189499	2.620507	2.95007773	2.67841508	3.06693157	3.18660767	3.11541879
COX18	1.46651779	1.45868815	1.86901493	1.58018543	1.72927356	1.85537972	1.51486031	1.56129283	1.99512998
COX19	2.24420219	1.34063523	2.47705541	1.76251685	1.94282349	1.42663851	1.86119475	1.9811096	1.84496648
COX20	1.46228294	0.61028089	0.89472145	0.88803256	0.66785217	0.8734173	1.11285909	1.51581263	0.82584841
COX4I1	5.60015272	5.23244762	5.53148619	5.35641551	5.57286729	5.63686058	6.32593111	5.92000493	6.18523584
COX5A	7.02329577	6.03847866	6.67835004	6.17357347	6.72985365	6.08532908	6.49245557	5.67768076	7.58750279
COX5B	7.17253542	6.33933319	6.58670617	6.08517326	6.30073098	6.20941073	6.65666985	6.81644763	6.80499512
COX6A1	7.40166421	6.29572903	6.47871724	6.41092643	6.24788678	6.40454774	6.76924909	7.2492396	7.21715761
COX6B1	8.73433076	6.95394727	7.2048799	6.85653476	7.68989835	6.71551213	7.02509842	7.30585934	7.66634793
COX6B2	0.09250087	0.14561358	0.05418351	0.28980158	0.06332578	0.20269144	0.27982076	0.0718211	0.0829345
COX6C	5.03542576	4.87156439	5.39706173	5.55751829	5.25570484	5.03517408	5.46549975	5.06659409	5.16479837
COX7A1	2.55989704	3.95808233	2.42062498	2.59620755	1.34877054	2.49591644	2.33012113	1.69838099	1.38422391
COX7A2	6.54451511	5.61010908	5.61444523	6.19283024	6.45293886	5.79146237	6.3645679	5.28907819	6.25513621
COX7B	5.25073111	5.42346864	5.25098668	5.13446191	6.68056408	4.81812628	5.26676788	5.47452417	5.4743745
COX7C	6.93863257	6.42218913	6.34763624	6.78400228	6.88133493	6.26102123	6.19217135	6.76396143	6.97380218
COX8A	9.47727812	7.68653108	7.96699225	7.36679911	7.72014878	7.84654993	8.6895373	8.03532516	8.39220734
CP	0.54646251	2.77999003	2.33695807	2.24904012	1.37600059	1.88237567	1.80718031	4.29317379	0.21432047
CPA3	0.99847913	7.51755945	4.74871342	4.13138563	2.28751888	5.02711441	2.15482357	1.61602381	0.47301597
CPD	4.13716704	3.98017527	4.9136564	4.46170512	4.95290356	4.39508347	5.02743684	6.9874245	3.77955619
CPE	1.44631315	5.26954831	3.75689286	4.33031006	1.76959443	3.37385029	1.85088324	1.72568366	1.53994308
CPEB2	1.35176347	2.10583406	1.84550726	2.48471683	2.06533791	2.43114307	2.23002892	2.72485263	1.72234871

CPEB4	1.55358328	2.67762886	2.22858123	2.39577599	2.64235368	2.64090731	2.41918819	2.34551355	1.96758737
CPED1	0.44776557	3.93536471	2.64362146	1.7083982	1.61170317	2.57810257	1.91126015	1.39025717	0.88048988
CPLX1	0.49308074	0.58177717	0.986387	1.31353915	0.31564628	1.11053472	0.4662199	2.23773844	2.16048189
CPLX2	0	0.07728962	0.0545889	0.03047644	0.0094569	0.07175077	0.00919007	0.10859301	0.01342992
CPLX3	0	0	0	0	0	0	0	0	0
CPNE1	3.46103975	4.7856344	4.27175416	5.69728927	4.44958758	4.74097351	4.92605644	4.62746436	5.86966079
CPNE3	2.85675549	4.56685553	4.27125726	5.15759852	4.71239281	5.07753545	5.21050605	4.62582523	4.5333699
CPNE5	0.64638064	1.07728256	1.85268669	1.8588658	1.73897637	1.41097248	0.68686408	0.91881776	1.68859313
CPNE6	0.13343359	0.31468372	0.03898986	0.00727494	0.02229352	0.01775646	0.00363387	0.07308252	0.03472304
CPNE7	0.88391215	0.68496177	1.03832566	1.37360484	0.55052929	1.21133673	1.16369605	1.32085937	3.99482116
CPNE8	0.51533141	1.86867674	2.48443305	1.65462114	1.49943761	1.62764989	0.92116321	2.42640896	0.44799783
CPPED1	2.14300149	3.88233401	2.70479765	2.75839843	3.24116727	3.09779427	2.87738644	1.74416232	3.54010529
CPS1	0.98344898	1.76479421	1.5174316	2.67638258	4.30918227	1.2499887	1.80756706	0.31527437	0.52851842
CPSF1	4.18181938	3.54724858	4.1665697	4.16394966	3.78170666	3.74959244	4.18102729	4.0943514	4.86570006
CPSF2	1.81772279	2.72213823	2.80085701	2.87357829	3.22453675	3.18130103	2.82711892	3.25122531	2.91589362
CPSF3	2.81261067	3.43724815	3.76035387	3.81278828	4.30377539	3.96015172	3.85607376	3.63766805	4.14706469
CPSF4	3.42040769	1.64363465	2.52342654	2.00608918	2.16659345	1.98840102	2.01385361	2.05231538	2.20433712
CPSF6	3.06301437	3.12165492	3.55113858	3.79157932	4.15541363	3.61627485	3.31881522	3.5730039	3.88889628
CPT1A	4.17855045	4.60023494	3.8061298	3.42553609	3.05660015	3.91917857	4.34979087	4.32115299	5.33976876
CPT1B	0.2295079	0.14512389	0.22536105	0.75149028	0.589361	0.36948607	0.05222736	0.29614887	0.73711417
CPT2	2.96692131	3.42075097	3.26012268	3.49064753	3.47886015	3.64454406	3.49851161	3.57827088	3.72469782
CPTP	4.51860855	3.0015318	2.84612237	2.81481202	2.66301743	3.60224458	3.51777093	3.06996122	4.02398236
CPVL	3.24466794	5.10877635	5.14133939	3.83794278	4.42666147	4.72987216	3.03459977	2.47714146	2.41613118
CPXM1	2.58179459	5.42075843	3.39379903	5.08717059	1.62727742	3.70892998	3.13095205	1.88585711	1.1372171
CPXM2	1.15169938	4.21165471	1.67175537	2.70782494	0.3143037	2.81089021	1.0215603	0.58971814	0.7859124
CPZ	0.32097304	1.30161078	0.31446873	0.46271032	0.06992506	0.52227383	0.4891312	0.16537491	0.10190768
CR1	0.34347838	1.82809886	0.92675168	1.04184924	1.11953734	2.48958743	0.34975316	0.43736306	0.22252945
CRABP2	2.14463873	4.42678664	3.61459561	3.15297085	0.83034925	3.0761866	5.47915996	5.03339728	0.89302567
CRACR2B	3.36135426	1.2188101	2.26620872	1.36535716	2.45369994	2.37215378	3.8443319	3.81664865	3.27809768
CRADD	1.31200061	1.41592144	1.55261072	1.16840696	1.36506796	1.39157615	1.39134197	0.77242707	1.55165429
CRAT	2.04334853	4.33406	3.53378997	3.75974963	1.90212193	3.90543096	4.16981458	3.53180168	3.56831453
CRB3	5.22928318	2.60642107	4.14965436	3.61390584	3.81551221	3.63069018	4.27365391	4.88083145	4.09149955
CRCP	3.77182281	2.74725416	4.02877137	2.94169957	3.24958428	2.88807509	3.12486281	3.13706849	3.08907077
CREB3	4.88312451	4.59334004	4.93743147	4.09415135	3.590958	4.67824704	4.4972675	5.28362827	4.04718097
CREB3L1	5.92280972	5.45015198	6.11987499	5.08330964	4.12059896	4.56441311	7.59325937	7.2327565	5.46952434
CREB3L2	3.00974045	4.4615967	4.46570099	3.87472777	3.65058093	4.2124065	3.71832348	4.05248683	3.26401788
CREB3L3	0.40132889	3.66407449	1.04484452	0.0947941	2.16752288	0.07311932	0.03222321	0.17280628	0.78983733
CREB3L4	1.41223827	1.29276911	2.60061042	1.47762072	1.32221586	1.95359048	2.11524354	4.53387433	1.85964337
CREB5	2.48834419	1.05458662	0.7214107	1.36736281	0.49901995	1.77643262	0.46959053	0.58776351	0.21600999
CREBBP	3.52587506	3.64148781	2.49914456	3.00300875	3.17192833	3.24446818	3.1331796	3.52236223	3.2739854
CREBRF	0.82888086	2.39123141	1.81441769	2.01971022	1.96663318	2.19442588	1.48603444	1.95867314	1.6479814
CREBZF	2.89152729	2.03567159	3.08915453	3.27874135	3.2095323	2.60334946	2.83673183	3.12318853	3.36855332
CREG1	5.96730815	6.29384953	6.88893371	6.31855202	6.65253596	6.58287951	6.10387814	6.10516386	6.00741408
CRELD1	3.28381794	2.2349544	2.51349026	2.75374253	2.30511508	2.27059257	3.23165484	2.63121592	2.74055066
CRELD2	3.65829976	2.32935178	2.63175857	2.55361114	2.62817089	2.62024152	3.32233536	2.43289147	2.54174675
CRHBP	0.02577267	0.51967481	0.68889135	0.14108435	0.30886981	0.32765793	0.02316251	0.06850537	0.07328514
CRHR1	0	0.04996345	0.15200814	0.0458563	0	0.03622816	0	0.00426966	0
CRIM1	3.74075312	3.68972746	3.73116647	3.04592359	3.91860866	3.71257133	3.57940558	3.4703558	3.76472696
CRIP1	1.93968757	1.03839658	1.17837892	1.65669437	1.84002022	0.82023702	1.4954322	1.83602302	0.71945395
CRIP2	3.36769863	3.58457251	3.57889387	3.04338958	2.04518532	3.52863759	2.64723511	4.48445969	2.1813598
CRIP3	0	0.10659637	0.18132184	0.04389405	0.07142437	0.08993886	0	0.05063753	0.02324045
CRIPT	2.47690926	3.44087419	3.73568433	4.09316136	3.60656618	3.53617241	3.23934508	3.44824729	3.92120401
CRISP1	0	0	0.40981071	0	0.26580904	0	0	0.04834403	0
CRISPLD2	3.25271538	4.68479746	3.58423122	4.90317203	2.60128155	4.83549623	3.21640686	2.36286715	1.77777671
CRK	3.59092383	4.47723779	4.72729002	4.86989098	4.52501329	4.87479273	4.6833616	4.05988941	4.54330834
CRKL	4.59427344	3.94427779	3.9733136	4.12216962	4.08280159	4.13184187	3.75769095	3.45169292	4.42626526
CRLF3	2.33311977	2.27907771	2.79621346	2.7795702	3.16998819	2.80099277	2.38686221	2.46633132	2.20588692
CRLS1	3.28746981	3.1045727	4.41439282	4.33206662	3.16596407	3.24221362	3.63576193	3.15493108	4.53682574
CRNKL1	2.57704485	3.10342261	2.80617922	3.59304711	3.57628473	3.04179606	3.31947736	3.58169704	3.90488902

CROCC	1.18815147	0.88101529	0.80811984	1.09500506	0.61994166	1.20917922	0.64545646	0.66846824	1.02560698
CROT	1.48601777	2.759514	4.21737352	2.07741995	1.90227325	1.57519346	0.66277685	1.81583062	1.07137142
CRTAP	5.1352019	5.27818632	4.66570178	4.77467509	4.42326358	5.21155242	5.03651145	4.78325505	4.00500258
CRTC1	2.52752463	2.14932736	1.46648929	1.70910494	1.53343229	1.5618369	1.15630021	1.31055855	2.03357656
CRTC2	3.70601917	3.05294471	3.91379803	3.54840868	3.5148655	3.68385621	3.80338784	4.17431214	3.46223165
CRY1	2.03880781	2.13804544	2.65781934	3.01291931	2.18125919	2.49497822	2.45364746	2.39674951	2.32429434
CRY2	2.39558304	1.98950052	2.45268771	2.38315585	1.90050762	2.45514295	2.187016	2.31686669	1.98152821
CRYAB	2.88483301	4.81219425	2.16636703	3.46931762	0.85633271	3.83964382	2.05483007	1.14393262	0.94370028
CRYBA2	0	0.52394162	0.17059196	0.01496024	0.04559578	0.14030401	0.02970065	0.10253575	0.6968865
CRYBA4	0	0.16978936	0.62301736	0.22427667	0.38628642	0.09581478	0	0.01721697	0.21875631
CRYBB1	0.79373174	1.3352238	0.59779595	0.80129717	0.40577819	1.38799307	0.33424899	0.3789512	0.47049266
CRYBB2	0.08000841	0.09129608	0.24536208	0	0.12144443	0	0.04842457	0.01576749	0.02146128
CRYBB3	0.46339244	0.09323351	0.32493039	0.25373466	0.34396968	0.39007504	0.0249459	0.06339224	0.24340763
CRYBG3	0.54594512	2.24859198	1.38024763	1.90206077	1.01504783	2.26318683	2.42070312	1.49390015	1.52950188
CRYGS	0.43544221	0.31286713	0.86632889	1.07947415	1.05016615	0.40450073	0.20747175	0.60028309	0.66580351
CRYL1	5.68425529	4.73258797	4.87847106	3.47120563	3.38004928	3.53558263	3.62864598	4.41680435	5.29132978
CRYM	0.172372	0.5222192	1.43480556	0.38356434	0.05246255	0.34478354	0.08403088	0.3996909	0.58019099
CRYZL1	0.97163811	2.11629203	1.54329227	2.23643244	1.97639938	1.96350044	1.7641229	1.91132883	1.9074964
CS	4.70029249	4.72420964	5.11302301	5.27604039	5.54234429	5.45567557	5.29968294	5.52107679	6.11466812
CSAD	0.8293147	0.84346988	2.02877238	2.04055575	1.37979607	0.8742304	0.53728935	1.54629427	1.15184899
CSDC2	0.18892129	1.20446387	0.62208302	0.89741181	0.06087145	1.47023413	0.5918349	0.43350358	0.0802479
CSDE1	4.53095821	6.56698802	6.09717119	6.69016234	6.22181613	6.43361292	6.52688958	6.47263909	6.71356608
CSE1L	4.48023697	4.79264634	5.19124342	5.50951849	5.8579568	5.21566567	5.49886905	4.69926913	5.69907957
CSF1	3.2710823	3.37455402	5.50784256	3.47590479	3.68544898	3.79343763	2.73617307	3.0927825	3.11620526
CSF1R	3.52047875	5.96968209	5.46567082	4.41769429	4.85093387	5.58998104	5.28994741	3.96433207	3.50134797
CSF2	1.6556343	0.7005539	0.63836426	0.62219881	1.48514573	0.9498794	0.13234326	0.68365464	0.57463116
CSF2RA	1.3467615	2.11762403	3.20177098	2.10176949	1.93660376	2.05915476	1.78076075	1.6935583	1.0956993
CSF2RB	2.58540032	3.18544633	3.16011794	2.98387774	3.67378229	3.84399615	2.61501494	2.18149997	2.16999307
CSF3	0.79879296	1.5791706	4.15277556	1.55190366	1.26844663	1.42263439	0.30052835	3.12197131	0.07564456
CSF3R	1.91541614	2.17934939	2.40543737	2.63894995	2.47084951	4.37515198	1.88472597	2.24037833	1.66771641
CSGALNAC	0.7903065	3.07074547	1.93178066	2.45052709	1.63895128	2.54765028	1.52716215	1.70595499	1.13399831
CSGALNAC	2.09919461	3.94813124	3.68596868	4.01942286	3.55838657	3.81802347	3.18459699	3.46489996	2.84184034
CSK	5.39209895	3.64201865	4.26833008	3.64170223	4.22292926	4.00885902	3.88541714	2.99937787	4.78038637
CSMD2	0.11195947	0.43109783	0.34183983	0.39196925	0.0933622	0.36032166	0.24677948	0.09287423	0.23598398
CSN1S1	0	0	0	0	0	0	0	0	0
CSNK1D	4.43438761	3.07876268	3.82811431	3.27385997	3.54815053	3.32188117	3.2738141	4.07004475	3.89619285
CSNK1E	4.29910792	3.19182993	3.44719572	3.75249336	3.35971575	3.47398748	3.28378181	3.30096372	3.89270576
CSNK1G1	1.92554307	1.84883302	2.14175561	2.2779031	2.54209828	2.52237069	1.88659318	1.52148067	2.12804855
CSNK1G2	5.43923838	3.66510419	4.02127113	3.71544174	3.92910237	4.17651309	4.05335636	3.85990227	4.29085502
CSNK1G3	2.18001697	3.11875922	3.08204876	3.42073552	3.49535657	3.12595273	3.05482861	2.68240774	3.58495151
CSNK2A1	3.92793665	4.35315085	4.72595771	5.07790835	4.69727009	4.43267282	4.73347942	3.78073693	5.3768236
CSNK2A2	3.32243154	3.37161729	3.37026646	3.92548108	3.48288712	3.73402561	3.8365919	3.31851275	3.67130217
CSNK2A3	0.60156892	0.6051731	0.49819348	0.98112416	0.87598879	0.88407534	0.63721228	0.5668811	1.65847124
CSNK2B	4.65252406	4.25519817	5.02450385	4.78316728	4.7652915	4.3369286	4.54057543	4.76895861	4.78387271
CSPG4	1.73469916	3.2530164	2.55276335	2.50316165	1.43838077	2.75641188	3.4057788	2.09096626	1.48065566
CSPG5	0.08114886	0.0654332	0.27880416	0.90672147	0.30480608	0.47188033	0.96530296	0.20089716	0.14593431
CSRNP1	4.36365288	3.73529085	3.67100126	3.49086107	3.09407653	5.40465082	3.43427989	4.10187985	3.47535299
CSRNP2	2.58709447	2.54297797	2.86142584	3.00336229	2.72377788	2.95702165	2.23826398	3.07422332	2.68041823
CSRNP3	0.02587127	0.67909644	0.23536529	0.31037965	0.07993861	0.60270907	0.09572026	0.31778091	0.07138604
CSRP1	4.38390306	6.48321823	4.52070752	4.94956023	3.85502758	6.32118088	4.22426958	4.00449668	3.96408494
CSRP2	0.44043194	1.71867185	1.82708353	1.92009381	0.89622317	2.28965201	2.14298942	0.47121012	0.34375099
CST1	6.81458827	1.25935589	6.90166777	2.22259257	1.02510545	6.7084435	5.16764639	7.18636039	3.49162216
CST2	1.14783082	1.69777091	2.11046279	0.96518224	0.27268297	3.377597	0.78892613	1.17798416	0.38049016
CST3	6.44463057	8.12777203	7.32668886	7.7029003	6.30056693	7.30319026	6.62073104	6.74913771	6.81276519
CST7	3.64896417	4.07374164	4.3371567	3.46450571	5.09166615	8.61291026	2.93858819	2.36339791	2.34986723
CSTA	4.36678744	2.33540659	1.90236708	3.25790167	2.1151508	3.70933979	1.09383835	0.9478245	0.6061982
CSTB	6.58679556	5.63021132	5.84392653	7.64564788	6.63579184	6.89650081	7.59457769	6.71879522	6.38862539
CSTF2	3.10828444	2.44173139	3.79803003	3.37039345	4.04237515	3.13898579	3.25492826	2.82945325	3.567539
CSTF3	2.3644886	2.35060733	2.86930229	3.01250224	3.57006684	2.9369101	2.75734092	2.50523637	2.99297568

CSTL1	0	0.03839531	0.93996754	0.09845192	0.02077392	0	0	0.05143598	0.01774395
CTAGE15	0.05527825	0.08614024	0.0888102	0.08213698	0.15613264	0	0.21868033	0.37134573	0.12069332
CTBP1	4.49476316	2.98754225	3.03851253	3.3758289	3.5510334	3.52934112	3.4917776	3.66129004	4.06701679
CTBP2	2.5538202	2.99993511	3.23751911	3.29143756	2.9270208	3.70544937	3.53263627	4.27394505	4.0546175
CTBS	2.51921603	2.93388926	2.80070724	2.87461516	2.92649329	3.03027103	2.74012984	2.9773482	2.3316701
CTC1	1.31175402	1.57873611	2.09118873	2.17559571	1.90005973	1.87975732	1.94694033	1.47589852	2.25612901
CTCF	4.10030463	4.15336969	4.10869355	4.52085411	4.68219115	4.14804955	4.62277141	4.63561594	4.59608005
CTDNEP1	5.27451649	5.00581766	5.33137872	5.25053131	5.11871053	5.50555621	5.14156086	4.58176004	5.4903014
CTDP1	2.20302487	1.71766348	1.58664903	1.85449854	1.9898097	2.32606672	2.72782036	1.60181497	2.46463765
CTDSP1	4.99668273	4.40726179	4.79734871	4.14891773	3.80915002	4.79439124	4.12642845	4.98605219	4.39852184
CTDSP2	4.89178435	5.6808574	5.37044511	5.21579864	4.93521022	5.66930253	4.91416229	5.89274654	5.26990739
CTDSPL	4.37855	3.90982106	4.46414503	3.97181606	3.73568558	4.44661516	4.89209369	4.95272484	4.25920579
CTDSPL2	2.04749129	2.56258078	2.88411544	2.69333014	2.87910979	2.80244641	2.31533487	2.06708321	2.09092274
CTF1	1.03387144	2.2551978	0.90030971	2.08800567	0.5987267	2.25757154	0.73413249	0.8999298	0.41700661
CTH	0.19724319	0.85536056	1.83613117	2.31263941	2.47116615	2.36636428	3.05490735	1.79761151	1.71248885
CTHRC1	4.27099342	5.33893934	4.26452216	6.5824868	3.51110928	5.66186331	5.04354557	5.06247535	2.81886532
CTIF	1.87808629	2.37051531	2.32239589	2.0331337	2.02509793	2.31139924	2.28946077	1.68819679	1.73245054
CTLA4	1.06782004	0.57669645	1.91863039	1.59264722	2.69514157	1.58325316	1.45440511	0.53612997	1.36296029
CTNNA1	5.39102278	5.25737735	5.04320106	5.55800421	5.54926491	5.69915906	5.51388277	6.08087662	5.58205397
CTNNA3	0.00978489	1.03097281	0.13100387	0.05016813	0.00402665	0.48206414	0.02331932	0.04348592	0.04404747
CTNNAL1	2.61811689	3.16246566	3.53424679	3.01553974	4.23791393	3.81123503	3.16261754	2.20389478	3.19862689
CTNNBIP1	3.50170911	3.12258552	2.80429317	3.0177592	2.38478444	2.62796512	2.83523404	2.15272123	3.26628865
CTNNBL1	3.38988081	3.00712264	3.25733303	3.12983344	3.46266909	3.2299261	3.26411387	3.40538452	3.1382377
CTNND1	4.99451712	5.16575667	5.79468026	5.59600726	5.94366556	5.28928919	5.69199514	5.88421664	5.62528846
CTNS	1.93266463	2.48955742	3.28291965	2.70723862	2.79589982	2.97030392	2.37260328	2.46355707	2.36895
CTPS2	2.18976623	2.69848624	2.49158037	2.47807352	3.29414	2.18457362	2.46612249	2.83387145	3.57142042
CTR9	3.38125283	3.23950052	2.54999853	2.90982008	3.89428838	3.51789038	3.63287923	3.66919174	3.70797829
CTRC	0	0.04712913	0.33544952	2.21110233	0.02553479	0.01907968	0	0.04365655	0.01094907
CTRL	0	0.07491105	0.02880456	0	0.02459759	0.00983067	0.03178887	0.01031048	0.02795116
CTSA	5.22945242	5.95615841	6.78778983	5.80521238	5.66770553	5.81495328	5.95506369	5.01386474	5.52109807
CTSB	8.03673601	6.91777058	7.56479278	7.44491665	8.04250713	7.9361834	8.26266373	6.79104902	6.76263087
CTSC	5.57909863	4.92634506	5.45408723	4.27258284	6.14819089	5.54157934	5.1195872	3.82860496	3.97005668
CTSD	9.16531055	7.33448898	8.00108439	7.36266091	8.07121067	8.56866978	8.50715183	7.83910887	7.39753257
CTSE	4.3556903	4.75304943	7.21625759	7.73924585	8.4590727	7.81067189	9.0776507	9.34182923	1.76602952
CTSF	1.37160383	3.48708286	2.97852544	3.11203138	1.50750979	3.08469624	1.84814415	4.51304015	0.79378664
CTSG	0	4.67038781	0.60220147	1.24353666	0.07947222	6.44318813	0.20674278	0.06984381	0
CTSH	6.38665287	4.2115906	8.80443335	4.58226021	5.97554732	4.27326342	4.93760976	4.55743083	4.74850971
CTSK	4.37756395	7.88334349	6.38721337	8.26876797	4.97074546	6.89214923	6.23140476	4.99017522	4.44211672
CTSL	5.0050723	6.09841101	6.30351796	6.5374504	6.89832182	7.209045	5.93605411	4.58616251	4.52952661
CTSO	3.04621053	4.82541394	3.78545004	4.30845273	3.92529485	4.68387058	3.27530363	3.71362698	3.1269814
CTSS	5.74584093	6.50658046	7.18396247	5.69302964	8.03486159	6.81233583	6.21651268	4.50839381	5.93351495
CTSV	1.69178265	1.18560126	4.09160783	2.05664226	2.58013122	1.31329046	1.73943998	1.66210912	0.55204262
CTSW	3.31889623	2.10423837	2.55675414	2.75267888	3.77053416	3.41043573	2.70779637	0.63697892	1.39499683
CTSZ	7.4255892	5.94483203	7.09143879	6.62914442	7.35438783	6.67983456	7.1824764	5.66946084	6.06696445
CTTN	5.10194425	3.70899594	4.45168988	4.34618481	4.03808012	4.55375311	5.19210344	4.89044039	4.71539898
CTTNBP2NL	2.34209932	3.41390796	3.31331067	3.31104916	3.36792135	3.2660105	2.9054935	3.27663554	3.43740549
CTXN1	0.56158327	2.53431284	0.3473071	2.51072074	1.27320368	2.45241056	2.48375607	1.23431622	2.27988516
CUEDC2	5.06100872	4.87355053	3.77124631	4.29761829	4.39712501	4.51827873	3.7339201	3.80998002	4.61641053
CUL1	4.23593332	4.55909505	4.4477511	4.77131864	5.29647538	4.80655667	4.93043018	3.97803348	4.58258165
CUL2	2.49269647	3.10746865	3.25964627	3.3714391	3.99343575	3.47535487	2.97672105	3.23511302	3.362816
CUL3	2.32589595	2.89592679	2.67517249	3.26948515	3.47199763	3.00163977	2.95455249	3.29682042	3.23016421
CUL4B	2.27203451	3.50641827	3.73489214	3.57924347	3.99781338	3.54168944	3.22815056	3.3444781	3.3742285
CUL5	1.81779633	2.67708525	2.52376182	3.0253563	2.86637934	2.92963707	3.03716574	2.5063712	2.93825817
CUL7	2.89551802	2.54565277	3.02630326	2.7490012	2.39384993	2.75962891	2.94103265	3.32159118	3.02032113
CUL9	1.71123078	1.26847929	1.84364003	1.35823604	1.78622746	1.65958059	1.29225588	1.81367024	1.78821925
CUTA	6.32296182	5.67668966	5.3648466	5.28700249	4.86278767	5.14953531	6.06193392	6.04618827	6.33296511
CUTC	2.16893601	2.85308092	2.51838441	2.87827965	2.61317856	2.74296319	2.34179387	2.27776532	2.36778442
CUX1	3.6207736	3.47205998	3.43329131	2.95113924	2.6311091	3.13241889	3.16398352	3.14749032	2.98331006
CUX2	0.01422551	0.15021526	0.33081474	0.03948034	0.04337576	0.13527592	0.00853232	0.05937052	0.01990063

CWC15	3.77460976	3.69622252	3.8911148	4.13173172	4.10148825	3.81086813	3.96356112	3.840981	4.02940806
CWC22	2.76524715	3.1777559	3.11425681	3.71711999	3.84745982	3.31663869	3.08621794	3.25639739	3.57647314
CWC25	2.87352985	1.89627382	2.65376276	2.56640334	2.79598783	2.37835965	1.86892589	2.37645996	2.61049101
CWC27	2.64811742	2.43991129	2.12667439	2.8609598	2.99076399	2.72166518	2.46476538	4.05822071	2.83780082
CWF19L1	2.47618101	2.21812254	2.45151921	2.6505114	3.10818922	2.39187781	2.35322241	2.5344764	2.60363256
CWF19L2	2.23158397	3.17778284	2.05829537	2.5855862	2.70661001	2.75687095	2.03545809	1.73202511	1.9770122
CX3CL1	1.80019921	1.90682717	4.51201034	1.13631353	5.26483206	3.44532445	1.00790267	0.86536952	2.05286379
CXADR	2.67861838	2.33428037	3.52414303	4.02428341	2.92827638	3.4054014	4.13505694	4.72566061	3.68589207
CXCL1	8.28258679	4.35004956	7.64464512	6.43692691	5.73038257	7.94255067	7.3448971	9.00962542	5.38290507
CXCL10	5.71456354	2.58092996	5.89349564	5.92780573	9.83868361	7.06394891	4.6995754	3.12644379	5.37549029
CXCL12	1.82539779	5.37089379	2.61506698	4.55371194	2.7431576	5.17838078	2.1306688	1.19720418	0.81231294
CXCL13	3.14083846	4.32192412	5.09207107	4.31353565	4.76659227	4.06203992	4.23635336	2.52504668	2.68235711
CXCL14	2.92843012	6.46654166	7.33526073	3.20096011	2.18704674	2.46739017	5.65843074	5.92186522	3.06616667
CXCL16	5.8012588	5.58149342	7.04190324	6.56085303	6.76509082	6.45020532	6.37204226	5.70861227	5.9112828
CXCL17	2.81432865	0.69658115	8.72620764	3.65091663	7.28668597	1.49111903	4.92196991	9.4773433	0.16715964
CXCL2	4.99183028	3.1491975	6.24442	4.28318154	3.48255845	5.43794149	4.52338637	7.16590058	3.6468236
CXCL5	2.897202	4.11934149	2.35016997	3.91294795	6.74409819	2.59355274	3.72791842	9.56084756	5.9424247
CXCL6	0.82808351	2.46309116	3.14709935	1.07781177	1.34473485	1.68681503	0.36065675	0.63924222	0.86003285
CXCL8	4.77641689	4.77514414	7.00171823	8.21958761	6.53987588	8.54432513	5.80137518	8.08380141	6.14834626
CXCR2	0.21932587	2.0210479	1.42095948	1.44192214	1.08232288	2.2432196	1.62856913	2.34230852	0.61754613
CXCR3	2.74564124	2.5016196	1.93860319	1.29413934	3.3595319	2.18356875	1.4629194	0.75532258	2.37288014
CXCR4	4.17569294	5.70068831	7.14673555	6.90318048	5.19416555	6.22615279	5.68475653	7.46348706	4.05631174
CXCR5	0.03253046	0.31983278	0.18386397	0.09083734	0.04980005	0.1518854	0.0146955	0.0801536	0.02570973
CXCR6	1.988851	1.3002582	2.47922435	1.30553885	3.70124076	1.83134789	1.86075397	0.94665325	1.71808925
CXorf21	0.77243073	1.89622039	2.07292551	1.28083949	2.16940391	1.95124421	1.2837667	0.71399791	0.89451626
CXorf38	2.52117441	2.2601684	2.3829715	2.31620787	3.55827395	2.56737978	2.24291782	2.30149154	2.84288091
CXorf40A	2.55913336	1.62962521	1.79599647	1.0217187	1.67606903	1.39912747	1.48255195	1.55379612	1.99155779
CXorf56	3.00367581	2.48660251	3.39234979	2.65370696	3.32621546	1.89799854	1.96394701	2.93178576	2.3422827
CXXC1	3.07322045	2.18521759	2.98887347	2.97330317	2.89579908	3.09585677	3.46120371	2.39338463	3.46401036
CXXC5	5.0525487	4.14148482	4.12321279	3.32055156	1.65503692	2.85567512	3.89960832	3.88390023	4.30119884
CYB561A3	2.24133711	2.00930496	2.37771043	1.99400539	2.38422595	2.16909124	2.23752779	2.30658375	2.088447
CYB561D2	3.58156506	3.25489251	4.1479818	2.63107176	2.63050146	3.45509159	3.9101989	3.8139643	3.59179052
CYB5A	3.57801076	3.10968191	2.87416237	2.59649969	2.60405599	2.18903588	3.3313176	2.05382802	3.21495451
CYB5B	4.11296679	4.05999387	4.57581632	4.27625791	4.33731778	3.8144941	4.21293002	4.29855958	4.6987764
CYB5D1	0.12281891	0.41234168	0.49845808	0.53918782	0.37101202	0.89391373	0.59007691	0.62352755	0.80790627
CYB5D2	2.58530013	2.83339357	2.59740344	2.74770121	1.93789311	3.10743771	2.53671599	1.782738	2.94829445
CYB5R1	2.90914641	2.6288337	3.40315236	2.82454418	2.31024798	2.99785018	3.63676691	4.58370216	2.2639726
CYB5R2	1.03087679	0.55877233	0.7910766	2.20007542	1.41934072	1.46581247	1.97868267	1.79648795	0.43695406
CYB5R3	6.63804654	5.6841365	5.17375655	5.27523518	4.88243459	5.47385719	5.18388585	4.69201919	4.94154503
CYB5R4	1.60522747	1.79075378	2.09658905	2.44668167	2.73193464	2.3493934	1.93547513	1.46695053	1.55129039
CYB5RL	0.72393455	0.83484069	1.3270812	0.6311771	0.85432591	0.74433091	0.6479842	1.0724258	0.8411487
CYBA	7.15970359	5.56946879	6.40172505	5.16622589	5.45262476	5.91715748	6.86007068	4.82099049	6.35246337
CYBRD1	1.7388902	6.33372916	4.82570057	5.60217133	3.15598383	5.3421887	3.87908231	3.04103212	2.01618506
CYC1	6.48194556	5.68757519	6.63498691	6.13346192	5.96161087	6.21610788	7.19880961	6.4963943	6.75148629
CYFIP1	3.98997743	3.73781961	4.32538863	4.04051675	4.28265311	4.46818548	4.53708543	4.54630277	4.23714477
CYFIP2	1.06910283	3.06740197	2.20307458	2.83736888	2.826368	2.84649295	1.17108855	2.19623627	2.42371712
CYGB	2.69461315	4.21497415	3.95939478	2.72175357	2.09663635	3.0907141	3.30170459	2.67092522	2.09084117
CYHR1	2.43594502	1.6153969	2.37370666	2.09861539	1.48214279	1.97470321	2.27256915	2.44067819	1.98911095
CYP11A1	0	0.92872345	0.25511724	0.55471054	0.04433098	0.72600712	0.0790354	0.02789424	0.01093344
CYP19A1	0	2.3847286	0.23822783	0.09526524	0.04712175	0.12606115	0.88857973	0.02966088	0.00905382
CYP1B1	1.20823733	5.31031844	2.30422786	4.33399055	1.25206548	4.85434423	0.98412662	1.27779235	0.46211124
CYP20A1	2.2548298	1.65798929	2.02160985	1.6471501	1.62026396	1.29908226	1.70904574	1.70201505	1.57681868
CYP21A2	0.01247426	0.04937881	0	0.02237495	0	0.16099206	0.00748012	0	0
CYP26A1	0.04823567	0.0209199	0	0.17962411	0	0.09586677	0	0.00471859	0.38767441
CYP26B1	1.02692041	0.30973698	0.31055868	0.59236429	0.90456977	0.37420707	0.54729536	0.43030612	0.64765519
CYP27A1	3.97282359	3.17982999	3.53793029	2.75736138	3.80420492	3.63089983	3.13111965	1.84461789	4.24329627
CYP27B1	1.37492525	0.11092564	1.1333788	1.26894484	1.70973718	1.23871436	0.98570478	0.99731386	0.58298341
CYP2B6	3.22897198	1.7905039	0.8178475	0.32581555	0.1371502	0.14539389	0.08268529	0.11288037	2.1130668
CYP2C18	3.54949011	0.84950932	2.383838	2.20487373	4.58896785	4.04105661	1.81826012	2.54397716	0.60046294

CYP2C19	0.03836683	0.107268	0.05511459	0.02314062	0.24873465	0.26861042	0.02308897	0.02476246	0.42222225
CYP2C9	1.3909656	0.11239822	0.8427826	0.68171017	2.52142336	0.57152976	0.11062164	0.49947549	1.02303457
CYP2D6	0.36684829	1.55134275	0.45788762	0.40337491	0.44689083	0.2088568	0.27554953	0.21339906	0.76651623
CYP2E1	0.102348	0.09447871	0.2505928	0.35659743	0.27726737	0.25802621	0.02472996	0.08641894	0.07185305
CYP2J2	0.15034336	2.49471558	3.17808266	1.95949367	0.24143455	1.93424102	1.40805449	2.51725229	3.81361616
CYP2R1	1.24518386	1.37788172	0.95124972	1.99526127	1.65865518	1.7579157	1.80790212	1.9784687	2.20682992
CYP2S1	2.89586136	4.08322249	5.24248406	6.10169773	6.48085336	4.7775469	4.4461949	5.10914859	6.53962521
CYP2W1	0.25254606	0.70610557	5.87820835	0.95203948	0.09402662	0.81816099	2.48185924	0.91710055	0.40144394
CYP39A1	0.02324152	2.35247923	0.55514755	1.24127947	0.11774819	0.89934639	0.19638384	0.61840003	0.60070479
CYP3A4	0.06799032	2.6664087	1.82104006	0.06132924	0.35885835	0.12254116	0.00692895	0.43986061	0.77060777
CYP3A5	2.12025674	1.84847108	3.17449362	2.47615147	2.62141392	0.91478462	2.31186232	2.9947006	3.49139146
CYP46A1	0.02702447	0.30472377	0.25483677	0.14871373	0.01433298	0.13391021	0.0345741	0.06461858	0.02437398
CYP4B1	0.30291494	0.09908622	0.39669115	0.06184976	0.06347228	0.0077196	0.01255571	0.03608243	0.12203319
CYP4F11	3.64151711	1.70810801	1.27683574	0.13962209	1.3633667	1.36929834	1.83986224	2.23685947	0.46382882
CYP4F12	1.33079937	1.00622384	0.75613581	0.81713419	1.45220285	0.09677884	0.05989472	1.96763614	2.28866178
CYP4F8	0	0.12477034	0.02958126	0	0	0.00189868	0	0.01388393	0.01890199
CYP4V2	2.21883804	2.57042846	1.92063183	2.19849604	2.18328539	2.53613751	2.07656966	1.3390813	2.07391985
CYP4X1	2.88068599	1.9063509	1.70321602	0.61295289	1.30920733	0.86940125	2.52003325	1.80064845	4.75568976
CYP51A1	2.40351219	0.55815465	1.03124668	0.9430576	0.40149119	1.06439878	2.14962151	1.36987973	1.23826069
CYP7B1	0.43748318	2.88092916	1.07838674	1.76168601	1.16863295	2.20278965	0.76758329	1.93687438	0.23212528
CYS1	0.82534597	1.2276062	2.12614769	1.38864934	0.06174817	1.60757535	0.4782474	0.3984028	0.13480535
CYSLTR1	0.27872429	2.17530888	1.74368622	1.28637572	1.33161572	1.49109817	0.61287546	0.59151619	0.52940288
CYSTM1	7.85486972	5.68433518	7.24546797	6.26199878	7.64281536	6.91302467	6.14657583	8.62060539	5.90900022
CYTH1	3.15650768	2.37142193	3.70139486	2.72030825	3.31691186	2.86290837	2.57231874	3.42881462	2.76774242
CYTH2	2.74382355	1.7846628	2.20122309	2.59980951	2.52832562	2.25389741	2.3185716	2.71703375	2.81975727
CYTH3	2.13364153	3.00953964	3.27159189	3.02956911	2.2020092	2.73492698	3.1549702	2.51279654	2.43932528
CYTH4	1.7597325	2.30891048	2.52690876	2.12768861	2.64301965	2.44991731	2.32990972	1.44579678	1.7904138
CYYR1	1.32224155	3.83124795	3.26051899	2.6700319	2.24503406	3.20147021	1.70668758	1.32336394	1.37277354
D2HGDH	1.95989454	0.91902289	1.87129108	1.241057	1.60965318	1.3736226	1.56806256	2.19007782	2.0641619
DAAM1	1.30404277	2.36410761	2.03831386	2.44280595	2.08545391	2.08251018	1.69739334	1.86841997	2.14203226
DAAM2	0.83496114	2.98498493	1.70928716	1.97430793	0.94404467	3.41861411	1.51114711	0.98610486	0.71238397
DAB2	2.32953256	5.3561938	3.95373386	4.42057748	3.66359973	4.66144514	3.40383199	3.05290406	2.24564236
DAB2IP	4.05226639	3.30478987	4.55463354	3.29544644	4.64943075	4.10516768	3.28908156	3.36211047	3.64897857
DACH1	0.07895373	2.45595616	1.01391287	0.53323279	0.1982143	1.53764093	3.96034648	0.40030618	4.23454577
DACT1	1.5114812	3.44028694	2.08115571	2.77817425	0.63650548	2.65589429	2.14682125	1.02764288	0.91398847
DACT2	0	0.04860515	0.10228093	0.10963569	0	0.03755765	0	0.39347752	0.01354514
DACT3	0.71858008	2.64815437	1.48061675	1.59789255	0.20645869	2.31675082	0.48888642	0.52010476	0.2961602
DAD1	7.69262797	7.36901669	7.44029494	7.37298949	7.2095879	7.17135816	6.65920727	7.65031096	7.09098282
DAG1	6.04436908	4.86634049	6.0703558	4.94739888	5.94173229	5.18990287	5.65465884	6.04097938	4.90866914
DAGLA	2.18165247	1.75067058	1.40689355	0.9753855	0.9824769	1.36737453	1.49718731	1.56730676	3.37964844
DAGLB	2.38702279	1.96292016	2.71261639	1.93158663	1.99745895	2.13300967	2.21041373	2.18602297	2.36911912
DALRD3	2.76499046	2.0496271	2.56970786	2.44615869	1.54122737	2.69325285	2.92129123	3.46818043	2.53695521
DAND5	0.20388777	0.4383406	0.77639391	0.10553516	0.21865693	0.1159136	0.01088332	0.10836784	0.11075167
DAO	0.00935538	0.016039	0.32631586	0.01679501	0.01151799	0.02054431	0.0278219	0.05326641	0.01472513
DAP	6.4256333	6.059941	6.63066344	5.46904712	4.9676843	5.88140445	6.25307476	6.59558698	6.04477946
DAP3	3.96264272	4.10682905	4.32038328	4.60222166	4.69867533	4.37116452	4.78167573	4.77473079	4.51437195
DAPK1	2.88776402	2.38421212	2.87571598	3.04773408	2.70396715	2.71769471	3.53064248	2.97058191	2.34406736
DAPK2	0.43574593	0.91494667	1.08010135	0.33786093	0.82141657	0.37702687	0.26299261	0.12827518	1.04631324
DAPK3	4.0195461	3.43316313	2.88296915	3.18739265	2.8853644	3.93476761	3.44429739	3.62990135	3.18413292
DAPL1	0.3993557	0.06773882	2.60432634	0.22798299	0.04407404	0	0	0	0.51835709
DAPP1	1.05874689	1.51181596	2.27869181	2.22002454	2.63888475	1.95743317	1.40734944	2.15897007	2.07336735
DARS	4.39493921	4.97185252	4.96176747	5.27274973	4.94225246	4.96000923	4.9099879	4.7703591	5.22017665
DARS2	2.39300853	3.2317436	3.21977779	3.43715029	4.13474136	3.85107002	5.07340621	4.28999535	3.82848021
DAXX	4.29340418	3.84875338	4.81029757	4.11215257	3.91699935	4.31270247	4.25125142	4.57576721	4.38064436
DAZAP1	4.30292711	2.54960736	3.21124331	3.10354007	3.9001559	3.42732077	3.69174931	3.22368209	3.70298453
DAZAP2	5.1406108	5.69445512	6.23009142	6.0043107	5.27947015	6.08580849	5.67567273	6.06620728	6.08484536
DBF4B	1.75664447	0.60444868	1.10042375	1.23977346	1.61668697	1.11529027	0.74783894	0.67269382	1.22060939
DBI	7.01819301	4.45743119	5.92807201	5.04374171	5.76696556	4.9664573	5.84708657	5.1379614	5.33954698
DBN1	2.34094752	3.73078861	2.82713183	3.92230377	1.11394151	3.96329002	3.11876755	4.55276443	3.53624301

DBNDD1	3.65293945	1.66655693	1.31362316	3.00921257	0.71646543	1.99753417	3.63709711	3.58598883	2.38051165
DBNDD2	2.18174081	1.74440727	1.89033855	1.49312521	0.86554875	1.80808385	1.59658512	0.93799733	2.23975489
DBNL	3.94109368	2.64681307	3.42834026	2.65489552	2.63806038	3.01010643	3.42277107	2.92006103	2.73706468
DBP	1.57547092	1.30217569	1.59415301	1.11124288	1.07763593	1.39722666	1.10795257	2.03165572	1.74141202
DBR1	5.08950193	2.49650987	4.12456813	3.14745837	3.61391824	3.49649711	3.2243044	4.18967938	3.10035674
DBT	0.90257407	2.06349268	1.63183708	2.05859591	1.80407968	1.95630894	1.78307603	2.07426147	1.69246085
DCAF11	3.96090409	3.6436755	3.52597686	3.16616039	3.27070487	3.65451137	3.7182794	3.86564691	3.82474359
DCAF12	4.2773149	3.6488048	4.47587765	4.09024514	4.10882382	4.14694611	4.14507052	4.33612094	4.24839279
DCAF15	4.17564424	2.30129712	3.01911762	3.11220921	3.43603144	3.22023544	3.15802317	3.01191576	3.65755394
DCAF16	3.2210123	2.26956621	3.14167669	3.32742316	3.87784648	2.69453381	2.5015077	2.82234286	3.45837678
DCAF17	1.38768893	1.82291247	2.14416259	2.19866649	2.55926934	2.13358586	2.0585818	1.80416365	2.24083938
DCAF4	1.20672049	1.42784424	1.81743757	1.39769754	1.71288632	1.54393374	1.95512948	1.50526216	1.84915093
DCAF5	3.20517701	3.30590024	3.14033726	3.05489793	3.14745197	3.10863771	2.75054231	3.28111423	2.94442334
DCAF6	3.11960278	3.32780408	3.52936584	3.12309473	3.08113573	3.122388	3.34351934	3.17624777	3.0506231
DCAF7	5.11330128	4.39536258	4.90708019	4.38811785	4.82681322	4.75466857	4.47535331	4.99126832	4.92180074
DCAKD	3.57416172	2.98799406	3.08569457	3.00776392	2.64757859	2.85451044	2.42350558	2.29965687	3.36333944
DCANP1	0	0	0.01670048	0	0.0071439	0	0	0	0.00609773
DCBLD1	1.89498549	2.28658791	2.65765265	2.91859944	2.71457551	2.76377772	2.66346895	1.92436226	2.13108805
DCBLD2	1.02199481	2.08126743	1.86726333	3.86701878	0.88561222	2.18795678	2.37914757	2.49773078	1.3113115
DCDC1	0.04095379	0.01891105	0.07599291	0.02960126	0.01020018	0.02321542	0.01484325	0.03167096	0
DCDC2	0.21636729	0.94187385	1.62055474	0.2486366	0.07444273	0.0654976	0.0259653	0.48291221	0.19348489
DCHS1	1.46833825	3.05126471	2.02634064	2.19321024	1.27736319	3.15904234	2.05436138	1.55583497	2.11573851
DCHS2	0	0.27682381	1.16769656	0.27546297	0.01372462	0.254222	0.02392019	0.42574254	0.00821304
DCK	3.65192826	3.35319868	4.15586466	3.84369428	4.34920685	3.85695745	3.55052495	3.65856886	3.92054194
DCLK1	0.06687877	2.16732541	0.29394782	1.00191936	0.14970444	0.91918454	0.19025026	0.14136939	0.05234476
DCLK2	0.48130255	1.01263042	0.97495449	1.05819845	0.44630354	1.02908812	0.51732542	0.52274333	0.87390493
DCLRE1B	2.47805911	2.04065801	2.90132108	1.97918732	2.68415921	2.37860492	2.36426542	2.11226974	2.445002
DCLRE1C	1.70673418	1.6834201	1.68253985	1.45004881	2.20288228	1.4527043	1.46682658	1.25968099	1.78641842
DCN	3.20065946	7.79962192	5.41239198	6.9317782	2.97562591	6.13516655	5.00182508	3.95444022	3.0695851
DCP1A	2.66807341	3.11967583	3.52878413	3.55978984	2.80617927	3.30874927	3.36935623	3.28509945	3.66930369
DCP1B	1.7494449	2.92107118	1.9061553	2.54526015	2.02954116	2.61238116	1.81143525	2.00673541	2.79984504
DCP2	1.53928153	2.01818238	2.52407005	2.48689878	2.75765304	2.20526197	2.22386134	1.49011796	2.02754322
DCPS	3.07179633	2.3409663	2.41521302	3.03609472	2.74242551	2.63938611	3.33036524	2.59096977	2.80928937
DCT	0	0.06228941	0.1396279	0.10345459	0.00594074	0.01764416	0.00288935	0.01482125	0.01766913
DCTD	4.34804663	4.72745139	4.3049979	4.97366394	5.16720313	4.97330509	5.03125579	5.09749998	4.79573418
DCTN2	4.37881844	4.43169483	4.51311911	4.62153613	4.18505548	4.59948703	4.50634018	4.69376781	4.63072329
DCTN3	3.41307133	3.34341874	3.77685454	3.70841355	3.48954476	3.52484031	3.07717629	3.44423744	3.56811434
DCTN5	2.46231987	3.11375389	3.26232449	2.72029636	2.85639366	2.82814581	3.36520899	2.98956084	3.17732972
DCTN6	3.31485665	4.45782437	3.7687247	5.11510572	4.39568192	4.6239909	5.03990953	4.30136762	4.28630914
DCTPP1	5.9423718	5.40243126	5.29611067	4.94413612	5.23897262	4.95827825	5.4617222	5.30430634	5.85407202
DCUN1D2	1.54793494	1.30740315	2.18938187	1.93798757	1.58405175	1.41574664	1.94213322	1.70309542	2.20329478
DCUN1D3	1.61828695	2.1585378	2.35823453	2.26555222	1.81482353	2.05804844	1.59035778	1.73200583	1.54866712
DCUN1D4	1.66750588	2.51638172	2.25030567	2.54005265	2.7086558	2.41376569	1.98799407	2.0646463	2.13612997
DCUN1D5	3.8393967	3.03727843	2.94468193	4.02667244	4.1145438	4.13688515	3.82515162	2.6936651	3.7242376
DCX	0.01430836	0.75771408	0.09303028	0.02776907	0.00221291	0.0209802	0.00215032	0.08213494	0
DCXR	5.8761581	4.34075839	5.22923693	4.85445822	4.13493644	4.02106313	4.63894544	5.27336528	5.71332192
DDA1	3.82471841	2.7039726	3.31132439	3.31514151	3.50089125	2.99775333	3.30503269	3.18332502	3.44792931
DDAH1	5.80530103	4.24200189	4.51911904	4.98079805	4.67285195	4.50751767	4.812399	4.73720085	5.70109985
DDAH2	5.91104314	3.84780105	4.45785756	3.23937341	3.66668039	3.80217312	2.99800123	3.58478114	3.74580092
DDB1	3.67966658	3.86118108	3.92464941	3.9931763	3.96116291	4.18703781	4.56510258	4.39445222	4.53518452
DDB2	2.79764352	2.749221	2.64671232	2.9091492	2.82038064	2.56385459	2.6768751	2.48767171	2.61147615
DDC	3.65901029	3.87677252	3.06784922	2.12074555	3.45478729	0.96571766	3.54962957	0.62894051	5.38940236
DDHD1	0.30791774	1.18273462	1.26090649	1.64037556	1.56222769	1.38217611	1.03742482	0.85693253	1.06453454
DDHD2	1.16268501	2.02685531	1.78480354	2.84474154	1.91175032	2.29532499	2.14229878	1.91555088	2.19885066
DDI2	1.67722389	1.87982949	1.91919115	2.15438617	2.32476314	2.14835842	1.88806802	1.88894999	2.43429807
DDIAS	1.26051109	0.80124271	2.48042181	1.67551284	2.42317775	1.83991941	1.75181878	1.15293418	1.4599045
DDIT3	4.60798418	2.83545961	3.62926489	4.10813303	3.57330843	4.20318081	3.85771781	4.23177689	3.06039021
DDIT4	7.49631122	4.63758058	4.04994361	5.39678002	5.20628579	4.91077826	5.48685746	5.12387847	3.77655239
DDR1	5.48327009	3.34379668	5.66328969	4.91958338	3.91584898	4.15249454	5.17382562	6.34070596	5.07354892

DDRGK1	5.29009259	3.96312853	4.61709477	4.11663165	3.86957789	4.26407665	4.66814031	3.97563982	4.49357203
DDT	4.55957487	3.76425011	3.08146758	3.17778194	3.01353261	4.15168257	4.10144768	2.76992322	4.38952616
DDX1	4.03407762	4.37354924	4.58654699	4.96023097	5.19947644	4.82620012	5.11076512	4.68186146	4.88588021
DDX10	1.69299774	1.85599204	1.67717649	2.33271278	2.41156593	2.36055978	2.21375641	1.5620824	2.55176194
DDX11	1.60659035	0.98405855	1.26255409	1.66120398	1.60608669	1.51540188	1.59746624	2.103851	2.24314519
DDX17	5.10062477	4.69998826	5.60422597	5.74507625	5.71846596	5.01825332	4.76639321	4.75344776	5.62966828
DDX18	2.81613588	3.38890419	4.05820225	3.87360553	4.15999394	3.82946496	3.85222419	3.71573178	4.12163117
DDX19A	2.00702489	2.4560403	2.23988119	2.60410535	2.56701823	2.75411119	2.72093513	2.5498642	2.55636831
DDX19B	1.9817145	1.82012201	2.00824972	2.17926578	1.73098724	2.10520478	2.10228654	2.02132737	1.84277273
DDX20	1.68903441	1.92626561	2.17158794	2.25763043	2.7941831	2.08978818	1.67843966	2.01474769	2.64397421
DDX21	4.00959007	4.6033281	5.35789038	5.59233733	5.7256526	5.7300294	5.41634947	5.37466856	5.61738413
DDX23	4.55069975	4.05722958	4.39175685	4.29063226	5.0128123	4.40220278	4.285486	5.19977933	4.62213195
DDX24	3.59635675	4.1285062	3.6551119	4.22338857	4.00146906	4.0155716	3.6875286	4.31297992	3.99003612
DDX27	3.38391237	3.33216242	3.73272304	3.00029937	3.63277766	2.89290486	3.59705078	3.66339477	3.55850481
DDX28	2.30691433	2.14698429	2.8072319	2.52043064	2.60402748	2.7105982	2.57457231	2.47059969	3.11106262
DDX39A	4.59826783	2.55021365	4.01701811	3.14443739	4.41195139	3.65088767	3.83182298	3.45644389	4.21622942
DDX39B	2.32659675	2.17596062	4.09629762	4.38418789	4.1217203	2.87024525	2.82053936	3.60001874	3.79774685
DDX3X	3.84574438	4.82425977	4.88901341	5.53985215	6.33932322	5.17782315	5.28109415	4.99730097	5.67254039
DDX41	4.09784054	3.42351536	3.8462117	3.67180254	3.59924284	3.72108678	3.50393069	4.08871681	4.17196882
DDX42	4.17461061	3.4659247	3.86181501	3.89363718	3.8382582	3.60751431	3.68521729	4.39330271	3.84080213
DDX47	0.33987744	0.32828485	0.7950148	0.88831785	0.76631676	0.63457835	0.35843437	0.5050828	0.98747681
DDX49	4.49996975	3.08664694	3.76185626	3.29906398	3.93202239	3.75670104	3.76876907	3.781553	3.93642214
DDX5	5.25942688	5.21736215	6.46264313	6.16098141	6.32024304	5.86930243	5.36718114	5.94871399	6.28198312
DDX50	2.72705237	3.68213144	3.68909437	3.58575778	3.94000846	3.97949723	3.36646356	3.59000634	3.7653017
DDX51	2.30713838	1.01325638	1.62237927	1.52064338	1.46487085	1.48740877	1.57598866	1.69778739	1.86602525
DDX52	3.47981995	2.19688473	2.33936426	2.6765655	3.04091608	2.34118837	2.07034857	2.433785	2.79369029
DDX54	4.48031775	2.81217075	3.01211046	3.12756268	3.32121274	3.57668259	3.51369669	4.14679504	3.88704363
DDX55	1.5475213	1.36322368	2.25270367	2.05900447	2.43692418	1.84890359	1.71074609	2.45991302	2.20516363
DDX56	4.55981433	3.52359625	4.21340652	4.17559602	3.92494974	3.87630126	4.18437375	4.04833754	4.38146843
DDX58	1.35020218	4.43426183	2.59244249	3.22912816	3.91989865	4.3820686	2.52848186	3.18785055	2.51960713
DDX6	3.44950374	3.61300497	3.1881208	3.63304361	4.09977474	3.44063072	3.58660391	3.32994772	3.60308209
DDX60	1.35226595	4.32883402	2.42764728	3.05770694	4.42971492	4.75634798	3.12395195	2.69111797	2.70049506
DEAF1	3.93644354	2.53132998	1.78033457	2.86748985	2.37897912	2.78181674	3.06710279	3.02820445	2.42878828
DECR1	3.59609696	4.76181557	4.19322701	4.61374805	5.18719146	4.85131748	4.6016918	4.23999517	4.67846981
DECR2	2.99107352	2.60347219	2.12791141	1.86161466	1.80887573	1.92984833	2.34053998	2.35252805	3.0826837
DEDD	3.82047078	3.7367078	3.91125271	3.903348	3.43997401	4.0475778	4.13188679	4.21283486	3.54773926
DEDD2	4.26626013	3.22390867	3.70741887	3.47374839	3.12710418	3.70297408	3.51280441	3.78664811	3.73328846
DEF8	1.13534389	2.33092869	2.44792959	2.39994302	1.95346745	2.41771913	2.50133211	2.38632512	2.3692143
DEFA5	0	4.47701474	4.3725302	0.0942491	1.12329414	0.11461759	0.77167974	0.47541922	4.02587012
DEFB1	3.28878666	2.23055705	3.75739143	2.03818312	0.95914488	1.00700024	1.13891451	1.05627527	0.55802059
DEFB124	0	0.39789417	0.07048597	0.39160287	0.11821343	0.12316952	0	0.12894071	0
DEFB125	0	0	0.23154539	0	0	0	0	0	0
DEGS1	3.90610947	5.15548339	5.27664353	4.9001187	5.40080671	5.0812231	4.98698506	5.29798198	4.4575321
DEGS2	5.91630383	3.57947911	3.54827018	2.22400897	2.75778331	1.08844269	2.63168385	4.95655069	4.65734823
DEK	5.00635445	5.20584686	6.04697288	5.59527751	6.10706732	5.60144618	5.84347819	5.83312391	5.68526349
DENND1A	2.04979578	2.17275478	2.10824517	2.44926018	2.91542294	2.57823758	2.15720584	1.71215683	2.65754558
DENND1B	0.9949557	1.43462208	1.75362333	1.23685068	2.00204474	1.64231288	2.24299314	1.14845887	1.65453376
DENND1C	2.74440016	1.79241871	2.52350938	2.30578903	2.73280746	2.22810685	2.05526368	2.75699475	2.39658236
DENND2A	0.38862706	1.72212367	1.61006931	0.85802465	0.61260915	1.08627781	0.99057128	0.67424087	0.89646812
DENND2D	3.58910914	2.79168692	3.78523975	3.38781658	4.25543485	3.7689865	2.16775463	4.31321343	3.38546504
DENND3	1.18431657	1.15958741	1.79110066	1.62035695	1.53707772	1.75089404	1.59619937	1.63901189	1.01507306
DENND4A	1.41905281	2.74832878	2.61931155	2.27333829	2.69103276	2.61738404	2.12209811	1.95694848	2.49742976
DENND4C	2.63286314	3.40850313	2.77254207	2.86339723	3.76168361	3.02895153	3.20485998	4.21533432	4.16102333
DENND5A	1.47150547	3.32893699	2.58636634	3.17586325	2.33906166	3.52408836	2.7487596	2.6577874	1.85577582
DENND6A	1.81325381	2.75720372	3.13755787	3.05515271	2.48183735	2.94018421	2.86571135	2.82578184	2.65226203
DENND6B	2.39290585	1.1781826	0.81268	1.47104866	0.94138135	0.83472135	0.67003714	0.72787702	1.24033165
DEPDC1B	3.32769592	1.28065962	2.63403975	2.45728464	3.38704421	2.18842558	2.00562353	2.79707274	3.06435291
DEPDC5	1.60865027	1.52087239	1.99739506	1.54112195	1.87490365	1.67722665	1.43945406	1.41633065	2.00425042
DEPDC7	0.16546001	1.79539521	0.50844822	0.99191665	0.26447218	0.6955718	0.31674071	0.19706968	1.62480171

DEPTOR	4.0629773	3.72687001	5.01788508	3.62334993	2.81843951	3.3420046	3.50197453	2.91013904	4.3932768
DERA	3.24260046	3.62537006	4.19889164	3.72052862	4.02045992	3.57373953	3.65921694	3.26916137	4.32312797
DERL1	3.47383849	3.71390794	4.6271498	4.027876	3.93283902	4.14178617	4.52933648	4.08169038	3.80261555
DERL2	2.19035365	2.57805317	2.89008845	2.93411556	2.93185714	2.71676704	2.40088713	2.05202037	2.74716282
DERL3	1.46303349	1.44518048	3.33577938	2.97146419	2.48538052	2.24520833	1.02555387	2.08650066	2.91615193
DES	2.56086415	9.8200005	4.12888724	5.63401469	1.72477872	9.67208423	1.69053664	2.05286957	3.63451121
DESI1	4.19378072	2.95832501	3.41032396	3.07268061	3.33074825	3.52131245	2.45896544	2.90224725	4.06161316
DESI2	3.31591523	3.79576212	4.08028864	3.9363675	4.14041698	4.10580952	4.73645942	4.45647377	3.67816181
DET1	1.75531855	2.04432388	1.5674302	1.74628083	1.45051154	1.42016381	1.02935974	1.50530552	2.27996437
DEXI	1.25921165	1.62869702	1.00493904	1.55831516	0.95597062	1.71843269	1.95291659	1.27788633	1.57242373
DFFA	3.70355703	2.87645094	2.72012358	3.4253368	2.7878051	2.95347838	2.5912348	2.56371801	3.10395744
DFFB	1.49630586	1.27771995	1.46909032	1.78561775	1.96024969	1.72038773	1.96322434	0.83384685	2.78045181
DGCR2	5.61813586	3.97960559	4.32276618	3.77618215	3.72267295	4.32096873	4.24839229	3.569623	4.39330259
DGCR6	0.18195734	0.06211914	0.13371445	0.04499951	0.18777035	0.27699685	0.14952412	0.16860514	0.51754114
DGCR8	2.55573613	1.55121441	1.95203716	2.30412345	2.55851583	2.22445936	2.06472426	1.90068459	2.63832485
DGKA	0.89000764	2.06256539	1.81670252	2.80338466	1.70254479	1.82545371	1.65443259	2.58179743	1.42194949
DGKD	1.59662536	2.00011055	2.33506464	2.19191269	2.30042146	2.20735234	2.17807182	2.66991605	2.80480182
DGKG	0.04895713	0.58453208	0.37027077	0.30092733	0.29157927	0.87737502	0.13280395	0.69277919	0.12687536
DGKH	0.89678443	1.34444138	1.5464974	1.41025149	1.46142467	1.58033556	1.61748718	1.32384207	1.57559492
DGKQ	2.7014864	1.95908569	1.4369905	1.98254512	2.27094395	1.97733792	2.25024436	2.8886988	3.63664514
DGKZ	3.91718432	2.94543386	2.73540101	2.40236902	2.97505864	3.32829716	3.53545878	2.73400447	3.53818262
DGUOK	4.83032307	4.58212984	4.85720037	4.78405871	4.62404224	4.4349831	4.27223671	4.20987366	5.06640033
DHCR24	7.19892233	4.22530074	5.53722303	5.52548033	4.82636372	5.82658743	6.06345428	6.5875688	5.75834629
DHCR7	5.07651973	2.90373694	3.59269021	4.48404678	3.17022524	4.27703232	5.1032211	3.98856734	4.72324623
DHDDS	3.04673942	2.42975719	2.38950729	2.82324646	2.88407605	2.67594436	2.89520026	2.21027982	2.95610594
DHDH	0.8436147	0.24780494	0.8427826	0.76639469	0.78225008	1.39110791	1.06427642	2.50221128	0.43959383
DHFR	2.51052055	2.02917098	2.42413545	2.11009005	2.55445976	2.69922176	2.45217582	2.47110152	3.32437444
DHODH	0.66024563	0.75875436	0.9823551	1.20896473	1.17149537	1.12266139	1.05916086	0.99813169	1.05002671
DHPS	4.06017095	3.114125	3.57193132	3.40527249	3.75406486	3.42059526	3.63393153	4.26375088	4.12990682
DHRS1	2.81043002	2.49645651	1.68142421	1.75639444	1.93428068	1.79562744	2.45584937	2.09032271	2.80011744
DHRS11	2.44483142	2.76590203	1.58407139	1.82557575	2.20028051	1.95366153	2.39148025	1.49204212	3.120882
DHRS13	2.74549499	0.57676821	1.267376	0.8668613	0.74100205	1.16084441	1.39411137	1.93770419	1.63939829
DHRS2	0.18532606	0.23880135	0.09441275	0.15877273	0.39519263	0.1432802	0.03423768	0.03126185	0.58239091
DHRS3	4.6032615	4.00761858	4.61710326	3.5395447	4.12592815	4.01987226	3.66505607	4.49326271	3.71267565
DHRS4	3.01237176	2.89180685	2.33288041	1.99225397	1.72640793	3.05250757	2.23871649	1.97041153	3.01537386
DHRS7	3.08044302	3.92243506	4.5224124	4.61767825	3.92887426	4.3949271	4.6395681	4.40198641	4.06343038
DHRS7B	2.89677012	2.07213696	2.56595867	2.09685796	2.19326129	2.07935128	2.00993338	2.30221097	2.08263305
DHRSX	3.60305605	3.00989715	3.38862912	2.94854109	3.48893635	3.28706375	2.21882299	3.45836652	3.40291212
DHTKD1	2.35440128	2.67509801	2.11716475	2.53209613	3.00825713	2.42804145	2.6289336	2.13982442	2.73113524
DHX15	3.70791832	3.63692755	4.64202096	4.77773184	4.97619679	4.58944509	4.1929426	4.72025943	4.8341542
DHX16	3.6310117	2.44472533	3.86082425	3.09936158	3.27124497	3.14614704	3.24798246	3.91129185	3.48252979
DHX29	2.54859321	3.38218557	2.77819662	3.03452859	3.86266658	3.24515851	2.96536822	3.26412306	3.31639553
DHX30	3.74267497	2.63229528	3.1772688	3.09634866	3.40815739	3.37556975	3.38315963	3.74042466	3.56541515
DHX32	1.79221646	3.2611356	4.00963068	3.29485271	1.62537529	3.20351869	2.9815376	4.03550563	4.03005953
DHX33	1.81775089	2.05884301	2.47012114	2.89824729	2.99940138	2.84311991	2.60490771	1.88275332	2.83078714
DHX34	3.04227825	1.38015701	2.41726539	2.05970562	2.5822916	2.07721773	1.8926704	2.39497001	2.32000535
DHX35	1.63980901	1.41369371	2.52160471	2.44827243	2.24700114	1.77130518	1.73069771	2.0316702	2.47576775
DHX36	2.67820359	2.37596113	2.48626813	2.73322153	2.94341438	2.36987332	2.33596944	2.3684629	2.39868078
DHX37	2.75213553	1.66798229	1.97405432	2.08267474	2.29933969	2.51019355	2.05898975	2.86462684	2.76565466
DHX38	2.44148894	2.62487408	2.74746026	2.92008613	3.21473371	3.04551518	3.33495165	3.19266423	3.38110852
DHX40	3.01473551	3.05683274	3.95519923	3.24761282	2.43913557	2.97698452	2.34598868	4.71099651	3.07737782
DHX57	1.42727769	1.99442928	1.86570387	2.23999993	2.37417204	2.15906859	1.6714834	1.80267198	2.0384421
DHX58	3.48568134	2.4642266	2.18207248	2.17689337	2.61986915	3.4672057	1.1783019	2.4058308	2.02930332
DHX8	3.26599608	2.74608328	2.97869705	3.05752182	3.04658279	2.99363633	2.94320568	3.43717699	3.41353084
DHX9	4.6497696	4.70185965	4.87897477	5.14647731	5.79713146	5.25149325	5.55829419	5.54144665	5.51204051
DIABLO	1.59021712	1.14567275	1.49249265	1.37719706	1.65012675	1.35361143	1.32062121	1.70338635	1.49585567
DIAPH1	4.9950548	4.30052109	4.97488704	4.80840638	5.27062994	5.30743017	5.19994073	5.76513838	4.55961865
DIAPH2	1.5224607	3.17696834	2.8800829	2.73673778	2.86529518	2.67388647	2.5378706	2.46932907	2.80814368
DIAPH3	2.08167079	0.94778692	1.38076543	1.62270904	2.17296966	1.94154331	1.80727988	1.15179931	2.26832571

DICER1	2.10915791	2.82309478	2.72432309	3.0604788	3.6920112	3.11412081	2.60084907	3.35041521	2.83422823
DIDO1	2.56438142	2.51925663	3.08612011	2.77867202	2.55505992	2.69113143	2.60741781	2.78261186	3.40165747
DIMT1	3.09640058	3.5557502	4.385892	3.96910389	4.18052261	3.8143413	3.06952282	3.35447771	3.8147919
DIO2	0.95641987	3.99449724	3.42184448	2.21611302	1.31914297	3.58848453	3.02952495	2.34507165	2.08673739
DIP2A	1.4921404	1.50810331	1.72805007	1.78447678	2.53876697	1.90776453	1.89809761	2.27781998	1.85522776
DIP2B	2.9494287	2.924977	3.05303327	3.74258831	3.61400768	3.40131774	2.96936061	3.71962001	3.111808
DIP2C	1.25763716	2.72928913	1.51357697	2.25706349	0.68801627	2.55904629	2.09338867	1.73847351	1.42464996
DIS3	2.33622703	2.77653746	2.58884976	3.10263241	3.27921714	2.96901546	3.07201663	2.74606085	3.40675684
DIS3L2	1.49137649	1.00506492	1.43294878	1.31416383	1.73707767	1.54954421	1.34894719	1.68129224	1.78964141
DISC1	0.21718244	0.87158327	0.82201008	0.65358605	0.71501153	0.7276083	0.47161128	0.47746114	0.26131472
DISP1	0.74088888	1.85438652	1.52708712	1.39687288	1.47570439	1.59512963	1.02629015	1.85363404	0.84353377
DISP2	0.57264697	0.32146429	0.27883534	0.54350147	0.21393204	0.6133453	0.08631358	0.12140099	0.28403431
DIXDC1	0.97443223	3.10343193	1.58860873	1.99466257	0.84150956	2.88002388	1.57519184	1.1331993	1.15077398
DKC1	4.13953286	3.76448905	3.90182562	4.4736284	5.31618164	4.27305266	4.35935242	3.87478075	4.85503127
DLAT	3.13006386	3.37337614	2.89333235	3.8820722	4.02948632	3.76020358	4.53541366	2.87189811	4.33050673
DLC1	1.05216977	3.72773649	2.47835719	2.97009634	1.89338124	3.24361553	2.37474359	1.82788825	1.86316789
DLD	3.85284252	4.53506184	4.14910437	4.45825284	5.05104683	4.41688043	4.8958414	4.01061041	4.69421729
DLG1	2.77969591	2.67199577	4.36951821	3.02512487	3.29304836	2.75234591	2.98894054	3.90915125	3.3736926
DLG2	0.06423942	0.88955013	0.31090228	0.47311769	0.03174394	0.6581456	0.04406703	0.05482076	0.04685787
DLG3	2.78861175	2.67461938	2.71489776	2.56589644	4.15334854	2.15510959	2.87172382	3.42092743	3.36675607
DLG4	0.97292194	1.8775032	1.59913367	1.62221121	1.01123809	1.65387804	1.0393605	0.63134197	0.62147047
DLGAP4	3.50610001	3.64839202	3.08647868	3.80846101	2.79578766	3.41653625	3.22493161	3.08798283	4.19807458
DLGAP5	2.27003809	2.17121498	2.78508208	3.17678964	4.2188275	3.21638989	3.04503888	2.749989	3.01873355
DLL1	1.06244112	1.79926317	2.40125375	2.28288349	1.20922607	1.81455792	1.99151759	1.77548426	1.8795384
DLL4	1.71607223	1.58795999	2.3474963	1.50906752	2.16154453	1.81201358	1.68166619	1.73513585	3.51108577
DLST	3.9951278	4.60541338	4.25566893	4.50427771	4.35242252	4.48009579	4.82842998	4.5072233	4.74845804
DLX1	0.01980057	0.017029	0.090712	0.01783139	0.01830651	0.0784088	0.03536629	0.03417075	0.00523034
DLX4	2.15456475	0.022735	0.19565479	0.45434823	0.04249681	0.2706094	0.29659033	0.12544517	0.26353496
DLX5	0.26298736	0.24276688	0.21875147	1.3648947	0.05805532	0.30463313	0.24310426	0.06521567	0.09770684
DMAP1	2.38365144	1.79842843	2.64940226	2.29853701	2.10128221	2.07588542	1.8188321	2.33529227	2.5242278
DMBT1	6.72287284	6.02758176	2.22245124	0.1947888	5.45776293	0.53101317	8.48709853	3.05816262	8.76804131
DMC1	0.12854448	0.1623091	0.48950031	0.38918732	0.80066494	0.26956134	0.12447766	0.04319276	0.1199065
DMD	0.10777111	1.92076625	1.11952078	1.05271758	0.46969117	1.77841089	0.30988361	0.34435112	0.19151897
DMKN	2.33489244	0.14400322	0.85915267	2.94989216	3.24829338	2.26990561	1.85457659	1.26273684	0.0355859
DMPK	1.74190621	3.7494311	1.52263319	2.21114455	1.68299428	2.84673016	0.96575726	1.69151582	2.43709331
DMTF1	1.55951277	1.69818744	2.7437032	2.55930781	2.7337475	1.9858098	1.59765949	1.93103263	2.42220439
DMTN	4.25980274	2.59687365	2.81697331	2.89355907	3.16523218	2.89508971	3.70733629	4.01674778	3.13384352
DMWD	3.33553413	2.31447393	2.20863814	2.12932393	2.29607779	2.50386615	2.04228525	2.56890178	2.25512028
DMXL2	1.62649035	2.12152586	2.39042143	2.56369456	3.4472476	2.36677117	1.93158338	1.16965084	1.41138968
DNA2	1.43149668	0.76187783	2.31248137	1.7803774	3.17966078	1.43151388	1.88630989	1.44594896	1.74139601
DNAAF3	0.93964161	0.12700633	0.16555469	1.29572566	0.68946876	0.20387878	0.13247346	0.13008226	0.38917468
DNAAF5	3.80816083	2.48758588	2.82707354	2.63586726	2.68665679	3.02637437	3.38364868	3.03927276	3.31806605
DNAH1	0.56100469	0.34619796	1.11129819	0.69747443	0.83344071	0.49995738	0.37826354	0.72914303	0.97934796
DNAH2	0.09415346	0.17008414	0.43923236	0.64830371	0.21057526	0.17264733	0.58602581	0.1263624	0.03947843
DNAH6	0.10904037	0.04561351	0.63946922	0.28533352	0.05380895	0.11700247	0.06779824	0.3102412	0.19835086
DNAI1	0.02002359	0.0057632	0.14537788	0.01204658	0	0.00369946	0.00602231	0.01929858	0
DNAI2	0.02534255	0.02899552	0.37947666	0.06008613	0.03116111	0.02329514	0.02277561	0.02926265	0.15890211
DNAJA1	5.69129685	5.96822175	6.0519807	6.45367622	6.89329622	6.858678	6.21796234	6.37521654	6.40185681
DNAJA2	3.2880687	3.36723244	3.95650668	3.79729579	4.18610759	3.67720232	3.7135145	3.64464004	3.94711874
DNAJA3	3.10294956	3.16092212	2.57300197	2.8888103	3.17276761	3.20144506	3.39283902	3.32188549	3.70019376
DNAJA4	2.38083215	2.85235184	3.33930357	3.58117644	1.41902791	3.58069794	2.73055877	3.27569061	3.15076692
DNAJB1	6.11693458	4.56500491	5.59162068	6.00439003	5.54467941	5.87962848	4.63434948	6.62440312	5.02878658
DNAJB11	4.52712326	3.06308364	4.35864483	3.83966347	4.08111144	3.4538768	4.49584567	4.62431589	3.50864365
DNAJB12	3.43208356	3.07437088	4.11747941	3.15400813	3.31717863	3.29910238	3.20153172	3.59587179	3.50308248
DNAJB14	1.33463483	2.07903427	1.94001506	2.4149678	2.36839819	2.31741211	1.73397604	2.3882923	2.09573627
DNAJB2	3.85048444	2.01821432	2.24282385	2.34444671	2.00127338	2.16814357	2.26063239	2.69898658	2.2600225
DNAJB5	1.09516163	3.04969793	1.52322216	1.91782943	0.99705735	2.6822166	0.96709474	1.07994515	0.59828817
DNAJB6	2.3160159	2.58412339	2.69565556	2.76358833	2.36486901	2.87846199	2.95773518	2.14971394	2.17226688
DNAJB9	2.7742647	3.62625601	3.95540777	3.73774707	3.52758677	3.56985973	3.19038974	4.42180753	3.48043377

DNAJC1	3.75753585	3.82162771	3.61929323	3.94129996	4.14576069	3.99939383	3.67219628	3.66878877	3.96352117
DNAJC10	2.76229699	2.73143851	3.43610003	3.25719526	3.09112761	3.25554343	3.64589623	3.7673132	2.65512021
DNAJC11	3.06613229	2.10888726	2.14930244	2.87031166	3.49973148	2.78084025	2.82634629	2.42213224	3.57272742
DNAJC12	0.37602052	1.66112834	1.34007368	4.22825212	1.02990517	1.12824452	0.47729009	1.27134335	0.40658616
DNAJC13	2.87380554	3.28625736	3.06774512	3.24727145	3.64404787	3.39140212	3.11445682	3.26853546	2.99528911
DNAJC14	2.85480128	2.10019363	2.35507102	2.20587609	2.07603207	2.49704911	2.29954077	2.74517763	2.36410804
DNAJC15	3.83476479	3.66275194	3.27406864	4.21856386	3.25774766	3.43710906	3.83240611	2.66152988	4.40656046
DNAJC16	1.70868536	1.92215413	1.71673836	2.32805573	2.44886829	2.07773966	1.85414309	1.50920595	2.80086451
DNAJC17	2.0366049	1.06810027	1.54963375	1.38480042	1.20226282	1.15852588	0.85216547	0.80736727	1.50385091
DNAJC18	0.46969292	1.67494965	1.18760389	1.30130657	0.91898175	1.36392932	0.64868359	1.17723432	0.95898374
DNAJC19	3.89029428	2.70219634	3.52157756	2.81461536	3.00234667	2.7501694	3.03234534	3.46614704	3.1121047
DNAJC2	2.37709404	2.15806618	2.84359807	2.66619221	2.93729571	2.36066048	2.36946198	2.5286431	2.6376566
DNAJC21	2.21964668	2.83781137	2.51095784	2.94354986	2.70075009	2.68519935	2.77096242	1.9805294	2.75470017
DNAJC22	3.133618	1.65294891	2.37037111	1.872323	2.51577704	1.82154024	1.6093206	3.39245919	2.94391239
DNAJC25	1.81169397	2.09725554	2.90687856	2.6278188	2.37530788	2.30164588	1.94182707	1.52544269	2.3842642
DNAJC27	0.46654942	1.00801542	1.02746774	1.26059146	1.00918091	1.03420904	0.63588712	0.79988411	1.07541529
DNAJC3	4.65094347	4.16689204	4.40905953	4.15413455	4.67588569	4.45663226	5.23399419	4.95636986	4.66286881
DNAJC30	2.58288123	2.66040936	2.90400922	2.53146486	1.99764487	2.36971065	2.02826157	2.08280966	2.63704464
DNAJC5	4.42773119	3.95826592	4.81964799	3.78534877	3.61124843	4.15671807	4.5295058	4.26885067	4.39106604
DNAJC5G	0	0.0264066	0.47333127	0.09016681	0	0.01135157	0	0.02371317	0.01621225
DNAJC7	2.99061351	2.62818646	2.90155763	2.70208558	3.20611458	2.69712079	2.52752078	2.91934234	2.84787102
DNAJC8	4.92263269	4.95708328	5.26606673	5.21111063	5.26231327	5.17657988	4.64368194	4.08605543	5.07446627
DNAJC9	3.03647881	1.60825752	2.59543897	2.25769631	2.55851583	2.57048394	2.56858513	2.26259496	2.52121218
DNAL1	1.21551155	1.15263612	1.38551193	1.28779848	1.11546487	1.07550212	0.95194215	1.05014477	0.98368002
DNAL4	4.19365841	2.05931837	2.76991493	2.06380349	1.84547769	1.85914016	1.65108681	2.02473105	2.39267611
DNALI1	1.41507148	1.30510597	1.33549301	2.08568998	0.25348618	1.24017064	0.40314836	0.32591625	0.15861709
DNASE1	0.6365614	1.69016821	0.81697694	0.76514222	0.87799811	0.55393857	0.48422722	0.78620402	2.33205385
DNASE1L1	3.13025538	2.49089228	2.46600001	2.23575148	2.79463407	1.7703859	2.69381389	2.74525632	2.20897129
DNASE1L3	0.12203864	0.98816351	2.56186723	0.63119578	3.56212733	0.21681481	0.25355978	0.79070401	2.53819175
DNASE2	6.1540601	4.17973419	5.42657737	4.40119645	5.47282326	4.79641179	5.01009703	4.76171242	4.37027071
DNHD1	0.18122186	0.30813433	0.31560329	0.45563786	0.40864203	0.40325591	0.14876985	0.24490572	0.42324755
DNM1	0.467228	2.22449265	1.15101175	2.18032477	0.4234184	1.4368153	1.26463704	0.90228514	1.04625381
DNM1L	2.78242182	3.35192484	3.31140491	3.6988983	4.20081272	3.80220002	3.28521341	3.3133659	3.66896248
DNM2	4.16863928	3.44761989	3.9340444	3.89778302	4.49681223	4.25185816	4.20943934	3.85130397	4.58970862
DNM3	0.17546716	0.423335	0.5905718	0.3420536	0.35019639	0.40448827	0.1976474	0.2118013	0.09012773
DNMT1	3.32229144	2.66241835	3.14947659	3.10453199	4.60197535	3.39889461	3.33340676	3.26562049	3.65010971
DNMT3A	2.2805444	1.47156389	1.8556565	1.4069544	2.08428151	1.67457316	1.1718616	1.47403672	1.49371264
DNMT3B	1.04920887	0.56380501	1.24115108	1.76944758	1.36347247	0.75326661	1.36962542	0.82860358	0.76290674
DNPEP	3.97036025	3.41107676	3.98573896	3.25423264	3.68968993	3.77952019	3.77649678	3.76916296	4.34544711
DNPH1	6.41093842	4.89840276	4.63559392	4.1052394	4.44979261	4.67808869	4.91720963	5.7844683	5.55676905
DNTTIP1	4.50247834	4.01242916	4.76852815	4.31576255	3.87882695	4.11852215	4.51371273	3.83261196	4.73820736
DNTTIP2	2.57830675	2.94141943	3.32162332	3.57445315	3.77957403	3.22187391	2.78968414	3.39809507	3.21423979
DOC2B	4.12941568	1.42388314	2.06398877	0.84921042	2.78832521	1.73698918	2.73020581	0.46520728	0.1890766
DOCK1	2.93701905	3.5145713	3.33349365	3.31007833	3.21353401	3.5151462	2.64696362	3.69592699	3.06070163
DOCK10	0.91518853	1.93336034	2.53270746	1.59339803	2.76587122	1.91931032	1.80661634	1.10201454	1.23710539
DOCK11	0.92191068	3.18090269	3.50942425	2.56764311	2.93298142	3.07063452	2.31552929	2.75158828	2.1670322
DOCK2	1.48899232	1.94114184	2.26224636	1.59847728	2.98754035	2.49610059	1.86376886	1.18827145	1.19453126
DOCK3	0.04103834	0.44392598	0.25815953	0.19150132	0.08465779	0.22866044	0.03447082	0.06739087	0.04735254
DOCK4	0.66894973	2.08016825	2.19737485	1.78635117	2.38055366	1.962085	1.82368688	1.14891796	1.066303
DOCK5	1.28709481	1.42320556	1.85089305	3.30532592	2.98730462	2.34836051	3.11032295	2.4732128	2.2649377
DOCK6	3.15586236	2.04173258	2.76185356	2.31562389	2.73346082	2.15910392	1.98014182	2.69339547	3.00876713
DOCK7	1.99787105	2.4171366	2.47111745	2.52715938	3.18908673	2.5503041	2.59447305	3.15148883	2.59989023
DOCK8	1.33511779	2.11186253	2.33711228	1.88812715	3.1875824	2.70954664	1.96856262	1.4021639	1.57513655
DOCK9	1.97091956	2.48324999	2.45242066	2.58508943	3.17142705	2.6690361	2.35908275	2.16546245	2.44364166
DOK2	2.90269949	2.69840414	3.42606773	1.90641577	3.26214964	2.77740496	2.77416711	1.35799086	1.92548084
DOK3	2.31378861	2.020931	2.37171958	2.54552312	2.76295497	3.22257229	1.83294971	1.38494533	1.55804155
DOK4	4.56698797	3.24673406	3.82821391	3.37868155	3.19455031	3.68459997	4.60242541	3.67902963	3.39781773
DOK7	0.20214148	0.29413325	0.38722447	0.35838274	0.02758339	0.99285008	0.23886788	1.6585215	0.46687774
DOLK	3.39568193	3.07887786	3.88383624	3.1731765	2.89257956	3.34193134	3.09898257	2.94117408	3.31220168

DOLPP1	3.78241216	2.58939404	3.82318049	2.8343979	2.50178326	2.72705762	2.75751242	2.27242384	0.91955446
DONSON	1.77540545	1.68156615	2.24978185	2.88965967	3.30156476	2.42635417	2.87335252	2.36273101	2.38670497
DOT1L	2.99008007	1.43618314	2.1835242	2.17891644	2.91640923	2.45251856	2.46352219	2.46739748	2.80265012
DPAGT1	3.20171087	3.61145398	2.98424677	3.69930963	3.51486426	3.44026214	3.88767349	3.28673992	4.19276208
DPCD	4.02351295	2.21889694	1.84907104	2.91735998	3.03746639	2.68595198	2.92961965	3.23422975	3.21738492
DPEP2	1.10801044	1.68767157	1.7743745	1.19880113	1.4326851	1.29830144	1.07793216	0.70403155	0.49177593
DPEP3	0	0.10952708	0.26179114	0.03214471	0.03299688	0.04571735	0.05306591	0.11372844	0.03748079
DPF1	0.50158269	0.23985456	0.48855094	0.254118	0.25644181	0.22432602	0.07094713	0.09895089	0.34293737
DPF2	3.30404405	2.64810357	2.77314106	2.65448618	2.74773202	2.92464259	2.96554182	2.74806299	2.82635354
DPH1	1.91411624	1.48523354	2.1022554	1.91437386	2.13060094	2.12697047	1.78578856	1.68428492	2.68055846
DPH2	3.52588509	2.528726	4.0049705	3.3646362	3.33605161	3.52452497	3.18467603	3.72305101	4.02114962
DPH3	3.11189629	3.52572782	3.48162899	3.46320041	3.92343245	3.58420693	3.61871436	2.94580046	3.40333595
DPH5	2.28394011	2.8464598	2.84805725	3.275798	3.50777902	3.17798419	2.38147369	2.87966333	3.27270344
DPH6	0.69272836	0.86813548	1.1253572	1.10316125	0.96043436	1.22642633	0.65188028	0.73978669	1.20261386
DPH7	1.80301176	1.36803015	2.59864157	2.03405086	2.19267011	1.90460877	1.65960214	1.62902899	2.44884686
DPM1	4.98274885	4.89936279	6.01273094	5.80828037	5.72261956	4.72581293	5.23585127	4.5692302	4.83804997
DPM2	4.87647192	3.32680569	4.66389785	3.63811819	3.67887117	3.45809117	3.79165523	2.89211508	3.973317
DPM3	5.55256451	4.55433049	4.13571154	3.90322724	3.53709661	3.96430857	5.03024846	3.47405302	4.6991763
DPP3	4.95095402	3.22610598	4.14974878	3.27348269	4.06744902	3.81235115	4.3015004	3.96378208	4.6374525
DPP4	2.20033144	3.94734422	3.17304683	4.31394766	1.53520593	2.89622747	3.63115309	3.26087697	4.93459703
DPP7	6.20873926	4.23305568	5.52190038	4.6464642	4.03193583	4.58438441	4.29922821	5.08980828	5.21377206
DPP8	1.8539908	2.66986114	2.89630806	2.75712238	2.72234499	2.84822773	2.30040363	2.27920938	2.65277843
DPP9	3.80371165	3.21191185	3.43128385	3.19285855	3.8405949	3.49694052	3.45641922	3.69533379	3.3728235
DPPA2	0.19676704	0.04458723	0.46748397	0.0466673	0.01614338	0	0	0.04006499	0
DPPA4	0.02888815	0.12787285	0.56418262	0.06000661	0.02229647	0.18584066	0.01304106	0.08462738	0.01904589
DPT	2.53830537	4.11345105	1.9978653	3.65732804	0.89734634	4.00826334	1.54694515	1.40993635	0.53215355
DPY19L2	0.02559169	0.4639392	0.28665565	0.52630479	0.11094309	0.38853367	0.05310651	0.04168599	0.10152516
DPY19L3	1.16017861	2.42638831	2.16801514	1.99389335	2.19803128	1.96747044	1.48882078	2.35715085	1.66904205
DPY19L4	1.62199476	2.59864183	2.82620706	3.1387055	2.45056852	2.61892737	3.11445113	2.67444451	2.40859168
DPY30	3.77318132	3.55955032	3.31167504	4.11857534	4.01281757	3.68955135	3.57046229	3.60181067	3.77385593
DPYD	2.71190335	3.63251064	2.40211366	2.73951745	3.51714321	3.73676312	2.5660089	1.7382253	1.46790928
DPYSL2	3.19030312	4.58567668	3.54733442	5.08209085	3.27029389	3.78079413	4.67672814	2.43447233	3.9507361
DPYSL3	2.8758025	6.32445117	3.51631803	5.00729805	2.30657095	5.97288191	3.46094614	3.16766122	2.17625993
DPYSL4	0.60877162	1.04053787	0.35791759	0.52986815	0.14769668	0.38610262	0.38899733	0.15897432	0.42976449
DQX1	0.54141413	1.67140594	0.77203582	2.2705481	0.25163907	1.48355118	1.26244825	2.66563567	2.78912275
DR1	3.12317412	2.61627842	2.96363301	3.08294913	3.3902055	3.28869049	3.06421014	3.2107692	2.98218349
DRAM1	3.50800332	3.98268654	5.65869319	4.23064526	4.39130643	4.46795564	3.72351485	5.28401524	2.84179235
DRAM2	3.83540695	4.38683204	4.36008167	4.24614338	4.29478018	3.81644426	4.15877735	3.66821096	4.19364873
DRAP1	5.82122259	5.04207734	4.66838852	4.20218667	4.67314155	4.81673238	4.84042949	4.44818846	4.50770627
DRC7	0.02107132	0.055651	0.24396456	0.03770681	0.02163054	0.05107769	0	0.01357081	0.02580125
DRD4	0.59750926	0.41477586	0.06960077	0.23197523	0.44173716	0.67536148	0.26875047	0.14466228	0.74352674
DRG1	5.08834372	4.82776675	5.05760381	4.95668915	5.31998982	4.78348626	4.73911202	4.0135144	5.0677914
DRG2	1.91243487	1.63721124	2.36444679	1.68564478	2.0632405	1.87239759	1.67605527	1.67477097	1.71958314
DRICH1	0.27908458	0.16639338	0.85838568	0.27444508	0.28113111	0.07025436	0.04941176	0.11404462	0.19254714
DROSHA	2.66847393	3.06535101	2.89744592	3.17750961	3.39168699	2.98062772	2.84260108	2.42461356	3.51969184
DRP2	0.10306673	0.18407909	0.20641355	0.21773586	0.07741372	0.26383689	0.04976178	0.05883413	0.05058084
DSC2	0.73789242	2.28542894	1.70688351	4.01595836	1.71344816	2.71805298	5.10011694	3.08499267	3.55889096
DSCAML1	0.62164586	0.22739354	0.14582713	0.15319137	0.02844064	0.03571952	0.0092734	0.24544048	0.03762804
DSCC1	1.85611297	1.55311808	2.4356718	2.81395329	2.95750302	2.34562921	2.65758812	1.9958871	2.35664895
DSE	1.00434145	2.42183049	1.98178212	2.2388923	2.07973452	2.47922079	2.26139617	0.97344371	0.94549658
DSEL	0.05256435	1.65732655	0.51493643	1.05859777	0.20395456	0.90576696	0.3624269	0.37661477	0.15425712
DSG2	3.37492547	5.37858726	4.86971809	6.08334097	5.75763519	5.11807386	7.64193173	6.55391917	7.05045617
DSG3	1.61235999	3.02723306	0.36444426	4.12815669	0.2664004	2.36818697	3.28014641	0.03516125	0.03087299
DSN1	2.61106069	2.39016586	3.29897076	3.08142876	3.24791219	2.90550606	3.18923656	2.71672671	3.21943367
DSP	4.06248193	4.82008008	4.43413674	5.97980283	5.35759783	4.42929745	5.37963765	6.71424044	6.63773162
DST	0.72725949	2.86750685	1.77484967	2.35551448	2.20331879	2.60015449	1.6901484	2.76386618	0.9438959
DSTN	6.05640811	7.63761861	6.6062002	7.24778085	6.00447716	6.98573735	6.5837704	6.08266324	6.82781923
DSTYK	1.24074543	2.18868861	1.65698641	2.13408577	1.84560255	2.21644472	2.04674091	2.16592248	1.22303849
DTD1	3.21513696	2.96633741	2.12454694	3.54379361	2.62534007	3.37331223	3.88663576	2.56115967	3.86489695

DTD2	1.95696298	2.51954343	2.53851644	2.32322819	2.64231138	2.20718043	1.95447207	2.01944939	2.96226078
DTL	2.35068453	2.11599978	2.46611408	2.70113065	3.6842963	2.8891025	3.64613638	3.06754917	3.0391351
DTNA	0.01720504	1.72137204	0.45701412	0.90559619	0.10452267	1.55917681	0.13349785	0.12148481	0.05357914
DTNB	1.75664447	1.20669398	1.72963841	1.18967939	1.72329581	1.14185841	1.08367571	2.39968057	1.72427202
DTNBP1	3.17849518	2.39438326	2.9743346	2.41823284	2.81397237	2.741367	2.45783958	2.91208805	2.76081714
DTWD1	0.61698143	1.72133682	1.29067413	1.90715146	1.3712446	1.518338	0.90919363	0.72870839	0.8635311
DTWD2	2.46022099	1.48326538	2.11118295	2.02941469	1.88089768	1.79773366	2.14917289	1.2491735	2.0091872
DTX1	0.37147412	1.97535091	1.3253745	0.94809485	1.20059078	0.93499338	0.46018549	1.29364534	0.52191189
DTX2	3.80235019	2.65287009	4.06603844	2.29062589	2.85618419	2.33521455	2.83335655	3.03204005	3.04305377
DTX3	3.53840753	2.32621796	1.71256166	2.4773285	1.57532938	2.06096024	1.0747067	1.5969047	0.49504933
DTX3L	4.46336387	4.80756348	4.08525248	3.87106078	5.7432611	5.19846116	4.63787037	3.88102416	5.18201591
DUOX1	0.49108239	0.54067701	1.2507203	0.61487304	0.22442101	1.49493653	1.60651972	3.55737862	0.24418514
DUOX2	0.0932751	2.43086649	2.98965034	1.3775116	0.68781804	0.17302315	5.10029098	4.49637939	5.06816004
DUOXA1	0.29962526	0.25632893	0.98261848	0.27229421	0.12145069	0.19179235	0.56118209	2.57176769	0.10903641
DUOXA2	0.1360738	0.6052414	2.52765038	0.77075995	0.48136631	0.16808048	4.97475595	3.83763735	2.90367009
DUS1L	5.08966985	3.02240264	4.02595899	3.4084443	3.5954325	3.4889105	3.92783927	3.9364005	4.55660979
DUS2	1.59024071	1.48992109	2.03332324	1.92447296	2.43259891	1.78709406	1.816623	1.52115207	1.96602381
DUS3L	3.4624243	1.74504463	2.69982715	2.38050835	3.07346919	2.02717897	2.45200823	3.12487914	3.58625155
DUS4L	1.16654845	1.06219191	1.74511471	1.5541642	1.35901585	1.44369481	1.2554089	1.13120648	1.25205453
DUSP1	5.34075452	7.19024839	6.82039341	7.32461705	5.49717968	7.99489638	6.34660328	7.20602133	5.31170095
DUSP10	2.04294956	2.42085168	2.38614447	3.27586895	3.86284155	2.96591077	3.20881441	2.25265061	1.92692115
DUSP11	2.60461277	3.00774214	3.47832026	3.44740058	3.43545013	3.21960531	3.20111197	3.10047404	3.16319393
DUSP12	2.7999525	2.36607627	3.26103549	3.32400615	3.50478872	2.89781396	3.83904994	3.4975447	2.95181492
DUSP15	0.1020529	0.32852937	0.17014202	0.38276452	0.10835223	0.15396682	0.11605372	0.06873604	0.79830483
DUSP16	2.9146619	3.7071679	4.16101489	3.57876798	3.57744876	3.80901832	3.05208253	4.62074485	4.34428045
DUSP18	2.27595699	2.0432024	1.72997292	1.53796956	1.58008665	1.41722411	1.04992391	1.86340945	2.63804268
DUSP2	1.98845627	1.54835019	2.28203011	2.52453356	2.73610371	4.44513106	2.1492804	2.18677556	2.51817636
DUSP22	2.50205889	2.82262883	2.7408126	2.28005013	2.13187721	2.81118256	2.31881522	2.78996652	2.87216863
DUSP23	5.06689545	4.91493702	4.74504141	4.35565439	2.87911171	4.83327839	5.03811237	5.52058935	3.99278695
DUSP26	0.11925718	0.65184831	0.40074191	0.28874562	0.09525711	0.41653849	0.01059268	0.03382695	0.05495406
DUSP28	0.64015435	0.78059363	1.03081265	1.11680954	0.84912064	0.72303166	0.80076526	0.75735814	0.99139088
DUSP3	5.12601755	5.10978618	4.46252517	4.50498495	4.3414737	4.96342592	4.23088567	5.37859923	4.03684336
DUSP4	1.655932	1.94534845	2.83467972	4.6125758	3.79867857	3.34504203	5.91407464	5.95826995	2.57851893
DUSP5	2.21172193	2.69310047	2.82920218	3.01685483	2.98788757	3.67065666	2.6197937	2.49644357	2.7457686
DUSP6	3.59218005	3.13037661	4.38063436	5.15213828	3.21387591	3.52206009	3.84157638	6.06368959	3.12385226
DUSP7	2.85408538	1.96067503	2.65669522	2.92775335	2.33008426	2.84412427	2.92645634	3.18757271	2.37363292
DUSP8	3.21329083	2.32370961	1.53496469	1.95952625	1.51482084	3.54114471	3.4906523	2.70625992	3.42302245
DUT	3.85907156	3.52602086	3.72465892	3.57772845	4.93324923	4.06797145	3.23148148	2.53125228	3.39898849
DVL1	4.34736279	2.74665239	2.55695352	3.62714764	3.40816256	3.70312071	3.88359386	3.64500059	4.14435873
DVL2	2.16044774	2.01436565	2.55034418	2.11862544	1.7215856	2.34398243	2.07896675	1.69611624	2.56843575
DVL3	5.785042	4.18000985	4.8316165	4.49469042	3.93086987	4.22714475	4.33301574	4.82216997	4.09610056
DXO	2.77609895	1.73596975	3.07320142	2.38848912	1.89047767	1.9456747	2.62823896	1.95245555	2.88777362
DYDC2	0	0.19755523	0.226866	0	0	0.02690915	0	0.01699478	0.01545993
DYM	2.083312	3.13738909	2.96090806	3.10973906	2.19386646	3.16787172	3.87779314	2.51987975	3.2443653
DYNC1H1	4.43843775	4.76570682	3.47200924	4.14424408	4.91140572	5.03662377	4.88888314	5.25378478	4.81996661
DYNC1I1	0.41338716	1.06327955	0.90379458	0.43476595	0.23112195	1.00270957	0.30841604	0.15716946	0.09729316
DYNC1I2	4.38450194	4.45278623	4.50403906	4.64390809	4.53367532	4.64670801	4.36989041	4.72527381	4.51310339
DYNC1LI2	2.68799656	3.77122257	3.3411515	3.71875714	3.42265782	3.88164809	3.45962077	3.54926418	3.61444427
DYNC2H1	0.05752133	1.14704844	0.57592805	0.99427098	0.17308989	0.76539355	0.25803766	0.42750277	0.14289166
DYNLL1	6.12791517	6.18754148	5.56872848	6.52166491	6.29222029	6.18236342	6.27781643	6.04360716	6.12690555
DYNLL2	4.33014751	3.13358053	3.08222877	3.05206675	3.23927789	3.15976935	3.08327371	3.87812563	3.28562656
DYNLRB1	6.18730619	5.41291264	5.94897714	5.84708484	5.43723547	5.2432844	5.05117076	5.31293894	5.99896538
DYNLRB2	0	0.18697367	0.15341177	0.18215987	0.0303502	0.07137804	0.01482532	0.03787712	0.03873121
DYNLT1	4.6395445	4.82052497	5.08529428	4.74108699	5.24484677	4.79702588	5.08582203	4.29286427	4.96137631
DYNLT3	3.23460023	3.95688663	4.36755534	4.03883818	3.81065859	3.50991018	3.68866259	3.30371963	3.08991844
DYRK1A	1.87728858	2.81989636	2.71865542	3.28928459	3.04441545	3.28500959	2.86029596	3.20306539	3.19490374
DYRK1B	3.30560646	2.57623784	2.67491182	2.31307345	1.92912339	2.56772512	1.10195487	2.43172923	2.55979488
DYRK2	3.99726646	2.81089168	3.37640631	3.36329159	3.0589508	2.83860427	2.82204935	3.38234706	3.10035957
DYRK4	1.73610931	1.94381186	1.93557457	2.05154126	1.9736429	1.89674793	1.72630315	1.36682294	1.90696624

DYSF	2.25898536	3.21179985	2.68235811	2.15910527	2.86794973	3.70069223	2.47423892	1.75409156	1.49048836
DZANK1	0.45840466	0.34356931	0.53275014	0.55297785	0.27691852	0.35191132	0.17660696	0.26132782	0.37730333
DZIP1	0.36324893	2.21375999	0.93629631	1.3593489	0.31541196	1.50640104	0.71137665	0.51593203	0.23337696
DZIP1L	3.31295454	0.56740098	2.7240462	1.24664797	0.6570354	1.11776494	0.87913191	1.57908451	0.18506045
E2F1	3.83508934	1.86927424	3.15799226	2.49368606	2.96152553	2.60237975	2.94372555	2.64595931	3.3540929
E2F2	2.14286226	0.80331396	0.94089778	1.25788236	2.36918482	1.44789616	1.81966875	0.63349375	2.0339174
E2F3	2.20176917	2.12236714	3.32775898	2.69195431	2.9092787	2.82350041	2.61184208	2.8142185	2.54261496
E2F4	5.07928417	3.6633945	5.21052264	4.1349694	4.5404216	4.33671768	4.36404729	4.11954015	5.0904389
E2F5	1.33905259	1.09261061	1.76220578	2.37156865	2.62197817	2.17525662	1.98050251	1.63105431	2.15510885
E2F6	1.97061561	1.73415771	1.84189127	1.92548512	2.30934667	2.10589049	1.72476732	2.04577455	2.1730658
E2F7	1.37358242	0.666941	1.61156855	1.84386587	2.23374991	1.51892314	1.64857574	1.41842295	1.46639417
E2F8	2.07367671	1.04441038	2.05533126	1.83589009	4.41294618	2.05075705	2.55758052	3.81080967	2.41498863
E4F1	2.41056545	1.39856643	1.39071662	1.6713315	1.44673529	1.72347549	1.94168506	1.86701452	2.12160349
EAF2	1.61126258	1.39781156	2.49569334	1.51937112	2.78467565	2.95106317	1.46016417	1.05911465	1.70070295
EARS2	2.47928422	2.77580118	2.44867471	2.33916368	2.71179749	2.63220137	3.15658768	2.71550945	2.87948001
EBAG9	2.7314368	3.30337047	3.67024922	3.65329699	3.01913841	3.49669477	3.59742634	3.3224611	3.09776526
EBF1	0.50291717	3.31257159	1.88409612	2.28716902	1.26790427	1.89328782	1.06802697	0.69822726	0.52236913
EBF3	0.19165523	1.99267388	0.72367698	1.24709988	0.33636325	0.74381384	0.45377838	0.20632219	0.13442749
EBI3	2.06599503	1.44584096	2.29954785	1.19320414	2.33921191	1.26446425	1.73122306	1.4158805	1.4527924
EBLN2	0.61032148	1.01217014	1.32821747	0.77662057	0.95318984	1.03975102	0.8988804	0.81053217	0.55248133
EBNA1BP2	4.17197477	3.67672127	4.4746614	4.48018261	4.54233098	4.49592132	4.16060693	4.37382115	4.61247267
EBP	5.18011826	3.42609091	3.97315756	3.776344	3.23534027	3.49120727	4.25054053	3.99036799	4.48563566
ECD	3.21254441	3.5963101	3.98595947	3.73458545	4.07281149	3.65336421	3.21104246	3.77963769	3.68678511
ECE1	4.34427925	4.38922965	3.98496654	4.37588352	3.67767888	4.54512946	4.01874426	3.52140847	4.04368456
ECH1	5.9352209	5.74481168	5.56455191	4.1227942	5.44894198	5.57039491	5.53897788	5.25732831	5.21188444
ECHDC1	3.98932816	3.50838885	3.56267308	3.63850089	4.16650555	3.57586736	3.66942765	2.77589836	3.59345806
ECHDC2	0.17757373	1.57200034	1.87953214	1.5224973	1.6456362	1.49934851	1.49852782	2.06885481	2.13635714
ECHDC3	0.53694385	2.28120698	1.01961759	0.36108351	1.13524151	1.23578228	0.40629148	0.50473673	0.36246929
ECHS1	6.374276	5.90469229	6.12663453	5.76222482	5.64847587	5.89308078	6.71284594	6.08755889	6.38391646
ECI1	4.67618377	3.66373751	2.86638394	2.73069976	2.97755172	3.25981012	3.98135068	3.88082973	3.91369738
ECI2	3.32482119	3.365463	4.80546971	3.15122896	3.39965536	3.41944405	2.91875324	2.42137344	1.77090971
ECM1	2.90754254	5.15165021	3.84364654	7.39165216	2.63795599	4.67023002	4.39234498	5.62612303	3.03361124
ECM2	0.48096605	2.74383276	2.08819181	3.09699993	1.67669991	2.36878863	1.46440438	0.70295309	0.93580997
ECSIT	5.00749979	3.23376363	3.7604474	3.13149223	4.01334018	4.09857218	3.3595921	4.37518508	3.37481521
EDAR	0.01637529	0.22247559	2.28938194	2.41922881	2.53330061	0.59416003	0.28933658	2.38748359	0.36178038
EDC3	2.91445638	2.29245845	3.08134935	2.78688731	2.8806071	2.34733567	2.47291858	2.18972321	2.8600976
EDC4	3.44940419	2.32230667	2.85976422	2.6544021	3.06287536	3.02460247	2.96125968	3.0475335	3.1812267
EDEM1	2.69931383	2.82479781	3.15368344	3.81847872	3.76294246	3.69001256	3.80024643	3.44900419	3.22081459
EDEM2	4.36557814	3.97094732	4.97336151	4.12001794	3.93060116	4.22441424	4.46100282	4.47267363	4.47392144
EDEM3	2.3165911	3.16974219	4.18961648	4.06894616	3.82733777	3.51273214	5.35748218	5.44521505	3.28224561
EDF1	7.78823321	6.51926202	6.4906702	6.31393793	6.51377726	6.24462481	6.48868951	6.55991152	6.73227772
EDIL3	1.2762093	2.322517	3.90976352	3.05367779	2.02988192	2.18452051	3.20761188	2.61856129	1.83632915
EDN1	1.3023182	3.51259487	3.0943787	3.90118138	2.70637662	3.80244341	3.0434819	3.08824667	3.37834512
EDNRA	1.48857251	4.34905959	3.18456227	3.7234635	2.28076811	3.17386317	2.74993734	1.87470972	1.86444849
EDNRB	0.87683903	3.73144164	3.14173723	2.07179095	2.20466892	3.00818667	1.92782222	1.64625251	0.89172708
EDRF1	1.09467636	1.1115697	1.74339795	1.82428819	2.1569971	1.4098343	1.23616106	1.78612423	1.48423269
EEA1	1.50424598	3.12403435	2.35648978	2.66881614	3.15913631	2.7504067	2.23151789	2.56489185	2.82813768
EED	1.74776758	1.41267575	2.0146788	2.04919875	2.42006392	1.80094206	2.11220746	1.71920234	1.70393114
EEF1A1	9.23261698	9.80368717	9.01504156	9.99519047	9.73891787	10.1133104	10.4345093	9.35923585	10.5052333
EEF1A2	0.07090082	0.92177888	0.77369761	3.95617469	0.16880394	0.34822343	0.32918326	1.48828265	0.1190933
EEF1B2	6.96776336	7.17527761	7.20364542	7.82836571	7.46445941	7.53399659	8.39621778	7.62969042	8.56680754
EEF1D	3.64675674	3.00179504	3.42093378	3.25471075	2.78661512	3.32705516	3.71032709	3.67711126	3.63840778
EEF1E1	2.374152	2.24364993	3.04439699	3.75044491	4.038205	3.21439725	3.48152188	3.13904833	3.18457876
EEF1G	0.52321123	0.40016498	0.54293942	0.67039147	0.38259298	0.44598853	0.88462696	0.47213472	0.47116693
EEF2	9.74758059	8.51957189	8.37991356	8.95662555	8.82360982	8.96565344	9.07591383	9.37817031	9.91404776
EEF2K	2.85721153	2.46408814	2.47459397	2.88396014	2.98895864	2.58570943	3.08114548	2.83650922	1.85636055
EEF2KMT	1.81545717	1.91491969	1.44115056	1.9874255	1.50124072	2.12005996	2.30591842	2.1051825	2.04119483
EEFSEC	4.50484833	2.86189335	2.95922961	3.10010757	3.00927078	3.4007566	3.41450379	3.48838479	3.33380879
EEPD1	2.50329696	2.42104681	2.39157853	2.08812108	1.6201731	2.05313976	2.09514855	2.35555756	2.55693967

EFCAB11	1.02903156	0.90551496	0.87087766	0.85933824	1.27330205	0.87941291	0.75886368	1.24405117	1.09031163
EFCAB13	0.37752595	0.45050496	0.89446309	0.34260623	0.81398137	0.67769693	0.08568896	0.71809223	0.36354824
EFCAB14	4.88133167	5.37037495	5.92934315	5.17136072	5.51181042	5.28002786	5.20555581	5.1211116	5.54431931
EFCAB2	0.46322241	0.56639294	0.56483639	0.88379559	0.64034592	0.5346708	0.52705739	0.88716857	0.55785928
EFCAB3	0	0.01077605	0.44290529	0	0.01158634	0.0274892	0	0.03593881	0
EFCAB5	0.08676676	0.18895392	0.3252663	0.03430581	0.04780972	0.05312909	0.04342562	0.06927291	0.10142473
EFCAB6	0.17241497	0.19375285	0.34823517	0.11299883	0.20282414	0.09829172	0.03525877	0.13169129	0.04626359
EFCAB7	0.69206959	1.05880982	1.04647278	0.91034079	1.25458473	0.8908721	0.46650383	0.83883479	0.95641561
EFCC1	0.22842677	0.73830868	1.34658425	0.78281522	0.15866662	0.90293546	1.05540103	1.11572706	0.477437
EFEMP1	2.60899895	7.41412873	5.28056235	5.60192951	2.85313969	6.12888006	3.71180277	3.09413808	2.20461852
EFEMP2	2.76639683	4.33815038	2.71801827	3.33005354	1.45807137	3.65712448	3.17431419	2.14006517	1.50606385
EFHC1	1.18703429	1.08220153	1.57160561	1.59195758	0.78358493	0.9353558	0.67964697	1.28667766	1.55183096
EFHC2	0.25130236	1.05439648	0.52597556	0.37497451	0.04659163	0.42950508	0.17321931	0.08624575	0.11105143
EFHD1	0.59758407	1.41879134	2.07627975	1.60218002	0.9317827	1.35964258	0.76856183	0.80182697	0.55996368
EFHD2	7.38312441	5.15911099	6.1362621	5.47196387	6.32078765	5.98685978	5.56004228	5.49210727	6.64232037
EFNA1	4.20332135	3.40094862	5.41804802	4.68608153	3.18244268	4.82554813	5.25640106	6.5978363	5.22937891
EFNA2	3.81182659	2.14255809	2.39763327	1.97766624	0.19860456	1.64264459	3.43504656	1.06886217	3.7286907
EFNA3	2.76750013	1.75056615	1.15916444	3.3166303	0.78704362	2.97371178	3.81943515	1.6540705	2.00426171
EFNA4	4.06013988	3.176745	3.41901341	3.24366178	3.08603599	2.83816021	3.56212753	2.87210314	4.32480158
EFNA5	2.6395328	1.11071029	1.88789709	1.38870643	0.37566627	1.4634752	1.80095438	0.34647541	0.10054499
EFNB1	5.20208774	4.93191281	2.82932402	4.51217704	4.56996407	4.00411496	4.22531189	4.8221024	4.82100602
EFNB2	2.60530202	3.81174703	3.85357715	4.32890306	4.54303884	3.34615922	5.46761281	3.976852	3.50418473
EFNB3	0.1604114	0.84028986	0.6264678	0.53761699	0.06805188	0.2121839	0.38930012	0.26599287	0.06963944
EFR3A	3.15614696	4.46492636	4.3554489	4.7194615	4.91698114	4.77999825	4.50333095	4.43965961	4.11190007
EFS	1.049042	2.48767385	1.14179937	1.66135011	0.51387427	1.91671746	1.19493709	0.71428453	0.37334333
EFTUD2	3.94461072	3.06081307	3.40543123	3.57349017	3.86852266	3.74805076	3.48689657	3.57562267	4.02923619
EGFL6	1.82719271	2.1527667	2.29670742	2.60707371	2.40219112	1.11621964	1.95926278	1.18730426	2.04699465
EGFL7	2.60783502	2.60905008	3.22160475	3.07596257	1.75066248	3.13664468	2.16058754	1.40464011	2.42324272
EGFLAM	0.48708518	1.29932984	1.32477855	0.99104439	0.92180167	1.12035156	0.93265714	0.45860675	0.98642748
EGFR	2.48335853	3.26434071	2.46063111	2.8049269	2.25150805	2.9098363	3.49628439	2.75322198	3.2731041
EGLN1	3.26915223	3.57827866	2.87392588	3.52978155	3.46690543	3.68050325	4.09558178	4.48274279	3.54567576
EGLN3	2.08190722	2.23764877	1.76582407	1.8435018	1.22043313	1.13135542	2.48882126	1.22012644	3.34059197
EGR1	5.2503328	5.2832108	5.4395803	6.1620258	3.79176955	7.72446179	4.92829957	5.27592787	5.02234159
EGR3	0.5178993	1.94458548	1.32620154	2.62370594	0.98845857	4.19194374	1.18912064	2.65267525	1.41299244
EHBP1	1.75788586	3.64302652	2.80045425	2.94955412	2.38628325	3.0255308	2.35650671	3.19994859	2.35834604
EHD1	3.92242242	3.11025591	3.34044471	3.25003127	3.88198903	3.99092347	3.81902083	3.7097029	3.6640224
EHD2	3.74993948	6.11369758	4.31179944	5.11541679	3.11967181	5.658172	4.4737792	4.83384929	3.0938828
EHD4	3.60074057	3.2550676	3.75627641	3.35410039	3.63853434	3.84505707	3.35279605	2.75644634	3.24495441
EHHADH	2.10798847	2.33673152	2.96119441	2.26165955	2.89533163	2.70436127	2.67562573	2.55342351	1.34948784
EHMT1	2.24824177	1.78943672	2.23653545	2.04633093	2.10031897	2.12821294	1.87707909	2.46842573	2.39025529
EHMT2	3.85822903	2.86807885	3.71904779	3.18336951	3.05565573	2.83005091	3.81412783	3.85590748	3.83981619
EI24	5.88489147	4.87928526	4.8750638	5.35118368	5.9167299	5.44910063	6.03415729	4.80668974	5.50390063
EID1	4.40333654	6.55733399	5.66393733	6.42891345	5.18282498	5.69990839	4.89768083	4.97559478	3.32396441
EID2	2.7524066	2.59977115	2.46558406	2.94951831	2.97850134	2.50129883	1.95523889	2.21929798	2.47630222
EID3	0.25072882	0.8484682	0.97078285	1.24906255	0.84579091	0.65712608	0.3548876	0.37770533	0.33688934
EIF1	7.59587398	6.53803412	7.03154843	7.24584795	7.09143043	7.02212798	6.34311648	7.05272909	6.74769024
EIF1B	4.32281891	4.4889707	4.20022303	4.68590287	4.41163648	4.19463942	3.87679241	4.42738151	4.40757849
EIF2A	4.83936957	4.80695529	4.7687359	5.65400663	5.46469583	4.96066103	5.12804966	5.10076052	5.95558902
EIF2AK1	5.21897031	4.65176003	5.77415277	5.00073801	4.90968034	4.56156896	5.30991296	5.23843715	5.18689977
EIF2AK2	2.55798231	4.59375465	3.13007121	3.67854751	4.36245304	4.3622829	3.0898027	3.02638765	3.40966307
EIF2AK4	1.66148478	2.56395165	2.65103831	2.76988175	2.83182922	2.896326	2.60772281	1.90306097	2.34578965
EIF2B1	3.93864162	3.60011442	3.86153505	3.83504983	4.15190582	3.72012437	3.69755622	4.3211737	4.13140993
EIF2B2	3.42381305	2.91433812	3.08061889	3.27542221	3.3360686	2.87445381	2.7668165	3.03836046	3.22660512
EIF2B4	2.14237972	2.39028863	2.59050898	2.50787219	2.18275614	2.617537	2.57472026	2.6808498	2.70525415
EIF2B5	3.68331303	2.36549152	3.71204372	3.01336418	3.20314001	2.89057436	3.15729495	3.55137543	3.48549819
EIF2D	2.95901539	2.0615205	2.34371097	2.10065608	2.58269898	2.45682797	2.99427859	1.9927073	3.12723011
EIF2S1	3.21882868	3.8859307	3.97755007	4.15510681	4.57769709	4.17988014	3.94027396	3.80909123	4.20402538
EIF2S2	5.2807174	5.09592923	5.6549308	5.78864028	6.23506241	5.6928738	5.81274026	5.43518501	5.97299156
EIF2S3	5.28061329	5.21220093	5.39126317	6.03053437	6.95306552	5.42546952	5.79675613	5.5555655	7.30536432

EIF3A	4.91136262	4.74939769	4.79629189	5.02561447	5.44166955	5.2387226	5.14300413	5.52064654	5.48656363
EIF3B	4.96840267	3.7267683	4.3747262	4.03400605	4.17155188	4.24951166	4.3117512	4.03284918	4.50070227
EIF3D	5.99015299	5.49047951	5.60020894	6.24498017	6.16788757	5.83405744	5.7614417	5.3014389	6.39083875
EIF3E	4.96738167	5.70644492	5.81223037	6.82374885	6.0820557	6.19981441	6.38146431	5.69720885	6.4292677
EIF3F	4.76126385	4.08061403	3.52713844	4.25666067	3.79375845	4.47020277	4.69995107	4.21446023	4.64438929
EIF3G	5.8896858	4.8103221	4.89386209	4.46208637	5.08367999	4.63946716	4.90201193	5.52420669	5.27545034
EIF3H	5.34844011	5.23756255	5.56719048	6.17077564	5.69296041	5.8811959	6.01212835	5.61299702	5.94460279
EIF3I	7.2547148	6.55177742	6.84854418	6.85492363	7.09528599	6.81962347	6.93250827	6.19913158	7.17830491
EIF3J	4.61462396	4.04223357	4.3317079	4.64784432	4.77044655	4.57898367	3.86580696	3.96262686	3.98636327
EIF3K	6.15300307	5.48217941	5.32426034	5.40137062	6.27703656	5.59793898	5.54068446	5.7313682	5.82709734
EIF3L	5.15426655	5.52850177	5.66115081	6.14221775	5.30325385	5.92701848	5.85479975	5.35617258	6.61222953
EIF3M	3.95083555	4.17400804	4.19394789	5.03271671	5.28759687	4.53805189	4.47728614	4.36896388	4.75847311
EIF4A1	1.2469145	1.1034393	1.76398108	2.13884514	2.14476006	1.8708225	1.44864085	1.26370398	2.298396
EIF4A2	5.68588793	5.50898115	5.83519219	6.58341123	6.00799376	5.30498104	5.06235491	6.20470532	6.21842306
EIF4A3	4.83157805	3.7032901	4.43310908	4.33205913	4.58573933	4.46738921	4.4646563	4.98224978	4.46409946
EIF4B	5.241697	5.49217456	5.38352163	6.39899308	5.61235488	5.88277822	5.79578512	6.81730322	6.28896549
EIF4E2	3.87385332	2.88895088	3.47156543	3.40512201	3.2115757	3.07527385	3.30986755	3.33125363	3.22691857
EIF4E3	0.59093133	2.38679687	1.61141027	1.65271161	1.57292216	2.79543624	1.34265206	1.45211511	2.28664727
EIF4EBP1	6.99271972	5.25777632	4.82616475	5.56380202	4.58686359	6.10435233	6.80858215	5.39871801	5.98855594
EIF4EBP2	4.35268957	5.17412607	5.36177609	4.80969666	4.5886533	4.95373458	4.81042522	5.32039634	5.10265856
EIF4ENIF1	2.92872771	2.32683507	2.64168685	2.42342355	2.65006721	2.36232821	2.08020454	2.01056962	2.52116392
EIF4G1	6.42780657	5.0334534	5.50766832	5.25225961	5.68797193	5.59770891	5.98843418	6.20910417	5.72172258
EIF4G2	5.88153165	6.8183308	6.59623628	7.43665602	7.21616437	6.99649554	7.41162112	6.92053613	7.1055965
EIF4G3	2.98279592	3.58428945	2.80826725	3.45185081	3.73576234	3.46107323	3.20268532	2.86346104	3.09411149
EIF4H	6.56746677	5.57391262	5.33509382	5.24807894	5.77655231	5.82592008	6.02244599	6.15761096	5.92661782
EIF5	4.42489981	4.58672427	4.69228691	5.14922098	5.31079882	4.88775695	4.84321303	5.56197288	5.08992504
EIF5A	6.91554147	5.85620399	6.50648806	7.01506268	6.84805611	7.41243799	7.24938553	5.80390083	7.85858885
EIF5B	3.89458857	4.52904331	3.8347467	4.69389867	4.8830766	4.47827458	4.43682657	4.36015108	3.56573164
EIF6	6.42130245	6.34039798	6.48146843	6.72538327	6.30970249	6.24225511	6.41152909	6.1977325	7.26517869
ELAC1	0.74372552	1.51911001	1.69341299	1.78697519	1.64006025	1.34258008	1.34849461	0.96281707	1.6784412
ELAC2	3.00916562	2.87440343	3.47646135	3.70023257	3.83432138	3.86833388	3.47777988	2.88670121	3.95853699
ELANE	0	2.2026987	0.40006101	0.51354139	0	10.3168011	0.04183154	0.07973931	0
ELAVL1	3.7689941	3.18919238	3.52339934	3.53742918	4.23002412	3.7854621	3.50701019	3.53270609	3.9876252
ELF1	4.49455676	5.00641682	4.7350983	4.62340953	5.0854581	4.84920369	4.72202908	5.07515578	5.56141211
ELF2	1.98011072	2.38571236	2.45964912	2.24739769	2.43091532	2.1917137	2.03075733	2.60751559	2.28447309
ELF3	6.50409223	4.22244212	6.29685537	3.97835937	5.19104204	4.56871731	6.38679306	6.65582878	6.11012398
ELF4	5.26943219	3.5100895	5.04928183	3.63495573	4.79577341	4.41678309	3.96118284	4.48232464	3.52399195
ELK1	4.00556839	3.76784153	3.6412934	3.29607053	3.58175013	3.99174194	3.79435963	3.58323633	3.57746061
ELK3	2.6514047	4.85221974	4.75098473	4.76016526	3.84053722	5.01621925	3.636417	4.47077575	3.40389905
ELK4	2.12020699	2.97335291	3.34609303	2.88623685	3.57174289	3.381402	2.60526317	3.28612328	1.95422464
ELL	2.97028526	2.19489855	2.4764949	2.17556402	2.64107318	2.66458103	2.21844078	2.39202085	2.36571408
ELL3	0.12129969	0.16424875	0.56098591	0.55936682	0.23009799	0.52113228	0.77301319	0.23777937	1.08431831
ELMO1	1.12603186	2.33474379	2.22022564	1.76186907	2.17080335	2.35915832	1.46728045	1.13249434	0.92215663
ELMO2	2.71521711	2.9145149	3.52876579	3.02040507	2.98511692	2.87803315	2.8261546	2.35198764	2.67524511
ELMO3	3.34245122	2.70696464	4.16677092	3.33956179	2.79260319	3.5343828	3.6929874	4.91564714	4.40485671
ELMOD2	1.20632767	2.39624902	2.3460081	2.57874882	2.45061854	2.57756278	2.22317962	2.44980459	2.46894027
ELMOD3	1.33983542	1.4245994	1.7892625	1.40795105	1.74124879	1.29728684	0.90523775	1.2469998	1.53997959
ELMSAN1	2.59892246	2.38123684	2.20639547	2.40119663	2.63023899	2.57304078	1.86923298	2.0047499	2.25656468
ELN	2.11131928	4.65419994	3.45557289	3.20833821	0.60949188	3.3427718	1.52569172	2.46736917	0.77262558
ELOF1	4.02550462	2.41906648	2.99482731	2.78622077	3.30877834	2.88648654	2.49990711	3.1182217	2.99028558
ELOVL1	5.64739555	4.74104645	5.91273601	5.46105595	5.08637952	5.61832171	5.08465238	5.52709386	5.18048835
ELOVL4	0.10398611	1.29044655	0.46881409	0.78443861	0.35768704	0.75925888	0.34869202	0.18196402	0.08557528
ELOVL5	2.23980681	4.73624817	3.88698679	5.44750266	4.06503928	5.35512755	3.29281682	2.56448156	2.45100149
ELOVL6	1.83261874	2.15586389	1.91694971	3.27559756	2.47445704	2.20115304	3.14635265	2.40683861	3.40685002
ELOVL7	2.07482058	2.05841632	3.19788028	2.91481081	3.9612591	2.90588558	2.18559142	2.51313876	4.3850537
ELP2	1.20081302	2.42330285	2.82402394	2.58483743	2.40630522	2.58491201	2.78392464	1.92501308	2.7035243
ELP3	2.68210425	2.80625564	1.94904386	3.17258464	2.96604769	3.07680506	3.29168254	2.45079403	2.88517763
ELP4	3.44532168	3.2807263	3.3216159	3.39802206	3.31757858	3.4551913	3.33886169	2.76192906	3.16714237
ELP6	2.86755539	2.19580095	2.39308389	2.40355396	2.16373546	2.46660101	2.67902618	2.12791286	2.60932523

EMC1	2.97797207	2.36630455	2.49804664	2.84893331	2.69364523	2.89355158	3.22830526	2.31972447	2.84156184
EMC10	4.01601995	2.60842783	3.91572308	3.31857586	2.57755793	3.25867058	3.46631421	3.72616348	3.67510843
EMC4	3.25449173	3.92882052	4.30437738	4.18252509	4.28366558	3.60151737	3.88480081	3.42355383	4.00613845
EMC6	3.10789237	2.17590318	2.60942032	2.31647522	2.27651018	2.96484345	2.54532786	2.05840991	2.90544295
EMC7	5.24689378	4.98508577	5.67760191	5.54821881	5.53988195	5.29237942	5.15576643	4.72857762	4.85275836
EMC8	2.88174333	2.63810575	3.30884501	3.12296477	3.09541929	3.19896128	3.39947242	2.98069608	3.37690212
EMC9	3.0027705	1.93878954	2.2186959	2.20999295	2.07196257	2.3530346	2.75115891	2.083139	2.92551349
EMCN	0.75204779	3.33538361	2.96637634	2.41498842	2.06621168	2.86169027	1.0715987	1.00602025	0.39956428
EMD	6.08227634	4.55678361	4.32976662	4.4251231	4.94194621	4.26444949	4.13168762	4.30552126	4.63814905
EME1	1.23250479	0.51651402	0.98599651	1.46822341	1.61569012	1.07352231	0.97719996	0.77098429	1.29586005
EME2	1.29370654	0.38952753	0.79220248	0.84326078	1.30106403	0.48716153	1.00175208	0.69604829	1.44366627
EMG1	1.5175539	1.56617547	1.8741662	1.78403956	1.65195743	1.72487528	1.47202655	1.72332088	1.90253301
EMID1	0.25191989	1.28943943	2.05239727	0.4158851	0.10599869	1.41433507	1.64323731	0.82141964	0.45557765
EMILIN1	4.92860673	6.19347472	3.9366643	4.44854391	2.58054854	5.76028157	4.70477352	3.60210582	2.60389191
EMILIN2	2.12185325	2.68844109	3.31688092	2.49504291	2.84426415	3.3389579	3.53670511	1.86822624	2.1934402
EML1	0.33799701	3.62980403	1.57288095	2.14009452	0.78575249	2.16232169	1.53171281	1.33325243	0.84542941
EML3	3.55558168	2.66805191	3.08289352	2.64019835	2.61071607	3.24725503	2.66404666	2.90411654	2.73335725
EML4	3.36601322	3.80823355	4.27873279	4.03600845	4.50640034	3.8452157	3.98545824	3.8757882	4.73708094
EMP1	2.84172864	4.76475838	3.96515697	5.45874752	3.98129815	4.79323573	3.58320695	3.45857954	2.8806468
EMP3	4.44367189	5.28643557	4.19621283	5.23627153	3.92682157	4.67676494	4.28244312	3.25737249	3.25263625
ENAH	2.9278033	3.5698999	2.8758884	3.55718364	2.56848998	3.44517399	3.55734037	1.33028979	2.88402484
ENDOG	3.85299035	1.55501729	1.90930555	1.88858487	1.67398161	2.41895663	2.62618953	2.22401165	3.02864327
ENDOV	1.7279938	0.6928392	1.0666647	0.92170404	1.06201023	0.64910115	0.75533082	1.40149148	0.76187128
ENG	4.54358723	5.95234807	5.84491944	4.67263275	4.53128184	5.72853295	5.15697653	4.1670206	3.92565313
ENGASE	2.34250674	1.20370105	1.86283688	1.63645613	1.50734944	1.27195672	1.43691886	2.38512115	3.96260881
ENHO	0.27515636	0.19799881	1.1469551	0.75391119	0.02060235	0.09575766	0.17092774	0.46250963	0
ENKD1	3.22130158	1.67730512	1.21513276	1.31162712	1.59250418	1.65446646	2.08789558	2.03693148	1.73318282
ENO1	8.95268311	6.89902404	6.87650263	7.57523149	8.08585481	8.16145209	7.88769886	6.97565586	8.32811309
ENO2	3.20660348	1.80227542	1.81013255	3.39065876	1.02576137	2.27944779	1.62483195	1.5081622	2.20586588
ENO3	0.65160939	0.32909324	0.90435721	0.73759762	0.55715573	0.46756544	0.33827517	0.24059447	0.81898418
ENOPH1	5.01400089	3.90052555	4.47702189	4.77518318	4.89965142	4.46645368	4.59659239	5.05184574	5.15551159
ENOSF1	1.90536875	2.021962	2.5044692	2.08286561	2.36909615	1.74648698	1.97246021	2.34727301	2.41691485
ENPP2	0.71871453	3.50431728	3.41096189	3.02819747	3.49895227	3.54038503	1.94888582	1.79369199	2.39571554
ENPP4	1.51478354	2.52996889	3.623944	2.94297965	3.63963122	2.99904835	3.34421386	3.92654631	2.92938145
ENPP5	0.08303522	1.11594173	1.24727989	1.3239502	0.44500642	0.92027353	0.18239088	1.66308767	0.73405025
ENSA	5.11918514	4.05402494	4.50563907	4.23196117	3.89662148	3.98312423	4.7611255	4.04738424	4.29075567
ENTPD1	1.53508224	3.0285781	3.35946105	3.10002237	2.7501738	2.98000205	2.76005433	2.01756499	2.14849144
ENTPD3	0.14417794	1.45950876	0.67989847	1.34247474	0.16381267	0.78002609	0.92164712	5.14331349	0.01134123
ENTPD4	3.01320648	2.80561571	2.7407004	3.64607822	3.50852394	3.23770439	3.67656356	3.33760254	3.04409435
ENTPD5	1.18301615	2.42659719	3.2229078	2.07018439	2.01838042	1.58046722	1.84476188	4.12943375	1.75685825
ENTPD6	4.35052258	3.56570346	4.22409928	4.17784814	3.9149553	3.82235888	4.58252575	4.53955507	4.97707061
ENTPD7	2.80366736	2.89100547	2.66219753	2.79211212	2.68290813	3.26593092	3.22985118	3.32522066	2.43896755
ENY2	2.47179448	3.07043603	3.23804838	4.06437884	3.22268616	3.22410034	3.26317097	2.5838123	3.25148482
EOGT	1.53254192	2.9996101	3.21410513	3.33919397	2.8867159	3.35715802	2.76544945	2.12142755	1.78900212
EOMES	0.54887783	1.17036074	0.9770542	1.28598528	2.74866273	1.06465497	0.44344356	0.35572411	0.66094315
EP300	4.74837755	4.04579842	3.81996878	4.09458112	4.52559326	4.10382354	3.96300045	3.72962809	4.20520532
EP400	2.33365135	2.16336376	1.90596575	2.20086672	2.7612455	2.32020936	2.1208014	2.62303763	2.4911081
EPAS1	4.14984465	5.52116493	5.25573143	5.38971443	4.78291577	5.16708297	5.2021979	3.15255655	3.71430996
EPB41	3.5087708	2.44831435	3.11467963	2.75214723	3.39623641	3.4067299	3.31449938	2.6849024	3.67910435
EPB41L1	2.53821419	3.70114765	4.18041122	3.6395653	3.84342144	2.26922422	3.31513477	3.93198958	3.26125898
EPB41L2	2.56516658	4.10703515	3.17486194	4.05846823	3.17851829	3.30537169	3.34292173	3.2158594	4.57777277
EPB41L3	0.91524788	3.03263293	1.9744749	2.36729224	2.1926363	2.5846806	1.96911567	0.93506971	2.55071666
EPB41L4B	1.19060456	2.10255904	2.0198034	2.14471698	1.84772257	0.77925527	2.50030072	1.97722356	3.19219597
EPC1	1.30876494	1.59911756	1.95788266	2.12563532	1.70366177	1.66426444	1.47266076	1.87884443	2.01778206
EPCAM	7.60741046	7.11996502	7.86085202	7.3489768	7.47907584	6.39783479	8.14442231	7.45587503	8.71781778
EPDR1	0.90552263	3.49330063	3.24126272	4.26015337	1.24115442	2.97705921	2.65196863	3.18551402	1.81075729
EPG5	0.92009676	1.96281192	2.00630804	2.00603767	2.90804719	2.27388055	1.97952775	1.41006794	1.94918115
EPHA1	3.49019216	2.34175198	2.75392539	2.50222242	3.0177239	2.63608823	2.88494063	3.1141461	3.00147343
EPHA2	4.664295	2.98931237	4.23120953	5.57170654	5.475819	5.79037204	5.34091063	5.79232772	3.96172763

EPHA3	0.37321655	3.77412368	2.0195363	0.84017672	1.13807255	2.9789483	0.70677596	0.70636023	0.39871571
EPHA4	0.15536217	1.51793452	1.26815977	1.47801151	1.3512253	1.02473097	0.29655549	0.36881524	0.12153337
EPHA7	0.00487974	2.35781565	0.50692478	0.43689975	0.0268496	2.04687028	0.31661498	0.16367598	0.07264987
EPHB1	0.17726624	2.3957031	1.62934889	0.39846925	0.18171963	0.50757061	0.19617003	0.34340095	0.92966345
EPHB2	0.64815024	3.61622078	2.29511608	1.23796489	1.15121757	1.44465121	2.4455624	0.55567871	4.79998036
EPHB3	3.87182094	1.81653122	3.54892078	3.74886566	1.72126829	2.81466984	3.26132078	5.8009805	3.98791454
EPHB4	4.76636522	3.76089271	4.55627956	3.89706813	2.99585427	3.75516212	4.60453275	4.68379651	4.04230341
EPHB6	0.62369304	1.04248081	0.60172489	1.33822429	0.33688325	1.01661693	0.27855971	0.26658305	0.30089239
EPHX1	4.7406647	5.66189722	5.86485531	5.07074048	4.62482274	4.88337861	5.58440137	5.44582308	3.5883674
EPHX2	3.92152108	3.42584541	1.26273456	1.82838707	2.44989718	2.07882796	2.70875366	2.47569679	4.89355967
EPHX3	0.67459339	0.75098769	1.1796144	0.76589183	0.52983411	0.35943563	0.50216068	5.19601448	0.51688832
EPM2A	0.60174267	1.59526065	0.5783817	0.92757339	0.72369608	1.20879312	0.80995186	0.50895731	0.64201003
EPM2AIP1	1.2938339	2.54638747	2.12282454	1.90800888	2.48002379	1.68523101	0.71219489	2.14859881	0.55962097
EPN1	5.03389464	2.67100116	3.45340437	2.14176168	3.26609778	2.93832049	2.57362827	3.55289402	3.53440639
EPN2	1.39415926	1.98471985	1.83995528	1.69060535	1.27048732	2.13002648	1.93605327	2.22717025	1.48016111
EPN3	3.71002529	1.93631584	2.54301199	2.1876203	2.1460873	1.9257395	2.44017857	3.28269042	3.06943147
EPOR	1.40550715	0.93486451	1.39833833	1.61237237	1.19143793	1.25318339	0.67289079	1.56894632	1.24574339
EPRS	4.56700411	4.72380558	4.70368929	5.04620944	5.60120881	4.86576308	6.2436821	5.39065759	5.62229222
EPS15	2.92354135	3.73371175	3.68871802	3.94009424	4.12214597	3.87301371	3.39146766	3.18761321	3.76120515
EPS15L1	2.62634409	2.12050012	2.28406836	2.40549154	2.68326779	2.30551584	2.19873961	2.35594027	2.27070268
EPS8L1	2.23949951	1.07034751	1.98396093	2.01708143	4.09824958	3.00936493	3.14770363	2.80345335	2.05740733
EPS8L2	4.12801973	2.96470697	3.42524934	3.66710174	2.21107594	2.91819585	3.58201018	4.59903547	4.30159115
EPS8L3	3.72072069	3.34241804	5.17244999	3.72681013	3.6626503	1.70414933	2.8050259	5.32833014	6.26272066
EPSTI1	2.63157457	4.56275805	3.61023464	3.76664125	6.02902053	5.31342669	2.76513775	1.99557302	4.87927432
ERAL1	5.58014242	4.03177541	4.56580349	4.50550274	4.96989321	4.30243873	4.29749637	4.36183475	4.81402949
ERAP2	3.05442969	4.55364748	4.28932506	4.14781399	6.07411215	1.28579011	3.50903547	0.92593287	4.22131893
ERBB3	5.12532232	3.93351256	5.27821151	4.50235033	4.51058675	3.70772582	4.6337207	6.99831477	6.00782707
ERC1	2.16318104	3.27058631	2.1160427	2.59454429	2.26111908	3.36413451	2.6215823	1.97855603	1.94924039
ERCC1	3.81643952	2.90549836	3.04665562	2.57476644	2.77724187	2.73037637	2.50403159	3.04955717	2.93163087
ERCC2	2.74554021	1.61432291	1.85831062	1.99343453	2.00487521	2.12246537	2.29520207	2.72253147	2.50678004
ERCC3	2.21579924	1.98765791	3.25766109	2.78311847	2.73062321	2.56557438	2.50977241	2.78251317	2.70857236
ERCC4	0.62895228	1.5138807	1.05114313	0.94709476	1.48208892	1.36299669	0.8064816	0.88970365	0.8049719
ERCC5	1.97643977	1.7373722	1.85214319	2.01931241	2.22705071	1.70544322	1.91820001	1.95932389	2.51454289
ERCC8	1.20348723	1.28271132	1.99081854	1.64588378	1.64111553	1.47455667	1.51572184	1.40554407	1.52709872
ERF	5.91815878	3.65208715	3.92405763	3.69692221	3.69004896	4.24257621	3.81223554	4.0635052	3.84429794
ERG	0.66835757	2.71467672	2.07661186	1.99856721	1.22351108	2.48681526	1.17344811	0.90597441	0.71872384
ERGIC1	4.74163185	4.11455423	3.76630423	3.60124538	2.78880595	3.55751678	4.67202192	4.60088436	4.26258028
ERGIC2	2.47884599	3.68337402	3.44114944	3.65114855	3.74124284	3.46446957	2.83981806	2.83490311	3.07164821
ERGIC3	5.41444231	4.69869083	5.5674831	5.64435331	4.14788946	4.85580781	5.92661019	4.87341516	5.62710454
ERH	6.67036903	5.74754943	6.14920658	6.02535846	6.70142072	5.41389831	5.44155683	5.77182131	6.10409126
ERI1	1.91437008	2.20053875	1.67720605	3.10442172	3.32909272	2.79487868	3.19629499	1.87280648	2.23575067
ERI2	1.1247905	1.87616093	1.93797327	1.77063214	2.22454547	1.93918974	2.16715564	1.63008576	2.24291783
ERI3	4.37666335	4.10897169	4.90984426	4.12107688	3.87204662	4.34024536	3.64040612	4.1846146	4.4353923
ERICH1	1.65205276	1.08019869	1.04934726	1.4973301	1.59955266	1.59149666	1.65404812	1.2818612	1.41594534
ERICH5	0	1.7582559	1.38205178	0.90908593	0.11040979	0.70707051	0.23918818	1.84076136	0.88912762
ERLEC1	4.58382898	4.85111695	5.8720499	5.17978397	5.0312634	5.00997383	5.47095018	5.32821224	4.77600862
ERLIN1	3.15570622	3.39552151	3.41512217	3.14056491	3.89481435	3.92171806	3.32072034	3.77734028	4.29594969
ERLIN2	2.81731756	3.18446294	2.53601715	3.49727736	2.7379963	3.73542299	4.23117096	3.32397042	3.51301942
ERMAP	1.57008652	1.82003703	2.09457602	2.4747951	1.85243103	2.27206553	1.46979082	1.28075135	1.97361881
ERMARD	1.22836512	1.68064549	2.46263187	2.2524499	2.25198078	2.02731074	1.48540727	1.73323595	1.90039947
ERMN	0.19378591	0.16590872	0.37291668	1.52792508	0.3836708	0.30148581	0.48470664	0.11960472	0.1801435
ERMP1	3.75729089	2.96356533	2.73279226	2.99746029	3.92474466	3.56109297	5.30020369	4.76478576	2.59417243
ERN1	3.08610723	1.60367113	2.81681022	2.09329877	2.20694877	1.98001332	1.6555881	2.70995824	2.32345819
ERN2	0.09863667	3.43913865	3.3404157	3.06918331	2.98525638	2.31875846	4.47544654	4.73067609	4.53323581
ERP27	0.22231812	0.86639502	1.18833688	1.95140278	0.63382471	0.44322296	0.1207039	0.29248399	1.1258211
ERP29	6.23430374	4.68167695	5.91984076	4.8595135	5.40227418	4.96303297	5.52855383	5.4249707	5.25483518
ERP44	4.37434047	4.59042294	5.469209	4.78525596	5.11255496	4.60458243	5.01678583	4.06088753	5.16307149
ERRFI1	4.61823313	3.98358823	4.31724231	4.84777136	5.25365014	4.32354641	4.28963369	4.25025359	3.71062745
ERV3-1	1.37635525	1.86954921	1.33811157	1.38973078	1.78646534	1.65115279	2.1702756	1.89297681	1.25734592

ESAM	3.30016097	4.04618042	4.64966394	3.69295018	3.49237538	4.01512606	3.47296227	4.51328922	2.75153028
ESD	4.9301745	5.08993151	4.03052392	5.08628471	4.87649589	5.18713978	5.22897258	4.91060892	5.97862421
ESF1	2.10403655	3.10574343	2.71151714	3.16116932	3.12483916	2.91841073	2.95021524	2.22507697	3.59033511
ESM1	1.86154577	0.36729803	3.75718284	1.22901448	2.50228154	0.82213821	1.51746367	2.07069818	1.62947875
ESPL1	2.45672994	0.89988297	1.41964634	1.55251189	2.19143754	1.31122816	1.57775858	1.85893426	1.99378591
ESPN	1.73791573	0.96202279	0.70387521	0.87854631	0.23107826	1.42113649	0.77866556	0.13027844	3.25428137
ESPNL	0.10484655	0.3385245	0.32800632	0.18713495	0.02490686	0.20455365	0.01618354	0.01563295	0.07314097
ESR2	0.25349808	0.16861497	0.41179191	0.1233703	0.46642621	0.20922957	0.12310424	0.50188886	0.13130397
ESRP2	3.46113043	1.46380025	3.74886548	2.96501037	3.79343356	2.85234765	3.09432401	3.8558203	4.27360394
ESRRA	4.87265466	2.66209237	3.54662996	2.63157649	2.95683993	3.29064252	3.68178042	3.90631561	4.73439404
ESYT1	3.96063172	4.51169068	4.30637639	4.5691271	3.92596009	4.74337495	4.95912027	4.71206403	4.41856141
ESYT2	3.65887237	4.64340183	4.51154586	4.85305478	4.05726158	4.75251474	5.57859387	4.2851125	4.36245524
ESYT3	0.02575836	0.26361054	0.09849021	0.2950978	0.0204398	0.41143109	0.30532931	0.79575088	0.47325334
ETAA1	1.52086391	2.16965977	2.38397804	3.00854849	2.54135905	2.36110546	2.41860716	2.29331445	1.43031016
ETF1	4.21827566	4.71255774	4.68037728	5.08885022	5.18546252	5.14178745	4.83650969	4.84660606	4.84137484
ETFA	3.66154844	3.8950508	3.93101955	4.03614743	4.81378188	4.27930137	4.1639215	3.22835704	4.41647217
ETFB	3.26321742	3.03942635	2.42596649	2.77269701	2.5660713	2.7223512	2.58840738	3.51570982	2.64844049
ETFDH	1.60984889	3.04147678	2.51807855	2.32891148	2.97671951	2.83834928	2.26600786	2.65447426	2.98361683
ETHE1	5.35377259	5.30780834	4.45372421	5.5515895	5.9300098	6.10400964	6.81957039	5.85868165	6.16217904
ETNK1	3.14350029	2.6721705	4.25661236	3.75614978	4.3845025	3.03404775	3.37294945	3.81314662	3.30741179
ETNK2	0.10980732	1.06379196	0.38073026	0.96017814	0.18360449	1.01369868	0.33384864	0.19918366	0.42380843
ETS1	3.96974758	4.29894771	4.31721826	4.21873157	4.74193559	4.58010587	3.69264208	3.09830272	2.64790508
ETS2	4.4500962	5.98019958	5.27562252	5.3922652	5.05377074	5.82297439	4.92714694	6.23874215	7.25249334
ETV3	2.47343984	2.00501579	2.4617688	2.43105797	2.21285718	2.52100375	3.34490929	3.00495941	2.53285287
ETV4	3.67817824	1.80372789	2.81793037	3.78426233	2.16823305	3.39096531	3.42532867	4.52114451	4.05706756
ETV5	4.50767228	2.02239221	3.2994444	3.46004525	3.20534966	3.43957531	3.09688507	4.32431202	2.12800246
ETV6	3.74652599	3.38725294	3.73944448	3.28615434	3.40317873	3.71622225	3.23361711	3.04625499	3.72239254
EVA1B	3.54602162	3.44223162	3.19665878	3.09747332	2.23691514	3.85312014	3.06692365	2.36868172	2.38446103
EVA1C	0.28062587	1.65110106	2.38691895	1.90442361	0.80503761	1.75503209	1.03499944	3.6599022	0.86196635
EVC	1.2775331	2.48888542	1.99725998	1.78438148	0.8800056	2.06082677	1.41180142	1.82665937	0.51887047
EVI2B	3.19093583	5.03790547	4.96322392	5.06478132	5.941792	5.20535463	4.39811593	3.467358	3.55659502
EVI5L	2.36787428	1.91294018	1.22644573	1.54234254	1.58199148	1.59125813	1.58858137	2.04947698	1.62954121
EVL	2.17484666	2.38836985	2.59371131	2.17050184	3.32164523	2.51379754	1.89617108	1.75614539	1.45301407
EVPL	4.01858877	2.05252326	2.46533764	2.31312925	1.65863337	1.78590616	3.2678534	2.94438648	3.76661146
EWSR1	4.84878738	3.60124263	4.18115688	4.30691704	4.76845908	4.17290185	4.00687407	3.60028019	4.84847234
EXD2	1.95452224	2.57652199	2.5286426	2.67835264	2.04730643	2.7828353	2.25732975	2.59787861	2.8448832
EXD3	1.51852589	0.66739884	1.13212922	0.84554826	0.82487494	0.54242515	0.87874802	1.20545405	1.46758788
EXO1	1.57386135	1.1549687	1.73066211	2.69034218	2.83333374	2.24544626	3.27261996	2.60737901	2.45013035
EXOC1	2.46130358	3.03152961	3.11766324	3.45235259	3.55796053	3.26843204	2.76742256	3.03335341	3.45165555
EXOC2	2.68015399	3.20761567	2.88437868	3.08391243	3.5270588	3.51488013	3.68408531	3.16511255	3.28871142
EXOC3	2.90672429	2.76530565	2.59428324	2.40277304	2.44946507	2.64491304	2.44734532	2.84053913	2.8397326
EXOC3L2	2.00603308	1.75441748	2.95814694	1.54801345	1.90750516	1.91497289	1.60942741	2.14786592	1.17786653
EXOC3L4	2.35089334	0.89907268	0.75447808	0.52156818	1.52741189	0.64164259	0.16564801	0.43266648	1.57547105
EXOC4	2.79669892	3.27879868	3.29950205	3.14413382	2.84197629	3.37978723	3.19257186	3.22033606	3.21288971
EXOC5	1.77852411	3.13153765	2.55424746	3.12385861	3.0882371	3.22078276	2.87501937	2.63908834	2.77364104
EXOC6	2.36983399	1.95812492	2.0367384	2.70780459	3.40759701	2.82657875	1.96918204	2.18783729	2.74025894
EXOC7	3.67035298	2.77681476	3.05648576	2.44848271	2.56195572	2.92269475	2.71651956	3.4975272	2.27980104
EXOG	0.72925412	1.24907959	1.35132389	1.66207607	1.6545606	1.49009541	1.03018407	1.01944402	1.21536957
EXOSC1	3.38927303	2.94417986	2.61943598	2.8621117	3.19539944	2.65150708	2.521613	2.85146024	2.72705627
EXOSC10	3.12117707	3.11884184	2.96254262	3.588021	3.93029873	3.83328194	3.48570045	2.79077166	3.78528271
EXOSC2	2.57603235	1.9825807	3.22703885	2.96323631	3.14821434	2.69241871	2.25607087	1.98798917	2.7820397
EXOSC3	2.62784838	2.18335416	1.90804076	3.0652096	3.02780503	2.62968914	2.29624096	2.58075378	3.12611111
EXOSC4	4.14839911	3.03362835	4.03991349	3.66041634	3.10411137	3.89910765	3.8188528	3.72698283	3.86129144
EXOSC5	4.53007121	2.88346409	3.80367459	3.40665326	3.93121	3.48123264	3.34432032	3.92153816	4.69819912
EXOSC6	2.95884721	1.96892289	2.36483327	2.0774464	2.33366023	2.29268153	2.38968987	2.2313639	2.002588
EXOSC8	3.45031525	2.68463878	2.91573062	3.17654657	4.23497733	3.03883494	3.50751756	2.61739355	4.10597318
EXPH5	0.55641866	0.34887906	1.60087502	1.59976797	1.55955137	0.26956308	1.23778118	1.54586804	1.21420864
EXT1	3.1530945	3.81924816	3.53981789	3.36140653	3.11570949	3.5209448	4.02565881	3.2401178	3.29578654
EXT2	3.9849515	4.03036977	4.39073352	3.75498426	3.86776063	4.05031721	4.35685858	3.97170747	3.74786672

EXTL1	0.12311916	0.32050316	0.19003551	0.21870085	0.0629384	0.15647947	0.04730104	0.03668185	0.00842009
EXTL2	1.06141349	2.25900032	1.64934781	2.50693175	1.26328398	1.95396525	1.80583705	1.93805454	1.53237276
EXTL3	2.53465353	2.48777709	1.7908174	2.43847014	1.90911164	2.82849641	2.90919496	2.27348376	2.11459138
EYA3	2.67206892	1.99265446	2.54090572	2.78574195	3.05350442	2.49280064	2.06645898	1.89289939	2.76390392
EZH1	1.77181369	2.35223646	2.33575551	2.50322943	2.30047839	2.27261087	1.46317401	1.90756925	2.27904837
EZH2	2.81305894	1.87022905	3.18076788	2.97527567	3.81532702	2.84156634	3.35231655	2.1381316	3.2525224
EZR	8.04296463	5.62155266	7.32362179	7.42352857	7.43191048	7.49467446	7.68234116	6.96002683	6.62882747
F10	0.21516218	1.85699119	0.98262152	1.52204275	0.22049703	0.49137107	0.88536542	0.57303029	1.80136212
F11R	4.25971904	3.21373373	4.52745585	4.43001218	3.53428762	3.7041678	4.97948108	5.08113159	4.38665567
F12	0.21935737	1.53971503	0.78661003	1.989891	0.36359707	2.59888707	2.41579162	2.66313551	2.55741646
F13A1	0.26864902	5.99974645	3.8415513	5.12897592	1.71789099	6.3711786	2.90318833	2.2171009	1.3107686
F2R	2.60752974	3.92931866	5.29650165	3.9008214	4.61878432	4.29378844	5.2090487	4.24429188	3.59540668
F2RL1	4.92900837	4.00468213	5.84249558	5.23908082	5.08064688	4.79344114	5.68716238	6.85949185	5.36359768
F2RL2	0.87379763	2.50798763	4.12088525	1.22057555	2.48359721	1.98102738	3.45677735	2.45881258	1.9550158
F5	0.14518804	1.39469417	1.90633628	4.32687937	1.59857926	1.98610047	0.70447141	3.84466334	0.53682765
F7	0.0190801	0.3713826	0.0138099	0.10551639	0.00590402	0.00703982	0.02846072	0.00369661	0.11633108
F8	0.51982286	2.28572923	0.73696327	1.39814298	0.76386085	1.87230412	0.50767317	0.73525825	0.38295927
F8A1	2.41566057	3.82896363	2.94314032	3.05912136	2.52618186	3.24320739	2.52780778	3.55574938	2.41078716
FA2H	2.06942681	2.51758123	3.95567632	4.59683939	2.86374643	3.58563578	4.8265558	4.83388038	3.54930321
FAAH	2.73162368	1.71275457	3.63380592	1.93043685	2.14027509	2.08289628	2.57328531	0.45604682	4.52706364
FABP1	0	4.28759488	2.22641094	0	0.92674043	0.06881003	2.82610741	0.31544416	2.81499076
FABP2	0	4.0714999	1.64207289	0.06891772	1.2746036	0.08396962	0.39761267	0.20076386	3.59600587
FABP3	1.90981725	3.06868452	2.71538603	2.59034518	3.49618993	2.45841371	2.56983978	1.60420058	1.07043781
FABP4	0.19505313	4.21241392	1.58508228	3.72515358	0.71946546	2.50777099	0.98967266	0.46842298	0.20437752
FABP6	2.55123606	1.23572595	0.77025052	2.55966921	0.01916058	0.3278803	0.01862171	0.05911005	0.74248148
FADD	3.16988881	2.29695059	2.63332769	2.33403216	2.40901743	2.91727557	2.67172355	2.93105036	2.80903203
FADS1	1.00007035	2.46297638	1.65360556	2.39651194	0.95275431	1.80383026	2.58867891	1.63824897	1.53747605
FADS2	1.74346436	2.47245959	1.69061754	2.31595897	1.28081875	2.17403814	1.7000885	1.46415087	0.98043931
FADS3	1.0548716	1.47281535	1.43877541	2.01929542	0.79340498	1.6845665	1.16936077	0.95882706	1.7469328
FAF1	3.00920108	2.76093764	2.85179999	2.89374529	2.91308155	2.97195146	2.77996286	2.84448567	3.18097116
FAF2	3.63330688	3.86884178	4.01086883	4.06007225	4.2904345	4.07515947	3.85458245	4.06546021	4.02375129
FAH	1.00876345	1.43098868	2.00186231	1.4733908	0.61032444	1.91965904	1.98586098	1.70214911	1.78959193
FAHD2A	2.23165627	1.9884307	1.3077287	1.50757801	1.37326971	1.30144732	1.18646562	1.13614268	1.66043832
FAHD2B	0.44425425	1.12762479	0.93496657	1.18246043	0.86271231	1.40936734	0.72035806	1.96498491	1.22146907
FAIM	1.61628856	1.48076588	2.30048046	2.37806673	1.31643355	1.79435089	2.17411044	1.69404886	1.4201412
FAIM2	0.05894908	1.81179558	0.46745669	0.74897844	0.20040394	0.47905057	0.38910039	3.24942707	0.18386113
FAM102A	3.62319045	4.01521764	4.37459444	3.68997994	3.56994821	3.82903875	4.03383086	4.58832741	4.09901248
FAM104B	2.35452386	2.37794191	2.27326755	2.46896489	2.97272183	2.45594573	1.61799998	1.78523865	2.02922122
FAM107A	0.30173985	2.03757429	1.40588055	1.56542395	0.60740924	1.83640249	0.34803247	0.34782472	0.36119093
FAM107B	4.02898894	3.66009208	4.40763641	3.8824543	4.88337749	4.22771455	2.31091099	4.50984373	2.59403402
FAM110A	4.80625337	2.32887633	3.03344305	2.42364799	2.26362388	2.37594556	2.31385977	1.46645503	3.13994406
FAM110B	0.32829682	2.04897504	0.80391163	1.35305865	0.2910532	1.71020821	0.43815619	1.52760124	0.20890025
FAM110C	4.44955043	1.4710676	3.00833728	2.05879941	0.15540818	1.37068925	1.91185741	2.96469903	2.44200782
FAM110D	0.79022244	1.51170189	1.05505237	0.85242309	0.3967692	1.21133673	0.52264622	0.32831691	0.30480189
FAM111A	2.12033176	2.08959173	3.20187538	3.28107355	3.49983027	2.66289873	2.51395165	2.57446513	2.33500288
FAM111B	1.29381069	1.72569363	2.35207117	2.79655701	4.06831213	2.06557734	3.18386941	2.81276851	2.95501532
FAM114A1	2.56093723	4.10423513	3.97222671	3.77391125	2.67070831	4.07744901	4.39032231	3.83696575	3.19990761
FAM114A2	1.53198278	2.54839578	2.19142473	2.27170525	2.61597066	2.45675017	1.7313521	1.97779879	2.02808501
FAM117A	1.1833205	2.39368036	2.25916709	2.37518581	2.02684943	2.45129575	1.0675685	2.35884103	2.32237519
FAM117B	2.3910679	1.5093454	2.24240066	2.58508022	2.92369233	2.01736132	2.59810796	2.1314388	2.57639246
FAM118A	1.69697783	1.90123359	2.03057649	2.25198485	1.93625992	1.80088755	1.41538059	1.11218362	2.64447331
FAM118B	2.00298555	2.81373021	3.07167696	2.74378325	3.14962097	2.46912829	3.17829817	2.30720394	3.56361424
FAM120A	5.35372415	5.23314394	6.54782658	5.66996538	6.38062802	5.71945338	5.51815995	5.75105397	5.88962074
FAM120AOS	3.34212496	3.38945642	4.85392172	3.4935146	3.38421862	3.79338325	2.97362235	3.798708	3.09143091
FAM120B	2.6914484	3.13713554	3.12118935	3.03068439	2.91374302	2.88567643	2.59549158	3.04908045	3.10739214
FAM122B	3.98334117	2.13770714	5.15393814	2.96351136	4.17871989	2.85944681	2.48924285	3.32354686	3.340952
FAM124A	0.53084502	2.01124255	0.70403147	1.09132806	0.61954575	2.23026306	0.51013859	0.65960455	0.53974125
FAM124B	0.19242308	1.3311599	0.73541631	0.79946967	0.19538724	0.80924102	0.30436748	0.28032637	0.17791463
FAM129A	1.66013578	4.60473715	2.69171368	3.17971962	2.89764276	4.37979197	2.60429541	1.74180946	1.36366007

FAM129B	6.1408579	4.83646066	5.38307409	4.93983867	4.84190597	5.28158289	5.75671531	5.12748271	5.13097345
FAM131A	2.38506917	1.33720269	2.36140137	1.33786384	1.01707064	1.74944403	0.58688242	2.07180795	0.97979606
FAM131B	0.13516473	2.42980995	0.18066067	0.13927787	0.10112292	0.28105378	0.22567807	0.10528986	0.44726711
FAM133A	0	0.21739342	0.07584749	0.04457416	0.01322033	0.03907187	0.00643819	0.01241019	0
FAM133B	2.10456105	1.81414121	1.65414888	1.89690128	2.19044884	1.78591173	2.49124403	1.53959258	2.1213045
FAM136A	4.32543074	3.29155246	4.52394051	4.60426194	4.46626401	4.30531329	4.56217657	4.00132541	4.73973732
FAM13A	0.65044768	1.97778221	1.37733499	1.80837469	1.93994071	1.99561231	1.9032615	1.26781192	2.98935583
FAM13B	2.28396602	2.59185986	2.52565684	2.75113034	2.64137304	2.40970581	1.79491727	2.12507279	2.09320968
FAM13C	0.19175335	1.87740392	0.78600749	0.81256961	0.34440467	1.06914791	0.41538203	0.20878281	0.13787316
FAM149A	3.98403829	1.5261403	1.79610977	1.78233205	0.59739083	1.35814703	1.87882777	0.83032718	1.08385292
FAM153A	0.00852191	0.03147673	0.1410718	0.03545602	0.01571036	0.01872047	0.00255592	0.03418131	0.00672334
FAM155A	0.08731252	1.36025293	0.4801961	0.22787941	0.0645454	0.22146956	0.22521186	0.24966794	0.04218327
FAM155B	0.01805006	0.08091965	0.00654633	1.25690629	0.0111463	0.0492038	0.00542616	1.98902186	0.03769282
FAM160A2	3.68213487	2.25867376	2.3197125	3.44853989	3.23098081	2.91979254	3.28094873	2.75667222	3.98065687
FAM160B1	1.82705391	3.2933576	3.30389076	2.96935587	3.28207124	3.1664209	2.63310513	2.61693696	2.50489987
FAM160B2	2.96725754	2.50351112	2.38522001	3.07829126	2.57515171	2.97429124	3.01834826	2.83394652	2.25628214
FAM161A	0.45455934	0.54879182	0.72837577	1.12450566	0.71372598	0.69941678	0.49746685	1.19318207	0.57578215
FAM161B	0.40028505	0.85135984	1.02572597	0.66678521	0.34929854	0.71752673	0.64365553	1.25140475	0.39811012
FAM162A	5.61919153	3.91901094	3.51860512	4.37205249	3.88212737	3.75196789	4.352161	3.90442367	4.60876695
FAM163A	0.19865483	0.11711127	0.15436202	0.08749013	0.0969989	0.30580876	0.07311121	0.03341084	0.03898079
FAM167A	3.43798229	0.78377151	0.63673626	1.29894242	0.53748503	0.28623861	0.71366552	1.21731556	0.42259797
FAM167B	2.48931748	3.32376809	2.58294798	1.77088832	1.88497984	2.63599	1.67680307	1.34455097	1.29102348
FAM168A	3.3865642	3.69165226	2.99684213	3.28325888	2.82603893	3.46790526	3.19216884	3.25026947	2.94635523
FAM168B	3.73420316	4.18006169	4.25445024	4.21993974	3.54565184	4.33753531	4.6493132	4.4357977	4.29688784
FAM171A1	5.3956047	3.60132513	3.20020115	3.6998226	3.31152712	3.40195682	3.58037253	2.71719905	3.70185518
FAM172A	1.65172472	3.22968145	2.54168556	2.68292815	2.03978738	2.71785122	2.08759865	2.32617503	2.57391589
FAM173A	4.00254905	2.69385838	1.78203649	2.24839577	2.02401599	2.58854886	2.83464118	3.7657567	2.40838027
FAM174B	0.67997803	1.13095031	2.40468246	1.66035654	0.94284123	1.68092259	1.72909292	3.51876688	1.94106638
FAM177A1	2.87019993	2.70268411	3.13228409	2.7188279	2.78040159	2.7554839	2.79803962	2.87782513	2.44916663
FAM177B	0.35776664	1.05163177	1.34625061	1.03834403	0.49215603	0.61475837	0.50413764	2.23359275	0.21814795
FAM178B	0.03626083	0.21458685	0.33575407	0.05934658	0.00564428	0.08513521	0.05921576	0.17316489	0.33060736
FAM181B	0.02759747	0.12722172	0.03970859	0.01662053	0	0.11159591	0.01107672	0.00714163	0
FAM185A	1.78399593	1.53252125	1.71978327	1.51905211	1.26815586	1.35294164	1.47728996	1.51540662	1.46029828
FAM186B	0.13173153	0.25298999	0.22981266	0.1190782	0.18992217	0.15975188	0.09324916	0.21627097	0.15635755
FAM189A2	0.03585943	1.03981186	2.84165068	1.23813645	1.82642063	1.11136874	0.95723303	3.98559076	0.14513108
FAM189B	3.80134774	2.8095726	2.7682037	3.06189476	3.08788927	3.7131762	3.5889694	3.9647009	3.66173085
FAM193A	2.99200171	2.42013643	2.22568318	2.45223681	2.83240172	2.75592441	2.27148983	2.75853178	2.63835919
FAM199X	2.90966853	2.51482374	2.85325998	3.21762197	3.7997618	3.0596163	3.12836432	2.95035926	3.15826728
FAM19A5	1.43070901	2.72646325	1.11228303	1.28271114	0.16054662	1.59936743	1.10336512	0.79102589	0.3086357
FAM204A	1.07851808	1.35826429	1.90926971	1.65930489	1.50923327	1.45893537	1.24268212	1.30435118	1.69700016
FAM206A	2.7670365	3.04451373	4.00609112	3.66668828	3.5880439	3.30075726	3.01895951	2.54943849	3.82698947
FAM207A	3.80459158	2.54609133	2.35064881	2.28393811	2.66428703	3.2020436	2.79107487	3.91691045	3.13041047
FAM20A	1.53900015	2.45202407	1.72153885	2.04268625	1.78707034	2.25059147	1.17187355	0.65343822	0.66588406
FAM20B	3.57809117	3.51113349	3.42820936	3.60234488	3.41880482	3.52940272	4.01943225	3.70579373	3.28525269
FAM20C	2.80121208	4.38064936	3.76561819	3.20343609	2.0414173	4.06126854	3.66321411	2.93679378	2.13913022
FAM210A	1.65931282	1.99273691	2.03136337	2.55325681	2.12690352	2.27287509	2.46873331	1.9747814	2.68532172
FAM210B	3.07665577	5.20529776	5.50994554	5.1354771	3.30065027	4.43142597	4.59065625	4.03329099	4.83607567
FAM214A	1.28922509	1.94842879	2.19264325	2.28229494	2.22506354	1.69229037	1.580983	1.56402717	1.837591
FAM214B	2.28177815	1.81265035	2.48809282	2.20360801	1.90452754	2.09574101	1.82229191	2.18210437	1.81106516
FAM216A	0.58946043	1.48145962	1.2341274	2.11642229	1.83446733	1.54516853	1.03501455	1.21360651	1.56709578
FAM216B	0	0.6128521	0.17939757	0.10206079	0.01351071	0.07876949	0.10183908	0.12985662	0
FAM219A	2.8369551	2.07382175	2.87311189	2.28544284	2.22609684	2.44545775	1.91336563	2.07214845	2.63873152
FAM219B	1.41788957	1.29457266	1.80126315	1.6328754	1.18063732	1.3849788	1.36030811	1.36191584	1.58744152
FAM220A	2.72033908	2.86580349	3.10262911	2.74995765	2.45851376	2.59038598	2.62700128	3.17130633	2.27234652
FAM221A	1.38719347	0.89340969	1.55969219	1.28495427	1.30758537	0.70243134	1.28903695	1.76421273	1.80797884
FAM222A	0.89155857	2.17330702	0.52738111	0.50529551	0.70607226	0.71448139	0.84829784	1.20005547	2.83227265
FAM222B	4.32482575	3.03899784	3.66115128	3.66154063	3.32564605	3.23312822	2.71526822	4.03772172	3.89908521
FAM229B	0.86750721	2.51527882	0.87382143	1.96658175	0.39859415	1.73176761	0.782006	0.64767992	0.31662615
FAM24B	1.13876018	0.68023414	1.27891134	0.91723662	0.50904168	0.64145398	0.2228304	0.02367321	1.14953117

FAM3A	3.72460421	2.7724772	2.62765419	1.89769036	2.05851698	2.23922427	1.9888451	2.42843325	3.17433087
FAM3B	0.07881852	2.65329349	4.08523309	0.77896112	0.54860936	0.40593881	0.71620579	0.62392277	5.56003446
FAM3C	3.51207942	4.14622831	4.6099104	4.06908577	3.93613314	4.28106049	4.34199242	4.15504458	3.39591515
FAM3D	4.7375579	5.36913155	7.44324267	1.49124593	1.91489024	0.67288432	5.95535279	5.42097554	4.3074585
FAM43A	3.5176696	2.69143794	4.21718052	2.44581348	1.09468343	2.39744778	1.62846143	2.2637602	2.14517078
FAM43B	0.04189543	0.74425709	0.08931244	0.3190301	0.02595296	0.36591733	0.07438842	0.11035792	0
FAM45A	1.47483281	2.20324562	1.90992186	1.99152507	1.85070739	2.16255514	2.12030499	1.94341938	2.26618178
FAM49B	2.73321862	3.02879119	4.13845891	3.8292318	4.49088403	3.7249216	3.79660004	3.34081553	3.23705418
FAM50A	4.96173985	3.79964498	4.24968257	3.87518797	4.49944444	3.95749413	3.59674006	3.85220175	3.87241171
FAM50B	1.01427189	2.32392655	2.44356824	2.47653762	2.70614363	3.03165757	0.80204224	3.12691946	2.35332656
FAM53A	0.46613006	0.14956176	0.13056066	0.24164852	0.11205541	0.20024451	0.18340098	0.37694733	0.2934142
FAM53C	2.82226143	2.79088171	3.10771778	2.77492606	2.59795451	3.1309318	2.58990453	2.45512813	2.445763
FAM57A	4.52747821	2.95205995	5.42502778	4.03944943	3.9259228	3.82930255	4.29512369	4.36312493	3.86437663
FAM71E1	0.70409586	1.07642484	0.83255653	0.37853525	0.89884533	0.70857776	0.18293206	1.22064746	1.32240498
FAM71F1	0.0143846	0.01646706	0.4835392	0.00864732	0.00887849	0.0367106	0	0.05466939	0.00757896
FAM71F2	0.08433393	0.07612823	0.47977322	0.301208	0.26143195	0.12020965	0.02211005	0.15405599	0.13083487
FAM76A	2.44358045	1.82795915	2.66209433	2.22825223	1.76206315	1.78292369	1.52132559	1.41171962	1.78754785
FAM76B	1.47783042	1.37173062	2.07206705	2.42820176	2.14673425	1.8769553	1.83404479	1.69308193	2.21871109
FAM78A	2.07105261	1.55289591	1.79876363	1.27465096	2.28519687	2.25778636	1.34010987	0.94428727	0.90993053
FAM78B	0.16117836	0.57286562	0.28927644	0.78757264	0.17023808	0.57855999	0.78383689	0.52157512	0.42808316
FAM81A	1.87834432	1.03787735	0.58868184	1.48055502	1.9168571	1.47619095	1.1680311	0.16855235	1.09618425
FAM83B	2.81357993	1.8876243	2.35949134	3.03770915	3.34754684	2.41538017	3.91921187	2.74477734	3.27048142
FAM83D	3.35141555	3.54902771	3.36541011	4.57981479	4.2215797	5.76012785	4.28634654	2.8400536	3.9995775
FAM83E	3.99306703	2.27305692	3.68850085	1.59721309	0.95258617	0.88293825	2.42837691	3.83263369	3.8827451
FAM83F	0.02644116	0.99129445	0.74113923	0.62038593	1.35942113	0.9272227	0.45319865	0.5954162	1.35549005
FAM83G	2.68125646	1.52119286	2.78168259	2.29465364	2.1589047	2.11685151	2.24492789	2.2997494	2.94358376
FAM83H	5.28523008	3.61626043	4.52931242	5.15622286	4.23877713	5.33336957	5.78385278	6.07629412	4.81367808
FAM84A	1.07023599	4.36173665	2.05307295	2.11129158	0.82703215	1.2083791	3.14970536	1.64394189	4.74407293
FAM84B	5.10767656	3.34826867	4.24695629	4.5749998	4.2375069	3.59537151	4.53003844	5.99568695	3.51110912
FAM89A	1.06861455	2.52829581	2.95643531	3.04528338	1.47568183	2.92134648	2.80228717	1.29151384	2.99695579
FAM89B	4.17938119	3.65895272	3.73793966	3.17557845	2.77593768	3.75248073	3.62724717	3.67880623	3.49247656
FAM90A1	0.1105332	0.18501793	0.91417201	0.09984614	0.33761306	0.33721041	0.09962908	0.06493091	0.06638115
FAM91A1	2.69221082	3.3555642	4.21631638	4.41329528	4.45846639	3.93405436	4.2936018	4.27431825	3.64924294
FAM98A	2.99758683	3.58462982	3.41078463	3.61006616	3.97868832	3.66822966	3.74208979	3.22457103	3.6357707
FAM98C	2.87581033	1.3316205	2.02861957	1.71122	1.59483343	1.46796517	1.56404874	2.11817727	1.96859834
FAN1	0.99943181	1.5541656	1.54214924	1.80056792	2.04633492	1.68472363	1.00771589	2.23322314	1.91692737
FANCB	0.43678607	0.52714324	0.58453051	0.90466772	2.02806671	0.67069226	0.64996108	0.47891443	0.77780435
FANCC	1.55955559	1.53432116	1.76019463	1.58764908	1.61878961	1.66086533	1.51004003	0.85674985	1.42761759
FANCD2	1.29120135	1.07406881	1.57011022	1.78559036	2.34826639	1.89265225	2.39744458	1.49147805	2.23582161
FANCE	2.14156675	1.14563436	1.59564249	1.64359719	1.70783907	1.83567141	2.37133288	1.61825129	1.93866556
FANCG	2.75696535	1.7749768	2.48556453	2.28041419	2.65948104	1.92187119	2.32798829	2.65394231	2.50984946
FANCI	1.91884287	1.84394273	2.85915152	2.78977455	3.80294184	2.67370985	2.82673755	3.05948584	2.97072465
FANCL	1.79128794	2.15752722	2.91554171	3.04680181	3.70267377	2.42428971	2.58869126	2.78692333	3.00640981
FANK1	0.40162727	0.50056679	0.88497664	0.44179774	0.10243062	0.22657677	0.2207497	0.91765931	0.75418937
FAP	1.0379608	2.91568398	1.88953181	3.70457295	1.53937129	2.95374537	2.40343039	1.40312676	1.00219502
FAR1	2.33957739	3.2143518	5.0340415	4.47336032	5.06859546	4.0489015	4.18636837	4.90727129	4.16448499
FAR2	1.16768719	2.99049604	1.25881937	2.05834006	2.8704502	3.25175896	2.67664243	0.84568606	4.46545076
FARP1	1.61879416	1.28470445	1.98961467	1.47254039	0.9311457	1.52299376	1.72269652	1.85625743	0.44320353
FARP2	2.5004674	2.07437754	2.40708774	2.56307571	3.23784841	2.2861191	2.65881479	2.59554175	3.08452134
FARS2	2.58152599	2.49744867	2.8547197	2.48283961	2.55870108	2.85398209	3.04421083	3.3031703	2.6626003
FARSA	5.46119654	3.63820355	4.32571866	4.09609246	4.80451077	4.64556218	4.43704832	4.85307727	4.97651437
FARSB	3.46371473	4.12869252	3.91657625	4.48339884	4.65033841	4.44556337	4.2458017	3.98471862	5.33944584
FASLG	1.20030003	0.66915241	1.14925815	0.70856007	3.20574692	0.97347863	0.81631006	0.16170769	1.05385148
FASN	5.65963923	3.39120215	3.72064546	3.9392872	4.05390298	5.12629445	5.31307279	5.48567474	5.13257084
FASTK	4.41203948	3.29387481	3.8681772	3.2996246	3.22143909	3.50309035	3.39808277	3.67822405	3.97001698
FASTKD1	1.40684968	2.17240858	2.75460634	2.9621957	3.26529292	2.59857606	2.70879589	1.91262887	3.50940679
FASTKD2	1.73066571	2.49305752	3.06220713	3.32878972	3.68056484	2.88465515	2.93390458	3.05371582	3.6358196
FASTKD3	1.66279843	1.67263199	2.49347599	2.02743875	2.53712878	1.66160346	1.41249465	1.72707355	2.43704704
FASTKD5	2.72801536	3.05842124	3.76069554	3.58944528	3.76557563	3.20968783	3.73295733	2.67808548	3.79601633

FAT1	3.5888743	4.61247196	3.46286044	4.36460569	5.20908053	4.03605548	4.6809368	5.97836642	4.62809887
FAT4	0.14739609	2.46049905	1.22583368	1.18414644	0.41799344	1.64186944	1.37167217	0.89758214	0.31491528
FAU	7.8833438	7.25224613	6.91512667	7.16304105	7.40726023	7.22111315	7.64161003	7.34457028	7.85233295
FAXC	0.00934498	0.86307404	0.03347515	0.43668499	0.00959403	0.82539357	0.03328575	0.04505909	0.01145311
FAXDC2	0.25807449	3.00909894	1.42794011	1.35689899	0.60342431	1.87198891	0.62116857	1.02434398	1.25922573
FBF1	0.6919797	0.43339466	0.60977152	0.41494244	0.53459455	0.3246359	0.29502456	0.44686038	0.31798964
FBL	6.88462878	4.47340404	5.5013338	5.63429687	6.18937965	5.31726671	5.40886481	5.64098215	6.0202252
FBLIM1	5.32004539	4.34575633	3.91925924	4.05082365	3.93032635	4.00081188	4.17460907	3.66672784	4.19015377
FBLN1	3.16061893	7.51592596	3.84914401	5.73834729	1.41576662	5.75770635	3.78935245	2.8640493	1.29566265
FBLN2	3.10900349	5.74389643	2.93019129	4.39922573	1.85195469	4.71323224	3.68233508	2.61474529	2.00876045
FBLN5	1.86452618	5.08395045	3.95214108	3.56007187	1.74557292	4.09133201	2.73000329	2.31736694	1.28324239
FBLN7	0.20134913	0.32027741	0.54926593	0.44788263	0.19746332	0.35385641	0.26579517	0.15954028	0.20338497
FBN2	0.13439405	0.53890499	1.19906705	0.40477627	0.54473832	1.23079462	0.93261296	0.70952828	0.2693628
FBN3	0	0.07442373	0.35583272	0.03207241	0.01325937	0.01318069	0.00215563	0.04107856	0.01881949
FBP1	6.85112616	4.84250517	6.78494963	3.858201	5.26911841	4.41275521	5.39528123	5.24043812	4.66583152
FBP2	0	0.13723114	1.13009987	0.36775482	0.30859727	0.03045942	0.08125507	1.16252978	0
FBRS	4.6841876	3.50159	3.40574013	3.07066051	3.16421044	3.5436835	3.5764848	3.54637571	3.61658545
FBXL12	3.9493163	2.36671988	3.11163073	2.59243551	2.79619374	2.60534162	2.28622703	2.60337921	2.85425032
FBXL13	0.44272742	0.33327864	0.72730885	0.27253206	0.34679141	0.2563812	0.15954003	0.29093601	0.16678019
FBXL15	3.42391886	2.37752226	2.11877578	2.31456754	1.83054292	2.66218848	2.49772264	2.06103736	3.006499
FBXL18	2.23079669	1.215632	1.46342803	1.04295063	1.43491243	1.619336	1.94381956	1.38685171	0.94595792
FBXL2	0.09655769	0.7913298	0.54085768	1.53822874	0.40366168	0.82789404	0.24546052	0.74161217	0.32334033
FBXL20	1.92387096	1.8588067	1.85626777	1.82940501	1.6476169	1.70389199	1.24153388	1.55306338	2.03480551
FBXL22	0.49815665	1.67360263	0.50502566	1.00767909	0.2079182	1.45214784	0.29389843	0.25902189	0.25224311
FBXL5	4.11421709	4.91507935	4.6997388	4.53946699	4.85326267	4.90043793	4.02946537	3.92933234	3.61217619
FBXL6	2.93764757	2.15701499	2.30197468	3.48056516	2.41657064	2.77427097	3.95993224	3.40177357	4.36822207
FBXL8	1.37292708	0.96861008	1.5989104	1.2480303	0.88380657	1.22856031	1.65972785	1.66363193	2.20217942
FBXO11	1.86354918	2.64687218	2.43883891	2.83945413	3.07802364	3.01013966	2.63447255	2.78697916	2.55223053
FBXO16	0.73794178	0.22801177	0.470891	0.99979105	1.02734608	1.16253151	0.29681121	0.94787123	0.60867213
FBXO2	0.33327715	2.71853486	0.59309872	1.75324371	0.163588	0.94634122	0.36924499	2.24848021	0.66042032
FBXO21	2.48508124	3.1595707	3.24176923	2.71931369	2.82881204	2.7946219	2.37182718	4.26170452	3.27771563
FBXO22	1.48934253	1.74920485	2.11807563	2.09772733	1.88101424	1.80856566	1.81735639	1.14562492	1.9255585
FBXO28	2.18804341	3.14844357	3.34719125	3.4624272	3.65295018	3.31892397	3.98245236	3.79770303	3.39137128
FBXO30	1.11908693	2.59035292	1.64108854	2.27571983	2.01875838	2.39426399	1.98644495	1.21990134	1.60192297
FBXO31	1.5988886	2.9235068	2.16218724	2.36225283	2.10198451	2.52216871	2.5156691	2.13558315	3.1034435
FBXO32	1.58583167	4.58102743	2.37864619	4.03036853	1.66624937	4.09504567	2.77618271	2.41707758	1.70675505
FBXO33	2.24001583	2.68660008	2.62475073	2.77094886	2.66989045	2.83162662	2.30920051	2.96768266	2.88545616
FBXO34	2.97414169	3.60589604	3.75171147	3.23172049	3.71295088	3.31785615	3.34037754	4.31009891	4.01589566
FBXO36	1.07134964	0.85647361	1.26131044	0.77461514	0.8231297	0.55866874	0.39851703	0.73242677	0.55040329
FBXO38	1.89741054	2.27419936	2.69650714	2.58227569	2.9778884	2.49618859	2.15217439	2.45439403	2.77238607
FBXO4	1.7172712	2.28945755	2.65498016	2.35709425	2.92870045	2.64754742	2.16088682	2.10915035	2.58716693
FBXO42	2.77107584	2.06773034	1.98025104	2.45656417	2.37291324	2.37957851	2.0645104	1.84317356	2.33874756
FBXO44	0.82662967	1.54394949	1.91790277	1.81831963	0.92608818	1.43437967	0.85625027	1.86575351	2.14476947
FBXO46	3.22886719	1.70827541	2.55933815	2.41652523	2.79284879	2.71683498	2.44141442	2.68699287	2.83167517
FBXO6	2.61665153	3.23472653	3.21158535	3.14703185	3.06277682	4.58938198	2.66538999	2.33733561	3.14612048
FBXO7	4.39553693	3.5984435	3.56035146	3.5185687	4.12560148	3.933304	3.57395493	2.79789634	3.82740319
FBXW2	2.50041769	2.51344325	3.15739423	3.09825581	2.91582501	3.05465017	2.57326432	2.0772769	2.67664884
FBXW4	3.91168107	2.23597731	2.20644031	2.29330884	2.05980532	2.56716294	2.39484372	2.92375515	2.33958366
FBXW5	5.75181008	4.41931438	4.90921493	4.43073644	4.39056515	5.1004754	5.07323256	5.1048664	5.16077318
FBXW7	1.09888954	1.59450306	1.67644462	1.82013496	1.81819572	1.6383678	1.25204815	1.4574593	1.5062395
FBXW9	3.23825297	1.05760764	1.64727154	1.56424193	1.53202673	2.11079561	2.09631412	1.88710331	2.60442885
FCAMR	0.5774897	0.39018919	0.76196805	0.00616174	0.28137154	0.01130048	1.21651869	0.11068221	1.30668346
FCER1G	7.56580375	6.65775888	8.04163135	7.45763035	8.28668986	7.2729456	7.03560416	5.43423016	5.89814838
FCER2	0.05828435	0.972544	1.1561527	0.26047621	0.18863668	0.208222	0.03517147	0.24695907	0.06121031
FCF1	2.92273867	3.15791129	3.05462515	3.08850131	3.06969388	2.93912719	3.0466759	2.88738863	2.95466864
FCGBP	1.98145911	0.55911103	4.02286208	1.29001952	1.38147182	0.27479885	3.20815622	2.71213534	1.64045168
FCGR1A	2.8193477	2.70093369	1.91217943	2.11653959	3.01812669	2.87406254	2.76136764	1.13430293	1.35060569
FCGR2A	3.24211069	4.63029627	4.95849953	4.4197902	4.79331727	5.43573025	4.60205312	3.52453143	3.5090517
FCGR2B	0.65767305	1.89115055	1.4598812	1.61317301	0.81094407	0.72601081	1.23403095	0.50857736	0.66715211

FCGR3A	5.39648468	5.75973469	5.23470087	5.20143287	6.74037104	6.05395704	6.42904297	4.13046486	4.32284466
FCGRT	5.57382812	5.81060655	6.17830324	4.67226456	4.86978073	5.35851311	4.27322209	5.21964555	5.77009442
FCHO1	2.36654562	1.37199509	1.67897337	1.63602988	2.17640024	1.92432936	1.20079284	1.77813184	1.92893771
FCHO2	1.88130589	3.60365368	3.61267877	3.59624562	4.36017797	3.79552315	3.48103372	3.94310431	3.46673894
FCHSD1	2.10527046	0.86742212	1.95456325	1.72833061	1.61487283	1.47298382	0.89069771	1.78581023	1.23342503
FCHSD2	2.66484202	3.36519729	3.29768113	3.32916849	3.68658057	3.52514367	3.18172385	2.71343495	2.41291538
FCN1	1.11872083	2.33782625	1.19518757	1.02037625	1.29810969	4.4304805	0.71953096	0.73347921	0.17030199
FCN3	0.56360331	0.67291144	1.23631152	0.73502502	0.50965392	0.61054476	0.86112358	0.24392949	0.66186044
FCRL2	0.04259735	0.08416858	0.45970467	0.33421233	0.33516955	0.10957125	0.03830574	0.17500879	0.12334623
FCRL3	0.06538752	0.31178045	0.76846023	0.30620268	0.96327764	0.51474038	0.27832722	0.34600244	0.40151434
FCRL5	0.09922	0.32519717	1.03718184	0.99522422	0.99907996	0.44306605	0.08526389	0.30107367	0.86654441
FCRLA	0.1277152	1.27133707	1.09170726	0.49287136	0.53273994	0.36950999	0.06512211	0.49292212	0.12326767
FDCSP	1.80710473	4.06813016	4.27406582	1.89966979	1.97892502	0.91497477	0.0750999	3.88041606	0
FDFT1	5.85850281	4.19191984	4.43608641	5.69891526	4.18172789	5.0838209	5.9939984	4.577567	5.2980072
FDX1	3.28484884	3.08767223	2.81205141	2.99851408	3.61149467	3.18824265	3.45440228	2.75724511	4.11079226
FECH	1.61949277	2.65360183	2.79084273	3.19626538	3.04632029	2.9003119	3.18105502	2.2865487	2.72543622
FEM1A	0.63506101	0.20457443	0.62500035	0.4099261	0.31421925	0.56833244	0.35680669	0.49884591	0.51230717
FEM1B	2.51667005	3.88555037	3.51000277	3.81900392	3.58939534	3.94246575	3.44749908	2.83898729	3.58807818
FEM1C	2.70018558	3.22643079	2.89696945	3.32279206	3.69398755	2.90844081	2.98004325	2.30424175	3.68284633
FEN1	4.39870348	3.00024718	3.35139301	3.59119493	4.1824924	3.95553681	4.43633609	3.82800769	4.15763365
FER	0.75249611	1.78966728	1.07448998	1.67998839	1.90569597	1.82387409	1.43444786	0.99210471	0.73568932
FERMT1	4.10130718	3.75793104	4.68393561	5.32599613	5.14710899	4.18548513	4.67218229	5.29780923	5.39087186
FERMT2	1.42231745	4.47192428	2.8794649	3.70931353	2.123504	4.05359121	2.58076253	1.85142686	1.30259409
FERMT3	3.99959808	3.57541508	4.07278623	3.05674126	4.15797834	4.40236359	3.59954194	3.84235978	3.01143774
FES	1.44523286	2.39469581	2.56957139	1.62211493	1.80333628	2.83643862	1.91302079	0.9678642	0.94449264
FEV	0	1.16536478	0.27949542	0.02118615	0.04317693	0.05135688	0.01060813	0.02716406	1.17867842
FEZ1	0.38554968	2.56875499	1.41141938	1.43304391	0.50261322	1.57342311	1.02077371	0.70318929	0.33022017
FEZ2	1.96549453	2.6420214	2.4975866	2.56827866	2.79943252	2.53107408	2.24200613	2.3456186	1.89594683
FFAR4	0.43793043	0.36589963	0.60117505	0.15314318	0.385869	0.26719598	0.79698628	0.29045415	0.36491957
FGA	0	0.06701937	0.32091668	0.01648685	0.02252463	0.03345812	0	0.4402814	0.07086768
FGD1	1.1372166	2.32020386	1.31857966	2.40898777	1.1118714	1.9155506	1.51631895	1.21896109	1.13681317
FGD2	1.00095568	0.84445041	1.71121906	1.01089762	1.91710105	1.39282474	0.97255361	0.54185051	0.85890587
FGD3	1.26966384	1.57654475	2.6220021	1.47674464	2.06754504	2.0509585	1.20588784	1.16228893	1.09658512
FGD4	2.11585921	2.2402164	2.89942694	2.28515841	2.7976734	2.60978381	2.56482463	3.19403284	1.96146048
FGD5	1.32959278	2.91608629	2.38503082	2.0151079	1.66542488	2.43258871	1.81723907	1.64158048	0.9905143
FGD6	1.57379776	2.17432505	2.52533877	3.0963133	3.41358714	2.58371116	1.90945633	2.60625307	1.70048415
FGF10	0.03586237	2.95902867	0.50409272	0.99757135	0	2.65088082	0.30572888	0.26749601	0.14514255
FGF11	0.04512945	0.06598979	0.06476362	0.08023759	0.07851318	0.03564944	0.04249079	0.0373732	0.09007292
FGF12	0.1004762	0.29886992	0.86303401	0.26536666	0.12842384	0.43114314	0.10025066	0.129597	0.04361229
FGF17	0.18692668	0	0.09305769	0.16334737	0.02034718	0.02423827	0.01977517	0.0337883	0.02312589
FGF18	0.08895501	0.9774857	1.0404081	0.91474797	0.1704073	1.41094941	0.16585062	2.88230126	0.71533197
FGF2	0.15297518	2.18981696	0.75669731	0.95406063	0.262843	1.71114032	0.342373	0.29351774	0.16796846
FGF20	0	0.03663222	0.23019297	2.31802994	0	0.55324814	0.01286672	0.11971456	0
FGF5	0.00606343	0.09430184	0.10168624	0.24730203	0.06942322	0.72376407	0.12866596	0.04382339	0.01903706
FGF7	0.47990841	5.26489515	2.1798752	3.78178019	1.52583864	3.90594946	2.16716427	0.92115755	0.47607549
FGF9	2.61490037	0.84557082	1.19324946	0.3185646	0.10950404	0.14044391	0.15911706	0.07829726	0.01260625
FGFBP1	1.66668857	0.38821836	0.24854998	2.80503668	0.01642686	4.00292226	3.22081259	0.50134545	2.44252877
FGFBP3	0.36496972	0.25496996	0.47222095	0.64134832	0.12660669	0.49412788	0.43989591	0.4144978	0.60572993
FGFR1	1.34810972	3.51247774	2.0863849	3.22702082	1.00710324	3.09926522	2.15611488	1.53874107	1.01926986
FGFR1OP	0.84523979	0.51651753	1.63043922	0.84786638	1.25950179	0.79166933	0.93037606	0.86205569	1.0499156
FGFR1OP2	2.41131265	2.48155706	2.92640565	3.00192064	2.95959008	2.82555208	2.16910473	2.2220658	2.55613767
FGFR2	4.62027588	4.2858342	3.90701159	2.90120839	2.17417196	2.43635575	2.29831365	2.84904094	1.76399513
FGFR3	3.67865544	1.23999525	2.91150333	1.39352998	0.61465518	1.51048079	1.04297106	3.63321132	2.98839645
FGFR4	5.88606601	2.71137103	4.24370782	3.33607914	3.36872013	3.60356812	2.60314443	4.88763366	3.51541813
FGFRL1	3.34050079	2.26980043	2.98659229	3.52280432	2.44799344	2.620656	3.5861987	5.72903163	4.28802178
FGGY	1.03873857	1.24946449	1.02243264	0.99825317	0.72427766	0.88468969	1.2634867	1.00139048	0.91919512
FGL2	2.91668533	6.53643957	5.52232453	4.29113524	6.14139846	5.09000812	3.81205821	3.18047209	3.57487091
FGR	2.76766556	1.82406281	2.70518666	3.0642252	3.17218339	3.34162355	2.65874503	1.83479929	1.45170003
FH	4.107661	4.29705825	4.46907622	4.69640031	5.39599435	4.98959799	6.08858168	6.70100797	5.23644463

FHDC1	1.62677585	1.32514939	2.47918103	1.06006696	2.54218845	1.37967336	1.10373396	3.25847646	2.36820953
FHIT	1.60357706	1.28802429	0.5881589	1.14544188	0.99690462	0.84280112	0.80790656	0.86525152	1.3283742
FHL1	0.80719499	5.7443621	3.16956887	4.17746455	1.48323617	4.79912434	2.45439125	1.70126072	1.52043561
FHL2	3.4362752	4.47633873	4.42873125	3.76563611	3.59999252	3.53202624	4.34943275	5.80664448	3.86939479
FHL3	2.81518733	4.6380661	4.04043285	4.31904115	2.28541629	4.65270469	3.66544611	2.76471815	2.75800843
FHL5	0.08292183	2.86044205	2.19589599	1.80391924	1.20461413	1.72237087	1.14017592	1.094594	0.58858818
FHOD1	2.12560784	2.0191595	2.90512773	2.57834766	2.12869794	2.26630153	2.58575953	2.21835172	2.57904651
FHOD3	0.1804112	1.02410209	0.54425875	0.91732801	0.09014428	0.88441689	0.31604233	0.23091616	0.12656434
FIBCD1	0.09212662	2.67748757	0.12500933	1.48340599	0.00584866	0.16496617	0.71321217	2.62571244	0.20043008
FIBP	4.81867573	4.15697103	4.02443275	4.06747769	3.60379976	3.82543651	3.95246446	4.13710137	4.07523988
FICD	1.10334941	1.11539531	1.77796733	1.18369395	1.45208677	1.49381215	0.9711455	2.56381209	1.28552068
FIG4	2.43550834	2.73810602	2.6115025	2.89259967	3.33788703	2.67353139	2.74691023	1.91775057	2.96245839
FIGN	0.05749124	0.94316584	0.81706659	0.25900351	0.6846755	0.48402674	0.13401614	0.11143734	0.02672691
FIGNL1	2.08807611	1.67369974	2.51244585	2.28800883	2.38399931	2.21688826	2.48826878	1.76152981	2.58588467
FILIP1	0.2371947	2.61495445	1.00150269	1.35451675	0.66800354	2.26172646	0.48441132	0.47197396	0.22463131
FILIP1L	3.1264783	5.64190417	4.14025923	4.77523829	3.93764904	5.35610044	4.07812566	3.28613899	3.20855339
FIP1L1	2.50308439	2.15034803	2.62299584	2.33947028	2.98913551	2.23393316	2.45089963	2.77455234	2.71661831
FIS1	5.91623005	5.0855209	5.27278357	4.46818225	4.01853206	4.65858852	4.73618963	4.84820984	4.70963634
FITM1	0.43746577	0.16023317	0.37442227	0.23751823	0.29830882	0.31799801	0.07833665	0.15700257	0.49731546
FITM2	1.53399467	2.09438486	2.41343976	2.48351951	1.7482757	1.4880323	2.45338713	2.03364279	3.06349151
FIZ1	2.44812805	1.21358081	1.71811627	1.45119084	1.62129155	1.52791381	1.36056561	1.66296419	1.62511413
FJX1	1.54998437	1.52216949	1.90928114	1.8669655	1.69981623	2.61934973	1.46418119	1.87755624	1.1918731
FKBP10	3.31907422	5.2488006	5.08651102	5.68044916	2.2843537	5.30083277	4.39148136	3.97135446	2.52625523
FKBP11	1.43952403	3.13171423	3.36460107	3.3939394	2.53298521	3.33950401	3.11640711	3.86807904	3.21661756
FKBP14	1.44704301	3.12902367	2.56494484	2.52483489	2.01498074	2.40674817	2.48238853	1.98337927	1.83509177
FKBP1A	7.25147561	6.55202368	6.97865235	7.05424172	6.40325308	6.85695693	6.59107621	5.28920533	7.22798101
FKBP1B	0.56758093	2.01335857	1.80861945	1.96385976	0.55902436	2.6621914	2.09532945	2.67794067	1.2007261
FKBP2	5.75922856	4.74023931	4.53819381	4.3476378	3.95758417	4.45027457	5.30551327	4.8641996	4.72522071
FKBP3	3.56608475	3.95496591	3.90131415	4.27739893	4.54296926	3.91009801	3.60536279	4.12275363	4.27210094
FKBP4	5.21686565	4.07346626	4.66508529	5.37756774	5.0659858	5.06803386	4.88667532	5.13115073	5.08795843
FKBP5	1.80951962	3.43894697	2.60151593	3.20583096	3.79003242	3.13959924	2.34299739	1.46362587	2.23287198
FKBP7	1.00045199	3.97916216	2.55063876	3.3500565	1.65415935	3.08911905	2.64236393	1.78125628	1.76060647
FKBP8	6.27334784	5.49817052	5.31412294	5.13739797	5.41070856	5.89973794	5.54676597	5.96023249	5.47099758
FKBP9	4.15726829	4.67453054	4.26073676	4.39725939	3.44350197	4.5262418	5.40556904	4.0853869	3.76367389
FKBPL	2.3166594	1.85798925	2.63473062	1.73662475	1.82599268	2.01060139	1.84232989	2.33943484	2.01813541
FKRP	2.39392739	1.29754095	1.54680164	1.60844269	1.56719672	1.85581565	1.06094286	1.56761201	1.5719628
FKTN	1.64000806	2.45523072	2.60424467	2.58005831	2.87913059	2.39651494	1.81054645	1.68467723	2.43052563
FLAD1	3.39283783	2.6951394	3.24046203	3.04206218	3.11938295	3.10827957	3.41648306	3.46804122	3.47078433
FLCN	1.28254957	1.23537572	1.94822915	1.97118962	1.86135029	1.92162776	1.53021829	1.29781654	1.84573832
FLI1	1.17907954	2.24046177	2.20306585	1.85399853	2.31892555	2.49794843	1.4664818	0.99282548	1.00767905
FLII	3.69911676	4.19227732	4.50750936	3.9899259	4.5427031	4.75567207	4.72448267	4.62195275	4.65094373
FLNA	5.57505633	8.11773605	5.25717687	6.57447365	6.67761389	7.94410765	6.3180175	5.44570666	5.31930235
FLNB	3.73133667	3.89538068	3.70136897	4.88725252	4.70055623	4.28374711	5.55915731	4.91397595	4.82976319
FLNC	1.11804897	5.19577328	1.26909171	2.5035767	0.49657334	5.23923972	1.7020399	0.71184735	1.80547492
FLOT1	5.82469865	4.92117794	6.00388652	4.76284962	4.34749874	5.0726252	4.80500463	5.09619175	5.03855604
FLRT2	0.10276956	2.31469071	0.60340224	0.84864684	0.11198253	0.92438893	0.29824796	0.19684524	0.06703351
FLT3	0.13380527	0.7441266	0.6712125	0.35270596	0.39841217	0.28799283	0.11101845	0.09792234	0.12775043
FLT3LG	0.60757603	0.8260396	0.6863523	0.4682438	0.79304706	0.81012719	0.37578793	0.57455034	0.39754628
FLT4	1.50769136	1.47175517	1.71835405	1.4321302	1.27740498	2.24748777	1.2376209	1.28088391	0.67267183
FLVCR1	3.86745452	1.42472579	3.47247419	2.24583073	2.82728534	1.83899937	2.78883191	3.37169341	2.20129591
FLVCR2	1.14630829	1.59052464	1.71407645	1.37566355	2.13607475	1.66136431	1.45203989	0.51405599	1.3466774
FLYWCH1	2.58253984	2.09389887	1.64958909	1.65509676	1.91913244	1.98996384	1.885975	1.21023703	1.60898333
FLYWCH2	3.19266335	3.47555471	2.54929127	3.41110174	2.21670577	3.24271742	3.45554788	3.60864465	3.39288035
FMNL1	2.37982325	2.95948511	2.96670555	2.55022457	3.07093748	3.16188657	2.43328391	1.88456256	1.84065885
FMNL2	3.73288414	2.63602691	4.18592885	3.74050174	4.33290128	3.51430696	3.47697294	3.06481724	2.72411843
FMNL3	2.20456495	2.24565082	2.67626447	2.34362064	2.43344714	2.62667986	2.01045557	1.57989752	1.37602188
FMO1	0.28381597	2.95622712	1.42067809	2.99113976	0.8543364	1.4913448	0.99636471	0.2565509	0.74700235
FMO2	0.58695804	2.15415058	1.31214357	1.648597	2.36334693	2.21932491	1.03184612	0.60967049	0.62746326
FMO3	0.47157435	1.10090279	2.35501959	0.68557	2.1247993	0.79665647	0.97455607	1.73707225	0.55365128

FMO4	0.90548214	1.81475635	1.49939392	1.51936085	1.02766666	1.31174825	0.61795729	1.56051681	0.94747359
FMO5	0.64662164	1.2423086	3.26271305	1.56297345	0.43121776	0.96384494	0.89641384	3.65464856	1.22776988
FMOD	3.03104648	6.6511324	4.60263235	5.31920606	3.28187335	5.81823672	4.78290234	4.17031123	3.35823209
FN1	6.30124476	6.71467284	5.47406058	8.33870356	5.86657261	8.33500566	7.63595443	5.75542326	4.83654754
FN3K	3.41003264	1.96345824	3.33316386	1.9592897	0.58726813	2.39169459	1.62001486	4.07977149	2.66046628
FNBP1	2.32186253	3.96259264	3.60315112	3.11217427	4.28315421	3.87805916	2.51366119	1.84357758	1.92162095
FNBP1L	3.62642882	3.23080916	3.83150003	4.09323875	4.28073132	3.27354654	3.73022175	4.59095814	4.55451446
FNBP4	2.35440786	2.26542662	3.33303239	3.3023034	3.64286862	2.6605404	2.71985462	2.56045755	3.23420479
FNDC1	2.74612669	5.10090716	3.01631118	5.1203309	1.4345128	5.4705375	3.66247609	2.16497303	1.1746179
FNDC3A	3.10876332	4.42777413	3.89840402	4.14450988	4.41544553	4.58347314	4.65294386	4.83731989	4.78696331
FNDC4	1.05892156	2.1164969	1.51139052	2.29723557	0.43651589	2.733874	2.01585434	1.35180767	0.89858865
FNDC5	0.05691432	0.8496711	0.4317293	0.76151483	0.12332074	0.43811057	0.11356153	0.0527232	0.09448296
FNDC8	0.05158117	0.05893981	0.24377174	0.03116719	0.01608555	0	0.03109782	0.04973305	0.05417689
FNDC9	0.03155176	0.1557311	0.0564944	0.11932458	0.04831042	0.06306426	0.01896985	0.01224254	0.01667077
FNIP1	1.7361757	2.54529245	2.84614943	2.7511653	2.81701001	2.60746444	2.44567818	2.74692615	2.6224422
FNTA	2.13795577	2.47198572	2.38804325	3.19177083	2.85740098	2.79588006	2.76711147	2.56874166	2.61722067
FNTB	1.77613524	2.04614577	2.09833749	1.70195927	1.89371777	2.07512556	2.25945761	1.80865057	2.33997103
FOCAD	1.3887418	2.31387117	1.79096124	2.50127304	2.43067025	2.70188584	2.43489903	2.3953026	2.54036433
FOLR1	0.06771655	0.96662262	2.13451414	1.59370071	0.34078352	0.12983824	1.42635054	0.54287677	0
FOLR2	1.51368315	5.77293102	4.47430413	4.26993004	3.54764682	5.62839124	3.65386077	3.33485837	1.90235207
FOLR3	0.12496192	0.14228599	0.24248584	0.93505269	0.03181239	0.57081524	0.0309215	0.63965647	0.06703163
FOPNL	3.27413377	4.57711504	4.50757766	4.24277626	4.47621529	4.08296052	4.48106951	3.92864377	4.43282958
FOS	6.21612993	6.20521489	6.42393988	6.8148297	5.1853784	7.44974091	6.40472822	7.11424229	6.45267944
FOSB	1.39430262	3.31103685	3.44224557	3.9580833	1.14996822	6.01334639	2.31730741	2.4729308	2.65347086
FOSL1	1.26643291	2.69490094	3.07848456	3.54425853	1.23926042	4.91613912	3.86067413	3.46342275	2.88958111
FOSL2	5.42019007	5.00990013	5.23825497	4.64490708	4.9838639	5.80385401	5.26841939	5.9895056	4.07340252
FOXA1	0.19649564	2.20198509	4.47887624	2.68083805	4.42851105	3.70787611	3.89419946	6.52255078	4.18956441
FOXA2	5.00381055	0.70324145	2.98682042	3.40457944	0.14745641	0.70588362	3.78942779	2.90634171	2.3039703
FOXA3	3.96893629	2.46859446	4.18400903	1.01671081	3.67721839	2.63320701	2.70157422	3.93402073	4.18281072
FOXC1	0.34510982	1.50732704	1.78847375	3.00466687	2.50321829	2.80350591	3.19259125	3.64094824	1.38213755
FOXC2	0.31575819	0.72596471	0.80229405	0.65528009	0.13813695	0.83114436	0.39091395	0.34162197	0.08990847
FOXD1	0.07229345	0.10638733	0.08324736	0.14381293	0.10564523	1.36860015	0.19868266	0.18730953	3.1838084
FOXD2	1.58982612	0.67848815	2.12731908	0.56540435	1.4281689	0.7611907	0.55802609	1.19218944	1.27510262
FOXD3	0	0.31357484	0	0.34349304	0.01072308	0.59616856	0	0.06581331	0.05407493
FOXD4	0.8833212	0.02099706	0.07788976	0.24399369	0.10947617	0.20809981	0.26203962	0.44889097	0.11199327
FOXF1	1.95111453	4.09206647	2.88958311	2.14629414	1.05041558	3.557117	2.84491452	1.96526704	1.27049639
FOXF2	0.29985083	3.58372429	2.47847771	2.01516599	0.93955893	2.24070298	2.37192231	1.60780966	1.28266983
FOXH1	0.24082281	1.15792129	0.18718321	0.05148431	0.13682063	0.05253056	0.23290976	0.05505513	0.09621521
FOXJ1	0.05416894	0.14287074	1.94118775	0.03274242	0.00847625	0.04983253	2.97548786	0.02111935	0.0428782
FOXJ2	2.44085353	2.8347263	2.59339172	2.75661598	2.88065198	2.93744901	2.67357031	3.06020966	2.63883925
FOXJ3	2.66690706	3.06817851	3.75231952	3.54669398	3.42093519	3.44961135	2.95466069	2.75494984	3.70528048
FOXK1	3.165169	1.95976986	2.03221658	2.1024605	2.33581628	2.4284579	2.47562167	1.9405209	2.03216055
FOXK2	3.5306039	2.28901307	3.00852975	2.51620914	2.39081927	2.74404719	2.68087171	2.92995159	2.8498598
FOXM1	4.66766826	2.59569924	3.93245875	3.25890686	4.29995162	3.86447936	4.16460263	3.54109917	3.99825528
FOXN2	1.76215806	2.60955103	3.25110712	3.24626848	3.69590672	3.62240898	2.83286435	2.28519417	2.93868285
FOXN3	1.92782928	4.1585106	3.69762432	2.90774037	2.94960592	3.5727261	3.02832815	3.36054747	2.92236504
FOXO1	3.49139247	3.48310132	2.51534394	4.00394076	2.99234216	3.88725325	4.15446081	2.9738268	1.79879416
FOXO3	2.17647546	4.11371087	2.83566153	3.51909786	2.88388183	3.82605803	4.26719602	3.33170903	3.09241734
FOXO4	2.28811163	2.46384616	1.60584166	1.98996193	2.04726995	2.42354148	2.69876544	2.35565218	2.39097396
FOXP1	2.72528141	3.13816842	2.6068317	3.57071225	2.45743132	3.18490395	3.16712682	2.94993717	2.68286692
FOXP2	0.02971248	1.97261336	0.42843179	0.49772088	0.07349429	1.26886956	0.2319075	0.10882545	0.42485495
FOXP4	4.61212506	3.6997023	4.94298722	3.4808511	3.95376821	3.81188871	4.35506858	5.15058878	4.56605464
FOXQ1	2.37386599	2.22608994	5.18649329	4.80222648	5.52211121	4.75197502	5.50727796	6.65412115	2.76536102
FOXRED1	2.16307298	1.78249708	1.66651202	1.99345214	2.26854383	2.15662436	2.41497993	1.27905971	2.44014128
FOXRED2	5.49738596	3.38785433	3.15482934	3.36184101	3.10031498	2.58844424	3.58631335	2.98906372	2.57346031
FOXS1	1.86512722	1.24567423	2.04995701	1.14925193	0.69021655	1.47891176	1.19158532	0.68945596	1.18170943
FPGS	4.63281393	3.04079128	4.45663272	3.39548097	3.3205702	4.24947516	3.90156118	3.43933409	3.94706809
FPR1	2.10152234	4.58657683	3.5658449	3.7134451	3.07093857	4.78210016	3.53726561	3.54020546	2.16047916
FPR3	3.47806119	5.58969277	4.72610205	4.65686811	5.86397497	5.22174607	4.87960577	3.52205863	3.75156561

FRAS1	0.0646967	0.4458538	1.9018167	1.60511842	0.82603665	1.50891623	0.0421977	1.66796119	0.2088007
FRAT1	1.22347531	1.40579843	1.26632792	1.17914361	1.79141254	1.74009851	0.88350973	1.75932297	1.50825303
FRAT2	3.81809283	2.56007912	2.71698661	2.54644957	3.6828281	3.05711163	2.94164061	3.88381886	4.2087246
FREM1	0.02017694	1.72209672	0.28340933	0.5740272	0.02891508	0.59262248	0.02212806	0.11293081	2.65829837
FREM2	0.10816678	0.45527662	0.8342985	0.85373179	0.00965521	0.10810318	0.00536907	1.44840217	0.00471566
FRG1	3.37608236	3.86757815	3.12775728	4.29219021	4.52291156	4.07422579	3.45263888	2.88967991	3.60069033
FRK	2.46855459	1.63020213	2.10979227	2.11060424	3.04100706	2.03323216	3.23281554	2.39555024	2.09150147
FRMD4A	1.4595744	1.62904983	1.71980717	1.21518247	1.40584939	1.55638072	1.2981125	1.16858617	0.70955022
FRMD6	0.85830236	3.67206275	1.62553504	3.34850709	1.06178379	3.2668206	1.79959463	2.24679381	0.73116271
FRMD8	4.35279874	2.59291351	3.57110036	2.99833321	3.13284784	3.25464918	3.78122869	3.16326492	3.0721269
FRMPD1	0	0.29211614	0.20077417	0.48317875	0	0.04715095	0.00741781	0.06552599	0.02909135
FRRS1	1.69604572	1.68632892	2.21199078	2.0019183	1.8265099	1.74795727	2.51174327	2.32186031	0.95651583
FRRS1L	0.01327712	0.60478642	0.18655524	0.20133984	0	0.4231286	0.00265903	0.02716077	0.00699418
FRS2	1.73255472	3.2141132	3.25332577	3.01059146	2.84747989	3.07943129	2.42388953	3.14898279	2.62154363
FRS3	1.58380932	0.88005731	2.02332198	1.17766846	1.00529772	0.97895834	0.85982758	1.45819345	1.61049584
FRY	0.55943457	1.57497225	1.27873646	1.08262371	0.96447182	1.34125075	1.67010903	0.81975482	0.39685489
FRYL	1.64646575	2.54466395	2.47342607	2.00359334	3.29045945	2.46217669	1.78766086	2.59975053	2.50743915
FRZB	0.85352076	3.87919787	3.69334397	1.51346339	2.87075934	3.09292331	1.76382001	2.0184448	1.53512144
FSCN1	3.75205156	4.63668845	3.83118775	4.12473391	2.71271926	4.59734283	6.3139365	3.20559283	2.69945024
FSCN2	0.67859107	0.04077664	0.02584936	0.49093849	0.12762692	0.26576865	0.28356967	0.43513055	0.5134807
FSD1L	1.46982387	0.62348122	1.82177968	0.88879449	1.47285904	0.83566218	0.17844965	0.42020352	1.36341301
FSIP2	0.00459412	0.06862695	0.09436055	0.15184013	0.01784224	0.01959092	0.11383024	0.20570682	0.03583166
FST	0.47652767	1.19630439	1.32893881	1.47323759	0.93511171	1.24254498	1.01702416	0.47192876	0.11170289
FSTL1	3.99975186	7.52614729	5.67804908	6.03834693	4.38303153	6.17199865	5.4144289	4.46038755	3.67235168
FSTL3	2.87012587	3.18786689	3.61677236	3.57978122	0.91671917	4.61683013	4.05076208	2.20988958	1.95359308
FSTL5	0	0.06479479	0.03401429	0.01219779	0	0.04916465	0	0	0
FTCD	0	0.07374206	0.09240431	0.03908877	0.01349852	0.07486534	0.006574	0.06633007	0.01152546
FTL	12.9836294	11.5694127	12.2715275	11.093307	11.6086181	12.0432589	11.500018	11.5739286	10.6670963
FTO	2.13789342	2.59140973	2.01831267	2.2952524	2.03070428	2.21127377	1.87135781	1.9321168	2.03550891
FTSJ1	3.59172748	3.64587078	4.02247702	4.2725808	4.51809154	4.12139824	3.67265746	4.51599691	3.96055218
FUBP1	3.80330853	3.47683777	4.05923012	4.07112654	4.62743247	4.23492826	4.19626392	4.51440135	4.64616472
FUBP3	3.34817277	3.25748867	3.70120658	3.64905613	3.35664652	3.63171835	3.4318867	3.06047964	3.53660923
FUCA1	4.86844904	5.93841212	5.93964467	5.4377361	7.16972658	5.9071189	5.65172138	4.98955735	5.55572067
FUK	1.89355919	1.47110501	2.16748349	1.55193315	1.69799798	1.19442581	1.60797418	1.85931744	2.33240676
FUNDC1	3.15817812	3.21967817	3.76712376	3.47768434	4.93234067	3.03769002	3.159277	3.34464806	2.54377639
FURIN	5.82148605	4.32893089	5.08910621	4.6213015	4.74144408	5.17968098	5.21830653	5.26678092	4.76808188
FUS	4.81762423	3.22063646	3.80925207	3.6856808	4.16920082	3.61868039	4.02103983	4.08832314	3.97496624
FUT10	1.17166922	1.46349614	1.3830788	2.21715236	1.95248078	2.16995804	2.14415944	1.83469334	1.65350592
FUT2	4.30119266	2.47823213	4.17364944	2.88726509	4.85008984	1.57925624	3.25814764	5.56173404	3.69144714
FUT3	2.57642315	3.93918627	3.90242174	4.27054342	0.56611827	2.18102618	5.47139526	5.39864534	5.42872846
FUT4	3.98991388	1.89720004	4.43979145	2.75770594	3.73088462	3.23115119	3.07380263	3.65778801	3.83200316
FUZ	0.6463263	1.7753006	1.50458845	0.82845067	0.81960881	1.38108294	0.91985933	2.40072977	0.41963484
FXN	1.89380075	2.35500697	2.98734827	3.26404756	3.22910443	3.13966627	2.81778374	2.6675544	2.94405723
FXR1	3.20434266	2.86809724	3.36173395	3.71072893	3.49643607	3.44891028	2.98707282	3.84242543	2.95531605
FXR2	2.82210044	2.26365424	2.87865551	2.05170734	2.46286569	2.32349644	2.61566518	2.41711354	2.61788186
FXYD1	0.07377176	1.1140358	0.20365831	0.64761498	0.26544056	0.44001552	0.08788544	0.08496599	0.03924701
FXYD3	5.08221758	4.42760526	4.69741016	5.76783086	6.50629957	5.8351983	6.50463766	6.78237091	4.23257817
FXYD5	6.20301923	4.99034618	5.7643548	5.93971073	7.18101609	5.7808783	5.95942036	4.81505943	6.04709447
FXYD6	1.22664171	3.47393588	2.0491801	2.11153602	1.22539052	3.36895036	1.36452127	1.05891189	0.49221753
FXYD7	0.0790108	0.13321449	0.13969851	0.08284405	0.13141557	0.14197891	0.0241052	0.04620461	0
FYN	1.81097279	3.42924137	2.95924905	3.42280405	3.59142122	2.80510708	2.6138954	2.14181909	1.92209167
FYTTD1	3.23822338	3.68146595	4.01333707	3.93852012	2.83074007	3.68115811	3.70872142	3.53622953	3.35933615
FZD1	2.96259213	3.72298482	2.03773004	3.09568354	1.79944032	3.65395804	2.41308038	2.42821616	1.39637825
FZD2	3.21180354	2.56206204	3.82119277	2.23793616	2.09229357	2.4583395	1.82155613	3.24806165	1.13393299
FZD3	1.20565506	0.99711553	0.66938572	1.85539548	1.64355152	1.75868005	0.69083498	1.04215327	1.91342988
FZD4	1.34272622	2.98664053	2.79136972	2.39944684	2.27012652	2.40038136	2.37248287	3.16201261	1.11466235
FZD5	4.88872768	1.97032684	3.63825242	2.5669238	4.6628764	2.73145834	3.837978	3.45071872	4.70434037
FZD7	1.82104609	3.65765795	3.22016923	3.48637521	3.81786352	3.89861247	2.41202221	1.1885122	2.28931402
FZD8	1.70337996	1.77854406	1.75210939	1.23042232	0.58689458	2.20878845	2.02879818	1.95239897	0.91306109

FZD9	0.03084411	0.2075494	0.2092934	0.34739572	0.02852496	0.14690087	0.08162675	0.0414544	0.35421731
FZR1	4.30714289	2.26999751	2.79469651	2.68966508	3.1108642	2.95466734	2.95619986	3.12393239	3.03077658
G0S2	4.21644401	3.95450405	4.92595782	5.70910023	4.19278343	5.0003147	3.95193586	4.80415884	3.92002216
G2E3	1.20446565	1.76682085	1.82653038	2.29908983	2.95812474	1.80447253	2.023126	1.90487986	2.11414314
G3BP1	3.59591608	3.67992086	4.22953306	4.5670922	4.38915612	4.37035366	4.072441	4.17912611	4.17104237
G3BP2	3.78858281	4.58284981	4.21349628	5.07892928	5.03869617	4.87308512	4.74594662	5.18515046	4.68820793
G6PC	0.02194688	0.01261197	0.41982978	0.0066187	0	0.01213695	0	0.06254994	0
G6PC3	4.59873009	3.88463696	3.9885947	3.55172486	3.26408237	3.90523154	3.43501387	3.87402501	4.2934555
G6PD	5.64752192	3.97592147	3.61164107	3.58055775	4.38664416	4.13001797	4.10041523	4.24932797	3.52450514
GAA	5.77974256	4.1075148	3.66257885	4.51888235	4.53207728	4.50595296	4.62389214	5.34564667	3.13085285
GAB1	2.00027054	2.73672391	2.05842726	1.98971005	1.74678957	1.98649913	1.66849704	1.67352814	1.91862457
GAB2	3.61278306	2.74082459	2.77817219	2.56010958	2.28610139	2.8389358	3.14241718	2.99687543	2.77856179
GAB3	0.75292826	1.34000654	1.98796378	1.21204363	2.39979322	1.36398429	0.77271045	0.59251405	0.87474521
GABARAP	3.71593098	4.66999332	4.28304018	4.54963939	3.88993832	4.53406259	3.88887361	3.43249496	4.59759099
GABARAPL	2.10482596	3.42711991	3.35067432	3.12598753	2.62222028	3.67510097	2.64421522	3.60479322	1.49894642
GABARAPL	3.70299525	4.24532417	4.43954968	4.26176404	4.31108168	4.19714388	4.11212321	4.47097698	4.11766188
GABBR1	0.44770005	0.83661085	1.07260411	2.19720041	1.2619639	1.09294777	0.29062173	0.61995033	1.13498468
GABPB1	1.69909431	1.61448121	2.08369683	2.02766879	2.23147831	1.82604155	1.5656886	1.21889693	1.53838591
GABPB2	1.35853858	1.22557867	1.55116057	1.44724787	1.49686782	1.00290475	0.68458748	1.24360948	1.83971839
GABRA2	0.00913572	0.23372906	0.13877187	1.10499787	0.09644654	0.04856083	0.0556037	0.03719383	2.84947735
GABRB1	0	0.05862711	0.43161047	0.11377292	0.00713256	0.008504	0.03432747	0.14864315	0.90391534
GABRB3	0.00571451	0.33116118	0.17141578	0.78962646	0.90900408	0.11422155	0.04055486	2.2954526	1.42357791
GABRD	1.08869766	0.88602689	1.95167282	0.64707362	0.60849051	0.55757105	1.01408707	0.82178988	0.32748729
GABRE	0.11649866	1.18877348	0.69678287	0.85384103	0.15278633	0.41587913	0.28717813	0.21417998	1.44209026
GABRR2	0.12515515	0.19251268	0.41743384	0.19247681	0.27424198	0.10980451	0.0762152	0.04343214	0.20766373
GAD1	1.19364537	0.01260892	0.77093586	0.18172218	1.42857245	0.29172601	0.08480716	0.35877147	0.62416826
GADD45A	4.21392633	3.71138886	3.63291339	4.71907351	3.61941444	4.38792809	3.03160658	3.13401435	3.62079823
GADD45B	3.74357418	3.99613396	3.80038383	4.69702298	4.24189226	4.73551151	3.07372602	4.34661856	2.46989362
GADD45GIP	5.77007042	4.15185692	4.11480065	3.83124732	4.05363101	4.37908835	4.20862435	4.26177891	4.52054776
GAGE12C	0	0	0	0	0	0	0	0	0
GAK	3.39843438	2.54969941	2.34689337	2.5170955	2.89720403	2.90050839	3.03231968	3.46964564	3.54463829
GAL3ST2	0.10266734	1.42188417	0.01909217	0.07767172	0.12548558	0.15726981	0.62372947	2.32635444	2.80326232
GAL3ST4	2.68284158	3.3590134	2.87465267	3.01125358	2.97837011	3.16470243	2.39142423	1.95410903	1.91659581
GALC	2.40625644	4.17647353	4.06335444	4.1634269	3.91610889	3.35070834	3.73445545	4.22646841	4.09654475
GALK1	3.504094	2.35451799	2.88037576	1.6111909	1.44781715	2.26246112	2.41440077	2.50107319	3.15604384
GALK2	1.10695232	1.89556228	2.00006181	1.38292385	2.16744581	2.28427358	2.06716396	1.57311166	1.45987038
GALM	4.04923227	4.01663928	4.09469377	2.99351413	4.52875068	3.76820012	4.6683441	3.65813354	4.55705057
GALNS	2.18517702	2.42517269	3.03652339	2.06543171	1.78679757	1.90333633	2.83209558	1.61970472	2.06287436
GALNT11	0.6362331	2.2454231	2.83385157	2.72966018	1.70019706	2.29528492	2.38361596	2.36074159	2.17533978
GALNT12	3.9929398	3.42951009	3.9354889	3.49212288	3.69220199	3.03748342	3.26371645	4.02031108	3.02292622
GALNT14	0.25531656	1.14010843	0.53761458	1.74110504	0.1929736	0.84696461	0.30847684	0.56943317	2.35529919
GALNT15	0.2685786	2.54498881	0.78768124	2.01806366	0.12851924	1.50439676	1.26819493	0.32623682	0.16553913
GALNT16	0.02342613	1.34053526	0.35857259	0.46344219	0.06899185	0.75186749	0.04571961	0.08704602	0.01937725
GALNT18	1.56080022	1.26561771	1.7549083	1.97577792	2.76756746	1.68991946	1.11317811	0.96169603	1.70436111
GALNT4	1.72948178	0.70708729	1.44572177	0.37580135	0.45307274	0.51753622	0.62345955	2.20487235	0.43214701
GALNT6	2.46055071	2.41917418	4.31173863	2.6524036	2.61494796	2.49930509	3.5031565	4.63216627	2.7043845
GALNT9	0.01560406	0.0843546	0.05194787	0.03103888	0	0.09120712	0.00936094	0.02992134	1.37985005
GALNTL6	0	0.02463504	0.19179255	0.01178111	0.02169977	0.02157138	0.04416735	0.02261975	0.01443713
GALR2	0.2895322	0.05995562	0.4730354	0.06273721	0.17056932	0.15792509	0.13721151	0.10905174	0.14676556
GALT	1.83743847	2.14124955	2.09757606	2.32973589	2.14001863	1.80821475	1.16928334	2.38825054	2.97299941
GAMT	0.82223405	2.2611949	1.29598388	1.57653628	0.52355674	1.29812461	1.0476194	4.48073597	0.53552846
GAN	0.46339046	0.86910448	1.21246045	1.69601048	1.06972916	1.16575662	2.18595518	1.00031242	1.30424641
GANC	1.14603108	1.4890727	1.68897048	1.81481508	2.12572071	1.80511095	1.74932279	1.13804433	2.020047
GAP43	0.03450964	1.08624145	0.60852061	0.60679104	0	1.28744783	0.02075669	0.11010822	0.03625705
GAPDH	11.1078532	9.63801268	9.42613191	10.2638602	9.93870478	10.2753894	10.556563	10.1416326	11.0279895
GAPT	0.45467537	3.49723636	1.62809455	1.67994612	0.84703395	2.27154361	0.59284561	0.88115977	0.31431237
GAPVD1	1.67675241	2.12158049	2.73451538	2.37673484	2.73269876	2.44511574	2.19127322	1.8136797	2.22529753
GAR1	4.05259964	2.46814868	2.68535149	3.28406213	3.56916061	3.04982637	3.0431867	2.81436064	3.56142593
GARS	5.73869728	4.88420853	4.98476643	5.86284946	6.39795441	5.59156369	6.08860569	4.65052365	5.35885256

GART	2.32633052	3.00314478	2.93402113	3.62424721	4.31070643	3.91078522	3.05759206	3.61426864	3.585584
GAS1	1.84095394	3.81795676	1.17561233	5.23308875	1.30439702	3.52353858	2.68586239	1.02620752	0.5416256
GAS2L1	3.49100289	2.21890975	2.53957099	2.34934229	2.23226104	3.15214795	2.37140399	2.27157339	2.32887861
GAS2L2	0	0.01378637	0.11739455	0.01443673	0.02217607	0.03076938	0	0.04586763	0
GAS2L3	1.12534084	1.35374212	1.66277059	1.40939098	1.10982165	1.64537248	1.59537391	1.07712196	1.67677903
GAS6	1.83418915	4.31490592	3.68552059	3.50092447	1.57010213	3.89073478	4.25457635	3.67789357	5.02239038
GAS8	1.39923973	0.93916198	1.298307	1.77106995	1.28595858	1.53653921	1.6745474	1.96959292	1.81109307
GAST	0	0.08620494	0.15897477	0.36761668	0.13662968	0	0	0	0.79456581
GATA2	0.36617617	1.86838828	1.18454667	1.15511493	0.40553329	1.59711145	2.1479571	0.24295815	0.20398215
GATA4	4.8065561	1.01390758	1.7136032	2.75369927	3.46311412	2.94244084	4.64178567	3.3958848	4.31881593
GATA5	0.56267337	2.11771598	0.37338212	0.15181747	0.15567636	0.10927903	0.04143427	0.13428837	3.55593355
GATA6	5.08815582	3.59551316	4.63104795	4.01467646	4.53761392	4.11643012	3.1581713	5.30832573	4.877323
GATAD1	3.34913808	2.87210515	4.02766839	3.37874258	3.0929968	3.03034395	2.71400236	3.15401188	2.69831668
GATAD2A	4.33076453	2.9185074	3.50284295	3.02164906	3.91822203	3.85318663	3.61228647	3.51731559	4.23372424
GATAD2B	2.6569668	2.77558861	2.82264806	3.13299192	2.91631581	2.98250659	3.53507579	2.83965099	2.75784288
GATB	1.04214444	0.96084099	1.08247858	1.02878224	1.19903898	1.40296981	1.21389961	0.96460253	1.24556039
GATC	2.6212639	2.75054423	3.10196269	2.63609932	3.332888	2.71282123	2.28119861	2.75606492	3.45026735
GATM	4.86078056	2.35568909	6.03977926	3.05027351	5.93329853	2.8007496	3.8272128	5.79752671	1.16034987
GBA	3.66787636	4.17789356	4.50891595	3.72086815	3.7992604	4.34295891	4.73834434	4.74377554	3.78181582
GBA2	3.61968303	2.99318471	3.69324185	3.14633984	3.46227529	3.12693016	3.16235263	3.81963466	4.73277817
GBA3	0	1.14989959	0.76840379	0.07271141	0.51057694	0.07128303	0	0.18836358	0.70843866
GBE1	0.7392275	2.79217862	2.35183105	3.29517658	1.73394953	3.32837632	3.59494114	2.56512947	3.4359132
GBF1	3.73272679	4.27673062	3.57646132	3.96570745	3.96448249	4.34539423	3.94911793	3.73166673	4.41526097
GBGT1	1.23506164	1.75250442	1.50504502	1.45358011	1.30273241	1.90198595	1.35375185	0.79647223	0.49698904
GBP1	4.42523546	4.23646772	4.29209296	4.81590792	7.29731437	5.95052813	4.29340256	2.92194675	4.31635757
GBP2	4.18038325	4.37902008	3.71343808	3.59824088	5.16246614	4.84269431	3.85007246	3.54553563	4.56462222
GBP3	4.03068774	4.40470335	3.68178339	1.76636179	5.66412492	4.63587396	3.72602334	3.31635546	4.11198473
GBP4	3.9835064	3.41849375	3.86444436	3.38771704	7.75167574	5.24676425	3.26950492	1.67919054	4.56796326
GBP5	3.21025174	1.73227065	2.58340915	3.0787127	6.62375854	4.22219601	3.16158122	1.32845239	2.16659382
GC	0.01522595	0.14184205	0.85798273	0.28264397	0.14349356	0.29865442	0.00913363	0.38591637	0.63940426
GCA	2.99590233	2.94297499	3.9227841	3.13907615	4.35872007	4.17300428	1.95939669	2.86483568	2.96323503
GCAT	4.30145363	1.68150364	3.17232979	2.31506375	1.28387929	2.25853104	2.96993384	2.35703083	3.63567946
GCC1	3.1459298	2.40916316	2.42285038	2.26634038	2.50214266	2.68210354	2.60167676	2.48987886	2.28356347
GCC2	1.83265875	2.83527592	3.03178977	2.01267263	3.20338377	2.72452585	1.63200287	3.02332225	2.97217699
GCDH	2.60880573	1.66087701	2.14972727	1.84721605	1.93999274	2.13451128	2.10851352	1.94072672	2.51586107
GCGR	0.03334655	0	0.08670623	0	0.06766214	0.01234203	0.07859541	0.04690363	0.03503577
GCHFR	2.9903307	1.11270398	1.79393385	1.51155892	1.26468233	1.16920716	1.53917231	0.89416221	1.87629547
GCLC	2.85510927	2.37877114	2.98279846	2.45906449	2.52903503	2.3750011	2.75105716	3.17715061	2.51630252
GCNT1	1.84456524	3.42360204	3.95187975	1.87892998	3.02065702	2.8200603	3.78545461	2.53296228	4.15613333
GCNT2	1.08533968	0.80974978	0.5772018	0.3984819	0.73878843	1.75785759	0.14436572	1.12143683	1.23957861
GCNT3	1.33877145	3.32214448	4.79523313	3.52418653	4.54190932	0.59895245	2.83257805	1.31274561	3.68567046
GCSAML	0	0.87075339	0.30332199	0.20844893	0.0610554	0.16985622	0.0231216	0.03264343	0.01222805
GCSH	1.42925329	1.89112976	1.26178429	2.22531053	1.52974331	2.19359942	2.63697111	1.59444287	2.08951763
GDAP1	0.47795307	1.59342188	1.0193007	2.96069743	0.75158006	2.33624807	1.32421802	2.38056312	1.02308668
GDAP1L1	0	0.1740756	0.6271901	0.0256093	0.03278805	0.08077004	0.04442365	0.0609396	0.04457874
GDAP2	0.96219905	1.54207568	1.67553997	1.65802769	2.15002615	1.78714432	1.69292648	1.97398496	1.75957357
GDE1	4.60184091	4.98025471	4.87636829	5.31292757	4.12780649	4.86333989	5.91406268	4.1138063	4.69974
GDF10	0.1809187	1.50121585	0.04222405	0.06096277	0.05378942	0.2357003	0.07774714	0.07786551	0.00777269
GDF11	1.47206448	1.49324411	1.50075756	1.5366862	1.00093374	1.23651614	1.02438332	0.57566205	1.36900764
GDF15	3.89213315	4.74036848	7.0738042	4.33790134	4.52059712	5.8033659	7.11081658	6.58058526	5.61054021
GDI2	5.64100067	6.03916241	5.92937594	6.30166149	6.43941703	6.40978938	6.18615511	5.48123499	6.78261466
GDPD1	1.59255724	1.0407014	2.6686721	0.97788089	2.96468474	0.79963719	0.53917898	2.69845191	0.97832736
GDPD5	1.18098198	1.24202636	1.31983669	1.16519843	0.53241669	1.0804603	1.05264391	0.62731921	1.34990667
GDPGP1	1.03204489	0.59059735	1.2682875	1.02022193	0.70270732	0.8101532	0.75557721	1.28731865	1.00968254
GEM	2.05579099	5.42809148	3.70266398	5.08778582	2.91590062	5.3797461	3.71724043	2.71195382	2.63927016
GEMIN2	1.75894234	1.84120522	2.16164674	2.39884947	2.51143878	2.03218702	1.9754064	1.58290522	1.85056087
GEMIN4	2.14033415	1.9507662	2.73721546	2.28353601	3.0419254	3.17667853	2.85591444	2.27995407	3.29198806
GEMIN5	2.17422343	2.28876018	2.22388471	2.83229332	2.83757544	3.01733906	2.77400052	2.92069206	2.90535565
GEMIN6	1.64427188	1.64776919	1.98809975	1.63346593	2.25133128	1.76043713	1.5309664	1.69568121	1.9113694

GEMIN7	3.59149717	2.28354011	3.1289833	2.40745817	3.01403606	2.72311425	2.54024324	2.58810568	3.01798543
GEMIN8	2.61527018	2.9808821	2.50859474	2.66581026	2.55090398	2.62778605	1.931118	2.58995942	3.87454439
GEN1	1.14474867	0.82239107	1.33782643	1.50207509	2.22500085	1.21949517	1.41605734	1.13930088	1.57880726
GET4	2.06227907	0.58929747	1.14076256	0.60407205	0.61180698	0.79209769	0.81558941	1.06892498	0.77542984
GFI1	1.00180949	0.6706325	1.50644832	0.77151524	2.34826655	2.56712245	0.90333621	0.37945591	0.86122604
GFM1	2.77044299	2.70183996	3.72166659	3.35839363	3.52010974	3.19915415	3.6365427	4.11343799	3.49580254
GFM2	2.26965019	2.68792911	4.12482544	3.50593323	3.71669273	2.76898578	2.81603455	3.08983076	3.33253597
GFOD1	0.38661257	1.54200345	0.78731399	0.97931783	1.56712164	0.98469766	0.85554346	0.25478781	0.51595667
GFOD2	1.7996353	1.21280022	1.61941377	1.65975981	1.39946864	1.66549682	1.69686083	1.71259877	1.83417985
GFPT1	3.58543396	3.98612837	4.69485711	3.52947843	3.76286203	4.58478464	5.6600565	5.44193172	4.71487731
GFPT2	1.7080606	3.39096872	1.22425261	3.10116728	0.72630688	2.95340673	1.74710893	1.03061256	0.49401156
GFRA1	0.01125326	2.84566105	1.66129208	0.69751481	0.07899277	2.68097782	0.11269971	0.46811185	0.04290294
GFRA2	0.01269588	0.55665368	0.38924925	0.29634358	0.08021334	0.79001566	0.07075333	0.16251267	0.09091457
GFRA3	0.03531217	2.42341287	0.79407386	0.62537335	0.03266086	1.67541481	0.15217609	0.09980158	0.03709969
GGA1	3.47743427	1.83152969	2.53455532	2.63358226	2.68480769	2.52041142	2.46194507	1.93490776	2.74239381
GGA2	3.0078366	2.95463937	3.40219094	3.07359453	3.62221846	3.18371131	3.25941544	2.95323547	2.8381949
GGA3	3.44766658	2.49166398	3.41058649	2.96930229	3.13825662	2.85988539	2.37647376	3.28185766	3.09375419
GGCX	1.6175734	2.36353442	2.42197731	2.22286772	1.91561791	2.35946158	2.82611288	2.46000545	1.73713937
GGH	0.87286524	2.07325112	3.56127864	4.5155087	3.16192075	3.7243758	4.90326485	3.22241221	3.16532377
GGNBP2	2.91320362	2.54404516	2.82599924	3.06710422	3.1909728	2.86041347	2.50865352	2.71901403	2.9583455
GGPS1	2.60411865	3.69247698	3.15941019	3.38489054	3.43588852	3.50604686	4.01378164	3.86877906	3.47147584
GGT5	2.39630547	4.28343054	2.46832801	3.17329834	1.5675095	3.7357366	2.63527958	1.779591	1.28359372
GGT6	0.22382463	3.39212265	3.26171928	0.91243304	0.14836271	0.12179765	1.83355997	0.2408994	5.06884439
GGT7	3.20594296	2.41208454	3.16288602	2.88705224	0.71380331	1.76297505	2.91382423	2.26540257	2.28466884
GHDC	4.33331139	2.92863194	2.8024153	2.40366942	2.38209678	3.09000506	2.39794808	3.54701117	3.29788276
GHITM	6.15674644	5.99400276	5.75229092	6.15299102	6.62014347	6.3296206	6.55508049	6.33328349	6.54053802
GHR	0.13667088	2.19646268	0.54432391	1.18285348	0.29951103	1.16343713	0.36285109	0.30185704	0.11418423
GHRL	0.5019568	0.34073657	1.63435283	0.57011003	1.01284324	0.45759206	0.31240172	0.6252535	0.34505796
GID4	0.76401715	2.42532967	1.87412189	2.01432311	1.32162704	2.37279916	1.57224584	1.42727447	2.02452611
GID8	3.90977689	3.46997844	4.69730636	4.13766657	3.54098794	3.82860003	4.33312503	3.69421276	4.18016155
GIGYF1	3.13161393	1.6386077	2.82040308	2.44394528	2.2795321	2.21237866	1.86369538	2.77609318	2.89344826
GIGYF2	2.57979146	2.47819346	2.29242904	2.17852044	2.76033788	2.74893596	2.52831189	2.96053159	2.72376594
GIMAP1	1.0810403	2.11015767	1.36836173	1.25239113	1.79073534	1.86063513	0.89868914	0.5520073	0.65322496
GIMAP4	3.77063248	5.08062883	4.62799562	4.01891921	5.55846153	4.45367968	3.93101955	2.74334218	3.08060562
GIMAP6	2.26765702	3.85403174	3.6483496	2.96086074	3.9056774	3.55313352	2.6593678	1.76072449	2.15306817
GIMAP7	2.89454767	4.46409314	4.0152582	3.19299851	5.17202261	3.93401643	2.83597006	1.91950305	2.48785039
GIMAP8	1.29222058	3.09060486	2.47237694	1.9424376	2.9171889	2.48166201	1.47601917	1.23532393	1.40442241
GIN1	0.60931133	1.09416393	1.11450892	1.37551016	1.48981678	1.33614658	1.04732834	0.74430468	1.19678841
GINS2	2.71472894	2.15204655	2.49736509	2.65064874	3.56003612	2.4066642	3.2175678	2.30726362	3.71090694
GINS3	2.12595877	1.1460989	1.39125821	1.76880977	2.4383584	1.76860777	1.61431493	1.48858672	1.80183318
GINS4	1.46359058	0.77923994	0.96103042	1.45189084	1.4791735	1.59654405	1.69843454	0.9499802	1.02545527
GIP	0	1.50540832	2.17555053	0.05981897	1.03088953	0.22468617	0	0.18394101	0.07811269
GIPC1	5.68620098	3.25623065	4.61227273	4.01124451	4.10141306	4.01623459	3.91159418	5.36456555	4.07676308
GIPR	0.15317437	0.08971677	0.63325865	0.54361621	0.55571069	0.05546847	0.21368639	0.56262151	0.44835741
GIT1	4.99676107	3.08932685	3.73696749	3.50456869	3.3851353	3.8425029	3.82567613	3.72362783	4.22853363
GIT2	1.3798476	2.33954847	2.31725191	2.00381448	2.53494932	2.47561509	1.81249246	2.16529918	1.97339736
GJA1	7.0396585	5.42384358	4.58881608	5.71920588	3.31573585	5.23388092	4.11410231	3.17536934	2.56742783
GJA4	2.37250908	3.14501428	3.52911392	3.09691788	1.96025576	3.49523394	2.21329696	1.93719925	1.43369669
GJA5	0.77059877	1.67903221	2.01959837	2.63067491	1.04849624	2.46825091	1.64468343	1.27479287	1.15719742
GJB1	5.29789	4.10746625	3.94451788	3.81497027	3.35514937	2.21606545	2.0541245	4.58347205	3.36594475
GJB2	5.98735081	3.27689339	4.74823317	4.70445789	4.72343881	4.51933587	4.50905803	4.78730425	4.6637232
GJB6	0.59128241	0.03673967	0.20730389	0.95516561	0	0.31022624	0.01931414	0.01865773	0.00851175
GJC1	0.61713072	1.54010364	1.52282689	1.020813	0.92623788	1.20370276	0.66921259	0.65284411	0.56552628
GJC2	0.37052617	1.17089903	0.38867994	1.22520258	0.12458781	0.73658937	0.36725542	1.14474968	0.17662839
GK	2.64197203	2.33941258	2.4935508	2.52529312	3.07624004	2.25572324	2.15321891	3.3083143	2.6191549
GK2	0	0.01070971	0	0.01121549	0	0	0	0.00721499	0
GK5	2.62758831	1.5422962	2.12664787	1.63017257	1.63487675	1.60364898	1.92022166	1.31772575	2.08384338
GKAP1	0.9681822	1.0525698	1.19542449	1.1057094	0.96554037	0.774859	0.70543364	0.72569656	1.16164731
GKN1	0	0.36697919	9.1423269	6.4773458	2.11047526	0.02312217	4.39893972	3.13125685	1.88466857

GKN2	0	0.28551212	5.8887892	4.23520503	0.96412021	0	2.78479266	1.78178354	0.4953692
GLA	4.43953733	3.81223382	4.87715127	3.9801719	5.08623207	4.275431	3.76815278	3.6782429	3.98852154
GLB1	5.14952925	4.5466659	5.27992039	4.85377111	4.87389694	4.83840253	5.63665002	4.93206831	4.74952177
GLB1L2	2.39538661	2.52466352	1.27437321	2.13055297	1.29365604	1.69490692	3.31175948	2.04083487	2.58264775
GLCCI1	0.95279528	1.50107771	2.19489286	1.67368679	2.36455863	1.59879585	1.60738517	0.77629581	1.36495148
GLCE	1.94772635	3.80589064	3.0636437	2.83177891	2.86465319	2.48621654	2.30753773	1.7666032	4.210888
GLDC	0.12728418	0.18095639	0.45255559	0.24554771	0.66136778	0.1265777	0.12557011	2.04556614	1.15096126
GLDN	0.1175166	1.04641573	0.49963751	0.40323092	0.21290723	0.3001398	1.57489059	0.12245643	0.07319421
GLE1	3.36612595	3.14405922	4.20988248	3.52813604	3.80221507	3.64205432	3.94620563	2.67423976	4.13217072
GLG1	3.47718472	4.06746977	3.92819101	4.0057185	3.7593273	4.08048092	4.806516	3.61562642	3.5521633
GLI1	0.83768129	2.01407936	0.95111773	0.72781714	0.1662771	0.87716999	0.92543106	0.48372358	0.2426882
GLI2	0.69670473	2.19268585	0.62494027	1.74474512	0.4271933	1.4820386	1.18788648	1.31204714	0.2202684
GLI3	0.4817805	2.47079013	0.83689821	1.90221807	0.64394371	2.08471906	1.07816206	0.59661383	0.35805368
GLIPR1	1.51219947	3.88491704	2.9926369	3.24162807	2.90063544	3.51623728	2.3628499	1.65021493	1.58716396
GLIPR2	3.07476929	3.66395673	3.1309729	2.86897426	3.90330676	3.75966371	2.67925368	1.70751449	2.79256384
GLIS1	0.10169608	0.77498873	0.45349249	0.81116777	0.1374809	0.57432718	0.36900079	0.10861305	0.08367903
GLIS2	2.97288009	3.30896142	2.31664822	2.88543599	1.7017868	2.84624892	2.00414359	2.40735405	0.84502347
GLMN	0.99250343	1.81600264	1.92015361	2.65102482	2.86043869	2.11434715	1.69816871	1.63281827	2.18153832
GLMP	3.77303527	3.99542124	4.7311456	3.92098557	3.59011358	4.21105655	4.39120401	4.83817594	3.60985826
GLO1	4.88185455	5.45145964	5.51103551	6.77726542	6.04876538	5.8535709	6.37793942	6.27172612	5.83243362
GLOD4	2.89059206	2.95673203	3.65638296	3.68192352	3.87706296	3.47142033	3.58568679	2.63410681	4.02533243
GLP1R	0.01169002	0.11620958	0.05817067	0.06202523	0.02153097	0.05500223	0.01398429	0.00451679	0
GLRX	3.33441421	3.57305108	3.94225529	3.11311356	3.68247493	3.10748322	2.20502742	1.94765788	3.13853943
GLRX2	2.54173024	2.77570535	2.85560976	2.96601406	3.31458484	3.0335394	3.77124097	3.24559571	2.71387585
GLRX3	2.88507852	3.27078661	3.38857985	3.86414621	4.1392454	3.79112071	3.69489379	4.06721006	3.75633664
GLRX5	4.94439869	4.11996533	4.03583364	4.2976717	4.44316903	3.99299884	4.13396838	4.28849741	4.51868358
GLS	2.55559118	3.01717307	3.51311136	3.80176266	3.70280406	3.2031554	2.9291949	2.18144754	4.2124849
GLT8D1	3.16667734	3.55582106	3.81531407	3.84360667	3.01820648	3.48407618	3.90707977	3.59115576	3.59267057
GLT8D2	1.12539267	4.17171586	2.4703381	3.90799237	1.21575952	3.19190761	2.48301182	1.74480371	1.19336132
GLTP	4.20751732	3.69816538	3.74216371	4.17889319	3.84080634	3.88302438	3.54867553	3.90980571	3.60414696
GLUD1	5.63288179	5.0490193	4.8437659	5.41191166	5.51136856	5.3714735	5.47505178	5.75896928	5.27916801
GLYCTK	2.35283728	1.15356788	2.4987627	1.7545635	1.07757604	1.47537767	1.93569221	1.81314329	3.57486565
GM2A	5.90092134	4.33717016	5.00387133	4.58533906	5.69248017	4.95536984	4.73690569	4.22085325	3.92322976
GMCL1	3.43608627	2.53843314	3.28771549	3.19775935	3.15862166	2.93281775	3.30075957	3.1155731	3.21110854
GMDS	6.28007527	4.17246389	5.46074121	4.01336145	4.52436876	4.49893028	5.38366267	6.39935306	5.59012265
GMEB1	1.77605137	1.47095418	1.93569119	1.95486053	1.92039488	1.78303824	1.63555969	1.49386793	2.07492887
GMEB2	2.85310463	1.81966893	2.90135547	2.0881639	2.35228079	2.26890415	2.32184597	2.58783173	2.64318213
GMFB	2.65513733	3.35003282	3.44166061	3.79458525	4.06473038	3.31599779	3.12250288	2.78884357	3.91187745
GMIP	4.06155083	2.72211117	3.77942031	2.75582432	3.7014139	3.53259602	3.26425322	3.72606187	3.19180244
GMNN	3.03436668	2.36273287	4.05157487	4.11172603	4.12295398	3.66884209	3.57382763	3.24339027	3.43269058
GMPPA	3.55831908	2.84516298	3.44861792	2.9630227	3.06690997	3.45129285	3.1688805	3.82383191	3.41202404
GMPR	1.16670091	2.30498363	2.21879165	1.85098705	1.22570298	2.52017334	0.72281631	2.43334202	1.15414541
GMPR2	3.7993301	3.48651193	3.74787084	3.21890057	3.4910534	3.5370092	3.31447306	3.8152914	3.86929001
GNA11	4.50772351	4.03128806	3.52166729	3.41068288	4.2751662	3.85564107	3.98513148	3.72664749	4.61759354
GNA12	3.76355272	3.91702852	3.98051919	3.6179782	3.32034272	3.87845413	3.69726643	3.31384028	2.93833958
GNA13	4.20296056	5.08093138	5.12581674	4.9291066	5.35100645	5.37612337	4.9576711	4.97275497	4.39791577
GNA14	0.86890375	2.6787351	1.85701163	1.11749272	0.4739582	2.35536987	0.71779283	1.14368236	1.65407027
GNA15	3.68615831	2.19799102	3.6410398	1.92436402	1.87271455	3.0224715	3.33342858	2.00653601	1.39294716
GNAI1	0.83009028	3.00477179	3.98393978	1.90041189	1.55610241	2.0629103	1.37461959	3.3348121	1.51759653
GNAI2	6.44604203	5.84360707	5.77075599	5.59490737	5.51814499	6.17387786	5.75735394	4.79891855	4.41990927
GNAI3	1.8676755	2.29355316	2.39379863	2.86550595	2.9829635	2.77969869	2.58776537	2.36371284	2.53507558
GNAL	0.15919205	1.61412199	0.64728027	1.10338565	0.41924716	1.12532772	0.3165112	0.58642191	0.4799134
GNAO1	0.30063694	2.98337284	0.53613447	0.57192822	0.2261262	1.98052104	0.11941714	0.15973061	0.16579472
GNAQ	3.81074125	4.2114664	4.24123012	4.1126223	3.95146433	4.24946008	4.04343921	4.71210933	3.5695865
GNAS	6.47721952	5.76566656	6.33390763	6.55763421	5.2505949	6.01103388	6.37189009	6.05606959	6.06595441
GNAT1	0	0	0.11922394	0	0.00812944	0	0	0.02025885	0.02071852
GNB1	6.23843929	5.69824182	5.41136186	6.00243949	5.64450192	5.95971067	5.8670283	4.98636885	5.94792267
GNB1L	1.15880235	0.49356876	0.7227424	0.42044509	0.69519883	0.53764503	0.55327921	0.37956825	0.70563446
GNB2	7.69202092	5.66117417	6.19473254	5.20732923	5.40647441	6.05757204	6.56109665	5.78032807	5.84472812

GNB3	0.0314655	0.12779274	0.34575119	0.82870932	0.22346144	0.18272614	0.27117051	0.07563312	0.30077258
GNB4	1.84934914	3.80833671	3.31322111	3.21299537	3.90417537	3.52465791	3.08813238	1.90262013	1.69955241
GNG10	2.8717779	2.94417007	3.20947224	3.011608	3.21851019	3.30135624	3.23851115	2.19692847	2.15844802
GNG11	2.06757845	4.78956252	3.46731259	3.63520038	2.60135834	3.93238553	2.59074707	1.83154621	1.73351368
GNG12	3.71811816	6.06059845	5.38667292	5.57173039	4.38663105	5.4218289	5.42641008	5.23928875	5.47110627
GNG2	1.21579427	2.99962133	2.60436197	2.58983362	2.97053081	3.02087973	1.873621	1.52168568	1.39614361
GNG3	0.15648158	0.19868515	0.14203652	0.14156549	0.3587446	0.25026196	0.24925609	0.22105362	0.29375281
GNG5	7.43324522	6.3587446	6.39453186	6.46561876	7.11140545	6.427462	6.60869033	6.45074229	6.54646003
GNG7	0.6512052	1.16583478	1.11131322	1.24633077	1.11791428	0.90380103	0.32802123	0.73456171	1.06269584
GNGT2	1.27553063	1.37489307	1.83662304	1.03553723	2.15549699	1.31252458	1.32945479	0.59745709	0.77856136
GNL1	3.03034522	2.23121559	2.76702365	2.27207476	1.62158788	2.47461863	2.37735709	2.68913339	2.09931854
GNL2	2.82758944	2.77098461	3.82097905	3.79403268	3.7281498	3.42976453	3.19820363	2.76181827	3.37788606
GNL3	3.82180903	3.77004866	4.95238903	5.08910814	4.25831339	4.36250635	4.84415103	4.64422555	5.53138605
GNL3L	4.15397709	3.09793102	2.84584844	3.06626064	4.05140239	3.02210532	3.04921763	3.7000547	3.2701073
GNPAT	3.98768939	4.06345255	4.37876406	4.12043773	4.34268387	4.28555246	4.60287797	5.01326987	4.67113099
GNPDA1	4.42610681	3.91412009	4.60837767	4.02420467	4.63005006	4.5992168	3.87227469	4.17287176	3.81152087
GNPNAT1	3.59726986	2.83655349	3.66050449	3.79847508	4.49716884	4.15955106	3.75491939	3.84567901	4.10404497
GNPTAB	1.83731338	3.11146007	3.8023879	3.39642677	3.41666747	3.7169059	3.11566552	3.69034346	2.85275371
GNPTG	3.80548746	3.9096722	3.49937495	3.58087558	3.41504121	3.35373249	3.63338016	3.49871705	3.35777331
GNRH1	0.15664989	0.0806377	0.35576459	1.27456235	0.7785511	0.45839085	0.35007854	0.33309188	0.58243895
GNS	4.67025919	5.97530479	6.19973152	5.64395659	6.10327865	5.81173978	5.49566099	5.6943585	5.19072277
GOLGA1	1.79228775	2.49009564	2.87999884	2.41371935	2.53702711	2.5792443	2.16982853	2.1081026	2.55470544
GOLGA2	3.90592228	3.01065339	3.69053174	3.09825194	2.85280425	3.29949548	3.41449663	3.10841213	2.86349598
GOLGA3	3.12785466	2.49533541	2.7769554	2.76633679	2.51572456	2.83343693	2.94207012	3.55839845	3.0917543
GOLGA4	2.73772629	3.58928844	3.50764429	3.59429999	4.57801694	3.50574489	3.72055414	4.22545096	3.85513508
GOLGA6A	0	0.01186301	0.10151348	0	0	0.00381541	0	0	0
GOLGA7	4.64360072	4.94702737	4.32533979	5.15975463	4.57113381	4.90222667	5.19079866	4.36943249	4.57158435
GOLGA7B	0.45587135	0.37629232	0.20135874	0.36587361	0.09420046	0.28335767	0.0724686	0.1988074	1.30338804
GOLGA8A	0.22454845	0.51257457	1.49136308	2.09016248	1.28744577	1.12329271	0.92133526	0.25493861	1.47115297
GOLGB1	4.00033708	3.83711668	3.6085765	3.37088811	4.11320049	3.43301026	3.80825243	4.34005086	4.02300015
GOLIM4	6.54582348	4.40530919	4.7202367	3.88489712	3.92625223	3.87420062	4.6013009	4.90747527	4.98042917
GOLM1	5.99384663	5.94503178	8.13492684	5.24534443	6.38197128	5.54394	8.10270826	5.64972964	7.2384975
GOLPH3	6.2340102	5.65818206	6.07615336	5.66147974	5.5724763	5.67415988	5.46682711	5.14442208	5.57984245
GOLPH3L	3.65304517	3.61049604	4.07227489	4.04615311	3.86547716	3.94571317	4.26826469	4.24693893	4.82734365
GOLT1A	0.15210183	3.0033154	1.40858547	2.60480708	0.18522237	1.76990224	1.8831963	3.19794298	4.18355606
GON4L	1.71083033	1.72607519	2.05762946	1.87417974	2.21126667	1.8944255	2.09986473	2.39685175	2.10840234
GOPC	2.56688941	3.34353342	2.78206751	3.5983794	3.43106693	3.4205838	3.0878128	2.84996441	3.29709853
GORASP1	3.3712756	2.77453227	3.26131054	2.87863388	2.86367245	2.93400923	3.09735637	3.07284885	3.27168982
GORASP2	4.77170919	4.54282141	5.67420076	4.9791895	4.87987642	5.02716685	5.30465034	5.26957047	5.10319534
GOSR1	3.17766981	2.73670752	2.66336248	2.91992354	3.07717175	2.99730147	2.86323406	3.58339349	3.11152858
GOSR2	2.09566292	1.79363074	2.25010653	1.96744107	2.19091016	2.22359008	1.73388058	2.16410419	2.11321177
GOT1	4.89192449	4.02200503	3.42224935	4.63788988	4.86075547	4.63525772	4.52998048	5.04530171	5.29913071
GOT2	4.88599831	5.38665107	4.97358127	5.27907824	5.47160452	5.57455909	5.38819981	4.872019	5.51616301
GP2	0	0.05564041	0.95440468	3.12684193	2.41382303	0.04237528	0.19621601	0.20053478	0.30205412
GP9	0	0.0232931	0.11385731	0	0.02503647	0	0.02433375	0.21943893	0.02138939
GPA33	1.82051018	3.67049973	1.74501372	1.50697125	3.04529594	0.97176962	2.75286617	0.33271039	6.18779876
GPAA1	5.07492228	4.5655325	5.2002259	4.55100517	3.69941754	4.76813169	5.59186559	5.49989757	4.62267069
GPALPP1	1.5482243	1.61885953	1.38364141	1.52614942	1.69114751	1.33022773	1.26908479	1.55347867	2.44123519
GPANK1	2.29348585	1.91083655	2.67822951	1.74779377	1.4343905	2.01763163	1.86745744	2.64171941	2.16154538
GPAT2	0.09910625	0.51367411	0.26192133	0.19407005	0.15703398	0.3348808	0.0928981	0.18310219	0.18035001
GPATCH1	1.30726894	1.57043502	1.83100758	1.67430881	2.21763977	1.22532105	1.49542388	2.04403344	2.00429404
GPATCH11	1.86838268	1.94925211	2.68177854	2.15909717	2.5821743	1.99768541	2.07048729	2.5451912	1.93234503
GPATCH2	1.14574817	1.37605152	1.75182851	1.77008445	1.69096461	1.45921368	1.70705382	2.02886988	1.6784354
GPATCH2L	0.91344217	1.50960357	1.3787143	1.63626823	1.56726637	1.66632637	1.02264654	1.37969689	1.43104808
GPATCH3	2.53669096	1.68308066	2.18900214	1.61217193	1.67320162	2.05729278	1.79371477	1.19590784	1.95475868
GPATCH4	3.16248616	2.28229365	3.10594192	2.8167983	3.35691784	2.57426989	3.43182623	3.53753447	3.42507752
GPATCH8	3.08188796	2.75585902	2.68602387	3.13187931	3.51264849	2.99860406	2.70923642	3.31239133	3.28313212
GPBAR1	1.61564209	3.1303269	1.0923363	0.54203872	1.16164466	1.36108991	0.85510751	1.03482561	0.50514816
GPBP1	3.09243345	3.46155993	3.937842	3.89049283	3.9745953	3.79075353	3.40194388	3.93083423	3.60274797

GPBP1L1	3.80871483	3.53917598	4.58152107	4.10620702	4.40284174	3.73076591	3.58961276	3.93236918	4.60470675
GPC1	3.58992432	3.03043231	2.97081467	3.68142683	1.62191887	3.5873124	3.78743842	2.90830365	1.55181752
GPC2	0.21210948	0.1252812	0.36295814	0.29823021	0.1816215	0.06831845	0.02261818	0.06106949	0.44807859
GPC3	0.99112357	2.85907865	4.56479737	4.30545872	0.48731176	1.22146306	0.85503479	2.0629563	0.91574223
GPC4	5.93894476	3.36422041	3.6214425	2.78604465	4.22609877	2.84342435	2.97279341	2.60876576	3.32585231
GPC6	1.52537538	4.37951293	2.45587934	3.76820996	1.71410615	4.06677722	2.61740683	1.6824837	0.92226442
GPCPD1	2.02635642	2.56760265	3.217884	3.35110202	3.35466992	2.40953359	2.02166182	1.77249372	3.08816756
GPD1	5.20041722	2.47347292	2.95157914	1.02625033	0.31690941	1.54875762	3.09744448	2.50566669	3.07998479
GPD1L	2.21096864	3.32290412	2.55675143	3.07708672	3.33445379	3.18751438	3.80110822	3.21263777	3.86426686
GPHN	1.86599186	2.20804376	1.84463027	2.33108071	2.22533622	2.39378674	2.31540327	2.20007023	2.29648457
GPI	6.35486633	4.71018428	4.92822727	5.18763897	5.6775447	5.61443244	5.88619985	5.43385775	6.10586798
GPKOW	4.00603981	3.48761123	3.55577995	3.12097581	3.9085059	3.5910443	3.49765512	3.82855611	3.49800548
GPM6B	0.33134004	2.0168398	0.67351549	1.73731656	1.20324667	1.62005795	0.55742554	0.25149278	1.07661135
GPN1	3.41827481	3.17174464	4.04293525	3.767135	3.93657634	3.79719499	3.79087931	3.59394238	3.75928352
GPN2	3.60551637	2.00702499	2.94565292	2.23777305	2.1448692	2.18330662	2.07494102	1.84596416	2.89696272
GPN3	2.63641248	2.60402275	3.18952676	2.74043927	3.50777902	3.00446282	2.81378002	3.15650385	2.92810616
GPNMB	5.0282284	6.43535964	6.02399486	6.41699477	7.20532761	6.16617176	5.95378558	4.62673228	4.58059669
GPR107	3.90427835	3.2342796	4.45894155	3.38901199	3.69608227	3.93506695	3.84278119	2.88475362	3.10488696
GPR108	4.59657296	3.84644664	4.60593885	3.91821076	4.22408908	3.94924669	4.14324946	4.83043443	4.11059182
GPR132	1.36504877	1.2159651	1.85436471	1.06085939	1.99108786	1.8116712	1.16719705	1.05355417	0.76443276
GPR135	0.15272241	0.80503598	0.26064932	0.38688185	0.46947147	0.74424959	0.14182976	0.18018167	0.25026557
GPR137	3.49859835	2.51520865	2.82938143	2.20223795	2.63861417	3.03849048	2.63125849	3.35222654	2.4567673
GPR137B	3.11773891	3.93256707	4.54991678	4.19602047	4.60725942	3.96718065	4.5110804	2.92182823	3.44662736
GPR146	0.40028505	0.55293381	0.5873363	0.32676432	0.13552918	0.58149234	0.24687016	0.59594997	0.16589219
GPR153	2.77832869	3.4430527	1.45611832	1.85353936	0.85761985	2.29266399	1.88919834	1.21351111	2.24614456
GPR155	0.8321895	2.29500565	1.72136405	1.35761178	2.07210842	2.1947702	1.34572146	1.02070099	1.25983182
GPR157	2.5246172	0.82404493	1.61136369	1.43512338	1.99939537	1.41445877	0.9541013	1.16096968	1.42400538
GPR162	0.20580996	0.87874142	0.69137038	0.5994731	0.28363548	0.70823629	0.49880125	0.1687774	0.21561118
GPR173	0.05432757	0.84859194	0.51773928	0.65889397	0.14364732	0.71239487	0.36233301	0.1085958	0.14615976
GPR176	1.00356736	1.90409192	1.64208882	2.2948999	1.2179725	1.79971873	2.25269101	1.681742	0.938588
GPR18	0.50962156	1.03854019	1.4496003	0.53880733	2.47492905	0.64102196	0.39727637	0.45681991	0.68923253
GPR180	1.1012444	1.57914563	2.03745786	2.53648086	1.91032991	2.04688802	1.86314725	1.45789495	1.7018687
GPR183	2.33820632	5.07074653	5.086613	4.77881455	3.85914974	4.64991428	3.55511418	3.36793094	3.10826025
GPR20	0.2224881	0.66231124	0.53179332	0.35626701	0.15444544	0.65617245	0.34425778	0.24655737	0.14427644
GPR21	0	0.03525067	0	0	0.01906297	0	0.01852683	0.02381368	0
GPR27	0.10066128	0.63221103	0.35999132	0.42958448	0.14810865	1.27418509	0.04390255	2.59580102	0.05378571
GPR3	0.33659154	0.66755041	1.17071078	1.85421899	0.53490046	1.54270224	1.22024437	0.44262166	0.93383416
GPR34	1.26422006	4.35373187	4.90708905	3.20516651	3.97775078	3.66274636	3.37034969	2.27891787	2.98955427
GPR37L1	0.06490225	0.48516398	0.2723474	0.22680379	0.03700885	0.08101813	0.2149641	0.20256849	0.28652146
GPR4	2.10746219	2.31208188	3.10953276	2.32742531	2.16305915	2.55688172	2.294037	1.71712898	1.7896296
GPR52	0.09714621	0.30963203	0.04760674	0	0.08026797	0.23774853	0.01991356	0.01285306	0.0175008
GPR55	0.03487289	0.13501068	0.31388113	0.0672343	0.13483994	0.09716465	0.03651064	0.07617859	0.11582359
GPR63	0.16746454	0.91450274	0.55497624	1.469739	0.1194	0.6283543	0.56824545	0.5886424	0.76596937
GPR65	0.99594037	2.27592014	2.17354019	1.66379191	2.52595592	2.17964599	1.40159272	0.91780216	1.06106835
GPR68	1.97112679	2.58846404	1.82327327	2.40091603	1.57056678	2.52246546	2.0645568	1.63638272	1.21698824
GPR75	0.2377609	0.75787415	1.09696815	0.49411579	1.01633339	0.60619059	0.33043518	0.57334481	0.31597395
GPR84	1.32684315	1.23870866	2.82629007	2.45632476	1.78457945	2.56898077	1.30190974	1.65504976	1.13908264
GPRC5A	5.37043891	5.09737712	6.1927972	7.3470856	5.97509032	5.51993259	6.38787555	8.35752211	4.7760866
GPRC5B	5.3076771	2.56618439	4.88182416	2.03987935	4.9835307	3.15684578	1.72836554	3.7640038	0.74431433
GPRIN1	0.8171083	0.76053167	1.01637738	2.14259948	0.67020717	1.75063517	0.76879099	2.11957156	1.03343489
GPRIN2	0.05451334	1.14131773	2.5230936	1.33618787	0.9973775	1.5103543	0.67052892	1.50358483	0.57123871
GPRIN3	0.82583052	2.35445141	2.28882743	1.85062266	2.84765926	2.07601342	3.35980618	1.03663574	2.71452749
GPS1	4.72477633	3.35932573	4.17509097	3.22495271	3.4397102	3.77661849	3.62564505	4.54311016	3.90035256
GPS2	1.07874833	1.25493325	1.66909516	1.67941483	1.35997723	1.45405006	0.96325429	1.14838243	1.83616116
GPSM1	0.807517	1.6706762	0.81820311	1.64521819	0.44857606	1.43833996	1.10752506	1.99131242	0.60700345
GPSM2	1.97181463	2.35058439	2.19273323	2.51258059	2.79600671	2.59461018	2.58544764	1.94092747	3.11242926
GPSM3	4.28565837	4.14584285	4.66413423	3.85273795	4.46118492	4.86866992	3.71820506	3.2037369	3.20509054
GPT	0.82370935	0.89611906	0.50303858	0.74063202	0.21176943	1.01947927	0.79056671	3.28184535	1.0119285
GPT2	3.08039333	2.26327301	3.51973802	2.12775417	3.03531499	1.80589306	3.52386936	4.40624117	3.01513604

GPX1	7.72207031	7.36367572	8.02530839	7.40922516	6.92612604	7.73855105	7.50143103	7.81126896	7.25764397
GPX2	8.29283563	7.33505339	7.88943602	5.64550095	7.91305198	4.86963867	5.22563924	8.13950386	8.11662569
GPX3	4.15799743	5.86878074	4.56888095	5.33068101	4.4600138	5.55821064	4.77911266	2.78871869	2.09149717
GPX4	7.26358734	6.30986324	6.90781127	6.69395037	6.13454484	6.58835832	6.53141792	6.09495541	6.65246653
GPX7	1.73572681	4.04036154	2.50966331	3.26366327	2.20976272	3.18873061	2.41364383	1.52168976	1.28974681
GPX8	2.21545225	4.47125928	4.19320484	4.17630607	2.80793955	3.98105419	4.2026342	3.66013669	1.58067048
GRAMD1A	3.96458404	2.87266804	3.9303068	3.58599252	3.79334528	3.54033356	2.54732542	4.07396356	2.64110712
GRAMD4	3.08366897	3.10197845	2.36583121	2.70474384	2.03440392	2.21781717	2.63239444	3.08476996	3.34912432
GRAP	0.36128598	0.57885384	0.72422968	0.36491012	0.33394708	0.60954272	0.34219724	0.21779129	0.79144646
GRAP2	0.84463109	0.99360359	0.85428575	0.44611943	2.14460052	0.74266407	0.50359624	0.23022897	0.5063481
GRASP	1.55469411	1.94830064	2.45003084	1.74496713	1.36976608	2.61620784	1.42712905	1.27993253	0.75700193
GRB10	2.16932496	2.36458717	2.20652549	2.63269721	2.62581055	2.78171364	3.12039338	1.37953245	2.40155712
GRB7	3.90591033	2.5448228	3.16200423	2.472635	2.67696895	2.47698391	2.57243302	4.217622	4.33520998
GREB1	0.10516251	1.59197465	0.47333127	0.53847226	0.12684855	0.31241661	0.24752943	0.09455969	0.05324745
GREM1	2.47977471	6.5825394	4.59797931	5.67190248	3.0130365	6.62165313	4.43369775	2.62655308	2.02118262
GRHL1	1.05003043	0.53369779	2.22479842	1.57912202	1.24722038	0.87083886	2.71670503	0.4577936	0.94992027
GRHPR	3.71950023	3.2274859	2.73663161	3.44539596	3.04123868	3.43390579	3.55100867	3.67737986	3.4400502
GRIK3	0.02501331	0.49350853	0.1991381	0.11628673	0.079905	0.4512173	0.05918394	0.03838357	0.00567279
GRIK5	0.14509437	1.65137816	0.28066682	0.62675634	0.13253334	1.63104254	0.02159285	0.13878238	0.04513457
GRIN2C	0.31561761	0.13472504	0.28850583	0.11042296	0.08145128	0.08196316	0.08161441	0.12093424	0.21544767
GRIN2D	3.88742407	2.15168118	0.86158408	2.52565697	0.51069091	2.84506651	2.45194181	0.23842162	3.02892169
GRINA	6.94743108	6.36451472	6.87103557	6.39129852	5.73548932	6.66111898	6.3015337	6.30839606	6.87239212
GRIPAP1	3.17025661	1.95401692	2.41410612	2.2562098	3.07353192	2.19080989	1.80006378	2.4300886	2.260853
GRK5	1.30169295	2.90232617	2.43531732	2.51764863	2.16755967	1.95111354	1.6441771	1.10918473	1.25269469
GRK6	3.69996166	2.74807069	3.56679149	3.03630014	3.22182761	3.66829188	3.3624557	3.17266906	3.47465165
GRM2	0.15102729	0.06893198	0.17453179	0.1629924	0.07700746	0.17065603	0.02740789	0.11230442	0.03730872
GRM3	0	0.05043398	0.30637595	0.0242314	0.01000176	0.00298991	0	0.01561326	0.01702638
GRM8	0.08759428	0.0224555	0.65682009	0.09500485	0.29461047	0.06884125	0.33604991	0.30653859	0.25992833
GRN	8.02107767	7.38722322	7.99985856	6.98918235	7.28891212	7.53433761	7.77760783	6.85911332	6.84751017
GRPEL1	3.19415869	2.93438466	3.31128736	3.53948702	3.68051526	3.49306525	3.01457375	3.36006209	3.67702868
GRPEL2	2.04708644	2.02438339	3.00230734	3.20600054	3.04766974	2.94137256	2.98972702	2.26191612	2.84418628
GRSF1	3.39647981	3.30300051	3.71552179	4.05393856	3.89580122	3.80653419	3.88059672	3.65289811	4.00548129
GRTP1	3.79425255	2.24836866	3.4213776	2.40270546	2.20572454	1.8672185	2.79338905	2.40614582	4.11401682
GRWD1	3.62064383	2.14723766	2.51166877	2.40379679	3.10233565	2.96403979	2.70794603	2.88416563	2.88722726
GSAP	1.56782889	1.10826009	2.94851565	1.99288877	2.50640074	1.75481388	1.12724407	2.52105162	1.7768388
GSDMB	4.08744622	2.69144387	3.18892205	1.35353451	4.13171494	2.65612947	1.62624858	2.67582519	4.90781894
GSDMD	3.70414037	2.6160388	3.4630813	2.41763853	3.1253872	3.18625717	3.09676721	3.44020317	3.84048516
GSE1	2.35043619	2.75442671	2.9765697	2.57989706	2.66457729	2.77192018	2.87836327	3.58181801	3.95700931
GSG1L	0.04134877	0.04339077	0.18464649	0.00418831	0.00858811	0.03301268	0	0.01073808	0.00367015
GSK3A	5.04718491	3.56262015	4.1215221	3.89649557	4.29351056	3.99554325	3.72815079	4.16193611	4.14327376
GSK3B	3.82769956	3.345827	3.45142791	3.83035506	3.58568056	3.65792021	4.01263156	3.67328329	3.90672878
GSKIP	2.91842314	3.71462718	4.01413932	3.86094437	4.8271351	3.99731684	5.31995076	4.07381922	5.09913541
GSN	5.00112472	5.27407143	5.14132625	4.69675395	3.69540303	5.16773067	4.75454705	3.49573801	4.21379958
GSPT2	0.46326187	1.74846418	1.27107597	1.36098183	1.08536246	1.36926711	0.72979969	3.04533244	0.40815379
GSR	5.71378775	4.10469802	3.88057342	4.16396101	4.36023992	4.48593606	5.68071633	4.8972425	4.85639418
GSS	5.22626423	4.31281729	5.08573907	4.98345579	4.32684575	4.87682301	4.90426501	4.44256152	5.54303113
GSTA1	3.86644349	1.47985707	5.91196934	1.66516757	0.6177922	1.978964	0.50132809	4.3000357	0.05532442
GSTA4	0.89489869	3.18319736	2.81761954	3.09770122	1.47220788	2.17107436	1.99372788	2.2222626	2.16346089
GSTCD	1.67404852	1.42363124	1.88833617	2.8522522	2.38268143	1.98844454	2.27748369	1.53109278	2.1616092
GSTK1	6.65532528	5.58196912	5.19900169	4.51107971	5.63731883	4.85464415	4.82946563	4.85293758	5.76802574
GSTM1	2.94816862	2.87505985	0.06155819	0.1487406	1.96093917	3.29461645	3.11490749	4.14294893	3.89592223
GSTM3	0.47324951	2.86120574	5.39180796	3.20565417	2.47693377	3.71702089	3.13744995	4.3374627	3.48982913
GSTM5	0.39976176	2.50541032	0.38241237	0.98585309	0.11134047	0.94573229	0.32364143	0.25812766	0.15968062
GSTO1	5.61291306	5.59838222	5.40713486	5.97593216	6.41773393	6.29175259	5.49004328	4.90143046	5.44103561
GSTO2	1.68494013	1.28056496	1.15749015	2.33011068	2.08005299	1.84571842	1.9642583	1.38647241	1.80632329
GSTP1	9.0454402	7.76850415	8.64450206	8.59017679	4.62245981	7.43382293	8.56793892	8.14517051	7.82185671
GSTT2	0.09486945	0.14250471	0.21869793	0.01947811	0	0.28265919	0.18348228	0	0.03395919
GSTZ1	2.26915279	1.89763272	1.13273451	1.31988439	1.5739498	1.47924213	1.36665495	0.79117739	1.54462971
GTF2A1	2.70238101	3.37827843	2.82991259	3.35761734	3.39305356	3.42003619	3.23678045	3.67802493	3.32814224

GTF2A2	4.1118024	3.49901122	4.2004428	4.64721999	4.59274155	3.48841592	5.16352237	2.65040271	4.54461207
GTF2B	3.60279164	3.90642371	4.27358053	4.79473551	5.06529331	4.47491836	3.81517809	4.35594057	4.32462978
GTF2E2	3.75066041	3.61959281	3.49598266	4.29334165	4.41845541	4.48909788	4.80904367	3.99664796	3.79699682
GTF2F1	5.30544118	3.98837328	4.00639178	3.78516251	4.36661417	4.19391432	4.08746122	4.67887828	4.35922
GTF2F2	2.89178474	2.99005936	2.21131264	2.7832392	3.37674478	2.85598362	3.17020299	2.79555477	3.54968444
GTF2H1	2.36768971	2.65888024	3.27336484	3.06887905	3.34664104	2.87162816	3.12702857	2.72398149	2.76393834
GTF2H2	0.27110193	0.35447888	0.04481887	0.81261997	0.37202724	0.62216913	0.23820432	0.43549704	0.35277888
GTF2H4	0.25489313	0.47240624	0.9375302	1.22635397	1.02741561	0.58968057	0.69327921	0.83354133	0.88918254
GTF2H5	2.25251086	2.22914328	2.42014396	1.87743628	2.25002869	1.77111456	1.93196985	1.79393978	1.74571108
GTF2I	3.11514561	3.07417845	3.03197044	2.9236621	3.36483924	3.08242652	3.28501972	3.15073316	3.35427768
GTF3C1	3.43548262	3.30515729	2.54939644	2.50080988	3.0358063	3.01100243	3.06070425	2.97107943	2.95239209
GTF3C2	3.69867054	2.96668462	3.5701281	3.02743201	3.57975783	3.63456482	3.5527093	3.65885331	3.92746322
GTF3C3	1.97833708	2.37891692	2.61157846	3.14913952	3.44832442	2.84433017	3.18323461	2.8106193	3.21196577
GTF3C4	2.71656407	2.44157206	3.0777804	3.13640813	2.99359814	2.89741868	2.45501838	2.84270794	2.78258359
GTF3C5	4.16696042	3.0951022	4.1341137	3.69760954	3.48022587	3.55356824	3.98394354	3.67521962	4.14912069
GTPBP1	3.83972018	3.22734984	2.94786032	2.79937758	3.41416563	3.55126727	3.07986533	2.74142361	3.64998755
GTPBP2	3.40731542	2.63247731	3.8835254	3.60256761	3.2884516	3.55348749	3.68203108	3.77044124	3.31483077
GTPBP3	2.20190186	0.90921799	1.78763302	1.77732047	1.97419676	1.89045162	2.08970008	1.76996652	2.33688615
GTPBP4	2.64459519	2.63007338	2.49589901	3.62760491	3.37477277	3.38080654	2.97628273	2.48548127	3.39844375
GTPBP6	4.12719298	3.0141275	2.97554591	2.68833658	3.28266041	3.48533102	2.89797829	3.49632732	3.60931688
GTPBP8	2.25741481	1.92493958	1.67927213	2.48354528	2.51188721	1.92619761	2.26309616	2.04649133	2.12244497
GUCA1B	0.1550819	0.20915336	0.33612703	0.16678557	0.32386768	0.21219357	0.21789878	0.17787688	0.28065541
GUCA2A	0	1.43500753	0.21459072	0	0.07683349	0.04631603	0.03784061	0.07222123	0.18905828
GUCD1	5.55595404	4.71246764	4.76432035	3.82423805	4.74075221	4.18103319	4.1304475	4.31694174	5.26959481
GUCY1A2	0.13392291	1.1480025	0.765986	0.6982213	0.44009521	0.80030718	0.46549921	0.30527963	0.26163994
GUCY2C	0.02594084	4.03943007	1.31610937	0.76269478	1.18062108	0.2387752	1.17391372	0.60388281	5.40110104
GUF1	2.7429505	2.91761463	3.52382633	4.0773031	4.70718922	3.53874719	3.42591013	3.52621127	4.34704979
GUK1	5.79583521	4.84433861	4.84910279	4.13560722	4.44467386	4.98835122	6.39268092	5.02786799	4.98880839
GULP1	0.1363426	1.87227219	1.05354808	1.78636246	1.9994225	1.18708713	2.38929319	2.51002018	1.37394289
GUSB	3.97793001	3.80418309	4.69655271	4.09314532	4.01581654	4.17558507	4.5233794	4.46689894	3.78097088
GXYLT2	0.68984945	3.28280181	1.27407879	3.33050928	0.34183158	2.66407254	1.15364693	0.76409112	0.49114598
GYG1	4.15558425	4.50033747	4.01229674	4.18705331	4.39924898	5.03492774	4.18500757	3.27828242	3.6716352
GYG2	2.29097887	1.49043062	1.56911415	1.01025996	0.15736041	1.1633508	2.09504135	2.16077582	2.09684963
GYPC	3.04651916	4.28277387	2.77113944	3.12789364	2.50106055	4.70548139	2.46338407	1.86505361	1.5012154
GYS1	4.45564686	3.50285526	3.61404874	4.1211526	4.19979598	4.19772654	4.25139692	3.72487463	4.34397098
GZF1	1.85305504	2.11770173	2.08311405	2.6809158	2.19110209	2.51512489	2.1207936	2.07680635	2.54100116
GZMA	3.8698571	3.90849526	3.88755054	3.47300014	6.78826889	4.18283981	3.92197637	1.74987843	4.66356749
GZMB	4.70303877	3.21507096	3.75155996	4.36024819	7.17494538	4.1839213	4.48210257	1.28408127	3.79917979
GZMH	3.31110743	2.41586736	2.10462235	1.9928355	5.34983482	2.88421098	2.73223484	1.27684598	1.86352008
GZMK	1.13137871	3.21213215	2.15084713	2.77813356	4.78942576	3.06254331	1.23760891	0.92540616	1.51635882
GZMM	1.53504301	1.30448591	1.03061409	0.46979442	2.01082378	1.55814196	0.75538551	0.24544048	0.64344328
H1FNT	0	0.1808712	0.1895267	0	0.01716796	0.01026395	0	0.01076483	0
H1FX	7.35728036	5.01611418	5.76862467	5.57606117	5.11196532	5.78482156	5.99014661	4.67821757	5.35858417
H2AFJ	3.23619921	3.50709845	2.97508412	2.86430432	2.70345191	2.95543091	2.97415971	3.58651137	0.99216014
H2AFV	5.40039491	4.72624923	5.4086448	5.13994334	4.8737909	4.92576579	5.2956273	4.27725853	4.72914242
H2AFX	6.7218218	4.31784968	4.80575061	4.94596037	5.38760828	5.17632917	5.57579233	4.13067064	5.63744585
H2AFY	3.72268419	3.44349899	3.6684824	3.94175592	3.97699553	3.46955478	3.67448722	3.34261014	4.86349369
H2AFY2	1.53655449	3.01331359	1.97156956	3.71000286	1.20950272	2.57862354	1.5482367	3.56053387	3.03016799
H2AFZ	6.63601835	5.52275096	5.36990938	6.37157864	6.85626933	6.35059555	6.37853728	5.77235651	6.41448877
H2BFS	0.95127849	0.55762497	0.21250859	0	0.26662072	0.08478955	0	0.57222852	0.08097496
H3F3A	5.91293312	4.34890577	3.3750077	4.02042904	4.00697466	4.21001811	4.67457938	4.59317631	4.45265894
H3F3B	6.84769717	5.83758553	6.36554296	6.51079269	6.40186957	6.16017035	5.9204186	6.52739467	6.04955508
H6PD	5.49107113	4.4403226	3.48179972	3.35209051	2.86347968	3.65648266	3.07141507	2.78347062	3.56259684
HAAO	1.19415447	3.43137451	2.62336694	1.35430189	1.88763614	2.33388294	2.66871999	1.06306043	3.28732896
HABP2	5.02019489	0.4310295	5.82794433	0.79373047	1.18150656	0.15930797	0.14732084	4.52835622	0.71286227
HABP4	2.07084027	1.5770275	3.6563973	2.25746486	1.7565344	2.04552545	1.09062465	0.96745879	1.22588001
HACE1	0.82245037	1.0051552	1.17154859	1.43628435	1.39451752	1.29504689	1.22790728	0.955363	0.8380911
HACL1	2.36717397	2.46924256	3.16692333	2.83326637	3.42147224	2.93213896	3.22043999	2.62362925	3.28210371
HADH	4.24741812	4.46325012	4.84904963	3.82593096	4.60742407	4.41574862	4.88705624	4.42683656	4.86316265

HADHA	5.50310024	6.03202937	5.60521744	5.50985383	5.59880632	5.98175289	6.11643738	5.58252683	6.25529004
HADHB	4.00563329	5.22996115	4.74521241	4.5965902	4.86147625	4.90464594	4.51249059	4.79121874	5.0901269
HAGH	2.97720132	2.01496361	2.0916004	1.67885141	1.58761468	1.97273098	2.11709265	2.10261958	2.15062039
HAMP	1.11541368	0.18218837	0.65628295	0.4674613	0.65433718	0.52393891	0.45603394	0.19605346	0.09244009
HAND1	0	0.01196157	0.02998553	0	0	0.99517819	0	0	0
HAND2	0.71227255	4.09705219	0.45415444	0.82734794	0.16479479	3.14461119	0.5227901	0.2467874	0.193848
HAP1	0.02898926	0.05269917	0.25881522	0.27435137	0.04265734	0.11771718	0.01742303	0.04445226	0.04246629
HAPLN1	0.23627905	0.01789514	1.32841498	0.10894289	1.57421272	0.17047477	0.7129335	0.31283882	1.03017669
HAPLN3	3.57241548	1.49518417	2.14511967	2.47142528	4.22392111	3.30163334	2.40239556	2.07018618	1.70689714
HARS	2.74514046	2.37249541	2.65791529	2.81522529	3.07185155	2.74495954	2.44125602	2.92566691	2.75285124
HARS2	2.68021658	2.2690788	3.05817192	2.94066889	2.97644184	2.52384671	2.86943318	3.19036165	3.06649854
HAS1	0.0321644	0.55079827	0.70949333	0.71588136	0.07800161	1.69630694	0.16537863	0.26285897	0.02541975
HAS2	0.85430914	2.45666538	2.01602448	2.93173228	1.04681433	2.59973555	1.35156095	1.85644855	0.7765638
HAUS1	2.45433416	3.14183146	3.54604591	3.38454301	4.02584084	3.30181987	3.99143483	2.18531659	3.64171422
HAUS2	2.40940438	1.96050039	2.54237292	2.70913369	2.93187089	2.74425542	2.54367545	1.54653551	2.62371444
HAUS3	1.2290039	1.02722293	1.24254204	1.7239099	2.15902266	1.58194252	1.20225183	1.3021128	1.69334854
HAUS4	2.9309931	2.81694625	2.92847856	2.67586756	2.68357034	2.87556795	3.03047766	3.25339941	3.53615662
HAUS6	1.44518069	1.77278128	1.7605706	2.43306956	2.71417384	2.42224002	1.93342622	2.59807019	2.2740838
HAUS7	0.60600784	0.48283212	0.39260644	0.4221669	0.46921034	0.40869056	0.34114993	0.31088077	0.53114003
HAUS8	1.40252179	0.83345209	1.53273734	1.2678003	2.14868315	1.17513598	1.03525846	1.02730809	1.46294607
HAVCR2	2.97086403	3.21309817	3.94512482	3.13898497	4.37315419	3.69391306	3.68995278	2.16873456	2.1979931
HAX1	5.54262238	4.6143721	5.09442618	4.92187215	4.87145637	4.87482172	5.51120239	5.60562464	4.86495665
HBB	1.59171837	3.33019807	5.04897832	3.78561486	2.65677887	6.97881453	3.96191266	7.26088467	3.06642401
HBEGF	2.08952086	2.64053436	3.8922192	3.05167005	2.41173713	4.77964771	3.13032449	4.66063959	3.42270216
HBS1L	1.85859594	2.08352596	2.26651758	2.72515002	2.77574847	2.32285439	2.71552592	1.71366033	2.66216531
HBZ	0	0	0	0	0	0	0.05666369	0.01848584	0
HCAR1	0.00762348	0.07673182	0.56036191	0.33416917	0.04633611	0.21123703	0.0226982	0.25825875	0.10459544
HCAR2	0.69228361	0.35862316	0.88389364	2.8758217	2.56111497	1.52283055	1.1530927	2.09128695	1.60990066
HCCS	3.28574968	4.24667882	3.93505256	3.98884933	5.09721924	4.01726473	3.71833027	3.66299893	4.17700914
HCFC1	4.89898568	3.4027792	3.3775231	3.34772289	4.39130268	3.88409734	3.6048658	3.53838192	4.37644842
HCFC1R1	5.59229359	4.9995308	4.0730323	4.27189205	3.63387436	4.0623847	4.2222622	4.16825659	4.01017214
HCK	3.57966361	3.22549283	4.27301408	3.26339546	4.08234468	3.89273614	3.63908587	2.6710348	2.71304541
HCLS1	3.14839692	3.50392928	3.85573559	3.07717136	3.99559211	3.73134407	3.39696768	2.50731664	2.27046043
HCN2	0.20871954	0.49323999	0.29898092	0.19195303	0.20821488	0.60166548	0.6891444	0.18901975	0.20895534
HCN3	1.49913893	0.44536592	1.00701011	1.01869536	1.12814301	0.87802356	1.29028546	1.46968006	1.56984902
HCRT	0	0.29360612	0.28854941	0	0.03839556	0.08996553	0	0.07124421	0.32555101
HCST	4.59700807	4.53828652	3.52478433	3.26734616	4.3926653	4.67578706	3.86225586	1.8751387	2.31780066
HDAC1	5.86588293	4.1168871	5.85739026	4.81364358	5.87311404	4.90270972	5.21189409	4.29028972	5.81494125
HDAC10	2.10987065	1.08958703	1.06682432	1.32896518	0.90114047	1.03551288	0.91746987	1.09403136	1.71001392
HDAC11	2.14567551	2.09753273	1.62476123	1.41077481	0.8302297	1.4679881	1.74207333	2.4877823	2.12988541
HDAC2	2.7251839	2.21839005	2.23068493	2.40930846	3.23307571	2.32746362	3.0744181	1.84731404	2.68202308
HDAC3	3.75924696	3.06106188	3.85776479	3.63821381	3.6375807	3.32958928	3.48764923	3.27796539	3.50073044
HDAC4	1.60628713	2.20032345	1.21848442	1.33621948	1.37631558	1.9013549	1.51699698	1.14246432	1.19741433
HDAC5	2.79197948	2.58388288	2.52434453	2.17676717	1.91146633	2.43513563	2.14927706	2.83873549	2.91920633
HDAC6	1.94515801	2.22393891	2.01039277	2.19055579	2.07040638	2.05075825	1.61394529	1.95018589	2.17749493
HDAC7	3.36209238	2.9314243	3.30275149	2.79714164	2.78498526	3.01436075	2.27314705	4.12857529	2.09292643
HDAC8	1.77341644	1.68312955	1.97383516	1.83806182	2.62461242	1.84053249	1.7658842	1.97404987	1.8916145
HDAC9	0.24446713	1.13101685	1.7471061	0.47601824	2.38865821	1.2503514	0.53985383	1.309051	0.16241232
HDC	0.02737634	1.55152552	1.00080001	0.88493211	0.32396544	0.61597816	0.0959987	0.07270193	0.03822879
HDDC2	3.11622982	2.80710984	2.12637867	2.86559539	3.00073903	2.78502977	2.66317175	2.08723027	2.42506677
HDDC3	2.06668158	1.68502461	1.95284014	1.57121484	1.48104959	1.83221995	1.63792546	2.87875806	2.59194669
HDGF	7.64223239	5.26236386	6.47648764	5.75205112	5.74896824	5.84575956	6.48703359	6.80329464	6.36434644
HDHD2	1.25755284	1.94773348	2.36170102	2.14669545	2.13781437	2.30000923	3.17065136	1.74115238	2.3340717
HDHD3	3.97388706	4.06299317	5.7746864	4.40767128	4.31819536	3.71916915	3.71358625	3.92686161	5.41987339
HDLBP	5.67068923	5.81011426	5.71920204	5.80550036	5.39195207	5.53462665	6.57278278	6.37343852	5.89803213
HEATR1	2.01738452	2.67699547	2.68378065	3.01280257	3.54403693	3.30281116	3.30747183	3.95165765	3.32735886
HEATR5B	1.8509767	1.93207576	2.48812343	2.43575413	2.91944106	2.26215393	2.65023814	2.38020889	2.73698239
HEATR6	2.59551359	1.96608214	2.95946975	2.38710827	2.49125276	2.23993288	2.04765307	2.54798871	2.25307237
HEBP1	2.22557349	3.92607896	3.57372644	3.44313487	2.9210498	3.89786149	3.42374071	2.00146883	3.68078722

HEBP2	4.00675331	2.72859766	3.55461133	3.05741096	3.0821216	2.86467559	3.89409065	2.63730596	2.97825124
HECA	2.06396507	3.22948624	3.14530404	3.02657178	3.18980755	3.26088125	3.21748589	2.22389101	2.79032353
HECTD1	3.87755799	3.50962814	3.41955889	3.61785264	4.09896916	3.21747793	3.45596897	3.88177968	3.62652712
HECTD2	0.32218855	1.42734156	0.86322723	1.38341649	1.28236031	1.24990969	0.4013922	0.79621846	0.20619712
HECTD3	3.55734031	4.04029355	3.88580292	3.72903716	3.81897058	3.93788216	3.61503384	3.9027795	4.6342647
HECW2	0.6073575	1.25548668	1.93405798	1.04375235	1.48920758	0.91670972	0.98417083	0.66756158	0.59906579
HELB	0.45982348	0.57614503	1.3648896	0.81844836	1.64863231	0.96593951	0.62943087	1.12432478	0.91714712
HELLS	1.73875965	1.05220163	1.52755776	2.3278222	3.11997796	1.95544701	1.75754559	1.64259774	2.11570517
HELQ	1.18844813	2.10983597	2.04479733	2.22076973	2.15498341	1.8835336	1.32355132	1.86936009	1.80283345
HELZ	2.27546838	2.88159236	2.52497141	2.50861247	2.9245594	2.76650183	2.51958929	3.09197657	2.62628824
HELZ2	2.75648506	3.25158502	2.68008602	2.46516576	3.35794331	3.40832081	2.82952107	3.25772168	2.86010493
HEMK1	0.9991086	0.53375399	0.88334703	0.762768	0.68173367	0.57601433	0.69777293	0.49262006	1.12784714
HENMT1	0.72771436	2.47891365	3.26735686	2.08060532	1.79747559	2.84760065	2.82829279	2.54995122	1.09657885
HEPACAM2	0.13978212	2.97114485	1.12170371	0.0687822	0.19428067	0.09899096	0.60185036	0.16550368	0.76600748
HEPH	0.99339518	4.64552916	2.19351302	3.23223472	4.06008719	3.02533	3.61025248	1.70483253	5.40820939
HERC1	1.55610495	2.48516995	2.06301422	1.79634903	2.79834992	2.56820193	1.94586518	1.56979601	2.06325137
HERC2	2.03933887	1.88979819	1.7629821	1.74732347	2.21115419	2.11404687	1.92018025	2.1672348	2.19483474
HERC3	2.82670703	2.04049214	1.89055996	2.0479226	2.78042881	2.24978601	2.40878259	2.13415498	2.3738356
HERC4	1.08450871	1.72051152	2.04784095	2.07337086	2.29730155	1.93364392	1.19663144	1.43002319	1.7883784
HERC5	0.48287952	2.88278738	2.01453264	2.1141896	2.02595957	3.03457187	1.46183628	1.09480472	1.21796165
HERC6	1.51923666	3.92292589	2.04668709	2.89654905	3.72088152	3.50569155	1.71318738	2.02407175	1.71400454
HERPUD1	4.55866473	4.44789988	5.43099094	4.79066452	4.73973041	5.0524362	4.88942713	5.34397538	4.84882761
HERPUD2	3.5751586	3.45634184	3.52673517	3.53009284	3.62957655	3.90274011	3.73068461	3.54240868	3.30166287
HES1	6.27353396	4.61932382	5.06038848	5.08473678	5.12273982	5.50121712	5.69405236	4.94283531	4.59628376
HES2	0.18331395	0.32680573	0.2124355	0.80372515	0.07287031	0.52207572	1.00691742	0.23666897	1.02772202
HES4	2.24818449	2.50393349	2.75406997	3.22715899	1.22422491	4.15273625	3.23673097	2.56994179	2.10892124
HES6	2.96273156	1.92554015	1.76982404	1.41903726	1.30266743	1.600379	2.85515257	1.37387637	3.86252715
HEXA	3.1802563	2.80422909	3.86425133	2.92422866	2.72892867	3.01520533	2.78173228	1.8656354	2.40804231
HEXB	5.347128	5.38294919	6.09937394	5.23365975	5.29371969	5.19388461	5.8206457	4.94274072	4.37330963
HEXIM2	2.18036262	0.76196967	0.97611319	0.72633088	0.80598171	0.48623172	0.83114157	0.74708038	0.82809473
HEY1	0.78818439	1.45971185	1.58447529	1.65859619	1.01835679	1.79244879	1.35503521	0.48610808	0.66676372
HEY2	0.76590343	1.18747084	1.5013405	0.96833071	0.83471889	1.60337196	0.81666673	0.47952618	0.63437166
HEYL	1.52103109	2.81948284	3.61664491	2.85738431	2.33293571	3.1301863	2.52565631	1.92009484	2.08246303
HFE	2.12900761	1.76632974	2.93315059	1.49640117	1.07014579	1.89970302	1.5622688	2.99781312	1.49708735
HGD	5.22390429	1.56545912	3.77302847	2.31416175	0.96309991	0.45446389	0.29645143	2.77726828	1.1959166
HGS	4.24459837	2.6070783	3.22518623	2.90195909	3.06849692	3.25833635	3.03691126	3.95722736	3.74382861
HHAT	0.81621523	0.97150312	0.91028475	1.06725547	0.21913549	0.8895208	0.55713758	1.98498652	0.43286081
HHATL	0.01255159	0	0.01810119	0.01504776	0.00774523	0.00462444	0.00752657	0	0
HHEX	6.33569434	2.13317341	2.57667192	2.77278866	2.10345126	2.56431039	2.37634219	1.37864202	0.88081076
HHIP	0.11873434	1.67334766	0.47035375	0.09122335	0.03671885	0.28580468	0.08932706	0.09890789	0.00791136
HHLA3	0.91755775	3.50150097	2.04836238	3.45244308	2.78316849	2.55863926	2.78457323	2.16152017	2.42151876
HIBADH	4.7699799	5.00231535	3.62030961	4.14904109	4.79615792	4.27200304	4.6412773	4.17159386	4.2487951
HIBCH	1.54375148	2.33024987	1.68182353	2.36018371	1.915429	2.09151634	2.80851388	1.53182327	2.93780715
HIC1	1.67228463	2.21488862	1.62918228	1.58411246	1.01240436	2.49034284	1.04870558	0.90628094	0.63708291
HIC2	2.02340451	0.72021437	0.98063493	0.76478447	1.10800833	0.85907191	0.94550896	0.4451498	1.01496726
HID1	5.43234798	3.20812462	4.83897389	4.11520249	4.23074851	4.41174385	3.48940151	5.84034392	4.178057
HIF1A	6.26164961	6.94489688	6.64740783	6.68247808	6.3496987	6.26069495	6.41281345	6.00726864	5.63773675
HIF1AN	2.35912997	1.75014932	1.59329756	2.04577947	2.29016976	2.15311787	1.81125011	1.9358331	2.10373524
HIF3A	4.02787477	0.96144426	1.93485144	0.43621792	0.55615562	0.58182416	0.06122993	0.98586418	0.12487897
HIGD1A	4.76376319	5.31895026	5.05157691	5.61847199	6.06073793	5.54104479	5.64760524	4.73237492	4.96766814
HIGD2A	7.77687551	6.63686455	6.15301768	6.55772155	6.5467187	6.35938027	5.96539897	6.41059428	6.9744637
HILPDA	2.85521363	3.18835666	3.06991514	3.95459492	1.64505673	2.06783022	3.22832427	3.54328149	3.67480372
HINFP	1.15051992	1.04002319	1.25574815	1.55351883	1.27359503	1.24212071	1.27533657	0.92433773	1.81353736
HINT1	7.22517269	6.0383889	6.46977582	6.61197888	6.87217175	6.52994247	6.3894656	6.14473667	6.54281807
HINT2	3.26248866	2.73182505	2.83939715	2.4120024	2.12627202	2.82229129	2.39558096	2.7649201	2.84991311
HIP1	2.24703903	3.03338184	2.30105447	3.03882781	1.88867023	2.85164522	2.32570781	1.9400849	1.46026095
HIP1R	2.92768452	1.86172043	4.52951129	2.34551229	4.03505977	1.89721084	2.05355117	4.30209611	3.07299217
HIPK1	2.99976464	3.56026049	4.02616058	3.60865765	3.53314875	3.91040808	3.81524602	4.28098216	3.47886574
HIPK2	4.59771975	2.88839123	4.18527001	2.29847109	3.82607075	2.74668711	2.91020407	3.92016561	2.67601636

HIPK3	3.22866736	4.75991035	4.2223277	4.09355789	4.27258101	4.98531356	4.66224528	4.45555096	3.68577819
HIRIP3	2.55836615	2.13790195	1.41759004	1.75157864	1.38911386	1.7357247	1.71589598	1.52229385	2.10327067
HIST1H1A	0	0	0.0400712	0	0	0.04074842	0	0	0
HIST1H1B	2.4168721	0.11768441	0.14691461	0	0.47351323	0.33223432	0.34080574	0.34669727	0.3482091
HIST1H1C	6.01053582	5.89069793	5.88037698	4.96863586	6.27255838	4.1930777	4.75760885	5.31886442	3.47966555
HIST1H1D	1.12157642	0.06136579	0.28596663	0.29580092	0.45824491	0.17047477	0.09506872	0.3370204	0.1884817
HIST1H1E	5.41555056	0.38618964	0.71017901	0.47547883	1.44428879	1.39579461	1.63593518	0.95912678	1.86218588
HIST1H2AB	0.40206284	0.05216414	0.30123225	0.1579414	0.56089609	0.13023312	0.10695905	0.03530645	0.22519798
HIST1H2AC	4.80205742	5.07450757	5.25307754	3.84731307	5.4226153	3.67143803	3.8533671	4.94561578	3.94494857
HIST1H2AD	2.7066434	0.88505754	0.30123225	0.9452721	0.90394284	1.01117949	1.67502117	1.94450799	0.98538264
HIST1H2AE	2.96598638	3.72105624	0.68141391	0.42983768	2.27532904	1.09332113	0.62170319	2.27502016	1.53211825
HIST1H2AG	1.31802669	1.62464558	0.94463286	0.65311209	1.08439543	0.71680069	1.17856276	0.41489344	0.8069406
HIST1H2AI	2.00479863	2.77394074	1.356253	0.83463607	2.70886711	0.6526334	0.90445272	1.71548872	0.50830461
HIST1H2AK	0	0	0	0	0	0	0	0	0
HIST1H2AM	1.58142596	0.66821436	0.59534337	0.62284167	0.93415536	0.41212777	1.00030686	0.76016589	0.49103249
HIST1H2BB	0.06332324	0	0.21671414	0.03832368	0.03933743	0.02359063	0	0.07297176	0.03363127
HIST1H2BC	3.79737308	2.70810903	2.93593321	2.10071975	2.74629432	1.52090046	2.54176883	3.31699511	1.08031814
HIST1H2BD	5.42906475	4.92938353	3.51801598	3.39241667	4.30063182	2.32695874	3.5391839	5.78055216	3.21382937
HIST1H2BE	0.4084123	0.45929268	0.36029537	0.30519328	0.64564292	0.13248963	0.10882735	0.3532796	0.18599467
HIST1H2BF	2.04133955	1.98635248	0.36447341	0.6013456	1.35171139	0.44934738	0.48222673	0.59559486	0.50408179
HIST1H2BG	0.64529365	2.31307496	0.92777829	1.34880755	1.74757067	0.73652079	1.0602534	1.71487831	1.10003759
HIST1H2BH	3.3988617	1.59113923	0.51714208	0.84445479	1.48399515	0.9635469	0.44064209	1.3698272	1.03248958
HIST1H2BI	1.04324633	0	0.36447341	0.26170713	0.21832723	0.03472493	0.11020234	0.14041104	0
HIST1H2BM	1.33627533	0.38275543	0.46802595	0.26170713	0.79512482	0.06863366	0	0.17347783	0.09721084
HIST1H3A	0.45289509	0.27648387	0.06268189	0.24413023	0.25014006	0.18332734	0.24362484	0.19173544	0.13346272
HIST1H3B	2.54983169	0.43516496	0.43855217	0.33128092	0.42307321	0.46875622	0.33061481	0.27882356	0.40389794
HIST1H3C	0.08605551	0.31782015	0.23587627	0.15137452	1.55143737	0	0	0.43865689	0.04587318
HIST1H3D	1.95932881	0.85579079	0.61623099	1.04920934	1.24069442	0.82198093	1.61371709	1.50983677	1.21699436
HIST1H3F	1.4111709	0.27648387	0	0.05224296	0.10531471	0.0945737	0.10243706	0.25037433	0
HIST1H3G	0.04465023	0.63983413	0.33041051	0.07078666	0.06375556	0.27248444	0.03574782	0.37272566	0.09232867
HIST1H3H	2.23779655	3.05704751	2.02983307	2.1554602	3.35887289	1.21915631	1.08723342	2.07966516	1.06689006
HIST1H3I	2.09003853	0.04991864	0.06268189	0	0.46325116	0.18332734	0.37221876	0	0.40389794
HIST1H3J	0.0462756	0.02668793	0.81029894	0.0822503	0.23967135	0.13199953	0.2092614	0.23374428	0.11858264
HIST1H4B	0.98584855	0.09230563	0	0.04907664	0.19171626	0.03025296	0.35185166	0.12289349	0.16512707
HIST1H4C	3.50296458	0.92457959	1.70734655	0.53897053	0.77863648	1.20262206	1.05495029	0.8879412	1.02705472
HIST1H4E	2.71956179	0.30087255	0.23325847	0.19626703	0.58380033	0.30800325	0.78827674	0.61400994	1.1597709
HIST1H4F	0.78969048	0	0.43399972	0	0.13718666	0	0	0	0
HIST1H4H	3.99579317	3.47198325	2.90341719	1.04888552	2.10191293	1.33323981	1.35710087	1.83798777	1.47128962
HIST1H4J	0.68664143	1.43664361	1.08771708	0.53302231	0.58560128	0.71982643	0.36040633	0.72471286	0.54872218
HIST1H4L	0.11232256	0	0.15961792	0	0.07022336	0	0.36827011	0.044333	0.27833745
HIST2H2BE	3.068412	3.54591233	3.10823599	3.94719327	3.01413881	2.54344275	3.57042381	2.65215908	2.53318587
HIST2H4A	0.12995578	0.30930191	0.12109018	0.0458838	0.08143281	0.22431074	0.17500805	0.14247781	0.16392059
HIST3H2A	0.64570883	3.20368301	1.29149513	3.9818801	2.88049562	2.59952642	2.85490944	0.47677621	4.07933767
HIVEP1	1.14272663	2.43524935	2.05199029	2.1147054	2.57008341	2.1742511	2.06407415	2.5297274	2.37795228
HIVEP2	3.10324709	3.30144056	2.68858528	2.67896559	2.90677981	3.17626021	2.78093372	2.36985381	2.45538223
HIVEP3	1.26485729	1.01894923	0.92849821	1.08251687	1.77220132	1.30003136	0.98021149	0.60037938	0.69337487
HJURP	2.26546708	1.28406952	2.58747241	2.83674726	3.14408126	2.64034073	2.85856512	1.99231642	2.9086077
HK2	3.81009522	1.58936874	3.18571147	3.93933322	4.16926516	3.48504627	5.37131499	3.97321701	4.35690102
HKDC1	3.49405826	2.75239585	3.62209993	2.79033168	1.56488301	2.46350584	2.5449444	3.25514017	4.34686891
HKR1	1.46862716	1.49365669	2.37385137	1.91901022	1.39335081	1.51962756	1.45216584	2.6932457	2.14813272
HLA-A	9.32683769	8.40991702	9.15120599	7.86066793	9.00795805	9.37563423	7.51699899	8.27648568	9.68004754
HLA-B	9.07504601	8.88014954	9.05838873	8.4347681	9.71422948	9.89423135	8.29367234	8.64431238	9.39589197
HLA-DMA	5.8642504	5.82885953	7.14012429	4.98453138	7.60419755	6.14686677	5.1460439	5.70636254	5.69584179
HLA-DMB	4.16355843	4.2313947	5.35459906	3.70740006	5.89817618	4.50017234	4.01963705	3.38915541	3.70587411
HLA-DOA	3.63042251	2.92534759	3.3700419	2.76525595	4.67546045	3.52367995	3.41162644	2.3055531	1.98870177
HLA-DOB	0.91360936	1.44420186	1.54371018	1.09345104	2.43894346	0.82319056	0.324582	1.07429378	0.8766914
HLA-DPA1	6.60248329	6.37676354	7.55404652	5.31992414	8.15467455	6.88011601	6.30316465	5.62408216	6.41428607
HLA-DPB1	7.37576654	6.76712739	8.19509691	5.92788955	8.02811222	6.67117724	6.81916923	5.7335905	6.03977841
HLA-DQA1	5.68173493	3.71207201	6.0649432	4.66090353	6.23384822	5.10301091	4.78317747	4.09966165	4.03043355

HLA-DQA2	1.66514137	5.5090476	2.38938002	1.68602808	7.26063979	2.61400415	4.64685511	0.41382378	0.35480602
HLA-DQB1	6.88842939	3.48622894	7.07238657	5.69691368	6.22141992	5.40382171	5.74273587	5.78455178	5.97201416
HLA-DQB2	2.78229412	2.69115805	1.88393329	0.20096456	5.89082753	3.34000949	2.45453558	1.79796801	0.79898525
HLA-DRA	9.85970832	10.2145457	11.1159035	9.18828679	11.781182	10.4506166	10.105906	8.93190928	10.0000357
HLA-DRB1	9.1812217	7.58591508	9.81135278	7.52090393	9.52743451	8.78565764	8.14284131	8.1631337	8.36987597
HLA-E	8.26907167	8.05649982	8.46142145	7.28839477	8.13674114	8.51654103	7.34606357	7.54028182	8.01252633
HLA-G	1.11145539	0.91564638	1.85561702	1.54875826	3.21685022	0.90607957	0.23893812	0.25494653	0.77864387
HLF	0.38104362	1.49188986	0.36766529	1.12582453	0.56644626	1.3714236	0.22871005	1.2891207	0.26439042
HLTF	0.46635011	2.68612319	2.23891392	3.57093388	3.78125565	2.93576118	3.68858537	3.02485768	3.33868748
HLX	1.05974156	1.49712536	1.16026281	1.06708029	0.68247949	1.60329895	0.95836735	0.58717327	0.62473884
HM13	4.46934164	3.8000038	4.67508814	3.95006812	3.91716809	3.99867874	4.17946386	4.53125537	4.43740459
HMBOX1	1.86349711	2.21762791	1.46502821	1.88142319	2.00855509	2.06694427	2.29281842	1.91435393	1.91846189
HMBS	2.44131334	1.91063238	1.90202762	2.48449342	2.21370737	2.82952779	3.06284252	1.99453354	2.83350887
HMCES	5.27009904	3.31442708	3.74073347	3.63712525	3.6898805	3.82922805	4.06954347	3.92969249	3.82900733
HMG20A	2.01359697	2.36685203	2.77165682	2.31720695	2.63368044	2.38278073	2.2615953	1.84442335	2.70405089
HMG20B	4.99818899	3.26339497	3.58514125	3.70093792	3.69725363	3.52861016	3.53182556	3.95558625	3.90570666
HMGA1	8.39423925	5.68976402	6.94027817	7.04408071	7.2995686	7.96038375	8.15853205	9.05647993	8.27991894
HMGB1	5.71975832	5.12922219	4.6286501	5.05454706	5.17764032	5.18223752	5.43802825	5.05007264	5.84607846
HMGB2	5.76650265	4.17858417	3.33190818	5.39044571	5.82468223	5.31878779	5.43057166	4.08381929	5.0763571
HMGB3	4.16142135	2.92223717	2.85751027	4.2659661	4.84828845	3.17359961	3.35615094	2.65003672	3.95109994
HMGCL	3.27693221	2.84372854	2.46443658	2.42965916	2.79972534	2.73862859	2.9084068	1.88347747	2.9848041
HMGCLL1	0	0.20632136	0.11082631	0.10114187	0	0.22671204	0.03146951	0.06857706	0.0039855
HMGCS1	3.92290287	2.99407487	3.27600735	5.03680113	3.04409546	3.84537206	3.94679812	4.00782559	4.02934076
HMGCS2	0.31688836	7.07849697	2.24086175	5.70542362	0.55791055	3.57190286	7.29553895	1.43719348	0.31951
HMGN1	4.37647722	4.24971447	3.91931696	4.9992415	4.9525917	4.6865383	4.25911856	4.56132957	4.9959891
HMGN3	5.75261716	4.61319726	5.10984105	5.58966509	5.50608833	5.20556225	4.53175205	4.73068173	5.15263936
HMGN4	4.29564573	5.03720768	5.69283059	5.68916097	6.1895255	5.61804652	5.1241509	4.91252534	5.94357841
HMMR	2.43371549	1.77046887	2.59792816	3.16707991	4.08420488	2.50373575	3.38717784	2.12407842	3.28257072
HMOX1	6.32626642	4.86818074	4.93382168	3.71005374	4.41490049	5.55469382	4.02382589	3.54842252	3.70567608
HNF1A	2.52428982	1.1031732	2.16498215	0.92238658	1.66434009	1.29660461	1.51381434	2.71868546	2.59868534
HNF1B	5.99103382	1.60690516	2.86484232	2.75112154	1.75948364	1.77435363	1.97992334	3.58951509	2.63703757
HNF4A	3.9261225	3.24139738	3.15677614	3.9812256	3.28802943	2.85267162	3.26338443	3.34195003	5.62173599
HNF4G	2.31890191	3.1645559	2.29278211	2.28747466	3.50036752	3.38432981	4.45815206	3.01768615	4.34411197
HNRNPA0	5.27809032	3.73524926	3.80226735	4.17399389	3.5056663	3.93588013	3.71087067	4.00498067	4.4818878
HNRNPA1	6.86640294	6.03115507	6.0952018	6.75555016	7.02949927	6.69731736	6.64766266	7.08111795	7.1796096
HNRNPA2B	6.61268932	5.43253765	5.5611273	5.99998293	6.9226436	5.95649052	6.16801398	5.86715082	6.22181123
HNRNPA3	5.39452894	4.78991922	5.01930834	5.1363978	5.79743914	5.20232616	5.08387616	4.82732606	5.58640751
HNRNPAB	6.84371941	5.58894244	5.93703363	6.25578161	6.63768751	6.17632841	6.09284046	5.88438533	5.89592857
HNRNPC	5.68541412	5.22020142	5.36821735	5.42990103	6.05349171	5.53870672	5.62518632	5.64132355	5.6708346
HNRNPD	5.78356632	3.96772869	4.07920488	4.73468735	5.16433716	4.58153594	4.60024994	4.78234257	5.20941355
HNRNPDL	4.93906326	4.30883493	4.86455825	5.31491015	5.47004751	5.05838761	4.59819904	4.85062387	5.56155255
HNRNPF	6.56411764	5.99767829	6.50408702	6.386644	7.4434078	6.77266643	6.59593265	6.86690835	6.95643387
HNRNPH1	4.35138956	3.50717139	4.32771525	5.13926867	5.01837904	4.25619282	4.27369337	4.24683222	5.05184824
HNRNPH2	4.74760245	5.10345706	5.8990851	5.55687463	6.30468619	5.40080975	4.98235306	5.40175161	5.48625154
HNRNPH3	4.2290695	4.08032252	3.88330437	4.40037545	4.79432134	4.48397821	3.83774942	4.03841637	4.23652891
HNRNPK	6.61509196	6.24201961	6.58276782	6.50236511	6.94312295	6.5854367	6.50202539	6.7168669	6.82441608
HNRNPL	5.41984421	4.04854543	4.68993332	4.83159352	5.08368433	4.70133696	4.69936239	4.53466443	5.31080467
HNRNPLL	2.83452941	2.94736019	3.43852248	3.38398093	3.73950692	3.17341681	3.17258835	2.67026059	3.03261204
HNRNPM	5.05332302	4.20065966	4.38153168	4.5710073	5.27084535	4.91312011	4.91524723	4.76976504	4.96476189
HNRNPR	4.18557903	4.06543662	4.01120243	4.60225368	5.30064757	4.56162287	4.62341719	4.24906429	4.67078059
HNRNPU	5.46458421	4.58918434	4.68424356	5.24006416	5.73936139	5.20725806	5.45491171	5.61327118	5.34193598
HNRNPUL1	5.79632536	5.01807313	5.29943851	5.2062294	5.69744237	5.39498222	5.29366518	5.627977	5.55710534
HNRNPUL2	3.83664151	2.58695252	2.55303505	3.10664137	2.71882874	2.66799459	2.6920881	2.67491222	3.04923192
HOGA1	1.68289023	0.08785822	0.16194745	0.13092889	0.07385408	0.02242247	0.01048072	0.17816733	0.02290879
HOMER1	1.54651471	1.1120604	1.80247625	2.29236126	1.54815383	2.26326688	2.03943944	1.70384754	1.16809761
HOMER2	0.06549852	0.20874892	2.10004506	1.36973109	0.18486012	0.36466779	0.65686937	2.34525952	0.02585722
HOMER3	1.94467501	2.40032772	2.01625692	2.38671728	1.57226526	2.75203405	1.63386995	2.82380068	1.21851698
HOOK1	2.63565592	1.94079232	2.65068837	3.11713552	3.66052414	2.83993812	3.07840306	3.5596111	3.89470627
HOOK2	2.38995828	1.38803679	2.65332412	2.35006293	2.05370073	1.99879951	2.41981297	3.00918505	2.4426996

HOOK3	1.63170668	2.59120855	2.46252282	3.07380963	2.14159107	2.55911241	2.34607117	2.33747147	1.69654428
HOXA11	0.01571169	0.02689423	0.06690328	2.09478594	0	1.82485718	2.76573767	0.0479053	1.00522392
HOXA13	0.00714801	0.31576758	0.05086256	3.08754414	0.00440767	2.5951111	4.40417576	0.01374343	1.88020967
HOXA2	0.34165684	1.07500453	0.28134201	0.5725117	0.13089052	0.74495971	0.15948651	0.08326977	0.57685701
HOXA3	2.9080628	1.72914667	1.09770188	1.37346081	0.58027259	1.91203466	0.49300056	0.4645299	2.18076635
HOXA4	0.30025371	2.10038384	0.47148224	0.99831855	0.34169055	1.71336905	0.36249111	0.22304662	0.33161956
HOXA5	0.50015877	2.15656888	1.2110573	2.47141807	0.27463209	3.04596679	1.76958926	0.7716912	0.3473933
HOXA6	0	0.06356155	0.02034621	0.71096816	0	0.75377392	0.86691311	0.22242463	0.08678482
HOXA7	0.04465023	0.07589474	0.17464361	1.42812904	0.01850518	0.39157987	0.11308186	0.11743513	0.0391908
HOXB1	0	0	0.30597545	0	0	0	0.01754352	0.01694693	0
HOXB13	0	0.66384984	0.21153936	1.17567512	0.01929782	1.41858288	1.51943276	0.00404584	2.14659701
HOXB2	1.151854	2.35940851	2.78467801	2.48709106	1.70121151	2.41223893	1.25025213	0.96369336	1.16296728
HOXB5	3.13913697	1.98417601	1.75259111	2.44769498	0.70740322	1.47174883	1.46641695	1.68626841	3.36777985
HOXB7	5.80617366	2.63642097	1.71428062	3.9680959	1.13868463	3.21309446	3.41934291	3.92690009	4.48510917
HOXB8	1.22046159	0.35862316	0.15792242	0.35704738	0.50316752	0.07629309	0.07260017	0.22761391	1.07524584
HOXB9	3.60729416	1.72674411	0.54559753	1.7712251	3.14870416	2.14050869	0.06608393	0.33375619	4.28847213
HOXC10	0.03093575	0.04409743	0.04448943	3.47300014	0.02860977	0.03406242	1.80368577	0.76344848	0.01634365
HOXC11	0.01111936	0.12252215	1.07503752	1.67610252	0.10602999	0.27205203	0.13390712	0.07134356	0.37956795
HOXC4	3.31140921	1.33570312	2.72080324	1.99317676	2.48914229	0.35151221	0.408612	1.75380069	0.42864829
HOXC5	1.31238283	0.13250934	0.20372408	0.17114203	0.03684137	0.03662453	0.03581139	1.18802372	0
HOXD12	0.02682554	0	0	0	0	0.03925514	0	0.19493321	0.04207761
HP1BP3	4.37143128	4.12493171	4.18010385	4.9484144	4.80286842	4.59592866	4.24662161	3.5134026	4.79875349
HPCA	0.39747325	0.24969881	0.24285414	0.15598644	0.17000361	0.14085789	0.0747323	0.22189747	0.58514964
HPCAL1	3.46687448	2.41201584	2.94174766	2.43639674	2.47084581	2.75272297	2.77107296	2.07045308	3.59140492
HPCAL4	0.00732618	0.06176529	0.07747712	0.15842371	0.00902118	0.10911127	0.02614161	0.00846761	0.00385555
HPGD	2.32788155	3.45976167	3.10244877	2.53872406	2.42448389	1.18639863	2.19106991	2.60123397	2.47088697
HPGDS	0.33716028	2.71347909	1.57522432	0.70009883	0.4604517	0.57767886	0.21112876	0.78971152	0.04467491
HPRT1	5.2767997	4.49470059	5.43405094	5.10241502	6.22286275	4.96716539	4.85564149	4.23817494	4.86872678
HPS1	2.24515761	3.50125397	3.78881486	3.20473822	3.76959469	4.38297212	3.46483467	3.79234454	3.91217266
HPS3	2.71820046	2.55590187	3.3547825	3.19066458	3.77601005	3.3992082	3.62033451	2.61873153	2.22926293
HPS4	2.68713632	1.8488855	2.25949898	2.22348421	2.30166702	2.28250963	1.75164773	1.72009267	2.31838538
HPS5	1.91397576	2.62133615	2.91715449	1.98345095	3.76438804	2.53109403	2.44620465	2.21505837	1.9411936
HPS6	3.47235355	2.58581714	2.58706812	2.78273782	2.62934226	3.16812936	2.70868268	3.34316495	3.35519867
HR	2.23833191	1.59749771	0.51190452	3.23201551	2.52129667	2.60317183	3.5353342	2.21448631	1.9452359
HRCT1	0.37016892	2.48147205	0.1323215	0.4676825	0.39093871	0.04167857	0.62405579	1.18973049	0.30756287
HRH2	0.58715361	1.75487887	1.10612846	1.1525786	1.32810556	1.65841283	0.79654009	0.48785746	1.28024224
HS1BP3	2.70764352	2.30746022	2.34530098	1.62898427	1.68066806	2.33575242	1.85495226	2.59599524	1.83927702
HS2ST1	2.97178194	3.08296561	3.00027477	3.26533919	2.91856427	3.02441275	2.86868268	2.83143786	2.69004115
HS3ST1	4.9707045	1.80672601	3.82368948	1.66600662	2.92592265	1.75026041	1.78269166	3.69493384	1.99592557
HS3ST2	0.76401715	0.65272049	2.41973521	1.15171724	1.61402001	2.58898832	0.71751396	0.8145313	0.40177318
HS3ST3A1	1.48416014	0.68810972	0.48830098	1.06746472	0.49426158	1.34074888	0.91982661	0.57078984	0.40971741
HS3ST3B1	0.78647642	0.8890552	1.02173574	1.18675815	1.37723274	0.99970967	0.99201547	0.62179913	0.73598404
HS6ST3	0	0.27658056	0.19529427	0.0111611	0.00287334	0.2382325	0.17852966	0.01432436	0.261861
HSBP1	3.07394256	3.58040784	4.00784086	4.07314863	4.10402164	3.49339587	3.80219212	3.52752507	3.9619332
HSCB	3.02435116	2.75707512	3.04051586	2.69224381	2.68453031	2.66285907	1.87113885	2.17513532	2.516736
HSD11B1	2.57098454	4.62748974	2.04411449	2.66200309	3.83477154	2.6707999	2.43232337	0.76128129	0.72963575
HSD11B1L	1.00971224	1.10202711	0.64281279	0.90813725	0.24769631	0.69932701	0.57080696	0.92481541	0.48953586
HSD11B2	3.55237473	2.70357526	4.16622109	2.80431847	2.90480158	1.70933544	1.9866977	2.34634503	4.0307125
HSD17B1	0.53699058	0.22816879	0.16681068	0.63145573	0.20323545	0.3343776	0.63775842	0.52487384	0.32050375
HSD17B10	6.07236734	5.43781077	5.31858494	5.15244138	5.75028836	5.23467103	5.27183692	4.90308394	5.28148814
HSD17B11	4.53354902	5.46292269	4.90898695	5.04106312	4.31180244	5.56243109	5.38353372	3.06302945	6.14569241
HSD17B12	3.01263035	4.26139681	4.43153312	4.75692791	3.18351282	3.38440099	4.98372412	4.28516826	4.5285316
HSD17B14	2.8305185	2.4523654	3.84293947	2.52511385	2.50826311	2.9547517	1.9517896	1.62056953	1.59517764
HSD17B2	0.07799401	1.33845759	1.47069519	2.17985814	2.83596224	0.93188191	4.03859003	1.82691181	0.99174421
HSD17B3	0.15131523	0.04005291	0.64902977	0.11248275	0.0640762	0.04488539	0	0.0470485	0.08151106
HSD17B4	3.50799895	4.05084095	3.73785119	3.88183879	4.21374452	4.06385017	4.67110809	3.1431129	4.21462727
HSD17B6	0.59594369	1.32367802	1.17706161	1.46522984	0.57174704	1.8291566	0.60956829	0.65857755	0.1893702
HSD17B7	1.34341367	1.09309423	1.77056742	1.6737061	1.75589866	1.90006639	2.01688748	1.84868467	1.97098917
HSD17B8	2.16825372	3.03416207	2.37643033	3.17384391	1.95255324	3.03650901	1.38636892	4.87827949	3.73125137

HSD3B7	4.19005348	3.67044371	4.02639508	2.8962835	3.78324287	3.40520565	3.34711621	3.2317335	3.75561429
HSDL2	3.83497713	5.00998725	5.05889986	4.21240368	4.24443746	4.4945915	4.34103482	3.24266776	4.15371898
HSF1	4.87907706	3.71903738	4.48014904	3.94182972	3.5675202	4.28660312	4.51946348	4.48371348	4.09578392
HSF2	2.35962703	2.71812525	2.459036	2.84424094	3.06561182	2.58698672	2.37312074	1.6933661	2.57733937
HSF2BP	0.30838201	0.09525498	0.42691059	0.3083615	0.55081394	0.17182247	0.19239447	0.29822586	0.21420659
HSH2D	2.44117477	2.2196879	2.83790056	2.63552851	3.6380291	3.08944738	1.69133561	2.87202178	2.90058842
HSP90AA1	7.37600352	7.67830421	7.03132631	8.0213856	8.44544875	7.82999438	7.78924459	8.72135169	8.06702625
HSP90AB1	8.54875854	7.8857252	8.91577139	8.70207015	8.60123381	8.5794721	8.83023601	9.10725183	8.84511076
HSP90B1	6.91608083	6.97041121	7.76143662	7.913366	7.7463992	7.4734393	8.8630743	8.23550888	8.03512891
HSPA12A	0.10407523	1.53621431	0.69733375	1.25235411	0.25525861	1.08033174	0.50909794	0.64890616	0.73168959
HSPA12B	1.31217382	2.46672303	1.71714266	1.79987278	1.72096357	2.33888077	1.31481621	0.86546072	0.69175977
HSPA13	1.92402822	3.69463912	3.83998661	4.03926104	3.77976687	4.05562026	3.67126829	4.00869415	3.48393368
HSPA14	2.64228497	2.47482696	2.7869563	2.89331984	3.7203296	3.09133824	2.81851841	2.14511975	3.18541995
HSPA1A	2.6367281	4.7497277	4.95470898	4.70592936	3.03256057	3.87849523	2.44353012	5.46929813	2.02867957
HSPA1B	6.32141752	3.25236361	4.29583825	5.43907972	4.22292092	5.19009901	4.31475233	4.40090915	4.24094994
HSPA1L	0.75288956	0.83586202	0.77418642	0.55644763	0.71034494	0.65334548	0.47748176	0.63330589	0.34050111
HSPA2	1.78791148	2.74863909	1.19269299	1.83527687	1.22579232	2.320248	0.98856742	1.32870717	0.82935987
HSPA4	3.98866866	4.27309256	4.42448973	5.03920448	5.33695808	4.9097017	4.55248108	4.69055427	4.82999757
HSPA4L	0.76180932	1.63311186	0.29782673	2.18337912	2.44484037	2.73197325	2.59914075	2.49201578	0.77844927
HSPA5	7.43508753	6.82942662	8.02157624	7.58831129	7.47019912	7.42155134	8.271538	7.31398346	7.50455416
HSPA8	6.91151128	8.24555722	7.98171145	8.8504518	8.93604659	8.88595209	8.36341558	7.83354148	9.35263048
HSPA9	5.11774862	5.10568283	5.40059269	5.9015864	6.20125086	6.03112583	5.93390219	6.31510549	6.29794404
HSPB1	10.5993079	8.42771298	6.94855493	7.49248688	7.15307101	8.32325203	7.62259807	8.11834266	5.80230393
HSPB11	2.61193603	2.29385616	3.07707144	2.66003732	2.63419315	2.30647899	2.09618435	2.49480209	2.07903578
HSPB2	0.33904266	1.61834383	0.40936527	1.00747848	0.12863301	0.502999	0.59428494	0.09171804	0.09710521
HSPB3	0	0.39370213	0	0.02762334	0.02835682	0	0	0	0
HSPB6	1.31981385	7.74879995	2.74459164	4.90054845	0.83693351	5.90013285	1.72663313	1.47855631	1.1394238
HSPB8	1.30231312	4.75381993	2.4540975	3.43415449	1.02807507	4.55718166	1.31674364	1.16953208	1.20744222
HSPBAP1	1.0262252	0.99896531	1.85453443	1.44702191	1.5872281	1.27669571	0.95235277	0.99207665	1.09344416
HSPBP1	5.16321137	3.28187142	3.31376445	3.17484408	3.64017056	3.74678176	3.79134404	3.15687777	4.29854563
HSPE1	5.4500963	5.35028593	6.08965997	6.03988467	5.87372599	5.47032603	5.95454609	5.50422009	6.17207682
HSPG2	5.57144743	4.94940811	4.24703374	4.82633992	4.52820051	5.28087166	4.5662323	4.14832329	3.67221677
HSPH1	4.09435347	3.80420183	4.00613616	4.34044176	5.4777859	3.20835006	3.33062619	4.30359399	4.44537099
HTATIP2	4.61474148	3.9494598	4.21708575	3.33984667	4.41108484	4.30953545	3.94055212	4.65415236	3.98855999
HTATSF1	4.73946065	3.86128268	4.99587013	4.34118136	5.15132355	4.11633639	4.00098334	4.16530104	4.38723962
HTR2A	0.06338934	2.16964273	0.41602782	1.04388785	0.14785098	0.84437165	0.21243144	0.13804199	0.20892767
HTR2B	0.19687965	2.23402489	0.77827083	1.81434059	0.87844852	1.33623466	0.32090367	2.63373655	0.16123155
HTR7	0.0345042	0.45873978	0.77986362	0.37326012	0.10374062	0.44850859	0.2193514	0.07857434	0.11745507
HTRA1	5.40097743	6.85799952	5.63367191	6.68171644	3.77990475	6.47816562	7.28248391	4.40337907	5.15631138
HTRA2	2.59709276	2.17164494	2.28328356	2.14538245	1.67281886	2.46027915	2.33377242	2.43625516	2.55375007
HTRA4	1.06020593	0.18667469	1.07475429	0.84460735	2.21639327	0.7274432	0.62419803	0.1764863	0.26642309
HUS1	2.69718846	2.27347103	2.68014765	2.72534138	2.39464948	2.19178363	2.52168832	2.55377364	2.63982521
HUS1B	0.135643	0.04016852	0.30038326	0.30635572	0.02173946	0.24013959	0.27087798	0.13091634	0.09054612
HUWE1	4.46761073	4.50037079	3.78721302	4.16525559	4.96001802	4.49688973	4.08181602	4.81143033	4.37682119
HVCN1	2.07702512	2.10422536	2.51127118	1.80677315	2.79697158	2.02249931	2.07118432	1.00040757	1.14388629
HYAL1	1.55769431	1.23207408	1.17918737	1.80009611	1.01335949	1.22254612	0.95716602	1.8173138	0.25703797
HYAL2	3.63611222	3.6576952	3.99365493	3.57542468	2.93472114	3.95529101	3.79762993	3.57880151	3.6124096
HYAL3	0.6400088	1.01678912	0.66234946	1.82309402	0.81287913	2.15413488	1.61926921	1.50402491	1.66825528
HYI	2.14118901	1.32181193	1.7731003	1.52639984	1.37628442	1.47427182	0.91727488	2.33145621	1.64275765
HYLS1	0.55749282	1.61136568	1.44155364	2.05607537	0.72128167	1.54081044	1.26408928	1.35520721	1.13743207
HYOU1	4.93029336	3.73228985	4.28239385	4.53728018	4.43071121	4.34267484	5.83602676	4.29517057	5.16797652
HYPK	0.26960321	0.20369922	0.63582703	0.70331045	0.52667345	0.35146221	0.23560798	0.26792939	0.43847743
IAH1	2.62815076	2.67486658	2.9814964	2.70443039	2.8058285	2.7528762	2.82196531	2.65042824	2.51427453
IARS	3.90504427	4.30446984	4.99920802	4.89637393	5.48569429	4.94375882	5.08442201	3.95590661	5.19784853
IARS2	4.78353462	4.31842959	4.86343933	4.97332276	5.50865928	4.98325924	6.78260399	5.94347167	5.2975842
IBA57	1.61897396	0.63302735	0.73497963	0.75798745	0.55786876	0.90733951	1.60154911	0.87023311	1.00038425
IBTK	2.60616698	3.10627123	3.22803872	4.3332632	4.367575	3.74814	4.33662685	3.71304567	3.54668795
ICA1	1.78150736	2.54890385	3.85951226	2.44865786	2.10418717	2.85024442	3.53510512	3.57348277	3.86267678
ICAM2	1.87036822	2.36569638	2.01799838	2.07243819	1.50483269	2.83401913	1.58962639	0.98239205	1.23339028

ICAM3	0.7306389	0.93533811	1.35908496	0.63678523	1.04657693	2.00285007	0.72334621	0.56419993	0.8050422
ICAM4	0	0	0	0	0	0	0	0	0
ICE2	1.24271635	1.78932739	1.84854642	1.9127163	2.30242505	1.9887313	1.53952721	1.31752575	1.80862906
ICK	2.07059491	2.69115117	3.11097564	2.86993518	2.82612247	2.52129293	3.08857371	3.86316588	3.06853349
ICMT	4.86532706	4.51574782	3.715467	4.47587829	4.50089405	4.51011035	4.90062728	3.82908959	4.45513615
ICOS	0.65355972	0.72334732	1.71844598	1.38095481	2.53924433	1.74917605	1.01675782	0.5605732	1.07344311
ICOSLG	0.05294255	0.16520555	0.02892764	0.02935638	0.34982195	0.10965355	0.13595929	0.13546156	0.30922943
ID1	7.0270522	4.55007368	5.93054241	6.13018153	5.68999392	6.6140851	5.93616001	5.00398323	4.7489605
ID2	6.09469383	4.90998722	4.53185057	5.56500817	6.51672294	5.43770612	4.75773874	3.40592245	4.31229562
ID3	3.98595141	4.74022228	4.67396069	4.94038196	3.84685572	5.60630683	4.56499393	3.1387354	3.95123059
ID4	1.17691678	3.43335734	1.68663094	2.74755863	1.21899351	3.29956033	1.97648658	1.23008751	1.21759302
IDE	2.66522248	3.41223395	2.93194043	3.50567519	3.75442554	3.3029503	3.67404064	3.81119248	3.92419066
IDH1	5.59577361	4.25031167	5.16687665	4.6540293	5.40316908	4.78045273	6.35405179	5.67224159	4.91638454
IDH2	5.56854271	5.94304454	5.41054962	4.67458194	5.41705081	5.30819708	5.54115165	5.02572568	7.12587054
IDH3A	1.79184502	2.24948766	2.70583294	2.06542807	3.06973651	2.1201891	2.28493333	1.63851043	3.78439933
IDH3B	4.88501791	4.34825676	5.12776158	4.76535687	4.753042	4.35907445	4.62258461	3.8392615	5.75171065
IDH3G	5.35389589	4.72846672	4.34949592	4.03966304	4.65894138	4.41270527	4.34075052	4.35462178	5.02227674
IDI1	3.95743333	3.67862561	3.73235944	4.97252201	4.44656577	4.28818127	4.70219661	3.58405005	4.52581022
IDO1	3.19612479	1.94437788	2.29138564	2.97791899	8.97306685	6.04409556	2.02331576	1.20086825	1.96468398
IDS	3.42322049	3.75405286	4.25901181	3.85753157	3.80474923	3.73556205	2.97114736	3.54161792	2.96914843
IDUA	2.67372532	2.16931434	2.25912408	2.2367066	1.84287222	2.03729041	1.67646768	2.74691785	2.44140354
IER2	5.68346836	3.33024449	4.4099804	4.18886606	4.82813856	5.5622894	4.05176598	4.67131473	4.56821482
IER3	6.30381891	4.57893789	6.90582975	6.11203458	5.07749037	7.22519826	6.44557215	7.37947457	5.94291448
IER3IP1	2.94497595	3.5215532	4.37842194	4.27173071	4.60035204	4.04403397	4.96984158	3.11470675	3.72094421
IER5	2.3713067	2.67501983	2.86386891	3.60800832	2.79143748	3.11205361	3.92267046	2.83964986	2.2889973
IER5L	5.83648818	2.70935611	3.5922524	3.13271342	3.29701334	4.65313215	2.52416681	3.16095979	2.61431316
IFFO1	1.11453588	1.47646652	1.75476898	1.57850902	1.52224819	1.45757152	1.03914568	0.64110062	0.75622313
IFI16	2.74772558	5.60360726	4.32332778	5.00098316	6.21789938	5.33800467	3.48435188	5.0196191	2.7669177
IFI27	5.24260494	7.1734477	4.73836815	4.10412654	6.17263911	6.54560809	3.96418295	6.3898924	5.93326719
IFI27L2	4.97823142	3.81078729	2.83561123	2.91948101	3.1001633	3.33432036	2.88502623	3.4455336	3.29812361
IFI30	1.59752283	0.75549365	1.6134022	1.28425722	1.79029593	1.30247749	0.94137103	0.84215288	1.16113271
IFI35	4.8173717	4.36893087	4.09318764	3.73400705	4.79671796	5.064779	2.81233396	4.05558842	3.87004254
IFI44	2.06115736	5.3628467	4.53493057	4.34907772	5.53641731	6.17733879	2.76918447	3.38671158	2.65915095
IFI44L	0.71493647	4.97143484	2.8879116	2.79052162	4.36331642	4.63536677	1.27361746	0.75076672	1.17493467
IFI6	8.8216762	9.43760683	8.54114025	7.17998339	7.0960819	9.53624251	6.14114323	5.99642928	6.15752318
IFIH1	2.07119542	3.94422065	3.30854523	2.92420379	5.09797476	4.7869664	2.43606659	2.9814926	2.9150382
IFIT1	0.80755257	5.57999506	3.3231693	3.68082377	3.26697223	5.44124694	1.98792793	1.44481546	1.39463945
IFIT2	1.30861698	4.2756284	3.03522442	3.60813092	3.94969153	4.79243424	2.27372897	1.79595933	1.76938364
IFIT3	3.3458141	6.34493427	4.53936577	4.91281592	6.51239919	6.40526178	3.62006295	2.96837148	3.50860257
IFITM1	7.97890473	8.64044713	6.39185038	6.95747513	8.47856648	8.54352925	6.66658727	6.82494515	9.25159766
IFITM3	9.54469014	9.69848198	8.22521573	8.47288415	9.10655143	9.43667961	7.23180775	7.72600509	8.88982887
IFNA8	0	0	0	0	0	0	0	0.02678846	0
IFNAR1	2.87548194	4.04608619	3.8197699	4.09825642	4.0840605	4.06061646	3.90491367	4.33843448	4.0102439
IFNAR2	1.81944636	2.63655335	2.98876328	2.48846685	3.80062739	2.91876521	2.30146312	3.13287164	2.08716303
IFNGR2	4.54649772	4.39088785	5.94435613	5.39610527	5.57074281	5.38333347	5.29777269	5.69020507	4.86384525
IFNL1	0.08263971	0	0.74972369	0.19090404	1.13651824	0.40589949	0	0.06411574	0
IFNL3	0	0	0.03375266	0.10919994	0.24081654	0.40603715	0	0	0
IFNLR1	0.13421566	1.06178973	0.83367802	1.4260334	0.49666565	0.94681125	1.37869937	0.17952121	1.53010375
IFRD1	1.75476637	1.5996473	2.22420951	2.38168145	2.84399204	2.45263978	2.67655915	1.43601111	2.19975301
IFT122	2.68760684	1.31941118	1.73782043	1.27704614	1.39924135	1.51893076	1.78515097	1.88390936	1.30309689
IFT140	2.09496154	1.67418376	1.08063668	1.23202548	1.11251327	1.19591251	1.34395961	2.13346605	1.50519808
IFT172	2.52382392	2.08342221	2.00942027	1.64227433	1.49807186	1.36423164	1.80521095	2.38973513	2.48407679
IFT27	2.01907332	1.29004856	0.95010658	1.25411496	1.61246881	1.2644068	0.61938596	1.6528109	1.40851149
IFT43	2.53947526	2.49230376	2.21400939	1.83232396	2.02500717	2.03999807	1.62656409	2.02297232	1.89825878
IFT46	2.19010906	2.9030307	2.38947339	2.84222395	2.32390835	2.65320564	2.20909259	2.15673333	1.87316714
IFT52	3.00467734	3.7408651	4.08348185	4.0585504	3.11415312	3.49924119	3.81936923	3.05450512	3.67278686
IFT57	4.49348443	3.70334682	3.58330784	3.30301962	4.26542142	3.49788703	3.56136428	2.97004823	3.50577189
IFT74	1.67194281	2.36571268	1.62531395	2.37785765	2.70220108	1.96235928	1.48756216	2.04147971	2.43176177
IFT88	1.17155588	1.61130827	1.39377572	1.76268905	1.55981205	1.61046081	1.47594464	1.36943497	1.85011486

IGBP1	5.42364467	5.6005257	5.17547871	5.52546642	6.0960521	5.06699111	4.69713093	6.05274682	5.92098935
IGDCC3	0	0.0804359	0.35548272	0.01176674	0.02007907	0.01200923	0.01174038	0.02508081	0.01031435
IGDCC4	0.28451161	1.64884193	1.07691372	1.23437153	0.38368694	0.89680911	0.84527132	0.46987598	0.29367065
IGF1	0.03999983	3.43509216	0.58593049	1.8914039	0.10698406	1.8096222	0.13141101	0.13898752	0.08508791
IGF1R	2.64579795	2.28518781	1.94204557	2.28210355	2.05895276	2.83253256	2.74486784	2.06668834	2.08264447
IGF2BP2	4.8365212	0.9637687	3.36736647	3.66809924	2.65340766	3.60100184	3.95974581	4.40113105	2.8424416
IGF2BP3	1.66434645	0.09262076	0.31800009	0.07264309	1.80339621	1.52394226	0.06515203	0.08180688	0.81412911
IGF2R	3.59047846	3.66005275	4.24892857	3.17614816	4.33811293	4.0382535	4.54563789	4.14447301	3.47055053
IGFALS	0.01534132	0.12677846	0.51855145	0.08096404	0.0375134	0.49416583	0.13223623	0.08090975	0.0080843
IGFBP1	0.021881	0.12106467	0.12211256	0.18590584	0.04018233	0	0.08954795	0.082568	0.5596829
IGFBP2	7.44759901	3.2353049	3.81225753	4.68549655	3.33045024	2.84281608	1.42120279	1.03549753	1.63886056
IGFBP3	4.45185195	4.79256239	6.30664744	5.21647657	4.003117	5.49959189	5.82511574	5.06874719	4.75779362
IGFBP4	5.99258803	9.93098162	7.02382713	7.84189253	4.90121651	8.91242309	6.8825787	5.67256986	5.08194974
IGFBP5	4.71251805	8.35670681	5.78257845	6.20892363	4.36315154	6.79294172	6.38497798	3.97183814	4.78251115
IGFBP6	3.25285424	4.22364694	2.64412418	3.05096075	1.52296215	3.21754596	3.05222228	2.56317118	1.42324145
IGFBP7	7.23426873	9.23732271	8.57765578	8.04674318	6.99988847	8.78900883	7.53516765	6.80109865	6.08493546
IGFL1	0.47943871	0	0	1.48206489	0.6476721	0.11701554	0	0.08855602	0.09635486
IGFLR1	0.97322554	1.21834078	1.67598692	1.1036017	1.89792216	1.50862725	0.60566368	0.51915981	0.70208661
IGFN1	0.0060035	0.14105085	0.05333122	0.03212372	0.00185187	0.00331182	0	0.01384548	0.01102559
IGHMBP2	1.96611647	1.46659714	1.74725751	1.5971627	1.77670255	2.06004794	1.65836402	1.54976564	1.7457862
IGIP	1.19265662	2.55147943	1.36628738	2.32742251	1.50393647	2.07848795	1.52626301	2.56669266	1.70563398
IGSF21	0.06919537	0.8077376	1.10278582	0.75092877	0.02166951	1.53828222	0.90874253	0.95642738	0.43150669
IGSF3	2.98668239	2.10588245	2.62108036	2.01193602	1.7410395	2.4000941	1.70491549	1.87407302	2.36670148
IGSF5	0	0.03885991	0.19487843	0.1187427	0.01684592	0.08820364	0.00820912	0.09735181	0.11793812
IGSF6	3.28074619	2.0758701	3.46060036	2.54921418	4.04367852	3.06680101	2.59025529	2.16933061	2.27789909
IGSF8	4.71488966	3.11144341	4.26207743	3.1772373	2.86990745	3.49212982	3.83397883	4.38938122	3.91532142
IGSF9	0.31931582	0.56162013	1.08454161	1.04334833	2.6209233	0.63649811	1.29339239	0.6385296	1.21427218
IHH	1.95774993	2.33991472	3.70429389	0.970081	2.83256247	1.82261611	2.56705603	1.82030248	3.89807487
IK	4.50878696	4.45898369	4.37671572	4.55020386	4.64378164	4.49269501	4.28396461	4.66427638	4.03231962
IKBIP	1.93650019	3.12487382	3.07868413	3.15028485	2.71506908	3.19644048	2.968823	2.59903111	1.52861191
IKBKB	2.21308648	1.79623347	2.00316956	2.2558109	2.30306062	2.26360309	2.18740666	2.09397565	2.32552157
IKBKE	3.83530758	1.68941365	3.19232282	2.184632	3.28205068	2.41046526	2.71730763	2.53224601	3.3654837
IKBKG	2.11390726	0.91506997	0.9760648	0.68692098	1.00074498	1.05908785	0.8037168	0.69421787	0.85216955
IKZF1	1.47186081	2.4761198	2.40487965	1.74612243	3.21545422	2.78217198	1.71220765	1.12184151	1.51498492
IKZF3	1.66598196	1.56304348	2.09844953	1.41211748	4.05132164	1.88561915	1.03889897	1.07659938	1.60750058
IKZF4	1.49856346	1.36215485	1.4526665	1.42623976	1.44670316	1.46043483	1.13421869	1.31874644	1.38031388
IKZF5	1.57599575	2.6025406	2.86776178	2.90152041	2.88622916	2.82028832	2.30669577	2.58553242	3.00094753
IL10	0.22160182	1.84678292	1.01074373	0.7369209	1.89921149	1.56630746	0.42656011	0.25929445	0.39562813
IL10RA	2.66697203	3.32964384	3.51893532	2.67282926	3.82370049	3.56139127	2.7042317	1.95058038	1.99312011
IL10RB	3.75455192	4.21346331	4.30030113	4.10067355	4.33894815	4.32463611	4.34248358	4.26581513	3.87169636
IL11	0.84354436	1.63820731	1.15256415	0.9103553	0.76976703	1.12302096	1.24148443	0.26799361	0.55805116
IL11RA	0.58760529	1.55810948	0.79846993	1.13245662	0.98826919	0.97445595	0.43728949	0.46778091	0.97592074
IL12RB1	1.69831862	0.99262123	2.15728381	0.86242755	2.62877932	1.54482026	1.14194924	0.75006021	1.35624372
IL13RA1	4.47547172	5.30624822	6.56305603	5.28209077	6.18204261	5.25859051	5.23759645	5.13328964	4.73666079
IL15	0.29275487	0.86478237	0.77443586	0.69678721	1.41527628	1.0772627	0.52471373	0.358879	0.88733526
IL15RA	3.95813524	2.75141877	2.68400923	2.55160466	3.62123891	3.51641705	2.82643706	1.76121555	3.28197788
IL17B	0.11562042	0.60951224	0.50790126	0.26332688	0.15789377	0.19613592	0.12089512	0.15388209	0.12130621
IL17C	1.05714758	0.11629055	1.53893317	0.15043634	0.13955719	0.64030053	0.27303826	2.12458532	1.20761468
IL17D	1.32389044	0.9393677	0.28620052	0.47218778	0.52576091	0.51282601	0.56290809	0.14832168	0.60676286
IL17RA	3.02208029	2.48395809	2.82980963	2.19777257	2.66347044	3.4579147	2.49157139	2.96370264	1.98642318
IL17RB	2.52722976	1.51718471	4.1349234	4.09960599	2.06211574	2.61293701	2.80282288	4.00335134	3.31426632
IL17RD	0.28917547	1.34176742	0.28631252	1.17545026	0.4311683	1.13233403	0.32363142	0.61980393	0.32671438
IL17RE	3.77337195	1.62124802	2.7530438	2.40628912	2.13685293	1.13549433	2.7276567	2.54109208	3.57274652
IL18	3.71384947	2.87094361	3.46038803	4.82632538	5.04780139	4.2488756	3.50921171	2.67678074	1.80780297
IL18BP	3.49778581	3.04556549	3.24704147	2.7607933	4.33589782	3.42306328	2.36124155	1.67317779	2.42763096
IL18R1	0.30793545	1.32337103	1.91347503	1.22464498	2.98899849	2.20140045	0.53419211	1.25505112	0.90255296
IL18RAP	0.61972043	0.66487458	1.31205016	0.58570153	2.13577993	1.33928851	0.42941812	0.43428888	0.93605446
IL1A	0.41469525	0.84564673	2.04233137	1.62126461	1.93112551	0.08566805	0.46931601	2.07025768	0.78675617
IL1B	2.31903444	3.245318	4.6892926	4.25201778	5.21299333	3.91068627	2.60515387	5.12155956	4.02629474

IL1R1	1.86645871	6.03064391	5.10256262	4.89387408	2.86599812	5.20361497	3.61182638	2.61589841	2.04049846
IL1RAPL1	0.00990186	0.30187912	0.29149004	0.23086825	0.01219027	0.16538089	0.00593548	0.03406027	0.03610329
IL1RL1	0.0498694	1.97775715	1.67571544	0.96314307	0.37620269	0.50915714	0.33705015	0.85865025	0.14272845
IL1RL2	0.06732453	0.30489326	1.28845093	0.42645959	0.05007033	1.26344594	1.27745184	1.16175847	0.0706933
IL1RN	4.17728057	3.01424852	4.1605138	5.36582504	5.9448656	4.89655797	4.46591046	4.71719911	3.19348315
IL20RA	2.06640771	1.9268354	2.18090312	2.28412496	0.05129056	1.28939821	2.76472047	2.14037248	2.58693569
IL21R	1.4307246	0.66442464	1.14522944	0.52407991	1.70117367	0.87735364	0.92099715	0.38550918	0.92683573
IL23A	1.26580375	0.52491709	2.29887393	0.86169187	1.6494004	0.83977268	0.51918341	3.11728638	3.41029579
IL27RA	3.49966495	2.27638734	3.33197244	1.99943559	3.33100733	3.24624524	2.24087863	2.49520607	2.88171795
IL2RA	2.03913548	3.09734468	3.34806009	2.68387139	4.41447988	3.19065932	2.58365855	2.07581407	2.1289454
IL2RB	3.51507583	2.21860035	3.04774612	2.41171039	4.34978864	3.69176711	2.80479608	1.712223	2.81157952
IL2RG	4.39098116	5.36185556	4.90479031	5.36578209	8.40965028	4.82993261	4.9548252	4.21555283	4.25725407
IL32	5.4596134	5.31343574	4.15383531	5.66564561	7.10528392	6.84096333	4.69171947	5.40413462	5.19256226
IL34	0.63629513	2.14028508	1.70300709	0.99344795	1.14577392	1.55637599	1.29231188	0.91465981	0.96453722
IL3RA	2.5939684	3.025359	2.69015912	2.33133662	2.75524783	2.77408781	2.20967122	2.13536902	1.62343366
IL4I1	4.0846785	1.59779367	2.70141308	2.1510695	3.0486116	3.17394185	2.62214306	1.94310156	2.32629367
IL4R	4.47490081	3.77840988	3.85248886	3.64289207	3.63423791	3.95380662	3.94590133	3.787184	3.52957154
IL6	1.73110917	3.28836646	2.801534	3.0434598	2.9210423	3.68596335	2.38931302	1.55291685	1.52628191
IL6R	4.68797311	2.16786604	2.35475978	1.76965622	2.32149514	2.81668625	1.43864716	1.08066634	1.14400868
IL6ST	3.08343388	6.29703745	4.67411839	4.68365785	4.77266057	5.26223178	3.76650054	4.70235738	3.26129602
IL7R	1.5260884	3.32564147	4.05967303	3.74117875	3.04701165	3.73341834	2.45132196	2.87196326	2.43351058
ILDR1	0.08390389	1.78865734	2.73176535	1.60291022	1.20928519	1.59354696	1.64155724	0.58607863	2.80239984
ILF2	6.00981106	5.80930273	6.20997105	6.58838152	6.94237153	6.31579681	7.0537642	6.86218112	6.61832161
ILF3	4.56537896	3.7339021	4.18934144	4.56619835	4.91790037	4.4082038	4.25832278	4.44107222	4.96325553
ILKAP	2.27779286	1.71268196	2.29332172	2.45644902	2.6644051	2.24522049	2.05108361	2.00583594	2.30316727
ILVBL	3.99304817	2.74201254	3.14507612	2.44293218	2.04872692	2.91444269	3.23428427	3.43488162	3.37755269
IMMP2L	2.56319479	1.56346065	1.42801678	1.43390137	1.44361798	1.29778935	1.49675158	1.16824038	1.33239865
IMMT	4.22210326	4.44775771	4.39606634	4.62553019	5.00033824	4.75444068	4.86408384	4.6663146	4.89896271
IMP3	3.74515851	3.37828635	4.14505762	3.98197957	3.86755542	3.65549588	3.69685657	3.5201413	4.34408098
IMP4	4.1890543	3.29603114	4.31353944	3.8001462	3.80015493	3.92533886	3.98262777	3.9887428	4.29598371
IMPA1	1.84016049	3.32093428	3.33077927	3.45328032	3.90868169	3.41595591	3.66480388	3.21917726	4.24555708
IMPA2	4.82184594	3.54214432	4.70135478	3.33252705	3.41507502	3.15002849	4.03306003	4.08189819	3.90092326
IMPACT	2.56455168	2.80840403	3.55148589	3.59918173	3.52665184	3.18111609	3.89317549	3.21401282	3.11389078
IMPAD1	3.53956656	4.28919522	4.22480283	5.28693527	4.24550957	4.93160952	4.80847424	4.41856805	3.59273604
IMPDH1	2.94453776	3.17837627	4.69093337	3.94239254	3.13161564	4.47738525	5.52297891	2.30070498	4.37030777
IMPDH2	5.31036156	5.67861656	5.10990369	6.28688794	5.54948154	5.86543326	6.25810779	6.11146002	6.75109436
IMPG2	0.00871656	0.11081577	0.04048354	0.06412095	0.05028084	0.0659298	0.04127259	0.11958319	0.04077875
INCENP	3.48827839	1.98194971	2.47020852	2.63802175	3.00252273	3.02948328	3.11391004	2.69910415	3.25318658
INF2	4.62278999	3.84471319	3.00929931	3.66466703	3.4098029	3.87539417	3.80658401	5.2976252	4.14032965
ING1	2.79734741	1.74664557	2.24135516	2.19615561	1.76331151	2.31143615	2.3711163	2.03030468	2.58875989
ING2	3.30381898	2.70713093	2.37792968	3.59644798	3.46754424	3.32219445	2.96886618	3.34329172	2.61167499
ING3	1.37248036	1.63006779	2.06291103	2.17170906	1.69001429	1.76991152	1.74630816	1.27006097	2.19475367
ING4	2.92280491	2.80463406	3.14479246	2.49480299	2.32317936	2.33353889	2.27867012	2.30121025	3.22084504
ING5	1.46418651	0.70796539	1.45677415	1.38805006	1.40763639	1.17259344	1.52841857	1.27505779	1.63690803
INHA	0	0.09225462	0.43720325	2.09650502	0.0253904	0.05250407	0.14210623	0.92582263	0.01088684
INHBA	3.56843904	2.44403978	2.72769586	4.53082767	2.82893659	4.2442024	4.06628088	3.00531454	1.67734675
INHBB	1.23641452	1.62385091	1.50582692	1.78869868	0.61756812	1.72379926	0.92351955	0.96881135	0.92233466
INIP	2.88132428	2.40921447	3.2441909	2.83809908	2.89848762	2.6352228	2.46729219	1.98657687	3.05540941
INMT	0.23828025	3.07662602	1.37860844	1.0359749	0.21578937	1.49149754	0.76067033	0.67013252	0.46214411
INO80	2.39723151	2.54336851	2.7092293	2.7442072	3.29500228	2.92939287	2.63029397	2.69514472	3.25998352
INO80B	1.69630172	1.12359147	1.27914755	1.52709132	0.87043797	1.05193891	0.87545072	1.92430057	1.7200642
INO80C	0.64281395	1.18214482	2.12769169	2.11708852	2.07290723	1.40703219	2.23976709	1.26398248	1.44128644
INO80D	1.39273678	1.62148072	1.88189874	1.98891762	2.16765985	2.2590697	1.98954846	2.15139839	2.20373716
INO80E	3.21959415	2.26400425	2.39568996	2.19674507	2.11083566	2.33444569	2.19342018	2.36060979	2.63408477
INPP1	3.31432425	2.98047964	2.48859413	3.09159135	3.31095086	3.54041239	3.02599051	2.62715731	2.78935251
INPP4A	1.81126801	2.01181548	1.81737463	1.93978707	2.14827853	1.85729072	1.95285182	1.15755963	1.33542498
INPP4B	0.53407205	2.26064156	2.17067765	1.57998247	2.83682034	1.2372787	0.87451634	1.9833131	1.63606073
INPP5A	2.93599607	3.08222764	2.96546078	2.94538128	2.36659621	2.75175033	2.96609851	2.64244122	2.94690093
INPP5B	1.61725	1.25424022	1.83468822	1.62883719	1.85222474	1.41080913	1.02478102	0.71657514	1.12770084

INPP5D	2.14380211	3.48685564	2.95940439	2.02166219	3.46243358	3.42885066	2.12035734	1.65640363	3.16498588
INPP5E	2.56918588	1.36355504	2.40503445	2.14859938	2.37088162	2.09726062	1.59374638	2.23109021	2.50169779
INPP5J	2.16641738	1.51323726	0.88285884	1.04209569	1.10578965	0.77909343	0.78348274	1.02418335	2.16727218
INPPL1	4.63411775	3.56650131	4.08458811	3.42352931	3.34004356	3.98513251	2.89517768	4.30497065	4.2808039
INS	0	0	0	0	0	0	0	0	0
INSIG1	4.947539	3.56203368	4.31727966	4.42207463	3.2884064	4.18731211	4.77713962	3.39400641	4.88440451
INSM1	0.05063158	1.34748559	0.1417209	0.05311325	0.02361445	0.06479033	0.1112708	0.01972542	2.57447825
INSM2	0	0	0.00871247	0	0	0.0132717	0	0.00465449	0.00634409
INTS1	4.66804189	3.31514074	3.38175232	3.07681042	3.46929881	3.71874641	4.12612926	3.74541684	3.89153313
INTS10	2.51375839	3.20933608	2.87463683	3.91152768	3.61321458	3.5745907	3.9616471	3.00853942	3.52824073
INTS12	2.6603834	2.8548471	3.1980164	3.18541545	3.60662527	3.11074973	2.70612202	2.59080235	3.13908948
INTS2	1.66422626	1.64173672	2.31568924	1.71293497	2.42878442	1.99336589	1.87383933	2.24617484	2.40167603
INTS3	2.45529644	1.9519807	2.40926624	2.46862806	2.75803835	2.55292918	2.5656012	2.91966275	2.64512293
INTS4	1.80029682	2.05454335	2.29370147	2.20962249	2.24197631	2.46949058	1.75385543	2.37354203	2.26943403
INTS6	1.03263932	1.36280079	1.3034134	1.27216727	1.32272776	1.64601443	1.29487262	1.45221543	1.82002529
INTS7	2.41241577	2.44241468	2.98440813	2.85220474	3.48013823	2.93772364	3.5456271	2.80399468	3.35588949
INTS8	1.60678837	2.54638624	2.81594072	3.0582134	3.2630685	2.98904235	3.17380108	2.35909058	3.10938681
INTS9	2.24302734	2.66840315	2.24584498	2.78306938	2.51227269	2.9782692	2.63606371	2.65624232	2.70003836
INTU	0.41844638	0.54085028	0.642631	1.04683274	0.76087782	0.85089182	0.66660034	0.74842524	0.34042782
INVS	1.07975366	2.05247455	2.02536448	1.81750481	1.58399492	1.91300762	1.55151926	1.62015364	1.59659365
IP6K1	3.87101704	3.43934022	3.5983451	3.34035201	3.28153132	3.87383367	3.75723947	3.89147983	3.40374508
IP6K2	3.79938023	2.80096955	3.74552143	3.34289147	3.18137386	2.82677194	3.1018361	2.86650787	4.17799954
IP6K3	0.09978489	0.14084486	0.81284378	0.03066451	0.03147786	0.07399153	0.15384353	0.29597097	0.0467529
IPCEF1	1.5100876	0.58362344	0.92455752	0.37506083	1.08298535	0.70954193	0.21043327	0.15839642	0.32996512
IPO11	1.45532737	2.33990688	2.73113679	2.76319833	3.0120367	2.82197253	2.04496966	2.36644276	2.53666198
IPO13	3.01560446	2.93437488	2.98203327	2.54357821	2.62338106	2.98195894	2.29196353	3.26454681	2.74418587
IPO4	1.13138295	0.72771534	0.77772466	1.11259801	0.85943312	0.91823385	0.73764334	1.05739527	1.06164811
IPO7	3.32032862	4.81926715	4.19046682	5.24942345	4.75359685	5.16314244	5.25503873	4.90498941	5.13756614
IPO8	2.6367741	3.37057726	3.06028312	3.44911067	3.54565521	3.5506973	3.41549024	3.99373714	3.64532832
IPO9	3.086935	3.04717406	3.18874948	3.44743405	3.562499	3.39432891	3.84808309	3.39369329	3.45311046
IQCC	1.17894124	0.63667099	1.41386775	1.25971895	1.2932082	0.800446	0.63154062	0.78159448	1.38566572
IQCD	0.77278135	0.3521452	0.66548168	0.53580282	0.20392463	0.28550584	0.29565478	0.74570754	0.33238446
IQCE	3.29166129	1.91945122	2.55500941	1.63813993	2.91099185	2.09481353	2.11994224	2.40634173	2.01926329
IQCK	1.59876086	1.23651123	2.19256488	1.57193455	1.95139401	1.26067029	1.04688795	1.34953689	1.4024621
IQGAP1	4.20333635	5.24099302	5.22482811	5.78326938	5.93828719	5.50710612	5.72257801	6.1073611	5.46844094
IQGAP2	3.44559787	3.31394562	4.47528936	4.0588333	4.85915393	3.48437262	4.29587552	4.55794753	3.4540277
IQGAP3	4.01502694	1.4567461	2.69576702	3.19714593	3.78965944	2.48353992	3.62503632	2.67113488	3.56634546
IQSEC1	2.43816421	3.24611562	3.15511447	2.91680272	3.03641969	3.31976488	3.02913419	3.09697465	2.39088713
IQSEC2	2.65647102	2.03523996	1.84714375	1.64873523	1.72643372	1.59190123	1.38376755	1.48103064	2.07706946
IQSEC3	0.21542915	1.08032785	0.15264153	0.70750301	0.54759352	0.79429527	0.098727	0.09716872	0.02715948
IRAK1	5.80865476	4.9805212	4.75175079	4.16967632	5.12587291	5.00502009	4.73348417	5.53851901	4.95952658
IRAK1BP1	0.48397026	1.3817523	0.54813537	1.46169839	0.60013181	1.02240571	0.79896306	0.60553712	1.17670236
IRAK2	2.42974511	2.0397245	3.1283925	2.96450662	2.44867415	3.47419832	3.15126743	2.0982163	1.52772736
IRAK3	1.91170211	1.99838175	3.83604051	2.12691097	3.09503069	2.14612484	1.55698178	2.58906634	0.56348504
IRAK4	1.70370059	2.36643549	2.96448879	2.28842778	2.75912638	2.2311908	1.71992648	1.70594325	2.23952469
IREB2	2.04459391	2.92782122	3.29318397	3.42539062	3.59770703	3.10605154	2.87563061	2.72281816	3.16313809
IRF1	4.09112864	2.63325958	3.97146332	2.99082687	5.87694755	4.56335249	3.01419109	3.51465739	4.92269818
IRF2	4.23166103	4.33475701	4.22271095	4.46943693	5.24176918	4.6921797	3.73043665	4.54364487	4.45356057
IRF2BP1	4.08666085	2.79077476	2.94583013	2.78643346	2.50425178	3.47434969	2.87115047	3.64190945	4.29599907
IRF2BP2	5.7107707	5.31967577	5.33844694	5.45797703	5.07154111	5.25822932	5.32614088	5.86126378	5.6507827
IRF2BPL	5.32920107	3.79376049	3.30154431	3.00132978	2.95554936	3.76431432	2.67028989	3.38195638	3.01863648
IRF3	3.72734086	2.550628	3.52791672	2.89054353	4.0044643	3.32374816	2.88072318	3.29500065	3.74114693
IRF4	0.5315111	0.85567658	1.46665071	1.64391028	2.24454027	0.85760952	0.54997848	0.46778413	1.55503949
IRF5	2.16963948	1.61533134	2.09722567	1.97352186	2.31213283	2.89960215	1.88866202	2.21991179	1.41973691
IRF6	3.73773729	3.21893396	3.55639515	3.94634247	3.55181012	3.41047433	4.81923769	4.98111688	4.42426583
IRF7	3.19096221	3.99189571	3.07923297	2.81637668	3.23492786	4.68756129	3.09970186	3.3748021	3.76031783
IRF8	2.48420645	3.44968996	3.90969267	3.0832466	5.01639885	3.30015142	3.7723088	1.29859356	4.8078563
IRF9	0.72466496	1.09011542	1.70313405	1.35046664	1.81496835	1.43831943	0.62112302	1.02264074	1.91796449
IRGQ	1.86356395	1.587728	2.04190957	2.10080584	1.7992722	1.92379807	2.20763805	1.92632073	2.03656199

IRS1	1.48778991	2.96660962	2.22826495	1.87493665	2.6331046	2.56085726	2.74851462	2.65200499	2.296205
IRX1	0.01948301	0.06588564	0.04189927	0.11312854	0.01203346	0.04260693	0.01169422	0.00754033	0.01027379
IRX2	2.15343318	0.09547288	1.5406215	2.00187659	2.63148522	0.09938076	2.8759715	4.08794988	0.18565944
IRX3	1.86319264	0.1236314	0.88470596	1.35135457	0.07513605	0.10210117	0.89710308	0.72731729	0.04534572
IRX5	0.23583341	0.04336755	0.23832175	0.45723153	0.03892817	0.41089417	0.59479848	2.69958985	0.21249285
ISCA1	2.81175889	3.98937852	3.7600146	3.97485948	3.47350787	3.98065247	3.20481651	3.44191526	3.97528624
ISCA2	3.00267602	2.61230662	2.63760695	2.13963027	2.5696429	2.63586518	2.22511312	2.95767343	2.87533995
ISCU	3.83674779	4.61773713	4.43613535	4.62155131	4.15464684	4.43198458	3.82613868	4.23091497	3.9958139
ISG15	5.44843949	7.58977505	5.43092797	5.55800142	6.08445725	7.78695455	5.56366193	3.98852895	4.99478298
ISG20	2.00936671	1.95350607	2.1817702	2.28993896	2.97659458	3.02346159	1.82562502	3.09333672	2.18443954
ISG20L2	3.79598547	2.94358706	3.71924488	3.65290343	3.68410306	3.75101762	4.10332867	3.93346864	3.57946778
ISLR	4.27200186	5.38350743	4.26273198	5.7075103	2.64376078	6.14175493	5.1970914	4.2506982	2.58045941
ISLR2	0.02956305	0.03715582	0.66568187	0.10703062	0.05062141	0.34918661	0.0492127	0.25278349	0.05241532
ISOC1	4.17041284	3.6551557	4.0164766	4.82532551	3.84119336	4.53751502	4.6630719	3.88130352	4.38334738
ISOC2	4.0375404	2.74528746	3.34284216	2.50576181	3.3064454	3.14805943	3.35737364	3.18581142	3.64954674
IST1	3.01911118	3.71057031	4.23727217	4.06458639	4.14459506	3.93765564	3.97513726	4.28981386	4.1399273
ISY1	3.4652349	1.97632588	2.1092461	1.94988624	2.20412131	2.06320598	1.94885675	1.91272838	1.96243275
ISYNA1	1.32636667	2.92524914	2.56383623	4.29336991	1.20065451	3.73552818	2.87681522	3.40356776	2.96290836
ITCH	2.68833904	3.38350174	3.81348754	3.93855547	3.88179599	3.62718709	3.77409233	3.55351575	4.05251787
ITFG1	3.19388617	3.70297414	3.70801507	3.48486246	3.28785207	3.37486023	4.12459873	3.29890405	3.26730521
ITGA1	1.27043854	3.65671582	3.18524869	3.01537488	3.18008998	3.71850805	2.54327546	2.41714861	3.30805563
ITGA10	0.03419127	0.35538147	0.73731676	0.59301388	0.18010858	0.32323796	0.10000984	0.69537658	0.15231908
ITGA11	2.55656797	1.42270265	2.42252081	2.8720426	1.72332856	3.11382449	2.93425845	2.35357502	1.13766565
ITGA2	2.60129545	3.0321123	4.01651901	3.70851815	4.74293186	3.39296024	4.63310972	5.91722271	4.41019736
ITGA5	3.93510089	5.38895459	4.09813105	4.93902663	3.43064286	5.9224831	4.38002373	3.60286762	3.22504981
ITGA6	4.71722442	5.13741873	5.80578482	5.87581595	6.29933236	5.46283751	5.85720834	6.15088978	6.4619998
ITGA7	0.97030573	3.0691426	1.59814152	1.75318089	0.77449979	2.71670533	0.57414564	1.01692021	0.98800343
ITGA8	0.31610661	1.66277728	2.07709259	2.56766487	0.66827203	0.59732785	0.93808754	0.71955763	0.48584007
ITGAE	1.78401224	1.32807609	2.52865475	2.04868139	3.50094337	1.87195741	1.91680289	1.06626625	2.12202176
ITGAL	2.82479635	2.43415114	2.99728995	1.99992901	4.46597564	2.76789213	3.00759114	1.48037696	1.93242488
ITGAV	3.60391972	4.69962237	4.90612508	5.2202322	4.65899069	5.08534332	5.075797	5.20222343	3.41272631
ITGAX	2.43760301	1.72923059	3.29774664	3.27072932	3.16055763	2.49423758	3.35599894	2.21127307	1.80281966
ITGB1	5.16634798	6.75336443	6.02048416	6.84894518	6.65301151	6.66537015	6.38130398	6.19186974	5.68645852
ITGB1BP1	2.68699485	2.8356298	2.72838718	2.37939018	2.79351343	2.44232261	3.06690788	2.21384814	2.2605883
ITGB1BP2	0.11148768	1.13952503	0.62972535	0.63947268	0.53151436	0.55808124	0.25383444	0.12230461	0.26917673
ITGB2	4.93014575	4.39163255	4.94608111	3.5635759	5.33330811	5.0371786	5.70194276	3.26288464	3.45537727
ITGB3BP	2.00451088	1.49620429	2.21436318	1.75672598	2.73092449	1.81896898	1.386784	1.07436678	1.88656465
ITGB4	5.44657963	4.60163413	4.99792517	4.65232832	6.29691863	5.07524423	5.23069006	6.94677127	6.51607776
ITGB5	4.61461898	4.27964538	4.23703954	4.65061208	3.51541271	4.46946121	4.94926143	4.15793318	3.27521308
ITGB6	4.26334822	1.73193816	4.18403449	2.75652673	3.27913669	3.621391	3.60477442	2.13955932	2.59461385
ITGB7	0.89727173	0.81728186	2.53552281	1.67890676	2.10060405	1.9649664	1.60965493	0.88379579	1.36207331
ITGB8	2.6641864	2.08223438	2.24091107	2.92618215	1.70623734	2.74594739	2.29694237	3.02521173	2.45775914
ITGBL1	0.97198162	1.78231308	1.49767952	2.07999501	0.41710191	1.906174	1.6942949	0.90220826	0.26563729
ITIH2	0.03200902	0.08402335	0.56115101	0.97754889	0.59585947	0.03524285	0.0064439	0.80685602	0.03916104
ITIH4	0.071591	0.05621988	0.22701471	0.08925436	0.10310028	0.03152403	0.11141588	0.29650828	0.02089175
ITIH5	0.30233345	2.88541544	1.6631942	1.79710321	0.61234706	1.94614234	1.3706024	0.99884337	0.60764427
ITK	0.6611446	1.089812	1.54475607	0.81058329	2.67009115	1.23832781	0.79318119	0.51830841	0.63459247
ITLN1	0.11792242	1.20019304	4.7318917	1.8827599	1.47713181	1.21059962	1.43448743	1.11678191	0.22665942
ITM2A	2.4965716	4.35408169	5.27899355	3.24536999	3.4733686	3.41377602	5.50367009	1.51166125	1.97792755
ITM2B	7.05712559	6.81525697	6.95778571	6.22689954	6.44427754	6.42871971	6.21437121	6.59916141	6.42743068
ITM2C	3.0572876	5.45304341	5.64383384	5.9090826	4.09380523	5.50564171	7.39192129	4.49121932	6.1244826
ITPA	3.99479256	3.63825276	4.38350623	4.19262813	3.37336119	4.11196666	3.95702104	3.13123193	4.56703455
ITPK1	4.48995493	4.1157399	3.69658303	3.87962707	4.30472129	4.7593005	4.1831806	4.06070193	4.51985279
ITPKA	1.13452584	1.46664126	2.20803839	0.989342	2.13981198	2.0156498	2.11030851	1.19483387	3.24294765
ITPKB	2.61434083	3.70476644	2.33897891	2.29560711	2.33370483	3.44753404	2.54065236	2.29386509	1.97968422
ITPR1	1.04937405	2.57064413	2.02095413	1.90699768	2.03491325	2.62263901	1.24826969	1.43315847	1.10517483
ITPR2	1.13404405	2.30494444	2.04332098	2.04899144	2.47256547	2.65277702	1.82442206	2.95031308	1.90466641
ITPR3	5.21149288	3.13113763	4.42943008	3.46640128	4.31460038	4.28939602	5.54305977	6.00384925	4.98527643
ITPRIP	2.04671743	2.62991221	2.45985555	3.38219794	2.69397829	4.05382913	3.19488844	1.97383784	2.16571314

ITPRIPL2	2.51076542	2.81214613	2.71662075	2.89554762	2.29000522	3.01075365	3.27452279	3.70945317	2.09472138
ITSN2	2.50311184	2.40580766	2.61079875	2.46784037	3.04469359	2.76351049	3.00632108	2.8127677	1.86341924
IVD	3.54836277	2.90830422	2.9383889	3.47169341	3.78575172	3.32792074	3.18718655	0.82537405	3.72326991
IVNS1ABP	4.70470242	3.4839573	4.49381663	4.50854891	4.55630479	3.64952268	5.27677018	4.71006715	4.53090938
IWS1	3.50803982	3.20498	4.16604019	3.84161541	4.12431899	3.59064425	3.6615251	3.66517486	3.8824219
JADE1	2.74246332	2.46191016	2.77054387	2.22875457	2.93096726	2.4229836	1.96914794	2.80058848	1.88169653
JADE2	3.31568567	2.73474165	3.33871026	1.99051703	3.40193958	2.62250578	1.78771781	3.3741604	2.94745351
JADE3	1.25505582	2.41092463	1.80049378	2.12447411	2.56112074	1.87739923	2.08741224	1.34964499	2.23946492
JAG1	3.85662105	3.39931825	4.43957742	4.37691011	5.50015763	3.70594667	4.37097837	3.34647873	3.86487571
JAG2	1.36935319	1.31520863	1.37880723	1.57907267	1.01734374	2.12637991	1.88366335	1.01417973	3.59854431
JAK1	5.24939969	5.56413091	5.27092894	4.61445225	6.03967065	5.44796215	5.03960276	5.47338328	5.04811962
JAK2	2.75692364	2.61286109	2.3311027	3.14397074	4.83023421	3.88776395	2.14030153	4.42927108	3.08489963
JAK3	2.13510852	1.6384192	2.0917925	1.78627787	3.01331842	2.07698549	1.2214019	1.10173596	1.45543955
JAM2	0.50311221	3.00080589	1.78986377	2.0993293	0.6857502	2.5931907	0.69541448	0.60427435	0.32784893
JAM3	1.1224205	3.95262819	2.47338491	2.8125205	1.68757524	3.07012835	2.33476571	1.71017195	1.23834296
JARID2	2.28208529	2.34413247	2.38504144	2.04418819	3.04791795	2.31425438	2.69443811	2.37234401	2.66299734
JAZF1	2.08919898	3.02399322	2.4875016	2.7547689	2.14601391	2.66195791	1.79864136	1.87920098	1.17746353
JMJD1C	2.27036638	3.28882439	3.36544642	3.28521721	3.24683387	3.18593299	2.75347371	3.15860332	3.1455827
JMJD4	1.66271907	1.06108436	1.75936178	1.29727195	1.13341362	1.45087438	1.31579948	2.02216748	1.5791455
JMJD8	4.99098419	4.56474423	3.57881723	3.95188042	3.36217367	4.00748736	4.59543726	3.93359679	4.30913414
JMY	0.90217247	2.32905122	2.45813058	2.37771392	1.48557394	2.29028009	2.01151572	2.21197854	1.87338942
JOSD1	4.60736351	4.25416648	3.96710626	4.42146589	4.4140047	4.5062737	4.5108572	3.80896017	4.29013795
JOSD2	4.12943786	2.98715857	2.74195514	2.72156425	2.37393452	3.26506098	2.41272714	3.22256274	2.74622228
JPH1	0	0.97217415	1.20725369	2.63621523	0.04045385	1.72259357	3.28578005	1.31616528	1.8682312
JPH2	0.67364107	2.80268568	0.50973708	1.18755024	0.15053502	2.61747107	0.41778039	0.19430104	0.18433471
JPH3	0.05418013	0.10174143	0.0436522	0.18942472	0.05923293	0.1313279	0.0716345	0.0488161	0.00643465
JRK	1.37949695	0.69813168	1.52223682	1.17500168	1.28028347	0.98031148	1.57382004	1.33589218	1.09038266
JRKL	1.36497009	2.07962692	2.1492479	2.54785644	2.23707084	1.86432317	2.31729214	2.01014607	2.03605171
JTB	6.51558993	5.77222147	6.78028713	6.12994965	6.12155559	6.48450061	7.29637822	7.27671747	6.44411362
JUN	5.38384777	5.30842079	5.5500831	5.1369474	3.75657438	6.19078819	4.7774056	5.72293835	5.69737517
JUNB	8.12514579	6.27196692	6.72657324	6.58695247	6.55625802	8.44465093	6.74351256	7.02315656	5.8679946
JUND	7.32803792	6.24626649	6.57987903	6.23299855	5.10560092	6.98679723	6.05940299	6.03518139	6.60910701
JUP	8.26398227	5.53370477	6.26161112	6.56881813	6.0609204	6.06611779	6.41678513	6.77907447	6.98603419
KALRN	1.02255371	1.22544319	1.73469571	0.92445552	1.70078807	0.82385506	0.39564048	1.31733988	1.18465329
KANK1	2.19497421	2.14146992	1.87813041	2.04914387	1.18204359	2.21025662	1.57273177	2.19933559	1.96195306
KANK2	2.79425706	5.14705075	3.02454926	3.63868046	2.48138747	4.49904273	3.00225234	2.31236091	1.88203624
KANK3	1.058278	1.261269	1.16288856	1.09095991	0.53206602	1.55683997	0.57344429	0.51676242	0.36533545
KANK4	0.16128445	0.32103973	0.77749793	0.66027637	0.0588544	0.73626998	0.38791973	0.16621624	0.03481483
KANSL1	2.73212768	2.07958066	2.25038592	2.33165217	2.49634699	2.34740468	2.05336861	2.72904138	2.36377313
KANSL2	2.70506505	2.2691711	3.25691498	2.82252692	2.921624	2.62525138	2.6360485	3.2107588	3.07845557
KANSL3	2.60814204	2.70768959	2.92365131	2.94948899	2.85573785	3.01811144	2.6177439	2.80145338	3.06938048
KARS	4.15713659	5.08525124	5.07538111	5.4262761	5.99419774	5.63361468	5.84873855	5.31822915	5.81362171
KAT2A	4.58231891	3.19749197	3.45234367	4.06963896	4.08149293	3.21327046	3.62929388	3.84405484	4.63258641
KAT5	3.59383214	2.70256206	3.17001283	3.01842265	3.06781264	3.23667316	3.43046824	3.15460432	3.01570432
KAT6A	3.07629093	3.25602886	2.36067704	3.34370855	3.51354078	3.62579778	3.5916271	3.28500095	2.9931151
KAT6B	1.60575993	2.09379107	1.95273263	1.95339096	1.91095093	2.009192	1.98674747	2.46793103	2.04903267
KAT8	3.14017808	2.97960844	3.04905108	3.00395067	2.87108094	2.80751698	2.764562	2.98413503	3.49831313
KATNA1	2.23921399	2.68888562	2.78639491	3.15695069	3.07562394	2.76619064	3.07551029	2.08687508	2.8333375
KATNAL1	0.79540666	1.76302595	0.96698612	1.61297088	0.98914284	1.79214506	0.76048067	0.92946031	0.85806182
KATNAL2	0.08865557	0.5307853	0.42590584	0.55480325	0.2295198	0.42597516	0.46402943	0.2176645	0.16626836
KATNB1	2.93592274	2.01713279	2.49039243	2.21656391	2.00607258	2.34420791	2.410035	2.90241606	2.94566875
KATNBL1	1.51722958	1.93669203	2.24928579	2.49820077	2.55812853	2.17769136	1.80164481	1.33314789	2.28672723
KAZALD1	0.9692996	1.25199666	1.62584103	0.76954422	0.82105821	0.87571638	0.96043626	2.59180538	1.04670625
KAZN	0.20644315	1.19590926	0.78263335	0.60086385	0.21871576	0.76953355	0.59519638	0.52189692	0.82271636
KBTBD11	0.12977056	2.37213728	1.41979153	2.62742283	0.28452784	2.56262975	1.75309625	3.01245924	2.97469223
KBTBD12	0.00625061	0.68646648	0.32302888	0.14300172	0.0115308	0.18548583	0.4504635	0.052161	0.00657038
KBTBD2	2.95179941	3.39345557	3.80801126	3.83200804	3.67548354	3.82265602	3.44669539	3.63609811	3.50389427
KBTBD3	0.3759365	1.10189447	0.87221953	1.06784757	0.59517527	0.87812163	0.95629158	0.60975876	0.88546417
KBTBD4	1.76972339	1.66210819	2.09493559	1.86907128	1.71002478	2.09773525	1.99656016	2.09524157	2.00287174

KBTBD7	0.42959464	2.05463982	1.66470678	1.86949473	1.19656209	0.97138342	1.98823188	2.32733438	2.11604924
KBTBD8	0.20749981	0.6005983	1.16653039	0.79845275	1.48579111	1.11900765	1.06707568	0.54239904	1.30073921
KCMF1	2.87842627	2.92794203	3.55027528	3.64781186	3.46018432	3.46330602	3.73023698	3.43457879	3.31309918
KCNA5	0.13650967	0.69611679	0.72404646	0.70779043	0.16642666	0.39132885	0.18956127	0.28577898	0.20383295
KCNAB2	2.34269854	2.21160115	2.72772918	1.83805037	2.74521074	2.82944163	1.90149882	1.41998755	2.39670747
KCNAB3	0.08497894	0.32509756	0.51638274	0.60677001	0.65465792	0.31169243	0.17474714	0.15126647	0.23493458
KCNB1	0.00307225	1.03141634	0.09042775	0.30340478	0.00378435	0.65957626	0.02011048	0.02118039	0.0096672
KCNC3	0.70294155	0.33925225	0.73371832	1.10225157	0.59997099	0.49528258	0.51967775	0.29996744	0.34570738
KCNC4	0.83346672	0.50052555	0.842861	0.54886771	0.59796734	0.60361132	0.38500739	1.55124029	0.41144663
KCND1	0.32459486	1.34761842	0.61756902	0.75618968	0.77866239	0.73822165	0.2605184	0.4722888	0.18257988
KCNE2	0.06773753	0.23753992	3.26238597	0.48930983	0.27178434	0.29820625	0.15717854	4.40251786	0.08837337
KCNE3	5.39189173	3.60130881	6.16119229	2.81441453	4.0037585	2.83980076	2.60675644	7.05169874	4.86522302
KCNE4	1.18918419	3.06371998	1.10178234	2.60032247	0.92169724	2.50280745	1.43174104	0.88378014	0.54639609
KCNF1	0.01583457	0.17175739	0.18338397	0.60698639	0.05768239	0.29998416	0.06521947	0.07764787	0.04936036
KCNG1	0.10345088	0.40854771	0.13401808	0.11215161	0.1452275	0.59659415	0.04736438	0.02814247	0.06880142
KCNH3	4.0521911	0.15310396	0.22794632	0.35696736	0.42544989	0.15748557	0.03036908	0.32057827	0.28812094
KCNH6	0.00862885	0.21171717	0.49303209	0.02062167	0	0.00317644	0.00517148	0.08736432	0.15512539
KCNH8	0	0.32675365	0.19218198	1.40287152	0.02174647	0.27302732	0.22918701	1.43932599	0.34969998
KCNIP1	0.05714638	0.71669663	0.23999482	0.24985222	0.11056846	0.49847483	0.09453885	0.14114916	0.02430697
KCNIP2	0.21542915	0.25493657	0.22183639	0.3517007	0.37062795	0.11332083	0.16267229	0.1574005	0.39443415
KCNIP3	0.07225403	0.65411235	0.16425687	0.50178987	0.01136638	0.36355197	0.10682805	0.15711479	0.0987748
KCNJ10	1.74952132	0.17439055	0.32389939	0.41065551	2.25021462	0.89336494	0.15610901	0.11473564	0.26387002
KCNJ13	1.23217894	0.14656703	0.33755528	0.04340892	0.19047951	0.08726766	0.0784391	0.25849702	0.22588418
KCNJ3	2.71564002	1.16860726	0.29503807	0.17455648	0.03068801	0.04167586	1.30700878	0.33494288	3.02289834
KCNJ5	0.81289674	1.14891444	1.02408919	0.70500702	1.00449354	1.37760124	0.56580654	0.35671215	0.22056932
KCNJ8	1.28930812	4.19292824	2.53366931	3.18047735	2.34796593	3.71057594	2.21992747	1.12160566	1.4695721
KCNK1	4.05202152	1.98120782	3.82101767	3.85892903	3.86676954	2.64176934	5.45400215	4.89859157	3.743357
KCNK12	0.24508727	0.1312462	0.18806473	0.07643823	0.02991435	0.09308035	0.10689736	0.10727523	0.03678051
KCNK17	0.15873291	0.47825603	0.8124916	0.37080056	0.0998334	0.25374854	0.27365297	0.22139328	0.15061532
KCNK3	0.02898926	1.81389233	0.32110333	0.7311934	0.01792742	1.7521209	0.09167909	0.12944747	0.06912042
KCNK5	4.81927744	2.42870906	3.23360865	1.67038735	3.06468034	1.70973576	2.2177334	2.84811406	4.00701199
KCNK6	1.21323694	2.42927755	2.73413205	2.19287816	1.20111443	2.1698109	2.92762982	1.92774179	2.53872151
KCNMA1	1.08194019	3.52269295	0.7327747	2.09026729	1.50516193	2.90982035	0.82368242	0.48398376	0.50251163
KCNMB1	1.07692691	3.74815059	1.59908764	2.22494061	0.98024814	3.13536127	0.78981446	0.5862251	0.56826113
KCNMB2	0.01025286	0.14563313	0.23920528	0.07809863	0.02513458	0.21393713	0.04845146	0.02360029	0.00539836
KCNMB3	0.01614497	0.15001856	0.2911144	0.20191695	0.06598051	0.10634153	0.14548218	0.29086775	0.05443151
KCNMB4	0.2277873	1.33513446	0.93915116	0.51825969	0.12775849	0.98880562	0.2272976	0.09749818	0.46614104
KCNN1	0.0091258	0.13982655	0.46519942	0.12109822	0.0279265	0.07216503	0.03251134	0.07560876	0.06123853
KCNN3	0.36715793	1.62259697	1.02963885	0.88611096	0.79186199	1.74861731	0.37879644	0.43615684	0.63840492
KCNN4	2.70540326	2.47109967	1.93733932	3.86591458	1.11368703	2.70110589	3.60663075	4.91804227	0.53821179
KCNQ1	4.40455186	4.33573841	3.38279894	2.18449206	1.84731201	2.30133401	5.42891582	4.06086936	6.07551785
KCNQ4	0.02590396	1.13318772	0.80772142	0.77717656	0.09351514	1.70829023	1.33039324	0.10364312	1.22233865
KCNS1	0.00800879	0.05859616	0.07902551	0.0473957	0.01476662	0.18527977	1.05634327	0.0516837	0.00421526
KCNS3	0.49720999	1.98299645	1.85952623	2.09988982	0.97398382	1.81791854	1.65056266	1.51108962	0.76892718
KCNT1	0.02479933	0.01956746	0.08764054	0.12911053	0.02103401	0.02053205	0.00932812	0.01438917	0.04848167
KCNT2	0.02543985	0.78362967	0.30023635	0.71591566	0.10663728	0.42352615	0.05723097	0.06006248	0.01876445
KCTD1	1.5098243	1.72602566	1.63040129	1.97037129	1.97057639	2.1818432	2.70986957	2.17088351	0.3657006
KCTD10	2.42867595	3.5372086	3.08307023	3.12179533	2.46043929	3.4942502	2.82487276	3.00098227	2.55730149
KCTD11	2.04137757	2.10282549	1.966577	2.41535634	1.0349414	2.41360273	1.99055232	1.66866996	1.80490588
KCTD13	2.24698699	1.71508181	1.67644083	2.35137473	1.94699492	1.51783093	1.31980071	2.1259705	2.000747
KCTD14	0.82100808	2.01499176	2.88552554	2.33782897	3.71475337	2.90807168	3.15054501	2.76201852	1.72885472
KCTD15	0.83430887	2.10673166	1.78595452	2.14599591	0.81364934	2.11493895	1.89894633	1.01302811	0.47374533
KCTD17	4.17511022	2.21761285	2.57347987	1.85248344	2.10288671	1.97273344	1.45824944	1.11120265	2.59402834
KCTD18	1.50590093	2.24545226	2.15247512	2.27473492	1.93281638	2.21265203	1.57299627	1.87824019	2.25525102
KCTD20	3.1739464	4.46025326	4.13756888	4.38376594	3.76543025	4.52645416	4.34463946	4.06413971	3.65526776
KCTD21	1.02673282	1.76899863	1.48614588	1.71782535	1.31467584	1.35883923	1.39338261	1.38333044	1.42423899
KCTD3	2.54296727	3.76738835	3.7317892	4.4522331	3.76427627	3.68266812	5.05555105	4.94225275	3.99464023
KCTD5	4.50236323	3.30400829	3.31225963	3.24855789	3.5781749	3.42460972	4.64311101	3.6138116	3.75800002
KCTD6	1.11717587	1.81110938	2.1692326	1.96704274	1.71037699	1.70215891	1.84897866	1.50782866	2.01374831

KCTD7	1.0593573	1.00142101	1.24813527	1.14393937	1.0058258	0.9162045	0.7913294	0.79100965	0.90025535
KCTD9	2.37576641	2.71938322	2.66122637	3.99534351	2.986818	3.85946717	4.46106665	3.03045255	2.79827596
KDELC1	2.30396753	2.65397968	2.31126247	2.87934986	1.75141747	2.30133835	2.11598175	1.58453083	1.58626709
KDELC2	3.35735972	4.02129033	3.3333706	3.14264473	4.24411105	3.60860893	3.92178773	2.76166008	2.24462794
KDELR1	7.56111607	6.67306098	7.10900527	6.9926654	7.02205221	6.88713423	7.53557514	7.10396304	7.1265704
KDELR2	6.8910761	6.70862972	7.79733879	6.20825021	6.44801707	6.24457834	7.28988623	6.97821676	6.64807071
KDELR3	5.19972838	5.38151586	6.28898347	5.55657856	4.23614308	5.79434009	6.40133469	4.79184364	4.74229633
KDF1	4.17487596	2.37080575	3.76027365	2.89058167	2.59935096	2.93290458	3.27099019	2.91521591	3.93201041
KDM1A	3.51827514	3.61922248	3.27556502	4.21669777	4.34679141	4.10475851	4.16276078	2.79351809	4.77253119
KDM1B	2.37856936	2.92906302	3.68970469	3.52463296	4.21837139	3.69511651	4.2294225	4.53709235	4.83751623
KDM3A	1.65757554	1.81792431	2.20046839	2.7455543	2.47353554	2.1007652	2.06593014	3.14374104	2.52190026
KDM3B	3.47663812	3.53019158	3.48917822	3.67480082	3.85564325	3.74082327	3.73943364	4.10073915	4.1715028
KDM4A	3.72790784	3.11812416	3.7604046	3.31969575	3.83759737	3.31100933	3.31804141	3.14108147	3.64555268
KDM4B	2.39665028	1.48359404	1.32515087	1.6119738	1.71369198	1.75123892	1.80400107	1.70139268	2.13333288
KDM4C	1.03481609	0.91444138	1.04800312	1.20547632	1.2262804	1.09496134	1.12531029	2.22600757	1.55487218
KDM5B	2.31366098	2.97416213	2.78499938	3.15117411	2.59574502	2.80218104	3.75709187	3.69468291	2.94149629
KDM5C	3.93460619	2.99376668	3.14032402	3.27679896	4.17872783	3.13299327	3.28495579	3.48678198	4.46532783
KDM5D	1.8155887	2.15913618	1.45194602	0.00461385	0.00237063	2.31622293	0.00230358	2.28564548	0.00807493
KDM6A	1.94430913	2.27770009	2.63520696	2.70622162	3.928675	2.41258644	1.54132382	2.89239	2.92515246
KDM6B	3.2683157	2.59643759	3.42949204	3.11078316	3.04653835	3.88925269	2.84054385	2.90238314	3.63456175
KDR	1.98198508	3.24662647	3.6327449	2.43160926	3.22255411	2.93144632	2.5338002	1.89618016	1.75716344
KDSR	1.89323652	3.36873804	2.63360912	3.66446714	3.66254038	3.46653467	3.95675311	2.55818845	3.26692871
KEAP1	4.90670975	3.5074724	3.97300378	3.51970637	4.04766824	4.1966794	4.00878189	4.19281089	4.22023407
KEL	0.59946169	0.09772005	0.10357879	0.39703026	0.4063073	0.12733365	0.07582721	0.09021468	0.07144929
KHDRBS1	5.82809704	5.19114799	5.19721375	5.61153688	5.98909756	5.57077362	4.98174551	5.07288118	5.84966835
KHDRBS3	0.15948036	1.6286494	0.63516339	2.49179901	0.25630592	1.12002191	2.38137177	0.99437695	0.62067185
KHK	1.63849049	1.71544212	1.22034866	1.13007367	1.67673352	1.47220099	1.18898217	1.01078028	3.33623728
KHNYN	3.0839341	2.93426964	2.50572811	2.76823962	2.94206344	2.73312506	2.44244109	3.22595239	3.31510818
KHSRP	6.45981967	4.67852309	4.84789304	4.98975276	5.8061721	5.24788085	5.26434977	5.27399913	5.63482062
KIAA0100	4.62434834	4.65840116	4.27112308	4.30270197	4.40792931	4.72470958	4.96986895	4.60281068	4.46242786
KIAA0319	0.53339224	0.01448065	0.38163792	0.14766295	0.26258727	0.06391108	0.01211639	0.07256823	0.02646632
KIAA0319L	3.19850773	2.92515032	4.61420166	2.71042709	3.09306524	2.95445706	3.16586693	2.9232679	3.44679331
KIAA0355	1.89513017	2.66726741	2.62700366	2.52064404	2.5237349	2.7841569	2.12408893	2.3142293	2.25089452
KIAA0391	0.58776712	0.70122234	0.90941915	0.73862037	0.79908833	0.58737036	0.64042083	0.73567336	0.998885
KIAA0513	1.7624229	1.81825362	2.86279611	1.16016988	2.01104858	1.46594674	1.17487111	2.48993628	0.74775377
KIAA0556	1.8918233	1.84857574	1.80024276	1.6389795	1.76359254	1.63771524	1.56548867	2.14802611	2.00024922
KIAA0586	1.07132029	1.65001647	1.40919574	1.82692988	2.06824386	1.69974338	1.68633513	1.73754125	2.01410354
KIAA0753	1.08058987	1.61054516	1.94198381	1.9844346	2.56835308	1.88099419	1.55686659	1.26791535	2.30263763
KIAA0930	4.25823988	3.51059721	3.01738117	3.00478392	2.8375161	3.45073946	3.07843129	2.56137095	3.01646963
KIAA1143	2.85976869	2.92561652	2.63517049	2.72173369	2.95031349	2.91404302	2.97149597	2.95559379	3.53694122
KIAA1147	3.00350054	2.96751103	3.14891876	2.58606416	3.07081283	2.93919349	3.16312192	3.39551127	3.66047926
KIAA1191	4.6266685	4.61997621	4.833703	4.94524644	4.50414044	4.75648757	4.63469168	4.93805634	5.01000492
KIAA1211L	2.46066238	1.73603091	1.69518144	1.3019752	1.28225953	1.52903986	0.88383893	1.71752592	2.56055769
KIAA1217	3.48417116	2.75055355	3.63996845	4.09465232	3.31371598	3.59440169	3.09383488	3.02904247	3.60966602
KIAA1324	0.20087755	1.41150623	5.50630555	1.14565994	1.93216591	0.31674441	2.73131355	5.40750503	5.44459208
KIAA1324L	0.08713601	1.24236328	0.52687418	2.06564025	0.66788368	0.94766235	0.207475	0.13064642	1.07459621
KIAA1328	0.32946218	0.74521478	1.01801629	0.76099849	0.73373643	0.74832618	0.78150373	0.49160706	0.63572496
KIAA1522	6.34164112	3.8901599	5.17037545	4.50565458	4.85390813	4.87915204	4.69556714	4.32247247	5.01607853
KIAA1549L	0.18314046	0.15871804	0.29476883	0.84878402	0.49112963	0.78017659	0.48056856	0.17483864	0.83399112
KIAA1755	0.69493145	1.28115838	1.02280254	1.14976225	0.53273877	1.31510258	1.03923731	0.58485462	0.22552811
KIAA1958	1.48663491	0.92182494	1.63580482	1.01936751	1.13548146	1.15543676	1.37973446	0.37337409	1.47843273
KIAA2013	5.66017337	4.33485355	3.67346304	4.05746579	3.93775397	4.66737362	5.06776198	3.60803002	5.51731586
KIAA2026	2.32316416	2.82335872	2.1121394	2.70351	2.7162066	2.81500587	2.41016274	3.53216866	2.51101767
KIDINS220	2.44555668	3.26588663	2.57620117	3.01218186	3.35854591	3.21802891	3.2420694	2.99389572	3.27818685
KIF11	3.51895595	2.38703298	2.5962411	3.81579173	4.53128564	3.76178658	3.57923157	3.0604529	3.53004101
KIF12	3.49716506	2.01431695	1.957281	0.8106281	0.99936998	0.7378257	0.81757781	2.12143812	4.50580931
KIF13B	4.24589784	2.8539272	2.24382728	2.86356948	3.38531351	3.29299359	3.95139813	3.39072327	2.9918114
KIF14	0.96434436	0.55119145	1.27770985	1.68246552	2.36902493	1.58371888	2.13397936	1.89844449	1.45554902
KIF15	1.6470065	1.15483373	1.92885348	1.8052589	2.72003506	1.76138873	2.35782005	1.63492252	2.29185956

KIF16B	1.86665059	2.6683653	2.18904553	2.64363981	2.47242199	2.35172937	2.75079494	2.07993634	3.41108503
KIF17	0.28735916	0.58041381	0.72175219	0.39541865	0.51070199	0.47762449	0.20866208	0.20473059	0.09133126
KIF18B	3.66368987	0.7781633	1.93465614	2.20804557	2.89703058	2.03581972	1.73234947	1.33973415	2.45901981
KIF1A	0.00294633	0.1136593	0.02952676	0.02977171	0.00181578	0.17721219	0	0.02031746	0.02001419
KIF1B	2.67771989	2.5208829	1.81049204	2.34701916	3.21225839	2.49638596	2.14836179	2.48384485	2.67514962
KIF1C	3.99955807	4.1912261	4.03864158	4.13815214	4.0334666	4.48011577	4.69300726	2.90207864	4.27437845
KIF20A	2.6547633	2.08996252	3.05299544	3.16117162	3.60110661	3.2769613	3.21577745	2.48797326	3.10074352
KIF20B	1.89863608	1.99052455	1.54236079	2.49147815	3.52230289	2.51056806	2.68823995	2.01734791	2.98995887
KIF21A	1.8369863	1.0357073	1.67711222	2.35301555	2.52045441	1.62871824	1.39571787	2.81256091	2.29449636
KIF21B	3.00421061	0.77884217	1.20873086	0.70922603	1.46527834	1.32872983	1.9666578	1.89428177	1.74685041
KIF23	1.91872551	1.42053422	2.45163627	2.97478559	3.76161356	2.84048166	2.44399254	1.31800162	2.6116257
KIF27	0.60498373	0.79707128	0.96464979	0.98257835	1.19139817	0.9673603	0.54493048	1.04583452	1.39228276
KIF2C	3.54901692	1.88237366	3.32050872	3.31224313	4.16381357	3.38609906	3.3249302	2.78861275	3.39406643
KIF3B	3.89639364	3.74442196	4.52622617	3.63844834	3.95362733	3.4423196	3.94586013	4.08757112	3.7599497
KIF3C	0.94350804	1.55752298	1.00853408	1.32777204	0.59724958	1.77128082	0.84659096	1.21026046	1.23522897
KIF4A	2.59403615	2.02192205	2.33176957	2.59341452	4.14136575	2.90394851	3.07138282	2.17781633	2.59905453
KIF5A	0.04583183	0.18441458	0.33610907	0.63629939	0.10375661	0.28090622	0.04460132	0.03326615	0.12310297
KIF5B	4.43081148	5.77927191	5.34375663	6.00860855	5.88242961	5.5840399	5.55990119	5.91716436	6.10002092
KIF5C	0.05433336	0.44981927	0.7848353	0.22374311	0.32575525	0.40086883	0.20696074	0.14903504	0.11963233
KIF6	0.01223144	0.09528605	0.27947668	0.1684789	0.02066096	0.03676165	0.0128106	0.05572132	0.00322471
KIF7	0.36504977	0.92807883	0.53493407	1.03034907	0.33145618	0.8832052	0.48453631	0.30745174	0.1899497
KIF9	1.91544906	0.81302099	1.06051715	0.98876592	1.6105553	1.02015022	1.6013811	1.28569037	1.72118313
KIFAP3	0.97020078	2.97559075	2.38805986	3.03837511	2.08683163	2.31638165	2.70813037	2.95742137	2.46500927
KIFC1	4.11248317	1.43067274	2.76549853	2.81922566	3.3013229	3.08421403	3.55794936	2.71741781	3.20370516
KIFC2	1.89349307	0.48565769	2.14226841	2.26714247	1.14914816	0.99228093	1.55829665	1.88820727	2.19727422
KIN	1.18954204	1.29548804	1.33036584	1.77790851	1.89335906	1.24885527	1.1271656	0.96370057	1.49761565
KIR2DL1	0.27651916	0.11960718	0.1634041	1.36982918	0.66510032	0.12995413	0.07614867	0.06561345	0.02270783
KIR2DL3	0.06945476	0.04019173	0.08968437	0.30651738	0.19640528	0.36324572	0.14572147	0.02716668	0.04419731
KIR3DL1	0.07496061	0.05052276	0.09847258	0.18055636	0.23293899	0.01406636	0.03038318	0.02935439	0.03988717
KIR3DL3	0.04247324	0	0.0459719	0.01287283	0.01321644	0	0.02557466	0	0.17074455
KIRREL3	0.03276198	0.15577832	0.05864208	0.11460843	0.01354535	0.20226015	0.09282229	0.01060392	0.00579433
KITLG	2.09586458	4.24455213	4.29086323	4.30518586	3.74868906	3.40638737	3.66127183	3.07767362	2.0924321
KIZ	0.86000992	3.03027243	2.81252477	2.40732466	1.70728494	1.86744862	2.71017011	2.07400953	4.5148935
KL	0.09078977	1.00738157	0.62766713	0.39478741	0.21801663	0.89321596	0.30948965	0.20671383	0.18468253
KLC1	0.95670221	1.16991418	0.84049298	1.3669697	1.37255916	1.35924989	0.8850102	1.75014267	1.10375546
KLC2	3.3072164	1.75906985	2.32400774	2.661043	2.81877712	2.78455237	2.71277129	2.98739961	2.74523308
KLC4	2.47502737	1.74822139	2.64904817	1.62268817	1.24450193	1.33630122	1.84893316	2.69058774	2.28122894
KLF10	3.51972399	5.34005391	5.42426544	5.03328094	4.87006244	5.44169045	4.1190229	4.23279665	4.05286603
KLF11	2.76481307	2.67054404	2.81009039	2.79455844	2.96399012	2.9151622	2.73721028	3.32683859	2.92877854
KLF13	4.07156938	3.39308947	3.31438284	3.50350976	3.54512139	4.28615599	4.17883299	3.37866373	3.03987793
KLF15	0.01274027	0.72719102	0.5909614	0.59538289	0.03888772	0.47620968	1.01396116	1.6726282	0.14696154
KLF16	5.06803899	2.62892651	3.07129085	2.65962195	2.92677929	3.98132569	3.45665675	3.25403891	3.53776969
KLF2	3.11038004	4.35830396	4.60646922	4.16356714	3.0893704	5.81940268	4.41124457	5.29748157	2.96902527
KLF3	3.79510125	4.42716171	4.60646318	4.84565817	4.98289956	4.85283686	5.23064069	4.85859557	5.04123698
KLF4	3.32332904	4.10317752	4.62015553	3.58164066	4.52286184	4.53974263	5.47618608	4.50207784	4.12427479
KLF5	6.63629461	5.64888466	5.61763793	6.68475074	6.33453966	5.97960291	7.22185479	7.13425109	6.75692102
KLF6	4.63757791	5.10391199	4.58879049	5.36895161	4.60523773	6.01658301	5.19934165	4.9676644	4.95099266
KLF7	1.03183041	2.60309	2.3007616	2.45899565	1.37641323	2.3535317	1.51004317	1.65282246	2.0268662
KLF8	0.24464464	0.70798301	0.48180437	0.81776026	0.27347589	0.63837472	0.20479869	0.16044357	0.22921819
KLF9	1.51347411	5.09076987	3.99704731	4.19259377	2.87088595	4.14479516	3.26912593	3.90305434	2.59563414
KLHDC10	2.83783955	3.10284616	2.96485543	2.94039246	2.77992827	2.75299705	3.2009658	3.29339922	3.76327142
KLHDC2	1.94652607	2.50450071	3.18706165	2.75989524	2.09312018	2.41839656	2.11328549	2.98286578	3.15944811
KLHDC3	5.59568	4.85980826	5.62027201	4.67845389	4.47628299	4.72570016	5.66439734	5.61178096	5.20127249
KLHDC4	1.20562276	0.7280239	1.32979796	1.25448706	1.61647531	1.17139282	1.66453585	1.44752967	1.59834141
KLHDC8B	2.55041401	2.51951504	2.64624993	2.6607307	2.18981119	3.09492752	2.18005035	1.97715285	1.56998107
KLHDC9	0.93402349	1.22504256	0.38704734	0.69315056	0.60001078	1.0258085	0.10986303	1.2855873	1.65713511
KLHL11	1.44219016	1.37345244	1.68063958	2.0369207	2.05685719	1.84717501	1.93262555	1.95013945	1.95635995
KLHL13	0.02586229	1.883489	0.27079554	0.90496683	1.68592096	0.89940951	0.99250882	0.13068333	0.64944292
KLHL15	0.86524613	2.26087141	1.56903654	1.7997854	2.01506787	2.09390754	1.84374995	1.64680007	1.21347672

KLHL20	1.64702254	2.60611329	2.41382209	2.80581576	2.40856287	2.56510098	2.72993225	2.60051189	2.1423572
KLHL21	2.23516837	2.04044377	2.05026783	2.70853988	2.278484	3.33014504	2.35786825	2.37698418	2.15753859
KLHL22	2.51178921	2.41895051	2.22067877	1.90939312	2.18499216	2.05883475	1.70579579	1.71464473	1.91872297
KLHL23	1.38884778	2.79357729	4.30650467	2.79658097	3.19017338	2.43945411	2.5572812	1.33742363	3.16487404
KLHL25	1.74181497	1.13661244	1.26034667	1.18146296	1.48322745	1.56094085	1.35646482	1.71873023	2.18411814
KLHL26	2.25415972	1.46235144	1.5964792	1.46126584	1.40138656	1.57408745	1.30208038	1.73994302	1.96382626
KLHL28	1.1806824	1.89175533	1.71577023	2.15053356	1.85396213	1.97478985	1.79638661	1.9312497	1.67060819
KLHL3	0.23757597	0.42939838	1.05821387	0.59834282	0.25298905	0.56175769	0.22294428	0.07503724	0.05703587
KLHL35	0.1702857	0.38641047	0.84518639	1.22336016	0.04817847	0.51501676	0.62289073	1.71414706	0.77056593
KLHL36	1.95653141	2.02215915	2.30624357	1.99647603	1.53583013	2.08185608	2.75179533	2.37754755	2.38350591
KLHL42	1.42598948	3.22427652	2.17933477	2.93758635	2.38301024	3.06373249	1.9607443	2.07254586	2.18047743
KLHL8	1.15244011	2.26701153	1.83133536	2.08678048	2.7848008	2.24210455	2.30445697	2.1243941	2.60216583
KLHL9	2.49025713	3.37063044	2.84750513	3.60736252	3.31790877	3.33530741	2.69879241	3.5957726	3.55324661
KLK1	0.22647097	3.7091278	0.75764684	3.48673573	0.24274278	0.75442348	1.74538693	0.18283672	5.70099549
KLK10	0.36567083	2.68491594	0.33401948	3.97301953	0.46853764	6.43167347	2.24972584	5.65041047	1.32142582
KLK11	0.44048042	1.03597312	0.92371643	3.33758666	0.78230439	4.25289494	2.44357422	4.79835337	0.17916766
KLK12	0.15606237	0.31659875	0.25824339	0.81762968	0	0.01506041	0.04850532	0.03141712	1.06239914
KLK4	0	0.18218837	0.09223854	0.04450611	0	0.08726985	0.01123068	0.02161496	1.09239359
KLK7	0.2966995	2.70417879	0.0634881	0.69837425	0.01833142	2.51336585	0.06997974	4.81706204	0.32930144
KLKB1	0.12974534	0.23438151	0.16018067	0.32364279	0.17182175	0.15465255	0.02199488	0.01420007	0.03840764
KLRB1	2.06183193	1.7942184	2.95761105	1.87950921	2.4173724	2.31447076	1.61718659	1.15300772	1.53060427
KLRF1	0.08426427	0.79491777	0.94636898	0.35236936	0.31848562	0.58584591	0.23881328	0.25481404	0.18523274
KLRG1	0.35837632	1.37457385	0.70685251	0.74563102	0.99592503	1.17705254	0.21782094	0.25233046	0.20580712
KMT2A	2.37428523	2.62688315	1.77820163	2.39681026	3.00584649	2.76400361	2.56287924	1.97651335	2.56529164
KMT2B	3.19561635	1.83156252	2.43999221	2.20920639	3.16237048	2.49279532	2.09484691	2.68255858	2.74599054
KMT2C	2.71727994	2.66171112	2.78823067	2.34210272	2.97886539	2.40003523	2.46679009	2.66297241	2.48627926
KMT2D	3.84893245	2.37766412	2.62059547	2.12029607	3.23798902	3.0062499	2.40569039	3.54899026	2.77734028
KMT2E	3.53461178	3.611857	4.15266391	3.80742011	3.85202461	3.68236562	3.44241618	4.10243716	3.39632734
KNCN	0	0.01478212	0.09955615	0	0	0.00475752	0	0.01491898	0
KNDC1	0.03605901	0.19073677	0.05816557	0.09791788	0.002809	0.16164828	0.01088739	1.00691359	0.09963994
KNOP1	2.22391419	2.23978875	2.18987162	1.08070267	2.25331181	1.33891716	1.97683789	2.17638582	2.0929375
KNTC1	1.47046339	1.22748178	2.1131034	1.97433631	2.7930337	1.50794803	1.35440293	2.03628623	2.18522211
KPNA2	5.7783745	4.65839389	5.78324386	5.89387873	6.47294192	5.74967798	5.68607216	5.00619829	5.54576945
KPNA4	3.49104561	3.85245441	4.45293024	4.15248604	3.72872711	4.45437917	4.43048401	4.4341457	3.8326333
KPNA6	3.46826262	3.88984084	3.84286114	4.0923402	4.22456913	4.06430574	3.67468072	3.25184505	3.70303781
KPNB1	5.36015177	4.6414769	4.76411224	5.26911215	5.62974002	4.8749566	5.06628797	5.03829034	5.5268527
KPTN	2.14578124	1.26915349	2.00575761	1.46968654	1.95039904	1.9114214	1.17186767	1.13213875	1.5456006
KRBA1	1.55846066	0.89278106	1.3461112	1.18229971	0.60592226	0.95799465	1.14580262	0.73943652	1.05125478
KRBA2	0.07621498	0.37893003	0.35713482	0.14859446	0.06289432	0.20099517	0.0536476	0.14799305	0.29085813
KRCC1	2.07996407	3.97808499	3.00307577	3.75136744	3.01670112	3.31366162	3.02853151	3.64094578	3.78276683
KREMEN1	0	0	0	0	0	0	0	0	0
KREMEN2	0.5013652	0.16579205	0.10675125	1.14520619	0.21504873	0.55802591	0.36004028	0.41305073	0.46569471
KRI1	2.77094101	1.87930625	2.62951848	2.57062781	2.70364662	2.71223841	2.36156446	3.15409964	2.4601991
KRR1	1.72998525	2.0161458	2.33273275	2.74670914	2.48794148	2.20539512	1.93700446	2.69801972	2.46597244
KRT10	4.5950544	3.40954774	4.04598444	4.34707169	3.35706432	3.66258627	2.74627794	3.64558098	3.42744815
KRT13	6.20321231	0.00714863	0.06192363	4.70848492	4.7921522	0.58213672	0.13570798	0.06136287	0.01309305
KRT14	1.76690953	0.14775243	0.61171663	1.15223058	0.16710935	0.15038685	0.38400062	0.0720668	0
KRT15	1.20883455	0.22890338	0.63699232	1.41345415	3.59034606	0.43917429	0.38504638	1.22574878	0.53125298
KRT16	2.36287472	0.63186184	0.21689299	2.12696656	0.54498948	2.56839825	2.44085195	0.73537214	0.08272378
KRT17	2.6478723	0.71263701	2.20516767	7.2344605	0.77531215	8.37059528	6.22720759	2.71558021	0.34133773
KRT18	7.15821759	7.37207331	7.89480211	8.31624532	8.55397473	7.70141091	7.52767018	9.12034644	8.86424745
KRT19	9.44171045	6.19549528	6.7010315	7.78137062	7.56990256	8.5370578	7.93508076	8.78722226	7.00465124
KRT20	0	5.58683715	3.59960462	1.43707251	4.98655274	0.01443535	0.76933445	1.25528474	5.94237696
KRT222	0	0.62180268	0.19405918	0.26407671	0.07590055	0.17862052	0.0137032	0.00883789	0
KRT23	0.08533566	1.3843458	2.1300036	1.60997815	0.08268732	0.46530428	0.15657545	0.9950114	0.50686454
KRT24	0.1646183	0.24442895	0.19602706	0.17494061	0.01188693	0	0.0115518	0.02223073	0
KRT27	0	0.14470053	0.12713567	0.11769813	0.01253744	0.03707068	0	0.02344273	0
KRT3	0.06153218	0	0.05568189	0.18554476	0	0.06215927	0.01869352	0	0
KRT5	3.48997358	0.10774322	0.20016357	2.39855644	0.15367897	1.24026312	0.06946239	0.02915804	0.01774395

KRT6A	4.07458851	0.16806118	0.11240768	3.73376951	0.29695062	1.41715561	1.05257402	1.00065203	0.2515316
KRT6B	2.48959094	0.90203545	2.2088093	4.62586943	0.06727685	1.26253783	0.16256857	2.73590586	0.32886765
KRT7	1.99538165	1.49241576	4.50048409	1.77782725	0.81904504	2.91197235	0.94936906	5.76796598	0.49775341
KRT73	0	0	0.01972138	0.00822139	0.16009777	0	0	0.01055452	0.01437522
KRT78	1.2363849	0.01908393	0.15311411	0.97599925	0.02728836	0.00819186	0.07179634	0.03826697	0
KRT79	0	0.008189	0.05088222	0.02557685	0.02625648	0.02610137	0.02551981	0.03277964	0
KRT8	8.34694027	7.29428544	7.76647768	8.17535681	8.39113561	8.25957181	7.97350416	8.78027405	8.54811424
KRT80	0.41252757	1.32205012	2.08085302	3.95784268	1.12944427	2.82311063	3.34334634	2.58394379	1.9836221
KRT81	0.32056492	0.03210609	0.06668297	0.23965241	0.04582068	0.9511893	0.09835594	0.30121168	0.04881941
KRT86	0.65059299	0.11718866	0.24240592	0.4711128	0.1763368	1.579635	0.53453468	0.21239818	0.09296363
KRT9	0.06229483	0.01811776	0.11062665	0.10138294	0.04820668	0.01164779	0.02830073	0	0
KRTAP3-1	0	0	0.26470019	0.02984821	0	0	0	0	0.05190153
KRTAP3-2	0	0	0	0.03081577	0	0	0	0	0.07962827
KRTCAP2	2.91770428	2.34611948	2.58933448	2.73738673	2.28339065	2.25836834	3.17933714	2.78315411	2.55998376
KSR1	1.48405804	1.6872212	1.29777709	1.26969888	1.59823969	1.21263827	0.77107651	0.84503694	1.15381322
KTI12	2.86365792	2.2499817	2.89569587	2.32609446	2.25613045	2.63446917	2.15938831	2.4725078	2.46173858
KTN1	2.74521778	3.71506715	2.74595196	3.74757326	3.78877636	3.4812883	2.93484019	2.72688044	3.67166195
KXD1	4.70506909	3.7027357	4.33853293	3.83516437	4.40640275	4.12912596	3.57200541	4.05631583	4.0256548
KY	0.09151722	0.31334102	0.19546941	0.07366702	0.00387514	0.19050993	0.00752138	0.22645596	0.04882771
KYNU	1.82279331	0.88439881	2.47038597	0.75535256	2.30428061	0.79900569	0.59571414	1.54605087	0.22434239
L1CAM	0.02916169	1.90558516	0.25905292	2.06174928	0.09084189	1.92111063	0.09386596	0.14908615	0.14941383
L1TD1	0.02813002	0.1345418	0.29025676	0.02254607	0.07390485	0.1040084	0.25874592	0.09872725	0.03927719
L3HYPDH	0.69600097	1.47927633	1.65347668	1.61909712	1.47327779	1.33823201	0.99776245	1.000225	1.77463194
L3MBTL1	0.21528452	0.37805881	0.76495513	0.54601246	0.36262259	0.23132534	0.05021248	0.39872575	0.85784936
L3MBTL2	3.50758187	2.43976394	2.95950424	2.34107315	2.95327212	2.70852301	2.46451998	2.10915337	2.83635941
L3MBTL3	0.39638294	1.78337564	1.38000162	2.03059175	2.33660695	1.96312848	1.67424577	1.61101793	2.00170771
L3MBTL4	0.22431328	0.41828703	0.47490212	0.74666747	0.94035161	0.45874731	0.14587045	1.08776862	0.38807611
LACC1	1.03124067	2.52784925	2.8296365	2.67645715	2.60061423	2.52176993	2.01589143	2.09124245	2.65528912
LACTB	2.56002077	2.51909448	3.28722527	3.19020653	4.04470406	3.07169825	2.7724099	2.00541397	2.40119798
LACTB2	3.08457742	4.65985836	4.05265911	4.36115129	3.91142177	4.66805234	4.9596929	3.9080542	4.8241143
LAD1	6.10043788	4.6807986	5.16500725	5.96425209	5.14777534	6.15524411	7.06899372	7.37177626	6.13866631
LAG3	2.53466847	1.44797195	2.50338865	1.65416901	4.52605775	3.3344751	1.86487317	0.56975325	2.55249158
LAGE3	5.42107351	4.36301722	4.29960821	3.58923014	4.019519	3.59237189	3.38751107	4.30186472	3.8043296
LAIR1	3.24568727	2.94549008	3.44862234	2.65517279	3.52366872	3.96830061	3.13808356	1.72478803	1.88101432
LAIR2	2.38352416	0.5155438	0.701694	0.46425089	1.31437017	0.27099931	0.34087613	0.10064813	2.4744504
LAMA2	0.50888234	4.35634716	1.56909148	2.54912092	1.25554017	2.81603194	1.77691295	1.17491724	0.39873038
LAMA3	2.65579495	1.72977722	2.95567476	4.31490397	5.40064868	3.36432088	4.42737505	4.01078901	1.6814576
LAMA4	2.07991606	4.35237217	3.62654104	3.99183957	2.8642147	3.96663917	3.61712076	2.74608038	2.27042924
LAMA5	4.35150691	3.05754814	3.46504663	3.24761762	2.4435235	3.47780597	3.84057999	3.97253021	2.10101941
LAMB1	3.89575217	4.9702858	4.68161935	4.90559727	4.41095635	4.64636765	4.90460537	5.5216916	4.87246751
LAMB2	4.56101558	5.1364124	4.10953414	5.06274331	3.86422611	4.92894948	4.77063453	4.64548247	3.44873434
LAMB3	5.30028851	2.86539711	4.47309591	5.20003713	5.17663838	5.20482814	6.09904677	6.35641938	4.74770327
LAMC2	3.99751677	1.80146233	4.04173963	6.00817997	6.96730971	5.21361815	6.87509924	6.71066909	3.69207847
LAMC3	0.24645196	0.40689551	1.01020079	0.5587561	0.92104844	0.26797313	1.06572068	0.2859838	0.35193483
LAMP2	4.64701887	5.59076411	6.33196286	5.52549982	6.20981216	5.66761095	5.3617545	5.52779298	4.59450547
LAMP3	2.27290409	2.08218835	2.80090245	2.31142531	4.87094423	3.49026612	1.32757465	2.34838572	2.45649097
LAMP5	0.11981761	0.74869223	0.75548364	1.00142942	1.4807624	1.52241212	0.18052612	0.21675147	0.51518534
LAMTOR1	5.55749938	5.0849186	5.77781436	4.8754476	5.12482485	5.36014723	5.03899167	5.23067132	5.00161054
LAMTOR2	5.11880449	4.29541211	4.59922671	3.91767005	4.19243439	4.27814055	4.85082589	5.24981174	4.43241367
LAMTOR3	3.95715008	3.89572744	3.49825006	4.04879529	4.29551855	3.88727219	3.60542242	4.02621815	4.02558685
LAMTOR4	5.63961563	4.3469914	3.93107768	3.84509091	3.84799585	4.1657341	4.20794701	3.95503387	4.08058174
LAMTOR5	5.26166822	4.50608215	4.93725875	5.17203988	5.2139912	4.51961875	4.67815339	4.91027617	5.09787589
LANCL1	3.66216294	3.64047119	3.56787134	4.39305702	4.37136778	3.8754889	3.92989519	3.88953958	4.17204766
LANCL2	2.5011781	2.30577302	2.68986513	2.64559202	2.49657132	2.52487222	2.77476296	2.22671317	2.85492923
LAP3	5.31385935	6.03276939	5.41987983	5.44826651	8.16342423	6.51089639	5.15665175	4.64088925	5.93803388
LAPTM4A	6.53510827	7.61603353	7.24680662	7.28202734	6.78398186	7.30941002	7.15518092	6.5152139	6.6866557
LAPTM4B	5.36415311	6.15992788	6.08982016	7.44000231	6.09395924	4.99886829	7.21087219	5.51021993	5.08141391
LAPTM5	6.8051712	7.14425503	8.01631532	6.84546532	7.90712425	8.12570959	7.86844254	6.13458822	6.21417616
LARP1	5.59719935	3.96562212	4.63637425	4.26670026	5.36166756	4.52505963	4.44452122	4.87648904	5.0190184

LARP1B	1.74553935	1.50923449	2.14368109	2.2956192	2.43730362	2.14729861	2.48343429	2.50868188	2.33602523
LARP4	1.69001213	3.0219358	3.37692236	3.65047802	3.3651875	3.34684001	3.40119991	4.21390338	3.61012121
LARP4B	2.88446351	2.37169283	2.5364328	3.03772121	3.16904939	3.199246	2.72027462	2.52903448	3.35948015
LARP6	0.49968306	1.88412301	0.80399568	1.63409457	0.21725067	1.29999597	0.71833078	0.31460627	0.21435307
LARP7	3.09248471	3.71138433	3.71445031	3.46186482	4.48601547	3.17966843	3.06142204	2.98030464	3.85210934
LARS	2.92070404	3.84517917	3.59173887	3.98298401	4.14903808	3.86338704	3.85160169	3.37941951	4.03849741
LARS2	2.55488836	2.41499757	2.55496652	2.35452835	3.29762832	2.71537973	3.14494545	2.64010552	3.3593377
LAS1L	3.04438015	2.44912212	2.42030832	2.43254313	3.83188538	2.67484372	2.35938356	2.95622996	2.84054302
LASP1	6.61218812	4.81331578	5.24291899	4.97120488	5.26176799	5.66560107	5.71138982	5.52999757	5.82613124
LAT	0.07220431	0.0824221	0.36094295	0.19369654	0.32934691	0.08224691	0.02924934	0.07114483	0.16722118
LAT2	1.81012175	2.10910155	2.964647	1.78000904	2.02065446	2.51020879	2.23259043	1.51357091	1.24323705
LATS1	2.29162908	2.90640397	2.96570145	3.24544464	3.26323226	3.19188242	2.66284096	2.36458331	3.0292596
LATS2	2.5596503	3.76713647	3.46357468	3.64308042	2.93621761	3.89136046	3.0677582	3.21286493	1.93660926
LAX1	0.51253858	1.01619321	1.69724375	1.28449821	2.29358957	1.20361291	0.49710798	0.56066102	1.14833083
LAYN	0.9136756	2.46695173	2.16444722	1.9766332	1.37233952	2.81319653	1.55970901	0.79147827	1.00373103
LBH	3.25726576	3.67077767	4.13590535	3.9062078	3.47861558	3.41943839	3.7385017	3.06899953	2.42296661
LBR	4.16964618	4.15635886	3.7775043	4.17046978	4.73307139	4.56371009	5.39829463	3.17807391	5.25458481
LCA5	0.12293735	1.33551583	1.28041151	1.26010817	0.9536495	1.09392347	0.51450321	0.9194223	0.47700764
LCA5L	0.10235343	0.29329276	0.50773633	0.48500525	0.33163575	0.15009886	0.22438493	0.29869627	0.24383897
LCAT	0.70942465	1.36851201	1.55570926	1.98619198	1.18563045	1.13663054	0.59712738	0.97950036	0.83516717
LCK	2.66822896	1.96956956	2.90984074	2.32292294	3.80194732	2.54105926	2.14233161	1.33963667	2.10324859
LCLAT1	1.61514128	1.53122229	1.7660473	2.18540497	1.9879448	1.86244098	1.83141511	2.20553111	2.34196575
LCMT1	3.59337521	3.97756091	3.5364283	3.78908107	3.7166056	3.55054192	3.66457603	3.58934941	3.96707259
LCMT2	0.9617886	1.0705741	1.274549	0.60263368	1.18021269	0.65659322	1.30540734	0.99033945	1.69463122
LCN2	11.1681954	7.91664116	11.8711445	4.9984774	4.58394889	3.71008008	8.06816288	10.0001991	10.186963
LCNL1	0.01787741	0.08987623	0.08821962	0.17748717	0.05969743	0.04233406	0.00537403	0	0.00942464
LCOR	1.8705095	2.20303042	1.88207258	2.47950026	3.03958775	2.53821477	2.53792453	2.82258381	3.30924282
LCP1	4.76554086	5.6983122	6.30029819	5.63000595	7.27786531	6.50459791	6.07225153	4.70288325	4.61071082
LCP2	2.68161056	3.27814973	4.16919798	3.85495305	4.62907713	3.84592401	3.27248687	2.84640982	2.7747792
LDB2	0.56497975	2.92753078	2.58310934	2.55407319	1.41014574	2.39013929	1.52813918	1.07386052	0.96409643
LDHA	7.94978477	6.98497281	7.09958979	7.77165584	8.49229816	7.1816761	8.07564567	7.09032087	8.29253889
LDHB	3.15536676	5.75914898	5.54506758	7.00598621	4.86185126	6.32946453	5.95688975	3.06916112	2.8894741
LDLR	4.98737726	3.36812197	4.41203609	4.17619401	4.87598766	5.40283086	4.70578834	5.80961558	3.98509179
LDLRAD3	0.58202789	2.4777475	1.37833043	2.48066797	1.0435537	2.90342304	2.37880434	0.71420474	2.22389861
LDLRAD4	0.40827222	2.06020018	1.30968209	1.21152416	1.02858743	1.64465399	0.94543555	0.73971944	0.72911854
LDLRAP1	2.39747328	2.64066384	2.29448216	2.28550019	2.22542241	2.29495088	2.95258594	1.65388551	3.30944417
LDOC1	1.67585247	4.16447897	2.18325161	3.07924998	1.04213592	3.26951694	2.00404225	4.65756545	0.87697193
LEAP2	0.18466097	0.83362375	0.77680354	0.79161649	0.63228633	0.30500181	0.29437744	0.67365847	0.7989559
LEFTY2	0	0.11728277	0.40934664	0.2814631	0	0.16387222	0.00909176	0.05755963	0
LEMD2	3.73654186	1.99357641	2.86992865	2.46326217	2.54420005	2.69639445	2.68758451	3.15758557	2.6556329
LEMD3	2.71352296	2.34165655	2.8904386	2.8796911	2.87416912	2.80444564	2.53976604	3.06637755	2.62215695
LENG1	3.13718241	1.8589663	2.24491111	1.56360669	2.04917873	1.65759535	1.7329942	1.75180396	2.0891729
LENG8	4.04499953	2.46130885	4.06593361	3.7867372	4.8544891	3.45214844	2.99934735	3.07357086	4.72847314
LENG9	3.93968371	1.81965385	2.05080717	1.34174435	2.13590219	2.20446112	1.28007869	2.62361367	2.89765299
LEP	0.02115695	0.13397394	0.44665663	0.35022151	0.10749577	0.01557725	0.05629808	0.04846318	0.03322298
LEPROT	3.09332712	4.86908955	4.99000198	4.92943911	4.02239412	4.72272188	4.27684544	4.50580156	4.11572675
LEPROTL1	3.19014008	3.7600127	3.50279292	4.2238931	4.39392787	4.1128394	4.2934202	3.68554563	3.82284508
LETM1	2.93173529	2.36299083	1.99817467	3.04410025	3.17446388	3.17267534	3.12215556	3.04313327	3.16505899
LETMD1	2.6529184	2.45382331	2.77324791	3.00679194	3.45874458	2.62495615	2.32006383	3.13688005	3.58550485
LFNG	4.15733579	3.00266755	4.3784501	2.4242865	2.81316232	3.32925408	3.6799822	4.18402648	4.0249587
LGALS1	9.16195111	8.05271162	7.27296327	7.98289429	6.29800082	7.56185081	7.61876452	6.26070566	5.86857303
LGALS12	0.01596637	0.82999309	1.19307065	0.35661464	0.05815559	0.66793479	0.23861935	0.14135378	0.02509781
LGALS13	0	0.0854986	0.07222649	0	0	0	0	0	0
LGALS2	5.80520961	2.38656876	6.66736726	1.65987928	2.08798773	2.31274531	1.60666156	1.59575708	3.75410654
LGALS3	7.91031342	7.54630247	6.97917625	7.82352043	7.84316948	6.56435095	6.95301677	8.10422194	8.25781077
LGALS3BP	8.43007211	7.64065879	8.99543723	7.48957752	7.5555853	7.5742244	5.69264153	8.3143725	7.66833907
LGALS4	9.3979391	7.25627345	6.59199538	6.21970429	6.15066381	5.46567254	6.77922099	6.98435481	7.81800789
LGALS9	5.80865721	4.67331613	4.48460012	3.1813104	5.39307223	5.25187735	4.15570737	3.85835931	5.09449103
LGALSL	2.49042398	2.38660971	2.9366833	2.71908594	2.43733035	2.7848809	2.7278937	2.4771073	1.58826145

LGI2	0.50240675	3.74549652	1.61011936	2.09273103	0.44950748	2.64789947	1.13580945	0.74071492	0.28565926
LGI4	0.22995261	1.54225952	0.62634865	0.93833156	0.46588043	1.15760888	0.31836199	0.2288111	0.34432256
LGMN	5.43721034	6.25466017	6.37892481	6.35303096	7.08445131	6.74240747	6.05355809	5.87685985	5.2613882
LGR4	3.98352733	3.95733831	3.89091261	3.51654394	4.85565372	3.67275959	4.81604565	3.70927469	4.03323792
LGR5	0.0206729	1.64494415	3.02114769	1.52988807	0.7396693	0.11278039	1.96452271	3.85524916	5.67374338
LGSN	0	0.0071523	0.1419947	0.04072475	0.01915077	0.04081052	0.0037417	0.09340083	0.01960531
LHFPL2	2.63372228	4.43242326	3.49212415	4.03855095	4.2442908	4.52104424	3.93894489	3.00963186	2.6396563
LHPP	3.02980964	2.73114817	2.56278015	2.10823235	1.30477677	2.05768874	1.87348963	1.24219598	2.24423106
LHX6	0.23363639	1.44993429	1.03183082	1.38608126	0.39403338	1.52748495	0.40731871	0.44803638	0.38142083
LIAS	1.84963501	2.11818155	2.28066025	2.52164733	3.19237764	2.16370766	1.76536767	2.10812764	2.37646903
LIF	3.08490699	2.69507656	3.85986935	2.92062931	1.90785912	4.60088209	2.89803576	3.74895454	2.80499098
LIFR	0.09796952	2.22226611	1.56625944	1.25540003	1.96064346	1.55015114	0.33635986	0.85794784	0.10432076
LIG1	3.13090382	1.91972802	2.49152404	2.35703525	3.31678637	2.4111695	2.40978426	2.44917101	2.96361994
LIG3	2.26811313	1.32242612	1.46994323	1.2844754	1.85503711	1.54901849	1.47404884	2.08173057	1.79145821
LIG4	1.23815222	2.11700895	2.4399992	2.63693819	2.60319012	2.39594197	1.92820953	1.91277752	2.14413296
LILRA2	0.42734561	0.72179949	0.75926693	0.85852923	0.39420009	2.36961046	0.78789065	0.56434743	0.33984388
LILRA6	0.99183809	0.93600056	1.17566679	1.44766149	1.60428967	1.5088859	0.55196713	0.76167247	0.36270949
LILRB1	2.0542113	1.91573041	2.32303349	1.86058085	2.84016458	2.05762716	2.04024534	0.95733531	1.2853359
LILRB2	1.98566276	2.78555499	3.21856565	2.69513419	3.29099156	3.12722333	3.07270755	1.50800912	1.73287021
LILRB4	3.11449249	2.42055567	3.07297064	3.2081095	3.94338884	3.3787898	2.76446156	1.87981206	1.92968509
LILRB5	0.46258408	2.19074244	1.82313611	1.20449799	1.36063727	1.32726006	0.45940484	0.31361682	0.52508785
LIMA1	4.13223884	5.13999754	5.01298362	5.34346588	5.80173312	4.85861235	4.5378609	5.93436829	5.47387046
LIMCH1	0.27972581	1.29790268	1.29576864	1.68953526	0.46132743	1.39763056	0.72624069	0.54846312	0.9920291
LIMD1	2.03128895	1.59446462	1.96893954	1.36391834	2.34151056	1.81295143	1.99172088	1.85897802	2.47561108
LIMD2	3.02396045	1.87511737	3.0848938	1.91061217	2.51424119	2.79630015	1.85711088	1.27985022	1.71031224
LIME1	0.75150871	0.28842257	1.21220128	0.61256116	0.60287856	0.28498033	0.44544867	0.57407977	0.68171149
LIMK1	3.60791715	2.84280323	3.59163448	2.60904871	3.28683991	3.06489944	2.88104778	2.71635525	2.70817673
LIMK2	4.10373993	3.01940011	3.90774808	3.81786613	3.86764727	3.39105725	2.86957879	3.65896194	3.39636899
LIMS1	2.90812276	4.09969197	4.19032043	4.31164636	4.1788784	4.34591614	4.74251134	3.45380586	3.29978934
LIMS2	1.08534549	3.03292255	1.38539633	1.5674944	0.73115186	2.79227868	1.01486171	0.81967526	0.47292623
LIN28A	0.03592129	0.02068497	0.20766898	0	0	0.00333713	0	0.00699232	0.01426815
LIN37	0.78710032	0.59262252	0.70862168	0.78255505	0.90877425	0.68608169	0.28671107	0.57557479	0.63749445
LIN52	1.92542804	1.76825737	2.40348003	1.86701454	2.40584206	2.0159499	1.78459289	1.91418595	1.757417
LIN54	2.2607015	1.85475164	1.84130891	2.16923978	2.83207851	2.29246671	2.33011731	2.27702859	2.23574342
LIN7A	0.18121318	1.1409695	0.79124123	1.03600558	0.41616733	2.12712775	0.4629146	0.33923026	0.18248491
LIN7B	1.19972006	1.08582752	1.44245504	0.89884306	0.78163683	0.75105679	0.84287022	0.76538107	1.08992828
LIN9	1.33252643	1.26347445	1.82340203	1.98483247	2.47728771	2.12155253	2.54282021	1.99152995	2.32442464
LINGO1	0.73979241	0.85973592	2.06847141	0.93747051	0.81974955	0.66110339	1.752383	0.60255106	0.62457673
LIPA	4.42954535	5.39066248	6.5698004	5.09231707	6.97021428	5.58809421	6.39126937	4.97118309	4.56404762
LIPC	0.07533162	0.34884815	1.18997491	0.27560166	0.31749298	0.14954279	0.05774587	0.11954908	0.07207667
LIPF	0	0.03513201	9.48353513	7.42275014	4.41779666	0.38336846	1.42964162	2.6731549	0.54964899
LIPG	1.58395012	2.77586642	1.6181261	2.04941237	1.70688811	2.02480554	3.76927903	0.43189081	2.5628037
LIPH	1.75349064	2.39454737	4.61194482	4.74061292	5.54935878	4.40571891	5.03510021	6.31943295	2.7292177
LIPT1	1.33894915	1.84672756	1.92404449	2.05849586	2.22879678	2.02820729	1.40356811	1.84870874	2.10554466
LITAF	5.16639813	5.55706052	5.8291393	5.12825244	5.53475592	5.57886416	4.82516351	5.36746843	4.34404246
LIX1L	2.05386016	3.38794447	3.13837291	3.50678302	2.50550536	3.15219226	2.33345023	1.93329341	1.3785676
LKAAEAR1	0.0816215	0.11550194	0.03001972	0	0.07558302	0.14656709	0.12049425	0.12397884	0
LLGL1	2.1739556	2.37806877	2.1224376	2.83767503	2.59018652	2.85692261	2.70985072	1.76412954	2.81047026
LLGL2	5.12363695	2.77783212	4.83927083	3.88464078	3.83630303	3.08750658	3.59156492	5.42456066	4.96255299
LMAN1	3.36130417	4.89230114	4.6050908	4.93734084	5.1029467	4.9713922	6.36471198	5.13934318	4.33730322
LMAN2	7.11003174	5.29043063	6.27876244	5.25811463	5.24369556	5.52685733	6.05843531	6.36851045	6.01882229
LMAN2L	3.80342367	2.72081688	3.18767195	2.88539737	2.88070435	3.02569254	2.97868745	2.66711754	2.89829323
LMBR1	2.44852562	2.70683899	2.79599234	2.90642696	2.6544008	2.63546608	2.95259826	3.23496279	2.66497954
LMBRD1	4.45958615	4.99782653	4.750045	4.99056311	5.18435292	4.72979303	5.20985564	4.3250319	4.96347489
LMCD1	2.63156701	1.74431511	2.02140891	2.67912437	1.17731562	2.12657095	2.12585702	1.8320814	0.5787217
LMF1	1.42301638	1.42423983	1.1341139	1.16871421	0.70939598	1.06206848	0.97535218	1.25959719	1.00809221
LMF2	5.20906718	4.68816934	4.17245064	4.13098368	3.82241875	4.68128426	4.94661904	4.22005208	4.71510651
LMLN	1.36632563	0.81212058	1.03699724	1.14525057	0.91689502	0.99269202	0.85517554	1.40598437	0.95342252
LMNA	5.92728468	5.38362496	4.81973956	5.30348486	4.73053352	5.58504151	6.06453049	5.95665263	5.14856278

LMNB1	4.96726462	2.82899531	3.84828655	3.93079861	4.81327747	4.35564943	4.29534382	3.0629069	4.43267518
LMNB2	5.28073299	2.69824962	3.72681912	3.58513446	4.3903545	4.0337242	4.37571312	3.75721257	4.30907536
LMNTD2	1.76280733	0.36846213	0.3348618	0.6773606	0.50490124	0.56950724	0.6574088	1.01347516	0.98701806
LMO1	0	1.8996778	0.1051341	0.05084573	0	0.9757168	0	0.04097072	0.0112911
LMO4	5.97122074	3.80595625	4.18457369	3.37065331	4.99490463	3.67023484	3.86019488	4.33286939	2.10767108
LMO7	2.23735606	2.31594149	2.62798531	3.78717869	3.39033348	3.70912072	4.21007082	4.20304322	4.09851147
LMOD1	1.66126056	5.78505154	2.27977664	3.92725192	0.88479495	5.41034805	1.47818554	1.4751142	1.18947755
LMTK2	4.01028443	2.57620365	3.56580992	2.42754601	3.04660296	2.56674948	3.18893115	3.13428004	3.12760987
LNPEP	2.0947363	2.51441558	2.65965495	2.52791407	3.02176384	2.50891973	2.25190664	2.74632484	1.9440164
LNX2	3.65679836	2.99527053	3.10248748	2.79871489	3.10284743	2.97204092	3.49757796	3.29215881	4.08299204
LONP1	4.7319815	3.00464389	3.12019563	3.27711585	4.14898151	3.73917158	3.77528384	3.97157527	3.92180746
LONP2	2.90591486	3.52952114	2.98070318	3.28213305	3.62170287	3.37684586	3.30737786	3.28911902	3.42963913
LONRF1	1.26820274	1.6936549	1.29151627	2.38336691	1.8294544	2.0647818	2.19795107	1.59335006	1.93679515
LONRF2	0.01560963	0.65946954	0.09859989	0.53096802	0.00643126	0.52573958	0.00780779	0.94710907	0.01232155
LONRF3	0.2705824	0.70189024	1.0686551	0.99829839	0.34434829	1.92509226	1.53094926	2.00769164	1.38592708
LOR	0	0.03355096	0.0831816	0	0.01813889	0.01084588	0	0	0
LOX	2.92372082	5.32177165	3.04255837	5.24276404	3.31441327	4.44450527	4.7128284	2.99638639	2.81247028
LOXL1	2.19661059	4.10471616	3.49144946	3.9895977	1.6004942	3.80076906	3.06050135	3.46626944	1.75251973
LOXL3	1.03420729	1.46522298	1.70436116	1.92857099	1.5312934	1.76449472	1.79975037	0.98036718	1.13091926
LOXL4	0.29655266	1.12459748	0.51062356	0.74525448	0.43679312	1.01537073	0.91522717	2.98096694	0.21856182
LPAR1	0.79489637	4.47352778	2.9251461	3.23596779	1.16978058	3.42331927	2.07399927	1.59134007	2.26762378
LPAR2	3.26748723	1.50604228	3.36326606	2.80132836	2.69092097	2.6606044	2.5106902	4.53631096	2.88986288
LPAR4	0.11256553	0.87709907	0.24805744	0.35333403	0.06349223	0.40922944	0.16535354	0.07044755	0.03044555
LPAR5	2.33111783	2.43924429	2.08141941	2.9976835	1.56249817	2.47166905	2.78158309	1.79801937	3.10793613
LPAR6	1.99295381	3.61217248	4.64779482	3.3067813	3.8106311	2.90034408	1.92191557	2.8323187	1.8536629
LPCAT1	2.57148322	3.79181326	2.82065083	3.18857067	2.45955392	3.61805436	3.42028463	3.10984026	3.69253599
LPCAT2	1.29679178	3.02479129	2.59553566	3.39960545	2.41877168	3.08364544	3.00637618	2.92806497	2.54618518
LPCAT3	2.82133467	3.90170844	3.29205576	2.78701148	3.49687683	3.22384774	3.06531119	3.53627982	3.58048816
LPCAT4	4.47508882	2.52101959	3.11107213	3.29953047	4.39288256	2.33407114	3.38585951	3.33299413	3.45539301
LPGAT1	2.63478433	3.38940909	3.20345907	3.56594777	3.72015758	3.42627669	4.62599463	4.22420371	3.53678617
LPIN1	2.54833459	2.21081487	2.24787775	2.13505641	3.37736094	2.01888203	1.84372691	1.68800219	3.6635727
LPIN2	3.99472146	3.64180985	4.31645728	3.46622737	4.29154722	3.80959159	4.45339468	3.60995719	3.47038697
LPIN3	2.52027612	1.0238494	2.92079419	2.30573411	1.59854863	1.59892314	1.48647248	1.97784404	3.69284474
LPL	0.21588576	2.11717741	2.20897675	1.60600484	1.18584067	0.85022222	3.65398869	1.03942123	1.0620216
LPP	3.59822287	5.05123099	3.56937542	3.62881424	3.02010849	4.65110657	3.507086	3.44816546	2.98470522
LRAT	0	0.45947993	0.23114929	0.1112568	0	0.14156759	0	0.66640918	0.37295178
LRBA	2.47603402	2.70376863	2.61377552	3.02896388	3.22509615	3.13334474	3.20203991	3.62863035	3.71599229
LRCH2	0.08610635	1.70896784	0.97827546	1.12074411	0.53753803	1.36204543	0.42281424	0.30227597	0.1581302
LRCH3	2.12427358	1.88557478	2.30765213	1.89190622	1.82381373	2.06774745	1.56315046	2.40810314	1.71775931
LRCH4	2.33387597	1.32820176	1.92794255	1.42583494	1.48787485	1.51581525	1.8005149	1.65275559	1.64716342
LRFN2	0.02212005	0.03157194	0.04752081	0	0	0.00816688	0	0.04232891	0.00584649
LRFN3	3.83975811	2.63036864	1.97082483	2.87527892	3.17308729	2.91305177	2.30378231	2.97439664	2.78531903
LRFN4	5.47407042	0.86693235	3.13577036	2.50689911	2.72410369	3.15220405	3.98876151	2.98363989	3.66418457
LRG1	6.08430334	2.54944408	4.12713178	1.84709177	2.40913323	3.27946451	4.64373952	4.75913838	3.65196741
LRIF1	0.41669878	2.18667028	2.24483948	1.43036662	3.15856834	1.5035151	1.74825557	2.96449288	2.73356504
LRIG1	3.84146707	4.23623298	4.83133974	3.15051029	3.2939623	3.95794444	2.50244022	5.30755556	4.5961235
LRIG2	1.25164045	1.23543245	1.54640729	1.65312472	1.62127809	1.2568452	1.0262844	1.69366262	1.60020203
LRIG3	2.6637963	3.0726141	4.39574815	2.92978018	3.88786965	3.04740749	2.92647065	4.7721169	3.84530298
LRMP	0.87760607	1.69019347	2.03151939	1.50838532	2.09217131	1.39901332	0.74344178	0.70684595	0.89703917
LRP1	3.87053347	6.10930691	3.88614516	4.45046271	3.53185596	5.2518446	4.67947586	4.64326798	3.23092039
LRP10	6.00674545	5.23242455	5.75356338	5.02986352	5.16816579	5.5477272	5.60320365	6.78564782	5.76982698
LRP11	3.30558034	3.36821516	3.82600576	4.34436721	4.22404414	3.51923628	4.19433319	3.22390123	3.5744289
LRP3	3.4840063	3.1727066	2.14783469	2.39171838	2.09879382	3.43235268	2.60786463	2.5732077	3.33676824
LRP4	0.07837792	1.05113214	1.31138085	1.65085541	0.19283302	1.04128128	2.47214499	0.36855659	5.02795959
LRP5	5.32956231	3.66005823	4.98645577	3.43764274	3.34226694	3.60472155	4.66926235	5.35139122	4.90698758
LRP5L	1.1791751	0.51164858	1.0633853	1.08041898	0.95062813	0.73137033	0.57338876	0.44038308	1.61400757
LRP8	0.93073745	0.4758475	3.05710862	1.76089082	0.58703559	1.658605	1.35345128	1.62806202	2.39862109
LRPAP1	4.77349479	3.40894457	3.8670807	3.37976459	3.11968238	3.75205511	3.92399236	3.57537194	3.20582472
LRPPRC	3.22013926	3.80818674	4.1038083	4.59691901	4.95940495	4.40289121	4.94284898	4.54076726	5.3534332

LRR1	2.44185106	2.50593456	2.6445834	2.59995551	3.38591364	2.64538593	2.47947098	2.01795621	2.91578344
LRRC1	3.31995054	2.28854303	3.14123121	3.21396323	3.72073565	3.326471	3.45603867	3.95956903	3.35245822
LRRC14	2.32715902	1.50198329	2.23086339	2.17308542	1.96025357	2.31346449	2.14506105	2.5657307	2.41419713
LRRC15	1.99475216	2.32681849	1.52932814	3.89927116	0.76067093	2.95134971	2.84113405	2.28148029	0.56620052
LRRC17	0.20308168	2.79302493	1.17366507	2.01565891	0.40063902	2.00714521	0.99660766	0.67549492	0.32567981
LRRC18	0.02217388	0.71261238	0.01605388	0.05265186	0	0.10296804	0.02650689	0.04243121	0
LRRC2	0.02569997	1.1012062	0.79111816	0.26242217	0.08151731	0.71710209	0.1333416	1.05944385	0.3168822
LRRC20	3.26399703	1.819968	3.12151747	1.90729824	1.38636275	1.88503628	1.85587976	1.92346521	2.39308191
LRRC23	1.77551559	0.99979841	1.33222017	0.66952991	0.66075667	0.85957745	0.94555264	1.61929389	0.94893828
LRRC25	2.80174939	2.85665164	3.62389533	2.59140858	3.11146725	3.53174509	3.30710263	1.9005057	2.05902493
LRRC28	1.36682734	1.77671997	1.92556806	1.53503777	1.80138332	1.65747254	1.14034329	1.87058345	1.87814476
LRRC29	1.03769622	0.67158049	0.61036773	0.39304385	0.41429421	0.5747141	0.43313757	0.3804729	0.50211013
LRRC31	0.6339557	1.67953786	2.43373415	0.43353459	1.84581213	0.05071654	1.12137851	3.35645389	1.61765916
LRRC32	2.88124076	4.84998374	3.62642266	3.78948243	2.74326254	4.37402297	3.96593989	2.90191389	2.46323295
LRRC37A	0.08695405	0.0473447	0.12401896	0.14686154	0.17525976	0.12793922	0.32310839	0.22909564	0.40100896
LRRC37B	1.09096279	0.819543	1.30297608	1.06062854	1.25998619	1.03402041	0.7843069	0.94634012	0.83366523
LRRC41	3.51207421	3.41178815	3.64586938	3.17481489	3.19292447	3.43949428	3.30612385	3.49344826	3.71095162
LRRC42	3.78150187	2.93088505	3.70692754	3.67584418	3.3674559	3.6397571	3.46731908	3.42768655	3.54923946
LRRC45	3.62548735	1.67035216	2.30233132	1.56490715	2.18780391	2.1443088	2.48441982	3.22098691	2.71457694
LRRC49	0.03246204	0.74657146	0.65238055	0.65944734	0.15738639	0.66473381	0.19953389	0.17458008	0.42391678
LRRC56	2.0742702	0.19700838	0.5010433	0.46222652	0.33173834	0.40979551	1.93485396	1.88632721	1.51148295
LRRC57	1.84410733	1.20054182	2.16482844	2.11201431	1.80143773	1.60496758	1.56042073	1.1816394	1.89460973
LRRC59	5.89562073	4.98627721	5.66027403	5.27996543	5.432007	5.68840884	5.72521178	5.80215453	5.89448827
LRRC61	4.90122488	2.28002791	2.75105701	2.62142246	3.02016	3.06307326	2.42287376	2.58622228	3.37976545
LRRC66	0.14574424	1.04653395	0.53040516	0.86392048	2.76954156	0.10851101	0.48071048	0.80426024	1.92060228
LRRC71	0.04096914	0.07723982	0.23071853	0.02471902	0.06670211	0.04509414	0.01648934	0.05756392	0.03595421
LRRC73	0.56209494	0.21027742	0.26051815	0.75358911	0.32975954	0.67369302	0.42462041	1.80998736	0.27840905
LRRC75A	1.38596584	2.23527394	2.21112767	1.30118945	1.34328204	1.12640334	1.26035396	0.94082016	3.40335549
LRRC75B	0.85761216	1.35393519	2.04823451	1.64835526	0.22436858	0.95492587	0.60124288	2.0510159	1.91180545
LRRC8A	4.35342092	3.74271303	4.18450401	4.10012719	4.03131126	3.92964839	3.86513157	3.53815629	3.40386554
LRRC8C	0.88838843	2.04914225	2.19301616	2.04648452	2.19450154	2.424313	1.71046271	0.99811479	1.0227517
LRRC8D	3.03112976	2.97656704	3.14202341	3.18382168	4.07366189	3.44189496	3.17745536	2.68167551	3.5191852
LRRFIP1	3.9563549	3.51776956	3.48931604	4.1677752	4.24054366	4.36860611	4.0403183	4.64481047	3.30454409
LRRFIP2	2.12845895	2.05588324	1.72517057	2.21130085	2.31226049	2.33941534	2.26313765	2.03046994	2.02683997
LRRK1	0.7792406	1.94808692	1.85077455	1.63080392	2.24065426	1.91588197	1.39028203	1.18243835	1.52044631
LRRK2	0.47142385	1.56722828	1.02924602	1.20095474	1.58692742	1.95938123	0.61783439	0.57205311	0.33381686
LRRN1	0.01699378	1.00322539	3.16229918	1.53861387	0.0967264	0.49713588	0.04036102	1.35202645	0.11232176
LRRN4CL	0.47601566	3.01852736	1.7717129	2.23252798	0.14843213	1.86926411	0.81596732	0.85282048	0.32979814
LRRTM4	0	0.15011887	0.22903231	0.02801777	0.00827628	0.07475959	0.0822916	0.02318052	0.02805349
LRSAM1	2.67012895	2.28153566	2.61692916	2.25211452	2.16339689	2.24951211	2.21228475	1.82581105	2.99555593
LRTOMT	1.18821749	0.70173826	1.05126339	0.89409247	0.75179859	0.78418046	1.07557017	0.98963361	0.72669858
LRWD1	2.8544555	1.05606972	1.72002397	1.39879815	1.58927093	1.51717382	1.75191214	2.0305263	1.67824581
LSAMP	0.30709128	2.9877537	1.1750031	1.57351233	0.22494149	2.21521447	1.14636281	0.8052338	0.12600225
LSG1	3.97719867	2.82164183	4.51072255	3.60081472	3.62194488	3.33306938	3.30621523	4.00296987	3.49674591
LSM1	3.81090073	3.67500728	3.19089823	4.48411905	3.96730403	4.11869971	4.15702728	3.57987244	3.40189634
LSM10	4.48182393	3.99929631	4.66324507	3.84629237	4.07320184	4.2781444	3.63485192	3.28015035	3.91770319
LSM11	0.82785211	0.97115564	0.94340784	1.48192111	1.17922841	1.29977258	1.27190326	1.26594862	1.19072481
LSM14A	4.66946261	4.2062963	4.6120707	4.96539658	5.08214475	4.49007649	3.79688515	4.3577982	4.60746086
LSM14B	4.096047	2.79411127	3.75968343	3.22722755	2.71476733	2.90545569	3.28978838	3.133444	3.82701581
LSM2	5.81046241	4.90204809	5.87904404	5.10170816	5.34774344	5.35227305	5.44763057	5.47021174	5.36519212
LSM3	4.26773079	3.91919976	4.12412418	3.97625172	4.58954074	3.67969834	4.3275197	3.42546279	3.96357844
LSM5	3.36783992	2.90290025	2.81256228	3.21198968	3.63432157	2.92908433	3.22008939	2.64249235	3.26147723
LSM6	3.4818893	2.36126911	2.41405705	2.74075384	3.21597923	2.5941053	2.14753708	2.37360387	2.98217423
LSM7	5.38623914	3.96010649	4.16678299	3.92183912	4.65090843	3.70390525	4.12946381	3.72393717	5.18499632
LSM8	2.96901624	1.98011302	2.62172376	2.48176995	2.95021343	1.97872887	2.25678011	1.93378724	2.43423693
LSMEM1	0.02785055	0.23712387	0.68937012	0.55936682	0.35860674	0.36743154	0.30940367	0.18298126	0.31124182
LSMEM2	0.07138699	0.08149235	0.10204765	0.04325149	0.02974699	0.06141497	0.09875894	0.25762551	0.0749538
LSP1	4.13489522	4.39224503	3.94774247	3.37439068	3.72324732	4.04017229	3.61713859	2.87235579	2.82569719
LSR	6.99894432	4.60186439	6.37878032	6.12664443	6.95275421	6.29105781	6.74108916	6.98995676	6.60586726

LSS	3.12436404	2.0944451	2.44504062	2.73504924	1.61545472	2.70499695	3.25946404	2.36942436	3.5236855
LST1	3.66745071	3.15448609	4.18859809	3.0707122	3.88440016	3.4950047	2.68024457	2.30942868	2.40494567
LTA	1.12300912	0.47667032	0.84681912	0.42254329	1.07175902	0.54022666	0.38371789	0.35011097	0.6747057
LTA4H	3.89715357	3.49852753	3.82277013	4.48832882	4.94491954	4.2954809	3.72070122	4.14374832	4.30857373
LTB	2.98704057	2.38491563	2.37107735	1.66097862	1.77528555	2.31051696	1.52687359	2.08016034	1.28450288
LTB4R	0.80473024	0.85613647	1.10338647	2.02931102	2.2469571	1.52567431	0.75489536	1.02712899	0.99514819
LTB4R2	0.27616331	0.32266466	0.3330516	0.81511342	1.23426644	0.39594325	0.17755467	0.41280479	0.52553552
LTBP1	1.91372015	4.96435916	2.91394478	4.48908817	3.39378104	4.98328351	4.22344485	2.54258938	1.97639132
LTBP3	2.0895841	3.72694096	2.64353182	3.06030388	1.23756001	3.38030074	2.38674145	2.67903448	1.84376366
LTBP4	3.47907542	4.37160617	3.63081504	4.33247682	3.18552643	3.81323277	2.83020762	3.4841749	4.11529782
LTBR	4.93593671	3.7506809	5.38541299	4.0287845	4.19118608	4.25623472	4.03148139	4.45003842	4.39595877
LTC4S	0.05988884	0.06840484	0	0.03622815	0	0.06587573	0.05388742	0.05774897	0.0317895
LTF	0.20000626	2.22429882	6.93716629	1.46244989	2.04334109	4.50861893	0.39713736	2.44194835	0.42980139
LTV1	3.05611232	3.2820501	3.51906712	4.01545526	4.4903888	3.72755648	3.65831065	2.75175044	4.03998454
LUC7L	1.59092771	1.7390862	1.98586494	2.67225869	2.44357915	1.97750432	1.60038338	2.00512643	2.69368561
LUM	5.62983105	9.42160159	7.90224897	9.6243176	6.26291898	8.59448724	8.280408	6.97059002	5.92121593
LURAP1	0.23527589	0.82591908	0.40582854	0.43457494	0.26478132	0.84076507	0.44241598	0.0881668	0.01029587
LUZP1	2.33972697	3.34314939	2.35334548	2.64981145	2.94333855	3.11843959	2.73994887	1.77844578	2.99443824
LUZP2	0.0082118	0.07132301	0.42691059	0.29801712	0	0.0563744	0.8653504	0.20251699	0.01721187
LUZP6	0	0	0	0	0	0	0	0	0
LXN	2.27690337	5.38385246	4.88226905	4.68815332	3.60217475	4.69324126	3.75570713	4.42640228	3.77937678
LY6D	1.66149036	3.19125808	0.2197429	1.56160676	0.05669899	0.1079508	1.19852105	0.17042234	0.3585734
LY6E	6.50983598	7.65672254	8.50076739	5.9517155	6.22831945	7.43316407	7.45416861	6.7299985	5.75779577
LY6G5C	0.4502232	0.83953028	1.61405844	1.26958875	0.52544612	1.19696712	1.14131637	1.39955744	1.14788429
LY6H	0.14722794	0.6919027	0.14630184	0.39422597	0.05847878	0.52523447	0.16425677	0.07280782	0.14520678
LY86	3.05530048	4.36328743	4.13561168	2.90965488	4.19863445	3.09837511	3.13190361	2.36782898	1.9202493
LY9	0.30185076	0.71747811	0.9816933	0.37207051	1.53756787	0.58869773	0.43642993	0.30566462	0.34416334
LY96	3.779775	5.18208132	4.41855376	4.68195151	5.1921949	4.83256065	3.62178477	2.69726907	2.95190289
LYAR	3.11585423	3.11579974	3.27158278	4.29951142	4.83462827	4.08013103	3.72749873	3.41481135	4.31687129
LYN	3.65155547	3.98694864	4.5827397	4.27161388	5.19181994	4.78524249	4.86618848	3.84463205	4.17397331
LYNX1	0.5168815	1.8372906	0.45280602	0.78684344	0.09007325	1.53283589	0.88076613	0.28982303	0.09809788
LYPD5	0.70941465	0.93106076	1.10002067	0.89095201	1.04742199	1.27623953	0.53161405	0.68025874	0.62528157
LYPD6	3.42994211	0.18650073	3.00365858	1.70153067	1.88244594	0.55426499	1.9306401	0.32141013	0.78483115
LYPD6B	5.01753259	0.38622815	4.17357079	1.80564026	0.23060551	0.5583442	3.21859178	0.28012281	0.60515267
LYPLA2	5.7231037	4.56037109	4.21483576	4.52154038	4.56931526	5.20013205	5.10451859	4.75555114	5.32087535
LYPLAL1	1.85049263	2.73956196	2.94387865	2.68636964	3.00555277	2.79158685	2.50985145	2.78637472	2.73720244
LYRM1	2.81258233	3.93324981	3.72645113	3.16616509	3.3005852	2.40911846	3.17676995	2.76934339	3.55012647
LYRM2	1.34798696	1.91236907	1.89898216	2.55559293	2.02431761	2.21624195	2.22053663	1.51657865	2.05848329
LYRM4	1.66672077	1.53075772	2.03904723	1.61164045	1.53121252	1.81162811	1.51964612	1.75821652	1.6108984
LYRM9	0.50852386	0.74654646	0.69064816	0.50276696	0.54589639	0.41090404	0.17900787	0.39891202	0.52539319
LYSMD1	1.64863153	1.29508368	1.9701412	1.79560009	1.13998254	1.67061428	2.22587753	1.83434095	1.72843335
LYSMD2	3.75455396	3.07677657	3.59336089	3.0352655	3.92781909	3.72633152	2.81048155	2.4438682	2.85517261
LYSMD3	2.14834591	3.29431772	3.12693149	3.14093927	3.45243189	3.62093083	2.87944919	3.0343134	3.28041319
LYSMD4	0.88166528	0.64979082	0.99501502	0.78320527	1.17063281	0.8789639	0.61435123	1.33597412	1.3035741
LYVE1	0.45808313	4.63741872	2.12411756	3.45590611	0.87369622	4.15718808	1.19302878	0.66902592	0.62312366
LYZ	8.22095592	7.81412481	12.0401989	8.58588381	10.268864	9.37983691	8.43143748	13.0818697	7.34010064
LZIC	3.17019031	3.56868445	2.98741903	3.54203464	3.34755282	3.57001365	3.38091062	2.59096081	3.6657523
LZTFL1	1.07385237	1.86647927	2.10344092	2.10368505	1.25546875	1.93511642	2.15115588	2.4415199	2.23179476
LZTR1	2.99531342	2.44436114	2.30295067	2.47955721	2.30811815	2.27572532	1.67238906	2.29519597	2.57979766
LZTS1	1.32750477	1.57311856	2.04101114	1.5183376	1.25264057	1.8027276	1.43794863	0.90453355	0.57855008
LZTS2	4.39487685	3.06423421	2.32441242	2.99546918	1.90696985	3.22872597	2.52268334	2.75347028	1.98813144
LZTS3	3.19114966	1.96521398	4.36056788	1.61447001	0.51290244	1.70668014	1.27899109	2.6690191	3.41619308
M6PR	4.84916577	3.6909284	4.9781592	4.31990535	5.11222308	4.75381833	4.15634624	4.2747499	4.14303068
MAATS1	0.03044896	0.17413329	0.12078141	0.44859918	0.01883376	0.14679458	0.01222845	0.67571707	0.00538168
MACC1	2.16717039	2.46777269	2.9878586	2.30891849	2.92039064	2.3322852	3.58935273	3.53532268	3.61234982
MACF1	2.08323732	2.91636564	2.75944091	2.61735613	2.52312826	3.20056234	2.24369128	2.35793411	2.26508874
MACROD1	3.83448167	2.8283123	2.78783011	1.94532912	1.04635115	2.22480621	3.56692887	1.89241979	4.39648609
MACROD2	0.44676586	0.31307787	1.22660755	0.25869533	0.10310163	0.30239363	0.09489028	0.1380532	0.33460068
MAD1L1	2.5941588	1.67061024	1.94483103	1.26573092	1.30835526	1.6419181	1.70344133	1.7920414	1.23821934

MAD2L1	2.63615192	1.92956147	2.79639336	3.27541629	3.76643696	3.06825779	3.50823724	2.14402196	3.50129535
MAD2L2	3.77362297	2.59581566	2.62062163	2.77127149	2.91196653	3.07894396	2.48759524	1.77376296	2.80572632
MADCAM1	0.15620186	2.27903817	1.53952544	0.17822674	0.1752344	0.24130737	0.26945326	0.3342969	0.16377274
MADD	2.59778245	2.42508031	3.03665576	2.83482849	3.21954498	3.1381352	3.18664579	2.9725355	3.29213894
MAEA	2.97041294	2.26762049	2.38293243	2.96132891	2.5406531	2.78744774	2.91249896	3.04770244	3.53677724
MAF	2.23626729	4.10153378	3.22430127	3.15407626	2.74219249	3.27842383	2.65286897	1.76163669	2.00131632
MAF1	5.90929047	5.35843283	5.83550086	5.11198076	4.69040762	5.57867679	5.6358831	5.63607356	5.62282231
MAFB	3.62690651	4.84698459	4.31129069	4.41107963	4.97205795	5.05359503	4.01651506	2.87949286	3.33444564
MAFF	3.68977747	1.68998488	3.03037882	2.44793662	2.53751645	3.19237467	2.15638169	3.01309714	2.32434413
MAFG	3.72881593	2.32990706	3.28299346	2.18799066	2.05767378	2.98816223	2.69297377	3.27705042	2.98949483
MAFK	3.43339025	1.59867047	2.68724052	2.05127925	1.64750971	2.64790365	2.80677089	3.07217206	2.55700988
MAG	0.02444174	0.05540043	0.11956623	0.04369812	0.00757281	0.05770079	0.007359	0.01418095	0.00646396
MAGED1	4.31104625	5.14693733	5.16430578	4.51536949	2.65366445	4.58611528	3.40830864	4.88281327	2.15875703
MAGEE1	0.17915928	0.94171565	1.05026154	0.54936884	0.22304985	0.56877505	0.18582983	0.99839535	0.15022127
MAGEF1	5.27683367	3.77899573	5.02302035	4.57000135	3.95833106	4.03492118	4.43254312	4.696225	3.80342935
MAGI1	2.16646999	1.98958954	2.6027854	2.87578192	2.79206631	2.38794347	2.57973417	4.01502234	2.11726875
MAGI2	0.11119749	1.02233746	0.34840646	0.69636561	0.14591573	0.73166059	0.21459246	0.30731406	0.19174294
MAGI3	2.14562766	2.13746851	2.194948	1.9074329	2.07429723	1.72544292	2.00344898	2.58932203	2.32092825
MAGIX	1.53084936	1.01030523	1.38302309	0.21074638	0.14222594	0.35191412	0.77703077	1.55433293	0.14952272
MAGOH	5.02903488	3.68134912	4.27708178	4.48959659	4.94760988	3.69729778	3.60198055	3.84832889	4.54227379
MAGOHB	2.10546891	1.98511323	2.39972445	2.23697407	2.63481163	1.99063884	2.2015834	1.71091812	2.16028116
MAK16	1.68035025	2.12109173	1.97039221	3.48527362	2.48177454	3.19825766	3.28629703	2.4254387	2.7674431
MAL	2.96283866	2.14933293	0.64494339	3.32611381	0.45821122	1.11489364	0.37960243	0.34175706	0.532632
MAL2	6.58385949	4.90175378	7.63315455	7.20754826	6.68394158	6.40277915	8.35639484	7.29470346	5.91288883
MALL	1.68235008	1.59429978	1.1214144	1.75430814	1.60372376	1.17084567	3.8549663	0.34194681	2.88961831
MALSU1	3.21184175	2.89316303	2.26186022	2.76992429	3.01868254	2.78361174	2.84805657	2.5268855	2.90747735
MALT1	0.94435094	2.05867612	2.1257083	2.48322859	3.06464847	2.29052422	2.56521275	1.36650879	2.13220684
MAMDC2	0.03733823	2.66338138	0.48313986	1.38967492	0.06269869	2.23979174	0.10899053	0.13556875	0.04405613
MAMDC4	0.8361203	0.60773862	1.4967489	1.51855773	1.06927766	0.68336737	0.63762519	0.58692314	1.37312593
MAML1	2.89337034	2.6788687	2.8240081	2.60134282	2.72064198	2.93938904	2.59660629	2.97103047	3.03355978
MAML2	1.83415132	3.09822866	2.88103815	2.72631777	3.02634205	2.63991948	2.63987035	2.18802375	1.06148927
MAMLD1	0.40298132	1.53323739	0.84020912	1.55501593	0.57633503	1.3354759	1.34805338	0.50421039	0.22023412
MAMSTR	0.52102064	0.72945498	0.80683049	0.64346247	0.44071893	0.43990691	0.25533473	0.5202064	0.48403302
MAN1A1	4.30284582	5.27742886	3.9110082	4.48177816	5.54102771	5.3858015	4.60150988	2.37694807	4.52567602
MAN1A2	2.83242581	3.31246171	3.02505695	3.27093007	3.1913285	3.33165044	3.0637554	3.34729529	3.2173249
MAN1B1	3.6454106	2.33341417	3.45345114	2.75694805	2.2373966	2.8343298	3.22153662	2.82817229	2.63393351
MAN1C1	0.70957422	3.12709265	1.70155626	1.87174793	1.16217821	2.4658866	1.06972782	0.90093587	0.69016218
MAN2A2	2.85365542	1.97543209	2.95037316	2.1772945	2.51970936	2.41964812	2.37262732	2.74300589	2.70106932
MAN2B1	4.17276315	4.14934988	4.87247936	4.22690487	4.70123743	4.85345156	4.50341109	5.25546096	4.02304881
MAN2B2	3.65245676	3.16327354	3.46011856	3.32808827	3.13038269	3.34922065	3.56880578	2.95830959	3.02223419
MAN2C1	2.09058119	1.63000899	2.45277474	1.9078138	2.14552941	2.05686079	1.7609602	1.82127614	2.363109
MANBA	2.53447233	2.64261546	3.08520716	2.75683962	3.28465116	3.10777922	3.93182497	3.04732749	2.40506294
MANBAL	4.79667484	4.21207781	4.81498013	4.58076467	4.23962374	4.16718311	4.43254312	4.30232005	4.88097859
MANEA	1.82466357	2.51603518	3.00982401	3.45042614	3.44039852	3.07455862	2.87823557	3.75923194	3.058614
MANEAL	4.28405045	1.36323027	3.87150632	2.33703316	3.3799829	2.22026625	2.44873772	3.21283703	3.19073665
MANF	4.96033439	4.16325333	5.19385633	5.00037143	4.82977981	4.44722495	5.81435427	4.97540168	5.10093163
MANSC1	4.10527414	3.5795733	4.18496839	3.42676209	3.30253978	3.3751589	3.96127823	4.85934699	4.15269603
MAOA	3.69691122	4.66992773	3.01288906	2.3478248	3.20437716	2.14794379	2.34599065	2.75726972	5.37790572
MAOB	0.72714001	4.03305432	1.50760252	3.51770898	0.80590325	3.44090319	2.37711904	5.53735834	0.46114071
MAP1A	1.32722245	3.65084081	1.03137609	1.89885719	0.82386038	2.703665	1.60885757	0.97421238	0.79561775
MAP1B	0.91995848	4.15354247	1.88273793	2.46005434	1.45732417	3.05523215	1.66316289	1.36927817	0.86564371
MAP1LC3A	1.92436868	3.62058422	3.30705619	3.43716923	1.26562766	2.84484927	2.15381818	2.2915013	4.31472432
MAP1LC3B	3.5841047	5.1164288	4.83816475	5.24808128	4.85225081	5.05054638	5.08728878	4.22261171	4.43137558
MAP1S	4.35703303	3.23539015	2.97158761	3.1157276	3.63334713	3.90617645	3.56453674	3.66797033	3.74853518
MAP2	0.14961441	0.82058273	0.85471599	0.62680277	0.42290401	0.88780876	0.22659451	1.22606936	0.14043774
MAP2K1	3.78049351	3.46758307	3.88310638	3.94466329	4.01995397	3.9171024	3.87594148	3.19559313	3.88351455
MAP2K3	5.7722891	3.80864994	4.27728151	3.94923566	4.02416354	4.89520113	3.80521593	4.74465784	4.60577978
MAP2K4	1.72778752	2.83407137	3.19747242	3.2208386	3.58173062	3.17358043	3.07483743	2.62880146	3.03058538
MAP2K5	1.89588326	2.13454157	2.13601667	2.01421883	2.18915109	2.27753173	1.6411011	1.59660024	2.81911142

MAP3K10	2.72465634	1.18721522	1.21848887	1.09437113	1.5869245	1.70057343	1.15404981	1.98384372	1.33729187
MAP3K11	4.81718855	2.49971375	3.24975056	2.48642461	2.83504555	3.08236504	2.38797535	3.38233731	3.06623157
MAP3K12	0.68570438	1.31951441	1.14931337	1.62920336	0.98414142	1.43610892	0.75099867	0.714816	0.57991171
MAP3K13	2.66204982	2.14722975	3.19447296	2.47573419	2.80258117	2.73973594	3.17585921	2.61025995	2.25306877
MAP3K14	2.86240438	1.60755325	2.47981342	1.50396803	2.49173766	1.93119246	1.1998697	1.57767055	1.92686501
MAP3K2	2.12603338	3.02870338	3.64908843	3.34178507	2.88603137	3.48193999	3.05506421	2.87584212	2.65922543
MAP3K3	2.98180043	2.95294764	2.85120643	2.48635548	2.56515131	3.17535414	2.22942043	2.22245908	1.89194876
MAP3K4	2.63198194	1.96877967	2.27453265	3.17622131	2.28847862	2.31777889	2.29043267	2.45754516	2.38524742
MAP3K6	2.65565846	2.59211687	2.02002342	2.58322747	0.86424971	2.7524302	2.79886548	2.1650178	1.58790426
MAP3K7	2.21374501	2.71767646	2.44950258	3.46315766	2.89752299	3.16786251	2.98046118	2.24690144	2.88921313
MAP3K7CL	0.96299384	1.36008791	1.8679507	1.14178691	2.00634924	0.95385982	0.73621844	0.78503748	0.7272754
MAP3K8	2.44190949	2.82940553	3.1711713	3.56582709	4.46643209	3.75197007	2.59335521	1.64892505	2.85361628
MAP4	4.42362567	4.77347107	3.96659995	4.41327294	4.45346815	4.87454858	4.50964716	3.8441208	4.09861446
MAP4K1	1.89373913	1.62937817	2.05907804	1.40106472	3.17679464	1.57045822	1.08203318	0.86017933	1.28352664
MAP4K2	1.94739099	1.63482659	1.95514005	1.553779	0.99925449	1.52345687	2.19854785	1.8512636	1.45114117
MAP4K3	2.2203215	3.14319353	3.27524736	3.54896887	3.26506548	2.87573827	3.53269818	4.32257353	3.29832718
MAP4K4	3.30896659	3.61436821	3.09152119	4.01319822	3.4841539	3.64541402	3.31520251	4.16369571	2.650735
MAP4K5	1.9880819	2.94759811	2.87433199	3.35344219	3.28588921	2.91895618	2.55188154	2.71806244	2.65795997
MAP6D1	0.68568313	0.3107631	1.03808809	1.46258721	0.23203983	1.37402379	0.9528507	0.39548045	0.70853828
MAP7	3.31347024	2.64857398	2.88745082	3.22840958	3.28725833	2.96171054	4.41724538	2.7530409	4.30867382
MAP7D1	5.20071845	4.2069272	4.02009737	3.74306657	3.87477737	4.05646191	3.47190988	3.18992698	3.3264213
MAP7D2	0.37229713	0.16226071	0.28811392	0.09194341	0.11816156	0.04221672	0.90898869	0.10221906	0.09323107
MAP9	0.15955068	1.63575592	0.69436921	0.95945186	0.47333092	1.03934188	0.45648844	0.3968107	0.11630584
MAPK1	3.8143082	4.16776064	3.81105383	4.31339499	4.12349562	4.50348912	4.19525736	3.75702644	3.9394232
MAPK10	0.05464413	2.35825986	0.70348595	0.84411287	0.33886626	1.25700468	0.31250577	0.23457	0.07787512
MAPK11	1.32404671	1.28528338	1.32613878	1.50779161	0.99696926	1.90901025	0.82433409	1.45154889	0.6444568
MAPK13	3.92881395	3.04193502	3.43615529	3.26814222	2.59979934	2.80539589	4.29132887	4.24480445	4.7226775
MAPK14	2.13230936	3.66295874	3.54978539	3.74354384	3.29140706	4.27605273	3.73170697	3.75151879	3.456828
MAPK15	0.06983636	0.26167074	0.22307391	0.97605727	0.68346587	0.32083315	0.69908783	0.41977013	0.96784212
MAPK1IP1L	4.46555732	4.26514934	4.48864258	4.67802013	4.52513461	4.44169233	3.93582133	4.47233457	4.24550766
MAPK3	5.4496235	5.04457461	4.26683441	4.71728138	3.69329448	4.177816	4.89049395	4.42553319	5.0582565
MAPK4	0.00478681	0.67432736	0.11321621	0.20356353	0	0.52444682	0.00286732	0.00921051	0.48907366
MAPK6	3.00987263	3.35385416	4.19776143	4.09714122	4.04046494	3.78273414	4.15420327	3.29420562	3.00988138
MAPK7	2.10471821	2.168302	2.21814187	2.20235523	1.90322226	2.34126157	2.04969776	2.17361966	2.37957544
MAPK8	1.39029659	2.09320791	2.44735412	2.5303326	2.08312249	2.18805911	1.9804476	2.19486309	2.56197197
MAPK8IP3	1.71555929	1.64941756	1.59339973	2.18909808	1.84294448	1.37352284	1.38239862	1.85688235	2.55142066
MAPK9	2.21645643	2.73313584	3.0933286	2.76912853	2.77966568	2.74274278	3.08647914	3.56160913	3.11093112
MAPKAP1	3.47095331	4.66869615	4.27996302	3.29541517	3.51158046	4.1451574	3.43246724	2.96800928	3.73458191
MAPKAPK2	5.13767533	4.87714972	5.04926277	4.79702006	4.88000985	5.05999692	5.69230574	4.12982453	5.24441445
MAPKAPK3	4.20378312	3.17593923	4.15443062	3.13563616	3.89871088	4.2991387	3.96927737	3.9858808	3.62859961
MAPKAPK5	1.647037	1.45187988	2.05565067	2.05235795	1.94486483	1.62612695	1.6913163	1.99332241	2.33360199
MAPKBP1	1.70748975	1.7168032	1.82273142	1.65854405	1.37782052	1.91047803	1.18748292	1.21987652	1.53989556
MAPRE1	5.25018529	6.01186446	6.07927525	6.14636854	6.16326159	6.0632306	5.96009505	5.61467611	5.88855183
MAPRE2	2.78798924	4.25472272	4.90491089	4.27747152	4.36872536	3.62571893	4.10789653	3.25978518	3.06407851
MAPRE3	1.62700508	2.13370781	1.24278875	1.98939952	0.61012578	1.56372773	1.07752982	2.22618009	1.44756422
MAPT	0.03618283	0.42580347	0.25344185	0.45925808	0.02091492	0.56585168	0.00583702	0.23892108	0.03049078
MARCKS	5.56839772	6.25893153	5.47720402	5.76389441	5.50487818	5.85386059	5.53849592	4.72923147	4.75344701
MARCKSL1	8.44412659	6.20406596	7.48056644	7.15595962	7.27724986	6.54841782	6.65828478	6.38308239	7.80139692
MARCO	2.13824181	1.03139769	1.8718064	1.75554594	2.09715254	1.93248078	5.07414406	1.53207363	0.1487108
MARK1	0.21232795	1.63702952	0.71084959	1.17927213	0.3051826	1.15498912	0.26422589	0.21184557	0.53464073
MARK2	4.36119067	2.7938286	3.60157754	3.23085011	3.72041991	3.34818913	3.90014778	3.58368864	4.03025701
MARK3	2.76170705	2.90441073	2.61823494	3.28689916	3.44300255	3.06671185	2.74951149	3.32262838	2.94955816
MARS	4.05341228	3.01812076	3.78086897	3.68466377	4.31432549	3.86571083	3.96357728	3.96120302	3.83748951
MARVELD2	2.79524905	1.89448984	2.5247046	3.50389153	2.66398888	2.95490254	3.29625649	3.59954204	3.38924616
MARVELD3	1.74812795	1.67700989	2.02673105	1.65166223	1.92161512	1.73854808	2.51542227	1.73251852	2.62249434
MAST2	3.42143522	3.09932773	3.67234258	3.09365982	3.21074432	3.26384074	2.63106341	2.82201218	4.16997244
MASTL	2.01694882	2.18103302	2.31055986	3.0618578	3.5063287	2.92749765	2.76316774	1.80809635	2.24187706
MAT2A	3.66345276	3.92559853	5.23667054	5.34342951	5.80378904	5.10231201	4.3938279	5.16925277	5.47877259
MAT2B	3.33399946	3.6729348	4.47989358	4.29365034	4.35247455	4.294334	3.86777551	3.62577279	4.63734264

MATK	0.75124423	0.57792043	0.63721152	0.38674148	0.82101914	0.65219985	1.32242498	0.28529891	0.20653875
MATN2	0.12577318	2.78402631	1.80877266	1.91194946	0.28685503	2.51580919	3.05845468	3.40486517	0.67067304
MATN3	0.28540374	0.54999042	2.46587344	1.34255856	0.1372706	1.77029018	0.67494046	2.47645671	0.08354865
MAU2	2.74136004	2.32459269	2.91025304	2.42107798	2.78299988	2.57315368	2.127302	2.9475833	2.8620549
MAVS	3.81166001	3.27151683	3.58466403	3.28696755	3.03726869	2.91426812	3.44807835	2.52343372	4.1889665
MAX	3.87635086	3.75352112	3.80160963	3.76047447	4.26122548	4.03135292	3.44207235	3.44350432	4.10114591
MAZ	6.31711973	3.71915476	3.7819529	3.48308036	3.77164318	4.19752082	4.80615554	4.13313824	4.19818208
MBD1	2.55318298	2.67659226	3.27472871	3.28114779	3.24333966	3.15836967	3.33680171	2.94487338	3.30814468
MBD2	3.5639455	3.85013755	3.62892639	4.72530617	4.6724594	4.18212251	4.54435916	3.29912313	4.22273852
MBD3	3.46764829	2.00101014	1.84463229	1.88029578	2.40186088	2.19017756	1.81372318	2.01237946	2.16839579
MBD4	4.2287761	3.27104859	4.32004565	3.31612735	4.27593342	3.27565319	3.29278638	3.45795833	3.5748744
MBD6	4.50633855	2.8778932	4.10769037	3.31153099	3.38411564	3.4067527	3.07501768	4.23490878	4.11255535
MBIP	2.36105099	3.12484348	3.87190433	2.92830192	3.1643263	2.97690921	1.99367559	3.12813712	2.01576393
MBLAC1	1.10953572	0.71786271	0.71276886	0.66765195	0.29612004	0.42510872	0.41888719	0.67352561	0.65512612
MBNL1	3.96811207	4.71759851	4.40736799	4.45347582	5.1104333	4.78747398	4.30668995	4.02912831	4.07939687
MBNL2	3.16472737	5.08931624	4.35964904	5.40854055	4.92914235	4.60696656	4.65544818	4.76373011	4.7735418
MBNL3	2.41526926	2.27911863	3.83432848	2.05809723	3.89737098	2.60989895	2.14244067	3.49251378	2.44070481
MBOAT2	1.31792773	1.61390087	2.30430665	2.95503902	2.14723967	1.99861017	3.89555235	2.48837113	1.76925732
MBOAT7	4.48388272	4.52288436	3.85506376	4.29350388	3.20235847	4.14916947	3.75992499	3.57323157	4.19935405
MBP	1.26414354	2.2281395	1.84252233	1.92937911	2.48449386	2.25565713	3.08931992	1.62029192	2.39250674
MBTPS1	2.7962961	3.6499295	3.882069	3.88419321	3.80479526	3.70643119	4.06643226	3.92937112	3.70169851
MC1R	0.13133942	0.26324638	0.28263045	0.7745773	0.46784973	0.3449959	0.33435619	0.59027676	0.45895543
MCAT	4.07620987	2.69413447	2.77539283	2.67400854	3.13226818	2.81320871	3.00294005	2.59051068	3.36027591
MCC	0.3505469	2.58553604	1.88037974	1.86905003	0.63525375	1.85070413	1.04139544	0.52640773	0.43395292
MCCC1	3.03093113	2.43123297	3.73466697	2.68551858	2.79097111	2.3433662	3.5995274	3.43348932	3.46541124
MCEE	1.51935559	2.38743542	2.78659447	2.38098961	2.22938549	2.18562335	2.61383913	2.17416563	2.45004275
MCF2L	0.55294965	1.3002652	1.93514504	1.60072967	0.48058593	0.67069226	1.12289777	1.16996629	2.61372921
MCF2L2	0.04534977	0.07803272	0.43443699	0.07659779	0.1323685	0.09632174	0.04490335	0.09001189	0.08059972
MCFD2	3.77192391	4.54456684	4.28812609	4.15560395	4.14033642	4.30847141	4.41746722	4.53807339	4.09056093
MCL1	5.9159385	6.51116002	6.99734777	6.77201387	7.13444543	7.15939206	6.78798119	6.43128191	5.99469934
MCM10	1.3540446	1.01158794	0.95129947	2.01338949	2.25895018	1.98406526	1.75536911	0.88314371	1.82842744
MCM2	5.37271485	2.16925895	3.27368015	3.30760918	4.09207139	3.84837636	4.25493431	3.40887076	4.15489689
MCM3	5.67704454	3.80971283	4.54199675	4.49216014	5.85428203	4.74583873	4.99914668	5.14045233	4.92315041
MCM3AP	2.22857249	2.56312598	2.78215932	2.75000333	3.27138183	3.00624037	2.64437453	3.21510641	3.32231884
MCM4	4.17044586	3.11738197	3.26239898	4.0416873	4.76061864	4.32878499	5.15129834	3.81550606	4.46780565
MCM6	3.63558103	3.54534682	4.25013109	4.29283878	4.88937549	4.49764453	4.5276838	4.00292756	4.39397605
MCM7	5.59271062	3.94460783	4.77173752	4.15942429	5.25648642	4.80936791	5.02611389	3.79759697	5.27409617
MCM9	0.93640713	0.98354611	0.89095504	1.39909599	1.41417779	1.41188576	1.3662594	0.836241	1.2040372
MCMBP	3.42978824	3.65065309	3.95165833	4.26566874	4.47036392	4.14852414	3.97416646	4.05769606	4.23891767
MCOLN1	2.8170568	2.59898655	2.88278559	2.74843193	3.1479202	3.23439015	2.63549714	3.01449319	2.26291746
MCOLN3	0.01641463	0.13529283	0.56240278	0.72637396	0.19620888	0.81408153	0.09558262	0.11609165	0.95030547
MCPH1	1.36826745	1.57418622	1.54841309	1.86719075	1.89870304	2.22164997	1.92131209	1.62979445	1.93154474
MCRS1	4.67878177	3.1589997	3.93578959	3.54219308	3.48260391	4.00639673	3.73185511	4.2039498	4.18508571
MCTP1	0.38183551	1.58328181	1.34218744	1.33885648	1.4745997	2.0330284	0.8292754	1.54200927	0.56130668
MCTP2	0.99332677	1.32812662	1.90931201	1.46339378	1.54942406	1.64212496	1.46680735	1.76137228	2.04893881
MCTS1	3.97688154	3.64637544	4.06426832	3.67637635	4.95292427	3.39154625	3.43539565	3.4207403	3.91221463
MCU	4.49078392	3.62247035	5.22592193	4.29398823	4.94032278	4.11157717	4.23424001	5.05101153	4.13536717
MCUR1	2.19090535	2.67731543	4.10245597	3.07794173	2.43260136	2.78043902	3.37811566	3.60433045	3.82666201
MDC1	2.56670126	2.04781187	2.80451364	2.36715603	2.3243209	2.46105627	2.44970288	2.09980273	2.63649698
MDGA1	0.39133806	0.59159343	0.3839225	0.61743763	0.73340027	0.50524978	0.30516766	0.18990559	0.05951907
MDH1	4.18724982	4.40992341	4.37556114	4.6685559	4.8633858	4.64731198	4.38653135	4.77370828	4.74365626
MDH2	6.88574954	5.96468323	6.24196917	5.93984694	6.18463649	6.21440744	6.71590465	6.22077481	6.79676998
MDK	8.76274905	7.33161644	7.36957205	6.54575577	7.90772888	6.72230933	5.3927793	5.66168659	7.09906969
MDM1	1.16234302	0.98462576	1.68503954	1.32037433	1.53774698	1.15073726	0.75736379	1.40125471	1.25265593
MDM2	2.03776523	3.38628334	3.61540601	3.85993261	3.62323839	3.1386374	3.05400393	4.0635587	2.79544715
MDM4	1.57442214	1.5578406	2.74808464	2.49036332	2.6631157	1.94483966	1.80401742	2.40993384	2.32677963
MDN1	1.5687132	1.45749567	1.40344455	1.89452456	2.53843311	1.61246006	1.72341475	1.31136244	2.02887025
ME1	0.76292433	3.06968701	2.86827417	3.28261378	2.3038971	3.41063501	4.38383015	1.19054747	2.01890292
ME2	1.98540821	3.17251325	3.29805101	3.80533143	4.54598913	3.76381708	3.85897409	2.42564281	3.87451003

MEA1	6.54919961	5.24307138	5.86830673	5.25185009	5.11970337	5.48379717	5.38292776	5.98185524	5.20733985
MEAF6	3.35773242	3.65039955	4.31087116	4.23121295	4.07641021	4.06309943	3.65639892	3.28261489	4.27687092
MECR	2.25727773	2.11610917	2.43103735	2.29706502	2.06155611	2.16846232	2.02196903	1.49171246	2.54214507
MED1	3.26489982	3.05758048	2.89119494	3.26231943	3.76176392	3.2969576	3.27240421	3.41906972	3.20062054
MED10	3.49389504	3.02275661	3.54938437	3.58546269	3.87039467	3.34598917	2.83047324	3.09169488	3.44656988
MED11	2.97110395	3.36417641	3.64716139	3.34384327	3.42433984	3.23777269	2.74913767	2.85913444	3.63639396
MED12	2.90197437	3.38459095	2.83046673	3.29182338	4.39931534	3.48210951	3.3026688	3.66995963	3.25443503
MED13	3.02041572	3.30971432	3.69543508	3.48285592	3.58886002	3.53190418	3.22053929	4.92889254	3.49390839
MED15	4.0142209	3.17717415	3.05877123	3.23295755	3.56082656	3.57324682	3.13845333	2.92578009	3.75996871
MED16	5.08535384	3.38102148	3.64767019	3.30623376	3.64304403	3.96019049	3.74721538	4.17495451	4.13593093
MED17	1.4725384	1.6573805	2.13363535	2.13345724	2.35163215	1.85360197	1.75609644	1.94437094	2.19437839
MED19	3.62892908	2.94656941	3.02609619	3.03226219	2.66235157	2.91688115	2.9710421	2.80414424	3.38932313
MED20	2.71627955	2.72860842	3.68907448	3.03215975	2.71459143	2.90766114	2.91571607	3.32617175	2.98379672
MED21	2.77015094	3.54564443	3.79006177	3.48859225	4.22390165	3.40851721	3.29196892	3.63301388	3.44478066
MED22	2.53462745	1.73357902	2.66351197	2.06079729	1.92619963	2.07366473	1.88520119	2.31066824	2.31866287
MED23	1.9265902	2.24324428	2.32157914	2.70597732	2.94993696	2.39201748	2.31033713	2.21819498	2.93849692
MED24	4.37251498	3.11176436	3.71560596	3.43971504	3.519825	3.17831961	3.23920864	3.42085937	4.00573369
MED25	4.42615199	2.6897948	3.12049773	2.73792155	3.61136715	3.17964625	3.17990867	3.27571898	3.44284484
MED26	1.4712323	0.83338615	1.10237942	1.05498091	1.33911098	0.99213211	0.87731119	1.03459051	1.17191879
MED28	2.94413275	2.24989403	2.47642122	2.61874044	2.4886637	2.43859073	2.11419984	2.5579188	2.59531697
MED29	4.10966771	3.8451802	3.75964984	3.50415409	3.65369115	3.64538094	3.24213108	4.11009175	3.63167772
MED30	3.34943043	2.89746481	3.60175523	3.20311556	3.14326172	2.99883201	2.99699216	3.19762853	3.29505411
MED4	4.42372672	4.00033946	4.05207846	4.13028061	4.40567731	3.92395286	4.19647287	3.57847866	4.55232024
MED6	2.13978039	2.37794229	2.45344507	2.93194511	3.04922301	2.52846234	2.26819246	2.25842824	2.58597736
MED7	1.8555765	1.92660108	2.23734534	2.51050052	2.39594266	2.25862144	1.67854384	2.03975644	1.9782474
MED8	3.99697164	3.37807677	4.61441524	3.6020216	3.77872954	3.8432588	3.83611804	3.82748062	3.77393253
MED9	1.64473491	1.10806583	1.61351612	1.32538036	1.52790509	1.43083552	1.30882087	1.39404167	1.80528271
MEDAG	0.84645292	5.60225347	2.60990709	4.27220543	1.03019121	3.88460919	2.704034	1.15197098	0.81614016
MEF2A	2.20292775	4.09254655	3.88874374	3.69117862	3.40603581	3.95777286	3.06444669	3.40560825	2.98936638
MEF2C	0.85177765	3.35050069	2.67209371	2.45770259	2.01941418	2.67727717	1.67047691	1.33287433	0.9743234
MEF2D	3.74309328	3.79239644	3.82292044	3.56141086	3.36844188	3.9896454	3.81682334	4.21486953	3.21965798
MEGF8	3.28902539	2.47219636	1.52732981	1.82165742	2.15156239	2.48228366	2.29672991	2.00586282	1.94275537
MEGF9	2.35257107	3.32846364	3.35263885	3.33783596	2.72716016	3.44537446	2.52277808	1.91823791	2.8070057
MEIG1	0.19362608	0.18103228	0.24073977	0.1192557	0.10758672	0.07419657	0	0.02964349	0.09229159
MEIS2	3.08673269	1.7802579	1.72335525	1.53138367	1.79504148	1.22920705	1.27000909	0.87999448	0.36898425
MEIS3	1.0871212	1.4881061	1.25464202	2.2655005	0.51746977	1.7520866	1.28905985	0.64400768	0.41901861
MEN1	3.88954833	2.69672621	3.09410796	3.13913741	2.93381612	2.98565649	3.20584515	3.29997022	3.60941241
MEOX1	0.57071937	1.81242392	1.23581608	0.91426464	0.67096569	1.91211463	0.26435967	0.27763672	0.36121164
MEOX2	0.08491892	3.50447606	0.52407905	1.89842685	0.07011369	2.22284777	2.0535932	0.23297339	0.29676889
MEP1A	0	2.11307775	2.34449733	0.30921838	1.25015811	0.08718241	0.90910273	0.55949135	2.19894448
MEP1B	0.014041	1.40434687	1.07677181	0.03346917	0.66942608	0.04591893	0.04162402	0.08960643	0.42146624
MEPCE	4.90187407	3.56813898	3.44118448	3.50994837	3.10342913	3.73625732	3.82855744	4.05047128	3.77323819
MERTK	0.97634181	3.03201757	4.04959943	2.37182036	2.91129426	2.98114539	2.72630669	1.58052027	2.04724546
MESP1	0.11810404	0.30825905	0.57080132	0.18186534	0.16123591	0.65262625	0.16514777	1.11035908	2.0354917
MEST	5.22372893	3.89796315	3.62551783	4.11826142	3.92037319	3.01990045	4.60607213	3.75365321	3.93944004
MET	4.5113217	3.86223117	4.54646155	3.44940254	4.88783958	5.22436991	4.81484756	4.86793369	4.36191982
METAP1	3.61726881	3.74148281	3.7445399	4.49201187	4.69461103	4.22600891	4.08774624	4.0538466	5.08435924
METAP1D	0.55971973	0.6938378	1.92158735	1.41106729	1.22133317	1.21562104	1.14997399	1.00559002	1.53608145
METAP2	4.1240097	4.15375482	4.28198231	4.60583563	4.78817601	4.55017819	4.23333524	4.65342707	4.42935119
METRN	2.40516355	2.24576592	0.96281722	2.33676982	1.16573046	2.18739156	2.84739472	2.70009885	1.60371508
METRNL	4.01357497	3.53283429	4.23843072	3.60294438	3.57842814	4.34331167	4.3468543	4.08671813	3.77045171
METTL14	1.49874677	2.09086963	1.8779898	2.2620449	2.4834916	2.28067321	1.52263242	1.92955876	1.95295018
METTL15	0.9641637	1.87610938	1.81246058	1.74101544	2.24010509	2.07163336	2.08292633	2.0744423	1.8836164
METTL16	1.63925076	2.11003861	2.27637907	2.37248119	2.32678699	2.29403821	1.94879445	1.75102627	2.48237636
METTL21A	1.61816397	1.20464262	1.72515802	1.75176088	1.37097309	1.64551686	1.62182303	1.51264103	1.47175548
METTL22	1.3839645	1.21198407	1.25882627	1.19240451	1.02777002	1.14916022	0.95594235	1.06346493	1.27243344
METTL23	3.48232692	3.05625485	4.08910568	3.35960369	3.11829132	3.4016092	2.81315207	4.0010373	3.40053305
METTL3	2.17320401	1.86067746	2.06032172	2.10576758	2.58199007	2.13023726	1.93440008	1.91339791	3.04238461
METTL5	3.46505602	3.86751498	4.31749372	4.23961616	4.71260595	4.23505986	4.07920548	3.67491272	4.1323065

METTL6	1.15356103	1.27690439	1.36258691	1.50897756	1.32858687	1.23058076	1.35693568	1.47275589	1.05215176
METTL7A	4.71250848	4.4616109	4.78310983	3.59146442	3.46360189	3.42199028	2.91396382	5.13955863	1.52919268
METTL7B	4.86194364	3.70794977	4.55038122	3.48342271	2.76711469	3.3454858	1.84422088	4.66890766	4.17832463
METTL9	3.71557688	4.28275689	4.05687868	3.95468934	3.68721027	4.03233732	4.08041334	3.59952532	3.87750103
MEX3B	0.69544769	1.58307394	0.80619537	1.08526143	0.35003001	1.31422857	0.64599279	0.60814723	0.59567514
MEX3C	1.86686796	2.43821016	2.54359626	2.89891179	2.60652758	2.96971506	3.3870867	2.17772872	2.93961895
MFAP1	3.72476054	3.76531079	3.79278025	4.15927242	4.27666699	4.13300308	3.7476713	3.35497441	4.06672435
MFAP2	3.14309414	3.88943583	2.50669911	4.18827659	1.1347921	4.03240177	3.44803908	2.10546067	1.61304337
MFAP3	1.55320994	3.04228783	2.62396347	2.81575145	2.77175315	3.00593237	2.66217631	2.92932928	2.4967562
MFAP4	3.67645627	7.30645185	5.76787404	6.65900827	2.47776678	7.0051742	4.89671116	4.22105979	2.07120969
MFAP5	1.91203981	4.61539881	3.08680658	4.28504856	0.85681772	3.4923615	2.41850206	1.8599797	0.66522551
MFHAS1	4.47193616	3.42602344	2.93247422	3.79553007	3.60543447	3.67952132	3.94908156	3.29983518	3.85763227
MFN1	2.6011286	2.63636085	4.15094383	3.14674468	3.51592902	3.10180735	3.29254974	3.81058749	3.24749759
MFN2	4.17252646	4.57959652	3.69562444	4.53618088	4.69733688	4.97056221	4.49700164	4.29116174	4.71856677
MFNG	2.50681341	2.47552013	2.71272466	1.80779781	2.49439773	2.77913229	1.79930887	1.35541964	1.36501221
MFSD1	2.90997893	4.31549424	5.27087106	4.35922907	4.56925826	4.64524193	4.62881982	4.37931068	3.51297113
MFSD10	4.79603206	3.12705746	4.04999422	3.9238323	2.98487814	4.6007787	4.62587629	4.41644938	4.05482985
MFSD12	4.42423675	2.83914149	3.36602147	3.19618139	3.65318067	3.75061376	3.89845301	3.05582971	3.61475267
MFSD2A	0.83838688	1.24184978	1.7540293	1.22819537	0.62641999	1.23151383	1.71509342	0.64274138	2.39003388
MFSD3	3.15133306	2.54249136	2.365888	2.30471662	1.98941322	2.83906755	3.38630316	3.53359124	3.30437088
MFSD5	4.38259693	2.99026863	3.86907821	3.08424584	3.30032964	3.35035837	3.23650719	4.46193802	3.25246573
MFSD6	2.85703963	3.43084053	3.95431299	4.42177658	3.86444326	3.88258998	4.36298689	2.86265152	4.06655595
MFSD6L	0.01656133	0.6126484	1.1491432	0.72344713	0.04047208	1.16538934	0.15131333	0.66260311	1.19701673
MFSD8	1.00782071	1.59287965	2.5449982	2.01487003	2.07691528	1.6102072	1.21886608	1.73464127	2.14601232
MFSD9	2.12462672	1.81349092	2.8716417	2.38410371	1.99891824	2.41744558	3.12579175	2.40255616	2.68016869
MGA	0.99614915	1.90607995	1.86710657	1.77385164	1.21513171	2.04957295	1.57399987	0.50004219	1.95119241
MGAT1	5.52490701	4.79845887	4.89353208	4.5035123	4.80248569	5.23744228	4.98338445	4.88114082	4.33235367
MGAT2	2.18108631	1.13808553	0.94391312	0.66594552	0.81926372	1.3549563	1.09165352	1.75110015	1.27103255
MGAT3	2.38072184	2.17421341	1.02391791	1.34721094	2.42464523	0.70578189	0.49865098	1.60206256	2.65695843
MGAT4A	1.68555041	3.06370338	3.05084804	2.43768605	3.61784133	2.97339639	2.89036923	1.49045292	4.33633603
MGAT4B	5.48978791	4.14118593	5.45149597	4.50713283	4.47861324	5.14205203	5.73510286	5.91706492	5.535396
MGAT5	4.20959353	3.56536301	4.2522511	3.38846575	3.60677457	3.63783884	4.07088777	3.99637264	4.59782479
MGAT5B	0.02264931	0.08258378	0.23184126	0.0471562	0.01747259	0.05351411	0.01360069	0.18744156	0.02084736
MGLL	3.89832467	4.13078184	3.3860007	2.72127985	1.97455343	3.05293006	3.35641726	2.68007227	3.41055137
MGME1	2.89421355	3.55727415	3.02102421	4.33845036	4.18806876	3.65968746	3.88448843	2.68220389	4.14293088
MGMT	3.80773771	1.96816103	3.13817727	2.01971747	2.00520604	2.4053731	2.61919067	2.14544585	1.9923997
MGP	2.80972417	8.47354418	6.25227605	7.57953317	4.48583201	7.11512368	4.01657181	3.95722844	3.28763741
MGRN1	3.85179708	3.26228164	2.62910088	3.10001037	3.20817839	2.99115075	3.10733285	3.32067276	3.51969423
MGST2	4.86471215	3.9707681	4.16211138	3.49124327	4.08850314	3.13701218	3.7040775	3.8579767	4.63364512
MGST3	4.27079414	3.69737437	3.34499385	3.75877413	3.44495272	3.03215931	3.71481835	3.85018356	3.98320597
MIA	0.43157457	0.26271906	4.10259491	4.32214492	3.4720211	2.1429322	0.01663393	0.99497788	0.07164529
MIA3	2.32156986	3.28066967	3.29642227	3.62402021	3.37882565	3.20140049	3.60508251	4.81736834	2.84824649
MIB1	2.09702351	2.83933036	3.06693825	3.08338209	2.59407423	3.20240055	3.59340648	2.98447014	2.45800035
MIB2	2.73534435	1.38362553	1.47286546	1.88831645	0.94206606	2.27782938	2.34805086	1.29236608	2.90322607
MICA	2.41421093	2.12824724	4.29240233	3.0561542	2.71591152	1.33178036	3.36243753	3.22795057	2.18074743
MICAL1	2.65504509	1.9822134	2.76368076	2.79786572	3.39947315	2.6498963	2.43176407	2.1384088	3.4996271
MICAL2	2.39289431	3.36012136	3.45500933	3.99042779	2.77013502	3.59854801	3.8272503	2.52994475	3.3725137
MICALL1	3.8459996	2.99118507	2.82114618	2.51241625	2.88786118	3.17617686	2.35685345	2.20844636	2.5280879
MICALL2	2.15963676	1.69822615	2.22072387	1.99044528	1.76123013	2.11030564	2.34923145	2.55359086	2.57706302
MICB	2.69806811	1.64408293	2.22413448	2.1696638	3.53825858	2.9218462	1.39624809	1.44860547	1.47735989
MICU1	4.3287061	4.07255927	5.21002449	4.3739729	4.75532313	4.22134733	3.33954731	4.48211757	3.86746227
MICU2	3.43014541	3.11266471	3.56745413	3.45943068	3.41207598	3.18831644	3.28532747	2.94821984	4.00735268
MID1	0.1346677	3.60944853	2.45590568	1.99948485	3.04226923	2.42539213	2.62570062	1.63431289	3.46204516
MID1IP1	4.26576925	3.58397277	2.95174542	3.50095723	4.30218437	3.77770751	3.7230321	3.49352014	3.47167669
MID2	0.44244015	2.45435129	1.45645381	2.00347645	0.90638563	2.48707456	1.55983897	1.7529328	1.83521025
MIDN	5.58149897	4.0085239	4.56925811	3.80595748	4.60587367	4.93533698	4.87322054	5.41331743	4.50482326
MIEF1	3.62431505	2.75849242	2.8365571	2.77096614	3.05564636	3.06397362	2.86213391	2.64243591	3.38307098
MIEN1	5.67631498	3.97706651	4.76455553	3.86743766	4.20022126	4.2029989	3.92957612	4.3164897	4.48660125
MIER1	2.48585078	3.19413766	3.80282626	3.39164636	3.83977486	3.5335389	2.44985539	3.14718262	3.07411263

MIER2	2.94248087	2.36538361	2.75822912	2.57940438	2.87344401	2.76040107	2.64675073	2.51691269	2.74845453
MIER3	1.72111833	2.50784834	2.63594599	2.87227647	2.95638429	2.58902849	3.03333402	2.40912126	2.97439909
MIF	7.46491604	6.04133214	5.61678224	5.85224269	6.03051719	6.18705859	6.71272219	6.23794157	7.15410705
MIF4GD	4.24057402	3.16564402	3.81131222	3.32507719	3.10899005	3.96755936	2.97248392	3.81512965	3.09092309
MIIP	3.69141672	2.08116536	2.61444938	2.39517752	2.80322482	2.94156665	2.82240063	2.00718321	2.79205076
MINK1	3.6662228	3.63837848	4.08269566	4.18720569	4.2702093	4.06767087	3.37523624	3.59443621	4.8183589
MINPP1	2.87094281	2.75233314	2.55218604	3.18436354	3.5694849	3.20717153	2.97065329	3.01830059	3.26346221
MIOS	1.88957844	2.06980162	2.99747677	2.50117113	3.00459584	2.36885335	2.81477874	2.33470965	2.7840246
MIOX	0	0.07915227	0.09913733	0.04789406	0.06118854	0.9293367	0.13886247	0.01555792	0.16129949
MIPEP	2.98781209	2.64470302	2.61757522	2.99551896	3.41609509	3.45382828	3.4905079	2.85909982	3.95875517
MIS18BP1	1.78633567	2.55705087	2.79118211	2.71164953	3.55143021	2.5914365	2.10114815	2.04711057	2.95374985
MISP	6.26067464	5.52553491	5.34891462	5.71650292	5.91311761	5.09886168	5.57732741	5.92646065	6.68917592
MITD1	1.93894142	1.98226089	2.63168883	2.47850031	3.19972547	2.32363317	1.83358246	1.77588511	2.30416796
MKI67	4.29035659	3.00476897	3.40858957	4.1266685	5.47953773	4.24249499	4.28286146	3.52723276	4.5078807
MKKS	2.54941565	2.81544272	3.53856002	3.55252078	2.92098687	2.85481305	2.99780392	2.17152103	3.6975669
MKLN1	1.84922577	2.89410149	2.24228992	2.20534794	2.77739018	2.61916503	2.62118425	2.59179111	2.40590656
MKNK1	1.77784353	2.14558606	3.1267204	2.50018498	2.7186934	2.51113226	2.08465193	2.0260665	2.21708503
MKNK2	5.30695143	4.58948363	4.30585947	4.73148884	4.4485839	4.62734034	4.45228797	4.88785486	4.53686509
MKRN1	4.06602893	4.06306758	4.38039977	3.95635727	4.05144837	3.98656724	4.15874028	4.05964996	4.56912227
MKRN2	3.30822498	3.20803757	3.46153685	3.58685001	3.78059761	3.79354928	3.93083541	3.31173033	3.61749473
MKX	0.11100607	1.54627752	0.4716596	0.92368359	0.0536694	2.18268839	0.62077152	0.1369692	0.09041787
MLEC	5.66382261	5.36858204	5.5074077	5.49827656	5.30688887	5.30491198	6.16722494	6.16175441	7.52761019
MLF1	0.31930964	1.34289006	1.61391039	1.48788815	0.32024616	1.32376053	0.48801517	1.69586897	0.61193945
MLF2	6.37733783	5.24470621	5.22421798	5.61320653	5.26941361	5.7000086	5.39855733	5.14401636	5.70111692
MLH1	3.0854924	3.37880989	3.30637807	2.4393707	4.2578454	2.73448921	1.79686126	3.43292892	1.17835565
MLH3	1.27122587	2.00754589	1.92761786	1.79422682	2.34612655	1.22831164	1.19486422	2.04064271	2.08319899
MLKL	1.44466003	2.1245649	2.52241301	2.72470362	3.32908989	2.9595223	2.76430716	3.05677306	2.68081449
MLLT1	5.08066788	3.80751268	3.84211878	3.76022013	4.06321171	4.15246602	3.62766556	4.15815757	4.07684723
MLLT10	2.27902048	2.13115379	2.49296226	2.54070001	2.98165157	2.27036201	1.83934936	1.8338911	2.72950994
MLLT11	1.04268809	2.11933665	1.40742592	1.5961089	1.01411659	1.72666269	1.19388123	0.77971185	0.56780052
MLLT6	4.70053966	2.88769578	3.48868108	2.96746085	3.20147652	3.44635478	3.16971968	3.6553005	3.76525587
MLN	0	0.32843639	0.60019278	0	0.7310843	0	0	0.14171058	0.03345507
MLNR	0.02911897	0.79556504	0.0210961	0.01754053	0.01800795	0.44316398	0	0.0556	0.0305963
MLPH	0.08476526	1.36578649	4.05444034	2.77043724	3.54503256	0.4882005	3.85193666	5.13709463	1.47942399
MLST8	4.17684758	3.54731593	2.86767029	2.82287044	2.55969921	3.35294471	3.6731887	3.32483392	3.63675388
MLX	4.3886863	3.25264046	4.28597551	3.93395655	3.91375015	3.76608579	4.07069175	4.42697685	4.44491923
MLXIP	3.51794577	3.71359875	3.25784462	2.898793	3.1394589	3.51793242	2.74009603	2.97592803	3.76071609
MLXIPL	0.05931611	2.16928904	0.56693902	1.39532635	0.14620894	0.20691022	0.50673961	0.15737905	4.41965559
MLYCD	0.1619493	0.87888298	0.65207477	0.5712249	0.24474991	0.79982037	0.78908072	0.86771375	0.83299382
MMAA	1.14035293	1.68298361	1.40461898	1.58679379	1.86367277	1.79947339	0.86035017	1.39490023	1.79993412
MMAB	2.07482659	1.78273871	1.90378955	1.59645875	1.15360905	1.80175517	1.98617658	2.2095192	2.2540902
MMACHC	1.25295403	1.5024967	1.53546117	2.01202234	2.02111804	1.56956957	1.30979304	2.0534879	2.79748155
MMADHC	4.68739348	5.05607687	5.92678479	5.60918537	5.98912013	5.54079972	5.65088662	5.40109302	5.24028377
MMD	1.29578966	3.23175251	3.62726852	3.08814401	2.72757172	3.16322063	2.17247361	1.84875075	2.19199026
MME	0.40380534	1.12780983	1.78627355	2.52158629	0.71565497	1.15527804	2.0629543	1.1377308	1.39438001
MMGT1	3.60057502	3.7344232	5.14435813	4.45880287	4.6506406	4.31442046	4.13471658	4.30539237	4.07865632
MMP1	6.03428646	4.47188164	5.48607272	8.67506768	6.14393279	6.85098129	4.96973732	6.03151102	4.59815807
MMP10	0.38046481	0.70092523	2.37109998	0.6577447	2.39692692	1.70474623	1.3736857	2.68178177	1.50356445
MMP11	2.2840034	1.47838763	4.24124083	4.63156951	1.55693591	4.30547941	4.64942815	4.45127775	2.66824409
MMP12	3.1732337	1.07852818	7.5170809	7.09076263	8.35184094	5.77580038	8.31365763	5.37518296	7.44232292
MMP15	5.79862797	4.48365293	5.47913169	3.95011864	5.09201025	3.83792534	6.01004782	5.61804472	4.70429597
MMP17	0.5652199	0.94514235	0.36427225	0.62779733	0.07645961	0.88284214	0.42627923	0.18113405	0.16701625
MMP19	1.05380806	3.41371815	2.69787907	3.46892008	1.9798724	2.62418197	2.48024348	1.70352327	1.48201864
MMP2	5.45995975	7.68047827	6.48143822	8.03159145	3.62726264	7.49221681	6.40901287	5.21692572	3.94620924
MMP23B	0.43690949	1.08743696	0.27317384	0.5712769	0.04078085	0.51348378	0.30335973	0.17064443	0.08896072
MMP24	0.37584653	0.4511781	1.2739382	0.40728975	0.22990524	0.80445976	0.19394426	1.36823948	0.34567365
MMP28	0.8372705	1.96011874	1.05602959	2.53717157	2.54211603	2.65983783	2.02084914	1.45998109	0.33893962
MMP3	2.65908194	3.73037445	4.07191831	4.85996565	3.72286506	4.58689118	4.57651802	4.15772958	3.08221815
MMP7	6.23073339	4.65282671	7.64270187	3.4355018	3.30985682	7.38515614	2.29830141	3.35166605	0.57684576

MMP9	4.36119856	2.44395518	5.06243247	5.19605309	3.95262052	5.32158249	5.54341324	4.55322796	3.50692471
MMRN1	0.2429234	3.53462096	2.15640171	1.97532223	0.84289827	4.18832192	0.69131312	0.50465861	0.38787648
MMRN2	1.85936113	3.42512886	2.89186269	2.63088078	1.92411186	3.48392228	2.18117495	1.7351475	1.42242139
MMS19	3.29753146	2.84643157	2.61656816	3.46003659	3.26373124	3.21418722	2.93878697	3.18666548	3.82101677
MN1	0.54569362	3.07450142	0.88327949	2.01851732	0.32056442	2.4724288	0.62160939	0.51249185	0.49297879
MNAT1	2.130642	2.03162695	2.26056426	2.43707056	2.66152679	2.26205544	1.95445246	2.51304123	2.01084339
MND1	2.01986187	1.38750951	1.8693823	2.36936182	3.27364622	2.32186862	2.31262232	1.35824966	2.40130841
MNDA	2.56555822	4.56205549	4.39392451	4.02968025	4.89084639	5.80667536	3.55573391	3.08821431	2.64968102
MNS1	3.58332991	1.12739257	1.29731983	1.64362127	1.19314174	1.13815302	0.30512243	1.07066163	0.65811341
MNT	2.09052047	1.93358653	1.95348708	1.97940986	2.10865024	2.36474552	2.03790459	1.77099699	2.1851783
MNX1	3.3519291	0.78504837	1.3943746	0.83987619	1.13989746	1.00204235	1.20109269	1.10540231	2.22403085
MOAP1	2.81232963	3.53215054	3.76819459	3.2562103	2.94572304	3.34652776	3.04299804	3.41512182	3.0331932
MOB1B	0.86466176	1.90269175	2.21907064	2.796602	1.94368613	2.42132953	2.02334623	1.95469058	2.02299589
MOB2	2.83125662	2.16908165	1.85020049	1.95164415	1.68473915	2.32781369	2.4466613	2.10194145	2.24351563
MOB3A	3.97474089	4.0485453	3.94212911	3.860274	4.05048576	3.98354583	3.42375566	3.40858085	3.59069945
MOB3B	3.71127092	2.75348589	2.569664	2.25794522	3.71722003	3.12095438	2.94804264	2.00609761	3.0881306
MOB4	2.76010256	3.31167181	3.45502863	3.30602023	4.18928384	3.43126182	3.21670049	3.16924991	3.40855908
MOCOS	1.1099054	1.77355053	2.57952941	2.02135748	1.93902614	2.70047859	3.37637737	1.387796	2.67792426
MOCS1	0.56411347	2.23864934	1.50116277	1.49221352	0.78145394	1.99785191	0.78888698	0.68341408	1.87540461
MOCS2	2.51077686	3.26846258	3.23631488	3.2545638	3.48871481	3.07236048	2.6177439	2.84276317	3.00823215
MOCS3	0.84522171	1.52834273	2.48915441	1.85873952	1.11794359	1.39138445	1.22200693	1.80258102	2.12602617
MOGAT1	0.03436321	0.09631007	0.43113579	0.04113572	0	0.14571574	0.21253366	2.73304642	0.08861873
MOGAT3	0.42404326	1.82017977	0.99685076	1.40051315	0.44874388	0.14900303	0.18441413	0.20619276	2.97905663
MOGS	4.79623044	3.38291983	4.28490669	3.77711435	3.72021415	3.9858538	4.0899165	4.42500936	4.52892549
MOK	0.8170677	0.9500112	1.36544661	0.75207371	0.667263	0.27392134	1.08464035	1.06076981	0.46838053
MON1A	2.65173512	1.69660108	1.9692304	1.47288813	1.65202161	2.1171331	2.29981052	2.26381506	2.27272933
MON1B	2.86986593	2.90913146	3.40256957	3.03374788	3.36503659	3.51309617	3.61148447	3.65683452	3.60078867
MON2	1.41201388	1.80183951	2.47029628	1.91208887	2.46791825	1.66053259	1.60525406	2.55724257	1.76714403
MORC2	2.21195845	2.45080585	2.25777429	2.89805748	2.47786255	2.57489236	2.32028394	2.84205613	3.04926909
MORC3	1.53612668	2.86165344	2.86677068	3.22481171	3.80612034	3.16011474	2.69746554	3.22998417	3.13097967
MORF4L1	4.31443647	4.84486851	4.8014339	4.92688561	4.45142076	4.84969472	4.58369984	3.81116626	4.19440476
MORN3	0.30549893	0.22046969	0.63576998	0.15062898	0.18551085	0.16085218	0.02801483	0.67892656	0.08207729
MOSPD3	4.68154187	3.78237278	4.24366863	3.80873134	2.59888999	3.23592843	3.95090938	3.67818195	3.78353485
MOV10	3.75248744	3.34941448	3.31430081	3.07833647	4.8010762	3.94935475	2.89664492	3.24600517	3.94261119
MOXD1	0.87595035	3.34573116	2.29778083	3.01558428	0.8571742	3.10757586	1.75817225	1.44356111	0.41267006
MPC1	4.56858028	4.47427349	4.99146441	4.87003573	4.53955126	4.47788061	5.5763788	4.65980734	5.16957774
MPC2	6.13019141	4.50023628	5.25206572	4.12471209	4.20194253	3.81022541	4.77880394	4.64195261	4.77642545
MPDU1	4.16210276	3.8461818	4.66901226	4.02956189	4.09772261	4.46860327	4.45036147	3.61027312	4.81367349
MPDZ	0.3230603	2.39018856	1.01893779	1.9584792	0.85516834	2.02104617	0.70171994	1.0327552	0.32159959
MPHOSPH1(	2.55883829	2.69685749	3.08657181	3.2921654	3.42012988	2.84302721	3.15260488	2.91580273	3.3646212
MPHOSPH6	2.41607564	2.98990652	3.47036587	3.73536032	4.46954741	3.33621	3.67345584	3.02974958	4.09574777
MPHOSPH8	3.41785563	3.65968048	3.13242536	3.28502941	3.57921835	3.37785508	3.19446115	3.62622656	3.92666807
MPI	2.31281277	1.96947789	2.06083537	1.97739463	1.66018781	1.93482618	1.68222077	1.88344549	2.87228901
MPLKIP	2.26653488	1.77524883	2.09512885	2.13931714	1.95147725	1.78859907	1.77803505	1.64586519	2.24659576
MPP1	2.06469801	3.43455586	2.92255275	2.88992782	3.13914171	3.62575511	2.53757853	1.56008757	2.67951087
MPP2	1.70124823	1.08690166	0.26661731	0.79183209	1.02209772	0.7446428	0.14903251	0.29785566	0.20960426
MPP3	1.1655052	0.44451991	0.56514028	1.48998241	0.6807283	0.68678141	0.79413387	1.64367805	0.90018899
MPP5	1.93685042	2.75867931	2.60724643	2.80226446	2.53398458	2.69506468	2.57165281	2.81540226	2.67583202
MPP6	0.05720021	0.98827412	0.9051165	0.82770693	0.22784883	1.22308457	1.74833539	0.2511745	1.77668678
MPP7	2.1979711	2.77993214	2.93876287	2.21249626	3.87004481	2.31596886	1.05752354	0.86345453	2.77431394
MPPE1	1.69957898	1.35978261	1.96754224	1.22086242	2.06553421	1.28850232	1.85916858	2.06524698	1.30483494
MPRIP	2.47322344	3.14788162	2.87985441	3.37919872	2.87951313	3.01286326	2.97693123	2.33550475	2.82031196
MPST	4.9788613	3.26986269	3.18362893	2.07323783	2.20827722	2.7366375	2.8927352	2.64847144	4.02990293
MPV17	3.29113703	3.85568409	3.68469078	3.60504681	3.46359816	3.62191534	3.12802436	3.0634736	3.03227936
MPV17L	0.16054782	2.28739965	0.67107355	1.63273102	0.04415349	1.29575186	0.04292211	0.11454719	0.06239167
MPV17L2	3.96901984	2.20551423	2.74269041	2.6114387	3.06007586	3.03013948	2.74756652	2.55123157	2.99831245
MPZL1	4.51473224	5.10802677	5.06005145	5.31134764	4.11084751	5.13341608	5.75477735	5.99674995	4.62357975
MPZL2	4.5380557	3.29072677	4.16149948	4.04831783	4.55400806	3.47197483	4.0360612	3.89375372	3.416867
MPZL3	2.37015503	1.1229155	1.87486938	2.75617766	2.25249931	1.39399021	2.57114064	1.80547219	2.42502768

MR1	1.80276382	2.70278376	2.57222857	2.11333645	2.85723164	2.33119236	1.58390453	2.57325538	0.82134339
MRAS	1.40006492	2.79357342	1.82510718	2.26954762	1.81188088	2.59517223	2.31872991	2.6519175	0.79611553
MREG	1.96401447	0.95067558	2.71106552	2.58354599	2.50844959	1.64714189	2.0242869	2.36583926	1.881761
MRFAP1	6.32314963	5.95956935	5.83869566	5.95114761	5.82562473	6.06171474	5.69310766	5.99665632	5.9081058
MRFAP1L1	4.44704695	4.36462325	4.63671299	4.41799136	4.53213218	4.2486838	4.12602972	4.47111181	4.3270472
MRGBP	3.22183648	2.48799259	3.51698419	3.34188998	2.39004144	2.68635958	3.34283239	2.85156568	3.59651199
MRGPRF	1.35834481	4.95561747	1.87909565	3.10156207	0.67361344	4.53713073	2.32134355	1.48238449	1.22617266
MRM1	3.02052657	1.35525064	1.49476594	1.68336759	2.12450068	2.17273198	1.39877262	2.34903482	2.50680115
MROH7	0.0159524	0.09338748	0.33234642	0.06123674	0.06284353	0.04067768	0.00479312	0.06940219	0.08988929
MRPL1	2.85093338	3.01476181	2.51719739	3.73261839	3.75688807	3.35731147	3.13849721	2.83662784	3.62019149
MRPL10	4.70228264	3.92478325	4.11348067	4.03441066	3.85365245	4.17652605	3.84344985	4.22040777	4.3813445
MRPL11	4.96653705	3.41103219	4.10571164	3.35703976	3.79278145	3.69776875	4.0061376	3.96478132	4.30941866
MRPL12	4.67381153	3.14178045	3.48877705	3.01112855	3.28764311	3.83790287	3.2726433	4.2675679	4.46805738
MRPL13	3.05384692	3.30651082	3.73304792	4.11158027	3.93595024	3.68996226	4.09432689	3.27523631	3.14758829
MRPL14	5.97324503	4.90326436	5.86970699	5.00530234	5.45153604	5.26331987	5.51123294	6.04721345	5.56677231
MRPL15	5.07223368	5.11948376	4.97280326	5.58518828	5.54637235	5.62221718	5.92000033	5.60998621	5.57433032
MRPL16	4.16967484	4.04624673	4.19078574	4.44549467	4.67619218	4.12113162	4.52232162	3.95215463	4.92065246
MRPL17	4.14692871	4.24102775	3.95995753	4.33946156	4.4651304	4.59232331	4.48545421	4.72303341	4.34501779
MRPL18	5.66799643	5.08038024	6.17784802	5.57097664	5.81882793	5.46273881	5.55063413	5.53020062	5.31021817
MRPL19	2.8389183	3.31659345	3.32140901	3.77247839	4.24928762	3.58647635	4.00534333	3.50650849	3.58929307
MRPL2	3.81083537	3.06993711	3.91057917	3.48868435	3.49162282	3.32932792	3.76958562	3.68576945	3.85467117
MRPL20	4.72382166	4.05051752	3.55524046	4.2961568	4.43090561	4.08180105	3.83678654	3.59715404	4.43192584
MRPL21	3.82404709	3.13597651	3.37220879	3.87741588	3.93916687	3.37028573	3.72646359	3.39495856	4.02176813
MRPL22	2.54581819	2.44859037	2.791156	2.8505139	3.39627632	2.49974237	2.32918412	2.4956664	2.71955604
MRPL23	4.31297286	2.95709462	2.45695785	2.5471532	3.14681935	2.4583267	4.10044427	3.95555035	3.72228738
MRPL24	4.79839317	4.3417091	4.70768866	4.08411982	4.11320049	4.3172914	4.44065652	5.22676915	4.343049
MRPL27	4.16990195	3.47444376	3.3497412	3.47300014	3.64691129	3.4423414	3.2989941	3.52258925	3.70890972
MRPL28	5.21522127	3.9697969	3.33230362	3.66830987	3.82249526	3.97823427	4.21382558	4.24504402	3.71171438
MRPL30	3.27661573	2.97278065	3.88366013	4.03393347	3.88552252	3.28635928	3.12476919	3.70052826	3.89251179
MRPL32	3.43584466	3.60231101	3.82097712	3.6554279	4.08843695	3.50069524	3.62066987	3.34842483	3.62083614
MRPL33	4.72477473	4.09721155	4.50018933	3.92606284	4.34797842	3.68380222	3.93512527	4.14788222	3.99569542
MRPL34	5.54346227	4.47350366	4.43870684	4.65221039	5.28817617	4.72040737	4.18749641	4.72926806	5.1070192
MRPL35	2.93799353	3.39549396	3.43864765	3.76289874	4.06357405	3.70329648	3.94069805	3.5962462	3.8840119
MRPL36	4.0829701	3.74796577	3.60498637	3.02941961	3.42722365	3.35545637	3.27635933	3.60835112	3.96810606
MRPL37	5.61867999	4.57515096	5.4963637	4.94571654	5.44775313	5.17707319	5.31592189	5.12845392	5.54699502
MRPL38	2.39372209	1.31571514	2.00696299	1.70081139	1.47073535	1.54631788	1.44220361	2.02126449	1.83011286
MRPL39	3.3515082	4.44663639	3.92182111	5.02349103	6.01633839	4.6548555	4.50094423	4.19924465	4.94183034
MRPL4	4.59847768	3.09795254	3.56866253	3.65744485	4.38927122	4.05637101	3.72870594	3.90774533	4.48304502
MRPL40	5.37310765	4.29718389	4.46549933	3.98687597	4.3910485	4.193579	3.83307117	3.66714994	3.66490689
MRPL41	5.69386751	3.91017495	4.81107345	3.89092094	4.04983246	4.18141971	4.17974032	4.57182772	4.54784272
MRPL42	1.71128604	2.32406148	2.45476085	2.98620123	3.05646389	2.64485851	2.39958248	2.46912179	3.06234169
MRPL43	4.46718832	3.61665263	3.4386899	3.75675871	3.60858127	3.78677093	3.41729614	4.28881953	3.91858747
MRPL44	3.97113104	3.78115735	4.06294263	4.28174856	5.25300996	4.29204623	4.18159013	4.04888617	4.33220066
MRPL45	4.81927401	4.30543545	4.64007495	4.85092127	4.58777485	4.56619921	4.46594762	5.00395553	5.3203206
MRPL46	1.70064448	1.22422865	1.43125388	1.2514521	1.56550286	1.43291606	1.15373133	1.93817503	1.44014847
MRPL48	3.1560288	2.33382661	3.18679319	3.11853885	2.67333935	2.92166563	3.33559114	2.26382918	2.91037781
MRPL49	4.47153862	3.95489171	5.0430052	4.36796929	4.06253998	4.35823553	4.47025259	4.05834953	4.5372917
MRPL50	3.16969009	3.26412185	4.46590119	3.80546648	4.81434379	3.23843277	3.29209581	2.5384744	4.23919372
MRPL51	5.61467752	5.00091188	5.32897729	5.48374881	5.40425604	5.22137695	5.12675711	5.16627596	5.28679373
MRPL52	4.31875935	3.25311447	3.70699105	3.00002115	3.23171906	3.30242761	3.2109834	3.68080251	3.54431304
MRPL53	1.11459846	1.08385586	1.69019915	1.78063428	1.26367839	1.21353729	0.85606722	1.65274782	1.57335858
MRPL54	5.14892837	4.19471202	3.99448537	4.12560353	4.65383772	4.60571727	3.9864507	4.44835613	4.63015002
MRPL55	4.17079117	3.00638162	3.40592262	3.04693675	2.78828245	3.24406858	3.99289232	4.01085133	3.21735823
MRPL57	5.18033432	4.37880925	4.42799992	4.74147825	4.65867353	4.51509274	4.09291813	4.50136909	5.26013367
MRPL9	4.99773467	4.3257906	4.55959216	4.83719155	4.67356173	4.65054096	5.07848045	4.23854822	4.73835082
MRPS10	3.92479074	4.54234839	5.27922295	5.02478374	4.99796951	4.67924245	5.17489289	4.80871973	4.55986098
MRPS11	2.65108251	2.33452583	2.59813205	2.13406846	2.46339918	2.44020251	2.04774229	3.05358229	2.46482278
MRPS12	4.55197632	3.32433315	3.82896493	3.15201994	3.90146166	4.01596611	3.55618519	4.02110511	4.2354812
MRPS14	3.48995201	3.58163425	3.51347691	3.13941455	3.38528981	3.17805256	3.7434711	3.57333611	3.57142922

MRPS15	4.78999794	4.36080386	5.39322344	4.76253126	4.77027012	4.58155111	4.24989035	3.73596115	4.82013584
MRPS16	4.7941307	4.21264603	5.05012352	4.18163432	4.31540887	4.44179824	4.40773095	4.42327135	4.9333757
MRPS17	2.32239701	2.04844362	2.87577269	2.75623319	2.5326129	2.33498051	2.46221714	2.06170884	2.46157317
MRPS18A	5.2704344	3.99078477	4.57799479	4.05144575	4.22835297	4.16777	4.60329461	5.00096484	4.87763564
MRPS18B	5.16680344	5.06525385	5.94282508	4.76562757	4.63687119	5.02989329	5.5679929	5.54043805	5.31959119
MRPS2	4.56735649	3.93153918	4.49104885	4.27367259	4.59870982	4.47690789	4.484245	4.51770602	5.06450969
MRPS21	4.68929355	4.26500477	4.34865211	3.68674843	4.58023354	3.70696107	3.30498703	4.05554537	3.95121296
MRPS22	3.43052075	2.93270244	2.74350772	3.13387515	3.51983096	3.13103693	3.30513549	2.82306411	3.39789644
MRPS23	3.0187211	2.21575925	3.31460442	2.65332287	3.04874835	2.66282959	2.31138098	3.40169529	2.77507599
MRPS24	3.5124952	1.54126231	2.07002022	1.62399355	1.51810333	1.69186516	2.2963433	2.42412146	2.34788494
MRPS26	5.40219209	4.17192009	4.74138398	4.69220031	4.41456106	4.79697385	4.79537376	3.53608882	5.48334479
MRPS27	2.93474071	3.01444266	4.01975038	3.50835158	3.64842865	3.70835543	3.49145321	3.71517421	3.87440196
MRPS28	2.70475797	2.75653099	2.68120656	3.43863699	2.8937884	3.08641609	2.86131489	2.37582801	2.7394717
MRPS30	2.65982039	2.99566004	2.80374645	3.12670114	3.20363142	3.20229225	3.00353456	3.26653585	3.64748039
MRPS31	3.96285283	3.0067253	3.02966689	2.84242643	3.93910662	3.08577461	2.87366251	2.76331902	3.95397014
MRPS33	2.85714884	2.76309401	2.98729031	2.59877774	2.72472875	2.4868654	2.71449449	2.2819838	3.13668337
MRPS34	6.88129949	5.73570679	4.49578305	4.88543888	5.18621837	5.60268233	6.12400334	5.66625678	6.11966679
MRPS35	4.68727864	4.77970188	5.37644945	5.51418242	6.33938864	5.21078335	5.36216356	5.63593215	6.0104904
MRPS36	4.50327565	3.72557482	3.57465833	4.16582102	3.98458671	3.81052755	3.75622923	4.11618409	4.32801694
MRPS5	2.90700564	2.45516251	2.90323644	3.01486146	3.24832885	2.710698	3.20816072	3.15598018	3.50080664
MRPS6	1.57259792	3.16246116	2.31798624	3.09260723	3.20322087	3.21847143	2.90560903	2.38010081	3.17927634
MRPS7	4.31474679	3.85181809	4.67329486	3.81897731	3.76788173	4.06429981	3.86317657	4.68162503	4.20109087
MRPS9	3.27893485	3.34024064	3.70663055	3.9643826	4.20917499	3.27149825	3.77001621	3.60948579	3.91748637
MRRF	1.68060547	1.87974279	2.90393896	2.52997142	2.68341911	2.35734913	2.14981541	1.69514931	2.88926741
MRS2	2.20703519	2.47244982	4.0358564	3.474917	2.89639632	2.90178851	2.98660113	3.31760145	3.38470331
MRTO4	4.10905156	2.91017433	2.8535929	3.61804131	3.51313138	3.74307252	3.32381298	2.65285474	4.0505021
MRVI1	1.47242647	4.60557614	2.88281371	3.23578675	1.67941348	3.90964565	2.17796577	2.15690537	1.154522
MS4A2	0.16238061	2.94005601	1.09995673	0.75198578	0.21507033	0.83151811	0.1265613	0.12963699	0.03820761
MS4A6A	3.18735623	4.72501118	4.96193153	3.92083583	5.12295815	4.56926743	3.36877357	2.70963092	2.82953107
MS4A7	2.75155677	4.18577148	4.61801848	4.37698351	5.28090291	4.69947063	4.09555496	2.59094546	2.38166354
MS4A8	1.00528451	2.39591261	1.35699688	0.95953098	0.76459103	0.0609061	0.11364858	3.25478118	1.79605359
MSANTD3	3.0050282	2.90151062	3.15280581	3.32413033	2.60080354	3.38070653	2.38558426	2.31028083	2.38424588
MSANTD4	0.71224109	2.25050272	1.89554084	2.29134261	1.76386973	2.34584986	1.33657547	1.54907496	1.81041777
MSC	2.73441608	3.15755657	2.84831536	3.08181345	1.73117693	2.77632472	2.13259455	1.84567667	1.20608808
MSH2	2.40019144	2.3325858	2.97306169	2.93213248	3.65417035	2.87003767	3.33756368	2.82132646	3.17855978
MSH3	1.67783314	2.87381325	2.66022735	1.80368938	2.70830739	2.48605257	2.87874108	3.21392353	2.58688186
MSH5	0.47826588	0.1733345	1.01140697	0.8143659	0.57945003	0.3013144	0.31050803	0.73174696	0.70341414
MSH6	2.11276045	2.29514745	2.34032953	2.65085048	3.30581137	2.7478095	2.71553575	2.43416838	2.77607948
MSI1	0.04642297	1.8286854	0.3929074	0.78325507	0.02877478	0.18672192	0.08898185	2.69447085	3.71774957
MSI2	3.06149136	1.55458515	2.70290878	2.56772162	1.47330014	1.83796965	2.53133243	2.35704266	2.21066426
MSL2	3.58462034	2.60291081	3.09609319	2.8895517	3.16002641	2.91561404	2.85513427	2.77558888	3.03440295
MSL3	1.71318719	2.75233314	2.72784599	2.9100399	3.57612072	2.67636531	2.71959475	2.38424111	2.43920845
MSLN	0.84286786	4.88700162	2.00922343	5.19245027	0.12993667	1.64081835	4.28361508	7.95654474	0.66266811
MSMB	0.07223612	0.08245829	8.06333576	2.88295086	6.95286184	0.66432306	2.15132777	11.7196091	0.87707939
MSMO1	4.90227779	3.26991716	4.69394513	5.26214009	4.09505254	4.13533796	4.78764363	4.1848182	4.42663076
MSN	5.00393705	6.4789823	6.0817342	6.12445998	7.74312582	6.52410016	5.89608772	5.91838014	4.40471736
MSR1	2.39295628	3.68107901	3.81563269	3.05724343	4.13425046	3.98969989	3.79536461	2.32752372	1.81518165
MSRA	2.1937721	2.1579364	1.65876069	2.36187589	1.03348304	2.50104065	3.04475725	2.37306803	1.37762183
MSRB1	4.40615236	4.63623441	3.22774862	3.5046034	3.57220358	4.59398337	3.82291806	3.97242507	4.99562635
MSRB2	3.80610481	4.60614065	3.18907955	4.0323407	3.39164893	3.78052265	3.18100549	2.5886305	3.77023077
MSRB3	1.23674128	4.60141468	2.34070534	3.61699831	1.24193932	4.37500618	2.31677535	1.5804597	1.08803343
MSS51	0.2225732	0.44814535	1.28608834	0.95940522	0.62247192	0.57562858	0.20917096	0.3530562	0.57311937
MST1R	3.69410485	2.31966663	3.07068559	3.63312149	4.195304	3.62967355	3.5750838	5.21355706	3.99477012
MSX1	1.02622209	1.76081568	1.55855514	1.82289357	0.40380374	2.02004552	1.48781136	0.3301994	1.64307445
MT1E	3.30521564	6.62717724	4.17698397	6.61365981	3.33807271	5.69568606	5.55540716	1.79154643	1.73225839
MT1F	2.40401871	4.79794347	2.88484684	3.479933	1.24340857	2.09339887	1.50649791	1.30088849	1.8025657
MT1G	2.21340579	7.35999747	4.01002149	6.71299393	3.99644049	4.8321962	4.47319377	1.39106834	3.27430431
MT1H	1.19710124	4.17565769	1.50727907	2.59239936	0.87928308	1.54242268	1.34921674	0.48954164	1.13341839
MT1M	0.64446493	3.97976062	1.06002224	2.51045857	0.30016715	1.7673094	0.79778714	0.30769025	0.17231423

MT1X	3.65510914	3.97029414	3.5965241	5.86072342	2.52979622	4.37298029	4.02385923	1.45748201	2.68139342
MTA1	2.71991307	2.65796969	2.33491651	3.15168128	2.8687772	2.72948653	2.69095326	3.87721934	3.34146698
MTA2	4.77363022	4.39210219	4.36360995	4.82578943	4.86899833	4.9696728	5.02601736	4.52305224	5.16685422
MTA3	1.93622173	2.25211657	2.23217817	2.3463917	2.29130637	2.09560277	2.0275952	2.17132112	2.36540765
MTAP	1.225065	1.53786567	1.47079666	2.05743311	2.27952432	2.16771458	1.76339087	2.02268365	2.12141089
MTBP	0.53207681	0.37731431	0.99967865	0.95687525	1.15938438	0.77127373	1.00445374	0.71236582	0.78979957
MTCH1	6.18812969	5.16113908	5.54596868	5.10637786	4.94487671	5.57311963	5.60776862	5.81890076	5.0770071
MTCH2	4.78638081	4.81232892	5.13938495	5.40252321	5.18141395	5.03006519	5.78352329	4.99789698	5.74195598
MTCL1	1.97675384	1.71840876	1.0906957	0.93768932	1.69382122	1.43230746	0.73142092	0.5675588	0.25955189
MTDH	4.70399146	4.95300785	5.28489275	5.71283045	5.35901972	5.92003543	5.87476471	5.59531813	5.26974865
MTERF3	2.48198727	2.87876097	3.52970754	4.16307256	4.09721761	3.69100336	3.32689195	2.93638312	3.37549302
MTF1	1.73948739	2.16537476	2.19894841	2.11247601	2.40065063	2.43519096	2.12999354	1.62168902	1.93226947
MTFMT	1.96894383	1.76511021	2.69183906	2.46865956	2.28732745	2.02870625	1.89304063	1.27070528	2.29163588
MTFR1	2.63498745	2.94112387	3.4675018	4.26702892	3.55806027	3.56702749	4.11197562	3.35079718	3.25783707
MTFR2	1.47704881	1.23056308	2.28719277	2.18612469	3.13441114	2.41316696	2.4705933	1.01813515	2.36044967
MTG1	1.33202275	0.78311656	1.32305014	1.33619432	1.58629362	0.89455913	0.90133727	1.40990889	1.84952615
MTG2	2.4910058	1.97782178	3.03994133	2.30862191	2.18183317	1.93248726	1.93203337	2.20980855	2.44983966
MTHFD1	3.22684821	3.35182246	3.23091579	3.51106325	4.51709352	3.81006209	3.7781	3.78545052	3.77394369
MTHFD1L	3.20728324	2.0165465	2.72036969	3.03706417	3.22972639	3.1756501	3.75312892	1.99433367	2.79708213
MTHFD2	4.32123424	3.04331968	4.12282404	4.44963087	5.22165282	4.01056452	4.86361814	3.63084271	4.13163851
MTHFD2L	0.83566974	0.75422613	0.77715101	1.23491525	1.13215483	1.03510566	1.15637406	0.72288524	1.25957329
MTHFR	2.4519698	1.81684257	1.35104299	1.59289228	1.80432291	2.14181726	1.7705046	1.07890242	1.56264247
MTHFS	2.49986499	1.41459762	1.77619777	1.53735083	1.97294153	2.00652259	2.22250075	1.73347633	1.39747546
MTHFSD	0.97137155	1.21665333	1.62022905	1.45543328	1.57620959	1.5239739	1.18634752	1.53726812	1.68062432
MTIF2	2.85087019	3.39222799	3.61293124	3.97248433	4.23225613	3.97506838	4.32182923	3.69613277	4.0926238
MTIF3	4.89378491	4.89788916	4.67635098	4.58003923	5.05152057	4.50804031	4.43502502	4.66495245	5.39414285
MTM1	2.20101721	2.9566038	2.66959113	2.54550338	3.21308168	2.82766146	2.5502496	2.48080957	2.49371527
MTMR11	2.8170474	2.28217293	2.83147171	3.15751832	3.95621131	1.86568673	1.16886569	3.25210396	5.14039965
MTMR12	3.20169328	3.33695152	4.17070967	3.39242788	2.9473418	3.23875059	2.9500533	2.90795691	2.85609636
MTMR14	3.46927583	2.4001204	2.90161227	2.70120745	3.19878462	3.05534	3.1574683	2.9597474	2.9375905
MTMR2	3.08277356	3.29197116	3.6311762	3.8955748	3.74153046	3.68673533	4.18077769	3.03114563	2.98238395
MTMR3	0.95511722	0.75923329	0.9422106	0.67247913	0.66124401	0.85508016	0.58137328	0.5006994	0.56953881
MTMR4	2.74732843	1.9924609	3.16812445	2.70778449	2.92197927	2.77603302	2.58369979	3.45337155	3.30011847
MTMR6	2.61015065	3.39879803	3.44382986	3.49004972	3.66162486	3.4644343	3.49246642	3.36292106	3.7061458
MTMR9	0.89814888	1.8261405	1.29951472	2.27570571	1.51132664	2.15604963	1.68513889	1.57563772	1.13588838
MTO1	1.08095901	1.48259078	1.39167124	1.77324376	1.8959106	1.28107869	1.77675923	1.00245064	1.69486559
MTOR	2.58561977	2.53065083	2.24191473	2.76186681	3.2341544	3.02766267	3.00741232	2.41769451	2.99232312
MTPAP	1.53989791	1.77920248	1.62161927	2.70092811	2.4903925	2.25915357	2.01044104	1.30483879	2.4319083
MTR	1.54654644	2.71916461	1.92722278	2.47504151	2.65485011	2.78626077	2.2764773	3.24419482	2.45833574
MTRF1	1.91160783	1.46626615	2.45810644	1.81452435	2.27890695	1.70354361	1.41644295	1.71652443	2.40708554
MTRF1L	1.39922994	1.82778824	2.14164585	1.78697669	1.76557107	2.08215	2.14935487	1.64112	2.08762648
MTRR	2.04461882	3.01140245	2.56578314	3.07090918	3.06220177	2.85412975	2.12523138	2.65081852	3.2374684
MTSS1	0.54692499	2.34974351	1.59428615	2.47770717	1.11912927	2.01744613	1.40159295	2.17971702	0.55665657
MTTP	0.00673582	2.67778608	1.44946738	0.02410141	1.58395407	0.28495638	0.0080605	0.27408765	0.25620332
MTURN	0.53467524	2.21125011	2.44056583	2.18880386	0.62369967	2.33417915	2.32137031	2.36282621	1.01034034
MTUS1	2.31907805	3.58583219	3.74269874	4.59653077	3.97643612	3.80630192	4.14778791	4.4685517	2.83157375
MTUS2	0.02566378	0.07231549	0.15924212	0.07565562	0.01323201	0.09658846	0.03067152	0.0247363	0.02696744
MTX1	3.20520099	1.84089914	2.39332204	2.04133261	1.74007228	2.18968387	2.71038729	2.6986475	2.59144819
MTX3	0.24499778	1.68993335	2.24686385	2.39924906	1.91907053	2.01615923	0.55387143	1.7943249	1.67236317
MUC1	7.85956139	4.00915145	7.13089759	3.50737557	6.98515352	5.9628533	7.43185717	8.01107322	4.12593897
MUC13	2.52912636	6.1888026	7.34865754	5.98787892	8.0776608	1.78482713	8.45570648	5.4077815	7.74145795
MUC16	0.01981941	0.01327476	0.05307747	0.02522051	0.03791571	0.12572997	0.45183759	0.03607972	0.00871505
MUC17	0.09199683	5.4562309	1.45091003	2.48786201	3.10777269	2.50896494	4.64705546	0.30304111	4.67079924
MUC2	0.03130659	1.05226968	2.6574926	0.22786155	2.45788699	0.53814684	6.58302224	0.29212566	1.14522476
MUC20	0.59010218	3.92219691	3.24275402	0.78583355	0.24706678	0.19154911	2.62802122	2.34187607	4.72778942
MUL1	4.11605987	3.78495484	3.18345133	3.92881092	3.42674456	4.04361783	2.40892155	3.44574168	3.98763215
MUS81	2.89350977	1.89390858	2.32464013	2.07589714	2.12686281	2.20910238	2.08942166	2.32463987	2.70678834
MUSK	0	1.85292989	0.45517379	0.19530137	0.05271166	0.64295129	0.04620237	0.0624465	0.0136773
MUSTN1	0	0.2344548	0	0.07094939	0.14211111	0.07238139	0.02398594	0.01548929	0.02108336

MUTYH	1.75900435	1.43048439	2.44115515	2.37284206	1.68236575	1.42920555	1.42788375	1.84213645	1.99612687
MVB12A	3.88747847	2.6864703	3.04515023	2.78442106	3.18065957	2.64359849	2.54851634	3.64824297	3.19860551
MVB12B	1.09374829	1.88386365	1.85432776	1.44286019	1.71363746	2.06223232	1.66852388	0.80805667	0.78876002
MVD	3.11051215	1.3113013	2.06631477	2.14780415	1.70485001	1.94605425	3.17019859	2.05949259	2.58145226
MVK	2.61359066	0.92666382	1.11775719	1.45153085	1.09895159	1.4777933	1.55302696	1.58231718	1.85173461
MVP	6.1075381	5.61421506	4.83540955	4.54092791	4.86024864	4.73257277	5.52706456	5.66453734	5.39472516
MX1	1.7295503	5.45871191	3.55328774	3.35617942	4.78120849	5.61232239	2.12407444	1.96251429	3.46050548
MX2	0.72126776	3.88882371	2.48742774	2.23723453	2.12554014	3.3561096	1.31979299	0.86463495	1.5406655
MXD1	2.62640516	2.91914293	3.56377944	3.75232117	4.80169604	4.30168261	4.22334715	3.48692202	3.0112507
MXD3	1.60125605	0.49558871	1.20136419	0.80965865	0.95983847	1.33339045	0.66878466	0.85028995	1.32753796
MXD4	4.52026	3.52134809	2.91546012	2.72533599	2.36940955	3.32576166	3.28603601	2.86190306	3.22109294
MXRA5	3.78900296	5.5310814	4.5136092	5.32516946	3.63545355	5.35112311	5.4327514	3.56873751	5.7519166
MXRA7	1.96882778	3.80547714	3.09110121	2.9876968	1.09180637	3.87434551	3.44919303	2.47740588	1.78058667
MXRA8	3.71004582	5.96354684	4.10854316	5.35662343	2.44545911	5.44186108	4.64493608	3.56672398	2.6327686
MYADM	6.08863492	5.49853669	5.16060107	5.60104336	4.99625916	5.87568044	4.44054039	5.33187169	4.62045677
MYB	2.05319551	1.36177283	1.7929864	1.5651019	2.68823679	2.81781794	2.63169146	0.44153234	4.80433016
MYBBP1A	2.8213074	2.22539017	2.82646788	2.9947231	3.42550261	3.36704141	3.38899094	2.10148159	3.92793326
MYBPC2	0.02918894	0.13941832	0.55344552	0.0464196	0.13294529	0.13766948	0.01754352	0.14065457	0.0607011
MYBPH	0.02005113	0.15345752	0.07115277	0.04765915	0.04891569	0.07234228	0.08221774	0.00776114	0.07244276
MYC	4.42777505	5.27321213	4.74894563	5.54940314	5.93082836	5.83983203	5.7354911	5.31350119	6.77429239
MYCBP	3.23835877	3.23835849	3.79136725	3.73046043	4.0902975	3.51013416	3.46485159	2.59445025	3.84193993
MYCBP2	2.44968909	3.10786868	2.38661648	2.7146406	3.58888857	3.13668743	2.61649824	2.27251707	2.78541198
MYCN	1.21435286	0.38499861	1.73798477	0.28156715	1.32950769	0.36218616	0.908854	0.89288861	1.4271533
MYD88	4.29693928	4.3844414	4.9100855	4.42637936	5.36098356	5.13443899	4.73272678	4.60664249	4.65326677
MYDGF	6.75741259	5.21792237	6.22427001	5.56069495	5.72289349	5.88500203	6.78322114	6.41804581	5.91584133
MYEOV	2.24556522	1.72472729	3.11184625	3.77961552	4.25107708	5.09371338	4.02704739	2.48179844	3.65789355
MYH10	1.84975433	3.82909304	2.02877874	3.62758203	1.40373927	2.93409306	2.4749643	1.69689915	1.56121866
MYH11	1.80388182	7.62828297	2.70639012	5.28585794	1.24960778	7.21466751	1.36291686	2.15058938	2.01660226
MYH13	0	0.06115759	0.52299429	0.03739414	0.0349361	0.05382023	0.03059863	0.02847844	0.03281425
MYH14	5.77410703	4.06265408	3.73005973	4.26321461	4.98778789	4.51700477	5.17111828	3.8411774	5.38933716
MYH3	0.23589037	0.14262651	0.32646392	0.30016374	0.25207661	0.17167705	0.03227565	0.08962478	0.16757605
MYH6	0.00560207	0	0.05177667	0.00671939	0.03754334	0.00411877	0.0067043	0.00432022	0
MYH7B	0.17300908	0.18733688	0.28251179	0.20762888	0.1063836	0.09316488	0.07764428	0.20749553	1.06692362
MYH9	7.30160055	6.78180213	6.33756399	6.8479932	6.53010138	7.19892472	7.03397699	6.20704559	6.64511133
MYL12A	6.99192155	7.0191285	7.43580295	7.52276583	7.73407591	7.27025069	7.54729497	7.19537197	7.30942416
MYL12B	8.29301854	7.68996604	8.49360352	8.26096872	8.78534278	8.2874171	8.54163656	7.99564998	8.20422443
MYL5	1.62439974	0.7818154	0.82847814	1.2402826	1.13523607	0.65595747	0.87103738	0.99484103	1.58582751
MYL6	7.05001754	7.03297145	6.87287535	6.55666512	6.12926026	6.63584071	6.4913566	6.67328104	6.3451666
MYL6B	3.15860428	2.51369712	1.96998415	1.94164845	1.65700274	1.994484	1.81918146	2.90992844	2.25732797
MYL9	5.68313969	9.13777171	6.35650847	7.34599719	4.26339236	8.38174422	5.96158006	5.03019711	4.46022687
MYLIP	2.92217407	3.85003018	3.66786641	3.57209773	3.39549215	3.53041953	2.56096039	3.97439298	3.48100633
MYLK	1.82196773	5.84992819	2.93250542	3.85852075	1.95807073	5.01148007	2.49154402	1.88166519	1.41590658
MYLK2	0.05996769	0.05505339	0.15900849	0.12653527	0.11592331	0.20517251	0.14627248	0.05555601	0.07524829
MYLK4	0.043284	0.18215889	0.33445733	0.15685878	0.12936674	0.11307485	0.12241234	0.12727963	0.16505988
MYNN	2.079686	1.80528527	3.1797491	2.23196247	2.43073195	2.09876729	2.02755902	2.4236384	2.00785691
MYO10	4.10944427	3.56265603	3.73882383	3.02714066	3.54246288	3.07298851	2.95666711	3.52494972	3.60741914
MYO15A	0.02081315	0.0754969	0.28064263	0.07029395	0.01518269	0.03937158	0.02488253	0.04341264	0.04536072
MYO18A	3.11033725	2.7013858	2.51142957	2.73541491	2.84085098	2.97711665	2.40621796	3.28971843	3.15882786
MYO1A	1.05367766	3.23749191	1.31775872	2.80458063	1.5293481	1.26378721	0.14930512	0.51601252	4.78230906
MYO1B	2.81346756	3.76491239	3.35280286	3.55684419	2.27072249	3.05312784	3.82633082	2.60364437	4.21186167
MYO1C	3.85721848	5.18561752	4.74920585	4.7606604	3.95782933	5.39408938	5.05378373	5.33211643	5.01790456
MYO1D	5.17394558	4.95896327	4.18292357	4.93262652	3.8722954	4.62562679	4.7355947	5.7568419	5.1559328
MYO1E	2.90900705	3.57569104	3.25460747	4.25551706	4.09756487	3.3885118	4.37365516	3.29620053	3.6009198
MYO1F	2.45861354	2.30051398	2.78792894	1.92223832	3.08822544	3.03459521	2.46020276	1.76429639	1.82848782
MYO1G	1.43715286	1.52502901	1.73030373	1.09895304	2.15713771	1.82464775	1.34063532	0.69998411	1.28198853
MYO5A	0.92788299	2.23195065	2.12844575	2.05941719	2.57487206	2.53468729	1.7384361	1.44383566	1.12013758
MYO5B	2.18673217	2.91052165	3.05158209	3.73559617	3.76512702	3.76811246	5.03451703	3.03924085	3.69591125
MYO5C	2.26528419	1.62970474	2.73701031	2.80465778	3.03224536	2.50803608	1.7460792	1.20211511	3.14278353
MYO6	3.93058212	3.24026706	3.41445317	4.08346515	4.60593545	2.98575783	4.47917127	3.4890872	4.36136367

MYO7A	1.32853219	0.65517035	1.38011435	0.75080007	2.03244903	1.19016256	1.29735332	1.01174218	0.6148528
MYO7B	0.61183304	3.47752037	0.93391854	1.07783064	0.83750572	0.21583455	1.69097547	0.2699804	4.75747625
MYO9A	0.80807958	2.0230084	1.50044475	1.43426753	1.28413677	1.73291357	1.24175987	1.15344058	1.12485205
MYO9B	3.97646297	3.54893622	3.77794289	3.45667669	4.25654246	3.96528236	3.55310087	3.3702988	3.00761826
MYOC	0.01729212	1.2559723	0.24237929	1.23881951	0	0.55979031	0.0309069	0.09722172	0.07135834
MYOCD	0.14816993	3.04161609	0.74727163	0.97817989	0.19381845	2.33612596	0.46462879	0.3620316	0.20864406
MYOF	2.73202207	5.11299607	4.05471653	5.48498674	6.10294428	4.9629355	4.97632753	4.62511356	2.66146635
MYOM1	0.34803938	1.54890193	0.79550844	0.60285814	0.20055066	0.87199203	0.16891575	2.71528643	0.19079127
MYOM2	0.21977033	0.60448949	0.2398918	0.1470541	0.13061172	0.32160589	0.23338018	0.31021627	0.22323031
MYOM3	0.03116725	1.00839552	0.21626179	0.59861243	0.13427056	0.08839779	0.10675294	0.05655685	1.99754049
MYOZ1	0.17778333	0.57364267	1.42953692	0.14820041	0.11208721	0.23225847	0.62266275	0.24272839	0.03682039
MYOZ3	0.00602434	0.56732188	0.29837434	0.04282088	0.01479988	0.11711129	0.0179565	0.05926161	0
MYPN	0.01028759	0.0551146	0.44289107	0.02151826	0.034564	0.03374291	0.0153684	0.26465295	0.01887007
MYPOP	3.41100306	1.89745157	2.61758455	1.71959292	1.98704814	2.2335415	1.7791723	2.20030339	2.2884148
MYRF	4.74357913	2.13194557	3.87236165	3.67836462	3.91975124	2.8697709	3.37715859	5.33603683	3.25082925
MYZAP	0.04207278	1.23635325	1.0735376	0.73362536	0.7645291	0.4190601	0.73744947	0.35335746	1.37104348
MZB1	1.7275613	1.62966426	3.49743162	3.42245578	3.1145906	2.55875709	0.84666264	2.13926432	3.34399913
MZF1	2.06314133	0.81835504	1.61580395	1.84086046	1.94716248	1.49071959	1.02349001	1.50109329	2.03137074
MZT1	4.18122112	2.93360326	3.66961438	3.87505515	4.04865269	3.87243883	4.65867132	3.50360075	4.37686282
N4BP2	1.20143816	1.39191962	1.43483725	2.16882504	2.27605119	1.38667157	1.14708875	1.58054719	1.38667405
N4BP2L1	1.00250839	2.03011632	2.1705327	1.78821206	2.27958772	2.03946082	1.09197439	0.87767139	2.33162099
N4BP2L2	1.68743125	2.17611747	2.59491245	2.59221219	2.65640118	2.25651697	2.03943669	2.04509519	2.96661037
N4BP3	1.7819554	0.74698003	0.63163535	1.24853331	0.28702878	1.17499354	1.15938492	1.27334775	0.41118597
NAA10	3.46281747	2.50223868	2.50687975	2.55514156	3.46067739	2.68781692	2.52473223	2.50309998	3.656449
NAA15	2.33847868	2.51864239	2.88725675	2.97225644	3.84806749	3.41467801	2.90640201	2.94950354	3.32809437
NAA20	4.32035385	4.19198804	4.12922653	5.01746933	5.20270702	4.44663763	4.81313194	4.21054405	4.73615272
NAA25	1.48714663	1.75589567	2.15699089	2.4672051	2.99149008	2.21927876	2.11918263	2.6864542	2.53405023
NAA30	1.8364875	2.33303329	1.95013535	2.51583004	2.32365238	2.35818097	2.16909167	2.376289	2.28881898
NAA35	2.16444522	2.37317721	2.93765673	3.33619428	3.24553543	3.0164866	2.92746935	2.97238939	3.20136964
NAA38	4.48209325	3.51707492	3.95095839	3.41263622	3.61131478	3.59429605	3.30792771	3.00277408	3.91241775
NAA40	2.79476669	1.65831591	2.7807372	2.52738079	2.35474816	2.02999183	2.52825346	2.52268429	2.7606591
NAA50	5.07684758	4.45285576	4.47486709	5.07929217	5.49341986	5.16243964	5.32473876	4.73053872	4.48204274
NAA60	3.06973448	2.45155096	2.05505337	2.03429499	2.06419621	2.27944322	2.30039709	2.80592657	2.48452333
NAAA	1.74707747	1.99710439	3.01377993	2.58492744	3.02082136	2.73502671	2.94381447	2.34077211	3.08263025
NAALAD2	0.01995779	0.58177717	0.40276637	0.48445761	0.09579073	0.58893491	0.07043167	0.10635609	0.17394036
NAALADL1	0.40580646	1.85280687	0.94836267	0.76623883	0.52861985	1.13323239	0.51980483	0.29149466	3.25454613
NAB1	2.1105656	3.0600571	3.247895	5.12963517	3.70314636	4.36100902	4.34290485	2.94897826	2.71112731
NABP1	0.89387423	1.22940064	1.73634244	1.93032399	1.94892557	1.44845036	0.80487938	1.65352188	1.09092487
NABP2	4.86678007	3.94468293	4.49243606	3.78214439	3.73332199	4.06728388	3.69960257	4.45108287	4.61985873
NACA	5.45142963	5.13725481	4.98982738	5.84370313	5.92828894	5.3501942	5.42536908	5.99967962	5.95312527
NACC1	5.65905942	3.63947143	3.96344593	3.82657347	4.78746577	4.18836703	4.08579159	4.32227818	4.2538104
NACC2	3.60403725	3.56515428	2.40791551	2.11744925	2.21688975	3.47986167	2.28161737	2.6695469	2.29915276
NADK2	2.33603067	2.98216444	2.13253331	3.34464416	3.03277659	2.7944465	3.15378453	1.80215526	2.53072717
NADSYN1	2.12581696	1.82347476	2.41613019	1.95800492	2.01029191	1.71246372	2.17583576	1.91391642	2.79101193
NAE1	2.54782484	3.15571853	3.4546842	3.83762771	4.25174518	3.70968946	3.80408381	3.05894889	4.13043056
NAGA	5.07285865	4.43607704	4.58009589	4.06951282	4.18553456	4.48743036	4.76726477	3.68442746	3.3460816
NAGK	2.57442661	2.83263755	3.15051826	3.03436355	2.88046153	3.14020988	2.60736083	2.23135502	2.52783647
NAGLU	4.57122486	3.67387363	3.52716385	3.40682853	2.9474732	3.74408882	3.48005784	3.90080698	3.68526872
NAGPA	2.00288467	1.86859098	1.74253464	1.58937098	1.55771422	2.06941114	1.9910567	2.02554834	1.72045706
NAGS	1.08335882	1.38973166	0.57050696	0.70817328	0.45362915	1.82875804	1.06197297	1.09197368	2.86705806
NAIF1	1.87106579	1.23307988	1.77157531	1.7105075	1.53158345	1.60304023	1.53404622	1.48066951	1.68478136
NAMPT	5.79612446	4.91939676	5.58129289	4.90747372	5.97076459	5.04781461	4.48382124	4.59525853	4.34980532
NANP	0.42667314	1.76943942	2.18724914	2.41981305	1.19637102	2.16784831	2.00355435	1.63571634	2.23722654
NANS	3.85678395	2.82306611	4.40024749	2.90752713	4.11866975	3.41347379	3.67641497	3.29346893	3.61277448
NAP1L4	4.07024272	3.18106211	3.10350997	3.82991252	3.86068905	3.84186419	4.21176558	3.64387355	4.1903732
NAP1L5	0.67682616	2.60430434	1.15997312	2.01773189	0.94004314	1.85094436	0.68923677	1.17266414	0.63154054
NAPB	0.88710255	1.6417514	1.7200183	2.06530769	1.57109713	1.46144801	1.75360147	1.2668304	2.37250333
NAPG	2.14072472	2.57858581	3.31690253	2.85768068	2.9501079	2.71327164	3.37649706	2.65145762	2.63252462
NAPRT	4.09014509	3.67495231	4.35764574	3.90586278	3.20371858	3.76112798	4.09087238	5.13305067	4.07752588

NARF	2.99066476	1.73467676	2.84762759	2.33322131	2.18570981	2.00652643	2.26600456	2.68579812	3.09926913
NARS	4.69024437	5.19026755	5.09076401	6.08097564	6.56704913	5.88522252	6.98161344	5.23913608	6.14400154
NARS2	2.42183149	2.50265189	2.9583525	2.70660723	2.81078011	2.63812133	3.24040094	2.66498069	3.3872224
NAT1	1.98780787	1.691064	1.47515161	1.98249803	2.64075091	2.01686449	2.18477607	1.6699691	2.72790297
NAT10	4.14238314	3.13718562	3.49451437	3.96488387	4.06347363	3.71312006	4.27811612	3.98587891	4.35593172
NAT14	3.14920412	1.53599504	1.29849034	1.79636268	0.78799494	1.67480918	0.72871837	0.82719157	1.00046526
NAT2	0	2.88599297	0.42774894	0.24297453	0.24895829	0.05952952	0.44999431	0.11238476	1.75293826
NAT8	0.07498633	3.55543807	0.27738093	0.08952057	0.11392235	0.02800666	0	0.23239272	1.93823781
NAT9	2.69412368	1.34903101	3.08676314	2.12104761	1.67911892	1.6958278	1.73085774	2.75477716	2.50999334
NATD1	1.32843298	2.33951122	1.61601511	1.7552686	1.32868236	2.20956327	1.34104166	2.42375802	2.33783926
NAV1	0.93032119	2.70024261	1.47049665	2.44562498	0.9940982	2.10615577	2.56834243	1.07909009	1.91164814
NAV2	0.38502974	2.01518008	1.56684232	1.49118719	1.45184158	1.70766308	0.58536564	2.20995396	2.07139141
NBAS	2.21909255	2.92864978	2.3601779	2.20565081	2.81026534	2.84089701	3.03400528	2.80709245	3.08896444
NBEA	0.04208088	1.57410885	0.35903185	0.93983561	0.17329976	1.07831642	0.38683714	1.01679788	0.5025589
NBEAL1	0.67674699	1.1684161	1.4237701	1.06302533	1.67462815	1.29802121	1.1432288	1.94496269	0.88418198
NBL1	3.72692029	4.40395946	2.92694693	3.73569129	2.61248881	4.06338822	4.13903741	3.03099342	3.69131926
NBPF1	1.99232671	1.34098886	0.83560406	1.45807895	1.56867235	1.34302175	1.12437854	0.65301069	1.1427571
NBPF26	0.40583978	0.52023141	0.1480048	0.18018355	0.73733416	1.12204804	0.38469612	0.54703655	0.37344125
NBPF3	2.68877603	0.45152237	1.25731201	0.99120802	0.20832873	0.84457254	0.53889103	0.58658046	1.21996417
NBR1	4.17924495	4.42999603	4.21227729	3.83488965	4.24587889	4.23489168	4.13652928	4.88982803	4.81154823
NCALD	0.3837167	2.34300756	1.32197633	1.19475723	1.57138433	1.59829553	0.6762249	0.42895423	1.15604126
NCAM1	0.14160721	1.8098794	0.71916636	0.40193224	0.19430735	0.97654153	0.37628599	0.28434667	0.07616327
NCAM2	0.03861981	0.95556135	0.16602293	0.5631245	0.11579038	0.79530959	0.40382152	0.2445649	0.07135834
NCAPD2	4.17694358	3.58243595	3.97321935	4.01211158	4.36691098	4.39064567	4.50300039	3.61850902	4.58861418
NCAPD3	2.38442954	2.01901183	1.93262623	2.52046707	3.25775112	2.6802703	3.37056506	1.75754055	3.02536405
NCAPG	1.88359323	1.6778224	2.3656289	3.13366332	3.49340185	3.08125174	2.84143794	2.15591118	2.89705005
NCAPG2	2.75983302	1.81133135	2.89488922	2.62285796	3.15979838	2.66013529	3.05106711	2.34153935	2.73648671
NCAPH2	5.44197214	2.3709625	2.83735738	2.54499848	2.73879946	2.98539633	3.09736037	2.68496882	3.25672319
NCBP1	2.86464329	3.50250875	3.81180183	3.76571509	4.45546274	3.46045511	3.62765832	2.74151846	4.04644688
NCBP2	3.95263699	3.26709254	4.84086049	3.92727882	3.93339655	3.83630064	3.74781195	3.90166984	3.48094959
NCDN	2.78079516	2.53379386	3.43678743	2.81459985	2.23941101	2.90384545	2.39996463	2.04497034	2.22759352
NCEH1	3.23527849	3.04333979	5.54167113	4.50841806	4.75907754	3.96313483	4.39036611	5.73031273	3.33548985
NCF1	2.57399652	1.27315512	1.70556259	1.30250048	1.88990131	2.07354415	1.62865428	1.13991966	1.53725019
NCF2	3.40868685	3.28498288	3.8611763	4.03327451	3.97036689	4.28235461	4.03931546	2.92864468	2.54007115
NCF4	2.95417088	3.94601078	4.12046894	2.99467282	3.33303438	4.03446947	2.42647517	2.21479126	2.99278245
NCK1	3.31818862	3.40352475	3.82801861	3.53014759	4.33563171	3.60814207	3.44410404	3.23488468	2.95447127
NCK2	6.01237643	4.59492712	4.70863075	4.34896046	4.04028005	4.50414697	4.40492602	4.47133721	4.79176766
NCKAP1	2.87359504	3.56311509	3.36768574	3.92385041	3.91747294	3.79453376	3.844575	3.77200931	3.38706125
NCKAP1L	2.30267986	2.99705285	3.12144816	2.08251043	3.80986782	3.4161694	2.75180967	1.66046655	1.72226985
NCKIPSD	3.44469664	2.96708205	3.50277206	3.18118068	2.8526205	3.38871352	3.60087768	3.41218009	3.4791548
NCL	5.38807477	5.35958905	5.40457214	6.28782932	6.47586753	6.044048	6.16733981	5.9459871	6.36868575
NCOA1	3.31952783	3.90726847	3.36364583	3.17135926	3.68397178	3.55937973	3.3571087	2.66221059	3.25648278
NCOA2	2.61528193	3.25196764	3.17609641	3.02254012	3.70680206	3.44909047	3.53060918	3.55377983	2.93592992
NCOA3	2.68750958	3.0564677	4.26112551	3.35222767	4.25086515	3.04712721	3.11554855	3.44729808	2.91661942
NCOA4	5.38330067	6.60512725	6.64441098	6.33767316	7.06167491	6.75956689	5.8119462	6.6281186	6.33851195
NCOA5	3.19643567	3.15162162	4.00994029	3.92111807	3.6005598	3.48187116	3.21257499	3.34165471	4.41509935
NCOA6	3.4350359	3.02538271	3.417106	3.43160535	3.43604069	3.04257421	3.35949687	3.42372893	3.97984348
NCOA7	4.80608857	3.86997291	4.75966307	4.71604185	5.07196956	5.51535806	4.70061	4.42841392	4.1317476
NCOR1	3.04557569	3.71303326	3.68293814	3.67094003	4.5189797	3.52727281	3.89158477	3.05986351	4.32203089
NCOR2	5.00248643	4.07999401	4.00752851	3.67261348	3.91830153	4.18948884	3.42487365	4.99687012	3.98208612
NCR1	0.16363176	0.21477356	0.71044951	0.23405792	0.6176405	0.29918571	0.29121674	0.13451501	0.25870795
NCS1	2.21990491	3.73161208	1.55037337	2.78902757	2.54747965	3.83035855	1.98624147	0.81731285	1.65146801
NCSTN	5.22524155	4.93766498	5.64886795	5.19080198	5.0646471	5.00349782	6.17864579	5.78402142	5.15253326
NDC1	3.28785351	3.01308979	3.65666316	4.06525432	4.57762643	4.32875591	4.22167088	3.52932068	4.21859577
NDC80	2.25556845	1.79741097	2.55258291	2.62818583	3.8058999	2.60570857	3.09705573	1.82467593	2.36753779
NDE1	2.77766725	2.10108837	2.32395019	2.59575634	2.68004768	2.24865928	2.26182341	1.77035694	2.12039096
NDEL1	2.04874922	2.43734862	2.64843267	2.96447184	2.8754638	3.04486634	2.93642491	2.23789342	2.64068698
NDFIP1	4.51400881	4.63251347	5.04104447	5.18003244	5.21977134	4.68933193	4.65154854	5.11917592	4.53270729
NDFIP2	4.19607166	3.81681364	4.12003457	3.96700928	3.78170622	3.47650332	4.46436667	3.69592139	5.18496512

NDN	1.76855458	4.87075294	2.48615523	3.61346019	1.38951131	4.0208062	2.54587139	1.80564683	1.25227889
NDNF	0.08941102	2.23355812	0.65988403	0.92222397	0.10352855	0.98082218	0.68154104	5.36838452	0.08371822
NDP	0.03922721	0.96730611	0.68469215	0.32920322	0.11756312	0.19767268	0.13618765	0.03781593	0
NDRG1	5.03557825	4.38489462	4.77500671	4.65652247	3.35338961	4.33873374	6.08531811	4.86500853	5.97189676
NDRG2	2.89288042	3.17844499	3.8707351	2.32504007	1.4155089	2.28138539	0.92394362	4.20418886	3.25512061
NDRG3	3.90161801	3.94556264	4.36648331	4.42273213	4.29876726	4.06843236	4.75873466	3.99053075	4.67315466
NDST1	3.46944166	4.02886013	3.33017504	3.40732912	3.42471944	4.0205275	3.22723056	2.96834729	2.95030587
NDST2	0.6803275	0.48333194	0.760523	0.53869546	0.54740285	0.5188122	0.31839185	0.48930078	0.43700287
NDUFA1	7.43064056	6.19931262	7.10026984	6.12669066	7.22372953	5.87503567	6.61134536	6.46221174	6.575478
NDUFA10	3.05303166	3.21652334	2.84296999	3.90303244	4.18242592	3.53948334	3.75998181	3.73070303	3.96151801
NDUFA11	3.28918	2.42378948	2.3789365	2.2962844	2.48265701	2.52144425	2.3680653	2.47386719	2.58356396
NDUFA12	5.29504042	4.76454418	4.08645794	5.00960104	5.46111766	4.65558615	4.73441724	4.96006773	4.86152891
NDUFA13	4.03246479	3.03236724	3.16556794	2.64120583	3.11061177	2.90265576	3.00145624	3.40986709	3.2549999
NDUFA3	5.92069439	3.86893021	3.85962509	2.95043823	4.04104233	3.02376439	3.4165644	3.24031259	4.00970378
NDUFA4	6.71095253	5.6330334	6.47970972	5.63772308	5.70800412	5.38450973	5.6667788	5.86287447	5.64404297
NDUFA4L2	2.95792754	3.5299141	3.94304652	3.04412842	1.83952818	4.0363103	2.91604795	2.42639025	2.94056274
NDUFA5	3.12030368	3.02010983	2.7696093	2.90959529	3.1870361	2.7207224	2.73180406	2.83766271	3.22274655
NDUFA7	2.96890644	1.91654829	2.38504678	1.96200648	2.50329681	2.16819516	1.81350483	2.37366867	2.61168588
NDUFA8	5.61498204	5.58113546	5.7531633	5.41726641	5.80885207	5.46696731	5.04437145	5.01023854	5.98346101
NDUFA9	1.83186297	2.07470787	2.17513841	2.3738305	3.01853818	2.38717264	2.43160849	2.14436879	3.01790889
NDUFAB1	5.39024737	5.51478506	4.93008387	5.30847142	5.78623137	5.30786556	5.78813661	4.93338223	5.55285678
NDUFAF2	4.59248873	4.03014162	3.9790375	3.81603204	4.12767008	3.89810244	3.70859139	3.76134772	4.26505628
NDUFAF3	4.61516465	4.13296003	4.01581629	3.46597155	3.62854437	4.2262691	4.22449497	4.4924183	4.28292947
NDUFAF4	2.72449659	2.7702236	2.55904708	3.52153263	3.97311068	3.3989644	3.28122858	2.83376306	3.99608214
NDUFAF5	0.84363813	1.11562871	1.24988086	1.61853434	1.4462949	1.22419479	1.28160523	0.65755747	1.74950039
NDUFAF6	1.03766364	1.31661478	2.12869123	1.82895614	1.88333656	1.33668576	2.02876174	0.87053135	1.39918725
NDUFAF7	1.35043153	1.75784409	2.25068928	2.21171996	2.44061126	1.77515923	1.67962769	1.7813286	2.28491389
NDUFB10	6.51857451	6.43723328	5.0738452	5.44928137	5.51761401	5.81883766	6.10183333	6.59704985	6.32886461
NDUFB11	6.24829046	6.09356112	5.93597731	5.49982307	6.21082615	5.57561833	5.53729904	6.42449471	5.99137908
NDUFB2	4.35526222	3.88748579	4.05946824	3.401904	3.74553395	3.23248413	4.12048341	3.38627615	3.72481116
NDUFB3	5.50488774	4.95674736	5.18229261	5.09639264	5.69074106	4.7446328	5.31563551	4.83896276	5.15131265
NDUFB5	4.21477149	3.68230986	4.64071398	3.77647261	3.69096893	3.62102729	3.82707019	4.17179182	3.86674513
NDUFB6	5.00304228	4.66881019	4.39858851	4.75481943	4.87334113	4.53488306	4.58357201	4.7407573	4.76721685
NDUFB7	8.23225473	6.04362062	6.55836977	5.50317687	6.2243323	6.08856986	6.24217911	6.61817029	6.73305286
NDUFB8	4.22922166	3.73395715	3.11284073	3.62731016	3.88704841	3.67316616	3.52258626	3.72946809	3.80113049
NDUFC1	5.18999723	4.1588944	4.22028841	4.18970908	4.33358497	4.08627417	4.2019867	4.44970555	4.68999951
NDUFC2	4.34060747	3.71350437	3.84582083	3.54186894	4.16662975	3.41125266	2.94590756	3.26497808	3.08243136
NDUFS1	2.64690044	2.94806847	3.36365122	3.25795785	3.4259128	3.19172432	3.87380581	3.90557322	3.87514737
NDUFS2	4.3572219	4.13896391	4.49336424	4.32228653	4.62574233	4.59764664	5.40292978	5.20525756	5.00376386
NDUFS3	4.07957556	3.66923009	3.98814376	3.91911357	3.8935476	3.88936675	4.41285876	4.00547758	4.37965153
NDUFS4	4.81296503	4.88909236	4.89441288	4.86717301	5.23582661	4.71589813	4.60973536	4.85840981	4.99260431
NDUFS5	8.5798651	7.40055169	7.66265365	7.76796871	8.05628071	6.99135085	7.45312255	6.74790048	7.43898777
NDUFS7	3.73341337	2.4150759	2.30079476	1.7977158	2.71626511	2.65748052	2.77093521	3.00119312	3.11449035
NDUFS8	5.11318171	4.17277798	4.46619874	3.96623019	4.01310398	4.0558016	4.48481689	4.57580069	4.56785009
NDUFV1	5.02403705	4.10909757	4.36699316	4.19588129	4.42576171	4.45994976	4.90752566	4.47417266	4.9713362
NDUFV2	2.39207368	2.29798522	3.23806568	2.47846703	3.00163461	2.41533719	3.37757682	3.02180088	2.43906483
NDUFV3	2.28506549	2.35703878	2.17929438	2.41817221	2.36936027	2.37032097	2.1687221	2.7914286	2.7781455
NEBL	0.07793205	1.10564671	0.49331807	2.47303414	0.12589041	0.61566917	1.23404657	2.44009892	2.19925166
NECAB2	0.01439028	0.00826029	0.27503947	0.24664134	0.03520454	0.15120522	0.01721134	0.09699785	0.02262737
NECAB3	2.75200789	2.01885017	2.77187467	2.24332641	1.72916178	1.66241154	2.03792842	2.65145891	2.70486435
NECAP1	2.83113058	2.86166638	3.50220933	3.22149752	3.52524933	3.37480565	3.20670289	3.4836586	3.53132896
NECAP2	3.98523769	3.42251271	3.8395737	3.78777016	4.03783382	3.93962045	3.71653801	3.0877184	3.49823221
NEDD1	2.13624181	2.53000685	2.57143141	3.04087089	3.46042587	3.30030401	2.70400682	2.98668414	2.94099646
NEDD4	1.67526218	2.64363753	1.55089549	2.30358763	1.53123567	2.27178754	1.60704573	1.34247334	1.26642653
NEDD4L	2.03166436	1.85714171	2.40783903	2.22619282	3.0716848	2.08718493	3.33953755	2.25639996	2.26719469
NEDD9	2.62979847	3.19386233	3.95695675	3.59295224	3.26155272	3.73630786	3.4607008	3.64825234	1.54161975
NEFH	0.1893269	0.66816134	0.48088484	0.21941226	0.27966739	0.29074507	0.36043042	0.1614246	0.12599641
NEFL	0.02503786	0.21882435	0.10552553	0.14408631	0	0.10439418	0	0.0257274	0.01759321
NEGR1	0.07927152	2.53397717	0.88127489	0.99161137	0.09393051	1.66423235	0.34282914	0.44122566	0.03943457

NEIL1	0.48595988	0.42218208	1.00350412	1.10808234	0.70711966	0.71675997	0.61318656	0.49258999	1.0130578
NEIL2	3.23772873	2.28199717	1.88305018	2.73512094	1.34170576	2.80420744	3.44666119	2.10705513	2.41525738
NEK1	1.02795708	1.74620046	1.16517018	1.69845755	1.79506219	1.73098112	1.47017195	1.53458662	1.07692709
NEK11	1.74587704	1.07208365	1.04736436	1.0155798	0.83093861	0.84165944	0.95421442	1.23321926	0.37579836
NEK2	2.89370017	1.72664463	2.72031776	2.62094804	3.10646768	2.67425807	3.970596	2.81557739	3.20949386
NEK3	1.84162009	1.93285704	2.47798914	2.01703195	1.40014924	2.29979488	1.6482214	1.93501487	3.31044402
NEK4	2.48770534	1.65576343	2.11997188	2.32893542	2.2127277	1.99964854	2.39218974	2.75478078	2.24103805
NEK6	5.02610507	4.08960516	5.03331743	3.93854762	3.9948059	4.22254833	4.1231362	3.72843626	3.90483402
NEK7	2.3159254	4.2384548	4.09011506	4.46720163	4.22793248	4.76725868	4.4979387	4.66344775	3.73103958
NELFA	3.23233435	2.00220872	1.77792185	2.58804068	2.39963235	2.63661886	2.49236475	2.5671615	3.32622478
NELFB	5.35719745	3.8266081	4.65050413	3.87729017	3.79914209	4.37051125	4.36064672	4.30596045	4.38359744
NELFCD	3.70145429	3.10360805	4.29479414	3.83020696	3.23476676	3.45024577	3.74818653	3.51364534	4.13258866
NELFE	5.12678057	3.87547983	4.86780802	4.14430839	3.92872369	4.27998486	4.2856411	4.88893846	4.26238784
NELL1	0	0.13503496	0.35827212	0.02044715	0.02930327	0.01754869	0.04051845	1.42217161	0.43579853
NEMF	1.33623916	2.50329426	2.16951354	2.6746422	2.95529185	2.35057301	2.36284022	2.42195084	2.60402569
NENF	5.29257852	5.27042305	5.3507823	4.88267227	4.41533941	4.9521612	5.75960925	5.17670329	5.38606358
NEO1	4.50846025	4.19692008	3.69568626	3.40435524	3.4729953	3.40796269	2.57603373	3.68906147	4.81995969
NES	2.54497922	2.4935218	2.27165258	1.98401376	2.02261046	2.31068465	1.64380534	1.93503065	1.06145187
NET1	4.95191527	4.88347151	4.10620627	5.65033253	5.35202592	4.81878428	5.48661675	4.8406456	5.48660302
NETO2	0.35645158	1.04936789	0.88672992	3.11841255	0.75934479	2.56272782	2.88508751	0.342674	2.99816024
NEU1	1.92904192	3.02802512	3.20843062	4.05357833	2.50334505	3.26192585	2.49842838	1.88500964	1.71373569
NEU3	2.09526892	1.03926185	1.69018629	1.74709901	1.82689278	1.7781074	1.61321889	1.60336122	1.60420304
NEU4	0.00775158	0.6402062	0.03330437	0.0046556	0.03780557	0.31667192	0.06815393	0.63057035	0.2229017
NEURL2	0.20831406	0.64821558	0.52092307	0.56328195	0.13184558	0.40944852	0.14353582	0.47420803	0.53513791
NEXN	1.50268491	4.07632425	1.85300709	3.18922239	2.63451356	3.72960089	1.56398796	1.19570904	1.29558664
NF1	2.20168477	2.6800822	2.23896181	2.81480464	2.85286637	2.3064894	2.40227361	3.32041175	1.8705403
NF2	3.6167532	2.11365804	2.3398458	2.5689074	2.62984825	2.31370927	2.10808698	1.75315462	2.69391455
NFASC	0.11045692	1.88830777	0.78732614	0.61632146	0.41534687	1.69765951	0.18188165	0.49863101	0.17692144
NFATC1	1.0858878	1.87751192	1.58620216	1.90986831	1.15625712	2.37700953	2.14194981	1.60663679	1.21372478
NFATC2	2.22738928	2.63765668	3.40673847	2.35176913	3.27092017	2.60751593	2.52321259	2.49815578	2.30982515
NFATC2IP	3.00713217	3.35788366	3.21499895	3.37637686	3.63090827	3.38050402	3.65847433	3.05953907	2.94431552
NFATC4	0.58498618	2.38804126	1.22322108	1.59412426	0.45496642	1.82516785	0.78283896	0.72018981	0.59748089
NFE2	0.99455175	0.79779248	1.11138572	0.22124486	0.13033294	2.8500745	0.16670702	0.30177884	0.13330782
NFE2L1	6.81737272	4.81741548	4.75884315	4.56356749	4.89518412	4.92783109	4.86304216	5.04857425	4.66976121
NFE2L2	4.05961727	5.02227206	4.89768026	4.72311884	5.06065221	4.83448917	4.40928311	5.40231434	4.710764
NFE2L3	3.85698192	3.71557871	3.45935404	4.29027	4.72686336	4.28233652	4.62095353	4.73671011	4.83071285
NFIA	2.09083714	3.96534879	2.41349771	2.55901495	2.02668456	2.67398622	2.19758846	2.40951223	2.37992651
NFIB	3.41656605	4.21612253	2.65444763	2.35568189	2.74098427	3.77171972	3.26983391	4.89655809	2.70353804
NFIC	4.36454856	4.86029424	3.41034251	3.67618266	2.75753582	4.13197963	3.65273425	4.24419279	2.910308
NFIL3	4.0609237	5.04925416	4.48328792	4.70436641	4.01162147	5.20791563	4.1716884	2.81419772	3.6910452
NFIX	4.69604937	5.45486364	3.81392373	4.19006324	3.97114805	4.5681075	2.95729514	4.49747162	4.76345813
NFKB1	4.27454573	3.62457048	3.71010282	3.84530442	4.25638267	4.31610809	3.6260652	4.21632532	3.13664718
NFKB2	4.51575286	3.12789475	3.86129167	3.89086833	4.11283021	4.66267934	3.63564341	3.94957116	2.98236495
NFKBIB	4.57911662	2.738151	3.49967239	2.7186104	3.69222606	3.39743845	3.13350308	3.17157524	3.14203541
NFKBIE	4.20641099	2.11628327	4.24386549	3.08823588	3.3204971	3.94038651	3.1304665	3.60389761	2.64804753
NFKBIL1	3.91788587	2.32244464	3.17024018	2.02092698	1.72275816	2.56686694	3.12823756	3.28315439	2.66544764
NFKBIZ	4.54224731	2.16395056	4.32519604	3.42873203	3.34373281	3.61330725	2.6024234	3.57999798	4.44056613
NFRKB	2.9357218	2.4112276	2.27250873	2.74954198	3.03651987	2.72242369	3.16169417	1.95555298	2.71295621
NFS1	2.00092136	2.11010169	2.59631606	2.35005503	2.35337032	1.87948506	2.14780407	2.55172173	2.52361049
NFU1	3.60328004	3.67165578	3.59672637	3.75193645	2.67592814	3.70305413	3.29932903	3.07282212	3.19428443
NFX1	2.43405452	3.20633695	3.44325615	3.23910789	3.25234232	3.14978777	2.92183734	3.36615873	3.67739854
NFXL1	1.36909399	1.51375737	2.23384327	2.45021577	2.68048544	2.46758127	2.35739381	2.34297217	2.56022611
NFYA	3.36735134	2.64235125	3.8536876	3.77633305	4.33750205	3.75764524	3.782918	4.58117391	3.46351424
NFYB	2.19960198	3.33595403	2.55203947	3.31484751	3.10048176	3.16853814	2.46914488	2.69361871	2.66199645
NFYC	3.03746979	2.81367041	3.34118057	2.98978331	3.0785676	2.9921718	2.66515966	2.38536556	3.40658734
NGB	0	0.02199249	0.02768396	0	0	0.04202742	0	0	0.02019433
NGDN	2.43640999	1.87851369	2.75213788	2.7055541	2.94881661	2.41028281	2.02750805	2.46599788	2.81383892
NGEF	1.11890736	1.64243377	1.44800744	0.97487337	0.14594411	0.72142378	1.35688533	1.6039214	2.98658166
NGLY1	1.73953411	1.83818195	2.10251853	2.29840343	2.38263173	2.27114757	2.14343219	2.43899697	2.44275735

NGRN	3.2039561	2.43216054	2.30312302	2.44151797	1.95314286	2.51278689	2.57452491	3.44821352	2.7225322
NHEJ1	0.47510248	0.339467	0.45399306	0.56338804	0.6786744	0.50988061	0.41223319	0.47984179	1.02402096
NHLH1	0.01415003	0.04806052	0.08961652	0.03372731	0.06843048	0.09600164	0.07464299	0.04319126	0.02959193
NHLRC2	1.77197154	2.23907387	3.01587693	2.67754937	2.78198651	2.70038051	2.35015095	2.69643175	2.75769034
NHLRC3	2.34021785	2.33768551	2.81379949	2.64763196	2.8938076	2.76587936	3.00307163	2.23054296	2.45072635
NHP2	5.25259057	4.54041252	4.66278406	4.59427543	4.6386712	4.88728669	4.99750208	4.89519373	5.43358016
NHS	3.29203012	1.06944002	1.54712797	2.02468947	2.81827834	1.54897258	1.54448364	1.13988888	0.30305944
NHSL2	0.30446809	2.00948405	0.46069773	0.80467059	0.84330482	1.75500048	0.68546282	0.3850933	0.14641472
NID1	3.20806101	6.20402427	5.23651044	5.21781174	4.3922894	5.75977302	5.30409258	4.202202	3.76664796
NID2	1.92075802	3.95265706	2.83278294	3.42242266	2.5069767	3.64726368	3.33642694	2.43960457	2.18131581
NIF3L1	3.57058363	3.07021084	3.91949291	3.57696188	4.41633506	3.50612854	3.96944826	3.77909186	4.03789198
NIFK	3.65813325	3.83366519	4.67246807	4.81267825	4.74115844	4.28785018	4.09889889	4.17833965	4.74079836
NIN	0.7110043	2.39930972	1.90445122	2.21644768	2.11062307	2.46034972	1.87970469	1.50341539	1.12307772
NINJ1	5.03211197	5.08722549	5.62937582	4.779254	4.84082556	5.78780451	4.44197406	4.08104584	4.27674227
NINJ2	1.35981511	2.25059168	2.14760179	1.61574459	1.32892753	2.51319928	1.5960447	1.15788997	1.02211856
NINL	0.21906944	0.84187311	1.47513967	1.65270624	0.24355801	1.24225941	0.64968898	1.22163367	0.85335677
NIP7	3.31139398	2.9695203	3.46241888	3.49646538	3.83599779	3.51831452	3.59569929	3.09150159	3.38541428
NIPA1	2.91103759	1.48692794	3.04437819	2.53676337	2.90248711	2.49318527	2.33375999	3.16391083	2.74527515
NIPA2	3.78435864	3.72959047	4.57519322	4.18596214	4.40159969	4.43471905	4.54418262	4.20667077	4.40444383
NIPAL1	0.25607287	1.54371979	2.10758789	2.22750051	2.65498955	1.76616711	2.3316571	1.88170747	3.04529007
NIPAL2	2.01631219	3.04560226	3.29723887	2.79415056	2.44918294	2.79384124	2.8707564	3.6824337	2.89875462
NIPAL3	2.31956114	2.2797595	1.98677386	2.51979713	1.74838916	2.40903996	2.27355364	2.0754441	2.60148246
NIPBL	2.38294383	3.34017303	2.75850849	2.76458252	3.60896175	3.16946789	2.81176883	2.69982981	3.26676022
NIPSNAP1	4.95025446	4.83197539	3.72882779	4.03632189	3.55670838	3.66672952	3.90998205	3.63851475	5.29513149
NIPSNAP3B	0.04934605	0.76348311	1.11977822	0.73211343	0.39439364	0.63203025	0.20507417	0.22635116	0.08537907
NISCH	3.01606961	3.16995977	2.97511004	2.8941276	2.34494633	3.03698739	2.82093934	2.7208229	2.76599858
NIT2	2.78108222	1.78884551	2.6855873	2.46002826	2.56322157	2.47110456	1.85658994	2.0088987	2.53255028
NKAPL	0.02206648	0.74068147	0.31611679	0.40451814	0.11829999	0.46406597	0.13945469	0.02548429	0.03464502
NKD1	0.12146933	1.33668842	1.01837695	0.60427463	0.06162527	0.99285589	0.27337893	1.46892944	0.88091474
NKD2	0	0.63386038	1.18366751	1.62062075	0.0077613	0.97701499	1.71832823	2.85128477	1.22611167
NKG7	4.96375135	4.01629809	3.62764076	3.77355919	6.68690483	6.15206758	3.69328864	2.04767172	3.67664323
NKIRAS1	0.74541673	1.92276281	1.84848909	1.90156453	2.10064542	1.84600622	1.42260198	1.27581006	1.20877287
NKIRAS2	3.96772103	2.85791353	3.56242303	3.11249226	2.83081616	3.2405093	2.74179471	3.32595051	3.07043573
NKRF	1.53871432	1.64549996	2.38930095	2.23092502	3.09623533	2.1430344	1.79648295	1.94482336	2.16844599
NKTR	1.16636562	1.71194908	2.15486776	2.61162891	2.76905025	1.995996	1.7579025	2.03146406	2.17316786
NKX2-2	0	0	0.04948588	0	0	0	0	0.01336677	0.03617147
NKX2-3	0.11679884	0.64054944	1.36473022	0.2484293	0.21818229	0.42311234	0.11973331	0.27373664	0.07129193
NKX2-5	0.01730033	0.23837641	0.30488881	0.04117446	0.01068226	2.96502238	0.04108313	0.0655675	0.01818182
NKX3-2	0.6155204	1.54937727	0.06434527	0.69799867	0.03953138	2.30741525	0.43969285	0.23545467	0.14312408
NLGN1	0.02138873	1.04524081	0.22312046	0.19518167	0.01321384	0.43913753	0.0213394	0.03554702	0.00565215
NLGN2	1.4121618	3.45066799	1.47628557	3.13243089	0.76162327	2.7200829	1.6946301	1.10488769	1.28847136
NLGN3	0.309906	1.68607522	0.56843839	0.65193923	1.60935258	0.54931571	0.3468548	0.45357331	0.13676833
NLGN4Y	0.21422325	1.97088128	0.75275764	0	0	1.28881432	0.00265612	0.56957814	0
NLK	3.03248191	2.14555732	2.54435845	2.3641627	2.35075751	2.04067511	1.93410312	2.26986124	2.68073035
NLN	1.58633125	1.17689373	1.57148065	2.29799064	2.48759542	2.62722656	2.3858712	2.14822101	2.31633079
NLRC3	1.05437332	0.80637823	1.44344658	0.92422009	2.34886794	1.23056373	0.76072851	0.55044575	0.87977203
NLRC4	0.60010785	1.31574736	1.54035579	0.86037379	1.78743639	1.87529	1.1870342	0.66510774	0.48478871
NLRC5	2.30283655	1.72666618	2.13873595	2.01679772	4.22618946	2.99808701	1.39633275	1.69122993	3.81119214
NLRP1	0.63619757	1.4858533	1.67103692	1.24000712	1.45286764	1.24907895	0.78257538	0.55411741	0.59659837
NLRP2	0.30110686	0.29233845	0.75040659	0.53738592	3.28994725	0.11806051	0.10011697	2.91028094	2.77819919
NLRP6	0.03369599	2.78599935	0.46601486	0.11778271	0.13999956	0.21310651	0.10493246	0.18859692	3.6175935
NLRP8	0	0.00529673	0.27353295	0	0	0	0	0.03875112	0.00970638
NLRX1	1.92479677	2.47259156	1.65736502	2.08280493	2.1806885	2.60212489	2.50627426	1.81786865	2.20450257
NMB	2.84883824	2.37598125	3.23160918	3.34339379	1.19806771	2.86126664	4.30571528	3.50533334	2.74497436
NMD3	3.58236271	3.37437341	3.76255213	4.14261592	3.35166543	3.66359727	4.2907403	4.47692849	3.58577764
NME1	4.87481906	3.54724961	4.50503186	4.71769053	4.81298954	4.37569621	4.07296013	4.15306337	4.67974599
NME4	2.3839019	4.514914	3.94500776	3.68347673	1.64751146	3.81587666	4.17821435	3.88991968	3.53393704
NME5	0.10289519	0.70944495	0.55880804	0.71364703	0.98250825	0.37284567	0.0537105	0.10200902	0.03949788
NME6	2.24781197	1.90183545	2.2850501	2.07839241	2.15114209	2.25724892	2.28494111	2.30847553	2.0543285

NME7	1.08898579	1.97667391	1.33195753	1.67476825	0.99677374	1.29350643	1.46643246	1.77618087	1.27153452
NMI	4.05246638	3.76760953	3.79878121	3.80377207	5.073474	4.52097282	3.55517065	3.12511315	3.73229556
NMNAT3	0.06201212	1.60301563	1.59717277	1.41859751	0.3758764	1.23441378	1.23779503	1.68596535	2.40542302
NMRAL1	4.04147536	3.35834868	2.87163007	2.83891474	2.81888373	2.8872116	3.04685919	3.60936037	3.36717168
NMRK1	1.34995236	2.28757511	2.47546864	2.43393316	2.35133864	2.36652264	1.78627244	2.52571897	2.54614765
NMT1	4.73882852	3.99699929	3.96473564	3.97699115	4.05915257	4.42827875	3.92309919	4.05230693	4.53744649
NMU	0.95064797	0.28996861	0.51654762	3.95158361	0.15280573	3.48853851	5.00435111	1.6309013	0.8065662
NMUR1	0.39401809	1.25486112	0.75377348	0.34089061	0.22635274	0.38288847	0.40158711	0.13023312	0.2694373
NMUR2	0	0.93601649	3.60413284	0.054888	1.07151513	0.03386143	0	0.07014699	0.1176114
NNAT	0.39136374	3.00112444	0.89402764	0.7959147	0.62287559	1.66373674	0.89745054	0.62339154	0.80517974
NNMT	3.68148798	5.96012632	4.08053048	5.28103043	2.63678161	5.03426059	3.71679571	2.95935609	1.72919835
NNT	2.88342655	3.46241169	3.33151326	3.00922433	3.57360348	3.51971631	3.5171035	4.02978404	3.63178354
NOA1	3.96982399	3.26642873	3.67325859	3.97135794	3.78429034	3.59434334	3.76106931	4.32866044	4.35424749
NOB1	5.17618684	4.39847761	4.67116498	5.04844431	5.08266995	4.82528821	5.09854946	4.89256984	5.54711798
NOC2L	4.13196321	2.49825328	2.36317859	3.38668782	3.5818641	3.44226712	3.81945868	2.69303202	3.89880561
NOC3L	1.8355929	2.54424319	2.1068573	3.23103237	3.77354876	3.18433193	2.25816955	2.99234526	2.93005642
NOC4L	4.51963526	3.1248996	3.67618958	3.15306226	3.56810994	3.78702719	3.81249058	4.25044302	3.76600464
NOD1	1.58148957	1.68673899	1.30401428	1.38958793	1.43417228	1.72247398	1.23013286	0.79512224	1.51275191
NOD2	0.55614303	0.80137038	0.89577534	1.52490478	0.96182025	1.46993911	0.81161064	0.43550746	0.58628974
NODAL	0.02136783	0.06037182	0.03077411	0.0631723	0.02628209	0.09192184	0.075316	0.02467835	0.03355273
NOL10	2.82595315	3.03511444	3.08303975	3.51345172	3.86555556	3.36575269	3.67941717	3.50332335	3.75458895
NOL11	3.47435	3.02253686	3.96588297	3.81575411	4.3964086	3.64104072	3.35532045	4.01944218	3.97022065
NOL12	1.72010359	0.8849213	1.43755466	1.03370427	1.4000194	1.00060879	1.01108354	0.80855447	1.6753497
NOL6	3.71248915	2.98485185	3.95123817	3.62616918	3.87612985	3.94518608	3.51263282	4.03786846	3.76595975
NOL7	4.61953761	4.47931223	4.52531613	5.00633084	5.06974089	4.76726854	4.95383682	5.22970836	5.11234144
NOL8	1.65443509	2.31727207	2.91724253	2.82689966	3.44348155	2.12574418	2.2151898	1.94434776	2.85474852
NOL9	2.35083	1.66203697	1.7408533	2.32145242	2.43809191	1.9916162	2.2034405	1.47001567	2.10770995
NOLC1	4.61952159	4.79910639	4.20632525	5.79818738	6.05108589	5.69023153	5.39885513	5.26590339	6.06416841
NOMO1	4.07689633	3.68301967	3.60435639	3.05456816	3.03882193	3.42378533	4.67421746	3.82338222	3.44188538
NONO	5.91584691	5.66402805	5.46997875	6.02021006	6.52882085	5.8158977	5.73150838	5.88400615	6.17738339
NOP10	7.28699792	6.21711828	6.90876783	6.7551753	7.12544659	6.52578544	6.28087276	5.63362588	6.63711944
NOP14	3.5593342	3.0715427	3.7059964	3.87810953	4.17876477	3.58465709	3.77728453	4.28244163	4.65105074
NOP16	3.11019736	2.30555489	2.73885985	3.22432039	3.6160283	3.40283835	2.84329862	2.90120507	3.61591984
NOP2	3.30090664	2.40697498	3.4341206	3.44343005	3.9147037	3.5102868	3.04375402	3.15212462	3.86327489
NOP56	3.87845289	3.72474455	4.49097155	5.25015473	4.8368617	4.54767035	4.59448625	3.64847339	5.59523774
NOP58	3.79729305	3.76382021	4.33604341	5.21354094	5.59733473	4.79196565	4.63752581	4.66834538	4.96680326
NOP9	3.26815355	2.43519207	2.23814201	2.13055764	2.48325254	2.35833369	2.61076646	2.59224818	2.87468172
NOS1	0.00277663	0.13515319	0.47622101	0.01162523	0.00853555	0.25059483	0.00166275	0.07411809	0.01164014
NOS1AP	0.6557054	0.52550837	0.46515565	0.35489821	0.72495776	0.70755594	0.94434599	0.62879141	0.91072743
NOS3	1.22709713	2.29315124	1.96015526	1.74301784	1.42273286	2.32710594	1.30496438	2.08350926	1.13460075
NOSIP	3.91189581	2.84583438	3.17056409	2.932155	3.40624956	3.05310564	2.94742068	3.24494178	3.46917353
NOSTRIN	2.5238163	1.98193485	2.16161663	2.93236367	2.14664854	2.0708037	1.79946984	2.89125908	2.07410329
NOTCH1	4.36409447	3.29329575	2.70966382	3.00463035	2.74898897	3.27395877	3.136948	2.55121737	3.76108598
NOTCH3	4.27851419	4.35347038	4.12191138	4.51543008	3.2050514	4.30603115	4.03862084	3.951964	3.33408471
NOTCH4	1.14418998	1.54636752	1.99637947	1.5824462	1.46680507	1.57001728	1.31928716	1.11875871	0.99496115
NOTUM	0.03576851	1.50857179	6.16242693	0.38540983	0.04394463	0.34545383	0.08420933	4.39508643	0.97322918
NOVA1	0.0428898	1.37704755	0.33733719	0.40693797	0.03095396	0.70497416	0.04489823	0.26261454	0.0283238
NOVA2	0.64287368	0.79894128	1.00178936	0.65301246	0.46458289	0.97856083	0.42363525	0.33355528	0.18601477
NOX1	0.13735687	0.27703095	0.78379553	1.67237545	0.69733618	0.22052231	0.24532568	0.17254684	0.68621569
NOX4	0.23657379	0.60391338	1.00643059	1.53578508	0.37988601	1.28169109	0.87848768	0.43613555	0.33998592
NOXA1	2.7868948	1.40438508	2.60905581	2.59883987	2.10800033	0.84520911	1.96746903	4.09654583	3.92039311
NPAS1	0.71012493	0.32444921	0.60026409	0.31792116	0.32555195	0.20736274	0.2690116	0.8414751	0.10030229
NPAS2	3.0024617	2.09587987	3.03052924	2.64277041	3.30932779	1.72108386	2.49173934	2.66593621	2.5370231
NPAS4	0	0.09439324	0.0379459	0.06248617	0.00654229	0.17992947	0	0.00409658	0.01114656
NPAT	1.36696661	2.56855045	2.17857696	2.31120895	3.04010481	2.49969255	2.57133539	1.85374484	2.34085665
NPC1	2.23373029	2.27207709	3.63477376	2.79236392	3.75403899	3.1448173	3.72832654	2.97901327	2.36816479
NPC2	6.67840517	6.42358128	7.27709861	6.01729217	6.52952723	6.40027607	6.34276406	5.66447121	5.61481398
NPDC1	4.61735705	4.07510665	4.42118594	4.13760029	2.12633007	3.86844158	3.93271995	5.05113563	5.19549795
NPEPL1	1.0823303	0.76289111	1.55907045	1.06002928	0.67697647	1.10130564	0.75591698	0.89439413	1.45393038

NPEPPS	2.93629001	2.93366994	2.98529725	3.28804772	3.33149436	3.1002996	2.95650204	3.03940537	3.30703637
NPFF	0	0	0	0	0	0	0	0	0
NPHP1	0.44216553	0.35111565	0.58158202	0.49012854	0.31892286	0.28798901	0.17775857	0.29586481	0.22706855
NPHP3	0.2159271	0.77901755	0.88486501	1.29083357	0.61143758	0.63773972	0.47800356	0.52767515	0.57533355
NPHP4	1.52135192	0.64704718	0.61435388	0.96659503	1.04194284	0.92131692	0.76698608	0.72244736	1.12426701
NPIPA1	0.22057671	0.17897858	0.41487812	0.30255506	0.53764791	0.24904066	0.28740212	0.23765539	0.57380703
NPIPB15	0.02125567	0.04824566	0.34264163	1.21835363	1.03024018	0.78685192	1.90853134	1.76665003	2.78782723
NPL	2.36273086	2.68029013	2.87848802	2.92108655	3.73501777	3.41873565	2.69674343	1.74518792	2.04081373
NPLOC4	4.28513696	3.07007547	4.20748578	3.35571699	3.54829957	3.70421495	3.41392435	3.97457408	3.49178086
NPM1	7.03999476	6.42330061	6.31140552	7.54154255	7.93819715	7.40921593	6.96920508	7.24633528	7.47726344
NPM3	5.17782364	3.01418011	2.90874696	3.69542382	3.21738885	4.04639447	3.88771199	4.03763814	3.65748985
NPNT	0.54846895	3.46488185	3.11716057	4.14906615	1.16611726	3.11759387	4.93491865	4.25290646	1.9794919
NPR2	0.73130331	2.62006114	1.96671445	2.43393316	1.23936603	2.10582558	1.44854438	1.0036928	1.25993145
NPRL2	2.68270526	2.36944483	2.93002845	2.52521062	2.42773279	2.31783016	2.91357469	3.13240765	3.41022643
NPTN	4.70833525	4.8078198	5.47114994	5.07896095	5.15608336	5.0418904	4.9845862	4.70486338	4.82799023
NPTX2	0.87122416	1.40621649	0.59870648	0.73406793	0.15512516	0.61289922	1.08617617	0.12484516	0.5307412
NPTXR	0.40402712	1.75237142	0.55596295	1.07972611	0.27805117	1.66137086	0.4908118	0.35550399	0.16269721
NPY	0	1.01522571	0.26082192	0.21978414	0.02417383	1.32865408	0.04661373	0.03018497	0.02065151
NPY4R	0	0.01063333	0.28048558	0.0440355	0.16265207	0	0.01111057	0.07008281	0.00976078
NQO1	5.42259211	4.76862671	6.40158373	5.77892766	5.63171032	4.85040155	5.83013446	7.1005295	3.46872571
NQO2	1.45405796	1.44036856	1.52401814	2.1310992	1.44472783	1.79535374	2.05918071	1.65486747	1.84932353
NR0B2	5.59558732	3.38722562	5.03338621	0.30566911	0.8215845	1.03690584	0.74865021	4.19640506	4.24264581
NR1D1	3.6976307	2.93846695	2.44993129	2.79332143	2.8886362	2.76327602	2.59338962	4.19780645	2.85632063
NR1D2	2.45287419	2.94912027	3.14398459	2.70329979	3.66018969	3.13229156	2.8634176	3.94599801	3.18070272
NR2C1	1.53593143	1.95069602	2.18411036	2.93338043	2.77444448	2.2698486	1.96509684	1.8536607	2.40028019
NR2C2	1.76729393	2.3655007	2.60101528	2.59025597	2.75329677	2.45652517	2.53978755	1.98437221	2.73753149
NR2C2AP	3.58126405	2.14136626	3.13796401	2.75556376	3.45397762	2.95066721	2.51183257	2.91347933	3.23165243
NR2E3	0.03331607	0.05058398	0.29943771	0.03331363	0.00690444	0.04470633	0.04632384	0.0426632	0
NR2F1	1.04568603	4.1120305	2.20481672	1.81630345	1.33274618	3.00546138	1.99293091	1.6609971	0.99457217
NR2F2	3.17401554	4.72711913	3.60688976	3.90296203	3.40651882	3.83326064	3.08289206	5.25845416	1.90001704
NR2F6	5.97149139	3.78931974	4.21161237	3.41825699	4.16562317	4.53271781	4.31690772	5.05278974	4.68292705
NR3C1	1.99079501	3.53290992	3.25403644	2.80767578	2.74217843	3.25214576	2.12543106	2.89556921	1.25076656
NR4A1	2.81187203	3.78926374	3.65888246	3.93995408	1.97963523	6.3286067	4.0328494	4.41496508	3.27292848
NR4A2	1.32723209	3.78308742	2.4287818	3.16380739	2.36799795	5.66798812	3.62085494	4.03873016	2.64427595
NR5A2	0.80067061	2.21262509	1.91246464	0.62794697	2.31305052	0.91386884	0.5221663	0.39710229	2.57124145
NR6A1	0.75221637	0.55103123	0.52670853	0.5114568	0.56404015	0.51069814	0.49318651	0.17611399	1.42290431
NRAS	3.92013972	4.26135141	4.89989086	4.97130169	5.02913563	5.0828657	4.74550782	4.73598334	4.48232235
NRBP1	3.89713633	4.52607972	4.46977365	4.58272561	4.80575851	4.61314408	4.31192686	4.68451337	4.21038657
NRBP2	2.44300494	2.33948167	2.38348542	2.6247813	1.1990008	2.03591303	1.91341975	2.04687155	2.51853346
NRDE2	0.83496271	1.00065344	0.99967612	0.85409554	0.95607852	0.89633204	0.65542993	1.31420135	1.0409576
NREP	2.40575263	3.11158224	2.75334443	3.28934728	1.98493805	3.13155149	2.37612831	1.84826753	1.58336562
NRF1	2.30921662	1.91368222	2.2816979	1.96307198	2.26276125	2.33248134	2.24241372	2.22060081	2.27125436
NRG1	0.01917548	0.17015695	0.84080911	0.17090457	0.07905442	0.29894056	0.68351441	0.18043034	0.51857125
NRG4	0.03577319	0.03686945	0.3996983	0.21744824	0.04827265	0.83965366	0.66276255	0.04940069	0.05234731
NRGN	2.68754764	2.37867223	2.24808338	2.2553514	2.68818641	2.32984672	1.91110602	2.18992324	2.0263159
NRIP1	1.82459687	3.43769584	3.30777213	3.06092638	3.20724867	3.80762337	3.12163646	2.70607763	3.35091723
NRIP2	0.67339142	1.8299842	1.53018145	1.14481351	0.76515676	0.98802852	0.65246908	0.8883209	0.8969915
NRIP3	0.25829554	0.68450705	0.85603549	1.2522671	0.49328595	0.87061685	0.9387888	0.26957001	0.52729162
NRK	0.17880075	1.99084363	0.12380403	0.91819914	0.03017136	1.60911603	0.03155731	0.03335958	0.0218462
NRM	4.3905764	3.8734371	4.10790729	3.54316336	2.89933692	3.48524086	3.88374023	3.35899588	3.31904435
NRP1	1.96614427	4.58062972	3.68064397	3.54754838	3.12193058	4.15602975	3.68780829	2.82253324	2.39901449
NRP2	1.72945336	3.13927019	2.23178459	2.61549119	2.50791058	3.44203897	2.30590462	1.89147844	1.18022461
NRROS	1.61230439	2.29961954	2.42191792	1.75263693	2.07338318	2.77738857	1.93813242	0.97104033	1.12554005
NRSN1	0	0.04903907	0.06158219	0.01041174	0	0.1078552	0	0	0.00912607
NRSN2	1.82056758	3.37415941	3.0333359	2.73402878	1.40607218	3.32786291	2.66830626	1.71394262	1.77869091
NRTN	1.79081944	1.73226439	1.32052667	1.86839441	1.11417904	1.00913542	3.07576709	2.07794187	1.24182032
NRXN1	0	0.37015317	0.14082011	0.10674761	0.01720932	0.41612481	0.00360029	0.01232579	0.01260625
NRXN2	0.11260852	1.38843585	0.15108056	0.22885037	0.07040475	0.55690006	0.05859248	0.03540864	0.04281431
NRXN3	0.19894125	1.50849683	0.37189939	0.38514252	0.18540983	0.80397739	0.30531441	0.14154783	0.08203099

NSA2	4.09445433	4.44997309	4.61163082	5.19264785	5.34414903	4.6016459	4.28325838	4.43105885	4.93922347
NSD1	3.03205714	2.79237622	2.8982348	2.85827776	3.20079761	3.00514619	2.37702984	3.24282562	2.78378172
NSDHL	4.79796921	3.43988818	3.32943514	3.7764545	4.27103504	3.4126587	3.61118946	3.80531311	3.78515399
NSF	3.63541556	3.69700634	3.91377706	3.97923321	4.19821221	3.73926732	4.04434893	3.7739596	4.07461686
NSFL1C	4.28171492	3.24742805	3.84429753	3.48021826	3.03997445	3.39356984	3.47815977	2.8420521	4.36454166
NSL1	2.99037756	2.83954169	3.18256128	2.99766419	3.29985859	3.04459329	3.49658691	3.02668774	2.8787731
NSMAF	1.97595434	3.08090319	2.92967209	3.44135354	3.32943122	3.22586342	3.58803184	2.79159431	3.04437079
NSMCE1	3.34576368	4.38045031	3.7418427	3.66887905	3.46455563	3.64726084	3.74181522	4.0338046	3.83683966
NSMCE2	2.26323288	2.59015802	3.50910696	2.79677653	2.68409577	2.68356803	2.42850549	2.6766969	2.35984732
NSMCE4A	3.62043069	3.47083616	3.98064924	3.55398168	4.55517936	3.41147108	3.23477751	2.94220672	4.2564696
NSMF	2.34070991	3.61217743	3.81727978	3.53200661	2.6075222	3.1773891	3.89489201	3.68281143	4.32138273
NSUN2	3.43663783	3.21875102	3.54267751	3.86959516	4.31635856	4.00377602	3.62301974	3.75264518	4.07089585
NSUN4	1.87224284	1.48244125	2.38820662	2.0679758	1.82666527	2.19253401	2.02809178	2.11045551	2.26992433
NSUN5	3.77661526	2.35845228	2.35358799	2.51128165	1.89228887	3.21135616	2.79119866	2.78611934	3.27518557
NSUN6	1.08363757	1.66338456	1.21413398	1.73032012	2.32821086	1.69459017	1.2814961	1.03123913	2.07431293
NSUN7	0.0229416	0.86167334	1.19489359	0.36927827	0.17423939	1.28627928	0.89061042	0.09763263	0.08267276
NT5C	4.80062689	2.7345086	3.66130071	3.29395727	2.65074029	3.18962368	2.89893903	4.10333241	4.14994034
NT5C2	2.39037888	2.96968039	2.97256382	3.63559939	3.9778689	3.39373924	3.05665521	2.82154287	2.90692534
NT5C3A	3.56747013	3.61386765	3.68077325	3.63107111	5.34683642	4.19680169	3.4342833	3.30988565	3.87480182
NT5C3B	4.12689139	3.22711165	3.8470435	3.59135261	2.44529653	2.95776666	2.8881549	3.92749951	2.5338026
NT5DC1	2.09293644	2.72434282	2.42015539	3.17588522	3.22770287	2.88014093	2.87521686	2.36032492	2.95809332
NT5DC2	3.66820862	2.62163161	3.90728926	3.98589943	1.31267082	3.6650816	5.17833753	3.82009611	4.02120696
NT5DC3	0.43344556	1.44645255	1.10912197	1.61803142	0.8855927	1.20789213	1.92139019	2.28596744	2.42596861
NT5E	1.2420524	4.88743855	2.72343443	4.20446594	2.66335883	3.77277731	3.67308737	1.30652482	2.19152557
NT5M	0.12455899	0.50649369	0.47600385	0.65252898	0.41501023	0.61363926	0.58614795	0.68768359	0.72250433
NTAN1	2.85822096	3.3752918	3.05136242	2.98914604	2.7388563	3.18074682	3.55691418	2.46653233	2.28311253
NTF3	0.10820748	1.03277536	0.59711972	0.22816147	0.07493538	0.16047009	0.2592522	0.31439795	1.69108951
NTHL1	3.14548294	2.8761223	1.47118226	1.54953785	1.96388947	1.80339512	2.77339407	2.59082894	3.30844486
NTM	0.74457961	0.74686826	0.72306403	1.78890993	0.51781927	1.44217752	1.63126108	1.0753061	0.58325916
NTMT1	3.03809563	1.98789927	3.17788639	2.08825767	2.31665966	2.88956929	2.33736423	2.27631902	2.51635346
NTN1	0.52139477	2.70687769	4.1177507	1.85653088	0.3232237	2.07107638	0.53853182	0.9363574	0.67352322
NTN4	2.67734304	3.43689756	4.6267196	2.12362494	3.32123723	2.49762489	2.89938319	3.96907171	2.15442111
NTN5	0.05734423	0.06550654	0.16740676	0.11465068	0.05653834	0.04235981	0.00698664	0.03122506	0.10665611
NTNG2	0.60869297	0.51204805	0.39658799	0.48333915	0.25149189	1.02352568	0.2188974	0.19091131	0.08303119
NTPCR	1.33348164	1.5848268	1.58531462	1.49480003	1.2288574	1.44735629	2.01599133	2.06009015	1.5131537
NTRK1	0.03308419	0.27810026	0.23385168	0.097053	0.08991448	0.232323	0.0540655	0.0475767	0.04332104
NTRK2	0.02484159	1.04179646	0.28107925	1.45816446	0.04022903	0.34568388	0.37274833	1.88680193	0.02979473
NTSR1	0.00857606	0.37530746	0.14192797	0.93249052	0.01580992	1.06414985	0.1420876	1.36277318	0.1213474
NUAK1	1.61611859	1.85771001	1.51704602	2.07435082	1.02961886	2.41331542	1.5725894	1.44947971	0.60507689
NUAK2	1.59712076	2.45169366	2.29338388	1.69945989	1.80359263	1.60099773	1.77040476	2.14494088	4.20014254
NUB1	3.83519568	4.20692637	4.17472703	4.2277091	5.32862837	4.79463584	4.17928737	4.01464608	4.36971614
NUBP1	3.3321295	4.04039147	3.70619416	3.65684802	4.350438	3.75698628	3.41321631	3.17363388	3.81642778
NUBPL	0.82279951	2.06414145	1.48595137	1.38153181	1.7010199	1.28576789	0.84393665	1.22048619	1.34853498
NUCB1	6.71034017	6.15237668	5.95916834	5.63722501	5.70644479	6.05154838	6.139906	5.51514468	5.43879765
NUCB2	2.73529751	3.11551097	3.12504127	2.9069765	3.3114092	3.90763341	3.63926712	4.2919278	2.20178235
NUCKS1	5.24400924	5.54527425	4.72135665	5.45904017	5.34264669	5.4709606	5.86283299	5.92179317	5.20714301
NUDC	6.04039261	4.66705597	4.88298718	4.72611286	5.45560836	5.13305009	5.01853498	4.72513468	5.14607368
NUDCD3	3.43655066	2.95057669	3.08780526	2.67799521	2.73715832	3.119552	2.69770607	3.09184836	3.18211098
NUDT1	3.85255433	2.56449423	2.10009701	2.1574682	2.49561752	2.38454453	2.52456912	2.03005055	2.40982123
NUDT10	0.01763518	0.67699183	0.15770393	0.63543388	0.06413723	2.09173843	0.14152412	0.02037148	0.00929658
NUDT11	0.10318664	0.96981706	0.28008125	1.01657023	0.14326446	0.77911243	0.41796357	0.21821503	0.09330333
NUDT12	2.27107372	2.87326484	2.73663974	2.76172478	2.27714847	2.92499043	2.51741865	2.55735669	2.04232558
NUDT14	4.70398404	2.86011245	2.9548474	2.33628758	2.12250953	2.36908146	2.74209501	3.24507139	3.66184118
NUDT15	3.99155212	3.78451716	3.90574529	3.68339671	4.14726395	3.97929551	4.59445499	3.45528236	4.8277566
NUDT16	3.18198621	2.17112183	2.22706477	2.40467046	2.1039619	2.43186737	2.37117199	2.68851471	2.41306432
NUDT16L1	3.8382172	3.13433825	2.7595224	2.78038396	2.2494828	3.02039528	3.64495733	3.01116706	3.30077872
NUDT18	2.67688347	1.80603423	2.05282297	2.44719837	1.71654782	2.62760892	2.27008378	2.44687489	2.45526541
NUDT2	3.34215914	3.71307171	3.63526088	3.65085535	4.66882566	3.83760202	3.31214545	3.83110762	3.39553763
NUDT21	4.1180722	4.34539774	3.87219172	4.46611841	4.66598178	4.34899498	4.80619418	4.15160851	4.52206867

NUDT22	3.18682048	2.25813131	2.64001619	1.94529041	1.81807562	2.64402939	2.73926154	2.94457675	3.02766755
NUDT3	1.90663373	1.71644533	1.7914687	1.86675799	1.63912935	2.10540661	2.0005275	1.91457378	1.81789458
NUDT4	1.81317187	2.88823128	2.87544292	2.57524809	3.02399041	2.79002867	2.35749951	3.85877966	2.40255356
NUDT5	3.21279461	3.25501951	3.24555626	3.77037316	4.08832601	3.65040448	3.61516539	2.7216155	3.99367079
NUDT9	3.05021846	3.46274682	2.73869536	3.355361	3.46393232	3.38312589	3.33759465	2.73025278	3.53307625
NUF2	1.64104611	1.09797982	2.19109791	2.62753639	2.9111356	2.26434122	2.47179602	2.05541978	2.36706661
NUFIP2	3.63839329	3.55512836	3.63709461	3.6359737	3.89862316	3.90594548	3.70629332	3.92726169	3.5499326
NUMA1	4.28035493	3.39634701	2.8367091	3.17900235	2.99892593	3.39457671	3.10194247	3.54735161	3.85459652
NUMB	3.39957709	3.34626059	3.665031	3.32991549	3.59267057	3.36394649	3.24146434	3.84828651	3.55139201
NUP107	1.99664935	1.88313428	2.69051752	2.50860172	3.44977098	2.53587361	2.18920252	2.37460462	2.65613473
NUP133	2.36011538	3.05173098	2.98532432	3.06755658	3.48278308	3.21701346	3.80302065	3.43941914	3.41142835
NUP153	3.44506886	3.43937888	4.10370942	3.8949714	4.41858523	4.04294228	4.07072654	4.13646627	3.99168354
NUP155	1.93389068	2.39367094	2.33068459	2.85356224	3.46254041	2.59033088	2.5031885	1.66936441	2.9769043
NUP160	2.19222686	2.84270973	3.09241715	3.23722265	3.41256716	3.26594211	3.73303482	3.15665745	3.33555539
NUP188	3.62504682	3.1757845	3.88129363	3.85094045	4.23366962	4.00365039	3.99707623	2.7293613	3.76551294
NUP205	3.32650196	3.1130029	3.62693547	3.47884655	4.22471926	3.24713011	3.79792796	3.2100768	3.90521569
NUP210	4.34427161	1.72899582	3.88344901	1.45675998	3.92116166	2.53625604	1.2276051	3.74039039	2.77450449
NUP214	2.65115855	2.72340233	2.88630268	2.59546768	3.19872585	2.66982852	2.59358668	2.96655069	2.93764837
NUP35	2.30625451	2.26782321	2.23092032	2.87613576	3.43045355	2.66669454	2.88724326	2.53983962	2.82402022
NUP37	2.27756188	2.67416666	2.89914625	3.12882528	3.69813392	3.11486826	2.94307626	2.80394241	3.19419168
NUP43	2.38504078	2.49469993	2.92775765	3.34111766	3.42593124	2.83599488	3.39638007	2.72672271	3.37540653
NUP50	3.5054022	3.18137071	3.21250941	3.32469663	4.32011449	3.63339364	3.55565721	3.29311973	3.6852816
NUP54	2.82546733	3.42959372	3.20862461	3.72784986	4.04682581	3.64601974	3.49264535	3.33473928	3.87904603
NUP62	4.47437627	3.08531013	3.53211658	3.26077762	4.38935955	3.85076668	3.46829831	3.6053696	3.82301278
NUP62CL	1.43555491	0.67516226	0.23383516	1.33229362	0.11550562	0.76214083	0.51528341	1.39345688	1.61472932
NUP85	3.32184995	2.58065742	3.55468233	2.95212487	3.3385993	2.72593019	2.33643716	3.19143674	3.28767835
NUP88	2.43411815	2.77016261	3.70545641	3.2024053	3.67471074	3.11611339	3.45946818	2.56954765	3.94975383
NUP93	2.2367961	2.0164055	2.30016265	2.53271084	2.82012987	2.7257877	2.67817433	1.92815281	2.48917414
NUP98	3.46078506	3.65593892	3.32153287	4.01494664	4.37969095	4.14350605	4.35068396	3.96630155	3.92340303
NUPL2	2.64604368	2.36068672	2.35543107	2.77813263	3.21968317	2.49754668	2.10575085	1.91360134	2.63172208
NUPR1	4.18824053	3.26446248	2.37157749	2.98127226	2.75051796	3.12505838	2.63527815	1.56541627	1.23715794
NUS1	2.82438633	2.98593751	3.20717836	3.52983967	3.51437699	3.6469384	3.83380868	3.20452348	3.49602786
NUSAP1	4.2740082	3.4614866	4.83255572	5.00319483	5.43240316	4.39032754	4.78789811	3.49979301	4.69301782
NVL	1.55422025	1.83612096	2.40394486	2.07124299	2.70902597	2.09967034	2.32090554	2.39957508	2.55728264
NXF1	3.31559616	2.84237804	4.12469475	3.92963365	4.06766787	3.28092536	3.28682468	3.51648497	4.18207948
NXF3	0.01029921	0.17800783	0.31414293	1.40160991	0.6461068	0.44427951	0.07827177	0.10373973	0.05333216
NXN	1.91002944	3.14007655	2.14722671	3.02818344	1.32839902	2.71533543	3.02669911	2.32589544	0.81456795
NXNL1	0	0	0.0546191	0	0	0	0.02287911	0.01477254	0
NXPE3	0.46880786	1.74637171	1.4949348	1.48032014	1.66070342	1.88602316	0.90664732	0.76279637	0.732523
NXT1	4.7813041	3.64458681	4.35081603	4.2358497	3.92640278	4.40598925	4.21090966	3.9137369	4.67507609
NXT2	2.61933907	2.72679479	3.50869936	3.40613581	4.58209731	3.34318631	2.65660328	2.88563038	2.94646679
OAF	4.75326349	5.35167068	3.65886951	4.21494487	3.60083342	4.43337742	4.6597962	2.99812061	4.67997488
OARD1	2.67862139	1.95085185	2.68434911	2.15391381	2.94605193	2.0908256	2.51774719	2.52531333	2.11718118
OAS1	3.57440833	5.09339338	4.04426037	3.57071937	5.3403665	5.15993625	4.98873257	4.13792683	3.5073586
OAS2	2.86056633	5.83783125	4.5612863	4.04180831	6.25111631	6.1927225	3.27228147	4.23363554	3.13626144
OASL	1.55901429	3.20159322	2.70402796	2.71821532	5.09908997	3.8900836	1.62340956	2.8707908	1.35148029
OAT	4.37566655	5.69614254	5.26205524	4.8789239	5.46254106	4.43946363	5.11493446	6.04047607	5.4266235
OAZ1	7.78542713	6.47529924	6.75463133	6.35999261	6.54507255	7.09176635	6.93956862	6.15696934	6.94295725
OBSCN	0.08864559	0.20964019	0.42919774	0.61346908	0.26826082	0.45866576	0.34455179	0.91388338	0.51865954
OCEL1	3.7391442	3.33057992	2.73126919	2.64436629	2.02707866	2.37046044	2.33158027	3.03044183	2.78725182
OCIAD1	4.16419711	4.37990687	4.68915848	4.92487133	5.04653444	4.59695825	4.49158491	4.63345674	4.76175551
OCIAD2	5.46890333	4.27520931	4.88823051	4.65160914	4.75443383	4.53306787	5.01605617	5.80105013	4.60289684
OCLN	1.88807452	1.06896128	2.27296288	2.47555017	3.29171975	2.95755644	3.35764732	4.22207505	2.99390128
OCRL	3.11920852	3.26466002	4.66241391	3.2739464	3.52385563	3.37926066	3.016356	3.20002364	3.3039379
ODAM	0.11218419	3.87693323	2.20489244	3.73476028	0.13701909	1.61164656	1.35538317	2.35444059	0.5110105
ODC1	6.55399166	4.19242592	5.23052281	6.10462024	6.35708265	5.38083291	6.15696653	5.59749767	6.39238681
ODF2	2.82401553	2.30136961	2.93577058	2.62538895	2.85628442	2.65185864	2.73917625	1.92281074	2.5484075
ODF2L	0.26781776	1.37405452	0.95068903	1.97103128	1.50841475	1.68263557	1.37921282	0.48014795	1.26668065
ODF3B	3.47822271	2.77044243	2.33488916	2.25126016	3.34062389	3.54913375	1.86468406	2.07001539	2.34808019

ODF3L1	0.19893776	1.65697467	0.43399972	1.69731743	0.30359426	0.84123705	0.28088767	0.27698517	0.2761346
ODF3L2	0.47738478	0.15897206	0.21091692	0.10894289	0.05073991	0.1111127	0.01249101	0.0476682	0.08554115
ODF4	0	0.05318311	0.25029826	0.07374157	0	0.04560612	0	0.01210016	0
OFD1	2.05494333	2.13725721	2.57308872	2.26236391	3.2118843	1.88366872	0.94185169	1.96313142	3.41010353
OGDH	5.01641765	5.10632253	4.94781057	4.69801567	4.53136732	4.75181316	5.07015845	4.81495226	5.40087948
OGFOD1	2.62705871	3.25638066	3.31139914	3.44191734	3.52781014	3.40966756	3.56749915	3.02243156	3.33890792
OGFOD2	0.54103701	0.36261597	0.40574479	0.28731382	0.43427626	0.22303194	0.36601124	0.62773315	0.5639866
OGFOD3	3.79512877	1.77222309	2.85478268	2.03126224	2.00174261	2.3769235	2.219713	2.51015091	2.29456251
OGFR	4.03731317	2.58366454	3.34265809	2.50840036	3.17304068	3.27994954	3.18860387	2.96655988	3.44451626
OGFRL1	1.40115685	2.864516	3.23735902	2.77413588	2.75812358	3.09257779	1.91075297	2.65906221	0.86865786
OGG1	1.9061365	1.36498229	1.54418376	1.28611811	1.10632292	1.25391234	1.52977754	1.05526684	2.15331469
OGT	2.68361292	2.83547091	4.29431087	4.20950509	4.84967798	3.52725148	3.09724425	3.71444348	4.90048367
OIP5	2.3642845	1.49292459	2.08192915	2.07892522	2.65263916	2.39035461	2.46003168	1.35153356	2.39053927
OLFM1	0.25872117	1.92558938	0.96124875	0.86400471	0.18686536	1.46082606	0.47126421	1.93748599	0.18784873
OLFM4	2.59458118	11.7887127	13.6164378	2.70356479	7.3872408	8.82758971	5.54581934	4.76968984	5.85423449
OLFML1	1.09235579	4.21157791	2.30175734	3.45531732	1.5349246	3.00227455	2.43791962	1.44361535	0.92632871
OLFML2A	1.62739236	2.1521195	3.4443407	1.83144624	1.95396973	1.91338069	3.00453123	1.79770579	1.89387668
OLFML2B	3.17172851	5.13098497	4.28632727	5.12212428	3.35381206	4.59597747	4.31910783	2.86467864	3.08292173
OLFML3	3.1313428	5.98283437	4.83454822	4.58238242	3.2909009	4.89274474	3.30524019	2.64859131	2.29146608
OLR1	3.27082106	1.74726471	2.10683177	3.42918369	2.20455293	3.42983478	3.44601603	2.49825501	0.80137553
OMA1	1.29912993	2.5926744	2.53256798	3.09994836	2.79743234	2.64730826	2.13489025	2.54646179	2.5100512
OMD	0.16836144	4.30403955	1.57281922	3.22472556	2.16628255	3.02777963	0.43504961	0.35422147	0.19710326
OMP	1.7711306	0	0.05254777	0.08625306	0.52890143	0.13004328	0.35232755	0.33028509	0.34470148
ONECUT2	2.16181375	1.70466282	0.95678137	1.44293862	2.29184218	2.11507911	2.80133533	0.84323722	2.75666553
OPA1	3.44754272	3.36345695	3.98052229	3.68041706	3.92118889	3.61192906	4.03240626	3.65482063	3.97177052
OPA3	2.39632537	1.46527532	1.6745153	1.56626546	2.18096106	1.68641329	1.48603899	1.6553543	1.64925867
OPCML	0.00479815	0.10611785	0.11026147	0.07855896	0.01765626	0.08069703	0.01431368	0.03296341	0.03000131
OPLAH	3.20167508	2.03173553	2.83419283	2.2365401	1.73295428	3.23900893	4.62867704	2.99218906	3.98132269
OPN3	0.58406725	1.22715583	1.37923574	1.0648834	0.90853029	1.55260779	2.16899002	2.35175186	0.84684363
OPTN	3.59303917	4.15782142	3.58035655	4.20191911	5.28910075	4.73666699	3.33040957	3.41421277	4.55011096
OR2A4	0	0.0656095	0.10868498	0	0	0.02842279	0	0.04447338	0
OR2H2	0	0	0.20136321	0	0.01410486	0.05799466	0	0	0
OR4F16	0	0	0	0	0	0	0	0	0
OR51E1	0.28296024	1.00932732	1.01802632	0.69284595	0.74877623	0.65797003	0.75378772	0.38043673	1.25631104
OR6B1	0	0	0	0	0	0	0	0	0
ORAI1	4.81444404	3.61948431	4.23874819	3.65129197	4.09001906	4.63055604	3.72688729	4.43500185	3.81305564
ORAI3	1.93342234	3.05369862	1.7986699	2.51874844	1.60278531	2.56304072	1.71243756	2.63776664	1.93489472
ORC1	2.52433372	1.11992591	2.52840395	2.44505891	3.35930488	2.58166957	2.52276453	1.7613468	3.04018992
ORC2	2.14692905	2.12521078	2.83146218	2.20895212	3.39342735	1.85022551	2.43654699	2.71218885	3.01468845
ORC3	2.45377953	3.44940006	2.82279147	3.57284171	3.76249202	3.25657384	3.04679108	2.45527249	3.48043902
ORC4	1.6698828	2.15192597	2.60724554	2.64104728	2.48265943	2.00170116	2.33838506	2.18810926	2.7335703
ORC6	1.54160945	0.96123322	1.77691424	2.07425437	2.6146716	1.58445413	2.04012052	0.95939097	1.78395426
ORM1	0	0.04561019	2.11843841	1.67968461	0.07288346	0.25789185	0.36212452	2.63677209	0.66857348
ORM2	0	0.02720973	3.01937162	0.46351559	0.40885062	0.19730385	0.02842364	0.73653791	0.2952907
ORMDL1	2.69655508	2.79357522	3.54815253	3.52207305	3.49492232	3.33276025	2.93648392	2.71248637	3.23685763
ORMDL2	3.66322371	2.96296413	3.78334687	3.18775507	3.00104755	3.42858287	3.12916063	4.18667164	2.94232756
OS9	5.58759821	4.97915702	5.59358779	4.98902565	4.82109393	5.04527188	5.57740463	5.75118449	5.19020066
OSBP	3.76246203	4.19892215	4.30614117	4.32332705	4.04216038	4.28021005	4.7959654	4.71961553	4.6535314
OSBP2	0.22609945	1.38824686	1.42553509	0.95345134	0.36515384	1.07035793	0.18035445	1.10398291	1.05115577
OSBPL10	2.2371314	1.92809913	1.46792633	2.71246029	2.44539487	2.08340579	2.59800653	2.18002977	1.38522986
OSBPL11	2.56670782	2.74272933	2.71964519	2.77711249	2.93134026	3.00369945	2.9388291	2.49849833	2.45251409
OSBPL1A	1.09970397	2.7870049	2.47665964	3.14967823	2.84490283	2.85458308	3.1912406	3.11249847	2.96252014
OSBPL2	2.40158599	2.32832586	3.33178451	2.27490882	2.03113624	2.00514426	2.79797953	2.60411158	3.35870212
OSBPL3	3.46456317	2.44954613	2.51326723	3.61018439	3.60415172	2.54035779	2.74769237	2.54639823	2.96829946
OSBPL5	1.4285628	2.21525509	1.5087317	2.33042431	1.20075331	2.46636367	1.52281768	1.17199767	2.02934372
OSBPL6	0.08920134	0.39362095	0.50374857	0.67006201	0.1555046	0.41547905	0.13930423	0.1083383	0.06840185
OSBPL8	1.95541155	3.70567149	3.61988789	4.07012182	3.83146383	3.70360007	3.73128388	3.57963846	2.52886974
OSCAR	3.30173363	1.67853798	1.76162511	2.08031491	2.37345672	3.0623346	2.17839233	1.11324617	1.18947654
OSCP1	1.84090618	1.2418316	1.89654091	1.26454847	1.05471318	1.12114643	0.76065391	1.20107793	1.38130605

OSER1	4.09454775	4.24106741	5.30670187	4.47436487	4.39469679	4.29766615	4.31614103	4.40681326	4.99690647
OSGEP	2.03188312	2.21225504	2.52904784	2.97458997	2.39860124	2.32579578	1.82367459	2.22364185	2.58453443
OSGIN1	2.52797176	0.5583377	1.16321951	0.81376741	0.72436908	1.17341396	0.7649191	1.2722811	1.45741621
OSGIN2	2.3885673	2.84011147	3.43215456	3.28324815	3.12372057	3.4190192	3.2726372	2.39671619	2.22362763
OSM	2.88384643	1.28534669	2.29850711	2.31584593	2.47807152	3.25924891	1.45700328	2.96309624	1.82021232
OSMR	3.76694016	4.50812805	4.15054799	4.04016307	3.40437949	3.88673756	3.96883591	4.57300412	1.68090782
OSR1	0.04727032	1.06365188	0.25815432	1.41376973	0.05395516	1.11627498	0.2554244	0.49275965	0.1506243
OSR2	1.90370006	3.69005862	1.50779491	2.39000366	1.13076417	2.73708982	2.33668554	0.27423012	1.99020467
OSTC	5.68409181	5.86492895	6.3484742	6.32064447	5.90355764	5.94064173	6.40203032	6.16961593	5.8832459
OSTF1	4.60223407	4.94310337	5.41208219	5.63840597	5.6243041	5.20151383	5.38670498	4.73852213	5.0624692
OSTM1	2.2615875	3.38406003	3.30754917	3.41374198	3.40803609	3.44216554	2.92323365	2.26452291	2.11900334
OTC	0	3.720108	0.95917447	0.02732986	2.71093312	0.01679861	1.51140329	0.21376821	3.17136496
OTOGL	0	0.07995035	0.33745255	0.1903865	0.18576988	0.06101107	0.03599429	0.04456222	0.03165442
OTP	0.01628383	0.00468249	0.0408257	0.06718549	0	0.01496448	0.0146298	0.02812653	0.03402705
OTUB1	5.42743905	4.13070513	4.88621151	4.67438327	4.60926063	4.64972376	4.96365704	4.84225078	4.96094617
OTUD4	1.93654072	3.04746777	3.17496344	3.3788129	3.77573743	3.75858865	3.25833068	3.38694106	3.58248687
OTUD5	4.39046664	4.14679275	3.89983126	3.86654356	4.24045642	4.05957573	3.52190003	4.13702089	4.20686551
OTUD6B	1.33598	1.48031132	2.01526132	2.48928326	2.29777604	2.36772289	2.43682552	2.23541355	2.10839478
OTULIN	1.72082727	1.97807429	1.99927352	2.06691077	2.543726	2.20061461	2.08487906	1.83649916	1.99210929
OVGP1	0.32868109	0.63451421	0.9759392	0.76272123	0.32725392	0.33380932	0.23477787	0.4718384	1.95073499
OVOL1	0.34379534	1.38558452	1.73677602	2.01148504	1.82009703	2.21677988	3.11716412	0.85959282	3.73134514
OVOL2	4.00054488	1.53077073	2.4675907	2.15357093	2.06241839	1.99538449	2.84972611	2.87904274	1.52318681
OXA1L	4.6424315	5.11721186	5.19937223	5.40173897	5.64281766	5.21282897	4.64555541	5.25388043	5.48679073
OXCT1	0.70703577	3.38970252	1.96601447	3.95806155	3.50824957	3.9060441	3.39884067	3.21749174	3.49045094
OXCT2	0.03937555	0.16203657	0.15048638	0.30147264	0.04836246	0.17907844	0.06996103	0.01530366	0.08157857
OXER1	0.48248929	0.83184508	0.86377425	0.37611501	0.62457242	0.68215722	0.56945806	0.34241043	2.05440977
OXLD1	3.67026393	2.51393475	3.47921992	2.99489114	2.71874094	3.02317178	2.55502724	3.14963152	3.6517856
OXNAD1	1.77420022	1.50945152	1.83437489	1.44135805	2.26512435	1.73285917	2.20473037	1.91087409	2.13131616
OXR1	1.83379999	3.19277286	3.98032638	3.25999597	3.04574066	2.97302419	3.26865464	4.0105014	2.66271236
OXSM	1.0545908	1.93469277	2.54727764	2.58768375	2.6443466	2.4930489	2.57911305	2.5403424	2.88418108
OXSR1	3.15729879	2.94979902	3.11449395	3.71586046	3.61633253	3.50069309	4.1479043	3.56664952	3.62344884
OXT	0.06934901	0.2255692	0.09916401	0.40597126	0.08497831	0.34641303	0.16080262	0.10556694	1.02528438
OXTR	0.33047815	0.61553178	0.63214178	0.50898579	0.39366721	0.54044625	0.27625702	0.22167097	0.16427997
P2RX1	0.17282426	0.90108072	0.86985947	0.6946154	0.56387798	1.98545184	0.31472828	0.33057106	0.62778214
P2RX4	2.44522685	1.69911154	3.13566428	2.02325203	2.5611959	1.67041734	2.0299978	2.35764704	2.15440706
P2RX5	0.33945467	0.36360287	0.44983489	0.56665222	1.33926992	0.89663357	0.25687462	0.1220779	0.52657357
P2RX7	1.2538097	1.5614887	1.71247093	1.59692504	2.5978133	1.8509807	2.00072291	0.73431205	0.71935602
P2RY1	0.23844301	0.85310525	0.87218668	0.46427922	0.9619888	1.01783891	0.53654461	0.45357028	1.38615371
P2RY10	0.83561091	2.36208631	2.19320329	1.79549562	3.34084183	2.3084819	1.46533528	0.8516903	1.49144712
P2RY11	2.16295201	0.60122767	1.15873381	1.29057223	2.35884853	1.47062286	1.55442861	1.43360749	1.50877467
P2RY13	1.69969816	2.21037093	3.01406875	2.61128105	3.76082802	3.07749989	1.37713985	1.56488839	1.71243628
P2RY6	2.08200695	2.08371858	2.35541388	1.51032755	1.91200927	2.51017497	1.45917234	0.92687458	1.17358364
P2RY8	1.58761852	1.82544223	1.8525291	1.33692784	2.27369786	3.10395721	1.17081626	1.05110356	0.9831336
P3H1	2.1476986	3.36828124	3.32986798	3.15352629	1.93440079	3.09577631	2.96943733	1.87666398	2.06799706
P3H2	0.37569213	1.60381833	2.98774146	1.03312173	3.24480318	1.37372634	2.02510949	0.82760814	0.58938637
P3H3	1.62077568	2.93159658	1.49199015	2.4119118	0.47401889	2.67906885	2.65113786	1.19721295	0.88183125
P3H4	3.09617733	2.7322267	3.27155323	3.30136966	1.81452309	3.2505603	2.95311024	2.95184131	2.09660593
P4HA1	4.28231309	4.35550682	4.46601634	5.30431187	4.2350764	4.98923207	5.61054827	4.5275357	5.15163242
P4HA2	2.55114558	2.89994239	2.52583741	3.12442202	2.24586958	2.89741908	3.73364773	2.82207239	3.20522416
P4HTM	3.06452699	1.61281012	3.01895999	2.3990179	2.02405318	2.04584035	2.28304365	3.31028049	2.63860453
PA2G4	5.3164283	4.91406152	5.07373546	5.81803077	6.36521366	5.54812429	5.32084762	5.62702775	5.95486192
PAAF1	1.42079224	1.57613611	1.50916307	1.5836948	1.41395141	1.32685881	1.24364202	1.89800266	1.78628403
PABPC1	8.17245882	7.83305979	8.93847875	9.25840601	8.234892	8.44198114	9.29676487	8.79536991	9.05053927
PABPC4	4.04234869	3.68583522	4.46125304	4.70933389	3.79659071	4.08584478	4.19009388	2.8740259	4.04736418
PABPC5	0.07211358	1.1038016	0.27060258	0.63554114	0.19452347	0.56713052	0.16136486	0.09073033	0.0966385
PABPN1	4.72701192	2.25978453	3.25135782	3.13828178	3.34572965	2.64254477	2.52208061	2.74354355	3.54279253
PACRG	0.23153733	0.56750681	0.37123941	0.25976323	0.06717618	0.15076549	0.06531711	0.15293181	0.11281812
PACRGL	1.24616635	1.42391665	1.83633723	1.99714433	2.00796547	1.60932788	1.26688777	1.54225863	1.81666169
PACS1	3.47353284	3.69501703	4.17320185	3.46542647	3.60395162	3.78600526	3.45764533	3.48051569	2.733277

PACS2	2.74743074	2.50750475	1.97518771	2.34373407	2.41453256	2.38613992	2.45460368	2.88020433	2.58773088
PACSIN1	0.08107663	0.45760598	0.12581019	0.26895828	0.22845999	0.1124958	0.03142418	0.06009525	0.06239421
PACSIN2	5.35669	3.98453549	4.50166666	4.38667052	4.2740868	4.02392131	3.76780971	3.88727881	4.24789322
PADI4	0	0.43367215	0.67988039	0.43508297	0.0950399	3.12685657	0.10614339	0.2229701	0.14116531
PAEP	0.20959753	0.0424417	0.42105314	1.18375911	0.1605771	0.57061092	1.46885707	0.04751232	0.17357968
PAF1	4.29318133	3.62786656	3.92284662	3.84577885	4.15324339	4.04730546	3.78460812	4.25140533	3.84229495
PAFAH1B1	2.79818262	4.03634483	3.97168064	4.40411652	4.09029269	4.26459909	3.91223222	3.38907082	3.97913148
PAFAH1B2	3.73645363	3.85253284	3.36565376	4.54784392	4.07123561	4.18281404	4.46618836	3.18041399	4.08110326
PAFAH1B3	5.98108246	3.12252634	3.51809408	3.62060768	4.15393481	3.65278865	4.10328634	3.98374057	4.74057818
PAFAH2	3.01339076	2.99165291	2.50418959	2.90632986	2.99339841	2.99201645	3.06517023	2.65151825	3.58540351
PAG1	1.09073295	2.37941704	2.47244309	2.27055006	3.18467074	2.38412432	3.43686124	1.54171943	2.54808776
PAICS	4.29433791	3.26111337	3.70943469	4.88171856	4.46017241	4.27939648	4.60021306	4.21942472	5.41123183
PAIP1	4.28851724	4.11451944	4.12273325	3.76546975	3.8145838	4.11678835	4.21663501	4.12153406	4.32921711
PAIP2	4.24110687	4.41482827	4.52871087	4.53463206	4.31815659	4.31160281	3.78653719	4.30094504	3.8535064
PAK1	3.82029642	3.76529004	4.00805564	3.86924123	4.51318569	3.56442749	3.66385174	4.10528504	4.46165637
PAK1IP1	3.10544098	3.2978768	3.59359928	4.2026106	4.43602887	4.09581014	3.99506787	3.84511675	4.33643943
PAK2	4.18392744	4.51864631	5.50150884	4.82408415	4.41918988	4.9263994	5.07090413	5.33419122	4.70398444
PAK4	4.62475038	2.3602363	3.22842845	2.55375859	3.1581124	2.82383369	2.73961408	3.47644352	3.97360455
PAK6	0.00563324	0.04456683	0.20492998	0.08226681	0	0.07279023	0.13535216	0.0215917	0.13310731
PALB2	1.99858229	2.66943836	2.67760155	2.6055008	3.01749833	2.25982662	2.52107623	2.50602123	2.85109289
PALD1	1.83277696	2.3677856	2.43172426	1.9908164	1.56612877	2.55988251	1.62830303	2.23791067	3.43422427
PALLD	2.93383327	5.8352328	3.88830273	4.94945277	4.35894667	6.00808555	4.58808628	3.21649579	3.21499588
PALM	0.91668697	3.02566566	1.34705914	1.86172693	0.55737118	2.57512859	0.63866242	0.66537931	0.44113286
PALM2	0.02195129	0.28980917	0.33165401	0.23237808	0.08809693	0.16372562	0.11449954	0.08818411	0.07550043
PAM	2.21799663	5.09430624	3.34224532	4.58049296	3.3845538	4.3709602	4.69220501	3.16188946	3.28358856
PAM16	1.65139066	1.28993315	1.0188217	1.4886405	2.01317945	1.47306449	1.77035038	1.54410062	2.04863236
PAMR1	1.08286491	1.12674932	2.16412221	1.20323458	0.95505516	1.06739298	1.20139722	0.90657092	1.07271683
PAN2	1.45419503	1.7229579	2.06189046	2.35662555	2.05668567	1.80630668	1.80403113	1.64716194	3.05669889
PAN3	2.00666475	2.21844651	3.06171999	2.95019567	2.90638357	2.69395379	2.71028451	2.98840059	3.61775147
PANK1	1.25246552	1.52951244	0.83876572	1.80825092	1.7767969	1.49215303	1.63621921	1.25282706	2.53214061
PANK2	2.08910227	2.04398726	2.43891372	2.3915439	2.29650186	2.19121885	2.11363142	1.3486037	2.12375977
PANK3	2.64727966	2.54533306	3.00962999	3.57583162	2.94541566	3.22117859	3.85295308	3.41575208	2.95694043
PANK4	2.61540351	1.80910137	1.62655032	1.87840342	2.24058485	2.1020213	1.77548871	1.5679884	2.45720714
PANX1	2.73866766	3.14891194	3.11964905	3.5356239	3.45033785	3.81553071	3.61726703	3.119346	2.94919298
PANX2	0.13451571	0.2914921	0.22727493	0.29248636	0.04979532	0.45210795	0.09524667	0.06637318	0.16881932
PAOX	1.02273707	1.51058119	1.69074295	1.39546964	1.13041373	1.46942407	1.15183602	1.55224703	1.73413466
PAPLN	0.60257542	1.30215603	1.58416829	1.70395737	1.51583103	2.00545237	0.98270796	0.45740014	0.3938513
PAPOLA	3.77298756	4.19762205	4.55297714	4.34639064	4.65349268	4.23784344	4.29701985	4.83635766	4.67510664
PAPOLB	0	0.01461993	0.08399686	0.03045805	0.03641173	0.0156245	0.01020137	0.00657655	0.00448781
PAPOLG	1.37284933	2.16268417	2.15659916	2.35568108	2.58747926	2.27728361	1.99066453	1.90332831	2.09081229
PAPPA	0.50275601	1.56095482	0.74548145	1.10685148	0.60700284	1.35021137	1.13803314	0.36576648	0.64358905
PAPSS1	3.31235249	3.72319031	5.09262685	4.68804662	4.16370511	4.23370069	4.47079843	4.04025925	3.97852921
PAPSS2	1.52029095	4.10754667	2.88508623	3.51950599	3.82385902	3.34855158	4.48658196	1.9267277	3.87243392
PAQR3	0.69706795	1.17853985	1.06679423	1.81744321	1.35309763	1.62311804	1.15004377	1.10593396	1.51149137
PAQR4	4.81412495	2.88037999	2.29442594	2.67471897	3.40074329	3.71101092	3.62423959	3.87520612	3.56493612
PAQR7	2.85689745	1.59399914	1.71883678	1.69130498	0.40159749	1.83033888	0.78248108	1.79074369	1.11901588
PAQR8	4.42298346	3.11624856	4.45852962	3.17552495	4.53185075	2.34226075	2.7369445	3.3309177	2.93268689
PARD3	3.06044629	3.040396	2.62724264	3.04497757	2.96264534	2.94261456	2.67922698	3.36894265	3.05183751
PARD3B	0.68335741	2.18361568	1.38515765	1.36874123	1.33853866	1.53817339	1.46896984	1.66878915	1.51777553
PARD6A	3.07654376	1.05617219	1.92931155	1.61233179	0.94137076	2.27201162	1.40399117	2.5302154	1.6474387
PARD6B	1.88727526	1.36215233	3.53803593	3.01565778	2.1721528	2.24530279	1.97369647	2.85193033	2.50254991
PARD6G	0.50450498	1.52983381	1.31150326	1.92822001	0.96387295	1.74183529	1.58209649	0.7365169	1.339665
PARG	1.84821486	2.29577321	2.35629541	2.07248851	2.69390588	2.49892173	2.13051855	2.5868151	2.70763349
PARK7	6.31792291	5.84314493	5.23267068	5.69041287	5.98682192	5.80897911	5.60340771	4.91846275	5.76984329
PARL	4.70255603	3.85396572	4.81790334	4.00263434	4.14960358	4.24739964	4.42705923	4.48585249	4.3376557
PARM1	0.62946019	2.98789003	2.74321775	2.92189356	2.62934226	2.30833894	1.12667617	1.32131884	1.11496081
PARN	3.1534635	4.06817431	3.72144852	3.81529368	4.05939069	3.62211456	3.99416941	3.60018405	3.49628654
PARP10	3.67472956	2.758791	2.98246572	2.34974665	3.11542758	3.603551	1.55829555	3.2792255	3.16789553
PARP11	0.90453179	1.70760046	1.56295528	1.77402049	2.28926458	1.79810161	0.9929388	0.94952495	1.67495504

PARP12	3.39201026	3.30886467	3.34382079	2.66698156	4.00603715	3.60339741	4.11376665	2.16130104	3.52703609
PARP14	3.50035685	3.78301286	3.27697031	3.53602222	5.46904679	4.90114773	2.8923503	3.25308932	4.29026657
PARP15	0.18601375	0.72389149	1.28195491	1.03847918	1.85696552	0.71854739	0.41980736	0.27547419	0.48320813
PARP16	2.68247004	2.08560376	2.36903649	1.84585527	2.19057703	2.0056247	1.89809936	1.35875255	2.61315154
PARP2	2.07069406	2.49257642	2.49756597	2.90569864	2.95659946	2.56880683	2.3488729	2.40988882	2.87979454
PARP3	3.67642008	3.49621919	3.01116453	3.44035231	3.70411461	3.19427839	2.23990017	3.66504861	4.77601304
PARP4	4.69743753	4.97295939	4.23637048	4.80047799	5.2476911	4.94432216	5.31169635	5.44831535	6.04308546
PARP6	1.61727144	1.72140928	2.78066646	2.5265257	1.64863555	2.10384535	1.0543354	2.02069667	2.78892381
PARS2	1.67128078	1.82629691	2.31203769	2.00332415	1.88702052	1.46665613	1.71406359	1.9495043	2.73132983
PARVB	1.46507555	1.89029812	2.34485025	2.47329878	1.74244361	2.66501859	2.5637923	0.86030281	3.00206066
PARVG	1.4797391	1.42763205	1.8512544	1.35420548	1.99438244	1.65777723	1.21130641	0.8441782	0.78195267
PASD1	0	0.02337657	0	0	0	0.00904185	0	0	0
PASK	1.20324187	0.49044201	1.30580209	0.92323535	1.61248966	0.88508482	0.92600472	1.0417594	1.1699652
PATL1	4.61134788	4.19624473	4.85154082	4.84994844	5.42887636	4.81254974	5.03355447	4.78747636	5.10971012
PAX4	0	0.26382889	0.01010708	0	0.00862275	0.00514901	0	0	0.8586037
PAX5	0.04232748	0.81913474	0.61162921	0.92696176	0.38420837	1.42689389	0.54291292	0.26213841	0.02442318
PAXBP1	0.85250148	1.70005469	1.91494701	2.79337444	3.08500546	2.05731865	1.72436063	1.92956338	2.93029812
PAXIP1	2.00172682	1.48886091	1.988971	1.83072497	2.22786735	1.95207947	1.73564922	1.9767878	2.20579034
PBDC1	1.9566683	4.16702599	4.11695634	4.56079855	5.14050773	4.24199334	3.82350647	4.17577217	3.88842148
PBK	3.11731218	2.94428098	2.38497758	4.66017934	5.18678324	4.21347853	4.98280211	2.68438839	3.99185695
PBLD	1.43543072	3.5392581	3.258184	1.18382566	2.94390724	0.96961837	1.38137082	3.70410155	3.09517086
PBRM1	2.80468007	3.36965225	3.01836923	3.29152957	3.40350898	3.44840938	3.4806092	3.46703049	3.63702022
PBX1	1.5967441	3.44073613	1.21846578	1.98029412	1.08890877	2.2718722	1.3779976	1.99365539	2.00207914
PBX2	4.6071377	3.48193069	3.71840449	3.69172126	3.54234822	3.41036084	3.47637461	3.95849746	3.67001651
PBX3	3.27069669	4.4017475	2.44701036	3.41292529	3.07711665	4.09506659	2.20037265	1.57632884	1.50353135
PBX4	0.9033559	0.30795808	1.53645603	1.46904605	1.1810444	0.68428941	0.58241171	0.78602653	0.89363507
PBXIP1	4.28335494	4.23977331	4.43462657	3.64881662	2.91814229	3.97312535	3.59750453	4.43568194	3.75995572
PC	0.67123712	1.12539587	1.41793393	1.31512013	0.8860348	1.15355786	2.35482386	0.94597548	1.89955279
PCBD1	6.61160986	5.60623432	6.41005063	6.15934788	6.28632399	5.98736126	5.50731089	6.00128814	6.51527632
PCBP1	7.35643571	5.97456404	6.35095875	6.18131245	6.44444723	6.74042984	6.58391672	6.50695992	6.67296266
PCBP2	5.21674329	4.16392954	4.5384632	4.83593448	4.74478171	4.65573782	4.55620116	5.13013959	5.03727555
PCBP3	0.18038991	1.48719722	0.36588782	0.76107848	0.15274202	1.28340499	0.21620207	0.1595358	1.04255222
PCBP4	4.19631673	2.46835077	2.58754644	2.73147054	2.31041048	3.23735127	2.65074564	3.33133867	3.09238435
PCCA	1.23822527	2.73510024	2.07343329	2.12263374	1.48962719	1.94664104	2.10541859	2.57045637	2.94698034
PCCB	3.4017305	2.54722006	2.711776	3.00551459	2.92312086	3.29258538	3.90800303	2.50312329	3.0225138
PCDH12	1.44879437	2.39224439	2.31980916	1.91756295	1.52733969	2.30886833	1.64653193	1.31391485	1.0047501
PCDH17	1.12850722	3.0505616	2.24341852	2.37886647	1.32890551	2.91614574	1.9447247	1.22369888	1.14539425
PCDH18	0.79326371	3.65833955	3.31082332	2.20225047	2.34952697	2.76701517	3.53753948	2.70177405	2.01456582
PCDH19	0.02957984	0.57334128	0.04254817	0.33277547	0.01146095	0.36650027	0.09308695	0.12144374	0.02337278
PCDH7	2.61615213	2.8376866	3.7646576	1.95116513	3.16303516	2.28746472	2.28180478	4.48134484	0.24665883
PCDH9	0.00939154	0.88287439	0.2256098	0.14954828	0.02517363	0.62929918	0.03138214	0.0300976	0.01723094
PCED1A	3.12537892	2.87976904	3.41615652	3.24110803	2.68508531	2.5383821	2.40479505	2.4241206	3.19301549
PCED1B	2.02448275	1.88643848	2.73716693	2.28649971	2.9821071	2.02009073	1.83139486	0.9594947	1.67863467
PCGF1	3.22498322	2.08842503	2.95892372	3.0913936	2.69499601	2.47545549	2.54531862	3.02966056	2.83627717
PCGF2	5.04360145	3.19472618	2.86997626	3.53197627	2.95017945	3.335053	2.86043696	3.638091	2.90348137
PCGF5	3.64414568	3.73024407	3.19476745	3.49363552	4.08816175	4.14663957	3.15373034	4.01834268	3.97750586
PCGF6	1.7726995	2.15019665	1.83605167	2.36377123	2.61538831	2.10907383	2.21595894	1.13003103	1.77352897
PCID2	2.56865744	2.61461738	3.04029391	2.75720457	2.83131764	2.71770514	2.61022869	2.63869128	3.53560822
PCIF1	4.89691043	3.86367817	4.80671516	4.14914558	3.69713638	4.04702374	3.71266245	4.10940685	4.38309498
PCK2	4.33722164	3.66002171	3.18851486	2.79660524	3.45320152	3.46402538	4.00316848	1.97656666	3.6295419
PCM1	2.3888491	3.37422091	2.41409972	3.42321168	3.56050406	3.47361817	3.82033323	3.06043023	3.307314
PCMT1	4.27482562	4.16902985	4.2802944	4.66938989	4.44511252	4.63021726	4.79004648	3.47616128	4.27578674
PCMTD1	1.40164643	3.49454273	3.37306845	4.0361815	2.87396228	3.40649167	3.08676016	3.13628205	3.10118576
PCMTD2	2.06217166	2.77228042	3.9386545	3.43372429	3.06922141	2.92502582	2.25619605	3.17680423	3.79539014
PCNP	5.24563913	5.09429216	5.19009982	5.54391048	5.36843698	5.2752066	5.25991469	4.8612908	5.03056504
PCNT	1.39106923	1.38723177	1.55625344	1.85564674	2.46201781	1.81795422	1.49811484	1.69615724	1.82865932
PCOLCE2	0.05143556	0.90549181	0.14390109	1.1501966	0.50625805	1.15848549	0.80161787	0.04764125	0.14644473
PCP4	0.06444313	0.39395866	0.28038537	1.11422405	0.040039	1.79729839	0.01959166	0.02517973	0.06767107
PCSK1N	0.37466961	4.40183133	0.64374734	0.24978748	0.15597835	1.7291504	0.09297467	0.16534825	1.92447475

PCSK4	1.10154092	0.36738037	0.21266491	0.41330754	0.34580861	0.29011996	0.35067785	0.93664243	0.99601753
PCSK6	0.37112606	0.58363542	2.23648156	1.29313291	2.08206444	0.72391541	1.36184904	1.73331297	1.36642128
PCSK7	1.4522624	2.11832653	1.38428343	1.32765931	1.68218352	1.94091638	1.61408411	1.3384514	1.50724402
PCTP	3.75876631	2.62820962	2.77822309	2.52042526	2.87448987	2.43699756	1.19111848	2.4244168	2.18298877
PDCD10	4.16935161	3.81990433	5.04018179	4.49188986	4.85787711	4.22248618	4.4198127	4.10130213	4.37725722
PDCD2	2.77471986	2.63991141	3.5499111	3.07642186	3.0283501	2.97917212	2.95952245	2.95736629	3.49290877
PDCD2L	2.62582835	2.14661523	3.24119138	3.4452129	3.70473612	3.32513926	2.93685519	3.27628378	3.3126427
PDCD4	2.74059754	3.90709176	3.70768051	3.73870158	3.5816744	3.36943908	3.90795223	2.05750594	3.53794289
PDCD5	4.49220835	3.92296196	3.98331166	4.51831745	5.11259466	4.40592359	3.9076259	4.14554113	4.28498938
PDCD6	3.66109398	3.43554101	3.62808685	3.48340329	3.551669	3.09248008	3.34135001	3.57493436	4.15351344
PDCD7	2.90452019	2.42080945	2.54412231	2.82280582	2.90608706	2.59671012	2.19608334	1.89348733	2.63243377
PDCL	3.02720191	2.81970023	3.68087896	3.08720709	3.27259998	2.99126554	2.80273295	2.44352325	3.18606895
PDCL3	3.28905081	3.25083082	3.34768564	3.50525884	3.93907689	3.69184574	3.77021413	3.24348646	3.84453867
PDE10A	0.20593811	1.00560335	0.70193235	3.04550916	0.42118134	1.49982442	0.47449783	0.43137262	0.29628181
PDE12	2.80208873	2.33156763	2.74226775	2.79720036	2.83733808	2.78046081	2.85566317	2.83785697	3.10661297
PDE1A	0.25314275	2.9832479	1.94892378	2.33259398	0.96178694	2.50228576	0.81020423	0.79076929	0.28254354
PDE1B	0.39872411	1.45711091	0.82880634	1.12548941	0.62419563	0.84936174	0.49051087	0.22648865	0.21240359
PDE2A	0.36954061	2.04293772	1.10340099	1.22651931	0.55175983	2.05786527	0.8017771	1.38733494	0.23344822
PDE3A	0.45416736	3.29117695	1.4625527	2.50119798	0.68177297	2.75334363	0.98386018	0.74594352	0.5815741
PDE3B	0.21640805	1.01590757	0.72117453	0.97794475	0.99494162	1.26693099	1.61608118	0.27549903	1.1764096
PDE4B	1.15727311	3.29403051	2.68822742	2.83879872	3.21214206	3.59471345	2.39931717	1.97689692	1.62146709
PDE4C	1.11737868	0.73389092	0.64787301	0.30804908	0.5917467	0.67656531	1.27691875	1.04991458	0.12879362
PDE4D	0.29590437	2.17946634	1.5391696	1.74104373	1.81298905	2.81745662	1.92825442	1.91805088	0.73390572
PDE4DIP	1.02778545	1.85294335	1.07903758	1.27922817	1.33310461	1.70056823	1.57136774	1.08585116	1.20724206
PDE5A	1.43183575	3.52575567	1.79809444	3.12737435	1.35536567	2.85549396	2.27397301	2.14223458	2.77424543
PDE6D	3.54935189	2.9734566	3.1757936	3.12017776	3.57410176	2.49758136	2.40683188	2.73420602	3.06052768
PDE7A	2.29047647	1.80104622	1.82938702	2.37021249	2.7475193	2.03612014	2.44571404	1.65258665	1.85024976
PDE7B	0.17307077	2.76741837	0.82658975	1.07641053	0.29596147	1.79602889	0.26718256	0.15018295	0.14107484
PDE8A	3.46290656	2.55104975	3.37507624	2.58836674	2.65638824	2.45407525	2.66718542	1.66470892	2.82001631
PDE8B	0.2005731	0.8211693	1.09628659	0.94025769	0.50496492	1.00275845	0.56470962	0.67437383	1.19943012
PDE9A	0.13847357	1.31095753	2.13519635	1.0359725	0.40929001	1.37874459	0.377008	0.63145524	1.84687428
PDGFA	2.12208238	2.32545785	3.28791395	2.97997333	1.88433198	2.62728665	2.21955728	2.97293993	3.15710774
PDGFB	3.27871587	2.70081979	3.87620648	2.66343517	2.53910338	3.12098446	2.31744992	2.40949774	1.97984279
PDGFC	1.1648401	2.13818949	2.21362117	2.68916693	1.42256006	2.88152009	2.73410146	2.22061805	0.84425142
PDGFD	0.58817088	3.34408504	3.45693119	3.70764026	1.79632182	2.2080304	1.95147069	2.35859703	0.81003795
PDGFRA	1.11296073	5.11168792	3.9030287	4.10793296	1.69898178	4.3905979	3.18242711	2.79569786	1.67597205
PDGFRB	3.63812292	5.81985127	4.90903007	5.49179644	4.12819872	5.16137604	5.19017345	3.78306695	3.65836703
PDGFRL	0.67601862	3.24873815	2.09263564	3.91697865	0.54464875	3.28312361	2.89172886	1.1717935	0.22758481
PDHA1	3.89220881	4.08387529	3.74343002	3.86531215	5.30563354	4.08340603	4.19292692	4.10819518	4.70532421
PDHB	3.06784255	3.53930535	3.60848201	3.78129115	2.91437503	3.70818591	3.85922394	3.28597282	3.77684597
PDHX	3.04309768	2.82697054	3.23966906	3.65727952	3.68459295	3.38366954	3.99356305	3.1060841	3.38596487
PDIA3	6.85286429	6.69696561	7.91255554	7.15366443	7.38575738	7.44533069	7.94013952	6.6186725	7.56054698
PDIA4	6.91512359	5.71870149	7.60399965	6.15356265	6.14364579	6.16860764	8.08394537	6.62009762	6.52693305
PDIA5	3.56841738	3.23074236	3.43599366	3.35299209	2.75981991	3.4506866	4.53690361	2.83913286	3.45987165
PDIA6	5.52832185	5.57104998	6.03983522	5.90354028	5.89426649	6.07288832	7.15136232	6.03573915	6.23704519
PDIK1L	1.61402408	1.75441352	1.86394011	2.59846702	2.60946947	1.79763249	2.16065624	1.35539855	2.66263747
PDILT	0	0	0.92455464	0.04725229	0.01959538	0	0	0.01839707	0
PDK1	1.08539144	0.68756998	1.678043	2.23691387	1.24539763	1.0678937	2.30309303	1.04707504	2.74382356
PDK2	3.17747907	3.12116564	2.67678134	2.27690036	2.17475113	2.53248005	1.98864189	2.76965312	3.29477812
PDK3	2.1166923	3.18534245	2.2109535	3.35134888	3.56755595	3.06883306	2.61156868	1.89124995	2.28763317
PDK4	0.26452933	5.69603653	3.33210605	3.515648	3.31673754	3.05769019	2.66615371	3.85307724	0.88918351
PDLIM1	7.00656224	6.99193877	6.39462651	6.96784085	7.20793763	7.03166632	6.74193965	5.5905407	7.15904417
PDLIM2	1.23513569	1.4519272	1.09482847	1.36295466	1.20334029	1.60274923	0.94963263	1.22750669	0.55531082
PDLIM3	1.92017567	5.02959854	2.42974997	4.61163648	1.49375025	3.84634569	2.51989801	1.51403246	1.72362633
PDLIM5	3.54637239	3.85464616	3.39012816	4.15524205	4.0389123	4.40728065	4.19780458	3.89651831	3.83298722
PDLIM7	4.34668121	5.02152946	3.63670889	4.9525425	3.66986061	5.30255382	3.96222787	4.03707211	2.74819576
PDP1	3.25047017	2.58676869	4.30370808	3.05182162	4.07952531	2.83442834	3.35855841	3.07113924	3.02343313
PDPK1	1.98530186	2.7507091	1.65601912	2.03278488	2.18162127	2.151553	1.66076486	2.3565458	2.92722065
PDPN	3.2351191	5.10978559	3.95411172	4.99254871	2.47158553	4.78034153	4.60993396	3.25696618	2.34683486

PDPR	2.02410973	3.07541558	2.62820991	2.5544441	2.3351551	3.19155095	3.15159109	2.38453335	2.72114847
PDRG1	3.37065953	1.92428389	3.47283942	3.35389064	3.20068824	2.62211434	2.4543175	2.75075999	3.52028786
PDS5A	3.084555	3.56739796	3.74902454	4.10040118	4.5557981	4.05888207	3.78449785	3.94414059	4.16508947
PDS5B	2.05413139	2.64925372	2.18458898	2.16325809	2.61526701	2.21776213	2.33971137	2.04631463	2.71498707
PDSS2	2.24048445	2.50515379	2.38432151	2.43936082	2.62223892	2.69813001	3.26200704	1.86440212	3.29434415
PDX1	5.57179788	1.50286434	2.12567495	0.94648465	1.88043885	0.41977606	2.45266598	2.19338237	2.94888922
PDXDC1	4.24466114	4.31870734	4.24899171	3.59736427	3.94637787	3.97602901	4.80639793	4.41917763	4.55995424
PDXK	3.31306163	2.5843391	3.13373644	3.25742334	3.98346286	3.98873437	3.8590621	3.36020142	3.23649641
PDXP	2.58762026	1.19783288	1.32640476	0.79014235	0.68483365	1.2648386	0.67553118	0.50125264	1.93843906
PDZD2	0.17530033	1.00864655	1.07217373	0.56144037	0.28908369	0.88546033	0.74231161	0.66797213	0.25320446
PDZD3	0.0832587	0.37286079	0.35230326	0.60668317	0.65012431	0.0069811	0.02822506	0.25502154	0.95368688
PDZD4	0.12073677	1.10209227	0.27023963	0.3315439	0.12383042	1.11313787	0.09716542	0.08176916	0.05200722
PDZD7	0.11430755	0.2586743	0.30834171	0.35161491	0.0933397	0.09351968	0.05514991	0.47131978	0.14395799
PDZD8	3.12448021	3.32112567	4.12498349	3.35979561	3.40270037	4.67376244	3.65592099	4.58510127	3.87382796
PDZK1IP1	9.20722017	5.90825736	8.77072633	2.0933132	7.01116117	3.90565334	6.23809667	7.56743207	7.38050734
PDZRN4	0.08487097	1.85174942	0.27135562	0.8047886	0.15753137	1.3801478	0.22424619	0.14277953	0.06133608
PEA15	6.56886386	6.24430146	6.567056	5.94667635	5.69478535	6.30917605	5.98776817	5.84320956	4.81134452
PEAR1	0.59610489	2.01920067	1.5033308	1.54927101	0.85187918	1.58851914	1.00256825	1.2415353	0.9782504
PEBP1	6.83661305	6.88506146	6.48604879	6.17509222	6.29889454	6.57540304	7.1473099	6.21149548	7.38894602
PEBP4	0.08000841	0.3097951	0.2536947	0.55342337	0	0.14373291	0.01632279	0.04166963	0
PECAM1	3.41632989	5.92888286	5.47119598	5.08827513	4.94715172	5.66332208	4.38789284	3.79334522	3.76525624
PECR	1.84958858	1.69480461	2.09786558	1.87471376	1.91010861	1.81208443	1.42347805	2.59516823	2.38028286
PEF1	5.16331925	4.84160934	5.15844734	4.67126448	4.59277624	5.03160507	4.36338057	4.22782335	4.92095007
PEG10	0.08977245	1.44348625	0.30728352	4.05370288	0.17668913	0.74482121	4.0366075	0.28663571	0.20272422
PELI1	3.02947819	3.89705732	5.24005812	4.4199629	4.57575797	3.84002902	4.07792551	4.28507382	4.43544202
PELI2	1.28957583	2.54942958	2.45565524	2.11325792	2.3005696	2.96172265	0.80860272	0.89346457	1.13284497
PELI3	1.94984806	1.64630539	1.3934332	1.33399382	0.81645303	1.63418016	1.08201858	1.40959254	1.24063473
PELO	2.70704185	2.58833792	2.7402972	2.39658185	2.31900468	3.1701169	2.78744912	2.82930946	2.20060021
PELP1	3.10517841	2.6232522	2.95806332	3.05436096	3.0505791	3.25688611	2.87420427	2.50160578	3.52285866
PENK	0.02094417	1.16059096	0.21878603	0.24929537	0	1.17457565	0.01881984	0	0.075609
PEPD	3.5114267	4.28802778	4.21286455	3.57259006	3.93199235	4.33131759	3.88978166	3.62918654	3.95020499
PER2	2.45939769	1.76497151	2.87527538	2.53714818	2.21094167	2.11739632	1.98779824	3.05651774	2.7713203
PERP	4.22775668	5.05038501	5.22739412	6.79715179	5.0943767	5.43145802	6.85006602	5.86535122	6.34873724
PES1	4.7931263	3.09714529	3.52067318	3.46172115	3.54750845	3.74592317	3.37125919	3.17801541	4.27415953
PEX1	1.7074358	1.7515049	2.57298419	2.24436696	2.20554994	1.92384673	2.20670947	1.86774717	2.23624353
PEX10	2.62924479	1.50237222	1.71083514	1.82211242	1.24349203	2.12706238	1.67041659	1.52784445	2.26584994
PEX11A	1.71299832	2.12081127	2.07040613	1.44647494	1.16455974	1.3211537	1.64692144	2.25042205	1.82106744
PEX11B	4.45136565	3.64316766	4.18650284	3.88223904	3.89340605	3.67825608	4.99072171	3.83968951	4.44092961
PEX11G	2.12439042	1.35029368	1.44913841	1.01684532	0.77504055	1.04077474	0.9473681	0.83486604	1.55054469
PEX14	3.83553109	2.44488983	2.26743323	2.41185634	2.42629562	2.83952882	2.57852453	1.9978544	3.03095275
PEX16	3.80603443	2.18403723	2.77127724	2.34181807	2.39538411	2.62867659	2.70170116	3.020134	3.33762714
PEX19	3.61760673	3.64408193	3.88067704	3.45299845	3.40129443	3.80110623	4.30543143	4.43288838	3.67663245
PEX2	2.65242204	3.2408684	3.7754237	3.85004039	3.3813799	3.60173428	4.16352812	3.50830949	3.21558152
PEX26	2.75526867	1.62920444	1.85068496	1.54171649	1.86349368	1.61901939	1.68781702	2.23214335	2.01999015
PEX3	2.55793257	2.92042863	3.85256597	3.10521552	3.7927378	2.99565055	3.12390356	1.77716495	3.42180893
PEX5	2.50506087	2.36163566	2.42575552	2.54761987	2.57109988	2.52418643	2.12121648	2.17910472	2.87657867
PEX6	1.78554117	2.82272093	2.84101964	2.55301752	3.05518366	3.20320855	1.30415832	3.74199044	3.50880372
PEX7	2.26704133	1.93172949	2.90585946	2.72062488	2.52124717	2.92713067	2.91989203	1.78937143	2.977175
PFAS	1.68588583	1.51624023	2.11934399	2.1927453	2.39548501	2.57531469	1.95829371	1.41690656	2.63650601
PFDN1	4.86513145	4.47258359	4.62755188	4.70497003	4.5817414	4.72387205	4.2482691	4.47205313	4.53392035
PFDN4	3.87386402	3.22644166	3.87633706	3.97439193	3.63156748	3.00888401	3.0880539	2.76708547	4.00843198
PFDN5	6.00649635	5.6062084	5.85564979	5.62749448	5.54582792	5.10688898	5.04349797	5.82246211	5.40649497
PFDN6	3.28813131	2.04311375	3.2382072	2.66512127	2.64117184	2.20129195	2.50989912	3.11313292	3.10873455
PFKFB1	0.13513966	0.08852074	0.05028934	0.13190165	0.19367765	0.14238949	0.06224183	0.04038176	0.15851603
PFKFB2	3.23049372	2.05171949	2.94873478	2.25062231	2.4016163	2.94848892	3.77877005	2.66304008	2.6532492
PFKFB3	5.41622808	3.38851359	3.94965967	4.12034752	4.44781561	4.53537554	4.11453024	3.84952537	3.82150469
PFKL	3.92265369	3.87693568	3.61081897	3.67355772	3.72750812	4.35112378	4.81472323	4.46092261	5.29935884
PFKM	2.05879638	2.59971769	1.91117317	3.07699501	2.61783887	2.97042928	2.28445469	3.16370158	3.00724977
PFKP	4.82533766	3.53208817	3.21000627	3.63752738	4.54489772	4.99117034	4.79981484	4.31286742	5.12761549

PFN1	8.93502545	8.16240018	8.69154892	8.37889891	8.84855171	8.83372047	8.45633259	7.27866041	8.67675152
PGA3	0	0.02056677	3.25514265	0.25398231	0.37460427	0.02110419	0.0552129	1.21152939	0
PGAM1	4.95205529	4.76351115	3.80721118	4.49187796	4.20910119	5.3538715	4.89328913	4.4765301	5.18377556
PGAM5	4.38930865	2.35071738	3.43475001	3.22537163	3.73269402	3.2128534	3.47220066	3.55173249	3.73197775
PGAP1	0.33038456	1.64488278	0.72185882	1.26105247	1.02183201	0.99692665	3.17825112	0.69762041	3.04322036
PGAP2	2.68072822	2.14323387	2.37298695	2.80748594	2.49745402	2.70398964	2.79724966	3.23816858	2.97623146
PGAP3	4.0400457	2.4142217	3.60138943	2.52159384	1.72385199	2.36353412	3.15395644	4.04950479	3.57429577
PGBD2	1.08467533	1.06889436	1.84172457	1.49477992	1.80821034	1.09001061	1.58520443	2.21996012	1.68150672
PGBD5	0.29567015	0.41119457	0.51566029	0.3056404	0.62980153	0.58131225	0.53847334	0.18751946	0.20094183
PGC	0.21682611	0.96374637	10.180695	5.69270039	5.42235957	2.11664399	3.70296519	13.8488246	2.12561355
PGD	6.60213553	5.22887977	4.57499972	5.51609645	5.686247	6.06875081	5.77578755	4.45398564	5.78371754
PGF	1.18878761	1.37818182	1.76161136	1.62860398	0.72698197	1.48365553	1.04278516	0.79347885	0.83148494
PGGT1B	1.65932751	1.93555463	1.84528713	2.14011518	2.04119566	2.08278677	1.99751272	1.34492672	2.53802018
PGK1	7.0581649	6.03668363	6.33731717	6.49501368	7.31003679	6.48593434	6.93343866	6.60243609	6.88991459
PGM1	1.6941575	4.34082593	4.71680138	4.3953099	1.88960265	4.40920995	4.62835055	4.86128318	4.03834657
PGM2	2.88832287	3.47482329	3.81930182	4.29677874	4.25803844	4.23854637	4.34125283	3.88933726	3.98792231
PGM3	2.15743753	2.79859167	2.66778223	3.57232158	3.32865308	3.42523597	4.22344244	2.66992548	3.31497181
PGM5	0.25568474	4.08973336	1.0352185	1.74377582	0.41263315	3.38963065	0.30592936	0.4033062	0.5752561
PGP	4.13067755	2.99595404	2.49002411	2.82037858	2.91017847	3.52401822	3.5452949	2.74148473	2.94852468
PGPEP1	3.36200237	2.18712607	2.89174984	1.99232689	1.19227794	2.05263482	2.4939206	2.11222368	2.92305617
PGRMC1	6.61982097	6.13288457	6.70608299	6.22125207	6.82247581	6.21887411	6.32145659	6.32808294	6.2699632
PGRMC2	5.13261536	4.22697726	4.83060331	4.2062615	4.05684825	4.02617902	4.16505249	4.23359101	4.2492587
PGS1	2.61077378	1.9644803	2.67637682	2.11955392	2.23799285	2.23941004	1.80253359	2.3214897	2.51542942
PHACTR1	0.45407458	0.93712115	1.10134986	0.93920709	0.54038842	0.84311509	0.32011473	0.51105997	0.48617491
PHACTR2	2.15900251	2.77889072	2.97755657	2.30811807	3.09304803	2.49189234	2.67530853	2.2678222	1.92968962
PHACTR3	0.00596713	0.35093941	0.1280627	0.55822523	0.03637381	0.07073805	0.0106983	0.11512202	0.10893146
PHACTR4	4.24139328	2.90588785	3.56508038	3.27422065	3.85137568	3.13958683	2.77928319	2.72126453	3.33275659
PHB	5.54327991	4.68402671	4.51504545	4.99066427	5.26476388	5.00939881	5.11665972	5.10635497	5.83602443
PHB2	6.99539021	5.70495053	6.12416487	6.19618683	6.38683537	6.51651405	5.93979998	6.25471662	7.21693139
PHC1	1.46185959	1.44624661	0.9366137	1.37170519	0.91588016	1.53328843	0.74021548	1.22240585	0.94283327
PHC2	4.97946032	4.45021544	5.18836533	4.6649534	4.31628323	4.8002259	4.29762749	4.49048829	4.68515711
PHC3	1.98579662	2.35220227	3.03530891	2.0696951	2.42715281	2.30115172	2.25316353	3.10079842	2.18504857
PHF1	3.53175896	2.77964292	3.43841677	2.96626504	2.53158848	3.1170749	2.82902075	3.70791952	2.51765257
PHF10	2.59553419	3.52562097	3.71802151	4.20947158	3.39878457	3.51589047	3.87944068	4.08310402	3.61284508
PHF11	2.31508331	3.50436844	3.32504728	3.12853291	4.0827761	3.25973573	2.36321186	2.42286813	3.52458339
PHF12	2.92972627	2.36333403	2.6479048	2.48764779	2.54606544	2.46738736	2.10173224	2.69655717	2.38889615
PHF13	2.69214507	2.34827108	1.99807687	2.53420588	1.86318936	3.12322621	3.00520456	1.97478534	1.95017058
PHF19	2.9358682	2.44076479	2.14254009	2.36227945	2.25812055	2.7736018	2.42386827	1.51437485	2.66237111
PHF2	3.1636457	3.18815819	3.51904479	2.31264072	3.02895559	3.30257165	3.1368125	2.82779325	3.19861013
PHF20	2.45112096	2.81676434	3.06686287	3.12863572	2.5319197	2.99721043	2.90348645	2.68513665	3.11871437
PHF20L1	1.10477971	1.7759613	1.90017438	2.28768843	1.90191115	1.8443121	2.01790221	2.12495329	1.91893665
PHF21A	2.1926383	2.35755692	1.97065115	2.2335731	2.19483059	2.34510543	1.68477084	1.71698268	1.54361297
PHF23	3.51356327	3.33538673	3.83523608	3.66588477	3.61482106	3.88076624	3.32947403	2.852071	3.77169877
PHF3	2.4949472	3.31205364	2.51158632	3.36739915	3.57273437	3.19269955	3.111936	4.10625656	3.3710905
PHF5A	5.85969612	4.11640887	4.84488852	4.50442258	4.71230584	4.19324573	4.02728512	3.54431799	4.77757627
PHF7	0.72816224	0.7240691	0.97412047	1.11380468	0.79285773	1.15719767	0.84377156	0.63063907	1.17973353
PHF8	2.46525083	2.27373677	2.47051531	2.49343229	3.05962449	2.51526993	2.67291573	2.75521708	2.78164389
PHGDH	0.45727873	2.0885758	1.08845535	3.23268884	0.68863008	3.65607188	4.52184395	0.48369638	0.46987214
PHIP	2.008388	2.73206138	2.15733501	2.85116859	3.18484064	2.9258456	2.72332409	2.86341469	2.84445348
PHKA1	0.13155969	1.10331381	1.78727416	2.11261811	3.16192958	2.0039911	1.36540085	2.25502274	1.96205905
PHKA2	1.64706405	1.72933922	2.02374008	1.80086923	2.16403209	1.83882001	1.58804491	1.68864844	2.0805033
PHKB	2.23624497	3.61523901	3.01982262	2.90635637	2.95427587	3.15404573	4.12353049	3.14466337	3.48872513
PHKG2	2.1100605	1.63873452	2.52275136	1.98657916	1.95093546	1.74619829	1.72413349	2.08371491	1.84849166
PHLDA1	3.25218281	3.27545548	3.7197733	5.14402766	3.82445485	3.82895017	3.42334444	4.67014793	3.57594929
PHLDA2	5.87183614	3.07715627	3.74856978	5.97155348	4.34414017	6.09862657	6.51459634	6.11324111	5.6755532
PHLDA3	2.26778275	3.84985312	2.95030478	3.5511662	0.70032703	3.95523908	3.73626214	4.1102564	1.8472558
PHLDB1	1.27082261	2.12080013	1.44086226	1.6964758	0.89674967	1.90165104	1.41650074	1.09356849	1.56609756
PHLPP1	2.58665117	1.57151151	1.70746212	2.08949309	1.90264518	1.72451741	2.79388004	1.93443742	2.72579715
PHLPP2	0.29213362	1.12372449	1.16179496	1.16026789	1.23972266	1.01015885	1.05805199	0.68656648	1.49877141

PHRF1	4.04143419	2.41119442	2.11161532	2.45398028	2.95457461	3.0465895	3.20314783	3.01343706	3.52504871
PHTF1	1.74112745	1.59364807	2.24929054	1.57663835	1.56239078	1.60107638	1.18246473	1.52847584	0.91474304
PHTF2	1.52102066	2.06805725	2.87100433	2.64750564	2.74192486	2.26441217	2.16253333	1.92367609	1.57365105
PHYH	1.62508177	4.1543435	2.91811337	4.03149682	3.29423841	3.64985245	3.2651671	2.95260375	2.57157526
PHYHIPL	0.00887685	1.20667604	0.45896464	0.12283076	0.01636301	0.21255637	0.05747122	0.05719288	0.16771694
PHYKPL	2.35419054	1.87622249	2.29952405	2.07658006	2.1389364	2.01874082	1.00438005	2.00506925	2.54576501
PI15	0.10317272	4.28808235	0.716069	2.50497414	0.50118131	2.32196261	1.18806246	0.12765969	0.24330591
PI16	0.17631219	2.82770581	2.07338548	0.95298297	0.0658668	2.11748002	0.43313264	0.89859451	0.14820101
PI3	4.33457851	3.77498365	4.2762852	8.34219715	5.21285372	5.71843693	11.4742937	8.20909236	5.69395162
PI4K2A	3.13510184	3.20602485	2.7466924	2.99754709	3.0375401	3.07276822	2.88969914	2.75145849	2.64807184
PI4K2B	2.44324638	2.98031621	3.17345243	3.27283174	3.40973497	2.79030465	3.10994999	3.18104451	3.39300489
PI4KA	3.5883983	3.32285373	3.13903718	2.75582432	3.53966596	3.2631357	3.01654741	2.9346465	3.52076878
PI4KB	3.84003449	3.61317471	3.91418235	3.32075608	3.59470428	3.70348414	4.11591094	3.63283828	3.68450384
PIANP	0.05474263	0.11069507	0.07844685	0.43086165	0.03396876	0.36086159	0.07800977	0.13928403	0.04617729
PIAS1	2.59514525	3.08303646	2.88493142	3.16403395	3.48097246	3.15089484	3.00497931	1.98843922	2.90380737
PIAS2	0.76593644	1.2018119	1.60203695	1.59907656	0.72008836	1.28775764	1.68547501	0.77253712	1.32850159
PIAS3	2.87379125	2.68742846	3.60398679	3.34882366	2.60753	2.86315221	2.92709296	3.33369204	2.39465558
PIAS4	3.86455505	2.0631338	2.56817217	2.4346342	2.66440897	2.74468519	2.41861871	2.93533872	2.73542874
PIBF1	2.08713655	2.51145498	2.3548183	2.11857233	2.45674379	2.37639673	1.98744483	2.14261845	2.87353677
PICALM	4.54971225	5.36421261	5.10632781	5.60745612	5.55110229	5.38426045	5.48181961	5.0160961	5.29131011
PICK1	3.58543882	1.83011407	2.81165913	2.03800476	1.91998767	2.30423505	2.26573554	1.84152687	2.81542614
PID1	0.26634233	3.73683172	2.19556309	2.1126768	0.31099032	2.4383286	0.87664867	0.97364536	0.87099148
PIDD1	1.97339057	1.39759037	1.26637861	1.71833308	1.34365535	1.22132626	1.69326404	1.45604651	1.87121865
PIEZO1	3.05802952	2.70458125	3.39131213	3.41702278	3.65170096	3.74528176	4.13589301	3.59615418	3.77519108
PIEZO2	0.19550313	1.15532127	0.96786993	1.11277285	0.52360761	0.72127827	0.69176314	0.44843004	0.18903432
PIF1	0.7700523	0.34525792	1.3339312	1.16990725	1.28920069	0.9131249	0.54393291	0.34929728	1.06900495
PIGA	1.68646096	1.80861847	2.02308118	2.68381819	3.09987394	2.12099763	2.45545841	2.00165927	2.38159357
PIGB	1.75958827	1.76777197	2.0917925	2.23794195	2.19226315	1.97618561	1.91329078	1.60442066	1.97857368
PIGC	2.11957776	2.35324576	2.71155876	2.55198249	2.44066806	2.37193085	2.80307555	3.0539118	2.53303592
PIGF	1.73959942	2.12536348	2.04648597	2.12774848	2.72975844	2.23472373	2.36614409	2.14176309	2.26816537
PIGH	2.79776432	2.6059778	3.04223214	2.53319977	2.76733855	2.84917133	2.50568855	2.66466667	2.71011022
PIGK	2.46296234	3.28511741	3.68088807	3.17431672	4.3513929	3.27986806	3.21085411	2.93332234	2.6362147
PIGL	0.91809062	0.90577243	2.08615113	1.51449131	1.83836307	1.12095077	0.82020882	0.523741	2.12058946
PIGM	1.9980782	2.20841116	2.50318529	2.4265009	2.17987962	2.12895402	2.09073347	3.38357245	2.49237356
PIGN	1.93967126	2.40566811	2.47647117	2.65251427	2.5934829	2.49416886	3.80496363	2.25031643	2.7013386
PIGO	2.64772921	1.93588759	3.29427216	2.26902967	2.35216883	2.42936957	2.30676965	3.61809039	3.04243402
PIGP	0.96358089	1.62386611	1.3584397	1.58723065	1.42612696	1.41460181	1.35898677	1.78623924	1.86045872
PIGQ	3.64130563	2.61526129	1.72866488	2.28524756	2.03458799	1.94477766	2.46801034	2.46509203	2.41574643
PIGR	9.83638017	6.84137711	8.8003233	2.80850155	4.88282137	4.87424569	2.13874591	7.58356203	9.51592744
PIGS	4.52525716	2.90141556	3.69959675	3.35511633	3.15853843	3.47689696	4.03824351	3.96013342	3.20022089
PIGT	6.46506651	6.56260686	7.43348268	6.51882284	5.38597713	5.92871526	6.73922737	6.03724768	6.7001548
PIGU	4.16667056	3.71562862	4.43646645	3.90111591	3.90332128	3.64722783	4.03377275	3.54634024	5.06489099
PIGV	2.81155055	2.56774807	2.73336196	2.25363006	1.59237928	2.56979869	2.25377018	1.78536112	2.73417125
PIGW	2.00843831	1.90897841	2.52604431	2.60512923	2.65631273	2.17580221	2.23620818	1.20659475	2.22253376
PIGX	3.08071451	2.39780753	3.3097534	2.41762917	1.9872301	2.21763863	2.25560709	2.69239196	2.21340933
PIGZ	3.13820941	1.14548709	1.8606026	1.72187096	0.40568719	1.01669164	1.10372485	0.73994702	2.16810986
PIH1D1	3.78198854	2.93312757	3.36811233	3.06146243	3.54193704	3.39142432	3.66094319	4.08276476	3.86803406
PIK3C2A	3.1801113	3.54862061	3.95410196	3.65788793	4.47914337	3.61746419	3.67507751	3.84965894	3.47364585
PIK3C2B	3.5958113	2.27579722	3.04280754	2.60620529	3.55906342	2.10994043	2.88524253	3.38055212	3.50411924
PIK3C2G	2.25497794	0.01053141	3.42614066	2.51393605	3.84252696	2.05811012	3.75468963	3.51224079	0.01287525
PIK3C3	0.64683988	1.82180522	1.88422845	2.11012614	2.03697892	1.84706935	2.06499541	1.47489569	2.05567549
PIK3CA	1.63434815	2.66376318	2.40265823	2.34553334	2.49656308	2.66574538	2.13382345	2.81214617	1.94279057
PIK3CB	2.90264136	2.4602647	3.22979466	3.13083896	3.8967626	2.88411331	3.62012801	3.67669944	3.13744419
PIK3CD	2.01578787	2.20281295	2.29100502	2.00485034	2.94343105	3.1849778	1.72758802	1.20023601	1.79332443
PIK3CG	0.72029425	2.20824642	2.34161354	1.27104271	2.65681859	2.61827836	1.6645262	1.15476921	1.22315531
PIK3IP1	3.5264384	3.83087957	4.00203654	3.06640055	3.32751804	3.48654495	2.99543275	2.81166425	3.31436578
PIK3R1	2.79896132	4.21617688	2.78137119	3.68915003	3.97555926	3.44474868	2.80761365	2.97417139	3.98098261
PIK3R2	0.58204553	0.48523003	0.27954456	0.41685203	0.34682159	0.35041017	0.35324857	0.36556867	0.45399219
PIK3R4	2.90211142	2.98452508	2.71907506	2.62996534	3.2876764	3.13132598	3.07627347	3.23651515	3.09895074

PIK3R5	1.23858943	1.46167336	2.11635856	1.60089173	2.07222507	1.82925162	1.41799732	1.13996087	0.91960846
PIK3R6	0.69301471	0.99617421	1.28928803	0.92716089	1.67643213	1.04260979	0.81515331	0.48986775	0.46858343
PIKFYVE	1.43924523	2.17843887	2.31946903	2.49167069	2.78369816	2.58390743	2.13637028	2.17140722	1.95177757
PILRA	3.56438435	3.91666871	3.18975129	3.82002993	3.97066194	3.9282139	4.12690431	2.72588136	2.19151588
PILRB	0.52872123	0.33466046	0.85896998	0.81979737	1.24583471	0.59345194	0.37457185	0.69442186	0.60685797
PIM1	3.47111136	3.42825849	3.85609661	3.89363399	3.59669863	4.43873806	3.73364351	3.73446871	3.9650092
PIM2	3.46307748	2.75918498	4.98389107	4.53049764	4.72173881	4.04594398	2.88908664	3.4106266	4.55209837
PIM3	6.98717562	4.05300593	5.87865053	4.32317221	4.29539773	5.7408477	4.97341287	5.42479435	4.78123625
PIN1	4.22153433	2.81258579	3.09623667	2.4358425	2.8892425	2.95364254	2.67437726	2.99668475	2.95088419
PIN4	2.05059132	2.3540596	1.8635758	2.17805236	2.94278908	1.41098544	1.72729437	1.37967376	3.00567776
PINK1	1.8775577	2.84912966	2.06007718	2.19897749	1.64334327	2.85821122	2.6914478	2.09478654	2.33156837
PINX1	1.6409295	1.85733509	1.48088241	2.56480429	2.07198897	2.40770592	2.20996648	1.55529316	2.12429693
PIP4K2B	4.12208575	3.80399917	3.40191136	3.50097259	3.1630888	3.75630978	3.03298578	3.51133117	3.26192288
PIP4K2C	3.7635272	3.23257875	4.50021545	4.13549294	3.87810791	4.31109888	4.15895444	4.6917643	3.9419773
PIP5K1A	3.47673574	3.24709723	4.15068547	3.86258896	3.7903207	3.72838728	3.87312406	3.7055044	3.58155028
PIP5K1B	1.12865702	3.19330431	4.03306279	2.10657911	4.6823299	2.47892256	4.27497493	3.89395535	4.92753982
PIP5K1C	4.05482242	4.01835286	3.52963473	3.20066026	3.67710954	3.89513051	3.3345681	3.14826593	3.02698222
PIP5KL1	0.28602079	0.20362114	0.28511229	0.44187747	0.12234359	0.14478896	0.30493035	0.29549763	0.1979322
PIPOX	2.03540179	1.59346268	0.91171641	0.33499524	0.52313944	0.5342548	0.33857924	0.3129786	0.34284201
PIR	3.11653896	2.6904193	2.64786746	1.91199817	2.16204923	2.6676632	2.14183957	1.96318023	1.46755751
PISD	3.07623029	2.59022753	3.74396457	2.88087528	2.92578956	2.98135572	2.43825316	2.04067943	3.33894938
PITHD1	4.26916888	3.14144361	3.09266149	3.63366385	3.77764927	3.7111447	3.73329419	2.59287117	3.741952
PITPNA	3.44357238	4.42125088	4.7521691	4.7325465	4.25841981	4.41658098	4.40372004	3.67233219	4.73061023
PITPNB	3.88902465	4.23062601	4.06540178	4.48274146	4.69445804	4.91728426	3.81210945	2.96587494	4.32192674
PITPNC1	1.57084935	1.95106135	1.89863766	1.90559502	2.00837813	1.70074686	2.15199654	2.81083372	2.01187043
PITPNM1	4.13468441	2.78070306	2.93234632	2.77450131	3.51683041	3.22260413	3.90901361	3.64904615	3.46247355
PITPNM2	1.45707723	1.61338112	1.56576042	1.58026003	1.65096402	1.77718874	1.13427829	1.02816222	1.31449091
PITRM1	2.92299543	3.08099459	2.57424089	3.53791768	3.05554245	3.52539178	3.30442065	1.63273977	3.65887593
PITX1	5.14418614	3.4445348	2.37997803	2.74187753	3.41690193	3.25701417	3.66650261	2.16714544	3.34910886
PIWIL2	0.16734793	1.67990693	1.17014815	0.1376916	0.85958137	0.11411458	0.33076893	1.27827936	0.68665766
PIWIL4	1.07618723	0.76418902	2.03109618	0.92178539	2.25483163	1.60137511	1.31784771	1.62719187	1.43987778
PJA1	3.69894673	2.74130616	2.58081356	3.23745631	3.52164337	3.07545614	2.93478421	3.58137599	2.61967373
PJA2	3.72007226	5.41009829	4.85991421	4.82402362	5.08077869	5.40757889	4.89275002	4.18627964	4.44015786
PKD1	2.19393507	2.4599889	1.22306895	1.75591919	1.39964112	2.33581507	1.7891499	1.58401278	1.71525234
PKD1L1	0.06718935	0.33102095	0.57701618	0.19684409	0.09562431	0.19656897	0.0908569	0.11189003	0.04547074
PKDCC	4.08856871	3.95104708	4.02497458	4.06447417	3.65641502	1.7855669	2.10621751	4.20892434	3.0360687
PKIA	0.17098806	2.05454767	0.86933428	1.77826026	0.4610126	1.23912746	0.35588222	0.7275855	0.29856106
PKIG	2.67909749	4.39910274	3.77465438	4.0290935	2.47449142	4.02370114	2.84390107	2.35702665	3.4371207
PKM	7.77374231	6.27075567	6.86597906	7.25706576	6.98352012	7.09350831	7.6500458	6.52113744	7.38818606
PKMYT1	3.29454657	1.73353448	1.13990004	1.70133218	2.20308226	2.1797953	2.49058401	1.70958139	2.29315017
PKN2	2.36862959	3.23946526	3.67380835	3.80578969	4.3894127	3.71708939	3.59099769	3.66948237	3.59269062
PKN3	3.57356592	1.88458666	2.55391206	2.10303788	2.19767729	3.32329313	2.9174369	2.7749887	2.14242429
PKNOX1	1.42075961	2.00748618	2.08329717	2.20647573	1.9389777	2.00165682	1.83895763	1.84383783	2.30282818
PKNOX2	0.02212676	0.94991316	0.36459656	0.59895988	0.05722826	0.57097129	0.0716035	0.11357493	0.04044867
PKP2	2.28290045	2.86690951	3.76707489	3.84107675	3.44788739	2.94634496	3.9471067	4.71878951	4.92331754
PKP3	5.75590979	3.27745709	2.94644025	3.9209971	4.40751456	4.68331889	5.80572769	5.0227043	5.3111743
PKP4	3.7870748	2.83569752	4.0596521	3.13304983	4.24643825	3.08753372	3.56502639	3.86205107	3.50243665
PLA1A	1.45011483	1.91070362	3.78801461	1.22783039	3.67276601	1.97589974	1.30026252	1.28773383	0.86879525
PLA2G10	2.37508148	2.68832744	3.23103397	1.42437955	1.23427966	0.86001909	2.06963456	2.72459661	1.67323374
PLA2G12A	3.10283952	2.95996125	3.3443652	3.07356538	2.87703283	2.97519935	3.04918569	3.48255748	3.63628184
PLA2G12B	0	3.02138968	0.53906453	0.07552493	0.30397176	0.01182262	0	0	3.36122297
PLA2G15	3.29523863	2.63135216	3.12482382	2.64751377	2.86711447	3.31274885	3.25294864	2.33511597	2.33922112
PLA2G16	2.21599594	3.31813679	3.86787505	5.01337895	1.60908153	3.01878185	4.80554857	3.62664692	2.04363948
PLA2G2A	0.10428379	9.77115305	4.21308346	4.97895951	6.41298441	4.14446089	4.15089475	3.21506134	5.1873808
PLA2G2D	3.81541457	1.58090099	1.4979358	1.41862846	4.92354865	2.7408564	0.43865792	0.51819532	0.51191546
PLA2G2F	0	1.73260394	0.07177791	0.16655728	0.5898117	0.00207781	0.04666359	0.01518783	0.27537856
PLA2G4A	1.82706253	3.40571199	3.85947214	4.97203232	4.4038649	2.70857438	3.96600567	3.54611915	3.25744817
PLA2G4B	0.0483637	0.03708232	0.14630184	0.05784069	0.09289157	0.08480189	0.00978061	0.02194788	0.09180891
PLA2G4C	0.39313145	1.30218762	1.6853254	0.75297041	0.77060698	1.16849007	0.56356441	1.20166368	0.20991399

PLA2G4F	2.08862843	1.13886038	1.18328695	0.13336734	0.31886018	0.03468558	0.88549355	0.27570448	3.44706506
PLA2G5	0.57936159	0.72678504	0.38739305	1.10115826	0.09175233	0.6537113	0.18803522	0.14348703	0.08558894
PLA2G7	2.86231054	3.86209588	4.78052769	4.25462063	5.79378626	4.10275724	3.35091596	2.77782181	3.27072954
PLA2R1	0.13136198	1.56766469	2.08744086	1.10953052	0.30810132	1.00519647	0.70419937	0.38058777	1.36229549
PLAA	2.75124331	2.95151708	2.64563179	3.30312739	3.7984967	3.32399536	2.94966231	3.23892096	3.6433009
PLAC8	0.72738706	3.43920891	2.87905835	3.22571151	5.58526504	4.29876825	3.55648017	1.44406192	1.36067626
PLAC8L1	0.34432979	0.15093497	0.55871691	0.52503354	0.75528773	0.59394005	0.09647054	0.1996507	0.66630832
PLAC9	1.05639966	3.37277812	1.57868019	1.70538727	0.48708305	1.66140392	1.06029377	0.9693315	0.53580972
PLAG1	0.05925212	0.19885101	0.28571823	0.3524058	0.26383136	0.52092752	0.34074761	0.85435151	0.24048087
PLAGL1	0.49121138	2.0501838	3.45575726	2.65454715	1.3338316	1.92913135	1.2247722	2.98978252	2.21027633
PLAGL2	3.57470857	3.34429324	4.52916571	3.07562089	3.63381175	3.01182282	2.81736256	2.96743276	4.09174847
PLAT	1.95535412	3.70374491	4.23465066	3.03644532	1.70165984	3.36122377	4.32369534	3.40793194	2.41137905
PLAU	4.37601852	4.0999192	5.45710879	6.18826709	4.21630383	5.65104608	5.54150288	4.7639098	4.40240218
PLAUR	4.40601343	3.62984268	4.74181505	4.94239016	5.60945164	5.160743	5.68408846	4.90980007	3.75371137
PLB1	0.30581825	1.54848741	1.10865813	0.49030974	0.47027567	1.24423978	0.50251914	1.54916284	0.74170033
PLBD1	2.74982303	4.97346511	7.15366592	6.28424026	3.99289687	4.79970919	7.15086762	5.11295314	5.61087425
PLBD2	4.22478226	4.74035788	4.53556911	4.58160494	4.618554	4.82101656	4.04425626	4.1776012	4.00115207
PLCB1	0.15762004	2.05765067	1.43818785	1.36065591	1.1356512	1.79404655	1.50391736	0.73441003	0.59927777
PLCB2	1.63545819	1.38752601	2.59944093	1.66226503	2.40952251	1.85310761	1.46633117	1.07457707	1.51459357
PLCB3	3.92148089	3.80683096	3.10117061	3.57693157	3.77775668	3.09943918	4.58016565	3.40399599	4.84857557
PLCD1	1.52884773	2.2900814	2.41813213	2.03514393	1.116895	2.36224784	2.64406911	2.66650883	1.77500302
PLCD3	1.9735053	3.18213873	1.77318373	4.3520245	2.40516293	3.22551514	5.11008027	4.70713763	2.39272511
PLCD4	0.28639892	1.04989705	0.66714506	0.91537095	0.72466695	0.83822374	0.4635001	0.5908672	0.36449735
PLCE1	0.71588293	1.88916817	1.57328606	1.63022248	2.15043341	1.85947037	1.70634808	0.95993471	0.92113271
PLCG1	2.61140738	2.73750785	3.14758303	2.83921482	2.73413474	2.63267131	2.51741865	2.07697576	3.06997506
PLCG2	0.71387584	1.72553541	1.4768673	1.43064065	1.84268094	1.6350402	0.88918051	0.7908409	1.83006747
PLCH1	1.44847475	0.96208079	1.75875432	0.90343904	3.06410952	0.87852925	1.10504383	2.01064942	1.68769384
PLCH2	0.14462422	0.25004641	0.15937963	0.42093771	0.20721059	0.29547219	0.02606952	0.22624172	0.2713282
PLCL2	1.9438167	2.64574754	2.68158628	1.92967931	2.52000249	2.47500693	2.43661839	1.95663537	1.40255038
PLCXD1	1.91009817	1.2317954	1.38381218	1.28366152	2.58792116	1.57409366	1.55790915	2.60958909	2.11108506
PLD1	2.40233359	1.83915381	2.13910094	2.22021376	1.21810496	1.81668951	2.09038484	1.93602259	1.33579852
PLD2	1.75808943	2.45544866	2.81199734	2.53599974	2.35698741	2.52619269	2.19319725	2.35199293	2.68916503
PLD3	6.08330268	5.78486214	5.67886018	5.33580715	6.01091589	6.09792395	5.25915864	4.74215119	4.84307741
PLD4	0.43985905	1.47114821	1.92438149	0.15944231	0.23460828	0.49894674	0.31774857	0.65150989	0.13047395
PLD5	0	0.07458598	0.26854304	0	0	0.02916418	0.0022838	0.04633959	0.00600847
PLD6	1.23986527	0.70257363	2.23704055	1.0913771	1.35058352	1.42347792	1.20133966	1.07762219	1.62047637
PLEC	5.52081108	5.00375097	4.55814042	5.15779833	4.25469421	5.5824891	5.43938646	5.88314507	4.58930094
PLEK	3.82807971	4.10094768	4.33652425	4.67108378	5.73500804	5.23181528	4.01624086	3.75969389	3.48352661
PLEK2	4.33137995	4.42624875	4.37449098	5.10170632	5.48897728	4.76894964	5.73092143	4.82468463	6.07185365
PLEKHA1	1.65803871	2.14834034	2.29309626	2.61278722	1.96353811	2.16784455	2.20612182	2.22327006	2.32487767
PLEKHA3	1.00149092	1.57229264	1.77455774	1.52737542	1.5954587	1.71587358	1.57563003	1.46767537	1.43241922
PLEKHA5	2.07606469	2.47104516	2.74068726	2.22643348	2.40535732	2.24237724	1.61934531	2.66102981	1.68727787
PLEKHA6	4.17882818	2.46816754	3.32148917	2.78122758	3.15195964	2.73792757	4.06677933	4.71019255	3.73340215
PLEKHA7	2.63106068	1.4159652	2.46298893	2.31548943	2.6735036	2.10976815	3.09922684	2.57166643	2.74886671
PLEKHB1	3.33334814	3.50033795	2.42375307	2.5660622	0.54655277	2.0359086	1.75911846	3.01183981	4.17368523
PLEKHF1	1.67601574	2.42393672	2.3049445	2.16605722	2.50552577	2.32054088	1.94847543	0.90426325	1.74740471
PLEKHF2	2.61010785	3.31582268	4.08095548	3.92547517	3.94313585	3.7336425	3.83108588	3.22049608	3.04529118
PLEKHG1	1.24860265	3.07025908	2.64207994	3.29021256	2.87209398	2.56024139	2.65633309	2.33841834	1.69998717
PLEKHG2	1.3926125	2.27243381	2.837877	2.7183792	2.57399673	2.40622952	1.87745379	2.42740496	1.34893138
PLEKHG3	2.05744602	1.7096063	1.03107781	1.9436082	0.51590517	1.77948862	1.37909897	0.46053065	2.76097544
PLEKHG4	2.87704851	0.5124941	1.75210568	2.33201949	1.82976235	1.80851877	2.19153312	2.99844328	1.71428333
PLEKHG5	1.42771294	1.36899485	1.34195875	1.77589684	1.49075291	1.53702263	1.42129564	0.77206797	3.73271689
PLEKHG6	0.84367644	2.65118929	2.83663612	2.56914264	1.22242799	1.19113246	2.17334334	2.78828943	3.2958938
PLEKHH1	1.15903274	1.40929384	1.82756973	2.15093348	2.99570344	1.31641543	1.1840584	1.52802462	3.19024569
PLEKHH2	0.24793286	1.3299453	1.72778132	1.1286788	1.21785174	1.14273584	0.99735249	1.37949558	0.35859492
PLEKHH3	3.12537186	1.59238819	1.91314202	1.75939684	1.69695785	2.18769626	1.64393793	2.20804213	1.54530146
PLEKHM1	3.03920353	2.38070508	2.36097674	2.21540423	2.35692069	2.77858183	2.30944599	2.8167133	2.46657927
PLEKHM3	0.48696809	0.95498077	1.01654667	0.89480299	0.80903973	1.18639863	0.733401	1.04279164	0.72516325
PLEKHN1	0.5928911	0.07886095	0.51114672	1.47862244	1.05808505	1.32034871	1.41433834	1.83898304	1.22392783

PLEKHO1	3.30803054	3.69592967	3.12525335	2.75848777	3.00513491	3.84999748	2.46327255	1.51590046	1.69826648
PLEKHO2	3.47889885	3.97723415	4.07094261	3.52694196	3.94144512	3.99763812	3.54419211	2.60217315	2.49569281
PLEKHS1	3.65558781	2.52604436	4.81815582	2.47558418	5.02730859	2.24504745	0.93128443	3.86010206	3.29095565
PLGRKT	3.20261407	3.75928545	2.88142502	3.11845042	3.83060488	3.62593889	3.12398051	4.3450236	3.97609673
PLIN1	0.09292555	0.53194011	0.2124045	0.82729927	0.04654506	0.03139578	0.03406836	0.07917395	0.03489142
PLIN2	2.0699401	4.83534566	3.38172043	4.43237841	3.92664553	3.54569934	4.27697414	3.33064517	2.88070827
PLIN3	5.64220781	4.7662502	4.55579886	3.89520594	4.72118601	4.71198066	5.21388709	5.19084428	5.43029106
PLIN5	0.19112614	0.08828891	0.36520902	0.18712975	0.02770935	0.22843406	0.13403358	1.08560187	0.04311362
PLK1	3.89459654	2.94142262	3.42321291	3.60444603	4.14754686	4.03362149	4.89438028	3.26474192	4.00924843
PLK3	2.65051047	2.11071367	3.10544654	3.69143471	2.56790632	3.7268819	2.64602067	3.09839379	2.444324
PLK4	2.01764308	1.05540428	2.11058645	2.54654089	3.01679328	2.25369293	1.95528895	1.51027851	2.36972267
PLLP	3.57059426	2.00722834	2.85121944	2.54952416	3.05918052	1.25644688	2.28667436	3.01616316	0.88848016
PLN	1.38035141	6.36563863	2.54673966	4.00380925	0.81735504	4.33918644	1.01989372	1.04315824	1.01099284
PLOD1	5.44336863	4.26845794	4.60433549	5.16447659	4.21186935	4.90631149	5.45655697	4.2689946	4.48978302
PLOD3	5.33788921	3.81837578	4.69449501	4.64705181	3.41296097	3.82632086	5.48285492	4.08844094	4.63233122
PLP2	6.57935768	7.12153589	6.58954958	7.65301511	8.1087107	7.06577048	6.75519739	7.51491405	7.83118782
PLRG1	2.55783548	3.1120662	3.19793766	3.74677578	3.90329129	3.48388375	2.85152816	3.20003537	3.66622068
PLS1	5.38406414	4.2536158	5.21795146	4.72148232	6.10826071	4.51870486	5.28461701	5.78421942	6.09159669
PLS3	3.52060503	4.03641085	4.52313727	4.49839165	5.47656556	3.84460052	3.4151836	3.70676542	2.85479499
PLSCR1	5.6406162	5.5632692	5.02186432	4.66832125	5.80260489	5.58261401	4.44425945	4.19332978	4.68728999
PLSCR4	0.69735821	4.15638857	1.97355831	3.32548816	1.15921015	3.06432752	1.96659814	1.33618899	1.92559901
PLTP	4.58466356	8.03316653	4.47452436	6.91993821	4.63616731	6.80454029	5.54934987	3.21893214	4.12692336
PLVAP	5.64890897	6.23461208	6.68835266	5.72083978	5.86275766	5.90399249	5.57682498	5.27013897	4.89211079
PLXDC2	2.0134111	4.46565826	3.16488098	3.92137052	3.1641127	4.09974552	3.64537104	2.37570261	4.05043583
PLXNA1	4.37629294	2.46134424	2.82039028	2.75546679	3.25040517	3.0090514	3.53098138	2.7521869	3.1217678
PLXNA3	2.76664488	1.53629457	1.99669983	2.06663479	1.69983068	1.93872326	2.46224016	1.64087733	2.4025713
PLXNA4	0.20508636	2.05615691	0.48447789	0.76540366	0.06698774	0.53731989	0.33289983	0.29533986	0.03924465
PLXNB1	3.99171571	2.5280195	3.57472288	3.18962604	2.53445272	2.6088188	2.40190521	3.54593083	3.62509492
PLXNB3	0.07103904	0.59658729	0.22242905	1.11204193	0.09005131	0.46761342	0.76120748	3.08733413	0.14055854
PLXNC1	1.39232191	3.01862684	3.26776574	3.05173209	3.52320131	3.42679305	2.36869344	1.78498791	1.78027075
PM20D1	0.14574802	0.43161089	0.41698057	0.2098219	0.21504873	0.30789715	0.17283123	0.1382469	0.13959424
PM20D2	2.01519523	2.72176327	2.3628895	3.90691248	4.02631932	3.68751812	3.11284057	2.42708516	3.69708743
PMAIP1	1.74944069	1.64318441	1.69120925	3.28807094	4.19071665	3.64474281	2.76662766	1.55296965	2.90453224
PMEL	1.07542683	0.60692355	0.88867752	0.58982832	0.35530049	0.44536308	0.35512953	0.73673126	0.71960876
PMEPA1	3.19177438	3.8465677	4.99276726	5.60300238	3.81565375	4.71007098	4.00515085	4.4468316	2.60649184
PML	3.69635478	3.42834191	3.38330554	2.86024358	4.16624601	3.88421008	2.48751452	2.57701379	3.47544767
PMM1	2.59108147	2.30884872	2.5057321	1.69983334	1.06572429	1.71099242	1.30967402	1.5813455	2.81629658
PMM2	1.77626815	2.51230689	2.77886503	2.09660191	2.29465158	2.14010953	2.59925252	2.29829041	2.81255163
PMP22	3.42402452	5.95491226	5.10094043	6.01306689	3.90055362	5.38646931	4.66420805	3.69783683	3.33267584
PMPCA	3.08647525	2.3290607	3.04107111	2.64078861	3.07904473	2.90173915	3.02357582	2.83059442	3.51161794
PMPCB	3.77899906	3.84123331	4.16790322	3.94137435	4.43013175	3.76684985	4.03646396	3.36927442	4.3346836
PMS1	1.05041066	1.87340697	2.0793001	2.43174	2.68487315	2.22018655	1.6685258	1.45359277	2.08627604
PMS2	1.98738335	1.90400791	2.13406871	1.78455564	2.27476227	1.83513167	1.93501258	2.11751421	1.79752111
PMVK	4.9513588	3.9671186	3.78850009	3.76032651	3.63276367	4.09085259	4.50925571	5.04176235	3.6129251
PNCK	0.03535671	1.32561201	0.09989796	0.29775384	0.00879329	0.87865131	0.1465652	0.03811943	0.16347114
PNISR	2.30154655	2.37588567	2.56177445	3.17912761	3.46844979	2.63270272	2.24386177	2.04287342	3.59421702
PNKD	4.68267631	3.66210187	5.03056282	4.10871365	4.7958578	3.95478113	4.6770403	4.77448844	4.35522318
PNKP	3.44177472	1.74774068	2.4999726	2.20627649	2.41528538	2.31017613	2.00065673	3.08315022	2.6336245
PNLIPRP1	0	0.04127248	0.61768874	1.16753426	0.01344598	0.00803457	0.00436886	0.02513467	0.17014089
PNMA1	4.00184581	4.49029885	3.25085755	3.86725494	5.05729042	4.0999735	4.37840654	4.47408126	2.89018767
PNMA3	0.02888052	0.06533566	0.08855228	0.02314878	0.12061135	0.12975127	0.00580904	0.0111997	0.01018638
PNMA6A	0	0.2509159	0	0.7180284	0.02018558	0.21371135	0.23632025	1.00037741	0.03427822
PNN	3.57021941	3.4415814	4.23190368	4.69750643	4.66386979	4.09648088	3.77033302	4.09303792	4.89867318
PNO1	3.00686151	2.95883927	3.44489813	3.51103304	3.7146407	3.46537023	3.24185136	3.37161605	3.61675262
PNOC	0.05053211	0.37792432	0.77611509	0.62460801	0.38674464	0.21603298	0.05041673	0.1915243	0.45259565
PNP	4.84098358	3.94910692	4.59207734	5.05586948	5.32185257	4.87879961	5.02982804	5.09099847	4.71554974
PNPLA2	3.86273465	4.34613203	4.12835423	4.71457048	3.76403995	5.33558083	5.69080678	5.81855019	4.88899372
PNPLA8	1.71650411	3.09272871	3.21838654	3.22466591	2.64060816	2.76036981	2.91643137	3.11171829	2.72124126
PNPO	4.17675651	3.12983556	3.49119295	3.53606961	3.20291224	3.43003054	3.38330581	3.71278922	3.85301992

PNPT1	2.10323536	2.68507463	3.06570578	3.26781221	3.58098677	3.37514243	3.34151764	2.93311404	3.45555908
PNRC1	6.25814801	6.37474635	5.52331781	6.14541476	5.51592877	6.16661979	5.08459169	4.66617349	5.23181654
PNRC2	4.68425685	5.22476646	4.65379625	5.01763465	5.21718304	5.13713809	4.62074892	4.16173819	5.82904819
POC1A	3.21849447	1.76910665	2.42921457	1.71656946	2.19118259	2.40938355	3.23247125	2.50581397	2.82752156
POC5	1.62669538	1.69643291	2.81002585	2.13533049	2.57755915	2.10714086	1.52460027	1.81739942	2.29764368
PODN	1.83904323	4.87597342	3.40475286	4.26113398	1.84590263	4.65571667	2.83868015	2.87354554	1.23569574
PODXL2	0.57081059	1.94843533	3.23889562	4.10863782	0.20991474	3.05512479	5.88679094	0.38661635	5.59814381
POFUT1	3.3669351	4.1373243	4.8368132	3.43795063	2.88814289	3.50230652	3.80945424	3.17235252	3.78412398
POFUT2	2.2749112	1.80144874	2.68685377	2.10314515	1.89162507	2.09251676	2.01392994	1.98117685	2.06751548
POGK	3.68977193	2.70723548	3.15911343	3.4903769	3.46750313	3.39282098	3.78474493	4.16089345	3.05737243
POGLUT1	2.1752944	2.02305221	2.06387153	2.15413911	2.3531287	2.08033478	2.1722274	2.29044753	2.57667655
POGZ	2.73739259	2.66806798	2.95581609	3.23149546	3.21076131	2.95046418	3.01951706	2.98831334	3.00254577
POLA1	1.89187399	2.45976903	2.26974524	2.22839427	3.68794303	2.43273321	2.17565947	2.28872762	2.552443
POLA2	2.57288768	2.14210124	2.14820265	2.204037	3.2092389	2.49922926	2.70767797	2.1593297	2.98926934
POLB	1.90693652	2.0618452	2.19615557	3.03458131	3.20370759	2.83355662	3.46363626	2.3921216	2.49255765
POLD1	3.40376489	1.7544256	2.18235091	1.85374804	3.16667865	2.33969357	2.22724872	2.08501133	3.00296323
POLD3	2.6012064	2.60984774	3.60522591	2.41590753	4.1863975	2.50622322	2.82061802	4.31174686	2.25994256
POLD4	3.15447419	2.40977534	2.66159978	2.69882168	2.03138232	2.51793691	2.53873939	3.01313312	2.76110019
POLDIP2	6.20243442	5.5801827	5.70283205	5.40313554	5.44334303	5.71851528	5.55371252	5.73877113	5.6491098
POLDIP3	5.09534599	4.44684441	4.31438195	4.4404007	4.33439845	4.60505326	4.22198933	4.25280165	4.61584671
POLE	2.24305096	1.29105891	1.69672131	1.75889764	2.71019828	1.94781366	1.99867245	2.37872917	2.31028347
POLE3	4.83351673	4.21019048	5.46098487	5.01377891	4.92104967	4.81031228	4.60036675	3.66242853	4.82150589
POLE4	5.34387346	3.09327253	4.03709588	3.31854609	3.34577176	3.47717417	3.21217026	3.08520871	2.77395907
POLG	3.5401779	2.54881087	2.71141076	3.06401705	3.15957705	3.23159429	2.71798254	3.92720269	3.35758251
POLG2	1.44881979	1.15706051	2.15825391	1.87426683	1.59506434	1.63921518	0.98365779	1.92891372	1.56732936
POLI	0.42947694	1.07982081	1.21172406	1.61782315	1.39866874	1.25028391	0.63305188	0.82206142	1.52471575
POLK	0.77188111	2.35772843	2.23396419	2.10685132	2.22911575	2.33748119	1.85563962	1.96230864	1.81560452
POLL	2.4115872	1.64390147	1.64839794	2.0652204	1.87476364	2.07061454	1.61438575	2.37699683	2.3755516
POLM	2.76170867	1.61233006	2.56400667	2.50112819	1.90703343	1.99905336	2.13544634	2.69287216	2.17020822
POLN	0.06461454	0.10930974	0.35226208	0.15555617	0.27455464	0.10532256	0.03627248	0.12537835	0.08432645
POLQ	1.44072488	0.49672455	1.27341851	1.09211038	2.72588902	1.19320767	1.15330181	0.75067652	1.57963859
POLR1A	2.62712004	2.04789351	2.15662244	2.6839298	3.132992	2.93638657	2.8089589	2.91224426	3.14644906
POLR1C	3.43857434	2.64360935	4.0926148	3.79302669	4.00029055	3.46415238	3.51511186	3.93487682	4.13592517
POLR1D	4.63038475	3.86505689	3.76350382	4.0004458	4.02793552	3.67183906	3.79285697	4.12849592	4.91826687
POLR1E	2.64604898	2.56542692	2.38838726	2.99323049	3.35135287	3.23811201	2.99693173	3.2372848	3.10986575
POLR2A	4.57972264	4.08412244	3.98345772	4.73080564	4.57912372	4.68742241	4.44373668	4.046523	4.69893283
POLR2B	3.3510605	4.11426526	4.41453543	4.62168108	5.01139969	4.4442861	4.14925383	4.45546183	5.0008639
POLR2C	4.69145359	4.07379916	4.61600569	4.01433395	4.18128114	4.06371027	4.48275826	4.4213315	4.34036242
POLR2D	2.84157026	2.36237588	3.38351519	3.12721437	3.15963024	2.96357835	2.88707507	2.74035415	3.07587413
POLR2F	0.01031379	0.00886604	0.05141885	0.0185107	0.0190038	0.01701323	0	0.00993914	0.0054305
POLR2G	5.28895655	4.36363692	5.0914507	4.86709847	4.45095682	4.60021062	4.65244608	4.17655807	4.6688642
POLR2H	4.74155004	2.96820005	4.98006315	4.26633259	4.23024475	3.66317895	3.9717012	4.62645619	4.5997894
POLR2I	4.68840194	3.63918639	3.7835753	3.91922561	3.90145006	3.37935366	3.68712512	3.83292674	4.1634894
POLR2J	6.11069968	4.35827827	4.95712734	4.04853225	4.43094226	4.26769018	4.66334376	4.83574428	4.72250109
POLR2J2	0.85788111	0	0.04804109	0.17787958	0.52878407	0.05683411	0.02673427	0.31831275	0.36447539
POLR2K	5.04801079	4.9314397	5.95351141	5.87312322	5.22601822	4.92016571	5.01443862	4.60032282	4.90173377
POLR2L	6.88610393	5.62278978	5.2227066	5.63650205	5.42119883	5.45873633	5.99887662	5.51735905	5.63942718
POLR3A	1.88192663	1.75794856	2.54001283	2.29249668	2.68586861	2.2888697	1.99446402	2.38861038	2.56923578
POLR3B	1.39660228	1.89508295	2.1038297	2.48022479	2.77666964	2.2299857	2.5352497	2.75489385	2.01318548
POLR3C	3.16484121	3.3172913	3.87415513	3.24345563	3.85860613	3.33375825	4.04209599	3.04393463	3.81204048
POLR3D	2.3406953	2.16313138	2.06612378	2.57381832	2.5507127	2.78601748	3.25366504	2.6198095	2.42771712
POLR3F	1.37155067	2.16956158	2.39430443	2.99219071	2.41316313	2.16268662	2.22244405	1.51206068	2.59091028
POLR3GL	4.24700992	3.66160544	3.04147067	3.43041891	3.30510976	3.54914177	3.43084855	3.30548464	3.00348949
POLR3H	3.79492293	2.32646724	2.47038105	2.23492553	2.2784718	2.57847004	2.44232838	1.77215404	2.73390192
POLR3K	3.55154056	3.05312092	2.62501816	2.75545771	3.45528772	3.42870165	4.34571916	2.59966747	3.31140324
POM121	3.48215726	2.37936423	2.75826149	2.34127433	2.64660204	2.73419988	3.12983625	2.69881684	3.35409397
POM121L12	0	0	0	0	0.01739408	0	0	0	0
POMGNT1	3.46289439	2.75940011	4.09626391	3.2615771	2.90990437	3.34276158	3.45664047	3.50808534	3.54779322
POMGNT2	3.33209758	2.82700808	3.40096354	3.14670538	3.26357589	3.63756526	3.74612489	3.7795178	2.82918304

POMK	1.80315848	1.88078338	1.61416334	2.7460334	2.150816	3.07508113	4.06957282	3.26464868	1.99214801
POMT1	2.89718484	1.66247626	2.48219703	1.8198993	1.39582413	2.05470784	1.7259711	2.09768744	2.03839176
POMT2	1.62485767	0.86922836	1.07982352	1.27586206	1.31471308	1.20785323	1.106966	1.47690525	1.37674329
POMZP3	2.84323908	1.57662329	1.43637529	2.80274506	1.21490176	1.41727799	1.22695887	2.37300886	1.35760609
PON1	0	0.09277914	0.39591532	0.10423373	0	0.13960159	0.74225862	0.04876606	0.00674861
PON2	3.47380501	3.88625542	5.65258123	5.71706943	4.22517952	4.10668407	4.60907868	4.13363574	3.53648335
PON3	0	0.39133225	1.99659662	0.10940167	0.02205986	0.6818823	2.62155809	0.05792831	0.0512389
POP1	1.72755731	1.09909499	2.14008798	1.75177269	2.01343004	1.53869612	2.06636376	1.28734821	1.78403434
POP4	2.56432376	2.17194905	2.6002129	2.29029768	2.83653399	2.45712286	2.12226131	2.37363774	2.49145253
POP5	3.18077499	2.69606333	3.29923862	3.12909109	3.2930314	3.00859326	2.5638962	3.23901192	3.23299523
POP7	5.79140506	3.92241962	4.58927875	3.70677757	4.18340322	4.18894216	4.67352143	4.42146824	4.46073012
POPDC2	0.72326633	2.8962468	1.26054157	0.76592285	0.7493628	2.94064245	0.51897358	0.48358676	0.37706636
POR	4.31595454	4.21720475	4.36772865	3.40009469	3.25397752	3.5282736	4.15194006	4.24835215	3.72066637
POSTN	5.12507457	6.00211529	5.93087898	7.73907863	5.83563713	7.2682093	6.67036646	4.69092927	4.54537275
POT1	2.07333553	2.46911051	3.12513464	2.6843842	3.38000265	2.50454554	2.24763618	2.51069612	2.21655282
POU2AF1	0.58894309	0.85110554	2.1610637	1.92981255	1.79315178	0.82352338	0.20530615	0.79074297	1.57286249
POU2F1	1.81264702	1.90870759	1.6911099	1.82607402	2.37920694	1.79858191	2.16619946	1.96323011	2.16580097
POU2F2	1.96447275	1.58452833	1.622294	1.09454645	1.14606992	1.24672711	1.02339218	0.63539628	0.83673308
POU4F1	0.00799298	0.01824842	0	0.00958558	0.01961678	0	0	0.0030859	0.00840163
POU5F1	0.72912117	0.23977786	2.41495332	0.74274991	0.37528285	0.50796536	1.38481463	1.08771875	0.94245664
POU5F2	0.03686608	0.10884484	0.56120359	0.0347821	0.06741154	0.06327197	0.07163604	0.08192534	0.01672657
POU6F2	0	0.08614394	0.14591302	0.01585139	0	0.01295655	0	0.01527777	0
PPA1	4.81016628	5.02448687	5.43759198	5.8101248	6.2494248	5.62053436	5.81369978	5.53961931	5.66087828
PPA2	3.24051777	3.46499342	4.1387633	3.94459064	3.81466741	4.02013852	4.17381471	4.08212229	4.24796096
PPAN	1.71743776	0.63133069	0.8181085	0.91533117	1.44526122	1.35182342	1.01339714	1.21836586	1.5370819
PPARA	2.22138775	3.04147123	1.67341625	2.04454353	1.62689452	2.31974189	2.494484	1.58127912	3.21101405
PPARD	3.5789793	3.35014163	3.80027455	4.13870946	3.26669552	3.51937537	4.8531265	4.57499174	3.68084496
PPARG	2.39968677	2.50352826	3.40462118	4.29404183	3.70093296	4.42916243	4.72918308	3.99274987	3.05975165
PPARGC1A	0.01002351	1.77065649	0.54932582	1.43797207	0.08618871	0.46732174	0.49181842	3.15019749	1.39550158
PPARGC1B	1.23049925	1.22816872	1.35797314	0.85519662	1.81799278	1.44724435	1.63012411	0.89150821	2.34327082
PPAT	1.98159369	1.74378589	2.42732332	2.9341537	3.21463871	2.64368015	2.64941926	2.65955828	3.08411434
PPCDC	1.75067282	1.07974138	1.59573541	1.8226661	1.53682762	1.64426558	1.07028017	0.97500956	1.58244037
PPCS	4.45105416	4.08182007	4.75762076	4.14955024	4.22692594	4.32790965	3.93972399	3.51830085	4.51739117
PPDPF	10.027529	7.70919328	9.07506137	8.24835731	6.85297034	7.46930516	8.13734528	8.68556941	8.49342869
PPFIA1	2.50765415	2.54286313	2.88973632	3.13753971	3.52483792	2.83304124	2.94523792	2.78820119	3.0048265
PPFIA2	0.0336144	0.32558978	0.25289589	0.27123479	0.0489199	0.16372591	0.15887553	0.09816821	0.04834115
PPFIA4	0.03340088	0.09165147	0.38981473	0.33248707	0.09401256	0.11273495	0.12479647	0.16008633	0.29200491
PPFIBP1	1.85907019	2.54438926	2.58134381	3.2087743	2.90005551	2.54972883	2.36196838	2.5475806	2.42942555
PPFIBP2	3.10465455	2.05177279	2.24213656	2.16694997	2.72683985	1.82542063	1.7492132	2.22623306	3.0369289
PPHLN1	1.84213561	2.11148156	2.40156425	2.58436703	2.46421258	2.39092384	2.02340362	1.46206786	2.47106075
PPIA	7.9809864	6.69735685	7.17674762	6.82619861	7.0961631	6.83780643	7.55727859	6.94515433	7.52775145
PPIB	6.58027362	6.68746845	6.85792203	7.10426924	6.52872542	6.78391642	7.19059152	5.80155597	6.81576415
PPIC	2.15566072	5.24880244	5.37362566	4.71491945	2.78753315	4.6152743	5.90052351	4.01986014	3.68206299
PPID	3.14458759	3.75892982	3.72628452	4.34682248	4.65727102	4.1276921	3.68630407	3.58280105	4.36810376
PPIE	1.53131011	2.02224165	2.62297671	2.16578926	2.0818688	1.82577423	1.79390817	1.56280202	1.68457378
PPIF	5.3704343	4.33546655	5.00706675	5.08787607	5.81968759	5.34050926	5.28130379	5.25238664	6.19948536
PPIG	3.11598488	3.32256316	3.52791361	3.63177175	3.86978634	3.22508761	3.29437037	3.2377824	3.52936952
PPIH	4.88767772	3.2011526	4.41008538	3.62964006	4.50974492	3.76469038	3.42901813	2.69488515	4.04151114
PPIL1	4.00909691	4.00409564	4.72519919	4.69968071	5.05681568	4.83996309	4.57540071	4.7426419	4.57427585
PPIL2	3.46615634	2.48404086	2.57691658	2.64569114	3.04357568	2.73197095	2.45533066	2.50605815	3.19043949
PPIL3	2.33831979	2.69424294	3.05141292	3.65039404	3.35376108	2.988089	3.10474302	2.90342456	3.34923142
PPIL6	0.19022037	0.36445555	0.42888848	0.46342712	0.43378049	0.17695487	0.3293803	0.36380035	0.30691913
PPIP5K1	1.52614917	0.94593072	1.36980641	1.53202193	1.38362419	1.31529097	1.60819499	0.98657424	1.74621614
PPL	3.02888044	3.00128817	2.19185281	2.83959672	0.24397766	1.98104475	3.2084568	3.41778039	2.3474616
PPM1A	1.77115988	3.09690608	2.64823236	2.92558298	2.75065767	2.99115878	2.6460439	2.75310534	2.87979754
PPM1B	2.79479685	2.56354204	3.11897844	2.77939325	3.17994008	2.7171737	2.98027361	2.50407481	2.95324136
PPM1F	2.00952497	1.93625279	2.06421074	1.69382496	1.61665923	2.31332049	1.4852622	1.37906738	1.54730295
PPM1G	5.37517674	3.96386825	4.47934647	4.45118263	4.94205931	4.85953172	4.85874043	4.82846335	4.87344596
PPM1J	0.2865779	0.26470485	0.36262313	0.62095473	0.49725934	0.79872844	0.73831145	0.56332566	0.56157756

PPM1K	0.72521594	1.65910412	1.30274881	1.48165665	1.6465799	1.48367164	1.12756955	1.01542698	1.23261189
PPM1L	1.44846264	2.16218921	2.92287215	2.07287858	1.64811517	1.83669975	2.39190316	3.49619745	1.54766999
PPM1M	2.37907582	2.41567728	2.90214569	2.27276414	2.534502	2.5015559	2.4430438	2.07070652	1.52123583
PPME1	3.24020619	2.50189425	2.98682349	3.21584208	2.74045067	3.26153476	3.63970471	2.89587467	3.3163415
PPOX	1.36692386	0.94130813	1.55637343	1.29324287	1.08737088	1.00818581	1.23207646	1.5568962	1.74125751
PPP1CA	6.78007593	5.22276023	6.31415225	5.82793647	6.06554651	6.10498501	6.57517587	6.13987341	6.08787522
PPP1CB	4.92087425	5.01317847	4.8143018	5.27926809	4.62896823	5.06250018	5.08869668	4.65306331	4.7674727
PPP1CC	4.03851013	4.42823558	4.78557664	4.58030272	4.67201406	4.73470349	4.28462649	5.30156369	4.94146364
PPP1R10	3.14445718	3.08697912	3.88333704	3.66349967	3.57229704	3.41617351	3.0643965	3.45398393	3.95686417
PPP1R11	5.49102283	5.13618589	6.39314701	5.3801983	5.1315728	5.29272479	5.34566197	6.03626342	5.47175572
PPP1R12B	0.91962139	4.67819312	1.81325111	2.59026452	1.97029979	4.30185761	2.20098738	2.33073115	2.09374397
PPP1R12C	3.37174698	2.97562773	2.57089971	2.38121642	2.56286433	2.83765581	1.92706241	1.77542171	2.41581875
PPP1R13B	3.13360964	1.97177263	1.85642468	1.8823747	2.24836164	1.81813155	1.71395064	2.38872796	2.40538194
PPP1R13L	3.57579495	2.13432014	2.30310951	3.21501925	2.87759731	2.83116745	2.32793573	3.14691435	3.31443689
PPP1R14A	1.45959559	4.80497786	2.8890424	2.58693688	0.77853626	3.58951188	1.28726698	1.20401818	0.88694633
PPP1R14B	5.74002882	4.58252429	4.41663074	5.03300102	4.3702286	5.36300987	5.53156243	5.29488416	5.54511826
PPP1R14C	0.11392853	2.20078695	0.92521156	0.94833012	0.07124221	0.28173217	0.5566011	0.0700701	1.09028846
PPP1R14D	2.05453534	4.04707439	2.58410915	2.15535567	0.91938771	0.41098339	2.31227651	1.97978088	5.45340876
PPP1R15A	4.30335331	2.86729383	4.8855272	4.79183894	4.39163045	5.01449874	3.55651238	4.89875804	4.02570752
PPP1R15B	4.12246249	3.74109154	4.81349005	4.49742727	4.73667502	4.69622301	5.00345925	4.77944247	4.04139677
PPP1R16A	3.36904758	2.11619988	2.18255205	2.21909335	1.37910693	2.16191253	2.81644759	2.90606489	3.66775823
PPP1R16B	1.763801	2.28211728	2.25654349	1.55323623	2.85686202	2.55193597	1.27079481	1.49387348	1.26705091
PPP1R1A	0.02719927	1.5471009	0.23163492	0.40852179	0.04441556	0.35844299	0.04848436	0.0417212	0.0931445
PPP1R1B	0.89945057	4.8667696	2.46062447	1.5818326	0.17613997	4.78637709	5.91325558	5.45413454	6.46736599
PPP1R2	4.29342298	3.96579769	5.34159388	4.08658818	4.15018972	4.08849456	4.03138789	4.19300829	4.18343185
PPP1R21	1.89492794	2.81142243	2.94690594	3.066088	3.02647206	3.04377971	2.44461608	2.48576636	2.51175846
PPP1R26	3.41249833	1.77008223	2.59041776	2.32485952	2.44886392	2.43479813	2.51832112	3.36029144	2.43277792
PPP1R35	4.26114113	2.85384489	4.04291226	2.80494358	3.27684595	3.31970315	2.86657669	3.45389116	3.99505961
PPP1R3B	1.68785441	3.08967082	2.2775561	3.17125237	2.68701703	2.98277541	3.19830018	3.15991506	2.26582464
PPP1R3C	0.56599982	2.98856338	1.71586933	1.56439712	0.19132501	1.76610116	0.73034272	0.98254751	0.21795994
PPP1R3D	1.05565977	2.10719978	2.66321146	2.30491189	1.45673554	2.303093	0.69290638	2.42976908	2.5203205
PPP1R3F	0.49737795	0.8898673	0.48335302	0.43540905	0.43355025	0.76607255	0.34273205	0.4552033	0.35257128
PPP1R7	4.22330895	3.59764504	3.67119194	3.3801502	3.96682099	3.7374704	4.12346975	3.65484518	3.73511466
PPP1R8	4.0041341	3.43964987	4.13163178	4.24383942	4.2792052	3.86348999	3.70042008	3.05515664	4.00192214
PPP1R9A	0.183868	0.90158364	1.67941195	1.63915896	0.04558102	0.54132158	2.03515834	1.77229732	0.03227721
PPP1R9B	5.5332536	3.8079106	3.77707297	3.79582115	3.68095684	3.94888544	3.50876008	3.62317596	3.67303569
PPP2CA	4.60991721	4.34401331	4.83240435	4.86839969	5.09163388	4.92512065	4.42583878	4.80845674	4.74084016
PPP2CB	3.88533968	4.44761167	3.79154142	5.01639058	4.36078123	4.69487726	5.1661024	4.36320878	3.90480159
PPP2R1A	5.57556952	4.4422012	4.92643234	4.62613214	4.90400257	4.88437134	4.87571822	5.20765873	5.22097633
PPP2R1B	2.48477829	2.54450086	2.80048867	3.21262348	3.61828127	3.17036254	3.46711089	2.70362752	3.22959969
PPP2R2A	2.63281659	3.20332703	2.74147742	4.08224393	3.47970775	3.65731747	3.8122545	2.93458084	3.70264618
PPP2R2B	0.14431968	0.24376479	0.72568366	0.11147976	0.72289619	0.27098996	0.08188313	0.05938894	0.06661694
PPP2R2D	2.15355303	1.99526255	2.21053825	2.31685052	2.12262194	2.23420488	2.0934536	1.89882588	2.34281573
PPP2R3A	2.92567351	2.21558727	1.37326169	1.60419301	0.59487097	1.99656124	0.72774105	1.454463	0.5847977
PPP2R3B	1.05578032	0.51941547	0.56540268	0.61154369	0.91500531	0.94530734	0.37879534	0.73951258	0.95256693
PPP2R3C	2.40856734	3.28264403	3.00370417	2.79492674	3.31373871	2.97845321	2.7872456	1.99062515	2.89761096
PPP2R5A	3.87619854	3.98521238	4.54265032	4.08644442	4.05054109	4.09862262	4.86905388	4.65606032	4.4168644
PPP2R5B	2.5591762	2.35705438	2.3204934	2.74736442	2.02013762	2.64032475	2.88330724	2.43090759	3.0262039
PPP2R5C	2.64768653	3.65349687	3.04210214	3.65444275	3.50957364	3.39329697	3.4167131	3.4169067	3.81095997
PPP2R5D	4.82529467	3.96355529	4.61464721	3.95309875	4.12630668	4.14232695	4.54137491	4.62251328	4.22697354
PPP2R5E	2.62115485	3.04589155	2.86960822	3.11498876	3.09348609	3.24498454	3.01500137	2.81561528	3.17072045
PPP3CA	3.94095864	4.31931433	3.64434537	5.94357348	2.79135875	4.49910931	4.91935263	3.53572747	3.70380682
PPP3CB	2.33248401	3.71190122	3.87251135	3.73847386	3.39415175	3.738782	2.80540442	2.9737911	3.5378499
PPP3CC	2.44469656	2.16155599	2.47781435	2.96183557	3.64740916	3.27497881	1.59640686	2.84527057	1.23340328
PPP3R1	3.97341256	3.31088445	3.80484858	3.8121691	3.80530946	3.76691276	3.66317197	3.73884183	3.65494837
PPP4R1	2.96593237	3.0158058	4.1198041	3.5897965	4.02086806	3.77820858	3.97385602	3.94220038	2.97312728
PPP4R2	3.25642447	3.3136802	3.65931142	3.65921276	3.83160992	3.72550042	3.75435534	3.40921787	3.80277896
PPP6C	3.66495908	4.11380286	4.79886921	4.57291329	4.53735016	4.4414659	3.9726724	3.59425037	4.53695513
PPP6R1	4.87117443	3.4477238	3.78921518	3.62566061	4.41788297	4.19988344	4.12036125	3.60292121	4.42502451

PPP6R2	4.27197493	2.86795821	3.29971995	2.7215884	3.15740966	3.00123431	2.58588882	3.26803231	3.29281144
PPP6R3	3.10455975	3.52999987	3.91116725	4.09494926	4.07234425	3.89406822	4.19100252	4.17341764	4.26109418
PPRC1	3.46391403	2.66052708	2.77504956	3.41117092	3.65764369	3.1469601	3.21283698	3.52955447	3.74210545
PPT1	5.28057261	4.88659285	7.15649803	6.36705858	6.56240123	6.2515593	6.21349699	5.28158835	5.23212965
PPTC7	2.54421561	2.82603907	3.30166981	3.86283946	3.41484161	3.63285997	3.30039602	3.75649067	2.93629424
PPWD1	1.91927689	2.40348039	2.41400383	3.12505925	3.31816932	2.59869875	2.28200375	2.37256824	3.05528084
PPY	0	0.06209604	0.07788976	0.04363998	0.02256992	0.04013768	0	0.01416189	0
PQLC1	2.91206583	2.51575883	2.3146883	2.49299216	2.2560003	2.88170338	3.40911574	1.93754591	2.9075229
PQLC3	3.37305201	4.62711282	4.51732398	4.29915252	4.74945	4.22831111	3.79166385	4.42136587	3.39421581
PRAF2	2.49247759	4.12752347	3.35034316	3.46807643	2.21120202	3.4479271	2.88118272	2.74740233	2.36336917
PRAM1	1.7147878	0.85093468	1.42643019	0.71049294	0.95822648	2.48228909	1.08212878	0.48116616	0.29376694
PRAMEF10	0	0	0.03412451	0	0	0	0	0	0
PRAP1	0.02444994	6.68559505	2.99579793	1.08251392	0.93018503	0.72518471	0.59604154	1.92087455	6.4397417
PRC1	3.27745425	2.05515269	3.33305467	3.5879323	4.19711642	3.66914238	3.81333304	3.57154015	3.54156803
PRCC	4.55952765	3.34005748	3.93890811	3.76583951	3.91249296	3.93353345	4.32045375	4.4615459	4.21498437
PRCP	3.44403353	3.63610239	3.80763932	3.16566977	3.15484325	3.70549717	3.23227146	2.73225482	2.31254073
PRDM15	0.45322385	0.52782325	0.76938131	0.87078446	0.86280076	0.74373283	0.5715069	0.7078941	0.99163219
PRDM16	1.56584995	0.95992007	1.19492896	0.92128882	0.2329913	0.30752044	0.07613348	1.46490955	1.41509878
PRDM2	1.87624352	2.35274408	2.1304197	2.68685689	2.45737645	2.14660916	1.72587069	1.69678646	2.58202475
PRDM4	2.78139921	2.88109969	3.25092618	3.37728292	3.58435367	3.26182622	3.14111644	3.68140169	3.61692168
PRDM7	0	0	0.11109364	0.01061648	0.02709733	0.01299269	0	0.01023162	0
PRDM8	0.49308074	1.12402497	0.78574231	1.09738603	0.50994416	1.38535749	0.34513273	0.99352111	0.20588236
PRDX1	8.65889589	7.14200526	8.85646722	7.76338166	6.90057763	7.59583624	7.98917723	7.99157991	7.29910833
PRDX2	7.12248667	6.55946112	6.56122157	6.79424932	7.07125317	6.4540257	7.21870832	5.85007602	7.8325096
PRDX3	5.43218445	5.83512288	6.29638263	6.26847281	6.57355294	6.24438111	6.25493605	5.86227863	6.15673168
PRDX4	5.60891463	5.96237858	5.91969858	6.03640479	6.06064217	5.56514864	6.12225264	5.72626548	5.65266405
PRDX5	7.84351327	6.42606876	6.55349973	5.97062038	5.75709411	6.46091089	7.1128791	6.74600173	7.03935436
PRDX6	6.17299385	6.02234218	5.86304242	6.68945542	6.19396747	6.60337056	7.28859102	6.54457018	5.24866218
PREB	4.27909542	4.0818848	4.12786459	3.94470422	3.65706443	4.1077604	3.94166594	4.21454185	4.11237922
PRELID1	5.96120149	5.02245272	5.27674435	5.15232816	5.32077483	5.75759396	5.21893485	5.52356829	5.81711651
PRELID2	1.39810389	0.94767735	1.97961217	1.43461585	1.69900253	1.04646053	1.63692421	1.42763439	1.94795564
PRELP	0.77205242	4.26577755	2.2461429	4.14126315	0.68155023	3.38961943	1.07930666	1.22551606	0.88050962
PREPL	2.1841645	3.25349849	3.18463628	2.94965342	2.76651288	2.98809561	3.29208187	3.4039092	3.68481116
PREX1	2.86807601	3.4802706	3.60708141	2.89871386	4.01099183	3.93117894	2.9423248	2.4030975	2.46533628
PREX2	0.13273583	1.86538549	0.88210228	0.89606927	0.72062173	1.67875031	0.54745483	0.46077088	0.28977102
PRF1	2.86404523	2.27079743	2.61881307	2.66063458	4.99175131	3.00654698	3.14621833	1.0536966	2.77678398
PRH1	0.25985362	0.13342751	0.24284167	0.09445274	0.02484255	0.02958455	0.07125617	0.04628074	0.13268001
PRICKLE1	0.09358329	1.91548951	0.64355963	1.11206214	0.28167062	1.11653321	0.29027254	0.54942273	0.14748523
PRICKLE2	0.49384634	2.6357999	1.10456359	1.8912305	0.50135455	1.8371594	0.79906595	0.52680642	0.25122169
PRICKLE3	2.48826197	2.33502265	2.40982511	2.54362764	3.04270137	3.02142182	2.25928922	2.8728853	2.51774927
PRIM1	1.98341662	1.95374575	2.40970352	2.50506602	3.17628838	2.5489905	2.49446826	2.60831495	2.70235674
PRIM2	1.76233824	1.81076366	2.04969669	2.39969339	2.75462101	2.2176765	2.37018739	2.31870579	2.19511079
PRIMA1	0.02782221	3.01679331	0.40952552	1.28966832	0.68147575	2.01628902	0.10796523	0.35352843	0.06260894
PRKAA1	3.16473192	4.35203789	5.06703195	4.68448738	4.37354718	4.2021515	3.82173965	4.69261777	4.62325592
PRKAB1	2.62688626	2.88454487	3.99431923	3.06301029	2.42631527	2.26385933	2.53628227	4.51401972	2.57936837
PRKAB2	1.18123796	2.94577846	2.72221858	3.1191884	2.29504126	2.56923747	2.54283966	2.28062484	2.30088943
PRKACA	4.39223937	3.72344593	3.53107722	3.38444291	3.2161148	3.48038879	3.1558891	3.14551397	3.2347475
PRKAG2	1.11479168	2.44410235	2.55502236	2.41559671	1.64029982	2.30368176	2.2965531	1.96959339	2.26550005
PRKAR1A	4.55406622	4.97538629	4.82257854	5.18075332	4.72634076	5.0774862	4.23672969	5.59446624	3.90639881
PRKAR1B	3.63313256	2.7592745	2.55497929	1.93022655	2.32858477	2.0694434	2.25841725	1.62351794	3.8968964
PRKAR2A	3.27506522	3.6237728	3.71665605	3.89979224	4.0490161	3.92830867	4.52669436	3.52501474	3.95694912
PRKAR2B	0.74729134	3.29276567	2.10750724	2.86518635	1.81526201	2.27985209	2.03661193	0.91371825	0.76388669
PRKCA	4.31722924	2.89169184	3.82535251	2.70766071	2.43293976	2.77946991	3.18251585	3.58303436	3.22261639
PRKCD	4.60276056	4.02006313	4.61129485	4.47818884	4.40098377	4.12137047	4.67792767	4.24611613	4.69657372
PRKCG	1.2341166	0.24370556	0.5660085	1.96689339	3.07930167	1.4486446	0.01922902	0.24052565	0.75471044
PRKCH	1.13477173	2.23744719	2.17963101	2.1441641	2.86541707	2.23893638	1.82749356	1.90601096	1.10341208
PRKCI	3.64882778	3.13074849	5.45060682	4.28486634	4.97501364	3.60426048	4.45662321	5.05851336	3.79098262
PRKCQ	2.04276348	0.91950646	1.11314482	0.753049	2.58536321	1.93665913	0.63720025	0.65645424	1.7016424
PRKCSH	6.58081067	4.90561034	5.34828046	5.24170895	5.43451464	5.16015699	6.03388364	5.8184633	5.87918837

PRKCZ	2.66436902	1.20040724	1.16993206	1.69724951	1.60603155	1.65379968	1.8496537	1.38638489	2.32830628
PRKD1	0.39142982	1.80600736	0.97126574	1.75884612	0.42441575	1.84009904	0.73019607	0.90667152	0.20933782
PRKD2	4.19939699	3.05611712	3.39608596	3.06269536	3.87822468	3.64472997	3.49418179	3.78010944	3.57333588
PRKD3	1.93399032	2.97075022	1.93299852	2.80294311	2.55905784	2.84803759	2.36028927	1.54215774	2.53860772
PRKDC	3.28077711	3.96887788	3.56906136	4.41419902	4.79193577	3.7837699	5.03001528	4.08047268	4.28830354
PRKG2	0.07630688	0.34976259	0.065401	0.54515822	0.0346878	0.69480093	0.03787712	0.34726468	0.48244472
PRKRIP1	3.06016206	1.82887064	2.6796598	2.1927135	2.31219155	1.95622411	1.95618523	2.24990358	2.23059105
PRLR	0.03569933	2.25626716	1.01277743	0.59334701	0.25808984	0.54132265	1.78576323	0.79275691	2.6678368
PRMT1	5.20231147	3.44971651	4.18438542	4.50806322	4.60796088	4.58781896	4.5487608	4.41408761	4.98950834
PRMT2	2.61671036	3.19281428	3.09120116	3.23812632	2.70246029	3.23831086	2.95635477	3.2784976	2.78126517
PRMT5	3.40744462	3.34320063	3.23773044	4.08594497	3.97438614	4.12503114	3.78217842	3.95019952	4.35230638
PRMT6	1.06432985	2.97024454	1.68013882	3.73269282	2.19947886	2.86324899	2.79181892	3.31646785	3.15924143
PRMT7	1.4535334	1.47947106	2.01061945	1.61539168	1.56303633	1.50990169	1.46065194	1.94928147	1.8994349
PRMT9	1.21410652	1.65673503	1.95232185	2.07353824	2.16260417	1.97139464	1.38009758	1.68556587	1.54249386
PRND	0.02721281	1.70064729	0.82405801	0.98729817	0.07688989	0.79848481	0.24168603	0.12500828	0.01436803
PRNP	3.61610952	6.69259816	4.84454838	5.92637013	4.53026506	6.26120332	5.45733808	4.39058204	5.1475828
PROC	0.13914122	0.27355643	2.08671891	0.16541714	0.04430599	0.96796096	0.32018977	0.32613798	0.19370259
PROCA1	0.33521188	0.22379516	0.33431358	0.30862595	0.32721782	0.19185825	0.15816118	0.21824124	0.23049039
PROCR	3.72039863	5.44995533	4.54887596	5.48518519	3.16630061	4.80726564	4.60818561	4.95738649	5.73649945
PRODH	0.35200826	0.10325739	0.06453989	0.19760462	0.61038305	0.10161812	0.02708887	0.0379112	0.35775097
PRODH2	0.26723588	0.08172443	0.26361501	0.0159245	0.00819771	0.00489491	0.00796631	0.04556343	0.06849387
PROK2	0.3760123	0.8542927	0.84199045	1.10864999	1.35292838	2.20122161	0.20174985	1.11483793	0.65682561
PROM1	4.68508895	1.86060399	6.74309498	0.45956403	0.34250337	0.51172709	3.70037387	0.51029904	3.38799199
PROM2	3.13882972	2.57688286	2.83971785	2.68019697	0.4319764	0.21884841	4.87445957	4.31244252	3.59633672
PROS1	0.54794366	4.43332415	2.42326493	3.52810268	1.78620766	3.50388752	2.85352347	2.0781796	2.60021221
PROSER1	3.60260179	2.83233439	3.00419841	3.67286229	3.95009882	3.44167032	4.24819624	3.22590848	4.5406994
PROSER3	2.30590445	0.98964747	1.2554859	1.72334562	2.07461694	1.45487758	1.05181077	1.77426095	1.53153474
PROX1	0.21571731	1.55741343	0.48611057	0.79497579	0.33709075	1.21948438	1.05230362	1.29815785	2.99285968
PRPF18	2.39690621	2.38424534	1.99446709	2.65500259	2.84127178	2.69919317	2.49723053	2.24266654	2.58622347
PRPF19	6.26223581	4.60180239	4.97197641	4.99716141	5.08285818	5.13398843	5.48712334	5.26447182	5.48998277
PRPF3	2.74885901	2.18399012	3.13453508	3.13786532	3.38475922	2.52456894	2.87594943	2.78359796	2.98320152
PRPF31	4.36396318	3.28510273	3.48022983	3.52153235	4.05033951	3.81420671	3.55304719	3.15743356	3.77267529
PRPF38A	3.09591468	3.05513787	3.40504427	3.55617134	3.45194282	3.22546453	3.28108023	3.32760684	3.5812542
PRPF38B	3.19671646	2.9268266	3.72023931	3.80908905	4.04329256	3.25660584	3.04625619	3.46348574	3.7255726
PRPF39	1.52338802	1.75299424	2.59101306	3.08347702	3.04347464	2.15306431	1.71689518	2.20559371	3.05150954
PRPF4	3.51851972	3.21509521	4.02998216	3.88434649	3.92611378	3.6578769	3.42484195	2.9437193	3.89399614
PRPF6	5.57710147	4.61284848	5.07878017	4.33975087	4.71735596	4.67812003	5.04057656	5.18896485	5.28116406
PRPF8	4.27405927	5.11969529	4.80854777	5.00369085	5.49196393	5.36952959	5.36828633	4.55640221	5.78075656
PRPH	0.03310673	0.34019706	0.01803301	0.56945091	0.00514855	0.22459425	0	0.13248241	0.11011069
PRPS1	3.48742025	3.48362752	4.15913115	4.20912383	5.13902303	4.33093476	3.84107484	3.75936693	3.77139347
PRPS2	3.90998954	4.30309045	4.64468386	5.21349612	6.92619406	4.92184086	4.71958917	4.73054551	4.44170959
PRPSAP1	3.16555626	2.47900952	3.11157015	2.9318952	2.81228819	2.92520071	2.90576772	2.92286767	2.87385304
PRPSAP2	1.65558152	2.21234629	2.92675886	3.12942123	2.84798533	2.54520987	2.22645718	1.9496889	2.79452381
PRR11	3.8984524	1.95597917	3.26198342	2.65639797	3.60821192	2.73405072	2.86217479	2.95038435	3.06548953
PRR13	4.54098796	4.76646904	5.1850699	4.60669556	4.81420837	4.77847572	4.43383177	5.70799571	5.2167266
PRR14	3.60697865	3.35145137	3.33381957	3.30931213	3.12110227	3.16065752	3.17801405	2.90060039	3.31483379
PRR15L	5.69967569	4.8785219	6.2102057	5.74178786	6.04696879	5.53245205	5.72969515	6.45415429	7.00891634
PRR16	1.44460624	2.31895849	2.3911191	2.9039866	2.00402598	2.88035879	2.51972076	1.34561085	1.56372206
PRR22	1.65081635	0.15337691	0.28962473	0.59637296	1.40842134	0.24490883	0.85105333	0.57065833	1.12193692
PRR3	1.38130713	1.43123397	2.29927927	2.32309627	1.54925465	1.44439613	1.23008603	1.74291149	1.86867796
PRR5	2.9021726	1.9319318	1.67415727	1.28768339	1.29995443	1.75519206	1.40045804	1.17747948	2.68671116
PRR5L	0.93262816	1.55794263	1.56893388	1.26833874	1.47907034	1.57576032	1.45023076	0.80050154	2.10351356
PRRC1	2.7554108	3.31624236	3.68207109	3.70366412	3.37711367	3.4900718	4.1122545	3.77350339	3.81879865
PRRC2A	6.06332408	4.56648809	5.07651751	4.63481124	4.76149061	5.0207325	5.08065643	5.34243009	4.99382031
PRRC2C	4.19240827	3.85571412	3.91985136	4.26744098	5.01730817	4.17440278	4.66623769	4.92987976	4.34395946
PRRG1	0.80573928	2.86618751	2.01814725	2.20934945	0.72878552	2.31615442	2.1975978	2.21244365	1.64979078
PRRG2	3.50099515	1.6524577	3.04680109	1.38554725	1.85827565	1.52186717	1.91596686	3.13402254	2.62657234
PRRG3	0.7710963	0.64722583	0.38292852	0.73090302	0.08227858	0.46535995	0.08001303	0.06051448	0.01497296
PRRT2	0.20857191	0.43544881	0.45720447	0.58547329	1.14743265	0.19594411	0.13956629	0.24193042	0.35050799

PRRT3	0.69213284	0.79773692	0.46943318	0.51307606	0.35225912	0.8048535	0.48069617	0.5383763	0.61282633
PRRX1	1.63299844	4.22405181	1.85177797	4.43467612	1.89072402	3.58055345	2.76094875	1.65161872	1.33164711
PRRX2	1.31250065	2.87487666	1.62023571	2.41241713	0.90952126	0.87053962	1.81333394	1.92029435	0.88022923
PRSS1	0.24133786	0.00999683	0.34695576	6.66085006	2.57485337	0.18683901	0.13022705	1.23684298	0.18141727
PRSS16	2.28899444	0.93834987	2.45912578	1.4014404	2.0213056	2.01522721	2.33726778	3.28619667	2.27365106
PRSS21	0	0.42049954	0.364719	0.10708645	0.26037802	2.68404057	0.08610715	4.3955374	1.33278976
PRSS22	3.51001853	2.22264488	4.64034036	3.44018169	1.65004438	2.58509396	4.85013126	4.08517226	2.54863303
PRSS23	2.01513333	5.62222756	4.27752673	3.9507551	2.48106258	4.03598541	3.34274145	3.25093769	2.29553828
PRSS27	0.7433866	0.15369635	0.3286477	0.47942925	0.14422003	0.08773954	0.4916733	0.30275989	0.12096476
PRSS3	5.16232186	4.43357086	5.51221054	5.61524021	0.7018663	4.93372385	5.90462894	5.04235165	6.08031252
PRSS53	0.18620409	0.07396856	0.24988048	0.46776116	0.24697208	0.13025576	0.18928056	0.18207501	0.29501809
PRSS8	6.51949127	5.93243143	6.76070312	4.72177813	3.93065061	3.40043643	4.10239156	6.12230891	6.53938005
PRTFDC1	3.25833968	3.46630126	2.09611162	2.74987316	3.63298101	2.49234459	2.52912993	2.2854962	2.94041268
PRTG	0.08972711	0.53606381	0.26510897	0.51153307	0.05444898	0.43278111	0.30528412	0.06228264	0.02735762
PRUNE2	0.32581885	4.43632147	1.02666391	2.11120723	0.28161322	4.38204268	0.44260976	0.1731923	2.56815679
PRX	0.76997749	0.45030206	0.45445611	0.3867898	0.44244143	0.60850886	0.20424523	0.7958455	0.28538706
PSAP	9.20779621	9.00280936	9.9130651	8.87637654	9.65646199	9.33445054	9.15314456	8.61553578	8.11296127
PSAT1	4.85155034	1.45728424	3.28663053	4.82900411	6.78685589	5.28401864	5.56118956	2.81253055	4.58937496
PSD	0.77354455	2.56614001	0.43742552	0.50433919	0.22882225	2.12560428	0.31147475	0.24968965	0.2568183
PSD3	0.50093848	2.53900553	1.13177131	2.50598518	0.63829735	2.27775764	2.87123432	1.62413394	1.81981536
PSD4	2.53037373	2.06643352	2.90939721	2.30205809	2.41974913	2.28930855	2.28954331	2.30375967	2.63639323
PSEN1	3.23438824	3.47965124	3.53599963	3.70958601	3.43819813	3.66881157	3.63830082	3.34355456	3.44640694
PSEN2	2.82370459	1.67728304	2.61286752	1.95308998	2.61801498	2.19288213	2.27527794	3.05395796	2.28757069
PSENEN	5.33599258	3.82216548	4.08117186	4.06018991	4.2714316	3.96433324	3.43110816	4.25235956	4.16351405
PSIP1	2.94413657	3.55456348	2.64641176	3.89090369	3.65144947	3.58530569	3.19389248	3.6456906	3.34655004
PSKH1	3.19169745	3.13284124	3.19596393	2.82341747	2.77858547	3.50796039	3.3603712	3.10706093	3.29147403
PSMA1	4.17027819	4.45375553	4.91583861	5.1208758	5.95188931	5.0473859	4.95913033	4.48154347	5.35359819
PSMA2	2.86345425	2.4879936	2.93592239	2.83435473	3.3897682	2.42712541	2.36345986	2.10766368	2.58300861
PSMA3	4.65585632	4.85279498	5.03238755	4.98400208	5.91302219	5.13292885	4.62322944	4.44807681	5.15617928
PSMA4	4.14452615	3.89774776	4.58079015	4.36481535	5.76448212	4.51561317	3.90501834	3.24059302	4.38075285
PSMA5	4.36700523	4.06839826	4.54757613	4.05891705	5.37453183	4.72458568	4.29694039	4.55878865	4.78592479
PSMA6	1.96652025	1.96272711	1.85936273	2.07704086	2.67616703	2.23855902	1.88784584	1.89440385	2.19401311
PSMB1	6.13479966	5.79139601	6.49075326	5.84680721	6.42960822	6.15732898	6.40451016	6.2268721	6.15298335
PSMB10	5.25462647	3.58810471	3.79931538	2.62890509	4.90324262	4.76910242	3.7525069	3.62637662	4.92063743
PSMB2	5.63465574	4.69938228	5.62649946	5.25785288	5.69075888	5.44842784	4.81278634	4.21372188	5.6892928
PSMB3	8.12016611	6.3285782	6.93919559	6.12381293	7.21885275	6.64937123	6.51225824	6.6513255	6.6897157
PSMB4	6.39204961	5.89716818	6.38501863	6.12396401	6.41657763	6.44158922	6.53675321	6.37498128	6.57249514
PSMB5	5.84958768	5.38863068	5.75472891	5.47605516	5.43557385	5.72401548	5.73643632	5.37469378	5.93672132
PSMB6	5.85483768	5.53662064	5.84147061	5.60328318	5.81880256	5.95906118	5.87680117	5.00956505	5.7696175
PSMB7	6.48325966	5.69088517	6.32427701	6.03079581	6.37716447	6.07157041	5.94201709	5.27803327	6.06829894
PSMB8	5.93177324	5.75324194	6.10506781	4.7668292	6.47078283	6.61994664	3.94680142	6.01035764	6.74132802
PSMB9	4.97576862	4.50582515	4.97323426	3.64557978	6.05256577	5.54940933	3.24884162	3.92107086	5.83740333
PSMC1	2.6050976	2.71455251	2.28968115	1.97897598	2.36248148	2.64877554	2.75032072	3.24007102	2.78456678
PSMC2	4.54878661	4.7314902	5.26531504	4.60589322	5.13404395	4.87304011	5.09742163	4.72849193	4.75777435
PSMC3	5.72549149	4.99883674	5.36449938	4.95010852	5.24603719	5.51102702	5.75717825	5.27763001	5.09009388
PSMC4	6.7488647	4.82504697	6.15032246	5.31150411	6.33686635	5.52005343	5.40252882	5.59514214	5.5379026
PSMC5	5.1241405	4.09352359	4.74190892	4.42553825	4.44277388	4.56693665	4.11807445	5.07886188	4.175038
PSMC6	2.69293664	3.16352763	3.42426877	3.65080882	3.88474086	3.25265828	3.29305534	3.31682569	3.47616916
PSMD1	3.82466791	4.74423792	4.47745575	4.76588233	5.64584997	4.88649423	5.04318794	4.59976588	4.96429406
PSMD10	3.75405088	4.11514801	4.36311306	4.3576325	5.31841171	4.32392742	4.54329934	3.82428744	4.14055564
PSMD12	3.50719899	2.84997107	3.70988988	3.65487087	4.04146851	3.52398527	3.37876128	3.72122098	3.7671324
PSMD13	4.39374042	3.97608924	3.99621666	4.36528448	4.33248156	4.49220561	4.70763883	4.10113941	4.74981259
PSMD14	2.65153828	2.68069358	3.61186564	3.33119072	3.83999538	3.47879738	3.48074521	2.9971446	3.39953392
PSMD2	5.83380782	4.80926892	5.61208278	4.82154464	5.14245421	5.32778334	5.76396749	5.84196125	5.12932596
PSMD3	5.48365191	3.89625351	4.54057526	4.24687919	4.7586738	4.74554915	4.38973065	4.75143902	5.15837538
PSMD4	4.89684603	4.22543954	4.64921384	4.50588917	4.58474873	4.7548485	5.32214416	4.69464464	4.64256449
PSMD5	2.36442921	3.62208262	3.99456415	4.15857155	3.29683044	3.97726393	3.12960905	2.93002926	4.03190317
PSMD6	2.62650662	2.51686556	2.73496707	3.11716369	3.44566549	3.00123051	2.91659738	2.74257165	3.08600974
PSMD7	3.93106957	4.51095502	4.87062048	4.68572839	5.3129714	4.9603147	5.35545178	4.92250099	5.06518516

PSMD8	6.46097119	5.06207675	5.80808056	5.39320725	5.96085811	5.64188146	5.59667963	5.7231169	5.70840619
PSME2	5.83225601	6.1981436	5.87621347	5.18644405	7.1833768	6.16762414	4.76393071	5.66904227	6.31018194
PSME3	5.33287726	4.42964945	5.14988295	5.28491619	5.67968663	5.13037102	4.76376893	5.0834518	5.36001429
PSME4	2.54894125	3.05322362	3.38452328	3.57821985	3.99217403	3.53328709	3.76080561	3.38309439	3.11247562
PSMG1	2.27410342	3.08397332	2.911459	4.10268918	4.07759173	4.00384299	3.75105918	3.13825434	5.01667922
PSMG2	3.99337523	4.52018873	4.34843855	4.69018114	4.98061068	4.46721286	5.05757284	4.16464182	4.72881304
PSMG3	4.27835426	2.87174559	3.6820092	3.2251543	2.97163418	3.02525025	3.85429941	3.08594187	3.38647205
PSORS1C1	0.109235	0.65827532	0.4434989	1.65764843	0.11204524	0.44152535	0.30194378	0.55074275	0.97093674
PSORS1C2	0	0.23407841	0.51449423	0.41085817	0.03852734	0.03451607	0.14430125	0.12846563	0.20176529
PSPC1	4.09915593	2.60672033	3.34271666	3.64084253	3.933833	3.23803119	3.67117961	3.80069344	4.06364194
PSPH	3.64664364	2.40487914	3.99698351	2.46219845	2.81097336	2.5769658	3.45979041	2.91101351	2.31633842
PSRC1	2.3520757	0.90046911	2.64049881	1.77652852	2.18193997	1.96104133	1.59106228	1.35337462	2.01663205
PSTK	0.56861838	0.58631644	0.69907986	0.93106229	0.64353872	0.57092712	0.38842275	1.001875	0.73101045
PSTPIP2	1.75135816	2.52227153	4.17606346	2.91959497	2.98066537	2.80535862	0.93098318	2.41076441	1.95654194
PTAFR	3.30509648	3.0524924	3.57025032	3.28639015	4.47360057	3.97989561	2.96691661	2.28604392	2.28178274
PTBP1	6.21501433	4.85934319	5.44590024	5.25548107	5.94371663	5.72334889	5.78451479	6.04405687	6.05706555
PTBP2	0.81363706	1.49787043	1.50281506	1.62102172	1.30450017	1.45106154	0.57734323	0.83284747	0.98490488
PTBP3	4.4774036	4.42384909	5.45224071	5.31394236	5.50862166	5.43205024	5.01243271	4.42822389	5.30769842
PTCD1	1.38892742	0.77223645	1.25868205	1.0389829	0.8535445	0.88051462	1.0806062	1.08775274	1.17801873
PTCD2	0.44879857	1.03552413	1.23529714	1.12578257	1.15160325	1.09088709	0.77943687	0.84513224	1.03870899
PTCD3	1.89878178	2.62609145	3.00537048	3.22846979	3.67205884	2.64405941	3.15286012	2.6499055	3.60214818
PTCH1	0.67661775	2.83026498	1.64651425	0.96418105	0.2582674	1.01346009	1.1980255	1.1986011	1.87229378
PTCH2	0.33905942	1.48612701	0.59154895	0.29472013	0.41736476	0.40148601	0.14499448	0.18174582	0.15506192
PTCHD1	0.00975412	0.44815807	0.15885686	0.12506783	0.00150654	0.1330207	0.01602201	0.00846251	0.00513535
PTCHD4	0.00702266	1.723824	0.18585121	0.10583392	0.01724378	0.1820132	0.07392619	0.08175085	0.04732023
PTCRA	0.79523363	0.05264656	0.39232944	0.17601574	0.578199	0.21279808	1.07071533	0.07041076	0.21307983
PTDSS1	3.73637555	3.50734152	4.15647075	4.38834426	3.94582151	4.41100822	4.87897882	3.80670392	3.95986263
PTDSS2	2.63675126	1.8285096	1.96750312	1.79879392	1.78512725	2.05490076	2.38682513	1.72103104	2.12588162
PTEN	1.55837	3.61618029	2.60478562	3.40370908	2.99019141	3.02304836	2.57305997	3.38831048	3.21182027
PTER	2.15239143	2.92610455	2.61461233	3.37391373	4.02936944	2.85591085	2.82568886	2.6071929	3.28892358
PTGDR	0.11541892	0.36851119	0.29000221	0.8355439	0.42629356	0.45448118	0.25634007	0.16192039	0.14414063
PTGDS	4.42000856	6.76763191	4.48673528	5.09076362	4.2737872	4.6843109	1.85596087	2.05545444	1.07423889
PTGER1	0.77492159	2.7814706	1.06467526	0.76886953	0.09181489	1.82144491	0.21365844	0.57853075	0.09141052
PTGER2	0.75223971	2.76521384	3.35193765	2.39991775	1.83491296	3.08728418	2.52232149	7.61727974	0.99129423
PTGER3	0.34133923	1.83745505	0.74250519	1.24051176	0.1268327	1.88264299	0.50412846	0.36904317	0.10152352
PTGER4	1.45474875	3.25025709	2.35201185	2.46636478	3.47417008	3.25453315	3.4496879	1.76826447	2.19543165
PTGES	0.5522091	2.98030203	2.18342873	2.29068853	1.4411999	2.00560955	2.47768829	1.74277964	1.03598571
PTGES2	5.15804355	3.49993367	5.70837369	3.84854068	3.60448537	3.97604657	4.10655513	3.4252951	4.99351372
PTGES3	6.29192292	6.10019505	6.31075287	6.7266313	7.20576222	6.71495295	6.62384113	6.97639645	7.08422707
PTGFRN	4.85609363	4.94126652	3.5702174	4.51328436	4.03567811	4.73553275	4.70671592	2.40251813	4.62101926
PTGIR	0.76396604	1.45115294	1.52793993	1.19852465	0.49828907	1.4462279	0.77582319	0.54452241	0.50528106
PTGIS	1.02638397	5.19142757	2.32121836	3.73726308	0.24718968	3.48028567	1.54414675	0.99494785	0.42261223
PTGR1	3.08742653	3.66210375	4.16285429	3.78342871	1.46481077	3.82725209	2.39223761	2.67553331	3.78738854
PTGS1	1.78925533	3.55871959	3.5555837	2.69043544	1.93033251	3.36088604	3.46391006	2.03532786	1.88902963
PTGS2	0.56505173	3.28897961	4.03683787	3.83306384	2.8040361	3.44112328	3.94499481	3.38746378	1.55605681
PTH1R	0.25380554	1.06075897	0.40517543	0.74495019	0.03220022	0.53030126	0.17134329	0.13598973	0.04782002
PTHLH	0.30961476	0.71609125	1.02690178	1.09757531	0.29373297	0.94990548	0.84564132	0.37192732	0.62538414
PTK2	2.52681962	3.38925934	3.71327595	3.48309092	3.04261632	3.22782604	3.80544937	3.79346585	3.34417796
PTK2B	3.08462622	2.56677449	2.45700743	2.70685254	3.47285838	2.98583004	3.14251699	1.8344429	3.08583123
PTK6	2.22834638	2.76005217	3.10204834	3.70877879	1.60078429	2.20752915	4.58146283	4.09950431	3.33523174
PTK7	3.51482146	2.62893335	4.39079256	3.57492819	1.19097081	3.2062545	3.38234554	2.13494132	1.15045854
PTMA	9.06936138	7.80748132	7.81879466	8.27499777	8.97475104	8.25362699	8.35091737	8.05204536	8.84497381
PTMS	8.45102275	6.54612781	5.92864482	6.68753383	6.02472277	6.69193187	5.72144767	4.29967178	5.77368393
PTN	1.00201868	3.52034791	2.65179372	2.48789588	1.61459518	2.89473374	2.11076666	1.72331555	0.74363527
PTOV1	4.6393938	3.15694082	3.50692565	3.2977413	3.32194898	3.52716281	3.55665928	3.91974336	4.00061278
PTP4A2	5.21926903	4.95092501	5.54558162	5.31565942	5.35510832	5.59861747	5.56423821	4.53450886	5.41825479
PTP4A3	2.89966007	3.45727508	3.44489593	3.2522981	1.96322993	3.14503805	2.23919109	2.41919063	4.55317533
PTPDC1	0.75965369	1.09507061	1.37852519	1.72409893	1.1131169	1.49377296	1.08937443	0.76818947	0.83435424
PTPN1	4.02764655	3.75608477	4.88167488	4.30507166	4.49609082	4.21249524	4.30554031	3.90860796	4.26827326

PTPN11	3.75613755	4.6067789	4.01312778	4.70571328	4.91734912	4.91779609	4.60036972	5.17337852	4.55639351
PTPN12	4.08541319	4.03342936	4.71598984	4.86206919	4.57258596	4.22212689	4.20255567	4.35166869	3.44984608
PTPN13	0.29722828	1.70814142	0.68293969	1.4191273	0.62061995	1.11703883	0.72082418	0.73944133	0.33192326
PTPN14	2.21136642	2.11529828	1.77522314	1.63346094	1.77412572	2.12273897	2.20990942	2.16302706	0.81818054
PTPN18	3.88315719	3.16739825	4.56322117	3.65796681	3.73054459	4.00676263	3.97487206	3.80988199	3.62954811
PTPN2	2.2847099	2.79922187	3.24417165	3.34453453	3.77964016	3.15169481	3.36228968	2.72255721	2.93071087
PTPN22	1.27460398	1.97711034	2.6759252	2.00444197	3.96967678	2.39917009	1.85707593	1.08994989	1.70694484
PTPN23	4.45721725	3.4925521	3.87303797	3.63497857	3.75958656	3.65555503	3.96800056	4.01831631	3.99911667
PTPN3	1.90196286	1.62581009	2.67505526	2.46868801	2.64678337	2.13772317	2.04517834	2.24092441	3.23370979
PTPN4	1.10785481	1.69708448	1.75436659	1.71083774	2.0280604	1.53311941	1.45117027	1.59623709	1.63983751
PTPN7	1.7780005	1.22358777	2.46248993	1.32115147	2.64967794	2.26635862	1.57987672	0.93481936	1.57132
PTPN9	2.91022621	2.94238159	3.22258374	2.98988736	3.08561055	3.00310057	3.05856201	2.28991166	3.11959493
PTPRB	1.36101138	2.08739648	2.49565525	1.62598006	1.45759774	2.02007165	2.46638224	1.18573561	1.78616594
PTPRC	2.48051575	4.07845899	4.41750248	3.6978439	5.48044284	4.33936207	3.67416982	2.85391616	3.18098387
PTPRD	0.05185181	1.68203536	0.82913751	1.10150511	1.69863324	0.64667832	0.2413418	0.26421954	0.28972866
PTPRE	1.59306236	2.21282618	2.86877974	2.62290475	2.44419536	2.51449571	2.39763114	1.78474476	2.51642823
PTPRF	6.19224911	4.52284066	5.2108623	4.9278426	4.15804574	3.5945098	5.10321665	5.36507425	5.01676589
PTPRG	1.65028265	2.75619378	2.80897833	2.83001961	1.38999981	2.72238109	3.93577642	3.11121785	2.41740086
PTPRH	4.18830395	1.93408688	2.63938809	1.99133871	3.69061409	1.72112286	3.56603175	3.53149663	3.74264542
PTPRK	4.82070007	3.92918003	3.72838748	3.92929596	3.83835182	4.76609553	4.51461112	1.55726139	3.93429616
PTPRN2	0.61156835	3.45784456	5.57811421	2.22118346	1.65136522	1.76986291	1.85178913	1.13926808	1.77999679
PTPRO	0.40330369	1.1581067	1.66004772	0.81550344	1.46212185	0.86073459	1.10458906	0.39753032	3.87102525
PTPRR	1.143102	1.37761534	1.50695764	1.45830453	1.80980559	0.9210672	0.38823201	3.78541342	2.27499824
PTPRS	0.47088729	2.24999066	1.11226267	2.38924515	1.28970304	1.46561164	1.60675335	1.88685894	0.32769143
PTPRT	0.00842278	0.07694286	0.26669807	0.02013119	0.00519461	0.01338737	0.00504783	0.06990627	0.02930223
PTPRU	1.58128219	2.15228558	3.14207769	3.30846354	0.92876701	2.65995144	4.04159912	1.57893388	0.51323697
PTPRZ1	0.03628972	0.84062991	3.96409991	0.41815836	1.09606673	1.07598063	0.03692139	0.18829151	0.00385827
PTRH1	0.02013421	0.04572258	0.14614703	0.04785475	0.01861514	0.01113139	0.00605575	0.00779344	0.03162332
PTRH2	2.8064606	2.14273781	3.13130911	2.66587863	2.95607262	2.42614501	2.04266912	2.96746763	2.87016526
PTRHD1	4.02089903	2.83982392	3.41585915	3.10925131	3.04287313	3.35588371	3.19479186	3.02070227	3.61132192
PTS	1.94448585	2.26444708	2.46743336	3.16123633	2.68133511	2.40594965	2.61834462	2.13748802	2.31717371
PTTG1	4.82118004	2.79888731	3.9905987	4.35172293	4.91941074	3.98656481	4.32611628	3.4133429	4.38688312
PTTG1IP	5.57944202	6.09245455	6.46291898	6.31894495	5.38542859	6.39664178	6.82198704	6.98774899	6.14225706
PTTG2	0.1018066	0.11604799	0.11001007	0.03130105	0.31931254	0.23219424	0	0	0.02746033
PTX3	0.27303136	1.74394242	0.94960741	2.16236651	0.28284177	1.95968701	0.40858091	0.33453951	0.32567392
PUF60	4.90000859	3.63311836	4.43672592	4.20204709	3.93923163	4.75454742	4.89248318	4.6765205	4.58009241
PUM1	4.25176668	4.57294049	4.62918495	4.92266497	4.72138009	4.58051069	4.57719594	4.93581906	5.13725718
PUM2	3.82553236	4.46118485	4.47475218	4.72939005	4.79749029	4.78061382	4.67822672	4.77861965	4.79318616
PURA	1.93339419	2.74724335	1.98154561	2.26486098	1.93597902	2.40515976	1.82238046	3.29793184	1.95288094
PURB	2.94521799	3.30485157	3.55487872	3.57888547	3.19311024	3.11241542	3.06752302	3.19421746	2.98329891
PUS1	2.3019295	1.09319358	1.72195959	1.645467	1.91221239	1.99199303	1.79902558	1.9595168	2.26417157
PUS10	0.83658469	1.27857724	1.74912174	1.1142157	1.6664709	1.02918689	0.80659164	0.73489576	1.31057426
PUS3	2.73729253	2.87339525	2.54586887	2.98035327	3.87890881	2.94579704	3.21578217	2.39580019	3.42902632
PUS7	2.70481712	1.78857819	3.06572934	3.30335542	2.55725913	2.47727156	2.86663711	2.36371257	3.09960459
PUS7L	0.82395417	1.67239847	1.83031844	1.95791084	2.07747157	1.85880835	1.77108816	1.27863098	2.1592929
PUSL1	3.26859813	1.57929072	1.6779617	1.65231835	1.43331353	2.37407973	2.59807483	1.58443781	2.56789164
PVR	4.18985084	2.85309046	4.06660775	4.09512333	5.29621107	3.80906229	3.69397687	3.73528323	4.11968691
PVRIG	0.1891097	0.17795507	0.10244487	0.10620183	0.20042183	0.18354426	0.10597145	0.39942694	0.22319985
PWP1	3.32997369	3.94904299	4.34954534	4.51303193	4.57598166	4.48731318	4.30261264	4.63261913	4.41846427
PWWP2B	3.37791804	2.53958049	2.77502647	2.11347592	1.69709599	2.40776028	2.58283748	3.32747951	2.72135107
PXDC1	3.51028828	3.21441652	4.7684309	3.02881381	2.20300317	3.23554893	2.40182652	4.39313468	2.01166686
PXDN	2.24857088	3.70079283	3.26583778	3.42612437	2.62762208	3.89611463	3.48211869	2.82505703	2.5143975
PXK	1.69442082	3.25403697	2.61272695	2.64671373	3.02841534	3.4481737	2.23784053	1.96367513	2.26884353
PXMP2	4.16917634	3.30139686	2.52534007	3.10063411	3.05028967	2.98221208	3.98181774	3.78077306	3.78732394
PXMP4	1.49723471	1.9976883	2.34718264	1.81929023	1.34586156	1.50381226	1.41537137	1.90463781	2.2715238
PXYLP1	0.24207508	2.2698293	1.36373706	2.19550809	1.1987383	1.88989188	2.376932	1.69142502	2.51091646
PYCR2	4.3429421	2.9306111	3.86231004	3.49506237	3.42643783	3.40651403	3.93060448	4.26361392	3.56401557
PYGB	5.1127571	4.9307041	4.94634144	6.65942894	4.80803355	5.74899053	6.6594954	7.17448484	4.43812897
PYGL	1.62031932	3.18115436	2.41893065	4.50468041	2.12656109	3.44302597	2.3568234	1.43972522	0.99932903

PYGM	0.10577763	2.10534423	0.34195813	0.64423274	0.20550818	0.90033055	0.14568591	0.16251532	0.08947092
PYGO1	0.12192227	1.53315709	0.70926507	1.45635776	0.24673155	1.01941959	0.45921144	0.24861472	0.14211408
PYGO2	3.91040746	3.17952488	3.79190396	3.70223383	3.36920151	3.80149842	3.62030732	3.74990252	3.66655754
PYHIN1	0.65410093	1.17912858	1.41018818	0.99512927	3.01135369	1.37151351	0.67399411	0.40559917	0.80262147
PYROXD2	0.7702161	0.80292617	0.71118131	1.46249434	0.74163791	0.82804472	0.73533252	0.94978856	0.42217492
PYURF	2.17843362	2.24237816	1.65945244	2.03020717	2.22356627	1.98790561	1.934383	1.56567068	1.99474881
PYY	0.17420272	0.37189741	0.43875228	0.12410554	0.09205339	0.0446555	0.05437999	0.19961912	0.79145853
QARS	5.34331289	4.85133035	5.13802945	5.08855362	5.04385467	5.01233072	5.87649654	5.31092558	6.15497504
QKI	1.36876495	3.4494695	3.14561617	3.25340083	2.69542702	3.37448	2.47297338	2.15376835	1.322824
QPCT	1.11528251	2.07846391	1.70848005	3.17284445	1.56462864	3.44950311	1.47227346	1.48834411	3.09267833
QPCTL	3.25080674	1.61038028	2.89609555	2.09433304	2.09543234	1.58070307	2.33680858	2.59233229	2.60978589
QPRT	0.64324116	4.0735308	1.46410183	1.43626033	0.5516474	1.07803398	0.94242676	0.61289491	3.94840523
QRICH2	0.58492127	0.16944547	0.48680029	0.3186729	0.53379638	0.38908337	0.12989877	0.89251937	0.19353773
QSER1	2.54843698	2.85243354	2.68869636	3.01299345	3.3926621	3.21866486	1.32753898	3.14107402	3.0481017
QSOX1	4.44670644	5.05925246	5.3818345	4.90225841	3.80701802	4.86854014	6.82956021	6.09244913	4.98217891
QSOX2	2.97846097	1.76911748	3.21463651	2.800194	2.86731715	2.8147961	2.83501818	2.5991924	2.77872835
QTRT1	3.07951686	2.28596964	2.75301311	2.26038339	2.70925121	2.39487434	2.06151999	2.90750863	3.96620679
R3HCC1L	1.87179373	1.97439563	2.18163313	2.40465664	2.93550955	2.25285025	1.99165712	2.7207323	2.37695279
R3HDM1	2.4620208	2.57873076	3.37727099	3.11636242	3.43254381	2.85417739	3.43794851	2.65555446	3.01160393
R3HDM2	2.49438271	2.39117451	2.99931984	2.75051713	2.71789851	2.15047102	1.77985377	2.19661609	3.11336571
R3HDM4	6.03247818	4.15611543	5.13974227	4.73926416	4.79119043	4.80091389	4.90546011	4.60749742	4.97705976
RAB10	5.48596357	5.78819204	5.57012041	6.01299035	6.103069	5.85573339	5.87511131	5.79303698	5.99062766
RAB11A	5.15282945	5.17229961	5.27100102	5.70604858	5.9898946	5.10327511	5.10701279	4.68873277	5.63820931
RAB11B	6.51622172	5.30486658	5.82371336	5.53070874	5.13126341	5.68262503	5.23752673	5.79359639	5.70627977
RAB11FIP1	2.96314068	3.85612112	3.14690678	4.31056445	4.33842746	3.49585397	5.03067943	4.5120431	4.74605097
RAB11FIP2	2.47660591	2.62244606	3.66279148	2.83582482	2.91239212	2.69792808	1.93964274	2.52529629	2.0309086
RAB11FIP3	3.63890249	2.86683721	1.93567466	2.28319676	1.82070621	2.45571551	2.13253528	1.46555756	2.93680125
RAB11FIP5	2.90168696	2.69188655	2.72705445	2.60509936	1.27770763	3.31515257	3.31731311	3.61467167	2.2866558
RAB12	3.35562939	3.09472198	4.32177137	3.92459854	2.88314852	3.73448486	3.76775261	2.77004655	2.80431837
RAB13	4.79246996	4.69723339	4.44437741	4.3777612	3.58597889	4.68278686	4.69062625	5.16648717	3.46020987
RAB14	4.14314691	4.76605783	5.35155708	5.0967876	5.13415915	5.12723474	4.9164834	4.21016889	4.98460082
RAB15	2.71268092	3.1356052	3.69801327	3.32324022	1.00413456	2.66335242	3.31881863	2.80159623	5.45499105
RAB17	2.66800707	1.43790961	2.08261384	1.94435828	1.56496869	1.07192865	1.20917498	2.56313738	2.78298275
RAB18	3.15374315	4.07536572	3.74917847	4.44698863	4.94926644	4.28048857	3.72602026	3.34269114	3.91953174
RAB1A	5.49214115	5.95312501	6.37666995	6.01064688	6.24272703	6.11981282	6.42147515	6.38307957	5.92942367
RAB1B	7.55015579	5.91045601	6.45625544	5.75338403	5.70167946	6.41468554	5.75905347	6.67991469	6.34117766
RAB20	5.96594132	3.983913	5.1208855	3.80110036	4.37825713	4.30131852	4.1670949	4.0457343	5.40066848
RAB21	2.56052522	2.36831762	2.93802226	2.78924076	2.48717224	2.67695544	2.34599161	3.02817551	2.34193788
RAB22A	2.12344997	2.82647832	3.36171493	3.53383828	2.93115032	2.96801624	3.36312858	2.95457185	3.14564991
RAB23	1.11377454	3.39550426	1.53821534	2.67590859	1.98786913	3.23380315	2.01731888	1.3801548	1.25756997
RAB25	6.75361942	5.0156941	6.18308561	5.92119353	5.72391377	5.4750996	7.11515929	7.05447114	7.07292981
RAB26	1.21409081	1.00860283	0.51142453	0.40365503	0.23283092	0.2284894	0.48660256	1.33249184	0.70466664
RAB27A	2.45653541	3.37163813	3.81422957	3.20012968	4.15475679	3.97680225	2.89842341	2.40627163	2.35139836
RAB28	2.88378333	2.75687159	2.97328688	3.36375584	3.1814117	3.30397939	2.71126973	2.64527489	2.8054101
RAB29	3.93208183	3.30160295	3.72503312	3.11760613	3.73238884	3.64143367	3.42418613	3.67813052	2.21713948
RAB2A	4.38019208	4.78542543	4.90376337	5.23863046	4.80983946	5.22057802	5.37719259	4.92265699	4.67174383
RAB2B	2.587451	2.46711141	3.1167587	3.03564306	2.97506846	2.63915129	1.9363972	2.50161967	2.73729527
RAB30	0.56388796	2.47676474	1.3532223	1.67315034	1.03533407	1.56194724	1.23868826	1.19708004	1.51239879
RAB31	3.27812731	5.48102519	5.16277121	5.45930115	4.220379	5.47974146	4.95230497	3.57529577	3.04691491
RAB32	4.4086058	4.35247156	4.75348651	3.72431055	4.2637342	5.23151573	3.62579275	4.1467069	2.07306222
RAB33A	0.62497419	0.98382009	1.73171586	0.70175005	1.74966291	0.74106819	0.80345215	0.41446159	0.49738712
RAB34	2.55041401	4.19277359	4.48885397	2.97089033	1.80228665	3.66006249	2.68885932	4.69649288	1.4004629
RAB35	3.93026889	3.58473681	4.05985376	3.77877534	3.82566637	4.2121186	3.70015585	4.81550932	4.02320025
RAB36	0.28857378	1.11045768	0.64146957	1.45512576	0.24108653	1.35579714	0.39283035	0.84712949	0.38284983
RAB37	0.44725355	1.01908825	0.94440997	0.47952112	1.17276386	1.35738252	0.26622568	0.40103192	0.8920014
RAB38	3.03091775	3.2664374	2.34657396	2.32893264	1.95954223	2.68189505	2.3788106	2.08290529	0.43668322
RAB3A	1.06883012	1.43045497	1.50442234	1.87487866	0.90545349	1.62911854	1.88556539	2.09318109	2.45922317
RAB3D	4.34153868	2.39201851	3.35832233	2.75945289	2.56179091	2.29961068	2.64267313	3.08429837	3.86880367
RAB3GAP1	3.29096698	3.61293692	3.44301686	3.43467093	3.60664033	3.58745405	3.47498494	3.4782837	3.30622239

RAB3IL1	1.18737031	3.00503283	2.02793307	2.45741618	1.03629099	2.97583592	2.14921435	1.44526935	1.24987458
RAB3IP	1.64393628	0.93170058	2.03804699	2.02345112	1.61093253	1.80826593	1.63980581	2.55765138	2.29363582
RAB40AL	0.06245498	0.07132694	0.03041857	0.01270823	0.02597798	0	0	0	0.01114008
RAB40C	3.66746184	3.26338216	2.34178118	2.71572969	1.95666426	2.80456865	3.33036029	3.28107589	3.79574285
RAB43	2.62355888	0.35097654	0.65117501	0.32428279	0.64558333	0.82216186	0.88623695	1.25166215	0.64526228
RAB4A	3.44000006	3.48753183	3.73647827	3.841784	3.38740838	3.27817386	3.8313721	4.43918842	3.91771036
RAB4B	2.48888942	1.54706125	2.41280361	1.77133857	2.4115482	1.80110594	1.21253537	1.23283349	1.62181099
RAB5A	3.95757442	4.17212316	4.57974443	4.59025729	4.39109814	4.41375384	4.76471303	4.74945306	4.29229636
RAB5B	4.32330865	4.91988513	4.74622944	4.83873012	4.54572528	4.9760318	4.720481	5.57904265	4.92230938
RAB5C	6.68519651	5.34618418	5.70750116	5.2194289	5.65707095	5.83177486	5.43624058	5.3957519	5.50169878
RAB6A	4.49847555	4.88622342	4.80751937	5.39234608	5.04642199	5.24193756	5.64877055	4.73398679	5.12364248
RAB6B	0.28041696	1.90480395	0.50529664	1.81199009	0.2656069	1.13103863	1.39190056	0.54963355	0.41325402
RAB7A	8.02548607	6.72750217	6.9246517	7.14019085	7.19753976	7.0349148	7.00547084	6.93729618	6.77357019
RAB7B	1.06329205	1.70191017	1.85371923	1.72359376	1.24362646	1.50626365	1.471073	1.25664872	0.53993221
RAB8A	3.6245634	3.63851629	3.56550983	3.91442701	4.68857102	3.8057843	3.66199574	3.41887885	3.89501826
RAB8B	1.88414661	3.99055898	3.50241581	3.67656507	4.25867481	3.45441222	2.85147773	2.62693606	2.76697455
RAB9A	3.57211077	4.40501217	4.26616477	3.73252847	4.76452389	3.88906759	3.66195332	3.5883105	3.46404656
RAB9B	0.2246594	1.74963981	0.92664112	0.59794033	1.18831666	1.20791873	0.09024937	0.45025618	0.5395125
RABAC1	5.66253253	5.4423839	4.96692397	5.06098318	4.1050473	4.9160808	5.08155326	5.18024411	4.6764293
RABEP1	2.07130979	3.57537835	3.24719351	3.62536739	3.87342391	3.74994238	3.09639746	2.860234	3.10261561
RABEPK	3.02862846	2.5037428	3.59662733	2.66316344	2.53263932	2.79803712	2.77918616	1.1669187	2.94773781
RABGEF1	1.05229385	0.79538451	0.72787296	0.75003037	0.69123451	0.7574143	0.91982194	0.60318466	0.57679778
RABGGTA	2.98538393	2.4546465	3.171642	2.96936575	3.14697798	3.0030509	2.99228168	3.43765972	3.5069182
RABGGTB	2.1736957	2.57716947	3.41633506	4.04254553	4.06669839	2.86961251	2.65216845	3.71159325	3.73718141
RABIF	3.06891032	2.94467531	3.57717043	3.27993055	3.05881182	3.22020309	3.44800099	3.12449045	3.05128742
RABL3	2.69587928	2.62775055	2.53225543	2.86210287	2.84259594	2.91744577	2.57267965	2.22241151	3.01107409
RABL6	4.16402795	2.26971768	3.40450528	2.69132373	2.7957154	2.81903241	2.38592881	2.96441913	3.26085064
RAC2	4.39668795	4.63869646	4.90226911	4.18230727	5.47543817	5.57741577	4.30835251	4.44697827	4.79092112
RACGAP1	3.3198273	2.81163328	3.55711621	3.70467959	4.36889822	3.70706495	3.86307917	3.60867755	3.75775188
RAD18	1.13818815	1.55355556	1.38258566	2.33689455	2.52446599	2.06788414	2.39078218	1.93434865	2.55005097
RAD23A	6.2174768	4.84055021	5.4147531	5.04065924	5.5590882	5.34589367	4.98980452	5.59994495	5.6277716
RAD23B	5.55347557	5.0786606	5.28511271	5.36285059	5.44959784	5.45049003	5.12110207	4.62351515	5.50449121
RAD51	2.66281203	1.41543962	2.58857219	2.56825022	2.98880397	2.6786718	2.96280392	1.08321714	2.50748327
RAD51AP1	2.06348065	1.93245672	3.01138515	2.43038075	3.84947292	2.74892516	3.01261279	2.03769648	2.99672379
RAD51D	1.39948766	0.99703432	1.10727838	1.04395991	1.45063129	1.06209363	0.98493624	1.66119155	0.9980489
RAD52	0.71288573	0.91121274	1.24614338	1.7951529	1.36237907	1.36333884	0.83151201	0.94995326	1.77850054
RAD54L	2.49776412	1.00655517	2.40324668	1.5438535	2.6840245	1.81075705	2.0063957	1.58962814	2.54108782
RAD9A	2.345978	0.97953686	2.02303928	2.09608661	2.10052502	1.61324443	1.34365495	1.78360342	2.38498488
RADIL	0.03863021	0.33575663	0.24471556	0.14361273	0.02052701	0.3791323	0.06545432	0.03408553	0.02621999
RAE1	2.65297862	2.09994354	3.62600163	3.55620355	3.15328305	2.4118151	2.44352515	2.50574968	3.46139818
RAF1	3.29395331	3.46570947	3.83784105	3.8121691	3.78012236	3.9106468	3.93499736	3.67748761	3.70896572
RAI1	3.02010583	2.29869971	2.29869673	2.13930812	2.38917456	2.41701988	2.47713386	2.45812541	2.83341427
RAI14	2.71667279	3.67176849	3.45540679	3.9815858	3.46062693	3.77165142	2.91586878	2.85660356	2.13507751
RALA	4.09661822	4.37218979	3.69224936	4.48730503	4.99834333	4.20290534	4.54436641	4.08435308	4.46578208
RALB	5.00189324	4.81799986	4.85449697	4.8214779	5.53440192	5.02507607	4.84462864	4.66880652	4.27889504
RALBP1	4.29688504	3.75824607	4.66621063	3.92340715	3.90021541	4.46625722	5.34268753	4.48787241	4.01875301
RALGAPA1	0.83406715	1.6658192	1.28474301	1.35397358	1.47433236	1.76376294	1.43402125	1.93937746	1.58305167
RALGAPA2	3.8105381	3.28086162	3.78197415	3.18808457	3.19196031	3.22684965	3.48669118	4.28898185	4.4458556
RALGAPB	2.14661029	3.01500567	3.17186699	3.44050401	3.28851521	2.81573346	3.03083048	3.19497003	3.43745475
RALGDS	3.69321233	2.74242016	3.58175665	3.45567767	3.27398933	3.07094343	2.97065716	2.78954227	3.78909517
RALGPS1	1.38585557	1.6194767	3.06643898	1.42713372	2.15893884	1.19462877	1.31590579	1.16341184	2.66148002
RALGPS2	1.37345307	1.75864224	2.34890181	2.70860933	2.89758872	2.16866488	2.99357852	3.49210145	2.17030806
RALY	5.10822676	3.83218515	4.57982686	3.74136653	3.52042147	4.16501789	4.09694408	4.09083682	4.99474266
RAMP1	1.10191835	4.97993233	5.01325055	2.656679	0.38147236	4.52408759	3.70959361	4.12913261	5.71926135
RAMP2	3.91276342	5.01557002	5.16765081	4.81607479	3.58586285	4.70532335	3.36636773	2.87154066	2.88229419
RAMP3	2.6374457	3.75083734	3.74065362	2.65530388	2.59416513	3.58795303	2.39795674	1.8532752	1.78934066
RAN	5.68818301	5.32693906	5.71525581	6.09086046	6.43189567	6.18440197	5.98374781	6.11175305	6.42208535
RANBP1	5.58095948	4.0920383	4.11861584	4.38094851	4.98903119	4.3074594	4.34153094	3.29045673	5.10927744
RANBP2	2.35419537	3.54946334	3.43715973	3.71967671	4.16367706	3.83093761	3.77845943	3.50055765	4.06741364

RANBP3	3.53310627	2.83861842	3.11201652	2.88440432	3.58787677	3.02522996	2.69825482	3.3294687	3.38704689
RANBP3L	0.01079654	0.37982843	0.14157791	0.47724197	0.03951051	0.27142168	0.09416563	0.08908206	0
RANBP9	3.62838204	4.54889033	4.55649773	4.91529373	4.45531333	4.70443165	4.68471168	4.94946667	4.2433688
RANGAP1	5.12871329	3.10890338	3.49553932	3.62576894	3.61365736	3.93963416	3.7400762	3.2335105	4.0615027
RANGRF	2.34164063	2.27089268	2.74783154	2.22083702	2.1422282	2.35779616	1.83198124	1.57022016	2.7186415
RAP1A	3.71312941	4.8271112	4.50219764	4.40615611	4.35224741	4.48935443	3.71524341	3.44812529	3.68723096
RAP1B	2.0240121	2.82848603	3.19553462	2.71128735	2.73695609	2.9993929	2.93807519	3.00356948	2.33754035
RAP1GAP	4.62304673	1.42740257	2.37110904	2.75860408	0.88471142	1.41207469	2.24540342	3.09972339	1.79827364
RAP1GDS1	2.18848053	2.64018828	2.63350914	3.4065061	3.30828336	3.01179669	3.25980092	3.12678872	3.25937754
RAP2A	3.52687931	3.44251062	3.55585795	3.62221309	3.66624361	3.49589436	3.49723744	2.69209358	3.30661934
RAP2B	4.62166946	3.06075952	3.82597321	3.67593621	4.25557845	3.82218983	4.31217624	3.64996082	2.57477379
RAP2C	3.83169217	2.77546941	4.52050336	3.53003648	4.57993046	3.55972073	3.18714293	3.00780215	3.05875243
RAPGEF1	4.62219215	3.68294639	4.7601559	3.28011391	4.15418803	4.01098099	3.902198	3.80174563	3.62908035
RAPGEF3	1.2230567	0.58686314	0.64768221	0.52902856	0.52802131	0.72661065	0.50403293	0.8758939	0.25386128
RAPGEF4	0.23966602	0.64922197	1.57355057	0.79894752	1.09580406	0.77363568	0.54835267	0.42964091	0.51251631
RAPGEF6	0.63098855	0.95520948	0.97550952	1.19753984	1.40337405	0.94891629	0.81331476	0.98640272	1.19569495
RAPGEFL1	2.10872026	2.34461172	1.22391818	3.71427434	2.08961393	1.9936057	1.35590591	2.45246713	3.59969332
RAPH1	1.06095818	0.77822589	1.12714152	0.93424005	1.67652867	1.12692166	0.9976497	1.87228685	0.94074294
RARA	4.05378015	3.36299511	2.98491796	2.87888408	2.35661193	3.22039205	2.30108537	2.59111001	2.85248921
RARB	1.00812031	1.15891579	1.36444797	1.46183931	0.93934608	1.42741387	0.96572032	2.20084307	0.30271119
RARRES1	2.34118381	6.16206211	6.68905247	3.25917353	1.55328656	4.86779909	3.4427863	2.74694312	2.38795655
RARRES2	5.11813189	5.75926837	6.42916089	5.83847942	2.97186069	5.19852916	4.66556934	3.64211961	3.16841758
RARRES3	5.14891891	5.88650227	6.13733478	4.44567733	9.0595635	5.72392332	3.87332444	3.51613192	5.48844007
RARS	3.69628114	4.29108424	4.56875822	4.94621293	5.42142024	4.75605963	4.62988487	4.66978326	4.92074828
RARS2	3.29342646	3.52729033	3.61151328	4.0636458	3.82491095	3.52811041	4.22638297	2.70412796	3.95024915
RASA1	2.59242116	2.84645196	2.6998884	3.64688362	3.55491196	3.2483725	3.14519533	3.4415862	3.29860597
RASA2	1.81930968	2.12143901	2.72653185	2.46512239	2.81345298	2.43916142	2.13462941	2.55271458	2.95193215
RASA3	1.69754381	3.52423017	2.28960701	3.26461441	2.40771906	3.01360245	3.41471013	3.42085902	2.83343261
RASA4	0.06614319	0.17502951	0.19200932	0.13431859	0.06787081	0.08216702	0.02676051	0.05627782	0.14002907
RASAL1	0.99800939	1.1509532	2.47060245	1.54172046	0.86751296	1.34640477	1.90050014	3.43121414	0.15460156
RASAL2	1.05776255	1.87845195	2.20101543	2.41599488	2.23755849	2.40602461	2.27888519	2.15202266	2.44936445
RASD1	0.44497938	2.44500334	1.67472388	2.37753716	0.70673632	1.87404031	1.73050853	3.74643233	1.31109448
RASD2	0.5004065	2.1448268	2.02513544	1.18576384	0.61266599	1.6648537	0.94581569	0.60616049	0.78564028
RASEF	2.42062477	1.75737233	4.37384782	3.34140883	2.89915964	2.31368204	3.02847967	4.40572837	2.48882987
RASGRF2	0.45231385	1.84855348	1.12890209	1.49703408	0.9027156	1.43011574	0.88910963	0.59838487	2.17434314
RASGRP1	2.84434732	1.09865384	2.90201206	1.75549587	2.95727517	1.84928191	0.90155012	0.91798904	1.25932642
RASGRP2	0.44491896	1.01966472	1.11963009	0.66031592	0.74880783	1.87029126	0.2912446	0.33256753	0.31296783
RASGRP3	0.74205392	1.68136619	2.08698994	1.45459176	2.0799077	1.69630793	1.4147564	1.51205978	0.84815509
RASGRP4	0.45882995	1.54590085	1.37192137	1.09360529	0.69715293	1.28692679	0.71357832	0.66165943	0.42868825
RASIP1	1.52371139	2.18154354	1.91167204	1.55207611	1.08253643	2.62159001	1.27585857	2.76325352	0.83260644
RASL10B	0.89406731	0.62798951	0.40393562	0.39132863	0.15774192	0.31786897	0.23516347	0.11927272	0.27326876
RASL11A	0.69796052	3.41030617	2.21098078	1.86806187	1.7139967	2.551827	1.03060716	1.86311883	4.216893
RASL12	1.32515951	3.31835385	2.22653613	1.98699762	1.25274665	2.48315203	1.48394846	1.20571913	1.24749705
RASSF1	2.34708965	1.9820546	2.78905312	2.60945342	2.5724038	3.04887908	3.12280041	2.8973136	2.82713704
RASSF2	1.78377842	3.85898429	3.46461525	2.96352816	2.91549675	3.56817658	2.0032905	2.20196424	1.7199136
RASSF3	3.88887305	4.54373439	5.34584563	4.4170647	4.01951388	4.52877464	3.99441225	5.48514747	3.94421231
RASSF4	2.53174362	2.06791658	2.94067799	2.27254281	3.30596143	2.64595347	1.92567848	1.39829961	2.40207972
RASSF5	2.06137888	2.72476092	3.15190967	2.5002735	3.32592361	3.14440264	2.69935689	3.76241865	2.04371596
RASSF6	2.77224027	1.04442955	3.18000202	2.29709409	3.20292381	2.14830438	1.74352913	3.62091726	2.68614988
RASSF7	4.8898397	1.66258748	3.34093864	3.10267842	3.09066231	2.7590327	3.77489136	4.10094069	4.30889144
RAVER1	4.31409991	2.59459733	2.71816429	2.65476079	2.98215525	3.3043986	3.33173844	3.26372512	3.80693452
RAVER2	2.20811399	2.83270302	3.53513059	2.78661102	4.26826195	2.51611077	2.32793402	3.03936206	3.66341763
RAX2	0	0.01719262	0.05353074	0.00902942	0.00927076	0	0	0.00580711	0
RB1	4.21118937	4.56970034	4.67436873	4.27990929	4.88232473	4.3839434	4.41453052	4.31617062	4.63137411
RB1CC1	2.10348397	3.26192562	3.12823389	3.39471666	2.99028886	3.3143715	3.4665059	3.18258629	3.0108443
RBAK	1.75279195	2.1283427	2.44969749	2.6840364	2.36831201	2.02546798	2.06227728	2.25103828	2.50101346
RBBP4	4.00068969	3.62730464	4.23956998	4.37274962	4.42121808	4.23288235	3.95618808	3.15316019	4.79210713
RBBP7	4.17635225	4.25173185	4.1812672	3.82489117	5.07861075	3.96364978	3.93386326	3.93933233	4.07674731
RBBP8	3.27996663	2.72973487	3.58735658	2.86599877	4.74512844	3.2855782	4.00187655	2.84918974	2.72699153

RBBP8NL	1.50389063	1.33225848	1.51717997	1.06024008	0.60763432	0.00955704	0.44077804	1.84542037	3.49125219
RBBP9	2.54152432	3.30042822	3.44663971	3.57821119	3.10193096	3.05284268	3.19259125	2.87421555	3.61423519
RBCK1	5.02356282	4.29844484	4.41128953	4.25160506	4.45179286	4.76984158	4.4193984	3.3321331	5.53489284
RBFA	1.71986342	1.76992077	1.79549205	2.21549945	1.92493706	2.18779457	3.23233053	1.38337352	2.52048113
RBFOX2	3.39027265	4.1232004	3.09449893	3.56921886	3.51799217	3.78990272	3.2357479	2.5810826	3.42217007
RBFOX3	0	0.38387869	0.22202382	0.05646181	0.00906764	0.64876722	0.01100631	0.06264294	0.01735808
RBKS	1.50930012	1.63023728	0.84840623	0.98123852	0.85024001	1.10410468	0.90446551	0.89698658	1.3748558
RBL2	2.20146148	3.7602693	3.76774644	3.31058715	3.46369973	3.43402081	3.66591019	3.89758789	3.7726437
RBM10	4.59631495	3.26617388	3.41956559	3.33179219	3.99585246	3.64428466	3.50472245	4.04632616	4.16774739
RBM12	3.2048923	3.28804299	3.74869006	4.09009774	4.19626947	3.92108754	3.99285937	3.94537612	4.25856005
RBM12B	1.35158011	1.48967716	1.59664336	1.87808199	1.96830548	1.71406292	1.622137	1.70979259	1.59380475
RBM14	4.41072732	3.00160588	3.74020829	3.43926437	4.03355797	3.95058961	3.86455574	4.02006224	4.25357659
RBM15B	4.13876945	3.33986652	3.54453736	3.4191283	2.94787115	3.79602018	3.61989575	3.51009887	3.9584868
RBM17	3.55769089	3.08934346	2.81886319	3.4716702	3.63841368	3.39113489	3.01420328	2.2998231	3.73088825
RBM18	2.41399184	3.04200298	3.79133327	3.44775374	3.20901004	3.07584384	2.90230557	2.74571143	3.27592002
RBM19	2.48593551	1.84575025	2.23969807	2.03720697	2.26730847	2.56796926	1.8330653	2.64619558	2.60419192
RBM22	3.85876272	4.0048868	4.39526073	4.85535461	4.68475689	4.51492864	3.98830576	4.17231897	4.731468
RBM23	2.58351269	2.60217043	2.98368577	2.20242683	2.57152666	2.64259874	2.68359047	2.5481265	2.67842165
RBM25	2.69477878	2.9771676	3.18702199	3.75272317	4.19819556	3.29399024	3.05490818	3.03422566	3.61848269
RBM26	2.78970149	2.78952931	2.8015221	2.98097168	3.13087042	2.8411303	2.333325	2.57799873	3.46683678
RBM28	1.60312478	1.17190904	1.84232375	1.79901084	1.88726208	1.61040554	1.70857027	1.54361502	1.7846994
RBM3	5.54710514	5.14110313	6.12375246	5.71279496	5.95986356	5.55300472	5.5860228	4.89834816	5.81166417
RBM33	2.24886672	1.8834993	2.76190003	2.18954525	2.40519109	2.31921525	2.18554864	2.37484943	2.39478536
RBM38	4.56244601	4.09246951	3.67681493	3.47763576	3.4708121	4.31359639	4.22802613	3.13285587	4.92480527
RBM39	3.30268816	3.26851452	4.37604712	4.53101548	4.25529164	3.70754191	3.71164378	4.01291156	4.91425985
RBM4	1.58818095	1.32934292	1.62274826	1.7330501	1.74487882	1.55531805	1.66627243	1.8306065	1.81740239
RBM41	0.85061857	1.74942152	1.46724628	1.91925202	2.92868778	1.59034766	1.56828208	1.8168298	1.64850863
RBM42	5.89299545	4.40796338	4.6007566	4.24009828	4.77153882	4.8941959	4.4056119	5.00639943	4.69128224
RBM43	1.40571079	2.31931533	2.33213277	2.07376127	1.75083145	1.72944627	0.73431127	1.86393313	1.06698252
RBM45	1.17424634	1.17327238	1.67517747	1.57685803	1.89510931	1.30601849	0.97848911	1.36584682	1.72289324
RBM48	1.7165504	1.78376141	2.27014131	2.03149507	2.01009635	1.64129955	1.60202723	1.59869388	1.92030771
RBM4B	1.81929733	1.90693495	2.02274967	2.13951482	1.99382904	1.90984731	1.71507271	1.69796511	2.20780984
RBM5	2.31297667	2.41317489	3.07556079	3.29858328	3.40195036	2.66224907	2.49669009	2.79818287	3.1847351
RBM6	2.36885573	2.08472852	3.43197856	2.99492119	3.37063254	2.58846277	2.37154855	2.51774083	3.01390916
RBM8A	3.94732192	3.57189029	3.61772497	4.3505732	3.80518809	3.96085096	4.32127816	4.1903356	4.5799188
RBMS1	2.144903	3.23024839	2.65337344	2.48747728	2.41862218	3.13454614	2.24536571	2.40104359	0.84090999
RBMS2	2.60975187	3.22318599	2.70710944	3.08389128	3.45271714	3.1893775	2.68415062	3.05067007	2.82937408
RBMS3	0.28648749	3.25092501	1.43477123	1.76431381	0.74814313	1.92181828	0.88664155	0.63780573	0.32656434
RBMX	4.9057688	4.64796956	5.18622692	5.07093822	5.64406596	5.02809206	4.80293575	5.14176249	5.51113759
RBMX2	4.0093664	3.09724925	4.46727681	3.5387787	4.04218123	3.08010057	2.7708226	3.26730378	3.19170236
RBMXL1	2.42225801	2.50206408	2.47600745	3.11880873	3.69071331	3.36763266	3.01290984	2.86994281	2.48860919
RBP1	0.93029828	2.23774534	1.99002803	1.41204158	0.74287159	1.60715119	0.95103132	0.7807912	0.67668947
RBP2	0.042498	6.42920447	3.11200442	1.11492638	2.1155215	0.32497049	0.02558966	0.71830729	2.89584973
RBP4	0.60693208	4.22306984	1.84078283	0.90966078	0.17489272	1.08473232	7.55901223	0.6518178	0.48873283
RBP5	1.03689217	2.33878273	2.2668676	1.29944663	1.28233549	1.57518687	0.50563041	0.72622681	0.54188402
RBP7	1.23243917	3.50919577	1.74539646	2.5677286	1.03444992	1.66793537	0.97524103	0.71770868	0.43798426
RBPJ	2.9006513	3.8854462	3.58449871	4.0413732	4.25004689	4.00887551	3.43479317	3.46939669	3.97946009
RBPJL	0	0.01503226	0.67308903	0.29266132	0	0	0	0.10299485	0.04101157
RBPMS	3.86758112	4.10068365	3.07696899	3.53366302	2.89806123	3.71163507	2.82999092	1.495442	1.2372192
RBPMS2	0.91819638	4.10074551	1.77550304	2.41169375	0.78734529	3.80249262	0.80019463	0.72948811	0.59638839
RBSN	1.8374151	2.3743837	2.12411387	1.88669504	2.13068976	2.24677448	2.00999185	2.01163801	1.85605379
RBX1	4.71981828	4.1923866	4.15464964	4.16393432	4.45798298	4.06071193	3.70441059	2.99601654	4.10531111
RC3H2	2.14975357	2.77939906	3.2641759	2.96131342	3.35410056	2.97736691	2.37612597	2.21539146	2.78376577
RCAN1	2.82716558	4.25086414	3.54550288	3.7677878	3.43391459	4.35012237	3.11010623	2.3471443	2.12133669
RCAN2	1.0952529	3.54367007	2.98239848	3.16640797	2.38222351	2.50865035	1.78248623	1.57292707	1.12700403
RCAN3	2.63163245	1.76049129	2.37591748	2.71970144	2.39913113	1.97017795	1.61948457	1.84145312	2.21178972
RCBTB1	2.65885698	2.62976356	3.23030556	2.86214422	3.27199952	3.06128948	3.24408843	3.45470172	3.20396274
RCBTB2	1.2781055	3.13955389	2.90435458	2.6799295	2.1849361	2.89039533	2.11201958	1.80367442	1.6047381
RCC1	3.76674031	2.39440467	3.7664189	4.16238512	5.1295526	3.83258587	3.61767963	2.45154488	4.18634514

RCC2	6.11638262	4.4483453	4.65332514	5.20262959	5.54146138	5.01278251	5.16657542	4.38851271	5.52287997
RCCD1	2.47250304	1.23873742	1.7539011	1.4757358	1.95188717	1.49727867	1.69184162	1.75397263	2.50635142
RCE1	3.8027717	2.13157104	3.51889854	3.13053581	2.76528989	3.10467741	3.25137766	2.97440918	3.31167472
RCHY1	1.54602855	2.09973513	1.95297848	2.51861254	2.65545078	2.43668076	2.55603765	2.08322931	2.78997185
RCL1	1.88166247	1.51461543	1.56630692	2.32872119	2.61897459	2.40856335	1.93216747	2.84565298	1.95230372
RCN3	4.2319412	5.27087526	3.45736108	4.57524391	2.55523445	4.50679474	4.27166787	3.03908687	3.30664541
RCOR1	2.94492291	3.15793981	2.58982336	4.03779712	4.03268225	3.94275402	3.72149077	4.51260508	3.45106518
RCOR2	0.22165953	0.74541753	1.25083808	1.97184299	0.17725192	0.87227085	2.30227072	1.63961608	2.47726917
RCOR3	1.70929003	2.26078953	2.61369601	2.63131033	2.37338629	2.17630753	2.56917869	3.10845917	2.52048034
RCSD1	1.57707855	2.49208663	2.58533044	1.89407122	2.76376034	2.78873682	1.81894722	1.41889749	1.26321678
RCVRN	0.01242324	0.21228275	0.07892773	0.014894	0.01529127	0.0182216	0.05854571	0.00958576	0
RDH10	4.11216612	3.86167128	3.93345781	3.89031018	2.98333758	3.66172701	3.77346143	4.03976813	3.21723035
RDH11	4.24938735	3.68489655	3.82446767	4.25199252	3.65791223	3.92041394	4.19488899	3.58832065	4.0813347
RDH12	0.11872962	0.24397557	0.97126574	0.28536508	0.17056932	0.140687	0.05451589	0.12554116	0.03216312
RDH13	1.47881235	0.6452831	1.42897216	1.37727519	2.23806658	1.6405876	1.6129217	1.35233035	1.54088558
RDH14	3.11378627	3.26274132	3.24596176	3.25941357	3.40067027	3.55527354	2.73551377	3.2806515	3.19105201
RDH16	0	0.05903984	0.11676206	0.24870041	0.14186365	0.05312909	0.02497446	0.12052797	0.07541761
RDH5	1.86815737	0.94234482	0.4956138	0.67305814	0.58073898	0.47398716	0.11926139	0.45980353	0.57065878
REC8	0.78483067	1.17698237	3.73866975	1.07040721	2.02888205	1.36554425	0.56169162	0.89228344	1.64199325
RECK	0.55799405	3.15909739	1.70679453	2.31437264	1.18544383	2.03780165	1.66850141	1.05150564	0.67579565
RECQL	1.44277849	3.14468609	2.99472401	3.06442436	3.78340444	3.22430203	2.94941629	2.608839	2.76388089
RECQL4	2.95655838	1.18315413	2.71702334	2.5319753	2.47201802	2.09025952	2.98909479	2.36679887	2.97945636
RECQL5	1.87520304	1.13319784	1.83390193	1.42809213	1.61740626	1.2749445	0.99789263	1.93275596	1.68327802
REEP1	0.10160783	2.52587058	0.50408322	1.18241234	0.0757957	1.67978756	2.20229115	0.21799565	0.51558725
REEP2	0.36133881	1.90668096	0.63760484	1.19674657	0.1615275	1.3814862	0.28809311	0.17689814	0.22628959
REEP3	2.68393289	4.19770554	4.53924587	4.74658842	3.71544479	4.21436315	4.10771598	3.90413367	3.16464139
REEP4	4.7719277	3.36779778	3.4556234	4.33310721	4.21738864	4.34346154	5.2877973	3.9571262	4.08774672
REEP5	4.88052702	4.92201052	5.49765117	5.43056714	5.24345275	5.18508506	5.16598114	4.36178277	4.95056826
REEP6	2.69769433	5.13573897	1.4561932	2.88974677	1.12140898	1.49916164	3.52318213	4.06622094	6.18055684
REG1A	4.10897346	10.2156944	7.70709722	5.13478586	2.58447771	1.81482674	7.44695491	11.4512258	10.9358771
REG1B	0	7.69197506	0.06479018	0.08027033	0.00938704	0.06587573	1.92977897	1.07503755	6.93565947
REG4	0.01039617	5.9426535	4.89038226	9.25697013	7.30796334	4.78501254	9.7308033	7.76368603	4.52469403
REL	1.35609861	1.67446871	1.63298453	1.52733976	2.09473394	2.08523814	1.96623591	2.15180884	1.11792664
RELA	4.64968868	3.84028279	4.42930324	3.98724083	4.0843964	4.51479446	4.36202869	4.59430682	4.40249131
RELB	4.03475194	2.83992616	3.4364123	3.34744479	3.3496223	4.19476821	2.87140602	3.68230686	2.46339415
RELL2	0.36952851	0.24359508	1.14353074	0.8431738	0.68299353	1.00337665	0.51119298	0.44366038	0.53107311
RELT	2.19920537	0.73245812	1.52862646	1.6041316	1.54234376	1.73932302	1.35857953	0.94183222	1.39499817
REM1	0.41552995	2.03346252	1.4265584	1.32828507	1.13512912	1.45085734	0.44708916	0.44011007	0.75348166
RENBP	2.91800025	2.25838044	2.70064428	1.74432578	2.40317528	2.65785412	2.06712397	1.31397244	1.61316974
REPIN1	6.21131283	4.4240907	5.761821	4.1476317	4.30615769	4.54354996	4.7534351	5.10283391	6.31965609
REPS1	1.98348791	2.13394712	1.85944682	2.63063291	2.54264297	2.1665838	2.57512771	1.97617972	2.65105229
REPS2	2.1474586	1.97764502	1.16920173	1.66235228	1.71843897	1.38041872	1.98108242	2.43741463	1.52874967
RER1	4.31507581	3.86440668	3.91164944	3.98200943	4.29582709	4.16174757	4.20737667	3.89214903	4.43888405
RERE	4.22212613	3.67629259	3.06846233	3.01680885	3.26917089	3.4668034	3.19343674	2.82337075	3.89585396
RERG	0.22669784	2.85968462	1.94131265	1.80779682	0.46660245	1.96031522	0.42040564	1.43442922	0.45137076
REST	2.57997811	2.91962111	2.92696765	3.55775875	3.37715278	3.22524516	2.92202326	2.7841545	3.35506621
RET	0.08498908	0.96296216	0.16068372	0.37636493	0.08507742	0.51659431	0.50356974	0.18388397	0.51056597
RETSAT	3.19466347	4.42115868	4.0185084	4.22517568	3.82898223	3.67005261	4.52694915	4.91516917	4.51552476
REV1	1.35474497	1.67285825	2.26543432	2.08549683	2.21090972	1.89498001	1.70736781	2.10593825	1.90625048
REV3L	1.19215777	3.12875147	1.82540829	2.56426634	2.43964528	2.42634509	1.98854978	1.5078389	1.67102455
REXO1	3.56386082	1.94798504	2.7772344	2.36327113	3.28089191	2.74271648	2.14895282	2.83795829	3.04232458
REXO2	2.84957272	4.20407421	3.07730784	3.88909355	3.48084912	3.91882416	3.80652253	2.69954177	3.32702767
REXO4	4.32731299	2.55170756	3.6950611	2.84375513	3.11110283	3.04375122	2.93854208	3.43180988	3.40546719
RFC1	2.73545929	3.22536325	2.90382912	3.59356196	4.19567628	3.22301783	2.83103694	3.33146105	3.5811298
RFC2	4.62956414	3.36178248	3.73773395	3.46943327	4.10339268	3.79225253	4.05909253	3.51279888	3.46799342
RFC3	3.57599614	2.91459286	3.07005428	4.11930274	4.79043913	3.11229601	3.55645606	2.82671063	4.06013482
RFC4	3.72303389	2.1051541	3.26738808	2.63782129	3.73124543	2.75909706	2.87723946	2.87223391	3.05185278
RFC5	2.92326709	2.26940019	3.13790397	3.10806819	3.79393785	3.1729717	2.89379716	3.05207434	3.35392242
RFFL	3.03193399	1.67947883	2.22430435	2.1148271	2.62749382	1.87853027	2.14746224	2.4077767	2.63828542

RFNG	4.29769599	2.64099407	3.11107164	2.95174319	2.17604721	2.92129528	2.99919752	3.7580969	3.01448401
RFT1	2.3599622	1.99732427	2.28124359	2.41046404	1.9277092	2.43450215	2.44041556	2.54460322	2.57005113
RFTN1	2.59471767	3.15257142	3.36312008	2.8789642	2.88426311	3.11192909	3.06046928	2.40971262	1.66763777
RFTN2	0.30017756	1.73105758	1.03862251	1.2448943	0.6696426	1.00202116	0.71539108	0.45881602	0.36194242
RFWD3	2.02686034	2.1815281	3.0178904	3.13087167	3.42580933	3.12522842	3.58782564	2.8240097	3.13357769
RFX1	2.60737687	1.97296854	1.94684871	1.89552628	1.68118643	2.17711152	2.14857084	2.23659242	2.84569753
RFX2	2.04150309	1.32951435	1.61781633	1.30349463	0.46992388	1.11270862	0.53502302	0.85892299	0.45663302
RFX3	0.90538455	0.90749463	0.69537271	1.14961859	2.17619498	1.12216441	1.13721747	1.61348528	1.45756873
RFX5	3.78734173	3.37624269	3.92480529	3.43984581	4.13950731	4.05367638	3.52672719	3.2001872	3.71564024
RFX7	2.02101815	2.53527532	2.25287151	2.56868546	2.99268425	2.67392162	2.1381526	1.95266399	2.1349973
RFXANK	4.76929467	2.8851888	3.43876294	2.88402639	3.35427136	3.31762793	2.94372555	3.41236184	3.29059184
RFXAP	1.65905305	1.63615451	1.53184053	2.09304431	2.26692734	1.75994223	1.61618952	1.46941586	2.79306063
RGCC	3.2801123	3.65484116	5.6419402	4.26493275	4.27441609	4.64153535	4.24076238	3.50217031	2.84835344
RGL1	1.25729199	4.22027993	3.62637543	3.07713991	3.23784903	3.73029536	1.9375441	1.59312237	1.71490508
RGL2	3.71634304	2.75182724	4.72293694	2.86947881	2.31083174	3.10254253	3.01549289	4.21230588	2.79835414
RGL3	2.38403549	0.17567392	1.46409101	2.11954508	0.12808931	1.40284034	0.7425756	2.14492676	0.30049429
RGL4	0.28648749	0.35185612	0.39345096	0.30410386	0.70523838	1.49111903	0.26164473	0.22822536	0.26973265
RGMA	0.63028653	3.23695387	0.85463413	0.71580993	0.18570643	2.6058763	0.31092607	0.29915637	0.29198406
RGMB	1.34337347	3.11492275	1.4367749	1.75840297	1.29475523	3.07001751	1.30253483	1.02488012	4.40757623
RGPD1	0.01056953	0.0151117	0.0190342	0.00635054	0.00976964	0.01935827	0.00317162	0.00408288	0.03034733
RGPD4	0.01327873	0.01897752	0.13338553	0.00798131	0.01634315	0.02911285	0.02768169	0.01534267	0.01047987
RGPD5	0.005133	0.00587884	0.00371023	0.01228776	0.0063217	0.01690464	0.00767485	0.0108594	0.01211208
RGS1	3.37343466	4.69711132	6.3839	4.85155549	6.04780209	5.33586099	4.74530818	4.29139469	4.82118906
RGS10	4.30926567	4.55756479	5.25828782	4.59367967	5.26683159	4.57447915	4.21893518	3.87186823	3.90379779
RGS11	0.03006794	0.33614175	0.2465738	1.19657463	0.05508735	0.48388744	0.1509615	0.03471025	0.52054657
RGS12	1.35940526	1.25165179	1.90709814	1.07620181	0.85733369	1.42443651	0.83982165	0.92368939	1.84665177
RGS13	0	0.65900294	0.32854845	0.17369186	0.13729713	0.11092491	0.02916949	0.03514156	0.05084248
RGS14	2.25869958	2.00107036	2.31610522	1.91002682	1.59679179	2.0372075	2.04902588	2.96685114	2.43838277
RGS16	4.0296206	2.37610122	2.88005466	4.37734905	3.79247883	5.05896554	3.91826018	2.418098	3.17761049
RGS17	0.10668894	0.36263337	0.46354083	0.42800122	0.24991326	0.37026225	0.20558231	0.16293567	0.22624288
RGS18	0.39516848	3.01807659	2.593165	1.83195571	2.64435072	3.01825669	0.99001479	0.90107724	0.96083501
RGS19	4.58089093	3.53904649	4.46384662	3.69319798	3.92054828	4.54327491	3.54199866	2.76849689	2.51951743
RGS2	2.89685434	6.37240821	4.90583769	5.59537745	4.47089085	6.70862003	4.55163018	3.85926681	3.76828781
RGS4	0.49842533	2.10343569	0.89272366	1.79606115	0.77499243	1.99161992	1.54902176	0.62629319	0.50599394
RGS5	2.69296238	6.25902123	7.15258809	5.61499401	5.91494111	5.10882989	4.48057802	5.1262585	3.84401004
RGS6	0	0.14436157	0.2384306	0.05755518	0.0396457	0.03551941	0.01215419	1.546848	0.20357769
RHBDD1	2.41002768	2.57425004	2.89021533	2.72480481	3.23217254	3.13962492	2.71870166	2.74696906	2.57247321
RHBDD2	6.10381446	5.31461667	5.56910226	4.88167462	4.25403406	4.67344703	5.23620747	5.15791368	4.9524407
RHBDD3	4.11146358	2.23606078	2.28730672	1.87187486	1.99249611	2.2933045	1.89503804	1.58166233	2.65028595
RHBDF1	1.87501622	2.5700103	2.18840736	3.28494062	2.53237625	2.21933354	3.43885954	3.07350945	2.28137519
RHBDF2	2.85065436	2.3404748	2.87718108	2.9175736	3.48479159	2.8693041	2.89963395	3.6143098	3.35069003
RHBDL1	0.43148657	2.01487001	0.43161047	0.53654459	0.54033867	0.25950069	0.46178286	0.75915743	2.65376507
RHEB	5.039098	4.54810106	4.80797195	4.22682133	4.46399236	4.62255687	4.76150688	3.91323345	4.15426392
RHNO1	4.33437477	3.13866062	3.58829156	2.64843717	3.89513889	3.61864573	3.20414303	3.32346441	3.46965091
RHOA	7.58524883	7.88160509	8.15854316	8.0879532	7.99199663	8.23416632	8.40774662	7.98313991	7.75935682
RHOB	5.26752963	7.79604891	5.9944413	6.4899997	4.95706833	8.05883743	6.35006059	7.07425013	5.44943209
RHOBTB1	1.41957655	2.64701048	3.32233111	2.69433069	2.17635015	2.57811808	2.47361801	3.28404178	1.79892314
RHOBTB2	2.67672562	2.18703898	1.6359756	2.28857073	1.53681642	2.1116846	1.78844447	3.60895872	1.52443902
RHOC	5.80755044	5.24003196	5.03614069	5.14268159	4.37049255	5.36500594	5.34776917	5.20629856	4.92013372
RHOD	1.33210658	2.96034821	3.1501292	3.48950951	0.51659805	2.97190854	3.51850414	3.84413772	2.8275472
RHOF	0.40367423	0.89646406	0.40577131	0.89961018	0.54671056	1.47012105	1.4265787	2.10910666	1.03446865
RHOG	6.01648574	5.73828948	5.85453676	5.77952817	6.01157513	6.31059182	5.85691452	5.52969563	5.40853585
RHOJ	1.22157897	3.87467515	2.6213583	2.68128139	1.91121391	3.51127669	1.72262594	1.56864954	1.14715973
RHOQ	2.92414787	4.32529625	3.69382775	3.94282105	3.22686976	4.11511089	3.37612275	2.9351396	2.56317854
RHOT1	2.54318588	2.71598288	3.02561314	3.05222694	3.09123846	2.86904389	2.54510666	3.02101594	3.04909358
RHOT2	3.67871757	2.82544297	2.77427748	3.05559916	2.89349018	3.01446121	3.40731042	3.14887493	4.1262616
RHPN1	2.73040449	0.44509423	1.99993062	2.55348679	1.35735741	1.51995789	1.6208082	2.22081299	1.42581373
RHPN2	3.49304374	3.32972965	4.28690859	4.33247905	5.06879745	4.00180395	4.20292927	4.70098019	4.35835638
RIBC1	0.94742641	0.55952954	0.23491262	0.42689539	0.27259995	0.19629715	0.13887721	0.31496158	0.39505489

RIC1	1.42960014	2.50221164	2.06869946	2.70449535	2.87580668	2.58264633	2.15664858	2.99045828	1.98432989
RIC8A	4.09000245	3.24168564	3.1819931	3.57788532	3.4674595	3.85823885	3.92804042	3.54472699	3.74913026
RIC8B	0.91930877	1.56028408	1.8624845	1.72081814	1.93620294	1.69800666	1.45933925	1.83917185	1.96384347
RICTOR	0.92068652	2.39295809	2.06538053	2.00598993	2.63611044	2.4638527	1.85880327	2.49285487	2.14885941
RIF1	2.20781282	2.22552612	2.49711262	2.49557968	3.19369714	2.27095564	2.59084429	2.66542078	2.59975866
RILP	2.4028222	2.80449073	3.02081753	2.22511182	2.12665077	3.11762949	2.6286376	2.30178075	3.16484455
RILPL1	0.92467657	1.21455831	1.11967667	1.17409226	0.67337072	1.43275682	0.57695377	1.30558617	0.68345238
RILPL2	2.57356722	1.7047252	2.43572143	1.72899701	1.99283352	2.37691148	1.69745296	2.1893391	1.45336741
RIMKLA	0.00405231	0.80801736	1.67108917	0.99564281	0.02969126	0.7678835	0.65303905	0.07763881	1.39873926
RIMKLB	1.14052608	1.4234135	1.16812923	1.74081089	0.42552448	1.37865654	0.43578792	0.75237354	0.17753727
RIMS1	0	0.6216219	0.16701943	0.03212648	0.02264522	0.15850572	0.00850482	0.01855335	0.0178653
RIMS3	0.36715047	1.0157774	0.4550335	0.51966734	0.51621218	0.95250425	0.35857903	0.1735682	1.46566199
RIN1	2.6654542	1.55030444	2.21229217	2.14304708	0.54130476	2.28679008	2.06532636	2.60260119	2.606205
RIN2	1.76115333	4.02429339	3.83417365	4.51339939	2.64197334	3.53749834	4.33270152	3.71368286	4.42713382
RIN3	2.04120573	3.24548922	2.30481128	2.26300067	2.06394686	2.79630553	2.75909852	1.89237494	3.04816561
RING1	5.09164453	3.72350026	4.49976261	4.13735136	3.50125009	4.01236266	3.56232539	4.57150126	3.70209492
RINL	0.94112842	0.78807311	1.61725774	1.32446937	1.07815164	0.97976217	1.03384681	1.23764448	0.99228173
RINT1	2.15791281	2.59069353	3.18890132	2.79365046	3.21963529	2.34652944	2.86811408	2.67678532	2.69566341
RIOK1	2.47647802	2.52063933	2.95398147	3.1352574	3.15519224	3.09201959	3.37904355	3.33824573	3.38786537
RIOK2	1.76022643	2.43011327	2.11005975	2.71283054	3.0203485	2.38706759	2.39658431	2.46963548	2.7781503
RIOK3	3.19425707	3.68070944	4.70321317	4.44320325	4.05669597	3.86753466	5.47271523	4.22646696	3.87188434
RIPK1	2.95026247	3.5426547	3.75588112	4.12566935	3.95242068	3.95629686	3.80649732	4.16040312	3.71236269
RIPK2	4.48463908	3.30595682	5.03018588	4.09585762	4.99615986	4.77081449	3.93243625	3.29175558	3.01133855
RIPK3	1.70729781	2.56549591	2.76821114	2.30254609	1.92631616	2.29185234	1.91023745	2.88620487	3.38980483
RIPK4	1.66892997	1.27975248	2.34020405	2.42708081	2.75313625	2.49385483	1.91347195	4.04768087	2.71824609
RIPPLY3	0	0.06167664	0.13019361	0.20203198	0	0.26318294	0.2097653	0.18186924	0
RIT1	2.71998257	2.87932101	3.49423687	3.18065225	2.88776663	3.12213417	3.42790561	2.98984787	2.27971292
RITA1	4.18140768	2.89208057	3.33933229	3.08230299	3.00896796	3.10727456	3.25002143	4.08480058	3.55224345
RLIM	2.79604814	3.45679153	2.99182609	3.24373115	4.65027437	3.60858639	3.35413747	3.47204431	3.06314517
RMDN3	3.87267547	2.00989482	2.75091271	2.49822738	2.87193802	2.64610922	2.56424211	1.69806631	2.6498654
RMI1	1.83655138	2.37170215	3.000841	2.61073607	3.15791301	2.60694329	2.33349402	2.82940069	3.32356558
RMI2	2.71271054	2.35757175	2.39050034	2.05237983	2.72254456	1.94311246	3.04442656	2.63850373	2.50718144
RMND1	2.15352499	3.16310004	2.95612179	3.680549	3.69401733	3.11281359	3.14581667	2.53310897	3.39174483
RMND5A	3.46700659	2.89892584	3.28695818	3.59636041	2.89324657	3.1190895	3.74742835	3.34904481	3.50327633
RMND5B	2.77930326	1.6679908	2.06691312	1.73900013	2.15234367	2.06498795	1.71359002	2.31284979	2.2867561
RNASE1	6.66952687	8.45262462	10.6085958	7.14823636	6.47440004	6.48326932	7.28357742	5.79610572	7.00507056
RNASE4	0.90311062	0.89830431	1.67498026	1.99953168	0.7628336	0.77734964	1.22097614	1.70069745	1.01899353
RNASE6	4.11744779	6.08115476	6.29826469	4.6827819	5.90848614	5.06643049	4.67483708	3.95751812	4.26656059
RNASEH1	3.10295082	2.46077146	2.97816303	2.36086921	2.80108167	2.61475689	3.07851217	2.81813917	2.68420559
RNASEH2A	4.62864778	2.6556109	3.66965278	2.95265844	4.23839789	3.32710052	3.24440297	3.53850808	3.81188791
RNASEH2B	2.42675863	2.10214583	1.98185606	1.72416925	2.45494214	2.27161045	2.12380891	1.48192452	2.52378621
RNASEH2C	3.66654669	3.08598688	2.61219338	2.20657258	2.04383708	2.58039949	2.03577963	2.97316899	2.71762782
RNASEK	2.03791922	2.29948026	2.73150422	3.23196664	3.09014909	2.6014335	2.57163029	2.15395181	2.76957818
RNASEL	0.95017426	2.29567103	1.54690878	2.07867431	1.60298439	2.00398681	1.85676171	2.67194694	1.31325977
RNASET2	3.22735408	3.14617964	4.74691953	2.82573086	2.95189162	2.86388107	3.6853991	3.53145964	3.51092706
RND1	2.5920432	0.67595967	2.96602569	1.70679114	2.78249999	3.17421381	0.85599776	3.56280915	1.08874829
RND2	0.12809889	0.53150065	0.73330219	0.35449432	0.05067691	0.18455284	0.02484363	0.09973216	0.03907356
RND3	1.21937272	3.99101694	4.03399736	4.63598816	3.86025235	4.42957922	4.29412533	3.40094631	3.23403789
RNF10	4.72890788	4.44400711	4.38964622	4.22887264	4.31149679	4.5171916	4.42707298	5.14598509	4.49489655
RNF11	4.3790691	5.38111467	5.03284744	5.34076769	4.77710663	4.97932489	4.5467544	5.04699468	4.09471009
RNF111	2.11951585	2.71401837	2.77996658	2.82857728	3.00699958	2.79665369	2.48790207	2.34706812	1.8743739
RNF112	0.0099451	0.17221844	0.18894299	0.14782873	0.01832643	0.42284367	0.04122176	0.13221799	0.03113503
RNF113A	3.20984888	3.13866857	3.96052444	2.81050861	3.70644	2.87012838	3.00741083	3.26726085	3.26702754
RNF114	3.93763796	4.40139828	5.03673108	4.22168204	4.25972703	4.39182945	4.52664267	3.73194475	5.17955921
RNF115	2.17962955	2.12268354	2.47780977	2.34901027	2.60604026	2.31678519	2.28204161	1.82721371	2.21911185
RNF121	2.98147467	2.36568063	2.79499461	2.33119772	2.70175465	2.50288743	2.64640512	2.84393206	2.51231818
RNF122	3.83728816	3.4390129	2.63854419	2.97227856	2.26771549	3.44663089	2.76299954	1.97042152	2.04533736
RNF123	2.40922882	2.15746011	2.17051226	2.0941548	2.2086555	2.41237754	2.24639925	2.75531572	2.78816155
RNF125	0.44876809	1.6826002	1.17219278	2.82290812	1.29225351	1.41611427	1.9668416	1.05876581	1.84775105

RNF128	5.51113559	3.36292126	3.44296035	4.51611427	4.65200114	3.01922845	3.71721998	5.33514671	4.92309345
RNF13	4.33739629	4.55940613	5.04692151	4.75692468	4.72015791	4.84257586	4.76774239	5.06763069	4.27308254
RNF130	2.13250422	3.63937729	3.29436363	3.38693806	3.44597626	3.48528514	3.05949864	2.81102681	2.83175098
RNF135	2.66806635	2.64207846	2.98975655	2.47604677	2.35396589	3.17298409	2.42313759	2.73837941	1.46948497
RNF138	2.36933229	3.18745121	3.8381497	3.58382399	3.6255805	3.34440181	4.1041417	2.47815914	3.75432476
RNF14	2.68487079	3.03078837	3.07161116	2.99982173	2.91410674	2.79744262	2.55925845	3.1772217	2.82566718
RNF141	2.12313143	2.8922905	2.38715688	2.95931081	2.92921704	2.82290425	3.51140474	2.9002576	2.92280961
RNF144A	0.82913785	3.15053755	1.64166891	2.94550328	0.80148516	2.87141559	3.614258	1.27316391	1.80082972
RNF144B	1.55481105	2.535588	4.83384082	2.63598127	3.43823873	2.5084764	2.06020915	3.93499387	1.23087568
RNF146	2.83644983	3.1327696	2.42496624	3.41126099	3.01835172	3.06774845	2.50514713	2.13384498	2.59590419
RNF149	3.56676476	3.46916519	3.97471569	4.01622763	4.27946473	3.84785932	3.74767299	3.51182247	3.63741675
RNF150	0.32740183	2.57085416	0.71371712	1.10693173	0.35767292	2.03407362	0.59162731	0.45390918	0.1439864
RNF152	0.36722907	1.44302932	1.12271996	1.30016054	0.46373184	1.46556335	1.59098236	0.48184084	0.27776592
RNF157	1.22679877	1.11417571	0.63852404	0.79757399	1.00955202	0.6528092	0.39820189	1.03966547	1.2050073
RNF165	0.05633459	0.27391463	0.27425963	0.3183102	0.00784331	0.10948723	0.01520372	0.0268117	0.01169585
RNF167	4.81925139	4.9585934	5.5948256	4.92602245	4.86864743	4.84007229	4.51381106	4.2008435	5.36553383
RNF168	2.99774151	1.73083007	2.54765035	2.27775005	2.28505995	2.15522908	2.24450335	2.5136617	1.89405731
RNF170	2.00289809	2.3688579	1.99857774	2.8763119	2.14433429	2.76075598	2.42370511	2.89872715	2.1614626
RNF180	0.12211303	1.35788349	0.68518379	1.08489026	0.35365776	0.99821085	0.4746635	0.35962968	0.13774932
RNF181	5.96477723	5.10729089	5.37540211	5.0401015	5.28209949	5.00661455	4.87515128	5.57575617	5.0782108
RNF185	3.93320763	3.83611658	3.83635464	3.39132531	3.11461972	3.70327677	3.35599107	3.12892958	3.46752995
RNF186	0.08491892	1.79794471	0.71582528	0.051545	0.76314955	0.13294528	1.47421563	0.26605668	5.09352356
RNF19A	2.58670274	3.58195525	4.62747167	4.02779604	4.55259014	4.05288701	3.17106869	4.13271567	3.02517813
RNF19B	5.54814381	3.45013405	4.28712867	4.82795058	5.65295796	4.7370802	4.04476185	3.68721248	4.55378808
RNF2	0.89693933	2.46160605	3.15304599	3.59204043	1.75228872	3.44555976	3.78051762	3.00889655	2.77100822
RNF20	3.26545333	3.62322524	4.01674885	3.47160112	3.65692163	3.49926938	3.28487345	3.30628612	3.49857976
RNF207	0.64870967	0.30465351	0.96599645	1.60150544	1.79577084	0.82457305	0.57891621	1.18086883	0.73065115
RNF214	1.96584678	1.73526025	1.53221219	2.09783997	1.85679738	1.99580573	2.06721834	1.58238721	2.22570727
RNF216	3.70952511	3.38600274	3.1902404	2.94443205	3.41680237	3.4873446	3.08550358	2.98127936	2.9368827
RNF217	0.32314584	1.71937776	0.86262503	1.28536311	0.80145697	1.69469594	0.77997401	0.46327322	0.27735857
RNF219	1.76470211	2.17438729	1.79578076	2.28885536	2.77277553	2.12301933	2.21615905	1.69365758	2.62525244
RNF220	3.16585425	2.01373857	2.91662696	2.53490406	2.35543895	2.53018195	2.41253409	2.6271999	2.85647106
RNF24	2.33971822	3.33105947	2.52134414	2.83373451	2.45234488	2.51651074	1.90196936	2.0689929	2.73758086
RNF25	3.52961774	2.68519085	3.45651037	3.22412683	3.03119705	3.06278851	2.73201487	2.99914587	3.08154856
RNF26	4.53874725	3.80366425	3.23191817	3.93001039	3.8323836	4.29229835	4.6790316	3.35174872	4.13939885
RNF31	2.72312418	2.2775012	2.51374944	1.92668488	2.55409398	2.43856562	2.06906987	2.53307941	2.52278147
RNF38	2.28231359	3.7408651	3.22117779	3.4123943	3.62596442	3.66069729	3.0581997	3.29332012	3.36307639
RNF39	0.78361282	0.76710274	3.48233375	1.10255236	0.86261139	0.66816367	2.85283994	4.75136883	0.77422688
RNF4	3.52627718	3.41833082	3.31354479	3.64762201	4.19869668	3.88104212	3.36949063	3.90126507	3.90723785
RNF40	4.07708345	3.15261611	2.79142524	2.6816696	2.74663749	3.13267315	3.31311458	3.54020525	3.14359348
RNF41	3.39320704	2.94575796	3.59933334	3.11788278	3.0787903	3.37569308	3.19626499	3.455715	3.10689787
RNF43	2.32060744	3.57091792	2.88144479	2.4797675	1.71502611	1.29100541	1.90313947	4.3421304	4.7941321
RNF44	4.18737448	3.19621623	3.67060644	3.41466374	3.71247594	3.44386214	3.3457652	4.23390188	4.65530851
RNF5	6.95725859	5.65687152	6.63623143	5.41392351	4.9882992	5.37227091	5.22269396	6.63164493	6.24822416
RNF6	3.52855134	3.51915366	3.58345993	3.7207626	4.46304008	3.79003498	4.3152761	4.02482811	4.56283184
RNF7	5.76055552	4.02087161	4.19966707	4.16493346	4.35397146	4.03473663	4.69997349	3.82859886	4.34037783
RNF8	1.38395833	1.73768197	1.46312945	1.94364993	1.76253076	2.04553409	2.19346842	1.51447281	1.93634998
RNFT1	1.89247578	2.01875592	4.36106908	2.92330979	2.95651054	2.89934823	2.24940671	2.36073228	2.0782466
RNFT2	1.12171211	0.21764473	0.96624477	0.57366297	0.32134632	0.43048793	0.603914	0.5804336	0.45038224
RNGTT	1.7977771	2.0883069	2.14862841	2.99469027	2.95168001	2.67249835	3.19885053	1.95373973	2.68644894
RNH1	4.37024259	3.4475182	3.04513198	3.31631754	3.10427972	3.91142389	3.93637795	3.47121703	3.54397286
RNLS	0.2542197	1.20083155	0.51758402	0.90075527	0.59496357	0.84233301	0.50496529	0.24870776	0.19556878
RNMT	1.96545236	2.70784676	2.93691965	3.29694006	2.93776623	3.09674522	3.59148666	3.1374076	3.22040074
RNPC3	0.8377371	1.04257643	1.604611	1.68719833	1.78651797	1.13501192	0.90460455	1.37577913	1.55460627
RNPEP	4.77121667	4.63848716	4.95914286	4.78067281	4.67897908	4.9403894	6.59555957	5.24038081	5.66786994
RNPEPL1	4.84939385	3.22639484	3.93441571	3.16215125	3.57214037	4.1049135	4.40489201	4.61768385	3.67533682
RNPS1	4.22448818	3.80279233	3.11544267	4.06023643	4.13165861	3.65268688	3.94826279	3.71469088	4.41246162
ROBO2	0.02386056	0.21061027	0.72871949	0.06792284	0.03066198	0.15815795	0.98690185	0.57723751	0.0408053
ROBO3	0.96819632	0.3469297	0.43090238	0.91246703	0.36507089	0.52465564	0.31156555	0.33560808	0.3790614

ROBO4	1.59218056	2.33188166	2.45799894	1.59828332	1.39185741	2.47089193	1.61040125	1.34056945	0.99638196
ROCK1	2.40269958	3.17286154	2.99923305	3.33547904	3.13764281	3.3767673	3.17620678	3.01915323	2.95547361
ROCK2	1.53353368	3.79236697	3.16125708	3.46065442	3.27581818	3.23934848	3.13972472	3.20776869	3.2793568
ROGDI	2.97182659	2.48762015	2.28982496	2.09196195	1.38632544	2.21227774	2.10745639	2.4481359	2.45834781
ROM1	1.04482676	1.01487059	0.76742512	0.43543856	0.24746824	0.63626473	0.6997891	0.45082447	0.32208511
ROMO1	6.67410556	5.59472823	6.16315108	5.17411573	5.0204648	5.48597968	5.55758905	5.84350689	5.84833038
ROR1	1.92701582	2.9021423	2.90659772	1.87669024	0.88251504	1.57834773	0.73102688	2.68450187	0.96286025
ROR2	1.12212963	3.29670437	1.28679203	2.50751622	0.66763642	2.82372253	1.43148419	0.69974002	0.41491243
RORA	0.3696637	1.38030639	1.18829328	1.02208455	1.52629242	1.38713506	0.69706327	0.5006228	0.47954395
RORB	0.00379492	0.53564586	0.09044472	0.19184059	0.11902088	0.17714834	0.09889116	0.05742431	0.00995247
RORC	2.31077288	1.20038056	1.52263965	1.06973356	0.23048091	0.96429901	2.85407859	2.14879892	2.45754788
RP2	2.30229155	3.43110197	3.54806609	3.1373623	4.3424225	3.69371893	3.36909555	3.54325272	3.51863454
RP9	3.23241387	1.86609428	2.1944175	2.25851469	2.11946369	1.9156289	2.0409589	2.01688995	2.50598137
RPA2	3.61892244	4.17792786	4.56394202	4.98274726	5.23373831	4.43234627	3.72827311	3.81440982	4.05254823
RPA3	3.99710119	3.01643895	3.55894045	2.94241775	3.64144586	3.06011281	3.27487828	2.54101542	3.05109085
RPA4	0.09054677	0.17614144	0.21882801	0.12093311	0.15021427	0.06708606	0.04135572	0.11254857	0.04830237
RPAIN	1.35071391	1.48415774	2.16370481	1.9950527	2.056078	1.52610953	1.4228284	1.32820822	2.36600483
RPAP2	0.8528776	1.19096939	1.21358423	1.33282315	1.6697328	1.12959043	0.93785035	1.21447372	1.23703161
RPE	3.45923952	3.25606718	3.7057467	3.34382464	4.46185349	3.62803432	3.73810859	3.64768956	3.80846925
RPF1	3.9746565	4.22900411	4.30279262	5.09987987	4.22679734	4.6096255	4.4016419	4.38355133	4.48795211
RPF2	2.99326754	2.96271632	2.41350281	3.495089	4.11206777	3.29749664	3.3537027	2.44917958	3.50976474
RPGR	0.71690824	1.41422296	1.04467105	1.60159781	2.08470268	1.3645122	1.01916867	0.6704506	1.89804717
RPIA	4.06823286	3.24248949	4.76766704	3.50357507	2.98129285	3.18290413	3.33617971	3.63302604	4.72247472
RPL10A	8.71377575	8.74963759	8.3132	9.23491648	8.93799536	8.89149885	8.97696077	8.82701426	9.56220675
RPL10L	0.28424579	0.16959208	0.29250091	0.37700011	0.58332723	0.19987537	0.05275789	0.13214884	0.29741406
RPL11	8.89393656	8.22029856	7.23339964	8.79708902	8.62393388	8.10248007	8.19368199	7.47043405	8.64540943
RPL12	8.42683068	8.45374075	7.92242187	8.41660839	7.94373391	7.70698345	8.65368114	7.5679664	8.89658559
RPL13	7.4024812	7.41951384	6.65482869	6.9281714	6.37971588	7.32261957	7.64425806	7.64930276	8.11466076
RPL13A	9.53681548	9.00437253	8.51914721	9.01539007	8.99828035	8.96776062	8.83741083	8.86243876	9.73277383
RPL14	6.03061387	6.21217115	5.30078874	6.39414157	6.07136326	6.20704438	6.58181758	6.1879525	6.84547336
RPL17	4.36504249	4.87316846	4.16954239	4.76371948	4.52535264	5.1579423	6.29157976	4.67054249	5.62178268
RPL18	8.10976873	6.8349147	6.73725499	7.18131551	7.18342207	6.78533417	7.14089089	6.91409496	7.80860951
RPL18A	8.71372661	7.18834836	6.87975142	6.65079961	7.1143111	7.38372767	7.28218399	7.31149209	8.00619406
RPL19	9.62876811	8.30718561	8.17766713	8.4556186	8.40724014	8.50051636	8.30778657	8.60942181	8.92411814
RPL21	8.19784144	7.6181576	7.15244658	6.96021447	6.93837545	7.42754579	7.83158349	7.48307773	8.73449145
RPL22	5.11176039	4.87785833	4.11911998	5.37494284	5.40552377	4.49933314	4.3339727	4.2275949	5.45838656
RPL23	7.50761119	6.25648303	6.00596622	6.98666955	6.50829964	6.02140976	6.09404434	6.44127704	6.70374746
RPL23A	8.79177336	7.47278138	7.72051555	7.97657907	7.62761259	7.38733127	7.70794993	7.69505807	8.41401822
RPL24	8.20325552	7.0001518	6.61044454	7.55008765	7.45378931	6.89155575	7.68844475	6.92506366	7.57340963
RPL26L1	4.05158117	3.66791117	3.1337122	3.9437059	4.3447715	3.88080496	3.83562681	3.76255737	3.84610893
RPL27	9.24117677	7.76448188	8.1835418	8.22238364	8.40964676	7.50024429	7.88259851	7.78661982	8.4114261
RPL27A	7.13272668	6.35091113	5.68121536	6.1167037	5.96609954	6.12748768	6.71757155	5.99981958	6.99041263
RPL28	8.61993412	6.91607471	6.94177303	7.21416695	7.39315805	6.83574215	6.70900418	6.03352602	7.85311057
RPL29	9.31908407	8.44199085	7.94926957	8.86457266	8.56104413	8.25207273	8.6660064	8.55727465	9.41083874
RPL3	9.07737321	8.12092398	7.80965737	8.55279152	7.92905835	8.20388976	8.30195962	8.04468278	9.08334747
RPL30	7.04515775	6.54412291	6.77152884	7.43298787	6.8519073	6.83523539	7.35944844	6.57074526	7.00975714
RPL31	6.60554317	6.36145743	5.47381922	6.46413204	6.35644208	6.04794933	6.43824744	6.16258256	6.50077359
RPL32	8.59943333	7.43544794	7.28551951	7.85011431	7.72999078	7.25202131	7.78419634	7.53359722	8.01294077
RPL34	7.08798608	6.6531041	6.03481098	7.00111367	6.87222447	6.35788385	6.42954209	6.6896044	7.0529281
RPL35A	8.12542545	6.71118967	7.49753938	7.33610839	7.23453798	6.46090437	6.96093251	7.14947228	7.21985245
RPL36	7.98270903	6.90697708	6.14938544	6.52411927	7.0355953	6.38726366	6.53908871	6.95423224	7.53892396
RPL36AL	7.75553614	7.56661787	7.26634686	7.94972644	7.95258514	7.40208394	7.1487354	7.95828104	7.71585396
RPL37	6.9602337	6.0079359	5.43954117	6.52179127	6.37383492	5.47632151	5.89033345	6.07511753	6.60836592
RPL37A	7.14916548	6.05937192	5.94078681	6.77871695	6.62488828	5.93175233	6.33142286	6.28629715	6.72099967
RPL38	7.73177895	6.08244808	6.08628337	6.51301507	6.53068768	5.65097207	6.05217092	6.75153281	6.66428705
RPL39	6.28824515	6.60979082	6.1391829	7.02093866	7.51702756	5.79695857	6.17113727	6.08194225	6.23493545
RPL39L	1.06782004	1.92020604	2.10627329	1.38718476	1.35401851	2.00438783	2.11710059	2.35213948	0.65648424
RPL4	7.87850528	7.35478269	7.5674036	8.2707514	7.71154554	8.20652096	8.02178524	7.17127859	8.59072566
RPL5	7.67849085	7.84564557	7.88092811	8.95363341	8.19375254	8.15443635	8.16979667	8.34348257	8.94836689

RPL6	6.94341211	6.92678134	6.47078081	7.45870671	7.28274818	7.09240753	7.12890087	7.35722846	7.4494869
RPL7	7.47451262	7.54534252	6.60949113	8.26364413	7.15184323	7.77655891	8.71340427	7.71313165	8.02938001
RPL8	9.37067352	8.24246116	8.5669434	9.25618655	8.00349711	8.58230034	9.48771535	9.48827666	9.24230728
RPL9	7.86244847	6.86903622	6.96393668	6.87924502	6.68981111	6.56134173	6.74223355	6.87209418	7.41978164
RPLP0	8.91742345	8.45657784	8.34587514	8.77166006	8.20605308	8.69730931	8.92333585	8.86039876	9.48067852
RPLP1	9.27278036	7.58011374	7.7725653	7.71602452	7.22304619	7.70169019	7.8832971	6.79092	8.27219956
RPLP2	9.1377831	7.93705672	7.42857744	8.17756054	8.11020472	8.02276641	8.66611123	7.79044996	8.88049142
RPN1	7.25578196	6.05336612	6.83729639	6.35688138	6.17189881	6.57466135	7.25150373	6.86774425	6.62365631
RPN2	7.22992393	6.7031223	7.71885433	7.47342211	7.14259947	7.0149963	8.12071666	7.25693145	7.52361548
RPP21	3.05831464	1.99032496	2.90474678	2.36785813	2.10903197	2.35797356	2.25572609	3.11183396	2.51001403
RPP25	3.73014573	2.79824886	4.01346274	2.79391953	2.79517257	3.50116429	2.98615593	3.65220156	3.44957758
RPP25L	3.7663543	3.77829075	3.6223128	3.44569456	3.77136476	3.7972854	3.27539164	4.07568006	4.7092146
RPP38	3.35441376	2.75818878	2.75880131	3.31387639	3.6116704	3.1787764	2.49728958	2.38054173	3.56511954
RPP40	1.31820548	1.93532853	1.86832591	2.65869412	2.50583992	2.46039486	3.09516373	1.8737531	2.2291178
RPRD1A	1.68721769	2.69861193	3.7188649	3.43973026	3.00356935	3.27587378	3.29100241	2.65629995	3.41700937
RPRD2	3.96049726	3.28199133	3.33930018	3.11658995	3.33462639	3.25644157	3.64960357	3.37136488	3.42257913
RPRM	0.0245653	0.31721057	0.12015115	0.01478823	0	0.41621273	0.05813612	0.05618572	0
RPRML	0	0.01897407	0.07051744	0.01986748	0.11826535	0.04819141	0	0.01279453	0.10150491
RPS10	7.4226575	6.29664867	6.30669717	6.69491363	6.43633196	6.34922968	6.88765309	6.79533558	6.88184682
RPS11	9.78614693	9.09213684	9.33552792	9.37057955	9.63660287	9.00060945	9.10450926	8.967116	9.88869324
RPS12	9.93034353	9.28539662	8.4633176	9.56985765	9.41857341	9.46925019	9.77263449	8.47944476	9.99655453
RPS13	7.82825855	7.97180661	7.48063349	8.11090048	8.09414213	7.70353158	8.24997829	7.43007966	8.42539643
RPS14	7.5022881	6.74898974	7.02374406	7.43525349	7.29974034	6.91570179	6.88907018	7.16442686	7.54942698
RPS15	9.35264774	7.4766978	6.81019159	6.838165	7.34390779	7.34355091	7.52431749	7.8074884	8.40361591
RPS15A	5.6820647	5.91581383	5.32759061	5.98083327	5.82751595	5.41692826	6.15651316	5.54325511	6.05458869
RPS16	8.40406933	7.49561508	7.83191773	7.91011387	8.24770908	7.58518515	7.76026645	7.62231944	8.42220062
RPS17	8.07666814	6.85244608	7.17889915	7.54672485	7.3477879	6.86930998	7.19422811	7.21847066	7.88836086
RPS19	7.62466836	6.6007527	6.62770513	7.04476493	7.30603314	6.78908077	6.80591169	6.56944915	7.19858256
RPS19BP1	4.47903278	2.8110404	3.17343753	2.61521701	2.48405228	3.0834169	2.50732097	2.84943128	3.32979021
RPS2	9.99171694	8.91284259	7.33644159	8.81156271	8.14934422	8.95518418	9.76909483	8.95943696	9.86468433
RPS20	8.53421839	7.76719601	7.38628034	8.50316413	7.78113827	7.84367138	8.17931735	7.47019887	8.01413054
RPS21	8.73455729	7.57970033	8.72595547	8.16858544	7.85096828	7.54330101	8.25079143	7.81343502	9.02891732
RPS23	6.19159522	7.41323251	6.3250953	7.6507491	7.05867361	6.67967653	6.50883184	6.6110926	7.45587088
RPS24	7.71078726	6.88964118	7.07096946	7.75695829	7.38692532	6.53563477	6.93380402	6.91732517	7.12799016
RPS25	8.70823747	8.09714393	7.28472275	8.25688558	8.06781027	8.03439568	8.34714721	7.05096409	8.80167205
RPS26	8.04314391	6.43576535	7.36510845	5.9984784	7.18350536	6.8061057	6.79392165	7.37418686	8.23270112
RPS27	9.70781772	8.76716881	8.1725994	8.36873788	8.28159168	8.24676495	9.12109474	8.87763077	9.09199919
RPS27A	7.39537901	6.87240934	6.87337427	7.17513715	7.04604396	7.25344866	7.43512992	7.03206035	7.49471409
RPS27L	2.55028292	3.91060429	3.3533659	4.08910866	4.27185392	3.45872509	2.98227644	2.33795161	3.43508639
RPS28	7.955041	6.20087178	5.96787232	6.30646299	6.088106	6.17131507	6.64367713	6.72297492	8.0526276
RPS29	5.95628515	5.10132328	4.82370142	5.15744371	5.18311232	4.49464085	4.85278174	4.9172919	5.59991847
RPS3	8.14877073	6.99632243	7.265423	7.62119185	7.15438869	7.02761152	7.9262914	7.27459677	8.1687366
RPS3A	8.21781248	8.02315238	7.75754386	8.07719433	7.62760137	7.85376184	8.30658032	8.20137473	8.88385056
RPS4X	8.82528202	9.0152671	8.37326708	9.56496595	10.2090107	8.34460644	8.70704768	8.7480758	10.277193
RPS4Y1	5.89875778	6.1229257	4.48770985	0.04727272	0.02930691	5.97891213	0.00955779	6.88769826	0.04150025
RPS5	8.17781163	7.2738149	6.9370266	7.6944078	7.95437535	7.4490282	7.48246824	7.27133022	8.40277112
RPS6	9.07935954	8.71756676	8.36082465	9.51978133	9.12117735	8.57508516	8.78748731	9.8899112	9.54025707
RPS6KA1	3.693247	3.41575924	3.23448892	3.30018151	4.19543757	3.97184836	3.80963406	3.47254078	4.25131589
RPS6KA2	1.53150625	2.78364685	3.09517761	2.77156672	1.89257532	3.08981826	3.131527	1.61128226	2.47226168
RPS6KA3	3.28544213	4.07343449	3.60683823	3.63445573	4.20307991	3.86005357	3.78190889	3.75484654	3.81451322
RPS6KA4	4.6442944	3.19422152	3.59498506	3.17151152	4.12375061	4.19650267	4.43847695	4.02647748	3.78630011
RPS6KA5	0.45600401	1.10764765	0.89351732	0.59612166	0.95784421	0.74493017	0.81133649	0.6876645	0.73280371
RPS6KA6	0.04224184	0.65558587	0.23391697	0.22130116	0.03134601	0.21336578	0.01022768	1.01706394	0.01345633
RPS6KB1	2.4295354	2.41552377	2.90060189	2.73264828	2.88978206	2.79633424	2.47200707	3.56893748	2.76235983
RPS6KB2	4.07508304	2.40388209	2.91559787	2.76883664	3.02827638	2.62418836	3.7914526	3.25028866	3.29767954
RPS6KC1	1.83800173	2.347904	2.33491554	2.3214436	2.31973356	2.54900142	2.36882906	2.77537519	1.67461733
RPS6KL1	1.07567533	0.81391349	0.94116602	1.05688413	0.38792335	0.52474538	1.07844057	0.1849428	1.11983537
RPS9	7.63580071	6.6366513	6.25816449	6.7423718	6.49131072	6.66627202	6.53319803	6.02863999	7.60571833
RPSA	8.10090786	6.69131526	6.78060897	6.85934192	7.11426445	7.51088212	7.9495021	7.16590913	7.83096488

RPTOR	3.4256244	2.30247212	2.50459903	2.09818703	2.50103253	2.6331658	2.19088124	3.24497313	2.76430971
RPUSD1	3.82328436	2.98813354	2.59977774	2.83837294	2.76673501	2.9380611	3.44131958	3.27732618	3.76221888
RPUSD2	2.19618073	1.55648682	1.92659484	2.07865865	1.98987051	2.25610896	1.88714257	1.14544446	2.48979469
RPUSD3	2.97514707	2.26103138	2.19529341	2.91416284	2.80921051	2.68377156	2.73752821	2.89022148	3.11679971
RPUSD4	2.5951325	1.93928242	2.00297206	2.67659945	2.68683593	2.56210812	2.91679679	1.99649833	3.08294508
RRAD	0.61554524	1.65173796	0.86575515	1.62482572	0.12573169	1.95549021	0.85215794	0.6079903	0.83571758
RRAGA	5.13474406	5.8617334	5.0563482	5.50248077	5.09058216	5.60167796	5.05121806	6.2949366	5.4130212
RRAGB	1.51302143	2.35481589	2.08915109	2.16404252	2.77206958	2.05925482	1.54325091	2.23424989	1.68359362
RRAGC	2.12068352	2.43506774	2.83427009	2.55314776	2.53852603	2.62272225	2.23601703	1.51403235	1.79692214
RRAGD	1.04673791	1.59583938	1.81951016	2.69405756	2.08305512	2.16547427	1.63454212	0.72144735	0.6490232
RRAS	5.92290978	6.23401163	5.21793164	5.83714452	5.25709476	6.24545681	5.01344219	5.7248722	4.43708138
RRAS2	2.59470121	1.98343012	2.53584974	3.24153394	3.36866733	3.06346783	2.29851096	1.53908759	2.74146464
RRBP1	5.44317599	5.46280016	5.4058564	5.44695502	4.86599539	4.8238138	5.99330163	5.43964422	6.08809466
RREB1	2.5643809	2.19645198	2.32419513	2.17174952	2.87557034	2.73348252	3.20201799	2.99899942	2.8414682
RRM1	3.97289829	3.87646623	3.57623947	4.40743365	5.02257437	4.50638215	5.19554663	4.1037574	4.60826268
RRM2	5.1464166	3.03123269	4.42559836	4.71859112	5.38330352	4.52888918	4.82948914	3.73150432	5.14625706
RRM2B	1.87508619	4.47156572	3.71826405	4.14049556	3.78887354	3.88004973	3.69022637	2.85609781	3.19331157
RRNAD1	2.40507887	2.01959645	2.20506895	2.07022745	1.93126195	2.30604285	2.69979742	2.55480143	2.43509759
RRP12	2.29300919	1.33833485	1.5243816	2.00367938	2.17039791	2.46937156	2.04404609	2.15743554	2.68942962
RRP15	1.08515528	1.7852702	2.11642732	2.42855646	2.37988819	2.52616271	2.33339553	2.37112267	2.4993046
RRP1B	2.44624468	2.93826473	2.75125356	3.7195262	4.26959245	3.63414127	3.37823283	3.59108086	3.85128932
RRP36	4.69021206	3.85489661	4.77187836	4.22215762	4.46408252	4.22780519	4.72340722	4.6681506	4.07499853
RRP7A	4.95227969	2.73190605	3.46374449	3.42974625	2.99855403	3.23606802	3.09901181	2.56459491	3.55988984
RRP8	1.63324628	1.46473823	1.48176246	1.65038273	1.89892923	1.74672831	1.62485439	1.81869933	1.9160437
RRP9	4.31978447	2.56437669	3.89854791	3.44400959	3.35624821	3.57280102	3.66247769	3.83696729	3.89409333
RRS1	4.06015306	2.83608509	3.24378281	4.24918184	3.6122716	4.07082349	4.41206812	3.96495016	3.98647902
RSAD1	2.8620147	1.92556071	2.55955064	2.48343239	2.26407472	2.29997866	1.87262669	2.10211672	2.86617875
RSAD2	0.92512208	4.38024494	2.56454071	3.57798538	3.88294067	5.37151564	3.97924384	1.02807264	0.94126441
RSBN1	1.87326721	2.48117254	2.34146991	2.52682836	2.64258453	2.45406216	2.25127974	2.60446168	3.0806624
RSBN1L	3.00266798	2.34263774	3.09677547	2.19525519	2.55176048	2.31168789	2.34289464	2.52744512	2.55749377
RSF1	2.06826716	2.57255707	2.02063366	2.24308828	2.56145302	2.41368319	2.2443336	2.48838035	2.33151117
RSL1D1	4.4241886	5.18434834	4.6279298	5.19524565	5.31702003	4.92756623	5.00788669	5.01385943	5.4088753
RSL24D1	4.31089603	4.38669634	4.77814871	5.03552285	5.11923743	4.76395447	4.5775017	3.86788206	5.10606204
RSPH1	2.10623682	0.86267809	0.5380319	1.2751459	1.61224267	1.22386995	2.02929624	0.7906902	1.70574198
RSPH3	1.55797081	1.93801638	2.04593085	2.09299926	1.65053123	1.83440121	2.41495558	2.59834325	2.32983179
RSPH4A	0.012776	0.27369048	0.4297704	0.41814151	0.21957517	0.1855685	0.17349844	0.06763347	0.07235391
RSPO1	0.04643776	1.18234452	0.4565127	0.43120084	0.04296347	1.25410923	0.06220239	0.04035598	0.0306796
RSPO3	0.9424482	4.40168295	1.88460923	3.38268589	1.08476841	3.76196541	1.05313436	0.61915478	0.42493203
RSPRY1	2.49801852	3.28965934	3.14309475	3.46268025	3.63611025	3.31463619	3.29061843	3.12985195	3.57586212
RSRC1	3.11386921	2.26575377	3.56939426	2.6766227	3.0073035	2.60811402	2.75380357	3.16765337	2.91443878
RSRC2	2.39899314	2.61785676	2.98082248	3.30313348	3.24081066	3.02106334	2.80834067	3.28614881	3.24967996
RSRP1	1.5783759	1.3566952	2.38312029	3.52081986	2.80440093	1.71287908	1.25509773	1.70021612	2.9495571
RSU1	4.85188426	4.85147265	4.09477166	5.021475	4.94376727	4.81232511	4.3587169	3.09792938	4.23894672
RTBDN	3.34219038	0.08338678	0.43077351	0.0717515	0.55130932	0.2350654	0.00813261	0.33993868	0.29086861
RTCA	3.70903862	3.09139497	3.72237233	3.98199822	4.48237758	3.72551467	3.92936672	3.56198003	3.98025844
RTCB	4.88979084	5.08345782	5.02122668	4.96863586	4.95297749	5.09090081	5.05591841	4.33594854	5.25432899
RTEL1	0.34446847	0.17675375	0.37667088	0.27781788	0.26912259	0.24961029	0.3360892	0.29174885	0.42103111
RTF1	4.03273415	3.73804271	3.76468498	3.99652798	4.1012946	3.90020395	3.60646408	3.09070267	3.98622755
RTKN	3.26160602	2.36258974	2.75810846	3.52186733	2.95716309	3.27430811	2.86839418	3.8757359	3.99475704
RTN1	0.75843633	1.1004804	1.4878887	0.8007873	1.05328582	1.28574411	1.11055191	0.86403309	0.4060637
RTN2	2.63131265	2.1051496	1.66192044	2.00516014	2.66111446	1.96909762	1.37694464	2.29983531	1.27189628
RTN4	4.87505733	5.48316493	5.26507918	5.77852411	5.12671464	5.36292021	5.03163259	4.83853742	4.91155643
RTN4IP1	2.20038518	2.19445144	2.06684739	2.19224536	3.06407653	2.17088113	3.36562261	1.73894612	2.51315911
RTN4RL2	2.45328764	1.0559093	0.78132588	1.78960449	0.31176543	0.97118276	0.713557	1.43996573	0.87969701
RTP4	2.60213655	3.08514448	3.32644602	2.16720277	4.11381652	3.95660017	1.22827316	2.2063659	2.18732582
RTP5	0.0815606	0.03950712	0.03985887	0.02495411	0.24503738	0.06529905	0.04126185	0.02670189	0.02188628
RUFY1	2.59320916	2.67037069	2.81099376	2.98508547	2.9967704	2.86506737	2.59020549	3.1109534	2.72154644
RUFY2	0.62310992	1.17114997	1.867457	1.64309671	1.50192857	1.33959087	0.96458474	1.23175047	1.23338842
RUFY3	0.98306576	1.75138744	1.98727746	2.13911453	1.78136707	1.96839904	1.55276733	1.86863293	1.56784987

RUNDC1	3.54128	2.33067252	2.99546878	2.66152877	2.70680908	2.619012	2.67324511	3.24078141	2.63428601
RUNDC3A	0.1070626	0.24169523	0.34579041	0.12346994	0.06252519	0.26983943	0.07349231	0.13354725	0.14088119
RUNDC3B	0.07537412	1.09745746	0.87123035	0.31562886	0.16639632	0.54902955	0.16607513	0.2536829	0.68105506
RUNX1	2.33217715	3.47494059	3.28683639	3.67384168	3.31357758	3.61740264	3.1270806	3.53095604	2.10905387
RUNX2	0.72842511	1.25173781	1.91937769	2.13035737	1.36979981	1.78500567	1.47385211	0.95967724	0.59356021
RUNX3	2.45986353	2.7655586	3.25249831	2.72450407	3.55020621	3.10278995	2.32634226	1.65754304	2.23057249
RUSC2	1.46961332	2.6523215	2.5492617	2.35719493	1.91523268	2.84358574	2.00742416	2.02951283	1.7495975
RUVBL1	3.0535166	1.96436722	2.40071627	2.72066836	2.82138277	3.09954947	3.08432816	2.61104607	2.91318416
RUVBL2	5.14475976	3.35286326	3.89579255	3.88641848	4.36871449	4.35252596	4.41847828	4.29138854	4.62962745
RWDD1	2.86904456	3.37216795	2.29759248	2.8354741	2.98236955	3.05628128	2.79487493	1.92579971	2.7346803
RWDD2A	1.26509421	1.72058647	1.76275126	2.25238243	1.72185407	1.85812474	1.99888085	1.45678144	1.96257806
RWDD2B	2.05223647	3.0984968	2.26806774	3.48263483	3.04309078	2.87739313	2.2547933	2.65727428	1.46468744
RXRA	3.67693242	2.79459358	2.62985436	2.22834701	1.90582149	2.70120825	2.74047783	2.97084412	2.80167611
RXRB	3.47393409	2.74799333	3.60461091	3.33148803	2.9104841	3.33349075	3.03939083	3.69840693	3.40734934
RXRG	0.02678611	0.80851169	0.11267918	0.06346572	0.02476946	0.24180023	0.016094	0.05620275	0.02116099
RYBP	2.66223192	3.04782046	2.95269684	3.32284968	3.09087517	3.15706203	3.1875243	2.9183112	2.97618809
RYK	3.22067208	2.86663137	2.87604398	3.24991209	2.7469194	2.81190445	2.9750413	2.50190029	2.93918457
RYR3	0.01580416	0.37466258	0.29556508	0.15237671	0.09994635	0.27416358	0.0202412	0.04479524	0.07668163
S100A1	0.13756441	0.21706738	0.46411275	0.3278018	0.28180537	0.20559754	0.21375456	3.14150509	0.17260009
S100A10	8.52153471	7.64335473	7.79791348	8.89740518	8.85574196	7.29160356	8.45378343	7.18161411	7.74191849
S100A11	11.2388164	9.56209718	10.1809386	10.8395473	11.276249	9.91097807	10.2716276	10.5748505	9.41761176
S100A13	2.94218883	3.24565986	2.61081024	3.71720876	2.07458664	3.86750406	3.10571166	3.70224852	2.16283819
S100A14	3.38896356	5.63948174	3.11443517	5.11564001	5.46403561	7.07813528	7.35995136	7.00967558	6.65105683
S100A16	5.64881041	6.1419803	5.19165599	7.42492535	6.27123819	7.56059664	8.46002324	8.21401576	6.63526233
S100A2	2.27885803	0.80567622	2.24092397	2.3342441	1.06518678	2.63791278	1.34163111	1.33472763	0.69473753
S100A3	1.04130091	1.89813439	1.08917701	2.24651021	0.63603386	1.57190739	1.81461749	1.92739081	0.90763199
S100A4	5.38843143	6.47054448	5.4197855	6.05085242	5.30833565	5.76935559	6.11460844	4.52590859	4.53631662
S100A6	10.2049868	9.01322547	8.36919615	9.78536541	10.0809318	10.3343992	9.85479615	9.19170337	9.0269416
S100A8	5.24367449	4.63672641	3.73139946	5.72972789	3.8083694	9.06380378	3.33973555	3.29669371	2.54699179
S100A9	6.6525168	6.95135252	6.00514879	7.21980418	5.56323074	10.260911	6.44403639	5.72164096	5.24811347
S100P	3.0940748	5.57213038	7.73011301	7.21464311	8.04181772	6.83588756	8.5490835	8.5467255	2.18518434
S100PBP	1.44798121	1.94021724	1.90203394	2.10606566	2.43681668	1.94936756	1.39683629	1.15190843	1.69214912
S1PR1	1.96485728	4.54289552	4.03021413	3.83498202	3.1855299	4.37091811	2.81393186	2.4415503	2.26971262
S1PR2	2.13957188	2.0976663	1.9985267	1.61976729	1.86453668	2.03439227	1.51475826	1.59564426	1.20236095
S1PR3	0.85899727	3.05387041	2.96822851	2.18341234	1.07311761	2.76278095	1.6713765	1.13755917	0.91172184
S1PR4	1.94638984	1.45373846	1.73819259	1.03381693	1.60350787	2.96750961	1.20702988	2.24277174	0.93936556
S1PR5	0.44372343	0.23938198	0.80030798	0.21665672	0.26262447	0.41942798	0.27521558	0.42228066	0.18552817
SAAL1	2.00715485	1.68281891	1.88247274	2.70799522	2.54997799	2.8402855	2.88337571	2.13222918	2.70752193
SACM1L	2.27237502	2.82086502	3.36722384	3.0908934	3.33020777	3.09372701	3.35546281	3.36800104	3.12689961
SAE1	4.61112803	4.31076142	4.55204526	4.63540919	5.14572426	4.79010521	4.4695866	4.45892364	4.50317487
SAFB	3.4138715	2.77282178	2.90944057	3.00516168	3.45879962	3.1047422	3.15632004	3.28331243	3.45774958
SAFB2	3.09689947	2.34259093	2.54810387	2.53085862	2.75599962	2.64683753	2.59817162	2.94321082	2.83244292
SALL1	0.15261184	0.02920852	1.09642821	0.39318952	0.21346859	0.07603377	0.90790376	0.61983974	0.37361936
SALL2	0.19006142	2.12010998	0.57367642	1.25598801	0.26939223	1.03945258	0.33812426	0.23989433	0.11949189
SALL3	0	0.00281942	0.17099213	0	0.00303205	0.00361604	0	0.01886572	0
SALL4	0.7521169	0.1786649	1.6931322	1.44386216	0.53823257	0.75918139	0.81221446	0.47591228	0.06457477
SAMD1	6.14093023	3.47698779	4.07858572	3.75600852	3.87017166	3.90120985	3.9874309	3.50099299	3.89159355
SAMD10	3.43486533	1.75332184	3.57015565	2.0252859	2.49183248	1.82177747	1.84363854	2.40777353	3.22368366
SAMD11	1.5982959	2.45644792	0.82097131	0.64261425	0.29581218	1.43193783	0.56091377	0.51006128	0.12003413
SAMD14	0.32428196	0.68748557	0.83850032	0.35463823	0.23838034	0.48766439	0.27646406	0.34552003	0.29749067
SAMD3	0.11171192	0.46842675	0.34145174	0.42728659	1.21200015	0.4930446	0.23833495	0.06951784	0.32643732
SAMD4A	0.81364298	2.07959733	1.2213709	1.77859715	1.22816068	2.31028141	1.41884453	1.53966625	0.8159737
SAMD4B	4.28228836	3.69590083	3.77548537	3.74478172	4.11898161	4.02198394	3.80958067	4.2201074	3.75706452
SAMD5	1.58618881	2.05325254	2.5229286	1.98259973	0.10282171	2.06639641	3.5974698	2.40045296	4.14216446
SAMD8	1.16101903	2.30677895	2.62908021	2.37712779	2.57492837	2.26675988	1.95424442	2.1523538	1.75291241
SAMD9	1.99590378	3.98274137	2.84118417	3.18354451	5.09715224	4.46178264	3.48490117	3.2798146	2.55282325
SAMD9L	2.08151319	3.93904486	3.12977828	3.33353874	5.69416621	4.39746587	2.38396593	2.21392859	2.50696496
SAMHD1	4.72941558	5.73945238	5.11464486	4.83649222	6.5988028	5.58844815	5.11354949	3.65903821	4.35523121
SAMM50	3.47615395	3.1598938	3.13228409	3.34909815	3.40349218	3.35491007	3.19444416	3.63656883	3.57879735

SAMSN1	2.02468572	3.93726985	4.21855924	3.29135817	4.53554855	3.70366022	3.04160043	2.2210344	2.37925861
SAP130	3.46994155	3.30080591	3.66371241	3.36633434	3.92694813	3.27421585	3.69458776	3.42951148	3.4601109
SAP18	6.13068483	5.1201909	5.15484631	5.25154469	5.30955205	4.91703827	5.44964896	5.43781876	6.05987758
SAP30	3.78891933	3.26381552	2.09653823	3.27332546	3.37308695	3.59224515	3.78500723	1.3829307	3.5734308
SAP30BP	3.43724784	2.47119506	3.02518519	2.87842304	3.17376161	2.82923914	2.4778232	3.17942695	2.70199515
SAP30L	2.40719042	2.7670688	2.45192955	2.2826841	2.56975593	2.91623551	2.17603422	2.56368195	2.60952623
SAPCD2	4.81585937	1.49570397	3.59107288	3.12428651	3.28566561	2.56163973	3.31954426	2.45445436	3.90486959
SAR1A	3.18068618	3.88529704	4.31737292	4.02355036	3.47622417	3.9330361	3.92443598	3.49388514	3.40582577
SAR1B	2.44247158	2.84179968	3.46663775	3.07281506	3.74427094	2.95377412	2.9877963	3.08808333	2.93445988
SARAF	5.95321018	6.37207945	5.93609863	6.98322587	6.25582877	6.38550515	7.21314836	6.31530167	5.98256712
SARDH	0.63926377	0.73722939	0.61122671	0.34747612	0.50533511	0.89615404	0.34006447	0.18512782	0.25641804
SARM1	0.26800829	0.69461195	0.83985666	0.67081745	0.40510555	0.89213145	0.65599937	0.75682897	0.79645929
SARNP	0.46401176	0.44591027	0.82690734	0.79236484	0.58447879	0.6085842	0.47237998	0.70044719	0.5929285
SARS	5.27161866	4.59887407	4.9355811	4.28060138	5.39743542	4.55905124	5.13917883	5.1315257	5.59111487
SARS2	1.42696566	0.5604374	1.06362755	0.97548414	1.28697123	1.05468648	1.00786227	0.97843156	1.46841584
SART3	3.07579846	2.59950335	2.89740531	3.0555907	3.2146011	3.13241711	2.72126551	3.44000451	3.26737462
SASH1	1.27077271	3.49332276	2.66684468	2.53082166	2.57762499	2.98320801	2.48231381	1.85027177	1.89688488
SASH3	3.28399586	3.00530148	4.00294638	2.79201341	4.2437144	3.56219812	2.94678152	2.19102281	2.8569777
SASS6	1.39753315	0.89510221	1.88102902	2.06297423	2.53983575	1.84646274	1.8526673	1.49258432	2.11202208
SAT1	6.84918154	6.18837294	8.30695755	7.88202225	8.56806758	7.05933654	6.80553765	8.56141577	6.88209249
SAT2	3.61164639	4.37409845	4.42290885	3.95428679	3.776294	3.79907198	3.09698805	3.26992947	4.14610192
SATB1	0.56782587	2.53388287	2.08477108	1.83465627	1.63689033	1.98241768	2.99467707	0.79297362	2.48639371
SATB2	0.14239972	0.74054513	0.58622956	1.25701469	0.17897157	1.20758362	1.21280615	1.29438569	1.75853252
SAV1	2.67556042	3.2289859	3.62406104	3.26721126	3.16229651	3.30419244	2.41115066	3.32013098	2.74833768
SAYSD1	2.679523	2.63554708	2.73400901	2.46286232	2.32527675	2.55124662	2.4397276	2.88513001	2.68672686
SBDS	5.46269147	6.51338433	6.13207005	6.15444826	5.883943	5.98408772	6.25862111	5.86833112	6.25130978
SBF1	4.52975789	3.15618322	2.54467013	2.920716	3.38408405	3.62094358	3.48621807	2.6962797	3.90391332
SBF2	1.77639032	2.41053552	1.5390041	2.0373619	1.84006477	2.21662092	2.43303171	1.58071175	2.11387852
SBK1	0.87575869	0.7838801	0.24220476	1.62987786	0.59575376	0.49852585	0.71562324	1.57703056	1.65500564
SBNO1	2.07185506	2.18540063	2.59809264	3.08589373	3.19384897	2.69753685	2.92905888	3.11338894	2.53807722
SBNO2	5.17476138	2.82680237	3.94588936	3.32004538	3.90376767	4.23384443	3.80281501	4.57899264	4.21171167
SBSPON	0.08084277	3.04897052	0.6473593	1.99176756	0.29541614	0.43233399	0.93159073	0.2788814	0.43124614
SC5D	3.29349416	2.69602764	2.52386284	3.79827942	3.07294387	3.04099958	3.87730111	2.60237325	4.09255934
SCAF1	5.06844467	3.35111253	3.51974803	3.02645891	3.82377141	4.07285507	3.52566514	4.11038325	3.99323714
SCAF11	2.82413495	3.35581054	3.25332422	3.51262441	3.86072717	3.42856379	3.32407722	2.65106213	3.59327702
SCAF4	2.56321133	2.67298482	2.62409699	3.21099002	3.45948272	3.12923649	2.84366091	3.21583563	3.63953065
SCAI	0.73621851	0.80502601	1.25092936	1.03102001	1.16507588	0.96188825	0.7220625	0.6915126	0.93674947
SCAMP1	2.73977949	3.81599672	4.16271412	4.03660745	3.48164394	3.93621115	3.80962609	4.70954885	3.77293776
SCAMP2	5.26994544	4.79872254	5.43876966	4.31641185	4.76705758	4.76368763	5.26096037	4.60213905	5.7199083
SCAMP5	1.2936796	2.64520338	3.72064204	2.35379185	1.63142192	2.23117697	0.87314645	1.91581034	1.94801516
SCAND1	5.1466875	4.17398214	4.20237281	3.5641126	3.23221575	4.07806457	4.09776714	4.14624357	4.64092145
SCAP	4.58980778	3.86853133	3.66464798	3.43350429	3.40500775	3.71032445	3.7649738	4.03150184	3.91751842
SCAPER	0.80970025	1.43694632	1.34513305	1.07250307	1.18909983	1.37746833	0.97654261	1.20207192	1.49551413
SCARA3	1.34931717	3.99556613	1.5003629	2.64789828	0.34948618	2.97737634	1.21442699	3.4444183	0.26020122
SCARA5	0.01634225	3.92374341	1.06669323	1.92164637	0.09311469	0.44800606	0.11347607	0.21989785	0.02992376
SCARB1	1.80724734	2.79348176	3.14083673	2.92674223	2.96218958	3.06013258	3.62868012	2.75875443	3.92139924
SCARB2	5.35257726	6.26801411	5.31142951	6.03447837	6.15804519	6.11239169	5.79302489	5.3416924	5.71231928
SCARF1	1.42517608	1.2665545	2.0373563	1.55578307	1.50977307	1.70242791	1.22723646	1.07511929	1.15610461
SCARF2	2.34068392	3.50571027	2.19063149	3.30903622	1.21226464	3.67638206	2.72827416	2.09111258	1.07403505
SCD	6.17413762	4.67200018	4.58386693	5.54402539	4.68118656	7.38789721	6.12342933	6.27450187	6.3960856
SCD5	1.82722484	2.60742006	1.62858926	3.24249406	2.16959885	2.37722835	1.10101683	0.59277257	2.42283263
SCFD2	2.69437039	2.57007162	2.5106459	2.1843133	2.54770602	2.3952169	2.24238565	2.83249728	3.03708838
SCG2	0.16450386	2.53743451	0.63887799	3.12526594	0.50948529	1.61601029	0.77557868	0.27652166	1.3355302
SCG3	0.04331891	0.68020409	0.17200848	0.07705569	0.30966543	0.28498033	0.05804588	0.02936023	0.02860862
SCG5	0.57980887	3.40963872	1.51264195	2.07828442	0.62160556	1.67901218	1.13630732	0.40453141	2.50130835
SCGB1A1	0	0	0.49329924	1.44292329	0.14659982	0	0.03700612	0.0474802	0
SCGB2A1	0	0.11545933	1.01140295	0.19591478	0.12381219	0.0751159	0.46186355	3.22550149	0.10629429
SCGN	0.57949416	0.8248386	0.54166373	0.79603263	0.01507059	0.11292519	0.01464617	2.57694075	0.01286877
SCHIP1	0.02832313	0.37745772	0.4211469	0.29395963	0.11021513	0.5304279	0.09923868	0.10964238	0.05168479

SCIN	0.17961162	0.95414915	0.86615153	0.4070696	0.11326626	0.41121761	1.2176927	0.39134699	0.3128926
SCLY	0.38960981	0.39449479	0.50587469	0.50438832	0.66683158	0.40009623	0.35723043	0.35294208	0.75538945
SCMH1	2.79887414	3.37721022	3.85534624	2.72979726	1.06959613	3.23312474	2.31867306	2.43432707	3.00198229
SCML1	1.43314772	1.86402631	2.77331098	2.45643793	3.56802372	2.40294346	1.30873647	2.29545027	3.22096813
SCML4	0.32837604	0.45212186	0.49343472	0.18705744	1.20924637	0.38860852	0.48710904	0.13555196	0.7153716
SCN1B	0.67881051	1.48489044	1.0872143	1.0253957	0.74240725	1.71713179	0.56302038	0.90584518	0.20793713
SCN2A	0.0318995	0.14831064	0.09826161	0.08206375	0.00360827	0.05496315	0.0052562	0.03240757	0.00615207
SCN3B	0.03424839	0.3350816	0.1353376	0.22620191	0.05812146	0.49262992	0.08938018	0.32902725	0.04778026
SCN7A	0.00886605	1.59018702	0.46315323	0.60546585	0.0190491	1.31568159	0.01851335	0.06533755	0.03464084
SCNM1	3.12203976	3.10666071	3.42231794	3.34659691	3.0886656	2.84002805	3.12444297	2.76871014	2.95817906
SCNN1A	1.00122482	2.67091705	4.85953808	2.42820016	0.34499014	0.17167826	0.74794661	4.62234957	4.46816055
SCNN1B	0.23943835	0.31040627	0.81879895	0.43097572	0.21273143	0.29563336	0.05463423	0.14322556	0.14854531
SCNN1D	0.17059693	0.13364913	0.19547138	0.53789573	0.24560258	0.15743312	0.0842161	0.38915779	0.2079594
SCO1	1.78022834	1.86537211	2.27424705	2.44127524	3.24062356	2.55591274	2.20282859	1.57005683	2.67095619
SCO2	3.99137237	2.72078853	2.71774693	2.72275909	4.23029281	3.4265728	3.08365192	2.43857766	3.75467212
SCOC	2.79663606	3.32201096	3.92432567	2.85533286	3.71890796	2.44915256	2.98670719	3.72135063	3.51958827
SCP2	4.50383643	4.32129207	4.13644527	4.14566049	4.56623288	4.14005198	4.02762815	4.19056285	4.42876584
SCPEP1	5.33571763	4.65041865	5.9202862	3.91588985	5.10512446	4.18165844	4.11394382	2.74488107	2.77575207
SCRG1	0.05954091	3.32994846	0.37212305	1.48273437	0.06109972	2.07726068	0.05940537	0.11021916	0.13867421
SCRN1	1.02884155	3.67363261	4.21548323	4.10317831	1.93786557	4.34157673	4.29909236	5.0092568	1.4642461
SCRN2	3.74800755	2.54966849	2.07748261	1.98675566	1.67534817	2.42517011	1.63552968	2.47825279	3.22594342
SCRN3	1.53398608	2.53894958	2.83400798	2.84929153	2.33149984	2.7110508	2.13182105	2.87280533	2.51921686
SCTR	0.66892025	1.47848649	4.37486931	0.01902061	0.13144557	0.0612194	0.00635381	2.21604047	0.13342551
SCUBE1	0.18044114	1.15200146	0.56536852	0.38099982	1.19623516	0.40273463	0.43579439	0.19056614	0.32637823
SCUBE2	0.16018469	1.72656682	0.88626811	0.66639989	0.11835332	1.49368625	0.25561206	0.56696222	0.71343391
SCUBE3	0.80340392	0.55208144	0.76944978	0.34237854	0.16697825	0.80798569	0.4542055	0.41383506	0.13892846
SCYL1	4.63664416	3.93522851	4.49938402	3.92299781	3.85850518	4.29726503	5.02772511	4.36966418	4.34367428
SCYL2	2.79740564	3.31290127	3.51351736	3.66314283	3.95165725	3.75048754	3.53547975	3.69816451	3.61327713
SCYL3	1.34566201	1.4367157	2.25574585	1.82237464	1.76698528	1.43895675	1.69248612	2.35710788	1.79706661
SDAD1	2.48227465	3.05369099	2.75615448	3.59602161	4.00366952	3.34096664	3.28025771	3.20113922	3.10300419
SDC1	7.47556828	4.90689795	6.85516024	5.98147629	3.86417364	5.31880696	6.32714177	6.5105369	5.89587432
SDC2	2.1655248	4.28909026	4.00214299	4.3939733	2.79753536	3.9021935	4.41430062	2.80722556	2.64626948
SDC3	4.80914307	4.79627468	3.70398005	3.93548025	4.45707498	4.9822436	3.75036972	3.05942032	3.04847914
SDC4	6.85626425	4.78732606	8.54064219	7.51477379	6.77370705	7.60985016	5.83367843	7.06368419	6.63254775
SDCBP	5.87939249	6.33156073	5.79520516	6.56405824	6.09342169	6.5307182	6.80337967	6.64535487	5.2564797
SDCBP2	5.66756131	3.6454404	4.31350195	4.42455133	4.58498149	3.49016477	4.33567896	3.87737543	5.05388257
SDCCAG8	2.2464429	2.36353408	2.69140394	2.51066981	2.39037566	3.11287721	2.87057041	3.29569536	2.01308342
SDE2	3.11402651	2.65121578	3.14961299	2.95323887	3.2311171	3.15559863	3.43900444	3.5799791	2.83589262
SDF2	4.39908136	3.54527228	4.24574604	3.87911858	3.45632282	3.45802511	3.37836506	3.77172179	3.4195173
SDF2L1	5.67617379	3.59685012	4.14483112	3.75722618	3.62390735	4.33485358	4.72323124	4.51840331	4.36609353
SDF4	6.06192916	4.65050363	4.46415482	4.61328031	4.41130885	4.77985646	5.5573853	4.21767187	4.86375783
SDHAF1	3.77953625	2.66971991	3.59623941	3.15548748	3.22425849	3.31821243	2.82068377	2.87174271	3.85130313
SDHB	5.31750692	5.16328537	4.80801039	5.28131938	5.95825551	5.45189042	5.43549449	4.48534039	5.78936234
SDHC	1.91273836	2.63544244	2.81683898	2.63517891	2.68291765	2.85943332	3.64858779	3.22944236	2.73922618
SDHD	4.79294107	5.55101738	4.72569977	5.15554495	5.74026273	5.47636354	5.84527279	4.6541995	5.73789696
SDK1	1.12373492	2.39436945	1.14730307	1.52718967	0.42705011	1.71573997	0.70429153	1.17737163	0.29875033
SDK2	0.07046859	0.41181969	0.53575565	0.15523216	0.06985129	0.33512989	0.10159339	0.20182409	0.10194114
SDR16C5	0.08628478	0.90932495	0.6280361	4.30087933	3.69758452	2.76373618	2.69468108	3.57652606	0.61399553
SDS	3.20984307	2.99025136	4.62766363	2.40893299	3.11385176	2.94261456	3.05474093	2.21632755	1.91105197
SDSL	3.42888447	2.52937995	2.6044315	2.35151156	2.24583089	2.48084054	2.21536033	2.16342966	2.0391037
SEC11A	4.40919907	3.87745508	4.49523289	4.37735787	4.41426948	4.13059527	4.27679459	4.12354595	3.68041675
SEC11C	2.5190444	2.45784995	3.17745727	3.63691582	3.60737691	3.21727198	3.88631955	3.40398811	3.16293547
SEC14L1	3.13821797	3.04549234	3.45213355	2.96681179	3.71353367	3.12710022	2.99936018	3.15109873	2.14583576
SEC22A	1.99821623	1.78343497	1.96130918	2.04023462	1.75775577	1.99395406	1.78233859	1.90853852	1.48273147
SEC22B	2.60294952	2.81230276	2.62473601	2.68494476	2.59177071	3.08470071	3.02457084	2.76836839	3.02745348
SEC22C	2.15753786	2.32833984	2.37564038	2.4780606	2.50704001	2.65276914	2.24662175	2.3779929	1.71801976
SEC23A	2.67545791	4.37086515	3.09061117	3.96254931	3.69241813	3.94868483	3.97121865	3.38904706	3.43993746
SEC23B	4.13744695	3.99645177	4.66009428	4.88298181	4.96364801	4.76259603	5.37002617	4.51485656	5.40969202
SEC23IP	2.67975982	3.52663962	3.66851466	3.61730491	3.6576182	3.58334891	3.5537392	4.34293045	3.57108149

SEC24A	2.82007101	3.29001133	3.8054471	3.60346465	4.27620704	3.53593317	3.78212125	3.95064658	3.70275779
SEC24B	2.90886176	3.73624931	3.60972654	3.98040958	3.99854168	3.84752444	3.61207366	4.31171883	4.04836138
SEC24C	4.03059659	3.51229329	4.08397268	3.37619699	4.18544678	3.93390423	3.98033181	4.36871714	4.16374296
SEC24D	2.79886227	3.60461336	3.82636732	3.52038357	3.32916696	3.83967993	4.42492202	3.87867008	3.55211682
SEC31A	4.42511358	5.19194475	4.34612669	4.70256517	4.4548056	4.21976872	5.16717631	5.15972751	4.01942731
SEC31B	0.31250346	0.20326087	0.6946314	0.82017797	1.12835432	0.31948727	0.22829434	0.33205191	1.21713075
SEC61A1	6.94548364	5.94845641	6.21313462	5.7676467	5.79543808	6.23240471	6.7497882	6.7867987	6.2909764
SEC61A2	1.27067665	1.03780008	1.60248157	1.74550169	2.11179655	1.21572547	1.04938565	0.81827433	1.35983851
SEC61B	6.63322745	5.74618535	6.02066322	6.01326482	6.17772821	5.94459235	5.59613657	5.12335102	5.950366
SEC61G	5.3706829	4.52869001	4.86683434	4.9375969	4.98727983	4.1700363	4.99550203	4.72062658	4.62699658
SEC62	4.94481846	4.68676271	5.62120221	4.5283368	4.04056946	4.20298914	4.28242157	5.02626611	4.08676805
SEC63	3.18988906	3.69364084	3.21155392	3.48290319	3.68194298	3.30792588	3.6777364	3.33567538	3.63474819
SECISBP2	1.92142864	1.73990505	2.82295583	2.14739616	2.0496508	2.02042095	1.93765087	1.67614034	2.83850622
SECISBP2L	2.02896615	3.28655358	3.11434293	3.19519019	3.41104949	3.2532085	2.6763417	2.7564747	2.11967351
SECTM1	4.95800795	4.18831744	5.11724343	2.9966871	3.57358162	4.47491141	3.65745893	3.7302676	4.12358984
SEH1L	1.8569223	1.82891493	2.35710575	2.81678009	2.62807228	2.66791092	3.50835076	2.26286095	3.05837706
SEL1L	3.38511484	4.12288969	4.5412051	4.34838871	4.39647157	4.25067443	4.41221333	4.87320723	4.20912859
SEL1L3	5.47202639	4.78659329	5.87296747	4.46476458	5.02184766	4.54648474	5.06457054	6.44741444	4.93150563
SELENBP1	4.71290805	4.42592113	3.89738838	2.8536879	2.38874066	2.59348538	3.57460031	4.65927835	4.931344
SELL	1.81620528	4.53282952	3.43721491	3.7007029	4.58105837	5.23849056	2.43014834	2.83559651	2.2529933
SELP	0.22216314	2.96416757	2.69025482	1.60663032	0.60992106	2.99949519	0.67621322	0.46405294	0.39593187
SEMA3A	0.24282796	2.37593589	1.18751638	1.11077688	1.54830758	2.08636707	1.8124995	0.41972393	0.37580719
SEMA3B	3.2055412	2.55009907	2.12023181	3.10988479	1.78990352	1.60732915	3.28440792	5.00236057	2.40482525
SEMA3F	4.18342121	3.01899315	4.44922505	2.44626887	3.39496148	3.03067092	2.78860282	4.66109823	3.43120956
SEMA3G	0.47319106	1.37033512	1.04254313	1.25595916	0.53017583	1.61340219	0.92315988	0.80414588	0.47734655
SEMA4A	2.1553465	2.27844774	3.16713375	2.74084187	2.46222174	3.06499654	2.14488429	1.70833436	1.4375168
SEMA4B	4.75647587	3.52385532	3.7719179	4.22061491	4.13364637	4.95829152	4.35032922	4.87475492	4.26436084
SEMA4D	2.31980193	1.35292052	2.81789418	1.86286806	2.27501398	1.75957409	1.34479873	1.03123414	1.71602059
SEMA4F	1.52927833	0.5252527	1.14051359	1.60619515	0.48271161	1.45547432	0.50847562	1.51870769	1.13506224
SEMA4G	4.6057158	2.16939142	2.4601469	4.01630741	3.87092962	3.12691649	3.31593091	4.24814949	3.98924076
SEMA6C	0.40250978	1.32740659	0.86394346	0.92608966	0.30538932	1.24933607	0.42612352	0.57960947	0.64368901
SEMA6D	0.0947443	1.65544271	1.12651474	0.82822635	0.37848336	1.32232678	0.48464776	0.24121565	1.63387902
SEMA7A	1.53578713	1.31086668	2.84133791	2.51694628	1.67945362	2.55976542	1.54404437	1.77303029	0.94446265
SEMG1	0	1.18892481	0.04676119	1.10977737	1.40999286	0.07840045	6.55802114	0.48373509	1.76758295
SENP2	3.53860835	1.96363564	2.95965714	2.34127703	2.56376171	2.35570761	2.62279708	2.9432579	2.51493742
SENP5	2.88451095	1.90530665	3.37404943	2.53817282	2.88875699	2.34580328	2.38272054	2.52387245	2.21911275
SENP6	2.25372251	2.76335429	2.42835028	3.36087033	3.23936595	2.87378506	2.85044189	2.21592929	3.14970311
SENP7	0.3521629	1.88004519	1.98929779	2.31572445	2.32825283	2.23147808	1.21219177	1.96793653	1.64522418
SEPHS1	3.87739363	3.52154962	3.11527567	3.59156999	4.19814081	3.63855485	3.47560286	2.71904956	4.41972186
SEPHS2	7.31689508	5.87468276	5.37182122	4.70769771	5.96293979	4.80534628	5.31116159	6.11969348	5.04719432
SEPSECS	1.21754765	1.72289107	2.07671693	2.14699726	1.98609576	1.81923647	1.49810378	2.22387004	2.55616577
SERAC1	0.99744526	1.34637007	1.8765184	1.50309045	1.73364307	1.5970214	2.0552901	0.81232716	1.46695633
SERGEF	1.64190335	1.70493631	1.8565394	1.76083371	1.2332293	1.8136222	1.77137459	1.92165412	1.76075951
SERINC1	4.82350451	6.53664196	5.54874053	6.42143741	6.01708305	6.27765771	6.12021817	5.57352534	5.55272408
SERINC2	5.22172055	5.2838657	5.44388033	5.83642052	3.02636225	4.45846313	6.65976847	6.412377	6.23016466
SERINC3	4.46614953	4.96891384	5.46472089	5.54727252	4.83519633	5.03253821	5.37388902	5.50722664	5.60268666
SERP1	5.87597399	4.76995586	5.15415266	5.00913205	5.20655499	5.21490663	5.28513116	5.55988299	5.21161591
SERP2	0.22702828	1.539676	0.9401202	0.88707549	0.32233638	0.66249494	0.36776402	0.20361232	0.12377201
SERPINA1	7.73102992	6.42944727	9.35628528	6.77021615	5.14984861	7.42607474	5.62817652	10.1985124	3.89555077
SERPINA10	0.07778786	0.08877177	0.03329534	0.02377814	0.05237677	0.00244506	0	0.02291995	0.02085384
SERPINA3	1.70192412	0.26265501	3.79431194	0.20738104	0.42143442	1.02956606	0.03449029	2.60186319	0.00382629
SERPINA4	4.23315036	1.72617645	4.89215723	1.87097425	1.91926875	2.16913005	0.21623266	3.75816273	0.048343
SERPINA5	2.44627696	0.98642498	1.36246823	0.76432682	0.11978354	1.64808954	0.3475443	3.63075945	0.0151442
SERPINB1	5.65221597	5.85507791	6.01924687	7.81784546	6.92469069	7.43979751	6.73326331	7.83909893	7.16940112
SERPINB2	1.09133445	0.37281173	0.4491952	5.45552744	7.41963502	1.99976064	1.36081966	2.08230314	0.13943445
SERPINB6	3.70670845	4.7940429	4.71389889	6.05127666	5.74107271	5.62485719	5.68688445	6.07547903	5.75837896
SERPINB8	1.00419135	2.04500489	2.02149076	3.69326502	3.52661158	2.91859519	3.17904947	2.0435997	2.63827124
SERPIND1	0.05600032	0.20543074	0.33856933	0.51341883	0.1342619	0.09636539	0.29287585	0.18075819	0.07315028
SERPINE1	4.35229199	4.85757054	5.10267227	6.25889625	4.34958005	5.52967524	5.03200778	4.62839715	3.46086127

SERPINE2	0.6227815	4.65342642	2.8560368	2.82105662	0.68470173	3.7806731	1.78196715	2.05292165	2.18633795
SERPINF1	4.31938761	7.65618226	5.01150574	6.41110004	2.86949388	6.20576837	5.06907081	3.82337366	3.32558772
SERPINF2	0.56963415	1.35218584	0.99633385	0.89913363	0.46235648	0.41792878	0.68869792	0.1691553	0.81377249
SERPING1	6.41204752	7.91329891	6.23388498	7.47508452	7.4079233	7.52982417	6.5211169	5.38316505	4.74126256
SERPINH1	6.31128694	5.97070316	5.67068629	5.75503724	4.44649389	5.73968697	6.19636627	5.09314474	4.40793045
SERTAD1	3.51200082	2.53560478	3.53946911	2.70653838	3.07389591	3.5066792	2.62567886	3.2454393	1.96882291
SERTAD2	2.09676825	2.96457752	2.59899221	3.42621705	2.96464677	3.2274306	2.56012745	1.05987448	2.88713065
SERTAD3	3.7072366	2.35884467	3.44173275	2.84478648	3.35819874	3.28976995	2.61034815	2.71311703	2.67672236
SERTAD4	0.15172683	1.51234759	0.60797411	1.19128074	0.10682798	1.18696069	1.0305398	4.17483412	0.22353419
SESN2	5.38873205	3.07836493	3.17871733	3.02213248	4.52146638	2.9911758	3.09221806	1.71914105	2.65161706
SESN3	0.44738432	4.12275643	1.78747616	2.8622357	1.66952534	2.90305515	1.21800269	1.02199249	0.80125049
SESTD1	2.00259054	2.92224274	2.94686482	2.65114751	2.24595568	2.7676496	2.63114334	2.39727723	2.09824833
SET	5.96935418	5.59842294	6.02628823	6.09010221	6.46023323	6.30573708	6.14192131	5.30649397	6.14651147
SETBP1	0.24857132	2.60381734	1.05104251	1.47674906	1.09962842	1.57541028	0.5403168	0.48622077	0.36798912
SETD1A	3.41923544	2.77883906	2.67541135	2.64426399	2.89591422	2.72691874	2.68739393	2.8035864	3.13500019
SETD2	2.98387961	3.21701829	3.40373842	3.23615731	3.85608132	3.53605486	3.20873286	3.4473645	3.24500849
SETD3	3.24077775	3.73032175	2.97965273	3.5028019	3.67815333	3.66315771	3.43288586	4.00190886	3.28968124
SETD4	0.83531502	0.90054304	1.19246691	1.32111702	1.42297411	1.12467647	1.00068633	1.32538342	1.49921228
SETD7	1.37609542	3.71734295	2.8052009	3.54741204	2.47606996	3.77082825	3.59461611	3.35342569	3.33234021
SETD9	1.38518465	1.88365456	1.68172747	1.81153099	2.1796278	1.46813719	2.36925058	1.86808464	1.52403205
SETDB1	2.26734612	2.32265697	2.644224	2.47669758	2.93217051	2.43595124	2.99715135	2.43536875	2.95779529
SETDB2	1.39112598	1.77686862	2.21845691	1.86102379	2.22640955	1.95807263	1.57158569	1.48423379	1.94014384
SETMAR	1.55952492	2.36794989	1.74350816	2.12040258	2.14420331	1.84988448	1.79028485	1.77332012	2.55772087
SETX	2.34912344	3.65245428	3.51800402	3.48843477	4.3178813	3.837968	3.10007098	3.60625019	3.36753881
SEZ6L	0.00508893	0.20389434	0.26811278	0.04219615	0.01872121	0.13234797	0.00304842	0.02917553	0.01067898
SF1	5.40683109	4.56390302	4.86432199	5.06256327	5.37188573	5.18698268	4.93021974	4.98221499	5.47946915
SF3A1	4.51859184	4.54962586	4.72604308	4.55637586	4.46499377	4.66360423	4.33954574	4.14384345	5.01508424
SF3A3	3.73166609	3.6796516	4.40093114	4.00740838	4.02560712	4.00648131	3.84213935	3.12522441	4.42290635
SF3B1	4.27103378	4.72641218	5.2618917	5.50054735	5.73310525	5.14350182	5.11166772	5.42437581	5.59457702
SF3B2	4.48929977	3.85231693	4.06978789	4.05780969	4.24233212	4.12007994	4.48486699	4.13383535	4.36702834
SF3B3	3.58575786	3.60542014	3.96227053	4.26345866	4.65341185	3.99127544	4.37943833	4.15044322	4.47389392
SF3B4	6.00688627	5.27446672	5.61268567	5.7424375	5.90317459	5.85161548	6.10819364	5.5717101	5.83991828
SF3B5	7.6722036	6.25861268	6.58959708	6.25523466	6.49428887	6.47087792	6.44323532	5.89963992	7.09948611
SF3B6	5.7169843	5.56851735	5.69750778	6.66706322	6.33536784	5.70127543	5.80290754	5.51194641	6.1649492
SFI1	1.53951906	0.94732086	1.29523779	1.16907583	1.45743168	1.0398087	0.73081511	0.88452292	1.45114749
SFMBT1	1.82065001	2.01755705	2.33116571	2.51016141	1.88242377	2.83402066	2.82367407	2.44070064	2.00809941
SFMBT2	0.64941745	1.32212668	1.6840352	1.44801253	2.35483112	1.6619605	1.13668052	0.74414552	0.74421695
SFN	7.95703431	4.80845823	5.50449522	6.11464746	6.19562375	6.61172119	6.54876211	5.11291807	6.23898944
SFPQ	4.85409171	3.73580302	4.903482	4.7918886	4.99947752	4.75503943	4.56898474	4.43166539	5.1703729
SFR1	2.05337019	2.36527449	2.08172921	2.92904772	3.21401429	3.22250972	2.06623815	1.77540705	1.94234518
SFRP1	0.26498896	6.04181238	1.94130226	3.22003353	0.51419868	4.26894549	1.25771604	0.280633	0.19538384
SFRP2	3.68343243	10.2262135	5.30524475	8.97769074	2.33230481	8.80807051	4.96823666	4.15644608	3.23622029
SFRP5	0.27069234	1.20188381	0.29705052	0.12539111	0.27727681	0.30164476	0	0.05258455	0.0411437
SFSWAP	2.1973727	1.47622744	2.17461255	2.09148709	2.36089129	1.94357176	1.53739333	2.45580401	2.30421018
SFT2D1	4.19331351	3.43778277	4.74828037	3.98536731	4.33858802	3.98773229	4.38717849	4.36773859	4.25191587
SFT2D2	3.37307227	3.48129832	3.74383453	3.48633393	4.64474589	3.73623266	4.30798203	4.0987784	3.24163699
SFT2D3	0	0	0	0	0	0	0	0	0
SFTA2	0.28611142	1.4309173	5.57580483	1.84323219	2.20270935	0.07550424	0.9071663	3.58719494	1.37571074
SFTPD	0	0.272765	0.07760274	0.14176963	0.01689664	0.19855967	0.01642108	0.3727495	0.01442936
SFXN1	2.78067037	2.40694792	2.82004694	3.65088956	2.93792895	3.03783656	3.14380163	3.10874331	3.5672079
SFXN2	1.12583377	1.12055443	1.24615986	0.96962217	1.56574417	1.02513824	2.24144136	0.82140813	2.11338789
SFXN3	3.1177821	3.24109111	3.00481633	3.79745824	2.86074583	3.35947786	2.95470529	3.75612468	3.25598059
SFXN4	3.15123307	3.04311535	3.7270488	3.45887589	3.59557123	3.51782602	3.26016144	3.58092287	4.3509175
SFXN5	0.95002509	0.83542158	1.22444611	0.78389688	0.47821929	0.67300673	0.91470695	0.37888577	1.04175802
SGCA	0.11426037	2.28253622	0.95997921	1.23489866	0.12281182	1.53994149	0.18355254	0.54264486	0.13419037
SGCD	0.61789992	2.52377207	1.26521483	1.68621512	0.75542565	2.61428401	1.86463019	1.36399682	0.51760457
SGCE	1.23805128	4.33928385	2.98845672	3.8793033	1.69705804	3.53071923	2.99568396	1.48370501	1.23602161
SGIP1	0.24275267	0.81638801	1.20023377	1.60872258	0.64427958	0.71648544	0.66407361	0.45501206	0.34621664
SGK1	3.67425937	3.10674346	4.65909031	3.90046783	4.08388685	3.97806929	3.25216817	3.42900986	2.39556302

SGK2	0.5983777	1.5680548	2.98133926	1.93638006	0.32083582	0.86658762	1.7942888	2.43434377	1.83522452
SGMS2	2.36894153	3.36692742	3.00370651	3.14550098	3.58209485	4.08907763	3.01658508	3.88782482	2.31339272
SGPL1	3.63687431	3.1437228	4.44759628	4.08300229	4.02903987	3.92685409	3.82625592	3.57404339	3.53053904
SGPP2	5.43527739	3.46653802	4.8552384	4.40219565	4.33335227	4.6696387	4.91479394	6.02744387	5.4505258
SGSH	2.86406171	2.34554453	3.22273297	2.49558404	2.26639055	2.4829511	2.61463985	3.05583653	2.64286999
SGSM1	0.06620678	0.29508689	0.47084432	0.29116981	0.04114427	0.34682086	0.03010021	0.3969708	0.24504617
SGSM2	2.00987634	2.03023645	2.64440053	2.23770568	1.86295462	2.02272731	1.62075588	1.94890901	2.36198799
SGSM3	4.09902466	2.77773037	3.52821638	2.68198117	2.91108125	2.88750936	3.19535925	3.8077958	3.31223844
SGTA	5.0969246	3.72921644	4.21518328	4.15111223	4.70721735	4.37488582	4.0885633	4.42711802	4.86352079
SGTB	0.71128027	1.72943869	1.83667315	2.081554	2.42268293	2.05157497	1.66466792	1.08267177	0.93893519
SH2B1	3.12753189	2.04575688	2.15326583	1.79189886	1.96471958	1.80816885	2.17213758	2.12715524	2.69569511
SH2B2	1.78565902	1.50542555	1.67677275	1.63130193	1.41391534	1.57425575	1.60222735	1.56377258	1.75154173
SH2D1A	0.86779573	1.51919987	1.82078827	1.29133507	3.29240956	1.62994832	0.92686232	0.48684499	0.75558327
SH2D2A	2.2933382	1.73179294	2.28894296	1.73242597	2.88382506	2.82864535	2.01005473	2.22597519	2.06971802
SH2D3A	2.58670853	1.17189739	1.99932782	2.52924155	2.52447479	1.97965142	1.85033586	2.45994664	2.60968873
SH2D3C	1.8425195	2.27185668	2.488167	2.0504797	2.16044908	2.53982483	1.52732979	1.42008788	1.30986819
SH2D4A	3.31769668	2.99328473	2.85403723	3.68525069	4.48880959	3.69476845	3.64784137	4.34133654	3.10588041
SH3BGR	0.63934019	2.67825253	0.43725519	1.16796253	0.84922853	1.4401816	0.97895191	0.95443274	0.81872523
SH3BGRL2	1.2957264	4.53358943	4.38312654	4.11018687	3.58459794	3.23082814	4.67321977	4.23352763	4.98527086
SH3BGRL3	7.91678204	7.32680214	6.91957574	7.71878952	7.66475297	7.63657331	7.4161888	7.05950183	7.66699211
SH3BP1	4.22871978	2.63483397	2.8871502	1.78618603	3.16150503	2.75382807	2.51425021	1.43859693	2.82105485
SH3BP2	2.26132691	1.61814035	2.53389104	2.10056484	2.041331	2.03745482	2.26561739	1.85773684	2.37108398
SH3BP4	3.41839442	4.00398897	4.43506118	3.5991667	3.51862828	3.14772757	4.3434447	4.30010392	4.6626306
SH3BP5	0.74870753	2.12231646	1.48047033	1.79220078	1.28059003	1.80347559	1.17041126	0.9376044	0.77027078
SH3BP5L	2.56974751	1.47804651	1.9480655	1.64469417	1.58042766	1.9048704	2.40418273	3.08661044	2.18895759
SH3D19	2.09396652	5.04785889	3.72612072	4.213895	3.56864621	4.2213391	3.84251679	4.71563591	4.7998817
SH3D21	0.99548868	0.4528071	1.18701418	2.385232	1.5023873	0.7470797	0.679539	0.62468733	1.81548067
SH3GL1	5.29446016	4.3254167	4.56277749	4.11675114	4.10575381	4.59275729	4.33076192	4.18590453	4.31494254
SH3GL2	0.01352513	0.03080324	0.20126618	0.84994997	0.02489765	0.00995121	0	0.68370021	0
SH3GLB1	3.33432376	4.48372747	4.42036668	4.53466684	4.21407347	4.66238965	4.27541691	4.05814289	4.13144471
SH3GLB2	3.64130127	2.420424	3.31487386	2.7201677	2.56935766	2.52454618	2.58440938	2.15820725	4.1619802
SH3KBP1	4.08899215	4.20918422	3.90907024	4.36184509	5.02470658	4.41675766	3.77491571	3.70644568	2.88130555
SH3PXD2A	2.68339633	3.88262748	2.80484072	3.61815285	1.65708394	3.60575943	2.76087823	2.38671144	2.63600254
SH3PXD2B	3.29193635	4.7648026	3.44471166	4.27537458	2.93115378	4.03334709	3.96404659	3.38368128	2.26761335
SH3RF1	4.58510447	2.95059151	3.92791362	3.09381945	4.43802423	3.81126472	3.79961218	5.14006424	3.78255858
SH3RF2	2.67181595	1.96020647	2.57808904	2.47730671	2.59447134	2.72489786	2.36062186	3.08245794	3.12201158
SH3TC1	1.90030871	1.1325806	1.56339593	0.91066364	1.02866256	1.54932102	1.21884441	1.61385922	0.67733308
SH3YL1	3.06197097	1.82328982	2.87752853	2.79892139	2.44787874	2.30002989	2.7541812	3.90090321	3.48734104
SHANK2	0.18885678	0.93885564	1.84786319	0.83289641	0.93429196	0.4014114	0.20718938	2.16290995	0.98807723
SHANK3	1.55162912	2.14783577	2.40101312	2.2388985	1.69594389	2.82161674	1.40329094	1.46561891	1.71598993
SHARPIN	5.01215742	3.64637438	4.17449436	3.61356922	3.20653496	3.96451918	4.0793149	4.44686311	3.80063759
SHB	4.77016522	2.5461234	2.67298622	3.10200734	2.60214139	2.18224643	2.29477834	4.34548864	3.08673214
SHBG	0.05440482	1.24619198	0.5023	0.23482963	0.14089816	0.05332557	0.04358676	0.02821442	0.21611889
SHC1	4.84791513	5.26407688	4.68761643	4.54145801	4.11723034	5.1470407	4.87318611	5.12488307	4.6683758
SHCBP1	1.30625451	1.31397402	2.13657892	2.14936916	2.45451196	2.42973756	2.65859314	2.11723642	1.57978048
SHD	0.81137736	0.44940737	0.24097639	0.25592272	0.25552214	0.01892999	0.22251396	0.0296748	0.86547136
SHE	0.34068065	1.44236879	1.2466878	1.06960124	0.63856939	1.3802961	0.45693878	0.37198558	0.34726371
SHF	0.47210619	1.11825137	0.59184154	1.16666916	0.19695234	0.8113297	1.06400228	0.24248596	0.60753293
SHH	1.76204717	0.47869693	0.99202058	0.12863379	0.40863861	0.17735201	1.6604344	1.42399902	0.71178723
SHISA2	0.4175369	0.69804633	0.86140898	2.21101233	0.1204341	1.76068301	2.41851384	0.76084457	0.21145619
SHISA3	0.08961139	4.27943855	1.27304809	0.69852592	0.19171626	1.2451074	2.74754954	0.31845059	3.14872917
SHISA4	1.85549928	2.94571025	2.7746566	2.67200545	1.10419779	2.70795598	1.91843493	2.10100039	0.61575224
SHISA5	5.92513758	5.85709344	5.54972512	4.77470118	4.6100041	5.7332405	5.32371447	5.01675955	5.41357463
SHKBP1	5.36130642	3.73265164	4.27929041	3.98340979	4.54541607	4.64271089	3.73021685	4.19149726	4.22818568
SHMT1	2.18785603	2.54134359	2.7793587	2.49245084	2.26183149	2.38392481	2.57622228	1.5500561	2.91244187
SHMT2	5.29299797	3.6606658	4.06247349	3.67498236	4.73361473	4.76253359	5.00815164	4.60315141	5.17144269
SHOC2	3.05125704	3.97616099	3.44598528	4.04721827	4.15970482	4.16825152	3.87049925	3.16068444	3.73802175
SHOX2	0.04270551	0.00825213	0.36265279	0.93603313	0.07373787	0.03409912	0.25318179	0.16801798	0.03377623
SHPK	1.35610685	1.68057005	1.62424049	1.64909116	1.72028536	1.80760289	1.48978512	0.77853429	1.65624969

SHPRH	0.53580077	0.85776108	0.82112354	0.79399034	0.91862192	1.04146609	1.1411009	0.85234223	1.05318708
SHQ1	1.64117582	1.99552779	2.31725552	2.31624456	2.62249252	2.33960799	2.12959193	2.24808774	2.50146265
SHROOM1	1.74618919	1.40240169	2.92017973	1.87622977	1.25746321	1.72863139	1.92285235	4.1835512	1.92824109
SHROOM2	0.25616192	0.4118022	1.3113337	1.50830802	0.25325447	0.52913764	0.38566397	0.51631513	0.12750542
SHROOM3	4.43312897	3.06535645	3.54349089	2.54389244	3.97531436	4.43289977	3.61311913	3.90393954	3.81420093
SIAE	2.23447733	3.93505045	2.75811763	3.25985315	3.42184613	2.66329898	5.05947749	2.08225642	4.43015804
SIAH1	1.31253394	1.79497785	1.85661874	1.6940503	1.48561692	1.58357911	1.50098456	1.2107572	1.60671696
SIAH2	5.16184011	3.65094089	3.83433866	4.11629299	3.82917967	4.2300071	4.30171182	4.08316999	3.62826313
SIDT1	1.34953464	0.90773098	1.01360965	1.04827767	1.41692037	0.8999395	0.22509476	1.335295	0.89016061
SIGLEC1	1.7828123	3.97312943	3.20628092	2.37083185	3.88983763	3.97986675	2.49090392	1.12943041	1.17060984
SIGLEC10	3.07489438	2.71366903	3.23589163	2.78787879	4.15461695	3.88997441	2.97790743	2.1517635	2.13875381
SIGLEC15	0.24189776	0.59141864	1.68556176	0.285746	0.57410666	0.5063884	0.34367127	0.31759419	0.06760676
SIGLEC16	0.20284581	0.09650307	1.27374163	0.14097443	0.21562053	0.53350949	1.05965499	0.34656802	0.08160227
SIGLEC5	0.30558684	0.39730855	0.89435328	0.9694784	0.5771627	1.5625221	0.60543925	0.53371202	0.21088256
SIGLEC8	1.83398627	1.32267285	0.89787743	0.5064997	2.87012827	0.90026742	0.43604459	0.61175398	0.72530217
SIGLEC9	1.54609145	1.54343695	2.35174619	1.49601225	2.11581367	2.22802104	2.0324776	1.07603627	0.86064587
SIGMAR1	5.58437215	5.34227602	5.24666214	5.88873123	5.28325514	5.73973629	5.59416988	5.83136522	6.27560872
SIK1	0.11466501	0.17292845	0.01607675	0.12426895	0.1483391	1.51685484	1.1360187	0.79353504	0.57188218
SIK2	2.65343963	3.1628191	3.06431685	2.8044535	2.49050967	2.89496839	2.85958163	3.58061005	2.67294055
SIK3	2.87595253	2.67321842	1.87212578	2.41006814	2.65959341	2.77259945	2.30159065	2.01957817	2.07638206
SIKE1	2.43913342	2.99809482	3.28044925	3.56823315	3.31902299	3.08382804	2.56142791	3.09746745	3.00197791
SIL1	3.85453043	3.23185819	3.87287212	3.14475572	3.19892285	3.301671	3.4592045	3.88987649	3.11611132
SIM2	1.53518736	0.56036239	1.53887563	1.68542194	1.85416031	1.91659145	3.24786655	5.17536198	1.59117457
SIMC1	2.40434329	1.83671593	2.58602491	1.96014563	2.95514572	2.14030569	1.844881	2.45358214	3.01017134
SIN3A	2.78640142	2.75624412	3.20573614	3.08860971	2.88988794	3.02469729	2.72520578	2.70224273	3.04508735
SIN3B	2.49191047	2.37595076	2.41055116	2.66469186	2.67905105	2.63565275	2.10469206	2.41222911	2.43402167
SIPA1	3.56862922	3.40141338	3.82957774	2.94292036	3.05832966	3.93269988	2.96412648	3.33232663	2.72815973
SIPA1L1	3.63244978	3.00765977	3.04449132	3.04280925	3.24309729	2.85174491	3.39856307	2.78308776	2.81889192
SIPA1L2	0.97237502	2.81382993	2.25659701	2.22142001	1.56127475	2.35487403	4.50148092	3.90529011	2.04666858
SIPA1L3	3.89137662	2.14965321	3.07478539	2.25717552	3.67613066	2.53097454	2.16613264	3.22389161	3.4178469
SIRPA	3.52136719	4.74595673	4.43430775	4.35383837	4.29723934	4.77418387	4.32520585	3.04009134	2.96620784
SIRPB1	1.92422821	0.72119213	0.82135117	2.32009093	1.48211933	2.63813388	0.96042963	0.69047796	0.43400968
SIRT1	2.26205786	2.63708467	3.41910463	3.14828706	3.14141674	2.65876386	2.61283095	3.73072983	2.56541667
SIRT2	4.18787682	3.02702126	3.31460682	2.59991842	2.97287996	2.97259187	2.95860479	3.11861509	2.97786498
SIRT5	1.27610175	1.71048449	1.73166145	1.66062557	1.68103435	1.80654609	1.14086082	2.03864344	1.83883679
SIRT6	3.76351873	2.0768984	2.58411998	2.39153028	2.85320582	2.48093298	2.53246426	2.9728292	3.23480847
SIT1	2.70079726	2.08727785	2.47309713	1.18126268	3.06231275	2.19792501	1.21529146	0.85950946	1.79142883
SIVA1	3.93667191	2.50842589	2.58730605	2.51009005	2.41514393	2.8334112	2.78366262	3.26805783	3.00412826
SIX2	0.4981858	3.92358516	0.43042575	0.93671614	0.26016625	3.26173967	0.64372067	0.16817532	0.31834784
SIX3	0	0.06471945	0.33389699	1.00661606	0.03516318	0.55307885	0.02571029	0.04925632	0.03005623
SIX4	0.8052558	0.78551856	1.51249173	1.15063874	1.0980451	1.43857292	1.28953884	2.35186425	0.12663051
SIX5	2.42081506	1.70775236	1.59557342	1.45136239	1.1282881	1.81722419	1.51731894	2.20435968	1.75484247
SKA2	4.27922334	3.22313098	4.0164734	3.75058697	4.69298998	3.66535501	3.08720153	3.33174944	3.84002807
SKA3	2.87420809	1.81980371	2.59780635	2.99121491	3.37207835	2.64874587	3.90503637	2.49195938	3.60111638
SKAP1	1.0618118	1.3851635	2.40807242	1.47806174	2.41609155	2.46785446	0.98296407	1.19107618	1.29231161
SKAP2	3.89399208	4.04118892	3.5988055	3.61522378	3.6842564	4.46591712	3.6038124	3.05962418	3.58212378
SKI	4.17073541	4.32339084	3.56916732	4.36931223	3.83039816	4.64163385	4.007823	3.30889369	3.85788939
SKIL	3.5940641	3.05279221	4.86251366	3.83616261	3.78895834	3.76615358	3.07032473	3.2630847	2.10833923
SKIV2L	2.85269963	2.4651067	3.58214885	2.75994018	2.89968328	2.88061011	2.83834348	3.62678655	3.00700112
SKP1	4.05542332	4.55741658	4.2208053	4.66118832	4.36581186	4.17034495	3.97793201	4.01332945	4.25564011
SKP2	3.96067537	2.93019481	2.84371778	3.00271888	3.16637266	2.6941657	3.55570221	1.56508356	3.95323964
SLA2	1.78894418	1.41629767	1.80282367	0.95607859	2.93001456	1.81069843	1.27208243	0.77980288	1.11151533
SLAIN1	0.79365788	0.83530796	0.89956492	1.01395505	1.18440625	2.12866724	2.79228359	0.9699669	0.2149205
SLAMF1	0.80860745	0.96529075	1.49412939	0.73148045	1.67889947	0.80744123	0.55691268	0.53308488	0.84000277
SLAMF6	1.31098767	2.39732336	2.38598961	1.84782697	3.59313532	2.07881712	1.75186915	1.28660682	1.45714452
SLBP	5.41474956	4.26396193	4.52230932	5.29320331	5.82226851	5.04621466	5.09526614	4.76837474	5.17720518
SLC10A3	5.06616855	3.98815268	3.70365607	3.71747455	4.06662639	3.74909041	3.78660069	3.78022349	3.6438284
SLC10A7	1.32179035	1.61251915	2.32696517	1.76945959	2.08627853	1.91793892	2.16430928	2.47439079	2.00586928
SLC11A1	1.50153891	1.83393702	1.65913476	2.45345135	1.34528596	1.13631923	2.75823312	1.36301876	0.90840455

SLC11A2	2.45955218	2.73158032	4.40102747	3.25001054	3.21747756	2.67851822	3.13350733	4.56361525	3.96489815
SLC12A2	3.36360722	5.1989803	4.67256537	5.9077596	3.73263952	3.88929342	4.4051074	5.12908195	7.14832324
SLC12A4	1.74817947	2.69692615	2.43909054	2.56704551	1.972939	2.7115492	2.19964519	2.43171095	1.4789349
SLC12A6	1.45728572	2.16698735	2.49308649	2.45754656	2.58239946	2.43048092	2.25760135	2.04250027	2.32182977
SLC12A8	1.96303462	1.97727855	2.66119169	2.05594542	1.17584346	1.94783895	3.05328982	2.37238349	1.85865707
SLC12A9	3.58536681	2.07546684	2.72648737	1.82683135	1.99334156	1.91245138	2.6173889	2.34451532	2.76145341
SLC13A2	0.95581845	2.41734318	0.95118802	0.00676198	0.06131481	0.02469269	0.00674681	0.13697499	2.4837652
SLC13A3	0.0818967	0.42409775	0.60544391	0.48334738	0.07231602	0.31111459	0.12750671	1.15607498	0.0343887
SLC13A4	0.04917051	0.20596152	0.27439831	0.17209476	0.26768336	0.05732074	0.04818095	0.12579343	0.13671949
SLC13A5	0.00809237	0.05919698	0.24970973	0.0431661	0.01492036	0.03534362	0.00242683	0.03552777	0.04203759
SLC15A2	0.13972849	0.26350099	1.03918692	0.51308545	0.48641723	0.58564065	0.24496704	0.17448821	0.44957704
SLC15A3	3.88563515	2.87156865	2.6512736	2.43795225	3.06967658	4.09362433	2.95264584	1.75038299	1.65955641
SLC16A1	4.42603768	4.32349208	4.24608726	4.96270804	4.71717797	4.45076041	5.09309781	5.5214193	4.92334807
SLC16A10	0.08806879	1.03616112	0.83023149	1.10827658	0.5264732	0.67105779	1.5448048	0.31939424	1.20745409
SLC16A2	1.00996963	4.38266851	2.95178879	2.19434251	0.99898344	3.10055965	3.58635162	3.42443434	0.76998388
SLC16A3	3.12948656	2.30157426	3.2598013	3.05646577	1.73271816	2.91713544	4.23960013	4.75365813	3.31044633
SLC16A4	0.59502521	3.47096089	2.1242239	3.0881044	0.58140303	2.10827807	0.70088337	0.4367447	0.47500964
SLC16A5	4.59063573	2.05887257	3.26694702	2.79017479	2.2881463	4.21434807	0.59837897	5.05490418	2.53109138
SLC16A7	0.15654669	1.53113896	1.84224437	1.01699796	0.78124313	1.3127383	0.39062059	1.74715699	0.17037552
SLC16A8	0.69529553	0.06485231	0.17957623	0.2951695	0.71618965	0.09411655	0.20290316	0.48767488	0.14073528
SLC16A9	0.45495602	1.49390113	0.77989348	1.23369113	0.06443936	0.46833092	1.49908616	0.06813847	0.03419408
SLC17A4	0	1.1101731	0.24026111	0.10904103	0.11185442	0	0.02335427	0.05648502	0.9709517
SLC17A5	2.16015597	3.04597308	3.94552028	3.45945943	3.48143444	3.67492825	4.57112073	3.63138568	3.68109546
SLC17A7	0.08816571	0.34701595	0.13372962	0.19048765	0.07635433	0.14400135	0.05342113	0.03462184	0.0221307
SLC17A9	0.84122734	0.51848026	4.68814395	2.45708151	0.95504756	1.79268578	3.37014534	2.63691601	1.20386423
SLC18A2	0.24332984	1.33480148	0.72280384	0.33441431	0.28993863	0.58666961	0.25615612	0.33030161	0.36704973
SLC18B1	4.35422412	3.09571095	4.54490844	3.97810708	3.94934745	3.44585482	4.31351487	2.94499441	4.43818522
SLC19A1	1.66006579	1.62889698	1.40510052	1.53576231	1.38547432	1.66217472	1.97829412	1.95808667	2.29298056
SLC19A2	3.07704194	2.36363146	3.53164212	2.93644493	2.8364664	2.58888405	2.79150166	3.01887421	2.17499893
SLC19A3	4.66049147	1.27668879	1.53301601	0.48646757	0.83401719	0.71454022	0.6896852	2.82346567	1.26474741
SLC1A4	2.50077506	2.44791866	3.40205776	2.42799041	3.16088078	2.88422912	3.29787801	1.31133627	4.11939363
SLC1A5	6.06913152	3.83723849	4.93519089	4.51363899	5.28970402	4.86219126	5.61664501	4.98970314	4.93872781
SLC1A7	0.30833066	0.69437953	0.38643452	0.50919478	0.03284201	0.31271458	0.07851996	0.10518093	0.01760696
SLC20A1	3.03519725	2.62185276	3.38329911	3.80239602	4.08275421	3.66049721	3.42012088	3.63917216	3.00428047
SLC20A2	2.14890965	2.81241219	2.8268952	3.18866854	1.97510002	3.17776018	3.59019944	2.58587569	3.05381934
SLC22A11	0	0.08537142	0.27464587	0	0.01709606	0.01022086	0	0.02665122	0.07505171
SLC22A13	0.06961399	0.06392657	0.22827638	0.03383169	0.10993824	0.04641414	0.05034304	0.07498484	0.08727921
SLC22A14	0	0.03939422	0.24434082	0.08696223	0.03635417	0.03256661	0.01777702	0.01527454	0.03619099
SLC22A15	0.37932684	0.56146528	0.94430194	1.14124591	0.77042646	1.27436251	0.72128454	1.24081042	1.10884215
SLC22A17	0.6314935	2.19169766	1.35694107	1.37728114	0.24994679	1.81781305	1.07960521	0.74105297	0.39681508
SLC22A18	4.45250445	0.90172784	1.90555192	2.28525678	1.49763806	2.04023178	3.87462312	2.81113816	3.67861005
SLC22A18A	2.63945511	1.37380152	0.77118798	1.10311174	0.43223516	0.68185216	1.99223889	1.82400819	1.77786172
SLC22A23	2.2157778	1.69059054	2.81230609	1.56998118	2.74375989	1.62194912	2.2210546	2.62838171	2.38823135
SLC22A4	0.61002522	0.68553222	1.01324844	0.86647338	0.71092274	1.599498	0.58562129	0.48007439	0.86003825
SLC22A5	2.04443639	1.30706224	1.66999803	1.02897682	1.58820262	1.08253642	0.89045861	1.23680311	1.4008673
SLC23A3	0.25024648	0.17964329	0.30233408	0.60187074	0.43762887	0.1280182	0.26995181	0.26313167	0.4014207
SLC24A1	1.88001563	1.11291081	1.13317138	1.23864083	1.20288725	1.24996923	1.37744488	0.8774205	0.89378528
SLC24A3	1.19194364	3.41729673	2.16806778	1.98251002	0.72152526	2.43457456	2.14717995	1.40529623	0.7816748
SLC25A1	6.47387986	4.99300239	5.03406031	5.13313507	3.87177468	5.11831147	5.04645974	4.35530486	5.6431963
SLC25A10	3.58578276	2.15931651	2.65033538	1.98362442	1.69794878	2.30493726	2.6571654	3.45700892	3.37609509
SLC25A11	3.89853251	3.73877824	4.45040344	3.83727385	3.72733337	4.19987622	3.85586572	3.61860269	4.51447223
SLC25A12	1.87129732	2.66092443	2.45153464	2.73528641	2.59811923	2.66127229	2.46539854	1.93134657	0.90567164
SLC25A13	3.66885021	3.32442957	3.85097093	3.15126518	3.01297163	3.21855251	3.54836236	3.38474968	3.58393392
SLC25A14	1.71156106	1.29129104	2.79577592	1.49740473	2.37408949	1.50667287	1.05692241	1.51955566	1.56527538
SLC25A15	2.81776896	1.85404955	2.29409558	2.3576029	2.30430381	2.26443148	2.99052628	2.64074701	3.23477906
SLC25A16	1.36774899	1.34089613	2.73063938	1.90613625	1.55168775	1.26747736	1.12326801	2.28749868	1.50076901
SLC25A19	2.12671321	1.03677866	2.18235883	1.76533703	2.05604776	2.14875977	1.80883051	2.14425804	2.21660488
SLC25A2	0	0.0857915	0	0.07516139	0.10685489	0.15231916	0.01531445	0.01969086	0.03999822
SLC25A20	3.85392816	3.58345635	3.63116656	2.61071135	3.46806693	2.95821827	3.01983762	2.31301833	3.5367219

SLC25A22	3.34712662	1.91148148	2.1996168	2.8771088	3.20028419	3.4058985	2.63983348	3.20664632	3.50566241
SLC25A23	4.57619599	4.16643284	3.48754441	4.87098368	3.25716679	4.06951569	4.62509787	4.79204234	4.14500248
SLC25A24	3.07229152	3.67437945	3.8015513	4.06874412	4.48124741	4.14447757	4.14786299	4.41757174	4.3524057
SLC25A25	2.77496375	1.77829872	3.33408873	2.15121386	1.73958537	2.70922526	2.27734582	2.43682937	2.73727818
SLC25A26	1.55315058	1.6986295	1.98004736	1.92229022	1.50498406	1.75631633	1.87992841	1.69014104	1.95452137
SLC25A27	0.03287527	0.8310217	0.75080692	1.39095981	0.26157739	0.70443739	0.11474502	0.61078563	1.36457492
SLC25A28	3.94025713	3.69453857	3.53176969	3.80970868	4.14111721	4.20494267	2.9535737	3.24105157	3.78599708
SLC25A29	2.12428405	1.51819572	1.99931159	2.18440654	1.56415273	1.76520512	1.54492	1.76294863	2.95828899
SLC25A3	5.42803194	5.07378777	5.41678514	5.75419659	6.00385348	5.56704669	5.62657838	5.84451904	6.0417459
SLC25A30	2.44841728	1.33259355	1.39979207	1.46587817	2.27608615	1.47301562	0.98825644	1.72438761	1.61536632
SLC25A32	2.41541502	2.98516219	3.99042844	4.07064391	3.63390406	3.79900789	3.7377724	3.02048876	3.36447579
SLC25A34	0.0991737	0.68425756	0.49700629	0.31257851	0.39682173	0.16608985	0.12423057	0.15017695	0.81942993
SLC25A35	1.03115418	1.49158893	2.81298018	1.64314572	1.40559922	1.11732642	1.2088707	0.84558895	1.24474125
SLC25A36	2.41864644	2.8801808	3.0353098	3.61170053	2.7516575	3.01977213	2.88976409	2.88309825	3.68027667
SLC25A37	3.08915752	1.87277925	1.78315563	3.47412765	2.90291547	2.8321532	3.12303402	2.29897461	2.80524062
SLC25A38	4.13801698	3.28169624	4.28390149	3.74502	4.35129569	3.6126055	3.74049624	4.11945425	4.09328753
SLC25A39	6.32196399	4.44910188	5.3760201	5.22986532	5.20693396	5.2298471	5.65046384	5.24399607	5.75752822
SLC25A4	3.26845383	3.11123178	2.0104985	2.99532142	0.66129225	3.1763353	2.46963649	3.24857256	3.2538258
SLC25A40	3.10047738	2.32145804	3.67477496	2.74485629	3.14218986	2.70132853	2.90515485	2.59950014	2.87732404
SLC25A42	1.24122252	1.18141839	1.32329864	1.11801633	1.32277953	0.97025543	0.51093852	1.08431004	1.03698633
SLC25A43	2.92932084	2.97386072	3.83555907	3.6288418	4.42447284	3.31527124	2.94653255	3.27778499	3.51218404
SLC25A45	0.84087597	0.71624598	1.04228688	0.64646868	0.69962054	0.35619691	0.62562938	0.46133132	1.05554848
SLC25A46	2.16737486	2.87982374	3.16292276	3.10937817	3.40814542	3.17938078	2.6773066	2.29698465	3.25225002
SLC25A48	0.00732029	0.05363866	0.31757376	0.01314894	0	0.03726523	0	0.03631044	0.3985605
SLC25A5	7.64187511	7.31552038	8.39261606	8.0264236	9.11954367	7.57946139	7.81710284	7.98071524	8.6080761
SLC25A51	2.03284011	1.69948791	1.67668345	2.42308938	2.04387142	1.89678824	1.55536823	2.04826003	1.82125504
SLC26A11	1.30056479	1.10499435	2.32021664	1.83416732	1.7747521	1.61158591	1.37031887	1.91615573	1.33148407
SLC26A2	1.00069113	2.30925441	2.54267539	2.41379762	1.7530458	1.9201922	1.63297598	2.04984625	1.33506981
SLC26A3	0.03197158	1.84165691	0.90628836	0.05081618	0.23248575	0.061999	0.00643629	0.08859665	1.46206456
SLC26A6	1.87174691	1.2567361	3.54901285	1.79332816	1.51884831	1.42886799	2.54843433	1.98355885	2.42359127
SLC26A9	0.92767227	0.28819387	1.93893767	0.62160856	2.34913894	0.04911956	0.3645693	1.94199624	0.03031386
SLC27A1	2.29322195	2.01870576	2.04481921	1.9604803	1.09599303	1.69611067	1.7567902	3.42886841	1.21809989
SLC27A3	1.96185694	2.25705869	2.08850955	2.07615613	1.55317397	2.19759045	2.21867861	1.91849852	1.76278606
SLC27A5	0.94285476	0.65717576	0.27408689	0.62305776	0.82364669	0.68144584	0.60414478	0.62850733	0.44084867
SLC28A3	0.07024474	1.45293494	4.62185579	2.13080643	2.4300958	0.293358	1.04493681	4.40190588	3.14902937
SLC29A1	4.11231988	4.85137186	5.11152202	4.20679219	3.97812418	4.44198819	4.48841161	5.63116479	5.88143236
SLC29A2	4.05174221	1.7584208	3.70235173	3.22620654	3.08759982	2.70439662	3.65501953	3.63361696	3.84266152
SLC2A1	4.81024995	3.41959754	4.50150938	6.51829576	3.39071847	4.31624656	5.51787827	3.67399439	4.7222164
SLC2A10	0.88263643	3.32321914	3.32630954	3.25564678	1.23124592	2.35226063	2.44113602	3.76706578	0.73228592
SLC2A11	0.68024944	0.69611679	0.53769053	0.92869887	0.50957533	0.45704884	0.56362542	0.45321094	0.55630482
SLC2A12	0.10990197	0.9864356	1.08631145	0.71466964	1.12943054	0.90656481	0.96173579	1.40340188	0.45574294
SLC2A4RG	6.3315243	3.66643148	5.09383256	3.61336784	2.64702071	3.8285326	4.5135145	3.92609829	2.9968325
SLC2A5	0.84206442	1.24134382	0.92173988	1.17135849	1.03794358	2.17088991	2.31052535	0.61444498	1.5084604
SLC2A6	1.90038134	0.82249145	1.61700437	2.29790115	1.6143739	2.13514517	1.22731817	1.03414344	0.97379788
SLC2A8	3.09378955	1.63728847	2.93261046	1.43996512	2.23156944	2.23857545	1.7351315	1.56712565	2.04388945
SLC2A9	0.42215703	1.70194807	1.06917917	0.7371014	0.96564679	1.0462393	0.83713512	0.49642317	0.70166631
SLC30A1	2.62062852	4.13298361	3.71983872	4.62822573	3.74579324	4.15284573	3.79364953	3.96800048	3.11808019
SLC30A3	0.02418225	0.10762996	0.26116115	0.35773922	0	0.00670439	0.03247639	0.02561809	0.05038278
SLC30A4	0.72322271	1.30959184	1.29092484	1.34876685	0.74057299	1.22544766	0.68595695	0.65118484	1.01419687
SLC30A5	3.23179629	3.15589618	3.27506855	3.58308209	3.59965796	3.41310955	3.51890217	3.76013692	3.52910695
SLC30A6	2.78997808	3.45317447	3.84930263	3.50373048	3.79746464	3.44298982	3.45222367	3.96804898	3.6911195
SLC30A7	2.31383381	3.09960073	3.5934847	3.43247003	3.67911138	3.50391231	3.44586733	3.16259528	3.2153625
SLC30A9	2.76045645	3.25243445	3.40068291	3.94394497	3.60887535	3.13315732	3.75615474	3.8664233	3.76244715
SLC31A2	0.68285448	0.96605172	1.38044721	0.77754925	1.84464578	1.25703204	1.34137222	0.53709313	0.74284286
SLC33A1	3.01429906	2.0709053	2.57790625	2.18589081	2.53993721	2.35090212	2.23577663	3.69423056	2.60667622
SLC35A1	2.2402066	2.75896149	2.68633242	4.22747707	3.32623374	3.12779531	2.50566674	2.63231882	2.42092283
SLC35A3	2.98283547	3.05530238	3.48994057	3.56950815	3.69256073	3.23422849	4.37838441	3.67546994	4.03541383
SLC35A4	4.97087824	4.36902353	5.04145971	4.58606237	4.74029628	4.97942554	4.5437281	4.57658176	5.12492089
SLC35A5	3.33018063	3.31482369	3.44899418	3.41659306	3.55918312	3.43933759	3.48444065	3.13657071	3.43927425

SLC35B1	4.38168157	2.57831563	3.55367532	3.12264384	3.35786199	3.06769022	3.58261588	3.17402881	3.34891761
SLC35B2	4.91269796	4.1737585	5.6759722	4.40928955	3.48730904	4.66342804	4.77023668	5.79044153	4.66702869
SLC35B3	3.13782087	3.73001754	4.01198094	3.91028706	4.26237545	3.84312414	4.20775125	4.60152279	3.71945456
SLC35B4	2.46281813	2.70624297	2.44532711	2.58942102	2.67126245	2.4516675	2.26019364	1.82454267	1.58608168
SLC35C1	4.30756691	3.36483041	3.66995796	3.31355898	2.80387059	3.66142533	4.62495598	3.92059157	3.8791891
SLC35C2	3.21237078	2.17387766	3.35265881	2.66545836	2.32414231	2.62120481	2.67429615	2.71655707	2.93180332
SLC35D1	2.34107523	2.71021853	4.45724056	2.92394917	3.85611708	2.92695986	2.7251734	3.67592606	2.96801165
SLC35D2	5.26064433	3.89071586	5.54781561	3.95120984	4.7760364	4.1152374	4.04972867	3.6885309	4.66122098
SLC35D3	0	0.37231426	0	0.02754215	0.0094862	0.01692958	2.12611028	0.01186007	0.59667468
SLC35E1	3.74184316	3.25609334	4.02619053	3.50201177	3.64443373	3.48839637	3.64772758	4.08671175	3.31300358
SLC35E3	0.83174329	0.72570507	1.31610197	0.64903904	0.52177691	0.6096961	0.58734943	1.17100401	0.60104374
SLC35F1	0.02971854	0.84938299	0.5031094	0.15769486	0.2224112	0.66796538	0.0871828	0.0897338	0.08429608
SLC35F2	2.38793434	1.96929897	2.27636729	3.72020943	3.33059043	3.55213723	3.5732538	2.30821491	3.37835975
SLC35F5	2.40481608	2.75469174	3.83708199	2.40860678	3.61076769	3.0360151	2.65238319	4.15790506	2.70172782
SLC35F6	4.42822254	4.02979995	3.61676948	3.70539387	4.38829011	4.11597922	4.47163089	4.10069048	3.57286581
SLC35G2	0.34603347	1.54790288	1.09536666	1.41229384	0.8174798	1.13856783	0.55497149	0.63613293	0.35475099
SLC36A1	1.86041773	2.35648849	2.899862	2.40401156	3.01494545	3.05756328	2.23474489	2.39774128	2.06638602
SLC36A4	0.34033456	1.28792123	1.34886209	1.98463792	0.92709339	1.62679866	1.11629865	1.41347395	0.52754492
SLC37A1	4.13305368	2.22064108	3.28822444	2.74909398	4.28636961	3.40029854	4.4160355	3.52151217	4.99074791
SLC37A3	2.32568983	2.15360322	2.50760864	2.26230299	2.43508204	2.31255346	2.66871333	2.45904311	2.47994177
SLC37A4	3.71687961	1.86336135	2.20698205	1.81602032	1.76447022	1.81526433	2.16303344	1.68460705	3.28180013
SLC38A1	3.27756768	4.14773452	3.40847543	4.36238289	2.74208908	4.41166822	4.98879705	2.3025925	4.77116776
SLC38A10	5.08710194	3.24233913	3.63592672	3.11280574	3.16717268	3.61679889	3.59977289	4.76209697	3.69985189
SLC38A11	0.10138162	0.85434431	1.34018849	0.26544417	0.20685022	0.36087309	0.18419585	0.48163908	0.57327634
SLC38A2	3.59560303	5.216165	5.41918811	5.07974861	4.86132646	5.11074456	4.67496291	4.02763976	4.38998107
SLC38A5	1.03632639	1.43760135	1.62618513	1.91510413	4.70981689	3.99938749	1.38744976	0.6838318	1.01827413
SLC38A6	1.37852474	1.56808857	2.15039802	1.72492553	2.67468172	1.69667802	1.50845674	1.36287311	1.03562378
SLC38A7	2.72738663	2.45009821	2.74561404	2.4487443	2.60667348	2.91025766	2.37206923	2.5257827	2.1578691
SLC38A9	1.16149313	1.16446095	1.57511046	1.32764298	1.964032	1.43465541	0.90090563	1.29928122	1.15189084
SLC39A1	5.6522905	4.59818719	5.63133177	4.52609967	4.13829041	5.33914553	5.42940888	5.80731417	4.50418685
SLC39A11	3.97568625	2.35348074	3.9652133	2.3768851	2.80706335	3.18924043	3.25931142	3.51282849	2.67460837
SLC39A14	5.48092266	4.84085964	4.89728329	4.86738938	5.05450387	5.08516958	5.20105537	6.09103562	4.76275614
SLC39A3	3.20720382	1.64019496	2.51070326	1.81438924	2.15919299	2.41659294	2.24304314	2.39232747	2.07938785
SLC39A4	3.04048455	3.82392894	3.64202674	3.55241427	1.66617258	3.02677608	3.3786759	3.67003709	4.2765671
SLC39A5	3.50971011	3.72104514	2.28471583	0.6594736	0.34259855	0.18989503	0.92292338	1.63673024	3.95630237
SLC39A6	3.1488394	4.02811092	4.63814905	4.86444534	4.69810907	4.47201302	4.63583679	4.24691225	3.79137022
SLC39A7	5.99255054	4.54272443	6.6939154	4.85585028	4.48217947	5.11732575	6.13173737	6.23197186	4.98648238
SLC39A8	1.23195535	3.20157172	2.43352613	3.36513923	1.95498057	2.94389839	3.98183227	2.77438052	3.19498065
SLC39A9	3.89294615	3.78331233	4.41532568	3.98912189	3.76492172	3.87226216	4.3523125	4.42581588	4.11474927
SLC3A1	4.00800827	1.88708196	1.96000667	0.0557372	0.19850685	0.06210685	0.14881307	0.21321785	1.55815956
SLC3A2	5.47187292	3.40928728	4.82928271	4.20032761	4.12456946	4.49263357	4.44309875	4.39703659	4.01006815
SLC40A1	5.21838833	7.16113916	7.08790918	5.67293493	7.48991353	6.45365025	5.1389525	6.71534727	5.57661716
SLC41A1	2.99100063	2.23370381	2.98007716	2.78824179	2.32830857	2.75171978	2.93565393	3.27402909	2.27601755
SLC41A2	3.53275758	2.51514445	3.33001742	2.86138998	2.95014395	3.11496648	2.51838921	3.23237141	2.78889599
SLC41A3	3.18119299	2.30583554	2.13836053	2.65629275	1.53038567	2.45944793	3.01625471	2.07112123	2.69397418
SLC43A1	3.62148886	2.77959888	1.57984708	3.05219837	1.85839108	3.11879112	4.76245906	4.44963053	3.39801804
SLC43A2	2.69983388	2.32786735	3.08790741	2.08924361	2.20784903	2.16617114	2.10169191	1.54165761	1.83351955
SLC43A3	3.4912916	3.18406733	3.61786857	3.701398	2.93923708	4.22895696	4.69323503	4.34984862	1.64674436
SLC44A1	3.95600757	3.70631939	4.3786859	3.91659853	4.17223096	3.50772628	3.82186514	3.18557744	3.9286154
SLC44A2	6.08107268	4.57057094	5.51078473	5.0422337	5.25501913	4.48060134	4.83850956	5.0796902	4.21455219
SLC44A3	2.58949633	3.41118036	4.21044537	3.72969484	2.93651837	2.26246348	3.20431565	4.45741942	4.88472122
SLC44A4	7.05161561	4.55547524	7.46668042	5.25632147	3.4522376	4.62990831	7.25856448	8.08546851	5.75140027
SLC45A3	4.22345935	1.92100981	2.476414	2.704581	1.76211013	2.96525066	4.26439159	4.53981458	1.81032152
SLC45A4	2.4121808	1.37025668	2.14400543	1.90424746	2.21502556	2.37037085	3.37145363	2.66135598	2.05683992
SLC46A1	0.46162153	1.08572444	1.21572676	0.72895622	0.75444416	1.07372092	0.86083717	1.07249981	0.33831881
SLC46A2	0.09852235	0.05725381	0.13070352	0.10935756	0.0089457	0.1949434	0.31938618	0.08714094	0.01523251
SLC46A3	1.46789492	3.86448539	3.21254143	3.13663429	2.77920042	3.17481622	2.4289909	1.94746582	3.78478299
SLC47A1	0.50999687	0.36278539	0.87664621	0.42708352	1.45355518	0.38058681	0.46545801	0.2455786	0.59309459
SLC48A1	3.08832964	2.48966767	2.59900868	2.32035706	2.41139333	2.47868891	2.07317702	2.55461578	2.05180948

SLC4A11	0.79628186	0.54689437	2.45327307	2.90671854	2.05966703	1.52880037	1.56206209	2.37424903	1.42340703
SLC4A2	4.91117656	3.30898787	4.31172059	3.35182116	3.11610481	3.72067758	3.95643914	4.23904385	3.88187105
SLC4A3	0.11021966	0.81232422	0.28531682	1.92651503	0.0730882	0.62260869	0.12707777	0.73598872	0.08404969
SLC4A4	3.24733644	1.14612033	4.09560259	3.29153787	3.25871169	2.41459456	3.90676235	5.17384605	1.58318259
SLC4A7	2.12345803	2.46081798	3.58159466	2.54952915	2.89332583	2.37307645	2.31938035	1.06173745	3.16473025
SLC4A8	0.04980465	0.14774967	0.72922979	0.15111075	0.49596473	0.10621609	0.13650926	0.73737671	0.11366738
SLC51B	0	3.14829604	1.0345263	0.40323092	0.51646606	0.66227541	0.36688637	0.20835194	0.74678657
SLC52A1	0.04559243	1.49604819	0.7015871	0.01107349	0.23635579	0.11447054	0.12730716	0.10332691	0.31776503
SLC52A2	4.76888368	3.77500714	4.65720023	4.09711907	3.22582552	4.48415976	5.34039045	4.60994383	4.82533619
SLC52A3	2.77335524	2.98510418	3.31898663	2.31529166	1.93313238	2.62911249	2.42779857	2.26850613	3.22119052
SLC5A1	2.55491327	5.29496502	3.68031898	2.54262653	1.2932082	1.06038673	3.09488141	3.38294296	4.90797931
SLC5A3	0.68416366	2.22167758	1.31256657	2.43046843	2.34583004	2.41665958	2.30236731	2.42745371	2.52031573
SLC5A4	0.12049712	0.36190416	0.74354485	0.25594197	0.15703398	0.34974669	0.62647598	0.20599264	0.21037288
SLC5A5	0.0099451	0.01704754	2.31265493	1.0558954	0.12928155	0.03620857	0.25153361	0.40045543	0.02083117
SLC5A6	3.32176494	3.01344682	3.04498428	2.92380905	3.20993357	3.00391339	3.27022597	3.17125117	3.89094521
SLC5A8	0	0.03942876	0.25363106	0.00522398	0.0053638	0.01276358	0	0.0365164	0
SLC5A9	0.10086341	0.37133103	0.38499377	0.11130874	0.03907214	0.16447041	0.03798061	0.19929707	0.78019336
SLC6A1	0.1615378	0.35649299	0.80880479	0.79024066	0.25529155	0.19150128	0.21455446	0.2468665	0.30833349
SLC6A11	0.02694454	0.00621811	0.07279942	0.01622585	0.02326832	0.01787631	0.01296614	0.07356638	0.00570715
SLC6A14	1.30116953	0.45559802	3.84285897	4.92360061	6.79611652	0.19893484	2.91786085	4.80152227	1.40333523
SLC6A16	0.18060976	0.26426801	0.4964659	0.39135509	0.32782522	0.36318925	0.07096637	0.22704335	0.14183286
SLC6A19	0.18150365	0.88768276	0.99227954	0.01237965	0.29527927	0.18996861	2.16518527	1.83170707	1.90527399
SLC6A2	0	0.09965218	0.10231815	0.01195765	0	0	0	0.00769313	0
SLC6A20	0	2.76094114	3.17895585	1.91579975	0.09155183	0.41223171	4.50342479	0.63810195	4.70097987
SLC6A6	4.10813658	2.93170825	4.07764748	3.25345208	3.30786371	3.421818	2.8970173	3.37247023	3.17331182
SLC6A8	0.79887878	3.71625972	2.10085304	3.49738639	0.61020931	3.20791004	5.02056074	3.94991106	5.34867687
SLC6A9	2.42958436	1.7479565	2.29856894	1.69273924	2.9744569	1.73702746	1.34084294	1.2065623	1.91731167
SLC7A1	5.35446414	3.51915156	3.7722005	3.90871913	4.2090208	4.63908566	4.67238168	4.72621722	5.73070514
SLC7A10	0.01166756	0.03316714	0.1751256	0.0278432	0	0	0	0.09606278	0.02442318
SLC7A11	2.1987637	0.49832043	1.33636572	1.63780884	3.07667418	1.86228799	2.87338046	2.92694513	0.70169031
SLC7A14	0	0.0413176	0.232043	0.02487132	0.00214504	0.06018175	0.00208437	0.01070432	0.00183045
SLC7A2	0.19384096	2.36960243	0.66787422	2.16529219	0.28746135	1.83601903	2.65157962	2.89035901	0.16687281
SLC7A4	0.07261039	0.94296678	1.05241987	0.0526413	0.14810865	0.7112674	0.0610962	0.03403734	1.58609525
SLC7A5	6.07123677	2.92177462	5.64579104	4.87830112	3.15925216	5.95595313	5.81070251	3.95504679	4.52152761
SLC7A6	0.80658199	1.09656237	1.88886674	1.57790919	1.3715061	1.43766811	1.49950247	1.41925899	1.99218385
SLC7A6OS	1.57709161	0.71734243	1.07883766	0.86581516	1.10478736	1.22691228	1.21253994	1.02583691	0.9905056
SLC7A7	4.01602347	2.93913556	4.13021404	2.85920121	3.73552649	3.37916886	3.28279547	3.02040345	2.68990268
SLC7A8	2.72331334	3.83754641	3.32396487	2.79176108	3.00197597	3.05825393	3.09755108	1.85330385	3.32747337
SLC7A9	0.32528169	2.83706793	1.00356243	0.08182951	0.11622352	0.1515921	0.00837616	0.20628366	0.30474311
SLC8A1	0.54233622	1.68192998	1.50878891	0.70532949	1.72868559	1.50184317	1.09124872	0.59950712	0.6135328
SLC8A2	0	0.91815115	0.0785322	0.19169375	0.06722944	0.69232107	0.00392832	0.02012101	0.02737204
SLC8B1	2.66221561	2.41224162	3.07584919	2.40342937	2.7589823	3.15481033	3.07077943	3.42288703	2.53432351
SLC9A1	4.32003534	4.28811259	3.73130776	3.95290678	4.6202083	3.34578771	4.07301226	4.49155231	3.68934565
SLC9A2	0.03013507	0.08796584	4.44163037	2.91010695	2.26368244	0.39662272	2.58338278	5.85638717	1.39071686
SLC9A3	0.02595623	0.33338853	3.98554871	2.14405272	0.57733361	0.01436309	0.15588703	1.33848228	5.62601993
SLC9A3R1	7.24598589	5.01886661	4.83718006	5.08757215	5.12519541	5.04276824	5.45516884	5.64232069	6.28526073
SLC9A3R2	3.47032096	4.25377535	4.1720934	3.48092889	2.93548802	3.75742862	4.16104737	4.15769321	3.71604824
SLC9A6	2.21823367	2.33832007	3.53685224	2.60133698	3.38227599	2.71057698	2.5063853	2.37636153	2.11940941
SLC9A7	1.84534923	1.23526969	2.4676136	1.51426636	4.30342999	1.89703854	2.13313468	1.52203977	1.64958055
SLC9A8	2.48652949	2.43343917	3.33238847	2.31927402	2.56725262	2.48114534	2.23830012	2.14685559	3.0479052
SLC9A9	0.95569303	3.53915157	2.2777737	1.94688368	2.2857868	2.55982384	1.25325852	0.98485632	0.64345916
SLC9B2	0.28242033	1.77198622	1.62853905	1.64322493	0.70601859	2.91106582	2.33363502	3.78565391	0.67845361
SLCO2A1	1.53760258	3.77385011	4.62655557	2.63853003	2.06601501	3.7618582	2.69056157	1.95763083	1.68339394
SLCO2B1	2.93276943	4.14871598	4.06719097	3.47344396	4.24239351	4.23564495	3.81656881	2.84754656	3.50443374
SLCO4A1	3.89420584	2.14233671	3.26310375	2.93230399	2.47008631	1.27806716	3.60775806	3.68331438	4.55029779
SLCO4C1	0.02707466	0.20403407	0.17197279	0.34151855	0.08975104	0.8539235	0.0522139	0.20122982	0.01429477
SLFN12	1.01058401	1.83708597	1.81230357	1.21132332	2.67581179	1.74602313	0.69235278	1.42330127	0.44946282
SLFN13	1.15488471	0.64721214	1.9138803	1.70694845	1.12943719	2.48146723	0.39625232	3.15997324	0.28255876
SLFN5	2.97101579	3.2920297	2.87941091	3.04420375	4.22653294	3.39388453	2.9599076	2.24527072	1.70943489

SLFNL1	0.10594012	0.05231866	0.23615393	0.09805442	0.12945369	0.04285368	0.04485134	0.04175777	0.07364832
SLIRP	4.50031324	3.78292861	3.4229528	3.97699756	4.76978554	3.82326956	3.60359703	3.65170427	4.22337857
SLIT2	0.39761919	2.97744095	0.88851573	1.81440386	0.3674055	2.338263	0.54990876	0.35580572	0.1726821
SLIT3	0.43754934	4.56837512	1.84466407	2.56425925	0.55080718	2.81630048	1.05183223	1.00804908	0.76810735
SLITRK5	0	0.46739324	0.2365309	0.02465514	0.02635552	0.37847166	0.00206611	0.1527058	0.00090749
SLK	2.99737644	3.87654462	3.05926918	4.52548165	5.06080738	4.69748653	4.11423688	4.22485875	3.5425602
SLMAP	2.67319592	3.77355922	2.97534943	2.97740984	2.72373801	3.79981014	3.2801831	3.10100278	2.60557047
SLN	0.22317066	0.29112791	0.11180157	1.55605773	0.02457067	0.31715776	0.22269015	0.22890452	1.65067371
SLPI	4.46372473	4.56978282	7.18127328	8.39441937	5.69013063	6.6572732	10.0608349	7.38800397	5.19912636
SLTM	3.42240636	3.18554276	3.31237764	3.78386788	4.17639955	3.2411729	3.28597284	3.10989687	3.63207208
SLU7	2.65282048	3.4436159	3.59956109	3.50518641	3.59774005	3.199707	3.36479828	3.50771206	3.61112208
SLX1A	0.02928275	0.01684578	0.04212272	0	0.04485483	0.01082834	0	0.01700163	0.01546617
SLX4	1.32680755	0.97213369	0.79849675	1.08782618	1.33124551	1.14830433	0.72203849	1.13762683	1.00001586
SLX4IP	1.43942716	1.28811363	1.87162409	2.02831264	1.66966583	1.29430822	1.66171298	1.02915298	2.16125147
SMAD1	1.98270851	2.59394421	2.15070499	2.32995614	2.281264	2.64930291	2.27623052	1.96917681	2.48788629
SMAD3	3.57922639	2.6622837	3.62799672	2.92395517	3.30227465	3.53462012	2.51666062	2.76651911	3.34782866
SMAD4	1.62875992	2.54229124	2.62088876	2.62296506	2.43949593	2.62135909	2.66706232	1.60175291	2.80866053
SMAD5	1.91386462	3.47617788	3.60154574	3.42889277	2.85559986	3.43533038	2.74558972	3.51818935	3.4191112
SMAD6	2.8267845	0.95445506	2.39579603	1.15252218	2.13820998	1.27691697	0.94776999	0.42587005	1.75051718
SMAD7	2.36357082	2.21686651	2.48599504	2.52653959	2.15451961	2.97604816	2.90296263	1.27271078	0.99468871
SMAD9	0.11030078	2.34935598	0.83034916	1.25080177	0.38656397	1.93860051	0.60457502	0.51986786	0.32259907
SMAGP	3.87314959	2.94221764	4.82555365	3.57140045	5.15653757	3.82728757	3.32155838	5.29604344	4.24647149
SMAP1	2.59575649	2.36711753	2.00477583	2.36827454	2.78305559	1.75611456	2.19981436	1.94222991	2.64815197
SMAP2	4.14772956	4.30522703	4.95897011	4.43288283	4.67766777	4.38192375	3.76797574	3.27498851	4.58642574
SMARCA1	3.68847556	3.48904006	4.67124792	3.78048513	1.17347492	3.52204959	2.6059619	3.52098597	0.77838253
SMARCA2	2.87796426	4.03152221	2.97268605	3.75474513	3.02327947	3.92583774	3.48766469	4.60973132	3.34904104
SMARCA4	4.16926773	2.98892096	3.24283145	3.57731764	3.887286	3.563041	3.47430671	3.73535782	4.18335487
SMARCA5	3.09590686	3.72724001	3.52599618	3.9967791	4.11726406	4.01006434	3.58724464	3.62689145	3.97492638
SMARCAD1	2.07766679	2.88025623	2.73375116	3.57194227	3.91227867	3.09166151	2.95465765	3.12366486	4.07475424
SMARCAL1	2.20528379	2.67789432	2.26817999	2.39240623	2.52723556	2.71720258	2.60082701	3.04651259	2.6212549
SMARCB1	6.25777316	4.53916822	4.86479632	4.7019772	4.47989583	4.87382863	4.5344219	4.17058471	5.37501003
SMARCC1	3.94148109	4.25508213	4.06413268	4.82749621	4.77137796	4.59142842	5.0213866	4.46907007	5.14436366
SMARCC2	4.08207143	3.07731975	3.18523029	2.95447784	3.587589	3.15618556	3.18152048	4.02331426	3.64311109
SMARCD1	4.18354146	3.41231987	3.96948983	3.9911486	3.66250703	4.04331146	3.51755153	4.19035043	4.30409691
SMARCD2	5.76603866	4.25381332	4.98661461	4.32978939	4.66711125	4.53280871	4.67568969	5.17494584	5.30450147
SMARCD3	1.18169543	2.57006398	1.53929339	2.12782234	0.76602148	2.33572172	0.93619607	0.78963377	0.62916687
SMARCE1	2.87658176	2.97852444	2.87223517	3.17916915	2.9072651	2.88094943	2.38029987	2.74593819	3.1709849
SMC1A	3.55948755	3.69699407	3.20314695	3.79633962	5.17836203	3.88488855	3.75453486	3.55499194	4.72995654
SMC3	3.5810413	4.13907794	3.34384396	4.217671	4.69992426	4.41677314	4.27831185	3.37334995	4.53735248
SMC4	3.58728488	2.65118791	3.48278729	2.8645634	4.47813591	3.27864415	3.25045198	3.01852623	3.54343896
SMCO4	5.46567185	3.72227436	4.4699246	3.38275329	4.42879063	3.83682171	4.22447537	3.44883144	3.85104953
SMCR8	1.89716761	2.76942625	2.90026804	2.62649401	2.98468782	3.18587805	2.82979521	2.41226502	3.01025341
SMDT1	5.58076124	4.13694129	4.14573922	3.68916913	3.08558967	3.53815122	4.05179122	3.29895753	4.51150913
SMG1	1.78030744	2.26256225	2.65825397	2.76252263	3.68512858	2.74673656	2.40601138	2.58416622	2.80515303
SMG5	4.85206105	3.31324766	4.4822615	3.57925323	4.44331635	3.9728142	4.17886027	4.61028234	4.30514227
SMG6	1.34972697	1.79004771	1.7314746	1.51003888	1.66673356	1.81902345	1.51879246	1.25214206	1.55954753
SMG9	2.7040623	1.43461035	2.02222946	2.13668677	2.73847787	1.77706291	1.60733807	1.97295032	2.24350693
SMIM12	2.49479127	2.09456412	2.9586166	2.49662028	2.52425118	2.64346146	2.09130993	1.53670229	2.3354438
SMIM14	2.16244489	3.64322474	3.36349637	3.23955202	2.9446097	3.16218152	2.80701021	3.4715435	3.0320296
SMIM19	2.74256834	2.86512349	2.3477209	2.86420285	2.70853885	3.0107259	3.26375858	2.44193409	2.80144758
SMIM3	2.10566334	3.84888293	3.76055988	4.10507203	2.7222394	4.21817689	3.10756537	1.85177792	3.48002796
SMIM4	2.92897302	1.67974534	2.16996427	1.19820636	1.41567149	1.25769718	2.08449272	1.16828763	2.0460379
SMIM7	3.17365908	2.82301395	3.70741493	2.98428171	3.44123976	2.93193813	2.59369285	3.2107198	2.57013042
SMNDC1	2.52406884	2.57928759	2.75512778	3.24747922	3.5454211	2.92505925	2.56187593	2.34993433	3.07591482
SMO	0.72796475	2.63987806	1.30641369	1.77050242	0.46366471	2.03575381	1.78885674	0.84941128	1.230217
SMOC1	0.00831769	0.98091142	0.78464929	0.18746202	0.09928135	0.54065492	0.71701378	0.05982834	0.16551985
SMOC2	1.70565783	5.93244602	3.49563935	4.42229611	2.20801767	5.11309369	2.98337296	2.39563221	5.00487478
SMOX	2.39082912	2.21551913	2.58779733	3.06560233	2.28610416	2.18210356	2.21868676	1.65755975	3.26505365
SMPD1	2.79564113	4.2233896	3.27114491	3.81425855	2.84505099	3.71743009	3.52253169	3.36865834	3.08888576

SMPD2	2.78297361	2.45502409	2.84402531	3.12697088	2.47481765	2.34181447	3.09015183	2.90824178	3.29207384
SMPD3	2.54076506	1.40143596	1.78448897	1.10612157	2.40479377	1.44845324	1.77861653	1.3628928	2.48185194
SMPD4	3.95062649	2.7733647	3.61818148	2.75927306	3.48521145	3.49952156	3.87742786	3.25562805	3.82206354
SMPDL3B	3.56476967	2.65911734	4.72249413	3.11299197	3.16754387	3.09131405	4.33382634	2.44111031	4.46736217
SMPX	0	2.7893486	0.02509783	0.62218145	0	1.2744309	0.02082604	0.56311574	0
SMS	5.16867307	5.56163026	5.19864876	5.46212071	6.6312359	5.46115586	5.57620792	5.6020822	5.56609628
SMTN	2.90055181	4.89367504	2.81024783	3.01237426	2.26262903	4.39025138	2.29475026	2.18274071	3.81824397
SMU1	2.93490803	2.90208946	3.20327652	3.4431083	3.44055454	3.34045549	3.13972732	3.10930936	3.43637071
SMUG1	2.83422267	1.80185993	2.62783287	2.04967019	1.79159589	1.99937018	1.72697048	3.03891262	1.98710359
SMYD1	0.04677052	2.89268596	0.12598588	0	0	2.15392928	0.0328223	0.01518783	0.06115331
SMYD2	2.4390905	2.18825489	2.88137068	3.11076532	2.70688286	3.01832452	3.62479678	3.08406719	3.24271774
SMYD3	0.8297289	0.89556986	1.04691098	1.06044631	0.59066197	0.87392718	0.96277622	1.08329508	0.88895708
SMYD4	0.91336504	1.36498598	1.52323386	1.2670443	1.69142199	1.36842176	1.21194214	1.01438506	1.60856214
SMYD5	3.32817553	2.65827113	3.26326859	3.23174648	3.10228519	3.63086778	3.20524335	3.46789973	3.57642855
SNAI2	2.03548241	4.54113401	2.52571864	4.66680709	2.55148429	3.34824849	2.79301634	1.98431601	1.76440779
SNAP23	2.5644591	3.45639427	3.80557107	3.4795491	3.95634192	3.61542388	3.67895429	3.13463081	3.4418287
SNAP25	0.03220704	1.0668347	0.55340661	0.69467217	0.09071922	0.73428536	0.11247612	0.81310036	0.08315479
SNAP29	3.7620413	3.53447204	3.41582719	3.13646758	3.52638821	3.47889085	2.91098975	3.1160926	3.28348533
SNAP47	2.39862223	1.85913078	2.32581131	1.99961627	1.93326724	2.2820323	1.9804736	2.82255341	1.84848315
SNAP91	0	0.19082183	0.08829498	0.0421171	0	0.1363007	0	0.03340162	0
SNAPC1	1.33444845	1.95679721	2.22110261	2.38950506	1.51765824	1.82541472	1.58145206	1.6393098	1.74834669
SNAPC2	3.32458526	2.95826882	3.01620624	3.01125518	2.07772128	3.02049264	3.26254622	2.77335363	3.28744412
SNAPC4	2.31712616	1.29100755	2.08726428	1.91031857	1.8993281	1.71477454	1.48331494	2.0859197	2.33091885
SNAPC5	2.73954608	2.73500587	3.48426324	2.52758304	2.5439469	2.90114798	2.08697685	2.05076914	2.34343832
SNAPIN	4.00911473	4.01883465	4.07071766	4.04177097	3.80237908	4.17621298	3.69690329	4.28178556	3.67702195
SNCAIP	0.05371706	1.64956727	0.5019126	0.79625534	0.10917102	0.92478056	0.42290889	0.27026049	0.05641819
SNCG	0.97994037	2.51962597	1.62895833	2.39003792	0.55996411	2.43268141	1.02201781	0.62175002	0.42303742
SNF8	4.42509721	2.82913064	3.18864646	2.79346954	2.79891235	3.09004167	2.99194557	3.19483561	2.78785753
SNN	3.02623524	4.24626654	3.07331935	3.04492857	2.53891979	3.43260964	2.85262582	2.32390189	3.48121537
SNRK	2.3183865	3.54991403	3.26986945	3.12579048	3.00958208	3.6019103	3.05346834	3.17393943	2.60418657
SNRNP200	4.56723395	4.05341859	4.21236575	4.53991831	4.82034647	4.45344867	4.94858372	4.82324665	4.95136451
SNRNP25	3.47631111	2.80653427	2.39954714	2.65979415	2.91655545	3.0511786	3.70576061	2.72455181	2.59200008
SNRNP27	3.93460307	4.06484894	4.26141335	4.08310145	4.50668551	4.03683667	4.07720607	4.16193811	4.04561388
SNRNP35	2.10359339	1.35591557	1.9545959	1.60148202	1.91816386	1.4257828	1.44417018	2.16584354	1.68318482
SNRNP40	3.29855932	2.79557095	3.69583932	3.44619971	3.83558942	3.57124987	2.97050967	2.20242776	3.64141151
SNRNP48	1.24093079	1.49817546	2.12077863	2.84257595	2.14184352	2.22265743	3.17636525	2.77663023	2.95644967
SNRNP70	4.89487206	3.43571105	4.04130676	4.21789436	4.58183944	4.17087701	3.91663349	4.30767739	4.66882942
SNRPA	5.49448905	4.30495751	4.54615507	4.90607467	5.6144756	4.84276781	4.77344209	4.40336449	5.53755539
SNRPA1	3.31580071	2.51819382	3.97305797	3.70957708	4.39486874	3.3427144	3.14188153	3.46035536	3.54193717
SNRPB	7.88786121	6.62000551	7.46732726	7.20034334	7.18178704	7.00027745	7.19082027	6.4227925	7.92970896
SNRPB2	4.27951675	4.63839631	4.46505328	5.23563752	5.07291077	4.53019373	4.42540873	3.38629597	4.93607437
SNRPC	6.69087722	5.39023687	5.82806055	5.97347883	6.24786044	5.62283159	6.19327878	6.12788657	6.2499418
SNRPD1	3.89966684	2.82814061	3.93217139	3.7635226	4.40859647	3.45100433	4.12613047	3.37012177	3.81983299
SNRPD2	6.90787612	5.75614959	6.27714926	5.83135217	6.68197857	5.70484952	5.75442734	5.73908392	6.10754589
SNRPD3	6.18789774	4.89436685	5.04390595	5.21205514	5.66538408	4.85426786	4.88448419	4.60628447	5.77219204
SNRPE	5.15527663	4.58027276	4.65995485	4.99817768	5.52448243	4.7386557	5.5983661	5.4215082	5.46700858
SNRPF	4.95891443	3.36405372	3.81933193	3.94848034	4.63293218	3.41948582	3.63916087	3.94228776	4.28327049
SNRPG	4.80116703	4.31495456	5.02457284	4.43082971	5.15139358	4.43473775	4.74119709	4.18543775	4.69899267
SNRPN	1.4076027	3.70935106	3.44509544	2.75106611	2.32917995	3.01345766	1.91515976	1.38831354	1.18505265
SNTA1	0.8637526	3.36263597	3.9049857	2.42790315	1.13928858	2.7942744	1.7663526	1.88190277	2.30960374
SNTB1	1.17267146	1.87496716	3.6773732	3.3941246	2.11689125	2.45505742	1.79860598	4.44990447	3.04617188
SNTB2	2.17958934	3.02406627	2.68562801	2.78781243	3.0903774	3.2048541	2.92483667	1.84759388	2.0518941
SNUPN	2.47009104	2.59685061	2.63011166	2.65974784	2.50886229	2.64887736	2.480372	1.82121312	2.61733744
SNW1	4.41255331	4.6349857	4.63902613	4.89651501	5.04864054	4.83529269	4.67864984	4.78967274	4.96543507
SNX1	3.19081687	3.67716134	3.32948828	3.20759971	3.07898207	3.54205561	2.81387721	2.37191714	3.44416342
SNX10	3.18127962	3.5527697	3.13705189	4.2654295	5.84977586	4.46331796	3.23910506	2.7100572	3.0192181
SNX11	3.09860601	2.07937179	3.03865601	2.33675876	2.74251766	2.84245119	2.39752873	2.63044984	2.3881027
SNX12	4.81507674	4.24619781	3.95569546	4.06723591	4.74959915	4.17367978	4.05105394	4.1750433	3.95766113
SNX13	1.33177339	2.43286967	2.38704264	2.21638608	2.22845251	2.12482562	2.50585541	2.33436227	2.26146497

SNX14	2.22099537	2.98999856	2.87670312	3.78446246	3.33170209	3.27590508	3.59125337	2.59524226	3.39097699
SNX16	1.6494356	1.71177385	2.6691136	2.17034559	2.19055692	2.0093923	1.55995714	1.98356937	1.46912864
SNX17	5.04108421	4.47220186	4.67592966	3.99663202	4.06451949	4.69456077	4.40808002	4.47216471	4.3964736
SNX18	2.81823659	3.62083025	2.64149859	3.00710546	3.84333315	3.5559466	2.76774656	2.72501686	2.33865671
SNX19	1.2837806	4.0005357	2.92301404	2.64306285	3.65044237	3.30946258	3.66246456	2.81676707	3.68661786
SNX2	3.51746323	4.51419152	4.3950398	4.74730136	4.89873512	4.7997315	3.93619341	3.81700667	3.75935241
SNX20	1.53654787	1.44365495	1.73882386	1.17234281	2.09144848	1.93040392	1.08017625	0.70589388	0.95204874
SNX21	0.90289049	2.07747247	2.23910702	1.80474786	0.62977342	1.79396704	1.39856879	1.57082274	1.47160204
SNX22	0.68997444	0.72890448	0.83066767	0.707841	0.71329744	0.79403046	0.78111898	0.46452744	1.59750599
SNX24	2.10255666	1.97925349	2.34392875	2.57965583	2.37840987	2.47977778	2.50082116	2.02953296	1.18617343
SNX25	0.88463554	1.62220064	1.56273873	2.42596323	1.60783549	1.80810475	1.659395	0.67337462	2.77239698
SNX27	2.84689128	2.52347619	2.83241471	2.80648717	2.9681971	2.63420057	2.81661627	2.63681976	2.57134892
SNX29	1.39506552	2.49278193	1.90603347	1.97936048	2.10676138	2.29684045	1.61851267	1.56039322	1.31834678
SNX3	6.15248525	6.82833283	6.40248798	6.82812893	6.6131921	6.8917223	6.89981819	5.60784549	5.85077835
SNX32	0.08908551	0.31550647	0.33605615	0.34633145	0.49373848	0.24759462	0.20478494	0.33648983	0.315462
SNX33	2.59854647	3.07894605	2.71439126	2.46454304	1.38676713	2.66495943	2.92881564	2.63822191	2.89245856
SNX5	3.30884666	3.95257818	4.00444532	4.8320693	4.70725803	3.9556896	4.39667656	3.1654685	4.74464591
SNX6	3.6672905	4.98875731	4.70485112	5.11091238	5.21490996	5.08469701	4.38820107	4.68876147	4.5627486
SNX7	3.8596631	3.8639219	4.39527078	4.33022633	4.37073437	4.08359782	3.39878198	3.97927296	4.30156308
SNX8	3.90883121	2.1477516	3.01574925	2.48260096	2.20462635	2.67383886	3.205947	2.56138938	2.01953746
SNX9	3.89386016	4.892843	4.32256206	4.77853397	3.89147875	4.78746121	4.98800776	3.96079192	4.70214771
SOBP	0.32055616	2.33905209	0.78381042	1.19308332	0.27456752	1.68688264	1.6181594	2.36071958	0.84653075
SOCS1	6.36230455	2.77048911	3.67802644	2.42421743	3.85966969	3.48718941	2.62002082	2.11179512	4.32907761
SOCS2	0.37656754	2.61925509	1.78138003	1.71334503	2.27365268	2.10013928	1.66004296	2.09095651	1.86301512
SOCS3	6.43271497	5.06342387	5.95807726	5.45782396	4.63319407	6.39579749	4.68997935	5.43499418	4.37116402
SOCS4	1.73302295	2.70497348	2.84300016	2.74563962	2.76088987	2.75034142	2.07230334	2.61462112	2.47623264
SOCS5	1.73267564	3.39704853	2.63370587	3.33776939	2.48544346	3.07720298	2.66128879	2.56380966	2.22233798
SOCS6	1.7153907	2.76523667	2.44782063	3.7161079	3.4726101	2.9814331	3.81012411	2.15939669	2.93761548
SOCS7	2.5161765	0.99297398	1.51857297	1.67933485	1.44760117	1.51677863	1.60987735	1.79866653	1.74235182
SOD1	5.83867239	6.01006828	5.23476755	5.94523665	6.29227053	6.11306971	6.64870445	6.52549804	6.4408341
SOD2	4.78047952	4.50814452	5.32202292	5.31981874	5.3674012	5.29652139	4.46387034	5.31790287	4.0354869
SOD3	4.08765435	6.15450866	4.59586672	5.73098496	2.86722331	6.3929757	4.41196593	3.04633371	3.33150016
SOGA1	1.17039245	2.66559881	2.2832595	2.31013425	1.33954763	2.13154598	1.75575407	1.21093322	2.08666483
SON	3.51247043	4.6821223	4.08526813	4.86450369	5.36959338	5.01541765	4.67826197	5.02645476	4.91980511
SORBS1	0.72983836	4.97338107	2.31792902	3.32119617	1.70540442	4.72621977	1.20417995	2.60452655	2.00162478
SORBS3	2.77180707	3.69561364	3.48946244	3.40025042	2.49513483	4.2105888	2.53955084	3.36194375	1.69548374
SORCS2	0.37842891	2.30714883	0.58756541	1.10542918	0.11932249	1.38100845	0.68529654	0.35498768	0.36510748
SORD	2.82596232	2.3865077	3.91893943	1.73674118	1.98415521	3.06874326	3.0132019	2.853064	3.88922885
SORL1	0.91420513	2.5199206	1.17108179	1.96852203	1.87866098	2.91146018	3.11993271	2.20065656	3.08963509
SORT1	4.2600744	3.7189409	5.16295928	4.2770217	3.64225477	4.32016221	5.19848328	3.72051156	4.28321654
SOS1	2.4975071	3.75355774	3.06841693	3.43523738	3.95721293	3.6481527	3.19033045	3.20766604	3.52253005
SOS2	2.2872883	3.34570997	3.05808531	3.114892	3.10800595	3.0862874	2.90048363	2.94161394	3.48776994
SOSTDC1	0.14032073	0.70139188	1.47434265	0.9220219	0.13840326	0.50244079	2.82957578	0.3950249	0.02305399
SOWAHA	0.01130562	2.35199138	2.24140212	0.68181784	0.15874744	0.41717481	1.7175429	1.02215145	1.96458039
SOWAHC	2.74089236	2.77232418	2.11995356	2.93707666	2.08909798	2.65251324	3.01064173	2.34868059	2.25983777
SOX10	0	0	0	0	0	0	0	0	0
SOX11	0.21692025	0.12382163	0.48090143	0.33427014	0.03057408	0.08208906	0.23924235	0.12640215	0.09357273
SOX13	1.66078535	1.86489425	1.26571466	2.66059854	2.82250931	1.71111998	2.61770962	1.82550701	2.73676321
SOX15	0.57704653	0.92708764	0.45116582	0.50884442	0.37057321	0.70702517	0.27031695	0.23500403	0.60122907
SOX17	1.12862518	1.5618807	1.21708726	1.28105696	0.72351357	1.77128003	0.72433277	0.49304705	0.41631428
SOX18	3.53580382	3.66306133	3.7106166	2.6179333	2.4528591	4.28235763	2.44726087	2.18481065	1.99451195
SOX21	0.05154469	0.05166611	0.66421311	1.00277269	0.08626995	0.02863553	0.07646904	4.04240382	0.02054079
SOX4	5.20098619	4.59648083	3.5529185	4.3831991	3.56217717	3.74404339	4.01186712	4.35469382	5.57847158
SOX5	0.07590302	0.66268846	1.3579273	0.54483808	0.23928469	0.50158831	0.25788456	0.42780311	0.15132876
SOX6	0.40511898	0.39791963	0.65139021	0.43092892	0.16001134	0.17811886	0.16367474	0.24361752	0.24852585
SOX8	0.07217253	0.33851478	0.23014496	1.74680411	0.07885797	0.07649149	1.88041209	0.08611981	0.02570398
SOX9	6.6617901	4.72178694	5.31105629	5.82059971	4.89086843	5.37019164	5.47815171	6.54989542	6.46374551
SP1	4.39758845	4.11354854	4.55458972	4.33094305	5.26971706	4.63238555	4.22596104	4.86353039	4.34358773
SP100	2.31674087	3.63839229	3.07357532	2.95939953	3.94825261	3.66738282	2.5834869	2.77733877	2.98515814

SP110	0.86440766	2.23239938	1.589682	1.45980526	2.27977094	2.01895398	1.17770765	1.21349274	1.17827046
SP140	0.85243931	1.08748495	1.35958603	1.09235196	2.31244658	1.38749392	0.84949766	0.63336711	0.64503497
SP2	4.31229868	3.39518069	4.02312605	3.86359621	4.12045295	3.88384209	3.66053138	3.53855109	4.18509887
SP3	2.41622594	3.08698713	3.85088086	3.37185071	2.94312406	3.31237652	3.38265948	3.49585127	3.09588887
SP4	1.20719104	1.40616622	1.11448933	1.84853861	1.99119527	1.84425138	1.66051746	1.1865036	1.21317794
SP5	1.67030061	1.01830359	2.3510527	0.26140502	0.08465779	0.28780207	0.16965783	3.12403026	1.39550483
SP6	1.70332545	1.53591745	0.46538752	1.17209371	0.61071734	1.09837935	0.56794816	1.13241712	1.98385435
SPA17	1.53971432	1.43516915	0.77550778	1.71878284	1.85683556	1.26935498	1.62164272	1.00861873	1.57884716
SPACA3	0.14102717	0.06633936	0.37490966	0.01766809	0.03605254	0.01084588	0	0.15162566	0.3074304
SPAG1	1.34436893	1.45438341	3.88319794	2.72914476	2.11676422	2.24384028	2.22727396	2.56822249	0.74649824
SPAG16	1.40379886	0.7210685	1.54307014	1.19904681	1.11922971	0.77056486	0.30168944	1.38511029	0.08557384
SPAG5	3.01631979	1.45416693	2.56385705	2.71160971	3.20818699	2.47317946	2.70335809	2.36588359	2.95435906
SPAG8	0.18803196	0.30322518	0.24004722	0.25686292	0.20709991	0.24798115	0.07038748	0.18386487	0.18471192
SPAG9	2.94253511	3.07827022	2.97155253	3.34190185	3.5026	2.98181796	2.84726878	3.16554307	2.97973937
SPARC	7.50122772	9.15177795	8.35642326	9.61861317	7.2862196	9.01642474	8.57950892	7.32637798	6.74327986
SPARCL1	3.2774877	8.19109877	5.92541017	6.83779452	5.35944484	7.24750588	4.40994081	4.03991229	4.18681907
SPAST	2.13810278	2.67278561	2.96197745	3.19227713	2.83486699	2.77925063	2.99731066	2.85524863	3.05845703
SPATA13	2.72231877	2.32025398	2.81472401	2.36927382	3.45976703	2.34803792	2.20863116	2.62815672	3.2515637
SPATA2	2.96193374	2.27584826	3.55819624	2.76245788	2.50764686	2.40113986	2.20657395	2.5021393	3.69009444
SPATA20	3.75849809	3.12145202	3.5344622	3.21159856	2.4744614	1.99685926	2.3946757	3.68909593	2.94349786
SPATA22	0	0.00728077	0.28328843	0.00762505	0.0310641	0.00467448	0.01517596	0.00978952	0.00668269
SPATA25	0	0.5064244	0.80519681	0.33373662	0.34170394	0.22855008	0.42179327	0.40222508	0.79636509
SPATA2L	2.6927776	1.78843556	2.31558953	1.72870401	1.83874972	2.53553768	2.56033458	2.69340264	2.58162229
SPATA33	1.00487954	0.47542674	1.10845692	1.27444273	1.03812525	0.87206718	0.70396809	0.84510574	1.28396692
SPATA5L1	2.64923976	1.34845925	2.00218064	2.07170567	2.40212319	2.01479253	2.42099501	1.33876528	1.78098222
SPATA6	1.6986028	1.62667769	2.04877632	1.54044587	1.43048473	1.26321965	0.94621636	1.77527245	1.12466545
SPATA7	0.64039655	1.48341716	0.9893576	1.3876942	1.14210661	1.15995368	0.5545455	1.02552041	1.18850026
SPATC1L	2.86827106	2.18513605	2.37874861	2.39371534	2.29736554	2.65245522	1.59254551	2.87070841	0.73631653
SPATS2	2.62366594	2.07716157	2.39537834	2.6528443	2.20499524	2.3141837	1.98961283	2.78514579	2.30021473
SPATS2L	4.92119596	4.20917313	3.7847779	4.32452363	5.1889179	4.74170548	4.42752852	4.22448449	3.24617524
SPC24	3.26637648	0.8603607	1.84168134	1.25257524	1.99349341	1.67524896	1.83053931	1.2501501	2.01775017
SPC25	2.55028403	1.92527342	3.15557491	3.51065391	3.76053831	2.78949743	3.13128468	2.27959371	2.93274968
SPCS1	4.75727012	4.16185997	4.58342365	4.36095392	3.8974363	4.1140546	4.75520203	4.49682362	4.28197427
SPCS2	4.66854609	4.09472097	3.98565066	3.85940087	3.95786261	4.18415014	4.40516024	4.34663091	4.2488319
SPCS3	3.96279919	4.34201958	4.69669796	4.80078859	5.18418139	5.23343183	4.35290763	5.00452432	4.73854458
SPDEF	2.8774354	0.4386348	4.6300597	0.89771371	2.92177944	0.70524414	2.27962281	6.2629432	0.55753695
SPDL1	1.56910691	1.43905238	2.24381272	2.88367334	2.7302972	2.37980412	2.19542101	2.08845706	2.07018455
SPECC1	1.2214443	1.1157184	1.83656399	1.65430531	1.82647113	1.53486355	1.60514503	1.03117707	1.66051795
SPECC1L	2.42402167	2.34139589	1.85756918	2.13136452	2.01078676	2.22071367	1.50045458	1.33312592	1.62864096
SPEN	3.49645871	3.20646797	2.6329878	3.16145812	3.66267576	3.369535	2.95247823	2.89201113	3.46939595
SPG11	2.52209734	3.1191011	2.92031364	2.71304485	3.31702743	3.15120128	2.54824874	2.38984915	2.69357235
SPG21	4.28792587	4.47248682	5.19859277	4.81177263	4.89896265	4.82308788	4.71061622	4.46456113	4.71738247
SPG7	1.82812281	1.80377891	2.39159181	1.80005979	2.03321055	1.92656437	1.75176272	2.10366111	2.54918716
SPHK2	2.44678981	1.7576837	1.5397159	1.59468428	1.58714022	1.31468868	1.44296391	1.65615562	2.62133224
SPI1	5.36564804	4.82894393	5.40318388	4.42648865	5.12547112	5.43148367	4.62529202	3.6087955	3.8115778
SPIN1	3.33174975	4.50922456	4.46738171	4.64002147	4.27774092	4.730038	4.27126808	3.26497808	4.20728989
SPIN4	0.2806694	2.00055051	1.78720091	2.61456152	0.84302131	2.47354466	2.35231622	1.85706321	2.31240401
SPINK1	3.41194593	5.60937297	9.60238409	4.18537264	8.03177191	2.29671528	1.54640272	9.6811916	7.0393797
SPINK4	0	6.1614731	4.400562	0.5720726	1.42733092	0.06015359	2.38078807	1.13158502	1.28779708
SPINK5	2.18649399	0.22459174	1.35972574	2.91954293	2.52606552	0.15077919	0.17780797	3.06284194	0.11348058
SPINT1	7.46659747	4.74692234	6.46343351	5.95599209	6.03610399	5.17847454	6.44472097	5.91974511	6.41879328
SPINT2	6.71826127	5.07324145	6.1122674	5.34608182	5.96767007	5.33543996	6.52504259	5.99312075	6.12158092
SPIRE1	0.3840086	2.05650039	2.31608117	2.42126342	2.66096871	1.96995574	2.67397062	2.49659586	1.15155006
SPIRE2	0.57626109	0.67366967	1.58172109	1.38386331	1.11044621	0.79346111	1.55941767	1.90053208	2.86699178
SPNS3	0.21412911	1.82123995	1.37737414	0.1543697	0.32114507	1.71170473	0.190111	0.45800251	1.09951177
SPOCD1	0.74933233	0.34203683	0.54959213	1.26372351	0.35744395	0.61407066	0.61288739	0.59307882	0.11431839
SPOCK1	1.82744729	4.19422446	1.98621425	4.18085878	1.55377191	3.58966227	3.75788036	2.30615795	1.12624067
SPOCK2	2.90374701	2.12988351	3.16971973	1.75054953	3.49469949	2.65632465	2.26113672	1.73229489	2.20166283
SPON1	0.46079669	7.1309303	3.10037417	5.1531376	1.24089829	5.17539204	2.95672927	2.09582918	5.07452886

SPON2	3.17917215	4.15530955	3.44957375	3.7644757	2.13860702	4.11003842	4.20283786	2.7517373	2.78856186
SPOP	3.28665247	3.8558671	3.28675732	3.50977067	3.39577714	3.33730466	2.58921058	2.94075157	3.31793388
SPP1	10.5289417	4.48678973	5.95298974	6.50419391	5.84186149	7.55650766	10.5922791	6.51961006	5.82206821
SPPL2A	2.99849175	3.18618369	3.51893687	3.38081486	3.70472016	3.32245358	3.30250057	2.68805246	2.56855597
SPPL2B	3.17051464	2.17796014	2.61459516	2.280381	2.23855471	2.05683843	2.1157026	2.75320586	3.77838298
SPPL3	3.74781461	3.34247552	3.58452218	3.4623359	3.21453534	3.44359648	3.0594467	3.69391664	3.79249934
SPR	5.81731714	3.897514	5.31317125	4.54502519	4.80478384	4.98005839	4.89787879	5.8767712	4.95555209
SPRED1	2.40682403	3.23398806	3.60489165	3.52367278	3.437598	3.4728974	3.38697695	2.53395371	3.24218021
SPRED2	3.3779496	3.39586755	3.57538958	3.43506352	3.40048624	3.48660921	3.94815792	4.0001139	3.93022919
SPRR1A	4.15713659	0.34251026	0.74497875	4.39887156	0.36534654	0	5.91970113	2.31227863	0.37303872
SPRR1B	3.43555874	0.575418	0.04185486	5.38623115	3.59026951	0.08390383	6.10340299	0.57222852	0.40190785
SPRR3	6.01935276	0.02868672	0	6.16325719	1.90007071	0.05470547	6.00768041	1.09431817	0.26630702
SPRR4	0	0	0	0.02952576	0	0	0	0	0
SPRTN	1.41814869	1.56202281	1.9979753	1.95291719	1.91634515	1.80880563	2.24823396	1.96948941	1.69768933
SPRY1	3.1800855	4.79265594	4.45822167	3.92198447	3.79700003	3.64840927	3.53891639	4.18568626	3.27072262
SPRY2	2.37135099	3.26045684	3.04036023	2.85518748	2.63212651	3.07348637	3.54003469	3.55290408	4.60991813
SPRY3	0.14591989	0.57932359	0.35530228	0.29891713	0.44427503	0.39835191	0.3002753	0.1351909	0.42550376
SPRY4	2.96721273	2.54264184	3.59597579	3.20107888	3.52848821	2.96508038	3.38056273	3.68547964	3.4665019
SPRYD3	3.97372109	3.67475012	3.71118062	3.67381407	3.36579898	4.17526522	3.99490596	4.59010775	3.56461687
SPRYD4	1.00051893	1.06445744	1.95985721	1.50186986	1.16083027	1.3029354	1.03616504	1.14900615	1.61107123
SPSB1	3.40880498	3.50294791	3.45337518	3.78612139	2.89952631	3.66563439	2.8025528	2.68169394	3.43892367
SPSB3	0.51334922	0.26238459	0.11109364	0.22179453	0.12135685	0.24975802	0.36623219	0.36027709	0.35469516
SPTA1	0.04297832	0.05715862	0.22115527	0.08058788	0.04628061	0.01594039	0.00867855	0.04551272	0.00381676
SPTBN1	5.36446904	4.75318877	4.28942295	5.45924068	5.28135225	5.72192921	5.94246491	6.23269785	5.48022586
SPTBN2	0.88441839	1.07734193	1.39829892	1.98641494	0.33410205	1.85390474	1.35931781	2.92787841	1.60838641
SPTBN4	0.46071579	0.27260076	0.30320355	0.10934563	0.09042009	0.21404127	0.02435046	0.25670183	0.02955262
SPTBN5	0.21884048	0.04988683	0.26799428	0.2765367	0.24078058	0.23485407	0.08580733	0.26451227	0.31907729
SPTLC1	3.9830502	4.07978407	4.61936292	4.43296682	4.49928576	4.5033081	4.26408408	3.57476602	4.42202014
SPTLC2	3.03858467	4.02111338	3.59461197	3.71450751	4.25654013	4.02281258	3.49537228	4.20392142	4.55079221
SPTLC3	0.08714346	1.75893191	0.4043104	1.19738328	1.19736626	1.11770895	0.43258277	0.77081182	1.61956201
SPTSSA	3.35518811	5.57094976	4.81468626	4.66620385	3.69649973	4.25162346	3.87029926	3.13652022	4.95373949
SPTY2D1	2.82778512	2.88376293	2.91581225	3.3321112	3.73947616	3.38139971	3.55523327	3.0952306	2.99214527
SQLE	4.3816684	1.92211453	3.3462241	5.1054897	2.56028532	3.77849897	5.05250754	3.56670528	3.75831014
SQSTM1	6.01309075	4.78715459	5.79650003	4.63026096	4.20341398	6.08979554	4.9228089	6.23910254	4.5048049
SRA1	3.88548861	3.3053189	3.89998736	3.53511557	3.55407765	3.61741319	3.45476313	3.77985275	3.40643719
SRC	4.59597613	3.48704787	4.81882946	4.12800605	3.90279538	3.21257539	4.07718394	4.91215921	4.67393105
SRCAP	4.18432958	3.62601454	3.44751843	3.38559811	3.83697963	3.4086322	3.66618815	3.73876431	3.79229281
SRD5A1	1.4025754	1.83724019	1.56727856	2.40926049	2.11447207	1.84310597	1.70397865	2.13827904	2.14513049
SREBF1	4.42871551	3.31554219	3.34989973	3.54654094	3.28571389	5.01645898	3.86493763	3.51780091	4.94026857
SREBF2	6.41236927	4.22718819	4.61021021	4.80614899	3.62270169	4.86011538	4.23167762	4.22774018	5.14255374
SREK1	1.51181987	1.8799326	1.75676157	2.51339824	2.50366781	2.05642881	1.7571059	2.1806903	2.35502853
SREK1IP1	2.69450525	2.55021451	2.11582155	2.81858973	2.76070634	2.68501283	2.19070128	2.78869183	2.79251665
SRF	5.13681821	4.20301211	3.98005497	3.92429653	3.88638577	4.75292098	4.06001424	4.35563224	3.69043951
SRFBP1	1.77391136	2.26304399	2.48374065	2.71124966	3.31748006	2.6754178	2.29514958	1.77102655	2.62898661
SRGAP1	1.78033011	1.02519306	1.50174278	1.01762174	1.22617174	0.83108507	0.60723844	0.87613728	0.38684103
SRGAP2	1.38493017	1.48404144	1.7835543	1.68834484	1.78379537	1.9147956	1.73123775	1.09663269	0.88063333
SRGAP3	0.18077217	0.6277771	0.63110765	0.34743923	0.62627939	0.39869802	0.18477909	0.37273778	0.32412088
SRGN	6.10070174	8.61987802	8.28634202	8.05348864	8.25327293	11.0254996	7.72614057	6.5587212	6.28508113
SRI	5.00952501	4.86850681	5.08798246	4.50269354	4.4020735	4.41978129	4.57562167	4.70752304	4.40672521
SRM	5.92947574	5.2562248	5.02880508	5.44470496	5.62189999	5.78362358	5.29844857	4.53456879	6.07930063
SRMS	0.18053904	0.33079828	2.74293225	2.07352374	0.11386454	1.39428834	2.78974254	2.02271875	1.07287545
SRP14	7.04351929	6.87175421	6.69544407	6.56036435	6.63893754	6.58311794	6.47256212	5.69904131	6.61835056
SRP19	2.12643603	2.17818565	2.74595786	2.96457859	3.0987764	2.60659397	2.35666796	1.74919656	2.53248416
SRP54	3.23878555	3.98690747	4.15085643	4.36227117	4.8670081	4.27173477	4.05498858	4.3093684	4.52664356
SRP68	4.14559491	3.28609041	3.69192092	3.64709515	3.70090856	3.7169581	3.4306032	4.5294352	3.75711074
SRP72	4.28986407	5.04797705	5.1407262	5.38304464	5.57211038	4.78691924	5.35466781	5.45473601	5.40103853
SRP9	6.17382274	6.73166036	6.65713062	6.95247764	7.24136	6.9813626	7.40917058	6.75599798	7.24724075
SRPK1	3.50039917	3.33950846	4.14491611	4.15491805	4.95718089	4.32933885	4.88514122	4.99369477	5.27228027
SRPK2	2.07279722	2.90362442	2.82993263	3.07446994	2.6949698	2.84398654	3.08666941	2.75954036	2.33446802

SRPRB	4.70723338	3.32148767	4.0375011	3.77164544	3.55667921	3.83106822	4.54847027	4.21198195	3.83577242
SRPX	0.3121499	5.63465242	2.22466045	4.46483225	0.50886628	4.00624253	3.23411263	1.0888255	1.06300445
SRPX2	1.64289723	3.69173188	2.54790036	3.3291274	1.60901744	3.76654351	2.65980042	2.11176823	1.12271882
SRR	0.82008234	1.72244831	1.48543719	1.61261205	1.78157251	1.30451414	0.98960516	0.85596772	1.47525934
SRRD	2.48774159	2.31035148	2.32728656	2.51063776	2.46048763	2.58050465	1.88046719	1.9370281	2.20168631
SRRM1	3.64729566	3.32515931	2.88478076	3.75217944	4.47397505	3.72580287	3.45347827	2.63188949	3.81941858
SRRM2	6.03486174	5.08554484	4.79398634	5.38522141	6.01300557	5.40028324	5.3416604	5.4609621	5.94991414
SRRM3	0.35539761	0.38202153	0.36079009	0.4708223	0.10091924	0.5729062	0.3480306	0.18395609	0.1099772
SRRT	4.86853672	3.2516996	3.92005339	3.93206129	4.34703737	3.87953599	4.00532744	3.95898192	4.42817057
SRSF1	5.11947958	4.98022601	5.25779551	5.68878824	6.27112772	5.68094248	5.43977166	5.59107803	6.01012081
SRSF2	5.09907655	4.84598839	5.59591419	6.25953229	6.45432156	5.74503193	5.19166297	5.13133688	6.02934502
SRSF4	4.63489793	3.97047461	4.55380312	4.30112838	4.56166484	4.5391418	3.85848563	3.68616821	4.56794093
SRSF5	4.20280053	4.16760875	4.61895396	4.71753053	5.00037995	4.26118101	3.8553212	3.93375649	5.16493024
SRSF6	4.16113382	3.72848023	5.24225769	5.66404665	5.05951948	4.79933984	5.08421793	5.10059825	5.93347986
SRSF7	4.65105752	3.86441895	4.81241999	5.18363804	5.72057266	4.98744102	4.65377513	4.50227468	5.77878266
SRSF8	3.64625491	3.60678695	3.56701099	4.04687653	3.37904448	3.63058701	3.70939365	4.27542983	3.97535326
SRSF9	4.81614891	2.88755318	3.76459943	3.74232935	3.74362107	3.69865671	3.81827602	4.08357314	4.06887748
SRXN1	1.87150017	0.98265649	1.12683324	1.14257974	1.24308291	1.82749696	1.2227725	1.03826121	1.43712015
SS18	3.25768662	3.35137168	4.03396505	3.74501334	4.01589983	4.05411532	4.0866675	3.86411924	3.16623998
SS18L1	1.75506499	1.47415043	2.81788953	2.49984931	1.88658533	1.60355848	1.87177452	1.79709029	2.63416911
SS18L2	4.12002686	3.45927335	4.23387009	4.1743083	4.50757718	3.71930652	3.91304277	3.56334298	3.72098064
SSB	3.1498206	4.08561919	4.40426121	4.994757	4.90249978	4.46842588	4.20364955	3.9265324	4.52774423
SSBP1	4.58909199	3.77032138	4.02758543	3.84948572	4.5626774	3.69365085	3.9103958	3.533029	4.08286537
SSBP2	0.4273519	2.22026474	1.10972954	1.34744065	0.65165344	1.55642152	0.78547529	0.35370365	0.40959681
SSBP4	4.94752964	3.44512911	3.71504965	2.97441949	3.98456426	3.96432527	3.28325019	3.16328619	3.91775903
SSH1	2.05511247	2.45292323	2.2863251	2.42084245	2.60297689	2.71101913	2.1000105	2.27553466	2.12871857
SSH2	2.71679097	2.30885266	2.4148121	1.89075884	2.60564499	2.14965425	1.6544787	1.68921687	1.88684208
SSH3	4.00999581	2.94865536	3.52497701	3.57713502	2.62668223	3.61647859	4.46667511	3.8154714	3.82170158
SSNA1	6.05558436	4.07065763	5.35386349	3.91454105	4.01268993	4.62337646	4.20641124	4.64476939	4.87432768
SSPN	0.89469523	3.61066486	1.64608011	2.74305288	0.8918653	2.8996087	1.56980776	0.98387937	0.96634717
SSR1	4.02075321	4.28908671	4.85144169	4.83360262	4.76781266	4.76291295	5.22705232	5.10408382	4.77093128
SSR2	5.4514142	5.1245311	5.54575325	5.21705777	4.88272439	5.03845125	5.72196287	6.09963918	5.35182241
SSR3	5.56369781	5.19909674	5.58831453	5.76121396	5.43655623	5.60752795	6.13347038	6.12760089	5.47052077
SSR4	6.8024935	4.98678953	5.46289329	4.49833419	5.36970179	4.79668422	4.69780955	5.22341171	5.28170322
SSRP1	4.69388711	3.8552047	4.64053985	4.67143188	5.01123409	4.65545402	5.2586838	4.74722037	5.0121918
SSSCA1	4.04981606	3.18123844	3.1942132	3.14069452	3.31378004	3.42172602	3.39491355	3.72559366	3.7511557
SST	0	1.41272638	2.06675466	0.93160207	1.37097309	1.52475206	0.34387228	0.17125442	2.84559811
SSTR2	0.16640039	0.48341693	0.85693881	0.39676501	0.40819302	0.512715	0.44374978	0.17973842	0.12607324
SSU72	4.49843451	3.67069468	3.33519395	4.03956015	3.98312821	4.04739291	3.79621388	3.11080695	4.30692937
SSX1	0.1339714	0.04745952	0.37325539	0	0	0.02048668	0	0.04265025	0
ST13	7.01811467	6.1825761	5.65233643	6.23918107	6.13923219	6.13803806	6.0657114	5.45874344	6.36322719
ST14	6.61546741	5.40329092	5.70326099	5.12431017	5.6226902	5.45378919	7.13634866	5.2019262	6.87756293
ST3GAL1	2.22329636	2.76663659	2.57364538	3.51735361	2.76657217	2.85357094	2.32642875	4.43819249	1.18571449
ST3GAL2	2.69959331	2.42581907	2.49747065	1.90926865	1.95234124	2.19255461	2.13248363	1.2665756	1.5574157
ST3GAL3	1.16682859	1.54992334	1.64325394	1.1579746	0.6707945	1.5856007	0.92746519	0.90640327	0.68409395
ST3GAL4	1.02528604	2.97583574	1.51385455	3.20972502	0.83246864	2.00959669	3.12069547	1.57319112	0.64620184
ST3GAL5	1.09941169	1.87279639	1.73918973	1.75424138	2.41266752	1.91902558	1.34156181	0.69231163	0.76649796
ST3GAL6	0.43673279	0.87604132	1.88837732	1.65754891	0.98735317	1.08732015	1.05643517	1.56422387	0.35234655
ST5	2.4684815	2.60244089	2.39571068	2.05965212	1.8252665	2.4252451	1.74910167	2.84072443	2.44643494
ST6GAL1	1.31814524	2.98828481	4.46978542	3.80918352	3.51129769	3.12596064	3.10789257	5.005113	3.98759723
ST6GALNA(	3.05007097	3.33782899	4.99314162	3.31524298	4.38260521	1.45221932	1.77806777	6.41190816	4.10284353
ST6GALNA(	3.64577729	3.58276196	4.22301069	3.19080081	2.12073422	3.69760601	3.86543739	3.36606125	4.08659155
ST6GALNA(	1.30081182	2.00874459	1.22122419	2.22859274	0.76320416	1.86434922	1.4438207	0.97365982	0.42171173
ST6GALNA(	1.95422827	3.19630282	2.59737714	2.5290962	2.16091273	2.81862547	2.40979979	1.78933972	1.65959973
ST7	2.70303794	2.73776076	3.15759487	2.82574312	2.71332178	2.59292949	2.93000326	3.08613515	2.7900288
ST7L	1.24890501	1.25684342	1.35068139	1.47352894	1.10514215	1.35405742	1.15373001	1.14404239	1.54207478
ST8SIA1	0.12294549	0.53266141	0.35728365	0.22979252	0.27700994	0.30773513	0.14736287	0.13153838	0.07349054
ST8SIA2	0.0122954	0.27304861	0.18793119	0.17752516	0.03011105	0.24362995	0.04368131	0.03525918	0.0289157
ST8SIA5	0.00920358	0.00922558	0.07157044	0.01515346	0	0.01518566	0.00963985	0.0017782	0.01208229

STAB1	1.81243011	4.19540124	4.29940474	2.88806292	2.93062752	4.23211619	3.01122096	2.67738094	2.59760327
STAG1	2.33547066	2.46279825	2.36776416	2.72287137	2.56148277	3.14553575	3.45347137	2.63878185	2.66667653
STAG2	3.06282041	3.84291277	4.95993119	3.86028158	4.97192499	4.05831776	3.77672214	4.27426833	4.07821535
STAM	2.54613211	3.62168116	2.94101827	4.00562744	4.10191829	3.79630685	3.27137767	3.68616567	4.06814129
STAM2	2.64318238	3.111862	3.75160466	3.01112742	3.30187536	3.30570804	2.92420713	3.14186165	3.05637767
STAMBP	2.78666094	2.51646725	2.94485126	2.90935643	3.01285008	2.8341349	2.95229552	2.95093275	3.01111218
STAMBPL1	0.3238869	1.35526246	1.09379741	2.28281939	1.98311875	0.95129264	1.92800886	1.27116717	2.42891037
STAP1	0.02185475	1.80035136	1.2500199	1.11304822	0.72963961	0.62667718	0.17366306	0.34553051	0.23451575
STAP2	3.95448621	3.00556566	3.91966024	3.70527428	4.3352578	3.81113226	3.71734369	4.37215675	5.08397119
STARD10	4.54563163	3.96260061	5.9597672	3.64318986	4.98464108	3.7177258	5.33315165	5.76960018	5.3435691
STARD13	1.84156901	2.96032192	2.31727396	2.13885315	1.83950686	2.84355403	2.06851488	1.42451797	1.51684691
STARD3NL	3.50504686	3.20996711	3.56891656	3.35525235	3.35735849	3.40799786	2.81864329	2.94986796	3.07974495
STARD4	1.72147431	1.59638643	2.18218032	2.91010387	2.19830808	2.82164959	2.52460705	1.30733768	3.96828772
STARD5	0.30465416	1.25019568	0.58034411	0.73937458	0.57390497	0.82866401	0.45642897	0.34868409	1.72430274
STARD7	4.5342642	4.6417483	5.03198666	4.3926031	5.23725286	5.25694908	5.2721547	4.81850691	5.58671523
STARD8	1.11642687	1.84728645	1.96570971	1.47116773	1.56695604	2.26078354	1.47898184	1.25332544	0.69915139
STAT1	5.55610863	6.3379175	5.95003423	5.68047023	7.69237205	7.48313392	5.84178908	4.86083415	6.35929443
STAT2	3.39721296	4.11336184	4.18057068	4.38915413	4.92358013	4.4301763	3.24043498	3.72441855	3.71440962
STAT3	6.32912591	5.40346831	5.24865078	4.87339806	5.4954414	5.33654505	4.94337215	4.99141334	5.0865185
STAT4	0.73098921	1.35193016	2.142068	1.19593167	2.25998996	1.56616976	0.95820398	0.52821695	0.91155159
STAT5A	2.72673748	2.99292001	3.28367848	2.59991459	3.90497723	3.97640332	2.89912798	2.82162431	2.48582646
STAT5B	3.11312938	3.91750612	3.10258603	3.08892298	3.37006944	3.59505383	2.98539067	3.10980136	3.2816441
STAT6	5.00393306	4.87640496	5.21475292	4.66282474	4.83448484	4.93277309	4.2524748	5.32515478	5.27363012
STAU1	5.22255872	5.37051553	5.95817663	5.58352623	5.3534189	5.30744934	5.55268332	5.96809664	5.91662475
STAU2	2.34382389	2.91460956	2.51802295	2.94808623	2.52034278	2.86434137	3.25916619	2.37174475	2.27319122
STC1	2.7801922	3.22860831	3.33203967	4.12537805	2.6570605	3.98476949	4.62268497	2.07712097	2.99002699
STC2	1.15604606	1.05432136	1.24488048	1.41652952	1.00224828	1.10813792	1.18792446	0.76475938	2.98882568
STEAP2	3.6252136	3.09220659	4.13788266	3.38303589	3.72147402	2.99661778	1.49844276	3.5116667	2.38670532
STEAP3	3.41301558	3.56188782	4.09713289	3.13432147	3.44619933	3.68088116	4.51479573	4.71952012	3.64956466
STEAP4	0.21629581	3.7047783	1.26732082	2.73713788	0.82331419	2.2506327	1.59767508	0.71128182	0.45126645
STIL	2.39362263	1.32214463	3.22080235	2.25799485	3.75482034	2.58905593	2.27162744	2.27955664	2.5987277
STIM1	2.98476658	3.20623198	2.86118875	2.77157794	3.22340969	3.27776615	3.40297544	2.93421443	2.65156023
STIM2	2.80887579	2.5644874	2.76011064	2.91735925	3.25725786	2.68876055	2.60147271	2.50912211	2.68295918
STIP1	5.48603243	4.48394676	4.99748117	5.38028531	5.751386	5.49373766	5.73794288	5.34518497	5.4822629
STK10	2.38456806	2.35380976	2.21564863	2.63212036	3.09257154	2.93553346	2.53474955	2.20418056	2.20961829
STK11	3.40678553	2.05156533	2.18425519	2.22994871	2.54561428	2.37009209	2.46134854	2.59240678	2.58311222
STK11IP	2.54274533	1.15043032	1.99308505	1.48782371	1.61646565	1.73808401	1.48710106	1.74907088	2.05168168
STK16	2.83163334	2.28271408	2.67108133	2.32211101	2.39243331	2.29068592	2.31450027	2.70860475	2.71704952
STK17A	2.15203674	3.19280749	2.95888386	3.22482603	3.63923328	3.18893914	2.55493899	2.96119463	2.42509643
STK17B	1.81495803	3.54335958	3.64796191	3.54887757	4.04177536	3.30590986	3.05890368	2.23650664	2.79766183
STK19	1.98712111	0.92619004	1.70970725	1.08094419	1.07986823	1.27512266	1.18007651	1.34566248	1.21816528
STK24	4.55860399	3.01196185	3.32289307	3.11041534	3.90371697	3.33545539	3.73493387	2.95783781	4.02118516
STK25	3.88720205	3.08878294	3.35693857	3.21527344	2.96315095	3.22634548	3.59599563	3.28887775	3.42703955
STK26	2.99402446	2.86679818	4.55193942	3.53617804	4.34025584	3.75075961	3.57146423	3.89547026	3.96171697
STK3	1.5355448	1.82822846	2.33888187	2.13083881	2.12451023	2.11527391	2.21534252	1.6999369	1.62866228
STK31	0.81216936	0.5189882	0.48690721	0.10566189	0.07775291	0.03212559	2.67865112	0.11564469	0.19394579
STK32B	0.07989146	1.29196867	1.20543356	0.58594508	0.29286985	0.91771543	0.24442461	1.50947155	0.20431488
STK33	0.53484553	0.66236967	0.35997366	1.40023597	1.40187612	0.4270122	0.52185922	0.03108684	0.65044536
STK35	2.69245469	2.62828532	3.27833568	3.18705527	2.7211651	3.11757864	3.52175455	2.18133493	3.79703257
STK36	1.74118866	1.22643918	2.09414864	2.09553508	2.03101381	1.70139975	1.62589366	1.97041201	2.05525198
STK38	4.01308085	4.33455683	5.18035044	4.42030189	5.00482598	4.21928181	4.18471025	4.72499615	4.37482666
STK38L	2.10052677	3.93708692	3.89311034	3.98375685	4.12147359	3.48875437	3.5489189	3.44228193	4.94114097
STK39	3.91619728	3.78699613	4.84795895	3.72180601	4.31950646	4.15024033	4.42877683	6.03287398	4.85696491
STK4	2.90632583	3.15416735	3.37848847	3.31782982	3.62531031	3.2051	3.06409807	2.96840856	3.23403901
STK40	4.42859486	3.69761203	4.37225821	3.34809358	3.35063519	3.83307092	3.47438073	2.82738385	4.04081515
STMN2	0.07515646	1.75046984	0.80055433	0.78665596	0	2.73010854	0.01835536	0.24548826	0.16099256
STOM	4.0414781	7.02159543	6.19788579	6.43010972	5.92545827	6.52129123	5.21875783	4.93712502	4.14532682
STOML1	1.32031463	0.69694357	1.12737439	0.65418388	0.6532853	0.75272492	0.54422505	0.45462933	0.81480593
STOML2	5.4118106	4.90321925	5.59678817	5.26944055	5.1547426	5.44626836	5.26784387	5.3099958	5.86881584

STON1	0.50199238	2.94924703	1.09835812	2.41656085	0.82633078	2.77869967	1.06799857	0.59478442	0.35894151
STON2	0.89165503	1.77732774	0.78393686	1.4149922	0.90274855	1.26235168	0.8550028	0.5440011	0.15237833
STOX2	0.0490714	0.92881179	0.78511238	0.61354501	0.8525441	0.86959666	0.13310511	0.60413513	0.03968001
STRADA	1.0298128	1.08858705	1.96917032	1.42397522	1.58914664	1.25206847	0.91609628	1.40416481	1.02003048
STRADB	3.1374724	2.59431458	2.4662534	2.15230543	3.27911469	2.40837526	2.74713515	1.28245504	3.04000606
STRAP	5.45961141	5.52663687	5.7246328	6.17284233	6.21433024	5.85044681	5.81215833	5.45194334	6.15903013
STRC	0	0.00504154	0.11915473	0.03398032	0.00542144	0.00807624	0.01051736	0.00339424	0.04785985
STRIP1	2.34051338	2.19661131	2.11565568	2.41723199	2.51342996	2.47510216	2.13680937	2.44442309	2.81142063
STRN	2.22402657	3.03734744	2.9507729	3.26705536	3.30076636	3.35966826	3.46878477	3.52378086	3.24335165
STRN3	2.61557261	3.11225458	3.0228099	3.31071903	2.99019956	3.16230093	2.66635193	2.83906909	2.81094619
STRN4	4.61059123	3.24460845	3.72877558	3.59665799	3.83213357	3.61794809	3.44614226	3.86346557	3.87132592
STS	2.04211622	3.23077146	3.38175211	2.52254686	4.73283114	2.23898947	4.51874095	2.31023718	2.47011409
STT3A	4.78099219	4.25450853	4.47716695	4.7709653	5.08520227	4.61794473	5.54665389	4.46002719	5.07506354
STT3B	4.71627343	5.12299405	4.80063101	5.76664377	5.60490541	5.57039958	6.08023753	5.72595793	5.44542598
STUB1	4.60149219	3.96818984	3.03884017	3.50412089	3.12777205	3.91560856	4.3266453	4.07997416	3.94614201
STX10	4.96341539	3.57102673	3.86956016	3.54743	3.92864579	3.81771362	4.25313588	3.6542522	4.08865446
STX11	2.5588376	2.59482824	2.89784206	2.8465922	3.60007087	3.72848228	2.45707061	1.64535632	1.85867474
STX12	3.79361716	4.34826299	4.56258449	4.24651395	4.70169952	4.4705287	3.96809038	3.68741126	4.13664194
STX16	2.67474767	2.76020002	3.97925708	3.50382076	3.41078442	2.89852151	2.88923118	3.09367243	3.45736638
STX17	1.637134	2.69387113	2.58534695	2.32377506	2.94750182	2.66089823	1.95398074	2.07681676	2.67893835
STX19	1.47965827	1.93036149	1.40004065	1.82869727	1.94450595	0.50263222	1.04106321	1.46762531	1.36229877
STX1A	0.98417521	1.007837	1.95280905	1.64235312	0.62236109	0.88517796	1.79797085	1.76853112	1.44100242
STX2	1.38300989	2.83452667	2.69721471	3.01204312	2.21827316	2.86740539	2.343099	2.26456891	2.23877983
STX3	3.70961354	3.9339977	4.62274373	3.52302249	3.65405287	3.43923804	4.14142728	4.3189282	5.20725156
STX4	3.72892001	3.35910123	2.59864872	2.78697531	3.21607044	2.96278666	2.97929834	3.21189184	2.82159322
STX5	4.38623533	4.08000865	4.73603907	3.97447743	3.95680433	4.47165467	4.36907488	4.53169795	4.2436628
STX6	2.71835381	2.83032397	3.02356646	3.16358575	3.23779188	3.02618332	3.31419604	3.25236755	2.54401203
STX8	2.44502955	3.36037435	3.1390247	3.14872637	3.61817839	2.87612161	2.45778553	2.1529225	2.79894986
STXBP1	1.4359705	2.56129066	1.98696188	3.38310044	1.47918456	2.21372596	1.24007027	1.26285083	1.94387986
STXBP2	3.56620321	2.16379453	3.29928978	2.71215255	3.13694878	3.0214049	3.2042744	3.40150917	3.05900086
STXBP3	2.22551243	3.30103302	3.5945979	3.97111626	3.66196353	3.4837607	3.0607649	3.41909798	3.67129792
STXBP5	1.23805532	2.42148505	1.88076035	2.41141404	2.50783486	2.58210668	2.29615186	1.45310945	2.34033485
STXBP6	1.21310269	2.39753979	1.68881679	1.36633842	1.9866469	0.56875563	1.4410547	0.29218156	2.51580304
STYK1	0.86893802	0.48694272	1.84203421	1.04117573	1.5584094	1.04637845	2.72825068	1.86234648	0.82200168
STYXL1	3.66920131	3.62415533	3.27297177	3.70399348	3.65302008	2.98885075	3.50362712	3.95588345	3.46946023
SUB1	3.87358631	4.61262878	4.79439484	4.82727168	5.2940629	4.1569881	3.74786705	3.05604526	4.32883869
SUCLG1	4.01745477	4.55323492	4.48306646	4.46136156	4.72159551	4.48056185	4.83425238	4.69220674	5.24465798
SUCLG2	4.17878904	5.15859425	4.87473275	4.35005054	4.53449993	4.95232719	5.82468296	5.64957523	5.24250737
SUCO	2.22358754	2.28865687	3.08746439	2.51435561	2.77135524	2.58314067	2.96496663	3.78866912	2.9238692
SUDS3	3.59050841	2.57685366	3.10459056	3.3168732	3.28729458	3.23673745	3.21452348	3.45760837	3.6799301
SUGCT	0.54575723	0.82361615	1.05629167	1.70246028	0.39608362	1.29598631	0.7508501	1.13722854	0.36530938
SUGP2	2.54862074	1.8325876	2.65650428	2.69292435	3.37807781	2.14779754	2.0137919	2.53549988	2.77367841
SUGT1	2.4562705	2.05756567	2.07083374	2.16491338	2.43382552	2.20405241	2.41729606	2.21513214	2.83001546
SULF1	4.71695418	4.76737681	4.67043593	6.30810838	4.24301295	6.01516264	5.94970435	4.55198562	3.23461295
SULF2	2.74981186	5.630759	5.48027574	5.97714764	2.36965774	5.84812289	6.12466326	2.84415401	2.59085743
SULT1A1	0.78971324	1.94076071	0.91140908	1.00389299	0.85222152	1.08758809	1.32708749	0.97212259	1.21816645
SULT1A2	0.3659225	1.61089194	0.62057828	0.86347756	0.23616199	0.25308699	0.46349299	0.82054285	0.26817637
SULT1C4	0.02493507	1.36893601	1.19599113	0.76176293	0.20208609	0.93084628	0.29204521	0.30078949	0.08969792
SULT1E1	1.50690687	0.49387447	1.14014726	0.15293296	2.14991904	0.83963042	1.11654126	0.20958641	0.14991163
SULT2B1	0.84535123	1.32329294	1.07886297	3.12659386	0.4823879	0.6498523	0.60893928	1.84441577	2.50396258
SUMF1	1.54707743	2.2519221	2.62075354	2.50992172	1.5660646	2.51221442	3.05799848	2.70257332	2.31905247
SUMF2	4.903072	4.57184386	5.78880669	4.70214365	3.79250667	4.62306333	5.27038704	4.86013862	4.76471998
SUMO2	6.37196669	5.51632955	5.95678839	5.56921874	5.73115574	5.56347208	5.65025152	6.00196367	5.75319781
SUMO3	4.80163078	5.33117684	6.07386982	5.94463293	5.53479951	5.74430996	5.88634191	5.77252111	5.60468439
SUMO4	0.16155027	0.37698833	0.47899821	0.26188674	0.40181992	0.31475196	0.37617582	0.16186991	0.10398536
SUN2	5.42788668	3.38116454	3.82058589	3.2238668	3.40732985	4.08736021	3.83176285	3.01419216	3.46750138
SUOX	2.63124446	2.37239693	2.46629214	2.3677117	2.05561595	2.02145536	1.61889587	2.481386	2.22084464
SUPT16H	3.44884548	4.11380692	3.95504706	4.61708143	5.01738551	4.53154775	4.09920758	4.31189599	4.57719345
SUPT20H	2.09328288	2.10461291	2.0749967	2.54273752	2.5210528	2.2965684	2.076749	2.20875208	3.14461091

SUPT3H	1.52762212	0.86621909	1.13466193	1.31325603	1.23719941	1.20050057	1.31468895	1.71954763	1.24174313
SUPT4H1	5.67657117	4.38438054	5.52792691	5.23361854	4.77024814	4.64855766	4.44786468	5.28173998	6.63300498
SUPT5H	4.20197013	3.34413869	3.64119251	3.70105082	4.15227045	3.7626466	3.7752111	4.26687254	3.8930288
SUPT6H	4.46373412	3.60653489	3.44197716	3.69304068	3.88060004	3.7931104	3.38234082	4.0202481	3.70743409
SUPT7L	2.96427742	3.04438274	3.53584952	3.60127029	3.46271644	3.01446585	3.01858963	2.8470023	3.22323213
SUPV3L1	2.41073094	2.41353741	3.33106486	3.14776518	3.69737454	2.88784529	2.54958467	2.74248946	3.43088518
SURF1	4.36223967	4.0128968	5.04091351	3.82367026	3.49878434	3.82545891	3.47339359	4.17798528	3.52118158
SURF2	4.35385374	2.70317572	3.88719515	3.4152056	3.00166128	3.37871069	3.09269865	3.67736811	3.20270765
SURF4	6.79942736	6.22953905	7.08796464	6.05014624	6.08276349	6.53988722	6.59770917	6.7252368	6.53595185
SURF6	3.30838806	2.35335832	2.63038777	2.49966622	2.36775927	2.53239872	2.4073822	2.81912526	3.37180742
SUSD1	2.5408292	2.8477785	3.29089367	2.64470166	2.81672343	2.69516701	2.56121069	2.13465459	2.96397914
SUSD2	0.80447529	2.2937308	1.33211638	3.08826834	0.98671881	1.70301978	0.95146426	1.02087705	0.91871568
SUSD6	3.89770319	3.99650798	4.29172134	3.91328813	4.22561993	4.20534747	3.80281339	3.53581825	3.72706916
SUV39H1	2.70163233	1.64462291	2.06379855	1.89800885	3.05241423	2.17959203	2.00779441	1.98663564	2.33148332
SUZ12	3.53597221	2.89022714	3.23722789	3.84370308	4.11441558	3.56971171	3.03308399	4.17251363	3.45875922
SV2A	0.51541716	1.88503038	0.81903586	1.17221069	0.27463885	1.61476012	0.7700328	0.4125152	0.1181342
SV2B	0.05455629	0.87790594	0.24088219	0.40193099	0.11599486	0.93243738	0.11930194	0.06936197	0.02893384
SVEP1	0.30033729	4.24577115	1.40677175	2.40426054	0.634818	2.45295828	1.47877701	0.75783061	0.4547684
SVIL	2.40982352	4.74300047	2.46561699	3.33814927	2.71258464	4.33905072	2.80773733	2.07309944	2.65343218
SWAP70	2.79007426	3.56035703	3.42997365	3.29379305	3.18507384	3.57779922	3.64095786	2.9003523	2.18572432
SWSAP1	1.720347	1.01000876	1.64347924	1.23265607	0.93027971	1.07577561	0.84013176	1.16174719	1.21214021
SWT1	0.5954724	1.61664804	1.55154473	1.76241072	1.54376265	1.17583638	1.88920901	1.62959261	1.49627118
SYBU	0.73582545	2.34978056	3.7995092	2.90630052	0.69829071	2.00130228	1.72496994	2.30313804	3.0940844
SYCP2	0.01122899	0.23485634	0.54325222	1.02741262	0.30270952	0.23809394	0.13824127	3.79935663	0.1436977
SYDE1	2.20752726	3.10523538	2.43920313	2.58948636	1.58766986	3.15086228	2.42407004	1.80928696	1.56516957
SYF2	3.51437728	3.95578891	3.29761343	4.20797251	3.83556597	3.86001713	3.35031767	3.31309629	4.18859614
SYK	2.22206776	4.12654252	4.62015918	3.61220598	4.21486967	4.62057606	3.87199747	2.85782515	4.37978389
SYMPK	3.49753138	2.55504262	2.9549101	3.02985192	3.48789476	2.7037799	2.41134642	3.54811159	3.55976298
SYN2	0.0442066	0.32431627	0.17435244	0.1771874	1.59630358	0.28938748	0.12846632	0.11857533	0.11028575
SYN3	0.03961812	0.12833077	0.16869335	0.02784446	0.03262131	0.05549246	0.03562296	0.03567799	0.03476658
SYNC	0.51341804	2.57201726	0.59733859	1.25489827	0.11742389	2.11699989	0.84286027	0.38023807	0.4086989
SYNCRIP	4.21496632	4.19143622	3.88032787	5.24217997	5.03611782	4.83404323	5.09576061	3.95779353	4.98905791
SYNE1	0.30546174	1.68294048	0.83325938	1.08023885	0.9885075	1.54609717	0.63824242	0.59012428	0.29903514
SYNE3	0.40798143	0.9560693	0.53418846	0.67800749	0.62511475	0.71034024	0.29055913	0.23300498	0.36024193
SYNE4	0.1374087	0.04871481	1.29342878	1.80735757	0.36662271	1.26456041	0.85402251	0.07576504	0.1164195
SYNGAP1	1.46508155	1.07081986	1.15853065	1.46044783	0.98966689	1.26972359	0.88802153	1.12044037	1.1010825
SYNGR2	7.77477799	5.08638391	7.48882358	5.16047068	5.34425795	5.97652528	6.15397612	6.94781402	6.39503664
SYNGR3	1.00859173	0.6322915	0.47462856	1.9791297	2.11617572	0.92678393	0.32935538	3.06434981	0.56321677
SYNGR4	0.40703477	0.21830965	0.43641577	0.13458908	0.121482	0.06338089	0.28630185	0.47777632	0.1880338
SYNJ1	0.92716343	1.96853365	1.73721545	1.86192083	2.62077372	2.10426532	1.50974513	1.81868507	1.87339071
SYNJ2	1.93598002	2.26110575	2.73362355	2.02524021	2.67965929	2.01956543	2.42654494	1.97900355	2.75142216
SYNJ2BP	2.90749833	2.73319064	2.94478379	2.80002023	2.85942129	2.79696303	2.57260849	3.49044739	3.23184256
SYNM	0.65838556	6.17758753	1.65523398	2.85473683	0.95711341	5.38853209	0.90773245	1.18987556	0.86682408
SYNPO	2.51125869	4.42504614	3.36003444	3.03557538	2.11639211	3.79448957	2.17216723	2.7678009	2.43499177
SYNPO2	0.70488351	6.32114294	2.36085851	3.29681722	1.46283709	6.13750382	1.05784735	1.28252002	1.20046686
SYNRG	2.42170321	2.05838341	2.28414884	1.98575367	2.7082219	2.40418847	1.86414403	2.29502987	2.4033706
SYP	0.50242057	0.34069675	0.55822043	0.14734575	0.22294315	0.33529192	0.1432476	0.95121223	0.26441729
SYPL2	0.13174212	0.73511211	0.4813529	0.20536017	0.07721105	0.73362631	0.24264028	0.13846002	0.08903943
SYS1	2.99924386	2.73771978	3.90794366	2.68727703	2.52925841	2.96503121	2.65991362	2.98822925	3.00012261
SYT1	0.02779109	0.46340578	0.68440863	0.75173209	0.03753764	0.93714686	0.07526892	0.12029705	0.0205031
SYT11	1.54056894	3.02766064	2.64197004	2.21278223	2.41898451	3.03164204	2.1552161	1.44981805	1.17433999
SYT13	3.22794787	2.96694261	1.65385711	2.09492706	2.71882897	1.40858294	0.04747605	4.59817525	1.45835261
SYT17	0.68398946	1.24641338	1.33508542	1.20971711	0.26334452	0.46902479	0.98474685	1.18576953	0.11710187
SYT4	0	0.24199451	0.0235437	0.01957839	0.015101	0.12979101	0	0.00316232	0
SYT7	0.15649403	1.16327404	1.12048265	1.59524942	0.10191562	0.83154025	0.54323469	1.38008064	1.09898852
SYTL1	2.58580045	1.74652907	2.35378147	2.25808175	1.31188444	2.31401789	1.59335655	2.11330891	1.56532161
SYTL2	0.99193677	2.38312036	2.54408608	2.39002515	3.47988806	1.81769058	2.36914578	2.54993665	1.37228331
SYTL3	1.43813433	1.84325687	2.03361326	1.24558675	2.63767128	1.58184329	1.37148799	1.02299686	1.13534807
SYTL4	0.71014119	1.62771185	1.32970661	2.11772689	0.33558161	1.44659613	1.19834305	1.20427354	0.73552003

SYVN1	4.24648802	3.54058861	4.50846873	3.84875604	3.88459115	3.95405496	4.34349492	4.94248765	4.36211974
SZRD1	5.48206766	4.77436972	4.37183226	4.7558276	4.93500536	5.05309425	4.75746383	4.08138326	4.99957721
SZT2	1.56440804	1.45459443	2.10584982	1.40513606	1.7667671	1.63322631	1.08902934	1.7499407	1.65984892
TAB1	3.72617958	2.50159125	2.7014783	2.24322998	2.55212366	2.69420527	2.0820508	2.17981786	2.81563418
TAB2	3.58214679	4.46067913	4.27103447	4.17374004	4.65014426	4.4524615	4.40870685	3.36549891	4.02986351
TAB3	1.7143662	2.13611709	2.58966946	2.00513102	2.9715788	1.96227239	1.87187836	2.28950387	2.14614681
TACC1	1.92403803	4.59389561	4.20726205	4.41874212	3.79945487	4.25731219	3.4365565	4.14698671	2.89605868
TACC2	1.71666874	2.4149203	1.92391857	2.19482991	2.23820608	2.08158269	2.07155684	2.28399647	2.15570526
TACC3	3.95130731	2.046854	2.45090883	2.87906945	3.47629506	3.08483517	2.97477496	2.61358931	3.23433829
TACO1	3.56907088	2.5744988	3.52270007	2.63489006	3.17953278	3.13260834	2.58110863	3.61129141	2.93613645
TACR2	1.80689962	4.75873	0.61128768	1.8845093	0.35824668	3.32246159	0.23766291	2.59684413	0.55753695
TACSTD2	2.53413962	3.57588534	7.49270424	6.46791194	5.94472728	5.57510556	6.39396555	7.76812752	0.57117183
TADA1	2.4213298	2.36485184	2.88627213	2.85911776	2.98689738	2.5350392	3.3037912	3.18063959	3.23582214
TADA2A	1.84813318	1.54315156	1.76071406	1.83402375	2.09884567	1.62827261	1.23796948	1.73099953	2.02984123
TADA3	4.86362433	4.16241945	3.96064843	3.96092346	3.7343786	4.33246326	4.34749828	4.09896408	4.29328468
TAF10	4.15456818	3.59589622	3.45629346	3.65646018	3.58348462	3.91892318	3.2885501	3.89246442	3.80016288
TAF11	3.82359445	3.22389845	4.00770273	4.17969784	3.87588305	3.80920043	3.89135428	3.92579489	3.63473148
TAF13	3.92263638	3.62505904	4.26683441	4.03319632	3.44535408	3.73962789	3.75688483	3.48124102	3.29861235
TAF15	5.29381856	3.49569676	3.77614545	4.33780094	4.6830203	4.0232654	4.21019172	4.18825802	4.27579489
TAF1A	0.94482418	1.2806565	1.78757082	1.82763375	2.06059892	1.77290665	2.16647377	2.36555585	1.32712661
TAF1B	1.91545394	2.27235245	2.25874507	2.39985963	2.90288247	1.96282914	1.64197319	1.99722177	1.41309562
TAF1C	1.79813568	1.49241771	2.57557482	2.69810754	2.2963707	2.22437437	1.8982971	2.55055173	2.77609727
TAF1D	2.52790236	2.33692783	3.21412185	4.08563792	3.80761054	2.72509317	2.87853624	2.91084631	3.62549492
TAF1L	0.03475649	0.02001081	0.0863185	0.01748162	0.03919384	0.02562191	0.00700234	0.04887714	0.04848576
TAF2	1.8058957	2.70459725	3.49508346	3.35631875	2.95963032	3.23482216	3.14730963	3.27308449	3.07886728
TAF4	2.21232213	1.62302125	2.56194811	2.11951848	2.0496557	1.81212598	1.72194115	2.10491005	2.52643652
TAF5	1.99133944	1.12285932	1.35192956	1.65833388	2.32058388	1.69386692	1.72235128	0.99213709	1.73826338
TAF5L	2.938487	2.16851721	2.71094157	2.64869684	2.94733902	2.70508864	3.44130669	2.96116378	2.12946061
TAF6	3.91374014	2.92081065	3.48277645	3.13271454	2.60425996	3.23758733	2.95427337	2.97937932	2.99471086
TAF6L	2.34575683	1.47419914	2.22480269	1.99073355	1.7345148	2.10968027	2.04815659	1.99857242	2.44366555
TAF7	2.37406623	4.87240898	4.98352613	4.69094381	4.75600991	4.4482539	3.56019444	4.82544833	5.73169134
TAF8	2.04940514	1.46672447	2.45239362	1.68472623	1.94762598	1.46319956	1.41990464	2.03429322	1.97346579
TAGAP	1.55272375	2.03808834	2.46386053	2.53326663	3.04974961	3.36264804	1.6915325	1.52541638	1.98371686
TAGLN	5.70403297	8.81524507	5.72024248	6.88730492	3.93647911	8.28077441	5.44450929	4.62866565	3.6116657
TAGLN2	9.08905149	6.85627745	7.50005313	7.35146098	7.69855097	7.76825289	8.33331043	7.84498926	7.54916152
TAGLN3	0.02020677	0.31310268	0.07169537	0.02421261	0	0.23413657	0.01212986	0	0.03173683
TALDO1	6.77771011	5.23859437	5.30885312	5.39713086	5.48966561	6.00156435	6.55127049	5.18230039	5.82187424
TANC1	1.66546527	2.1614699	2.31405696	2.3210484	2.49548494	2.03880377	1.4632757	2.43557097	2.05569433
TANGO2	2.7045261	1.89587516	2.32497635	2.0257535	2.33026119	2.39015289	2.00399811	1.60179014	1.85526182
TANK	2.64860217	3.57175844	3.7822103	3.77954409	4.20604525	3.58119607	3.18698716	3.42677751	3.23693401
TAOK1	2.58610108	3.28554416	2.71584435	3.17808064	3.36659869	3.53171968	2.89920808	3.27203717	2.64250448
TAOK2	3.69833531	2.5547068	2.7094839	2.65781396	3.15598383	2.71257318	2.57002876	2.54336935	2.51720759
TAP1	5.7977624	4.35389219	5.70465121	4.40922886	6.95533584	6.65911765	4.12130036	4.86573851	6.17904862
TAP2	2.63102606	1.87480953	2.67763818	2.75473484	4.17609491	3.69924786	1.68801288	2.7779104	3.65362402
TAPBP	6.74319469	5.25146915	6.77697753	4.45182738	6.08645322	6.20959653	5.90877843	5.37636114	6.11564087
TAPT1	1.53643128	1.81156707	1.80226094	2.05447781	2.33447025	2.08867948	1.87413928	2.22930308	1.78280711
TARBP1	1.27258187	2.08929406	2.73587615	2.30627901	2.61142356	2.19023886	2.68508758	2.91863998	2.70303431
TARBP2	3.23644546	1.93077774	2.6215031	2.27691807	2.26908909	2.3162842	2.23974821	2.63868668	2.59035153
TARDBP	3.59116999	3.21672937	3.53678311	4.03105464	4.24890761	3.82083336	3.66102003	3.45808826	4.05910517
TARS	3.94922027	4.2373246	4.00987744	4.65684744	4.98207169	4.70104998	4.70830865	3.06661448	4.64654688
TARS2	3.25373234	2.43131514	2.82538323	3.02061573	3.13535153	2.85511842	3.6678337	2.80023239	2.86574656
TAS2R13	0	0	0.03181533	0	0	0.12528002	0.01326574	0	0.01165519
TAS2R14	0.39148224	0.39399533	0.58211149	0.49191207	0.59446653	0.28360415	0.29303909	0.2415937	0.50399127
TAS2R3	0.0960365	0.1948025	0	0.0197241	0.0793458	0.01211133	0	0.08665123	0.08447523
TAS2R43	0	0.24104425	0	0.01949548	0.03975539	0	0	0	0.03398931
TAS2R5	0.06197697	0.24923137	0.73635893	0.57260005	0.39064115	0.28419376	0.25950716	0.38352558	0.31297444
TAS2R50	0.03599224	0.04115855	0	0.04308104	0.02227877	0.03962299	0.04298555	0.04153613	0
TASP1	1.80559643	2.02619624	2.23085017	3.09931732	2.05495947	2.24532768	2.07085236	1.51608202	2.71531588
TATDN1	1.45944592	2.10872983	2.31950551	3.01207908	2.27891325	2.43658329	2.67075949	2.2196367	2.36278557

TATDN2	2.77673448	1.94343189	2.28437972	2.00886506	2.31370278	2.20572981	2.52679082	2.06899666	2.00952863
TATDN3	1.62012755	2.11087027	2.52249377	2.17405713	2.19612066	2.02944418	2.3872394	2.79920556	1.85401042
TAX1BP1	3.64632863	4.14741146	3.78579884	4.76838098	4.51359041	4.16901543	4.54984507	4.48855976	4.17980447
TAX1BP3	4.27479966	4.83849889	5.0747071	4.97317303	4.13914673	4.34543533	4.15514981	4.02662285	4.64569445
TAZ	3.13777408	1.99691111	2.04930437	2.01097342	2.06840041	2.0907565	2.04929407	1.974751	2.17767696
TBC1D10A	1.96879139	1.28165563	1.79738324	1.63483754	1.15993489	1.44996282	1.47674593	1.3348885	1.30765513
TBC1D10B	4.18342481	3.19270352	2.961468	3.00446486	3.0440462	3.15722651	3.52709042	3.42404368	3.24212181
TBC1D10C	1.52204986	0.74686325	1.38916675	0.7274007	2.06428964	1.48419139	0.72082223	0.5461669	0.95316606
TBC1D13	3.40058927	3.03729571	3.53904133	2.65142492	2.71226632	3.07649757	2.92890458	2.14984596	2.73310791
TBC1D14	1.90627155	2.85443142	2.78624918	2.66008871	2.64763714	2.65579458	2.72553257	2.74929415	3.42180996
TBC1D15	1.99443888	2.82344656	3.54725099	2.8643264	3.32306303	2.86608954	2.51141422	3.41325923	2.98625129
TBC1D16	4.58805249	2.79404618	3.05095782	2.26021181	2.33315575	2.59926255	3.09323559	1.87487811	2.71209441
TBC1D17	2.98955965	2.69394572	3.42682663	2.75132989	2.57902304	2.95137858	2.57579519	2.99687505	3.22762916
TBC1D19	0.29636578	1.58569014	1.36353425	1.59098522	0.7866744	1.61461864	0.80983281	1.31364608	1.12390838
TBC1D2	2.82940979	1.985507	2.72628369	2.60425943	2.67876	2.89627488	3.147102	2.29475747	1.95956739
TBC1D20	2.79845116	3.42092504	3.80462894	3.632277	3.13331452	3.444272	3.14484247	2.4017953	3.56998033
TBC1D22A	3.73083225	3.02954505	2.62717911	2.73373276	2.59712553	2.89911087	2.80625577	2.45244193	3.40918407
TBC1D22B	2.53638435	2.34344819	3.17621877	2.81226022	2.79174074	3.00196375	3.11022337	3.3222664	2.67122524
TBC1D23	3.0322111	3.14354414	3.29906903	3.33628344	3.46619192	3.27287453	3.32663942	3.10337895	2.75658694
TBC1D24	1.39914946	1.1875917	1.01087384	1.33643642	1.3114827	1.11778466	1.55618427	1.25173544	1.24800301
TBC1D25	2.44323473	2.18253993	2.51257	2.27931556	2.30110513	2.44819806	2.20123459	2.58070265	2.04894751
TBC1D2B	2.81647489	3.53163296	3.07949242	2.54413122	3.00217107	3.04340926	2.29963708	2.22433481	2.50175189
TBC1D31	0.76650534	0.7789223	1.58431186	1.34185833	1.7470294	1.1825379	1.46721946	1.27711495	1.22842843
TBC1D32	0.43387524	0.9308974	0.84379804	0.97758884	1.04138417	0.72358374	0.37820472	0.34694365	1.19595553
TBC1D4	1.28794063	2.10912505	2.18840877	3.18646778	1.82023572	2.9979214	3.6245001	5.01536763	3.84601301
TBC1D5	2.82775137	3.54414746	2.79267833	2.9893456	2.97921854	3.44934411	3.02601941	2.6000356	3.03782193
TBC1D7	2.66827861	1.14625027	2.36128477	1.33089762	1.40802179	1.10331254	1.72690799	1.24496624	1.31766948
TBC1D9	1.96977435	3.52977293	3.45514054	3.10267988	2.56534055	3.14575222	2.89049219	1.88835697	2.42047962
TBC1D9B	4.2299304	3.26760709	3.47471508	3.43532518	3.26503582	3.71473268	3.72818755	3.81034713	3.93692075
TBCA	4.77923598	3.82991955	4.22554764	4.29208437	4.61747686	3.90703595	3.55160324	4.18582166	3.49377548
TBCB	4.40983004	3.34143496	3.70416029	3.66437776	3.52410491	3.72742055	3.32224014	3.35884793	3.48856184
TBCD	2.76983603	2.18304043	2.80337206	2.50422067	2.4351313	2.85656041	2.22323068	2.81272672	2.96998851
TBCE	2.62167696	2.90952426	3.49708329	4.04372236	3.67864193	3.17548769	4.04890577	3.92840939	3.6420369
TBCEL	0.46241494	2.36955657	1.39225081	2.11047177	1.29137115	2.10661019	1.07238137	1.16613161	1.12077423
TBCK	1.52838447	2.1628991	1.67919559	1.92034328	1.71431522	1.54282603	1.09144265	2.20393045	1.9705997
TBK1	2.3950077	3.30389719	3.86655356	3.4899916	4.23573669	3.48313546	2.74758761	3.63401044	3.20524817
TBKBP1	0.74231583	1.69364031	2.02055852	1.63550152	0.9022798	1.99815074	1.47544798	0.45501914	0.68255383
TBL1X	3.37496733	2.6609001	1.88151064	3.08619684	3.76769439	3.04236397	2.94021561	1.60897333	0.79656257
TBL1XR1	3.91661496	4.20870106	4.65990999	4.7315268	4.20007026	4.24137072	4.72187309	4.35750529	4.38320083
TBL2	3.55994334	2.90130941	4.14553365	2.99336381	2.69855141	3.27049295	3.72117328	3.86605161	3.38649197
TBL3	3.05802918	2.36770165	1.64004572	2.27893588	2.33448729	2.44463878	2.87531777	2.66123635	2.85678669
TBP	2.91072383	2.56221315	3.68544703	3.17972443	3.53781048	3.03007468	2.87948606	3.01608304	3.48835435
TBPL1	1.66709585	1.97522026	1.84384002	2.31242881	2.53588693	2.00485264	1.94744539	1.70517163	2.31816508
TBRG1	1.82168562	2.16855972	2.08548951	2.54522557	2.59666984	2.36652092	2.49602293	2.0449017	2.77964306
TBRG4	3.60478343	3.07607661	3.34682465	3.18322104	2.96464519	3.25537572	3.8247704	3.0739886	4.07118451
TBX10	0.02335322	0.64233043	0.23492711	0.12183207	0.08449844	0	0.02791446	0.2417178	1.50804116
TBX19	0.50845857	0.64102212	0.82020436	0.86618219	1.09427884	0.68550295	0.89398251	0.36250594	0.73032231
TBX2	1.31224258	1.47676304	2.50818595	1.5224613	1.22948192	1.31807305	1.9228083	1.3760368	1.16773039
TBX3	1.1018721	1.91081803	0.7805253	2.22830045	0.34711407	1.45465464	2.3300559	1.36044144	2.82083405
TBXA2R	1.10950493	1.19861506	1.41835829	0.76092625	0.4635065	0.87770448	0.87055815	0.63947147	0.3620517
TBXAS1	2.28189954	2.42623412	3.52762848	1.92916665	2.73941056	2.55688114	2.333159	2.82659209	1.62162467
TC2N	4.57588025	3.75861454	4.84780033	3.75098181	4.98822173	3.82321744	4.48636535	6.43908787	4.10479915
TCAF1	1.63124583	2.51179368	1.51005175	2.39268617	2.07926292	1.9555584	2.06189423	2.1702716	2.20114834
TCAIM	2.33891517	2.17205531	2.32530097	2.39309592	2.50598785	2.09368021	2.32086369	1.98618535	2.3544008
TCAP	0.76726923	0.10430112	0.46283925	0.30519328	0.51112371	0.24321023	0.17403735	0.38655847	0.35778467
TCEA1	3.35768343	4.24536308	3.84801195	4.72906475	4.14046697	4.50585128	4.82426679	4.53812441	4.02426047
TCEA2	1.67760241	1.93601777	1.82326949	1.42682797	1.42155117	1.57870173	0.81727451	1.49088636	0.4807034
TCEA3	0.83460197	3.55894372	2.93862551	2.02043234	0.3549331	2.23574283	3.82638363	2.54300596	1.22483926
TCEAL2	0.04310116	2.85348416	0.23299989	1.13804689	0	2.06775428	0	0.01676489	0.05637743

TCEAL3	2.89399229	3.28089679	2.68518743	2.62175497	1.61316469	2.80317946	1.61042505	1.07208495	0.55249483
TCEAL4	4.3060257	4.42111192	3.94842837	4.5545354	3.95470463	4.04377284	3.31443191	3.8973192	3.09083861
TCEAL7	0.49343554	3.20163598	1.53965316	2.34537487	0.56168554	2.12151839	0.99224465	0.49938373	0.21156432
TCEAL8	2.64404084	5.35628086	5.1992636	5.63639306	5.36919151	4.51905608	3.27235934	5.2779159	3.61429985
TCEANC2	0.8291553	0.93532594	1.27097322	0.8921247	1.15415564	1.10881713	0.76540095	1.10923979	0.90720562
TCERG1	2.56124727	2.21770011	3.01531372	3.1287331	3.6607948	2.66560431	2.71026849	2.67227439	3.49408866
TCF12	2.99348521	4.01113692	4.2174932	4.14811321	3.54402637	3.96752193	3.28426975	2.00017456	4.15572843
TCF15	0.42347609	0.52341497	0.14486959	0.34812353	0.07044374	0.35675349	0.15923608	0.07909062	0.09533551
TCF19	3.74210944	2.23180849	3.59274418	2.77101983	3.08889519	3.10874853	3.66752866	3.0632772	3.02008276
TCF20	4.34728317	3.37921121	3.24337305	2.99885391	3.92379564	3.31679296	2.99274699	2.75582008	4.0510261
TCF21	0.31525959	3.52936997	1.2286236	1.54673273	0.58753015	2.05488141	1.48761543	0.84295	0.50307651
TCF25	3.10919555	3.59770756	3.67025035	3.34951139	3.44400931	3.79110942	3.84575288	3.89300401	4.3066768
TCF4	0.73495813	2.75474058	2.07524003	2.11619365	1.46134157	2.49301431	1.71351965	1.23389377	0.85537541
TCF7	0.90388195	2.44483704	2.56922588	1.56679187	0.70024564	1.45734819	1.66033693	3.06351809	2.80924375
TCF7L1	0.95925825	2.25792193	1.50586495	2.04784462	0.91990825	1.80397515	0.96271009	0.83435238	0.76376712
TCF7L2	3.64823284	3.79287292	4.57710829	3.87987308	3.73387417	3.64744838	3.83501858	3.12270233	4.19045783
TCFL5	1.66396462	2.30378892	2.42815994	2.87374772	1.78861217	2.19340726	2.04582972	1.75471055	2.65052048
TCHP	1.33057776	1.21020242	1.398784	1.30923151	2.30287732	1.42345725	1.25676328	1.50203763	1.68395832
TCIRG1	4.53760761	3.64263631	4.6500749	3.69076986	4.04083731	4.24376374	3.93651785	4.66086624	4.85218048
TCN1	1.45214529	3.7856722	3.37071653	2.85791424	5.00384422	2.52701106	2.49654716	4.94536075	0.42574406
TCN2	4.39080254	4.69761833	4.28252047	3.76887737	4.73910459	4.21955082	3.30215004	3.10696797	3.31943935
TCOF1	3.21983324	2.10172634	2.33595907	2.56358851	3.10994725	2.79175913	2.69866655	2.74090008	3.08444769
TCP1	4.68875171	4.71212714	5.90151274	5.79205868	6.23688748	5.63348908	5.82842878	5.37668857	5.65863633
TCP10L	0.04738887	0.01373195	0.0512853	0.09777244	0.09336718	0.12699901	0	0.08560664	0.03749302
TCP11L1	1.18874966	1.95323586	1.48957927	1.76150394	1.37995343	1.76880631	1.21455685	1.64885046	1.52693961
TCP11L2	0.36614426	1.08015039	1.23249784	1.26194579	1.07276291	0.99739496	0.66929858	1.32249729	0.70461189
TCTA	3.72583172	4.00112161	4.23562907	3.21691416	2.92706734	3.28858333	3.72893215	3.72938979	3.80766108
TCTEX1D2	1.81311736	1.0884592	1.00767589	1.381617	2.08231539	0.92702855	0.77908404	0.67959113	0.76837955
TCTN1	0.77187379	1.97014748	1.07961327	1.65495148	1.33422304	1.39783218	1.03458891	1.76999359	1.00860054
TCTN2	2.3097486	1.18062713	2.05772789	2.15640246	1.64917484	1.7791453	1.62386175	2.59995648	1.5078213
TCTN3	4.03162017	4.20772904	3.92832415	4.81732246	4.01276836	4.13296266	4.06168223	4.2601512	4.39529633
TDP1	1.91176213	1.9348321	2.33390866	2.14074534	2.47598749	2.21642552	2.08555335	2.27318202	2.26558064
TDP2	3.93728297	3.84417237	4.71327281	4.01968791	4.4078952	3.91995198	3.75297563	4.57112144	4.07951678
TDRD3	1.89719588	2.01241026	1.74947056	2.13632321	2.24935077	2.01738922	2.0780353	2.01254553	2.59346433
TDRD6	0.16728301	0.82230407	1.20921891	0.89397809	1.67566394	0.68781568	0.38709242	0.38021479	0.53401468
TDRD7	2.15573111	2.64972589	2.85427702	2.5661244	3.63872182	3.08244777	2.31207733	2.09508117	3.3430397
TDRD9	0.06501806	0.23500752	0.29842159	0.12965632	0.27179403	0.68227436	0.09251453	0.13684202	0.04135094
TDRP	0.20642034	1.19761334	1.16844375	1.02582441	0.28649425	1.02706099	0.40758166	1.7886762	0.20709124
TEAD1	2.46842823	3.75666935	3.11723967	3.06419837	2.43676036	3.54723029	2.49393965	2.46090386	2.55631799
TEAD2	4.50200976	3.40462294	3.181409	2.61627539	1.08950272	3.46936144	2.06932198	1.81384207	2.63847658
TEAD3	3.983707	4.28457148	4.01479505	3.74570157	3.14027498	3.9959902	4.23171985	3.69575985	3.74862319
TEAD4	4.47775648	2.66525952	3.29455284	2.89477143	3.36097841	3.87553231	3.35118628	3.47256918	3.10982842
TECPR1	1.71950205	1.51732769	1.78992875	1.30614271	1.54213206	1.74707683	1.41032054	1.97560783	1.8729194
TECPR2	1.52557561	2.2267222	1.26788586	1.78595301	1.52803567	2.08214479	1.30993753	2.2014021	1.54185429
TECR	4.9790671	4.19190032	4.92282029	4.49565764	3.94190875	4.08139119	4.35820071	4.86880363	4.88904095
TECTA	0.02813726	0.17598138	0.08953748	0.13549045	0.06279435	0.14866622	0.03915033	0.02712122	0.02956529
TEF	2.52682674	1.79640577	1.67218998	1.63926817	1.17051882	1.84660251	1.26202834	1.90551275	1.71047839
TEK	0.63903072	2.76233694	2.57083597	2.12719806	1.54786067	2.61146268	1.66229885	1.23609117	1.01809954
TELO2	3.44526797	2.29024945	1.98470512	2.27817909	2.45388425	2.45703666	2.95407444	2.44903121	3.41250753
TENM1	0.01126021	0.24914617	0.29779614	0.1249813	0.0762909	0.17667054	0.00675098	0.01732314	0.00444959
TENM3	0.32290481	1.23630792	0.39365884	1.66629457	0.14259287	1.27942512	1.77567537	0.3137355	0.12839053
TEP1	1.76248353	1.92453028	2.22046581	2.43020576	2.73879692	1.77312564	1.72481732	2.2936562	2.20376803
TERF1	1.7199193	2.28979859	1.82287431	2.42386227	2.14017869	2.41932359	2.3876228	2.95556498	1.95584803
TERF2	2.79333468	2.70298561	2.66842106	3.08673621	2.75200877	2.88886738	2.60451152	2.99679799	3.19748243
TERF2IP	3.68365262	4.06832601	4.04120985	3.9601272	3.75287383	4.00920232	3.97608362	3.86935634	3.51864781
TES	4.74155389	4.76882861	4.29499927	4.83916007	4.27651586	5.3611465	5.05146446	4.34630435	4.49661947
TESC	5.88743751	3.29243316	5.55068433	5.2533169	4.19818785	6.1696533	3.96235649	8.69268348	0.47817184
TESK1	4.05069236	3.37396229	3.62254669	3.27719035	3.28028272	3.62120192	3.28245764	3.49564083	3.27028032
TESK2	0.94690551	1.5421024	3.05430118	2.00235922	1.60080547	1.93536656	1.91539894	1.66262355	1.71649231

TET1	0.21470873	0.53007573	0.44733522	0.63975691	0.65968084	0.59803454	0.23568193	0.26958584	0.08217834
TEX2	2.72734508	2.60532961	2.73365725	2.48063718	2.7690558	2.80047146	2.26340684	2.53623564	2.96479884
TEX261	4.56609123	4.39904802	4.78886118	4.38212168	4.18607015	4.34726656	4.4014955	4.15443804	4.64265673
TEX264	3.83450467	2.83047239	3.37601375	2.65712947	2.39354404	2.92147265	3.51368077	3.4701647	3.42421864
TEX30	0.50812588	2.09020176	2.19661934	2.82359704	2.65273572	2.02127985	2.16642935	1.89923798	2.23605642
TF	0.04193561	0.08747448	0.12929216	0.19100842	0.01304746	0.0431248	0.00254488	0.05470947	0.01114008
TFAM	2.82626864	2.91665654	3.33989551	3.4732343	4.10835633	2.86623067	2.61283193	3.12604654	3.72746084
TFAP2A	2.40394973	0.25889133	1.27565837	2.37756752	2.51569049	2.96191819	2.90757769	3.26822581	0.67871482
TFAP2C	0.21378238	0.25412335	0.86867542	1.50378521	0.16235515	1.04441803	1.54462144	0.94609911	1.51170985
TFAP2E	0.24445461	0.13639367	1.60533827	0.91020009	0.3769479	1.42403371	0.96592597	0.16361758	0.1256381
TFAP4	1.45777201	1.11756484	0.99151848	1.34770372	1.48345948	1.28162999	1.35117706	1.29525603	2.36234111
TFB1M	2.19901908	1.84881477	2.87125535	2.42806168	3.00750413	2.6873927	3.08748167	1.7497145	2.4437992
TFB2M	2.57913039	3.15261611	3.86389807	3.73355809	3.57354544	3.4881233	4.29815614	4.36488434	3.98856139
TFCP2	3.21066185	3.03943865	3.51771187	3.70127266	3.32823204	3.34489436	3.23517177	3.85078904	3.43459886
TFCP2L1	0.12293735	0.07803887	3.14342091	0.66338156	0.3266044	0.72651156	1.08281321	1.94160695	0.17133183
TFDP1	5.73823899	4.33215913	4.8917034	4.78048595	5.20474468	4.85583224	5.59520749	4.17140513	5.84049857
TFE3	4.44096262	4.10685581	3.87896863	3.69210947	3.89283459	4.18881151	3.32899164	3.73218667	3.24524707
TFF1	4.13346357	2.49107342	7.49658685	7.91662119	5.6912359	5.84715344	11.6920001	9.97914822	3.43406185
TFF2	0.27863982	1.15078846	7.03653821	4.60612926	2.79570035	6.00863373	10.6276622	5.61198767	0.74150931
TFF3	1.15885835	6.92544763	3.988732	5.19452024	1.3479901	4.09256723	9.41961243	3.75173745	8.52516724
TFG	6.26636282	4.75110602	5.27371504	5.24136723	5.26411257	5.07355343	5.19949571	5.14650964	5.47864345
TFIP11	3.23439096	2.43155244	2.65410676	2.48550543	2.82504585	2.68053315	2.2837651	2.3155699	2.654382
TFPI	0.54957686	3.07998507	2.47638083	2.68747401	1.5038669	2.38911112	2.49690293	1.01068187	1.28464326
TFPT	3.85866465	2.86355488	4.03002029	2.49685969	2.89982932	3.04495763	2.54330407	2.02506324	2.59030763
TFR2	0.17590801	0.48538297	0.91564183	1.09563255	0.10264535	0.76088204	1.98828027	0.42869037	0.27468449
TGDS	2.57923746	2.80442652	3.15074127	3.68088787	3.61429319	2.73103232	3.36436847	3.24378866	3.85277291
TGFA	3.38993705	1.8725923	2.81143109	3.20679841	2.89846732	2.64807707	3.59510491	2.14128722	2.44820863
TGFB1I1	2.80128546	4.30862879	2.7445587	3.51147914	1.53941457	4.15957625	2.94386225	2.09019992	1.83916917
TGFBI	3.76914714	5.82741532	5.68515945	5.16721118	4.18066408	5.88184656	5.40157443	4.32670487	4.19747686
TGFBR1	2.9377948	3.83716163	3.9968687	3.90709921	3.14816791	4.39313239	3.51488574	2.42843011	2.90194529
TGFBR2	5.20954114	6.74281756	5.48502634	5.36997434	5.81230888	5.74231233	4.44809863	6.09730195	3.52932357
TGFBR3	0.70719903	3.90091208	1.62185285	2.29328575	1.10521369	2.73521961	1.46543192	3.02331052	2.0615452
TGFBRAP1	2.7093637	2.9182031	3.3865511	2.96241791	3.19179904	3.37754279	3.21488504	3.59480083	3.38646271
TGIF1	3.20746359	3.25091166	3.72582019	3.82970421	3.40317016	3.44884188	3.44800639	4.4782014	4.02369398
TGIF2	3.14364532	2.09277145	2.94291795	2.54655192	3.0043985	2.79817774	2.22913877	1.72146955	3.36838188
TGM1	1.47094835	0.10766467	0.2217373	1.07294536	0.56070944	0.26756648	0.17057765	0.25762551	0.32880323
TGM2	6.2311678	5.84991753	5.03422824	4.71992424	6.72627379	5.71030368	4.96201037	4.59770576	3.62026961
TGM3	2.77089222	0.6601985	0.25066797	2.65826415	0.04917463	0.35909641	0.31203186	0.21297914	0.70587662
TGOLN2	6.27968157	5.43663739	6.22817426	5.17946907	5.38494669	5.27654673	5.72199511	5.71316574	5.23160897
TH	0	0.17554967	0.06353279	0.0200865	0	0.00823468	0.03980863	0.12441837	0.12984185
THADA	1.76705908	1.85933001	1.88566885	1.7469666	2.18732409	1.96999761	1.46464638	2.25044424	1.99097522
THAP1	2.10908981	2.54522953	2.29472813	3.12323374	2.50365988	2.79994391	2.98476955	2.53825628	2.67657861
THAP11	4.37108137	3.63027592	3.72232914	3.45958674	3.22124486	3.9548054	4.07908039	3.88288823	3.88014337
THAP5	1.43335953	2.3341636	2.59454542	3.14390938	2.0507472	2.24431988	1.98984205	2.67564667	2.68799183
THAP6	1.10802943	1.48168518	1.39010093	1.57933622	2.01963672	1.67379375	1.39596044	1.81801786	1.65281138
THAP7	3.99722554	2.54225102	2.93184054	2.73076238	2.45873411	2.8039786	2.13178058	2.63728309	2.97083154
THAP8	2.4949489	2.29771406	2.18907324	1.74424929	1.24664806	1.95364592	1.48441968	1.63319349	1.72528618
THAP9	0.44524806	0.97231554	0.87512773	1.11904362	0.9025467	0.89798896	0.95011246	1.19682041	1.29989299
THBD	1.70049811	3.7207631	3.86396642	3.61859377	2.56092536	4.32790307	3.13480545	3.88128523	2.18633875
THBS1	4.38367005	7.30760783	5.79526567	6.69618602	5.4278113	7.43550568	6.44020548	5.08209804	3.89307314
THBS2	4.25834821	5.89425315	3.8408876	6.23555342	3.29052288	5.99601026	6.47773431	4.22246626	2.80712493
THBS3	1.35772435	3.39739821	1.8036636	3.0781287	1.36857386	2.72236889	1.94204365	2.07802558	1.74023642
THBS4	0.20614314	5.54166771	2.18116436	3.50346428	0.11701139	4.85207232	0.36989255	1.12278355	0.51274005
THEM4	2.29997324	2.31758992	2.37820477	3.32893233	1.78641154	1.83631838	2.35801265	1.74264752	1.73902921
THEM6	5.06809856	3.20737845	5.62421403	3.72020213	2.48802984	4.05004432	5.23794011	4.51293097	4.23656891
THG1L	2.51673224	2.2390401	2.21727035	2.88685229	2.81484538	2.56781742	1.97469038	2.58161873	2.78596335
THNSL2	0.22516268	0.50208003	0.78785579	1.17082609	0.26696136	1.12758867	0.09198958	0.87276728	0.37094154
THOC1	1.13981855	1.38061677	1.95729788	2.13162724	2.59321386	1.65916136	1.92302714	1.54910919	2.11282053
THOC2	2.35260524	2.65849607	4.71372175	3.44468549	4.28327421	2.86529851	2.78329343	2.98735509	3.48469913

THOC3	3.03412888	1.45809305	1.43680066	2.09422101	1.60697809	1.80618786	2.69469269	2.20804103	2.59213629
THOC5	2.69887046	1.9991234	2.29587586	2.39857508	2.32540564	2.19364843	2.0690031	1.69354074	2.18630508
THOC6	3.87678449	3.46815138	3.13922199	3.43437503	3.60522481	3.39728674	3.52914888	3.23048601	4.34671797
THOP1	3.10686238	1.47591257	2.03406303	1.78440528	2.66107627	2.27797824	2.38071084	2.16063799	3.17678312
THRA	5.03400415	3.29517295	3.49106499	2.36791384	2.5688219	2.68087638	2.60056291	2.903434	3.66767974
THRAP3	4.77655296	4.35187357	4.54039565	5.10272415	5.22415157	4.81030639	4.73687214	4.26304837	5.16358089
THRSP	0	0.27444644	0.18892144	0.34608024	0.09258501	0.04491765	0	0.02373321	0.01622598
THSD1	0.44591231	1.64649513	1.34635637	1.27045665	0.60904704	1.22420001	0.57318109	0.59351647	0.44877527
THSD4	0.3057674	1.73625914	0.65222756	0.78613167	0.17218353	1.24397004	1.38766343	0.6574427	0.25135877
THUMPD1	3.10688909	4.11364253	3.40870317	3.9029799	4.09877577	3.81356655	3.71754646	3.76337434	3.58080315
THUMPD2	1.16496097	1.12492788	2.17580176	2.11703926	2.48435185	1.71466037	1.84432219	1.83991767	2.04352435
THUMPD3	2.30758255	2.87382711	2.68618023	3.52856237	3.46382643	3.18889701	3.33122273	3.27313629	3.41179095
THY1	4.57634452	7.05408525	5.01793702	5.47724713	3.85462681	5.9412195	5.06684891	4.05307256	3.15922275
THYN1	4.18230156	4.06816855	3.31201601	3.70933328	3.83277853	3.57689468	3.89323509	2.85410367	3.80570294
TIAL1	3.35360398	2.99997216	3.91522093	3.91596934	4.02060191	3.42150382	3.18400561	3.35277629	3.87393498
TIAM1	0.63353752	1.31027833	1.19594899	1.58096192	1.43964879	0.74470598	1.19695413	1.09864082	0.53519829
TIAM2	0.40512676	0.6330494	1.40030958	0.79465703	0.70027434	0.90556738	0.66671903	0.72992022	0.8900365
TICAM2	0.03331607	0.16978936	0.07896456	0.07230041	0.16341053	0.26337574	0.10377766	0.08001031	0.04075603
TICRR	1.49210912	0.41027531	1.08906442	1.45074728	2.03779178	1.3296665	1.33183186	1.1723842	1.71669905
TIE1	1.67642241	2.79496345	2.26731348	2.14217465	1.32030052	2.9761969	1.62846735	1.20638587	0.92342012
TIFA	2.08185107	1.83338555	2.56798821	2.3538061	3.01597782	2.7739639	1.74497354	2.54160568	2.18265785
TIGD1	0.62191519	0.6179537	1.65141146	2.16955661	1.67247824	1.05550711	1.13633831	0.95210352	1.69414308
TIGD2	1.02611976	1.99191255	1.90528232	2.60710581	2.70264425	2.01985865	2.1011178	2.30201822	2.61214019
TIGD5	1.59006394	1.19953251	1.34326699	1.34054095	0.60653084	1.50981536	1.46418119	1.93347965	1.73787672
TIGD7	0.07610885	0.34751403	0.63436932	0.29572445	0.24085176	0.30641642	0.16208006	0.45785091	0.22364717
TIGIT	0.79072182	0.69178878	1.12865383	0.63910933	1.82047477	1.29409005	0.99498487	0.30448445	0.89616156
TIMELESS	3.2929617	2.10111885	3.06715479	2.88100482	3.47078204	2.75105289	3.00318606	3.17756981	3.04535107
TIMM10	5.50290945	4.48166638	4.95286692	4.28764938	5.11887434	4.77860577	4.88890403	4.6927033	4.90775009
TIMM10B	3.22346469	3.24332095	3.53290137	3.85711566	3.33612821	3.41085462	3.68032196	3.66918822	3.89328864
TIMM17A	3.57915362	3.80941617	4.17064558	4.58776423	5.00262069	4.39255168	5.05824785	4.71812367	4.2698214
TIMM17B	4.96035604	3.73714454	3.73593494	3.2712175	4.3617118	3.70192113	3.00534544	4.12779031	4.02815934
TIMM21	1.37350015	2.11941905	1.70655624	2.6091506	3.21089729	2.55223518	2.97793338	1.70166023	2.79029303
TIMM22	2.71371999	2.57767707	2.941052	3.07405464	2.98616013	3.11971374	2.75741137	2.05061641	2.86427916
TIMM23	5.4802497	5.42310213	5.4822021	5.57165154	6.00173568	5.51267206	5.33763944	5.41030619	5.7096198
TIMM44	2.98457686	1.969338	2.57040703	2.38575361	3.10000408	2.75930485	2.64883321	2.35480461	2.95532418
TIMM50	3.46289856	2.34918694	2.9576168	2.86583211	3.1331157	2.93837872	2.68303621	2.7983616	3.23651572
TIMM8A	2.02333697	1.50503718	2.35715089	2.51638099	3.1416868	1.86706146	1.78913358	2.15192645	2.10541315
TIMM8B	4.79588603	4.45926581	4.12520627	4.61015532	4.9076523	4.5071367	4.74948208	3.53353132	4.88341297
TIMM9	3.98062204	4.35317394	3.99027611	4.29362524	4.4038649	4.00994341	3.7365313	4.21749102	4.6273383
TIMMDC1	4.5599504	4.67710848	4.80915778	4.58331394	5.31170917	4.72825026	4.7856692	4.64336058	4.43059638
TIMP1	8.30830645	10.4833973	8.57057409	9.21345529	7.25784191	9.26152335	8.76964467	8.23480739	7.23305665
TIMP2	4.64239221	6.83399805	6.31870558	7.2809947	5.08818754	6.84977107	6.14556415	6.20153546	4.41219726
TIMP3	2.58751742	2.14821405	1.75488821	2.26194806	1.25469798	3.12249008	3.32308786	2.30593847	0.97131095
TIMP4	0.02192048	2.24362711	0.95619379	1.32032329	0.15468751	1.539518	2.20791501	0.11451578	1.90156224
TINAG	0.35765002	0.86328592	0.21525676	0.51501331	0.65122198	0.01237435	0.03993616	2.68598463	0.80877994
TINAGL1	5.14794288	3.72886924	4.45930596	4.67730462	4.04185299	4.89099375	3.89493608	4.15286241	4.84631378
TINCR	0.05466325	0.16563243	0.2347393	0.4630105	0.08334793	0.06013857	0.1019205	0.0213143	0.02899107
TIPARP	2.68698847	2.69294909	3.82670621	3.01213167	3.10473105	3.45178103	3.47691727	3.67264275	2.96439266
TIPIN	1.61500053	1.56258627	2.04270164	2.45943523	2.99161078	2.28123244	1.91947477	1.13622457	2.45860681
TIPRL	3.86748413	3.75073305	4.07978734	4.0789163	4.54432292	3.9703875	4.48335035	4.17615959	3.86709629
TJAP1	2.39427557	1.45575359	2.66671992	2.32639257	2.18972323	2.05467277	1.38069115	2.28503201	2.0232648
TJP1	2.47540604	3.21470588	3.23270928	3.89064534	3.80304462	3.89379974	3.01645155	4.24628341	3.07747915
TJP2	4.8600534	3.90255082	4.2858045	3.81657559	4.83554505	3.20828657	3.98477582	4.99615848	4.81447819
TJP3	5.63263153	3.06708378	4.08416584	2.83998429	4.22716853	2.93075452	3.16246235	4.63947406	4.69000141
TK1	7.01447199	4.0481389	5.23714414	4.49237911	4.81562057	5.03717079	5.4007342	5.24392094	5.28441141
TK2	2.10610444	2.3232259	1.92516519	2.1709018	1.95548134	2.09437842	2.41254707	2.19449394	1.37456727
TKT	5.59253084	4.42943744	4.12149637	4.59893971	3.70826877	5.6249078	5.38104736	5.17441334	5.46328763
TKTL1	0.02964803	0.34884469	0.15704892	0.70221256	0.01225013	0.07853425	0.00596469	0.04918114	0.15416359
TLCD1	2.76143732	2.1801266	3.77275708	2.39031857	1.2634714	3.583517	1.99963461	5.01262011	2.84569679

TLDC2	0.7843607	2.04566126	1.24643908	2.66917875	1.45915099	0.812464	0.4512619	0.51312092	3.06073974
TLE1	4.52209129	2.60730911	4.155297	3.34697178	3.6827355	3.14066099	3.82985259	3.78935022	2.75057165
TLE2	2.53252618	3.20370558	1.38350864	2.17051781	2.88102869	2.31781497	1.10663125	0.75668109	3.17160814
TLE4	1.7015404	2.55187207	2.19749361	1.69268063	1.25442755	2.48850354	1.65194967	1.84118643	1.84079172
TLE6	1.08916486	0.29377584	0.44341086	0.98506857	0.79322657	0.44725253	0.5040713	0.91465981	0.41630757
TLK1	2.66259003	2.64408886	3.42901921	3.1656827	3.17078515	2.89060087	2.93122004	2.99537001	2.65149089
TLK2	2.18741077	2.01374381	2.47425393	2.15711293	2.22377126	2.36407285	1.95877792	2.55751933	2.17831731
TLN1	5.46007547	6.15324507	4.8706729	5.10092065	5.27660323	6.27896185	5.17788559	5.30328719	4.91801414
TLN2	1.44843741	1.37775459	1.41590083	1.51110871	1.34520964	2.20251632	2.43786292	1.22841396	1.91836523
TLR1	1.03367566	2.98562049	2.85394895	2.23047862	3.41876688	2.99920264	2.60131438	2.07076247	1.39073059
TLR2	1.66233792	3.1295676	3.34607374	3.66005987	3.73353219	3.70850501	2.97431334	2.65891847	2.42896408
TLR3	2.15709214	2.58524933	1.41644294	1.51009211	2.65218542	2.57290715	0.60999127	1.12249125	1.65741029
TLR4	1.50385387	3.85888452	3.20298506	2.9472722	3.67796524	4.26989396	2.9336197	2.48960828	1.77941761
TLR5	0.87873957	1.89992779	1.84885499	1.94052193	1.60239742	1.92822639	1.19100846	1.40609924	0.55269404
TLR6	0.48732528	1.26317535	1.19044553	0.95735828	1.3223839	1.14731122	0.66112859	0.57574647	0.37276636
TLR9	0.0093867	0.01074831	0.06648089	0.0168511	0.00578983	0.01034323	0	0.00362506	0.01477434
TM2D1	2.88442726	1.92960502	2.68174098	2.44292193	2.60568678	2.01697961	1.83652052	1.88294382	2.41366738
TM2D2	3.21894302	3.50320624	3.23247868	3.79479205	3.67728464	3.86717159	3.94067242	3.45721455	2.92581864
TM2D3	2.00802658	1.58732087	2.04944462	1.77391125	1.74637775	1.66528903	1.34462593	1.70493434	1.73742674
TM4SF1	5.69772473	4.98727207	6.91145758	7.60649123	4.93593009	6.9379495	7.24219996	7.787088	2.77153675
TM4SF20	0.03912193	4.51704891	0.83279281	0.54771522	1.21815749	0.37809857	2.17323091	0.1360368	4.54668743
TM4SF4	0.33308014	3.33258018	1.19931556	2.04872999	0.13032129	0.30359009	1.94134678	7.36384214	1.5198824
TM4SF5	1.97354666	3.5423303	2.74382278	3.32790448	2.03177847	1.6739934	0.50777254	3.75427943	4.79697158
TM6SF2	0.17248924	3.20274662	0.91393045	1.00316399	0.21858186	0.54832778	0.28825225	0.28982825	2.25909803
TM7SF2	1.85758972	2.05326001	1.35824345	2.66876028	0.74093778	1.24649945	0.48016849	2.73356688	2.54768767
TM7SF3	4.40866201	4.60870266	5.35633819	4.16646227	4.98515113	4.64904086	5.46353017	5.23396827	4.12808058
TM9SF1	2.00277025	1.70618405	2.12092766	1.44294674	1.35003877	1.73592601	1.49223744	2.20212794	1.79376572
TM9SF2	6.30430089	6.35199917	6.84454115	6.16923493	6.37743231	6.38603274	7.64562943	6.79925687	7.22726758
TM9SF3	5.45896839	5.53313826	5.23833673	5.92084961	5.82437617	5.43632446	5.9414153	6.44002435	5.97959276
TM9SF4	4.46508796	4.48138986	5.03040785	4.80710554	4.36377993	4.35243055	5.16710011	4.8421931	5.15807676
TMA16	2.80826624	2.41832423	2.54129663	3.08714775	3.16001981	2.39842996	2.3141401	2.70506647	2.90786475
TMA7	6.18484789	5.51989832	5.41429742	5.85444367	5.95528734	5.44392881	5.83422946	5.19927632	5.69081239
TMBIM1	5.91311685	5.39698664	6.32455442	6.1173747	5.92645888	6.22546749	6.34889244	7.16700712	5.62223712
TMBIM4	3.27729407	3.28506032	3.95221723	2.97753568	3.28870936	3.45703347	2.81826005	3.59457361	2.89566197
TMBIM6	7.44122855	7.4307518	7.63198616	7.32093271	7.39449167	7.56953729	7.67745065	8.37069826	7.38546967
TMC4	4.80194692	4.03320139	4.54433014	4.21999734	3.34316955	3.06401024	4.30173487	4.84818038	4.88072181
TMC5	5.33091375	5.43759664	6.03928101	3.94992574	5.30664059	2.9088384	3.98208003	6.21663429	5.4395753
TMC6	3.95014186	1.87603363	3.55323927	2.12497623	2.24905001	2.67044409	2.62370102	3.53368288	2.91230489
TMC7	0.70246826	1.86430365	1.48366236	2.06354008	2.01499812	1.23161909	2.06384775	2.30071355	0.74699335
TMC8	2.03088938	1.67330109	2.51870042	1.52585168	2.58707465	2.66653489	1.4742467	1.28964036	1.52741144
TMCO1	3.97128912	4.281709	4.7515639	3.91389097	4.51621138	4.08593977	3.59180089	5.3320633	4.54125545
TMCO3	3.23989832	1.98960554	2.87673221	2.52369075	1.78572657	2.71779603	3.77376404	3.64470817	2.82370017
TMCO4	3.29898466	2.26556564	2.45163869	2.53890083	2.66703495	2.43735873	2.05378296	2.38463992	3.53391703
TMCO6	1.27564007	1.22827394	1.92940972	2.05333027	1.68193797	1.6453687	0.94415601	2.10717735	2.31870962
TMED1	3.45300149	2.1638817	2.61792058	2.29179882	2.10146009	2.66383377	2.67063531	2.90803053	2.7657714
TMED10	6.15408443	6.43605975	6.47112367	6.67151743	6.25696425	6.40631917	6.9577913	6.64921367	6.2884864
TMED2	6.12897281	5.87932454	6.81976308	6.66285806	6.49005733	6.63000696	6.71105473	6.81847436	6.42492189
TMED3	2.52909411	1.96289579	3.56648302	2.20876228	1.98367825	2.6141983	3.39437914	2.42884367	2.32661949
TMED4	5.27930833	4.32939527	5.18709978	4.89099029	4.18074269	4.06236229	5.39590379	4.67708311	4.94609539
TMED5	2.84654977	3.63390308	4.14245517	3.78436842	4.32792914	4.16496301	3.93955557	3.76285089	3.80570363
TMED7	4.44357384	4.88959533	5.40484318	5.25685629	5.3640353	5.29294026	5.2759862	4.49380926	5.22975699
TMED8	1.72854186	2.51971486	2.47090191	2.40839763	2.81594282	2.74692389	2.24182316	2.73551195	2.30018492
TMED9	7.05923862	4.92836	5.97950065	5.2905071	4.97492218	5.6048948	6.19695331	5.71728542	5.19876055
TMEFF1	0.02764982	0.01590256	0.01004922	0.03311434	0	0.0942932	0.00833138	0.01604974	0.01459981
TMEM100	0.02486702	3.09821391	1.07405191	0.6397382	0.12570268	1.0869766	0.14283564	0.22758372	0.10179719
TMEM101	3.97682551	2.36092151	3.23439725	2.53939864	2.45014889	2.5976532	2.7205909	3.07030557	2.90204605
TMEM102	2.82759238	2.1678187	2.39048549	2.26986884	2.07846304	3.15940205	2.71714034	2.50199365	3.38184462
TMEM104	3.30034175	2.45281527	3.12868237	2.21434377	2.41290829	2.59331116	2.46034065	3.59042167	2.29783621
TMEM106C	4.49346601	4.48454339	5.36158034	4.47876382	4.53823257	4.24933734	4.65953835	5.26035771	5.09197271

TMEM109	5.75395477	5.50448827	5.39056367	5.25329699	5.34512056	5.70250341	5.55198054	5.58691939	5.00861164
TMEM11	4.21402902	2.76334883	3.06499441	2.75253515	2.96184009	3.13070319	3.00923309	3.10634225	3.23310488
TMEM115	4.76150304	3.65364842	3.92884551	3.48667824	3.31930007	4.15360461	3.90621849	4.27767568	4.1466102
TMEM116	0.63245342	0.77399701	1.9998234	0.74936959	0.9066117	0.65863036	0.93105166	0.79788082	0.87528708
TMEM119	2.94664221	4.68252407	2.84300179	4.13639114	1.63152311	3.29685163	3.31370473	2.56450207	1.52083257
TMEM120A	4.46349019	4.14462625	3.80502614	3.1365611	2.58755715	3.72658652	4.41951765	4.273637	3.7204292
TMEM120B	1.38160453	0.73805015	1.34981125	1.15856232	1.31727442	1.1594241	1.07578671	1.4705066	1.33057456
TMEM123	5.10568792	6.23663946	5.82133397	6.56689966	6.13865237	6.32576931	6.56125055	5.49651832	6.55841342
TMEM125	3.14382055	2.29422288	3.308378	2.43200437	1.89314565	2.44190471	2.51797836	4.26682726	3.84645592
TMEM126A	3.93629927	4.49685639	4.49320494	4.89183415	5.15065618	4.65170167	4.31175321	4.70586593	4.42894396
TMEM126B	3.34177996	4.08161674	4.02452662	4.0134302	4.44278473	4.06255531	4.2720366	3.48644077	3.89613256
TMEM127	4.47215829	4.33525554	4.55080902	4.20987089	4.37277665	4.42925395	4.39542291	4.67125464	4.03759417
TMEM128	3.57203305	3.06132772	3.94237938	3.74871173	3.62556063	3.40412818	3.3408881	3.4016593	3.26992051
TMEM132A	3.39357985	1.17360205	2.32439707	2.56744816	1.57728758	2.05284254	2.38456915	2.23343573	2.62143384
TMEM132E	0.06985123	0.71451471	0.1158685	0.07890866	0.06700431	0.14709612	0.07422142	0.35901765	0.04120566
TMEM134	3.24758557	1.72724878	2.07667273	2.03729445	1.60612794	2.0571551	1.95877291	3.00681532	2.19283595
TMEM135	1.78274181	2.68122037	2.28650099	2.53226945	2.69376896	2.73540446	2.84659958	3.2295067	2.83705426
TMEM136	0.13982386	0.90892811	0.79853895	0.9303145	0.25707267	0.76030157	0.30674398	0.76140755	0.20359894
TMEM138	2.35354919	2.21065987	3.18524201	2.9695749	3.15401323	2.36158522	2.35107494	2.23531501	2.7522563
TMEM139	4.72889391	2.81642966	2.83793945	2.94211841	2.17449174	1.32534505	1.30709972	2.16907404	3.13484013
TMEM140	4.29836372	3.94554496	3.6037288	3.35230711	3.95725543	4.21289579	3.44341305	3.2036025	2.87535876
TMEM141	4.80347659	3.69297337	4.36573388	4.41294497	2.87181042	4.12917664	4.27734485	4.72376973	5.006693
TMEM143	2.70982572	1.50862629	1.25440508	1.21326501	2.03675379	1.21359882	1.56020692	1.73017567	1.56683179
TMEM144	1.44891699	1.78725204	2.7820299	1.77547342	2.53610028	1.90591479	1.70603451	2.87521387	2.76970169
TMEM145	0.05418908	0.10666223	0.42830969	0.67541054	0.04190751	0.76224655	0.21460093	0.0927275	0.18371241
TMEM147	5.56377081	4.52946442	4.70855057	4.83346086	5.30124162	5.09681422	4.96440556	5.15161409	4.99271554
TMEM14A	4.8161712	4.17056238	4.83126656	5.03333373	4.65121612	4.15794962	4.43969186	5.13004144	3.98835598
TMEM14B	3.6785839	3.45958933	3.78184228	3.54790634	3.55337259	3.47761818	4.30004447	4.16990268	3.49156415
TMEM150A	2.51787726	2.25099593	2.45030882	2.28386358	1.64738397	2.27676113	2.13805274	2.10151911	2.54516239
TMEM154	0.35754408	1.57741633	1.10104146	1.72223952	1.05180654	1.95953875	0.94898429	1.25233513	0.81446052
TMEM156	0.57822176	1.1320532	1.82211754	0.87041762	1.95802037	0.6396195	0.4055885	1.02139544	0.44107723
TMEM158	3.15612443	2.16343329	2.65189759	2.75160731	1.57668817	3.03618611	3.25916871	1.96544704	1.82135127
TMEM159	4.24366308	3.1606031	3.74110251	3.53379822	2.84053806	3.1694821	3.76464979	3.26620791	2.79677436
TMEM160	5.63390894	3.9016964	3.99275287	3.5023992	3.36717689	4.15035763	3.90290314	4.14065366	3.89134921
TMEM161A	3.53726744	2.79229692	3.14546882	3.06165558	3.49488782	3.07822597	2.88786997	3.60592184	3.57421681
TMEM163	2.10081584	0.44071966	1.14911768	0.69134671	0.14730647	0.26694784	0.23063852	0.56499014	0.25248118
TMEM164	4.13535497	3.09858022	2.96308538	3.8984679	3.67740386	4.00512182	3.94038165	3.80832681	3.5089326
TMEM165	4.03885083	3.50901622	4.51569105	4.12114121	3.87689046	3.5475441	3.93887017	3.09941904	3.63219162
TMEM167A	3.72455177	4.53514434	4.70619482	4.90864402	4.86025657	4.64584789	4.34601355	4.315374	4.39836363
TMEM167B	3.33889502	3.63829417	3.84265979	3.77057542	3.58370762	3.59402008	3.61554079	3.32497599	3.22214318
TMEM168	0.99889021	2.54093898	3.89189727	3.08991369	1.83567669	2.51757664	3.26987559	2.85672643	2.86354493
TMEM17	0.02423854	0.94257684	1.29667691	0.85042313	0.39300432	0.65895719	0.63481852	0.94411498	0.55778322
TMEM170A	1.51770239	2.26748669	2.45179082	2.46555195	2.65046008	2.4058236	2.73401381	2.45424637	3.38659415
TMEM171	4.00967522	2.28799666	4.05304554	2.46779175	3.22870313	2.78186774	3.47729324	4.22177397	5.42358706
TMEM173	2.83888609	3.50852194	4.00031079	3.52934051	3.67019992	3.87900506	3.89269741	3.0515282	3.39020571
TMEM175	3.35497676	2.46495349	2.23652077	2.17972215	2.19879998	2.23168073	1.89744037	2.8982241	2.39774563
TMEM176A	4.75733974	7.07005871	5.48891824	4.41784448	4.91845336	5.6987407	4.50809421	3.52571589	4.76726142
TMEM176B	5.98454849	7.96215412	6.92980113	5.81587571	6.30823128	6.92940777	6.19047706	4.66661471	5.68397672
TMEM177	2.55355419	2.34843632	2.86878743	2.30316241	2.22283182	2.52856564	1.88732619	2.39574375	3.3262785
TMEM178A	0.18009239	0.62743658	0.24454986	0.52170994	0.20315451	0.67919468	0.11863973	0.11473861	0.49423044
TMEM18	1.91823006	2.38877326	2.60065073	2.59573288	2.35756961	2.39151271	2.82800007	2.85765069	2.69854408
TMEM182	0.65509389	0.96495704	1.82675538	1.38010883	1.19648702	1.06354842	1.63866184	1.24060254	1.24755607
TMEM183A	3.76946879	3.11480736	3.87322065	2.852545	3.04872152	3.42251164	4.72052667	3.7909347	4.05458051
TMEM184A	2.7751166	0.97654631	2.32555	1.01118966	0.9833175	0.45275668	1.28430812	2.16883788	2.42050351
TMEM184B	5.05505332	4.21103418	4.59155694	4.1941712	3.55971413	4.4207738	4.40048276	3.80461238	4.47280063
TMEM185A	2.10630382	1.57965253	1.9692235	1.53367491	1.52741276	1.53079816	1.25855953	1.33747625	1.11172043
TMEM185B	4.11070474	3.53429844	4.54606278	4.01144785	4.00984342	4.17617989	3.5873965	4.16081777	4.03097412
TMEM186	1.80866671	2.74703035	2.31252872	2.03135406	2.44708178	2.17462033	2.29921136	2.15717254	2.49193554
TMEM187	2.9218222	2.89048261	2.30905096	2.24301019	2.08800099	2.56928215	2.1841745	1.79473433	3.41068076

TMEM189	2.73967304	2.01355023	3.12097227	2.59390843	1.58093981	1.95958319	2.32198517	1.73713868	2.48435057
TMEM19	3.06801253	2.7127197	3.25274562	2.4219532	2.5448907	2.39082973	2.2373169	2.66195285	2.39703948
TMEM192	0.74066769	1.67102191	1.74135096	1.53616433	1.44934193	1.44083382	1.60788093	1.79769257	1.87668247
TMEM198	0.38268696	1.01144065	0.58749547	0.84290424	0.32600407	1.12493576	0.26235418	0.24954937	0.69428627
TMEM199	2.41133004	1.44830358	2.24278089	1.82204778	1.9894978	1.86865632	1.5626956	1.68194233	1.73343
TMEM200A	0.68265946	2.17186833	1.43986192	3.53814028	2.64072832	1.80487256	1.82893446	1.12875816	3.90095577
TMEM200B	0.76450719	1.71893537	1.29820151	1.81841821	0.42441575	1.65326867	0.99786524	1.22605106	0.29445762
TMEM201	2.46807224	1.26181156	1.37020645	1.78652789	1.70425819	2.22162703	1.88601848	1.28831434	2.50435869
TMEM203	4.30419362	3.36885432	4.2903598	3.66568756	3.60460357	3.84450765	3.63250816	3.75291587	3.92919107
TMEM204	2.92410267	3.17701286	3.57337708	2.88694513	2.09135186	3.58062295	2.70449965	2.36789046	1.72369719
TMEM205	6.14583891	5.15664214	5.66376931	4.6909142	4.64287598	4.8774613	4.50184662	4.92153672	4.97086764
TMEM206	2.68605634	1.68912418	2.57994162	2.12429187	2.48633854	1.74856763	2.5565997	1.91962682	2.39522812
TMEM208	4.35514346	3.63238564	4.1177975	3.54988631	3.64633695	3.89515196	4.20281349	4.2509463	4.00021642
TMEM209	2.49145345	2.79581658	2.64829367	2.83979438	3.05006112	2.74644903	2.86369638	2.62478102	3.13589624
TMEM216	3.61624622	2.71455971	3.2780898	2.78488704	2.96108949	2.76701293	2.87812305	3.13963051	2.87498057
TMEM218	2.0362665	1.53842652	1.62266907	1.82714408	1.70145562	1.53323213	1.38330032	1.20701443	1.50389601
TMEM219	5.77667304	5.20650333	5.00638077	4.32829521	4.29186938	4.7875409	4.69937897	4.57100867	4.87209844
TMEM220	0.3891819	1.59707319	1.51466254	1.15789098	0.6405892	1.3036117	0.56831873	1.42476835	0.36052202
TMEM222	3.95304887	2.82326267	3.75721852	2.77347582	2.61631277	3.13734922	2.78703594	2.77548093	3.47650314
TMEM230	4.94403865	5.96519555	6.03110106	5.85197196	5.10901657	5.08015888	5.1176132	4.62966772	5.74616295
TMEM231	0.68236602	1.2470872	0.7138837	1.49915589	0.4154941	1.08635907	1.79128196	0.72025449	1.5839261
TMEM234	2.25414411	1.21975653	1.99447205	1.4180286	1.11341934	1.32776386	1.43193377	1.14263839	1.71179296
TMEM237	1.66916185	1.40497559	1.20507483	2.12347106	1.63331288	1.68051676	1.40017378	1.09472171	1.34100077
TMEM241	1.09974928	1.03792158	2.54669413	1.65845919	1.34161861	1.19113325	1.70215134	1.51785682	1.26223998
TMEM242	2.01073818	2.36027973	2.56566642	2.95886577	2.47702774	2.3037341	1.9384357	1.36194493	2.04145882
TMEM245	3.05466774	3.64852463	3.99098447	3.34165203	3.55835662	3.64118678	3.02445844	3.13652337	3.1699477
TMEM246	3.47474656	2.31877806	4.18132136	3.04462512	3.66226893	2.22672003	3.13971197	3.70518304	2.74099971
TMEM248	5.34486179	5.17367138	5.4931516	4.85081781	4.5906994	4.64153781	4.8853225	5.19863685	5.03203638
TMEM25	2.25518419	2.18132146	0.71111812	1.02779274	0.17205245	1.2242093	0.33322335	0.26393567	1.23983341
TMEM254	3.2605661	3.44382407	2.70870078	3.33926078	3.48564983	3.25932928	3.81791806	3.06195312	3.39020571
TMEM255A	0.80983114	0.92040564	1.64341178	0.98703669	2.07688563	0.85468367	0.17047272	0.71014466	0.15073888
TMEM255B	0.70570053	1.1001505	1.40762546	0.77685423	0.5847093	1.35129652	0.58707004	0.42873985	1.87775026
TMEM256	3.59840089	4.63717704	3.87339108	4.62447399	4.13544099	3.92271826	3.08872439	3.72863163	4.8048491
TMEM258	4.98585961	4.08649225	4.50089144	4.31580189	4.15503787	3.8082763	4.18913154	4.15976024	4.44171084
TMEM259	5.64459655	3.57527876	4.67491043	4.23143566	4.62557099	4.47890287	4.3214404	4.67032485	4.79174345
TMEM26	0.17377897	0.74920129	1.42109431	0.85048554	0.92352395	0.98915093	0.86299924	0.38035546	0.48925381
TMEM260	1.25241841	2.04229038	2.12731493	2.19719079	1.93312964	1.77550121	1.5259164	1.52026528	2.3551709
TMEM263	3.80971558	3.98246894	4.53848466	3.91896092	3.30562367	3.8587722	3.96216292	4.46398869	3.18077063
TMEM30A	4.56004839	5.15312108	4.87722825	5.55903953	5.73031492	5.51561319	5.68910331	4.63327603	4.5600265
TMEM30B	4.80395857	4.40256864	4.40611733	3.83674418	5.31619625	4.26834056	3.9635626	6.35253603	3.87309546
TMEM37	4.22842834	4.06403943	3.76099453	1.57713073	3.44079928	2.91304026	3.18198102	4.56895555	2.24213203
TMEM38A	2.15273215	1.67616065	1.38374981	1.04909811	3.42579696	1.04009051	1.78267099	3.07321856	1.45470646
TMEM38B	2.22107829	1.98107338	3.41322812	2.91548809	3.27189576	2.48720722	1.90126377	1.73970299	2.98449355
TMEM39A	3.33756828	2.78347567	3.63100069	3.0463311	2.71796452	3.09357298	3.24202082	3.49994607	3.2258946
TMEM39B	3.1841416	2.41848624	2.54780057	2.42476568	1.81638591	2.69236326	2.17729954	1.59773807	2.71390693
TMEM41A	3.22221287	1.7812526	3.30436116	2.3326404	2.56674579	2.2810302	2.74854725	2.4951624	2.6821971
TMEM41B	2.98203275	2.01315774	2.49227157	3.08498567	2.80598507	2.97057886	3.44035581	2.94528671	3.44987095
TMEM42	1.95574176	1.49886673	2.0752361	1.70453401	1.46919449	1.73461354	1.40888457	1.43075157	1.84770365
TMEM43	4.06056889	5.24257689	4.61879564	5.3775176	4.11621284	5.23562697	5.12573439	4.37774331	4.64360916
TMEM44	3.40125171	1.17397176	3.26170005	1.50297265	0.65059061	1.15385887	1.96139998	2.09435816	1.97728538
TMEM45B	4.75690859	5.12206046	4.04081186	5.2131454	5.14398211	4.27463076	6.9090061	2.42179374	6.27815368
TMEM47	1.74649957	3.96629265	2.98702972	3.82065306	2.12142449	3.58400094	2.58186711	2.20241922	1.39748973
TMEM50A	5.20843449	5.60722348	5.38095652	5.66540098	5.78561158	5.55066781	5.27054947	4.86809725	5.37508702
TMEM50B	2.1576568	3.90378142	3.37391289	4.29762302	4.14352908	3.67257831	3.73874222	4.10050333	3.59251135
TMEM51	4.7400696	4.08753538	4.22029653	3.7689448	3.86545774	4.1117498	3.90700417	3.44242788	4.85194488
TMEM52	0.59180982	1.3206719	1.13894972	2.08806383	0.50569942	1.68117292	2.74244303	0.62632152	2.35947311
TMEM52B	0.65922244	0.27680834	0.82714784	0.5549745	0.51419584	0.84646134	0.33347917	0.33925836	0.16089484
TMEM53	2.37783069	1.89175552	2.32672335	1.92036416	1.72391162	1.94184675	1.79192035	2.02487564	2.32718515
TMEM54	7.58513409	5.57090088	5.92234606	5.08685901	5.77937702	5.07013749	5.73178017	5.26636686	6.59075043

TMEM56	0.01469589	1.21261729	2.26585927	2.07400789	0.13870633	1.87810815	1.90366331	1.82266854	0.44801615
TMEM59	4.84241421	5.11246545	5.96763428	5.17740358	4.85916124	4.84471753	5.53364036	5.53147486	5.13934079
TMEM60	4.52369654	4.28024123	4.15570178	4.52823352	4.25226075	4.28069193	3.90815749	3.21858736	4.13121814
TMEM61	0.12712085	1.00178192	0.09294815	0.47180336	0	1.13290759	0.07743279	0.95664981	0.69360793
TMEM63A	3.48050598	3.11827639	4.09835562	3.33192127	3.29025801	3.17935321	3.9781124	4.5728782	4.32785287
TMEM63B	4.42054286	2.83879026	4.15136897	3.74734037	3.60146426	3.24591434	4.4320356	5.06754306	3.69143822
TMEM64	0.80992294	3.19330114	2.57734475	4.15238858	2.684129	3.62606488	3.42219195	3.22159286	2.24809573
TMEM65	1.04877741	1.60943817	1.17069853	2.36018073	1.15411275	1.90921386	2.45772617	0.87209986	1.08849301
TMEM67	0.6898926	0.66322462	1.53594179	1.25648196	0.66056124	0.8491228	0.66555059	1.03806405	0.75522697
TMEM68	1.56525638	1.72258118	1.8051778	2.5265885	1.91725612	1.87390088	2.15029362	1.82663249	1.91775877
TMEM69	3.58241805	3.57915475	4.45873351	4.46290223	4.43188084	3.91716099	3.70971107	3.65130241	4.57687303
TMEM70	2.97331091	3.7698278	3.28840933	3.84926155	4.08086306	3.92920906	3.90309124	3.16352838	3.47353313
TMEM71	0.26867612	1.63313758	1.23444828	0.95383163	1.52250561	1.93111179	0.98901117	0.86017639	0.45062611
TMEM74B	0.46174987	1.2359258	0.82212913	1.88295165	0.64463317	0.55206858	0.62984248	0.27675708	3.76930663
TMEM79	1.60924394	0.90883967	1.36897642	1.68872822	1.22624139	1.12450394	1.50541999	1.55408802	1.34801848
TMEM80	2.77513068	1.63091114	1.61034709	1.80809128	2.39884479	1.57814071	1.98431913	2.59834819	2.84352461
TMEM86A	2.16635945	1.77598644	1.81757984	1.47077239	2.49186546	2.05995908	1.65165127	0.76543328	1.24913168
TMEM86B	0.56656427	0.31318842	0.54468168	0.61445208	0.78492412	0.46278205	0.5294755	0.34978237	0.53104589
TMEM87A	3.14181216	3.62258441	3.87434839	4.11784986	3.9034577	3.93229597	3.96534518	2.91940685	3.45772387
TMEM87B	3.17876215	4.08937898	5.62950728	4.02680816	4.39053058	4.02320253	4.29723519	4.83921798	4.68001176
TMEM88	1.58114262	3.32375536	2.29290605	2.16955661	1.54638054	3.18149274	0.93805989	0.83099317	0.87550369
TMEM8B	1.75920167	2.01744826	1.55139332	1.34930041	0.77551064	1.27004802	1.25127579	1.51420823	1.91114286
TMEM9	4.62229952	3.85923626	3.64013898	3.2202216	2.37675609	3.33311461	4.75461203	4.02015065	3.73611037
TMEM91	1.07813088	1.05770605	0.85029653	0.8883279	0.91028407	0.95318341	0.52414227	0.27788625	0.43955177
TMEM92	3.87154121	2.38356484	2.04238669	1.05327414	1.44697506	1.51386838	1.1016242	2.98435838	3.87293053
TMEM97	5.60241808	2.92340159	5.5807409	4.97116541	3.75763839	3.08663685	3.80154729	4.53867356	4.48495343
TMEM98	6.23214522	4.20701489	4.86080365	3.62252429	3.54013872	3.67093286	2.17314568	3.39461734	4.0255166
TMEM99	2.51513711	1.20084132	2.57732375	2.22350664	1.41873449	1.55834364	1.01745144	2.12143215	1.53612296
TMEM9B	4.30787529	4.30090986	4.27351483	4.30969459	4.36600768	4.40036604	4.80766723	4.27694156	4.29471644
TMF1	2.30702349	3.0994468	3.48655734	3.20442945	3.45434764	3.11058092	3.51926403	3.56061428	3.38777958
TMIGD2	0.79961527	0.78437655	1.46692715	0.26802769	0.63683679	0.77687336	0.43027479	0.36228041	0.81358123
TMLHE	1.89206999	2.59536794	1.91587536	2.1237011	3.03245293	2.20298268	1.94170949	2.01827593	2.43150716
TMOD1	0.11337132	2.55434984	0.80175168	1.30535655	0.50984035	2.17294676	0.28231223	0.77311774	0.48653579
TMOD2	0.33153172	1.67237806	1.32251622	1.37604865	0.9482365	1.67698534	1.09215796	0.62029602	0.822117
TMOD3	3.25712512	3.54052598	3.46649493	3.74205647	3.7895288	3.68439301	3.50209016	2.94857343	2.85864423
TMPO	3.95684694	2.86513845	4.10851849	3.79628849	4.92378755	3.87132475	3.91355631	4.33139629	4.30718482
TMPRSS2	1.02906321	2.90150683	5.76736271	2.93594	5.3438263	2.13564933	3.79874579	6.41418941	5.20427219
TMPRSS3	1.22035111	0.88456454	2.69276599	2.80696335	3.22204141	4.04211228	2.40201401	3.17718533	0.85736359
TMPRSS4	4.86424262	2.97979872	3.43081275	3.82453661	3.73630244	2.8095267	2.05456319	3.49488794	4.62686076
TMPRSS5	0.05374948	0.11308144	0.43259106	0.93858123	0.10567712	0.12057591	0.45337579	0.69757962	0.13063461
TMSB10	12.0161429	10.3293512	10.9012694	11.3926601	11.6874781	10.6001594	10.9517116	10.7504297	11.6124222
TMSB15B	0.73376851	0.01876935	0.18324462	0.1140932	0.77858203	0.08927867	0.02349934	0.09351295	0.13558383
TMSB4X	9.41750859	9.87223553	9.11438238	8.7186849	9.62946317	9.15641555	9.52408352	8.03573439	8.08045531
TMSB4Y	0.22217923	0.51953162	0.51244727	0.01304175	0.11625697	0.74236116	0.02590876	0.84736413	0
TMTC1	0.4016696	2.60211884	1.06253896	1.47511809	1.24653434	2.18810542	0.98511212	0.78890813	0.47143529
TMTC3	1.75067595	2.71570445	2.34696407	2.80622482	2.18627072	2.41855097	2.56765968	2.37457445	2.23674985
TMTC4	2.36979242	2.28292453	2.64572842	2.08668049	2.48100019	2.10685224	3.12672512	3.16856546	3.69499967
TMUB2	4.05550701	3.10428291	3.99828706	3.15163168	3.05161092	3.20832837	2.82755224	3.41020427	3.61487032
TMX1	3.62880882	4.1239157	4.11771695	4.46762531	4.59445379	4.40314567	4.03687566	3.78871062	3.87634964
TMX3	0.72966467	3.0442463	2.79959812	3.30114741	3.54695759	3.24593409	3.15997006	2.14255802	2.55850776
TMX4	2.52927391	4.16597771	3.51726815	4.1596418	3.36404432	3.99296375	3.52177264	3.01513348	3.43011658
TNC	2.71781602	5.65465659	2.6138596	4.12049641	3.21490864	5.8657325	3.42395792	2.13586255	2.62308149
TNFAIP1	4.44491214	4.40344457	4.90020299	4.60709657	4.3637282	4.78843269	4.4518282	5.13148699	4.33255093
TNFAIP2	7.21335771	2.85787608	4.61653113	4.1423932	5.48122769	4.99488776	4.54277567	5.00618173	3.34025075
TNFAIP3	3.61976576	3.9500545	5.78487159	4.57424616	5.36462019	5.36564466	3.5758534	4.10786033	3.97710091
TNFAIP8L1	2.38433247	2.12280505	2.09188633	1.591737	1.81214171	2.22078174	1.98548126	1.48651457	1.84605312
TNFAIP8L2	2.67898137	2.52756423	3.50255538	1.77889674	2.7821576	2.93398593	2.47099147	1.6203788	1.99941225
TNFRSF10B	3.36464335	3.36609671	3.77562804	5.25369678	3.97014172	4.52347868	4.51560954	5.32166366	3.84691408
TNFRSF10D	0.98418508	1.35745216	2.0519504	3.32811843	0.49924149	1.75005733	3.3598415	3.5282193	2.63268905

TNFRSF11A	2.68289973	1.78007595	2.28455768	2.75781914	3.1484163	1.42237767	3.76922828	1.85773026	3.07577421
TNFRSF11B	0.47838018	1.71043579	1.03269676	1.82641088	0.71243456	0.6337427	2.96619284	0.69899686	1.6364347
TNFRSF12A	5.05158759	4.79793853	4.54101918	5.17299772	4.57927503	5.9239447	4.96056984	5.33792495	4.90861784
TNFRSF13B	0.11821583	0.16118435	0.33004347	0.1683967	0.22228837	0.13665567	0.04238892	0.14660691	0.06334426
TNFRSF13C	1.08030822	0.65291175	1.29183334	0.71860196	0.89850607	0.36149772	0.11172146	0.50304586	0.46513217
TNFRSF14	4.00630758	2.44960212	3.36866752	3.11754446	3.39904674	3.3402781	2.40040942	2.78804879	3.97724488
TNFRSF17	0.18342014	0.90084395	1.92895413	1.93857233	2.33647087	1.14541941	0.09441022	0.37417381	1.72427493
TNFRSF19	1.66400979	1.13001366	3.29469305	2.46322082	1.21092994	1.36801039	0.50285071	0.91346697	2.96520312
TNFRSF1A	5.55015763	5.33780531	5.28137004	5.07450137	5.1181915	5.52870054	4.90441445	5.04757721	4.72300375
TNFRSF1B	4.46440892	5.01581451	4.54178344	4.03468496	5.05844652	4.9065876	4.53934995	3.45434617	4.12908811
TNFRSF21	7.02563845	4.91500468	5.81896549	6.2038797	5.80217415	6.16636539	6.14396448	5.34613151	4.07997855
TNFRSF4	2.63479596	1.38642739	2.21331684	1.32025236	1.66046387	1.81058579	1.58234806	1.23395946	1.44799598
TNFRSF9	0.73669773	0.38544785	1.37526538	1.10050291	1.79918527	1.09900804	0.85385584	0.53205513	0.91463604
TNFSF10	5.69871992	5.61265342	6.13939194	4.39320775	6.52635415	5.83080159	4.03144665	5.27919306	4.93781936
TNFSF13B	3.81058524	4.18054808	3.75225048	3.4309812	4.71221538	4.73778301	3.02032997	1.82371778	3.03586717
TNFSF14	1.13761484	0.79065042	0.71520242	1.23539179	1.79717021	1.01564473	0.43435026	0.38252865	0.4566992
TNFSF15	1.68233963	1.64423513	2.96588537	1.45041839	1.60861709	0.80690678	0.57228143	1.16371845	1.34297045
TNFSF4	1.04529366	0.79421993	1.9281443	1.17700597	1.58760199	2.0024472	1.8877585	1.76165507	0.61562145
TNFSF8	0.56375602	2.26877406	1.52812067	1.77397081	1.7988693	1.97763504	1.1625126	1.08113199	0.98058778
TNFSF9	1.92283945	2.47349781	1.06016716	3.83600168	1.21281195	3.69820101	4.94010569	0.1968484	1.82214834
TNIK	2.56418904	2.2986863	3.3717304	3.44288383	3.27751713	2.36128599	3.34311812	0.5497183	3.48802933
TNIP1	5.2806951	4.05583568	4.49621162	4.73221705	5.20978498	5.05957441	4.48440041	4.94544312	4.92792199
TNIP2	4.14517236	3.12340149	3.08538896	3.29893545	3.13059851	3.6438819	3.11755124	3.71386365	3.69363018
TNIP3	0.32741443	0.50444979	0.82779793	0.53654459	2.13585124	0.59915645	0.45757278	0.43723075	0.42420161
TNK2	2.50481812	1.60426678	1.77129458	2.25475854	1.63711154	2.3077559	2.90200774	3.02159368	2.27396552
TNKS	1.63865336	2.62585034	1.80617735	2.58701071	2.13703315	2.62286373	2.74891495	2.59613185	2.60202968
TNKS1BP1	4.87268533	3.67146072	3.63359812	3.41654548	2.78553284	3.80883978	4.12814012	4.09643659	3.13432235
TNMD	0	0.38468566	0.0162621	0.02690941	0.17076914	0.05707639	0	0.02593922	0.04682637
TNN	0.12538254	0.45095843	0.31940081	0.10115544	0.02224345	0.33706029	0.11307016	0.22545042	0.07454537
TNNC1	0.38160967	1.24800951	2.90993685	3.17091269	0.1109557	1.94295129	2.70669994	1.0358544	0.22716027
TNNC2	0.1197051	1.27534078	0.56480097	0.35255555	0.07491038	0.23893956	0.25082313	0.12289349	3.07250249
TNNI2	0.86168045	0.54071325	0.57449644	0.74187776	0.88642688	1.02105117	0.50515313	0.36507858	0.27336915
TNNT2	0.0149839	0.0171526	0.18685734	0.22799495	0.33519912	0.03282832	0.02384502	0.47680107	0.04418032
TNNT3	0	0.48565246	0.04045603	0.28505059	0.01738734	0.15327285	0.05011174	0.03246396	0.02221625
TNPO1	2.86123922	3.94144183	4.04134105	3.76859447	4.41478707	4.08204992	3.83065678	4.25418903	3.98141017
TNPO2	3.59155063	2.99651665	3.32109069	3.65390921	4.11616916	3.339039	3.08300536	3.32184996	3.79806338
TNPO3	4.30981947	3.83577311	4.03609173	3.98799805	4.34197376	4.14742119	4.23881362	4.23957117	4.06698539
TNRC6A	2.36281025	2.72822718	2.36074212	2.58494487	2.9638131	2.50423378	1.9889708	2.41143121	2.46036946
TNRC6B	2.41455651	2.56918132	1.83035982	1.92180462	2.14992893	2.14456458	1.94796867	1.74021838	2.16049518
TNRC6C	2.32591087	1.55673867	1.19054977	1.21063184	0.73975451	1.4638713	1.0700252	1.58475789	0.73052415
TNS1	3.01529584	5.76358617	3.36411945	3.84602733	2.49805563	5.43582474	3.18456523	2.86110961	1.74820022
TNS2	2.13622895	3.80054158	2.99712771	3.06637165	2.03824773	3.40200515	2.11923164	2.60333931	1.64374387
TNS3	4.0443263	3.22731474	4.29734962	2.77948237	4.08827017	3.7759721	4.03595985	3.68342172	3.3770697
TNS4	0.38104625	1.3348644	1.00655878	2.73137102	5.34389633	0.3073737	2.9380894	5.93365853	4.6278592
TNXB	0.2655805	3.73644623	0.91761728	0.89266355	0.18455687	1.90075261	0.41463837	0.50530011	0.25039772
TOB1	6.10725064	4.8202961	5.45665586	6.11492546	5.75593006	5.14532568	5.19401402	6.70939187	4.82727072
TOB2	4.959788	4.23664626	3.82346928	4.16874215	3.59935618	4.51355642	4.32150913	3.88163445	4.33882684
TOLLIP	4.24499756	3.19687374	2.79982571	3.14778502	2.65320106	3.43439348	3.72330187	3.67676885	3.48945329
TOM1	3.55227579	3.07694846	3.16117866	2.92272946	2.51891688	3.04470822	3.02041816	2.76504855	3.4205173
TOM1L1	2.73175952	1.94270332	2.78037489	3.02529612	2.50980577	2.4783526	2.72785333	4.21352085	3.54566644
TOM1L2	1.88073739	2.88508801	2.38653636	2.51758419	4.07342973	2.70219027	2.56405533	1.84216169	2.94217472
TOMM20	5.08139743	5.25164277	6.20083836	6.35882855	5.16409601	5.77757877	6.59704059	7.30174302	6.18813544
TOMM22	5.33988516	4.45475728	5.1293791	4.83201058	4.21674404	4.89305305	4.59757348	3.8903602	5.44281232
TOMM34	4.03558916	4.40142122	5.02199689	5.07784599	4.75650424	4.95300057	5.29845876	4.84241	5.33573776
TOMM40L	2.15833123	2.42101722	2.03823817	2.10103008	2.2195701	2.58036801	2.87853174	3.10318287	3.00010512
TOMM6	0	0	0	0	0	0	0	0	0
TONSL	2.36437711	1.33693742	2.0714322	2.38876581	2.46102823	2.29742362	2.78861275	2.22113641	2.60075358
TOP1MT	2.84499289	1.62201137	2.28995524	2.35220777	1.74766504	2.27339772	2.74473565	2.36355921	2.50844027
TOP2A	4.33533068	3.42042005	4.38548394	4.53974444	5.64526862	3.81081766	4.83501696	4.12970258	4.77665865

TOP2B	3.60253889	4.5100905	4.16681642	4.95959565	4.95153093	4.83497022	5.04799931	4.57976265	4.93348113
TOP3A	1.70078337	1.8616942	2.4816103	2.34193367	2.78851074	2.41642072	2.09313481	1.68328376	2.54064217
TOP3B	0.20909153	0.06754949	0.13970854	0.10475495	0.10117194	0.07657001	0.06110392	0.05804047	0.10296779
TOPBP1	3.50793568	2.6834067	3.36698101	3.41743824	4.3038696	3.35860877	3.58686447	3.18301142	3.67908574
TOR1A	3.12111481	3.5139968	4.57296137	3.88513924	3.80269178	3.91365271	3.34525913	3.0962217	3.71158873
TOR1AIP1	2.65399056	3.56339513	3.42202961	3.28399024	3.1014628	3.61925677	3.63015608	3.90606429	2.3630136
TOR1AIP2	1.75337561	2.05012849	2.15467811	2.65408106	2.04283435	2.41296352	3.00045649	3.20297397	2.38270281
TOR1B	3.67635252	3.81435907	5.27281238	3.44384556	4.36933191	3.83657952	3.90936323	3.15977942	4.2433905
TOR2A	3.81253278	2.19213059	3.19054653	2.03699083	2.45504235	2.39216281	2.27448436	1.67427142	3.1972665
TOR3A	5.1367262	2.59514105	4.06154464	2.85080761	3.82482465	3.46590673	3.77867195	3.78828007	3.53103777
TOR4A	2.02481927	2.97419835	4.85495018	2.84148908	1.74032336	3.98733601	4.43157152	4.89670547	4.15404841
TOX	0.6038706	3.16659982	1.60459452	2.00171842	1.67639348	2.07715192	0.66511187	0.70841427	2.87489392
TOX2	1.15232666	1.33711409	1.52322505	1.45906535	1.69618643	1.1881792	1.1227741	0.73255376	0.85728359
TOX3	4.03871789	2.5693613	3.76000738	2.06703396	3.36261585	0.70735102	0.20951305	4.3823853	3.40049348
TP53	2.35445648	3.97283006	4.33338517	5.10369056	4.92865978	4.31269275	4.57741792	3.58024642	5.77385123
TP53BP1	1.76618223	1.94581104	1.62584503	1.77580614	2.03907788	2.14270739	1.69736071	1.51270234	2.05069382
TP53BP2	3.49894012	2.8508979	3.69157199	3.33767387	3.47680919	3.22888056	4.00777661	4.08149961	3.43899584
TP53I11	4.26283772	4.36483809	5.66942906	3.26309273	4.92045649	3.81587049	4.16707148	4.49003805	5.14070638
TP53I13	3.73506513	2.10621224	3.3047609	2.23299658	1.60235318	2.65137672	2.48100688	2.95051299	2.84868053
TP53I3	3.21106222	2.77384363	3.29882543	3.89443658	2.79427753	2.79443197	2.35261242	3.63567141	3.03901945
TP53INP1	1.16746955	2.7584186	4.25546407	3.56505739	2.74123861	2.72406155	2.46843039	1.44478314	2.67423139
TP53INP2	3.35994465	4.59389658	4.02203717	4.17582028	3.206939	4.05500106	4.37354112	3.97998594	4.68937728
TP53TG3	0	0	0	0	0	0	0	0	0
TP73	0.42213697	0.24106301	0.65269612	0.73796306	0.27154362	0.44315693	1.00606039	0.25100294	1.84466398
TPCN1	2.4188067	3.03826936	3.10764889	2.88697094	2.55498079	3.00030808	3.12981421	3.14640528	3.60812616
TPCN2	1.85472749	1.60717968	1.78671749	1.93302681	1.61540418	1.97504003	1.90329858	1.49580195	1.745211
TPD52L2	4.63558596	4.10323884	5.50331958	4.96431516	4.26313542	4.78242739	4.99187476	4.13290139	4.57084991
TPGS1	2.09408439	1.48215816	0.94263647	1.01876355	1.08307133	1.56101501	0.93926243	1.48553717	1.29618941
TPGS2	2.32754593	3.16609923	3.27537614	3.35922146	2.64107961	2.82186296	3.64317132	1.98759947	3.12974534
TPH1	0.01289808	0.07236563	0.72707896	0.06088092	0.12236334	0.0375884	0.02308083	0.09656426	0.02364061
TPI1	8.58040495	6.63875632	7.20911174	7.19201387	6.95667457	7.4520168	7.31248152	7.30707663	8.0750158
TPK1	1.18419582	1.14449204	2.27411349	0.89279013	1.89972782	1.0413776	1.72293282	1.33256197	0.9626703
TPM1	4.56764951	5.6418096	4.41764938	4.96410079	3.75637062	5.37259061	4.48758067	3.82798353	3.88652896
TPM2	4.76642353	7.16116346	4.34926379	5.55537887	2.66739042	6.95925218	4.29666698	4.65011899	2.80012111
TPM3	5.53777572	5.38197217	5.63471883	5.81870257	6.27943636	6.02733486	6.29505099	5.90375403	5.98137223
TPMT	2.87836292	3.6022526	4.81326336	4.39339678	5.05845664	4.26337723	5.24860261	5.00329321	4.9844151
TPO	0	0.30425875	0.14628151	0.19129606	0.00654897	0.24265161	0.0654618	0.01633357	0.03321817
TPP1	5.68137251	6.83006705	6.17512112	6.50919171	6.71897355	6.545858	6.51481575	6.04391313	5.88387547
TPP2	2.66509137	2.88359474	3.17827314	3.57941178	3.94146278	3.02039292	3.6692643	3.19311662	4.12699442
TPPP	0.40382151	2.318667	2.27043738	1.87192752	1.92257204	1.1560205	1.39627563	1.32401791	1.78094961
TPPP3	1.10963428	3.25889533	5.5456673	2.86417513	0.94213887	1.66759508	1.57589337	2.40779167	1.94122677
TPR	2.93389085	3.23618621	3.13041874	3.64386621	4.02358094	3.43360233	3.77995403	3.88679724	3.58420934
TPRA1	4.2830769	2.50787721	3.07034306	2.43972741	2.43790818	2.71760821	3.06913513	2.89921961	3.09676921
TPRG1	0.18284198	0.41396726	0.85723645	0.72949132	0.3806665	0.24008158	0.20841845	0.30672841	0.03767673
TPRG1L	4.05244756	4.58169009	4.08212157	4.77579562	3.93837962	4.71866782	5.27068157	4.02807526	4.62141694
TPRN	3.47972199	2.02739512	3.10326362	1.31649683	1.28008791	1.64136144	2.15302465	2.70996438	3.86783637
TPSAB1	2.51387267	6.92055754	4.07145309	3.92634655	1.72936601	5.0710319	1.98926859	1.93617457	0.82604825
TPSD1	0.9681894	0.05350118	2.00074398	2.26269517	0.01458121	0.04305447	0.30672336	0.50533402	0.131381
TPSG1	0.13834832	0.50087598	0.40046308	0.06805949	0.18447424	0.37387873	0.06790991	1.07683479	0.13122636
TPST2	2.87719514	2.18136848	2.47356193	2.52498981	2.46722242	2.49711022	2.28298977	1.34531525	2.5947283
TPT1	8.83485508	8.53925789	8.00365589	8.35607454	8.42287732	8.25127544	8.20037653	8.56567637	9.30939086
TPX2	4.44890395	3.50330405	4.56777928	5.41832146	5.17360054	5.12124674	5.25242734	3.97347674	5.10467383
TRA2A	3.77260203	3.03380613	3.59667283	4.24995683	4.52065258	3.45974775	3.32031028	2.95648249	4.03171497
TRA2B	3.77820223	2.93389639	3.44398518	3.7520488	3.89148356	3.65584771	3.53976137	3.54784439	3.72799162
TRABD	4.98503579	3.26376068	3.27365532	3.62012128	4.00884163	4.20790459	4.05031399	3.65127537	4.73477907
TRADD	3.62143693	2.54369684	3.26877928	2.8228025	2.6987923	3.27953601	3.27634863	3.82388757	2.92412045
TRAF1	1.57252367	1.07922161	1.89296035	1.57586892	2.18194777	1.52517821	1.07020092	1.21944976	1.3646486
TRAF2	3.12271234	1.58820208	2.97833018	2.16206748	2.48462659	2.72908696	2.24369195	2.6717195	2.50033514
TRAF3	2.53600789	2.44339636	2.03209971	2.47550809	2.67501381	3.01617448	2.16845638	2.77020029	2.6909341

TRAF3IP1	2.54381697	2.11381933	2.11382047	2.09849788	1.81746804	2.12741088	2.2726321	2.24648233	2.31125027
TRAF3IP2	3.43801654	3.10810147	2.99477138	3.03965415	3.34243063	2.74499639	2.97208608	3.34333438	3.33689351
TRAF3IP3	0.80172452	0.85027573	1.38837186	0.89110651	1.8768419	0.90541365	0.71711961	0.40230117	0.66067972
TRAF5	1.0935639	2.05420915	2.45436861	2.26754082	1.92452216	1.8204235	1.59200377	2.26976884	3.32912981
TRAF6	1.82001137	2.50858296	2.25716641	2.62556134	2.9145535	2.48392672	2.33133005	2.41291661	2.77783871
TRAF7	5.20545685	4.90399161	3.83411489	4.39121292	4.27293853	4.43724133	5.20605898	4.77475042	5.1494762
TRAFD1	3.23843102	3.70391007	3.79369719	3.39196699	4.64243848	3.84677769	3.10292883	3.30939062	3.72173376
TRAIP	2.02078265	0.79587737	1.58071577	1.54282709	1.92221936	1.53329565	1.57251338	0.97144704	1.45935196
TRAK1	3.45784916	3.11767647	5.05344136	3.83404624	3.9486955	3.11444488	3.40407943	5.55835526	3.46432783
TRAK2	2.87567073	3.51317302	3.31738866	3.30022919	2.89165539	3.31109063	2.71050887	2.65863301	2.72680992
TRAM1	5.77610513	6.42247849	7.27179007	6.26521854	7.0629968	6.56175864	6.46139861	6.17242165	6.18869553
TRAM1L1	0.01790375	0.88843734	0.36590557	0.49779023	0.08616082	0.56730274	0.21035627	1.08173381	0.07383412
TRAM2	2.65675537	4.10082482	3.28033517	3.66780067	2.97737768	3.70682339	3.49060604	2.74106923	3.14351992
TRAP1	3.59708289	4.01428341	2.83698072	4.08891149	4.3235092	4.22349389	4.26455443	4.03406025	4.72710159
TRAPPC1	6.45700328	6.10464016	6.57923544	6.36315446	6.63397872	6.2113354	5.53014827	5.27233695	6.45415575
TRAPPC10	1.20759451	1.70271352	1.77698875	1.93749135	2.603485	2.26613314	2.11792912	2.26096368	2.25423798
TRAPPC11	2.4645421	3.03675924	2.65088645	3.46149208	3.82691117	3.17338356	2.94244902	3.47678243	3.2823765
TRAPPC12	2.6435207	1.93321563	2.08405399	1.86176522	1.93175855	2.28392055	2.01429294	2.68221164	2.28080032
TRAPPC2	1.29634273	1.54088605	1.65202253	1.76651584	2.23893401	1.3821042	0.95675178	1.17090113	2.27236088
TRAPPC2B	0.56534343	1.87864011	2.48154196	1.74144459	1.68362069	1.74281366	1.61327471	2.03970808	2.05259329
TRAPPC2L	2.97664791	2.29097543	3.24383245	2.40392787	2.8680826	2.54172284	3.07038336	3.04313961	2.68210428
TRAPPC3	3.91125362	3.47256761	4.33611064	3.50374927	3.58972383	3.58118248	3.1843449	2.85904488	3.46378321
TRAPPC4	2.79021683	2.45314722	2.3588107	2.78107722	3.31774383	2.70013174	2.97412044	2.84111489	3.07372186
TRAPPC5	1.1249043	0.65938404	0.90492774	0.61795145	0.73494053	0.90887525	0.78705493	0.93117408	0.71938044
TRAPPC6A	4.23387866	3.70087904	2.89824896	2.86523328	2.29859045	2.78853303	2.62153834	3.20200131	4.18863891
TRAPPC6B	2.33191797	2.67687355	2.84886455	2.90103087	3.2748457	2.80343649	2.47687461	2.53190374	2.66250604
TRAPPC8	1.47331958	2.84169671	3.36202076	2.89077209	3.38378069	2.8108351	3.18730238	2.32394069	2.59789229
TRAPPC9	2.37162786	3.05410522	2.75612367	2.70951445	2.02574388	2.97427997	2.70432059	3.41100033	2.92443251
TRDMT1	0.54872453	0.62015562	0.51389315	1.10010764	0.99149746	0.64592761	0.38486512	0.32458551	0.98042193
TREH	0.27072336	1.87372968	0.47510557	0.47072346	0.14104743	0.16297681	0.02716707	0.08984572	0.26340008
TREM1	0.75082023	1.21431529	1.32485491	2.9198955	1.51347436	1.98037942	2.84104667	1.68296374	0.9039048
TREM2	4.36685559	3.75639795	5.17995819	2.80022248	4.02863178	4.12818832	5.24465122	2.94733038	2.3224299
TREML1	0.09509645	0.34846005	0.5893505	0.14038298	0.1711116	0.13768718	0.33926981	0.13550834	0.07549547
TRERF1	2.26196587	1.92159978	1.62159709	2.13070235	2.46376193	1.66738391	2.1359197	0.85974862	1.2005744
TRIAP1	4.1149726	3.96628671	4.2156666	4.45111487	4.50618596	3.90067062	3.83553577	4.44555306	3.73760491
TRIB1	4.25878339	4.3280973	5.02177027	4.5684302	4.14337384	4.87792627	4.6719523	4.79394264	4.7373918
TRIB2	2.91584296	2.97156496	3.83976147	3.13413277	4.48148134	2.68478568	3.99582114	3.31486739	2.1424568
TRIB3	0.58518575	1.05276136	3.34367683	3.16877438	0.88907214	3.27807525	3.00188565	0.8724258	3.39400122
TRIL	0.59145933	2.47948935	2.22983645	1.40554837	0.5893352	1.61594094	1.33157787	0.98157754	0.53003275
TRIM11	2.72524484	1.16590059	2.10241267	1.58001904	1.96031867	2.013703	2.34255796	2.24614666	1.98137518
TRIM14	4.0396813	3.56364313	3.84617229	3.23685112	4.44933042	4.18052667	3.73860733	2.85206078	4.50746398
TRIM15	2.91243425	1.16526363	2.56384055	2.4739982	3.19366636	1.80825822	2.48224221	2.62846972	3.37602148
TRIM16	0.87075557	1.80053113	2.29257394	2.85807472	2.59483438	2.70443412	2.1054116	1.68939278	2.37331818
TRIM2	2.32363827	3.49081994	4.60993299	4.90343653	3.3798182	4.8488655	4.77364853	4.66633414	4.9728813
TRIM21	3.92684396	3.96184624	3.48775477	3.64232648	4.61618081	4.42837274	2.80834935	3.57378151	4.14508831
TRIM22	2.55691802	4.60150703	3.94034665	3.77941433	4.98573554	4.52395926	2.56029003	1.67203596	3.06473468
TRIM23	0.76463571	1.73677025	1.29966763	1.62482917	1.38349783	1.57021784	1.16718646	1.62299774	1.25500964
TRIM24	3.24654111	2.52607324	4.01167835	2.52250143	3.17095331	2.09956146	2.44664018	3.23539742	3.03207664
TRIM25	3.70734983	3.3765833	3.26873059	3.17696867	4.31998909	3.83049832	3.28668532	3.11985217	3.3497831
TRIM26	4.70630361	3.2478437	4.3818109	3.35541435	4.19636641	3.84095627	3.65827111	3.90438062	3.95429679
TRIM27	2.98514154	2.54138707	3.82480369	3.10763916	3.23308933	3.2499736	3.21742959	3.81675864	3.4891661
TRIM28	6.78897632	5.07708605	5.45654578	5.96046233	6.21089949	5.97726575	5.75402132	5.86149478	6.18646676
TRIM29	1.23560919	2.85276525	0.15152093	4.69112538	0.26793838	2.31602156	4.31855738	2.6259955	1.05380843
TRIM31	4.17170202	1.98530766	5.63819912	2.6363927	3.87299561	2.80457384	3.71074304	3.20327949	3.06843524
TRIM32	2.39058573	2.80862235	2.90758304	2.87164138	2.53820863	2.88445314	2.45642213	2.07714007	2.85574793
TRIM33	2.44659424	2.92085786	3.05527473	3.40009285	3.61316874	3.23124576	2.88289882	3.21587585	3.10835251
TRIM34	0.27094922	0.51634932	0.42869537	0.23914554	0.58814879	0.40903074	0.14640545	0.4379818	0.37426018
TRIM35	2.70116234	2.29417507	1.94703166	2.55793963	2.26368866	3.01344448	3.17363434	2.25744047	2.52113154
TRIM37	2.8921426	2.44562172	2.9766304	3.16608384	3.23918394	2.805352	2.53047858	3.04933459	2.82714359

TRIM38	2.12964634	2.91649789	3.29599997	2.58983683	2.9359867	2.75755712	2.03749812	2.35405421	2.45868892
TRIM39	1.21478987	1.12291207	2.14275866	1.50942737	1.38160303	1.48820185	1.27535132	1.80154385	1.37813815
TRIM41	2.05963704	0.71739567	1.32006403	1.1205564	1.13492579	1.31308843	1.02896677	1.32479983	1.24870746
TRIM45	0.57684689	0.61668259	0.86970991	0.87945646	0.76712238	0.52166623	0.46189835	1.0323911	1.07070647
TRIM46	0.46357402	0.33177615	0.39842437	0.48323227	0.5357924	0.2564324	0.3336565	1.17409548	0.31737454
TRIM49	0	0	0	0	0	0	0	0.00647947	0
TRIM5	2.07662334	2.80113452	2.3088302	2.08192737	2.77960496	2.64863212	1.48569263	1.85943595	2.84184452
TRIM50	0	0.22468837	0.40326908	0.16573093	0	0.13345524	0.05163397	0.06075586	0.01530553
TRIM51	0	0	0	0	0	0	0	0	0
TRIM52	0.86392721	0.74015169	1.36567873	1.45251941	1.3577614	0.80240787	0.64578538	1.25986017	1.42742363
TRIM56	3.71827524	2.74658946	2.44223796	2.18712446	2.94504623	2.45289448	1.86038991	2.98008265	2.94427546
TRIM59	1.42732085	0.61339713	2.03159621	1.86393858	1.36791059	1.13917705	0.7784054	1.40135939	1.10868315
TRIM6	0.30662232	1.24555755	0.60644575	0.78077471	0.23079833	1.49816646	0.40252821	0.32378656	0.27331348
TRIM62	2.07311681	1.68994411	2.09886195	1.55643136	1.68110035	1.77441342	1.76084466	1.38714586	1.89409379
TRIM65	2.68395716	1.91068426	2.70239897	2.73072167	2.22202276	2.74533121	2.28319909	2.65578901	2.53014972
TRIM73	0.06428397	0.00751131	0.04672799	0.01569024	0.02010784	0.00241333	0.01175725	0.03997946	0.09364553
TRIM8	4.92489398	4.9819498	4.59028375	4.45040435	3.85501913	4.81104521	4.25812464	5.02560838	4.83448284
TRIM9	0.02424123	0.99745361	0.25806113	0.43011469	0.08527811	0.56803668	0.08523316	0.06749101	0.02547327
TRIO	2.05604142	2.7757492	2.31605365	2.76221335	2.33392618	2.92732775	2.23056561	2.03270553	2.58119917
TRIOBP	2.5083958	1.90332453	1.57625586	1.44304524	1.11340011	1.60811542	1.54178333	1.28075818	1.17267018
TRIP10	4.11462164	3.76999122	3.92242137	4.14608489	3.77338251	4.165387	3.85119023	3.5961477	3.1115277
TRIP11	1.83376216	2.8501888	2.20993647	2.68119517	2.99257442	2.72660731	2.10509055	3.42859095	2.68469227
TRIP12	3.38869205	4.23333134	3.89572328	4.12711016	4.5551646	4.53099025	4.30617459	4.3853957	3.92719998
TRIP13	1.85535993	2.0727747	2.28863928	3.0302051	2.56933365	2.82056936	3.05342382	2.24780488	3.39489057
TRIP4	2.82123024	2.91019913	3.45503612	3.07313539	3.27532503	3.03673083	2.98406811	2.16497784	3.17753118
TRIP6	5.50282753	3.19845331	4.814297	3.24115636	3.28591409	4.16377196	4.78452055	3.4100873	3.54767712
TRIT1	2.39487055	2.24604479	3.06241742	2.81642636	2.8386756	2.24060033	2.25242717	1.64012113	3.36239667
TRMT1	3.10803453	2.05860254	3.02881105	2.86402237	2.95410876	2.64654759	2.53738477	2.72572151	3.64593588
TRMT10A	0.99439102	1.08643724	1.19661136	1.65027082	2.00236346	1.40431978	1.10892005	1.40466147	1.8727779
TRMT10C	3.44288387	3.58748833	3.96495098	4.46834868	4.27843567	4.00700667	3.71262521	3.57685924	4.126353
TRMT11	2.22978884	2.59589367	2.2587662	3.3922087	3.65196951	2.90236468	3.45360497	2.74974852	3.52657896
TRMT112	6.17484244	5.04491193	5.18143304	5.49076719	5.53049823	5.45920124	5.37531244	5.41561148	4.98156325
TRMT12	0.64481528	2.04782879	2.88076571	2.45069686	2.1434701	1.70738427	2.49528447	2.51704422	2.09223587
TRMT13	1.2218521	1.63581905	2.13756634	2.36385304	2.52215086	2.05884395	1.63913291	1.70137592	2.48312957
TRMT1L	1.76812995	2.61095635	2.47553552	3.04841057	2.8479467	3.02271265	2.7296263	2.90109108	2.85555254
TRMT2A	3.36192517	2.11556194	2.67435827	2.08380146	2.43769795	2.42564595	2.01203973	2.13758092	2.81444028
TRMT2B	2.49440278	1.78871234	3.43931643	2.02053632	2.8591006	1.94304982	2.24272653	2.55084675	2.64366741
TRMT44	1.32072241	0.82065462	1.34381377	1.13420152	1.356972	1.17437512	0.77456191	1.51119722	1.04192926
TRMT61A	3.33943266	1.90918013	1.91005182	1.69131134	1.96576082	2.40370797	1.86444701	2.9909988	2.79625159
TRMT61B	2.11836564	2.42883959	2.60137218	2.40534321	2.89557519	2.52336427	2.24376779	2.53298511	3.14834001
TRMU	1.95995376	1.08593651	1.48936906	1.65436246	1.86299392	1.61609129	1.46420042	1.0744581	2.01389205
TRNAU1AP	1.90847462	1.86322968	2.44945941	2.09408169	2.28065709	2.49398786	1.99967804	1.75432214	1.94222266
TRNP1	7.56003835	3.02628142	5.91950711	3.92573028	5.30995897	3.28345532	2.84466975	5.44842524	2.15074901
TRNT1	1.56753902	1.91022101	1.78970956	2.18630877	2.61856142	2.20889343	1.92628117	1.98514464	2.06061166
TROAP	2.80981732	0.95643303	2.62194222	2.75151854	2.80455216	2.23755714	1.73346559	2.11900847	2.74031232
TRPA1	0.35756102	0.47772904	2.18276505	0.47977312	1.07248494	0.18868969	1.40751589	1.48547367	0.79149674
TRPC1	0.15219872	1.25880206	0.66506835	1.38141075	0.27757305	1.13694582	0.3334979	0.48356522	0.28689142
TRPC4	0.05108704	1.22776256	0.42065746	0.34927896	0.2222368	0.66843799	0.28186922	0.13693073	0.17564507
TRPC4AP	4.70539453	4.70946015	5.43176646	4.84320049	5.15547996	5.14036547	5.23857834	5.11196301	5.64723606
TRPC6	0.28568951	0.8971566	1.38686654	1.15817095	0.98410825	1.00537304	1.06705384	0.52214423	0.54166007
TRPM4	3.22444476	2.58602623	3.36642606	3.01925541	2.3236719	2.8027036	2.43261175	3.57302031	3.41675739
TRPM5	0.12335371	0.25968475	0.10259462	0.11657372	0	0.01010389	0.03803856	0.09600307	1.03019847
TRPM6	0.00790714	0.45074379	0.51281776	0.07868311	0.02181456	0.2953371	0.23792717	0.05398413	0.18000282
TRPS1	0.50105559	2.4147691	1.61063213	2.13981956	1.44976996	2.41591002	1.93575391	1.10211838	0.3337044
TRPT1	3.94831145	3.13072356	3.60858803	3.03576004	2.25531629	2.65733033	3.37777406	3.76833693	3.84605611
TRPV1	0.00981652	0.01682767	0.06266531	0.02053659	0.02108326	0.0126116	0.00588425	0.01698336	0.05834561
TRPV2	2.40307877	2.52420751	3.09351751	2.26306144	3.1946203	3.10106733	3.01866071	1.89902469	1.76993189
TRPV4	1.13502183	1.21433851	2.20699088	0.96162084	0.83387195	1.68143423	1.36115266	0.82104407	0.64308073
TRPV6	0.13414948	0.81225432	0.03705559	0.04512617	1.79618458	0.01674609	0.01093514	0.05091893	0.14070287

TRRAP	3.32902973	3.05303006	2.9306989	2.80456164	3.54361649	3.22711721	3.30584345	3.10598097	3.22357746
TRUB1	2.42693097	2.93399662	3.59625781	3.64173395	3.50741356	2.79679872	3.00911211	3.39729487	3.67929934
TRUB2	3.02927943	2.67285358	3.51311393	2.77861577	2.52005141	2.46120205	2.68183064	2.37358348	3.08615964
TSC2	2.93007872	3.06336532	2.35913906	2.45334944	2.49612659	2.63780549	3.16021012	2.9066583	3.40823552
TSC22D1	4.12416824	5.05804481	4.21576137	4.73644386	3.64034793	4.71089447	4.50305016	4.62830558	4.99371912
TSC22D2	2.61482122	1.97940395	1.71324939	1.952321	2.02486764	2.27535115	2.11945369	1.75982111	1.60363671
TSC22D3	4.50898363	4.25236407	5.23870445	4.60495451	4.17324628	3.66415753	3.77017547	3.9230941	2.80416069
TSC22D4	5.71169962	3.19412089	4.01834679	2.94233673	3.41752583	3.58744292	3.73301865	3.83907039	3.26245946
TSEN15	3.44843754	3.83765416	4.40856339	4.15831755	4.43175706	4.00885292	4.38246352	4.96961081	3.92417239
TSEN2	0.63068106	1.06546058	1.46719615	1.65912699	1.96952422	1.35479127	1.58686917	1.38870372	1.78207087
TSEN34	4.22344982	2.95168966	3.15531162	2.78493629	2.19975315	3.25123309	3.20784982	2.60924333	3.34727372
TSEN54	4.36661876	2.04303696	3.88176574	2.69322693	2.80802533	2.64088981	2.7529658	3.55210884	3.4143591
TSFM	2.88934217	2.96520422	3.02771088	3.03662153	3.63783148	3.06691332	3.39382886	3.34668499	3.53717997
TSG101	4.02264353	3.94399033	4.34889374	4.3535571	4.46939276	4.21863561	4.48521042	4.39668475	4.41582055
TSGA10	0.60143321	0.46532847	0.6725784	0.75217316	0.63492576	0.50624877	0.32918326	0.48544359	0.77803052
TSHZ1	1.96838162	3.11152432	1.69268079	2.29981789	2.28264095	2.48252455	2.56769273	1.89328541	1.79467549
TSHZ2	0.4704295	2.81237992	1.00277481	1.43926763	0.69574604	1.7661293	0.79842657	0.42028512	0.19350674
TSHZ3	0.95135597	2.52572334	1.49555712	2.23338109	1.27056903	2.25316102	1.5439402	0.98244632	0.84898217
TSKU	4.09705827	4.26857534	2.82357171	3.75955121	2.30355488	3.89823135	4.6622527	4.21385866	4.4879844
TSN	4.22548378	4.15814539	4.83969703	4.76656089	4.41095376	4.47756508	4.80917513	5.03029644	4.74792566
TSNARE1	1.78730313	1.18220038	2.10295269	1.48854007	1.05803735	1.4850831	1.58443504	1.62351045	1.87143456
TSNAX	1.64947194	2.42004138	3.02679046	2.72776247	2.22049954	2.57348054	2.7553477	3.01260754	2.54447571
TSPAN1	1.64372601	5.80148849	5.44966373	4.66781117	6.60496583	3.22769178	6.83221978	7.19153441	6.43550516
TSPAN10	0.71269559	0.208548	0.58497933	0.11509466	0.08721143	0.05807852	0.03183029	0.30803805	0.03981808
TSPAN11	0.59589893	2.23027444	1.41425117	0.97781783	0.256673	2.21716745	1.34436323	0.88541465	0.66215028
TSPAN13	6.28352031	4.59933608	7.06880665	6.00694096	5.48149415	4.41698921	7.29778647	6.05560207	5.94893273
TSPAN15	6.00647682	4.69083041	6.25451253	4.99892931	5.56031824	4.62432623	4.94422282	5.61224867	5.43125344
TSPAN17	3.9562856	2.56547161	3.45785446	2.66088002	2.95088469	3.10370683	2.98154793	3.47545293	2.83209914
TSPAN18	1.62211091	4.1045488	2.41841511	2.65056752	1.27862813	3.41514762	1.5555643	1.23159571	3.0567838
TSPAN3	5.94514175	5.0889226	6.36288988	5.85885456	5.89651399	5.31108267	6.51041698	4.85469247	6.09742534
TSPAN31	2.67901367	2.59027618	3.03894805	2.18645605	1.99573621	2.22542793	1.52538907	3.13660481	2.53506779
TSPAN32	0.37077134	0.27023495	0.48477546	0.20893497	0.32697452	0.69955379	0.17322546	0.05806832	0.02006215
TSPAN33	2.67008601	2.29557637	3.80883442	2.58332287	2.45766385	3.11744941	3.01283874	3.0956446	2.54194014
TSPAN4	2.63895433	2.96167328	2.3861347	2.58310799	2.05839154	3.32405699	2.94584615	2.84583203	1.41117034
TSPAN6	2.83338197	3.58745223	4.74071262	3.60314716	4.0159592	2.77899937	3.27345898	4.04315838	3.22867895
TSPAN8	3.36967699	8.04239859	8.0062029	5.08598402	7.92531048	3.79012246	6.72071617	8.57506833	6.79321163
TSPAN9	2.57647578	3.26339764	2.41615907	2.50155384	1.53385474	2.80678304	2.22851239	2.34510109	2.36923456
TSPO	7.9921275	5.53662939	5.831778	5.34130233	5.78647513	6.87765305	6.78029229	5.91955541	6.504491
TSPYL1	5.1539614	4.32089961	3.82247356	4.98533523	4.4581578	4.32184176	4.92892099	3.46286081	4.07839439
TSPYL2	2.35473017	2.05397808	1.82880773	2.41195421	1.79581093	2.08137306	1.16794104	1.59590227	1.02564771
TSPYL4	1.84504859	2.93870477	2.38703985	3.62460433	2.86335346	2.96947436	2.62772075	1.92060312	2.83731053
TSR2	4.85529143	3.79938913	3.62645508	3.66539682	4.03651576	3.88767947	3.31767576	3.5856571	3.63831208
TSR3	5.58518893	4.32264338	3.31563403	3.70696028	3.5098947	4.76961724	4.68552664	4.72857097	4.03224283
TSSC4	4.16122806	2.29811123	2.21556577	2.23765715	2.68642285	2.91419178	2.8305714	2.70850484	2.72966151
TSSK3	0.09650288	0.17898202	0.58475982	0.28848284	0.3038025	0.11733063	0.09628597	0.17487968	0.29900783
TST	6.51810511	5.19595933	4.58095529	3.38461391	3.94159596	2.95093286	4.40414219	4.51477472	4.8081943
TSTA3	4.70064309	4.91892942	4.87422138	4.45017253	4.7140735	4.97434471	6.97439985	6.66546094	5.50566062
TSTD2	0.94123531	1.82947994	1.96860292	1.78582833	2.03068002	1.80513275	1.48897565	1.09662199	1.91407013
TTBK2	0.87891638	1.58045036	0.95469985	1.03654161	1.19327926	1.53778511	0.78826733	0.64437474	0.77254662
TTC1	4.32134473	4.82942158	4.87647702	5.17602947	5.01423712	4.93559536	4.24741441	4.82707836	4.83371131
TTC12	1.07081141	1.17163872	0.79629424	1.1094968	1.85292118	0.98956994	1.15471377	1.21319482	1.56240463
TTC14	0.2761681	1.50806992	2.12131846	2.05201693	1.61047036	1.21867232	0.37547669	1.74171922	0.86713856
TTC16	0.03392602	0.03560596	0.04876783	0.08011506	0.10532269	0.04552155	0.02040401	0.02405466	0.05314146
TTC17	2.24106822	2.51155133	2.91129609	2.7984229	2.76860599	2.94419023	2.89593365	3.16762324	3.06299719
TTC19	2.38565546	2.82581806	2.89281572	4.15479728	3.60025358	3.16889099	2.57109381	2.26672546	4.17738692
TTC21A	0.31822186	0.39687464	0.66648078	0.46779523	0.30231381	0.32919773	0.2306193	0.28618041	0.47960256
TTC21B	0.82165819	1.6420776	1.7518433	1.74951573	2.11850925	1.79443968	1.43143144	1.72940376	1.85645734
TTC22	0.40644893	1.30013674	2.12657588	2.15759543	1.07040872	2.41176648	2.47061187	3.14806711	2.31939398
TTC23	1.66239143	1.93697467	2.09869413	1.84236411	1.67221112	2.03596006	1.14413696	1.80564548	1.42936926

TTC25	0.07110836	0.28681293	0.55308162	0.27037397	0.03548274	0.14268186	0.06817524	0.14932095	0.08191958
TTC26	2.28118291	1.24082488	2.12271857	1.60425836	1.56012388	1.42440761	1.49776857	1.18261385	1.59140326
TTC27	2.01355227	2.40910204	2.72961187	3.01617678	2.99237783	3.19714507	2.98678833	3.12260896	3.17200086
TTC3	2.655603	3.68959063	3.13446492	3.76548727	3.90004299	3.4132416	3.28153031	3.83600172	3.83577242
TTC30A	0.40658953	1.05496912	0.96794956	1.19124469	0.75275058	0.94897692	0.41309692	1.65951874	0.81742976
TTC30B	0.76920878	1.40587839	1.41297997	1.53683053	1.0052344	1.18615701	0.89105459	1.68823256	1.53968857
TTC31	2.26858925	2.05613933	2.33791014	2.61011557	2.54046873	2.33746903	2.23411982	2.32205392	3.18115416
TTC32	0.80774575	0.77124057	1.31540554	1.64577272	1.78443489	1.28932473	1.20767027	0.94970333	1.95916714
TTC33	1.63166121	2.85333813	2.81495328	2.65347388	2.17670011	2.543298	2.20012585	2.74001902	2.69490096
TTC37	2.96910998	3.76680483	3.44402181	2.98730564	4.02805863	3.15280473	2.94999265	3.66361029	3.5213441
TTC38	5.09194125	4.15669622	3.3636821	3.18838694	3.74137765	3.58858408	4.26356313	3.25549116	4.91998646
TTC39C	2.42827486	1.45697677	2.32821709	1.77334321	2.09177324	1.95972848	1.75134726	0.93784381	1.16558583
TTC4	0.83244423	0.79099196	0.81611542	1.09094857	1.11427184	0.91397121	0.82865383	0.93846956	0.97916346
TTC5	1.35714419	1.55861992	1.40352532	1.39526765	1.437957	1.15512992	1.15361304	1.69430292	1.47084125
TTC7A	3.40412584	2.51825785	2.99227023	2.3561293	2.69201445	2.59954169	2.97516484	2.50653508	2.58191023
TTC9C	3.14621316	2.67615239	3.09342014	2.67074989	3.36554308	2.67348873	2.84070304	2.93425178	2.97849417
TTF2	1.79342727	1.59640087	1.91028686	2.03801684	2.50134277	2.15195488	2.29595476	1.57650606	2.62130511
TTI1	2.67623914	2.91556103	3.1988121	3.56000961	3.3277831	3.19512014	2.85514563	3.23624849	3.6706065
TTK	1.61768601	1.5022448	1.8102463	2.82042196	3.60742284	2.592898	3.01088543	1.29035965	2.65557167
TTL	1.2388319	1.81376381	2.03676995	2.11482424	1.80724934	2.26449098	2.1784576	1.2968191	1.29480813
TTLL10	0.08958935	0.03142145	0.1338278	0.16241155	0.156342	0.03688131	0.07545248	0.04558365	0.06181624
TTLL12	4.75194114	3.4663053	3.47154353	4.3548293	4.19957546	4.36525255	4.24799698	4.47591204	4.71075846
TTLL3	0.67986681	0.54112522	1.03646432	1.31105504	1.41011204	0.68690578	0.32804696	0.87510643	1.25157679
TTLL4	2.29718901	1.73432273	2.44888718	2.65696198	2.94273158	2.45280532	2.73703585	2.54400983	2.83048256
TTLL5	2.01281553	1.84752522	1.89756625	2.12909621	1.66311733	1.66079315	1.6570651	1.87306301	1.9490369
TTLL6	0.58074313	0.27171242	0.51930055	0.20742043	0.52573332	0.04653063	0.16560853	0.04343333	0.75955852
TTLL7	0.03753225	1.53374917	0.33897	0.73975355	0.13023128	1.42446308	0.10185074	0.2080759	0.07436482
TTN	0.01562321	0.08295739	0.09743587	0.10045544	0.08563757	0.29339273	0.06506856	0.09275254	0.05355304
TTPAL	1.60944741	1.96215765	2.33914187	2.48157611	1.97516874	2.16222195	2.07729363	1.99629673	2.16527258
TTR	0.31513814	0.78170897	0.85971967	0.53938441	0.08037576	0.01836949	0	0.8920388	0.06885707
TTYH1	0.1700448	0.21118349	0.30146302	0.19096096	0.20680065	0.14925414	0.08684463	0.10166217	0.11207712
TTYH2	0.55580196	1.98694665	1.6503715	1.27231702	1.53283342	1.94458687	1.01323833	2.56599231	0.95697063
TTYH3	5.66681774	5.08317033	5.64982647	4.66193425	4.46962646	4.89198963	5.20665065	5.60467612	4.75998311
TUB	0.09443229	1.39480538	0.34653836	0.6532316	0.10621342	0.78998945	0.24423831	0.18599486	0.05863924
TUBA1A	3.95720153	6.15724452	4.84559379	5.35960541	3.65692374	6.2965916	5.026438	3.86295611	4.11158524
TUBA1B	7.4824913	6.67153384	6.66781678	6.98503066	7.52163874	7.5525196	7.93537328	6.80982403	7.56185944
TUBA4A	4.51525166	3.94376181	3.43517485	3.68775389	4.34159672	4.28555182	4.37724948	3.46522827	5.35370571
TUBA8	0.04919948	0.09967863	0.08426897	0.04725995	0.06628357	0.0363166	0.07016734	0.03807219	0.13549194
TUBB	8.12248334	7.53375622	7.68005874	7.91531508	8.08561469	7.9909412	8.22181555	7.24193781	7.78887319
TUBB2A	2.6134447	3.01434455	3.76856062	3.23915048	3.16734336	3.32070247	3.13778236	3.42064151	3.29331709
TUBB2B	0.29898677	1.36896397	0.65440967	0.52817628	0.48255401	1.32298356	0.48517566	0.48937134	2.41994944
TUBB4B	8.03529926	6.16715746	7.20497338	7.05226587	7.01756422	7.33995952	7.39777332	6.86325831	7.64168643
TUBB6	2.93803094	4.50101402	3.37638062	4.16343893	2.45598495	4.46889933	3.27764364	2.43515068	1.98579614
TUBD1	1.34481651	1.38761403	2.62870734	1.48896547	1.75048825	1.64850073	1.38269179	2.08790549	1.5110719
TUBE1	1.27724502	1.34468493	1.10718054	1.63814604	2.18753069	1.54503372	1.32981224	0.85475785	1.61193502
TUBG1	5.00722534	3.93166722	4.17429616	4.5050071	4.79500031	4.58717665	4.7963773	4.27366596	4.47523368
TUBG2	1.68867939	2.16909499	2.23271158	2.83371373	1.74273851	1.75446287	1.24443366	1.79314844	1.64912778
TUBGCP2	3.35645004	2.92394505	2.96091812	2.7598603	3.32503713	3.41923712	3.43421927	3.47831831	3.57617334
TUBGCP3	2.56379315	2.20678096	2.44404822	2.42585602	2.28967792	2.56109591	2.78135065	2.4829923	2.82621927
TUBGCP5	0.828651	1.52757411	1.93362682	2.0157176	2.14747994	1.64114046	1.55916605	1.99455737	1.83857996
TUBGCP6	2.81259904	1.70826812	1.90910252	1.91509624	2.22949553	1.93079241	1.63337667	1.68298306	2.51682235
TUFM	6.60531432	6.3964078	6.18564973	5.99968748	6.09424039	6.34841892	6.94235779	6.3435158	6.58832441
TUFT1	2.89451547	1.49804353	2.98601372	2.46206211	2.64824351	2.46685517	3.00270141	1.8644229	2.75978705
TULP3	2.8969402	3.59140455	3.36139784	3.73253429	3.03759448	3.54589275	3.3529851	3.56277093	3.09544567
TULP4	2.63283935	2.15835553	2.61128476	2.09274343	2.10856236	2.24806989	2.39309167	1.48576365	2.59715206
TUSC1	2.69835371	2.77074954	2.56304304	3.06032545	0.78280907	3.15561686	2.51695436	3.16760379	1.94866943
TUSC2	4.43715817	2.94480053	3.92455859	3.07282226	3.42679296	3.17193259	3.3630722	3.28525037	3.61376984
TUSC3	0.76630112	2.55695245	1.67231827	2.19724379	0.92110019	2.3174566	1.3372828	1.04653317	0.68379453
TUT1	1.79718985	0.98946998	1.36640024	1.1099715	0.97139153	1.08473979	1.10326164	1.21202648	1.30089848

TVP23B	1.30170304	2.14364297	2.93431925	2.85004999	3.09550877	3.05437351	2.85550249	2.68397871	2.63615243
TWF1	4.45535573	4.93261035	4.59185892	4.81294661	5.13717453	5.12837305	5.1688829	4.01936146	5.31171012
TWF2	4.79624378	4.35807111	4.34846557	3.93389854	3.36693937	4.72594256	4.95742856	4.58759356	4.05697013
TWIST1	1.59119363	1.36343393	1.89185196	2.85594855	0.82630654	1.57821765	1.70869236	0.71523263	0.84546241
TWISTNB	3.16201259	3.09560221	2.91430287	3.42660926	3.64671069	3.39868666	3.32199303	2.86196655	3.35410189
TWSG1	3.29868456	4.60744547	4.75595683	4.6060939	3.86589338	4.1930633	4.82226467	4.20732885	3.6569335
TXLNA	4.07307245	3.05928726	3.75141171	3.68772625	3.75363424	3.79752495	3.21497014	2.72327614	3.58922318
TXLNB	0.13385218	0.90114574	0.40633002	0.7258553	0.30616136	0.81056142	0.17067015	0.15112997	0.0865943
TXN	7.78088458	7.06476612	7.78632704	7.49560604	7.54519809	6.92577656	7.32625746	6.98812144	8.46289215
TXN2	6.41863985	4.88619585	5.14749972	4.52461147	4.50823061	4.82881381	4.77068138	4.30001966	5.21980725
TXNDC11	4.96510077	3.907661	4.42112473	3.73364762	4.09614146	3.98091954	4.02724473	4.32428149	3.94616151
TXNDC12	5.28322908	4.84957977	6.08819771	5.13042606	5.03420257	4.83792929	4.8529123	4.93144097	5.00648276
TXNDC15	3.31723919	3.06539759	3.38439464	3.32379479	2.92774114	3.02280315	2.75489256	3.16009504	2.71115801
TXNDC16	1.31763319	1.75510835	2.42313769	2.16026373	2.53875831	1.96805927	1.71576407	2.91093332	2.6401733
TXNDC17	2.80356476	3.49554445	3.36914719	3.95261594	3.90486364	3.66650955	4.16093298	3.32422295	3.84496165
TXNDC5	2.56220173	2.4426222	2.32132707	2.21022357	1.98753986	2.37843792	3.11307571	2.71407787	2.4524863
TXNDC9	3.00595563	3.46003108	3.66287669	3.47904782	3.97727405	3.13021434	2.93407184	3.60458429	3.19050556
TXNIP	5.88623942	8.41378193	7.18626994	7.21305404	7.56722522	7.09070381	7.07058482	6.80482689	7.14689062
TXNL1	2.33480483	3.02551267	2.44593189	3.22299438	3.52548958	3.06735229	3.552669	2.33589303	3.21320523
TXNL4A	3.05593997	2.72460958	2.6970202	3.24848215	3.02556331	3.23481755	4.04898122	2.57493878	3.50741446
TXNL4B	1.5422875	1.69043554	2.61988848	2.63268177	2.94230719	2.2112329	2.08373284	2.39338951	2.72164347
TXNRD1	4.06568163	3.73005705	4.37729093	3.93072819	4.06210729	4.44025787	4.33417712	4.12738014	3.95712747
TXNRD2	1.87163643	1.05493524	1.71454133	1.48970168	1.09130358	1.20418746	1.72826016	1.72327585	1.67592865
TYK2	3.87549125	2.80803601	3.58683594	2.72365209	3.95105739	3.02450616	2.82638229	3.51538543	3.28883972
TYMS	4.46925438	3.80850692	4.73829679	4.75610346	5.18147163	4.55998418	5.55221	3.42072177	4.60092034
TYRO3	1.37950676	1.19555259	1.4271576	1.04604431	1.27781161	1.25747865	1.65905148	0.57201272	1.19664508
TYROBP	7.74959717	7.63904975	8.12279556	6.99542227	8.24390019	7.51046005	7.46795822	5.5679803	5.91739559
TYSND1	2.18410394	1.66820771	2.45790289	1.97488046	1.78841714	2.63629137	1.53179839	2.74587521	2.56890329
TYW1	2.78043412	2.87621254	2.88833304	2.62803682	2.73136557	2.80557494	3.03140569	2.77226787	2.97097248
TYW3	1.90583881	2.41472804	2.64764426	3.07837473	2.88927956	2.98313167	2.02951477	3.24380913	2.82340448
TYW5	0.64004382	0.8354625	1.20747454	1.2960299	1.26727984	1.03788444	0.941573	0.94056372	1.06211257
U2AF1	0.15826209	0.05528671	0.03972973	0.14229339	0.14592225	0.19892927	0.10413131	0.14309058	0.17816853
U2AF2	6.40520117	3.84067135	4.91885411	4.17384879	5.10185153	4.39960044	4.45058661	4.9287278	5.27110524
U2SURP	2.89648095	2.80382997	3.49266593	3.79160974	4.33219603	2.87030771	3.39088199	3.47678028	3.90123851
UACA	1.71610693	2.74321518	2.86795258	3.37648114	3.61797736	2.68191635	2.77886321	1.99907476	2.45774556
UAP1	3.28921788	3.98246354	4.2719985	4.23448164	4.31792916	4.17423565	5.29660079	5.14100376	4.32138443
UAP1L1	2.46557973	1.29697009	3.60264527	2.22936873	1.54044193	2.02540814	2.20637845	1.42265368	1.98305415
UBA1	5.87589854	5.57681993	5.3330013	5.63204027	6.46202208	5.9028192	5.74703027	5.4515462	6.31215883
UBA2	4.03640345	4.21452996	4.74829302	4.70367703	5.50313901	4.49994331	4.19808206	4.74066619	4.52311935
UBA3	2.68539355	4.23887872	4.30074317	4.72341367	4.54420749	4.17654732	4.32483771	3.73280675	4.23980365
UBA5	2.28406375	2.14571879	2.23834796	2.75816035	2.84577296	2.39865234	2.46198506	2.59829954	2.44526709
UBA52	7.62821327	6.40773521	6.58381489	6.87819172	7.29892267	6.53434114	6.47508621	6.49036344	6.99505529
UBA6	1.50175639	2.22400904	2.66741608	2.86131932	3.45048578	3.0401875	2.49542052	2.50111486	3.02479893
UBA7	3.46222474	3.60921349	3.71225012	2.76672211	4.25002416	4.00898116	2.20266348	2.06243455	4.1477904
UBAC1	3.97774562	3.16211266	3.8854127	3.39351168	3.59081441	3.64494878	3.60736682	3.83254183	3.69390937
UBAC2	4.6441417	4.05706784	4.16592295	3.91070554	3.65959148	3.89248927	4.28678663	3.9551665	4.6825186
UBALD1	3.61003494	3.38128637	2.39819331	2.78762087	2.80709463	3.21196228	2.97471599	3.18340646	3.15730286
UBALD2	7.39634463	4.42741354	5.89874145	4.26940213	4.72531706	4.78310295	4.57725125	5.76628043	5.51054394
UBAP1	4.95676543	4.52926591	4.99242939	4.56588129	4.46363186	4.68394361	4.36422781	4.502808	4.65151931
UBAP2	2.82301907	2.64388774	3.1825239	2.728457	3.12886421	2.60995922	2.75672231	2.75213701	3.28475959
UBAP2L	4.56576108	4.09050365	4.4467286	4.50170612	4.69430904	4.45694658	4.62299259	5.06183499	4.71254408
UBASH3B	0.77574308	1.50601792	2.17829233	2.18337682	2.22073697	2.11920816	1.98803761	1.34895385	1.08501685
UBB	8.56945568	8.34827911	8.98153228	8.44799811	8.93742469	8.406366	8.759785	7.96638031	8.5712792
UBD	4.33600629	1.118649	6.85162959	2.66451114	7.81753995	6.41092782	1.41650559	6.51602256	3.88670228
UBE2A	3.47222597	3.24781772	4.45197164	3.62573278	4.14382442	3.38284226	3.31857521	3.35717708	3.43266559
UBE2D2	4.93254849	4.37515363	4.82450817	4.85169536	4.80746337	4.62416546	4.43856287	4.48597504	4.78981025
UBE2D4	3.58802962	2.79447445	2.78367742	2.66186985	2.14433019	2.37925389	2.37583014	1.53531632	2.08023569
UBE2E1	3.99978422	3.86966301	4.22713834	4.38739693	4.14785245	4.18192681	4.00741632	4.19226894	4.122936
UBE2E2	1.63525733	2.90032155	2.69591614	2.74084666	2.63781434	3.35882529	1.79152167	1.16547294	1.25278379

UBE2E3	4.56706541	4.13564513	4.02225841	4.52050718	4.18135298	4.49053266	4.90701339	4.3134818	3.79643077
UBE2G1	3.12973083	3.61809751	4.08293174	4.28527614	4.29757136	4.28055794	3.89036709	3.15209966	3.89824614
UBE2G2	2.29118288	3.05167538	3.49714032	3.37046856	3.68165449	3.29840134	2.7893594	2.72209016	3.56794996
UBE2H	4.11388416	4.73584825	4.47007962	4.61483349	3.7280494	4.46447357	4.71278993	4.80980909	3.92450174
UBE2J1	4.23228374	4.61843368	4.72257511	5.3119222	5.34219942	5.1634126	5.49618291	3.87853723	5.21573564
UBE2K	3.96643529	3.956271	4.46918055	4.73360825	4.62337516	4.68541515	4.3775859	4.3825879	4.68859304
UBE2L3	5.69744129	4.95503584	4.79975465	4.92373126	5.44167755	4.99568519	4.647641	3.83672576	5.31961661
UBE2L6	6.29965443	5.92700146	5.5204137	5.18680109	7.04084072	6.74218653	4.66166627	5.15963965	6.0067021
UBE2N	4.24212798	4.02830265	4.41889216	4.50141235	4.93641565	4.23942232	4.06537757	4.0612623	4.5005856
UBE2O	3.35379458	1.90258128	2.75606115	2.30280687	2.61733166	2.68987753	2.29266847	3.09245087	3.13050546
UBE2Q1	4.63359856	3.6446704	4.4204147	4.16984125	4.30887719	4.02857772	4.46098632	4.78892403	4.06952667
UBE2Q2	2.6551064	3.9883966	4.52464798	4.43462481	2.71172482	4.44287426	3.92257308	3.61267299	3.76254327
UBE2T	4.11035241	3.11485457	4.44991951	4.92402185	5.33245802	4.31114439	5.22629064	4.30781928	4.83816095
UBE2V2	2.36324907	3.20692045	3.17316337	3.86066352	3.06178466	3.41808109	3.56546356	2.76013862	3.10670538
UBE2W	1.55979831	2.32477101	2.83140674	2.95995177	2.49690361	2.72951925	2.48364964	2.25634558	2.18159579
UBE2Z	5.08535901	3.87282255	4.42731855	4.55754143	5.08029673	4.78117145	4.11746357	4.48228695	4.47037968
UBE3B	2.25493603	2.27474356	2.32474626	2.25742466	2.39167865	2.41626797	2.16317704	2.89061314	2.38885992
UBE3D	0.48443274	0.70409784	0.78181563	0.76690788	1.10189466	0.67502139	0.6088378	0.64188502	1.06798568
UBE4A	2.38561137	2.99633397	2.85356935	3.60483329	3.79970886	3.08802225	3.29486485	2.72140661	3.51109948
UBE4B	2.58384015	2.88061865	2.47110029	3.19311029	3.24957493	3.10516822	2.90924589	2.36137453	3.18698246
UBFD1	2.63009897	3.17551021	2.602026	2.8575241	2.98070422	2.92175146	3.24318896	3.24509085	2.96721859
UBIAD1	2.74449138	2.3294271	2.15534924	2.59882633	2.37634864	2.87530519	2.80402441	2.27539065	2.99146133
UBL4A	4.7585463	3.86305418	4.21008663	3.91715503	4.51538967	3.92412128	3.97235581	4.0924998	4.18838158
UBL5	6.26250995	4.95263098	5.60224037	5.07290333	5.58136229	4.67431246	4.58165641	5.11252372	4.99623921
UBL7	5.01969252	4.27514043	4.8005195	4.33973694	4.71177965	4.74690245	4.17626118	3.98640188	4.65599559
UBLCP1	3.19772884	3.87422683	4.24572487	4.61795079	4.79750689	3.98027995	3.33356693	3.57869219	3.45663829
UBOX5	2.02918367	1.82288531	2.13539909	1.71120712	1.74633001	1.75710092	1.38502209	1.17743401	2.66549959
UBP1	3.35288635	3.16491769	2.94590987	3.61366767	3.82515011	3.4495091	3.51185936	3.34507898	3.66199366
UBQLN1	4.04661947	4.0437435	4.79142615	4.68336578	4.94258116	4.69337558	4.27595138	4.85844528	4.70369906
UBQLN2	3.67411212	4.50818427	3.57742152	4.07966274	4.62581415	4.08918543	3.93103204	4.17605348	4.02597476
UBQLN4	3.65329963	3.33849682	3.76936379	3.9914802	3.83946075	4.2072725	4.89690439	5.09278887	4.33325251
UBQLNL	0.06168843	0.22516288	0.05582357	0.1352556	0.00967423	0.09520679	0.02802203	0.04189602	0.07267839
UBR1	1.47567888	2.81613345	2.66328666	2.64700535	2.77675904	2.58470603	2.39136554	2.37315136	2.61570698
UBR2	2.01455213	2.98621797	3.08369111	3.11069123	3.62638953	3.23503854	3.4823881	3.64826511	3.48280723
UBR4	2.6476171	2.86032364	2.43871154	2.91883309	3.23142971	2.97432525	2.84050641	2.83579038	3.11038762
UBR5	2.32642796	3.2515371	3.33062469	3.19844331	3.62932194	3.52583236	3.20443821	3.23094527	3.36620935
UBR7	3.10738334	3.54137235	3.5332968	3.5635619	3.87528395	3.64795656	3.72682143	4.12990515	3.63167728
UBTD1	3.46267119	3.07843709	2.993555	2.86938998	1.73384776	3.46440415	3.01317515	2.22860575	1.9789751
UBTD2	3.0861113	3.74849716	2.89014169	3.94039559	3.28330377	3.55900713	3.52523569	3.28514769	3.71015276
UBTF	4.35703621	3.70608829	3.63406551	3.89566232	3.76933768	3.97644327	3.81938723	4.11800485	4.16613132
UBXN1	5.33838771	3.67625952	4.24341736	3.63746539	3.5485141	4.26527751	3.83616177	4.51770602	4.22330237
UBXN11	3.22010382	1.14533589	1.72786311	2.37978904	2.17104856	1.41111434	1.47055461	1.07967225	2.01239737
UBXN2A	1.64710917	2.12466996	1.93888733	2.58757493	1.80933224	2.01450788	2.01520342	1.32391857	2.01914591
UBXN4	4.52574469	4.86780593	5.70517755	5.49242908	5.91799862	5.22604166	5.06011795	5.28268116	5.64758527
UBXN6	4.44710216	3.32273865	3.27872066	3.00808993	2.94594643	3.39012847	3.34917046	3.56246125	3.46714055
UBXN8	1.30359425	2.2137633	1.73624956	2.49582998	2.15353127	2.17446696	3.13645181	2.48862874	1.61337791
UCHL1	0.68602945	2.9931828	1.3007505	2.6704633	0.90001744	2.41130236	1.20503761	0.51264104	0.86429562
UCHL3	3.15435721	3.0765987	2.8127167	3.1692259	3.31519479	2.78565144	3.47004356	2.42625875	4.02987242
UCHL5	1.86826991	2.1487563	2.32517046	2.75288457	2.72454898	2.91232124	3.47574651	2.68223934	3.00126984
UCK1	3.38343532	2.84719475	3.43443824	2.83025193	2.82355157	3.06322603	2.47862865	3.14259523	2.44909799
UCK2	2.94496403	2.27762324	3.13333678	2.81307693	2.97364037	3.1283581	3.54361029	3.43427699	3.16096863
UCKL1	3.72690956	2.67715784	4.02652989	3.63974895	3.32017964	3.18430518	3.51923753	3.56891583	4.65405125
UCN	0.32677435	0.21885563	0.2432477	0.58078719	0.64863289	0.57565656	0.31877955	0.61034218	0.6942054
UCN3	0	0.57955465	0.33176335	0.01810703	0.03694241	0.01111599	0	0	0
UCP2	6.87717426	5.48566866	5.84505901	4.57611483	5.80770963	5.68401984	5.44717968	4.3191415	5.40272384
UCP3	0.26165984	0.12996398	0.41350729	0.19925258	0.29147132	0.22814021	0.08620074	0.1756857	0.20336849
UEVLD	1.74013319	3.15678927	3.276519	3.09159299	3.31235579	3.19017141	3.01674705	2.99456781	2.87046441
UFC1	5.2789486	4.92196971	5.32501147	4.98025812	5.02484164	4.90906278	5.92178806	5.66819647	5.47316374
UFL1	3.17310497	3.29163815	3.32694718	3.74141217	4.28823458	3.51435781	4.11113049	3.29326365	4.01614189

UFM1	4.10572998	3.70408895	4.08689049	4.42298281	4.6073561	4.03962489	4.16069612	4.40911667	4.72111043
UFSP1	0.56744842	1.15078846	0.92438329	0.75253895	0.71423883	0.84914623	0.96827539	1.16445477	1.49899638
UFSP2	2.37253267	3.7305498	2.70468634	3.57482378	3.19429004	3.39488401	3.2956789	3.22981404	3.36940778
UGCG	4.26348192	3.64465606	4.75666891	4.11942813	4.22081616	4.38549417	2.92522651	3.13640717	2.99694558
UGDH	3.39473952	3.44222338	4.39951497	3.70967843	4.74146467	4.16759843	3.99445609	3.88699823	4.23784711
UGGT2	1.36432286	1.46246933	2.32031781	2.0368126	1.87297559	1.61147502	2.03813719	1.67710911	1.86840787
UGP2	3.22745191	4.03120901	3.88365359	4.06617103	4.25511813	4.16752341	4.15271039	4.23872898	3.78511047
UGT2B15	0.12728745	1.30996175	1.47642409	1.57451919	0.40013091	0.61309531	0.64703496	4.9255867	1.60232353
UGT8	0.08288753	3.29606851	3.50470971	5.12735459	3.95258116	3.25354437	3.43240798	4.78759428	5.06838598
UHMK1	2.86148251	3.65513815	4.17286293	4.34455644	5.23472151	4.42666316	4.85926867	4.74967243	3.86677982
UHRF1	3.24331474	1.15276861	1.64376714	1.8330146	2.45647033	2.0926448	2.20545156	1.82820413	2.50762536
UHRF1BP1L	1.42225562	2.91780251	2.51529339	2.92160366	2.90673582	3.05714723	2.49214593	2.79850645	2.4198725
UHRF2	1.49618773	1.80330165	1.90647181	2.36113311	2.38763825	2.08296424	1.60326287	3.04576802	2.34492426
UIMC1	2.33816427	2.04816787	2.49684497	2.54960848	2.69617158	2.36683251	1.91201557	2.48756045	2.45057197
ULBP3	0.1735659	0.86406182	1.31976637	2.38786938	0.42924561	1.77953967	2.05943648	1.38744996	2.28809137
ULK1	2.23131649	2.39879863	2.38659469	2.34308064	1.8682892	2.49841047	2.06288325	2.83442441	2.68005182
ULK2	0.20983314	1.82506077	1.57004183	1.96438327	0.80817965	1.75140982	0.97776073	1.50882687	1.35660997
ULK3	3.32229482	2.45028828	3.67204539	3.0497229	2.81592045	2.73205425	2.38856009	2.58921686	3.79553147
UMPS	2.89780615	2.01484396	2.46778994	2.5244012	3.10940012	2.59384232	2.98030129	2.5454731	2.74514074
UNC119	2.12748252	1.96479477	2.68265931	2.28005229	2.02114248	1.91983395	2.04982195	0.92164399	2.57123638
UNC119B	2.61935099	2.15865189	2.41173345	2.62586836	2.16090053	2.28230894	2.60255063	2.82333535	2.59099139
UNC13A	0.05110196	0.31643814	0.40666024	1.15374798	0.16271244	0.09028838	0.04219195	0.05289907	0.04044156
UNC13B	3.24259944	2.93249653	3.39881805	3.25302448	2.87813937	3.16981624	3.46300611	3.83628124	3.66219987
UNC13C	0.02417067	0.00773565	0.16614945	0.0193674	0.00166744	0.00298214	0.00162027	0.00520966	0.00142286
UNC13D	1.88301755	1.60858578	2.68566115	2.83295478	2.22829892	2.46590233	2.37576249	2.9475685	2.43033751
UNC50	3.92973944	3.78139884	4.49759761	3.96145872	4.20800486	3.77642699	3.84657938	3.44886728	3.72189571
UNC5A	0.22426199	0.32476342	0.19741497	1.14683922	1.08528964	0.87933883	0.21373746	1.36876574	0.97549716
UNC5B	2.68773968	2.0741048	3.00535279	2.92722917	2.04859595	3.25104898	3.26018211	2.43199234	1.72420822
UNC79	0.03453417	0.1337432	0.30260037	0.08317859	0.02137213	0.09824149	0.04632228	0.08076602	0.0422422
UNC93A	1.5499599	0.78556102	3.98573636	0.04780679	1.06334119	0.02459593	0.13125588	0.02578999	3.47827243
UNC93B1	3.81988689	4.11176757	4.52553159	4.09411754	3.41036054	4.49912961	4.95640872	4.62090789	3.8209489
UNG	3.71305916	3.30011092	4.04121059	4.06526413	2.97056771	4.06861088	4.15434008	4.01978199	4.50756141
UNK	3.58682124	2.43348318	3.41554009	2.76352009	2.83672686	2.62747965	2.35313593	3.20100879	2.56055584
UNKL	0.98618489	1.22771023	0.94883264	0.88548715	0.83253797	1.15456333	1.01653424	1.28199055	1.25757127
UPB1	0.02130226	0.03641388	0.34025647	0.09648362	0.04554513	0.04464093	0.02546643	0.08433423	0.01403196
UPF1	4.21593745	3.25560569	3.32591879	3.38268812	3.89384146	3.71869504	3.65008923	4.33092931	3.93114542
UPF3A	2.43372262	1.82456727	2.52336456	1.91262316	2.11249407	1.64647666	2.05369808	2.08477281	2.97116529
UPF3B	2.09550036	2.12675838	3.09466505	3.11017015	3.63365842	2.29550411	2.06298818	2.27590103	3.00447548
UPK1A	0.1029575	0.5415092	0.09329408	0.40245813	0.03250324	0.10399181	0.07772252	0.02042084	0.0277789
UPK2	0.12613034	0.27420716	0.1143769	0.36487553	0.34180974	0.08235533	0.13186298	0.04992751	0.06767107
UPRT	2.10122333	2.68238575	2.86256077	2.38465669	3.03173849	2.30241416	1.87198907	2.74797761	2.59313352
UQCC1	2.41164049	2.45823429	2.93182005	2.56689719	2.57278291	2.43843212	1.18752533	1.92851647	3.21076841
UQCC2	3.04465623	2.35083398	2.96988641	2.40919848	2.57090974	2.18120806	2.1094455	2.78684883	2.466787
UQCR10	7.77684821	6.17049803	6.56651388	5.80285361	6.53496227	5.81847606	6.05991724	5.34250488	6.59015934
UQCR11	6.26209364	4.63775448	5.05824328	4.5149865	4.95400934	4.57842448	4.4908323	4.88168185	5.08858623
UQCRB	3.85804618	3.93293831	3.75751972	4.90568126	4.41412974	4.13374846	4.34149592	4.161168	3.89184258
UQCRC1	6.56649663	5.6181856	6.15299577	5.64126104	5.90812106	5.97407119	6.86276901	6.25816291	6.55664621
UQCRC2	4.93935097	5.6079153	5.601575	5.1567358	5.56873809	5.12932368	5.754275	5.16836027	5.82093013
UQCRFS1	4.64576892	4.514027	4.61548251	4.68141754	5.68393407	5.09673606	4.80901044	5.01668011	5.53340845
UQCRH	6.87314207	5.83130014	6.76938779	6.02959694	6.65180365	6.12832976	7.05451476	6.68742355	6.94589516
UQCRQ	6.8290827	5.85956357	6.12411838	5.46908035	5.75145562	5.41077483	5.72236566	6.28817666	6.42962759
URB2	2.02011424	1.73206517	1.72742488	2.01733641	2.43109557	2.45071287	3.00140477	2.54270679	1.90789693
URGCP	3.1093835	2.36111871	2.31781681	2.26466594	2.65870048	2.60731617	2.50182336	2.50061899	2.58725419
URI1	2.99057443	3.73438751	3.87920169	4.12011517	4.44792276	3.78516352	3.7752111	3.55403577	4.0408038
URM1	4.9187968	3.49399704	4.62746404	3.50697888	3.5251296	3.74199001	3.4543333	3.16973841	4.36468404
UROD	4.01966455	3.48293493	3.89250326	3.29624849	3.17600869	3.49211403	2.96570987	3.61979462	3.51637969
UROS	2.48772891	2.54811817	2.96290825	2.12755254	2.38510594	2.28111006	1.84051751	2.44035544	2.35830293
USB1	3.44097499	2.55564932	3.29402443	2.68886532	3.03302016	2.8290052	3.03135506	3.23688822	2.61887462
USE1	2.69053656	2.91257405	2.90717172	2.79911238	2.273386	2.62429976	2.32722302	2.356492	2.98061756

USF2	4.67309444	3.89441743	4.20907193	4.18273868	4.43610148	4.27606127	4.20449318	4.17160328	4.18704363
USH1C	5.13894541	3.07850562	3.00072433	3.85853569	4.19662604	2.07381845	2.52632636	3.21645082	4.83586559
USO1	3.57646098	4.56382867	4.08163948	5.12649116	5.3492221	5.10316817	4.94788104	5.1259922	4.85241444
USP10	3.50363467	4.0050688	3.96438443	4.24569543	4.59389505	4.49206949	4.17484592	4.19698571	4.73481835
USP11	3.77159306	4.09764227	3.73221267	3.99563716	4.01256474	4.1090094	3.32319742	3.6343518	2.90142913
USP13	1.73233078	1.64743684	2.36810231	1.96682675	1.93330323	1.63811249	2.23535531	2.24171307	1.37590566
USP14	3.3838927	3.73041614	4.06301865	3.95280643	4.53708817	4.36635199	4.72311199	4.03252646	4.18970752
USP15	1.02265066	2.02903435	2.48706547	2.29926086	2.82564783	2.43447904	1.92348584	2.19283842	2.02854947
USP2	0.16667378	1.40691611	0.51634377	0.63325769	0.15910902	0.98230898	0.34093951	0.28297054	0.59372275
USP21	3.33541919	2.40960486	3.22339541	2.82458642	2.52887897	2.60226718	3.12592251	3.39245195	3.05354041
USP26	0	0.02000601	0.23942861	0	0.01615887	0.00322696	0	0.04010312	0
USP28	2.37993525	2.63154997	2.44418753	2.93178646	3.64632309	2.86153587	3.04165598	2.17327868	2.87732431
USP30	1.7803778	2.25664739	1.97793734	1.68684669	1.60389	1.83532495	1.71080128	2.64653627	2.55950962
USP31	0.54217871	1.31165979	1.2364806	1.32459112	1.59589938	1.60400693	1.38722206	1.17366174	1.35377861
USP32	2.39080266	2.45858347	2.75172992	2.60484013	2.79492904	2.98140778	2.47684016	3.27320892	2.4741238
USP33	2.53923328	3.15314044	3.26149732	3.17740552	3.85524155	3.36914734	2.53554889	3.19757597	3.34910329
USP34	1.65432972	2.77430092	2.47910069	2.73211103	2.96573178	2.97913335	2.80208418	3.11063238	2.59672679
USP37	0.70803932	1.32459129	1.42734343	1.42418467	1.72831572	1.69910649	1.49810272	1.8841635	1.40957905
USP38	2.93430791	2.93310607	2.85158319	2.94653059	3.38279435	3.01119426	2.89557108	3.19056545	3.11518901
USP39	3.70093149	3.58658686	4.13463502	4.29106812	4.19957171	4.09847804	4.19731001	3.63962493	4.30121365
USP4	2.79904144	3.23010547	3.4031195	3.40322724	3.09687473	3.38336632	3.40431344	3.42384479	3.18141863
USP40	1.94578692	2.37701176	2.0304622	2.32042565	2.25824115	1.86865632	1.98750487	2.23396004	2.24960332
USP42	2.6742615	1.99392319	2.46502593	1.99702139	2.60432717	2.39477169	2.29912137	2.20212794	2.46549823
USP43	1.80416812	0.5763629	1.49681151	1.23003718	1.88359522	1.65707821	1.47886125	1.01032531	2.24373469
USP45	1.42320911	1.30880522	1.30653699	1.88291433	2.10094718	1.53371929	1.47246133	0.73039084	1.66469959
USP47	2.06694245	3.05465108	2.58697502	3.14273352	3.20112961	3.0946316	3.09387595	3.16247855	3.03558163
USP49	0.53940602	0.65538175	1.00794628	0.86407819	0.86569652	0.61864782	0.38669329	0.64060947	0.64651182
USP5	4.72081758	4.04908841	4.15424539	4.083955	4.17181723	4.38178239	4.44850203	4.25923869	4.79084128
USP51	0.22185637	1.12131568	0.46571137	0.7042939	0.32682675	0.60427535	0.24941191	0.20469347	0.08851341
USP53	1.17086047	2.04955559	2.90717981	2.50426495	1.95242225	1.85933335	1.79445413	3.42070711	2.48491858
USP54	1.21858568	2.07380132	3.40100603	2.42936596	2.9628135	2.48437342	2.73826436	2.88965956	2.81231663
USP6NL	1.49915457	1.63694463	1.55856346	1.93699076	2.35433105	1.94196753	1.81220429	2.12431013	2.04852824
USP7	3.57922202	4.27819679	3.93654251	4.04074428	4.55607679	4.04644882	4.03919183	4.317685	4.60245549
USP8	1.43497784	2.08612728	2.23856627	2.3354013	2.5245967	2.03178796	1.84281745	1.58619431	1.56451123
USP9X	2.95430281	4.61427301	3.80030811	3.9781684	4.86110037	3.90635114	3.85457443	4.1553148	4.93820851
USP9Y	0.21922758	1.6075995	1.07676321	0.00582573	0.01984304	2.12164426	0	1.62147558	0.00510535
USPL1	2.30547251	2.50403947	2.51202796	2.97468048	2.79103703	2.52639469	2.25692496	2.46901939	3.32839487
UST	0.17998107	1.71789439	0.94405712	1.50931226	1.98687081	1.49805354	3.01885343	0.6247806	1.06035444
UTP14A	2.98148543	2.47235784	4.15628325	3.09782477	3.81982563	2.87503191	2.64647849	3.13899575	3.55229277
UTP15	1.49691884	1.54514326	2.67203374	2.32244021	2.51592889	2.05607	1.8249835	1.73707514	2.33151496
UTP20	1.6609589	1.60598286	2.05872767	2.2063628	2.51317469	1.96350288	1.85793966	2.90183295	2.44894487
UTP23	1.73465691	2.58310431	2.8734104	2.73341548	2.23748865	2.63293326	2.37394012	2.40371504	2.47272029
UTP6	2.44811586	2.29645109	3.11477324	2.98422051	3.40834089	2.78056922	2.82310934	2.98817756	3.02712951
UTRN	2.38900604	3.43364363	2.80138076	2.7469673	3.22696873	3.34424466	2.0013919	3.22305949	2.52443777
UTS2	0.04013443	1.08061183	0.4932454	0.32638538	0.2836011	0.85062061	0.04791929	0.32200736	0.19947483
UTS2B	0.0188093	0.14435388	0.366438	0.09876829	0.03457206	0.10398233	0.07184596	0.07465267	0.11013081
UVRAG	2.104634	2.59320829	2.73805858	2.42843568	2.77364156	2.41046924	2.05462136	2.52013649	2.13170078
UVSSA	1.24363255	0.66522719	0.99827376	1.14389182	1.26971677	1.16650752	0.97782627	1.19580225	1.36577076
UXT	6.33099415	5.90177346	5.72087916	5.78175429	6.67611884	5.42260288	5.2472957	5.46363158	6.03815791
VAC14	3.90849151	2.72020955	3.30491138	2.77593577	3.50817646	2.81843851	3.29543118	2.96237266	3.30461005
VAMP1	1.13259176	0.80549525	1.7789315	1.95244913	2.01228861	1.17799841	0.92256461	1.05070687	1.80579161
VAMP2	3.92380205	3.75483833	3.90964282	3.94876087	3.80837135	3.69314844	3.22815289	2.83719784	4.04342092
VAMP3	4.71740153	5.28129355	5.2692538	5.28822894	5.95662919	5.70702337	5.21059806	4.45202439	5.73778063
VAMP4	1.50397803	2.23423206	2.42083912	2.47645484	2.15605729	2.37027358	2.34570729	2.58978964	1.82600495
VAMP5	5.34027475	5.13189506	4.5218093	4.3734217	5.09895056	5.32058821	3.76862988	2.35760193	3.78067418
VAMP7	4.12851497	4.51813956	4.33004731	4.53444264	5.27407514	4.41208009	4.20216804	4.22203176	3.86908961
VAMP8	6.98319417	5.29032387	7.05742106	5.76344509	6.90987155	6.44187677	6.19683512	6.58146168	5.98080461
VANGL1	1.62534783	2.41252167	2.84173255	2.84964004	2.44659566	2.68838709	2.27441799	2.89656323	2.45479074
VANGL2	0.30077076	2.44536801	2.31166534	1.0054403	0.31253295	1.04039889	0.91343273	0.33217195	1.44135658

VAPA	3.46589646	3.82727421	4.37044045	4.00507707	4.58221968	4.08826222	4.32918379	3.97047233	3.5882545
VAPB	2.86988344	2.91949054	3.62588118	3.28199495	2.87107084	2.94094905	3.24165531	2.89292557	3.63975824
VARS	4.08021087	3.32217931	4.07104322	3.95330806	3.93503781	4.29649136	4.81952372	4.82275981	3.95939067
VARS2	2.36972429	1.47666363	2.31094567	1.69152155	1.91548489	2.15091576	1.62123637	2.54010715	2.0371324
VASH1	2.67203243	2.00797184	2.38759574	1.80685655	2.16737936	2.4678986	1.91453758	1.51969814	1.14605382
VASH2	0.2052874	1.09133351	0.68582575	1.01496039	0.31009477	0.50988827	0.51035039	0.25315752	0.63043718
VASN	2.67030565	3.69869934	2.81738943	2.75076273	1.66380882	3.4919711	3.13806365	2.89866717	1.64810345
VAT1	5.80374381	5.4938616	5.07873148	6.13401055	4.45027966	6.01302112	5.71243184	5.52834398	4.10710777
VAT1L	0.32936956	1.03020023	1.28579857	0.78076925	0.29803195	0.89649539	1.05032165	0.89088653	0.54496611
VAV1	2.02466078	1.84727288	2.59166371	2.06802673	2.63858425	2.77868356	1.8378416	1.8079221	1.34258283
VAV2	3.28468091	3.02831204	3.64522235	3.21859715	2.74525599	3.17688327	2.93207914	3.78266984	3.44554243
VAV3	3.10696667	2.53161051	1.38624143	2.08024394	1.35631182	1.5991751	2.17332164	0.39324277	0.70814387
VAX2	0.12180675	0.32458994	0.13154968	0.68510787	0.03862678	0.17565218	1.25356499	0.0242872	0.0811809
VBP1	4.10840908	4.23572568	4.31088813	4.53935787	4.98397413	4.34434378	4.27213711	3.91858451	4.16803828
VCAM1	1.99795073	3.63458692	2.70783889	3.83838023	5.51812208	3.99088737	2.37429128	2.00415274	1.19166769
VCAN	3.04813344	6.04947921	3.63884321	5.39329865	3.48330665	5.351377	4.90986451	3.5795194	2.4019046
VCL	4.2437549	5.50448867	4.47026175	5.14572369	5.07757497	5.39533086	4.9107972	4.69917503	4.25488288
VCPIP1	1.58603774	2.35171699	2.09713201	2.56357881	2.49958644	2.67044673	2.69907721	2.23081284	2.3372929
VCPKMT	1.83559958	1.31774248	2.62498848	2.73960153	2.32367719	1.75906843	1.61718181	1.62511747	1.93983715
VDAC1	7.09150341	6.18940732	6.36967998	6.82767736	7.030431	6.46506866	6.30412081	6.51355158	6.8519362
VDAC3	4.58216483	4.98071923	4.45860268	5.80592211	5.88683415	5.46064327	5.81042465	5.07770196	5.44262346
VDR	3.03312936	3.36251378	2.5172996	3.52525632	3.1231015	3.3262226	3.88022508	3.68682438	5.92810922
VEGFA	4.78389419	1.80930399	4.68549945	3.23809476	4.13659722	3.26031935	3.34311033	3.70439546	3.68198934
VEGFB	5.37050275	5.28422684	5.69998634	4.99993257	3.85571739	5.47420615	4.65169347	5.19795672	4.78119374
VEGFC	1.53077486	2.68731976	2.26784816	3.19145907	1.9444102	2.98882738	2.21505194	1.39475215	1.07987817
VENTX	0.16541973	0.30870638	0.40709842	0.3097533	0.10412799	0.25254905	0.20244497	0.07978373	0.13796615
VEZF1	4.01050484	4.24174464	4.61643529	4.09513339	3.82613796	4.34731437	3.71649359	4.8714365	4.11068258
VEZT	2.74545852	2.96827151	3.29847545	3.79187609	3.63271425	3.08620345	2.95486939	3.74132141	3.11014938
VGLL4	3.33969953	2.88609967	2.90260396	3.24666439	2.28697633	3.18414634	3.28657629	3.3145167	3.69077995
VIL1	2.61393347	4.12412486	2.92775674	3.20334427	3.09849775	0.94321461	2.60992166	0.2661647	6.16953392
VILL	3.20949055	1.99139149	2.09107457	3.45422897	2.59952643	3.49229854	2.72030504	4.0697265	1.89406192
VIM	6.31218295	8.23512887	7.03103722	8.08069195	6.83298869	7.85222745	7.13250055	6.01357537	5.82969732
VIP	0.02282664	2.7868535	1.01823979	0.5165858	0.10915666	2.9333867	0	0.01762749	0.07079782
VIPAS39	2.6169188	3.11629597	3.11450411	3.25520124	2.97082377	3.05786913	2.67207734	2.89222769	3.10382567
VIPR1	0.10469247	2.22051075	1.28159939	0.82015473	0.22998277	0.20268813	0.9742126	1.0455068	2.76694406
VIPR2	0	0.98616733	0.35962361	0.68920028	0.05069217	0.97854404	0.02895174	0.07085812	0.02184469
VIT	0.03876817	1.44980242	0.53861413	0.4168208	0.02994538	0.36760639	0.05763688	0.33621007	0.0457393
VKORC1	4.43474537	4.54200495	3.57737511	4.03484114	3.14545485	3.67546708	3.58347636	3.50344117	3.2557015
VKORC1L1	3.96176061	3.38931314	4.11874759	3.61569708	3.46269173	3.72543714	4.39777118	3.64470642	3.48995151
VLDLR	0.17265857	2.12300734	1.60046418	1.62088727	0.19111061	1.78903936	2.02273994	1.10917995	0.10290711
VMA21	3.35564013	3.15732857	3.35986175	3.93084403	4.37889135	3.30613548	2.85585813	2.98787422	3.0341357
VMO1	2.29305805	4.02828791	3.02442844	1.64744115	1.65590326	1.63399132	3.80671989	1.3378533	1.34361165
VMP1	4.29219168	4.46803333	5.87748147	5.64266366	5.38885963	4.92914745	4.42248531	5.15481524	5.44715368
VNN1	2.23284692	3.18757426	4.07344593	1.25447016	3.81425652	0.89811179	2.92078598	3.99173679	4.90350521
VNN2	1.85950487	2.87182959	3.43125891	2.33530196	3.49093922	3.22404634	2.9126509	1.84782458	2.34479244
VOPP1	4.329246	3.40499039	3.68796692	2.83873227	3.16506601	3.46031759	3.42644888	2.968741	2.94089163
VPREB1	0	0	0.10100333	0.0286739	0	0.1190982	0	0	0
VPREB3	1.05431308	2.96494642	2.05015457	0.84403557	1.29524316	0.85315366	0.13640068	0.68394607	0.30969814
VPS11	3.21603217	3.31162732	2.90998408	2.92680656	3.19563907	3.18413835	3.00212488	2.70306454	3.24030729
VPS13C	2.1676491	2.67452939	2.94050162	2.21404696	3.52858699	2.61544381	2.06023735	2.02731361	3.1408008
VPS13D	1.87215453	2.60037086	1.60099292	1.9731605	2.68404753	2.54492781	2.05967114	2.32121741	1.9521484
VPS16	3.05166037	2.70553335	3.32669403	3.04186542	3.0238761	2.79845962	2.62724673	2.52416737	3.26674289
VPS18	4.16501895	2.94029105	3.27318969	3.02867499	3.17519029	3.41009244	3.23198745	2.77772318	3.02084169
VPS25	5.7229019	4.2186163	4.86205426	4.62818891	4.57444017	4.3014339	4.58744113	4.71858856	4.98990891
VPS26A	3.90708387	4.4293498	5.28450165	4.84902656	5.17712049	4.77584079	4.4881302	4.44427026	4.6987898
VPS26B	4.00543572	3.45111208	2.67159059	2.90671083	3.28643067	3.43544443	3.47277409	2.90369464	3.34331146
VPS29	4.45927922	4.87126861	5.02637895	5.266801	5.6912779	4.88863432	4.54335645	4.59598949	4.70468138
VPS33B	2.0326657	1.7086659	2.42168222	2.02546327	2.0435364	2.12176144	1.81621424	2.44131313	2.29535586
VPS35	3.52176742	4.41282629	4.14184377	4.32757384	4.51498508	4.24342055	4.60934491	4.3700813	4.67703043

VPS36	3.36786427	3.72237227	3.4431134	3.09106748	3.49269955	3.66267974	3.95816464	3.20973523	4.46058456
VPS37A	1.95408929	2.71335508	2.15867268	3.09423235	2.54943448	3.04702068	3.06047316	2.59293114	2.59083088
VPS37B	3.2910652	2.69680251	3.79455223	3.76362193	3.36731691	3.7931822	3.4505099	4.71789702	3.49206271
VPS37C	3.67464892	2.44447599	3.10353378	2.92492718	2.80186512	3.14140701	3.17665092	3.54359894	3.36668146
VPS37D	1.85202573	0.99761464	1.06264172	0.86413366	0.18141158	0.84274287	0.29066058	0.81104254	1.30924075
VPS39	3.06144704	3.43182496	3.31888762	3.1557123	3.32050669	3.40546936	2.76469269	2.56518287	3.23515812
VPS41	3.05519695	3.00806008	3.42075604	3.15906744	3.10744889	3.2070643	2.94834911	3.68642792	2.23854392
VPS45	2.15092128	2.78113207	2.7851596	2.80484255	3.02326166	2.66155308	2.70334694	2.14982438	2.70103169
VPS4A	3.48405309	2.55880653	3.06873651	2.7206982	2.84584033	2.83698619	3.18454026	3.14694559	3.2186285
VPS4B	2.67756552	4.19087279	3.55634133	4.59751918	5.07347089	4.43471537	5.57381568	3.80912386	4.23683138
VPS51	4.46280068	3.48902888	3.65302444	3.26399167	2.96909453	3.97817877	3.69286371	4.09487487	4.78092249
VPS52	3.48961956	3.11341574	4.17949934	3.27253198	3.36409731	3.36374547	3.86296652	4.02082863	3.71268046
VPS53	1.52526795	1.44021949	1.73925998	1.71911818	1.86679306	1.64555521	1.68896061	1.13236626	1.85887039
VPS54	2.65976523	2.78372848	3.31334714	3.17646334	3.64488998	2.98961321	2.86907276	3.33211084	2.59868868
VPS8	2.2466016	2.09302046	2.68792347	2.17887313	2.5198064	2.44984544	1.88476437	2.63045894	1.82276876
VPS9D1	2.12688646	1.65274465	1.86421554	1.96848843	1.69312539	1.92176051	1.53886906	2.35327773	1.71436235
VRK1	2.44535029	2.67088615	2.878099	3.06869922	4.19806009	3.35296493	2.64173867	2.63018984	3.22531997
VRK2	1.62991368	2.52780595	3.0498976	3.40148411	4.18612452	3.11176587	3.24634598	2.81339959	2.81080517
VRK3	2.44446482	1.75624053	2.21599815	1.78753196	2.52993365	1.99787281	1.86319957	2.49380806	2.64100498
VSIG1	4.54635387	0.27638373	4.63816688	1.33802908	5.18300938	3.37439764	2.47659772	7.9553079	0.17227113
VSIG10	2.08205725	2.2735021	2.41054947	2.08341828	1.49710025	1.90734124	2.01005834	0.84698034	3.18370363
VSIG2	0.2558627	0.98561197	4.90417461	2.25332707	1.25764124	0.91684865	6.84767039	5.97075706	0.43548181
VSIG4	4.22509329	5.49239736	5.71117008	4.6905265	5.08137934	6.02523865	5.5876682	3.02544937	2.91269024
VSNL1	0.2523273	1.01652998	0.31363357	1.81505752	2.47810683	2.30163026	4.13857593	0.76645063	0.52949611
VSTM2L	0.27061674	1.4283984	1.41555227	0.76238291	0.03397905	0.72994962	3.22052251	1.40331877	0.05751157
VTA1	2.58801018	3.28027461	3.25509133	3.65411504	3.55711763	3.50156103	4.0821989	2.71133517	3.77116248
VTCN1	1.59435867	0.16286335	2.0832899	0.55645342	0.07232767	0.03927433	0.03559326	0.35595917	0.04976108
VTI1B	3.41431587	4.1220132	3.7073044	3.79692494	3.93001642	4.12185889	3.54320233	3.97358111	3.59686834
VWA1	5.91900501	4.47716065	4.61368538	3.37084784	2.20290861	4.14685432	4.02529774	4.32427317	3.1030289
VWA5A	2.82529514	3.39113743	2.50450217	1.8149565	1.55796289	2.74505228	1.62433432	1.00696225	2.04446638
VWA5B1	0	0.12533765	0.13772406	0.00684132	0	0.00419359	0.31801688	0.03696817	0.00300093
VWA7	2.78852192	1.29615738	1.70598557	1.51292001	0.40610787	0.67767693	1.24591392	1.96091102	1.24633464
VWA8	2.08969741	2.54402607	2.275712	2.85216164	3.04547117	2.69529575	3.18441595	2.89734117	3.25868392
VWCE	0.05141482	0.73037621	0.65343318	0.57958315	0.12353628	0.4754883	0.08263229	0.1109038	0.50543411
VWF	3.41923149	4.57702397	4.25639809	3.39546718	3.48280701	5.19456041	3.83953768	3.40555085	3.37905986
WAC	3.20285336	3.764528	3.39945632	4.00161172	4.03687253	3.79754938	3.53767661	3.09010307	3.78228487
WARS	6.01495448	4.79123879	4.88914432	5.09807414	9.79291284	7.32997137	5.51200249	4.98050454	5.12005504
WAS	3.31382437	3.70341545	3.60775259	3.07307308	3.89174349	4.079033	3.02927053	2.25776616	2.43299707
WASF1	1.11259953	3.11749659	1.10652335	2.9094513	2.30958683	2.28792185	2.34933081	0.56642088	2.28377401
WASF2	5.35433633	5.3597468	4.88396645	5.13650198	5.03943866	5.50484581	5.20584803	4.2928802	4.98364445
WASF3	0.17279271	1.86860188	0.67582694	2.03268824	0.28340557	1.53544631	0.41254063	1.08944732	0.1379256
WASL	3.78469296	4.55774662	4.64263869	4.44113587	4.30212987	4.36862966	4.64270715	4.33945262	4.63866935
WBP1	2.3543332	1.54451138	1.87675009	2.03415754	1.57473192	1.72722988	1.58362542	2.33728084	2.24872283
WBP11	4.21132247	3.99957833	4.14247798	4.671641	4.77511756	4.59963703	4.28237618	4.09563834	4.68008803
WBP1L	3.83203972	4.94287778	3.84234812	4.15541274	3.66157509	4.70095496	3.89103561	3.28193128	3.24867738
WBP2	5.22505641	4.17377908	5.01853691	4.25999455	3.83470007	4.46401419	4.30472471	5.60912916	4.26910747
WBP2NL	0.1825559	0.19260547	0.35343476	0.04870183	0.35275755	0.06978827	0.08565905	0.21499186	0.08428403
WDFY1	3.660112	4.14308143	4.0114287	4.74017407	5.36558686	4.50119407	4.46567584	3.92552932	4.19688734
WDFY2	1.26992319	1.10657525	1.25252037	1.05840804	1.21183215	1.29368937	1.01733706	0.86721485	0.95812557
WDHD1	1.45252537	1.56131412	1.76475606	2.36101464	3.11160707	2.30137987	2.0653689	2.09040368	2.16926886
WDPCP	0.47552494	0.43579327	0.61940033	0.53650654	0.42610082	0.48421481	0.34444849	0.55219398	0.28540511
WDR1	5.50392958	5.34062707	5.46636823	5.58541018	5.60074522	5.87553194	5.74942549	5.45819108	5.69568241
WDR11	2.23747604	3.37387637	3.07759458	2.81247461	3.0083839	2.53449687	2.65495944	2.87247512	3.02151117
WDR12	1.56709416	1.6728577	2.31612113	2.66123114	2.55332407	2.30182457	2.56757859	2.27397678	2.71993078
WDR13	3.24657016	2.73715819	2.49545245	2.48498716	2.58351791	2.64591852	2.45935772	3.03970697	2.76920966
WDR18	4.92594114	2.77104423	3.87751438	3.61352456	3.84832416	3.94015379	4.41099406	4.268953	4.56531794
WDR19	1.03601454	1.32116877	1.40690093	1.74504145	1.61474808	1.64220193	0.9904184	1.42987615	1.44808932
WDR20	1.92438504	1.65007281	1.58611136	1.74839198	2.06686931	1.90730623	1.77597368	2.50846549	1.89875223
WDR24	2.57539828	1.99537639	1.26961594	1.58882975	1.85767593	1.86762885	1.97496075	2.15468993	2.61918375

WDR25	1.74058663	1.31149154	1.19224052	1.052661	1.39952028	1.56161842	1.27482032	1.73311507	1.64237328
WDR26	2.7164366	3.53245697	3.80508203	3.81150824	3.60475771	3.73419434	4.09823726	4.14346453	3.43493015
WDR27	0.43840386	0.5672246	1.0401803	1.38551171	0.69583251	0.64902012	0.77153249	0.94255781	1.34404196
WDR3	0.94442314	1.77848602	2.20064283	2.50633265	2.61371949	2.39929897	2.37613164	2.56162405	2.6591411
WDR31	0.80542534	0.80569476	0.83239384	0.48164351	0.55277151	0.60876662	0.50273778	0.48204085	1.06175884
WDR33	2.2530012	2.01075948	2.49832307	2.46297113	2.50328816	2.36529711	2.1350605	2.22780088	2.41107359
WDR34	5.58618655	3.1450565	4.72855431	3.58008742	3.65740559	3.92233212	4.64122744	3.74360138	4.15107944
WDR36	1.8334114	2.9757023	2.92751533	3.47402859	3.73333691	3.53916017	2.86726512	2.50200156	3.71025064
WDR4	1.65099942	1.4053769	1.72522904	1.66442606	2.05073004	2.07276061	2.38086293	2.18889816	2.62295113
WDR41	1.84220269	2.16104054	2.86652477	2.70937855	2.95101098	2.81587648	2.7708146	2.38937226	2.1307048
WDR44	1.81514833	2.83937644	2.87773181	3.22271063	3.82474975	2.86040387	2.55489913	2.77867371	2.61261411
WDR45B	5.99478734	3.9830436	6.02798068	4.97198354	5.20848691	4.83709266	4.84336455	5.72236429	5.39159254
WDR46	3.60009269	2.87683773	4.09199861	3.30158858	3.04772596	3.52958334	3.80439076	4.17445589	3.78626252
WDR48	1.89103917	2.99181567	2.92789777	3.16292705	3.48198107	3.00797538	2.94837422	3.15702788	3.47396589
WDR5	3.89060681	2.91973532	3.84017228	3.79925062	3.97193768	3.0592436	3.64298027	3.58979653	3.91664605
WDR53	2.64560651	1.51605005	2.9866855	1.86387695	2.16394501	1.87998019	1.62617199	2.08775972	1.85725929
WDR54	3.11550683	1.84821028	1.58676064	2.41829845	1.4053034	1.90893233	1.65139662	1.58906918	2.43333461
WDR55	2.34615251	1.70426219	2.51666464	2.53323353	2.31152683	2.27021553	2.35443013	2.72014138	2.47027815
WDR59	1.01352234	1.72136842	2.13160385	2.28446076	1.92209161	1.91348539	1.82922912	2.00738473	2.37138321
WDR5B	1.37619848	1.37129125	2.06827931	1.88321123	1.70999461	1.37399149	1.31957258	1.60726234	1.64661656
WDR6	4.03125421	3.05318417	3.87216359	4.09220837	3.46548619	3.78357492	3.73018283	3.44913501	4.53049553
WDR60	2.31131888	2.00293272	1.6611077	1.81600993	1.58629389	1.73273156	1.62288432	1.21786799	2.14589961
WDR61	2.27762102	2.38523145	2.83498698	2.87129562	2.8709715	2.4640969	2.33895278	1.94551673	2.80167541
WDR62	1.78764943	0.33403276	1.29100324	1.07202981	1.8005799	1.10426289	1.18274174	0.79914012	1.04424017
WDR7	0.7697914	1.72666268	1.24057398	1.56013321	2.10336358	1.6834302	1.7050789	1.26614419	1.44006315
WDR70	1.47325037	2.21686496	2.29570103	2.36913572	2.26583572	2.02212765	1.63274651	1.25708278	2.23532836
WDR73	0.81428711	0.45553644	0.93219061	0.76741469	0.8233803	0.53250748	0.43665253	0.83747535	0.68927393
WDR75	2.77593356	2.90980443	3.197022	3.83200196	4.24803494	3.3480615	2.92708483	3.15240103	3.96585307
WDR77	4.63452246	3.03768632	4.85298404	3.61781947	3.60964697	3.37908901	3.38326021	4.00922657	4.59866825
WDR81	2.18788504	2.19836394	2.32704629	1.98100321	2.47323032	2.62345753	2.16163447	2.0556385	2.58390136
WDR82	4.42843179	4.60898726	4.50963317	4.6502038	4.13687829	4.91331404	5.03825461	4.6680364	4.39494867
WDR83	1.83551536	0.94087811	1.61579144	1.64713175	1.54543101	1.16783375	0.97168689	1.73414698	1.51436937
WDR83OS	5.19078463	4.91497555	4.7215493	4.8121634	5.03400854	4.79684703	4.51457503	4.9245942	4.78198928
WDR86	0.34324728	0.72665222	0.57716901	1.01123881	0.31317575	1.10733858	0.45043636	0.20071996	0.25547277
WDR89	2.07714335	1.9498395	2.3214064	2.34768436	2.44162154	2.22891638	1.61541324	2.31107038	2.2029207
WDR90	2.2246297	1.28500204	0.9410927	1.47345991	1.88082348	1.23366713	2.01092027	1.7154088	2.41990948
WDR91	1.44204646	1.27507961	3.31027847	1.49395425	1.32281444	1.37696868	1.11317811	1.61942691	0.96907426
WDR92	0.09507952	0.30349334	0.46031329	0.3121002	0.38429106	0.23571278	0.1376286	0.25212013	0.29998143
WDTC1	4.19230997	3.75977954	3.69779875	2.87530975	3.44926876	3.89180946	3.24941297	3.33066905	4.24759554
WDYHV1	2.20227565	2.58544829	3.13303509	3.13372839	2.50004113	2.8053874	2.95330949	3.15980019	2.56110241
WEE1	2.33063941	2.09799373	2.09998553	2.94845004	2.51254473	2.75541803	2.74114879	2.03909039	2.48297442
WFDC1	0.97539938	1.57443235	1.3421313	1.23211382	0.31962374	0.78288015	1.32745718	0.87977432	0.47818403
WFDC10B	0.11129304	0	0.08125581	0.13254793	0	0.02110004	0.06764665	0.06538435	0.27592463
WFDC2	0.05180114	2.99515671	4.1222594	2.47563684	0.51390239	4.17088872	2.05735739	0.51576586	2.1690835
WFIKKN2	0	0.04145485	0.0584742	0.0803162	0	0.14122959	0.00548344	0.01757896	0.00481613
WFS1	2.62576893	3.40811383	2.57873061	3.31174498	2.63869225	3.52501848	3.12378477	3.67355819	3.11709696
WHAMM	2.21996843	1.39646544	2.28780164	1.8681857	2.2157661	1.83482733	1.5008454	2.09450526	1.80678942
WIPF1	2.87897691	3.88787474	3.76871135	3.53869584	4.19451823	4.18278583	3.13948282	2.40185267	2.76953841
WIPF3	0.40586478	0.55112851	0.55230027	1.09559091	0.56292517	1.90986429	1.56059478	0.96136321	0.10064003
WIPI1	2.51481019	3.04919126	3.99317957	3.47473557	2.38971136	2.95873283	2.57975351	4.41780222	3.18075266
WLS	1.46065009	5.16463567	4.87046039	4.38076941	3.95227073	4.80756309	4.29080797	4.02299438	3.79497225
WNK1	3.14526708	4.27183272	3.81260586	3.94196215	4.52739777	4.36679751	3.56840707	4.42079205	3.67160355
WNK2	0.11920019	2.07613117	1.64978137	1.37576634	0.88948882	1.55941786	1.69751105	0.32797424	3.1465939
WNK4	0.35946178	0.89208254	0.15673487	0.53279142	0.64965556	0.14744865	0.95087288	0.53184798	2.51407544
WNT10A	0.23596093	0.21555872	0.37800276	2.57032744	0.42936431	3.74114478	0.30438649	3.14802784	0.69837218
WNT10B	1.06723348	0.11444602	0.36912129	0.73375621	0.47579644	0.10791732	0.06490411	0.07292665	0.20354371
WNT11	0.07396644	1.20650208	1.87799763	1.50751638	0.04601321	0.60585449	1.7219493	0.02321351	2.16661412
WNT16	0.01113294	0.11677441	0.21698562	0.15858353	0.04072907	0.064249	0.26231091	0.01286484	0.01170147
WNT2B	0.15657012	2.68180019	1.12419845	0.43157276	0.39472327	0.40613419	0.88841115	0.46447193	0.39044056

WNT3	0.04959228	0.46879132	0.73216756	0.34399617	0.43498162	1.07092965	0.59064645	0.14481115	0.67576223
WNT4	0.10103556	0.81355391	2.08314571	0.16847781	0.1416028	0.22925026	1.36736597	0.88548762	0.53191103
WNT5A	1.43331366	1.33001044	3.24037569	2.78207845	1.79296189	2.39117891	3.16373899	1.94706056	2.20010298
WNT5B	0.27649411	0.57602148	1.53568146	0.7570449	0.27029159	1.00028865	1.68231979	1.93964006	1.55738312
WNT7B	0.21417778	0.04074878	0.28736545	0.60606352	0.05665771	1.56952623	0.62188889	0.32260842	0.07752548
WNT9A	0.12308573	1.49482587	0.26017885	0.47831423	0.02799614	1.18366686	0.29239974	0.15735139	0.19802913
WNT9B	0.01095886	0.00628741	0.08487623	0.02616084	0.00676097	0.05550461	0.05174113	0.00845487	0.00577076
WRAP53	1.72429878	1.14518838	1.59620229	1.75468906	1.62019205	1.45260457	1.50138601	1.22873098	1.92631504
WRAP73	2.08376084	1.03992925	1.31920466	1.76458383	1.79758622	1.49004907	1.55822611	1.17791867	2.53876754
WRN	1.19336191	2.04461788	1.67493825	2.67359289	2.78198743	2.03753763	2.51834827	2.00251261	2.3758683
WRNIP1	4.01571859	3.01769351	3.56229455	3.86596951	3.73018641	3.69099638	4.4286488	4.1599552	4.07999919
WSB1	3.09151261	2.68443973	3.93606753	4.27204538	3.81836975	3.17972737	2.44980282	2.69333694	3.26738861
WSB2	4.79528381	3.78617248	4.50099194	4.52921727	3.98127175	4.25769961	4.45763854	5.42582984	4.38069134
WTIP	0.24584676	0.50737686	0.69181064	0.87872198	0.20537097	0.67028447	0.24487465	0.21881029	0.07475168
WWC1	3.94376676	1.16306875	2.82754273	2.35441749	2.47691742	2.709275	2.19561554	3.20223413	2.79290836
WWC2	1.55039619	1.50936605	1.63419944	1.76285029	0.90527486	2.21228514	1.48988707	1.38460509	0.39152239
WWC3	2.703695	2.87377652	2.6775567	2.81596751	3.02545225	2.75764598	2.47495555	2.56330753	1.7505612
WWOX	0.96062388	1.90736506	1.8222258	1.46565209	1.22117469	1.57871778	1.67130187	1.0731073	1.43797857
WWP1	2.88424818	3.97923379	4.75160324	3.81308445	3.67468474	3.78511295	3.72851359	4.60145933	3.7244389
WWP2	3.09813398	2.90731771	3.52849817	3.10190593	3.28546994	3.33797482	3.66178318	3.73713454	3.78256574
WWTR1	3.67915608	5.1115867	4.5945396	3.55120266	1.82139083	4.89358012	2.62008245	4.13039731	1.43709677
XAB2	3.94649769	3.24884642	2.88089075	3.14017974	3.6689352	3.607922	3.34452635	3.74921797	3.7266674
XAF1	0.78708334	2.61989113	1.77848595	1.71456598	3.0766454	2.73982342	1.02217668	0.74212792	1.65436445
XCR1	1.17186897	1.15482913	0.52857246	0.38488431	0.64573926	0.10050831	0.1494577	0.1004142	0.25598607
XDH	0.1270503	1.76012725	1.48322994	0.84465067	2.17920502	0.19280657	3.14897598	1.49859304	3.9696014
XIAP	2.49735115	2.54606676	4.61734789	2.92499525	3.595663	2.80734611	2.61916191	3.2714957	2.98755788
XK	2.32286564	2.53746829	3.1271487	2.06117182	3.77469273	2.31183714	1.66307069	0.36724158	1.58970799
XKR8	2.0074552	1.73165702	2.40186927	2.04906396	2.18996248	2.51183871	2.03694231	1.60116523	1.91787388
XPC	2.21518668	3.04175892	2.79999087	2.83223202	2.94505928	2.89228881	2.76868628	2.88757849	3.0216211
XPNPEP1	4.00407702	3.64492954	3.08763422	3.85934372	4.38414884	3.60439974	4.0802384	2.9789803	4.86829799
XPNPEP2	0.08098652	4.3997871	1.18417252	1.77608136	0.48101724	1.62002505	0.17030331	0.3851551	0.61102702
XPNPEP3	2.05601986	1.34402066	1.8022692	1.72252737	1.7617433	1.41862694	1.39939376	1.3698421	1.71496735
XPO1	3.52074035	3.58712816	3.91940523	4.42475897	4.82904417	4.19980475	4.22923001	4.09218172	4.04610314
XPO4	1.9124024	2.37785834	2.31927292	2.69389132	3.55347869	3.06882714	3.16856493	2.48834278	3.31849983
XPO5	2.73012364	2.46154021	3.449164	3.20197749	3.30111213	3.22269013	3.60358862	3.43056221	3.29215155
XPO6	4.35437623	4.05936642	3.62532351	3.9759468	4.09050652	4.40046654	4.51352119	3.6438971	4.09017015
XPOT	3.43214322	3.42231451	4.36640113	4.27978263	4.33139516	4.06154001	4.01772545	4.18827112	4.00004693
XPR1	2.73812822	2.95476364	2.5996292	3.25202358	4.0613582	3.14912506	3.13436298	2.8579721	2.8927244
XRCC1	4.13331865	2.81141103	2.88136413	2.97240274	3.60646734	3.0929147	2.80062288	3.31566359	3.11090835
XRCC3	1.88705782	0.8935534	1.06291949	1.24785085	1.6973423	1.29805053	0.82004214	1.51986384	1.5420548
XRCC4	1.69899415	2.66502783	2.77501725	3.40288912	2.77128238	2.72434012	1.86576937	1.66213735	2.53068837
XRCC5	5.22590794	5.65848685	5.61786031	5.81768604	5.96466736	5.89631352	5.9324405	5.80923321	6.14082013
XRCC6	6.83601884	6.59774289	6.64252227	6.63848919	6.8597955	6.78455397	6.68828449	5.93589854	6.9694769
XRN1	2.25133067	2.54899869	2.67867786	2.56076009	3.98160769	3.08536775	2.56669269	2.35172917	2.8635371
XXYLT1	3.24437229	1.85130798	3.18070983	2.362927	1.9836037	2.36531995	2.45676853	2.27806419	1.79885459
XYLT1	1.13340336	2.42940908	2.05584511	1.87764027	0.99581871	1.58085588	1.76526826	1.73730157	1.45864691
XYLT2	3.91901788	2.1281441	3.10396121	2.40410414	2.24050587	2.32770106	1.7778331	3.02792793	2.50057835
YAF2	0.88259522	1.54516362	1.54656533	1.55483961	1.68756993	1.54464193	1.09843064	0.77217138	1.41472594
YAP1	4.49353397	4.58336724	3.77696679	4.58950572	4.16045595	4.7590276	4.49959857	4.33555051	4.20077212
YARS	4.50888269	2.94472423	3.61914873	3.58181241	4.42250319	3.82693631	3.75858116	2.73067403	3.87239042
YARS2	3.38862298	2.88906492	3.16814375	3.43811548	3.72873605	3.39077191	3.08825912	3.74185324	3.75103717
YBEY	2.78063936	1.98653613	1.59454531	2.43385436	2.58585445	2.62345114	1.49362521	2.52954421	2.44318588
YBX1	8.32865992	7.72120734	8.20847739	7.91151007	7.99574625	8.48501828	8.51251476	6.89874813	8.39304107
YBX2	0.02348364	1.33144705	0.19190921	1.87765245	0.80302323	0.22009714	0.82868475	1.04741702	3.21608111
YBX3	4.37983971	5.10595962	3.73870158	4.78278862	3.65269807	4.87564488	4.42092685	4.00155944	4.20821509
YDJC	5.65947039	3.09565968	3.69781066	3.59218053	3.29951638	4.12182094	3.59126615	3.77148105	4.81532234
YEATS2	3.02049247	2.17441757	2.83173332	2.68823975	2.71185968	2.48448073	2.29289886	2.55432002	2.73470634
YEATS4	2.66677056	3.44213446	4.60654109	4.44608287	4.1550441	3.8509802	3.65149217	3.55148844	4.29397653
YES1	3.60873401	4.01910087	4.3274923	4.08619203	3.82259053	4.29985395	5.29317741	3.98752841	3.99147795

YIF1A	5.2934072	4.50085693	5.18108049	4.45993598	4.17233448	4.9534012	5.17475851	4.98067657	5.04352896
YIPF1	3.91975867	3.94617888	4.42759777	3.82988402	4.4505351	4.02979436	3.57404942	4.57825523	3.95307544
YIPF2	4.66726565	3.6404097	4.08038141	3.57949784	3.22873845	3.78140159	4.36260181	4.11705551	4.29545156
YIPF3	5.96910973	5.17738059	6.10512527	5.45046785	4.99203805	5.35025594	5.80861049	6.52772888	5.87181924
YIPF5	3.37741413	4.31863083	4.25682154	4.45429408	4.12268214	4.40930728	4.11734173	4.32547205	3.87510755
YIPF6	2.4769237	3.17125597	3.40425687	3.07740615	3.43388034	2.83880237	2.92092034	3.19411018	2.90770095
YJEFN3	0.36766908	0.14103078	0.21457725	0.68202727	1.77812608	0.22187623	0.18647786	0.52368902	0.36925675
YME1L1	4.03727481	4.43973656	3.85557751	4.65190998	4.72445831	4.60377756	4.45492577	3.89868993	4.34543173
YOD1	1.21955503	1.34102177	2.47414072	2.6163031	2.82293588	2.99048658	3.18661139	3.56665853	2.17914514
YPEL1	0.36073563	0.47499809	0.40207763	0.53945492	0.50858656	0.61516675	0.1535442	0.34554043	0.82535662
YPEL2	1.62344423	1.88738626	2.56331358	1.7666812	2.13954165	1.86217484	1.68832423	1.62632118	1.49418088
YPEL3	3.04587571	4.17312652	3.5861705	3.90212112	2.86910896	3.81505845	3.18216696	3.51914398	3.92609649
YPEL4	0.14745937	0.32171625	0.24384358	0.33143261	0.28254314	0.18568004	0.10378889	0.07666316	0.05064365
YPEL5	3.92950212	4.80290351	4.79159324	4.49780905	4.16918089	4.63855995	3.90254237	4.08581758	4.16750038
YRDC	3.1737649	2.57355087	4.40664781	3.34529263	3.65722147	3.86511332	3.5412155	2.95724669	3.76962062
YTHDC1	3.10458964	2.85127389	3.25301733	3.51004414	3.63195586	3.09079027	2.82710069	3.20177027	3.4248995
YTHDC2	1.28888626	1.80916904	2.16492385	2.79782419	2.75970127	2.54107321	1.85878628	1.4902446	2.48133169
YTHDF1	4.42329557	3.75460107	5.29638928	4.54225759	4.17032002	4.01847208	4.20904492	4.23495005	4.93023824
YTHDF2	4.31461187	4.33749291	4.48636602	4.56803973	4.613685	4.56097316	4.17766125	3.91006951	4.88185603
YTHDF3	3.29585574	4.63596879	4.32362545	4.79497596	4.65365909	4.83321049	4.90498375	4.64488607	4.29248223
YWHAB	6.42084272	6.94477149	7.29550163	7.07322118	6.9188251	6.96944848	6.95397498	6.67703734	7.28816721
YWHAE	5.98212655	6.20593226	6.81962404	7.28775443	7.05254328	6.7863746	6.77553922	5.86597665	7.33191751
YWHAG	5.84663974	5.47568206	5.86342813	6.01549789	5.43423451	5.97760813	6.05579942	5.67882364	5.95031046
YWHAH	7.00461972	6.30195826	7.06976669	6.77439347	7.7491881	7.00952614	6.7745082	5.97642515	6.38523586
YWHAQ	6.53461565	6.85919623	6.63903624	7.13853105	7.00154528	6.98595677	6.86984762	6.7550257	7.0239601
YWHAZ	5.998985	6.36332991	6.95869822	7.36516864	6.98250517	6.99094332	7.48986445	6.76370745	7.0422045
YY1	4.00552872	4.05986794	3.55503629	3.99036193	3.75544064	4.17816312	4.05330043	4.63845524	4.15595467
YY1AP1	3.58760639	3.4255893	3.67460845	3.65997538	3.73178572	3.52649172	3.88579793	4.03834898	3.77327063
YY2	0.40334246	0.5028859	0.34065966	0.55580997	0.84387509	0.2627809	0.24801803	0.45730318	0.27298965
ZAP70	1.28879829	0.66285456	1.64204891	0.7678992	2.63948259	1.15080159	0.71808085	0.50884065	1.15966874
ZBBX	0	0.02604203	0.32505856	0.02726511	0	0.01007854	0.00548206	0.01057041	0
ZBED1	4.09973194	3.92388625	3.81720745	3.24464162	3.57825639	3.91565884	3.17573959	3.99766702	3.88868218
ZBED4	3.17115386	2.60110339	2.12693397	2.72634744	2.6316216	3.00133691	2.90566425	2.41433261	3.52798885
ZBED5	1.34733073	2.04441962	2.07308654	3.05576122	2.18714931	2.08868144	1.62062561	2.12508143	2.59838778
ZBED6	1.12227453	1.95998269	1.71946344	1.57318933	1.98234466	1.90756755	2.06749849	2.95102559	1.73314537
ZBED8	0.52653041	1.08607876	1.28397685	1.41038612	0.84644714	0.9436687	0.54096653	0.86302946	0.82886022
ZBP1	0.45827862	0.74845357	1.13886506	1.00277559	1.64513051	1.20805579	0.33364167	0.34914699	0.93094524
ZBTB1	2.14992349	2.92095776	3.28138056	3.32184898	3.54138333	3.14048881	2.52824162	2.90016383	3.15620564
ZBTB10	0.32448988	1.82762011	1.49590952	1.31927987	0.67961694	1.54000326	0.73775102	1.94042464	1.27926487
ZBTB11	2.07574614	2.23308203	2.46616897	2.62156085	2.74223763	2.72701724	2.32857925	2.47431418	2.42509944
ZBTB12	3.06981084	1.09906393	1.65587474	1.46155186	1.46010256	1.27248505	2.09843373	2.41292903	1.85328055
ZBTB14	1.50249067	1.98319743	2.37095663	2.4533544	2.35961211	2.35944292	2.1394953	2.21391417	2.18230597
ZBTB16	0.03949137	1.10826702	0.39198439	0.50206636	0.07884593	0.61510998	0.06689973	0.13058241	0.0297551
ZBTB17	3.18158395	1.82057951	1.80237557	2.02773847	2.06679204	2.17982881	1.7534761	2.00377028	2.53003958
ZBTB18	2.57230094	2.28647947	2.53221761	1.78266946	2.52934142	2.23480449	2.19933921	3.52407772	3.31137551
ZBTB2	3.16130823	2.7944794	3.20779386	3.02180097	3.56046186	3.2796212	3.24577467	2.79483183	2.73726952
ZBTB20	0.70089842	2.15837075	0.83039385	0.57767646	0.5315655	1.2075587	0.41405745	0.70518454	0.31845809
ZBTB21	0.99288752	2.62925117	1.93297313	2.374784	2.53911192	2.86855507	2.32000651	1.86776383	2.14649438
ZBTB24	1.77263589	1.7860906	1.88561437	2.36209679	2.71568418	2.13437326	2.12768131	1.58946253	2.38605866
ZBTB25	1.04677944	1.22323659	1.29402458	1.6740795	1.56672267	1.42897596	0.95773184	1.13216046	1.21637117
ZBTB26	1.26491284	1.78727613	2.39482614	1.96733744	2.03529371	1.7697095	1.3428948	1.25303555	2.13903395
ZBTB3	1.39908023	0.97364424	1.32454159	0.96059543	0.80216738	0.72134911	0.69491046	1.02935056	1.24626834
ZBTB32	0.27471416	0.1770818	0.53409161	0.39763219	0.8004114	0.2358406	0.16362891	0.46058325	0.31554047
ZBTB33	2.10903491	2.85426287	3.63956776	3.29701916	4.12949378	3.15915413	2.56446908	3.20145432	3.68076737
ZBTB37	0.41527445	0.83355869	0.69747975	0.67838734	0.7488146	0.81958147	0.80968422	0.73967583	0.49709778
ZBTB38	2.8384103	3.68908902	3.1482255	3.42042807	3.43037416	3.2653414	2.69797667	4.01448801	3.84426365
ZBTB39	1.84465914	1.41037202	1.6268235	1.56329721	2.18628993	1.47645104	1.18862228	1.74197791	2.20110768
ZBTB4	3.25832231	4.52417599	3.60772776	3.88730761	3.18649638	4.21034592	3.36737345	3.35368582	3.32784572
ZBTB40	1.80168821	1.77545049	1.70532918	2.03548797	2.28181332	2.1789157	1.63793982	1.30285636	2.37966778

ZBTB43	1.30793512	1.47242539	2.03941451	1.82036967	1.89910016	1.99889995	1.6877442	1.28280658	1.5133375
ZBTB44	1.91046814	2.81217531	2.29253028	2.47714849	2.31663638	2.87203938	2.75015557	2.18732136	2.9627278
ZBTB45	3.21949995	2.20644178	2.3155596	2.66822115	2.40422833	2.76562014	2.26580022	2.8118513	2.84337077
ZBTB46	1.66520086	1.04149974	1.44141549	1.13470256	1.14230417	1.12688237	1.14108625	0.78788959	0.47351673
ZBTB47	2.34546174	3.13866394	2.10163973	2.37205925	1.17335604	2.80474375	2.20147077	2.04569446	0.98792647
ZBTB48	1.94900738	1.32230458	1.61111038	1.73220781	1.405179	1.69348745	1.26526455	1.21615316	2.11606727
ZBTB49	1.23056724	0.97207067	1.35278807	1.34913453	1.50704252	1.27695093	0.83603802	1.50251533	1.54884052
ZBTB5	2.01471795	2.13831391	2.06729524	3.15443304	2.25916857	2.28913056	2.25047465	2.62530435	2.5241553
ZBTB7A	4.99452863	3.67759877	3.9286099	3.86808782	3.62058732	4.41890665	3.91214243	4.60596723	4.40817011
ZBTB8OS	2.89785037	2.38089638	2.70833409	2.70102934	2.92808773	2.27435572	2.4105712	1.6263689	2.7174065
ZC2HC1A	0.76627742	2.00643335	2.08518564	3.28281097	1.74198698	2.58428227	1.87404036	1.55229633	1.25380704
ZC2HC1C	0.64797879	0.55493127	0.69440395	0.36357967	0.23221799	0.43614425	0.35224092	0.65871754	1.18927885
ZC3H10	0.74117513	0.80070977	0.99725271	0.74422008	0.63032817	0.9069652	0.43695875	0.8149326	0.71100253
ZC3H12A	6.11673623	1.79480106	6.83436343	3.74875409	3.89528011	4.29950147	3.59462006	4.56583085	5.54842221
ZC3H12B	0.03676844	0.89915814	0.80402932	0.73465794	0.58966346	0.62239505	0.62911708	0.13683488	0.95577436
ZC3H14	1.2906096	1.70568914	1.78805388	2.03926792	2.10826708	1.71915988	1.79121837	2.08128563	2.09298348
ZC3H15	3.76323277	4.24884733	4.6839246	4.84554451	4.93831063	4.63726106	4.55407206	4.53132827	4.67028415
ZC3H3	3.87681023	2.75271965	3.17739432	3.01005826	3.09868008	3.38246065	3.72153479	3.8406809	3.27080464
ZC3H6	0.64026279	1.53066463	0.85987806	1.10985122	0.96102067	1.06833401	1.09345734	0.76652765	0.9597767
ZC3H8	0.77238716	0.72920228	1.71306432	1.66031986	1.65013928	1.04683513	0.86775914	1.04724175	1.73367938
ZC3HAV1	3.40161113	3.33228031	3.39001279	3.43447011	3.59158328	3.79605035	3.22376966	2.97492576	3.17781165
ZC3HAV1L	2.71424082	1.40646675	2.32431211	2.49196855	0.62436251	2.34997419	2.73899956	2.33915465	2.91253796
ZC3HC1	2.72342623	1.97858862	2.40034471	2.20918225	2.13948034	2.4978861	2.31544301	2.08078262	2.38901785
ZCCHC10	2.81183004	3.10901495	3.67603803	3.27538267	3.4726455	3.05460398	2.61013475	3.02395023	3.54518391
ZCCHC14	1.98934243	3.04655339	2.62906368	3.30963068	2.59991062	3.29620712	3.5095477	3.70392739	3.60108946
ZCCHC17	4.0459105	4.29814589	4.85694814	4.23741425	4.12141625	3.77025883	4.15687208	3.12334895	3.98430231
ZCCHC2	1.17237902	1.86542054	1.52489969	1.9266295	2.48451734	2.31120837	2.65425702	1.29822799	1.77217614
ZCCHC24	1.80143247	4.92291684	2.28707463	3.09279066	1.54419093	3.94256746	2.28953352	2.46775329	1.11234467
ZCCHC3	3.39921857	2.46162242	3.14067564	2.99991995	2.78118083	2.67475109	2.66304024	2.38748576	3.36286583
ZCCHC7	2.01345572	2.79512665	2.36106143	3.32708957	2.88850578	3.03657918	2.61832244	3.10676127	3.29786528
ZCCHC8	1.91136113	1.4114936	2.07206729	2.05908521	2.38876938	1.92295734	1.53356949	2.14643904	2.03359628
ZCCHC9	1.97695289	2.33305134	3.01884481	2.90128175	2.78548326	2.42692417	2.0985323	2.41151092	2.44885741
ZCWPW1	0.52765617	1.07567019	0.68755777	0.53825091	1.20113494	0.56125848	0.38946838	0.86057809	0.42241224
ZCWPW2	0.03728093	0.48012087	0.53366072	0.54665784	0.19552495	0.28453363	0.19692916	0.37027094	0.23179443
ZDHHC1	2.45148778	2.64163811	3.41864579	2.42584001	1.63217098	1.9813959	1.08240167	2.86845247	2.9066537
ZDHHC11	0.1586767	0.09019506	0.2207832	0.92618425	0.70393229	0.39364383	0.24472059	0.45088844	0.04074695
ZDHHC12	5.52290008	3.66176609	4.56461975	3.86536229	3.50448179	4.12673211	4.26187746	3.36267742	3.97690745
ZDHHC13	1.38987023	1.5759827	2.94799203	1.8173608	2.83680034	1.83569127	2.11339116	2.25289046	2.48969716
ZDHHC14	1.05271866	1.30239261	1.94692119	1.07772003	1.72820219	1.13487826	0.97084318	0.81766855	0.9307356
ZDHHC16	3.82251747	3.17913384	3.10557709	3.13984709	2.83857489	3.16704423	3.30773763	3.53249873	3.58297138
ZDHHC17	0.87606248	1.75743058	2.19718467	2.09151912	1.73893389	1.81807636	1.43268465	1.77200534	1.5071054
ZDHHC18	3.39277796	2.09651613	3.15160744	2.55969734	2.87881771	2.90891459	2.6691427	1.94605993	2.46120775
ZDHHC19	0.0149083	0.13900633	0.33645834	0.02672305	0.03646242	0.16623729	0.02666347	0.04548146	0
ZDHHC2	1.67546611	1.96283576	2.72981727	3.55345682	1.3945237	3.91341493	3.63060414	1.22924521	1.97298829
ZDHHC20	4.77128501	4.35193832	4.80749373	5.20520846	4.80293628	4.39441034	5.25076316	4.42378958	5.08294546
ZDHHC21	1.24130468	1.18141796	1.69521408	2.12979614	1.41281179	1.85492041	1.97076713	3.17042475	2.05551455
ZDHHC23	1.58645301	0.66341104	1.82331333	1.77421983	1.71774419	1.46704101	0.99535718	2.02855343	1.90954182
ZDHHC24	2.79880498	1.89900455	2.61431973	1.7897102	1.6534458	2.38655382	1.93856009	2.75571534	2.19910177
ZDHHC3	3.92469582	3.50521155	3.83552257	3.98995712	3.91412607	3.46616332	4.21244826	4.23366693	4.14672562
ZDHHC4	3.91894212	3.49671558	3.75860247	2.65936875	3.15333491	3.36822224	3.64725474	2.94653999	3.151246
ZDHHC5	5.00121332	4.39891997	5.00173578	4.68216191	4.93625363	4.7654706	5.0104415	5.04813689	4.85919959
ZDHHC6	2.9772534	3.40987642	4.53815545	4.05106426	3.82610349	3.20737264	3.51723878	2.68989819	4.02786877
ZDHHC8	3.61896486	2.70361635	2.80534205	2.76212967	1.92586434	3.03502679	2.45354582	1.34624752	3.03392052
ZDHHC9	4.83226838	4.32004867	5.98211741	4.22605988	4.16650441	4.05863637	4.38370173	4.62498878	5.00213198
ZEB1	1.09296985	4.21227105	2.77936967	3.07686464	2.23730839	3.52566682	2.17547726	1.75777102	1.15280905
ZEB2	1.07955788	3.19341753	1.9045302	2.29529176	2.28062093	2.55245731	1.76706132	1.05620514	0.91973119
ZER1	3.9191557	3.99578499	4.0785397	3.28953127	3.23004856	3.87519927	2.96404494	3.14264669	3.73665562
ZFAND1	3.09951794	3.30196062	3.80840408	4.48794348	4.27567739	3.44576032	3.92766144	3.34497896	3.82137368
ZFAND2A	2.7638671	2.49335834	3.07742689	2.36906139	2.65873552	2.76867445	2.4924604	2.19035209	2.97194031

ZFAND2B	3.48618083	2.10077109	2.54383426	2.03778953	2.07776514	2.32072872	2.36208639	2.32260527	2.92379933
ZFAND3	4.962323	4.4301225	4.19847939	4.12185632	3.84016624	4.48314103	4.49561223	5.21639578	4.32072903
ZFAND4	0.92591443	1.04015634	1.34173137	1.2399431	1.20383931	1.37165051	0.74169099	1.14965512	0.65366292
ZFAND5	4.54423146	4.76938596	4.91494237	4.99278874	5.36719316	4.88511383	4.40036004	4.72963809	4.54658158
ZFAT	1.09447819	0.85572142	1.10460868	0.91756154	0.99760863	1.20716086	1.14277033	1.41533496	0.91778448
ZFC3H1	1.19731721	1.88614316	2.75987856	2.45153449	2.66553314	2.07405721	1.50379921	2.35105207	3.13021133
ZFHX3	2.05585359	2.79474398	2.32878959	1.8460374	2.08828103	2.28473169	1.58955296	2.40325233	1.75825728
ZFHX4	0.18508078	1.53016011	0.47844116	0.93000131	0.17246527	1.5327585	0.40268547	0.17517108	0.05275596
ZFP14	0.78836732	0.9200801	1.3118274	0.83297313	1.31910519	0.79393692	0.53099957	1.0055007	1.0767172
ZFP2	0.11913954	0.65561656	0.67916593	0.76563807	0.64679684	0.51476969	0.20021629	0.71499039	0.73000577
ZFP3	0.26759792	1.35886779	1.69292998	0.6830567	0.80267849	0.65941273	0.30144154	0.21980904	1.12494645
ZFP30	0.35465776	0.82831359	1.14120743	0.93412527	0.37282529	0.33365461	0.6661355	1.10088388	1.12009248
ZFP36L1	6.46441848	6.71294761	6.05383883	6.58845378	5.66605442	7.07977805	5.57792019	5.68578311	5.99035678
ZFP36L2	7.05448914	6.96506361	6.37768884	7.02288604	7.28559678	7.45393964	7.01802294	6.7139852	6.67943011
ZFP37	0.21785669	0.55718817	1.08810383	0.74418465	0.46171628	0.30472587	0.35707553	0.06060774	0.47156246
ZFP41	0.96655544	0.82765539	1.04756748	0.95838402	0.68937358	1.02452417	1.04666482	1.68291563	1.06320411
ZFP64	2.0487269	1.96705595	2.71506377	2.38330802	2.18719051	2.15390404	2.84938382	2.22409744	2.78104715
ZFP82	0.16499133	0.74889486	0.59280664	0.54234042	0.50257162	0.39542103	0.18010406	0.19406572	0.13859946
ZFP90	1.80701029	2.18714823	1.960899	2.44585155	2.52236479	2.20045947	1.50306869	2.26183298	1.90483658
ZFP91	3.31792152	3.98435949	4.07994296	4.45336038	4.14989736	4.32660115	4.34108048	4.17733882	4.05120419
ZFPL1	2.49718673	1.93245979	1.86208605	1.83353509	1.70660322	2.19029807	2.53645355	2.26454184	2.33473995
ZFPM1	3.00143431	2.11032684	2.94371362	1.40844013	2.36360696	1.79306646	2.33386989	3.8002647	3.3904193
ZFPM2	0.23321464	1.78079401	0.51557609	1.48200598	0.18917414	1.28529874	0.44752793	0.35106127	0.09994084
ZFR	3.44489384	4.34383188	4.18288071	4.51173926	4.51510705	4.2281096	3.73007221	4.12795349	4.40461169
ZFX	1.38257622	2.45715239	2.50827301	2.73846844	3.45966275	2.41640465	2.13041633	2.32109551	3.03868866
ZFY	1.21752535	2.1783945	1.15385366	0.00395069	0.00405647	2.15795104	0	2.45132511	0.00346189
ZFYVE16	1.43734149	2.56746408	2.97074532	2.86995945	3.24381865	2.55466686	2.27996154	2.47558615	2.38488656
ZFYVE19	2.79413744	1.68542647	2.16195893	1.79645586	1.97241452	1.88814414	1.88963301	1.58429605	2.27580545
ZFYVE21	2.27019526	2.69082404	1.71547563	2.74905314	2.26098866	2.40120142	2.56482761	2.42158058	1.8670213
ZFYVE26	1.6687539	1.96288213	2.06685955	1.73695643	2.50935737	2.12928746	1.68457259	1.63527512	1.77313625
ZFYVE28	1.76181889	0.77751239	0.59285304	0.69501209	1.43441986	0.71911508	0.40455413	0.39909225	0.87774008
ZG16B	2.59523283	2.41489925	3.14760198	1.51296979	0.77110946	1.11088498	3.45618734	4.99203971	2.74845453
ZGRF1	0.59248627	0.47285785	0.93978768	0.86721916	1.57681906	0.65821843	0.48578423	0.54377245	1.03785847
ZHX1	2.3880919	3.37310549	2.81673128	2.98310015	3.47969093	3.37814958	3.76128661	3.09390957	2.6337129
ZHX2	2.10960629	3.16037859	3.54657414	3.5363103	2.03513242	2.62901589	2.73609926	3.12010966	2.98564838
ZHX3	1.26037452	2.40687366	2.01620242	2.01344187	1.36523616	1.96409873	2.05638679	1.59734617	1.84320246
ZIC2	0.02185475	2.18716106	0.71334339	0.9986895	0.01350258	1.42357218	3.11308006	1.73282754	3.99124605
ZIC5	0.01562748	0.89357599	0.09339924	0.70824075	0.00964721	1.26457467	2.22958693	0.02699727	2.26940836
ZIK1	0.16984689	0.64801886	0.82433095	0.45314731	0.57667728	0.34498293	0.26811365	0.14478828	0.19723694
ZKSCAN1	3.2512285	3.93917758	3.74834037	3.61425423	3.08154517	3.34103447	3.41554239	4.91081569	3.8325183
ZKSCAN2	0.82392307	1.01459521	0.76808227	1.08539475	1.02909383	0.98167467	0.91101401	0.58344585	0.88858289
ZKSCAN3	0.81697844	0.78508647	1.33069188	1.4277406	0.89884533	0.91503696	0.8968238	1.18322321	1.08330338
ZKSCAN4	0.86315478	1.39993959	2.38158648	1.5145002	1.44189195	1.39273963	1.18081555	1.81607016	1.34975721
ZKSCAN5	2.11116428	1.57221112	2.11238363	1.90511316	1.94093994	1.78496871	1.73539153	1.89415872	1.8472967
ZKSCAN8	1.41484199	2.63377578	3.13189727	3.00863946	2.95716348	2.78460741	2.31436276	2.82347118	2.90243801
ZMAT1	0.06171455	0.45592786	0.46859767	0.82580189	0.32023409	0.62390721	0.1170625	0.68617241	0.19523137
ZMAT2	5.10097017	4.45222003	5.05006576	4.40880304	4.43435644	4.10965454	4.19442618	4.85852161	4.60090517
ZMAT3	1.6553981	3.38613231	3.57495927	3.37466138	1.81294577	2.97678352	3.26754534	2.80145352	2.70092653
ZMAT5	2.77270473	2.10161637	2.04454166	1.61811155	1.57498888	1.94749134	1.32077627	1.56384234	1.455651
ZMIZ1	3.7685628	4.039633	3.55793201	3.73422264	3.84828915	3.78924813	3.4021196	3.87492284	3.53673577
ZMIZ2	4.82463149	3.63959489	4.10446364	3.79602433	3.39316638	3.54587429	4.01094231	3.54428252	4.35747417
ZMPSTE24	4.17456844	4.47028051	5.75200213	4.38803448	4.99362313	4.32061565	4.20589574	3.8731506	4.63672689
ZMYM1	1.21971906	1.64105284	2.28754023	2.3829178	2.39452519	1.99827596	1.25362117	1.34752465	1.80963375
ZMYM4	2.34570949	3.3622292	3.58094289	3.21451927	3.07902917	3.28559619	3.0223452	2.74558264	3.4667159
ZMYM5	1.87727707	2.01286605	2.10867023	2.77251188	2.64134514	2.01872207	2.20808072	2.45661467	1.99628699
ZMYM6	0.87366518	1.09697988	1.62197366	1.41529073	1.31494024	1.09042606	0.85379898	0.80500798	1.15576818
ZMYND11	2.61892118	4.07502631	3.35551135	4.3651551	4.00752748	3.84912103	3.9437854	3.27583792	4.06900403
ZMYND12	1.15797311	1.52214922	1.49190024	1.36029517	0.73677799	0.89077578	1.71686698	0.18023841	0.42741042
ZMYND15	1.11270909	1.21054452	1.86440459	1.41447998	2.30830469	1.33838987	1.07582417	1.25959114	1.81747733

ZMYND19	3.556689	1.79265437	3.28287199	2.72185592	2.6450638	2.73514422	2.72467581	2.72220872	3.19300153
ZMYND8	3.87311686	3.09831743	3.92630174	2.93409423	3.52851059	2.31805047	2.44475355	3.18151091	3.4985539
ZNF10	0.29625504	1.05917534	0.69530256	1.43073656	0.40379796	1.03582646	0.49224542	1.33640133	1.27992255
ZNF100	0.85900352	1.23818402	1.58153725	1.0986265	1.43762611	1.15139145	1.28736965	1.24992358	1.61752874
ZNF101	1.57126258	1.14611862	1.82150688	1.38677597	2.14432789	1.34690222	1.01190582	1.15153337	1.23148864
ZNF106	2.15497906	3.79756507	3.0034046	3.05847302	3.57001158	3.4790691	2.71515352	2.60282449	3.08388475
ZNF107	1.9154712	1.53462307	1.51226992	1.34874829	1.78611921	1.24242042	1.22174758	1.2284401	1.70243132
ZNF121	2.3663325	1.38978929	2.60210511	3.11793124	2.68673523	2.13871131	1.81783454	2.31846265	2.45398449
ZNF124	1.1500295	0.76140792	1.75866811	1.25646508	1.79074186	1.00494972	1.76709925	1.67159757	1.87778415
ZNF131	1.89306743	1.86075235	2.20913576	2.39376204	2.47350966	2.05040757	1.8577727	1.92163298	2.24744636
ZNF132	1.07433894	0.88433916	0.94274546	0.81303638	1.2541388	0.71916672	0.34089491	1.02937472	0.4711191
ZNF133	1.1413159	1.31656511	1.62860097	1.7735377	1.4226136	1.28302392	1.30538256	0.79402587	1.70540796
ZNF134	0.65557851	2.22850175	2.03596681	2.05425923	1.35947838	1.56913346	1.20032951	2.61715175	0.44552751
ZNF135	0.09344289	0.73560956	0.32875583	0.52530059	0.19596174	0.39694688	0.25768778	0.09731938	0.08124486
ZNF136	1.34821591	1.62564354	1.63660675	1.65588394	1.75252939	1.71323073	1.28588429	1.68950309	1.6537886
ZNF140	1.14892699	1.50128619	1.60457229	1.89370437	1.85859332	1.81199835	1.10681238	2.4562059	2.05409845
ZNF142	2.43277521	1.91387119	2.4652465	2.4596504	2.6431114	2.66181854	2.39132482	2.72590345	2.43926007
ZNF143	1.93911667	2.53334226	2.44953945	2.68756176	3.10727887	2.80629558	2.61418438	2.64994982	2.58426074
ZNF146	3.95998835	4.15079314	4.28616819	4.78514248	5.21341995	4.5611176	4.17110896	4.53366491	4.93589321
ZNF148	2.71773925	3.08590776	2.67860262	2.85469467	3.18734946	3.11823982	3.19469019	2.94668459	2.98445498
ZNF155	0.81008699	0.5995897	1.1848663	0.87821122	0.90618629	0.867883	0.390494	1.19422741	0.59579288
ZNF16	1.69948266	1.10394245	1.72096854	1.53673058	1.18763587	1.35094702	1.68468827	1.60471039	1.33126882
ZNF160	0.24007701	1.81750351	2.13006278	2.38611752	1.76743024	1.82681938	1.13120379	2.06595613	1.8564138
ZNF165	1.83420046	0.71856808	2.12459945	1.45834492	2.12453753	2.0652106	1.93377579	2.22455457	1.98162512
ZNF169	0.39551596	0.44792192	1.06659617	0.71818891	0.85098268	0.49480122	0.45917511	0.4446836	0.90625983
ZNF175	0.59678146	1.22661138	1.36282579	1.01909789	1.09365377	0.98284848	0.49668051	1.4064345	0.7333445
ZNF177	0	0	0.0189889	0.00791486	0	0.00485234	0	0.0050896	0.01384035
ZNF18	1.2982727	1.67168124	2.16676749	1.97583745	2.0428999	1.63159722	1.17641935	1.11134986	1.60252428
ZNF182	1.43149513	1.59888554	1.86823061	1.60043036	2.29032515	1.48859036	1.27793476	1.44408246	1.68257253
ZNF184	1.94418931	1.87382027	2.71740967	2.33452325	2.57042982	2.42261042	2.43759831	2.50140222	2.47934599
ZNF189	1.93848214	2.64913024	4.57135861	3.0035102	3.64967001	2.6149867	2.293573	3.01618926	3.53725237
ZNF195	2.09423507	1.85224351	2.10511277	2.63118053	3.17176702	1.95761044	2.33551676	2.13211529	3.14489849
ZNF197	1.35848555	1.70967734	1.46667506	1.88968122	2.18757529	1.81262218	1.69161085	2.0459298	1.76379347
ZNF2	0.99459068	1.03315583	1.3649608	1.34690467	1.24540719	0.96347925	0.74945949	1.13989202	1.15456228
ZNF20	0.36996855	0.17513602	0.63225247	0.22863778	0.29290636	0.19591019	0.10153089	0.21656023	0.3178724
ZNF202	1.36526175	1.09599714	1.44336165	1.87203315	1.86184348	1.43129599	1.40835411	0.91606801	1.90928446
ZNF205	3.55921419	2.11600062	1.64641461	2.07940316	1.74461108	2.3411974	2.14836023	2.49527466	2.56376211
ZNF207	3.50007874	3.31870078	3.99512877	3.9719283	4.35745321	3.8506673	3.59171383	3.82232279	4.03608177
ZNF211	0.45953476	1.22758912	1.95039272	1.47610588	2.04989495	1.60634429	1.60149283	1.85950041	1.32010856
ZNF212	2.3084758	1.80404773	2.53336783	1.97774897	1.68942617	1.69349034	1.90932513	2.01378676	1.96201768
ZNF213	2.98696714	2.20517233	1.62783156	1.68309842	1.58493242	2.10784819	2.55222	2.06748683	2.16776163
ZNF215	0.07067734	0.47700931	0.61401762	1.2814721	0.36301922	1.07669289	0.1686165	0.76270128	1.03729991
ZNF217	3.61632961	3.24333948	4.3412258	3.46223449	4.08557309	2.90875046	3.33292477	3.5103046	3.49150429
ZNF219	3.74682097	2.42777059	2.01580092	1.79729748	1.60459903	2.19047305	1.22950533	3.14805676	3.16866601
ZNF22	1.75558919	2.94890105	3.11950238	3.15505165	3.21386772	2.9855669	1.85129194	2.58676328	2.87064557
ZNF222	1.14453365	0.71628474	1.49222172	1.17863396	1.44204655	1.21741653	0.46991592	1.36453292	0.76782968
ZNF224	0.66282681	0.8646173	1.42508689	1.49451068	1.85511914	0.92119264	0.50522112	1.20633211	1.25740511
ZNF225	0.52305358	0.71439254	1.00920515	0.91143383	1.07074515	0.72578983	0.31353917	0.98910644	0.65044847
ZNF226	1.27043924	1.19916069	1.79952391	1.34688168	1.93075545	1.16457786	0.65893766	1.41511029	1.29179177
ZNF227	1.49263255	1.45785992	1.84057065	1.65386896	2.01961128	1.73989848	0.74604206	2.02413008	1.16962063
ZNF23	0.15177294	0.0780632	0.18618374	0.17763842	0.2063496	0.10282851	0.09556104	0.17068923	0.2236005
ZNF232	1.49432604	1.04743824	2.22172192	1.71770079	2.24240023	1.69112182	1.6621592	1.14580152	2.47694085
ZNF234	0.92740957	0.98173782	1.56470883	1.23609223	1.65016632	1.16918552	0.47999055	1.63858166	1.00666991
ZNF235	0.52896344	0.40989222	0.8637322	0.67099103	0.89537856	0.78165808	0.21296856	0.71129915	0.52506187
ZNF236	0.94721457	1.46295678	1.15347056	1.83863434	1.58384293	1.67793577	1.71263528	1.31473285	1.52998967
ZNF239	0.99767238	1.03129783	2.32214098	2.25196321	2.92355953	2.27388918	1.32582228	2.35865921	0.91366263
ZNF24	2.24726011	3.05566081	3.85804608	3.42999504	3.6474126	3.45589327	3.72274736	2.98258551	3.59437525
ZNF248	0.86777337	1.35745468	1.40601633	1.4076422	1.15966393	1.18645966	0.86187236	1.30021714	1.67753668
ZNF25	1.1273132	2.4992612	2.16869838	2.35242237	2.02474294	2.26071816	1.44978257	1.44149605	1.19672155

ZNF250	0.75129753	0.95323241	1.18434891	1.25609599	1.03951626	0.92372807	1.02131739	1.26313891	1.27741944
ZNF253	1.99846147	1.84554433	2.07940418	1.61580031	1.9204544	1.51778636	1.94893121	2.2409231	3.07995505
ZNF256	0.30121882	1.06847219	1.08670934	0.55936682	0.69158306	0.61251493	0.43025121	1.65431115	0.45010014
ZNF26	0.41334466	0.47486137	1.1162569	0.78476961	0.57176967	0.52784634	0.37133591	0.88848141	0.63039938
ZNF260	1.87730131	2.45110721	2.55995349	2.59254089	2.85360921	2.39173711	1.89766384	2.99491323	2.8039205
ZNF263	2.63176562	1.97507278	1.83063215	2.12456196	2.23870074	2.02970673	2.1198918	2.40819983	2.33614397
ZNF264	1.5760428	1.88779501	2.04244952	2.03208623	1.69110626	2.12099068	1.19148824	2.21094155	0.42350364
ZNF266	1.50678006	1.56367489	3.25682602	3.23611145	2.91431833	2.26391145	1.98342579	2.65742657	2.92459583
ZNF268	0.32711142	1.18262929	0.92592906	1.28738969	1.30037851	0.68686564	0.87130176	1.97664312	1.20462225
ZNF274	0.8579408	1.30192061	2.04910983	1.97921688	1.35686155	1.45409012	1.00775709	1.53014683	0.95972252
ZNF275	2.550267	2.33880162	2.08566514	2.56916464	2.72798618	2.35461287	2.08351919	2.15148206	2.17133118
ZNF276	0.99014705	0.78476574	1.53825796	1.55513801	1.53630205	1.25077957	1.26596563	1.44566809	1.77356852
ZNF277	2.59027534	2.8900648	2.81051406	3.01225205	3.19185357	2.79116834	2.71545912	2.78030144	2.85380641
ZNF280B	0.37868558	0.43719841	0.70894709	1.04029652	1.24353926	0.48770625	0.08870461	0.37787177	0.89771598
ZNF280C	1.41877042	1.00617816	1.98898228	1.69906089	2.68112612	1.16725704	0.7417696	1.25629143	1.39215836
ZNF280D	0.6468058	1.69272328	1.66023644	1.91721552	1.35122003	1.49456016	1.06072574	1.05079381	1.66906545
ZNF284	0.60647654	0.34789582	0.97126574	0.67414178	0.83455105	0.28907549	0.0685425	0.48138003	0.26474128
ZNF285	0.22103095	0.26075095	0.45082357	0.08351897	0.09796654	0.14638845	0.01893966	0.72819023	0.03582284
ZNF286A	0.38179406	0.71232325	0.8586664	1.25371241	1.12567457	0.77788343	0.56475239	0.50391405	1.1447436
ZNF287	0.15490046	0.71468284	1.23021715	0.81272056	0.51807591	0.5181036	0.26219985	0.55006998	0.594681
ZNF296	2.15765784	1.23413561	1.76402456	1.68137762	2.25806619	2.25500634	1.65947738	1.90563129	2.67594762
ZNF3	2.94348627	2.7632039	3.05397429	2.86864476	2.60709501	2.12064518	2.40648215	2.7139323	3.60649867
ZNF30	1.17682408	0.61082874	1.1133803	1.53296478	1.45443113	1.00062849	1.08290569	1.2721617	0.15846134
ZNF300	0.08813727	0.77555544	1.37459356	0.77793491	1.15559915	0.51101574	0.41045807	1.10877303	0.10871605
ZNF302	0.52281781	2.0774628	2.40245317	1.97363819	2.8956965	1.86156022	1.28833643	2.02526677	1.79600223
ZNF317	3.02186344	2.86832853	3.2900123	3.40792453	3.79844487	3.17708494	2.83979668	3.42495086	3.35631373
ZNF319	2.32914934	2.09324464	2.0712875	1.70749846	1.90793531	2.27809664	1.75145736	2.49812124	2.3079706
ZNF32	2.57977374	3.8339735	4.56054618	4.19364786	4.24633403	3.96359189	3.24764249	4.29064353	3.5843039
ZNF320	1.53905068	1.06591933	2.16164063	1.70882603	2.03241021	1.5188719	1.79191497	1.90774266	1.99572991
ZNF322	0.87098257	1.5083508	1.78338002	1.08640132	1.0796829	1.24520258	1.62591854	2.23766565	1.64916872
ZNF324	1.47947894	0.85586512	0.95431934	1.00824636	1.12892436	1.18997116	0.99892458	1.08225614	1.15300107
ZNF324B	1.42958806	0.72068027	0.82546627	0.86486091	1.04449345	0.8156429	0.63222374	1.18080272	1.0525813
ZNF326	1.58206622	1.57339436	1.90428953	2.20852371	2.13436635	1.88548755	1.33651077	1.63758139	2.17216854
ZNF329	0.40320247	1.42171126	2.1585355	1.6338158	0.97666021	1.80592174	0.75816939	1.45052202	1.24755185
ZNF330	3.37344948	3.31504271	3.55215159	3.8645543	3.90310319	3.70825802	3.24021876	3.51830234	3.77857476
ZNF331	1.19815916	2.23434034	2.16611133	2.34572392	1.84628588	3.65628677	2.31898975	2.68427398	0.69011214
ZNF333	0.71529871	0.89328535	0.98724827	0.88936854	1.01337937	0.72028442	0.43748832	0.63702418	0.70498805
ZNF335	2.04863467	1.43644291	2.58176392	2.15704564	2.33788119	2.04474274	1.74260327	2.33573546	2.49111922
ZNF337	0.42611122	0.50602318	0.70077842	1.00501076	0.68760374	0.60825947	0.39664037	0.77231388	0.66313291
ZNF33A	2.18884651	2.64407239	2.64605251	2.97411331	3.01378177	2.82099551	2.51186828	2.96558975	3.36606454
ZNF33B	1.7793913	1.92504123	2.4616028	2.58166225	2.80242876	1.6695006	1.61898158	2.83174907	2.94829692
ZNF34	1.69077768	1.25917585	1.76484453	1.62309769	1.16778574	1.4105294	1.3359585	1.84384176	1.18071788
ZNF341	0.90683287	0.86131931	1.34394494	1.18397502	1.24071984	0.96531936	1.2048803	1.18744213	1.23742877
ZNF343	1.28473693	1.31776793	1.73081976	2.05311188	1.79365046	1.49526697	1.55796045	1.08962414	2.11400694
ZNF346	1.65332388	1.60605761	1.76020427	1.64680851	1.77442907	1.72230637	1.03930373	1.87493073	1.43457517
ZNF347	0.14421658	1.07132745	0.58466329	0.5408145	0.49121953	0.70507965	0.23941576	1.73915342	0.99405776
ZNF35	2.04960594	2.03272284	2.84846545	2.55099041	2.53406184	2.45018199	2.58835018	1.98737562	2.36557561
ZNF350	0.94817881	1.66346924	1.33795255	0.94729498	1.67858894	1.14974783	0.9581545	1.84533435	1.39797375
ZNF354B	1.06904417	0.97427912	1.62888551	1.7116746	1.68343764	1.06455971	0.93762604	1.35637795	1.65991048
ZNF358	5.06204425	4.40669696	3.23852366	3.6654814	2.80603412	4.02462415	3.25381731	3.45628306	3.88353672
ZNF362	3.75144328	3.80931055	3.41925271	3.09257662	2.54600683	3.19603349	2.6029817	2.8361529	2.8816982
ZNF365	0.07437009	0.13183938	0.22523639	1.28089563	0.02939324	0.20851334	0.11325705	0.06656049	0.02304054
ZNF366	0.39111041	1.58947753	1.2660098	0.63137854	1.02123636	0.84310671	0.55750769	0.56806327	0.41219597
ZNF367	2.44209747	1.64121633	2.19030839	2.36416535	2.80761579	2.4024517	2.01883573	1.42063164	2.55407565
ZNF37A	1.22390431	1.91759599	1.53121193	2.13623949	2.333459	1.96719168	1.43785451	2.42125915	1.85965734
ZNF382	0.07528328	0.2531091	0.39445242	0.21996965	0.23388897	0.23628884	0.11303529	0.17179292	0.09338915
ZNF383	0.92172548	0.88961902	1.17756996	0.80806643	1.04999427	0.77784936	0.55826821	0.98869904	1.05484
ZNF385A	4.16780663	4.36231594	3.84102729	4.3611759	3.77749567	3.82929833	3.33828144	2.6387814	2.24184302
ZNF385C	0.1675581	0.10214112	0.27922287	0.59579505	0.06002914	0.17904054	0.05836389	0.24618851	0.17564665

ZNF385D	0.04660532	1.2844987	0.28918397	0.58851329	0.04311869	0.87098452	0.06726624	0.09950375	0.04378846
ZNF394	3.20870386	2.24874002	3.00904578	2.17487944	2.43534927	2.43469621	2.54080469	2.52776929	2.26297733
ZNF395	3.09436243	2.70330687	1.90108116	2.6855152	1.78760878	2.55682514	3.2546718	2.69407218	3.36664422
ZNF396	0.01199395	0.40095777	0.84246616	0.56883611	0.21661045	0.40447042	0.52583033	0.40245707	0.20337745
ZNF397	0.9297134	1.45122067	1.84645665	1.89451599	1.67378998	1.46601085	1.53053266	1.33165149	1.8137072
ZNF398	2.33926353	2.23874726	2.7094561	2.27007293	2.25520146	2.02060791	2.76869832	2.21037957	2.34409191
ZNF404	0.64197675	0.6418703	0.95780202	0.68280553	1.24781039	0.44697804	0.18167411	0.92256242	0.26085255
ZNF407	0.57441184	1.11559925	0.7762373	0.96889918	1.29819284	1.25123887	1.16542377	0.8905084	1.29973296
ZNF408	2.92260972	1.57944664	2.38452515	1.96355026	2.14664101	2.20084949	2.50763697	2.63895218	2.32787419
ZNF41	1.35486727	2.14334307	2.18882257	2.25398029	2.720693	2.17800468	1.83329563	2.09167157	2.35272939
ZNF410	0.44744026	0.57793036	0.81313887	0.54341863	0.59974817	0.62844665	0.53775514	0.6182061	0.57247932
ZNF414	3.64751359	2.07770798	2.2077954	2.12898185	2.28249528	2.63450384	1.76617388	2.41265409	3.04030038
ZNF416	0.19907251	1.17630413	1.88230742	1.32715943	0.83502551	1.22263626	0.72719419	1.83746212	1.45529301
ZNF417	0.35887078	1.00363434	1.60837053	1.07256271	1.18250999	1.05938715	0.89226477	1.78381547	1.72772477
ZNF418	0.07878017	0.3845003	0.46587091	0.31708173	0.25116016	0.18670839	0.10738271	0.11972307	0.04607379
ZNF419	0.13874364	0.21738413	1.07092614	0.51371999	0.33166512	0.31563703	0.31451751	0.83036197	0.11048539
ZNF420	0.65782095	1.46781379	1.68022314	1.14113276	1.14960133	1.45518153	0.95584107	1.85254637	1.47115984
ZNF423	0.24868323	2.5201577	0.66929963	0.93204115	0.20007259	1.29064229	0.54482959	0.35524447	0.16101111
ZNF426	1.29861386	1.55943751	1.93445562	1.78258062	1.10680591	1.02561342	1.31770588	2.1042644	1.05020093
ZNF428	4.91976202	2.55962038	2.61636785	2.27295176	2.1768298	2.66412111	2.32693635	2.23237355	2.41624263
ZNF430	1.34842213	1.24349623	1.69108933	1.8854302	1.71784422	1.62740877	1.70486661	1.75202326	1.3926592
ZNF432	0.93166826	1.02315218	1.70469621	1.55693786	1.22756451	1.02790247	1.00221125	1.45795044	1.67446345
ZNF436	1.68971536	3.3134705	2.32958561	3.29575905	2.3752393	2.63247685	2.44035286	1.93935477	2.53330589
ZNF438	1.86645441	2.04732567	1.97761273	2.38787334	2.86039213	2.34008938	2.07408481	1.14659576	1.77706657
ZNF439	0.17758656	0.6011079	1.19745803	0.57433828	0.38388444	0.72768339	0.52458923	0.47397516	1.12479641
ZNF442	0.49327147	0.22331044	0.86801857	0.37330854	0.15537956	0.21194849	0.20405527	0.2720276	0.17551674
ZNF444	2.9650171	1.44838867	2.03952617	1.75185587	1.48731764	1.7124456	1.31580509	2.12247413	2.16043489
ZNF446	1.37335531	0.73743407	1.01398757	0.78015235	0.79884506	0.79831544	0.59738181	0.93637351	1.12346271
ZNF45	1.95427991	1.71195794	2.07572735	1.73348878	2.35812683	1.91130389	1.18062461	2.38034383	1.71062787
ZNF451	1.03092836	1.6621335	1.87944685	2.42384808	2.11004271	1.99163366	2.06122573	2.0256233	1.90384337
ZNF460	1.57628906	1.227566	1.21249369	1.26103677	2.20468749	1.47896891	1.69176717	2.05217442	1.06946089
ZNF462	1.22361564	1.7608702	1.46529346	2.09925261	1.54442862	1.11553043	0.39862414	0.22939868	1.21772201
ZNF467	2.89891419	2.25667726	1.98934558	1.5004723	1.11562528	2.32464032	1.86764338	1.38583504	0.92486648
ZNF470	0.14399672	0.75308423	0.82812177	0.89619386	0.2201886	0.65147019	0.41637962	0.09868264	1.16260278
ZNF471	0.02661786	0.81161351	0.42117875	0.6277869	0.24590844	0.51014993	0.18395211	0.1286913	0.08233258
ZNF473	1.68345266	0.854085	1.47265572	1.57725865	2.45015459	1.59440429	1.36032217	1.68590905	1.7956306
ZNF480	0.3760123	2.55144068	2.54271209	2.84213435	2.35497108	2.2018236	2.26749087	3.27371405	3.10852071
ZNF483	0.05606015	0.59155957	0.74629644	0.47329291	0.10922995	0.33761649	0.16846605	0.38076764	0.06527268
ZNF491	0.0588824	0.44140838	0.43844303	0.38395893	0.32366036	0.26915789	0.11076212	0.43982907	0.44730891
ZNF493	0.35030352	0.81346758	1.09270196	0.64734741	1.0447595	0.65178144	0.59943018	1.08879764	1.24416843
ZNF496	2.09813425	1.6068817	1.83526299	1.83606962	2.03886175	1.997842	1.9769076	2.80550773	1.66391004
ZNF500	1.79959983	1.39350575	1.25635354	1.25286271	1.21208288	1.05334157	1.10487454	1.39231043	1.58223912
ZNF502	0.6303993	1.2289602	1.63069477	1.39250227	0.64252707	0.6376809	0.82502712	0.2332874	1.90869385
ZNF503	3.35908124	2.86854571	3.50108453	2.67037066	1.04597795	2.92074367	2.2951611	5.32384239	1.1849138
ZNF507	1.30843574	1.64057009	1.55367958	1.73211105	2.1277286	1.76058663	1.4610866	1.7694274	2.1356506
ZNF510	1.20510309	1.85004634	1.97388293	1.78857758	1.88929711	1.525056	1.04077093	1.28521723	2.08882966
ZNF511	3.27728441	2.91863613	2.7726842	2.5374624	2.26282527	2.82930096	3.23574265	2.83783708	4.54484119
ZNF512	2.05828021	2.3798306	2.38528037	1.70819995	2.68040624	2.54473906	1.78580055	2.39124588	2.45589941
ZNF512B	2.98196589	1.62333195	2.39951627	1.8609741	0.80688548	1.4089075	1.25466936	2.76542051	0.46849958
ZNF513	3.31949261	1.66261321	2.63435073	2.13002068	1.94210998	2.30460454	1.02561594	2.95909289	2.77382142
ZNF514	0.60321864	0.95985894	1.42057749	1.44809833	0.88731136	1.03301011	0.78911443	1.35162181	1.20528381
ZNF517	1.51883635	0.92569374	0.92663833	1.00159265	0.69998355	0.89555559	0.98965487	1.37823084	0.91882128
ZNF518A	1.5599243	1.82819722	1.85098879	2.28599316	3.21997625	2.22676079	1.72701093	2.00297358	2.43365248
ZNF521	0.42329916	2.9160638	1.63901375	2.10680554	0.73136077	2.24556387	1.26866976	0.85385381	0.48880701
ZNF524	4.36301931	2.80091629	2.66775939	2.30271423	2.47656539	3.04283946	2.99083939	3.74411297	3.67303364
ZNF526	2.57447123	1.74614842	2.21035998	1.75293709	2.10107061	1.74663397	1.74913373	2.33850158	2.19191673
ZNF528	0.11492989	0.68767616	0.99477251	0.70868136	0.5901131	0.60005332	0.34421789	2.25368403	0.139056
ZNF529	0.18003024	1.20243706	1.81236283	1.32311315	1.38978713	1.39550401	0.86365757	1.66363522	1.59352155
ZNF532	1.94462177	2.55090631	2.43490172	3.00803703	2.77926065	2.7482985	2.88776774	1.70051782	1.18689932

ZNF540	0.13721379	0.33597838	0.43504433	0.42013217	0.21299795	0.17829866	0.0740964	0.28084461	0.16787957
ZNF546	0.10154465	0.5533567	0.87564198	0.56188715	0.48452211	0.42119379	0.17110073	0.45726589	0.23680889
ZNF548	1.17706219	1.25960876	1.79858	1.61124379	1.75773821	1.59428054	1.12446518	1.48977711	1.43873646
ZNF550	0.17519163	0.78450304	1.55275638	1.43201072	0.72654852	0.78300919	0.91521223	1.52870196	1.59969796
ZNF551	0.14002823	1.03409605	1.64691432	1.5129124	1.86546042	1.43456014	1.58680086	1.65549522	1.75578587
ZNF554	0.76635399	0.78727436	1.31278335	1.00437984	1.03070608	0.61442776	0.44177631	1.02351489	0.95256107
ZNF555	0.62039406	0.67529482	0.88330898	0.84418958	0.79320667	0.55868571	0.66281761	0.76340036	0.58464513
ZNF557	1.33076923	0.90149521	1.72986325	1.49353889	1.81928812	0.91597105	0.72097288	1.35228376	1.41461192
ZNF559	0.70977466	1.61153329	1.75566843	1.32537421	1.17008493	1.76882178	1.23605078	1.51122905	1.61246869
ZNF561	2.39874388	2.4803052	3.15137151	2.90664816	3.11270143	2.88887653	2.36732739	2.78507116	2.59522479
ZNF562	1.9583064	1.49341676	2.15481873	1.95923633	2.42188105	1.79394229	1.64931261	1.717019	1.97261987
ZNF564	0.32317731	0.25792181	0.53952572	0.32315593	0.44457706	0.2474046	0.29331102	0.30945871	0.34212572
ZNF565	0.76804211	1.02567851	1.2456306	0.90328856	1.21585072	0.83720642	0.53363518	1.06597664	0.91337141
ZNF568	0.1185726	0.61402832	0.6828652	0.3627445	0.31697067	0.42044475	0.17810463	0.92103415	0.08091655
ZNF57	1.66995182	1.40819931	2.02104126	2.41445805	2.79882921	1.91843642	1.95076764	1.80130249	2.817347
ZNF570	0.18588149	1.03686611	0.96320848	0.73750272	0.57129351	0.63901026	0.52184193	1.2181941	0.72107138
ZNF573	0.50611504	0.63448181	0.88939338	0.42156951	0.87424105	0.61958777	0.50184086	1.11704921	0.66040591
ZNF574	3.21109131	2.00588967	2.3127256	2.37358734	2.57511866	2.6340913	2.2624986	2.66973284	2.98550422
ZNF575	1.04155986	0.90400015	0.6987359	0.65431991	0.99287972	0.85328189	1.37090955	0.67383006	0.93105025
ZNF576	2.37296589	1.67196682	1.94762754	1.52868996	2.10561327	1.6839704	1.3411134	1.9749917	2.16926943
ZNF577	0.10157006	0.59738192	1.15488328	0.99591758	0.5551446	0.85476679	0.69902293	0.15171472	0.81591518
ZNF579	3.72673514	1.464806	1.90391249	1.63405753	1.51855583	2.0885545	1.54222076	2.81785843	1.8523658
ZNF584	2.04149044	1.35290553	1.72077913	1.60701656	2.04560487	1.7411581	1.26995061	1.7154266	1.50743359
ZNF585A	0.4870117	0.89280867	0.93048431	0.58101281	0.73964669	0.83253449	0.61478505	1.06576711	0.84686319
ZNF585B	0.74295584	1.48374545	1.36658277	0.98048684	1.34255433	0.991865	1.03786778	1.71415022	0.67710143
ZNF586	1.92173438	1.44683438	2.4386507	2.00753929	1.58934082	1.85655831	1.88707043	2.04788231	2.03374074
ZNF587	2.4690522	1.24740943	2.77299162	1.86451674	2.30460913	1.3945352	1.57990228	2.38599463	3.1022741
ZNF589	1.2297218	1.04366448	1.68917396	1.80316146	1.56374976	1.16719458	1.00964554	1.34156373	1.32791507
ZNF592	3.76419557	2.61431973	3.0876065	2.70011505	2.9559611	3.02783625	2.83265376	2.76211217	3.23651865
ZNF593	2.11935044	1.38144925	1.45671195	1.46003138	1.56035668	1.65734203	1.44573235	1.31517813	2.09723765
ZNF597	0.90351642	1.40792461	0.74729888	1.38307489	1.36465342	1.26349224	0.99688502	1.45767195	1.33331707
ZNF598	4.04888681	3.23549007	2.34711282	2.9501771	3.11662572	3.27817093	3.62439521	2.93397015	3.96325147
ZNF606	0.10581147	0.80932909	1.39781816	0.46169716	0.84392889	0.31821085	0.44410264	1.28370376	1.03960946
ZNF608	2.3479691	2.13786865	1.1198827	1.0470499	1.45306411	1.1314063	1.5802287	0.30931493	1.99800491
ZNF609	2.81986804	2.61727558	2.93083197	2.51821165	3.10304136	2.78160758	1.94731638	2.15516392	2.7355753
ZNF611	0.33516668	1.03321296	1.50893593	1.0779525	1.43082316	1.20069725	0.90263953	1.68289065	0.81387119
ZNF613	0.98611861	1.17241289	1.5175661	1.02618739	1.0952231	1.2404879	1.13082339	1.61962568	1.02954756
ZNF614	0.95507281	1.48013881	1.80829481	1.63898926	1.87543637	1.44258269	1.63799024	1.91341021	1.65930409
ZNF615	0.31313585	1.62006063	1.79201373	1.16402483	1.20729265	1.35270395	1.46778097	1.88343626	0.71827966
ZNF616	1.55680585	1.58634964	1.50569616	1.68786389	2.03082057	1.71666089	1.85548096	2.02841615	1.88060939
ZNF618	3.34576807	1.42551835	2.83789242	1.83956293	2.62782722	2.15851786	2.08462125	1.8453856	0.63926299
ZNF619	1.13593497	1.37292802	1.38134883	1.34736304	1.67942155	1.54621528	1.3773687	1.62763602	1.497642
ZNF621	1.62172342	1.67718058	1.98875781	2.21052008	2.08135958	1.9580641	2.08718483	1.96206878	1.79495594
ZNF622	3.61710873	3.80787162	4.15384345	3.45120149	3.73649061	3.73187948	3.63036337	3.40114578	4.11491133
ZNF623	1.59722324	2.15394101	2.49844785	2.64171504	2.02062073	2.38800174	2.22844512	2.78137368	2.21821286
ZNF624	0.45129053	1.09950293	1.38994418	0.94785832	1.12646092	0.79483556	0.62358734	0.47927776	0.87562091
ZNF625	0.15654886	0.10059845	0.16891198	0.04877012	0.02023105	0.01210039	0.00986464	0.12214499	0.08440077
ZNF626	0.15039981	0.89837041	0.40833141	0.43464739	0.35497194	0.46003531	0.18589877	1.46368315	0.11170918
ZNF627	2.1308226	2.05033153	2.92784272	2.54387122	2.38207102	1.97801163	1.84640778	2.27017641	2.56051946
ZNF629	3.39055113	2.93212347	2.36363251	2.69104773	2.31264119	2.44934898	2.47988474	1.91410336	3.1029022
ZNF641	1.21079962	1.65683358	1.69785304	1.98680294	1.63013587	1.80444403	1.16823279	1.95964365	1.05415943
ZNF644	2.02137723	2.86914376	2.85797218	3.40368578	3.2940397	3.05109861	2.45325073	3.2788212	3.32547701
ZNF646	2.35918687	2.17601101	1.8291137	1.57947484	1.81242354	1.87748599	1.74422226	2.08808933	2.38860564
ZNF649	0.41755875	1.68157379	1.98558825	1.422512	1.21802036	1.85078534	1.9439896	0.3698323	1.80130173
ZNF652	2.95149522	2.82581907	2.74838358	2.58021591	2.53722406	2.62988394	2.42953953	2.52326802	2.89009027
ZNF653	1.96361901	0.7479806	1.13007195	0.96037074	1.25746643	1.00880644	0.73883103	1.00204517	1.32216935
ZNF654	1.38265159	2.15661361	1.82354839	2.49776526	2.23007517	2.34963984	2.38249469	2.29267734	2.34871786
ZNF655	3.15223887	2.5339916	3.69929363	2.66161083	3.09726341	2.49039577	2.34602197	2.72323982	2.22003751
ZNF658	0.19009083	0.53423015	0.34633734	0.62423607	0.57690373	0.64119945	0.22864308	0.72717164	0.52200043

ZNF660	0.06563288	0.50353025	0.54553207	0.36314201	0.14109949	0.38873895	0.10961577	0.11996031	0.02911739
ZNF662	0.09054677	0.50252119	1.05902238	0.49216	0.15519372	0.46166214	0.12363733	0.08603972	0.07365665
ZNF664	4.7711282	3.73576355	3.94745969	4.54528869	4.2445502	4.27762039	4.25646622	5.20957297	4.95098517
ZNF668	1.67606655	1.13186707	1.01792483	1.02851467	1.07272444	1.38592438	1.35192905	1.06771773	1.32675793
ZNF669	0.29825011	1.68123491	1.96136151	1.48481252	0.75843832	1.65182016	1.84052399	2.96723799	1.7342611
ZNF671	0.44107139	1.22376604	1.26632792	0.94625344	1.35848769	0.96637613	0.57759375	1.55613903	0.41640105
ZNF672	3.59548901	3.06628272	3.05196812	2.92283777	3.56467931	3.63752149	3.51868314	4.10553306	3.54600546
ZNF675	0.15276124	0.8512003	1.65498122	1.21143083	0.85205157	0.85258938	1.16008557	1.21245463	1.69722443
ZNF677	0.06480041	0.90536848	0.53704215	0.57023826	0.38917924	0.6525247	0.26956208	0.17124147	0.08746342
ZNF680	0.13088987	1.50941426	1.80980566	1.66843532	1.72663608	1.51232324	1.55097774	1.75518758	1.91248552
ZNF681	0.06042146	0.72275636	1.16453947	0.80252337	0.28731664	0.75337911	1.30372649	1.21148956	1.32671939
ZNF683	2.61810174	0.40974003	1.39727838	0.30534744	3.25941627	1.41042172	1.13003604	0.37713716	0.61901709
ZNF684	0.96171691	1.06375154	2.08098013	1.02667602	1.112658	0.98801504	0.52394408	0.84619077	1.01609071
ZNF687	3.6093346	2.52517768	2.78450229	2.40122593	2.54924178	3.02217893	3.54917951	2.97892913	3.17189123
ZNF688	2.82786995	2.55168449	2.14112284	1.60193177	1.47570637	2.01533244	1.59019782	1.94499939	1.98088853
ZNF689	2.71452567	2.41079051	2.3225234	1.73491362	2.24430828	2.06434313	2.26070938	2.68140805	2.33581531
ZNF691	2.16159984	2.21153753	3.09250059	2.48743871	2.67552673	2.24434562	1.89466712	1.85241055	2.4387768
ZNF692	1.88014657	0.7273071	2.41141341	2.23007325	2.31863809	1.56366473	1.61491732	2.5689694	2.67057417
ZNF696	1.37463606	0.86577372	0.99398931	0.97328037	0.77059011	0.94342129	1.27108493	1.52888777	1.33104052
ZNF7	1.13736228	1.16773067	2.05492459	2.03409501	1.37215948	1.29519502	1.51718572	1.92494374	1.45065642
ZNF70	1.30895131	1.05688528	1.08422558	0.95696748	0.849911	0.87378187	0.53159864	0.71600131	0.77847828
ZNF700	1.78693962	1.64301592	3.00099473	2.44564281	2.89669382	2.06531858	1.70480269	2.37954926	2.21184817
ZNF701	0.17944666	0.98839082	0.89623887	1.12586601	1.53428889	0.79197965	0.80306057	1.14788366	1.42867059
ZNF703	3.77636939	5.03961168	3.70647174	4.33446546	1.57059226	3.63442499	4.54623965	2.60795843	5.81766806
ZNF704	1.36296065	1.64206341	0.85299618	1.54783234	1.48815672	1.21629198	0.61257087	2.15696566	1.49159215
ZNF706	3.40250036	3.47545521	4.24765178	4.1051444	3.64308698	3.51687389	4.09035164	3.44289727	3.76872532
ZNF707	1.50011372	0.63548094	1.41068003	1.07825569	0.73261698	0.9897394	1.12002816	1.12554846	0.77876199
ZNF708	1.45319894	1.21029653	1.61457749	1.35248272	1.45892444	0.90226016	1.08184696	1.5456606	1.90957209
ZNF71	0.49929488	1.15176117	1.1303389	0.9308563	0.47345166	1.32788831	0.67627662	1.40374356	1.25326603
ZNF710	2.95332678	2.1652864	2.67351058	2.20600578	2.6061224	2.43751958	2.29816409	3.95804769	3.50345982
ZNF713	0.55373962	0.37168656	0.84847505	0.46383759	0.31350895	0.26913824	0.35068444	0.58026443	0.42083722
ZNF721	1.17703085	1.69276459	1.38452206	2.05385702	2.18071763	1.82867735	1.19368712	2.20130522	2.0024807
ZNF740	1.82799059	1.43059457	1.89083463	1.71339834	1.89527469	1.61476012	1.48043274	2.05044035	1.83052177
ZNF746	2.51189711	1.68292148	2.16278531	1.72526762	2.01197239	2.02540505	1.94159098	1.81804635	2.10174961
ZNF75A	0.25610324	1.09004426	1.45331826	0.89161942	0.76442477	0.60764865	0.32775892	1.49680648	0.65058797
ZNF75D	1.63766896	1.30482819	2.39457306	1.66515665	2.64152961	1.34838794	1.06117071	1.40785664	1.67122434
ZNF76	2.1292591	1.51205635	2.05427064	2.0137576	1.72510312	1.79058118	1.49900017	1.86403748	2.26017742
ZNF763	0.39042216	0.47677353	0.4896911	0.71773014	0.48471998	0.40379631	0.20682015	0.43562243	0.57085179
ZNF764	3.03953713	1.64778882	2.40207988	1.50605665	1.53837497	1.62904663	1.64955066	2.23266324	1.84535898
ZNF765	0.09704473	0.75238614	1.17328666	0.98891544	1.27324621	1.03804511	1.26757395	1.19460574	1.05060749
ZNF766	0.33935157	1.84643206	1.75007052	1.92433967	2.01522941	1.88569468	1.60355852	1.88022233	1.97418201
ZNF768	5.49032089	3.62105952	3.45313862	3.28941268	3.12745527	3.36635494	3.42269347	3.86532532	3.74502757
ZNF77	1.42184398	0.77611234	1.76085278	1.39814227	1.78483074	0.97629202	1.49789222	1.45938423	1.49283735
ZNF770	1.93153141	2.72191316	2.48380241	3.3228009	3.36644178	3.31182108	2.82553367	2.46654104	3.27703924
ZNF771	0.99305273	0.8321628	0.85034934	0.71123921	0.47432175	0.82959169	0.71705581	1.33339989	0.81241325
ZNF775	1.83810567	1.2576212	1.54810756	1.04741358	0.98714886	1.24249397	0.86243845	1.41348147	2.40090157
ZNF776	1.60656533	1.55419909	2.06403721	1.94977855	2.23974952	1.80868313	1.87546731	2.44932763	2.09218527
ZNF777	3.46270535	2.3916096	2.65810816	2.48149169	1.89143765	2.7308605	3.18307839	2.75468781	3.02399411
ZNF780A	1.1871526	1.1633092	1.15367452	1.42766859	1.61513036	1.13699196	1.03623164	1.42054896	1.33669939
ZNF780B	0.24322051	0.98408316	1.35744153	1.425165	1.68794837	0.96765908	0.86419366	1.37166629	1.33253511
ZNF782	0.74650787	0.72075621	1.06065441	1.19037518	0.97303412	0.66756683	0.61045849	0.85511785	0.91444988
ZNF784	2.84283194	1.14819916	1.38626028	1.58999278	1.27597866	1.42587138	0.93442576	1.42196387	1.78195051
ZNF786	1.82723728	1.13382404	1.5864289	1.44307537	1.33482688	1.24694895	1.21834687	1.33911367	1.43127793
ZNF787	4.70578821	1.99416259	2.64040453	1.68942867	1.93323512	2.99700711	2.44047601	2.7824115	2.73020405
ZNF789	1.25306895	0.81733091	1.58449236	1.27885645	1.3136369	0.69919333	0.94858699	0.67170337	1.51788978
ZNF79	1.6607651	1.41453304	1.96117163	1.24854383	1.79390674	1.51131683	0.77906631	1.17116826	1.3184472
ZNF792	1.65439097	1.6299187	1.70620351	1.73254847	1.96347995	1.57933264	1.93952255	2.03394029	1.69424802
ZNF793	0.47864296	0.32824556	0.52382284	0.19395746	0.59146542	0.18990783	0.08296492	0.6046332	0.0439955
ZNF804A	0.24897155	0.27822486	2.02006811	0.22789578	0.45207385	0.56029091	0.30846863	0.18510314	0.35938891

ZNF804B	0	0	0	0	0	0	0	0	0.00408319
ZNF81	0.49865973	1.35204229	0.86163463	0.89518346	1.41482387	1.06008121	0.80476967	0.90621087	0.86345372
ZNF813	0.08499817	0.98061828	0.91952086	0.70153782	0.46866038	0.46523791	1.38433017	1.13179852	0.82534302
ZNF821	0.8963601	0.73424116	1.06653326	0.63451907	0.60548345	0.64342498	0.70592208	0.9825293	0.78088006
ZNF827	1.47320437	2.35124189	1.99437046	2.47543039	0.61839651	2.21407178	2.01094446	0.79531153	2.48364107
ZNF829	0.11747773	0.58222564	0.48619977	0.27203274	0.32106723	0.24151047	0.09540143	0.70381121	0.11870027
ZNF83	0.42206483	1.65631265	2.08004011	2.93529366	1.99609011	1.42343128	1.36546961	2.56183172	2.12738415
ZNF830	2.95181329	2.27108318	2.85773654	2.49980538	2.90809033	2.41662041	2.36384114	3.0538837	2.97198892
ZNF831	0.27123814	0.35619172	0.43638908	0.23940329	0.93370383	0.49326839	0.17253045	0.14077735	0.14890082
ZNF839	0.55580196	0.68786098	0.55915556	1.10586798	0.90410123	0.6850095	0.48580076	1.22076037	1.09934134
ZNF85	0.61894933	0.73678499	0.94898704	1.11907946	0.7627913	0.69278066	0.99134414	0.97198639	0.40521049
ZNF91	0.24742674	1.45400274	1.34850766	1.47728835	0.71819664	1.03551999	1.22263254	1.59586288	2.5545571
ZNF92	2.60535974	2.00277211	2.43615032	2.09898176	2.76656335	1.88791695	2.04624123	2.04224304	2.8706789
ZNF93	0.30770022	1.12313293	1.22446483	0.82036882	0.96961483	1.38418858	2.06146388	1.04799498	1.9779421
ZNHIT1	5.47884101	3.97663902	4.30093236	3.23735709	2.99109838	3.2550894	3.88394549	3.24837974	3.83698938
ZNHIT2	3.06182312	1.97269284	2.58564882	1.98522093	1.64203176	2.694297	2.61301215	2.86781056	3.12886839
ZNHIT3	2.14490426	1.73761334	2.03866102	2.5761784	2.2111707	1.97355506	2.15993572	1.91931868	2.06251876
ZNHIT6	1.80520315	1.76667885	1.83038772	2.43746969	2.06411509	2.22605924	1.69603309	1.48083504	1.92050264
ZNRD1	2.64364838	1.79197072	3.62201843	2.36055601	2.49153777	2.08853359	2.09416996	2.67234036	2.45618607
ZNRF2	3.44222286	3.42972147	3.31863225	2.7546904	3.23719465	3.34142725	3.68354996	3.01782148	2.91293138
ZPR1	2.962914	2.33068585	2.72638751	2.88999811	3.02531686	2.95546067	3.0368329	2.55003318	2.92756573
ZRANB1	1.47884079	2.16247742	2.1676755	2.14952739	2.05846776	2.18873479	1.8272007	2.16086236	1.61171064
ZRANB2	2.71042247	3.23465393	3.86294661	4.10917467	4.54115508	3.54890831	2.84790686	3.27114437	4.45935318
ZRANB3	0.50717016	0.75070666	1.00081643	0.67529674	1.09794555	0.53917358	0.87406485	0.46606501	0.82782228
ZRSR2	1.96601019	2.1779698	2.09678746	2.48656527	2.8916592	1.84115817	1.80922012	1.69004215	2.79077706
ZSCAN10	0.02179594	0.01252495	0.21346084	0.02611397	0.01346614	0	0	0.02100722	0.03422208
ZSCAN16	2.08842226	2.07156506	3.63463882	2.48523931	2.65998147	2.14331033	2.01039239	3.01477109	3.13078641
ZSCAN18	0.31767723	1.27063277	0.7111738	0.84436245	0.31879521	1.03347311	0.33378201	0.27616581	0.18582661
ZSCAN2	1.24632102	0.93183825	1.40597357	0.94085838	0.65466941	0.68606353	0.65965114	1.0086014	1.53200423
ZSCAN26	1.44953437	2.32507465	2.55696271	2.55912595	1.92254924	2.15668536	1.51403138	2.5094187	2.00383182
ZSCAN29	2.00297415	1.80179397	2.15553927	2.13507633	2.47942138	2.1082587	1.91263555	1.74276829	2.3921415
ZSCAN31	0.55468491	1.60842864	1.17579448	1.36091809	0.87338607	1.44203359	0.9670485	1.0513265	1.16882904
ZSCAN32	0.63009096	0.62035863	0.54080884	0.93656252	0.81564361	0.54165793	0.61831894	1.05803134	0.90321806
ZSCAN4	0	0.08121048	0.72176648	0.1477278	0.25550131	0.09225178	0.03828893	0.13694153	0.0584263
ZSCAN5A	0.76083372	0.58533682	1.20927344	0.62922749	0.88437706	0.62757451	0.75088397	0.53297654	0.82621182
ZSWIM1	2.3207433	2.25013057	3.53749523	2.99613871	2.63848625	1.94204862	1.97949078	2.54467847	3.23069145
ZSWIM3	0.90654382	1.43076374	1.57263023	1.25595059	1.2648496	0.78210969	0.79592191	0.9331067	1.86332843
ZSWIM8	2.98226646	2.50254084	3.13185998	2.53710527	2.68615316	2.89867543	2.22509031	2.73013349	2.92441638
ZW10	2.34905334	2.95083798	2.73301289	3.54572508	3.75085679	3.41248182	3.69464082	2.70537323	3.45168882
ZWILCH	1.96027839	1.99425296	2.73323616	3.23097826	3.52450648	2.84260579	3.01363632	1.76530385	2.85887705
ZWINT	4.62582598	2.69248053	4.3115719	4.06102258	4.04983246	3.93336335	4.16412535	3.70775182	4.0609761
ZXDA	0.22135976	1.17373944	0.81093001	0.83880212	0.46691696	1.0851327	0.82245064	1.31005594	0.11198017
ZXDB	0.26990487	2.16704915	2.14052405	2.24766071	2.75447623	2.12503837	1.9119531	2.65406384	1.78518733
ZXDC	2.90490618	1.63435933	2.15402514	2.06162537	1.83596379	2.17307106	2.38966506	2.04933179	2.13348427
ZYG11B	2.03668605	3.16894948	2.54757422	2.79531568	2.7901508	3.02536244	2.47263422	2.54354041	2.28233955
ZYX	6.66949409	6.53537207	5.98770308	6.17741343	6.0250714	6.97109537	6.34763608	6.04464893	5.98440896
ZZEF1	1.87940207	2.45945328	2.30189437	2.12934663	2.99397853	2.57715983	2.49105201	1.93661819	2.86370292

TCGA-BR-6	TCGA-BR-6	TCGA-BR-6	TCGA-BR-6	TCGA-BR-6	TCGA-BR-6	TCGA-BR-6	TCGA-BR-6	TCGA-BR-6	TCGA-BR-6
0.11581417	0.02853851	0.03350691	0.04936132	0.05594024	0.04974781	0.0112737	0.14340063	0.06338697	0.0292174
7.54246949	6.60469935	8.01097598	8.55519403	7.11588845	6.76098217	4.99337342	8.84777104	6.2631931	6.31698663
3.23891902	1.92982569	2.88957474	3.77370124	2.23125327	2.94220708	2.91773827	4.16010062	4.27896413	3.11684515
0.05025936	0.9793713	1.19584634	1.63901749	0.2464872	0.02522095	0	0.22263704	0.22568613	0
3.53107731	4.02326248	3.28943311	3.22451554	3.80480594	3.7519598	3.77071993	3.31566539	3.86389014	3.32930379
1.32827033	1.82049146	1.3710964	1.75547084	1.9187417	2.17176765	1.48462412	1.43786176	1.04020871	1.53222768
0.25041829	1.24714444	1.86349468	1.16534815	2.17488036	1.66392748	0.34948085	0.90741327	0.82751132	0.35662608
1.44112875	1.63942462	2.63382026	1.84004213	2.07235129	1.80424508	1.27086599	2.02833768	1.52862368	1.31935515
2.08476283	2.18126844	2.41478182	3.1242716	2.15185498	1.98758418	2.05169243	2.57119815	2.44495563	2.08100405
4.8813055	5.14258609	4.94213355	4.90125823	5.15116175	4.95021615	5.5528769	4.65910535	5.60939761	5.04870426
3.85840794	4.50850194	4.38329951	4.26190906	4.83157033	4.57395442	3.25984369	4.06522266	4.02243284	4.57627004
4.63246146	5.18101204	4.34408484	5.14912576	5.71779455	6.50630915	4.80504843	4.79729614	4.77971422	4.99344039
2.37233849	3.41639834	2.00913988	2.22360166	3.20775523	3.07121806	2.50673346	2.52785677	2.92744312	2.07028472
0.60924297	0.75824495	0.32250254	0.48332045	0.54211507	1.3820123	0.35134406	0.7974237	0.78966447	0.51078799
1.47078416	1.96683665	1.34484047	1.04964065	2.27581936	1.2786189	0.93478701	1.2093162	1.51388569	0.92188002
1.97745115	3.37725249	2.27263887	1.91356405	2.66420216	2.59364079	2.17464462	2.24123515	2.51582976	2.132695
3.9718104	4.37548911	3.72282588	3.59398056	3.63856544	5.85267344	3.73913604	3.71138709	4.41923662	3.93297805
1.94141466	0.38087731	1.22474463	2.45329672	1.02480345	2.42020876	3.05005725	1.80254735	0.56730173	3.21921794
2.05416085	2.73422961	2.61058091	3.04315789	2.75621191	2.22070843	1.76579955	2.83993122	2.5434793	1.31552888
2.05560708	1.74988713	3.58724292	3.26976346	3.54343305	2.92876846	2.76844322	3.56371799	1.86846679	2.87423761
1.63838663	0.80764689	3.43997446	2.46076582	1.52979156	0.90801645	0.53105511	2.78482494	0.84743137	1.5090499
0.04351602	0.04390911	0.08497335	0.08504717	0.06358957	0.01094703	0.00551795	0.12046185	0.01051272	0.0257209
0.73399894	1.4693582	0.58667803	0.44070903	0.44478331	0.55928993	0.36892194	0.45478168	0.88939421	0.77510169
0.53564109	0.24343621	0.44073398	0.69542158	0.53654173	0.38236274	0.06269708	0.96489554	0.2518448	0.11092926
1.94819946	1.59905441	2.41838701	1.62327637	2.97401398	1.28739518	1.35438558	2.91680907	2.09018563	1.86580171
1.50068757	0.64263187	0.80629721	2.14904926	0.87644203	0.33312442	0.04564599	2.42428079	0.22079445	0.19082637
0.52114354	0.51511604	0.65967508	1.12088641	0.4899297	0.49754459	0.0197091	0.9422861	0.26323486	0.05583172
1.8024593	2.36796612	2.37473179	1.78763328	1.99284802	3.34199485	2.24939373	1.98010495	2.31034087	1.85862223
0.73066256	1.62934662	1.10929304	0.66147561	0.75462004	0.63695624	0.46506829	0.77772594	1.10974395	0.70192997
2.83540938	2.69583053	3.23315969	2.59219328	2.91144843	2.74748349	2.53704019	2.72902632	2.36456721	2.76562364
2.21704084	2.48041849	1.42347975	2.30099529	2.1536415	3.15599388	2.93512923	2.79609545	2.075667	2.76683384
3.09696356	3.83884439	3.99714293	3.79246185	3.71676057	4.34269346	2.58777346	3.63090588	2.78882909	3.26031093
2.06688432	2.66976883	3.20865523	2.32466836	2.73825345	3.18047447	2.16842666	2.39319191	2.26723778	2.49252074
0.41771934	0.29499492	0.15341922	0.08505724	0.08311167	2.82561061	0.34459092	0.21662982	0.06116619	0.73361955
1.05564647	3.12621181	1.79477513	1.84740079	2.53075976	2.52841165	0.57565486	1.93195064	2.75062216	1.17625946
1.39596835	1.33226683	2.8752707	1.42041974	2.10682618	1.59525262	0.71853718	1.96165045	2.457516	1.48587935
0.9754436	1.40883517	0.79911994	1.20769816	0.60272613	2.15316158	0.8077145	0.95234512	0.69147473	1.48756232
0.013302	0.01131223	0.06437268	0.18665898	0.0111937	0.04901655	0.00999906	0.04642063	0.01902342	0.00348467
1.2827029	0.59714405	1.84456382	2.87127904	1.09256379	1.34616096	0.56038585	3.07153699	0.89219744	1.18642616
3.25820943	4.4255169	3.37009833	4.1033075	4.64992439	4.77356139	3.87241252	3.66801414	2.68727066	4.28581234
2.18019301	2.3640532	1.96396546	2.07683698	1.83075429	1.78006663	1.51783323	2.48327451	2.1316751	1.90315128
3.02002656	4.26124548	3.39376889	2.55124792	3.57851391	3.75892971	2.54179531	2.89960562	3.76844013	3.05257866
4.21911114	4.70613878	4.44286219	4.39464367	4.71297864	4.91576539	4.26388455	4.32677969	4.82122484	4.00062437
3.4178716	4.39925792	3.85782249	3.75763623	3.60372184	4.52107401	3.80155134	4.31762372	3.99523697	4.59393618
3.30075093	3.45753918	3.40727615	3.12746632	3.33045449	3.56726652	3.03302902	3.15505228	3.32302668	3.9230396
2.5622599	1.93599512	3.39462678	2.46045663	2.7453456	2.34104889	2.06107723	2.34128077	1.74909927	1.79229206
1.76742954	0.37534872	0.7833881	1.05985288	0.83517446	2.13060609	0.45022963	1.18587061	0.55593714	0.46681782
0.0704702	0.0182958	0.12724451	0.24395279	0.04484237	0.03898757	0.05323083	0.20066407	0.06079369	0.06992574
1.09671282	0.00720297	0.02882816	0.676227	3.82313642	0.26914286	0.53417395	3.03210264	0.06351883	0
3.25213364	3.26254586	3.12920116	2.62204615	3.27451642	2.77098325	3.40623507	2.74549998	3.11329651	3.52274167
4.76425725	3.66426294	5.45255452	3.68005606	4.37255024	5.60326696	6.33755845	3.89829159	4.05911089	5.36762251
4.91491305	5.56302537	5.20669363	5.01239717	5.71300143	5.38875729	4.67179919	3.84018657	5.42674171	5.30324557
2.29997106	1.89287785	2.10772521	2.07098324	2.33559497	2.70883874	2.14807013	2.06780541	1.93789991	3.4087716
2.99300626	2.91034596	2.02120388	2.8171839	2.35410093	2.38294307	2.76027341	2.91551177	2.42314784	2.82505707
5.63426075	5.10344832	4.05787123	4.91203322	3.71062102	5.41118206	4.87869504	4.78727825	4.95985066	5.58826629
2.96978737	2.91375099	3.31157394	3.05273913	2.99332073	3.53541685	2.37353172	2.93420963	2.42171545	2.24129008
1.16975194	1.38357644	1.10614303	1.18030412	1.38651502	2.1186423	1.22146807	0.95816459	1.73029171	0.98751507
3.91807411	3.7120141	3.92937119	3.81219532	3.52657184	3.25082793	3.55463179	3.87529878	3.71687625	3.35288974

4.33514834	6.33406868	4.36652015	4.4128245	5.06826193	4.36594318	4.61539644	4.27170023	3.81673724	5.98094246
2.44833343	2.78073758	3.18639268	2.29972917	2.97217205	3.16496118	2.24031767	1.88382333	2.57678783	2.44097293
3.57239229	3.5836395	4.06151638	4.50218534	4.25220415	3.49901242	3.256334	4.30475003	3.33520634	4.39810152
1.7939142	2.84515642	2.60576514	2.25143838	2.29120763	2.20126685	1.92369634	3.31604377	2.28297136	1.85595745
2.45833066	2.59447855	2.4235621	2.24174295	2.59411355	2.07272141	1.77817136	2.06717804	2.51416352	1.83459189
2.83900753	2.02793283	2.75357942	3.72093246	3.24769032	2.51249936	2.02810735	3.15929675	2.03346852	2.50401772
3.96319346	4.36703175	5.01046227	3.89111203	4.21780962	4.92018453	3.74072085	3.57646237	3.81431627	4.6305724
0.93662434	1.69370568	1.64732619	1.53204837	1.65080012	1.36604153	0.99133551	1.51166447	1.46852115	0.89742351
4.34585659	4.07357974	3.45480842	3.6174857	3.79955196	3.12523323	3.54969516	3.60883377	3.83024923	2.04150496
2.83445573	2.27487971	2.52299329	3.57063886	1.70499328	1.71881361	0.25314424	3.0712434	1.26877688	1.00779182
4.26305234	4.65130506	4.66334672	5.12307111	4.51095643	4.88715931	4.23623301	5.21913059	4.6461553	4.05967295
1.61507422	1.938621	2.41308777	1.88697555	2.23925185	2.1818737	1.01134965	2.71733402	1.73355336	1.47980341
3.83362481	3.71755194	3.79496758	3.60471531	3.9760179	3.8763874	3.21896635	3.70238853	3.45801664	5.59059747
2.22152446	0.23439208	1.46150669	1.44479497	1.8474723	1.20234885	0.64256395	1.4338904	0.29637235	2.32415474
2.10213236	1.25824312	2.05797115	2.45856057	1.3048416	1.30815711	1.21421523	3.30289209	0.79811464	1.78382525
1.41142982	4.34331874	0.5283794	1.32287095	0.78481151	1.31578056	3.23997237	1.81684678	4.10614542	3.09604662
3.86947078	4.00725869	3.79633487	3.56373821	3.66911519	3.93746146	3.49959757	3.87529194	3.69579045	4.31519576
2.95782229	1.66217641	3.23563792	2.82450085	3.12942362	1.95036279	2.87025576	2.69125333	1.56587492	2.6872086
1.75963031	2.49371394	4.07289821	2.6330017	3.39324921	3.38180521	1.95197988	2.8290065	2.32486268	2.64019
2.61181169	2.76121646	2.005903	2.49335279	2.62852956	2.46537244	2.53127148	2.6123991	2.46463184	3.02201918
4.78796432	3.65269701	3.89161806	3.59630169	4.94074792	5.09358273	4.08074282	3.71571945	3.68224829	4.56954286
2.07094379	2.44010356	2.38740953	2.32142917	2.44282713	4.59964622	1.84831142	2.82585552	1.95500229	2.73012593
1.65393104	1.25338757	1.55370574	2.30248445	1.92974134	1.0940668	0.54744049	3.56419443	1.14775153	1.70391043
2.20581668	2.57460613	1.67592718	1.91631354	2.14395295	1.96894819	1.57670225	1.6645196	1.9287147	1.78205082
0.03677585	0.06628558	0.06681073	0.06438396	0.09732009	0.07730452	0.07398212	0.0851025	0.07430432	0.09280735
1.70781574	2.41121893	1.3758026	1.31674723	2.07269952	1.68427632	1.31454231	1.33185853	1.98839675	1.39831212
3.39152484	4.15336632	3.33574546	2.98365347	3.36973235	3.32725703	3.23927559	3.28335932	3.5596166	3.91787238
2.77149365	2.89355483	2.59072281	2.77198483	3.26446963	2.09473653	2.54739245	2.70246931	2.8061222	2.03371235
1.43099037	3.75838637	2.2755127	2.30151137	2.37942186	1.83955365	1.5443676	1.82190519	2.18518449	1.62815151
6.02306289	5.5052541	4.607682	4.72859375	5.34989137	5.26129932	5.56112155	5.01901803	5.65993329	4.95229467
0.86028684	0.9185666	2.87677181	1.17866957	1.54190253	1.2802263	0.90532641	2.01553638	1.72416084	0.92265479
3.03010416	1.3952199	1.08147966	1.44532987	1.41878784	1.3699207	1.07257206	1.59658743	2.10621773	0.4760731
2.4188953	2.32863964	2.64201533	2.24835871	3.03972144	2.74861238	1.96956521	2.47208146	2.25973685	2.40045864
2.84778206	4.17896325	3.43310638	2.84151667	3.01545987	2.68567353	2.70386664	2.95948072	2.45532699	2.47778663
1.92937093	3.08050313	1.88607566	1.38027971	1.67311052	2.88052871	1.626244	1.34536304	2.66843819	2.8486878
3.67383472	4.36104954	4.11297382	3.86465438	5.2652814	4.47011852	4.30331948	4.13759794	4.09103482	4.13503841
3.03073939	2.85673209	3.69885364	2.86208712	3.24885967	4.23615789	3.51687989	2.64016497	3.43535157	3.14119054
1.53080869	1.79415633	0.53153273	1.03296777	1.89649681	1.14156328	0.55512809	1.22125873	0.8254716	0.90904595
3.26844984	3.5261251	3.52112316	2.98587057	2.95929103	2.93227984	3.33731934	3.13771579	3.21719286	2.9996314
3.02108015	1.81470677	2.74457435	2.82750423	3.02189368	2.94383718	2.01211599	3.53694449	2.29293805	2.18544474
1.37212699	0.17083546	4.29593731	0.38341132	0.32439301	3.1780403	1.64951537	0.68566832	3.2585859	1.84674916
2.25116855	3.26876232	2.70189356	2.54116189	2.5132584	2.01541552	2.24923953	2.10995482	3.2999622	2.01872366
2.13723386	0.48953422	6.358743	2.12195051	2.35249947	1.52781835	3.62153719	1.61943792	0.92005877	2.10559049
3.70587532	4.28790481	3.58421477	3.60460224	3.8795761	4.08136058	3.49173609	4.16106345	4.53627979	3.49605192
6.09287557	3.14423866	3.04945827	5.66052127	2.50149992	2.57369123	0.56317871	5.60923909	1.28582528	2.39860834
0.24327804	0.23639676	0.53110681	0.54490238	0.26050014	0.1560297	0.11546246	0.77522748	0.13579996	0.20895495
3.48361196	2.9633627	4.11415513	4.57687458	3.3848666	3.10444613	3.44660835	4.72904884	3.6192965	3.11168983
1.73127519	2.04128495	0.487781	1.49712859	0.82087765	1.32458171	1.91273856	0.96600255	2.70081836	0.35754161
4.44993989	5.07922496	5.18504509	4.68282372	4.71555489	7.18196946	4.30308326	5.11956978	4.3399269	4.6738767
0.02152101	0.05424603	0.0138649	0.01028198	0.10544549	0.10150834	0.00811705	0.22405001	0.1048413	0.02525094
2.86114483	4.02182782	2.791491	3.18161414	3.08980859	3.26626187	3.01470554	3.52469188	3.77088398	2.21901893
4.79893529	5.65311168	4.35215565	4.73511413	4.77904469	4.67246189	4.70190303	4.8434841	4.51492665	4.94415622
1.34446387	1.01441034	1.28483751	2.12067317	1.31889983	1.43549666	0.80915904	2.8092598	1.2314405	1.09672241
2.03644708	0.64773891	2.12945439	1.39918035	1.4888026	1.87572372	2.4893519	1.44730174	1.20656223	2.0383543
2.22995847	2.63583201	1.69742204	1.83241316	1.77189816	2.9035186	2.47124885	1.83693043	2.67003043	2.81010829
1.43358466	1.65091081	2.01235493	1.29804576	2.19943771	1.88395832	1.31625068	1.86306627	0.79609242	1.39930289
4.35017118	4.37613212	4.47635476	3.59111656	3.26920541	4.760708	5.53293835	3.1096217	5.02468997	3.43950104
3.04916718	3.31157039	3.10267119	2.73950665	3.43288617	2.84453638	2.6207698	2.20917602	2.6347122	3.08501694
1.96772087	2.51030833	3.70368702	2.50735138	2.51566917	2.91501724	2.49088586	3.05244621	2.53063457	3.13312879

2.83063331	3.44670446	3.87175182	2.99439198	3.5259391	2.94733396	2.56530289	3.04380906	2.90806236	3.04431483
1.9356339	0.6542423	1.27602651	1.7052561	0.7372016	1.11466899	2.58503038	1.69026622	0.36460943	3.03928441
3.64795004	4.08857052	3.71778758	3.41257175	3.74644599	4.06037894	4.56021541	3.44676515	4.45276187	3.72793397
4.00425086	4.60475557	4.45514506	4.22548736	5.08021886	4.34759247	4.51793072	3.89485004	4.20670905	3.85073013
4.94383059	4.8927086	4.50914823	3.530358	5.10530993	5.26337658	5.16726234	2.86289736	5.31477954	2.93254675
1.23211871	0.92518207	0.95684922	1.05026953	1.41308628	1.41965911	1.1250789	1.29253548	1.36016566	0.94492862
0.08540395	0.03690881	0.08239961	0.14589562	0.03652544	0.05369797	0	0.19348732	0.04142467	0.02278907
0.03264531	0.06179081	0.03262933	0.04807535	0.04778681	0.04048878	0.02728552	0.04672156	0.06174336	0.03126555
0.02604087	0.01112262	0.01678616	0.00831299	0.07533544	0	0.0260696	0.09955517	0.08528872	0.02718369
2.92969118	4.01230394	3.10529837	3.06630018	4.15239329	3.59663333	3.25797952	3.01892007	3.44016725	3.18379976
2.96865982	2.80024424	3.49752988	3.02974105	3.19920846	3.41833709	2.31626406	3.67512112	3.69266367	3.55735045
5.43120071	4.22982769	6.47895889	3.78904645	3.47429448	6.04369943	4.42766889	3.9196134	4.19451786	7.12201687
3.58464296	4.02097661	1.88326624	3.60944409	4.28552595	3.88134919	1.6377296	3.16386692	3.06232341	3.75332296
5.16134872	4.07557533	4.37987558	3.76503237	3.59582712	4.7094516	4.82143176	3.53896395	4.95212398	5.50329941
6.71752157	6.12042611	7.77668646	9.27799427	6.8806608	7.00439966	6.2472539	8.42423475	6.03376003	7.59480758
10.7221922	10.5575753	11.5115248	11.1548732	11.1129308	11.0229741	11.6168541	11.2270693	10.6177862	11.1625021
0.40578972	1.83383555	2.04229	3.14485368	0.2857869	1.08243789	0.31877248	1.64802445	1.39808983	1.69606263
10.3733396	10.7177276	10.2406323	9.93949495	9.91303241	9.82545175	10.8264027	10.287221	10.0364192	9.30764466
5.08248293	6.07393466	7.08816752	10.0578286	3.70698791	8.22508762	4.2691953	8.3098442	4.0906084	9.00458241
1.7139636	0.29116736	1.37371086	1.88795407	2.16410022	2.79909833	1.57327797	1.47770377	1.1837846	2.15681904
3.37877694	3.78563394	4.29485817	3.28193369	3.97991502	4.69659371	3.51349499	3.19247755	3.50530109	4.18469343
0.03742493	0.00803282	3.00645973	0.13757632	0.03153478	0.00939983	0	0.24555477	0.07068012	0.02939415
4.20676462	4.94378486	5.80191007	6.68337457	5.36250345	5.54179082	4.56207443	6.1501639	4.01871846	6.22644093
0.04303017	0.31454726	0.94595064	1.31269313	0.09009656	0.92448436	0.03242801	0.64668019	0.03096007	0.16164608
7.00519864	7.30685049	8.73288232	7.66684268	7.84099446	7.84466596	7.88921574	7.55114517	7.25585896	7.75828066
3.02779177	3.64568788	3.86758333	3.3459845	3.25280472	3.22219012	2.9085596	3.32008169	3.17175111	3.35050605
4.87800499	4.8832274	5.31445748	4.99542812	4.28945446	4.87771677	4.80286125	5.09317851	4.72968922	4.76667551
4.73663774	5.16986054	4.37421149	4.51529729	5.65988139	5.23658977	4.89446614	4.2875773	4.7613412	4.39903655
5.33096426	5.84018599	5.76475071	4.88207266	5.92856352	6.22432621	5.5947949	5.33510967	5.57125037	5.2645142
4.0515247	4.04467015	4.90000304	3.75133162	4.84834278	4.25566028	4.46355428	3.97545307	3.80371778	4.12130823
1.00038874	1.57081081	0.90691987	0.91040601	1.26437865	1.97143082	0.88983947	1.26681022	1.49418902	1.26871984
2.4604256	2.66937829	2.75846316	2.2482013	2.60864983	3.06676306	1.9258568	1.89043342	2.71226628	2.89062712
1.88553173	1.70791052	1.97605207	1.46493589	1.75913366	1.93426041	1.83486974	1.53224346	1.73124824	1.67851402
2.19608853	2.84345067	2.21141375	1.96355375	2.28756414	2.51218912	1.69147571	2.1187855	2.56187093	1.95694264
0.53488698	0.13723076	0.79721339	0.76165959	1.00988789	1.12173668	0.6741182	0.83679686	0.12398713	1.55861007
2.93164593	3.41750003	3.60630992	3.49239663	3.11565486	3.14203776	2.75315505	3.69339943	3.11667342	3.36639852
3.38476245	4.36377958	3.78604745	3.80212598	4.61005404	3.66580362	2.74889214	3.53811926	3.64387273	4.51086051
3.54492903	2.63240119	4.17341177	3.65125942	2.71821494	2.77673576	2.27718007	4.07013732	2.52908275	3.44838286
1.60342699	1.75853189	0.75855442	1.05191627	1.79366501	2.42458642	3.06543446	1.13222386	1.18201681	1.50650712
4.63375988	3.02528151	1.20371856	3.91909595	3.3300193	4.24570218	4.21809347	2.23661696	2.77321666	3.88200373
1.40507575	2.09100641	2.7213747	1.15928389	1.55017358	2.12682369	1.64004077	1.71695038	1.94510479	1.53870831
1.51136628	1.11874836	1.32391376	1.34761801	1.16287168	0.90170241	1.20226791	1.26838937	1.41215941	1.24710446
3.30757116	3.93700235	2.11080382	2.28165344	2.10110675	3.70616379	2.77287473	2.6338445	3.69081366	2.46998076
0.11296894	0.01860386	0.01408809	0.04134437	0.02297528	0.01634792	0.01098685	0.1555123	0.031232	0.01145894
2.19259296	2.65665254	2.71879747	2.16919373	2.90777208	2.88994904	2.75813654	2.3196749	2.95759526	1.93028189
0.11551375	0.11622137	0.20442915	0.3559072	0.13335936	0.1265339	0.08310572	0.2831352	0.27198793	0.04874298
5.0857205	4.37679887	5.09656484	5.22106594	4.99337701	5.39426483	4.80426179	5.60065032	4.23127928	4.55981749
1.79957111	1.99124508	2.68355166	1.92206725	2.53728081	2.53053949	1.48836771	2.49501844	1.56058965	1.82145147
3.27529676	1.93375596	3.93238049	3.0959113	2.43099884	3.51719454	2.50303019	3.59904279	2.37531282	2.4727498
0.65036122	0.17088079	0.75140273	1.1525208	1.47216429	0.20456026	0.37435627	1.01252368	0.75349186	0.15253864
0.67781083	0.54713275	0.88935612	1.52137312	0.48628019	0.62057278	0.28137541	1.27444568	0.32984047	0.45433445
1.82803255	0.8316042	1.48788099	2.85287391	0.60868203	1.24029895	0.72874349	2.88657001	0.19399921	1.12435323
2.93885872	2.1343947	2.6997646	2.66502783	3.92948531	3.12106858	3.41344275	3.16678005	2.23313978	2.69670489
3.43813663	5.35276481	4.00221216	3.95655213	5.45798599	4.21228827	3.73451542	4.02461138	4.87780271	3.62537388
3.41722071	3.5082933	3.32610932	2.0741488	3.84244868	3.00595385	2.99175024	0.92523655	3.07124343	0.6766009
4.18331536	3.1534715	4.32375511	4.92122283	2.98987515	3.11896307	1.70108309	5.22042239	2.26433892	1.98510903
0.4034261	0.9132919	0.52896524	0.30312255	0.53233911	0.84928328	0.19994607	0.78644079	0.9046863	0.51096382
1.98081123	0.97232403	2.28588731	1.54807538	1.45861929	1.33705859	1.65817045	2.88174926	0.95539473	2.14773443
1.49313959	0.79768139	0.80067881	2.36858834	1.204891	1.20636125	1.57015783	2.76175216	0.15187352	0.33347136

0.22038797	0.0300383	0.21195632	0.77445472	1.23754452	0.24341027	0.20240396	0.82203249	0.11594761	0.12628908
2.08769293	3.50317345	5.3956368	3.82334892	4.34260792	4.98827113	4.91827003	5.12267691	2.56737021	3.79995457
2.12155579	2.13958593	3.14616364	3.25734445	2.33628141	2.70492614	1.98956297	3.59440408	1.71155716	1.71408535
0.30964165	0.22038275	0.66776585	0.32589191	0.71584224	0.57564512	0.23173614	0.63938598	0.20667158	0.29593851
1.00625273	0.70846169	2.125154	3.54539331	0.20381646	0.7837504	0.15208179	2.40931204	0.44854932	1.98842822
1.50542419	1.43893607	2.68700652	2.83708536	1.86896047	1.12343373	1.04381531	3.10753354	1.40832904	2.48041737
1.49431431	0.9526238	1.41657122	1.77140049	1.10445185	0.49062747	0.07693265	1.22534767	0.50711966	0.6612407
2.2796676	1.51821917	2.97057613	2.71687118	1.77284562	2.11553569	0.89525518	3.03864056	1.09286826	1.45848713
1.40661412	0.89611455	1.96789675	1.02754721	1.08750583	1.75063059	2.58551266	1.87304537	0.21061739	1.69320235
3.18113395	1.26415962	4.07931335	2.54891888	3.50875667	4.11073516	4.27672253	2.35082529	2.59067981	4.2390967
2.8790221	2.89840443	2.90849676	2.30491462	3.24147964	2.37670359	2.69151013	2.63252406	2.65581901	1.28055276
5.20643319	5.67477193	5.8428547	5.32569382	6.20389167	6.18964027	5.29908083	5.81922086	5.24239282	4.99616799
1.90800825	2.34467705	1.90248259	2.44273084	1.73697056	1.40271202	1.0477006	2.69219774	1.71344122	0.95950591
2.15149954	3.45548612	2.38254457	2.09359171	2.83657927	2.57292354	1.49284535	2.46986322	2.51422984	2.28484243
0.69712921	0.97442685	0.89548413	0.44751035	1.12740288	1.14838349	0.6176427	0.7303075	1.52648852	0.86041982
2.93224078	1.40692102	3.23506135	2.16975557	2.0676258	2.10030552	3.11495744	2.26573797	2.08093495	2.86529903
3.65236585	4.10248196	4.33388844	3.32276131	3.28863221	4.50909797	4.14176187	3.53305472	3.55052461	4.32086804
3.76726246	3.43261274	2.85949615	2.79995595	2.57246674	3.67426878	3.22224156	1.41711044	4.10045354	3.07459116
0.35261584	0.13046158	0.42533462	0.69188751	0.46207299	0.25289571	0.1327667	1.17136856	0.20531346	0.14930225
0.05406399	0.09757359	0.41690843	0.57595006	0.06371574	0.24297124	0.16472865	0.44005964	0.09313776	0.35130222
2.8468122	3.4033079	4.06177798	3.73094542	3.74850106	3.76364553	2.52793641	4.27334415	2.69267961	3.53213815
1.61834388	0.87709745	1.37122666	1.4296967	0.96873857	0.9766644	0.41054648	2.4980763	0.55789532	0.70836465
1.71610961	1.45540555	4.00698815	4.02792897	1.0029259	2.03847723	0.15947564	3.20011247	0.58516406	1.97583098
2.04804724	2.62763323	2.80124351	2.56661679	2.83676367	2.74907693	2.05056632	2.92047419	2.42155613	1.94903683
1.59108973	1.71225559	1.88764608	1.40365768	2.68122634	2.00788398	1.24552959	2.03526875	1.58572103	1.33420778
2.05377863	1.51903838	3.27092788	3.41044509	2.85648628	2.5851748	2.00843195	3.06651366	2.22467689	1.72494177
0.9316975	0.40328595	0.90255467	1.61968614	0.19621309	0.21420138	0	1.11003549	0.01132595	0.03074449
4.43534336	4.69893959	4.1558865	4.6719243	4.22326586	3.95240161	3.54984311	4.44866563	3.98331105	3.83310316
4.5221422	5.94640962	4.01998899	3.11875287	3.69684941	4.81469588	3.97244048	4.23847321	4.46291734	3.9573941
0.37471933	0.21350199	0.03396365	0.3487678	0.05785462	0.01981853	0.01000495	0.65812805	0.03782128	0
4.47148451	2.03619909	1.87384507	4.06833783	2.33460607	0.58752766	0.05120274	5.78712937	0.85153816	0.56302864
4.56029763	4.62904319	3.08081514	5.61846778	3.80953363	2.40563038	1.72522885	3.24994988	6.55059163	0.09828502
5.47470688	0.03870151	0.08447321	0.37901697	0.03829976	0.12317174	0.3217275	0.2997183	0.05196536	0.47255442
4.53504445	4.67497223	4.12385797	4.40834434	3.79562481	3.82288828	4.09627573	3.95085084	3.98945923	3.6608821
3.3819677	0.62812693	0.11028515	1.0131931	0.16344673	0.69368714	2.7345576	0.09375987	0.60851291	1.1321312
0	0.04270387	0.02475416	0	0	0.00723073	0	0	0.08119462	0.01513757
0.43465694	0.2917132	0.44970268	0.70673692	0.25954183	0.33611566	0.05340642	0.96025552	0.34698469	0.2747787
3.88521374	3.77466282	3.17503432	3.5481545	3.04848907	3.71363602	5.18946357	3.46884759	3.64875264	2.7677931
1.73829939	1.10296597	0.31260376	1.43367012	1.00924162	0.02421522	0	4.94408032	0	0
4.96872323	5.25994747	5.96294234	5.37677489	5.79316248	5.50364018	5.41840319	5.35475392	4.77912804	5.55131774
3.24447239	3.570337	3.78798868	2.94796672	3.54503732	3.72938627	3.41826699	5.04860501	3.24788966	4.52612874
0	0	0	0	0	0	0	0	0	0
3.15283038	3.13896788	3.34007355	2.89239147	3.79228278	3.57384012	2.5836017	2.69337333	2.90485703	3.0495083
2.16359221	1.50606967	3.08834426	1.85745222	1.72322448	1.52833293	3.67540937	2.06982376	1.87581734	1.94579283
2.50686232	1.34008463	1.69698072	2.63631723	1.81378239	2.61873636	1.50082674	1.34025013	0.57621421	3.27194499
2.23062568	3.0991573	2.49193888	2.36575907	3.02227708	2.76794724	2.1685536	2.37846222	2.3567109	2.09176124
3.05995963	3.5711758	3.87036197	3.3456947	3.99604364	3.34147299	3.14429942	3.52062116	3.11376596	3.17049821
0.35558584	0.16053981	0.08096632	0.20416589	0.12602798	0.12843744	0.08910237	0.23412929	0.26334681	0.04988559
3.00773783	3.50404064	3.02374218	2.34595287	3.67618106	3.30312948	2.6293499	2.99409316	2.92762805	2.53479778
1.79930139	1.21107445	1.79892635	1.81231077	2.02962963	1.63446649	1.07023111	2.48948794	1.08690482	0.93031661
1.42380472	1.11814932	1.1698856	1.42649955	1.81879705	1.95964039	1.22127779	1.50686801	0.66306398	2.26468661
4.01962894	4.1713234	3.97237889	4.02448955	3.99667018	3.97773188	4.42872759	3.6892786	4.55186846	3.60447129
2.68965683	2.38287244	1.56912563	1.8506931	1.47728852	1.90585364	1.79635269	1.89571646	2.53929183	2.0127202
0.23954577	0.10668117	0.42269665	0.42635372	0.60785665	0.10014547	0.01298494	1.034593	0.22964751	0.14240342
0.60887903	0.30923191	1.14073253	1.68083081	0.34442854	0.76524056	0.06358084	1.21661069	0.17502579	0.87980533
4.07643831	1.04860891	1.37930675	3.92816302	1.20413847	0.98240468	3.16003805	3.5385304	0.81617659	4.87372434
1.55585758	0.89298767	0.8349058	2.63687074	1.12508687	0.43420797	0.83675789	1.48968321	0.47025625	4.44770988
1.49438611	0.25545239	0.81046994	1.23853386	0.57967706	0.56635686	0.21379604	0.95913496	0.34484381	0.47548144
5.84086339	6.28886326	6.59129519	5.85603515	6.39937923	6.46094536	6.22381133	5.70618005	6.35421357	6.53136426

3.16460711	3.9274396	2.47331161	3.02078428	2.87515249	3.44426148	2.75613177	3.25129114	3.00802544	2.61954466
3.23139052	4.05139021	3.17227223	2.96399211	4.25184864	4.30077139	3.30405008	3.52602274	3.36356376	3.28210065
0.64979944	0.25861503	0.83002382	0.83149555	0.48538181	0.33295997	0.85882529	1.52207295	0.35074951	0.45609089
2.62620361	5.65881009	0.40845608	1.89402844	0.76526537	2.90227538	2.75115548	0.45197622	4.06636598	0.16155396
6.40146612	5.8490589	8.04155708	7.92031497	8.13015945	6.71485462	7.89636461	7.31456602	5.48445712	6.2693627
1.99529501	3.46714295	2.38039572	2.28766234	2.65854652	2.80094491	2.10417994	2.41966493	2.99171977	2.55850007
2.66324738	3.48660866	2.51806398	1.81647485	3.16016384	2.6250582	2.27215514	2.52687897	3.1957392	2.80573875
6.49275571	6.15989437	6.28534146	6.10843786	5.62067111	5.7119776	6.21821801	5.8061072	6.40651714	6.41046601
2.68390229	2.56535529	3.63752167	3.52698808	3.55537173	3.36222342	2.34088669	3.7844446	2.94025926	3.2707264
1.52807229	1.47948888	2.15266741	2.06397335	1.83223081	1.44889824	0.89689834	3.29278075	1.58847228	1.30169411
2.07637385	1.00000869	2.08814105	2.0763004	1.95504535	1.50439096	1.15327107	2.64176118	1.0868728	3.29987391
2.43422877	4.08458557	3.60502604	3.26613579	3.24901837	2.94978827	2.45839102	3.45118445	3.12156135	2.75158881
1.65571582	0.28802838	0.4130564	1.05160517	0.18100667	0.15895125	0.06609605	0.67575962	0.11424246	0.06710921
2.45011005	3.49034362	3.76674469	3.02304263	3.76767999	3.19141333	2.38868481	3.1410255	2.65563085	2.6279133
3.31753108	3.52753919	2.69624305	2.69376838	3.29087903	4.04775344	3.22864212	2.91944646	3.07464109	2.78074271
2.23000045	2.89698061	1.69848435	2.54769276	2.26062101	1.78161666	2.19625417	2.02365815	2.51391953	3.1105053
2.59468228	2.95017272	2.88399416	2.19809677	3.0344235	2.99654759	2.79093972	2.12030569	3.50703074	2.4442551
2.68295654	2.47318131	2.12698507	2.66428702	2.60689931	1.84244387	1.60049138	2.25257017	2.04929158	2.06569085
2.06152625	2.56437265	2.55351974	2.85235083	2.47196389	2.42396567	2.25964151	2.64350655	2.10481076	2.50884685
1.81794487	1.34622257	0.88396092	1.4885193	1.3625262	1.18245953	0.98956959	1.42620722	1.34862535	0.41023009
2.44385251	2.8444188	3.17553317	2.64401454	2.67196834	3.64873377	3.26990146	3.141451	2.67039556	3.58094763
1.93226422	2.83397819	2.21090313	2.88502241	2.85415104	3.18622295	1.90394051	2.58088971	2.45384771	3.23296314
1.23277721	1.0779521	0.60825485	0.62017885	0.9705213	1.0893622	0.40633433	1.00563113	1.81242089	0.69203536
2.85051869	3.47588259	3.49850866	2.84061105	3.65160553	3.30052415	3.16600251	2.85766563	2.91103341	2.75685175
2.87913358	2.69679409	3.96331522	2.78160771	2.45328563	3.21920102	2.78067526	2.87109359	4.25538432	3.87640271
2.59611728	3.02048732	3.25003205	2.3948279	3.06682578	3.03409845	2.55989344	2.62254038	2.6046083	2.80205551
1.84752033	2.82781524	2.14196072	1.83629989	2.09147647	2.65043577	2.35911772	2.32906188	2.38368891	2.56061978
1.71931185	2.38743215	2.28615244	1.78921484	2.50844727	2.13551988	1.57914783	1.51894534	1.76407145	1.60622344
4.09714842	3.89663683	2.98595984	2.73143732	3.17641241	4.35722425	4.1182855	2.81927965	0.64504829	3.85731509
2.16833002	2.90563214	2.15039616	2.43444818	2.44364596	2.52734698	1.7218556	2.50012583	2.33877248	1.94174465
2.36617267	3.52384605	2.79034309	2.66003289	2.89749471	3.62534121	2.07809003	2.86468287	3.42585117	3.12380458
0.84367682	1.15013247	1.17594812	1.10701059	1.4215109	1.06994868	0.51755049	1.27898444	0.96740886	0.73927414
1.62393171	2.08459631	2.44436549	1.80955501	2.23311526	1.97099859	1.09875233	1.79511696	1.07164996	1.81351237
4.15156039	4.80456546	4.44393243	4.69888067	4.42683321	4.46736479	4.2732129	4.69021157	3.77550249	4.32489418
5.85157131	5.74831537	6.93868313	5.10302949	5.9241912	6.46385816	7.32047078	6.15231319	7.03246515	6.13569893
3.20887696	3.91584907	3.95236153	3.03220156	3.3292155	3.47539221	3.29562434	3.37647569	3.58929758	3.70946823
0.64012137	0.41152086	1.55121619	1.17097248	0.93516842	0.80125301	0.7676008	1.41432573	0.37956477	0.60550904
3.56442001	4.36285189	2.59071447	1.74123259	3.34051389	2.64792578	2.96347089	2.25865289	4.41681832	1.94299463
2.52774062	3.29594662	2.80109784	2.42211658	2.90910582	2.9164385	2.92638948	2.96713327	2.57908161	2.64227013
8.36132448	8.84801013	5.4689222	8.94449599	9.08232647	6.95825422	9.39229214	5.32861636	8.79569307	5.2064307
6.05153846	2.81495821	0.45517816	3.53115783	1.94280231	4.48054155	6.62330803	0.46689758	4.27850448	0.39052381
3.59462523	5.65343951	5.60479856	4.88090405	6.53253587	4.22748614	4.71335468	5.2408381	4.97224828	5.44638668
5.43336476	4.04379229	4.62272288	6.12803918	2.3672245	3.60630222	2.37593041	5.40326523	2.68826773	4.6771336
0.49484763	0.32993789	0.48758498	1.45775816	0.18955284	0.73899421	0.13670967	1.31436679	0.10782462	0.86396676
3.7313718	3.96184285	4.42026301	4.41470425	4.07506458	4.22005417	3.89412181	4.04739573	3.73906219	4.60075657
0.3150533	0.00430931	0.03863468	0.1293646	0.00426404	0.00504377	0.08393859	0.01584823	0	0.36370721
1.68187348	2.82208369	2.32139101	1.76399754	3.26693193	3.32265933	1.77477783	2.27551698	2.39764363	2.0093961
6.02721571	7.22702575	5.95070122	5.56733788	5.61215815	6.6321689	5.95554544	5.19273079	6.57595828	5.9596244
4.94071643	5.03362098	4.86170139	4.87317871	5.07504363	4.896271	4.49167661	4.38453678	4.87079247	4.7360451
3.26935623	2.15898509	4.83823523	2.71162474	2.196453	2.53890688	4.33043169	1.89882242	3.36410335	3.57708992
2.77758091	2.1783061	3.06891875	3.87954969	3.24338972	2.8573723	2.51223363	4.00144422	2.77863383	2.63838453
1.34405717	1.34046135	1.6737598	1.14287487	2.01109926	1.29143325	0.88486642	1.2700322	1.18564974	1.1797394
4.68783215	4.59578914	5.7297296	6.15820167	5.91071445	5.26395374	4.65587228	6.38654463	3.96297995	4.65665247
3.98912598	4.30317459	4.93227648	4.35655012	5.33626587	4.58235614	2.81296934	4.37168948	4.20381805	3.72762936
0.50009502	0.37719366	0.16908764	0.79642672	0.51923039	0.07155323	0.04648385	0.28843016	0.32639763	0.17388256
4.68515084	5.419822	4.84991106	4.32682585	4.2919729	5.36645254	5.36028593	4.65776305	5.58916341	5.13934235
0.20032017	0.03384181	0	0	0.06622005	0	0.11620439	0.12119913	0.21422826	0
5.22853727	4.58547373	3.61654144	4.05540507	4.53911499	4.29566138	4.73813295	3.5757517	4.99891813	2.60571991
2.57828933	1.10796535	1.71985659	2.59297432	1.59811832	1.45816325	0.45175634	4.29303389	2.52594774	0.69203669

4.07963581	4.14631987	3.83718133	3.14188452	4.02242132	4.6905615	3.87054814	3.68969208	3.72846464	4.30749391
2.70503089	3.53364224	2.679642	2.61171268	2.52681428	1.96328757	3.05770849	2.36364838	1.37582078	3.32066261
2.06971853	0.99225653	1.96282155	2.33211646	2.54805432	2.77677439	1.64137545	1.14635985	1.18194169	0.2079174
3.82052152	4.24281018	3.92396915	3.428225	4.62668829	4.46961732	4.30241375	3.80699751	4.25877952	4.43716193
5.30143945	5.40208876	4.30273032	5.63073818	4.6594948	4.81980928	4.60923836	4.87530892	5.00551873	4.86814398
0.13543146	0.03655628	0.05692101	0.04237355	0.01215992	0.00480803	0	0.03748277	0.06332952	0.0200799
0.87425191	2.78444472	2.27875985	2.37047408	3.11362735	2.5872527	2.26468449	2.03178836	2.48785556	2.92054474
3.98249686	4.85604379	4.06065341	3.75716749	3.8955964	4.28750653	4.38488917	3.75812854	4.93799337	3.78802314
4.29884107	4.49131179	3.71415854	4.20858673	3.70406196	4.35928808	4.22421877	4.03687002	4.36879398	4.05958455
2.18283903	2.23210842	2.41310213	1.77327655	2.29421734	1.1810091	1.56939737	2.4164029	1.58422636	1.2653129
0.52958261	0.82276125	0.60597523	0.45507081	0.69325372	0.48278947	0.21833453	0.38656985	0.47409042	0.53245248
3.64127003	4.73793289	4.71617153	4.51638492	3.99117855	5.02525728	3.97728675	4.21136161	4.22214075	4.65081677
2.21652079	2.80127772	2.37835013	2.05361087	2.54677912	2.1847612	1.65039891	2.43676555	2.40407688	2.02142374
2.36272085	2.44004024	3.21238705	2.70664783	3.11497979	3.3500154	2.4061558	3.06489415	2.2738989	3.48563219
2.43503442	2.31477224	3.8194433	4.27963344	2.52364561	2.42393754	1.39828421	4.15287805	1.31356567	2.35398805
3.20919976	2.71409362	3.30070116	3.31981033	3.38702748	3.16011946	2.48126224	3.64926745	2.91437382	2.50870167
0.0754113	0.01635475	0.01648658	0.04830627	0.0791618	0.1463657	0.14723146	0.11662144	0.07212379	0.02007138
3.22664797	3.54648578	3.52358955	2.80791133	2.96678216	3.42717859	2.970972	3.27563464	3.20365778	3.22876348
0.32831191	0.17930313	0.33926171	0.58412042	0.16029356	0.19534715	0.06484019	0.58609084	0.09064348	0.08752565
0.23638605	0.27428053	0.69081863	1.18200422	0.34028076	0.55709445	0.04408938	0.70354807	0.08093872	0.68637652
2.18533357	3.84886742	1.20586382	1.12943364	1.54567585	1.06179218	0.77839533	0.84466439	0.60930361	0.43763905
2.85800635	3.09560993	3.53587881	2.54844096	2.98247997	3.71601808	2.51561597	3.11124802	3.27247107	2.67265319
2.65934268	2.83866431	3.13130455	2.39007247	2.72880525	3.11676997	2.08546625	2.77304821	2.83079202	2.64578798
2.12180383	2.47161486	2.42395527	1.78075097	3.64874113	2.21743402	2.11405786	1.87027747	2.30524964	2.10247673
4.19927669	4.09494545	4.23818427	4.04065801	6.75963067	4.10888902	3.53174718	4.0528533	3.85917155	3.76869712
4.50279228	4.70059924	4.32508193	4.13268864	4.280541	4.50225298	4.1682402	4.43693347	4.29406425	4.60796173
4.23060669	3.26004238	3.28011967	3.26080796	3.2978854	3.16596463	2.5528046	3.47948343	3.64117036	1.52150537
0.0363948	0.00391004	0.08059515	0.05168527	0.09010643	0.06716921	0.01833242	0.02862834	0	0.35629704
5.62272737	5.39743324	4.40647835	5.17302819	4.7084823	4.51701061	5.26315063	4.45195591	5.31922253	5.21851378
4.50715392	3.41681168	3.44061594	3.98861457	3.72282197	3.05317909	3.10130442	5.82644344	4.09043489	1.88423647
5.84418072	6.17681344	1.86884182	4.14834351	4.86556691	1.90304194	6.62701601	2.63143033	6.81856671	1.35624525
1.12444872	0.32048253	0.6560442	0.70487452	0.31288986	0.25503975	0.08216567	1.77911183	0.15296069	0.16327064
4.96345739	3.67195226	5.04934395	3.28548139	2.3660159	4.62425694	4.75657018	2.74962856	1.36004346	5.61356591
0.94815357	0.13430966	0.13534903	0.26031095	0.08686824	0.7807209	0.23953749	0.0546393	0	2.77772519
0.47471008	0.14385921	1.91693269	0.47232581	1.29749197	1.58325545	0.19285904	1.12558833	0.15272529	1.24068986
5.80657221	5.08773446	4.81277718	5.46869921	4.83707725	4.95156883	5.38287815	4.82951269	5.0536138	4.855989
5.41894717	3.42425502	3.30194561	4.71449375	4.73597844	4.99895466	5.33310657	3.85165106	3.91312658	3.64968287
3.66243895	4.5689656	4.3261468	4.11881161	3.82562859	3.99829409	3.84846769	4.26813042	3.68290608	3.48638951
3.90026679	4.41891705	3.91880633	4.38052926	4.72966641	4.45516953	4.42560555	4.73502807	4.45449241	3.92055403
3.0177323	3.65829267	3.28694894	3.30169216	3.51550391	3.98269466	2.97705558	3.56198591	3.39564531	3.59439345
1.56129311	1.51940295	2.16412798	1.87837042	1.8236457	2.68629278	1.59925953	1.8997691	1.82234188	1.81975812
2.99945518	3.93519493	4.40683241	3.40025734	3.45337002	3.77298249	2.88903022	3.6237806	3.76193373	3.37136282
0.01777143	2.8600925	0.00573332	0.03364385	0.85209777	0.01328159	0	0.02088944	0.98319441	0.01393757
2.39320195	3.65276736	2.33135921	2.13534645	2.97408648	3.08714978	2.28516073	2.33761495	3.65108581	2.04065471
3.74560611	4.31037445	3.43884535	3.63912977	4.2377348	3.75435615	4.05179175	3.2738174	3.97096397	2.41746019
4.71034369	5.43869166	5.1152156	4.50809646	5.84932043	5.5968203	4.88803477	4.65130682	5.723547	5.2997033
4.15318136	9.75986686	3.94710823	5.02133233	4.06139455	5.13426938	3.34106539	3.8294939	9.47649562	6.60955544
0.17769166	0.28100768	2.93902343	0.2162634	0.77811069	0.07136991	0.01047899	0.41286034	0.03470494	0.04056495
2.56828661	2.39545036	2.81337217	3.12888353	2.32042169	1.93120693	0.98949668	3.24387181	2.20180333	2.00468871
4.56531374	4.91111442	4.7658237	6.09657702	3.6461259	5.29597424	4.91635508	5.40590536	3.74863143	5.26770846
0.70685476	0.66006645	1.63922121	1.74957819	2.56213826	1.40014238	0.95344295	2.08285007	0.49097931	0.93461196
4.29803596	4.4177405	3.90679488	3.96984376	3.216707	4.22410143	4.74630519	3.62085733	4.36657037	3.80961696
2.70673775	5.11038987	1.12248341	3.05588557	2.08315163	1.26722314	0.27805662	2.34818487	4.77121348	2.37976057
3.63705016	3.62951521	3.23982804	2.88173852	3.16902011	3.60452238	3.05660369	3.01138145	3.84417976	4.01073375
3.10527008	1.79748036	2.72539382	3.83274065	3.55562458	2.4745872	2.62023062	3.61123362	1.5387483	3.79940279
1.65279344	2.57028101	1.50259434	2.20382127	1.57633315	3.14333586	1.68097222	2.16500556	2.67758545	4.43823039
2.00681016	2.29179218	1.31868301	1.93469172	2.46585368	2.60588576	2.02760427	1.58345029	2.05624135	2.84928655
2.97432545	3.29466531	2.16692706	2.62747688	2.59498928	3.47900186	1.90646101	2.59676554	2.35190523	2.32158205
4.47945875	4.81627788	3.93168428	4.1435365	5.02202183	4.57668086	4.05099563	4.3034345	4.32203208	4.12193618

7.66845983	0.60123105	0.65129661	2.86115156	0.20261095	2.25647661	3.68701964	3.19512215	4.90502083	1.62285596
2.99436839	1.3317185	1.91254281	2.55360549	2.13711298	3.78326967	3.37361073	2.12295565	1.24013201	3.89453993
2.90383343	2.83859012	2.58658207	3.16802159	3.11077906	3.23084615	2.5869582	2.506699	2.97150789	2.71364471
0.5630224	1.13959729	0.95036993	0.68175245	1.23976041	0.91413269	0.63968938	0.8842268	0.81064723	1.15703332
1.85162335	2.25609345	2.24634798	1.87650186	2.08324816	1.96514759	1.76685527	2.09600405	2.10678214	1.90482547
1.13128926	0.74836721	0.9556567	1.09019892	1.24996759	1.11583728	1.13037128	0.75723264	1.12836459	1.19736112
3.48463626	3.65681913	3.83822177	3.37787778	3.68110454	3.59923733	3.340399	3.4774004	3.92076553	3.47956382
4.39090264	4.98491192	4.71350016	4.50145533	4.97661426	5.04079457	4.87088655	4.05476231	4.66161976	6.07857808
4.15439003	3.92908906	3.43299437	3.92854413	3.44172675	3.69653655	3.84369546	3.81406294	3.77878365	4.61975481
3.26221318	4.86359085	2.28007928	3.95853613	3.41383258	3.57964376	2.83195796	2.82189341	3.76581591	3.365699
0.92877959	1.44162052	1.0465784	0.94093864	1.02694181	1.04822124	0.64413747	0.74513216	1.17472028	0.87385564
0.12108435	0.02203959	0.06564993	0.18641175	0.03527489	0.06357967	0.00977768	0.07002653	0.049086	0.02368413
2.24657289	2.44029564	2.11863897	2.02423215	2.09640873	2.37409476	2.0547932	2.4069616	2.66587603	2.1859595
1.86075844	3.21548277	2.3501285	2.52775464	2.61714194	2.38625472	2.78969507	1.92448477	3.56757583	2.44871319
2.52274003	3.31669764	3.60838299	2.33802418	3.35018815	2.62328877	2.98477673	2.06244385	2.1460755	1.95408268
2.1297282	2.61836604	2.424372	2.65700899	2.46798966	2.48013837	2.49446409	2.52103728	2.90159511	2.79517121
4.46201753	4.48520472	4.18174689	4.47407561	4.42356702	4.52416589	4.45676981	4.68995301	4.60149826	4.46450165
5.76568861	4.65023668	4.77924163	5.18381797	4.47597727	4.35917061	6.27137382	4.59822301	5.44019853	5.42240436
1.1575496	1.76460077	1.45564931	0.90711052	1.41186221	1.20356961	0.6997408	1.19522103	1.24549442	0.88563246
1.02180913	1.82870136	2.28994984	1.30438723	2.12346629	2.53886818	0.89080418	1.64408926	1.27441091	0.72926784
0.10942068	0.17705835	0.08509091	0	0.13016307	0.07439111	0.35395716	0.8898812	0.03221656	0.76330137
3.40827073	2.49615139	4.11198571	2.26693241	3.33541363	3.61367281	2.36679777	2.57809325	1.71162639	2.4902015
4.92584467	3.34845096	3.94519508	3.96452984	3.79364959	3.59582993	4.55250488	3.71677978	3.59529856	3.29737118
0.97289124	1.29355435	1.32328703	1.07524603	1.43039137	1.25467951	0.67160069	1.00075104	1.35595612	1.04446944
0.56925264	0.07358922	0.27792387	0.6176425	0.21876869	0.47245394	0.29073536	0.36719821	0.1230504	0.15891541
3.80815389	1.39639285	2.36455178	2.93935978	0.81503846	2.35712488	1.33976766	3.9307442	0.87007815	1.25042445
1.16834306	1.65610179	1.51187952	1.19476905	1.62563947	1.4261845	1.39575758	1.60947652	1.29434745	1.29409596
0.99370117	0.36752263	1.21314317	0.73101004	1.01546275	1.47118667	1.00576288	1.11353554	0.27333837	2.61819403
5.22974846	5.56210111	5.02685916	4.54764478	4.67064115	5.38737193	5.78524797	5.06660068	6.13103407	4.6118706
1.68471999	2.11639675	0.9700531	1.03265682	0.66340279	1.19199094	1.61773284	0.81429673	1.20270601	1.51260735
2.57562283	3.5684414	3.65593849	2.81204705	3.31217659	3.12459857	2.28225016	2.88182867	2.73706699	2.51142782
3.42134974	4.11143194	4.75440546	3.06471522	3.92061735	4.02990886	3.88102583	3.16329674	4.18450197	3.3312629
0.19917077	0.09697022	0.06358164	0.19017747	0.31694706	0.28109412	0.05790196	0.10211721	0.03186067	0.06034825
2.35949365	1.87466441	1.81658741	2.11454789	1.91653148	2.21671107	1.93147608	2.26523788	1.96295912	2.08149481
0.93708248	2.67272152	1.49673235	1.54735681	1.4664189	2.37143938	0.86748195	1.59657693	1.80318593	1.63345093
4.22919933	4.61944075	4.01070219	4.1962712	3.95339246	3.94446672	3.75853494	4.0816354	3.61099534	4.1878763
0.05059629	0.02709463	0.01644931	0.07170666	0.15387233	0.03167016	0.0255481	0.23266441	0.03643056	0.01338154
1.47821356	1.48120968	2.00506789	2.24627593	2.4561786	1.87518505	0.28319213	1.49529321	0.64123964	1.83910013
0.02094618	0.02003211	0.01349361	0	0	0.02342463	0.00789926	0.04881995	0.02992156	0.02457733
1.59771299	3.84021079	2.43290333	1.53373144	2.83499948	2.18033644	1.90748214	2.08369872	2.40811405	2.221923
2.21674452	2.48840964	3.07570739	2.343432	2.74290045	2.42641811	2.19461802	2.91518649	2.28131452	2.51951273
4.90472358	2.57837075	2.29067834	3.99899367	3.03239218	3.55984685	5.3804773	3.19496441	3.71676042	2.5276675
0.74951064	0.9617766	0.54889473	0.71559342	0.9106589	0.61613315	0.53850641	0.7403478	0.80033874	1.30291211
1.72957499	4.99191776	0.72803039	1.83693821	1.90303715	2.51660755	1.28454627	1.96004868	2.38659195	3.86002877
0.54710679	0.09690044	0.08305903	0.44267656	0.15876802	0.26328506	0.35275799	0.06947089	0.35201289	0.00944324
3.25526867	3.38467677	3.80100731	3.2849082	4.00622117	3.36318635	2.77942005	2.95811735	2.62705258	2.84713129
0.4672439	0.17351538	0.74022983	1.00269869	0.29532053	0.3653161	0.35164706	0.99412576	0.16656381	0.46570272
0.71175492	0.62390587	0.52535803	0.96501036	2.01484414	0.82962075	0.6237657	0.77628402	0.7629508	0.51728086
0.23389531	0.14179619	0.14523433	0.1248703	0.15186023	0.17536433	0.07373939	0.16982723	0.19927729	0.10058524
3.16365341	3.57958405	3.17909036	3.346289	3.13680353	3.41477691	2.91820868	3.26074338	2.97719485	3.15821639
1.03159073	1.90937105	1.7450184	0.88204852	2.50751416	1.63545439	1.24277859	1.39597289	1.27896642	1.09189369
1.76560334	1.86628286	1.78701096	1.553275	1.51469021	1.57227603	1.80583356	1.44846804	2.03153092	1.54746322
3.61425099	3.78093556	3.55814291	3.61158692	3.43467945	3.60597775	4.16688201	3.39946363	4.11591841	3.56551613
4.29565584	4.22833344	4.20544143	4.45278566	4.61233787	4.51007441	4.57414833	4.66657215	4.37186965	5.26576509
2.29009089	2.54013362	1.94130322	2.50527258	1.72167367	2.19206359	1.97537381	1.78366425	2.00596582	1.71114197
4.5947872	4.05311671	3.92241481	4.08386173	4.25762749	3.96427349	3.86234508	3.81403632	4.26188945	4.39830174
2.93123474	3.48945088	3.20411509	2.81508711	3.27565463	3.18390872	2.70647517	3.63725062	3.83642654	2.8894083
1.43482939	1.81281662	1.68689779	1.15231348	2.05400726	2.00251339	1.17241524	1.31153466	1.85324736	1.45242698
2.75904314	3.82363814	3.04520791	2.85876646	3.8945478	3.39510194	2.74848851	2.72949021	3.07201749	3.08953712

2.52891864	3.15637421	2.58263335	2.47800921	2.98008212	3.05673167	2.31614657	2.60353468	2.89684173	2.63334352
3.39606837	3.48603241	2.49224414	3.10760629	2.35254489	2.75363963	3.96583739	2.17962592	4.58529208	1.615058
2.49229566	3.07741137	2.41847728	2.72233819	2.60909779	3.03552765	2.12877601	3.29166352	2.66648808	3.47076464
1.45236752	1.98331754	1.83803813	1.51230161	2.16278693	2.07144041	1.43353782	1.87419618	1.63089029	1.57283205
1.22308008	1.09092957	2.02730924	1.32501032	1.15908933	1.03313225	1.25517223	1.95427171	2.60862532	1.30931603
3.196739	3.63517252	6.21024376	5.82792017	4.15869959	5.04962865	5.51602904	6.13443299	3.96108587	4.96595545
1.11604213	0.80372767	1.02463346	1.92558458	0.46440904	0.70204697	0.08577712	1.6023741	0.31081929	0.59398969
0.35598944	0.42530236	0.43577753	0.34070573	0.43371633	0.33072975	0.33880707	0.40604111	0.16322692	0.351775
0.73475559	0.66176904	0.62673626	0.80461581	0.42256022	0.60438985	0.25862738	0.78071796	0.62410255	0.32230099
1.31404078	2.92022693	1.12237272	1.50215328	3.0632455	3.35323425	1.47267525	1.2175715	1.19490107	2.76157188
2.14333954	3.33210778	2.65136613	2.55088458	2.91392367	2.73511716	1.85318505	3.05020228	2.14800973	2.1511894
2.93929422	2.80197557	4.11123652	3.9119459	3.55915218	3.36769636	2.68203959	3.85705527	2.54560386	3.88379115
0.90569061	1.30055885	1.00548477	0.78699546	1.02502926	0.99415909	0.82329878	1.38009339	0.98539781	0.7260544
2.25814143	4.06250682	3.78823194	2.41905468	4.19199211	3.46259875	2.84789297	2.31301927	3.60272382	3.71350852
2.37931692	2.67303324	2.38759711	1.98788741	2.1792601	2.1812731	1.72967628	1.8340821	2.16510317	2.26407679
0.05977062	0.33670395	0.04820028	0.12510672	0.19751914	0.14088704	0.03396245	0.07008081	0.02169799	0.08131001
3.61639351	3.24761451	3.62698414	3.2773755	3.44333452	3.60857116	2.52326318	3.67416862	3.40804854	4.07180847
3.13324233	3.36103843	3.01409541	3.42767138	3.52798727	3.21946645	3.20529588	3.73218617	3.62958949	3.05489519
2.71368371	2.0747733	2.11872358	2.22576072	2.31065296	2.53766255	2.59890267	2.08136236	2.77918191	2.16125827
3.06651201	2.73285438	3.30244791	2.98728038	2.99638373	3.19179228	2.26338092	2.90108496	2.61558502	2.65659368
1.72150591	2.04402191	3.13450463	1.83726412	1.83964508	4.01119964	2.11233925	2.69923789	2.83282047	1.26261758
2.20815192	2.36245649	2.69952123	2.32602583	2.85689428	2.36160397	1.53841974	2.31051249	2.21669412	2.21810346
2.40606359	2.68561201	2.42010825	2.9899832	3.00029097	2.52084517	1.97827119	2.27628422	2.36420821	2.10067984
1.63241695	1.50374784	2.40337247	1.91858047	1.29769418	2.63392469	1.52867153	1.84165279	1.08242967	2.64537245
3.08239051	3.76799791	3.03165512	2.92769064	3.88499141	3.33802342	2.98319083	3.2503962	3.3249306	3.03870968
0.10603737	0.15919839	0.10890343	0.10110208	0.31464451	0.241369	0.05405167	0.20110357	0.17778626	0.12116312
1.25632894	1.02958936	1.36921324	0.8581632	1.1166579	2.20678697	0.72680812	1.04089254	1.26820789	1.48006046
2.21062357	2.87961267	3.79994791	2.23105933	2.58022616	3.366823	2.12324645	2.42249162	2.40775855	2.57938739
1.17887006	0.36282029	1.1035405	1.35171865	1.46981218	1.2964941	0.28839548	1.18125179	0.81570939	1.00940055
0	0	0	0	0.00379192	0.00448544	0	0.00706709	0	0.02801844
0.56373679	0.43496932	0.55035573	0.76913529	0.58739258	0.41279989	0.37280409	0.87222221	0.853936	0.34701372
0.63803004	0.41406754	0.47442611	0.29707474	0.82914053	0.53178572	0.44267033	0.75497662	0.79750552	0.37477415
1.18492893	1.45267725	0.73737409	1.13534257	0.6813638	0.71510125	1.19225125	1.02210026	0.88637347	0.91310698
2.8808805	3.92075723	4.02773542	3.57915101	3.18420518	3.41999767	2.98346848	3.79857215	3.2494378	3.28365358
1.91801579	0.84192652	1.55756359	0.90897928	1.36393924	1.08714481	0.47715277	1.05574489	1.18758431	0.38085265
1.66207392	2.3724333	1.73907436	1.66288438	1.85118436	2.19769163	0.925291	1.19570018	1.77908773	2.31110672
1.88774784	2.51440569	2.28305207	1.42106872	2.43948329	2.35207495	1.39808523	1.62354033	2.05202632	1.84744334
1.35141804	2.50765717	2.49903932	2.50236323	3.19773671	1.88582555	1.42587408	2.67090866	1.76874351	1.34035718
2.30757892	2.71937105	2.2145018	2.36852101	2.32714224	2.96872068	2.08264758	2.19658202	2.56373924	2.15922736
0.52073912	0.33094963	1.51920984	0.8431808	0.99306338	0.40120715	0.22857849	1.07031737	0.28218183	0.50505688
3.31320183	2.25778969	3.52478219	3.35504635	3.39403039	3.53163296	2.96133849	3.00684914	2.43784705	3.17014979
2.77650784	3.18857301	3.16456366	3.74043959	3.46817861	3.78567479	2.68279393	3.65150623	2.88985759	3.11394907
0.07711869	0.06302238	0.24922938	0.31416625	0.0374111	0.1060723	0.01021872	0.2256825	0.03480554	0.06892951
0.95328607	1.10167984	1.19341508	1.12752348	1.37496912	1.42816068	0.80316909	1.41081411	1.37685731	1.22108473
2.89417508	3.68017034	3.12304662	1.47057533	0.11354641	3.92384866	3.35761028	0.40947387	2.62321976	2.55704137
1.77093514	2.14192398	2.56997825	1.93238616	2.9974841	2.7975877	1.55498111	2.74851356	2.62862497	2.29675221
2.16295105	2.19722133	2.94822942	1.7471129	2.87607881	2.30165344	2.2445732	1.8559909	2.38938019	2.04945594
1.71066265	3.28745681	4.48662403	1.47123942	3.47003367	3.67871513	3.45770734	2.49804635	3.214958	2.76939062
3.75886807	3.18226583	4.36237391	3.75970368	4.22555891	3.41886453	2.42037382	4.50162542	2.85714959	3.59779348
2.83100422	3.61171924	2.41933194	2.65989563	2.13097996	2.33192516	2.39885986	2.81870638	2.45594118	3.43833993
0.21070344	0.15171686	0.13886781	0.30155537	0.06255239	0.09748262	0.05685293	0.56207031	0.15712831	0.08443404
0.07253335	0.98135401	0.85500868	1.07427946	0.08050082	0.43139499	0.01957931	0.43651961	0.1028405	0.38039116
2.7713139	4.11336071	4.08947656	3.65904996	3.5899158	3.91748702	2.46725184	4.56156079	3.09270037	3.34001754
0.22985272	0.78161791	0.78254687	0.75321822	1.17236276	0.31504095	0.46258133	0.86520724	0.10967814	0.64535593
1.60339358	2.30554049	2.5697252	2.06644802	1.98779227	2.40343961	1.60796012	2.22951367	2.43372641	1.4462839
3.48055026	2.38363398	2.79635739	1.53117301	2.63846514	3.52815093	3.62661905	1.74534123	2.30099721	3.18825859
3.88358381	4.65275039	3.02639931	3.23456286	3.89962219	4.03662035	4.1694772	3.87908078	4.72797024	3.71675957
6.85941034	7.12993344	6.49204479	5.83208201	6.38840993	7.14246136	7.03740413	6.49974602	7.68805059	6.32714008
9.89206591	1.74043274	8.31802139	4.68300044	2.19088654	3.63306256	9.69122878	3.23073203	1.8765562	1.49250766

3.29138283	3.32718933	6.43411855	4.97307825	5.32520481	4.89686793	5.44663718	5.35090228	3.64015431	4.52322107
2.44882301	1.79631388	3.83033697	3.88876384	2.58117014	2.77405269	3.31347111	3.61143173	1.53914878	2.735489
4.53997645	5.76038942	4.24494787	4.47299105	4.16372904	4.49175494	4.28187175	4.94506414	3.50032946	4.33203047
3.67979282	3.53060246	2.03633284	4.05685161	4.43775468	1.57637546	0.5961149	3.65142127	2.38413121	0.05128002
5.35390812	5.01940076	6.11161702	5.75864899	5.94555987	5.34011764	5.84846136	5.39389526	5.09152871	5.78158662
5.07869827	5.10005718	0.2899358	1.61943041	3.15600603	3.34978527	3.47229804	2.88989912	2.38279665	3.85469801
6.25503257	5.49547786	6.47476829	5.98654958	6.60394373	6.4526987	6.85873022	6.2079318	5.83404613	7.29314829
1.7594755	0.64066477	0.42630089	1.10573511	2.11990683	0.6975699	0.71843523	0.94004503	1.14182194	0.26210061
1.91288326	4.70870349	3.66841693	3.08157733	4.57557568	3.67185567	2.78687938	2.52580841	4.46156998	5.26637854
3.69786928	6.16239539	5.4270339	3.42806494	3.45216063	5.55692899	4.98727866	3.30869467	4.5519474	4.61757326
6.4227009	5.38373265	6.45466525	6.90140547	6.80151713	6.37938631	6.7852889	6.7530963	5.81411533	5.78993483
5.81295593	4.06333815	4.72110663	5.74398577	4.74932621	4.527259	4.17355279	5.74212916	4.09938585	6.30823831
0.02914347	0.19291261	0.01344871	0.03558459	0.17465528	0.0432798	0.03123715	0.14579557	0.07056482	0.03256959
1.58769612	1.71653214	2.01222783	3.04324166	2.05361984	3.29028862	2.4685928	1.85454962	1.81260601	3.98861577
2.19828997	2.24587416	3.7028636	1.52812752	3.00705752	2.45071618	1.65071462	1.35477279	2.17906843	2.31035775
5.74678229	5.91956874	5.09038612	6.02694775	0.77193517	5.78209463	6.90512635	4.55940066	2.94288477	6.42626148
0.23055185	0.23353179	0.25376796	0.22950147	0.1560752	0.26988194	0.16128078	1.06604345	0.50346074	0.19142269
4.3070906	3.38051601	4.78269437	6.26845409	3.4200039	4.22585709	2.62974884	6.59295808	2.6801311	4.28271782
5.12760231	5.4057869	4.91546729	4.72032413	5.08851777	5.12130425	4.78434093	4.8000123	4.71082484	5.34882431
2.62683881	3.45151006	2.83578932	2.62322568	3.68516375	3.91649088	2.60416357	2.93207769	3.13891101	2.67426855
2.59745957	3.34941509	2.72958648	1.87068946	2.25743465	2.9363112	2.40514059	2.68180861	2.8619504	2.61669126
2.177648	2.07814522	3.10398815	2.46322196	2.02382007	2.95364088	2.1966717	2.61723173	1.76189542	1.90906014
5.07371129	5.95225399	6.00959131	4.67845659	5.13606141	6.21253107	5.73625633	5.0099913	5.56945719	5.6358198
5.23917187	5.24035509	6.15392319	4.4505005	4.72493255	5.81327401	6.07635207	5.17230521	6.61501414	6.60235528
2.33028056	2.87226638	3.24547995	2.82022774	1.81127243	2.24122237	2.03665335	2.30768587	1.91293894	2.83044251
3.58639664	4.06320634	4.68637001	4.19934366	4.65075824	4.48505614	3.62279478	4.50145103	3.5832912	4.10654629
3.05993104	4.10557719	3.4376033	3.3761608	4.0634751	3.36235868	2.56411246	3.42631924	3.00185174	3.24042858
4.83309011	5.34071668	5.48184971	4.98830023	5.04395779	5.55663294	5.29511884	5.04074532	5.07096844	4.7341256
5.9251734	6.42174499	6.36195049	6.27368012	6.0726714	6.48545273	5.80062277	6.14787148	5.96994054	6.78858291
5.73527099	5.19198363	5.46151503	5.2924324	5.69541196	6.22993612	6.32310161	5.49067794	5.94058519	5.93358752
2.83148381	3.55990218	3.31032754	2.79728086	3.4072342	3.36446886	2.18075546	2.8718426	3.19272523	2.9414979
0.15178371	0.03643504	2.53008846	0.26027984	0.0547866	0.05729277	0.0127283	0.29929487	0.01456581	0.02380752
4.05600255	4.63736446	5.02478928	4.62989334	4.82574858	4.34354991	4.46520766	4.85180675	4.27818447	4.78501992
3.02314464	4.0810428	4.00066168	3.36164072	4.19251289	3.57208293	3.04265907	3.24693224	3.2831708	3.43345634
1.58372342	2.02091764	2.12490818	1.31346892	2.09003335	2.55140343	1.486811	1.78268868	1.79510457	1.70565417
3.60999062	3.69161557	4.31535422	3.44178376	3.11435891	3.86010853	4.19999501	3.48187383	3.81187784	3.40250373
1.8735853	1.73958574	2.09166482	1.73788802	2.21968929	2.17195852	1.76854969	1.90434082	2.03237265	1.86624214
1.94900233	2.27602115	1.76633162	1.59017551	2.9335426	1.76906048	1.46564576	1.45815253	2.12700513	1.5730151
1.03518967	1.35241505	1.84368936	1.10278285	2.01628495	1.60776399	0.80163284	1.35959527	1.10379168	0.75363278
1.97923996	2.32191835	2.64282429	2.07678318	2.20079305	2.73806011	2.67601544	2.35174226	2.31672788	2.87344048
1.09672016	1.31952807	0.70421017	0.97469086	0.7685095	0.94187262	0.7769921	1.40236872	0.9974351	0.64412488
2.85557654	3.36057486	4.05012427	2.97919444	3.62798208	3.50355169	3.39875318	3.25292956	3.76529218	2.89186202
2.70632808	2.54219343	3.37810396	2.83911183	3.84820881	3.45430265	2.46045991	3.1263548	2.67551506	3.35499867
1.56994856	2.44437994	2.67743382	1.28884524	2.3100201	2.22435552	1.44822199	1.70110759	2.19410859	1.58106703
0.6807596	0.34558659	0.8054679	1.05388196	1.45430891	0.49445488	0.278065	1.30257194	0.43896457	0.31421528
0.95258999	0.73476621	1.20768025	1.02315802	0.94859347	1.06436656	1.56247367	1.22703086	0.89535643	0.54886703
2.80743686	2.66379743	2.66758211	2.70116876	2.41237557	2.0727958	2.25141422	2.45681192	2.55169157	2.47611026
2.2349568	1.34420628	3.8119255	3.00558702	2.54906762	1.75548067	1.156104	2.71167242	1.18603927	1.67689152
3.68831842	2.78599287	2.89558581	2.37379312	3.45628068	2.59189473	2.1187579	3.43111921	2.75116832	1.63028723
1.72537511	3.64754562	2.24569708	2.81477266	3.17107109	3.07258318	1.44414015	2.81959976	2.06042907	1.71749858
1.44550444	1.65394672	1.36404854	1.09819566	1.73008455	1.35999853	0.66117547	1.51255994	1.70340686	1.06082799
1.50782615	1.69453568	2.36994736	1.73126663	2.01121376	1.89687586	1.2801298	1.85796919	1.27062532	0.6800145
0.4472714	0.93175358	0.58611347	0.74237778	0.55680045	0.33491496	0.35796915	1.15347181	0.71763867	0.52672388
2.4860777	0.98937246	2.41004272	2.8621743	1.18007718	3.29511239	2.16249689	2.935601	1.14144303	6.80950838
0.30906373	0.56937529	0.6441799	0.96346365	1.44589457	0.39716838	0.35505116	1.27014897	0.18515065	0.32133576
4.60999772	4.74450483	4.06816381	4.02103367	4.63288179	4.41134036	5.0132658	4.11824558	4.31815813	4.63403472
6.73101775	7.16018795	5.80699107	6.22933837	6.1782441	6.64321322	6.55288711	6.30816527	6.72912329	5.86499333
3.88263156	4.20679203	4.25429602	3.6888947	3.45644021	4.52280223	3.971929	3.7384825	3.81525069	4.76691641
5.79968553	6.23948082	5.55950602	5.34740298	6.11904096	5.8721611	5.82655659	5.66147795	5.87163258	5.11796528

1.48240945	1.58290855	1.50867028	1.48456511	1.80869711	1.28753593	1.0212228	1.94516549	0.93886951	1.04621554
4.09780447	4.562612	4.67292195	3.8014354	4.92246976	4.68663115	4.22938977	3.73507334	4.0410613	3.95506529
2.05813754	3.2709244	2.38761043	1.60835643	2.14975068	2.06067861	0.99650034	1.42945056	2.56478699	1.62975108
5.12562322	3.14402796	3.86770903	5.06819136	4.14404963	3.18064907	2.15964594	6.20139828	1.93205123	2.29228194
1.10855995	0.32814036	1.86281799	1.66378492	1.0461293	0.36788058	0.66013253	1.58198932	0.40043262	0.67877036
5.17399211	5.63066782	5.26626754	5.61173472	5.75714772	5.8948307	4.5087102	5.45682639	5.6548573	6.32765276
3.18318842	0.65204885	0.2264793	1.87096947	0.35395502	0.61012448	0.23456922	0.42854621	0.54923732	0.0807309
2.31972294	0	0	0.72168252	0	0.40996312	0.04544704	0	0.05757941	0.03175604
0.34113273	0.02572546	0.02849993	0.26322803	0.03175196	0.03304521	0.94892091	0.13486163	0	0.17829517
2.20955084	3.25813987	2.77370854	0.23787211	0	1.79046098	3.79916142	1.2371731	0.88231661	4.29442033
0.34057228	0.04023918	0.34906012	0.2042113	0.2848656	0.56250618	0.24681986	0.26114769	0.1563468	0.20530461
0.84376836	0.23374296	2.85198942	1.02118718	2.13655145	3.03393387	1.68755181	2.87291869	2.6622758	2.05201613
5.02942043	3.9780955	3.50645113	4.3848172	3.9861567	4.39609747	4.38742827	4.78895296	4.57709495	4.39614381
1.88686111	1.76148265	1.5825892	1.20324066	1.33024584	1.78532682	1.45418248	1.97807635	1.88431947	0.96010895
2.29820955	2.2951623	2.5337759	1.98265127	2.50183336	2.46621364	3.79483626	1.89069055	2.69788007	1.33183797
6.43315705	5.75945609	6.02410093	4.6941611	6.29476821	6.2038904	6.81525673	5.47933944	8.00365178	4.41769058
0.71916673	0.56096598	0.66500937	0.25006149	1.01729209	0.26042405	1.01449161	0.15961641	0.89125661	0.40539499
0.56883401	0.04377243	0.02222963	0.3774245	0.04331883	0.81341858	0.07646949	0.1554227	0	0
7.45823073	4.9829549	5.26361216	8.62401691	4.1719561	6.30850317	2.32961158	7.26314764	5.51164676	2.9462111
8.49431479	7.78111071	7.94477049	7.52663918	8.47429098	8.59273825	8.54398082	7.46992783	9.23999352	6.14673416
3.91937574	5.34393417	4.46279314	4.86541337	5.01409755	5.10648972	5.47894891	4.97846299	5.11846755	3.79388408
3.43098018	3.8441689	3.61435814	3.75469649	3.5769011	3.78627322	4.31419597	3.88728936	4.28523618	3.04603742
3.0135127	2.43445534	1.95957675	2.78882479	2.99771521	3.40577525	2.71545448	3.13061691	3.4675888	1.72971079
3.33040318	3.96397442	3.75471384	3.50741587	3.90857452	4.62227659	3.96091	3.59435269	4.60717941	3.7189148
3.06487866	2.54773358	3.18146714	3.31015944	2.1990143	2.15858614	0.71750057	3.97410071	1.56280107	2.20604762
2.8787413	3.63710377	3.50452786	2.95632922	2.89044788	3.31114353	3.46066736	2.77505345	4.22215761	3.6780732
1.99347287	2.71352442	3.10198974	2.38973924	2.53637826	2.71194092	2.0961372	3.23236991	2.71065971	2.6245011
1.93792905	1.8676811	1.93926045	1.98865084	1.78816377	1.85014264	2.11533599	1.95410409	2.31871061	2.14077484
6.86666196	7.32675622	6.78649413	7.19910272	6.44771106	6.04259545	6.52378125	7.23658267	6.64601212	7.87367133
1.8732902	2.01155839	2.22620104	2.0539769	2.27686539	2.14411438	1.5508861	2.22819829	1.80321328	1.69995402
2.95785266	3.75048244	2.72209472	2.79478574	3.23782609	3.03733418	2.66749369	2.69519834	3.45820088	2.54441544
3.15497197	4.37779256	3.72881264	3.12954851	3.82898058	3.30419911	2.88992706	2.75550566	3.62896898	2.9713135
6.12508064	6.01052827	4.54777767	5.20663569	5.93603412	5.66288059	6.99996918	5.69020345	5.86253879	5.59832212
6.50711124	6.68406157	5.3453657	6.96255394	4.36431126	5.46674968	3.46472937	7.22156227	7.62900869	4.60972018
0.13807984	0.08160894	0.02517171	0.07333352	0.69770408	0	0.02937134	0.06070879	0.03726742	0
1.37763435	1.68514279	1.0895439	1.55437698	1.06017906	1.63468501	1.44962088	1.20496923	2.17937738	0.8808469
0.01395903	0.01334846	0.01791382	0.27839351	1.70602654	0.00522387	0.04663127	0	0.01001507	0
0.09303835	0.08907357	2.32044675	4.87902299	5.72552292	1.19684648	0.09313865	0.36447952	0.11042498	0.08524721
1.19586113	0.19821577	0.24092607	0.49522049	0.36619201	0.65965187	0.52878736	2.88645729	0.02449945	0.79869508
0.06237436	0.01347935	0.42295448	0.02008307	0	0.0467968	0.03155896	0.07312241	0	0
0.57691974	0.48113012	1.32465817	0.41413862	1.20001226	2.17603228	1.90659922	0.41678543	0.69697279	0.76056985
2.06905627	2.82109328	2.54691355	1.85696339	2.28865495	2.11947328	1.98324638	2.14955913	2.50129584	2.15209881
0.26679994	0.14464421	0.77885665	1.02637475	0.38609037	0.26946955	0.12079117	1.37100141	0.15707733	0.34565629
3.96374017	4.10019532	3.930909	4.35362499	4.16567793	3.53153618	2.68970392	3.91905538	3.46082843	3.08756592
2.04000321	3.06489601	2.5701792	2.5773867	2.18326582	3.20352132	0.80890994	3.23813302	2.89208519	1.62295167
0.36387283	1.84620342	0.94907606	0.83122346	0.2613191	0.32263432	0.57902136	0.82025342	0.02864379	0.39818814
4.97739834	5.97976509	5.64295457	5.20572432	6.22192946	5.41858562	5.32462286	5.25223214	4.80857907	5.29746437
7.27472777	7.41965607	6.98484639	7.19906245	8.25643419	7.79780111	7.76545282	7.41002632	7.17693948	7.53826921
5.09165531	5.79578859	5.37296979	5.21703594	5.62167898	6.07331337	5.55574383	5.2596068	5.8420389	5.62444282
6.09925592	6.52923168	6.29624612	6.20299143	6.73762755	5.95271855	6.67689912	5.9951567	6.80272559	6.18435432
5.56911017	5.7671892	5.36044524	5.28607666	5.28258104	6.19880431	6.53270154	5.47576485	6.03125488	6.34099177
3.25707448	3.70842473	3.67714835	3.553896	4.26124563	3.84100502	3.31138191	3.81656395	4.07849238	4.37502932
3.74702508	4.93975647	3.91030276	3.63386823	4.2324545	3.71658226	3.26066352	3.46970435	3.93691356	3.76577785
3.6092419	3.61539058	3.62283614	3.75193976	3.16030956	3.94283511	3.55285441	3.62876908	2.99488584	3.74795365
2.55879529	4.54149127	3.8412448	2.75246027	4.08738663	3.99905454	2.00457037	2.6852344	2.93321909	3.69746753
2.86361779	3.4321945	3.41547995	2.75452362	3.32710328	3.22448404	3.00980576	2.52864439	3.31841861	3.04606232
2.57887637	3.00078422	2.40267068	2.43732714	2.36644027	2.3851514	2.20719826	2.37063042	2.89997054	3.41096854
3.6080306	3.78062253	2.94245803	2.75800322	3.66475319	3.8209326	2.87444248	3.41489285	4.47582099	3.71086799
4.53624687	5.21040881	5.68262026	5.5325524	5.24515903	4.81957327	4.98977706	5.72991599	4.68493349	5.09836563

1.00126589	2.47571187	1.60038277	1.82606921	1.43441377	1.427338	0.86572621	1.72761662	2.09125289	1.02787873
1.81688791	3.15413864	2.57379195	1.18695983	2.49107302	2.4988444	2.6945011	2.30515898	2.98356718	1.61010286
2.37104444	0.953935	0.79893821	1.21715119	1.02359256	0.81585608	0.60361495	1.02407453	1.04890428	0.3932006
3.26570108	2.74109943	3.64423244	2.90002116	3.06905044	3.30091163	2.78981273	3.28757596	2.80138007	2.84908405
2.92573661	3.52914716	4.22998219	2.51442069	4.2806085	3.43841774	2.64599146	3.10080824	2.94890985	3.19595398
1.2639542	1.9851019	0.87759027	0.782651	1.81779737	1.48041338	0.64441455	1.36811455	1.30934654	0.97829423
2.11740199	3.39757266	3.90729989	2.78259866	3.51790585	3.81709291	2.3075706	2.91307972	2.68140086	3.29055775
1.27272579	0.56351265	1.07871844	0.79424176	0.93699753	0.35453573	0.45973327	0.97773603	0.43218317	0.30098632
1.89550675	0.6921291	1.78886244	1.86285922	1.25011577	1.00654464	0.64553227	1.97695383	0.91638544	0.86785977
3.50885455	1.94954373	1.65907156	2.17698101	2.29634537	2.00374562	1.38147832	2.02076034	2.389818	0.89051535
2.01838681	1.63724557	2.54870302	1.84851583	1.77223601	2.06515722	1.37259304	1.46188513	2.38083456	1.95509324
2.93169406	2.40250933	3.63183751	2.07110367	2.18455403	2.85268584	2.59055249	2.23087899	2.84770696	3.32608953
1.03384619	0.73482358	1.5841649	1.30328754	1.8270303	1.25895992	0.71344229	2.32808516	0.68399119	0.55360562
4.24713746	2.92217448	2.38609855	3.01582166	3.38047146	3.0465439	2.61788953	2.72507461	3.60160714	1.30611988
3.19462196	2.88141222	3.32609196	1.78958453	2.50217494	2.93082247	3.34403866	2.1249555	2.3847156	2.77747094
0.99902777	1.74668147	2.6487953	0.79466064	1.66356146	1.45091872	0.75197927	1.29633917	1.8728343	1.0423032
3.08148041	3.52731285	3.65254989	3.75969371	4.45134824	4.11497437	3.05987236	3.96454668	3.52917016	3.49000877
2.24159031	2.81202284	3.15083397	2.49922931	2.97524467	3.73889329	1.97990109	1.73449058	3.33344002	2.11879598
1.43427693	1.5976432	0.9002795	1.6722139	1.00391044	0.71463921	0.45500062	1.24437944	2.72522251	0.72548951
0.32381801	0.42742652	0.47305962	0.54898481	0.25469698	0.80308134	0.48534532	1.02250786	0.68974634	0.76525317
3.69007336	2.1588064	1.62654942	1.74104466	2.21335337	1.88224098	1.41038329	1.73117647	2.34560952	0.44495001
7.06593607	6.88307833	7.38207048	6.99925312	6.74724738	6.82145957	7.54422607	6.99828225	6.49459665	6.74517245
7.51828302	5.78989586	5.44305325	6.05571193	6.00079687	5.56577658	5.66467609	6.1850481	6.25226983	3.99536404
0.39921165	0.01575876	0.37117913	1.66964973	1.85344868	0.77163214	0.00930287	1.74507904	0.01770274	2.71231809
3.91266927	3.59684571	3.44522096	3.22670139	4.30930913	4.30496937	2.37735348	3.99427933	3.78512346	3.15157754
1.56586034	1.45171942	1.99834875	1.94272388	2.79774721	1.28589934	0.82156775	2.30142854	0.8029964	1.33970048
2.91013598	3.86181455	3.82558956	3.46871017	3.92023769	3.73913782	2.9708848	3.75050378	3.57206269	3.3029931
2.16515029	3.10841069	3.11825637	2.71857577	3.10058265	2.70128968	2.15858199	2.91419131	2.33777195	2.8766718
2.47455912	1.54843169	2.20934365	2.91229917	1.77749142	1.75471325	0.9786259	3.91108409	1.67967957	1.452579
2.73101382	3.17475433	3.16892399	2.12507868	2.93237895	2.9354929	3.66124874	1.53340407	3.41563181	3.48779571
2.27220522	2.23909617	3.54959516	4.01829748	3.54447025	2.62142592	2.08861075	4.29853241	2.23274618	3.04193488
1.32986601	1.27404782	2.08604688	1.35560676	1.35262731	1.57344231	1.25008097	1.67449996	1.51913465	1.32408459
1.86078752	3.64725528	1.38328386	2.12308454	2.01990853	2.64767822	1.7458256	1.94138846	1.46555593	1.15130037
3.69331002	3.34604304	3.79098135	3.47649389	3.61923548	3.57306765	2.91257901	4.12225165	3.04299216	3.20052834
1.93126358	1.48991534	2.46318483	3.47235886	2.09946703	2.1379723	1.5054537	3.22848651	1.35022974	2.19227016
1.06116862	0.5380418	1.61828114	2.37714666	0.72599131	1.84127662	0.48032128	1.91900908	0.21218365	1.34532975
0.79440398	1.60956979	1.53493491	1.07963816	0.38788775	0.89991741	0.42232666	0.92511223	0.85627307	0.88683113
2.61561335	3.72334891	2.2774259	2.53758134	2.37499763	2.3543552	1.57428594	2.54968688	2.14702135	1.97753707
1.29999649	2.53864036	1.81067132	2.64108466	1.64479785	1.94525311	0.7874763	1.85521644	0.33240186	2.46617526
0.94901955	0.99339714	0.60056064	0.63161733	0.10184032	0.50203919	0.43115427	0.11700572	1.1953041	1.13344772
1.26195373	2.48532647	1.27256724	0.71716256	1.60114004	2.48883384	1.9954446	0.8264908	1.55075566	1.36995543
2.67519238	2.56421247	3.05949796	3.1248324	2.5491655	2.77060041	2.92724907	3.5473011	2.30463279	2.4429694
1.43947454	1.40389562	2.69805939	1.24458293	1.45536754	2.64574984	1.91289881	1.86946254	1.51900398	3.27839603
2.73193328	1.91050582	2.33400927	2.38996882	2.36627727	2.00696968	1.21945247	2.81789891	1.62890979	1.01549007
2.46483403	2.32227653	2.67478442	2.51452533	2.80673641	2.5760433	2.1539127	2.89553652	2.26596935	2.8864846
1.88297753	2.86852098	2.379151	2.3973231	2.08147906	1.69596065	1.38483238	1.95642913	1.78582563	1.98525384
4.13529233	4.99973809	3.99700507	4.42068755	4.55266408	3.99555753	2.8695722	4.77780101	4.13936053	3.74462035
2.93104493	3.40236464	3.06021108	3.13869437	2.76333105	2.98479511	2.55776769	3.2981455	2.77868965	2.55311902
2.4855978	3.34240596	3.74263142	1.64492355	2.64768502	3.00822312	1.89017694	2.43002716	2.74916226	1.93062707
2.44463233	2.3257588	1.96404946	1.95976837	1.86824157	2.05414224	1.90251273	2.01641149	2.46501335	1.7746038
2.37681936	2.46555307	1.89916152	1.77228374	2.57265018	2.33760135	1.9080471	2.04196359	2.49620982	1.75462698
4.21079746	3.46938836	3.99464369	3.89198174	3.92336341	3.9602298	3.37506229	4.14818307	3.46957629	3.24394527
3.58184473	2.51442511	4.07835134	3.90267719	3.22573632	4.04586202	3.37615397	4.277344	3.02193295	2.44975497
1.57234899	2.10287883	2.08429671	1.70027844	2.18586554	2.18581369	1.5416619	1.80881434	1.76827041	1.93429356
2.64617357	3.20517574	2.63686729	2.48005756	3.04360003	2.85347994	2.33498803	2.5881177	2.72894398	2.60084907
3.44329075	3.74918613	3.79464002	3.81785098	4.05082517	3.71945377	3.78169077	3.27947228	3.79278002	3.6521052
0.45879994	0.28826388	0.86135873	0.84490748	0.78809291	0.71414744	0.48302319	1.22714463	0.40546497	0.48448852
1.24001617	0.69650555	0.51833445	0.5696737	0.88105937	0.75335766	0.8682991	0.54935862	0.66708761	0.30524696
1.60186791	1.59290662	1.635693	1.60310885	2.27067392	1.44653849	1.22827467	1.88187926	1.34762446	1.11898009

2.88840854	0.97744489	2.18713492	1.95554071	0.7773561	2.15429415	2.75615295	1.6380586	0.76150725	1.83137617
2.28041976	2.94077147	3.11026947	2.25774298	2.45799646	2.13251964	1.3908024	3.06596954	2.55993088	1.96542493
2.55270602	3.00383051	2.39260816	2.49187912	2.41690099	2.04083431	2.51282677	2.43800286	2.99960298	2.59977548
4.69513922	5.45625702	4.85656718	4.72695559	4.28770729	5.14546329	4.66287114	4.83398093	5.20848556	5.30179228
2.51349087	2.47556011	2.85845714	2.50985124	2.76033117	2.65637212	2.42233664	2.88071644	1.75091301	2.67128284
2.18921759	2.31719316	2.62697874	2.36622911	2.12025546	2.416503	2.16044444	2.2296245	2.46185843	2.11588028
2.35938617	1.9920172	3.76445424	2.1506127	2.53138909	2.82604274	2.98612684	2.4417208	2.23433406	3.36171941
1.57837686	1.04206006	1.61373412	2.61253469	1.5878375	1.65119608	0.79167557	2.21285269	0.51387632	1.63923447
2.99866479	3.74751711	3.00573249	2.65403166	2.79267463	2.92713535	3.25574316	3.00065663	3.08167572	2.59510903
1.9836071	3.19385091	3.94258457	2.03182594	2.95720644	4.29694315	2.22902096	2.16803355	2.57210246	3.05477333
5.88626701	5.28206534	5.65328144	5.13834267	5.87611887	7.02116521	5.66620609	5.51872854	5.82380751	4.93309695
1.05389127	1.05935048	0.75826067	0.87816214	1.07112172	0.82775161	1.29735118	1.01317811	1.22475288	0.98003585
5.86251507	5.20251294	5.40733263	6.64403578	5.29252895	5.52153629	5.72180483	5.58135214	4.94590933	6.06861916
4.01570252	4.16678022	4.75403348	4.12507977	3.79275106	4.46889866	4.02644465	3.74667765	3.84221297	4.58694849
1.99800007	0.15915845	0.98018414	1.2874187	1.34612286	0.56961649	0.97217186	0.97027692	0.51510101	0.48945958
3.50160813	4.20115937	3.89790916	3.26654477	3.44777192	4.05015778	3.27586992	2.93710087	4.14777624	3.08964621
2.69061524	3.34683125	3.84181015	2.93872609	3.20278021	3.9603344	3.50735682	3.16834417	3.04754819	3.83857775
0.21704021	0.02689018	0.04047061	0.09793112	0.1406302	0.09231202	0.031625	0.23102693	0.0597671	0.0165811
0.38438332	0.73584641	0.85849733	0.47892084	0.84649298	0.825075	0.23568945	0.72663333	0.67787201	1.38735605
1.86918552	1.97187062	2.06826399	1.99075029	2.2896426	1.98032776	1.49243405	2.21191441	2.47907673	1.48097097
3.14913242	3.94413557	3.60570346	2.92079065	3.15785186	3.72264646	2.95735964	3.26482965	3.51637243	2.80382657
2.77279807	2.78000262	3.48583299	2.71993581	2.74902961	2.32974026	3.16792307	2.77131362	3.3398805	2.88838078
1.65367753	1.80078904	2.30631142	1.7581841	2.16238716	2.19902121	1.49353875	1.87899915	1.69383747	2.11176538
0.88009534	1.16696261	1.84801436	2.36585955	1.65488632	1.27550528	0.88803152	1.84391942	1.22136242	1.17128633
2.14117557	1.58294765	2.57638113	2.99173524	2.64334753	1.77183746	1.65922219	3.04006913	1.29508045	1.83274337
2.96040738	3.51928164	3.46052422	3.29505689	3.93641313	2.2768074	2.43297446	3.64082199	2.14306557	1.71940325
1.42856713	2.23246332	1.78686259	1.44563764	1.8443046	1.8175431	1.2110444	1.71220058	1.64105953	1.69877443
2.05417629	2.84487452	2.18227523	2.00616099	2.61785561	1.01178619	1.54557172	2.05870178	1.87765978	0.8768469
3.02669669	3.10379457	3.93227843	3.34682134	3.91782432	3.10949147	2.41768712	3.45896612	2.50487159	2.79738212
1.01573317	0.35906668	0.87757921	2.12539103	1.02861305	0.67000812	0.96196524	1.56699052	0.40644858	0.54546401
1.13000336	1.80686314	1.48611297	1.18301312	1.78288028	1.43022974	1.15932247	1.17601219	1.32854019	1.31274382
1.55975595	4.00058309	2.51208342	1.84173721	2.6865708	2.71849739	2.22624427	1.74361487	2.64437491	2.6629109
5.17014128	5.3480092	5.85421349	5.24301114	5.5778918	5.47896301	5.5075307	5.52313261	5.20095272	5.51329472
6.07326433	5.08860931	6.49880891	5.61952919	6.03035375	6.12806157	5.73504154	5.8874298	4.93567377	6.78944946
5.7538423	5.39686463	5.8191105	5.34326292	5.83955233	5.4545287	5.99821266	5.40796787	5.58808182	5.19348501
5.9207848	5.97915579	5.84391975	5.24219143	5.7344417	5.97421301	6.1539908	5.03806717	6.35571618	5.84942295
5.09280948	5.19299026	5.05737123	4.75447958	4.63778126	4.83121676	5.58013877	5.01992857	5.54014981	5.03747746
4.02261048	4.03068654	4.09965402	3.30638619	4.13128383	4.23202536	4.0457256	3.67791563	4.17275214	3.66371337
3.44877307	3.84890582	3.97939341	2.81259591	3.84319144	4.11772056	3.91592285	3.26438838	4.78614049	3.25478519
2.68218591	2.65936372	2.2071374	2.52220312	2.91767561	3.22518982	2.44973814	2.67324242	2.51282346	2.62364359
3.73946694	4.40705429	4.75892036	3.94448188	4.26359096	4.43935771	4.41785114	4.19006881	4.07473893	4.25244328
3.76254507	3.70626414	3.41610449	4.02150736	3.00043135	3.4862388	3.11659309	3.6288803	3.24097403	2.54535346
4.92033744	3.69767195	3.61663217	3.8669994	4.20098979	4.19112615	3.92636988	3.56939669	3.69093403	3.12610552
3.98276882	3.74639382	4.75207157	3.11838615	3.99983811	4.76344414	4.74444227	3.60974178	4.40612185	4.31382242
3.40472009	2.69085093	3.61662351	2.91749125	3.08119449	3.15719758	2.14930732	3.50067262	2.87665074	2.33049401
3.60954768	3.76905946	3.39614856	3.75521954	3.41153188	3.81698195	2.9615921	3.52686651	3.56079188	2.637461
4.13887405	4.40904294	3.37048242	4.90701934	3.25049799	3.95498026	4.53026159	4.23120816	2.89205563	3.71125257
2.847597	1.95616271	2.77717636	2.586588	2.69439035	2.98339953	3.47201723	3.00551044	2.3037452	3.89621956
3.45159464	0.28431777	2.68360536	2.63499005	1.83888798	3.67765502	3.94207403	2.20774257	0.23528705	0.87304517
0.73742995	0.90674656	0.8150986	1.14577005	0.84597882	0.43642281	0.37118982	1.39098103	0.70334748	0.60886481
0.7025601	0.6023737	1.90129124	1.32133985	1.09119036	1.54615847	2.1758686	2.4267634	0.30861358	1.33588601
0.99952432	1.09198446	1.56286469	1.24290423	1.17859454	1.11235919	0.66909308	1.11785466	1.20159422	0.85846262
3.18577766	3.68353576	2.91794783	2.94984803	4.250527	3.6979346	2.76817707	3.04011516	2.93939801	3.43847552
1.24948965	1.58290689	1.79146298	1.94852656	1.55096066	2.24810072	1.23451071	1.80441367	1.33981581	2.30865362
0.16826308	0.01332074	0.08512921	0.89439713	1.17468932	0.15578415	0.4208819	0.36863751	0.01496553	0.00819906
0.01250498	0.01592174	0.32390264	0.24158461	0.16451016	0.04611349	0.03723527	0.2316129	0	0.04836412
5.2747296	5.08969824	4.58170755	4.86931167	6.46467765	4.52321541	4.4840398	4.25240419	5.08465021	4.27798779
2.53473959	2.4220439	3.58998117	2.70459045	2.47273526	2.80684998	2.1012166	3.14404714	2.26609268	3.15391561
1.50111671	2.3712572	3.25227737	2.36607546	3.21385036	2.81276706	2.1150885	2.7698212	1.81208292	1.96067751

2.09086223	1.44759407	2.20806681	2.94505098	2.47624099	1.73113892	1.45858496	3.06542398	0.91364217	1.25107109
1.89083103	2.0189899	1.80763216	2.03827161	2.09732627	1.64061873	1.38963063	2.37411157	1.66781627	1.67052
0	0	0	0	0	0	0	0.02961266	0	0.0099227
3.84883596	3.54739169	4.36360279	3.73600018	3.7172672	4.83834537	2.77279844	3.38276357	2.80566216	4.58831758
1.99628235	1.93904222	2.16064392	3.74098904	1.07927479	2.29761982	0.39836115	2.59282296	1.52619778	2.40318708
0	0.21922754	1.77903991	1.6538962	0.02323886	1.57293255	0.03852812	0.69428679	0.04196716	0.98556554
2.43688131	2.84490688	3.1178085	2.74360312	2.93827785	2.90048919	2.48712556	2.97304547	3.29632322	2.49987759
1.49301347	1.91951078	2.06667889	1.55829943	2.84788349	2.00020483	1.42689629	1.79434255	1.96777846	1.6073956
3.01924095	3.09134457	2.67863415	2.93322703	2.97498357	2.66920978	2.39610741	2.70473982	3.00398304	2.59868826
1.29423439	0.44495625	1.05743231	1.0143509	0.86920447	2.18759644	0.53160373	1.50824431	1.87884634	0.77191738
2.11582684	2.61907492	2.47928782	2.49003153	2.48612728	2.6852372	1.89318616	2.20358698	2.5386008	2.29529882
3.50338933	3.83415717	3.42993579	3.3928168	3.59348464	3.65499263	3.43032197	3.84434151	3.90347778	3.84993006
2.15098497	2.82163577	2.17712661	1.71463182	3.16478054	2.48489909	1.59962731	1.83910952	2.30731488	2.13872665
5.2695236	1.1346157	1.80041156	5.31101889	4.3534295	6.53972423	5.64478911	1.36869408	1.22896993	6.49697569
4.01192407	5.44487162	4.6598863	3.19284482	3.88753064	4.22212345	4.22744588	3.36742913	4.52826966	3.6043438
0.39865074	0.19201384	0.18893709	0.38176644	0.35411134	0.65914978	0.62762817	0.38826208	0.49371588	0.20547634
0.29505232	0.17449261	0.14091962	0.27261277	0.21290394	0.19439802	0.19552917	0.21193678	0.06787956	0.08303717
2.42138209	3.40378605	3.3790116	3.06718749	4.11804298	3.08274045	1.89276013	3.46764804	2.6403846	2.32879461
3.47606602	4.34000748	2.66916353	2.91946301	3.5328754	3.77461862	2.66482457	2.87207963	3.65938729	2.85936914
0	0.01708966	0.03763234	0.0504521	0.02026948	0.03184813	0.0320441	0.06212077	0.06055141	0.80953485
0.37519211	0.46321881	0.57940375	0.11067798	0.31051282	0.38611542	0.1413365	1.24010667	0.36035071	0.41579022
0.91662109	0.49721059	0.46492932	0.38652387	0.43963302	0.83416138	0.38179071	0.70758653	0.62259897	0.3007135
3.77550559	3.87958699	4.54213744	3.68810031	4.08002895	3.90579882	4.36620622	3.40966807	4.04655904	4.05722463
3.68289353	3.0286092	2.52504947	3.20583516	2.17868139	2.52197383	3.36392705	3.02315588	2.46370092	3.1295302
1.96636873	2.46418451	3.29125106	2.67936339	5.11612545	3.62728961	2.82821716	2.38647408	2.40638443	4.10278984
3.50423404	4.11940612	3.87081847	3.0673399	3.31700785	3.43845781	3.57595977	3.58321903	3.43411779	3.33614027
0.8543514	0.30946962	0.27110981	0.62707579	0.24199195	0.1322931	0.06437486	0.8906209	0.35234228	0.07861025
0.13918741	0.13334507	0.04619719	0.26886258	0.20377444	0.10511189	0.14347971	0.53300554	0.05141976	0.26101527
2.59259071	2.92091976	3.43814886	2.76929104	2.91626892	2.9391605	2.66898715	2.95172442	2.38240051	2.24883271
1.2318807	1.05980424	3.28159045	3.27054585	3.85979532	3.0778227	1.45769493	3.09085427	0.77686549	0.43774217
1.86523265	1.03491344	1.15946414	1.63251245	1.6393383	2.253384	2.60929784	1.12988058	2.11803908	0.84861069
0.95136564	2.51187388	2.30671583	0.58748832	2.4996121	2.59382025	1.785739	1.57375153	1.7177959	1.11195727
3.40480322	4.08802661	5.47710063	5.8193571	5.44621479	5.03095665	4.87455422	6.25295138	3.46318851	5.61820101
0.76703075	0.27300749	0.36772016	0.84482218	0.5407262	1.5076865	0.73469819	0.77624015	0.38190074	0.37143926
1.91380556	1.83068818	1.68600294	1.73194786	1.56218048	1.95847074	1.72889278	1.82150764	1.81808943	4.38753605
4.99445703	2.08429885	8.86213021	5.17199077	3.85252026	8.67914739	7.82028292	5.16610966	6.14888838	6.56429531
2.11579992	2.23671994	1.97349652	1.34500363	1.92450897	2.18723851	1.48644541	1.5166071	1.86155215	1.77810604
0.49463657	0.68112248	0.54983065	0.68734424	0.42983456	0.45731263	0.40916381	0.7203613	0.57722568	0.35031149
2.9341755	3.6349227	3.72955606	3.06925378	3.90325187	3.63677797	2.00899214	3.34538563	3.19961867	3.00206572
2.007034	2.39165355	2.74076272	2.40920441	2.97234134	2.87361744	2.11000143	2.63803147	2.06592642	1.96901739
2.70899404	1.32526505	3.53433215	2.63706114	3.41711914	3.49823707	3.00544718	2.85994629	3.07918368	2.95923903
2.48501049	4.14007708	3.89471714	1.85014298	3.06757458	4.14961072	2.17313508	3.31975388	3.56152495	2.41642475
3.37711759	4.19877442	3.30215812	3.16933366	2.9775855	4.02009834	3.90819534	3.40230311	4.32936754	3.35686715
1.7656138	0.27262105	0.31833342	0.81680908	0.50749907	0.29607867	0.66439752	0.7706933	0.44576826	0.63452144
1.99172036	2.64528874	1.70464986	1.89533919	1.85471962	2.41956764	1.54205616	1.6629513	2.71353826	1.67703098
2.41267852	2.16702375	3.55003183	3.00870154	3.12863604	3.63903494	2.23529472	2.36224988	2.09474891	2.40243634
3.03051397	3.10622833	2.99183529	2.9076873	3.5659245	3.091306	3.01225158	2.87529871	3.0415734	2.95317506
2.70656471	3.23314978	2.76328281	2.6858165	2.8637653	2.69833809	2.61682395	2.88117488	2.73165041	2.77939335
6.39524561	6.5816245	6.23797305	6.71503925	6.55207207	6.61190494	6.58448314	6.34336321	6.77766652	6.50896493
4.10573457	5.53039852	3.35112248	4.03634416	4.3192072	4.64114387	5.09292856	3.65778502	5.31362206	3.67959173
2.65283363	3.46826405	3.45372623	3.04010519	4.09712915	3.25286013	2.89814138	3.595333	2.98367654	3.02985808
2.63923253	3.35896477	3.12350587	2.59956173	3.43337336	2.80676684	2.20027124	3.01542626	2.66318373	2.42583874
1.28930133	3.08162538	1.01468844	1.25518049	1.93005924	0.82549163	0.31430137	1.97256478	3.28211391	0.72596805
0.90741232	1.03574342	0.88089549	1.03245539	0.87629761	0.77944786	0.91920399	0.78122596	0.8061051	0.78404383
2.83387741	2.64858068	2.86144454	2.89258715	3.01508096	3.14003489	3.16229022	2.91669359	2.83218646	2.93077053
2.28212714	2.4549152	2.62449658	2.13524209	2.14595893	2.04832497	2.31398744	2.20398899	2.5744743	2.1164199
3.84238959	4.64049123	4.55702632	4.16491445	4.91162755	4.01883876	3.68730077	4.13051343	3.90014262	3.87946552
2.36542821	1.87455767	2.97253616	1.96789852	2.73307874	2.40453971	2.60287654	2.1978258	2.19710341	2.58001531
1.5572603	1.03855459	1.14161791	0.94633172	1.2620912	1.30436189	0.57406101	1.09781114	1.73928516	0.55379506

2.86414479	3.5753422	3.97468168	3.23158146	3.91479175	3.3297347	3.05513242	2.97952445	3.11803408	2.67726477
1.31356322	1.35344111	1.91241276	1.58575256	1.75251436	1.65603387	1.05458565	1.78923238	1.09381415	1.250171
4.09180653	3.41986052	3.57541942	2.90430945	3.49079954	3.16188097	3.5578346	2.93455878	3.3924083	2.94390634
2.21613847	2.22437631	2.78539707	2.09767794	2.96928962	2.05234453	2.00228023	2.66889568	1.84072308	2.50345694
3.13541785	3.47843092	3.01001564	2.81509005	3.05848724	2.85859625	2.92009525	2.90040943	3.22965743	2.83964975
2.3423246	2.47042711	2.0280544	2.06241654	2.55801867	2.03497144	1.67025173	1.98026959	2.26859141	1.88694088
3.55104906	3.98743958	4.51913999	3.17985996	3.45719116	3.6394615	3.65955538	3.24584196	3.35636022	3.29702204
2.86686725	3.15206473	3.86723301	2.4829977	3.57710291	3.21661263	3.10572373	2.5102715	3.20651981	3.08059504
2.02691996	2.09862035	2.1487539	1.91687406	2.09604533	1.86024894	1.47361383	2.19791511	1.80265156	1.73745098
3.52517225	3.66052705	4.96062442	4.04269797	4.31015616	3.59623745	3.77473533	3.7720225	3.26846264	3.9834805
0.37991235	0.18088181	0.75173601	0.17349622	0.24594112	0.78923365	0.93149634	0.87062809	0.1294114	1.77878838
4.28133808	5.84352986	4.55868898	4.26229257	4.29314931	4.54467387	4.74385658	4.40898825	5.17987694	4.62144468
0.69527962	0.97582251	2.23012931	1.21717682	0.72055791	0.8120802	0.32039896	1.23953914	0.82404071	1.01082055
2.74739168	3.72368327	3.41403772	3.32301802	3.33162254	4.00223979	3.23098267	3.13647765	3.10022098	3.19541253
3.15385355	4.62486035	4.48213211	4.11552395	3.76159841	3.75743213	3.48106307	4.30414316	2.05407819	3.996707
1.47841717	1.39895241	1.50076709	1.11304222	1.78421453	1.45579189	0.45973164	1.38403927	1.1436501	0.90161029
3.0401069	3.54839224	3.16839688	3.18466885	3.62275115	3.47312897	2.84357625	3.34303891	3.28036808	2.98947026
0.5954073	0.74967677	0.44219305	1.13598427	0	1.36023738	0.56164308	0.03177093	0.21746465	0
1.33085611	0.59389708	0.91645178	2.01635615	0.54647802	0.58114872	0.14201586	2.33136096	0.46471413	0.77129413
3.17842822	2.61296682	2.74706612	3.09160246	2.69081516	2.94164917	3.71866131	3.00999088	3.55528999	2.83309361
1.67296717	1.57424862	2.03550093	1.96661694	2.39787633	1.75039057	0.96705039	1.87509137	1.37232065	0.88263908
2.03496276	2.78409015	3.1673892	2.82796148	3.9356372	3.7265956	2.32395261	3.70226189	2.84308689	4.61935218
2.37754351	3.80953887	4.02002817	2.45973051	3.31472821	3.614545	2.74128019	2.57196112	3.67386452	3.32827413
1.65671402	2.69919243	2.48691722	1.7256928	2.10077371	2.13219514	1.15869281	2.54041464	2.00877321	0.9841314
3.86118143	4.21293435	4.0185056	3.71855199	4.03838429	3.3924565	3.18298377	3.8752378	3.97112678	3.58918308
4.2998968	4.64525128	4.92284414	4.31958842	4.68531039	4.61821861	3.37584486	4.67188576	4.38788097	5.05956495
0.52110717	0.57340798	0.06312527	0.39474282	0.18407743	0.0479042	0.03353814	0.2687655	1.23362488	0.01564586
6.63525258	6.77544379	6.72112474	7.03745041	8.74340185	6.85023376	5.8932323	5.78067614	5.94682643	9.16567789
1.03097613	0.81759315	1.32864581	3.00499858	0.51773825	1.28431557	0.04953294	3.00760172	0.1374967	1.35409418
6.7167227	7.43868072	5.38948421	6.61640773	7.48171385	6.74611917	6.90455165	3.58190992	6.92916223	6.99176651
1.37688214	0.54606861	1.33739725	2.29205584	0.86543447	0.52939003	0.34324362	2.74536025	0.53461164	0.67392728
5.48384988	4.85897001	6.19571201	5.15461362	5.2300106	6.16698364	5.65889851	4.95825377	5.0897015	5.94570952
3.96080313	5.29652427	5.00510901	4.73219032	5.0953761	5.05530454	5.01095622	4.741649	4.66186589	4.38664488
5.74654761	4.08661415	2.83300742	4.32404196	3.56460911	3.76922508	3.37265188	4.26431479	4.49747462	3.674252
2.78000627	2.48914136	2.92537821	2.3392103	3.04766592	2.64937726	2.29710365	2.22685426	2.39462586	1.88705219
3.17061877	3.31678156	4.48824784	5.64581555	3.78240954	4.47875553	2.9099412	5.69652145	3.62530797	4.00472315
2.80563801	5.16573251	4.15486673	3.05669789	4.129995	3.72660177	3.26934875	3.35832168	4.46166368	3.07654487
3.15229313	0.15378946	1.58630146	0.9900221	0.0830332	2.4817889	3.02136343	0.14252881	0.68506391	3.25854914
0.05334662	0.01476903	2.63155369	0.0579366	0.04340661	0.01154	0.02313031	0.06257253	0.07584545	0.01812757
0.27615972	0.01287512	2.942488	0.01918478	0.05029968	0.04472201	0.01515512	0.11470026	0.08469228	0.01580533
4.88805865	5.27336771	5.51419277	5.16557231	5.48283372	5.04857771	4.921715	4.68833305	4.90128561	5.8649847
5.07456387	5.25986219	5.77317233	5.32843164	5.21702612	5.64500415	4.95238784	5.22824153	5.13496755	5.69890921
2.78802121	2.72905429	3.25818591	2.80254983	2.50910974	3.76189274	2.55750049	2.77688132	2.42465133	3.35688404
1.23582272	1.00751622	1.58074522	1.29245636	1.61226648	1.63268641	1.18587964	1.17925307	1.26968487	0.95221213
5.34152323	4.87247144	5.20636322	5.19271282	5.27101814	4.53321019	5.52975146	4.28252178	5.34041292	4.90995634
4.31410056	4.33077768	4.57000656	4.26877482	4.54213915	4.4192507	4.83332647	3.91099843	4.23533672	4.40266393
4.37950064	3.32127203	3.66952355	3.83007095	3.85805235	3.78360445	4.17339056	3.59963587	3.4243588	3.9162727
6.74970287	5.385057	6.48467144	6.45204385	5.82665916	6.09999997	6.6286269	6.04809943	6.38628804	6.48089601
3.07980312	1.63961227	4.12689683	3.5664081	2.77595994	1.72617341	2.04563537	3.1515928	1.87957611	2.83963841
2.99122758	3.70818587	3.80045973	2.98184358	3.78549617	3.44093348	3.08873976	3.19774006	3.37689365	2.9844756
0.08828714	0.02159845	0.13930201	0.22982134	0.06319173	0.24897257	0.15385061	0.39572564	0.18351231	0.10318308
3.99101973	4.06861268	3.49631301	3.29542127	3.97391295	3.71762166	3.48216755	3.14132847	4.16909334	3.5178136
3.23843605	3.35612459	3.99881248	3.11719871	3.30630274	3.99636088	2.56886916	2.7797306	3.53342586	3.62366388
3.08625755	3.39852094	3.50504566	3.23075672	3.05516678	2.764869	3.6127514	3.16158415	3.36921202	3.43111335
4.86205518	4.84190125	4.73768373	5.09081433	4.40826392	4.68604981	4.72218704	4.69672269	4.87231998	4.89808434
1.04656767	1.10692719	1.06484796	0.93826606	1.19951315	0.87233303	0.83610611	1.03479699	0.58451178	1.0347052
6.90101273	6.51032813	6.41525752	6.34130047	6.07240381	7.0721018	7.57678964	6.62990286	7.44055222	7.67895276
6.1054147	5.64918914	6.01915215	5.61260706	6.02461896	5.82507385	6.10766842	5.69436132	6.73254568	6.11013832
0.73364227	0.17692104	0.20986394	0.91889486	0.14340193	0.30188362	0.13112082	0.48692579	0.29099662	0.22398985

3.05277302	3.07990989	3.90553869	2.78116367	2.85730946	3.16176856	2.61024939	2.27113076	3.28354234	2.85231952
0.99193943	2.49775	2.28082922	1.67069625	2.28400389	1.71992539	0.74989512	2.3065659	1.49638352	1.86341228
2.64835385	4.60725799	2.06661106	1.85775204	2.3856037	2.58378408	2.41640402	1.95489143	3.96928903	2.14618258
1.56209631	0.96382875	1.47979592	1.11082842	1.98697445	1.77077424	0.96236563	1.30644837	0.57275718	0.42962383
0.11956118	0.08607087	0.27708453	0.23617875	0.1192552	0.02997102	0.06594537	0.30067717	0.12715872	0.10717492
3.71978092	5.26746387	4.42545228	3.27605461	3.8532501	4.59575692	4.19141459	2.9894027	4.18542947	4.31181778
0.43370063	0.36306734	0.40666784	0.47003804	0.43553818	0.25940219	0.13537822	0.57288453	0.14754779	0.08661626
3.07520274	4.9605652	4.71405296	4.31688721	4.45598955	5.03758582	1.98018662	3.91592573	4.67594472	4.9347024
1.20477775	1.44872938	1.15374836	1.6787054	1.36228481	1.34819664	0.84874391	1.39306813	1.68325036	1.23222457
2.98942484	3.82593263	3.14023122	3.06132691	3.41130545	3.12047214	2.71993061	3.18362914	3.21048123	3.45357855
2.21325045	2.93776681	1.35956569	1.52762335	1.75973861	1.90709374	2.02896501	1.77629909	2.32015278	1.82950141
1.14132503	1.84512429	1.99812377	0.9409358	1.97567799	2.11909046	0.63999117	1.35104911	1.48414384	1.67678001
5.74289749	5.62220211	5.24403834	5.8495049	5.80154664	5.73044663	6.0820447	5.50185044	5.77450838	5.605259
0.12981963	0.27267392	0.1751292	0.13955927	0.21207252	0.25909236	0.0857919	0.12252348	0.15954315	0.04881485
2.91898601	3.95519791	3.3980497	3.39528436	4.80596981	3.54724646	2.17988295	3.35329286	3.70897167	2.07948825
2.47137818	2.7360358	2.50185287	2.36165466	2.94998063	2.58818737	1.99585322	2.52183095	2.68724019	2.6048692
1.44897379	2.01681602	2.21338911	2.44363007	2.41720351	2.02328922	1.48414258	2.52541576	1.58361518	2.0250548
2.15921296	2.28579959	2.56092964	2.14722283	2.57691606	2.0470733	1.99466906	2.35365596	2.23395615	2.00081696
4.5808291	5.40676265	5.25606096	4.58781345	5.21757309	5.36102646	4.57580923	5.0153842	5.11315083	5.02194678
0.76471361	0.84061707	0.74389151	0.66432403	0.6683125	0.77342822	0.53180751	0.93480844	0.98503096	0.64127353
1.23633717	1.26589444	1.04187439	1.01459483	1.53206514	1.64667812	0.926758	1.10962368	1.17330017	1.08300383
2.8811474	2.48590367	2.64856192	2.26632828	2.47490049	2.61464738	2.13161426	2.22172732	2.86170123	2.19953331
1.01210207	2.13789378	1.51015475	0.81642734	1.6894505	1.92167186	1.8070541	1.06645449	2.29952857	0.95558649
5.65546411	5.95366031	5.39353093	5.65441842	5.79722464	5.91150937	6.22762215	5.29250394	5.84933181	5.37824795
2.99566181	4.36189847	4.16415658	2.43882362	4.50834554	4.65491713	3.82145063	2.97664058	3.41806065	3.10001103
5.69163142	5.55932568	4.80355018	5.27034215	5.12653934	5.02751459	6.64991847	4.99336234	6.34709282	5.30802098
3.74535363	4.15598047	3.41016818	1.8918186	3.38744001	4.40751585	4.33515917	3.12883231	4.34887273	2.79019816
0.35538359	0.21951149	0.34393805	0.1270125	0.40623313	0.18432037	0.48052947	0.15546052	0.90881221	0.11655665
1.4706377	1.83560264	3.73486719	2.60932746	1.24751563	2.27042002	3.15997565	2.58461344	2.56172634	2.9071711
2.63700965	3.33581143	2.82907698	2.7456658	2.59210066	2.78101834	3.38983402	3.09293572	3.34660165	2.62683009
1.91433282	2.44287648	2.68060753	1.79093195	2.39896691	2.80679779	2.19677987	2.03511173	2.08358218	3.55932367
4.70799462	3.73581055	4.71329782	5.00216195	5.27974397	4.65849985	4.47896825	4.39082792	4.59550726	3.72192259
0.84815421	0.55996278	0.55931147	2.19776911	0.64358462	0.61506653	0.43346756	2.44021374	0.2523046	0.2337964
0.82645839	0.11827382	0.02213181	0.1046608	0.00312381	0.03653383	0.06550511	0.01162276	1.81625142	0.01545162
2.99835584	3.17609901	3.38007733	2.87578825	3.51330411	5.11426022	2.74376002	2.97537523	2.8774816	3.65472566
1.49471245	1.74119001	2.01760906	2.32365368	1.85860918	3.84520251	1.85315376	1.87836427	1.17788565	5.42670967
3.43540815	3.51441278	4.514828	4.94638922	4.60947011	3.92295483	4.19078943	4.73160185	2.99258922	3.3379354
1.11825877	7.328898	2.75051082	3.79325431	4.72670946	1.94578391	1.87438407	4.11692204	4.91009431	5.51635783
1.69957285	2.10241441	1.97188501	1.82683229	2.06443618	1.70287523	1.57289275	1.97112104	1.43318746	1.60875211
4.18371643	5.42483652	5.29932475	3.77740119	4.07767878	5.19135678	4.07156402	3.6866491	4.79854239	4.57868325
0.38549646	0.42182772	1.15577974	0.82487896	0.85192917	0.70990629	0.30425294	1.38444951	0.45454066	0.46190255
9.7472035	9.55688359	9.27698648	8.96937145	9.53126886	9.00151333	8.76969745	8.71258546	10.3358524	8.55173146
0.98594216	0.55772538	1.72742217	1.76352993	2.39496272	1.08150828	1.36188951	2.4772928	0.5161707	0.72971908
1.22402925	1.57146168	1.7242385	1.66421123	2.35163011	1.92976884	1.01998935	1.53621204	1.09137089	1.13100175
2.95106232	1.37346803	2.646709	2.89742863	2.41576648	2.70370795	2.5114739	2.28626883	1.57806137	2.71191072
2.80909206	0.21440669	0.94838539	0.38674092	0.04451939	0.673978	3.26734446	0.57212841	0.92484003	0.62857099
3.49174913	4.40977634	3.3783544	3.67478502	4.13880054	3.34411025	3.29510818	3.81287674	3.15634672	3.5243746
0.19728842	0.19799999	0.23455922	0.05187782	0.1244715	0.15148851	0.03721596	0.15884912	0.27811906	0.16784988
3.7611244	4.71692533	3.26318367	3.63364105	3.98403478	3.41767697	3.71030981	3.51944656	3.88476212	3.52073187
3.46510769	5.54304329	4.41244776	3.78990405	3.9463997	4.14231567	4.09156928	3.75378268	3.74981103	4.95730771
4.78057661	5.09271452	6.8294013	4.68552453	4.57223282	5.72366293	5.90973164	4.6466514	5.02066419	6.513301
1.90231097	3.34834743	2.56709911	1.29488635	2.00105106	2.91052673	1.70338139	1.82551152	2.20836482	2.88466695
0.011452	2.18605615	0.21892134	0.24129195	0.21516347	0.00855399	0.07567514	0.14163829	0.0956139	0.02676574
5.90161002	3.3214307	2.27830863	3.73609774	5.69440364	3.13749132	2.45078591	3.67172502	1.07792141	1.54635708
4.65279363	0.63709373	3.60553682	2.57882554	1.59518729	4.09588935	4.94996332	1.92064121	1.41374183	2.93498462
2.68666887	2.05651688	3.30901515	3.68651718	4.48961939	2.54851142	2.5717069	3.83420321	1.49026724	2.29436008
0.39661595	0.18481622	2.61939306	0.85115427	0.84391556	0.27123194	0.26571838	1.19298192	0.24775799	0.46780402
2.41567978	2.78580433	3.38909594	2.64641008	3.59737002	3.00020851	3.35016936	6.14022104	3.77256029	4.29986259
0.65364666	1.25503646	2.5149881	0.68147462	0.63967868	0.1479007	0.08201021	0.64442306	0.13183159	0.45005359

3.98917214	4.75821933	4.28133337	4.53149258	4.95669155	4.41072629	4.22821658	4.57832123	4.13753954	4.17684704
3.52780967	3.50968533	3.90357201	3.42416732	4.29777737	3.67654558	3.68298254	3.91087558	3.77985359	3.5586173
1.87743293	2.32159935	2.64838452	1.71758204	2.3045611	1.84660082	2.13605875	1.84335885	2.39510311	2.49182484
4.36724706	5.15065339	5.63506653	4.60753681	4.80451621	5.06747646	3.97530247	4.96137261	4.9721882	4.61555247
2.49201716	3.00604073	2.60968333	2.91173869	2.80470787	2.59973099	2.99257638	2.67877441	3.05697154	2.7453476
3.70929365	5.013462	3.84424955	4.39272313	4.32066392	4.01606201	3.79542488	4.05770924	4.43556794	3.5103115
2.38244949	1.77504414	1.9977989	2.28537125	2.56422128	2.72267801	2.34584506	2.44723904	2.69863883	1.26751103
0.79764687	0.37519252	0.30116043	0.72495533	0.14491788	0.13957676	0.10392702	0.73053226	0.30169129	0.12461896
3.57986406	3.58872088	3.73244419	3.55558897	3.30525635	3.969917	3.58858143	4.06907591	3.89461344	3.67965475
3.93717496	5.02049191	3.47016128	4.77234098	4.72011639	3.07203154	3.10971097	4.68689199	3.70595735	4.74311758
2.21473605	0.1721857	0.53402071	0.70215519	0.82823599	0.46278411	0.22477997	0.94566021	0.17467204	0.15778513
4.53008435	4.47370901	4.58855849	4.48995926	4.28308529	3.65144318	4.18980962	4.0631511	4.81748042	4.03079869
3.84016727	3.4435263	2.787554	3.10962156	2.96857163	2.9134603	3.20359762	2.73242529	3.58345895	3.08462223
4.20823563	3.7845515	6.29678239	5.61375266	5.43983701	4.92231298	4.79191609	5.15070601	3.786041	5.00046812
2.18483242	2.82431801	1.90960843	2.05816698	2.29197659	2.77576412	2.25518871	2.14094903	3.38250535	1.7537281
3.0948688	3.43005713	3.25955928	2.9191901	3.31168426	3.50595148	2.94291035	3.23915498	3.12911579	3.04145128
5.57352085	6.05884508	5.90383608	5.72496898	5.78063581	5.88179803	5.38344379	5.56711645	5.45482949	5.68843586
2.28665533	1.16317567	2.13266908	2.32754436	1.70167617	2.18887568	2.1924959	2.11663832	1.02488996	2.89125933
3.03047864	4.36279329	5.00843553	2.81739473	4.05836545	4.10860295	3.85532509	3.26800083	4.46970688	4.93764743
4.66118843	2.46273052	4.11410705	3.31170349	4.63415404	4.39660201	5.5332436	3.61558855	4.35024071	5.92087801
4.9086255	3.98223761	4.21148752	3.86143287	4.65918073	5.54223349	5.21145526	3.85509787	4.58863052	4.7878884
1.89266928	2.14912763	1.26332798	1.25974193	2.58275151	2.36089591	1.46046606	2.74050012	1.43174616	6.04234636
6.31856509	6.73023648	5.83462861	5.82068917	5.96873683	6.57131721	6.46633447	5.52266104	6.92354373	6.58989149
3.61279838	1.04379146	0.53254923	1.66989969	1.66726973	0.84238625	0.17687642	1.14958108	0.4705105	0.70688958
1.45761975	1.38381933	1.40090949	1.24088974	1.60193531	1.52540586	1.22303696	1.65459664	1.4986726	1.271389
3.90425868	4.00865508	3.65819053	3.8989779	4.14864544	3.81747057	3.61100097	4.1496567	4.03621923	4.23304882
1.11956875	2.29287182	2.22832758	1.07706061	1.68952404	2.00693148	1.51712342	1.22869828	1.91571154	1.00944903
1.42589691	3.76000946	0.9862075	1.6921622	2.62218393	0.89345111	1.59966267	1.03063419	2.19812873	2.17635355
5.33228784	3.42666174	3.61129137	4.35037442	3.9483068	3.79673572	4.46644222	4.57922117	3.87001823	2.56297596
3.28938033	2.4855683	1.95150626	3.31373879	4.12134059	3.37873744	3.13365285	4.30900296	3.51922986	3.08630152
1.90487655	2.28929115	3.79391381	2.51442374	3.0254289	5.22875515	4.34492279	1.80480302	2.92016027	3.69011696
0.91026451	2.14629834	0.78131274	0.73187648	0.74939728	0.71780842	0.70090603	1.53462585	1.40436574	0.38082683
4.77464364	4.74339286	4.53574872	3.51418917	4.59195516	5.25742009	4.6718794	3.89706342	5.47515591	3.90409177
3.46322889	3.53855714	2.96934566	2.21516539	2.82963032	3.49530551	2.89532138	2.94643961	4.0570201	2.60165941
3.87363639	4.07794821	4.70744967	3.8478173	4.96129434	4.73741955	4.38109531	4.27674946	4.19587088	4.1417253
3.45415537	3.83779727	3.95294062	3.65157246	4.29027606	3.83163779	3.5281394	3.79674355	3.66937717	3.72029135
1.42543109	1.41319659	1.18108039	1.05017213	1.3617465	1.19453208	1.22074217	1.2388325	1.62234961	1.29800193
0.19716651	0.36834599	0.34883936	0.45709976	0.4029164	0.34412103	0.18336165	0.48207158	0.40282491	0.2240988
1.2507629	1.6698034	2.16426615	1.87387327	2.67073523	2.23429688	0.59170408	1.89777248	1.17074298	1.89431067
0.88088773	0.83600222	1.56131512	1.08812745	1.15496491	0.94728421	0.50247718	1.05403539	0.42307591	1.06934732
1.3835352	1.69525655	1.44413608	1.6256618	1.68188411	1.69193377	1.46978719	1.75232221	1.52490594	2.41873616
1.56664569	2.38083556	1.70155459	2.01401183	2.59124961	1.87077368	1.02157948	1.81906563	1.5614759	1.80604237
2.01419343	3.04685915	2.79128954	2.46945337	3.25114664	2.31462057	1.9137008	2.38046323	2.35350123	1.74606667
2.12097034	2.49036285	3.1627949	2.34793413	2.45942434	2.38847076	2.29384054	2.31697608	2.38177791	2.38247087
5.95709969	6.35314653	6.7684748	5.71734092	5.98575624	6.30743478	6.16892196	5.07376912	6.15763759	7.4965906
3.17013706	3.63447613	3.95995386	3.75987356	4.12301534	3.58987855	3.95574866	4.13560181	3.43197492	3.6697562
2.34958116	2.13561449	2.37123956	2.35630461	2.24032029	2.07126221	2.20540237	2.33461516	1.44933215	2.22251751
1.36017703	0.79620072	1.08427566	1.76522953	0.35857437	0.45828242	0.92036211	0.28997429	1.1647258	1.64454962
4.58920486	4.87435338	4.05132756	4.27122187	6.10862023	4.2209444	4.44184856	3.8746696	4.95475478	4.07564526
1.26887665	1.6857147	1.45071643	1.78446296	1.571828	1.23253778	0.802631	1.57877269	1.2497007	1.37098931
1.79840105	0.4442484	1.69031784	1.63295586	0.81608232	1.4433842	0.88397587	0.77502574	0.60599473	1.14718294
0.85918436	0.80448615	1.66074616	0.78295394	1.80163416	1.87774019	2.10707193	1.85787216	0.75064962	1.18770068
2.95889195	4.83812091	3.23032858	2.79610507	4.27964388	3.54010683	4.0163126	3.27437629	4.43667256	2.9595851
0.82875378	1.22693885	0.83819513	0.90914358	1.15986941	1.02319208	0.67775849	0.85393463	0.70365966	0.75838588
1.39010339	1.51306537	1.14334723	1.31883312	1.78309704	1.61481778	1.06496273	1.29638481	1.34770653	1.21801613
4.15683786	4.40243502	4.19329685	4.3281324	4.33139029	4.4915352	4.4599586	4.12142123	3.77514479	3.85425604
3.22754421	2.81317164	4.04214711	2.53552882	3.06594279	3.3647218	3.40200638	2.63582742	2.9961392	3.0485909
3.06743539	2.98402692	1.51540366	1.60483966	1.37400153	2.74016334	1.67778918	1.43894696	2.61798887	1.97110985
2.23041693	1.34398343	1.31279386	1.48043857	1.33187035	1.9775959	1.27995355	1.35156479	2.3613497	1.53966708

2.43912478	1.12424647	1.60836865	2.03206851	1.41698436	1.78698447	1.17681057	1.99331768	0.91695487	0.70183158
4.26584789	1.86135102	1.91774052	2.05811559	2.46801973	2.761077	3.97300339	1.85726435	3.09049485	1.59307943
4.52127704	4.52637928	5.18758845	5.09554298	5.33045926	5.34044092	5.20751318	4.08232362	5.05415643	5.97646475
2.45726418	3.21881967	2.44019033	2.19100098	3.01276192	2.34515513	1.88196747	2.64181391	2.6923348	1.84196847
3.41174359	3.70406103	3.56457841	2.87698983	3.9065699	4.00092097	4.38081525	3.19661625	4.00680567	2.93401033
2.70309028	3.17985577	2.70516257	2.80954862	3.1453781	3.05038477	2.53782678	2.98900045	2.28071421	2.77990892
1.80975975	3.79338192	2.29380052	1.93919492	3.2209494	2.35352178	2.5565408	1.33878113	3.17103397	3.06896244
2.91927309	3.51294977	3.14178724	3.4253696	2.81105231	3.09764871	2.56412518	3.51685741	3.44162393	3.38701494
5.65891765	5.68442693	5.55632649	5.61809057	5.80706029	5.52820992	5.61036839	5.27762723	5.61984343	5.34243656
2.76054166	2.94080959	2.94883295	3.85250638	3.05301372	2.58545372	1.92086509	4.04859111	2.88235518	2.35728343
2.6766066	1.90035485	3.04347061	2.77199287	2.50159412	2.3644538	1.30430827	4.2507796	1.98892217	1.78403803
2.32580275	3.00358846	2.44215909	2.24523475	2.46820505	2.62168273	2.07617752	1.64127317	2.46885006	2.07838057
4.4008373	4.17168202	4.77852642	4.36488425	4.80585822	4.51720776	4.64413896	4.39918607	4.21147874	4.50751111
4.26062811	3.67854552	3.53192049	3.97825374	3.65489431	4.81756484	4.5297038	3.96947119	4.14782961	3.45152425
2.27599295	2.89022826	3.11118488	3.22535041	3.48106371	3.03947423	2.09999953	3.19033869	2.40657702	2.76786508
3.01255544	3.70635325	3.9844946	4.15281911	4.24458952	3.74710632	3.99449034	4.65404414	3.4030116	4.02847087
3.61346732	4.11522673	3.69954642	2.99259372	4.25576326	3.69687668	3.46946151	3.24311975	3.90744784	3.2143041
2.21941097	2.76648009	0.82831694	0.58792508	0.06977214	3.01398948	2.08926335	0.81265667	2.11998222	0.57211724
2.54174529	2.50996984	3.39725326	2.85342461	3.56518249	3.00498385	2.27173778	2.90570409	2.49033377	2.54081132
1.97599499	2.03133345	2.72982545	2.17632954	2.99141159	2.52642869	2.28257971	2.90718813	2.3524157	2.23144307
2.57152943	3.3055092	2.59624517	3.19150119	2.94045181	3.05631121	2.80169656	3.45557426	2.66775264	3.02535373
2.52832593	3.26507948	2.2559916	2.39564505	2.86180008	2.61695921	3.10722293	2.44898134	3.02895711	2.53906485
0.15518249	0.09384593	0.28969231	0.27623726	0.16707178	0.13601107	0.18656494	0.2287414	0.11801388	0.1616856
1.76328305	2.2001778	2.21636665	1.37948327	1.88213334	1.87516163	1.46868624	1.77310518	2.13280378	1.31884923
3.05123531	3.14103395	2.83860008	2.97515298	2.86929408	4.23510316	2.80578099	3.01435517	2.50356879	3.19572904
2.03322381	0.20910857	0.69836375	1.66908954	0.54892879	0.65051033	0.49730799	1.12093619	0.20700927	0.75702164
0.46284902	0.13156611	0.5021358	0.75224927	0.60371846	0.14195648	0.13173148	1.26124484	0.06064691	0.4225161
0.16695263	0.06435052	0.03741952	0.27986621	0.61232047	0.1463997	0.14726565	0.33876194	0.08215854	0.01150633
1.79614219	1.82567659	2.1285063	1.63726884	4.18631575	2.03186227	1.96970558	1.54809409	2.06471056	2.25133073
4.16034437	4.88594178	4.48885816	4.39208593	4.81442601	4.31926982	3.89935954	4.11948353	3.56356095	4.31899021
0.25553364	0.12780632	2.69910216	1.6205563	0.16634123	3.7460008	0	0.93515011	0.14290767	0.61466195
2.04686564	0.15500538	2.76253481	1.23576389	0.40650908	0.49738571	0.05670182	0.79321265	0.56999148	1.44801982
3.75623755	2.59508676	3.49020382	3.86092125	3.0260752	2.72703041	2.52522323	3.39919226	2.79942971	2.47365891
0.97430418	0.1705446	0.28101689	2.09104228	2.0194945	0.5702634	3.35389672	1.47708229	0.77001682	1.34595488
3.7330426	4.3118751	4.34327921	3.58477407	4.40219149	3.99219661	3.88226401	3.86043305	4.20722043	3.84543857
0.50099109	2.00513782	0.41066413	0.85016174	1.16064588	0.54104951	0.23309207	1.06462328	0.64805709	2.52501921
0.37736875	0.1677439	0.12485759	0.09350056	0.37791565	0.30592663	0.05604753	0.49966926	0.35299403	0.23764264
7.83407385	7.74631951	9.55812671	9.56200196	9.80393743	7.99998721	9.16027574	9.91899197	7.32129907	8.19927126
0.45992898	0.48175711	1.21008243	0.88621815	1.09178332	0.58856515	0.24500682	0.8181635	0.59017675	0.69409268
1.62531502	0.50293676	0.73796023	1.57162711	0.73778678	0.95836281	0.31133539	1.63870207	0.74081466	0.20885747
5.41534935	5.94462021	5.77910781	5.65122276	5.32591385	5.37658532	5.67204266	5.57470422	5.10255281	5.67431074
3.5688637	2.4709977	3.56742658	3.89236417	3.37769441	2.78112855	3.14027361	3.5987906	2.4901475	3.16867514
0.02551275	0.03778099	0.01644466	0.01624106	0.05832649	0.01908001	0.0318561	0.010061	0.02438209	0.00670438
2.09594741	1.33626309	2.64306341	2.78742179	2.01505407	2.42028699	1.89799388	3.16090164	1.21371929	1.3374783
0.79638093	0.75645452	1.54476645	1.19890759	1.52919175	0.92081926	0.7694237	1.28638237	0.66867951	0.98052622
2.69169872	2.86728234	3.05237107	3.19640725	3.26957914	2.96590108	1.74080777	3.63930778	2.90416722	2.38851476
3.12505843	3.31333052	2.87506604	2.81270244	2.73062711	3.39969193	3.25701612	3.09960164	3.74171956	3.94386355
3.82090578	4.24791057	2.30767736	3.29887051	1.27887499	3.58439631	5.31578861	3.59523682	3.86108199	2.84636943
4.1180445	2.04754821	3.18716377	3.6139186	2.93469528	3.8417435	3.31153779	3.43752319	3.7130529	3.43998899
3.32005293	2.21383977	2.13209978	1.76522387	2.46012665	2.25860599	2.27506348	1.57706585	2.4521111	0.88130929
3.70472207	4.31502865	4.87021851	2.81719298	4.01941588	3.53980691	2.92695746	3.04608687	3.282146	3.08828369
3.93019325	4.55411401	3.5047314	2.29827637	2.51133688	3.30662659	3.97362746	2.27570742	5.05340558	2.50449435
2.39842189	3.12615029	3.25758576	2.19985234	3.08986416	3.05252647	2.27577506	2.61721775	2.17022468	2.0569894
1.0226583	0.64488463	0.80650502	0.10636831	0.65497175	0.81542639	0.85984622	0.51165094	1.13115557	0.17111123
1.64473358	1.9820804	1.82221747	1.63612385	2.04355569	1.85232361	1.30303196	1.47339512	2.07843658	2.82213265
3.14843417	3.11101283	3.14798558	3.15510368	3.70092029	4.01929168	2.83519731	2.83469411	3.28259501	3.70370358
0	0	0	0	0	0	0	0.08016353	0	0
3.98001071	0.52486296	0.38732751	1.45515356	0.62162244	0.66174298	0.12211982	1.15075282	0.29985441	0.03405937
1.13408094	2.18102942	1.41952554	0.51473683	2.04575412	2.10725674	1.12282346	1.2665977	1.5222087	0.88613145

2.33956445	3.30975761	3.03462007	2.4974056	2.70871048	3.80796672	2.26315461	2.81215185	3.08027339	1.55297548
4.85634378	3.51793398	3.93235898	4.19354674	3.80563961	4.23586256	5.02963289	3.8759121	4.83237562	4.28023639
3.60121731	3.24214542	2.77579101	2.76707891	2.5775086	2.80105923	3.76446968	2.78994424	3.57574648	3.20922804
3.40291483	3.31354701	2.64709439	3.10857345	3.55055554	3.03302111	2.8844985	2.93072178	3.04789437	2.93923564
1.92953778	2.22208566	1.67559923	1.74909808	2.11519531	2.67029377	1.58511721	2.0190721	2.2737207	1.89894708
2.54944798	2.71308209	3.01202973	2.49061484	3.03560951	2.62783284	2.34172033	2.56565139	2.53788832	2.42161267
3.6456485	3.49718569	3.87538003	3.84359802	3.97989879	3.97729115	4.32635641	3.84275384	3.12220901	4.13070903
5.77734146	5.01776232	5.09599211	5.47727198	5.3813416	5.75292173	6.05294614	5.88647219	5.86795101	5.09907669
3.00213356	2.84465461	3.286726	2.84989801	3.1150709	2.69890111	1.62694597	2.4965051	2.67332024	1.89426941
2.96107576	3.72133611	3.57526577	3.31914074	3.93184524	3.57752696	3.02493405	3.08121144	3.55947325	3.29536175
2.66810533	2.85635375	3.5518079	3.19765356	3.72347562	3.04458295	3.63906314	3.96583426	3.17056639	3.04420844
2.47715871	4.70944051	3.76546472	2.66277223	2.0828436	2.28598268	2.68248951	3.71125127	3.88340175	1.7475461
0.08684702	0.09103731	0.36037723	1.48336953	0.20979376	0.66745187	0.01963357	0.69461466	0.05187338	1.18401549
3.56197118	1.14806573	3.28832774	4.55685571	3.10743779	3.85326577	3.72975663	3.81068426	2.2702373	6.26807842
0.19759044	0.07609797	3.57866407	0.88982596	0.19987583	3.10201896	0.06543945	0.49475016	0.22163161	5.42690002
0.76545318	0.22155569	2.58727386	1.1927441	2.43602736	2.01632535	1.80199354	1.66408082	0.22539768	1.08749583
1.4948247	1.77944246	2.43829163	2.28181323	2.49906491	2.18567652	1.41510166	1.97087263	1.67046511	1.85668316
0.05168028	0.14766625	0.98548498	0.86434024	0.42762693	0.69467062	0.17243901	0.70322949	0.24524341	0.30907114
2.51854389	3.68991411	4.03927077	3.52962274	3.62937963	3.08236502	2.37526457	4.19315063	2.70033384	2.91133921
2.49504443	3.1530394	2.92754896	2.08312932	2.79237436	3.09206325	2.61725051	2.72799803	3.03942466	2.27481712
1.02230993	0.55674578	1.11989517	1.87225001	0.95714357	0.9708748	0.63385725	1.96083674	0.3973135	0.76881902
1.95356447	2.18694027	1.96303879	1.7090716	1.69573888	2.07698805	1.96272234	1.64130872	2.23249266	1.47518941
2.63771457	2.1074172	2.65461814	2.47619657	2.8855285	2.90048379	1.87183742	2.78257576	2.17587238	2.44054454
1.56991672	0.76935547	2.78156098	1.87304193	0.9955991	2.01945473	2.38341272	2.01918011	1.24703924	0.94858456
4.07760564	3.83059787	3.74327081	4.17924852	4.04867096	3.85894393	4.09931914	4.11947237	3.95970351	3.22375693
0.08119228	0.12119362	0.07833211	0.09609657	0.12601647	0.22994971	0.06887812	0.10653356	0.11497582	0.10256602
2.3745076	1.63268903	2.61745859	4.35462982	2.39396309	1.58643047	0.71939079	3.94812827	0.86288481	1.34345096
3.71747174	3.94576515	3.83932904	3.74179607	3.76658678	4.01846715	3.61948574	3.84425506	3.9778923	3.19584429
2.40466067	2.17704687	2.00386862	1.96671937	2.39368953	2.13295496	1.68140863	2.28834365	2.04723898	2.05816854
4.21998555	3.72731608	4.75073079	5.10743099	4.57533718	4.31567399	4.32970118	4.99930613	3.38345959	2.77834346
0	0	0	0	0	0	0	0	0	0
2.5296798	2.80595139	2.0776353	2.28362722	2.40917267	3.20999329	3.88912933	2.38013172	3.14703187	2.14321336
1.69170503	2.34486401	0.92626136	1.26668111	1.32339015	2.81802788	1.59969096	1.62055117	1.58626996	1.33181226
0.04787876	10.567233	5.58969692	5.3174212	3.87740432	0.00724701	0.04320909	4.01224619	9.33155949	0.57684144
0.01228117	0	0.03135601	0.17656795	0	0	0.0092309	0.75194969	0.01756618	0
3.68997018	3.5265316	3.23026528	2.88853342	3.79323133	4.23061378	3.06080389	2.85158187	4.09571245	2.78932856
2.80490529	3.28449342	2.75983431	2.60987869	2.91570319	2.66312197	2.22879422	2.97909115	2.88050022	2.41491147
1.5476645	1.79861918	2.11069822	1.55021651	2.00121545	2.00861544	1.48694956	1.92131164	1.76711909	1.84579022
3.37589636	4.02447037	3.77114967	3.19927651	4.7917775	4.14054299	3.36844749	3.77658267	3.80410998	3.75860223
1.28738163	2.63988078	2.06953829	0.92136438	1.75497328	4.1038873	1.35629848	1.3087391	1.67850414	1.09613929
0.77345956	1.75664228	1.13445958	0.38419205	1.28049639	1.5628743	0.92799433	1.15315485	1.55347932	0.91288209
3.09598675	3.83382345	3.64518544	2.54881033	4.59093436	4.08779978	2.98251664	2.70274042	3.58817168	3.88251546
2.91857681	3.59173224	3.12641247	3.15980811	2.77757779	3.28507324	2.85056894	3.47890091	3.16203397	2.74034765
4.76397568	5.36011737	4.98535735	4.74327883	5.01093036	4.80840417	4.70789089	4.82690842	5.36711588	4.45415783
2.84613739	3.00195724	3.11668913	3.06219819	3.30305897	3.18927854	2.33155583	3.40285047	3.40947068	2.60745284
3.543116	3.60307125	4.03803454	3.75880521	3.33850301	4.29168245	3.78240147	4.3141064	3.84438082	3.47785511
2.92501481	3.45254345	2.54693193	2.70990938	3.27946081	3.66270514	2.85993693	2.82044573	3.38202955	3.23683986
1.90874647	1.97116246	1.83909666	1.80534426	2.33028951	1.86187432	1.57520277	2.01746431	2.09557596	1.63677634
1.02465694	1.71714625	1.51907676	1.19620154	1.22664938	1.30573442	0.77814902	1.46341722	1.52728831	1.00042226
1.86343909	2.35411878	1.3194657	1.7391406	2.18836048	1.98106814	1.48494943	1.56704664	2.16252452	1.69288071
4.51221365	3.46193155	4.42126668	4.11262401	4.20668428	4.27344171	4.50347742	3.93451044	4.35196229	5.3326687
3.73019706	4.423292	2.55651169	2.60944629	3.34205942	3.55908611	3.70673924	2.36943208	3.77830639	2.64766337
0.63467322	0.93306111	0.63108187	0.43704112	0.78437054	1.19973957	0.25838018	0.81282237	1.42462495	0.2685494
0.13286215	0.02443205	3.29859386	0.41312247	0.12151641	0.0114929	0.02303629	0.28026384	0	0.19775591
0.52342424	1.47304282	1.3446776	0.32108847	1.1530876	1.21963468	0.71979021	0.40445169	1.42089623	0.48752634
2.51689976	3.18463423	2.34680628	2.01895493	2.42483299	2.62893313	2.87527389	2.19772843	3.17015471	2.21941533
6.94672746	6.35462443	6.64438646	6.65267398	5.92409982	6.13865632	6.67057477	6.79584291	6.94050098	6.11098058
4.08390934	4.51609494	4.25553958	3.85180469	4.22317755	4.49971812	4.62359747	3.72413204	4.33154327	3.97233311
3.04497778	3.43160608	3.36450251	3.04959153	3.93506251	4.11059548	3.28208073	3.21228091	3.31970914	3.03995857

2.60726442	3.10164008	3.27815623	2.70987699	3.01651172	2.70424279	2.54312485	3.29069505	3.05954094	2.6122602
2.74202143	4.32140227	3.34057088	3.34268329	3.83273354	3.970523	3.09896061	3.53074139	3.37861281	3.45537534
0.84437808	0.52711088	1.61449254	1.38848085	0.70687642	0.83566183	1.00753307	1.40126664	0.62173359	1.3857609
0.42175088	0.08129856	1.05872208	0.50427851	0.39768528	0.53398599	0.24690436	2.03064798	0.18779129	0.25952546
1.38652258	2.21548215	2.13554221	1.24832788	1.8622904	1.5433749	0.91345652	1.57020377	1.60659319	1.45974126
1.16971681	2.35530925	1.74352072	1.18036546	2.01108938	1.95190316	0.88977516	2.47482819	2.36678623	1.70417189
1.28743692	2.41859123	1.29567763	1.73597492	2.0388111	1.76993213	1.48093261	1.43392111	1.40122618	1.65898613
4.13362895	4.03562421	3.78810515	3.83534761	4.02186383	3.32475748	3.48359514	3.71916948	3.87454216	3.70941122
7.7739595	7.72933627	7.69848048	8.02530133	8.2192808	7.07999028	8.08842156	7.94718156	7.93503004	7.7422263
0.12812147	0.17093019	1.47735146	0.45885189	0.10739287	0.0992597	0.03041567	0.50324382	0.21741903	0.15856792
2.83124039	1.01794613	1.36194931	1.99804813	1.66861424	1.25938394	1.39180753	1.8787266	0.95300124	0.70712694
6.91589381	7.82243824	6.18184268	6.87267495	7.06061775	7.86891305	7.85034645	6.47871536	8.41230219	4.26140775
1.73056584	2.36165293	2.09916192	1.6561173	3.06199977	2.803054	1.08910544	2.17451756	2.46179484	1.64988589
3.66748276	3.72942507	4.19236032	3.46197595	4.79120862	4.08222593	3.92619055	3.78541719	3.48544177	3.88448459
2.41394584	3.21323806	3.77170346	2.65936177	3.60935464	3.28718955	2.51507566	2.75632597	2.92895405	2.93640311
0.04934209	0.05492906	0.32366145	0.59769881	0.26000718	0.27225926	0.41476081	0.39758174	0.15338769	0.58238745
0.14319727	0.02116687	0.02133721	0.23458803	0.03130578	0.02474993	0.01250503	0.23556138	0	0
2.39806017	3.41809741	2.56680489	2.98106049	4.10677173	2.62593079	1.57314769	2.47895793	2.38085794	2.91409873
4.09726642	4.75415319	5.12695591	4.29549886	4.59047982	4.55041172	4.34575362	4.59788623	4.51900806	3.30664196
1.83985976	1.93182263	2.22670424	2.23369076	2.30787486	2.21557532	1.52404419	2.50438823	1.79336318	1.98784051
1.82777286	2.46660402	1.71367093	2.04279558	1.91903609	1.95188411	1.38844892	2.15207033	1.6266426	1.64354188
0.77690621	0.61402497	2.07999149	1.90480168	1.94825048	1.39527734	1.09271531	1.28862682	0.61586228	1.92370814
2.55206447	2.95863302	2.32735864	2.7079134	2.92586257	2.07038413	2.21540643	2.68971355	3.01223291	2.12099799
7.13090672	7.54378908	6.74932065	6.80711958	6.3294116	6.52813615	7.31669703	6.71213108	7.7684877	6.4621425
2.18970713	3.19187776	2.70238379	2.42213592	3.05207769	2.60470622	2.06916523	2.50960491	3.25029724	2.677515
6.20317521	5.18259313	4.48742231	4.87480613	4.70778597	4.27024387	4.14626843	4.64018317	5.40830381	4.28728504
6.48604201	6.20413223	6.01403799	6.99004691	5.68706826	5.82460622	5.43273053	5.91027503	6.99869819	4.50729295
3.08065817	3.61001542	4.28301617	2.98847897	3.45705716	3.1673907	3.04562972	2.97968429	3.72938572	2.67695591
3.38931681	1.41245207	1.31625791	1.33066129	1.6175314	1.5298735	1.07447156	1.09839168	1.16699903	0.39537653
1.95444993	0.37390774	0.25871545	0.37444329	0.40974032	0.40165972	0.1006486	0.26283685	0.43167928	0.12257129
2.57727322	3.03740068	2.782447	2.37194645	3.46933823	3.0883186	2.20991958	2.55192363	3.11791837	2.15789856
1.8083129	1.41230757	1.09986976	1.32588966	1.86333346	1.87203137	0.80456671	0.84230358	1.62784302	0.91369873
2.96671116	3.15137356	2.68920786	2.56464775	4.31587543	3.76505585	3.29466147	2.68672474	4.07294067	2.35509827
3.87222554	2.75383375	2.63308453	2.90476019	4.12394839	3.91175345	3.38603657	3.19954012	4.11391864	2.59383841
3.24002601	2.94840334	2.31983482	2.81005913	3.90159392	3.46335537	1.94567882	2.66954256	3.54466336	1.8965394
0.06041859	0	0.01315506	0.0386302	0	0	0.0153605	0.04760955	0	0
3.87834137	0.55057936	1.18113203	0.50217556	0.09804245	2.62706686	2.56875836	0.01448784	1.09964543	0.9860761
3.77708269	2.06293321	0.94247798	1.83536045	0.25304794	2.61302902	2.13742834	0.17058774	2.24866713	0.75279907
1.77318999	0.32109672	1.60378811	1.03978708	2.1386185	1.41817351	0.35880323	2.78357381	1.40837225	0.13769295
1.53702994	1.99904858	2.30592291	1.67756393	1.74205975	2.05727019	1.32702636	1.96795116	1.71215164	1.96256296
1.69502214	3.20643211	3.37274527	1.31384272	3.71967983	2.96031266	2.96811462	2.43324635	2.69238913	1.73382492
3.738496	4.40278656	4.76152297	3.82777754	4.59906919	4.35620145	3.65529902	3.41983758	4.11290097	3.14439647
3.24514946	4.26791176	3.45534894	2.79709955	3.71795396	3.87387961	3.10538847	2.86836861	3.54248105	2.83803713
4.1998261	4.04548921	4.15714403	3.89603122	4.04721386	4.55597403	4.7340424	3.9819142	4.46329519	5.05000437
3.001823	4.0720321	3.81701833	3.38239553	3.59443376	3.52573802	3.32941023	3.28258593	4.01624865	2.7663901
3.20695357	4.47935322	3.98329747	2.96619429	3.68755526	3.47020957	4.05536652	3.39736977	3.34770541	3.41720718
4.14605511	5.40626624	5.07387005	3.9694585	5.49081277	5.61910292	5.09370903	4.1200592	5.02798382	4.30313881
1.05317253	1.6154628	2.71838997	1.49597912	1.8776079	1.92053355	1.12013868	1.51737543	1.46359022	1.24739286
2.11331304	3.03140238	1.0549084	2.13254111	2.10566	2.59414083	2.1049504	1.52458187	3.13052053	1.87234995
4.2537566	4.22267188	4.55385305	5.08804728	4.94010536	4.17330144	4.93893231	4.61462098	3.50293585	4.03010268
0.46413847	1.25534514	1.79222769	0.37569317	0.70488186	0.51072131	0.38965305	0.79535887	0.32872782	0.80884942
2.04613021	2.9601479	2.67727946	2.39230224	2.88118882	1.78557199	2.1969217	2.2819654	2.72166096	2.71837606
0.91513602	2.61476301	0.20942948	1.46060867	2.18549361	0.89167629	0.66612804	0.43020674	1.08400849	0.42230646
2.02665649	3.06641858	2.22874399	1.93656195	2.40862773	2.42673136	1.23942556	1.49306709	2.18722216	1.6775881
0.1072901	0.3041364	0.0326581	0.10229854	0.21076793	0.10478463	0.05056689	0.12544825	0.29838561	0.08470013
5.23914886	4.86715942	4.82032803	5.33539036	4.71303087	4.85335135	4.83157126	4.95922667	4.33207321	4.74875055
1.45835746	2.59687755	0.92856769	1.1568517	1.26059404	1.56387238	1.19149527	1.09775605	1.66194533	1.45864623
1.62625914	2.27679762	2.222609	2.41993064	2.39321484	1.5911773	1.96727519	2.25069509	2.2521351	2.03260766
1.82611761	3.09170593	3.14922211	1.5330953	1.96444711	2.19519158	2.16910326	1.49150637	2.07653659	1.27197461

2.93167053	3.56039913	2.00061331	2.40918708	2.59557989	3.06032075	3.665855	2.59764959	3.07111965	2.88633478
4.01770563	4.10202956	3.41021103	4.07981344	3.86252581	3.79856948	4.36475746	3.78238784	4.30403867	3.61160775
1.31519964	1.83551301	1.78057484	1.28766809	1.75719531	1.92948247	1.66748541	1.24245894	1.62805184	1.33561247
1.61321078	1.70270347	2.13946815	1.58515862	2.28851933	1.80907152	1.58857741	2.39728652	1.60701863	1.84476364
3.42543747	2.7431375	2.28162083	3.1338121	2.73218412	2.93877848	3.38940386	2.89400399	3.97459199	2.69323041
2.71380441	2.42721699	2.53772284	2.96658277	4.31462719	3.14472844	2.48480641	2.57254805	2.46298605	2.76564472
1.80375644	1.95968672	1.63382551	1.6290444	1.79815619	1.68220636	1.73233947	1.47063312	2.12799952	1.76640521
1.31794572	1.42515036	1.11476818	1.25175393	1.69293076	1.46724396	0.9640813	1.17648988	1.08730792	1.38670671
0.17952536	0.31279924	0.24079019	0.08250975	0.04743812	0.10099264	0.05630276	0.08728859	0.18808161	0.09651926
0.70936061	0.54560758	0.56610679	0.65905855	0.74633083	0.6999721	0.52725868	0.66282872	0.88127544	0.66303566
4.31478254	3.84003285	3.7256191	3.89558168	3.61077331	3.87986069	4.28131968	4.10367145	4.37167324	3.78771565
1.25163674	0.96552681	1.24981549	1.27668314	0.91954414	1.15090131	1.01487096	1.18160258	1.02856088	1.12104749
1.95072688	2.64241302	1.35929411	1.7287741	1.9837263	1.61922916	1.02378203	1.76181821	2.22505448	1.84485349
3.89121836	4.12010314	4.23949333	4.04393572	4.38307973	3.67816943	3.93336553	4.72001635	3.95938745	4.24826698
0.68999057	1.41049851	1.38928742	1.25479401	1.49323042	0.83471118	0.38623467	1.38256693	1.54795852	0.72047006
4.90766488	6.6586012	7.19772775	4.26970433	2.58438616	4.73320975	7.41498355	1.999003	7.54224002	1.58814273
0.25553364	0.59001293	0.51041896	0.65084117	0.83000606	0.80131816	0.56185348	0.83811573	0.54040181	0.56170215
3.445357	4.2301098	4.03360022	4.24157103	4.92981069	3.6834394	3.83511588	4.10210239	3.41269026	3.89973849
1.89493547	2.18478699	2.06223764	1.88552644	2.58777076	2.39993198	1.82352008	2.11767679	2.12876813	2.0970234
1.99205395	1.99623957	2.34706806	2.04461023	2.61954614	2.75965892	1.88915978	2.32838966	1.90634821	2.02711278
1.24408348	0.54083962	0.58842591	1.16086523	0.94276012	0.53993836	0.41550732	0.84671155	0.61956737	0.36067909
3.86164216	0.72964554	2.56991093	3.06560605	0.48777266	0.51665782	0.01092596	3.37523225	0.24927486	0.48998418
3.55957255	3.36995096	3.26685036	3.03542493	3.47434728	4.28847246	3.79894304	3.34760517	4.23182202	2.97062354
0.96173464	0.86974099	0.54141307	0.84467954	0.55787034	0.78655949	0.8199477	1.01536581	0.96135553	0.58996569
1.30013444	2.28018344	1.89211006	0.97171333	1.7173576	1.2988281	1.70903774	1.53260576	2.18040382	0.8882494
2.36650957	3.77201949	2.69088475	2.38979469	2.63006268	3.04379733	2.64724907	2.46992361	3.43859265	2.78536804
1.17961039	2.21385779	1.64916888	1.04397747	1.30679631	1.74442004	1.13687291	1.18809349	1.780159	1.34274039
1.58145236	2.41205845	2.26500914	1.86561861	2.15575699	2.09263002	1.21262912	1.9652049	2.2379498	2.03699725
2.21807947	1.17321288	0.78613767	1.067087	1.06751568	1.40807361	2.24662397	1.143134	1.35006885	2.29670542
2.20401446	2.73459702	1.76512179	1.89646279	2.39754081	2.65634061	2.63202962	2.13772727	3.24653544	2.36803765
1.43544553	1.84601673	1.72620888	1.42822318	2.12122649	2.01775869	1.23797961	1.6752035	1.98871288	1.28863464
1.16162139	1.35263259	0.96663478	0.98026597	1.72924236	1.09615215	1.11090999	1.05260207	1.38490237	1.27173411
2.39855707	2.14818185	3.34431308	2.59123521	2.51276024	2.05229208	2.39104509	2.82302993	2.27228118	2.08193923
0.37555313	0.17785658	0.37175265	0.30972103	0.26537107	0.47771872	0.04871927	0.24901909	0.57688158	0.02561565
4.75514666	4.11986999	4.01962189	4.30161734	4.23517553	3.79586419	5.22218234	4.20296953	4.69422289	4.82521128
3.11455985	2.9540167	3.30598959	2.89575409	2.49252745	2.08986426	3.177737	3.11375147	3.11876782	2.69618687
5.29251096	1.31713334	6.68645563	6.19479934	5.96332656	6.19345813	8.25353286	5.2110738	4.9216484	5.98528764
0.77005079	1.0795198	1.21605487	1.20860335	1.3314732	1.40960546	0.57234158	1.72999048	1.36765405	1.06608317
2.30477156	2.66346945	2.63919831	2.39441553	2.6147338	2.83284518	2.4753244	2.4234596	2.80689799	2.17643271
4.58559056	5.2910366	4.11963714	3.51253097	4.77338839	4.93396267	5.71591784	4.17315398	5.00465282	4.34133392
6.00956134	5.99850159	5.75176325	6.0377244	5.64596111	5.37146958	6.7244765	6.13386086	6.62768647	6.05305944
2.56644785	3.80194467	1.98271673	2.39054842	3.16195545	3.6511063	2.536093	2.72967571	3.59033935	2.58786704
3.26385718	2.85859289	3.26150926	3.13385659	2.76384534	4.25484092	3.16697183	2.96067618	3.14193959	2.66171075
1.68804032	1.69647181	0.5557401	1.17526793	0.98236381	2.08147551	1.93738455	1.08773496	1.94929045	1.25578593
2.59678134	2.95895305	2.75548867	2.22209374	2.57108181	2.96563169	2.62299779	2.29660253	2.69902767	2.65756242
2.25073525	1.59539195	3.64526685	2.24687277	3.30396525	3.5328457	2.90742954	2.20166374	2.01682434	2.94473147
1.71977961	2.28976123	2.17469561	1.83215221	2.25802093	2.23745429	1.68636591	1.94455862	2.14479053	1.90133155
0.70292623	1.54387795	1.69901976	0.60162992	1.97563494	1.693769	0.77263013	0.85841006	1.27637748	0.677029
3.93887118	0.71796645	2.01774846	2.79885716	1.88421942	3.00750616	2.68852138	3.03834411	0.72265298	3.40800717
3.14208797	2.47199732	3.66587082	3.83478483	4.70898752	4.35788405	3.13180735	3.52754579	3.47671467	4.16984769
3.79292749	3.76843676	3.93402716	4.14464055	3.77253177	4.1486259	4.22276156	3.94738949	3.84680911	4.18465886
1.93446099	1.90976236	1.06560757	1.47755255	2.88715846	3.05292657	1.63710585	1.69525969	2.48785104	1.80035937
1.50784882	2.1197951	1.91188234	1.55934348	2.26292867	1.79191225	1.55761741	1.51308026	2.57803839	1.65150017
4.48910534	3.00121894	2.79622865	3.47482678	3.18606989	3.14160557	3.65574253	2.83748066	3.20525438	1.65786946
3.20405673	4.28293055	4.17250262	4.7539688	4.21503022	5.20157471	4.01456477	4.50985561	3.81555897	3.81088174
2.27896293	1.62306386	2.5706438	2.56922588	1.92853849	2.39903682	1.5039437	2.31218741	1.96658319	2.97903943
3.83593731	3.57521797	3.58589351	2.75316234	3.06593906	2.93629374	4.59737413	2.28278161	3.46585127	3.82799081
1.82034327	2.03640973	2.69854087	2.46542372	2.27628094	1.95562218	1.81559067	2.58853091	1.48619102	2.20583059
2.16137701	2.49048877	1.87653572	2.00530142	2.76285694	3.02330273	2.84375559	2.17126352	2.82883181	2.19665978

6.00152095	6.51919407	5.9653282	6.11555164	6.60323652	5.92910188	6.11795698	5.93775699	5.75015719	5.97343585
2.33809401	2.28256461	1.90815344	2.33090002	2.09100631	1.82259929	2.48306995	2.1465934	2.3832118	2.10748871
2.05360823	2.03174861	1.86948287	1.79231951	2.11067584	2.05809068	1.77649682	1.63348073	2.45669282	1.95265857
0	0.01863238	0.54138424	0.01624434	0.007711	0.38614599	0.01100374	0.0454401	0.02092828	0.00957012
0.57674073	1.28407996	0.41469562	0.6584259	0.86838203	1.44377785	0.69952917	0.78705084	0.79066814	0.83217784
7.28115104	7.52857266	6.69939372	6.95046377	7.83479707	7.36563706	8.1374064	6.47373527	7.76706043	4.92752467
7.2670814	8.13044082	7.4484095	7.03388656	8.60839306	7.84381369	8.42328272	6.25728138	7.92130446	5.31738658
5.85067692	6.55792855	4.20370294	4.51610749	5.1997502	5.66689419	6.51206524	5.17431791	6.45034865	5.27726664
7.40064205	7.94072957	7.68959382	7.25568977	8.59032139	7.72713011	8.37429812	6.56008867	7.61409675	5.39725453
0.86725533	0.22008793	2.43645837	1.91994991	1.25465657	1.92511175	0.58254711	2.11999053	0.47889613	0.99079119
4.46487431	2.11639524	3.8601495	5.26523505	4.30444807	3.60831697	4.3775292	5.62319046	1.69443604	3.87906239
1.76281819	0.84658828	1.53367131	2.38160037	0.76973498	0.97460204	1.13146481	1.86570017	1.05784183	1.02567717
0	0	0	0	0	0	0	0	0	0
1.087506	1.08943871	2.58768487	1.93752814	2.88945088	2.56748936	3.12019034	3.65194087	1.64743843	2.57393962
0.92572815	0.56115466	1.28849434	2.19938618	0.51627671	0.49689704	0.12068359	1.05399789	0.40303665	0.27665966
6.30351332	5.16228805	6.26775983	7.18776086	5.9728972	5.29088938	5.65965312	6.78317669	5.00304008	5.03053165
3.25837103	3.43907301	3.36993442	3.88144733	3.44872407	3.00363014	2.74158515	3.74062929	3.02314805	3.4439384
6.02599156	5.14356949	6.48837308	7.04566037	5.89533582	5.76663113	5.20834713	6.85901721	4.69476586	5.06230474
2.60425441	2.59469855	2.31366248	3.47576028	3.04525347	3.92199613	3.12346648	2.24251688	2.99881674	3.18459997
0.12996875	0.23240625	0.22737547	0.22810683	0.4909409	0.66168834	0.15810922	0.45746565	0.43949945	0.22380061
1.32026986	1.69230875	1.92393758	2.2472766	2.73964129	1.4273008	0.42854518	2.2998881	1.32353231	1.02733051
4.8314025	5.55877193	4.5034928	5.23540012	5.30087958	5.58605357	4.29582555	4.85298479	5.80282543	4.98944578
1.16623828	1.8051303	1.74105317	0.48260396	1.18044427	1.44297922	0.68087505	0.8078558	1.45896902	0.72578579
1.29476824	1.5716691	1.5752392	1.30427369	1.61637905	1.5353049	0.72705018	1.48190165	1.49471927	0.701045
0.13793063	0.08072622	0.10749476	0.10620445	0.15560646	0.15353734	0.10477683	0.16099206	0.3484016	0.05559661
0.20758312	0.25974742	0.31012125	0.4659559	0.22925565	0.54112467	0.21913757	0.58828111	0.28895338	0.58407959
2.3545796	2.28257273	1.60287726	2.36577502	1.61115718	2.03541666	1.67186192	2.35957387	2.26333076	1.99596172
1.33136347	1.92870447	1.62863607	1.79139125	1.97301387	2.04460107	1.10456282	2.15494226	1.87241081	1.73656409
2.13310926	2.15840121	3.17778012	2.46592669	2.29423675	2.17171304	1.68354171	3.10641108	1.96110698	1.53025605
1.44789612	1.89626576	2.05012903	1.50335961	2.13981317	1.93534151	1.65692431	1.47489988	1.35355745	1.68211581
1.53415306	2.44349259	2.02008994	2.12565131	2.28485729	1.95910982	1.18267575	1.82088128	1.58223368	1.45865803
1.2613629	2.53924191	2.66087353	1.73810813	2.67333821	3.14069102	3.08102729	2.41273105	1.85260483	0.74470803
3.60247434	0.87016475	4.0606322	3.62244134	4.26720897	3.19678259	1.87068117	1.92285316	0.68504768	0.64206696
2.55728086	2.12982847	2.60576573	2.15795924	2.8046929	2.68678637	1.95102727	1.96085982	2.56572078	3.69606291
1.23776727	1.37922024	4.15185401	4.62280279	1.08541865	1.97895141	0.23941072	2.84709561	0.58450864	2.26694312
1.97618906	1.6450583	2.37048996	1.79466336	2.09626381	2.30595152	1.48135575	1.98472928	1.54839145	1.89290367
1.78809695	2.05992984	2.15445416	1.75579535	2.92953656	2.71830245	1.96839003	2.17711631	2.32922635	2.09400614
0.02040816	0.05147088	0.16809906	0.04809605	0.08188592	0.10354972	0.18104979	0.2235964	0.04351497	0.07068
2.52471029	2.63389688	2.74482548	1.97638156	2.04530014	2.39414854	2.79495341	2.15781129	2.93067047	1.97323315
1.82991266	1.70762606	1.51548743	1.74623144	2.22753118	1.21507676	1.60902692	1.78656082	2.06918418	2.05499741
0.7308007	0.38865438	1.01772707	0.84306118	0.52624115	0.81374439	2.61720935	1.12337019	0.47513775	0.23592446
7.55439208	6.13565616	6.28332164	7.97068674	6.8275146	6.22826045	4.97028287	8.04981987	5.68508947	4.2507127
3.07129437	3.43717273	3.84012412	2.94401754	4.27095442	3.48726431	3.24423675	2.49525065	2.9178169	1.68529955
0.50634649	0.32530197	2.44544181	0.87683661	0.4999156	1.81287097	0.43419625	0.81687368	0.46371912	0.46582479
0.98505325	0.63255723	1.3751422	1.89645398	1.16572561	0.90322441	0.77498147	1.60745903	0.53845771	0.84182253
0.96441343	1.42635783	1.26236861	1.15841901	1.38640079	1.0175636	0.77136447	0.79789431	1.31591905	1.21004827
0.22689321	0.10274595	0.0917462	0.14240924	0.26018788	0.13969002	0.14051818	0.19445956	0.22151123	0.16012704
2.6136887	2.64395	2.48167075	2.32124184	2.52671037	2.58217152	2.50144519	2.35040748	2.68336576	2.2696677
0.96008485	1.36341095	1.94309265	0.64071881	1.11516012	0.94943348	1.07308869	0.93515011	0.67598428	1.09438559
1.53474964	0.62949843	1.74488836	2.64675068	0.94055707	1.35392676	0.29436082	2.03147065	0.84629265	1.51234576
1.49961174	1.49268943	1.9186372	3.27106949	2.43086805	1.63730146	0.79609618	3.60863144	1.24612951	1.5388325
0.49812136	0.55805348	1.92393297	0.52763358	1.19674764	0.80342069	0.75857315	0.99511929	0.15159452	1.20289873
2.32750727	3.50809294	2.2965282	1.9891135	2.38844504	1.97510661	2.35888526	1.59789442	2.71711303	1.70507521
4.86793919	4.66399859	4.53123444	4.84659233	4.28215655	4.39208079	5.11823329	4.71049474	4.64092265	4.52644264
1.01456456	1.57504068	1.20621929	0.6824136	1.43743057	1.22246368	1.03443303	0.54237677	1.25145576	0.90963006
1.77797154	2.30476432	1.97355217	1.49349768	2.09886296	2.06559047	2.05642275	1.55633796	2.55784475	1.38071465
0.32546718	3.09393322	1.48171117	0.57912667	0.84478292	0.56837656	0.95996226	1.55646089	0.39192767	0.42624632
3.74454659	3.3579296	3.87661732	3.08275371	4.04260667	3.43444125	3.33171976	2.61051277	2.72677589	1.98569756
4.34794632	5.3230382	5.05848581	4.64804444	5.19403505	4.85584568	4.94630319	4.48694347	5.29453368	4.04006681

2.62761591	3.26951162	3.50110987	3.0561564	3.05459162	3.05325518	2.81683572	3.1109851	2.99899946	2.87938614
1.96038896	2.79424836	2.72831046	1.73224287	2.37296231	2.98463922	2.75428975	1.61852385	3.59828947	0.64621653
2.6204351	2.65866433	3.15946656	2.68568436	5.09092412	2.76762416	2.73671739	2.94655566	2.7100705	2.89462244
1.15860843	0.89486779	0.85193513	0.97325303	0.87638413	1.05340109	0.80292083	0.98002497	1.63153954	0.78998535
0.90981586	0.20413305	0.11786209	0.50194918	0.57278431	0.03753984	0.03777038	2.34757482	1.78086548	0.0049814
4.82818871	5.15654145	4.68825808	4.78629284	4.79958155	5.50272658	4.66737468	4.88294898	4.78221134	4.54881328
0	0.24757784	0	0.02499072	0.01660711	0.05810232	0	0.06102164	0	1.75992351
2.46538358	2.4406385	2.83638869	1.78487951	2.37871135	2.5345084	2.58894026	2.02153625	2.90605063	2.47840634
2.82090797	2.77054862	2.56579878	3.54167874	2.40704751	3.38918975	2.92138452	2.86600665	2.6732052	2.30723917
3.6182326	3.66251927	3.89493716	3.79464226	3.42817814	4.06978226	3.62070022	3.38441107	3.29230305	3.21696648
5.5005751	5.4575328	5.19846779	5.42090881	4.44370809	4.23608545	4.8934669	4.99127023	4.97063485	5.1707854
0.03893729	0.19938611	3.64743352	1.0296553	0.04894206	0.01465053	0.10015888	0.19517281	0.18549132	0.17448201
3.51939362	3.77525255	3.52920833	4.06359923	4.03288969	4.03948335	3.09710555	4.19394831	3.37242493	3.73143317
4.52969288	2.97775172	3.92049806	6.30091271	5.46380176	2.52754717	0.04697093	6.2045253	3.13233411	1.63641854
0.19125095	0.16761979	0.30566141	0.19036802	0.21223342	0.35486042	0.18941149	0.22256293	0.12740931	0.31293904
4.1470029	4.19334353	4.31782031	3.95296946	4.64955686	4.4996034	3.86841495	4.01084187	3.95724156	4.41297429
2.89450819	3.02478986	4.28536189	2.40656316	3.28845526	3.36963045	3.30910588	2.97352721	3.28441232	3.66194407
3.57091341	4.01739686	3.40904253	3.66770022	3.97578109	4.24403362	3.50434925	3.94072083	4.09878404	4.23332955
4.24962089	4.91897409	3.90116886	4.1179954	4.91790165	5.16937777	4.02592564	3.71489976	4.3515972	4.58088573
0.75280536	0.83841529	0.65969761	0.30865851	0.82743385	0.94308679	0.35870811	0.55766345	1.38083888	0.56969885
0.41479484	0.13952395	0.44671123	0.81408052	0.38008703	0.2300968	0.27096249	0.7203793	0.32082303	0.3214155
1.12422136	0.97166783	1.31722324	1.31225536	1.46193063	1.05527902	1.53351168	1.52151553	1.70897511	1.40626157
2.09861511	1.9647741	1.85018375	1.34607637	1.40771362	1.99558921	1.58802549	2.10907131	2.08598849	2.10951614
0.31846562	0.97531194	0.60351363	0.8577133	0.68611623	1.53275988	1.08486938	1.32545674	2.73271519	0.96325945
4.36903956	4.25895243	0.87579565	2.69637606	1.58374742	4.12281492	4.1153613	2.71854786	4.1686326	4.03034189
6.16322551	5.26243882	5.99496436	5.68934019	5.30144991	6.01203685	6.42238961	5.49730864	6.16672035	6.60241151
1.73724784	2.13298811	2.65166427	2.3229471	1.64017116	1.90148352	1.32409702	2.15908467	1.71775001	2.05927985
2.1045463	1.87625698	2.35782931	1.43023305	1.57507032	2.83737494	1.86939504	1.836589	1.75879947	2.3721349
0.25328209	0.17395424	0.30437357	0.1087396	0.14240576	0.52932761	0.32640513	0.20232935	0.49155914	0.33076449
0.05485604	0.08084468	0.09275546	0.28844892	0.10208742	0.6584447	0.06832443	0.14588669	0.05244838	0.16532279
1.87828735	4.05370398	3.0907332	2.56372651	3.38146659	2.88258158	2.34059306	2.95081977	3.82647442	2.62981671
1.28256505	2.01626952	1.67629654	0.98252815	1.36288093	1.9551244	0.74325734	1.19395377	1.87632953	1.21046068
3.50005787	3.6471549	3.41413738	3.16122292	3.39333576	3.52146842	3.34026347	3.30052797	4.27336639	3.58578213
1.43304406	1.34308598	0.87407559	1.08238671	1.14073661	1.2036316	1.37610856	1.30494981	2.23161759	0.92336018
0.83347165	0.00943023	0.61870901	0.21000391	0.04152385	0.12714638	2.18212818	0.03451827	0.34796102	0.25020755
2.32309461	1.51814617	1.76459274	2.39041147	2.77917826	1.9708732	2.12006969	2.33543762	2.14103629	1.49003932
1.94174962	0.64992463	4.4858841	1.25155723	0.44844761	2.34989018	3.17639101	2.52478809	0.27886489	0.87659643
3.58273668	4.25132971	3.10340149	5.62439554	3.65330708	1.35995705	2.84997437	2.21124393	7.65807456	1.71605191
0.7557749	0.63093498	0.32228857	1.1590509	0.77754192	0.70970265	0.00978049	2.59770115	1.66948586	1.11823102
0.59396054	0.15063335	5.87485503	0.22967272	0.0233758	1.24623852	0.04601908	1.85643945	0.01773903	2.30518091
0.88552298	0.74665905	1.41759976	0.89739976	0.82241753	0.74057314	0.3207508	1.15228056	0.45373535	0.71595424
1.50323088	1.36865895	0.32590905	1.33876426	0.12301696	0.29175802	0.09848231	0.24258651	2.59714355	0.23355961
4.73279738	0.44300254	1.80244143	4.05635052	2.62867641	3.81962031	3.88417936	0.2168707	0.14762531	4.22319643
2.83132824	3.57454009	2.62025585	2.21665413	2.76323068	3.35807244	2.75498755	2.23306477	3.66348133	2.55270991
3.71105841	4.46277394	4.86956105	3.7451013	4.3787032	4.26744528	4.21756545	3.81515456	3.93023919	3.85659895
1.42704338	1.94227172	2.04593176	2.64073614	1.43607996	1.97767475	0.59911084	2.1226785	1.13626944	5.13952993
3.71813236	5.05738936	3.59720183	4.27040446	4.11854755	4.2096554	3.31295184	4.38647894	5.35057069	3.29633967
2.2409318	2.47574879	1.66835573	2.8040943	2.32273835	3.21191195	1.2229579	2.30496636	3.11465536	3.19125626
2.08033027	3.59158698	4.12007473	1.99800986	3.6146258	2.66421497	1.75651503	2.07077025	2.18320817	2.98712916
0.20069064	0.09348037	0.07001119	0.22448791	1.59215506	0.11436008	0.05290779	0.20113927	0.09934776	0.04307307
0.08979077	0.10255441	0.04107868	0.11845225	0.08508223	0.1193578	0.08114678	0.18398292	0.1147893	0.07768736
3.59198021	3.63387626	4.00429161	3.21572701	3.90914599	3.28651593	3.38917596	2.86305414	3.71125277	3.00817494
1.50780462	5.77812559	1.4519173	1.98565999	1.95023248	1.20132609	0.39181138	2.50312541	3.36809205	2.00345488
0.85193552	0.12807511	0.23551049	0.62116358	0.34704005	0.12066209	0.16091323	0.51903053	0.10319335	0.10641897
0.00608851	0.00775679	1.28540237	0.12241752	0.01721261	0.00454563	0	0.07689009	0.05573892	0.01426548
0.7125473	0.62335526	1.43707519	2.48341825	0.87494249	1.20406297	0.53599535	2.37672616	0.5397174	2.075275
0.78957083	0.38587837	0.44987541	0.93095001	1.00940633	0.53591685	0.05427368	0.35570342	0.05574563	1.38934394
0.35114103	0.07284838	0.04830697	0.1706173	0.0432756	0.11333209	0.02529016	0.37776245	0.05493592	0.10889339
1.53820739	2.36212643	3.89665733	4.25477453	1.7703364	3.24320187	1.95400935	4.08051694	1.80843082	3.43929395

1.42936515	0.71460655	1.58203517	1.92976733	2.76759324	1.21994328	0.61862168	2.05265344	0.64998972	1.23369794
0.34647189	0.13917585	1.04510004	0.74624645	0.37921228	0.41518506	0.12363779	0.68497578	0.20388344	0.29649961
0.54309839	0.10717873	0.25797893	0.37693211	0.10229845	0.11137176	0.3591161	0.3555645	0.08105936	0.16264446
0.611707	0.92078414	1.15103564	0.9710808	0.65013992	1.01064736	0.36011019	1.0943455	1.33931162	0.54577703
0.59055947	0.29933682	0.87752767	1.43800815	0.35003482	0.70335029	0.1760718	1.12126576	0.2542043	0.56699337
2.01326066	2.23886747	3.07478866	2.11362965	2.13044361	2.65051676	1.97631671	2.29905716	1.71953163	2.35166334
0	0.12905989	0	0.04579159	0.41659317	0.02411413	0.01218246	3.34059758	0.06839833	0.02530047
2.35738523	2.7640861	2.68813004	2.42871555	2.46099398	2.17748072	2.17857863	2.83174899	2.75033412	2.35610252
2.04351618	2.58332797	2.55682369	2.33289319	2.4403914	2.36989534	2.19954405	2.03305075	2.51686923	1.91278506
3.73825026	4.73439245	4.18870048	3.30128999	4.02829345	4.70740767	4.450802	3.19558275	5.156333	3.27349091
3.3086542	4.20657435	3.57969117	3.48402371	4.1061469	4.25472982	3.47313155	4.02590589	3.34913616	3.22367165
0.79347747	0.62745653	2.6952578	1.80384905	2.21138323	0.74757471	0.80165257	0.96513218	0.71102052	2.06074868
0.07364508	0.0267195	0.10122858	0.39072649	0.02081572	0.11899146	0	0.26264202	0	0.05572108
2.05291689	4.16827647	1.85453835	3.54024092	3.19016636	2.93882258	2.13295605	2.55831591	4.51375022	1.60915714
1.52685529	3.18432375	2.01904567	2.74459297	2.90859736	2.84232637	1.63022771	2.60581809	2.27890273	2.28584904
0.96069166	1.02979358	0.86488275	0.68546968	1.59218052	2.30539792	1.13110965	2.89432718	0.0499359	1.1395472
2.82036502	2.63895731	2.99427695	3.91209005	3.18874845	3.36111386	2.15402167	3.46696462	2.65027918	3.10444331
3.80676323	3.39960145	3.87174947	3.65953333	3.24924189	4.03931205	4.05722746	3.42063364	5.02014812	3.17557747
2.52995806	2.03571021	3.38641365	3.24607122	2.68785485	2.38944884	0.97017007	4.20474366	1.91174941	1.65670565
4.53733674	4.82389191	6.44066191	7.06668044	5.2568475	6.03612396	4.94688151	6.55280518	4.46349243	5.82090779
0.03902537	0.00628955	0.01265312	0.04631313	0.04301128	0.00736066	0.01477485	0.04581367	0.01410211	0.00772498
3.48125928	2.65149683	3.33053837	3.95930592	3.46121133	2.82675476	2.52417715	4.33222932	2.3588879	2.10836076
6.06631759	5.60783861	6.16223676	5.97661863	5.22632415	6.04953599	6.27025126	5.87104761	6.03326246	5.24133239
5.7675309	6.31562171	5.82974864	5.38999326	6.12375133	6.03970866	6.25647614	5.32453862	6.45231768	5.84338975
6.81516644	7.41533866	7.2034034	6.80593966	7.08262458	7.60915833	7.45812172	6.99104419	7.28342167	7.00080677
1.91801892	0.81980828	1.85535355	1.07794406	1.09911561	2.41337047	2.85433975	0.99796387	1.31506534	2.48219739
0.15991086	0.02466167	0.01662077	0	0.04044769	0	0.01940334	0.22621774	0.09031613	0.03024637
8.04052674	8.75086664	8.06511807	8.40594624	8.29899964	8.40396331	8.86335937	8.03437263	8.41754788	7.97764242
4.31925077	4.90379395	6.0233875	5.67051088	5.49101484	5.52745694	5.51451501	5.92159612	4.46718098	5.86011623
0.19661823	0.05642214	0.14694023	0.8055435	0.05583997	0.0442245	0.02241927	0.33549366	0.04248925	0
1.80441997	2.77826657	1.65333047	2.03225386	1.61460372	1.87340014	1.342942	2.2308064	1.30238914	0.93808535
2.74084204	1.35809132	3.09033602	2.43814831	2.63679186	2.53063763	1.04757308	2.75978618	2.3123184	1.11217964
2.84762165	2.87517989	3.51129011	3.71714577	2.96199395	3.28338178	2.2799202	2.94852571	2.58558174	3.43501945
4.3224482	2.48305882	5.68410983	5.3445835	5.4537123	5.61913839	6.55975427	5.60218799	3.08358102	6.96994105
1.13992446	0.64705445	1.25765657	0.99087011	0.87763013	1.88293733	0.64822723	1.29838845	0.53041048	0.71782487
1.67678432	1.18059859	1.25386611	1.34610162	1.83976727	2.23674163	1.33779082	1.2818782	2.06038133	2.05885177
0.03063914	0.00492592	1.24417893	0.02917741	0.01939873	0.35647278	0.00580106	0	0.01105112	0.16050854
1.02812653	0.07548276	0.02580797	0.03806728	0.02533529	0.05924846	0.2249476	0.0469179	0.05693525	0.06212649
1.70567962	2.72646421	2.65208629	2.30964197	2.40661396	2.99321178	1.68873338	2.84586641	2.53671649	1.93729831
1.52117538	1.80411171	3.18103753	2.21321477	2.879869	2.49376774	1.6215591	2.7897613	2.16788125	1.92721027
3.52797634	4.04512224	4.83691183	3.45872823	4.16413735	3.87569531	3.99706527	3.21253827	4.29778005	3.4492622
2.13477639	1.75580431	2.14067013	1.9467256	1.82706775	1.81428143	2.08153869	1.60975354	2.55901171	1.88774678
3.38454209	3.36937138	3.73449842	3.72384442	3.44720481	3.43480895	3.21489821	4.0516254	3.52329477	3.74409052
2.56739373	3.27052837	3.27203123	2.48866998	3.53249302	3.37907568	2.75371594	2.6266649	2.99470527	2.39313396
0.93876511	0.59583562	1.41109958	2.48351534	0.80679071	0.80948936	0.27770641	1.71137571	0.29857149	0.83265917
4.95519597	4.73861455	5.15053427	5.43204686	5.38167533	5.13212264	5.79352226	4.54489238	4.59241092	5.72375087
6.00480375	7.04037977	6.27789433	6.31181478	6.4227802	6.57930519	6.28818852	5.88826883	6.27324509	6.10068808
6.44621378	6.36910851	7.0597733	6.316713	7.89359814	6.38312506	6.46638236	6.47584429	6.16648585	6.33618908
1.01327591	1.69147561	2.37443381	3.13195236	2.20615951	2.71660684	0.96384107	2.45125408	0.73087096	2.07342704
6.17546505	5.69622666	7.54728436	5.07543025	5.5961015	7.32410456	6.4712323	5.17891897	5.92585185	4.46847165
5.44069344	5.99237172	6.08250746	5.09194474	5.84383792	5.67959432	5.51451464	5.35581559	5.11361263	6.13958904
1.45062339	2.03123152	1.91484203	1.55854422	2.12918131	1.84013025	1.6470132	1.74101641	2.03619028	1.85279451
0.35957634	0.13944219	0.1651037	0.30536631	0.21401897	0.13330589	0.10469709	0.53016499	0.10574466	0.11521197
0.28390733	0.39288236	0.74905386	0.54671599	0.89575488	0.7645874	0.18851863	0.42503696	0.16432198	0.90164451
1.35171817	0.16118119	0.54705459	0.97035529	0.46079767	0.23571692	0.84244603	1.68503245	0.11989593	0.31398643
4.00408296	4.13418405	4.11242202	3.722957	4.62259713	5.89271453	4.17414535	4.08969404	4.27807623	3.86067081
3.72524174	5.33142218	4.48100051	4.29766779	4.36560093	4.6141276	4.61273475	5.17479695	4.63215619	4.36299216
0.32296912	0.10715662	0.08859517	0.10097247	0.16179332	0.15532191	0.01928368	0.10273971	0.19060875	0.07881006
4.25247143	5.47655906	4.34575688	5.39957235	2.7451371	3.45429126	4.70748136	3.10774993	4.89050289	4.47025421

2.13079071	2.35744311	2.62117898	2.16350549	2.85381923	2.55077976	1.86002222	2.10854347	2.19825573	2.21581421
0.33144152	0.03663826	0.30865322	0.51620792	0.06462167	0.71227157	1.21855004	0.11832706	0.09679081	0.09698365
6.81993288	6.95052236	6.80916798	6.69756153	6.88314893	7.48107943	7.5421071	6.76641312	6.86916949	6.86748165
4.57901399	6.2897741	5.53225718	4.56597369	6.21825654	5.65185109	4.65970848	4.79040794	5.36797349	4.64641579
0.90905599	0.87786444	1.39044169	1.09876261	2.0613681	1.40630538	0.5128983	1.27868211	0.74757419	0.92186852
1.15322484	1.23792383	1.3954847	2.84146024	0.84226873	1.12528556	0.37638675	1.18497116	1.08234369	1.9113063
0.1900357	0.12133721	0.18229982	0.2114239	0.60119397	0.39268988	0.42290677	0.25271202	0.0878246	0.47215136
3.32616968	3.97665801	4.11170275	3.18251378	3.33409358	3.71252131	3.7912839	3.40548391	3.67242082	3.85993493
0	0.11888978	0	0	0	0	0	0	0.82577227	0
6.84628133	6.65886337	6.46280895	6.75838322	6.50360852	6.46391006	6.96386982	6.79586533	6.77000866	6.4975697
3.40212286	3.48907472	3.35690672	3.11584766	2.86056759	3.89995801	3.74140911	3.27556312	3.66005385	4.40336324
3.62966554	1.75599386	4.79818567	1.82855603	2.36691611	2.6939518	1.09650739	3.77145216	1.99107044	3.21697827
2.59296222	1.83646893	1.52144402	2.28438927	3.99038842	1.88811694	0.98718441	2.58745527	2.21000129	2.39478238
0.54829638	0.81772099	0.65083613	0.52017478	0.94265845	0.68146148	0.5223457	0.79206452	0.6244956	0.23409842
0.37636872	0.63358	0.80392567	0.51735153	0.61959122	0.57009149	0.20519254	0.49925353	0.48116496	0.63795281
3.60585903	3.53906173	4.28991366	3.58463443	3.10221079	4.45157295	3.90475598	3.50971702	4.13570378	3.27268445
4.2115462	4.78878024	5.31136072	4.51812958	4.42078624	4.96145614	4.89497683	4.83097466	4.59030916	4.54204551
2.05764615	2.71699715	3.36088534	2.41155211	2.97434387	2.81555204	2.36771564	2.58799632	2.20735258	2.64845853
2.8238119	2.80523867	1.82019045	2.1309972	2.58327642	2.58601299	3.01275503	2.29103823	2.60619142	3.38684536
0	0.44571862	1.12647648	3.56412068	0.62546116	0.28489249	0	1.80363814	0	0.23295027
0.56463535	0.39598133	0.54587783	0.6373782	0.76968321	0.27444841	0.2053411	1.04442543	0.23090926	0.39010643
3.79917757	4.3708976	3.96599806	4.21137724	3.96051763	6.36453632	3.17222199	4.19895541	3.83627681	3.6576794
3.14804643	3.80527853	4.32886408	3.71982125	4.43000325	3.35104676	3.42165811	3.83886937	3.50300326	3.35458568
1.84186273	2.14697546	2.8074087	1.66925952	2.96224153	1.5877161	1.28150985	1.90039503	1.74649276	2.01592834
2.94157038	3.4598516	3.44495547	3.14309758	3.73292115	4.01787681	2.39977384	3.06238831	3.35649266	3.58890859
2.29609017	1.98482499	2.71541481	2.07793408	2.69844148	2.56820103	2.89290709	2.43941001	2.38494365	2.09440209
2.46865027	3.52624516	3.99799928	2.09877982	2.53044114	3.33420669	2.95705927	2.91052899	3.1570059	3.32970357
3.93008702	4.48365848	3.03543969	3.30113059	4.01970192	4.3560439	3.70530226	2.99380732	4.1687855	3.4037763
3.46051487	4.45200389	2.22894833	2.84829203	3.47259514	2.43902273	3.01074073	3.11325568	3.87701476	3.49982361
1.02751475	1.15269508	0.88335621	0.1044347	0.38598361	1.20453203	2.32403834	0.76221997	0.22593545	0.61860878
3.89019268	5.47311749	4.05116746	3.07170468	3.34403909	4.07057578	2.98087847	3.0845503	4.62288482	2.97408505
2.53299998	2.90636026	2.89393609	1.87701866	2.87570311	2.6318573	3.14092877	2.5243752	2.80723939	1.9145554
1.83263705	2.27134855	2.03680335	1.8224065	1.882763	1.62736364	1.72607024	1.64027187	1.39697184	2.00632137
1.43727824	1.51816371	2.63477408	3.70353084	0.80205318	1.41037628	0.0403648	2.63497842	0.34568686	0.8755122
3.65579184	3.52413575	4.58000122	3.61049393	4.11437271	4.43909455	3.88848424	3.83418675	3.09392932	4.39271941
1.66580973	2.56839102	1.79284263	2.35234268	2.5789566	2.07704055	1.63807236	1.76667292	2.26508117	1.9764657
5.72649917	6.45975112	5.23760919	5.0732767	5.69287049	5.0324137	3.94607334	5.16138319	5.1597359	4.44715316
0.19910153	0.10361629	0.14416895	0.2362389	0.24690202	0.22470926	0.32361406	0.32954921	0.15264738	0.30790473
0.14521733	0.26602201	0.23709018	0.30962486	0.32244394	0.31838798	0.12360032	0.32103186	0.44402891	0.24685568
0.20447972	0.17656923	0.15645385	0.22174367	0.35470865	0.22533561	0.04748603	0.29543334	0.48454304	0.12455829
4.48870025	3.55388802	4.36761181	5.39131693	3.52339964	4.39549848	3.26990834	5.8643975	3.55886064	4.20565696
2.32560221	1.94354584	2.6285114	3.0399433	2.25955566	2.04204622	1.70774152	3.7317007	2.09392441	2.25257021
2.62307489	2.70281915	2.4232676	2.62796286	3.03392198	2.84844783	1.66448509	2.24232542	2.31146001	2.65143699
2.79125996	0.69682358	0.76002198	1.54657426	0.81760306	0.71656842	0.37260954	1.90707232	0.62980232	0.36227098
1.99901431	2.51779664	2.80090228	2.33774263	2.90594679	2.71609384	1.5494238	2.71015017	2.12038068	1.79394947
1.6607137	1.44913771	2.21906216	1.72164897	2.50552939	1.80873527	1.35205944	2.45203081	1.559619	1.11665292
5.0813596	4.9477836	5.60682906	4.39975979	4.78385343	5.79964238	5.88087444	4.48529818	5.22894532	5.40700552
2.18375803	2.66047378	3.55437861	2.18146384	2.7850304	3.1599147	2.48725308	2.62876942	2.53566618	3.00662123
4.7062535	2.872039	4.2724979	3.78630234	3.66747357	4.06500087	4.44664406	3.5687349	3.8384765	4.06254277
1.81731323	1.03545899	2.91335994	2.37148113	1.58962136	1.90246861	1.86711699	2.24145034	2.2925841	0.99677625
2.17569181	3.15743207	1.81813438	1.55260657	1.46681638	1.72298322	1.71983003	1.37046369	2.47355052	1.66375334
0.01768814	0.0084825	0.61266607	0.05810186	0.02227512	0.17781715	0.04602709	0.09619582	0.03153094	0.04460526
0.88807516	1.23490305	0.74800431	0.39801977	0.34390493	1.02647666	0.86265737	0.53617524	1.33316683	0.50931494
3.44507945	4.2033733	3.65685462	3.66073065	3.68868638	4.11305444	3.36454215	3.80604607	4.45834908	3.28469275
1.98770245	2.79273654	2.14375026	1.23818488	2.36771984	2.52231079	0.56365262	2.36291799	3.470203	1.21057628
4.59577817	4.35747	3.54170715	4.58728722	5.74937962	4.59867438	3.82096679	4.55074085	5.13604601	4.63003713
2.9821754	3.37496417	3.11608872	3.12959163	2.9914878	3.28661362	2.00322596	3.76150796	2.4683822	3.31166454
3.49822257	2.76814035	3.52247636	4.75829685	3.04212209	2.95676412	1.57262384	4.88652703	3.99274723	3.75233744
2.7615107	2.11400288	2.44575158	3.49005872	2.10228533	2.23089079	1.14331976	2.94740347	1.98780628	2.39983311

1.64893336	1.94883162	1.2045523	1.67398188	2.67618391	1.68714117	1.57769878	1.77352265	2.01150301	1.61850247
2.87698277	3.02419078	2.45227723	2.89346582	2.42731342	2.86889081	2.31669463	2.69016936	2.97895848	2.5210684
3.93344782	4.06976717	4.46531161	3.68560776	3.59387018	3.62412851	3.63199063	3.56913284	4.43240592	4.22362983
2.22312012	2.6803909	2.57506507	2.01639374	2.46248042	2.40271163	2.29079011	2.32723521	2.18683533	2.59946841
0.93795065	1.15534355	1.61121779	1.91571914	1.21468609	0.89921187	0.42966977	1.6216005	0.42752899	0.55392555
3.91150446	4.38459277	3.00468719	3.47919849	4.1396714	3.37384474	3.52442504	3.34033918	4.79744228	3.4524185
0.21300434	0.05736317	0.2109255	0.15715812	0.36397709	0.26807052	0.20826083	0.23386748	0.23612757	0.05294892
2.38484349	1.96852596	2.32034699	2.93838184	3.07770772	2.52148585	2.03265284	3.34429174	2.35704117	1.74640571
1.17028329	1.21318981	0.91424344	0.68142375	0.79987953	0.48336809	0.35469185	1.19751875	0.88109763	0.22571995
0	0.00984766	0	0.00735927	0.00488044	0.01152229	0	0	0	0
3.13158365	3.82641926	2.91595885	2.82394571	1.8814826	1.65807671	1.62945623	2.72043502	3.88146897	1.7194933
2.93381547	2.75306407	2.97738626	3.43514022	2.55925948	2.43842727	3.15638835	2.7952262	2.82039496	2.85950745
2.03295524	2.62168112	2.15137204	1.8879692	1.73965103	2.14513224	2.24223121	1.55545541	2.02922844	0.97587363
0.04834315	0.04841844	0.05316039	0.03304229	0.26250472	0.08150399	0.0517916	0.1564848	0.010029	0.10090161
3.39551038	4.29985168	3.34014132	3.29370047	2.93049286	2.88190034	3.57416049	2.96911232	3.29535142	3.69861673
3.2419897	2.69469206	1.8168384	2.41480853	2.41065471	2.45086041	3.02961275	2.4733581	3.63139629	2.95630211
1.95189948	1.63665374	3.50872891	2.20149986	2.93806135	3.08991885	2.7724561	2.21117461	2.08107506	3.23062458
5.09250751	5.77730516	5.09303177	5.35989925	4.62932479	5.79360394	5.82882105	5.35583282	6.31443213	5.04967438
1.22832944	2.0853935	1.31918019	0.79977059	0.38350282	1.25341057	1.32628079	0.82371974	1.65072092	1.15809124
1.46605966	1.86452178	2.18922921	1.31437166	2.14783586	2.09963648	1.13719665	1.38754204	1.59001488	1.89154914
1.37411322	1.16277371	1.18250847	1.47200926	1.26057358	1.10391204	1.34229632	1.47746837	1.29813279	1.38791087
3.01122151	3.1376411	3.1333377	2.5992359	2.87457493	2.5117958	2.55694669	3.01202696	3.26118385	2.68979063
1.99968335	2.84102488	1.41842503	2.40959525	2.73862536	2.4236255	2.82021188	2.15431643	3.19323484	1.57898946
0.28668165	0.28417757	0.87193526	1.33307112	0.4120811	0.80070253	0.1228408	1.75696518	0.27308732	1.15473412
1.2032729	2.06219065	1.75563181	0.86084096	1.64645119	1.71983013	0.66664689	1.3234486	1.80706435	1.72756501
1.19574738	1.64619905	1.63895268	1.25524434	1.89510504	1.73600679	0.84202968	1.48300811	1.34666305	0.79051503
0.0173521	0.14568855	0.0074585	0.00368764	0.05872872	0.01296773	0.00726348	0.03383663	0.07315838	0.01210257
0.81227559	0.71409806	1.0938391	1.58817462	1.06945882	1.35567808	0.38984147	0.95554118	1.25463675	1.2256851
0.78864684	1.35975292	1.45371686	1.67143391	1.8801147	0.71137617	0.52893411	1.68635895	0.46665821	0.83066151
0.13390389	0.20527823	0.16455329	0.04096269	0.15639333	0.21971133	0.09950063	0.16617122	0.30856392	0.0990894
0.27843923	0.2098395	0.1715438	0.28476192	0.33134723	0.26497884	0.28115605	0.56728984	0.24819968	0.16587269
1.91277658	1.51486787	1.02079776	2.0434168	1.59612539	1.22952922	1.65524655	1.62787729	1.63025735	1.72905595
4.23917695	4.29888102	4.01075689	4.09507803	4.051952	4.51874388	4.68368424	4.15613088	5.21496916	4.86666124
0.33858077	0.27349614	0.18083454	0.25316725	0.26611595	0.36363792	0.08606759	0.36628033	0.49170875	0.18664632
0.16912156	0.34928547	0.23365836	0.18830617	0.15822641	0.16300587	0.08148369	0.22742783	0.45658237	0.08636887
2.57704178	2.58951334	2.17045992	2.2214019	2.24416004	2.17415714	2.09272487	2.30667909	2.63892268	2.6017966
0.24170445	0.05484819	0.0511062	0.19772897	0.0419354	0.01502507	0.01009629	0.41162866	0.01920782	0.01576669
0.94771082	1.11440006	0.83237116	0.33534421	0.73589831	1.21387986	0.57623908	0.523242	1.39747657	0.43208734
0.87940669	0.30265947	0.94412069	1.55501997	0.69568264	0.55243121	0.83022195	1.28685574	0.34344956	0.43273792
3.36798774	3.33593712	3.65448563	3.04198149	2.79130659	3.53388266	3.11800128	2.95487897	3.09908389	3.8177567
0.89509293	1.06241727	2.16623496	1.9226469	2.54296004	1.74639107	1.20709036	1.52149663	1.13632696	2.59918406
4.20914471	4.16098897	2.40283802	2.87647234	3.34224647	2.79099986	2.96571374	2.5682417	4.75207284	3.01567997
3.61454428	3.73274894	3.62227537	3.15450343	3.72534872	3.05047145	2.64745601	2.69837285	3.88271387	3.55412789
2.19755416	2.30537707	0.97021871	1.68368724	1.54148209	1.23398215	1.05780506	1.67907267	1.75409521	0.80764049
3.51006835	3.09920055	3.69813732	5.16855134	3.26260759	2.81774871	2.00743391	5.46820565	3.02952731	2.48475524
0.02586796	0.01654101	0.0249398	0.03679194	0	0.01934626	0	0.02285353	0	0.20011043
2.49331947	3.93378065	2.16456992	1.73632052	2.84333463	3.05090401	2.53571355	2.2022407	4.06985575	1.99487755
4.29097355	5.70428289	4.19704127	4.06671399	4.37563479	4.41026895	4.04851388	3.94335465	4.9958321	4.2953439
2.92210211	2.82097896	3.19350179	3.2318361	2.93806429	3.35117667	2.12112516	3.25273879	2.62572959	3.28429138
2.84455847	3.12724561	2.11286206	2.48426945	2.69645166	3.11421656	3.77622447	2.25417103	3.25718434	2.49357799
1.42389067	1.05378954	1.10637179	1.2285275	1.34695875	1.45861142	1.79720551	1.76032804	1.41898868	1.23507188
3.01504364	3.4694601	2.51434603	1.95418239	2.79055126	2.93796519	4.06758932	2.60684881	4.76334342	3.51474928
2.82337701	2.94044593	2.00102202	2.31146625	2.42878688	2.71346721	3.12221224	2.20130307	3.31892988	2.39572862
2.9111154	4.14836989	4.55291279	3.63397299	4.28590025	4.11613734	3.4406949	3.67260783	3.55612651	3.08792522
2.68756126	2.67376093	2.57204215	2.60951745	3.1287484	3.01722842	2.24122776	2.29878571	2.65433697	2.44859332
0.9026192	1.11784653	0.65101107	0.44022553	0.88996247	0.83478698	0.48560129	0.73266035	1.86419829	0.63892261
1.362675	1.8379721	2.0653065	0.92587274	1.68445894	2.16123002	1.95898117	1.46416109	1.42164944	0.89829753
4.34054381	3.03651631	3.42363658	4.83689115	2.84351255	3.65001433	1.93230571	4.57357891	2.70081074	3.46624837
3.28217542	2.68002552	4.35867498	3.50107212	3.0912648	4.11783234	3.27460273	3.39187304	2.25823117	3.45797844

1.04335854	0.91406313	1.86550431	1.62806668	1.48622945	1.40844109	1.94469314	2.37455845	1.18697347	1.15048894
0.08492644	0.10740977	0.64746267	0.30484486	0.28760621	0.43457629	0.13495125	0.6260661	0.02864063	0.09681261
1.27770871	1.75571316	1.29781249	1.02978258	1.4378487	1.68364077	1.27347171	3.39123732	1.63968879	1.34342699
0.1387234	0.83838317	0.59367536	0.1511788	0.29766414	1.56101852	0.36933946	1.02380627	0.70410891	0.05592536
2.02427286	0.90763916	1.94803424	3.05581227	2.55987615	1.31455917	2.03239903	3.36953698	1.02576	1.48586374
5.41049236	3.57917244	4.19674945	5.78289671	4.60690647	3.63116455	2.9519802	5.97883961	2.56758835	3.3289807
1.61890008	1.93858424	2.18748763	1.29169416	1.95483195	1.85939224	1.17884593	1.61691336	1.57049927	1.61366239
1.1220472	1.29441439	1.08192097	0.9240583	1.60156525	1.3750372	0.74337899	1.23013564	1.47505862	1.12537705
0.40691691	0.30390979	0.31790293	0.5111647	0.31646678	0.20041033	0.03931081	0.83126012	0.17380419	0.0695594
5.66343741	5.43253325	4.97457857	5.17890959	4.48263678	5.40520785	5.40062648	5.69544453	5.5662214	4.355252
0.05100087	0.27072395	0.16973508	0.29401226	0.37801728	0.37334836	0.15506769	0.10596842	0.08661441	0.22539658
1.41449008	0.91070187	1.40158143	1.56466746	2.61882776	1.34874754	1.17930973	1.91447776	1.1726576	0.92669602
2.13298862	1.27893124	2.7756534	1.34199664	1.79620168	1.59577103	1.38029643	1.41851101	1.79317981	1.31351676
3.02897689	3.37854533	3.53621635	3.30038152	3.70917877	4.01171065	3.70766349	3.40880847	3.42545625	2.86648653
2.22483994	2.88157842	2.29406732	2.56667841	2.37358118	2.15189094	2.14873678	2.19441573	2.20648396	2.18271729
2.70040676	3.12616787	3.15620527	3.05834736	2.83418509	2.58503916	1.88159849	2.99438964	2.22193598	3.13717096
2.32007009	2.01314006	2.36783364	1.79692792	3.53927597	2.29725	1.76403536	2.31842139	2.03733517	2.12821025
3.86460599	4.5641453	3.96261991	3.94277935	4.62962715	4.50991634	3.55960212	4.11909001	4.21815462	3.40489918
2.02052221	2.46505324	1.79908881	2.21850419	2.12281639	2.91692014	2.14509579	1.86349872	2.14015676	2.50904551
0	0.33762586	0.45971015	0.18773285	0.06494007	0	0	0.42612995	0.02493722	1.00045509
0	0.02870525	0.71653279	0.40338934	0.02840625	0.12198016	0	0.14016397	0.01620427	0.17587408
5.50697945	3.66052272	2.96430857	3.93788208	2.14169029	2.84396978	3.33029417	4.17019846	2.94971541	2.20173135
3.8629758	1.37783646	1.24775987	1.62444055	0.97202139	2.67695402	1.84940582	2.12327968	0.57855685	1.15880063
0.19408653	0.01099238	0	0.04860587	0.01087718	0.02560797	0.02576589	0.1173279	0.22891692	0.04011534
4.85119794	3.81764012	3.40816976	2.19501054	2.38231116	6.58184933	6.77698215	2.22238395	6.34301609	4.04538165
8.01877699	4.34189341	3.41606075	6.23249524	4.20650142	3.16977498	1.29635199	5.95519867	3.74715526	1.27107054
5.51913723	4.39565267	4.86659781	4.72168326	4.03547594	3.80144678	3.59323734	4.33150562	4.05952534	3.58418722
1.4651264	6.42425212	4.46379597	2.08180907	0.75526086	1.71328559	5.10500321	1.17966654	5.52828525	3.92546706
8.62920451	4.49432103	6.70395688	6.41723416	5.30623365	5.66213168	6.00359812	5.11081538	2.55295015	2.62759074
3.95128823	1.63200114	1.20691355	1.74254835	1.15424422	1.58721255	0.90647383	2.12813632	1.4051307	1.02920153
1.90715654	0.1718735	0.17318654	1.14704391	0.30158171	0.70912073	0.59550918	1.30348371	0.21728228	0.46213308
4.10259927	2.32178359	0.40807357	2.73329885	1.69871099	0.99065692	0.17291929	1.08240859	0.74885761	8.05857598
0.77520346	3.75165283	1.77354854	1.36737542	0.82088437	2.62147974	1.83169596	2.3938436	0.98329538	0.63896439
2.30759995	1.10427131	1.71099483	2.018389	2.46977066	1.10860924	2.24618596	1.27869754	0.65083906	0.30086622
1.68403896	1.11148419	2.01835997	0.81555616	2.35773603	1.85326243	3.46249186	1.15041829	2.53541193	1.014599
5.96186619	6.04189656	4.40701193	5.32746137	5.34232316	5.60908458	5.73028952	4.41347767	7.44845478	2.08084804
1.78920887	1.04850056	1.47552567	0.95009015	1.92792911	2.05156141	2.79316266	1.43245016	1.75624846	0.95300614
2.96107245	2.2674836	3.28924389	2.69718771	3.0567089	2.70271903	2.87015659	2.86151436	1.9721474	2.49154512
2.0576428	5.20078228	1.69149251	1.89154742	1.29815217	0.90710865	0.46744827	2.83783532	1.26462728	0.8818141
3.47894937	5.35830407	4.08941305	2.1933976	3.77296652	4.57013794	4.24786065	2.80607201	4.6688567	3.37233522
3.81242455	5.44065184	4.61830832	3.03889884	4.14373692	4.67787638	4.84319418	3.82379055	4.99351411	3.47066194
3.7481755	3.95273178	3.16209358	2.75224519	2.91286719	3.14576992	3.09002646	3.49725939	3.45609308	3.5261581
3.39284907	4.67088852	3.55858293	2.31273238	4.03513043	4.33755927	4.05905656	3.30321635	4.2894192	2.88677883
2.73382665	3.56982012	2.32335491	2.1832031	3.30628299	2.9866686	2.95548211	2.65072683	3.58207367	2.51308854
4.0321145	6.2028009	4.85336818	6.32261863	6.01880075	5.65367838	5.44610489	4.84148535	6.1091674	5.34354273
4.37236053	2.66779188	3.71106663	4.27278279	3.118759	4.96762614	5.05947572	4.75172276	2.69708626	6.14511363
4.30557283	5.76612213	5.79307871	4.27301219	4.10463922	3.69282413	2.90477957	3.66215544	3.37230917	3.43413208
2.91674081	2.80485025	2.80527656	2.55570832	2.96413707	1.89167889	2.05073079	2.32428561	2.273627	2.12515682
1.25519191	1.15657036	3.64033303	1.29910092	2.69281904	2.25656487	2.65522142	1.51797198	0.66722724	1.79424335
2.19091292	3.43234073	2.81215752	1.69674193	3.07603729	3.48388942	2.62831057	2.78372812	3.0020181	1.94586692
4.90065205	6.06006141	3.93078778	4.3265139	4.29684843	5.30307834	4.75955739	4.29957682	5.58111655	4.15372048
2.40785424	2.98626355	2.92259958	2.24495635	2.19446717	2.52571909	2.16779835	2.32511038	2.95073299	2.45171785
6.77271796	6.41852945	6.57361253	6.71600545	6.81280516	6.22114693	6.63256595	6.36082447	6.35155491	6.11904815
1.01684088	1.69571433	1.7030528	1.13719196	1.50512127	1.62216612	1.18710575	1.37508204	1.28319678	1.33996334
2.68866432	2.7500395	2.6456376	1.92859115	3.06073166	3.23613385	1.59701061	2.41199755	3.18330377	2.88156417
2.5600897	2.99304521	2.70446989	2.17773362	3.45158631	2.81678148	1.79135068	2.58601428	3.4787226	2.96667785
2.3093293	4.23604477	2.93460255	2.5444722	2.53806588	2.30094527	2.09582877	1.61787119	3.59777024	2.39717737
1.9588403	2.52772014	2.24539422	1.99987845	2.6442827	2.41023406	1.8585245	2.06025394	2.10282114	1.97241186
1.55037766	2.22182997	1.8569742	1.26007429	1.91019611	1.63213279	1.21751499	1.74816919	2.25138925	1.49263989

3.56617905	4.04531457	3.64643135	3.96976116	3.75241932	4.53313829	2.87891413	3.7063958	3.84214936	3.99004853
1.99559315	2.85261932	3.06335578	2.16168145	3.27978536	2.79878294	2.29939558	2.94131288	2.33686597	1.3776156
0.95215616	1.3933701	1.5897754	1.02367388	1.93507744	1.92572863	0.94133864	1.13079869	1.15709023	0.89890937
1.40466848	1.25336288	1.57880385	1.73926833	1.67185758	1.56366104	1.54681883	1.59587804	1.63618876	1.53150483
2.36552542	2.93866088	2.97523326	2.04580735	3.17893583	2.98218228	2.59186063	1.83225452	2.61025875	1.68469065
0.5728311	0.6035946	0.49120477	1.04600272	0.45194853	0.40919811	0.2821339	0.87724614	1.28173695	0.40647953
1.09630124	0.74445444	0.5856412	0.94345812	1.29463522	1.02083435	0.42778319	0.81446788	0.68033309	0.22262505
2.98255623	1.43652745	0.9994902	1.07958359	1.67443421	1.41143513	0.48654539	1.45969637	0.71326045	0.32067479
2.01354907	2.39833007	1.61607107	1.42311952	2.61145721	2.20229779	1.30321658	1.28800785	2.37335898	0.51453176
5.85135121	2.16466929	1.59518917	2.89861872	2.47167437	1.97095161	0.42716924	2.86672457	1.81680078	0.33505336
3.49108759	4.04918	3.03498698	3.2010717	3.27100997	2.99089662	3.22823063	2.9259162	3.44027273	3.07730222
1.13285494	1.60625162	1.56990449	1.13060261	2.08087002	2.29122502	1.26904428	1.33439819	1.61077874	0.95961858
2.15645427	2.27966986	3.19537176	2.89781957	2.96402161	2.91139639	1.82549745	2.63310286	2.10793628	2.26482299
4.48121395	5.95642503	4.53076238	4.20655531	5.00257776	5.24336302	5.06308459	4.16830565	5.39448081	4.18819806
5.53338753	6.83903533	6.46327758	5.56281308	6.34917067	6.39694545	5.98213447	6.07930049	6.31184611	5.60503862
5.21373379	6.38016917	5.75258162	4.89051426	5.82420286	6.05216688	5.62840379	5.29762922	5.74137257	5.38917848
4.53093739	5.89160273	4.91490187	4.39731686	5.08004935	4.8910815	4.75640828	4.52639831	5.2622668	4.40890056
4.60601653	6.16400675	5.6176626	4.58241075	6.45142511	5.98369842	5.30069207	4.81901983	5.80210173	5.41541472
0.20124771	0.36314712	0.41021521	0.19795892	0.16974476	0.44394594	0.12517556	0.28205012	0.19573631	0.33884269
5.8195571	6.84752478	5.87255462	5.61760102	5.83333559	6.77891478	6.58854769	5.88607617	6.36761326	5.84399954
4.99605951	6.32068862	4.47058205	4.41889088	5.20439002	5.44780229	5.45112816	4.85699619	5.74439019	5.08593885
0.72320519	0.75584335	1.34779327	0.60441612	0.94684676	0.75670846	0.67020574	0.64069692	0.80028554	0.9121765
0.95630641	1.03235904	0.48590041	0.61915087	1.31385216	0.96988073	1.1257843	0.5235217	1.0299367	0.5271108
0.97460046	0.70405176	2.37347849	1.88245659	3.18283383	1.43467167	1.02515886	2.41034024	0.41742827	1.1418765
6.0522699	6.99996642	6.78104656	6.77711031	5.87077459	6.36596854	6.32842702	6.70911578	5.92877087	6.77187332
2.23075676	2.74217049	3.06084609	2.15402671	3.55498213	3.32998208	2.21616095	2.31311322	2.2083848	1.05068303
1.39874405	0.77623599	0.47747324	0.58891099	0.6974664	0.51967537	0.22119061	0.94575429	0.93492372	1.0687078
5.75692503	6.99880559	6.1538413	4.95950584	5.93113808	5.24890126	5.54160458	4.93220922	5.95372542	5.74882678
2.67598612	1.14687423	1.40794741	1.40311486	1.7459791	1.40016734	1.34876865	1.08767153	1.07798981	0.31037234
4.67690653	0.79337258	0.85875083	1.85722403	0.77467444	1.55924346	0.65957517	0.93235507	0.77227764	0.109161
1.45802667	0.64157276	0.39436315	0.69396994	0.27238364	0.14314144	1.22307766	0.47272181	0.06063274	0.10862845
4.07116033	2.10116749	1.14938706	2.63029661	1.24515648	0.69625879	0.71901457	2.01959716	0.85999322	0.54290189
2.25998951	1.57694055	0.79916446	1.19028888	1.13811593	0.74783364	0.33917042	1.0880341	1.04603892	0.57987674
2.03453873	1.3641607	0.64861991	1.25849364	0.81137899	0.27817399	0.37804002	0.92165466	0.2842822	0.05269717
4.5750105	3.99347302	3.53852039	3.49341931	4.19020519	3.96799548	3.47102979	2.70374518	4.46791672	1.30122448
3.45097351	1.84441288	2.59557907	2.82518185	2.61324849	2.41366962	1.35335532	3.60364433	1.69415979	2.170518
1.8426166	1.24071019	0.34051243	1.22828295	0.24319081	0.1046033	0.66768134	0.52086612	0.36604834	0.15576361
2.37767064	2.16611	1.37498513	1.88370788	2.39641485	1.80374022	1.79836431	2.29791297	2.12558979	0.62854485
4.57906825	0.60591753	0.93390551	1.40920404	0.74234508	0.88903307	0.32195919	1.10000548	0.50955755	0.14594376
0.47165772	0.36133868	0.34948631	0.23657308	0.52931362	0.39116834	0.14535209	0.19693857	0.34785441	0.06608707
7.43371247	8.80627508	8.78157767	6.87959079	9.18788496	6.77985382	4.46642041	6.21248472	9.00262434	5.72374001
0.70483877	0.87634761	0.33212696	0.33109628	0.7115145	0.55051366	0.71676886	0.32189422	1.40488861	0.07263426
2.39770217	1.33968532	1.14188905	1.24053002	1.6063916	1.40972053	1.60274874	1.20564832	2.10955505	0.19183417
5.25892126	4.02998735	5.75150019	6.11430937	4.39154414	4.17351153	5.28357252	6.56824858	3.81953387	4.8291195
5.04051542	2.91579865	1.81073173	3.49809067	2.79577351	3.06820094	2.45417552	2.44723904	3.43361206	0.61800486
3.41918731	4.34516603	5.39368521	4.48428299	5.05367028	4.12797548	4.65776096	5.089269	3.02168406	4.62226593
3.06872989	4.47163567	4.94234386	2.62168103	4.50369477	4.21407294	3.72436837	2.73661811	4.39252185	3.48040649
3.65769378	3.96029265	3.99525493	3.84209212	3.91791635	4.29251	3.51139597	3.81157082	3.99195271	3.82939882
2.47254749	2.55987045	2.05275814	2.04598571	2.68697428	2.02081738	2.97278324	1.68239037	2.41768927	0.95589563
0.83701459	1.21892496	1.00756901	0.68168817	1.63886412	1.2692139	1.32900927	0.88295508	0.72453041	0.57165553
0.98342244	0.58043847	0.48078253	0.65859167	0.41138715	0.24332877	0.20188711	0.49305534	0.38814482	0.20245562
1.99728255	1.61138845	1.58971365	0.92852694	2.15010075	2.08564774	1.87236425	0.78067811	1.84601635	0.62865552
0.90896231	0.10930972	0.04824623	0.26483992	0.19839773	0.02354705	0	2.63717251	0.01812169	0
1.86180034	2.38205621	1.70943085	2.04559783	1.59351677	1.37954587	0.42979808	1.87384948	0.55678898	0.68471348
4.82399827	5.21028731	3.59793661	4.23753194	3.53698997	3.76611661	5.08366256	4.47024564	4.99716514	3.53560368
0.69595587	0.70249583	0.54286311	0.53611398	0.85301497	0.55368932	0.62702919	0.50210571	0.51260168	0.26004789
4.09530444	2.82164893	3.42461899	4.26834297	3.3056499	2.712084	1.90267915	5.09146025	2.99005473	2.66594199
7.20048826	3.17066291	2.70194373	4.0755215	3.74168471	3.25540264	3.45444358	3.21041275	3.26599811	1.63575795
1.61099868	1.48237484	0.82609855	1.18443797	1.39761533	1.74556453	1.93769786	1.02476852	1.83687483	0.18737038

5.08086769	3.57438202	2.93430809	3.53508541	3.53234345	3.42169984	3.93316738	2.85389648	4.81331536	1.0903229
5.30271792	3.87174923	3.28638359	3.84786238	3.85846386	3.79569018	3.76289755	3.15473524	4.519342	1.25291886
1.14154522	1.63164384	1.63272471	1.32291293	2.03831811	2.41054736	1.87228129	1.77183587	1.93276438	1.46209954
2.22225078	1.53362367	1.39316452	1.30424694	1.73375305	1.6363418	1.4696797	0.98712584	2.2581554	0.20642889
4.81539858	4.57717458	4.35155677	3.93418592	5.33226131	4.34096267	4.10579849	3.65795135	4.21738954	2.41058135
3.92394093	2.29613376	3.80461269	2.91806958	2.94800215	2.97103741	2.46158237	3.21163987	3.06107105	4.39705763
4.84795583	4.75105479	5.34146909	5.00593152	4.12997277	5.0848073	4.08156175	5.24167084	3.53946571	5.04305082
3.58841431	4.43997362	4.28526434	3.79168702	4.91667534	4.60057917	4.01881017	3.75305792	4.05734515	5.1358812
3.58282072	3.83042486	3.87095276	3.32950537	3.81333283	3.49514245	3.5363432	3.07553867	4.62458424	2.99827369
5.71112579	2.67492792	2.1617915	3.30669566	2.76490777	2.60741939	2.6261626	2.27385155	3.46693641	0.84883052
3.89514008	2.35395322	2.50799611	2.7054276	2.84958955	2.18859935	1.38667899	2.17535455	2.27100412	1.33432553
7.96001171	4.86387028	4.71449269	4.99218429	5.04073964	4.89614345	5.15665452	5.03752035	6.61363101	3.2023252
6.05703691	4.43968302	4.26488032	4.32054782	4.90499859	4.62875747	4.32571245	3.49679895	4.47397684	2.38077049
5.30064828	3.50287909	5.11674324	4.50475447	6.0490876	4.55366494	5.6724079	6.56496876	4.0121162	4.75603985
3.27060792	2.85882288	3.32372172	2.86365542	4.71463788	3.52153186	2.68057865	2.46734902	2.90863843	3.98779697
5.36309262	5.60579662	6.0152809	6.05488427	6.50888936	4.85120261	4.98476064	6.0289399	5.01290409	5.51253802
2.98289842	2.26684799	1.870444	1.8511091	2.37485972	1.81831881	1.3657737	1.86931948	2.45011953	2.11490996
7.08891992	6.80842304	7.05049171	7.49495903	6.96711756	6.70967047	7.7478472	7.1067903	7.41305456	7.18248897
4.43747916	3.38635548	2.8548536	3.1219372	3.13170626	3.29176116	4.13499953	2.83972899	4.05527707	0.81650234
3.47239773	1.16915254	1.19578279	1.16053419	1.74268906	1.35550073	1.19249425	0.89391775	1.23034091	0.61588617
11.3624184	9.95302869	9.33867006	10.6462571	10.4235768	10.5836575	10.2838115	9.01152126	10.4313684	8.26510717
9.07078287	3.66944083	3.2680817	5.9650408	3.67635099	4.58142701	4.60866964	3.95119761	5.09345217	0.96258393
6.3125159	1.37317699	1.07416264	3.0985434	1.30205662	1.55065912	1.24328545	2.47463211	1.81093175	0.37790805
0.42922572	0.38776509	0.72712505	0.17719083	1.00437603	0.54938626	0.49307312	0.23504946	0.34674663	0.08603387
6.02909378	6.22542553	6.30874646	6.79516553	5.92465527	5.53775758	5.94289064	6.40572769	6.04561226	5.33008941
4.91785701	3.84615673	3.50448812	4.35143695	4.13729828	4.5923933	5.43755673	4.31455682	4.52725438	5.54349822
4.98134625	2.72956513	2.78829546	2.44656604	3.21594854	3.12914185	2.67906781	2.28615942	2.58116837	1.91452613
3.04246418	3.90790836	2.35048874	2.56749014	3.23666974	3.15493455	1.55654098	1.95608815	4.00692464	0.54157843
5.43481285	3.97468362	7.26418785	6.3106921	4.65927977	6.39174073	6.66040178	5.45689403	5.84151304	8.78910116
3.9193202	3.43101013	4.3649377	4.62918553	4.06545559	3.59192207	2.47164908	5.73903987	3.00098447	3.06938633
2.31951272	1.82920597	1.62263188	1.36148193	2.0829231	1.81133121	1.41333305	1.17137544	2.29532808	0.23085232
6.48511285	5.9783714	5.74417694	6.29227769	5.23917969	5.49719161	6.38255128	6.02110658	5.42371978	5.76623516
3.18348169	3.2095409	3.21123679	4.0800343	3.18035104	3.32162747	2.16692776	4.07448439	2.0972397	2.62500391
2.4984177	1.72821614	5.83662892	2.96062062	1.45450222	1.75864046	5.22636304	3.2695629	1.12655218	7.21254351
1.03251305	1.82712835	1.2854871	1.23010132	1.44285332	1.37897699	0.88763759	1.22179285	1.36072913	1.46514243
1.46570684	2.22915288	1.8133204	1.64206081	2.22618925	1.76772163	1.3099244	1.84421814	2.1093316	1.79626058
4.22617119	4.39745313	5.21273705	4.14468841	4.26510876	5.72544493	4.53264197	4.13458584	4.61483522	5.10274782
0.73811509	1.43195552	1.48987588	1.07968491	1.41184814	1.25102758	0.94037921	1.15495728	1.37797884	0.35414578
1.4648415	1.74791077	1.87613148	1.37708755	2.18067653	1.8426928	0.79323241	1.84274852	1.82478863	1.39903367
3.89725019	4.13533557	3.84372156	3.43689249	3.98976247	4.09867801	3.59179915	3.50340527	3.83243974	4.89984646
4.70751953	6.01984674	4.79709874	3.81532883	4.92855497	4.89109043	5.72743861	4.84032845	5.39469309	3.84308929
2.43669268	3.67897229	3.33664102	2.33019592	3.08058222	3.30994725	2.248875	2.52659545	3.24208051	2.74348102
1.79935663	3.34795129	1.82686322	0.72945462	1.90343055	2.55251709	2.03129808	1.40975192	3.36585568	1.30167141
4.34739365	4.65594299	5.26788963	4.24536114	6.02544417	6.05682044	4.320008	4.74889024	4.26870252	6.26839935
1.75551059	2.58585622	2.32017961	1.12648726	1.64270704	2.36638611	1.93000823	1.32382339	2.33769691	1.18241154
2.54269996	2.51091719	2.51187119	2.1005799	2.42480529	2.74736331	2.46119976	2.38781693	3.25550549	2.39362488
4.63490478	4.7551711	5.29520446	4.58764077	4.27893658	4.2346051	5.47477944	4.43367328	5.40824162	5.5246243
5.72847801	6.11194475	6.13200228	5.68565603	5.62421274	6.42753098	6.24295019	6.32639923	6.51007985	5.91219498
1.74088571	1.58803884	1.84178133	1.6548075	1.395622	2.02315821	1.55278917	1.73381893	2.24143756	1.67051924
2.01847442	2.41585781	2.5619963	1.58977722	2.62573526	2.24166136	1.54918622	1.7576484	2.05388129	1.69775026
6.03623223	6.09174748	6.11820723	5.59499138	6.03188244	5.95223058	6.00594414	5.89580642	6.45298726	5.82045466
2.17964692	2.90397251	2.75448303	2.67128041	3.87829898	2.916232	1.80959064	3.31011111	2.55888853	2.30810909
3.55401886	3.93143773	4.28772823	4.35729624	4.4880932	4.37059302	3.96509674	4.72652199	3.84803954	4.30886351
2.00228377	2.90000484	3.29381213	1.41786523	3.386589	2.62797431	2.44972171	1.71037733	2.3230114	2.95172464
6.48843745	7.99862042	6.32577348	7.36977107	7.32265224	6.93629185	8.23541607	7.3109673	7.88729415	6.71772005
4.09835363	3.22662948	4.43133034	3.83799246	3.75723809	4.00427716	3.99487839	4.65504095	2.21435511	3.38421637
2.26757647	1.7571874	2.5429503	3.46518013	2.45780462	1.97814707	1.68559569	3.08337704	1.58774584	2.61522797
4.34770386	4.76331873	4.02989633	4.99059629	3.95989822	4.09352813	3.74477122	4.28453322	4.46953139	4.12137879
7.2618598	5.04096712	6.75303372	5.9855261	4.19307266	5.92891203	9.00657886	5.80190901	6.3784342	6.58725957

4.19547139	4.31853779	4.8483364	3.67777645	5.10553421	4.51266544	3.65890738	4.2549809	3.84616889	3.95848018
3.98150243	3.92202244	4.20288281	3.25865313	3.30580563	3.98418824	3.70725628	2.89107692	3.47840919	3.54360328
1.80439317	3.61563924	1.87188638	1.48839307	2.69221997	3.17697322	2.4993737	2.01952635	3.39728004	1.79286728
2.82639308	3.59298582	2.73574286	2.73547818	3.37980686	4.15992612	2.75678828	2.79871207	3.05837066	2.77645398
1.83468803	2.98778748	2.48781534	1.51976442	2.55071227	5.23840741	2.26655685	2.03136767	2.90717975	1.73995204
1.89338663	3.44874534	1.72482439	1.05344755	2.95191396	2.11258329	2.18216337	1.21227166	2.84499261	1.06385882
2.31500084	2.67086543	2.25551864	1.4396286	1.98823602	3.15972391	3.32760976	1.64426995	2.45906505	2.41152507
3.10629375	4.96259463	4.67557028	2.66685008	3.48672806	3.99448166	3.58105109	3.75848446	4.28252458	2.94339355
2.64431314	4.15560917	3.58465349	1.90127287	3.44933663	3.7153367	3.38516509	2.55768902	3.87618436	2.38346535
3.93641721	4.49685747	2.78796901	2.97840609	3.62329507	5.17562628	3.85645087	2.99181495	4.33099781	4.13747214
2.93299493	2.62753653	1.50007928	2.75277994	4.86765696	2.61919951	1.53295932	3.03869303	1.26219225	1.72130939
3.57807888	4.56328285	3.9761616	2.61234814	3.58898989	4.26848949	4.1300218	3.21080612	4.24335732	2.8978115
2.51555957	3.81668462	3.90498326	2.57881528	3.78860787	3.13981842	4.0637693	2.85765884	3.6892662	4.28593989
5.3121103	6.65959058	4.95188908	5.085505	5.56509313	5.74001201	5.32933825	4.5081705	6.21688747	6.05104798
0.50907072	0.04146168	0.06731189	0.0614854	0	0.03646878	0.00618065	0.01928496	0	0.00644668
2.60181574	2.6307774	4.14657677	4.20843605	3.9616656	3.43510009	3.17602664	4.2001242	2.38035569	3.06280027
0.05126937	0.02881732	0.07354661	0.28119983	0.25226643	0.03842832	0.06231356	0.39390992	0.00931834	0.06981861
0.8260109	0	0.0158318	0.09872855	0	2.70929387	1.61496049	0.18233358	0.29827638	0.00645371
6.6028982	0.40208752	6.79852398	3.45782752	0.42771834	6.41795987	6.53860703	0.78212723	0.71199001	7.86659832
0.45749394	0.18290684	0.19747451	0.64490995	0.3683564	0.1581749	0.09473476	1.21414178	0.1883896	0.22169192
2.12479951	2.62136758	2.31373244	2.31326287	2.25231778	2.49182247	1.79641718	2.55789739	2.16110383	1.84534643
2.52576591	0.82900591	3.78118712	2.06841511	2.45990827	4.15488101	2.9913374	2.10746126	0.58509318	3.32419631
3.854014	3.02357187	3.56004702	4.08493131	3.51578518	2.89134387	1.93281617	5.30206332	3.02693098	2.81410506
3.1564709	0.44536226	1.26787387	1.16848478	0.19921887	1.05281033	4.3036767	0.41808164	0.36287108	1.97117968
0.42814398	0.26244398	0.64305843	0.41626959	0.43309194	0.69133657	0.45212969	0.51339904	0.75318459	0.8314202
4.92859481	1.31289327	6.12467201	3.95453663	2.7503013	4.47227125	4.54515936	2.79204519	2.38190101	4.09336089
2.91009749	1.74561029	3.44283283	2.97122857	3.20955398	3.73190525	1.8725757	3.28500868	1.89500733	2.67715052
4.84521249	4.75294443	5.58027023	5.48132719	5.25812514	5.09276834	4.45177159	5.0673415	4.55919291	5.13658866
2.62886728	4.50158792	4.18556274	1.99260407	3.76456276	3.80677278	3.42994017	2.554322	3.92138614	2.67049337
2.67022156	2.79365516	1.91539538	2.27924223	3.21162254	2.82046918	2.26492694	2.36314223	3.0453476	2.86783719
1.64522358	1.16894985	0.79505018	0.60920736	1.15140999	1.22572327	1.10198749	1.09188704	1.33972072	1.15532294
2.14653806	3.17500192	2.64656302	2.18765388	2.91275784	4.02719834	2.19879989	2.52757845	3.17128218	2.15736595
2.54587701	3.03105503	3.66709923	2.54397061	3.25348641	3.3896687	2.23449535	2.87377895	2.94794576	2.91089961
1.98151009	0.71843639	2.03763784	2.02467436	1.92432859	1.81750733	1.53926684	2.3217573	0.89808419	1.45518587
3.8603916	4.6380246	5.2407063	3.96070584	4.17137729	4.4973608	4.87463354	4.25748683	4.12718735	4.40189521
2.10716874	2.56964295	2.49354212	2.20997883	2.6764301	2.28288399	1.3371179	2.43621985	1.96498043	2.03896738
1.92984093	2.42037867	3.50655834	2.0243913	2.52961396	2.24025575	1.66101032	2.03781867	3.0023229	1.86682294
2.62405526	3.17899502	3.76966061	2.51122928	3.44857865	4.05278206	2.85006223	2.55326975	2.74890691	2.43376109
0.50241769	0.65561987	1.35562187	1.11911554	1.02159706	0.92172843	0.29599431	0.97321631	0.65147068	0.83529142
6.13041503	5.17476862	4.8281677	6.43690436	5.63636894	6.70218823	7.21029838	5.4604869	5.30269119	5.69254951
0.06547464	0.19907623	0.10377467	0.04704098	0.18787706	0.14265549	0.03723527	0.1136358	0.35924834	0.07662301
4.82354469	5.22901306	4.25126132	4.58611693	4.84680081	5.29758867	4.86901587	4.49344212	4.60154094	4.17338451
2.6189305	3.02047853	2.3649291	2.46701381	2.42520893	3.42316725	2.76955928	2.84349343	3.35292115	3.53677404
2.14803509	3.18619027	2.97514683	2.12813152	3.02830235	3.00728219	1.7367164	2.08431737	2.99759747	2.54104958
2.12683127	3.11137544	2.59994626	2.16920314	2.73363362	2.6945322	1.69117085	2.46651198	2.80689799	2.1524262
2.85951153	3.67764581	2.65513511	2.97547097	3.53607281	3.14430368	2.74199961	3.07874331	3.56372739	2.73727388
2.72783276	2.50493067	2.48431239	1.9319419	7.37578082	2.9173849	1.8249137	2.66335702	2.15108979	2.81895025
2.55567803	3.10502321	3.96429016	2.279106	2.86935269	3.39003174	3.63304618	2.56241559	3.28759738	3.07029612
2.48442231	2.87606114	2.84779747	2.84662128	2.79549864	3.40120052	2.78061228	2.77544256	2.46631624	4.02374219
4.03588463	4.41320188	4.19673942	4.05052942	4.26804542	4.17873211	3.48517107	4.17324591	4.49774135	3.84825841
2.14397339	3.43060884	1.84588345	1.77016487	2.36617924	2.46554483	1.49215459	1.80474708	2.16553432	1.78531595
0.32798655	0.11716536	0.68718116	0.43971845	0.32323788	0.23606395	0.17020297	0.57783578	0.14067113	0.20072817
0.0548418	0.07921888	0.87917794	0.4528905	0.35531029	0.17381313	0.12621911	0.18494959	0.03392607	0.14279786
5.85420814	6.19006973	5.92948866	5.67355938	4.66146369	5.17814549	5.15068857	5.26757223	4.29589126	5.90980903
4.13586734	4.87775716	3.847435	4.29756721	4.26613716	4.37539019	3.89366753	3.99589782	4.56825867	4.52031849
3.22773168	1.66049411	2.87348096	4.10295508	2.55939219	2.44960158	2.4082033	4.41941866	1.58237412	4.15098678
1.87521631	1.09350057	4.4427267	1.28429574	4.54881548	1.2210208	0.9480253	3.21984871	1.74645546	4.07919412
2.96893866	3.33609368	2.14942384	2.64305726	2.7992198	3.08787996	2.70413669	2.41117435	3.74187288	2.32755406
3.39628866	4.5326542	3.07053222	2.59845377	2.61604852	2.83625272	3.20039272	2.91025701	4.33762905	2.80322186

2.08187403	2.62846711	3.73304601	2.72371176	3.47361856	1.90599081	1.79834684	3.82156126	2.77048605	3.16241778
2.79918873	3.21560983	2.61797611	2.64593345	3.00876976	1.27582461	2.30871149	2.33910051	2.7113817	2.17705645
3.51903485	4.03261712	2.54988252	2.29128298	2.86737292	3.92641086	3.61119706	3.11417873	3.65313883	2.62298199
0.47138132	0.9553365	0.4069656	0.37723286	1.33525656	0.46306458	0.11317325	0.40799059	0.55309002	0.52113443
1.33349239	0.74010292	0.78134315	2.38216667	0.88764878	0.69021284	0.23812524	2.97705952	0.4395651	0.40270593
2.53380872	2.4305128	1.70197685	2.42261011	1.85597503	2.62383916	2.21057186	2.39898265	2.57308089	2.19300373
3.94539369	3.3749251	4.26764591	5.40538979	5.49496658	4.21853913	3.34338965	4.76481657	5.27071132	3.5041802
0.04644148	0.02132874	0.02576227	0.01277826	0.10652653	0.0735597	0.02010903	0.12166633	0.06609417	0.18324505
0.30120644	0.11398293	0.07569819	0.07757986	0.06875091	0.11344838	0.00696918	0.24139705	0.45382104	0.20356119
3.13854291	4.54078998	3.06783769	2.23454411	2.48713264	2.87570574	3.29003271	1.37595136	3.68237788	2.28908651
2.47537156	2.58953345	2.75790575	2.90079886	2.95185747	2.94373334	1.76918284	3.01280479	2.4009776	2.55149023
3.40916544	5.15391447	3.95748291	2.65587432	3.99774249	4.63601098	4.57189085	3.35230102	4.69442778	2.63874972
4.7834417	5.24695371	5.1607067	4.51836216	4.94203702	5.48272698	4.8651903	4.94606492	4.82467571	5.26230332
7.19892794	0.20382825	5.99233656	3.4615738	0.32798877	7.11678736	7.85224399	0.35632868	0.18455749	5.63431386
3.71583011	0.05046159	6.36199595	2.66114909	1.27218735	5.08673373	5.24517994	1.53598061	0.18679376	5.47429341
2.2854621	2.79877224	2.85623723	2.50080694	2.7100264	3.22405083	2.40984438	2.75379622	2.58743385	2.65198051
1.13805663	0.77862991	1.89659979	1.27222077	1.14859307	1.33875207	0.95756877	1.60973686	0.8387795	1.23176491
2.48035685	2.4972052	5.84144958	3.28884983	2.37928809	4.13959203	4.56211409	2.49020247	3.3667965	4.24066577
0.14889061	0.07902022	0.23221442	0.08162049	0.05459459	0.10574945	0.316012	0.08945032	0.10821436	0.11080114
6.22680326	2.1947353	10.4731195	2.97252818	0.72676122	8.36134747	8.07269021	1.1044007	2.28361746	8.02376124
6.21587419	1.70274426	10.0976402	1.66898428	1.23220813	8.48725296	6.63441808	1.11231848	1.55853493	5.83601284
0.23119519	0.61232494	6.95953515	0.8089687	0.02846996	1.17732018	0.47047876	0.312327	0.12507808	1.73672308
4.90926184	4.36351945	5.60829595	4.02219534	4.56068951	6.39784319	4.00293401	4.41545403	3.81494466	3.56790986
5.84476729	5.85705981	6.47824274	6.70464789	6.06854723	6.37075459	6.38070856	6.20990408	6.16718489	5.72558105
6.56960586	6.33793158	6.36270321	7.21478482	6.08859283	5.93358357	5.68656477	6.5142791	6.42896916	4.90493916
3.56458157	3.0125893	4.85421641	2.84895206	3.93005918	4.83634411	4.12698967	2.99716192	3.29341061	3.5964563
3.16229093	3.8631512	3.48269628	2.65084858	3.80344976	4.0020205	3.23732412	3.19055735	3.24968752	3.07089668
4.35696302	0.20070524	0.34578947	0.15243981	0.09615655	2.72321964	3.96778835	0.25446855	0.12364734	6.01761058
3.21943978	4.13575895	3.59175865	3.3190448	4.14846805	3.97208941	3.00372836	3.65909631	4.0399899	3.03526762
2.96611521	2.09262122	2.28996252	2.70487197	2.19695061	2.24879476	1.43445858	2.6275214	1.82973309	3.09984814
0.01673238	0	0.0267843	0.02383019	0.02107332	0.0062655	0.0063044	0.00986818	0.0120081	0
1.25843652	0.73873694	2.35507784	1.88339105	1.83193606	1.38184499	1.64169346	2.72915114	0.48347136	3.00739501
1.05977935	3.13072902	1.47188603	2.41920058	3.18897704	2.58894308	0.66709258	1.5869686	2.47713655	1.01865679
0.39206157	1.5369701	1.90236501	1.23511154	1.42077397	1.55998865	0.80440438	0.95402516	0.780458	1.67273457
0.59266165	0.99324501	4.61984804	2.78041112	2.75891052	2.99254654	2.36645031	2.48498032	1.616308	5.6903169
1.95648555	3.08177228	2.91529263	1.70493476	2.45226169	3.29341876	2.62815318	2.0887242	2.64332109	2.09255666
5.81894443	6.06859875	5.56773024	6.19820331	7.23768291	6.11476591	5.60270572	6.00597515	5.47829119	6.44384308
1.81658019	3.69642851	2.08184912	1.26035671	3.45572073	3.45789152	2.47039986	2.74563333	3.03759181	1.95502968
2.12963777	3.73771254	2.12292476	1.16526386	1.92588331	2.947769	2.58657968	1.7051879	3.64151302	2.00865227
0.48248834	1.58513923	2.22409485	0.45775398	1.56161205	1.80176251	0.72010471	1.03868867	1.26002248	0.82996915
0.99031846	1.66394968	1.62941054	0.73542981	1.53529901	1.78790874	1.20698767	1.17261656	1.56091776	0.73846442
1.03103163	2.31561001	2.06810456	0.57499224	1.13540881	1.63923408	1.32061848	0.8613142	2.41489707	1.08759217
3.37752014	3.96756051	2.45942762	2.53480674	1.98076919	3.26282494	3.92200683	2.31473438	4.42466159	2.58859051
1.83079806	3.37206874	2.03754956	1.45219833	2.92305076	3.05090177	2.1954868	2.04737034	3.10401599	1.95450376
1.55486051	2.59169444	2.32795879	1.3900301	2.28395438	2.26071177	2.12063616	1.89795559	2.28860716	1.3356393
0.52625249	0.79870178	0.78424433	0.46925552	0.75470328	0.91329175	0.45482322	0.74101845	0.93774248	0.55445078
1.66066763	1.47004531	1.86014097	1.21526154	2.56372711	1.77567854	1.77056294	1.4357533	1.82528904	1.37765576
2.25991223	2.25944008	1.85287853	2.13107363	2.33038192	2.59746863	1.84969807	2.43337195	2.44062424	1.93921228
2.49257554	2.91037075	1.26181925	2.01032072	2.94733933	2.11490354	2.43738082	1.58165512	4.04414498	1.86038413
4.38547485	5.04654408	3.30862419	3.14822596	2.76967035	4.26259102	4.35672134	2.97010215	4.80563171	4.02633429
3.89206673	4.00301696	4.12579913	2.8607557	3.6251698	4.58626705	4.30719661	3.08684213	6.39341002	2.28575107
2.11595192	2.41291138	2.65212529	2.43449072	2.39318737	2.33140301	1.99373896	2.34938369	2.37316957	2.34101925
0.48811826	0.41477352	1.03937676	0.88369552	0.59247463	0.49554658	0.3451299	1.10321793	0.3200196	0.54953007
2.03171926	2.37780087	2.77468074	1.95270478	2.3189983	2.46456772	1.51867227	2.08973146	2.32207312	1.85041716
1.99519158	2.97288629	2.36363957	2.39894666	2.57819553	2.45958962	2.26171999	2.55900102	2.86315477	2.10092048
0.97382556	1.4154119	1.23704496	0.54020625	2.3039797	1.01907897	0.7186424	0.7904982	1.12210842	0.57111157
0.59197898	1.76440502	0.81336048	0.34007158	0.99549899	0.96786336	0.35278586	0.69427621	0.9830373	0.41263725
0.95926481	1.01159041	1.14634433	0.7919562	1.16150899	1.0494931	0.43624552	1.13827646	1.13735649	0.86667629
1.34074467	1.8049682	1.66837911	1.5597703	1.91448523	1.9350325	1.15045733	1.94446219	1.60267059	1.31512048

1.16701115	1.46572047	1.68984157	1.30422336	2.16922497	1.52111393	0.80603138	1.81891321	1.41659868	0.77041377
3.75802998	4.40889488	5.1185209	4.26934046	4.10816049	4.71494965	4.36738139	4.46281608	4.23522055	4.39794789
1.53827075	2.33351718	1.51360635	1.23633389	2.04381458	2.36006889	1.14671829	1.47940861	2.11596349	1.14308545
1.94770528	2.63903112	2.10546648	2.088436	2.15999306	2.9482293	1.41954217	1.81653322	2.43501484	2.35421581
0.75960884	0.89455757	0.97882292	0.58910427	1.23458989	1.09117876	0.74385335	0.90306319	1.06634947	0.80564534
0.73654139	1.32737692	1.44853055	0.97713011	0.97934015	0.96674339	0.63710889	1.42829142	1.08519408	1.60906541
0.70825287	1.11698212	1.27485332	0.76404016	1.1161888	1.16446841	0.65539096	0.76633925	1.10576259	0.79721713
2.41453148	3.5399197	3.14331612	1.69915026	3.89652201	4.30955968	3.26719621	2.93363389	3.53356447	2.50725499
0.94415159	1.39621633	1.61516402	0.71734998	1.26395144	0.86058541	0.77476838	0.8934682	1.14854434	0.61839217
1.0989419	1.427839	1.23136581	1.07358948	1.32368058	1.53621126	0.84297777	1.18626414	1.17057915	1.76266001
2.04218308	1.86844063	2.31379221	2.61819531	1.94601231	2.34590947	1.28879396	2.6567848	1.88568417	2.33433139
1.85155529	2.07208875	1.5858473	1.47981995	1.86145498	2.03510341	1.58397713	1.29524368	2.42111722	0.8308813
0.88690591	1.29416358	1.00700861	0.69203371	1.20511806	1.61936443	0.58200682	0.72314573	1.58321774	0.90019031
1.50057485	2.53292267	2.52588857	1.24060898	2.17708043	2.22854575	1.67267684	1.57104744	2.32797898	1.41453186
1.27571444	1.57633916	2.38945754	1.5682278	1.96152486	2.16484659	1.55421344	1.74752498	1.43053337	1.58019589
1.35040251	1.77880858	1.54504791	0.95276402	1.66210224	1.8402495	0.97274014	1.2114953	1.60501486	1.40916266
3.49735636	2.67564991	4.86114258	4.47578851	4.13061538	3.50372674	4.83723817	5.08725459	2.24242265	3.84927632
4.00903326	2.60176389	3.26831042	3.88870075	3.3413907	3.40573213	2.4534588	3.7841911	1.90965126	4.63286082
4.40584916	5.60380514	6.50710612	4.92756425	5.9636715	5.25491379	4.86884502	4.87966485	4.49869774	5.1896985
2.06829997	2.4110144	2.19986746	2.23300671	2.37108988	2.18128805	1.67290709	2.39358536	2.04395788	1.90568146
2.12857839	3.80544693	2.96186213	2.70511285	2.9713351	3.4044184	3.05161788	2.27390175	2.92000511	2.91495249
1.62658216	0.70601456	2.9333469	4.29155098	0.77022936	1.99381266	1.57250653	3.40494573	0.82032588	3.41724102
6.89866159	3.70162949	2.71856088	5.22329499	5.4235843	6.53333518	6.15046788	3.85155167	6.10013553	3.11134942
2.76581082	0.49379047	1.7966565	2.52071744	0.48938516	1.02036115	2.70935609	2.33828081	2.33720106	0.11508108
0.53451572	0.08187948	0.16058312	0.27635029	0.66070798	0.62604735	0.17213056	0.37302237	0.2213665	0.13016632
3.52856548	3.61383389	3.65999235	4.1512286	3.65966578	4.41428451	3.76381335	3.51384437	3.7521751	4.78979962
1.62656779	1.39230374	0.72930434	0.72215907	0.76241415	0.65005306	0.87699205	1.21590094	1.60521488	0.32373776
3.74775254	4.06448993	3.45259002	3.33508676	3.9211368	4.52320275	3.60031912	3.48982819	3.88147673	3.34166019
2.4584014	3.47053989	2.82158359	2.99310167	2.63672278	2.77984682	2.50599373	2.87252819	3.60473922	2.26504894
0.32537952	0.58410691	0.24044592	0.16084756	0.76837002	0.57213685	0.07801621	0.38940759	0.74267104	0.20959593
0.17732751	0.05540942	0.04164283	0.06906872	0.05657221	0.02537482	0.0526897	0.16511438	0.00409086	0.0089289
0.05130105	0.0195362	0.05094894	0.06623853	0.08713997	0.04876367	0.01595261	0.07881414	0.02695928	0.01663683
0.24269001	0.38732509	0.60210385	0.28704648	0.45083087	0.49229666	0.19679263	0.34115516	0.35122063	0.18435659
1.55947562	1.27983735	1.5171164	2.51695517	2.19834386	1.80481848	3.12275409	2.04076905	2.20377494	2.02629428
8.32087163	4.75668815	5.73880934	7.06218994	4.2142861	4.06667781	5.0938108	8.03827381	3.98392137	2.77574233
3.19700676	3.04869842	2.45514678	2.85827064	2.53574251	3.50855863	3.161741	3.03981367	2.87177309	3.48663027
4.49092337	2.55065062	3.27830805	3.7480166	3.72710287	2.67699672	2.27013936	3.97077076	2.29360394	2.02893651
3.04306755	3.65462226	2.10903892	4.16121353	4.82497181	2.03529757	1.32681865	3.85592885	3.28468513	2.22131679
8.51625685	8.02975292	8.02148982	7.53757057	7.66097019	7.99223844	8.46144862	7.29785487	8.20397979	7.77398717
1.65865437	2.43935549	2.76931916	3.6298156	1.71922889	2.59664937	1.04527451	3.09189753	1.68060353	2.635199
2.39381261	2.42908129	2.67576803	2.25561867	2.61966758	2.26321813	2.44825782	2.73174261	2.70461626	2.05777885
2.22700102	0.98082788	0.50611358	1.10671564	0.4744418	0.35980174	0.15486572	0.95338195	0.45101032	0.17039997
0	0	0	0	0	0	0	0	0	0
3.49406569	3.53185256	3.68387663	3.49751521	3.68782632	3.45566377	3.00385557	3.52253801	3.10763431	3.23346951
2.73119236	3.90903826	4.44279422	3.0766703	4.39240823	3.7129767	3.89874081	3.47373966	3.77428494	4.20451661
1.39806905	1.63308766	1.55367371	2.47080801	2.02879138	1.41057343	0.31599562	2.51717191	0.79112541	1.03703726
1.98499648	2.96345279	2.94021034	2.54670672	3.24834663	3.43879516	0.88908655	3.04963179	0.63864178	3.18848138
3.79023096	1.15975933	2.56389638	3.45102152	1.48165375	1.00690288	0.75674659	2.73076533	0.91789076	0.93649119
1.24040898	1.01726108	3.16640028	1.70274938	2.42768113	2.08925584	3.99271021	1.43253131	1.94167554	3.14099208
2.03742779	2.61102194	1.51744391	1.29135944	1.63606655	2.62433863	2.58112154	1.6002725	2.35304198	0.8231877
0.46806512	0.33236887	0.40962441	0.26179888	0.37069524	0.3821831	0.27414814	0.40924191	0.48481709	0.41852254
2.73164424	3.71874716	3.46394033	2.16640045	3.04951167	2.81427127	2.95370855	2.51751133	4.0438018	2.22042336
1.39366665	2.72151561	2.44647037	0.85872362	1.83637428	1.91826512	1.75808868	1.43474902	2.43777699	1.41434708
3.29361923	3.21655105	3.23931761	3.04430501	3.63901894	3.6121501	3.22993439	3.06394077	2.66167849	3.47193052
4.26970764	4.04103062	3.82712037	3.72350627	4.07702329	4.00995754	4.52069234	3.60001918	4.81650179	4.0236017
7.47394551	6.25890317	5.50885151	6.12596197	5.40964635	6.17559744	8.23206526	5.94712559	7.30076472	6.52160409
8.03945876	7.84799873	8.03619571	7.52566871	8.35185902	8.22941094	8.92935682	7.32785242	8.54036649	8.34141418
4.0742587	4.80053166	3.69605348	3.69637413	3.54310287	3.56516581	4.55982284	4.05757907	4.23214776	4.27712172
3.16732527	3.16538241	3.1264429	2.53604966	2.66967206	2.84883903	3.23229833	3.3739173	3.33457732	2.72368933

2.53860094	2.30313892	2.09411384	2.46609108	2.82688881	2.18912622	3.442212	2.25308465	2.89649296	2.53412112
2.28406838	1.44032779	1.85144464	1.62119037	1.73186554	1.78545961	2.12656221	1.45782213	2.32024802	2.54324679
2.33506457	2.68331958	1.885895	1.80424763	2.19739862	2.66212699	1.64844128	1.39445206	2.83487171	1.64194548
2.06564546	2.6777174	2.69043464	1.55348579	1.97083359	2.5025278	1.71409541	1.89459622	2.25306105	1.91349939
3.33984697	5.02648552	3.15550257	3.03740966	4.18074068	3.87377431	3.6509549	3.37178463	3.58461626	3.2318485
2.67837842	2.67746523	2.01602248	2.05689632	2.84701222	2.21539679	1.87906598	2.45116104	2.56488816	2.35965762
5.33468955	5.37981805	4.75881505	4.93943908	5.05280268	5.14397647	4.80232067	5.01605013	5.01565505	4.88711327
0.05098669	0.03650437	0.36997047	0.32163674	0.02859466	0.11384417	0.00913972	0.21022929	0.02601173	0.17539566
1.8826336	3.16824662	2.44784389	2.41121031	2.88721232	2.35526277	1.13621061	2.41047935	2.29211984	1.87532204
2.18600276	2.1901984	1.99523755	1.47902715	1.83098292	2.86006552	1.47282504	1.52866773	2.56039028	1.68099153
2.83997154	3.63602635	3.44934692	3.16821533	3.38485868	3.38502138	2.46989681	3.62085851	3.04888272	2.80136162
1.72601831	2.17484637	1.91882784	1.66848033	2.20036978	1.70227958	1.215756	1.88854197	1.24392447	1.46264841
2.38452947	4.04425411	1.76064685	2.23499291	2.68149104	3.30289552	1.94250677	1.61359641	3.09973405	2.8174217
1.51464388	3.15116456	3.181861	1.04055672	2.25081492	2.68572076	2.05506524	1.30788465	2.58671635	1.22323582
1.94284231	2.14721525	1.27036662	1.31383717	1.50032351	2.73696092	2.18374003	1.61499326	2.52527027	1.4017914
3.39376235	4.23325217	4.22319965	3.6772392	3.95082195	4.38086146	3.43107057	4.15665847	3.96841408	3.68238249
0.84436504	0.64080008	1.56697994	1.00183133	0.83676545	1.52840843	0.40675453	1.42810831	0.67068528	1.53972373
4.59057792	0.06667608	3.78171865	1.8012029	0.02434722	0.87235781	0.01454263	0.63758418	0.7034502	0.00760328
0.89729662	0.39745276	2.08411557	1.21621638	0.71457716	0.74041029	0.27031402	1.16259441	0.4144365	1.90945996
3.2899588	2.99513196	3.47256106	3.27201491	4.66130346	2.83541876	2.84311698	3.25099455	3.33972086	2.86402276
3.51683255	4.47558294	3.47640803	3.88600899	3.84016854	3.46867654	3.30960614	3.65784872	3.9670432	3.36872188
2.22860796	3.08926775	2.7952227	3.09896354	2.71128469	3.6868758	1.15578475	1.93845862	2.65678622	1.99145956
1.02794891	0.79690409	0.6586944	0.66615111	0.54472116	0.83232677	0.86538131	0.71002814	1.00188048	0.74693426
1.23612891	1.8084582	2.84801533	1.46606605	2.2749234	1.68465595	1.04877156	2.14164873	1.34099966	1.4634297
1.02493355	1.82773816	1.45029916	0.88305785	2.48838057	2.33019191	1.30041952	1.17600559	1.29220837	0.99193447
4.88496853	5.27707155	4.84628784	4.91064048	5.11566316	5.73040987	5.11133891	4.93332585	5.09691042	5.01007168
4.1842446	4.37659957	4.07439353	4.32049619	4.19376216	4.40723756	4.03332123	3.82620114	3.99861423	4.23411436
5.35886538	5.2830522	5.12926893	5.35549861	5.49949635	4.67609762	6.01311375	4.93656343	5.17738444	5.53273905
3.78045529	3.92006998	4.05337534	3.81455981	3.69763354	4.26533978	4.1911329	3.4137441	3.82571326	3.88908089
3.87109868	4.22961872	4.16558198	3.90169375	4.03123969	4.66004219	3.89192751	3.78606349	3.81131461	3.96410278
1.42361114	1.31441255	0.81841287	1.15169274	1.22319035	0.98348064	1.17844423	1.24961105	1.54296212	1.12562869
6.70957588	6.60036108	6.66046934	6.45305336	6.82838791	7.21044893	6.98339937	6.11007594	7.06746875	7.80636172
4.24484903	4.47592112	4.63322222	3.88526972	4.09247524	4.38425475	4.32245678	3.87411091	4.86824959	4.65197844
3.66440685	4.16165893	3.41540791	3.98899424	3.40044171	3.60354056	3.54363116	3.60294245	3.75363687	3.51893583
4.26182238	4.57267534	2.98516194	3.55125597	3.71428594	3.07140656	3.13253597	3.12454485	3.89937458	3.11118066
2.12419076	1.02747369	1.8424029	2.15903401	1.20558942	1.60201718	1.58995921	2.34402058	0.95688384	1.52582236
1.33203198	3.2471807	1.46254066	1.34361597	1.54675434	1.35127313	0.42277847	1.01843174	1.40979068	2.11761643
0.24206969	0.04893476	0.10287118	0.26260712	0.0543694	0.09853365	0.02182218	0.22305386	0.01392062	0.23900971
1.2526315	2.63205699	1.47218892	0.87923498	1.59589949	1.93959883	1.35796388	1.21682666	2.41227545	1.25835076
4.37847146	5.51988047	5.16348583	4.65712822	5.4251932	4.88981169	4.74597253	4.52405823	5.4185915	4.70606053
4.83953429	0.00805636	2.31120481	0.51246513	0.07020879	3.62471279	4.3298948	0.84484206	0.44901124	0.66440242
4.98581119	5.20634227	7.06585978	6.5324323	6.63869499	5.57703739	7.28516639	6.86967256	5.02474666	6.80509203
3.76411529	4.14294031	4.88713978	4.39361268	4.93864912	4.54231933	4.72589671	4.79788118	3.75889773	4.6890463
3.03218418	4.7815932	2.60768832	3.44844667	2.83855357	3.38210544	3.29723506	2.26740151	4.76595028	2.78880741
4.18637626	4.36981088	4.4231503	3.65926511	3.93377199	4.92608104	4.1106248	3.15145733	5.00967699	4.70904182
1.15892828	0.83333122	1.6219886	2.57574608	1.3006539	1.16553754	1.07132617	2.38465859	0.92368124	1.04938194
3.37502039	2.43925211	2.56622271	3.80141923	1.92138253	1.64308675	0.08423835	4.88951131	1.25380313	0.7852501
5.57113485	2.29452594	5.23384127	6.24432522	1.20220489	3.65778741	1.4695354	5.67528558	0.84315169	5.05207124
0.38864063	0.0740622	0.08538496	0.0975073	0.06533938	0.12310883	0.41494998	0.09062963	0.16147399	0.03417963
0.0144397	0.48450757	1.78643416	2.11817349	0.11009486	0.92369591	0.01085533	1.24855035	0.18971611	0.91709153
0.2552737	0.41745458	0.22545365	0.3225723	0.50085201	0.45391518	0.12831091	0.32780419	0.43832449	0.2337153
1.07523702	0.98314242	0.87134869	1.03500107	0.85192025	1.6553458	0.79043083	1.17089663	0.88914325	2.41457716
1.58096768	1.76751856	2.60689827	2.69110342	2.66174906	1.69021423	1.35285713	3.65917895	2.39964696	2.15374226
1.04177352	0.60147211	1.06203083	1.40435849	1.07249447	0.78859387	0.48823613	1.54099967	0.56025451	1.19964846
1.57449928	1.3369493	1.61352826	1.80376894	1.77438488	1.40089884	1.16919309	1.84472836	1.55189111	1.36619709
1.17848977	0.28241063	1.33676834	0.54251469	1.31630727	1.54184244	0.96243996	0.39682337	0.61956737	3.22730152
2.77110999	3.34677809	3.22421391	3.41245376	3.22833988	2.4306211	2.82205639	3.62546429	2.46317255	2.34533115
2.7652738	1.90281222	3.18947414	3.50136328	3.43097274	2.51785296	2.22435513	3.65268683	1.6681269	3.04531777
1.2999793	3.08746365	3.08632274	3.17619516	2.03490894	1.80196633	1.3801226	3.52187286	2.61961827	2.0078856

2.9895716	0.01727478	0.04031042	0.41769033	0.92139222	0.91721245	3.6128258	0.05250723	0.07609961	0.02819662
0.3496989	0.06008284	0.43052778	0.34537823	0.60308922	0.26422249	0.55159648	1.45205502	0.10347946	0.32823769
2.43722438	1.34061961	2.35063507	2.74976195	1.76986551	1.20781989	1.03584048	2.68792243	1.30296097	1.11107472
2.87662729	2.99211272	3.20601401	3.48795781	3.78230396	3.10364626	2.65751875	3.7607339	2.5492482	2.54722617
0.71169129	0.54327279	1.55223411	1.61292204	1.39628223	0.98512609	0.90907809	2.1564628	0.65879205	0.82716454
1.88303877	3.31976581	2.64294704	1.84368263	2.64013757	3.67143309	1.92682487	2.05495677	2.8077338	1.25083674
4.94336021	5.42440802	5.01657385	4.57886348	5.14611208	5.33242996	4.69906295	4.8549219	4.62096227	4.29497121
3.52207503	4.28374373	3.57069617	3.16246604	3.89975042	3.9157232	3.76539442	3.41563269	4.31544626	3.25325202
1.72722087	2.54081278	2.24436977	1.56462743	1.9967549	2.45268715	1.69335666	1.74399093	2.02774614	2.1195058
2.74261712	2.84759282	3.4767107	3.23273675	3.11505851	3.47844737	2.6040291	3.1592841	2.96822951	2.74140376
3.93772437	4.59099917	4.25176116	3.73115599	5.04150133	4.78817227	3.98232371	3.96244795	3.92408326	3.8864381
3.66013855	4.13691401	3.10033182	3.32892099	4.00982381	3.71105872	4.17411559	3.48778402	3.91497381	3.18880748
0.52853889	0.33030359	1.98285913	1.13063293	2.41484258	0.94184913	0.26082825	0.94304157	0.41543912	1.01536598
6.6034015	5.70622033	6.58266416	5.87537598	6.46326393	6.23085343	7.15797011	5.5207754	6.1976021	6.58823729
2.20727368	3.21883964	3.51829491	2.69726682	2.01153186	3.65861287	2.93380484	1.99408346	1.14780611	2.46910737
3.90982891	4.09269116	4.20452627	4.47638631	4.62026226	4.30171579	3.76344788	4.76819467	4.26022574	3.6175306
0.21046083	0.10444143	0.14420368	0.18527211	0.49846829	0.10853135	0.06242798	0.16762653	0.05312467	0.14079125
2.44759643	1.05686576	0.83911089	1.51161089	0.82172442	2.95947322	2.78802015	4.96234883	0.14925154	1.34021822
3.21017794	1.85609726	1.18354818	2.40177393	2.4505799	2.82511873	1.35865435	2.09531605	2.33856621	0.71209866
2.3626925	2.67558191	2.18681454	2.28032972	2.38429059	2.4440711	2.64285179	2.72727771	2.81290153	2.48722462
2.03551988	2.40012954	2.7123471	2.41404004	2.21515054	2.4016741	1.83251951	2.2950475	1.80775159	2.56722044
3.66871802	3.41941627	2.57599959	2.9425714	2.97731656	2.84806876	3.91204268	3.06791453	3.96289961	3.10268866
4.35396192	4.52003134	3.51201737	4.33739526	3.89594956	3.42400693	3.31967418	4.16808594	4.31151011	3.51408893
3.75218366	2.19271847	3.69333817	3.30792592	2.90421352	3.38627574	3.11369464	2.72119123	3.0171616	3.02756734
3.34361509	3.62984946	3.68405328	3.19057331	3.06981564	3.35484524	3.63060999	2.76553645	3.860744	3.34404374
2.41892013	3.2337724	2.48357536	1.80801955	2.40003814	2.85558151	2.62890284	1.91271614	2.50772736	2.17857984
1.03809574	1.8753013	1.82125835	1.25409365	1.44461305	1.71442164	1.10493561	1.35291391	1.73757342	1.11823102
0.88303591	0.05365983	0.18589987	0.58302279	0.23876282	2.92795206	2.64379003	0.30785843	0.04834545	0.47628395
4.65849406	4.84765922	4.82366734	4.74948749	4.24666354	4.05612778	4.29929972	4.90866067	3.67744707	3.28709082
3.28488122	1.99642358	3.27884968	5.3106458	6.16866683	5.21740811	3.43654333	4.86977488	2.3315515	2.80807533
4.5775917	4.68413663	5.42199207	4.63704159	5.04499524	5.09064033	4.36458866	4.95333566	4.72354851	4.82244276
2.95285214	3.85916949	3.565792	2.08317684	3.71642903	4.02864908	3.20605086	2.98776514	3.38740157	3.7056943
0.81383737	3.15298732	2.83590753	0.58861757	3.04889384	1.56845625	1.7473727	1.65396202	1.85149168	1.31356447
3.29669237	4.88924455	4.87385949	3.16916871	4.68810204	4.52020463	3.58917664	3.5637554	3.83383429	3.51890434
4.2326097	3.98578995	4.59819624	6.3539764	5.24508882	5.23616694	3.00743077	5.49872911	3.02411888	4.68613842
2.73944274	3.38302256	3.25515533	2.32123614	2.80551469	3.00866027	3.02131797	2.45301767	3.56979898	3.08691807
3.46597299	0.79382043	2.33952301	1.1571377	1.92348793	2.35580444	4.11436109	1.25948201	1.40042469	2.44199473
3.21131928	4.2732673	3.82603428	2.43422971	3.76075337	4.18845317	3.90491175	3.61711402	4.44148419	2.89723095
6.3225511	6.33539647	6.47871832	4.43524667	5.41998974	7.05955348	6.87437288	5.42833545	8.15203898	5.66035131
2.17517131	2.78420212	3.00542084	2.78752499	2.61288271	2.4870534	2.01274169	3.06961651	2.38388288	2.36781715
1.42910302	2.02020091	1.69881593	1.58885401	1.75622333	1.79349608	1.10553451	1.63264457	1.53181608	1.36372385
2.86083895	3.05329854	2.38520416	2.472128	2.92916179	3.36209813	2.27972695	2.90736242	3.34469453	2.43148947
0.27862583	0.82112679	0.27903233	0.05900524	0	0.46332716	0.0135112	0.04193362	0.07570007	0
1.87360687	2.90329606	2.28342367	2.64818132	3.05517003	2.35646308	1.93851236	2.13368352	2.17505355	2.05545353
2.60556171	2.38995857	3.04102362	2.93670469	2.04060894	2.18844396	3.15774569	2.60877393	1.30618836	2.72473438
1.99653433	2.40603409	3.69508693	1.52864357	3.20294019	3.39642324	2.24814344	1.49260897	1.67581275	3.1858862
2.80211602	3.01244162	3.27195234	2.72265634	3.67325418	3.2835734	3.3837894	2.57454074	2.65146292	3.64873099
0.99018519	1.28233445	0.86551742	1.70453202	2.08632949	0.77076199	0.88165946	1.97487404	0.16823619	1.5787177
1.15690143	1.6294605	1.45945743	1.13168744	1.55286011	2.4918597	0.57080957	1.36697981	2.17774342	1.78257709
1.61931081	1.37721551	1.65775131	1.92245137	2.10899516	1.86402739	0.75381427	2.08429965	1.55619121	1.46718341
3.12092262	3.18840513	3.45251347	3.43085686	3.47376682	4.20699626	2.37738048	3.31307057	3.07011852	3.43614468
0	0.00552563	0.13318639	0.04075603	0.09538026	0.02569559	0	0.34394058	0.02468115	0.05342346
2.52013391	3.08276888	5.78494368	4.57100289	3.20403408	5.61231901	3.02089837	3.20617046	4.54588492	6.46440461
2.31256263	3.35207512	3.18157895	2.28158919	3.81327694	3.13141792	3.26325618	2.28680117	2.85984412	3.06399085
0.07224016	0.17748508	0.41645445	0.2660073	0.31636448	0.39777064	1.14873799	0.46445661	0.27527797	0.16433695
6.16632955	1.80314416	2.29134575	3.51057121	1.76929034	4.84146402	4.66512514	2.90756092	2.4427205	4.74031241
4.0135678	9.43783897	2.89651379	7.01932887	6.90363118	5.51968762	3.2400078	6.44097646	9.21658855	0.1503498
0.58808738	3.16983051	2.55629114	5.27211198	2.45370734	5.28696676	1.63713902	1.56418996	0.20101257	7.63116431
4.17771236	1.13294338	4.33763087	5.74402349	6.7429734	2.72119935	3.34029626	2.78029348	3.81552795	3.39532477

7.93841325	1.64405211	9.92979042	6.70008103	6.07549559	8.37878897	9.47658649	5.9579173	1.2420064	6.98923255
6.64565968	6.15743629	8.83186478	5.66466605	5.78190774	7.23810474	7.50547624	6.00732613	6.53640607	7.70452128
5.05313684	2.87439344	2.82741635	4.52677755	2.7351267	2.34885635	1.79872993	4.95044092	3.06114822	2.41695241
0.14836175	1.89646606	0.04394701	0.38876444	0.95124316	2.48485293	0.03858746	1.86155564	0.14204678	0.01353472
3.28466913	3.39492543	3.64367093	3.1205887	3.1506702	2.86275768	3.08281978	3.33844243	3.0531721	2.76352214
0.72398239	0.46548442	0.55119073	0.55114204	0.42494695	0.83472331	0.62640851	0.63587638	1.46678575	0.46763582
2.59939882	2.62910899	1.22973924	2.57376851	1.45840679	1.14278101	0.2750327	1.84130101	0.68464727	0.19667555
4.80866271	3.17278361	4.58187634	5.2237103	5.00271097	3.64656654	4.98297553	5.58918114	2.94792308	3.73778285
0.23915636	0.69210592	0.18988963	0.18248752	0.93639962	1.08703572	0.2725019	0.2130356	0.6270597	0.22210545
4.59246008	3.06566965	3.57962998	4.584645	3.35033016	2.87392051	2.37477539	5.18639661	3.70317482	2.83125057
2.07500027	1.98166146	1.98206883	2.16915248	2.46520496	2.41324604	1.98069171	2.67125253	1.99461301	1.89759931
0.02437968	0.0062548	0.05578041	0.05058694	0.00309789	0.02184951	0.00368742	0.03430716	0	0.0115082
2.1924199	0.99645722	0.72704453	0.50388737	1.08618598	0.9002952	0.46881319	0.98330264	1.42516314	0.32977041
4.96297761	2.20077741	2.61716222	3.32903015	1.68244447	1.5187729	1.79673546	4.06472459	2.02387271	1.80408216
1.66577762	1.41470773	1.22875812	1.08378568	1.88329534	1.8660684	1.50588069	0.95753885	1.16398764	0.58403138
0.18306307	0.13357663	0.08374371	0.12238196	0.03796376	0.03595943	0	0.1368485	0.06828137	0.0095231
0.59746604	0.24185958	0.25297522	0.33479775	0.13464526	0.12304673	0.17046446	1.6020869	0.04850808	0.10401551
0.21054382	0.2555998	0.91020727	0.60503956	1.14502052	0.89122715	1.4696859	0.65312305	0.28438374	0.77784119
1.07985658	1.62982	1.53441169	0.83928959	1.75628058	1.86047996	1.14735343	0.81890412	1.20327709	0.60806005
1.71639543	0.48854982	0.19969593	0.27302352	0.65552047	0.36778068	0.14999401	0.44120102	0.48623201	0.10593851
0.18045135	0.4509982	0.38488193	0.20663386	0.50603834	0.32025768	0.08020155	0.5994608	0.36332587	0.35560723
7.65738647	7.45349808	8.16748925	7.34546875	7.39919224	7.77807073	8.58753138	7.14690546	7.9200136	7.75140628
3.13328201	2.04217695	2.41350352	2.80815244	2.29116272	2.17429752	1.38102868	3.07871647	1.85549162	1.29878139
2.53182339	1.64643583	2.25749812	2.55436091	3.09349687	2.03726894	4.35507914	3.15863308	2.83086116	3.95264869
4.45928588	4.61782583	5.97535716	6.06269459	5.92913574	5.37146514	4.4818861	5.95481053	4.57956221	4.72237272
1.06334485	0.81010181	2.23576429	3.36300729	0.55092718	1.17258728	0.79644937	1.4279675	5.7952386	0.50629558
3.7808085	4.58417656	4.22837461	3.64189849	3.74690008	4.18122945	3.21007573	3.37867768	5.00345689	3.18348047
1.82403063	2.30221802	2.6413519	2.46782164	2.48532585	2.75648891	1.98719428	2.55000438	2.43319386	2.24409884
3.17346946	4.05849001	4.64144745	3.55701918	4.36635897	4.33967937	3.94152088	4.17540335	4.45447844	4.84480472
3.0829532	2.46265462	4.00108422	4.84950415	2.7762039	2.97681832	2.48946979	4.09039555	2.02743916	3.0441006
1.21346007	1.22220439	1.75694586	2.02839175	0.97520005	1.52000782	0.5527268	1.6498384	0.95402815	1.07505834
3.02513461	3.19016677	3.39963073	2.07890341	3.09143471	2.84399453	2.41113148	2.95678697	3.5400811	2.89207157
3.23523621	3.74780227	3.1561245	2.89790762	4.11498094	3.66838298	3.04865783	3.68447271	3.65573432	2.8298265
1.74876261	1.79561541	1.39721567	1.39246951	1.49933576	1.71815382	1.36411673	1.52326938	1.64752147	1.48751724
1.73027983	1.93152318	2.40534751	1.81436207	2.57746476	1.93908319	1.32779911	2.21132034	2.29621682	1.19490421
2.60481739	1.69373074	2.65660226	3.65076946	1.90190988	2.73859163	2.55131869	3.69524318	1.22378483	1.9276077
2.82858865	1.99038334	3.12847829	2.48652911	2.27344898	3.20582502	3.50917568	2.24212793	2.73209281	2.68066091
1.81990002	1.68375205	1.59936449	1.85815491	1.82212729	1.54307825	1.28847107	1.54458482	1.522769	2.74033648
3.94294609	4.43360972	3.36634369	3.32468387	4.4393548	4.72266751	4.60357653	3.94129515	4.94871516	3.5636548
1.74551076	1.47562329	1.22521572	1.10389164	1.5786307	0.91211036	1.1152022	1.57804925	1.65788428	1.25356394
0.37865229	0.21294523	0.05820391	0.09410585	0.16233577	0.15801456	0.07820459	0.1622679	0.26658186	0.03016599
3.95776194	5.07016737	4.11043653	4.34303354	4.05872043	4.24199076	3.8555409	3.43912803	4.42318234	3.78214701
1.84852317	2.3166386	2.54016382	2.2028399	3.91826309	2.13582955	1.3795114	2.16207692	2.4167032	1.76956015
2.33734325	2.9305604	1.84793767	2.24617854	2.47267963	2.67327347	2.07465888	2.34299355	2.32953663	2.23542702
2.2070293	3.22147642	2.83402896	3.24896703	3.04102037	3.13282493	2.53189517	2.69875868	2.75403235	3.15312495
4.84782882	4.92586125	4.90505668	4.46717498	4.32843134	4.26994285	5.33014022	4.26135575	4.90629074	4.4556773
5.21818707	5.76082858	5.47761709	5.61062261	5.78640265	5.52945591	5.12627333	5.07199524	5.20897941	5.25298802
3.94823021	4.39825636	4.10461105	4.30043358	4.44100203	3.90451582	3.88622759	3.93882003	3.938192	4.73650226
2.70283074	3.41781599	2.71045087	2.37399994	3.48033564	3.43370204	2.69438907	2.79754209	3.2571919	2.53516349
4.24402053	1.37733786	4.78304648	3.07049313	0.17124257	4.00544277	4.65208645	0.20118178	0.55441873	6.03337924
1.21101619	2.07368719	1.88901258	0.80208378	1.90462274	1.79631558	1.74886245	1.12651435	2.09981109	0.94177712
5.26326022	5.64331296	6.01436619	5.82942186	5.70298667	5.10238359	5.34336314	5.56883356	5.0922254	5.93041556
0.40865308	0.15682778	1.3050596	0.98245635	0.9080729	0.3141403	0.63497578	1.98538369	0.46154206	0.34853023
3.98523089	3.37370154	2.7596927	4.17065367	3.24672211	2.98629905	1.76986969	4.10944159	3.1974307	2.65658364
6.00044827	4.90721017	6.74581648	5.87473092	5.57620653	6.83508873	6.76048769	6.58519121	5.92882041	6.64650508
4.56323668	5.38168722	5.51653269	4.73350091	5.43487725	5.20464034	4.92956801	4.81720593	4.51400397	4.64000053
1.1284391	1.99268811	1.35356338	1.62379854	1.79751552	1.68026178	1.2729705	1.87644987	2.00817674	1.46433499
4.69088766	4.98723287	4.05923781	3.9992335	4.65202089	5.21460714	4.71878784	4.76971287	5.34736128	4.1445242
1.51117231	0.97745743	0.47585199	0.91015102	0.69520141	0.83542169	1.1811647	0.63731522	1.15592513	2.28237419

3.32367001	5.67665269	4.64666367	3.17234888	3.66387304	4.33622498	4.17040655	3.52445354	4.30408885	4.87677034
4.77240921	5.10380415	1.30126343	4.46205226	2.98131941	0.93464369	3.6225874	3.52332171	0.93449051	3.35490276
1.06521369	0.8940354	0.85125053	0.86435163	0.78686347	1.12126072	1.13983793	0.6272377	1.28365485	0.95465188
1.25216581	1.69584664	1.03043542	0.95936975	1.26197333	1.33200217	1.38639718	1.01383861	1.98077002	1.21630724
0.09490085	0.08497672	0.14385052	0.12512403	0.06057359	0.04326199	0.01465545	0.19410489	0.02784193	0.20051507
4.22777889	4.21740704	3.58188299	4.0448042	3.48632234	3.73177507	3.86044578	3.89408936	3.65743412	4.20279836
2.20648531	2.01202492	1.81429769	2.1880156	2.81277266	2.29518893	1.70808242	2.23280035	2.02223895	0.88737864
5.73247028	5.90006273	5.60204654	5.3747506	5.21629425	5.29097616	5.77393099	4.65188565	6.4277875	5.79296782
1.69716299	2.32544491	3.46751645	1.79984163	3.01562102	2.78024947	2.02826168	1.92570981	1.99405671	1.27571509
2.26609934	2.36119533	1.79683311	1.88148964	2.51403481	2.26756514	2.23509052	2.14453891	2.86305705	1.79499399
2.87578067	3.77294525	1.96315386	2.54398837	3.8762462	3.01880289	2.92698168	3.38761964	2.94522759	3.1082151
3.55474894	4.13777854	3.37248219	3.09077793	2.78676816	3.29293798	2.92798131	3.20678056	3.83910592	1.56447113
3.385138	4.45474308	3.2616077	3.10169998	3.25971519	3.26995212	3.62018003	3.16528046	3.90426147	4.11913426
1.88732601	2.06134189	1.87824604	1.46752692	2.30280221	2.25808963	1.47736242	1.61470368	1.62869987	1.87063564
0.16292475	0.10846706	0.39178473	0.44173527	0.18738394	0.29240317	0.06104415	0.46323269	0.07143396	0.14882534
7.15481619	7.15216674	6.90948227	6.74627665	7.02729231	6.41647812	6.75403385	6.62535984	7.11514411	7.41902783
3.84140154	4.23477467	3.12695494	3.24900484	3.59065505	4.59223238	4.48228832	3.05307586	4.49845883	4.18481829
1.39393855	1.93684836	1.76460261	1.20858787	1.75379922	1.68212483	1.40247871	1.2252798	1.83868399	1.50589025
3.94839988	1.58056818	2.87005951	2.68942837	2.83363573	3.62563948	2.31816906	2.81645534	0.32038568	2.38221058
3.90969292	4.57064947	3.88315986	3.91619931	3.56439297	3.8640219	4.24312895	3.63996975	4.30608693	3.68765467
0.33684329	0.45830511	0.92857887	0.86262282	0.63097473	0.52383152	0.45642784	0.65198013	0.44610802	0.50577224
2.60652707	1.95048987	2.9905395	2.59090852	2.83874541	3.35249679	3.19854956	2.66288604	2.50800815	2.23977893
2.04129778	3.04412369	3.49194651	2.56005965	3.50610276	2.6490111	3.07439594	2.56302307	3.00971156	3.60711079
4.32190639	5.23593222	6.64427564	9.07890565	3.74055039	7.36154196	4.21274177	7.47883895	3.50543467	7.78033542
5.62412614	5.3034374	6.34233735	5.70583222	6.25345097	5.4203244	5.76335529	6.32983443	5.40796208	6.07730709
5.34076298	5.23894523	5.64187538	6.19658499	5.35821635	5.78789558	4.81547223	6.19830029	5.00300588	4.33904351
0.1000276	0.12908358	0.88199996	0.92893762	0.82184827	2.0677796	0.04865951	1.96319987	0.0529864	1.85490919
1.05799611	1.63835403	2.03873812	1.51276385	1.43401188	1.24250387	0.80565368	1.7148015	1.53583771	1.16484998
3.57460851	4.34394298	3.75391068	3.79051534	5.07175244	4.31506002	3.56412079	3.52506111	3.48892606	2.97850112
3.27113303	3.62404799	4.69473474	2.79953463	3.58631977	4.44370769	4.6230753	2.47348489	3.70255385	2.86146725
3.82571235	4.90899916	4.09414488	3.5951317	4.80065306	4.89098496	3.57888911	3.93020572	4.02445854	3.85213875
2.41689215	3.08477707	2.63744859	2.06827272	2.7830318	3.00798443	2.32884001	2.36650828	2.86417848	2.18314071
4.18781614	4.7346782	4.69410824	3.83636407	5.58297735	5.5417007	4.90696733	4.1418683	4.47283681	4.58132517
2.49323222	3.11791332	2.03296066	2.14892291	2.67551085	2.43247235	2.20344857	2.17626522	2.68545858	2.32707643
3.20517141	3.80809637	3.49345679	3.35117716	4.24557915	3.13730269	3.0948558	3.38908811	3.55507552	3.03627842
1.90954369	2.18483271	2.34941092	2.02432798	2.21105024	2.31110641	1.74925155	2.30062622	2.18243734	2.26684352
2.47753409	3.00284073	2.76999383	2.20326659	2.63656679	2.88190475	2.56494041	2.43722872	3.06164531	1.98429905
2.79881059	2.70445338	2.64813253	2.19100118	3.21208496	2.53432998	2.34666313	2.31778367	2.82955671	2.11846072
3.19752123	3.56485941	2.34543924	2.43529026	5.09870434	2.65635075	2.68690806	2.29012581	3.74186427	2.68578423
3.89969134	3.85511673	3.62518725	3.3759955	3.2596316	3.76303507	3.31037925	3.38190094	3.71259887	3.51068762
3.26574447	4.02555982	3.32735844	3.73176095	3.76888146	6.00017145	3.41436062	3.7692306	3.64167071	3.60459811
5.22131361	4.71568435	6.03421395	5.11222889	4.92032735	4.80415965	5.39085307	4.27454643	4.26953588	5.27713558
2.69436738	3.04466414	2.02201817	2.680576	2.89241899	2.49257713	2.91730749	2.22978182	3.67907431	2.4717011
6.11624642	5.79524699	5.86811896	6.23037647	5.80295637	6.45527599	5.55352786	5.63993982	5.71519853	5.49074401
2.26456047	1.27025022	2.22428741	2.66905515	2.03691155	2.25684498	2.0811929	2.48463608	1.56701524	1.61960952
2.23808233	2.52365422	2.29175098	2.01100378	2.84717081	2.6408673	1.25082056	2.17835666	1.93190064	1.62489695
1.45132271	0.02803951	1.22167358	1.94948767	2.16430467	1.06180904	0.88573695	0.97542148	0.06229164	2.44079974
0.10748326	0.33408749	0.09895573	0.13047478	0.24023373	0.36578134	0.04709958	0.22531236	0.2700353	0.15381338
2.64941279	0.40987938	3.35564513	1.40155565	0.49200498	2.82692367	0.03186207	3.54956658	0.07973527	2.43779133
0.44897092	1.14825773	0.56092554	0.64479616	0.84453281	0.41478694	0.31695648	0.76460599	0.43258944	0.38752962
1.07611762	0.80157131	1.07136217	1.34596683	0.52602066	0.41008429	0.18410776	1.0340064	0.41371766	0.58807663
1.51381681	1.89130229	2.97621889	3.89778972	1.64762143	1.94270308	1.0430357	3.27338924	1.59885433	2.51826677
1.2006076	0.12383799	2.42937969	1.24912678	0.56548147	3.55377436	0.36656022	0.26784881	0.03151257	0.2925543
1.68911118	2.13827513	1.64639434	0.87286529	2.25744164	1.56807691	0.74627936	1.57892797	1.64784366	1.23629804
2.27314814	3.11723497	2.24957091	2.10243476	2.44578358	2.4492792	2.40534573	2.68785984	2.80907822	2.28947739
1.75459338	2.09207957	1.96216209	1.50259897	2.40603031	2.02797951	1.61755044	1.61378403	2.17194923	2.02511615
5.52432923	4.52965077	4.00568505	4.58231523	3.79521335	6.50870717	5.85186752	4.03346665	5.33675157	4.88687593
4.06504539	5.15526055	2.98460642	4.65298449	4.11134074	4.35971504	4.84111313	3.66417155	4.55366838	4.06349202
2.49000411	2.41159819	2.31770432	2.18842114	3.43403556	2.99266362	2.35025822	2.33398032	2.91268726	2.3961898

3.98194991	3.69710266	3.30281564	3.09742657	4.14784182	4.66744135	4.32920455	3.73372457	4.9920401	3.9627126
2.29697226	3.34566173	2.39995346	2.26223972	3.08599064	3.00878762	2.53014028	2.62334996	3.14186422	2.49164612
4.01401129	4.56507606	4.11978879	3.99811068	4.33666491	5.93416447	4.1881976	4.11791464	4.50104331	4.26306048
1.04929182	0.69111915	2.69300949	1.96580376	2.32882877	2.33232599	1.76845435	1.86501847	1.52531739	2.82216891
1.2570584	2.2633383	1.71417601	1.55694831	1.69996623	2.01434833	1.41156489	1.60747659	1.51915593	1.57344877
1.71399596	2.18932945	1.6644212	1.70406887	2.82776166	2.53319717	1.70984189	1.8705491	2.03593478	1.81520053
2.45066179	2.44037107	2.41160459	2.13874164	2.95798962	3.0339223	2.41923662	2.65559588	2.19350193	3.74501143
3.18073791	4.02737834	3.31288521	3.24774924	3.95344589	4.19659244	2.93145511	3.46317803	3.14076661	3.13329865
1.93178663	2.9318415	3.23701363	2.11400566	3.04631638	3.06841939	2.10360979	2.1798105	2.11414945	3.02392981
1.60433026	1.97363324	2.1551124	1.72720442	2.3588379	2.08050297	1.48114071	2.03727133	1.76028088	3.23144958
2.46970007	3.09892563	3.22122996	2.70434499	3.64101667	3.38785355	2.53905431	2.85321056	2.63857722	3.00020098
1.93264587	2.40958098	1.41369194	1.83786831	2.20383242	2.6892927	1.89481365	2.1953227	2.19742317	2.02787663
2.29754905	3.12895169	2.65103337	2.33805107	2.62946111	2.90967851	2.46928848	2.37226982	3.01374675	2.68753648
0.53029567	2.90989456	6.17137778	4.3148046	5.04334743	4.15405122	5.97872415	4.79841605	3.50261459	4.33140146
0.09198414	1.50417106	3.18907676	1.82678798	3.56324867	3.41167546	4.53157364	2.66977181	1.64945045	3.19048948
1.91522405	2.88514945	6.26797153	4.22442595	4.44302087	5.22390395	5.8722076	5.42300637	3.08239569	4.58952275
0.66104793	0.60498143	1.13887018	0.87986503	0.57113889	0.53872151	0.75708359	1.17151467	0.16314655	0.34516415
5.43018385	4.23725866	5.27754853	6.04277289	5.55189295	5.42923313	4.65424542	6.65648134	3.10807517	4.55905259
2.69191835	2.2418725	3.55031568	3.91748413	3.96450149	2.47919783	2.8156237	4.24390339	2.21004051	2.69329495
2.84947586	0.99136454	5.2979441	0.61942922	0.27195986	3.01895689	4.41200671	1.85345072	1.03235851	2.37386343
4.89132338	4.34986349	5.91051885	5.99997733	5.98382263	4.68894574	4.2652433	6.84256194	4.45719097	5.73304813
8.1816531	7.916174	10.4120284	10.1503385	10.3096763	9.15720824	10.6704979	11.2803882	7.14703392	9.48362753
7.24250171	6.74890577	9.25811832	8.72484109	8.7036439	8.01681665	8.99948537	9.55352087	5.98726363	8.00989076
0	0.00874143	0.00176673	0.00261633	0.00346613	0.00205153	0.00412562	0.05089186	0	0.00859387
0.55593852	0.09724466	0.21597145	0.88745409	0.41552792	0.30833932	0.03167803	1.02138734	0.07735233	0.13391059
0.19450394	5.10597839	2.29181403	0.38716608	0.70077021	0.34397162	0.25417684	0.53893506	2.15283302	0.32332105
0.02502635	0.06296664	0.59738965	0.29504638	0.40358495	0.45641368	0.7777004	0.34218886	0.05761775	0.29814246
0.73360418	2.98467363	1.5801178	1.30011356	1.94681373	2.32001618	1.26285094	1.7890831	4.23354658	1.30007674
0.01819445	0.00582326	0.08008802	0.00868744	0.0229117	0.10852937	0.02723635	0.03722022	0.00654439	0.01426978
8.24134514	7.1350595	9.77357631	9.42548136	9.52728238	8.69797771	9.50384899	10.083234	6.65759976	8.7263365
4.85671855	5.97270665	6.87533011	7.03225988	6.71292574	5.78443019	5.00323458	7.70736743	5.78693576	5.97704678
5.33436299	5.97555634	7.02263396	7.44076637	6.94081828	6.02663235	5.43152149	7.89870654	5.81947051	6.31632423
0.76323494	0.29294774	0.26356056	0.4205026	0.39227388	0.14996138	0.01483123	0.11592752	0.19900546	0.08309023
2.19630503	2.20056788	2.6468889	2.21108791	2.1207968	2.26611073	1.90893405	2.27911661	2.07272925	1.65383057
1.94761645	0.93249963	1.104128	2.08236627	1.52309782	0.68632948	0.18670018	0.88259953	0.44430129	0.20048805
0.59468755	0.45034673	1.77169672	2.14013827	0.80295066	1.29242384	0.02571328	1.68237434	1.46678575	1.14400163
0.09442581	0.16256672	1.33574208	1.71328945	0.11811665	0.94636844	0.00730844	0.87636031	0.19583773	0.720798
4.57199532	4.47108289	6.75323888	6.49386187	6.35563573	5.58482233	6.81336015	7.25081229	4.29046302	5.73907575
3.56717215	3.93482261	6.6556518	5.27450531	6.08122406	5.37354195	6.01547808	6.18792431	3.65102233	5.14463869
1.49090606	1.54636226	3.87657229	2.59321985	2.43856651	2.96678942	3.44140072	4.12760311	2.11506715	2.43947255
6.38798639	6.21873039	7.11903125	7.88675476	6.7893385	7.06703103	7.42720851	8.32203372	5.46766559	6.14452583
7.24429935	6.44527105	7.75793535	8.56731519	7.34772729	7.45957545	7.91762498	8.80662484	6.09050985	7.07987115
5.13938809	4.69717148	6.58260814	6.80884224	5.6187514	5.7637136	6.20056018	7.08267148	3.90004992	5.67482007
0.42657785	0.58265442	2.62314187	0.78366213	1.05168991	1.85855992	3.54479943	2.27613234	0.61949628	1.38451966
2.14590713	3.17526591	4.49147265	4.80427505	4.37417094	2.72691711	3.17612612	4.38644756	2.23486173	2.86825124
0.31738691	0.55446829	3.04209965	0.35860356	0.14121852	0.11350475	0.2089179	0.35905004	0.35658259	0.97353567
1.5710894	0.66726022	0.90911611	0.79027299	1.30947793	0.74926032	3.08921756	1.13255189	0.49753239	0.83017828
1.05566108	0.2381405	0.27684777	0.79747821	2.58027491	0.88511984	0.10844147	1.75290938	0.20813016	5.89294997
0.5529419	0.36524074	0.58824417	1.14053522	1.14504529	0.07890224	0.10236649	0.99483874	0.33237398	0.26377158
2.08856218	1.09109125	1.83679408	2.66774503	1.3737202	1.41691091	1.46228851	2.84197815	0.97695524	0.91062474
4.12248683	4.86288472	5.6808115	4.6012899	4.48909875	5.34606583	4.79847947	5.40659161	5.05223952	4.40995397
0.50757923	0.23548707	0.32437253	1.73431994	0.52928573	0.17493759	0.02515475	1.23098302	0.27976455	0.21764636
3.13101094	3.00874393	2.46902127	3.02000188	2.89301802	3.03119464	3.62123398	2.83719836	3.47638626	2.53695663
2.86832404	2.86815672	2.65265	2.66714677	2.6658122	2.94348711	2.52061377	2.52290489	2.54093172	2.76146746
2.27084063	2.74573801	2.90369976	2.30046436	2.9969376	3.05586232	1.94976191	2.52568334	2.52773419	2.94278787
2.3738053	2.14737998	2.11919811	2.18367455	2.35455177	2.30054215	2.05649323	1.6258454	2.49980826	2.53808548
2.77093345	2.57591178	2.89968988	2.60069651	2.98754621	2.91058961	3.40707127	2.86629964	3.45099305	2.83673472
3.230658	3.21646529	3.21043422	3.2336055	3.1091347	3.6923912	2.98784289	2.7420414	3.80682457	3.58930379
4.53148124	5.42536019	5.03385122	4.66927707	4.92747697	5.15056623	4.17402751	4.08272572	4.96283642	5.20245907

2.82302948	2.76668068	2.72057678	2.67599747	2.98031812	2.59934736	2.82051857	2.58010361	2.81866742	2.33004174
0.89828014	1.86948033	4.42728834	3.94861094	3.89814527	2.62098634	1.41801737	4.5886819	1.2350539	3.28823369
4.47548637	4.36394652	4.00931342	4.52629465	3.69867839	4.60802974	5.53052682	4.19620815	4.50934339	4.98123868
4.61300601	4.03996754	4.70494017	3.90388504	4.55177784	3.95045621	5.7468409	4.20972322	3.87064827	4.04431583
4.90046051	5.48296943	5.76826988	5.31109917	6.57299634	5.68206483	4.90852652	5.49442145	4.68128766	5.2579311
4.10883562	5.36069442	5.22261799	4.03464698	5.43541047	4.67398987	4.07399591	4.04209303	3.97745442	4.30206399
3.74086482	4.27900633	4.47392798	3.94045476	5.08247273	4.46580011	3.90663896	3.90265968	3.48485626	4.74635498
6.15463559	6.11764146	6.10509842	5.79666801	5.67110159	6.49928273	6.54646695	5.68767034	6.10770916	5.82445944
5.28072104	6.0679277	6.05778269	5.59496225	6.34752682	5.04339555	5.92515186	5.53038293	5.20337265	6.62583677
2.22680794	3.58253597	2.89452346	1.81631565	2.52230081	2.83182336	1.82367287	2.8347704	2.50787225	3.33353109
4.38071934	3.69083026	4.00980482	4.5090268	3.71472923	4.38707451	4.66947963	4.17194716	4.005156	3.85066574
3.85315045	4.67895473	3.63865796	3.40729868	3.87359295	3.88766768	3.98445822	3.8889692	4.333223	3.98167164
3.13550581	3.38782862	3.24958355	2.95690172	2.82924407	2.93501481	2.89231625	2.806183	2.97340714	2.67517318
2.84120751	3.1823879	2.82260903	2.17006091	2.46510759	3.14980932	2.36430343	1.85774696	3.41686491	2.20526868
4.8860602	5.43700571	5.30278454	5.13272687	4.90064353	5.52553527	5.55023438	5.12005673	4.7659015	5.58307865
3.94801746	4.25448303	4.10856245	4.5836393	4.15193721	4.3610085	4.39605209	4.30657029	4.04272726	5.37033166
2.39054353	2.79343497	2.67358704	2.49064009	2.64241041	2.28448036	2.63503581	2.45469166	2.5569066	2.42693441
3.2164988	3.82162809	3.80198954	3.89857121	3.68138718	3.24760973	3.40612762	3.79457793	3.55443114	3.24394984
5.14956121	5.67100493	4.70320289	5.11520955	5.78318859	5.45502015	5.49785892	4.79527207	5.63115969	5.46016608
2.9874582	1.57963401	2.88331427	3.73571962	2.93866574	2.26704785	3.22023288	3.8806979	1.88972471	2.43325822
1.52177794	1.1381808	0.88430922	1.26698438	1.43142609	1.07885012	0.85918767	1.08669536	1.35250001	1.02941621
2.73523916	3.11588479	3.61803887	2.70760189	2.87527709	2.72858467	2.90357806	2.87973833	2.67523522	2.83271821
2.87197882	3.43251072	2.30568775	2.67099451	2.69536278	2.78760832	3.29922465	2.574942	2.6170267	2.62408044
2.33001924	2.36019634	2.07101617	2.15133247	2.38644245	3.10173424	2.22075751	2.18223251	2.39853992	2.07267018
4.0047029	3.91861722	2.85131935	3.59993519	3.85623189	3.90707417	4.08781482	3.35422138	3.78616959	3.69006034
6.16160856	4.07982625	3.24750366	3.95694665	4.13787648	3.8053914	3.7963879	3.47068835	4.43706995	2.90779311
4.72540033	5.5683946	4.86535518	5.34471646	5.24779831	5.07823863	5.26193105	4.57375168	4.63074504	5.56947976
3.62323689	4.32630581	4.60200766	4.53778441	3.85129409	4.81121635	4.42161542	4.25353184	3.55487157	4.20439053
3.61848835	2.60944658	4.21272784	1.91321852	4.47565722	3.8698222	2.93625646	1.84179052	3.68484464	3.75648438
1.66555649	0.6420202	0.98949253	1.61009664	1.1812269	0.37066806	0.20574603	2.25576857	0.2388156	1.88823308
0.19572465	0.13184836	0.302642	0.48176549	0.18192772	0.44509517	0.09638869	0.54758653	0.10613789	0.34406892
1.95069318	1.21963691	1.60555422	1.09878716	1.56136975	1.75163174	1.58739235	1.44043076	1.2763162	1.56611567
5.92923431	5.5027943	4.87175015	5.05478979	4.89855651	5.74077986	5.86949776	4.98131302	6.17087423	3.42749132
2.97845428	3.23425373	2.31388735	2.26658012	2.17903079	2.89233735	3.03873023	2.80602851	3.29445481	2.46911992
2.32093681	2.65680615	2.38285081	2.31762385	2.21266258	2.60831507	2.34165233	2.01312267	2.81298392	2.15905495
4.68216508	4.2592585	4.20709934	4.47612281	4.27719753	4.24151753	4.6244127	3.96318124	5.09251051	4.42013952
2.95584617	3.79781819	2.53043042	2.54487828	2.98607602	3.42581001	3.34912533	3.01449073	3.59859385	2.97946406
3.6351079	3.15105286	2.84078883	2.62892961	2.71088249	2.9861919	3.55300252	2.95999038	3.55296384	3.38291755
3.59347282	3.75920947	4.16301681	3.04192785	3.63578261	3.23120293	4.34507448	3.49761519	4.12923577	4.40563791
1.28510382	2.05218317	1.23815441	1.06796896	1.43747898	1.39979079	1.49570467	1.16291077	1.90075332	1.52946314
1.86826116	1.6693097	2.38792438	1.51149002	2.76887215	2.62740178	1.74437335	1.61439387	2.32160574	2.18560599
0.97363825	1.03778191	0.92485614	1.01415655	1.17746276	1.2218096	1.24079968	0.91921613	0.94009696	1.14391653
6.56927439	5.82631971	4.71730642	5.5776005	5.12708126	5.42110553	6.8649119	5.42838629	6.68476199	5.5110887
6.91077311	6.59752302	5.98279463	5.92097792	6.00230627	6.49281925	7.26294645	5.55210903	7.33811419	5.96844669
7.26012299	6.69270107	6.13654758	6.28430167	7.0557221	7.18950741	8.48132256	6.11956668	7.36333889	6.74747722
6.94945915	6.51669893	6.22821414	6.48064223	6.26198967	6.52904776	7.88549863	5.78803516	7.33258561	6.49643325
8.28618327	7.47905127	7.69888912	7.42783682	7.71950403	7.77078326	8.80088549	7.35563017	8.51744051	7.71477091
0.09197516	0.05444662	0.22985113	0.08767464	0.09652225	0.08596951	0.50601599	0.25538552	0.15062605	1.39888696
5.46197707	5.71824392	5.10757013	5.12544989	4.02902312	5.29933702	5.40145868	3.87327872	6.16011546	5.48889769
3.43272832	1.99877502	3.3290277	4.52842424	2.85699435	2.63953156	3.55605902	4.4532231	2.99018214	3.3809592
6.11396715	6.13268021	5.74337186	5.3682495	5.41234569	5.09072234	6.7474653	5.2441712	6.37682605	5.53406644
5.49344609	5.00953757	4.60663743	4.72677098	4.40535605	4.90522974	5.63816306	4.64616699	6.10853828	5.35706945
6.67207098	6.59536215	6.07493176	6.13284689	5.61917499	5.87261549	7.0660087	5.8539882	7.31269917	6.10645077
8.65229246	8.75339554	8.07138432	8.05721225	8.07957077	8.12338998	9.25927465	7.76898152	9.11648844	8.09544563
0.75076278	0.56996226	0.42921049	0.66877141	0.11772643	0.85695117	0.70769186	0.65425804	0.84261069	0.14188292
4.42306078	2.06458612	3.87505121	3.34358909	1.97650795	2.29149025	1.43780852	2.92277559	2.19222861	0.40945129
2.66953524	4.13795162	3.58837757	3.31428934	2.97500211	3.68738714	2.59287832	3.03701736	3.80401204	2.43818079
4.29955464	3.31487091	5.78343036	5.39013185	4.14432136	2.330022	2.71527058	5.27849743	2.78880981	2.89833666
1.57669715	1.60880424	2.20432967	1.99909185	2.00773001	1.75068257	1.6851272	1.86477001	1.62725083	1.60057356

1.42596865	1.88690916	2.35052054	2.05419791	1.97248934	1.85029982	1.59199516	2.22430747	1.72398272	2.29229778
1.81024031	1.66961997	2.92608822	3.50446438	1.70840519	2.14244859	0.55087977	2.86845544	1.00300588	1.96960436
2.2017925	2.46270361	4.24297683	2.03183079	1.84947324	1.51927012	3.86482157	3.1805014	0.67383994	0.77549774
0.10654709	0.0268564	4.25944099	0.28885397	0.00802448	0.02516812	0.00954842	0.20058837	0.04204291	3.72428941
0	0	0	0	0	0	0	0	0	0
5.13032568	5.53177729	4.77523114	4.96369645	5.79581362	5.59959657	3.65858682	3.62733119	5.16462273	5.58024459
3.75410538	5.68502515	4.62406923	3.83479588	3.83141869	3.76432959	3.45607406	3.14782729	4.50028762	3.25573593
3.56209722	1.36968108	1.42914044	2.55176775	1.66054406	2.06459069	1.9482623	2.53649012	1.54456799	0.61161734
0.1662798	0.0473561	0.01928343	0.40298938	0.0126472	0.12240926	0.02250831	0.33669132	0.04964528	0.05418424
1.73746246	0.72044303	1.31937699	0.60053928	1.23329511	3.19641394	0.45542148	2.600714	2.26237572	1.59294482
0.91775616	0.59460692	2.55891806	1.03847676	0.86656524	0.87609509	0.47030341	1.53487462	0.70600854	0.62119228
1.70885932	2.45541038	2.68840395	2.39111281	2.28921945	3.6141164	2.77357623	2.02998264	2.48234847	1.71156691
5.85859849	2.48421407	0.54052308	4.70151507	1.70747873	4.83804864	0.29358375	0.39353395	5.73566699	0.11944848
4.81713552	5.24936086	5.09821488	4.40184747	4.41586706	5.46796616	4.11514449	4.03022898	5.15927339	4.89741144
1.94116848	2.91520654	2.42569448	2.03110396	2.57084451	2.85911377	2.15572135	2.42177683	2.14087286	2.18304088
3.0961952	4.01859105	3.61209836	2.89680868	3.81121125	4.38431978	3.63498808	3.26969181	3.40433912	3.2376652
2.34124763	3.33340191	2.61412583	2.19854948	2.84897343	3.16271088	2.53029104	2.74951667	3.18147005	2.75992075
3.01258526	3.89589913	3.41005028	2.84303635	3.71340342	3.78924075	3.16164636	3.06477509	3.79100795	2.86768014
5.00594356	6.44574832	5.41336132	5.74335829	5.11640607	5.03535574	4.00690413	4.42476267	4.50565402	3.32024905
0.27402588	0.31107636	0.31334322	0.16530154	0.41295694	0.56528564	0.05954553	0.35858216	0.63069461	0.06205978
3.18456733	4.12248012	2.91298423	2.85784832	3.6522006	3.3636668	3.63795199	2.7838505	3.08211866	2.9839028
4.38404329	4.00220694	4.3873981	4.42443171	4.40286789	3.91657891	4.69545562	3.97116254	3.59683788	5.17639263
3.77619652	2.80869093	4.883451	4.55103042	4.33353962	4.4699797	1.8239508	3.67191317	2.47072714	2.13942188
4.70129936	2.52714061	4.22400311	4.73296293	4.22476881	3.54175865	3.81672638	6.18257185	2.81669072	3.31017871
2.59950347	2.89214635	3.98566485	5.12068502	2.43089432	2.86073647	1.6028108	4.35486929	1.92207095	3.03125309
0.65141391	0.67440139	1.14615666	1.13880266	0.50320711	0.42686569	0.790249	0.94231955	0.32458037	0.16817483
1.12861982	0.35132972	0.40203208	0.41530692	0.42922764	0.640115	0.13036209	0.47604943	0.30499265	0.04467805
3.09001214	2.73878578	2.36635007	4.22015262	4.9512488	3.43805939	4.7425464	4.89135034	0.6530744	3.15824382
3.24525544	4.28564589	1.36010729	2.97677857	3.06115392	3.96518831	4.04549914	2.35854483	4.93536812	3.54670485
1.73326013	1.55137049	1.170961	1.61087066	1.52595658	1.30128398	1.39996895	1.23475658	1.55713412	1.05195004
3.66293207	3.36383955	4.39685895	4.24691216	4.29038917	4.21879189	4.6589099	4.48886276	1.73731296	6.46625731
3.85411414	4.20183252	4.98271913	3.63210441	4.05119342	3.64683344	5.02525205	3.07468538	4.37313383	4.32239557
2.94339841	3.65814418	4.17741244	3.1317843	4.27212687	3.94680394	2.86236304	3.5517745	3.69429385	3.89680977
4.56770391	4.56124827	4.47177411	4.89299965	4.67518962	4.54317133	4.83084574	4.31905159	4.64442545	4.61766356
6.06682562	5.16518662	4.97123081	6.35997897	6.81540948	4.5188772	7.21519544	5.43401206	3.8643812	4.50630212
3.06243821	2.91522518	4.20805174	4.22481667	3.44033621	3.57225647	3.1855655	4.22749579	2.55074761	3.16209794
2.89669442	0.30346241	2.34263035	1.58894956	2.14417305	2.1488186	0.69251451	2.27615559	0.54510674	0.41198899
1.64761427	2.8591825	2.16120588	2.54153522	3.244331	2.5258405	1.55440402	2.14323313	1.78984162	1.78881376
0.55726716	0.89737459	0.51566216	0.56674455	0.49031625	0.49638328	0.43004216	1.25838416	0.25107786	1.02787799
3.39651978	3.34904356	3.7712148	3.88378367	4.06572493	4.02892294	3.11690712	4.19657348	3.61457193	3.13852033
1.60391987	1.14280662	1.94653561	1.70539423	1.78376096	1.49546743	0.8429884	1.58245668	1.32490279	1.50761975
2.27149622	3.10503016	2.99243736	2.32097903	3.23673322	2.76015521	2.19810051	2.42560558	3.08448411	2.24864537
5.8331499	5.74312449	5.94255098	5.79105161	6.52971386	5.89882654	5.24028065	6.09112759	6.12797525	5.81434061
2.61054606	3.52933505	3.07035026	3.45344113	2.7527209	2.58432989	2.33658421	2.98595823	3.06458211	3.00201878
2.78734544	3.02103683	2.43571071	3.97282059	2.88077592	3.22322978	3.61690368	3.22107682	3.05987624	2.55992215
0.83508149	0.18046099	0.44339697	0.29561307	0.19062974	0.10087875	0.07869088	0.40607101	0.16051688	0.04157779
0.027313	0.01166892	0.02922995	0.0599729	0.04564232	0.00684205	0	0.22023805	0	0.00718077
3.32793737	3.69812258	4.12679561	4.6942919	3.4214959	3.8595399	3.25242205	4.37966429	3.73330903	2.40765404
2.41379906	1.04461474	1.56768761	1.40231232	1.50472549	1.34461924	3.48128397	1.84656441	1.11004617	0.8517776
4.5677183	3.20529626	4.70125656	4.75454668	4.02700892	3.27088545	3.57040543	5.38371932	3.39463027	3.49023051
0.88998508	0.02334526	0.03901107	0.10176269	0.0458392	0.99963336	0.23800711	0.1114513	0.1505969	0.19787429
2.86475119	3.01683945	3.48501935	2.84155676	3.08459738	3.25811153	3.25411631	2.8770609	2.98985452	2.71832931
0	0	0	0	0	0	0	0	0	0
3.37392573	3.44347007	4.00397185	4.85460178	3.29093147	3.44250225	3.15462335	5.13960383	3.05450703	3.23871154
4.2417814	4.37281608	4.10507016	3.99343545	4.45380663	4.16182033	4.14480293	4.42032259	4.38743594	4.05500644
3.69592529	4.55378783	3.84409982	3.75224896	3.93513715	4.12217069	3.66342709	3.9349773	3.79315215	4.02175766
2.83684125	2.51493831	2.54625622	1.82330902	2.20831113	2.80648474	1.69208006	2.07962932	2.49066906	1.79206058
3.84668053	4.3018874	2.30639656	3.44630147	4.42433652	4.09804668	3.54059233	2.65565627	4.17433843	4.00954748
2.5055498	3.38884729	2.43502756	2.67109358	3.56563752	3.60781539	2.29429214	2.4082798	3.16959152	3.11122045

1.47934901	1.24660968	1.76148915	2.11661035	2.08220761	1.38153789	1.77159682	2.01427833	1.21943725	1.17642343
0.93198571	1.41776462	1.95969524	1.24381068	1.1859024	0.94973544	0.40949175	1.22369156	0.92405347	1.11023877
4.98525062	5.11648364	4.9645902	5.53363115	4.82303264	4.81652784	4.53258598	5.80408777	4.7350107	4.82202497
2.62904484	2.45605602	2.41530461	2.7199052	2.06930234	2.58331894	1.70312537	3.03416148	2.52658445	2.04037387
3.90449722	3.40972679	4.34996248	4.07557594	4.26338421	4.14825983	3.90808705	4.06604213	4.01514181	3.64010595
2.02126601	2.23337084	2.38215525	2.31224764	2.60943418	2.57920499	2.04145689	2.41012885	1.94026077	1.88142548
2.83796626	2.78868157	3.12093241	3.13550803	2.93713448	2.3858358	1.93004696	3.29482284	2.1782736	2.33332627
3.43411826	2.20735861	3.90954457	5.24327981	2.54533613	2.76132341	2.74016913	4.99661776	1.83588921	3.43514912
0.5465487	0.03916892	0.07791255	0.13211352	0.02595696	0.01541223	0.28231013	0.30698335	0	0.06363127
0.33119047	0.04669342	0.07003527	0.0350066	0.02329003	0.05451668	0.0815116	0.08505883	0.10293409	0.3594727
1.26623153	0.58137416	0.76174071	0.91510601	0.70909332	0.51108861	0.78844305	0.57689406	0.52146365	0.9202534
0.03368123	0	0.0431407	0.23861446	0	0.02520684	0.05028644	0.18773643	0.18318812	0.39452022
0.25472309	0.31749093	0.30176005	0.27115745	0.16579749	0.3450302	0.14892558	0.39225797	0.53571537	0.57681196
0.86966355	1.13253132	1.30398206	1.43711711	2.00037674	1.03074899	0.54036108	1.93570489	0.23004373	0.6354529
0.46408963	0.17661137	0.27058882	0.15202278	0.34481084	0.46207335	0.10171963	0.47431661	0.51823026	0.38384244
5.14075857	4.7129267	3.7282414	4.07593947	4.06840986	4.54362916	5.02229725	3.41655178	3.5923357	4.45676362
0.44326894	2.16384823	0.73233739	0.44722531	0.09733668	0.73891005	1.00393316	0.12586362	0.0670553	0.17634933
1.43157452	1.92548156	1.6784755	1.69940166	1.27937214	1.26490422	1.35579811	1.33511437	1.71235948	1.46986064
5.09797669	5.4467457	5.19071883	4.63566479	5.36694345	5.53308757	5.60001823	4.86332696	5.12555519	4.80768738
1.0347655	1.36397054	0.75371973	1.06905711	1.48433933	0.96331272	0.31428707	1.60498086	1.57250817	0.94279099
0.93202385	0.28880794	1.9636285	2.14204608	1.31403562	1.2298347	1.25557116	2.36311939	0.35255602	1.25517149
5.75829139	6.21527375	5.97969946	6.04917152	8.00499067	6.20094929	5.69520449	5.79904821	5.70758868	6.04086882
4.19929468	6.44777689	5.21176092	4.00069261	5.46668601	6.26759167	4.44102188	4.03202221	5.73753358	4.84456002
3.91820063	4.65174078	4.23230216	4.65507565	4.02946246	3.95507682	3.37926536	4.56011119	3.81833984	3.48989266
3.79866475	4.41345804	4.15351081	4.11074613	4.97807397	3.87717182	3.4922285	3.86190677	3.48618611	2.3768689
0.14583158	0.49239286	0.56405597	0.0712424	0.13832213	0.35366074	0.35561094	0.20971231	0.29705304	0.6047251
2.03114143	2.0256565	1.89432902	1.35335953	2.02030292	1.58199028	0.91547355	1.24853064	1.10501465	0.50093648
3.89118139	2.52856633	2.38618766	3.26680051	2.89496454	2.73470108	1.66234696	2.85605634	2.17654579	1.01870159
0.77942952	0.66250132	1.16850464	0.42326881	0.04226118	0.18097874	0.14557548	0.85582042	0.01935822	0.30599483
1.54559422	1.38371802	1.35177158	1.36129816	1.83953906	1.24998895	1.16570502	1.20537285	1.2118817	0.46179913
1.99513179	1.2088843	1.76519897	2.31989493	2.60949676	1.58871118	0.97786746	2.86321858	1.35797534	1.60179641
2.78241314	2.55176266	4.13437025	3.18182768	3.17623397	2.81155846	2.14153067	3.09419847	2.37316388	2.56603115
5.54245691	5.45376139	4.434146	4.27720151	4.49622168	5.03154991	4.63389735	4.32236017	5.55581238	4.74816943
0.07453292	0.06019844	0.48152546	0.36940385	0.21944094	0.30125809	0.52222813	0.72099065	0.07203854	0.25373085
0	0	0.03886258	0	0	0	0.06744344	0.03562594	0	0
3.76610356	4.0781596	3.41539499	3.69397452	3.80040993	3.49751163	3.37975355	3.90708593	3.82744511	3.80656089
3.95416156	4.02749442	3.59679248	3.92592826	4.15297418	3.85425678	3.77952944	4.41528618	3.97383835	4.21170479
1.5124375	1.89448277	2.00945539	1.48018081	2.35970181	2.15714588	1.37428795	1.86277733	1.84132729	1.13120609
4.61292225	4.9177656	5.26435992	4.52804347	4.86847663	4.38387593	4.51397154	4.88348326	4.95000554	4.75582271
2.50682356	2.81335423	3.4290267	2.53718557	2.82090547	3.11302851	2.69057401	2.57763698	2.72812094	2.28148687
4.07682036	5.04335458	4.59018086	3.99147862	5.95178069	5.03588066	3.9767457	4.01852039	4.66871454	4.93572836
2.90244063	3.44373957	3.26447546	2.98823085	3.57393698	4.29572348	3.39587306	3.64530261	3.2117703	3.1254549
0.58737175	1.07186728	0.46452099	0.55013522	1.52282118	0.99462253	0.7362353	0.73194651	0.65243836	1.13244799
4.59590381	4.38929182	4.2720666	4.05370219	3.84358785	4.21036746	4.81953139	3.95814048	4.9517751	4.11471897
1.60276826	2.317293	3.53265284	4.77216564	2.81860087	3.09910931	1.34001412	4.59022107	2.59520963	3.28441623
0.30995554	0.0348374	0.38395468	0.22503838	0.93580347	0.59596276	0.19857062	0.78407163	0.10508282	0.06357423
4.42876219	3.77082171	4.5979119	4.74821252	3.88413552	3.50699951	3.4896451	5.09594373	2.9342275	3.35630402
2.24775212	2.73042294	3.3874083	2.85647236	2.96041973	2.95773599	2.42452259	2.87443901	2.59733338	2.38102924
0.21192121	0.04523002	0.32488693	0.42143146	0.33880257	0.06657538	0.03563999	0.55233911	0.05074621	0.30124169
4.59400808	4.86357788	5.80146943	7.29500896	4.392841	5.81087237	4.61220186	6.15305737	4.06158811	5.99176571
1.91545872	0.75352598	1.52021622	2.1403358	1.37731831	0.70973108	1.04604653	1.85125709	1.58575288	1.11172443
1.47300493	6.47701954	7.51835074	6.50275885	5.54754691	5.73344788	6.8381676	6.17498187	7.95056658	8.04610505
2.28954402	3.27302335	4.34650059	3.84214679	3.75411284	1.01648956	1.55221356	5.11877894	4.09058702	3.71349332
7.93028019	7.03392801	6.54799721	8.13170144	7.69896116	7.17282005	7.05819974	7.19527341	7.1940075	7.77523799
5.47130429	3.83752367	2.92474112	4.10550629	3.97296004	3.57668954	3.56236965	3.58684738	5.0197016	6.65222095
3.04534262	1.26242017	0.88913752	1.86411391	1.50389334	1.30952396	1.28807435	2.33217733	1.86507504	0.88566763
5.79323702	5.17922565	7.47755717	5.21408138	5.36624009	6.05991198	7.31601823	5.25051443	6.66711808	6.09790421
2.730376	3.81272511	3.80399382	2.69667664	3.59169511	4.05390399	3.14384261	3.5605548	3.03061165	3.16342721
2.314767	3.4370495	2.66667274	2.06941206	2.94158627	3.01009705	2.09658726	2.16647199	2.92838284	2.02501625

0.05520715	0	0	0.05259451	0.15147637	0.02084542	0.06202935	0.06474767	0	0.08556678
0.01162395	0.00741959	0.03701609	0	0	0.05132842	0	0	0	0.45819855
4.11643704	4.60214709	3.54705188	4.00789903	4.44507021	4.08310468	4.23981801	4.20104858	4.42735664	4.29744423
2.64477281	3.57515707	3.89346058	3.14991557	3.09826959	3.63082847	3.13314469	3.01919471	3.12123723	3.0784237
1.88960639	3.13098735	2.37403868	2.30993725	2.62167603	2.49237648	2.08423859	1.8612996	1.16650733	2.10987045
2.47047595	1.72962114	2.1970944	2.00588753	2.04105783	2.00042892	1.72271937	2.28839659	2.11665777	1.93543555
3.950131	4.55764628	3.84267375	3.6786669	4.45211877	4.27292909	3.76367005	3.78927678	4.10527304	3.62189604
5.84749463	5.79257425	5.09882665	5.2929479	5.43298123	5.51746801	5.82332508	5.60187373	5.76392725	5.30416959
2.63592996	2.79829317	2.19357773	2.74078614	2.3963881	2.69831587	2.42369372	2.58695991	2.68517166	2.39741631
5.23115612	4.80645297	5.14131651	5.40978837	5.48031425	4.72309593	5.49692529	5.39394826	4.72742474	5.16121758
5.16402117	5.79429217	5.93359971	5.91986904	6.18286356	5.82363345	4.78715096	6.02709473	4.2343034	5.49066995
3.38284623	3.05910216	4.04076071	3.68599879	3.80622685	3.64409171	3.92425829	3.94320929	2.69929922	4.06700507
1.78526844	2.30900939	2.0641677	1.72608963	2.77759301	1.97785019	1.35749618	1.90269099	2.16594036	1.56997212
1.79288445	1.06363833	2.29609634	3.23319631	1.84228506	1.84782951	1.71600145	3.63520151	1.21139823	2.06069488
0.7302049	0.98809357	1.28850083	1.16204328	1.917429	1.35195035	1.61515503	1.65182771	0.81376913	3.08913281
3.41927276	3.75463959	6.05103118	5.1656133	5.35246835	5.1430846	5.64150734	5.64314945	4.36959097	4.55748675
2.47514301	2.71612103	2.43986074	3.68019318	3.29359953	2.56185484	2.02603801	3.75831498	2.97664489	2.59281166
1.93853771	1.17529081	1.33474182	0.57394515	1.77738254	2.19301111	0.90087904	0.8398822	1.30518515	0.28272294
4.85563607	5.47877075	5.78892266	5.02651421	5.19513546	5.25635375	5.2258854	4.98822088	5.0440752	5.52364724
0.06104899	0.25441052	0.36038528	0.76957092	0.06845596	0.24330867	0.00609425	0.43468579	0.06086022	0.16629031
2.91144018	3.66078577	2.56499853	3.20372211	3.73753616	3.6371023	2.44267719	3.35084548	3.02085793	2.31425773
3.98167946	4.81849517	3.56429229	3.37214085	3.06353786	3.32708737	2.72353223	3.18791437	3.55247308	3.9312912
3.69738657	4.06365224	3.85528689	3.42540485	3.77767305	4.08172344	3.07270527	3.1694529	3.78094127	3.53238721
4.43760645	6.26720523	5.48600997	4.77363834	5.50944392	5.36412314	5.11754373	4.97001714	5.44158699	4.94800702
2.67837727	2.65522209	2.6313317	2.76751323	3.03790665	2.54010582	2.35752115	2.98042904	2.58593272	2.76720157
2.09815763	2.75323024	3.3523052	1.93213253	2.0295792	3.15728604	2.74983843	1.87156598	2.36838156	3.06345434
3.20263264	4.03553038	3.59658335	2.72429451	3.60296394	3.35931103	3.0021619	2.92629938	3.42095968	3.03139126
0.01721906	0.00550981	0.01108749	0	0.03240694	0	0.01294785	0.01015591	0	0.0202086
0.05455863	0.01410681	0.0212787	0.05197613	0.01395913	0.04090538	0.00832572	0.03873328	0.06237398	0.01731566
5.80919952	5.28551411	6.13435665	5.90114451	6.17282946	5.94619333	5.70598128	5.35314502	5.63654784	6.36137345
7.21156562	7.10380429	7.29199182	8.0531964	10.4877975	7.82920899	7.74810232	10.5315826	7.40977239	7.11234998
4.16589433	5.36534841	3.96518585	4.33733766	4.52891043	4.62906711	2.91864649	3.65071899	4.42939157	3.34167919
8.26713024	8.41549427	8.68733779	8.53004751	8.89361211	8.52580737	8.54836427	7.74118493	8.46865351	8.15720925
4.71785064	6.32901584	3.01047507	7.20564613	9.17592648	4.2639473	5.78782208	7.83803855	6.82441307	0.34484836
3.4376625	1.91563182	4.13482178	4.41122639	3.85200948	2.85039826	2.82755889	4.14922551	2.02698442	2.05598214
2.55435486	0.33584627	1.55472597	2.24498475	0.35453362	0.44752371	0.1413365	2.18548131	0.14942844	0.17788686
5.442196	5.22291416	4.35043499	5.62531844	5.5968051	5.77472984	4.18641315	5.13912493	5.7890135	4.60413911
6.31145862	4.72391668	6.55573067	6.49976171	5.19989659	5.88194216	6.49813117	6.74036628	5.01877794	5.28744418
4.4564432	4.47783484	5.76602873	4.81857798	5.13114248	5.23614659	5.37338535	5.14482733	4.55368918	4.3030376
3.77638904	3.14829153	4.18245436	4.39233818	4.01873844	3.58573803	3.74008812	4.15704386	3.07226659	3.74792034
5.95216533	5.17094963	6.5485237	4.99767816	5.60231998	6.07219518	5.50075638	4.26798163	5.99854723	4.685618
1.78432035	4.67776792	2.44294731	1.76682435	3.78598747	1.19900803	0.53072657	1.73396626	4.05704136	1.09789067
2.84001793	2.60652339	2.42777892	2.75436077	3.23144815	2.70159559	2.01250482	1.84519238	3.05479454	0.76220548
6.84503619	6.68950177	6.62775323	5.98679035	7.1941335	7.03993595	6.8592013	6.2295176	6.62464698	7.22767212
3.62548857	5.17939007	4.6709092	5.05259197	4.81132113	4.73364548	4.28885864	4.36266951	4.31203846	4.23533441
2.23785198	2.2934335	3.4705707	2.54861145	3.31942274	3.02045174	2.20211189	2.72852033	2.3012528	2.52767155
2.29560996	4.58889847	3.65533993	4.34520063	2.4337534	3.50871274	2.0557115	3.68795081	2.07084563	2.33340231
4.81673322	5.35880079	4.47736856	4.76013273	4.4498318	4.89345834	4.58244008	4.91877892	4.75467685	4.30107551
3.68833832	4.42075834	4.41837627	3.71690885	4.13617152	4.70848265	4.15296454	4.11305399	4.19990938	4.47012107
2.23269211	3.18071971	3.62846913	2.70972678	3.1973134	3.87642919	2.25778897	2.59068508	2.9350861	3.33196673
2.12304051	2.62467072	2.96737176	2.12222841	3.08811579	2.45825844	2.32361956	2.07700625	2.07069948	2.30514539
2.63018594	3.21900113	3.75430735	2.76238047	3.97478999	3.22617614	2.43963872	3.14326295	2.84405128	3.29321522
2.0403408	2.7997339	2.82806656	2.02074389	2.77310827	2.59016223	1.85027835	2.20326459	2.2356333	1.94463001
2.55459015	3.47327606	2.93715517	3.26790143	3.87063326	3.92821316	2.70729733	3.17881928	2.72601619	2.57317359
1.69806672	1.98685124	1.92726548	1.96912631	2.38314572	2.45431956	0.9427635	2.05796494	1.80705826	1.52799156
6.03812321	5.25257955	5.37100285	5.79392192	5.05774999	5.60331834	6.05747298	5.31910552	5.38305501	6.22066494
2.68328934	2.60442114	1.94271122	2.13655373	1.77357648	2.81996694	1.88643728	2.43048782	2.33200712	2.15344441
3.12496575	3.41686737	4.17579224	4.06167053	3.63338645	3.92167847	3.3858407	4.06627506	3.02050721	4.68949379
0.1211011	0.03725779	0.03260462	0.03709431	0.04654597	0.01756743	0.0088651	0.06805126	0.04456081	0.01843374

3.91417212	4.10247791	3.47532193	3.22371198	3.54554479	3.67038888	3.78502364	3.14982086	4.06750721	3.59573908
2.88811843	3.16762536	2.65524073	2.37040026	3.07334731	2.96137485	3.12270066	2.8585614	3.19184475	2.66009764
2.47021823	2.45928531	2.10277803	2.03041618	2.21494462	4.25855766	2.08624274	2.46421255	2.85958837	2.41815869
2.3745076	2.960431	2.22371993	2.1139585	2.45559483	2.42762484	2.44608069	2.21656989	2.81349534	1.88571451
2.313324	2.9681061	1.96670331	1.96317172	2.31550358	3.00825966	2.22496944	2.67414137	3.02398737	2.28408922
2.4609648	3.11472038	2.29544655	2.07748196	2.61600952	2.57911951	2.09688116	2.17444799	2.88715132	1.94547809
2.77209691	2.34480442	2.47456709	3.09383126	2.29479536	3.71384224	2.79946147	3.164237	2.37860955	1.34308949
2.23519803	2.77336415	4.96953023	1.86795752	2.75908722	2.68564293	2.86556117	1.98261011	2.30596037	2.77827997
3.09715765	2.87377148	4.89084964	2.76435686	4.60234846	2.17967942	4.98858837	2.88452505	3.37777288	4.07986276
2.70020067	5.52982622	4.85415202	3.72382932	5.73425086	5.93093631	4.70413376	3.06903423	7.12433054	2.21346006
4.46193067	2.62815272	2.9548252	4.37062867	4.21038311	2.75950077	1.41424621	5.94427832	1.72445911	1.68093822
6.90901279	2.8289347	1.75592349	4.42653447	3.31286677	4.78002299	3.22594386	2.47965924	3.6896746	1.44263273
7.23721801	4.12031995	6.6454855	5.5678757	2.7078274	3.13299632	2.36098401	5.44110906	6.99435437	5.27873955
5.44369014	5.63228176	6.0404329	5.99537714	7.3843365	6.48920516	6.63864094	6.22139058	6.31099846	5.69157835
0.21214803	4.31249249	4.10203712	7.1873165	1.46727323	1.98372809	0.19581666	0.36733331	8.5850405	0.55455586
1.69878104	2.80052128	3.65948493	3.30703862	4.57225369	2.4505518	3.19872328	3.16918378	2.64571472	2.51894263
2.92693256	4.18517442	2.14664927	6.09713645	4.85559258	4.99215344	5.9437001	1.20176733	6.15138156	2.90881586
0.69024178	0.80360843	0.35297825	0.80589628	0.98069536	0.09001631	0.0510107	0.84875881	0.18570773	0.20184979
2.43271918	3.11449337	2.75694356	1.35075372	1.7494377	2.36011723	3.86574325	1.60215145	2.11213629	3.60983093
0.62675459	0.15781374	0.19093554	0.34626328	0.33506985	0.13946748	0.23871755	0.23303412	0.19975831	0.62557452
3.24162095	2.90238923	1.84458712	2.148492	3.08448618	2.63028618	3.6572779	1.87303678	3.67441539	5.20922512
8.32570132	5.33667058	5.16734311	5.64853861	5.75414649	4.82346495	4.13142055	4.80200651	5.1846422	3.06144325
2.00309776	0.12871693	0.06002886	0.43364948	0.14695846	0.1077348	0.0101962	0.20866356	0.09447904	0.00532701
2.01933109	1.9338231	1.50276126	1.69402415	2.07423732	2.27650358	1.26353062	1.34967309	2.33400155	0.37859964
1.99628235	0.89754395	1.01068599	1.04480188	1.69020894	1.22560617	1.10001205	1.00644381	0.90534529	0.3189423
2.09046175	2.18146392	2.40272266	1.43518781	1.97652485	2.41303481	2.17074641	1.74651234	1.95726955	2.44439685
1.72016037	1.5687026	1.88364348	1.69489496	2.02776766	1.75137475	1.4832152	1.54012024	1.23378964	1.96333671
2.24718666	2.41342557	3.58652453	2.0849794	2.99229342	2.97967854	2.21477903	2.61712834	2.86098789	2.58342886
3.58547327	4.29901888	3.08434206	2.95027442	4.6579682	3.44479617	3.1291819	3.31580575	4.08904236	3.05804814
4.62953752	3.7033724	3.92907797	4.38069301	3.9650542	5.1004819	4.08702987	3.55499156	2.42007114	5.19476062
3.26016265	3.1060708	2.08134088	2.38819154	2.64323037	2.43893741	1.90084453	2.26848694	1.99479363	2.24727797
3.20110207	2.88894033	2.51243171	2.61538257	3.32518494	2.63294885	3.71620944	2.58061596	2.87010548	2.94829907
2.95345508	3.29340816	2.14471078	2.74556655	1.80792822	3.02738974	2.60294856	2.34262036	3.65337513	3.22028776
3.54867827	4.02852567	3.64643846	3.10880446	3.53035269	4.92353327	4.24090953	3.22299554	3.95755539	5.23978037
0.27540172	1.55112202	0.42720061	0.52147666	0.60782775	0.47346976	0.37418517	0.75958601	0.4453828	0.40637854
2.85866637	3.4361372	2.26929489	3.18255355	2.78862557	2.5992936	2.93215153	2.97074651	3.03592965	2.63537394
2.81757683	2.63582607	2.72657954	3.21260969	3.59733051	2.44675467	2.27013833	2.92486357	2.27753516	2.53971942
0.45680185	0.41031036	0.88708323	1.11321853	1.0249437	0.69230021	1.10788306	0.90815967	0.58724367	0.27243739
5.8392748	5.93399021	6.27250015	6.68362097	5.52323451	5.62106023	6.00729899	6.7339032	5.58342872	6.17538795
1.66937291	1.55015928	2.01425133	1.07312837	1.22379888	1.67999464	1.17048717	1.13331423	1.54924339	1.0661834
0.96023766	1.19053786	0.60745941	0.90406662	0.58205634	0.87439124	0.391992	0.76084261	0.82670387	0.93459704
7.44229676	6.13432993	6.13080047	6.05686883	6.65671706	6.46519833	7.47743616	6.0344813	7.33654001	6.23934759
4.64086725	3.8875413	5.05419858	5.59753537	4.67875642	4.12257328	3.31991278	5.52548032	2.87091421	3.06398251
7.51965383	7.55677038	6.18039641	6.2349993	6.65038607	6.96072254	7.26145732	5.10662143	7.68236632	7.00584993
3.34849683	4.29828666	4.26294353	3.34077175	3.96736332	3.64247234	3.56777261	3.89769353	3.50419651	3.6008247
3.66900462	1.5175555	2.02055914	2.61399502	1.77649586	1.57623992	1.2030603	1.82048223	1.54806949	2.10046672
4.67067919	3.95305652	4.9411597	5.11400335	4.16368078	3.85989679	3.81624442	5.41374925	3.94195642	4.14588535
2.6648412	3.24032753	2.73330045	2.83930508	2.93949519	3.20035943	2.2244964	2.2377115	3.08816254	2.71524376
0.57683729	0.04877797	0.14776704	0.61414539	0.21262041	0.09369455	0.02574394	0.89030298	0.14154294	0.09184765
0.06643976	0.06467444	0.06957604	0.72182134	0.11907743	0.03430312	0.03451402	0.66336459	0.02036751	0.02500764
3.68397019	2.62632782	2.94674788	4.85869345	4.53979298	2.42287208	0.96232642	4.61424725	1.54093795	1.88472123
2.44742548	2.43730027	1.96761466	2.14053253	1.92539832	1.92258224	2.07717871	2.29089171	2.69236534	1.98855996
0.04093004	0.04557949	0.05880551	0.19383172	0.10719437	0.0900663	0	0.32650039	0.05829717	0.03215548
0.04992139	0.02567055	0.05129767	0.18998183	0.07490493	0.06666122	0.08874661	0.03542464	0.0430245	0
0.75579033	2.10460679	1.26926936	0.6342239	0.65901077	0.34939264	0.81927718	1.54598114	1.18124689	0.21431653
5.31712955	3.41638119	4.80653979	3.79782933	3.63355014	4.29780175	4.58519502	3.24214173	3.87898676	3.94041866
0.49946768	0.51642996	0.28558553	0.8426177	2.18434192	1.37988865	0.8401524	0.43379623	0.79267838	0.29715109
2.0907193	0.85616375	4.71296614	1.63904507	0.24799148	3.82530436	4.31348229	0.18552756	0.36075388	2.47099369
2.34384391	0.81662058	0.20878438	0.81384659	1.17690002	1.01925775	1.02840198	0.41801996	2.87042456	0.14855905

0.17187327	0	0	0.06982031	0.00675567	0.08554404	0.02398481	0	0.0454344	0.00838513
0.44926325	0.08611788	0.05639304	0.13542445	0.09113824	0.20703185	0.14210496	0.05735647	0.67144405	0.03082464
0.63860399	0.25229611	0.50977829	0.32511393	0.46276661	0.33580587	0.36674571	0.82057556	0.50321152	0.60087
0.10293758	0.07597188	0.12729839	3.13057086	0.05247143	1.81853745	0.42543885	1.63057114	0.02673231	0.06488268
2.23346954	2.64506691	2.64140456	1.33662519	0.47805728	3.16175036	3.16040379	1.38187626	3.0620868	3.18972619
1.87367596	0.38319855	2.2284542	1.15173191	1.86243334	2.38274598	1.38652878	1.25074731	0.55074456	1.77439509
4.99566671	5.47880983	5.29012741	5.08472758	6.93204675	6.80375859	7.39445451	4.15267554	6.80631554	4.0795068
0.87198108	0.3165207	0.21415685	3.26818987	1.63623378	6.33495663	0.56168565	0.51225491	0.35747306	0.75333033
0.91168947	0.21243605	0.97371936	1.43465126	0.69924436	0.81391338	0.32486388	0.93978805	0.21893471	2.96774365
1.30206865	0.02430093	0.01841109	0.46519506	0.01207382	0.09035583	1.17675434	0.28632374	0.02728874	2.15361869
2.10290622	1.78135959	0.87426309	2.46516125	2.48345142	1.77685078	2.05284089	2.982562	3.19639372	3.28318687
0.17319757	0.0545157	0.09822537	0.17078091	0.07536796	0.05204897	0.06632509	0.25366175	0.10266317	0.08350877
0.55162665	2.54933712	0.08703197	1.98308127	0.00549285	0.02581336	0.0195231	1.11112903	0.16522692	0.04043546
1.23020218	0.38009182	2.81980565	1.88975084	1.79545416	0.03318312	1.99600761	2.04650747	0.83945817	4.19337008
2.48425787	0.21664825	1.81579251	1.03283261	0.24187115	1.70176688	0.97750586	0.34133465	2.54650305	3.17842856
0.02547687	0	1.29615913	0.00407166	0.00269916	0.00319305	0	0.00503214	0.01222526	0.19139283
1.89313787	1.51154263	1.11286542	1.37429573	2.05097516	1.35822543	1.52740227	1.40451388	1.34897007	1.3425572
2.13493263	4.09484951	0.52937933	3.49572276	0.70201572	0.45178336	0.1518658	1.56656915	0.29115821	2.19432318
1.07695684	1.68846912	1.61024154	1.0156995	4.16930394	1.73576336	1.57652177	0.81692061	0.85576289	2.51688737
0.99309801	0.90376553	1.61744801	1.6052886	1.37002609	1.28515329	0.5473013	1.98829875	0.36637888	0.45786612
1.39610149	0.88598657	2.64109588	2.44806798	1.77788862	1.61208668	1.59193884	2.48811016	1.03798059	1.42903882
1.65306487	0.68541316	0.79793282	1.36958834	0.68392411	0.74463944	0.28354767	1.09793458	0.41548663	0.2163914
6.30226546	7.15322964	5.36541642	6.50050738	6.19750595	5.47910242	6.299949	5.41507158	8.27108122	6.25464917
3.75881394	2.86917279	3.10072018	2.83949497	3.20712072	3.00697982	2.52053312	3.3510546	3.16153264	2.48321499
2.51955232	2.78248849	2.83023364	2.59956041	3.0227967	3.16350532	2.70031805	2.56637969	2.71647716	2.29354873
2.96935282	3.20003491	3.79153923	3.72612792	4.42976996	3.10587318	2.50326183	3.91550228	2.80199293	2.76604933
3.02806429	1.81916863	1.76867574	1.82951743	2.54582013	2.08814677	1.67509405	1.86793916	2.10113143	0.86980263
3.19792488	2.10570704	2.59836786	3.30631536	2.28913629	2.06668747	1.19800173	4.13841681	2.08762668	1.84788494
1.90738174	2.79450579	1.56254892	2.03488478	2.47517976	2.76825533	2.27849323	2.50325787	2.44273144	1.98062634
1.97185512	2.07046939	1.89053633	2.08636103	1.77395413	2.07736063	1.30612638	2.28162299	1.83479173	1.50396744
1.72956104	1.51044945	2.82807858	3.98974721	1.86663222	2.16412135	0.90433257	3.58556139	1.27133574	1.93535327
3.84005884	2.73832518	3.89977558	3.57045102	3.62039756	3.51369727	3.09046138	4.10848623	2.95855426	3.17794122
3.65209612	4.05596582	4.40888091	4.41090952	4.16391515	4.59159025	2.76375503	4.49437269	5.02653172	4.06809475
2.35952015	0.18413577	1.80540416	1.47400628	0.21007293	0.85246985	0.08969928	1.05868523	1.70457302	3.7124514
2.24431808	2.19247694	3.62720838	3.57237674	2.79998609	2.54201021	2.75262567	3.92391131	1.79822726	2.28814025
0.08334063	0.02708573	0.16887955	0.39619342	0.38547418	0.4343493	0	0.42032277	0	0.11821553
2.069414	1.51281225	3.10183642	4.27575016	1.93688855	2.26257503	1.20250668	3.53859512	1.05243639	2.51235369
7.22615264	7.11029173	6.64128929	7.19305892	6.57861841	6.6554224	7.8078708	7.00551767	7.69185192	7.2048408
4.18543738	6.75727883	4.8793324	5.69539407	5.89149356	5.1419768	4.66235144	5.34752441	6.40964352	4.71599439
2.10703394	1.77939926	2.61683214	2.13335568	1.65346519	3.00849692	2.30090699	2.01180057	0.5648554	3.29497485
2.37531661	2.3351845	2.58482082	2.45821342	3.12593744	2.71526959	2.21550625	2.69803521	2.55113585	2.99013975
2.77223013	2.807943	2.25257001	2.67298562	3.06336796	2.83875944	2.78615093	2.62685139	2.05663812	2.47702453
0.15740154	0.11123628	0.21612152	0.61226441	0.05610508	0.14988114	0.05567127	0.13573666	0.10445062	0.1987999
0.00775472	0	0.14711711	0.03653776	0.03870657	0.00579045	0.02889955	0.00912084	0	0
5.66182947	5.70533252	5.50353771	5.82070527	7.02955264	5.359027	5.11895942	5.56175587	4.8716354	6.31277114
3.96611438	4.60284956	4.47738415	3.84782261	4.56316257	4.4514362	4.21753869	4.09841557	4.22853202	3.79598487
2.01617156	4.03254917	3.37424363	2.36727482	2.87562674	3.05259973	1.66203768	2.6623443	3.55050924	0.99281591
1.0182692	0.54459361	1.48741825	0.41537817	0.4453779	1.36979686	0.53740819	0.69969693	0.54423776	1.48120531
3.64983845	3.63788279	4.42411274	4.36414157	3.7847171	3.80671093	4.24407631	4.55392656	3.89316203	3.77378139
0.43043537	0.16874794	0.02555301	0.36869054	0.02508496	0.67033868	0	0.25864775	0.26214676	0.03108805
2.21264413	0.76883912	2.00993749	1.03951368	1.09207378	1.48148907	1.87470842	1.25142755	0.7420706	0.18059399
4.63533921	4.96624718	4.63957379	4.10121207	4.12138731	4.58731942	4.80085389	4.44002659	4.12093425	4.48027218
2.65244002	4.08839465	3.57321554	2.68649336	4.51838989	4.8555811	3.28148008	3.61531283	3.24841758	2.70061511
4.28627939	4.31586682	4.24581266	4.02857745	3.97275576	4.68123203	4.15089551	4.20119663	4.2080975	4.03309757
3.79736607	4.41927632	3.70870485	3.25178508	4.10753725	4.03434246	3.9105485	3.92028531	4.67538613	3.31960984
5.92756716	4.9069185	5.41749721	5.79818947	5.81712475	5.4180549	5.53713654	5.16818218	4.56679624	5.56139435
0.94519183	1.81707922	1.54845351	1.12004142	1.19832871	1.44583994	0.89000146	1.62846223	1.76037699	0.77683069
5.34798051	5.35682683	5.61942964	4.28292275	5.38308007	5.20289315	6.18518688	4.49092589	6.11993302	4.96115005
3.66683348	2.65119399	4.15201307	4.69857156	3.82845827	3.44979988	3.12586999	4.92433954	4.02022333	3.57673921

1.84060146	1.78545399	3.32573517	2.71526004	4.42951809	3.13482639	3.6766658	2.96449983	1.2300658	3.27497032
2.12742465	2.59988917	2.83880964	2.44151933	2.46271504	2.34941106	1.66006703	1.82707716	2.25244269	2.48285738
3.72362384	3.95964258	3.50053284	3.45736068	4.19547362	3.43511512	3.57284766	3.46015566	3.22618765	3.99557603
2.5479978	1.93474115	2.11056411	2.16127751	3.24565927	2.04394986	1.93591527	1.6234234	1.89077784	2.28907325
2.38185898	3.10684667	3.02059145	2.54001544	3.36597183	3.04001441	2.30981431	2.42963068	2.64559804	2.87245355
1.298079	1.80422235	1.39629557	1.43141116	1.82555242	2.03379005	1.14732922	1.32008124	1.61924169	1.49047466
3.66524371	4.10608919	3.35215589	3.65106931	3.58689916	4.00190346	3.11621872	3.39784584	3.25734105	3.60701018
3.69662077	4.33237718	4.0068889	3.39997212	4.32309232	4.69432173	3.4548289	3.82434811	4.52676217	3.76371265
3.96824548	4.25723881	4.42380933	3.41235812	3.79561482	3.31315892	3.75802054	3.88324895	4.18324096	3.72496518
2.73693717	3.66225482	2.77894934	2.45618897	3.10380723	3.05576131	2.6339791	2.45266834	3.606128	2.76327665
1.44606298	1.89572171	1.50880252	1.29679095	1.64083254	1.63920254	1.47893856	1.42334737	1.79059883	1.56289104
1.58792774	1.63932222	1.8822562	1.55660769	1.44197425	1.78238916	0.93949752	1.72200096	1.37623384	1.79737605
2.73202125	3.17045746	3.13655929	3.19570424	3.12489451	2.7131392	2.24988428	3.43416166	2.99135814	2.80435071
2.84254572	3.23131906	3.89011427	2.84052689	3.59909167	3.22847302	2.49436144	2.81265824	2.77898122	2.3793699
4.43678742	4.78131842	4.51777296	4.34587024	4.61319944	4.62525401	4.11161544	4.39237044	4.48897691	4.60948885
2.9541068	3.88983013	3.18503864	3.42893517	2.97197788	3.4768339	3.42815045	3.64844611	3.41143308	3.86595463
0	0	0	0	0	0	0.0072131	0	0	0
1.68244984	1.84630812	2.99368807	1.70401139	2.40093421	2.24202012	2.16855008	2.5395438	2.04965255	1.1052788
0.67266647	0.62580105	1.43150364	1.67475518	2.32471666	1.07216427	0.5999814	1.74807621	0.62540272	1.79923506
0	0.00874725	0.00881795	0.00218206	0.02723287	0.00512679	0	0.00807653	0.00656027	0.02140363
0.01203637	0.19093747	0.02692877	0.06742505	1.01794196	0.39583176	0.07514708	0.31212159	0.17127639	0.64552808
2.61735479	1.84287139	3.51951797	3.70798903	2.65147689	2.98251915	2.22691124	4.29300761	1.8970438	2.57218105
0.00924268	0.01762174	0.28652415	0.30154094	0.26623857	0.03418652	0.0124775	0.18605089	0.01848278	0.13442742
4.11842627	4.41555952	3.89291385	2.65589465	3.99872857	3.44704316	3.56312396	2.64746248	3.76174332	3.51578918
1.20987925	0.54162329	0.68362278	1.27603467	0.48480824	0.38073494	0.06475945	1.5308677	0.14858488	0.33209473
0.55961373	0.66068658	1.59879836	1.9050651	1.06684764	0.86884743	0.71949091	1.81117872	0.55592128	1.017375
1.74319339	2.60766827	2.25128529	1.76185424	3.37056011	2.36229509	2.10374086	1.71430329	2.54617845	1.60138764
2.13561909	1.8015786	2.33185685	1.39220031	2.1094567	2.49530365	1.24708951	1.51445471	1.65202415	1.88446166
6.16813998	4.24071546	5.54296099	6.60956222	5.32129093	4.71261674	5.08651936	6.42923877	4.31881965	4.13681501
3.54142735	2.78883779	2.73222679	2.85575609	2.88962732	2.99150319	2.70125514	3.01256809	2.84631429	2.76154623
2.66960039	2.70337659	2.3949616	2.79366563	2.72149738	1.92291547	2.03768627	4.47847451	1.33115351	3.35706638
2.10072445	2.01345213	1.95593354	1.2781298	2.12553083	2.15359671	1.23756925	1.64423742	1.89231047	1.52000728
3.02103505	4.19552291	2.60567401	2.62527216	3.63399643	2.92008243	2.9411217	3.11481596	3.00254305	2.61579592
0.02775054	0.00254898	0.02549257	0.0338874	0.0225425	0.00893272	0	0.02798747	0.00572439	0.22588892
4.15118602	4.85940116	3.66556477	4.20944265	4.55398093	4.12028719	4.34948112	4.12031545	4.44912696	4.50925748
4.49970088	4.6837409	4.81338308	4.7706299	4.51191219	4.53700467	4.53864666	4.41627823	4.31640104	4.44052587
3.45274897	3.40079768	3.3976319	3.26166621	3.07347978	3.22317795	3.33573221	3.39614877	3.67932726	3.2078103
2.28543732	2.81539855	2.85642816	2.4630767	2.97535385	3.25841831	2.05013673	2.77146193	2.424619	2.95697565
3.9005654	3.48097726	3.22185053	3.53383401	3.79899955	2.9504294	3.64385021	3.18868277	4.21678883	3.67875071
5.69720722	5.42680738	5.19156277	4.80085639	4.87900667	5.8302691	5.4677414	4.74638716	6.19027344	4.48802293
1.61854077	1.72866017	1.65553879	1.40100303	1.8454098	1.99623825	0.95071221	1.38677785	1.64717994	2.29716221
1.23873439	1.75355174	1.7038401	1.81892435	1.43549171	2.01677121	0.99159105	2.35248474	1.49278138	1.7646134
1.46574673	1.92428135	2.08693513	1.69429888	1.90107517	1.70260703	1.47213907	1.88122446	2.59222308	1.54261104
3.13021403	4.57108435	4.47549227	2.93427123	3.92674523	4.25592967	3.88121281	3.10763826	4.01059564	3.02072401
0.69168009	0.1443837	0.03963583	0.49233369	0.01866109	0	0	0.16849458	0.00426129	0.06387685
5.64883808	5.62369327	3.98676861	5.06281169	4.50059515	5.76337492	5.63977282	4.59783656	5.684342	5.46067394
3.43102074	3.56736538	4.32447267	3.67923682	3.26876687	3.78725352	3.78378594	3.82263766	3.60380508	3.48266459
4.11692452	5.60227894	4.02831522	3.9198506	3.73041535	4.97268676	4.87320543	2.90014416	5.27933182	4.565532
5.31935086	5.14475146	5.48083892	5.41320543	4.36822545	5.47996717	4.78355872	4.75822185	5.60483039	5.80949818
3.8728699	4.93621942	3.67051424	3.96865271	4.51131193	4.5395121	3.58813694	3.94053664	3.64497813	3.64613122
2.96018592	3.95209634	2.71924966	1.86390609	2.67531275	2.34444806	1.98144165	1.99899396	3.26641213	1.97039992
4.23966691	3.36727946	3.54771497	2.45031298	0.79969693	4.94454929	5.11171695	2.74440688	3.23206692	4.07268831
0.92628586	0.66653412	0.92202193	0.85716322	1.05285146	0.89854564	0.52039059	0.96933511	1.01516495	0.47229111
1.96942652	2.50327954	1.99544337	1.69127512	2.21716799	2.23630737	1.09834117	1.74642019	1.75933406	1.78708854
1.74879342	2.00929917	2.41508065	1.65918062	2.60741414	2.23012568	1.59818538	2.02509455	1.97840593	2.06422501
0.56768012	1.63396078	0.97426403	1.10374837	1.68148886	1.66861001	1.0110544	1.08150171	1.24521375	0.74980303
3.60778285	3.25631941	3.2635662	3.79231068	3.98602602	3.38525194	4.0532609	3.26854719	3.34585449	3.49902373
5.28960078	5.57215787	4.62063805	5.51496374	4.66872326	5.13623704	5.09488367	5.46824906	5.6334578	4.77918775
4.05755579	3.7888136	5.3837336	5.02852977	5.59681219	4.5923431	5.31810947	4.26838857	4.61215074	5.03458177

4.77789784	5.35685533	4.38738305	5.21708737	5.44553862	5.06620232	4.96348875	4.13981404	5.82499755	5.61001291
3.12895221	3.01780669	2.32975515	2.86468203	2.55553981	2.92450806	4.34524116	2.33510278	4.46664546	2.75299659
4.06201635	4.94123535	4.8114691	3.96951026	4.55326991	4.93174823	4.46652174	4.1013897	4.3959261	3.85760214
2.04317153	2.69282176	2.05255176	1.64860936	2.23031114	2.57604375	1.56887464	2.02938375	2.2284016	1.99119267
1.94824033	1.94354498	2.00392056	1.36307716	2.17867995	2.61900431	1.73413655	1.91915501	2.1121489	2.02196914
4.79217555	5.29269392	5.05288537	4.84707047	5.47579054	5.4805553	4.24165354	5.26346488	5.26607044	4.79514171
3.22601239	3.66107251	3.22414066	2.63608811	4.28670792	3.50249205	3.58038337	3.19166711	3.59102811	3.02618509
2.08743986	2.2493667	1.8256417	2.27413018	2.41836595	2.98019351	2.04004666	2.38606632	2.24957612	2.12107823
1.72454449	1.90203062	1.46644257	1.51768516	1.98002977	1.86056116	1.69657279	1.77314622	2.23891813	1.38171574
1.8786368	2.33469194	1.98881601	1.61903514	2.13785016	2.170748	1.54538458	1.57741757	1.8935154	1.73364162
4.32386838	5.17279405	4.54375477	3.67368826	5.04174497	5.11178574	4.09394268	4.37641639	4.74454836	4.10887153
4.13041401	4.55144731	3.95988024	4.17645318	4.62762923	4.21109847	4.00913152	4.06226397	4.40216786	4.19533944
4.07019484	3.89884083	3.89785555	4.13828054	3.77356106	4.1099658	3.63197869	4.20524309	3.90467695	3.7274866
3.83057308	4.36944986	4.20911456	3.47630758	4.54912611	4.79655508	3.04600516	3.42716376	4.12044001	4.0775558
2.98866387	3.02282829	1.93622695	2.55270807	2.86390119	3.23629523	3.11394862	2.5784484	3.00674857	2.11021439
4.75673381	4.88627511	5.32166971	3.66587746	4.1928835	4.27176746	4.73467413	4.67775362	5.10800681	3.48841379
2.62247681	3.08461627	2.59809459	1.91847869	2.94204227	3.12949747	1.65253763	2.61700469	3.16220412	2.23144837
4.02748357	4.82186473	5.24006343	4.12845486	4.74510368	4.92250386	3.58474138	4.72443827	3.98680438	4.27725104
3.99159033	4.32423484	3.66078675	3.92575182	3.74304655	4.02584413	4.15502305	4.00347809	4.66049311	4.04396701
3.39501092	4.1411891	3.25933429	3.42538631	3.4905796	3.62598579	3.10795452	3.66566516	3.69352578	3.03260355
0.41828916	0.50402336	0.29801659	0.37453252	0.44785196	0.52294316	0.36382418	0.50113491	0.64640902	0.51858337
3.57933615	4.42321066	4.05516859	3.77803103	3.88164447	4.60459919	3.80855213	3.83131843	4.32556725	3.91230634
5.24109574	5.87771319	5.64088777	4.91192071	5.59813149	5.1458057	4.49140295	5.34708977	5.47301136	4.88025852
3.21645327	3.91287027	3.5794723	3.18390331	3.67267517	3.64892198	2.85712088	3.19822898	3.00928062	3.01460126
2.0136716	2.80559984	1.62337978	2.37498255	2.22863005	2.43728776	1.85078841	2.41084171	2.92884295	1.93643776
1.75926249	2.37667218	2.2648918	1.53653012	2.2164146	2.43179747	1.90013599	2.06023033	2.22417391	1.90321835
3.98177609	4.64135867	3.87480366	4.13810618	4.31969148	4.28445956	4.651619	4.16650733	4.64839517	3.95368508
1.67696403	2.29095837	1.56461378	1.45026112	2.26572625	2.07050021	1.60951413	1.71175692	2.20667273	1.80321102
4.24719951	4.696188	4.64681046	4.06797944	5.43580697	4.8524447	4.7104419	4.35619878	4.58454027	4.74045599
2.34122251	2.70392652	3.2500007	2.1102327	2.55769954	3.26398749	2.08706001	2.37631592	2.20259923	1.92372441
3.22890564	4.4632622	3.6483238	3.1059943	3.70225953	3.72343897	3.10455852	3.47458046	3.93817785	2.87424941
1.97904246	2.81173096	3.56914728	1.52892216	2.59241993	3.4446653	1.97141797	2.01793172	2.8502069	1.6291133
3.09186451	4.5694785	3.09713021	3.16704815	3.37780823	3.11450171	2.7502662	3.51017261	3.97807628	2.96147591
4.70593403	5.53233733	3.8717185	3.57354347	3.58005886	3.67909133	4.09468509	3.01780047	5.07758668	4.07299956
2.24268688	3.02038466	2.92260139	2.20584536	2.55962353	4.87559132	3.33127237	2.03375085	2.67168201	2.5779625
3.47495567	3.7280657	3.87958014	3.81889926	4.16146175	3.92539029	3.61097247	3.86192846	3.62062458	3.98654915
4.27727789	4.03404263	4.50567465	3.64477551	4.29693977	4.43270131	4.34148684	3.27313554	4.3941578	4.57640672
2.74068075	2.6467163	2.61418432	2.78500125	2.7905102	2.935627	2.16201153	2.90776085	2.75121503	2.76500375
0.30223196	0	2.59249272	1.10665718	0	0.04932421	0	0.07703411	0	4.50132341
1.38953662	1.26019391	1.66272992	0.62456002	0.55523189	0.40784226	1.03699203	0.07247588	0.52523525	1.59607286
0.04083316	0.0261591	0.05226517	0.03889162	0.07630604	0.06052194	0.03076656	0.13926422	0	0.03207914
0.02380306	0	0	0	0	0	0	0	0	0.01867639
4.05041952	4.86762995	4.70524994	4.98274801	5.27313101	5.11495557	4.28632523	5.0477997	5.05702792	4.29857767
3.99619899	4.05569157	1.38619428	3.96538087	1.27942986	3.98039541	4.8052107	1.01444603	4.33133693	4.29337881
4.78032327	5.48277101	4.8472528	4.04261447	5.23054753	6.05609484	5.02615358	4.09206568	5.69825547	5.27155352
2.24001334	2.19223354	3.14050145	2.20859088	3.19092929	2.86442188	2.63583289	2.47129629	2.78490871	2.61218419
0.78873265	1.20178713	1.80246237	0.61595445	1.36203301	1.22364512	0.76839452	0.64286731	0.96305475	1.07120982
3.5910115	2.72801007	3.38273959	2.18591683	2.81780218	2.48647363	2.38434403	2.13062115	2.87128858	2.35062792
1.57913689	0.66029113	1.10036762	1.82147367	1.1918627	0.51064268	0.9424624	2.107792	0.85111832	0.70807084
3.83952735	4.08391722	2.80646544	2.69982795	3.08426456	3.0030085	3.53381116	2.40845782	4.24659616	2.49936201
1.84768838	0.99553744	1.7731858	1.19787366	1.37131681	1.55975661	0.96670646	1.542857	0.92869363	1.12056292
1.94147009	2.25249272	1.98236823	1.75246622	2.48706192	2.00212853	1.2068556	1.74331249	1.55266312	1.49870858
2.76370469	3.5416593	3.21394915	2.31264547	2.56712055	3.62728961	2.35162798	2.35134424	3.20957968	3.00754985
2.57261033	1.90099824	2.84196355	3.2016231	2.55375941	2.47403293	1.70904121	3.47293456	2.09543019	1.93655852
2.03417961	2.41108251	2.5860627	2.18192801	2.66265448	2.49532535	1.95817516	2.28074623	2.18852874	1.94854045
1.36129379	1.171113	1.09584915	1.4248366	1.20501759	1.24541244	1.90925002	1.4964964	1.16868338	1.3563791
1.69017641	3.97578966	2.49362327	0.99973507	1.81318654	2.81383347	2.39072116	1.21007414	3.24820842	1.41038362
1.7977727	2.36505286	1.43282287	1.39415945	1.54415661	1.99551767	1.19673133	1.90892755	1.67268056	1.37291366
0.56385036	0.2096516	1.79168329	0.76104944	0.71969385	2.17677159	1.26776216	0.52290813	0.07601046	2.62520312

3.51400378	3.61866222	2.95021367	4.26264817	3.03886343	4.83791563	2.91995723	4.1371809	4.79452641	3.19505394
3.37706964	3.80408932	3.43506212	2.99185735	2.87017666	3.96807749	3.78503958	2.71350408	3.37492436	4.10007132
3.61837583	4.06398504	4.16551178	3.60233846	3.54278802	3.85898853	3.2555282	4.04869981	4.21040755	4.16174548
2.50999208	2.65899147	2.16779456	2.29616684	2.24264314	2.4997881	2.35310065	2.25326876	3.02467109	2.51931385
3.25896472	1.81824284	2.13773955	4.19052939	2.60073068	3.39499465	3.96652517	2.48866613	4.07176888	1.21225986
4.71757099	7.72483685	9.00134534	11.2044031	4.42155397	9.4132115	4.57588819	9.81093335	5.09857404	9.82059987
3.387113	4.34169418	3.30910139	3.24204318	3.24872695	3.92089823	4.24516256	3.51738575	3.29456329	4.25143445
2.81047245	3.65306484	3.47945818	3.09884675	5.13830408	3.98291012	3.07004495	3.84012265	3.21300574	3.07355826
1.50711701	2.43325985	1.48073637	1.64193316	2.65154446	1.74672228	1.07010436	1.65935342	1.68824967	1.94681205
1.80381452	1.64625081	1.48339464	1.59661444	1.48998556	1.5913187	1.43149952	1.54415967	1.13858283	1.23950885
3.16771817	3.88793266	2.83647733	3.31759925	3.20721276	3.04054263	2.91042397	3.39765585	4.27798663	3.13581373
1.97489386	1.90575485	1.54327262	1.19656237	1.43575664	1.83446597	1.57993097	1.27822573	1.9396633	1.50066198
4.575778	5.01493956	4.34219629	4.92071469	4.62187881	4.44921632	4.55797937	4.72912653	4.61141667	4.72586858
0.52753819	0.09892939	0.24054652	0.3085793	0.43151666	0.72240981	0.76702669	0.43902065	0.16308919	0.24340925
2.34880951	2.97151211	2.27396011	2.16089966	2.61793007	2.64029634	2.00805346	2.42757943	2.80096941	2.09324031
2.82638996	1.17639213	1.83416962	1.70834849	1.86023466	1.64384404	2.49002747	1.84463478	1.19644332	2.11744027
3.0436686	1.80380434	2.73828474	2.58219712	3.32469793	2.70775832	2.55008135	2.80287341	1.98166195	2.64402116
0.36283781	0.19789258	0.41088334	0.92460815	0.91078192	0.2874585	0.16195546	1.06365506	0.131735	1.26245592
0.82244357	1.26994748	1.6230989	1.0539853	1.6059391	1.5571837	0.69327533	1.86883866	1.10839935	1.30447146
3.05485561	2.79648815	3.08516903	2.83999579	3.79823186	2.77887478	3.42251433	2.7384416	2.66587313	3.62627282
3.78465354	4.31754225	4.3837527	3.50699743	4.11506893	4.42913135	3.36492103	3.62281031	3.74527576	3.43755658
5.03380571	4.73114481	4.62676383	4.20701983	4.85042462	5.40550848	5.59498668	4.29160799	5.05375857	4.83569117
4.20219218	5.25969335	4.85631963	3.85687995	5.93584821	5.78557346	4.9186468	4.949776	4.89124712	6.77517719
3.7827257	5.38849155	3.20174969	4.28873786	4.00091828	4.99320462	3.65966858	3.1590718	4.43652329	5.51467412
2.55959038	2.95117626	3.08781389	2.79734716	2.93745776	2.42044315	2.53296454	2.93370172	2.75950397	2.64158633
0.84298017	0.76837041	0.71970903	1.78691665	2.05313643	1.20172885	0.82408235	1.52007216	1.05792247	0.6284198
2.59136654	3.35479257	2.50008786	1.57911848	1.91877059	2.98372485	3.0962613	1.6452733	2.9675662	1.96339904
1.19989146	1.76450161	0.77984037	1.1488948	1.59887991	1.8331833	1.13818588	1.40266552	1.82869495	1.01995808
3.88128642	4.0609726	3.6976924	3.75392868	3.49103106	3.09652756	3.68139342	3.62985471	3.87554218	4.03600402
2.5889277	2.33877486	1.6093738	1.56524799	1.68784388	1.81407258	1.73047675	1.76871396	2.21014195	2.65583269
4.66997919	2.17759321	1.50791078	2.30943515	1.34028804	4.45623421	3.25158977	1.86476553	2.8300295	2.49633398
1.4171308	2.51058433	1.23389388	1.46424204	1.83365674	1.83485182	1.30322338	0.90300355	1.63626299	0.92326223
0.00399033	0.44186905	0.11351884	0.13080494	0.08561064	4.11324655	0.52694455	0.19744198	0.15717018	0.06127319
4.5124838	5.09360547	5.00179081	5.00063733	4.48807382	3.44851082	4.65603458	4.77283254	5.77959162	5.34996845
3.78964008	3.41234932	2.10499291	2.72621649	2.34721315	2.6517893	3.48434085	2.85374041	2.66317475	2.45231534
4.02306091	4.98913424	4.38209539	3.62091227	2.91579859	3.01902028	4.00629713	3.2409185	4.00780445	3.75438831
2.38830431	2.56272901	1.74534157	2.33812618	1.70665803	1.91522172	2.34361851	2.42636265	2.73861864	1.96362452
2.94818091	3.64812227	4.13329567	2.92971876	2.64223096	2.82515299	3.46524833	2.72813388	2.71159067	3.96121292
2.59568011	3.30978077	3.69570741	2.59707083	3.42922516	4.00356947	2.16103859	2.51698157	2.69217164	3.2133149
3.42813958	4.41549242	4.06141942	3.0969022	4.5162587	4.03562935	3.39243132	3.4513654	3.89399072	3.36572215
2.95446691	3.57845502	3.68745974	3.39810677	3.94336416	3.68659477	3.07206722	3.52364174	3.21573282	3.15399823
2.15608741	3.29396634	3.22513973	2.40492769	2.65791592	2.85096688	2.25419842	2.34732464	2.82015816	2.65954547
3.27505755	4.38375093	3.38539264	3.32641011	3.82715292	3.4156824	3.40567797	3.50556954	3.81373635	3.21450202
2.96562773	3.31469901	4.44449398	2.88114403	3.10918567	3.5108323	2.80940381	3.07430961	1.2752739	3.19834283
1.88115908	3.49905197	1.94868993	1.84329998	2.7173355	2.56753385	1.94081625	2.64305598	3.16874122	1.82724078
2.3875327	3.45813287	2.87087589	2.41491639	3.53675583	4.07860203	2.4534738	2.97715144	3.47497503	2.76094258
1.95535834	2.65853208	2.19232482	1.89028979	2.73601205	2.66028209	1.1551751	1.78770468	2.05555948	2.30545075
1.8494423	2.3327159	2.41550902	1.80922471	2.16195607	2.5125227	1.65524789	2.14419828	2.26359089	2.59728605
2.50513019	3.13554507	2.48075631	2.46173803	3.36256224	3.01417588	2.38054736	2.93328758	2.94012482	2.64117062
2.89694549	3.54828574	2.87823557	2.99470432	3.48653423	3.92550746	2.63970572	3.44616992	3.16014786	2.86898916
2.35794266	3.41230469	2.84514115	2.62335192	3.00247716	2.48649689	2.12700854	2.71745663	2.87222999	1.82677234
1.22444933	1.82135707	2.07027426	1.47826457	2.20217023	2.22247607	1.391488	1.71467321	1.47712646	1.27286192
2.16519393	2.10579431	2.2418502	2.10318624	2.22915414	3.27436632	2.28455421	2.16749515	2.36279742	1.82806652
2.65600542	3.13740687	2.98841394	2.86111409	3.12834738	4.4156306	2.41401493	3.13043303	2.53455248	2.67350402
4.59983763	5.54116866	5.11367919	4.20880235	5.67262961	5.47416556	4.40147935	4.6432496	5.09178556	4.27846391
1.1463417	1.44784106	1.1646019	1.20492871	1.35081383	1.31108526	1.45036377	1.0373197	1.39140021	1.0733335
4.47631106	5.98181757	5.65099771	4.08375127	5.00630582	5.16881512	4.56020491	4.64729231	4.85263941	4.34968841
1.97449687	2.94200759	3.37502436	2.16857777	2.42938038	2.70678422	1.87531402	2.74725001	2.00863505	2.80673244
1.38812439	2.01280256	1.6632896	0.88804718	1.48732572	2.22942611	1.60776318	0.74425985	1.65076448	1.19636328

1.84160067	2.26455471	2.60240523	2.00142327	2.60462042	2.51124993	1.73443398	2.51585433	2.03575307	2.21945498
2.70207075	3.28122932	3.39333047	3.07454162	3.89021601	3.54380158	2.35640155	3.14340975	2.87010783	3.21951608
3.3124774	3.50013589	3.03917545	2.7679932	2.84162638	3.49282454	2.98374613	2.9168786	3.70404094	3.25830371
2.92102531	2.85075354	3.49752348	3.5515417	1.73556106	2.50966581	2.79890258	3.20352887	2.1274521	2.59127534
1.6611675	2.24573731	2.45583491	1.51311898	1.87596723	1.63597302	1.40941926	1.89855556	1.95032638	1.40158116
2.32755924	3.39299862	3.09651169	2.44999075	3.29182731	3.1651333	2.23094765	2.50890479	2.25616431	2.46586258
1.58422303	1.88970096	2.70639737	2.98869129	2.40998212	1.68810977	1.24432905	2.94489049	1.48422206	1.64389256
2.21920061	2.60219429	2.42553881	1.86902401	2.78928201	2.68012673	2.13110415	1.87333673	2.21142649	3.04110775
1.3664301	2.01823109	1.62591596	1.48410502	1.83190976	1.5856593	1.75101549	1.57582611	1.89707845	1.40424467
0.56716118	0.26984048	0.51001119	0.51974432	0.56424041	0.41833038	0.15947777	0.91631138	0.30142861	0.25465194
1.14614291	1.3315875	1.23523425	1.82934916	2.40971328	1.41283629	0.42395057	1.7567518	1.24533188	1.12745431
0.28479681	0.20762672	3.27207152	0.42106306	0.23125528	0.39470747	0.49848896	0.5107525	0.56298318	0.39492135
1.22069707	2.04632873	2.44044194	3.29721636	2.07570061	2.36444145	1.0348609	2.86972765	1.20415272	1.9574473
3.79870053	4.76495108	3.95921377	3.28100951	5.08497591	4.91985207	3.91917341	3.63183033	4.58518083	4.71349054
2.84781782	4.83025976	3.28394268	2.76408188	3.70398337	3.64251053	3.57204546	2.82507588	3.50752826	2.80918703
2.39523558	1.82308374	3.39033785	3.24412344	4.03131328	2.26142028	1.41623035	3.73939365	1.50564171	1.65858285
3.36966367	4.0234367	3.96994811	3.04461658	3.6363459	4.34005568	4.05394893	3.45286175	3.79029398	3.91728359
2.01026725	2.84645536	2.2810051	2.2054937	3.21982452	3.04108389	1.55987743	2.22932639	3.06448605	2.93345149
0.16474864	0.11159167	0.18743967	0.55506057	0.12789628	0.09221012	0.0195926	0.64174313	0.07849804	0.14529139
2.04972076	3.53152164	3.55000127	2.1508664	2.82567206	3.55686253	2.76837647	2.1488164	2.95948712	3.52467771
1.82020427	1.13456958	1.75703309	2.46751804	1.96388475	1.2331076	1.30524317	2.66061135	1.38842061	1.1437335
3.54649034	3.97966973	4.52027101	4.36170004	4.17444315	4.03932595	3.54891345	4.48027202	3.51053894	4.48702729
2.20200417	3.58057422	2.79200714	1.37081326	2.97258772	3.19056043	2.72447212	2.59216958	3.18137886	1.79013386
2.83923833	1.32960438	2.45332793	2.29347983	1.5931217	1.58691891	2.12462609	2.52693231	1.48719059	0.9673361
2.45711181	1.95582515	2.96162185	2.25327105	2.28831992	2.15588145	2.35407594	3.29929906	3.03324461	2.44682997
4.4057618	4.46245149	4.20936365	4.23471238	4.39018503	4.55823667	4.38345841	4.38851282	4.15169813	3.85639679
0.01642293	0.10650677	0.04698354	0.08397239	0.08593019	0.01227233	0.00618739	0.25742206	0.08054661	1.72441652
0.08056384	0.56513486	0.12730903	0.14968473	0.33432091	0.14683353	0.06685866	0.72139455	0.42829907	0.39984837
0.15112843	0.10759253	0.56114066	0.83013731	0.44591396	0.22010139	0.13685358	1.0646205	0.15156921	0.36226448
2.68169026	2.8237085	2.014544	2.36108559	2.55108916	2.09007027	1.9453732	2.38350452	2.7017911	2.09227698
7.77587977	6.72227651	6.43988402	8.79970849	5.35557693	2.84948509	3.06440754	4.01633347	5.85535731	1.7038922
0.1922302	0.19864611	0.16129563	0.18353075	0.31868366	0.36020669	0.23130955	0.17093467	0.07184427	0.05270892
0.57304186	0.91057871	1.48715453	2.12917944	1.00266321	1.75785671	0.09499377	1.98537397	0.38314364	0.91953453
0.26256334	0.88562636	2.05889126	0.54621977	1.38106576	0.14664147	2.02175801	2.9502682	3.46720385	0.57204577
2.59585747	2.34534742	3.66265651	4.66203154	2.60942261	3.41518035	1.91075753	3.90392983	2.2030948	3.45433768
1.67210837	2.0102093	2.28511534	1.22717024	3.21528515	1.95992836	1.08155039	1.83301161	1.78347785	1.77962428
3.06747166	2.9792272	4.36841538	3.51075145	4.13877261	2.88945307	4.19026822	3.06746156	2.99383791	4.20672025
2.91941841	3.1423051	3.60528036	3.46245315	3.83876992	3.47521966	2.83992764	3.83806689	2.95170726	3.02830647
1.22646027	1.36334059	2.20243554	1.0040074	2.60221389	2.01282259	0.83798297	1.10223255	1.56306406	0.6733514
0.87852416	2.83851168	1.97302583	0.66844149	1.91202585	1.83855126	1.07103551	0.843502	2.34979551	0.62815809
0.0800322	0.33174712	1.05601596	0.2691597	0.27244287	0.05357665	0.39405114	0.37664739	0.48502491	0.13656018
2.68167142	3.92622118	3.63761843	2.9085473	4.06422934	3.81963491	3.34687501	3.22554153	3.3726203	3.57399884
0.87165082	0.74003197	0.66845786	0.57497934	1.02785247	1.29275404	0.42082248	1.13055188	0.81990365	0.66868483
0.04994018	0.04832172	0.060631	0.07157719	0.55160394	0.10949046	0.0260756	1.55973142	0.00751302	0.18158437
0.08670833	0.06406042	0.05354462	0.08665772	0.35207402	0.36612967	0.02988329	0.23034751	0.13148583	0.06333413
0	0.01588232	0.02658608	0	0.02091706	0	0.00625742	0.02918692	0.07007896	0.00652674
0	0.013437	0.02696486	0.06888765	0.01329631	0.04665144	0.00792965	0.04900515	0.04482243	0.00827075
5.59001383	6.32917606	6.0337778	5.01647659	5.56513492	6.27925616	5.66543348	5.28588625	6.86549357	5.53895189
3.35481968	3.78676661	3.24645907	3.12757616	3.67651513	4.18814469	3.27414004	3.24480515	3.72212435	3.22336921
3.1546677	3.68353917	3.22032915	2.90722892	3.32739428	3.90982729	3.15345277	2.97407129	3.17853544	3.15457069
2.16351547	3.6383853	2.48454792	2.51847424	2.5931902	3.44675981	2.48267827	2.42333098	2.06835637	2.84847333
4.97714781	5.07096426	6.37103093	4.97641927	4.69658425	5.41381782	6.03956343	4.91199772	5.80577857	6.02648631
3.0606645	3.79260609	3.51730886	3.29839481	4.07370996	3.98912717	3.70809886	3.29497755	3.68444494	3.03923125
3.14693254	3.77947211	3.62140868	3.44365345	3.83146599	3.35089132	3.24337407	3.2885317	3.62892077	3.34447822
1.38749628	1.24786004	2.02475433	1.26196738	1.54623644	1.50472164	1.24252558	1.40574434	1.55916186	1.6406943
2.86657478	3.04154434	3.99385436	3.26139423	2.84081778	2.76322193	2.93294824	2.98705407	2.70439357	3.92517367
1.75102806	1.38899393	2.26800575	3.53737024	1.32901533	2.60699112	1.16542355	2.9117913	1.21949697	2.40946284
2.28958425	2.24774976	2.63160027	2.46522128	2.22406828	2.73783407	2.44470651	2.48909807	2.55345178	2.47603209
2.95277747	2.56511764	3.66275489	3.44253562	2.85480837	3.14916724	3.23045886	3.03034427	2.88453323	3.80673759

3.8726248	3.89092115	3.92290998	4.65377448	4.49014548	4.10722086	3.93721569	3.90423033	3.79099248	3.75750242
2.06962553	1.94133402	2.35992014	2.35656743	2.43519882	2.09367979	2.3117106	2.47290065	2.09148834	2.25376556
2.65604038	3.57046094	2.5963604	2.52248172	3.05835534	2.83621386	2.98014619	2.84439371	3.45791455	2.36824394
0.95409689	0.46188182	0.64547542	1.42785878	1.85590947	0.51302169	0.53796045	1.33400147	1.80843082	0.80065939
2.22015498	2.62685929	3.4612983	2.25163616	3.17813601	3.03716474	2.11743795	2.69324667	2.27557873	3.10389784
1.96419201	2.90089088	2.39031253	2.48223869	3.01569939	2.58626125	2.02553608	2.37099368	2.21284562	2.26495012
3.92311955	3.16801018	2.33240574	3.39387875	2.91428794	4.27992464	4.79832913	3.79586123	3.89922354	5.08887418
2.18699826	1.9012549	2.08715045	1.65721725	2.04630829	2.04786092	1.90363464	1.71774747	1.38266015	2.00466308
1.58242552	1.34824432	1.38067605	1.3193548	1.38501765	1.15209207	1.64654212	1.51484964	1.73613149	1.51994639
1.169559	0.97878774	1.35736206	1.49757166	0.78150609	0.70872416	0.45664285	1.56328485	0.61414893	0.64146436
2.66579917	2.79887402	2.55914251	2.28277065	2.52027285	2.98068828	2.70319091	2.04187016	3.14883206	3.11145939
2.13289421	2.5939844	2.86495	1.46630159	2.47257241	3.06897912	2.05904481	2.36651174	2.86342062	2.77575094
2.24846646	2.82709436	2.23983367	2.04253696	2.06428707	2.41160651	2.03163698	1.89510805	2.26817363	2.173942
1.69448161	3.472671	2.68454235	1.73628175	3.33006236	2.72508611	3.18760408	1.88012218	1.55628355	2.0473752
1.64168913	2.00072822	1.67553122	1.47873603	1.97625886	2.11750911	1.70235787	1.48534575	2.48940512	1.64018114
0.74289816	0.75109655	0.99267865	0.75456223	0.93754233	0.91303024	0.42015725	1.05463101	0.81764	0.55792767
3.23260305	4.68168237	3.79036684	3.91661029	3.73044128	4.02735123	3.63186341	3.49349913	3.84733238	4.21693817
2.87442777	3.07259792	2.14048484	2.74400355	3.17991543	2.66197999	3.21859995	2.6760541	3.30480731	2.90380155
4.4421432	4.89146625	4.77734057	4.37999696	4.9688507	4.7793798	3.39622982	4.23357234	4.60364047	4.99621682
0	0	0	0.02425253	0.01611519	0.00955531	0	0.02992444	0	0
2.62083187	3.21230576	2.49156846	1.97865682	2.65959804	4.75689827	2.63935828	2.40841316	3.04552452	2.47166109
5.0441043	4.97568031	4.44927507	4.69291497	4.64372889	4.79103226	4.7291959	4.88835975	5.26626728	4.73227856
2.32661201	3.05225305	2.73868259	1.90880105	2.71290596	2.77562124	2.52500278	2.01163186	2.94114599	1.98367436
0.82015763	0.97374356	1.11469254	1.10749666	1.01481583	0.90988691	0.67291809	1.17358448	1.04496552	0.94438052
2.55236758	3.21974305	2.55056403	2.86862874	2.82773381	2.36858312	2.26565532	2.68526743	2.95660176	3.31388175
0.70844126	0.34415999	0.86219772	1.87352278	1.78144575	1.90325277	0.4092017	1.02394338	0.32853814	0.30929371
1.74434698	2.15802106	0.97495618	1.21542351	1.60220259	1.69870283	0.64276241	1.16589423	1.69710075	1.98712817
2.30963013	2.64198187	3.37079685	2.47702222	3.04348906	3.08777788	2.6778183	2.46773283	1.99960019	3.69622409
2.33968899	0.5519813	0.27418252	0.47748373	0.18163153	0.18764034	0.0495484	0.81460712	1.3734526	0.03707917
4.75114083	4.70646371	5.62302299	4.999648	5.115748	4.64364609	4.84977868	4.59039175	5.01657375	4.95053735
0.56330088	0.33761414	0.5141592	0.26658714	0.5813338	0.90392542	0.08411745	0.74845243	0.2940912	0.41125984
1.90137625	0.93490388	2.60741201	2.14283646	2.43269819	2.51770255	1.85118206	2.8264583	0.52313756	2.35628698
2.52659418	3.54405281	3.33233478	2.84549753	3.38597656	3.49606436	2.80532918	2.74235213	3.17415408	3.21884964
4.19627287	4.22234786	5.07689147	3.97478897	4.08276209	5.12960222	4.72495746	4.13230429	4.08191469	4.54377356
0.42013627	0.43010941	0.93923878	0.55700811	0.65262585	0.26949577	0.09486228	0.79832027	0.72118695	0.2094647
3.12376393	3.96408256	4.33098081	2.76926715	3.66203989	4.66901921	3.00291553	3.51065787	4.02658153	2.78097243
1.91792234	2.51368632	1.88513604	1.8487113	2.22975565	2.46938344	1.46923015	2.48160509	2.56680748	1.67111038
0.62095114	3.02638852	1.78590366	0.9576852	1.59644334	2.22643818	0.42521943	1.94719448	1.87011058	1.35484901
4.27265969	4.28692354	4.39271376	4.18329037	3.87037731	4.32578623	4.9235767	3.72630578	4.17924146	4.13344531
5.95206973	5.60141143	5.28166347	5.51710906	5.23051692	6.44048667	6.19191352	4.99331102	5.80889085	5.44335622
4.04961394	4.70077009	5.46052303	4.63728187	5.09161652	4.77553765	4.18444919	4.15743477	4.79555069	5.86617454
2.36222054	3.00707325	2.5995998	2.18500623	2.91901475	2.94876359	2.3711098	2.10701071	2.90018592	2.38357054
2.34199798	1.40192236	1.32881051	2.44422157	1.19119663	1.1319605	2.11236464	3.02402324	0.48540235	0.8607686
2.44403005	2.35298575	3.31801791	3.37877385	3.07373945	3.07229587	2.1420973	3.72557976	2.0072868	3.05866134
1.88121244	1.26206107	1.60485952	1.00066333	1.81085494	1.27572222	0.61992804	1.50392444	1.24077577	0.42300112
2.41771442	1.684282	2.26110206	2.33805726	2.90083032	2.91463433	1.01674839	2.58049632	1.10268169	2.57201913
2.77502659	1.66449383	1.61664216	1.37990574	2.04405631	1.81554452	1.00147874	1.36308482	1.32897528	0.38144678
0.16288022	0.08534697	0.42162906	0.57890838	0.2213574	0.23679219	0.05337648	0.43467366	0.09559629	0.18719043
0.90765465	1.05511001	1.53875072	1.24970447	1.95553859	1.08511301	0.46114595	1.64364315	0.81429914	1.17786926
1.52032976	2.67917987	1.69351083	1.39094619	2.13006613	1.45041013	1.63869026	1.24781861	2.88015854	1.68586969
2.49483249	2.80703937	3.854534	2.97615529	2.57965918	2.70529025	2.48044966	3.68533985	2.85529947	2.84891181
1.33474571	3.05124447	2.78187623	2.05848151	2.36092258	1.76748012	1.51142714	2.01447183	2.19418408	1.8889561
2.86024323	1.63583117	1.90391658	1.39590084	2.12937674	2.01422769	1.04675469	1.91972687	1.4348391	0.57869589
1.98476273	1.81694264	2.59462971	2.36084694	2.12275382	2.84452106	1.73025187	2.95640799	1.93818295	3.12863071
3.62189023	2.99313697	2.43707782	2.65682358	3.37297599	3.03388466	2.78782128	2.56090562	3.27105482	1.35436121
3.12260785	2.0458905	2.12190669	2.02646564	2.14385538	2.27744605	2.11581119	1.99391449	2.46038214	1.20726262
3.20708127	3.03393352	3.75404615	3.23769709	4.79584621	4.08949444	3.95381156	3.66630278	3.67738271	3.60451511
0.74409854	0.17933638	0.22255112	0.70272555	1.27951831	0.14857386	2.04348982	0.78826949	0.15262942	1.02925845
3.25722642	3.61041497	3.98168359	3.73897576	3.79915922	4.05062638	3.34445851	3.82630944	3.20280838	3.19602051

1.86870696	3.57734497	3.98115054	2.73735739	3.21513674	3.59562363	3.50962584	3.10053359	4.06014913	3.54923157
1.91461705	2.94304339	3.13516009	1.62072256	2.38070432	2.33872085	1.71422261	1.57038184	2.93706423	1.74886599
2.79287163	3.17779611	3.14464902	2.64675965	3.46275616	2.8841196	2.67974253	3.1822145	3.40077844	2.4672032
3.45320972	4.05112611	3.03468435	3.6222345	3.58961899	4.15973044	3.61183072	3.11643717	3.44997713	3.47875424
2.16867768	2.65424635	2.73869671	2.46818107	1.86199591	2.4687935	3.45302073	2.61628673	3.29244014	2.47026994
2.43065926	0.8547269	0.91686665	1.07753614	1.32943494	1.00000887	0.97841343	0.95856736	1.06266844	0.34685182
0.51116035	0.13638721	0.00958156	0.06950183	0.11772882	0.05476851	0.10817829	0.08544763	0.04225409	0.04612804
0.16617509	0.21213237	0.12386051	0.09834924	0.09962134	0.30742542	0.10033166	0.51180382	0.57876252	0.07206844
3.00511686	3.69397492	3.3748576	3.2161558	3.33230566	3.12166005	2.56974652	3.055908	3.11136912	2.99895061
2.30891864	3.10117402	1.84131252	2.03081115	2.6422857	2.05097812	1.8462744	2.44592629	2.56623564	2.07047336
3.32291906	4.1919413	3.40988134	3.36270103	4.04443109	3.58440236	3.44056587	3.11524769	3.66195562	3.4685486
2.47031125	2.91210064	3.41931089	2.75462506	2.88808939	2.71059636	3.04557624	2.84136434	2.77104743	2.68378971
3.01716824	3.34957629	2.26399459	2.6379919	2.65572805	2.46240967	2.81815104	2.28458367	3.05316907	2.16073341
0.85475552	1.34003764	1.04098377	0.64799938	0.76358894	0.81036749	0.75651666	0.70704781	0.98460501	1.34085325
1.92294029	2.30293144	2.4809118	1.68159519	2.14639489	2.42279402	1.94663818	2.08076875	2.70827208	1.80655875
5.02564756	5.43753989	4.7903138	4.48253621	4.96964418	5.67441467	4.48894055	4.12977966	5.66791296	5.34050357
4.06790571	4.12876413	4.27511272	3.68596805	4.12696197	4.47079184	4.27773913	4.18199646	4.92259995	4.16894011
5.25851879	4.34649647	4.15188626	4.8170603	4.79261643	4.35633185	5.71106602	5.09668144	5.40652175	4.8106525
4.32396595	5.26292675	4.45791829	3.79360573	4.32501419	4.5746773	4.48052075	4.22523851	4.40453614	3.9882709
3.76254072	0.65797544	2.20500058	2.68976462	2.00423862	2.7030983	4.74731282	1.80024731	0.52311175	3.71630566
5.4890152	6.11039937	5.3512495	5.7025737	5.93873135	5.97600868	5.81372332	6.0076171	5.82318231	5.28591409
2.24813216	2.38063757	2.36630318	2.04376233	2.59603859	2.36047134	1.7146403	2.33747082	2.0556093	2.17540328
3.62219935	3.80644128	4.55538204	3.64035241	3.97997084	3.85069041	3.76339068	3.92325731	3.83878967	3.88386124
0	0	0	0	0	0.29376094	0	0.07508016	0.03094899	0.03379857
0.12717982	0.02292804	0.01544936	0.00574068	0.00760263	0.02237316	0.01803704	0.0070939	0.03423029	0.0094357
4.53928592	2.33570227	2.91797893	3.91196656	1.82763757	2.85310151	3.70635519	3.2862533	0.69712521	1.37168933
0.09318197	0.07310146	0.21359381	0.25585465	0.11345902	0.12966621	0.00800809	0.42715339	0.08192159	0.09717393
0.9027697	2.09007374	2.09394621	1.55854325	2.04370936	1.34633346	0.71740329	1.67278934	1.13677482	1.17657844
1.79100469	2.56327623	2.94714252	1.91847322	2.36054602	2.56173047	1.34061222	1.84189571	2.47500541	2.53736906
3.5489454	3.5562361	3.55191195	2.96739178	3.35498503	3.54995971	4.05699402	2.84963704	4.24076847	3.26553033
2.05561853	1.72431471	2.08234958	2.46304235	2.9753663	2.14560181	1.43288968	2.37867671	1.5923478	1.02172038
3.84223473	3.96036853	4.60320258	4.45932653	3.51764278	3.46064948	3.45214189	4.68132824	3.44961813	3.79823123
3.85294793	4.06400895	5.5712099	6.48058528	4.69750647	5.12103034	3.74596511	6.12135545	3.33993628	4.95453245
1.379191	0.28498146	1.3089708	1.13614061	0.73544833	0.46321008	0.60827994	1.39839143	0.31672642	0.27075545
2.26339423	0.37789449	0.93716569	1.85410182	1.92138253	3.18506746	2.56750078	0.26427458	1.61374991	2.21605916
2.71543523	2.88554343	2.81382103	2.52497103	2.8575607	2.93263234	2.64081814	2.4285921	2.72092729	2.27855885
2.258535	3.15451801	3.40942224	3.13531622	3.27361015	3.62423321	2.67172072	2.73971724	3.2847468	2.48131726
4.31231363	4.22792604	4.04061021	3.51868215	3.69199501	3.517985	3.60307752	3.14019227	4.02851487	3.56989095
5.40633573	5.22169186	5.1564981	5.22003252	5.04294203	5.48921958	5.96633598	4.88110352	5.55861915	5.359597
0.04614613	0.01116456	0	0.00556827	0.05796628	0.01739002	0.00877531	0.06737577	0.02498042	0.00915267
1.09324762	0.76161833	0.86345062	0.76663059	0.8656159	0.92501326	0.95323517	1.30290255	0.87378285	0.47344318
4.76957614	5.24693471	4.00055709	4.46398109	4.21255209	4.93228312	4.6394277	4.42787148	5.06429763	4.37019265
2.11604153	2.63348637	1.8646769	1.94350663	2.18342073	2.22493882	1.68987999	2.37210357	2.29184491	2.24086409
0.34851875	0.12724257	0.1007029	0.02190475	0.2663282	0.52985107	0.0173072	0.29510186	0.12714704	0.21867096
2.51556145	2.94266365	3.02932048	2.53174336	2.83856998	2.8608775	2.51042285	2.82838658	2.77475515	2.48197343
0.13037858	0.11732571	0.33662317	0.28854471	0.51808436	0.17350985	0.04632111	0.43823204	0.01576727	0.13501401
2.20426038	0.24413025	2.91878508	1.13299411	2.29545954	2.8840766	3.04487356	1.41585092	0.43197031	2.36452411
0.28352204	0.06681501	0.1956875	0.22985811	0.06354031	0.0472549	0.14693299	0.20229102	0.04241982	0.02994
2.17423057	3.14744727	2.68700776	1.55764428	2.1678675	3.54582168	1.41474083	2.97262042	3.20749422	1.99679043
1.19307389	1.15148669	3.14597936	1.53453869	2.79786752	2.00909167	1.06016095	2.11792912	0.84065563	0.90975661
0.46682548	0.17652167	0.59928731	0.72244569	0.62429559	0.39280966	0.37123812	1.17359954	0.11344444	0.37384678
4.92072079	5.17501069	5.34827205	4.03177171	5.39870351	6.15600225	5.92665886	3.94205017	3.6197745	5.95567335
0.55179366	0.02417771	0.01745054	0.02577525	0.00687644	0.52700004	0.14805456	0.05674365	0.21089738	3.87282999
2.87763448	3.92700483	3.85563796	2.2814605	3.69009668	3.8800373	2.46484966	2.247702	3.40479385	2.63793168
4.44030255	5.77393302	5.02354242	4.00259835	5.40515667	5.85231773	5.16579561	3.96195507	4.70357841	5.42208145
1.35893675	1.09463205	2.27426347	2.36937175	2.10909798	1.63574219	1.03339028	2.75765203	0.69759435	1.94806054
6.17352714	6.30162859	6.48083392	7.46988243	6.33122051	6.81678848	5.82176444	6.54868403	6.04355687	7.68968409
1.67737299	1.79483025	1.90851588	1.8788174	2.05464784	1.8641272	1.00989859	2.06369467	1.60533101	1.43414791
2.92258277	2.70342261	2.47519697	2.88215929	3.22369659	3.71282723	3.0971023	2.97949712	1.24444545	3.02842542

1.98495771	3.07330075	1.76311476	2.00132069	2.28242291	2.23116531	2.44712524	1.66902189	2.58411357	2.29619901
1.49238021	3.52126485	1.93569487	1.32147867	3.0780968	2.99161181	2.20636542	2.1097787	2.94678598	1.60117679
0.40923624	0.29015544	0.61056198	1.37692682	0.43738588	0.53597571	0.09881468	1.26392839	0.11829597	0.71449887
1.71173281	3.33518433	1.41024621	1.54487666	1.63485505	2.36332339	0.54988649	3.0948598	2.58350853	1.29528275
2.92794704	3.05290137	2.47284245	2.53207902	2.67838203	2.99161483	2.25516833	2.21885302	2.7204946	2.67387581
0.8881908	1.15413838	0.98742442	0.953738	0.99588087	1.11368967	0.60293678	1.02543696	1.12392794	0.87678537
1.13828509	2.17227627	1.53553051	1.30520918	1.70793946	1.53816931	1.43386344	1.3335092	2.08225021	1.33313002
3.74860286	1.97381135	1.72332349	3.4187391	1.29462668	0.71747346	0.75812451	2.70692748	1.5665189	1.27933985
3.19343543	3.20720595	4.76980435	2.86738281	3.34176808	4.08512255	4.19424175	3.49399388	4.71905855	4.19568707
2.10011404	2.89200693	2.74881398	3.36105489	2.70608925	2.06975148	1.43467741	3.18273378	2.05491211	2.42748985
3.41434102	4.31890307	4.9522345	3.30602885	4.72373298	4.41386791	4.03798847	3.9345042	4.22006918	4.31831564
0.46840982	0.18866151	0.59140403	0.51688352	1.24051539	0.32475152	1.54393086	0.91527083	0.32497055	0.57243981
0.68584379	0.15391099	3.63035962	1.09103223	0.10994351	0.3979096	3.25141986	0.09438975	0.18938512	0.05889823
0.03209677	0.05579579	0.42190869	0.10426322	0.45300946	0.25465745	0.74836473	0.29280493	0.01158061	0.1159607
0.01177353	0.11578409	1.03032602	0.22846083	0.09380541	0.22044104	1.64558973	0.02755691	0.03348838	0.00922947
4.19238223	4.89201432	3.83649197	3.89073829	4.13135484	4.41255915	4.47459047	3.9776173	4.76498765	4.31248498
2.54106009	2.44790516	1.47045082	1.56118404	1.9463497	2.42733237	1.78473862	1.70876218	2.39274584	1.39363578
3.15956595	3.69251682	3.45486855	2.7009211	3.15737267	3.03926116	3.01283215	3.2313363	3.61543235	2.43579281
0.89378886	1.39285248	1.31375171	0.63166188	1.33011699	1.67516991	0.90495548	1.02634269	1.14241106	1.34481685
7.35506549	5.89966577	7.83995763	7.98263593	7.29067979	5.84008719	6.92010059	7.79293183	5.56958192	5.18348297
2.57595736	2.24204526	2.5855039	2.23209041	2.06023862	2.73185156	2.78192752	2.47381985	1.83706761	1.78293512
2.78397193	2.73241779	2.91119466	2.54163114	2.94203319	3.25592554	3.05815894	2.69347068	2.96565806	2.35380244
2.77976921	3.1031639	2.85348627	2.25917305	3.5781981	2.81102808	2.45333606	3.29353714	3.21546902	2.05759054
0.58191854	0.12628916	1.48275893	0.6484414	0.47862522	2.16110382	0.22643715	0.89334269	0.36152134	0.41927084
2.96221054	3.30532696	3.48305197	2.94791935	3.13381948	3.644731	2.9445299	2.79970917	3.05642709	4.35460817
2.04030508	1.11704641	1.25885063	1.77469638	1.22139437	2.15368053	2.07616188	1.57320363	1.28815986	2.26010254
4.12550348	4.2042227	2.94665346	2.44459114	4.01367504	4.14628554	3.50817757	3.35272845	3.4155341	0.63363278
3.1337486	1.99421887	2.34065373	2.91466714	2.54992283	2.53761209	2.04071627	2.84319887	2.33493619	3.50816017
5.76884047	2.85295889	5.36037303	5.17321781	5.28820501	5.1299037	5.78776305	5.80694463	3.41045012	5.32197617
0.63295242	0.4201019	0.8049261	1.77278016	0.21512085	0.1657695	0.05420678	1.04830936	0.19678934	0.4848062
0.83991648	1.14767599	0.78971033	0.78661707	1.13231683	0.74663061	0.90514511	0.89259568	0.85482912	0.76780156
3.72485583	3.41712677	4.6042494	5.20742009	4.48739246	5.0784091	3.71582135	4.79052936	3.16893934	4.3672875
3.44513476	3.75339125	1.72873086	4.96621069	5.65723836	2.57168712	3.67620499	3.29766169	4.40903026	1.74666109
4.28917333	2.70243125	5.74499644	3.59402709	2.91093347	3.16067777	2.21082873	3.49858291	2.5027102	2.7477267
3.61631978	3.36478262	4.35140353	3.37202402	3.72633546	3.31631173	3.36673209	3.65969661	2.41097931	3.05685814
2.32990982	2.39000991	2.91003669	2.86138554	2.80419531	2.77389282	2.88350486	3.24032511	2.58664518	2.56113426
3.79026517	4.17363594	3.37779674	3.12433737	2.51041521	3.36600265	3.39150608	3.14270727	3.41773479	3.36307908
3.33257771	4.31107729	3.7090872	2.86253574	3.3077231	3.58038525	3.48217814	3.08299589	5.00958397	3.35985667
3.70253092	4.5604068	4.23931549	4.37290963	4.6771443	4.46505943	4.44463006	4.24953833	4.43892006	4.9964146
2.64195392	3.12004658	2.90733721	2.72485471	2.88934087	3.15358137	2.16930719	2.88449175	2.63529623	2.98570202
4.01142621	4.80643703	5.3460424	4.86017508	5.11761569	5.30085993	4.04455598	4.88166997	4.51437428	5.25120023
2.49889582	2.83054438	2.30842236	2.38397589	2.76312574	2.86328312	1.84838725	2.18921274	2.6913372	2.4806206
0.01219683	0.00778582	0.02341936	0.10128428	0.02299008	0.053822	0	0.08398614	0	0
2.74585737	3.24575396	2.93883338	2.8300282	4.36556096	3.73262607	2.48701874	2.93777779	2.75555222	3.11704249
4.07908663	5.03561124	5.0268928	4.93905885	4.81049268	4.84500964	4.10074628	5.32315756	4.28694722	4.34072616
0.42118322	0.5502021	1.01541083	1.6927153	0.82493635	0.73989115	0.10062482	1.13187061	0.18632938	0.52383375
3.85104271	4.53606526	4.07492229	3.54267534	3.7399369	3.73119816	4.43562731	3.86746866	4.04235613	4.04875095
2.42862326	3.31485985	3.3857824	3.25689336	3.64703959	3.31707669	2.47002437	3.50949742	2.9714658	2.97943943
0.35596362	0.39165799	0.62943185	0.609505	1.66218514	0.35106736	0.16621152	0.85485929	0.17653663	0.21778778
6.09424555	5.81528649	5.71538765	5.52795468	5.81272885	5.7550631	6.58117341	5.38484698	6.47358276	5.6807068
3.00656059	3.72225385	3.41238471	3.43922364	3.1280685	3.52628904	3.77659569	3.44210241	3.97726853	3.64737286
6.20980608	5.99685832	6.46668993	6.06768289	6.05817184	5.87320107	6.56440575	5.47497591	6.12976253	6.93241892
0.04066815	0.05164356	0.06478022	0.05771839	0.03850112	0.01530751	0.04572204	0.11650665	0.02925389	0.03194912
4.39900941	4.12447494	4.41250488	3.64655292	3.81488274	4.31273496	4.36740271	3.5746411	5.15938393	3.9245429
2.89096004	2.91289386	4.50317663	3.01724535	3.08082056	3.34496382	2.55647934	3.43413815	2.86918451	3.52298021
2.78133978	2.72170087	2.94222959	2.64080843	2.84448668	2.6861193	2.3618512	2.92913891	2.59355357	2.26125597
3.31461651	1.98652033	4.34180238	3.68865244	2.39590408	4.04215282	2.964984	3.04206832	2.22738664	3.32884702
2.55157981	2.84971952	3.28356391	2.77555867	3.18861891	3.11328631	2.81206151	2.671148	2.88003272	2.70401606
1.72215324	1.63706184	1.56078391	1.8437682	1.67232843	1.69911805	2.33366711	1.93520428	1.86228381	1.63687184

2.09565508	2.09648461	2.43553404	3.12232388	2.11447705	2.45966647	1.35978595	3.80890594	1.92058216	1.72271346
0.16775628	0.31246623	0.27168973	0.33550609	0.38854927	0.60540633	0.07018837	0.29944984	0.16066084	0.42676878
0.94602839	0.55203447	1.48314821	1.50900128	0.96962896	1.02454943	0.59648811	1.57108285	0.58080595	0.87657689
0.43573693	1.27961884	0.90631784	1.11946075	1.55254764	0.55326663	0.38774932	1.48353842	0.8259948	0.31461097
2.93175608	4.25895352	4.53696688	2.60721202	4.01483935	4.18098993	2.99882936	2.35959976	4.01080317	2.91496789
1.71956366	2.316685	2.20708437	0.88221768	1.54736325	1.37037545	2.20053406	1.29991941	2.43052629	0.76542126
1.72860377	2.59011076	2.45044477	1.54262869	3.02069183	2.99575843	1.7358884	1.90568446	2.48227262	2.22419054
4.7598342	4.92899507	4.31263745	4.32966057	5.22073656	5.31709682	4.68921185	4.75606538	4.79043628	4.76225325
1.71706531	2.11368955	1.78178722	1.35566023	1.57175399	1.66718835	1.18081042	0.81916357	2.26886509	1.02637396
1.30249513	2.09310787	1.7657154	1.4169221	1.95672937	1.84780084	1.39743068	1.59570904	1.62609451	1.84781768
0.53099172	1.54046303	1.24597987	0.25814348	1.64598303	2.07838693	0.61917508	0.84876168	1.35647321	0.9159329
1.82690405	3.69274839	2.1724608	2.19374144	3.53092554	2.66475923	2.15384495	1.94681952	2.85391217	1.68224096
2.07722063	2.53772939	2.70811172	2.07202838	2.6015894	2.66882162	1.99976386	2.32872817	2.29061812	2.11384728
2.33109752	2.72231544	1.14766438	1.22602312	1.8872781	1.46704443	1.42108835	0.76184823	2.34093073	1.0907146
2.28436202	3.27996626	2.32971596	2.2576894	3.21281167	3.24466914	2.29103712	2.5468637	2.99953354	2.16024853
3.36076877	3.64947273	2.37474397	2.94066939	2.55493333	3.38102325	2.715126	2.10823039	3.59258329	3.69754785
2.95946902	0.89979117	1.43524191	2.52338944	1.30441159	1.14290409	0.73672058	3.45959473	1.35237823	0.77488171
1.25686798	0.49540511	0.78598681	1.60926722	0.61180573	0.66392329	0.29199467	2.25604345	0.39107586	0.39567487
3.51286819	2.07739471	1.4968503	2.06894213	2.50514379	1.42136252	1.38026468	1.09192846	2.55041253	0.32805061
1.13526861	0.53096349	0.26330314	0.40089159	0.58037777	0.40657214	0.2588624	0.74389958	0.47813922	0.69156938
3.87131171	4.37724062	3.81410105	3.64071397	4.04376721	4.34122205	4.20363452	3.82519543	4.1742076	3.66737364
4.41940466	4.85168679	4.7094897	3.35677011	3.76567935	5.26147283	5.14811893	3.55636495	4.90894811	5.71557843
3.04213347	3.56635225	3.51761706	3.26803815	3.61947741	2.76429407	2.93998845	3.08078205	3.38336987	2.68341611
4.60092565	3.88373428	5.24504766	5.05573161	4.02668504	4.47379096	4.15233739	5.63360774	4.08672746	5.27497506
5.11701217	6.21584635	4.31255735	5.83889485	6.28288605	6.66860535	4.07498109	5.59533093	6.00857474	5.36495107
3.08192234	3.58654171	3.26362184	2.53933451	3.29662745	3.0264561	2.02764043	2.5903472	3.43683543	2.84932567
1.72580379	1.52608343	1.57479781	1.47143905	1.85320626	1.28202796	1.26138766	1.40444127	0.53439103	1.82709288
1.1668058	3.69794192	4.4777654	1.091149	0.83125171	1.97200728	1.89746448	3.21298533	0.74202374	0.31910599
6.40051739	6.45081215	5.43835388	6.54900431	5.89738186	6.7693398	7.10189876	5.71443325	7.39524064	6.17840103
4.31628049	4.29625155	3.81781747	4.08082448	4.11713944	4.32340977	4.72628306	3.46319801	4.40513085	3.47661278
2.24446127	4.38113743	2.18320839	2.09761782	2.05527884	1.48050545	1.14493713	2.82975745	2.60459353	0.98349435
3.33181614	2.94061312	4.54767663	4.06521644	3.68162521	4.21006746	4.8304446	4.60461306	2.53871744	5.00689065
1.79810716	1.68684886	2.03147201	2.35751952	2.60463068	1.65997091	1.37217387	3.35224502	1.26120663	0.93628247
4.51444596	4.95333524	4.44214097	4.21850746	3.70304495	3.45193815	4.4448561	4.83470458	4.74214801	4.06771053
0.14893855	0.09261686	2.6155243	0.61028715	1.62501501	1.33584128	0.70972789	0.95215302	0.82019582	5.05653643
2.6755568	3.37919258	2.88303198	2.59812402	3.09763411	2.69140435	2.27403773	2.81894621	2.92144639	2.10931895
2.87573002	3.84620116	3.08747843	2.88541338	3.73178984	3.58459041	2.55200022	3.34307224	3.07685281	2.85598997
2.88288557	3.01082379	3.27966573	2.79294346	2.69386145	2.84053475	2.57858038	2.63482008	2.69310846	2.61127947
4.39313237	4.87918524	4.84789514	4.53946302	5.09995081	4.79645955	3.92974615	4.19367775	4.333363	4.93368604
2.52607054	3.88789392	3.60310456	3.03795267	5.35790498	3.56629631	2.50727112	3.11939493	3.22620144	2.49884311
7.4559346	7.01008087	6.56670737	6.65719565	6.48340202	6.63359635	8.04398383	6.59780428	8.38328076	6.92046584
1.58433474	1.69331676	3.72658781	3.81064429	2.87926622	2.40896664	2.33947193	3.09145389	2.0930469	2.15960895
3.20876506	1.94394019	6.61869264	3.17229293	3.86992554	4.75085768	3.03489689	3.77172075	1.05438159	2.87998022
2.27851061	1.60236028	3.3484768	3.98536234	3.14079486	2.77486571	2.05108272	4.02422536	2.24804402	2.83004375
2.02868903	1.004036	2.46790541	2.82259544	1.92029974	1.2714242	0.56815105	3.57748689	1.99007449	1.21050102
1.00216641	1.68834057	1.85512601	1.0409612	1.4909454	1.62003743	0.5686308	1.12870497	1.32596211	0.86518721
1.97903128	2.40491298	2.98638921	2.0037736	2.27382444	2.49142654	1.94605218	2.14990759	2.33986081	1.74957993
1.80967196	2.35933097	1.47038477	1.57201655	1.82052886	1.97176788	1.54455744	1.27175126	2.09564108	1.27109391
9.91300278	10.0488721	8.39743257	9.22981231	9.04016598	8.85350272	9.36954604	9.2962954	9.26104739	8.85329988
0.53466416	0.26241464	4.82014355	1.70664888	3.76691093	0.10815344	0.1501242	1.77334101	0.12392791	0.37201679
8.10201311	7.58862222	6.15163552	6.74782357	6.24348932	6.38780461	8.17071509	7.15945526	7.8029007	6.62980879
4.3911457	4.26260424	3.41693483	3.90329484	3.7616287	4.28847745	3.72713102	3.38750619	4.37058384	3.84073927
2.29381272	2.8500906	1.57764517	1.99203638	2.69205586	3.00205142	2.66102353	1.99714931	3.52762274	2.27359524
0.76654757	0.87956339	0.33050547	0.78135187	0.49385515	0.67104156	1.05387849	0.68481875	1.34813066	0.97054328
9.7825171	10.0975433	9.58705735	9.97803695	9.40319543	8.63218628	9.88904064	10.1322664	9.58819013	9.34405329
2.07369916	3.00755293	2.66377456	2.81361844	2.93861571	2.8970488	2.07156473	3.07200629	3.0723161	2.36484162
1.89907171	1.79985349	1.97836385	2.09531616	2.13503188	2.84410551	2.18796401	2.15836101	1.52080624	1.82648884
3.89724954	3.98175481	3.79267372	3.6583042	4.21199786	3.81041006	3.59135274	3.82193746	3.42607223	4.26397658
2.25379593	3.41406336	3.84770393	2.50819779	2.39623493	2.57258602	2.31644631	3.13316151	1.40705796	4.55097573

0.86335977	1.1002877	0.81263355	0.64066534	0.67318276	1.13633083	0.71180265	0.83439524	1.23547863	0.75284215
0.41412282	0.31023278	0.27213105	0.35349638	0.43311073	0.38841534	0.11427313	0.39367522	0.64324917	0.38092615
4.60202063	4.74582513	5.2843079	4.97867055	5.08146086	4.76136634	4.34010908	5.02926827	4.68220575	5.07742765
0.27973539	0.88332728	0.37152417	0.58486046	0.60246843	0.51967666	0.51134459	1.01946973	0.5004201	0.37103433
0	0	0	0	0.00983248	0	0	0.01829205	0	0.01220092
0.22919938	0.0464646	0.05763782	0.08066311	0.03529501	0.12752402	0.04509828	0.10386291	0.16504663	0.06343807
0.0822898	0.05081927	0.0879496	0.17380744	0.09473176	0.05171927	0.01318584	0.16091373	0.06427569	0.11419147
0.50039039	0.87566107	0.9652621	0.56065257	0.95736674	0.60683528	0.21384979	0.69751124	0.89295548	0.5199654
1.60720167	0.72835812	1.65353833	1.6557583	1.33558183	0.57322184	0.93537872	1.53515682	1.01314018	1.39243523
4.96753467	5.15474342	5.23698458	6.1693181	5.27836044	4.04387611	3.18376342	5.82650259	3.37126372	3.52408374
3.84346078	2.73333857	3.96790185	4.62377903	3.70317834	3.09773497	3.89804295	4.65964311	2.74558542	3.12739541
0.66660952	0.93413019	0.6804986	0.98071338	1.67324743	0.87757653	0.25202562	1.28323883	0.99751637	0.95482553
0.13038061	0.20504189	0.46977652	0.80256642	0.33623772	1.11561096	0.06019888	0.45521967	0.05122189	0.23002072
2.52406027	2.3392019	2.14660402	3.43162278	2.07785738	0.99838513	0.57445411	3.57302835	1.8416652	1.61716137
6.88417486	6.46185576	6.36493265	6.28703361	6.63604292	6.38485981	7.29106222	5.70055685	6.82024568	7.06993976
3.90491135	3.89733771	4.85380559	4.14909761	5.54851308	4.12639806	4.18331197	4.13792408	4.14673698	4.93606202
4.21878478	3.47327476	3.31955557	1.20249405	0.88626092	3.84582284	4.96889612	1.38343689	1.27621345	3.12157703
1.5012015	1.87545436	1.56473643	1.2366333	1.41106083	3.00631416	3.04325471	1.24331426	2.53389086	4.33252879
3.88132689	3.40601749	3.31528054	3.25997854	3.73447056	4.25561494	4.00932939	2.86363508	4.05722701	4.62783397
1.11076767	1.40031063	0.60062813	1.6624471	1.56570818	0.25299421	0.1827041	1.78230608	2.13822942	1.08468971
4.44217983	5.90985266	5.94924261	4.74274911	3.80729566	5.36258616	5.32957021	4.70115156	4.1788709	6.69875099
4.11523763	3.44311935	4.79108663	3.79098018	3.6978443	4.85561335	4.05072212	4.5043087	3.99859039	4.84117829
0.6640999	0.52074223	1.14425177	1.29445399	1.25621065	0.57014821	0.93138551	1.83206969	0.29087393	0.30929637
3.73161121	4.81683297	4.28972117	3.6052563	4.04652683	4.61552863	3.35195951	3.27069463	4.90697369	4.41458892
2.1185333	0.89306406	2.58034737	2.76745376	1.93108131	1.63832596	1.69286741	3.11200441	1.03342587	1.37013469
3.35792791	3.90252787	3.29628603	3.10515587	3.51190579	3.71977676	3.46010024	3.59711049	3.44968946	3.32269749
0.45340629	0.62034124	0.77986292	0.62526287	1.40899188	1.38479274	2.14520865	1.20388075	0.93263197	0.27200487
4.24242683	2.60495224	4.45909239	3.83745829	3.53646415	3.59717882	3.51040562	5.03448676	3.49846987	2.93574966
0.95850337	0.68546382	1.03238933	1.33981554	1.12471445	0.66838509	0.5713345	2.26400833	1.05970472	0.76687821
2.11042303	2.12098145	3.81968876	3.16538933	8.48110712	2.83006838	2.77142621	2.99101771	1.07449677	2.89572722
2.71615288	2.71480684	2.7848208	3.24995761	3.93146683	3.43921773	2.88330323	3.24660327	2.48608773	2.82384256
1.30798891	4.38958553	0.97840799	2.20673465	2.01564519	1.82292901	2.79411253	2.71482185	3.13479713	3.03229004
6.76049717	5.91434422	7.44548721	7.0604636	6.825787	5.7134748	6.85843503	7.85988425	4.41526191	3.8660232
3.45441094	2.24633953	3.04410896	3.03859952	2.43983553	1.75986607	1.495152	4.02530254	0.75329426	0.42330152
1.92441315	2.74550102	2.86315276	2.69322844	2.68360506	2.72564492	1.81000071	2.89336514	1.97316376	2.562186
4.54270512	4.04036685	4.26588847	4.18262194	4.36091509	4.29201111	3.8630936	4.15878287	3.89873382	3.69874416
5.17784617	4.36804106	6.20155639	6.70119912	5.34588215	4.98165248	5.10490544	7.31143126	4.18282265	5.29852517
3.27387174	3.24205955	4.06559114	3.52031824	3.52983791	3.45725132	3.19328143	3.98438297	3.08132684	3.40866598
1.91100391	2.70299163	2.03812654	1.54171962	2.31754758	1.65978576	1.295328	1.7720904	1.93124948	1.18898037
2.26841235	2.62905378	2.53929741	2.12918939	2.65501824	2.63612682	1.96076194	2.56312512	2.73444327	2.07714024
3.72618218	4.50101937	3.66192308	3.9893963	3.83446408	4.48713692	3.40418399	3.81368508	4.34457992	3.59933808
5.59068583	6.57760681	5.85996677	4.85899456	5.28843907	5.6180283	5.2771512	5.1030013	5.89058846	4.95569725
5.15149114	5.81342612	5.27084045	6.03703773	5.45948307	4.98063565	4.12474831	5.80284964	5.11448857	4.96382636
2.45676431	2.56775245	2.91525793	2.56241234	2.91507096	2.8535139	2.2242094	2.2642301	2.99616153	2.23982949
0.5434378	0.39932836	0.64181625	1.00493574	0.58096308	0.72305841	0.43743669	1.13376999	0.55693195	0.39341026
6.66169049	6.56741876	6.79339936	6.71818	6.19786735	7.86665542	6.88294238	6.494407	6.86226751	6.53410039
4.09415585	4.22704974	4.24932664	3.85505159	3.64708761	3.69750225	3.88898632	3.70517406	4.24956534	4.13162687
4.01279095	5.23267465	4.4025422	3.79236184	4.59515532	4.83964307	4.27194995	4.04903943	4.50913739	5.13616535
4.89053673	5.40888467	5.83840239	4.57992015	6.28634601	5.80023861	5.26210643	4.65968943	4.8604212	5.78514283
2.86172624	3.34284648	3.85369806	2.75171694	3.49769993	4.16563407	3.30135929	3.28653943	3.28810233	2.65538613
1.78821548	2.50980581	2.39599856	2.09767583	2.92596735	2.19200295	2.04579881	2.17758117	1.54778777	1.72079445
3.45253632	3.94398516	3.3788109	3.35191629	3.7672652	3.60662432	3.31980443	3.4005333	3.797711	3.67600358
2.56249098	3.06563838	2.75397278	2.73516124	2.36023687	2.53822871	2.9496601	3.06223476	2.98180947	2.64016185
2.80067349	2.91901463	2.27860173	2.78739806	2.66228061	2.98776466	3.08433712	2.66328199	2.65673288	2.31716037
3.14041349	3.52737718	3.17866759	2.76782271	3.18660957	3.38986178	2.87123835	2.87962584	3.30871636	3.72181245
2.38328953	2.89713129	1.77944278	2.55303818	2.29480155	2.40311429	2.30509416	2.99438682	2.48740198	2.64299478
3.32289155	3.95684976	3.60917203	3.20368491	3.59930128	3.24420287	3.62455845	3.7179236	3.76986455	3.14030685
5.19902495	5.96454163	5.44689592	4.91523872	6.26270346	5.80244637	5.48038822	4.78812029	6.22274885	5.6234433
5.01729316	5.73343378	5.96361793	5.15313808	4.93679482	5.78819285	5.56592932	5.45840041	5.61439112	5.85613259

4.57124456	5.40775201	5.25776589	5.01207824	5.43803038	5.1666482	4.88476962	5.03111786	4.91158719	4.55623129
4.3019802	4.89144342	4.86435643	4.19154139	5.36256819	5.56388525	4.77881713	4.63257972	4.84025818	5.02842566
6.07970192	6.30296555	4.97251792	5.82679232	5.55250864	5.55779078	5.79709592	5.81158374	5.5859167	5.73287404
5.83531629	6.51566418	5.12620628	5.25318079	4.76409812	5.99219952	5.43688344	4.8445295	6.5454096	5.77122365
4.78055883	5.04291973	3.8881712	4.25464974	4.12268185	3.83888845	4.31820809	4.33130107	4.42809379	3.88118253
5.62302304	5.80002137	5.02495121	5.50516802	4.70832833	5.47693799	5.70119281	5.53433065	5.67504412	5.2443336
5.77275606	6.16026228	5.12384546	5.33879338	4.90601756	5.83139295	5.40477935	5.04966343	6.10040082	5.65857418
6.68727847	7.16605047	5.76217761	6.60119863	6.36314342	6.13452353	7.17362943	6.48631385	6.8706937	6.55630712
4.32727268	4.85075284	4.04447211	3.80661277	4.64392164	4.01057542	4.63648581	4.17513074	4.98266472	4.36907669
6.23405093	5.68168128	5.56422024	5.47289652	5.3666996	5.65996065	6.40333518	5.51392314	6.48136892	5.25393878
5.78952822	5.49295611	4.83105177	5.7782984	5.22083152	5.29054093	5.10840348	5.73411226	4.06052678	5.11869517
3.75529314	5.07754839	3.97765178	3.49862677	4.73273823	4.11301134	4.26978302	3.88414194	4.51614491	3.67299487
1.3753994	2.34552614	0.81256498	0.87329969	1.65198406	1.57670482	1.73774763	2.06277839	1.66277165	1.30856048
5.20079018	5.62742024	5.25770052	4.81235691	4.88662102	5.04946009	5.08234377	4.83194636	5.16190554	5.0896894
3.95560582	4.27265974	4.10140745	3.71140801	3.80590927	4.29855	4.44733178	3.93909066	4.12086678	3.41715195
5.77281187	6.18309096	4.96892644	5.51423455	5.69180435	5.58002744	5.54569329	5.61620377	5.65798174	5.1434518
3.06248176	3.39117043	3.0327148	2.66699786	3.10721413	2.72736811	3.65730082	2.80338218	3.67430088	2.81714651
1.39342542	1.01942727	2.34607158	2.01564892	1.61966049	1.73535664	1.72199448	1.60308501	1.12776579	1.19887743
7.09002447	7.1635406	5.22471205	5.74095416	5.96954137	7.11727279	6.23215847	5.26685875	7.38528224	6.3582534
4.77815262	4.85088848	5.14471228	4.84399643	5.57315382	4.91138233	4.17992418	5.16120554	4.46867595	5.26876836
2.32108683	2.63696682	2.22105546	2.48796917	2.56458153	2.65367722	2.03264649	2.50053254	2.40012736	2.3733256
4.92893098	5.9650158	5.8279673	5.51137172	6.14493513	6.08835239	5.66591257	5.97878168	5.85344796	6.18248876
6.01925353	6.9033508	6.70668405	6.34712364	6.84614242	6.59727867	6.09170183	6.26564845	5.94837917	6.16377456
2.87209833	3.0011227	3.47563668	3.17857218	4.04314235	3.13628647	2.32263759	3.62385094	3.36760683	3.46219104
5.69382771	6.4189406	6.35737087	5.75215431	6.67449181	6.3595827	5.88188017	6.09324554	5.74442554	6.07472893
4.37960159	5.15219557	4.27130535	3.94565184	4.28756042	4.54723738	4.34717698	4.15379085	4.34876745	3.71650258
7.40559724	8.1004203	6.93151893	6.48155697	6.99419267	7.46190153	8.67075689	7.20224682	8.48998977	7.35851349
4.35883446	4.42022507	3.71518267	3.76516233	4.77533486	5.00074808	5.03332529	4.14115664	5.39969281	3.89141545
6.8861038	6.89793248	6.69428256	6.34471806	6.60914622	7.31109835	6.6246651	5.75042483	7.62097553	7.50481574
1.34142378	1.49967745	0.977263	1.04172496	1.22022441	1.38557611	1.07875319	1.09853785	1.19166381	1.1652786
3.55125631	4.22001257	3.35497515	3.17079455	2.83394357	3.9117822	3.79805295	3.93604632	4.13665186	3.52210072
1.30203832	0.0730006	0.19394615	0.9486666	0.08976297	0	0.08561738	0.56473247	0.0818089	0.21343853
3.4699194	4.17286053	3.77236329	3.29721845	3.32214164	3.7103629	3.57007617	3.49998597	4.01795621	3.37578666
4.43516942	4.988052	4.36033411	3.89061926	4.78083948	4.74110226	3.70836353	4.52193546	3.87202659	5.26642217
1.93223903	2.60923638	2.27854331	1.88818127	2.3182652	2.11841696	1.36560846	2.11964027	2.40943396	1.68504524
5.55155269	6.68016492	5.45906857	4.97071142	5.47780258	5.55584499	5.30769318	4.43014969	6.61320534	5.46670241
3.87899471	3.83406458	5.21385833	3.69475332	4.5510886	4.81993595	3.63174594	3.94232857	3.76892872	4.6402455
3.61643146	4.96795512	4.48295647	4.33742088	4.29574813	4.46590656	3.66932645	4.32940744	3.78627417	4.61167494
3.82385215	3.01585921	4.74962554	4.37906354	4.35692964	3.72935718	2.77722988	4.82175399	2.58348332	3.53964852
2.09703276	2.57585763	2.5665358	2.14421505	2.87289787	2.72495829	1.47546594	2.71577048	2.58352783	1.63377863
2.71228992	3.00698586	3.32627141	2.94871668	2.70283032	3.3311806	2.50505994	3.11173782	3.0125357	2.50139387
1.31595013	0.85271009	0.55278054	0.3601671	0.19742591	0.56282962	0.65840934	0.55610681	1.20915082	0.62889142
2.77467183	1.58904272	1.78564335	2.15885343	1.85009237	1.55029976	1.08950983	2.68416251	1.47503632	1.04039046
2.67558838	2.96885492	3.58684003	3.18107008	3.10693053	3.32139048	1.94601581	2.89281922	2.77697913	2.92330704
4.44288635	4.41109285	4.04843796	3.65664055	4.30824066	4.38904565	4.96743264	3.26125467	3.0082609	3.53588363
1.49841072	1.61405683	2.0787055	1.53139054	1.99834626	1.89870415	1.38666436	1.50750159	1.63844354	1.84484108
1.35273314	1.62470758	2.17976275	1.27095275	1.50314404	1.66380328	0.95727331	1.90218031	1.43847775	1.29790131
2.35196508	2.46488064	2.98404028	2.96098163	2.53366284	2.55192397	2.14489474	3.26362116	2.56006186	2.47028173
4.28302533	4.81332546	5.41267803	6.40288529	3.99144228	3.10441314	2.88587428	5.59088636	3.22671263	4.49458799
3.70389621	3.46677007	3.69651871	3.64595591	3.14725353	2.92695006	4.03039288	3.69359537	3.97893565	3.36805964
5.15285661	5.45843359	5.68980388	5.12266902	5.05260139	4.97316538	5.77881998	4.9755072	5.62849129	5.73018802
0.52636789	0.13862149	0.65503915	0.8098539	0.2201733	0.44291548	0.38378894	0.74150925	0.21130211	0.38044828
3.67626432	2.92490228	4.46111767	3.9846931	3.93506763	3.34739455	2.25296272	4.35105703	2.4508209	4.18888752
2.06273166	2.18237319	2.36406086	1.46076066	2.18491126	2.27161319	2.06221801	1.63030929	2.33221641	2.21451672
2.22135255	3.52132803	3.52092906	2.01533897	1.6757945	3.32622937	2.69456873	1.16898996	2.37873566	0.76425478
2.16991392	2.73489139	2.00164354	1.9585691	2.67718111	2.5286668	1.6208802	2.24699114	1.92043883	2.15305293
2.82698437	3.79375743	1.93324503	2.37596563	2.70473855	2.21560423	2.48400954	2.30137154	3.21741672	2.15727693
2.98939316	3.76037023	3.03655118	2.79174068	4.14583362	3.35816311	2.51146554	2.92451806	2.81301471	2.4897341
2.5734864	2.87919663	1.90136845	2.24664587	2.11586506	2.23127499	2.52773977	2.18449266	2.94625392	2.29675848

2.25368506	3.18602751	2.77179356	2.57264353	2.99746244	2.38122597	2.46409184	3.00073507	2.77639008	2.0586297
4.12446981	4.78521009	3.74831228	4.27354298	4.76973093	4.55817206	4.43711127	4.06984194	2.29544393	4.16978557
3.69010365	3.87135319	3.46614854	3.47717833	3.24898824	3.11249699	3.71358233	3.4712212	3.92576105	3.62618389
2.46404213	2.76414985	2.38727979	2.20179469	2.66006498	2.59848465	2.99788607	2.59926644	3.09853315	2.69078758
4.68353627	4.79381531	4.65190841	4.7455203	4.32561919	4.08936324	4.83720362	4.7661501	5.10356816	4.71996513
2.96489689	3.23815988	2.61745965	2.80000349	3.20617343	3.43379851	3.38099208	3.01104693	3.59665068	2.87795305
3.11823388	3.60844537	2.39488449	2.57100078	2.48873254	2.98247546	3.23583961	2.72209942	3.52827964	2.50221106
2.61005082	1.3122164	1.69098582	2.54602373	1.53385126	1.29484532	0.45904203	3.51264017	1.37936826	1.26901501
4.94614576	5.30007897	4.97065279	4.881606	5.07477807	5.2944143	5.43842672	4.64412522	5.44848469	5.60792377
0.47254439	1.88790715	0.67447161	0.39446248	1.11235718	1.42638938	0.89426076	0.79299034	1.57758329	0.52396054
0.76272239	1.32533349	0.80358612	0.68038674	1.52595725	1.935591	0.75238628	1.18879241	1.57497075	0.56716407
1.79814122	1.84077508	1.26443518	1.44445711	1.42374821	1.70309731	1.91114459	1.45724607	2.13416132	2.24421706
1.82620901	0.41378734	1.37101374	1.45644786	1.19225826	0.59761273	0.55728662	2.03440046	1.01598266	0.86107073
5.79162011	4.94888485	6.33892773	7.20241914	5.93098383	5.71415158	5.54004111	7.18448745	5.01514	5.46955316
2.26553659	1.92380284	2.47282483	2.02198605	3.21350579	3.00938174	1.9933201	2.24339565	1.78519405	1.70797345
1.74490086	1.09604502	2.85160987	2.86721956	1.08290396	2.08386781	1.3265099	2.54803017	0.53761341	1.59571056
3.55316816	4.12002857	3.18042775	3.71353441	4.01008419	3.53339731	2.77254973	4.03240734	3.28391277	3.52475739
3.38349408	3.81380578	3.87628668	3.03910214	4.28203996	4.57364198	4.19646712	3.33498038	3.48384041	3.02214545
4.44730132	3.48727122	5.34717882	4.42601501	3.56839242	4.15878134	3.94263111	5.1560506	2.21369392	4.82407955
5.84282063	4.06033926	4.5489914	5.18939936	4.51577518	4.94042809	5.86009112	5.31660621	4.64168183	4.05863349
2.32266362	3.90086586	2.87891715	3.63906453	4.0915258	3.37595154	2.37772779	3.68885911	3.14309861	2.49664619
3.91199328	3.5312986	3.21116235	2.64034025	2.8445064	3.9008554	3.80788458	2.96106275	4.03821237	3.20314321
1.06795463	1.2086835	1.21530411	0.93906544	0.59930427	0.92435295	0.72619892	1.67968377	1.36011029	1.07853242
6.16641932	5.22965427	5.84329366	6.61500877	5.62154193	4.79925857	4.57273499	7.01235842	4.59639377	4.56313105
2.21341745	2.05628847	1.98087364	1.41066898	1.98284981	2.64660492	1.73404638	1.70221313	2.2242586	3.01086846
0.67771031	0.30361308	0.23142126	0.56428482	0.27186913	0.21258245	0.12053171	0.12570448	0.25581893	0.33059753
1.76146631	2.46362162	1.91675813	2.46485414	2.64086473	2.10214194	1.69625464	2.44602268	2.77233142	1.98598226
7.91687086	7.89039336	7.66178775	7.47854261	7.75075076	7.72833028	8.57752256	7.4032015	8.02985866	7.40807648
1.89833159	1.37243595	2.90244621	3.16669155	2.91145082	3.0019978	2.73072564	2.5539406	1.61329831	1.8168902
0.72967428	1.47226911	0.48648712	0.41592689	1.2345427	1.19638844	0.48445738	0.83678992	1.20576786	0.37616206
4.26997344	4.97448218	4.2195967	3.90002761	4.37541981	4.39207894	4.32106428	3.66803175	4.61398039	4.61936443
2.21139917	2.14941972	1.22815661	1.78445811	1.92782743	2.37189422	1.17608024	1.7371087	2.41275169	1.69821905
3.70891149	2.9963281	2.13528125	2.5059221	2.29316816	1.93870894	1.11142171	3.15699298	2.74513718	2.05383327
1.19623169	0.94801635	2.66646956	2.06571574	2.21616283	2.46010312	0.57192408	2.18844824	1.67166416	2.46876844
0.41548572	0.22518318	1.45459461	0.97600117	1.0373245	0.22913318	0.09763455	0.83161994	0.14469344	0.9687791
4.77665296	4.80601456	4.95091535	4.41401909	4.88344402	4.67228981	4.8603895	4.35580421	5.08013938	4.12128275
2.51894652	1.99513942	3.0807472	2.78128152	2.62551044	2.60071968	1.73413432	3.0300332	2.09661617	2.03056809
0.45094847	0.19245188	0.34680099	1.4323818	1.15300067	0.146314	0.28072435	1.41211866	0.02548815	0.33690033
2.45091545	3.35286252	2.59744726	2.58960143	3.52245877	2.36813017	2.23242741	2.21399367	3.33109622	2.81937351
1.58507911	1.86791173	3.3372486	1.59498718	1.58695014	1.85579518	2.07108747	1.7939477	1.68014428	1.37664956
4.26107595	5.00764124	4.24799382	4.32135316	4.51290744	5.21193459	3.95319753	3.0864086	4.53028719	5.81562034
1.83108683	3.90188068	2.71039507	2.06384834	3.06706283	2.9990351	2.18560133	2.46252696	2.97972326	2.30707585
3.00567241	2.86531471	2.51707349	2.46377608	2.26251081	3.30952111	2.97442323	2.0268445	3.42490551	2.88165674
1.5461673	1.84105037	2.98854028	3.07516736	2.41860085	2.29458859	1.52274051	2.97574106	1.74105751	2.5968169
1.90157256	1.32586149	0.4479797	0.96201085	1.72230837	1.20069749	0.41710201	1.06898049	1.89974989	0.10596994
3.69217721	4.24883824	4.03203002	4.24770461	4.4254864	4.20369833	3.74206816	4.5300332	4.37014597	3.64981219
2.05334047	2.87107189	2.40043085	2.64411818	2.77184423	2.63294671	2.10510747	2.72232763	2.63733365	2.20515904
5.42354233	4.16123338	5.17517682	5.94494771	4.83195993	4.7293715	4.14384191	6.65603798	4.10931019	4.89214116
3.26059995	3.48751457	3.31266641	2.57386075	3.17553773	3.39688922	2.11235247	2.58314451	2.90048594	3.34143937
3.02867694	3.80080846	5.05087651	4.2172485	5.32825556	5.34735702	2.81808136	4.45145614	4.70496648	4.69294993
3.3809049	2.66263922	4.57064171	3.06109993	3.91993614	3.70515086	3.74708764	3.6894162	2.2306257	2.88785129
1.38953005	1.30095478	1.32276038	1.57346856	1.48967222	1.39980813	0.9047765	1.90246187	0.93562419	0.51788959
2.38789471	1.85289568	3.05362019	1.43103256	2.18735038	3.23657345	2.39831822	1.43479595	1.15665363	3.38559003
1.7856394	1.72606457	2.01798674	1.67609371	2.03158982	2.15275393	1.39984363	1.63287182	1.75169767	1.86581594
7.57664878	7.28822904	8.53719838	6.30783881	6.52793222	7.8474673	8.75090156	5.67893005	7.35842883	8.31558739
1.81298739	1.79622131	4.42872828	2.95999541	2.51809558	2.25660489	2.09422698	2.99986806	1.65377226	4.59875411
1.4907955	1.8241444	1.76566381	1.44220787	2.21730326	1.99584691	1.03660325	1.83802154	1.68477544	1.44933644
3.2841758	2.59970159	3.5129272	2.71545417	4.39557144	5.17007688	3.67910179	4.91548065	2.31771722	2.75908726
4.10542578	3.51271695	4.7258341	4.17969706	5.59056222	4.67476862	5.01945338	4.0868905	3.46429782	4.91138722

1.11327388	1.2768099	1.94673915	2.42398299	1.14036214	0.86310825	0.20838242	2.60080211	0.81507016	1.41555229
0.72338777	1.67496968	0.65533186	1.14255383	1.65413436	0.61669577	0.14546549	2.13007815	0.64865063	0.38510338
0.30213687	0.26347832	1.10476809	1.80870669	0.07949236	0.95636205	0.08844643	0.633742	0.10084062	1.1817248
1.65450547	0.27383704	1.70309615	1.58576764	0.30040149	2.55156118	0.08541421	0.79608698	0.15885818	1.93648129
4.03253115	1.74552391	2.92374516	3.42386124	2.39254549	3.92967723	4.50865204	2.8019074	2.28451737	4.23048243
2.70653614	4.42901575	2.67207781	4.33177865	4.74823656	1.70048086	4.38770932	2.38913702	2.02280799	5.39467332
4.10399958	5.05355498	4.7546454	4.59660107	4.43862022	5.27208389	3.97561152	4.72932172	4.05617138	5.04908909
2.05788496	0.76458536	0.82185652	1.40580815	1.30005077	0.86716069	0.48982071	2.47626728	0.56980997	1.66453485
5.51205587	4.90698379	4.26364718	6.17351192	5.01072135	4.54633125	4.2545929	5.93409821	4.23391733	6.37575897
4.55430111	4.46955506	1.62026795	2.28256445	1.82157395	2.21779671	2.91343289	2.19317735	3.8615827	3.48897858
0.68441869	0.29038937	4.84001414	0.41464329	1.00883021	0.5840435	0.66641945	2.27080796	0.43706953	0.52929684
0.89337828	0.94373121	1.14794395	1.40789592	1.09124106	1.04394763	0.38741991	1.02514368	0.78268299	0.83320618
1.86896027	2.15819991	1.7952002	1.95342949	2.06134803	2.05570242	0.75566114	1.76047212	2.00677206	1.82440038
4.41780721	4.53772517	4.25921171	4.14218101	4.5641907	4.01068561	4.43574286	4.05260851	4.66965684	4.16108346
1.59563374	2.10586889	2.5978599	2.31970359	2.37150783	2.04992857	1.65336976	2.55387191	1.73440589	2.05994836
3.31261092	3.85509792	3.66249897	4.07589835	3.03648578	3.36057858	2.1056158	2.52972551	4.17641448	3.03249293
1.20902881	1.15726835	1.34577558	1.36293114	1.40385067	0.84501414	1.07199502	1.55756179	0.8701753	0.91357957
3.84121253	4.88425333	4.35698896	3.93941056	5.36291442	4.99898565	4.600948	4.12571504	4.36611975	4.31575785
3.100158	3.30137049	3.34686235	3.10269997	3.30434586	3.02030233	2.67148961	3.21077165	2.92951107	2.57352884
2.44380937	2.57568373	2.77246164	2.46706375	2.38722226	3.11389614	1.94753761	2.58652115	2.4961713	2.24328783
1.46637146	1.7855502	1.49326749	1.58090062	3.13936989	2.11542778	4.22559345	1.92026988	1.06300654	3.51027212
3.21846307	3.71168767	5.2230973	3.1708131	4.67861522	4.44084343	4.32456768	3.23871229	3.4192427	4.42045086
4.55007953	3.79916109	5.6991417	4.10540871	4.33465392	5.39127534	5.56251811	2.53832163	3.82742865	5.14277433
3.19741207	3.50266726	3.80782944	2.28314251	3.26889921	4.59518606	4.2015066	4.35544445	4.1814488	2.96854872
4.88630797	5.32123565	4.43565601	4.62696769	4.53325992	5.32436442	4.94770991	4.85980662	5.55872083	4.37012565
3.44998704	1.17162528	4.01152631	2.7936769	4.31313374	4.56813199	0.63859135	2.77813306	1.34221716	4.08336678
4.69295861	4.78647234	4.84438858	4.7072789	5.40927705	5.38845226	4.99944306	3.51154996	4.54308167	4.57757247
2.06623583	2.13588321	2.58574095	2.96426239	3.04579809	2.79567092	1.90651896	3.97673688	1.92939671	2.43698703
3.3295198	3.38465488	2.71870699	3.34374073	3.45454817	3.5783141	3.4069068	3.31502284	3.28147003	3.09524756
1.94683747	3.0777357	1.94248766	2.56324595	2.75139929	2.65476179	2.21931467	2.53872245	2.49573238	2.14423231
2.07847267	2.87729734	2.90811827	2.03486827	2.53934648	2.30926028	1.92616414	2.53808061	2.39024205	2.17040694
0.77605388	1.05661027	1.16234287	0.92377948	1.29423282	1.1637826	0.4693416	1.06460461	0.75807619	0.70288045
1.92194894	1.76883901	1.49491306	1.13000364	1.63479639	1.70767863	1.72832596	1.44388582	2.5241556	2.34814133
0.80127963	1.7642522	1.13469516	0.91031909	0.96975567	1.09579609	0.6542651	1.23668335	1.24327365	0.87866356
4.78192836	5.1298232	4.9749243	4.64071219	5.34893703	6.02458875	4.92781288	5.19781435	5.77053022	4.44386899
2.0786571	1.346774	2.03419178	2.43006973	2.0960216	1.27011172	0.48721903	3.28006747	1.15134868	1.29501014
4.18992181	3.35637511	3.76559536	4.67780564	3.79912663	3.98498033	3.79360995	3.69643802	3.31140401	3.94330134
2.54029769	2.78231279	2.73039861	2.65210806	2.90069924	2.87668581	2.71579569	2.5759128	3.18046933	3.13178423
5.97226265	5.96921982	5.31112314	6.09575414	5.99619574	5.63888758	5.27648906	4.97316434	5.67444129	5.98640572
5.79417667	5.96750667	5.13344454	5.31390078	5.17558007	5.83497593	6.28905831	5.49645025	6.86491598	5.51579378
1.91065608	2.56198461	1.87181141	2.30548044	4.10485805	1.84695915	1.92436058	1.65634076	2.86571762	1.52950084
0.83691736	1.96427154	1.9874894	0.85145337	2.01208865	2.67394815	1.13243046	1.04515452	1.48128444	1.10051432
4.53428429	4.5385298	4.2321715	4.5249981	4.51796887	4.28589277	4.24183782	4.07512506	3.94525322	4.47527374
1.85803571	1.71522986	1.28399425	1.07447101	1.62816542	1.25049345	1.45418248	1.40646946	2.21649646	1.29835052
0.863942	0.05362992	0.80597667	1.49627631	0.41118388	1.43436133	0.01281609	1.28710981	0.16247608	1.22672053
4.38225856	4.81142695	4.73712885	4.63362518	4.86460718	4.13340787	4.67913753	4.44672228	4.06179485	4.63903591
2.82250015	3.46570149	2.78645415	2.23597534	2.5974128	3.10894053	3.47005991	2.92334627	3.25602463	3.09360365
2.57263166	3.4523801	2.40779456	2.68440548	3.15025641	3.54383647	2.54429756	2.61437177	3.02936663	2.63774876
1.78632487	2.00669295	2.15461361	1.67521525	2.37085221	1.33003029	1.37748375	1.91883425	1.03496338	1.72738655
1.33412569	2.04887069	1.71678732	1.2003598	1.74029237	1.51727072	0.94141093	1.46524254	1.54887736	1.57996556
0.26313662	0.24145402	0.06898187	0.08262971	0.1004487	0.32058453	0.52244224	0.23803672	0.30112145	0.11878266
2.39786404	3.40760198	3.21808495	2.46442993	2.88103176	3.66814155	2.28195509	2.27172917	3.85492189	3.24937091
1.52323332	2.01217727	1.91033537	2.0176181	1.70042868	1.81961942	1.19454156	2.08380717	1.88954587	2.11180383
3.99231803	0.19759656	0.64864126	4.13876766	2.77929033	3.2377216	4.15543319	0.9641843	0.48131145	2.88402002
0.54320446	0.77802241	1.02610422	1.04207856	0.75317498	0.2390768	0.27946425	1.43582827	0.30435666	0.12145923
5.82951146	5.54446874	5.0175168	5.66313023	5.38597562	5.25700877	5.60194648	4.93672215	6.21744551	4.91948038
4.18506445	4.62042305	4.45608153	3.86583567	4.47625202	4.53856718	4.49609846	3.87458202	4.93282121	3.94634013
3.48529752	3.99242487	5.0780591	3.91143327	4.07064316	3.94661325	3.53100129	4.33770409	3.34828544	2.80793802
1.05961343	3.10624444	1.76882463	1.84445587	3.336847	3.75734922	0.62232565	2.61357515	1.45972299	4.489648

4.71777003	4.77075442	4.76741599	5.02852832	4.31555948	3.80856733	3.48650159	5.96594609	4.3599341	3.77939799
4.51922456	4.68441464	4.11777766	4.41119952	4.04336401	4.50434276	4.57601988	4.86520187	3.05175807	4.81226761
2.94291841	3.27083032	1.80234408	2.45042142	3.08573966	3.34524035	2.72553014	2.46612169	3.48321003	3.29310011
0.18875342	1.94148428	1.93700982	2.00562445	1.70602654	1.12953298	1.45286659	1.23972481	3.90138814	1.83210767
1.12704671	2.65283859	2.38659836	1.03465556	2.53639247	2.28542642	1.66231312	1.62698801	2.4558942	1.86259778
1.91950933	0.21238682	3.5931449	3.26216129	2.38527514	3.03960587	3.42214833	2.95580938	0.46300407	1.93058385
0.38342482	0.04591774	0.27579158	0.26821929	0.48161035	0.19612829	0.25509469	1.60150443	0.07085056	0.05197513
0.32570654	0.13187448	0.05303461	0.22996182	0.39686203	0.06602772	0.12993387	0.2824703	0.33360549	0.06681118
2.73183107	4.6088075	3.57572767	2.38053882	4.26338471	3.50818675	3.93875696	2.68022941	2.9029841	3.13304131
4.44008307	5.48508397	4.89520416	3.68772025	4.44681335	4.60506634	4.43943202	3.7099124	4.17021484	4.51921396
4.91097237	4.36178779	4.85569188	5.06511279	4.94865554	4.77383215	4.38927347	5.03915135	3.7022489	3.96878805
3.56971001	3.63853602	4.26561409	4.32569046	4.06173759	4.46934542	3.90048494	4.33753187	3.05251023	3.89339277
0.06906492	0.02718869	1.00101132	0.49975409	0.53916277	0.48143723	0.14280791	0.32004742	0.06464365	0.21034683
1.38345158	2.26177155	2.12043566	1.39425129	2.27588722	1.90191987	1.26955559	1.79598798	1.73236541	1.53352322
4.00051483	4.7556269	4.29983471	3.97252504	4.31104935	4.57257377	4.31071708	4.18097553	4.47605198	4.03820792
3.88530578	4.27977826	4.05113364	2.86069635	3.68518039	3.87296923	4.24339152	3.37691344	3.49905095	3.60625225
4.07425486	3.23808597	2.6481831	2.48302281	3.13129187	3.41708236	4.08970528	2.91232465	3.59281319	2.84977865
2.70787461	1.95485828	2.36656321	1.82750203	2.26075406	2.20624152	2.30494083	1.94696815	1.84387915	1.95139095
6.40159882	4.36695759	5.90513648	5.24537483	4.43214272	5.59385087	6.71906386	4.27289181	5.74597858	5.44939679
2.64633059	3.02171745	2.08205963	2.07050294	3.2169897	2.75774751	2.18250352	1.86025027	4.0147752	2.7240341
0.89613856	0.56290467	1.23641772	1.59985846	0.88291207	1.20976234	0.37703218	1.63137978	0.30416935	0.40731254
4.76632027	4.37269459	4.12802696	3.91102426	3.90580685	3.83670127	2.8343858	4.80022962	3.82153075	2.5944514
6.09884738	4.67262116	7.15927947	5.27964445	4.11180193	6.27303971	5.29194619	5.17815146	4.07881201	5.54489161
2.30933834	2.30561661	2.52045524	2.21355751	2.69348321	2.52352183	2.22018012	2.81407521	2.16018384	2.18773153
2.61565146	2.79243392	4.12622883	4.45265163	4.94572113	5.54178108	3.18216956	3.55245909	0.68430569	3.33660219
1.20297235	1.70382286	2.22449643	2.99508907	3.63349429	1.38379308	1.71861502	2.4139623	1.78008637	1.54370603
3.22914061	3.8411005	3.41939803	3.58699255	3.29492725	3.34890713	3.51415716	3.26579337	3.32053043	4.05100473
4.077391	3.216317	4.13390454	4.61598419	4.18401474	3.71812166	4.44624665	4.99777978	3.90607532	3.56581673
1.95955902	3.06727447	2.23195366	2.53155199	2.62377291	1.59956006	0.75965032	2.72757198	1.56391357	1.71453231
1.50966751	1.3986613	2.99457021	3.25460079	2.40811713	2.14872412	1.95515317	3.58503237	1.51529585	1.81563656
4.99123111	3.6271475	3.33053837	3.54010715	3.91433613	3.61322266	3.26643691	2.71159455	3.8277941	1.77763309
2.10226873	2.03863379	2.37209385	2.81050944	2.25730304	1.7400387	1.84044489	2.96686435	1.72979293	2.11074986
3.4684398	2.36231409	2.70040473	2.79524621	2.57971031	2.1001819	1.9312851	2.74300064	2.74553703	1.7323353
3.12002237	2.35883051	4.38803443	2.5630502	3.16681926	3.94600963	4.16557534	2.75243026	3.4365963	2.66912961
4.36885483	5.18739404	4.31097539	3.89263558	4.56443769	4.50713913	4.49081681	4.12819228	4.73998986	4.16986531
2.05463838	2.68742169	2.34160769	2.4249188	2.25211022	2.25022721	2.08544041	2.29873225	2.19736596	1.93505171
1.17257688	1.1770396	1.26519439	0.90691517	1.16521426	1.3482683	1.42408061	1.41134226	2.08418148	1.54779963
1.15271823	2.50708787	1.26103156	0.79188882	2.58699009	2.88111852	1.85857612	1.40150008	2.129469	0.92805467
2.29918228	3.13885393	3.53294416	2.24722584	4.22103344	2.99112648	2.32351384	2.4349907	2.57073796	3.18766368
2.6678538	3.52876327	2.96654261	2.49800967	3.7052832	3.15795776	2.27901465	2.82952878	2.82465504	2.53342064
2.84904427	3.04635542	2.75113885	3.02597968	3.23629657	2.71993164	2.57033992	3.06021555	2.85417362	2.86601233
3.34134548	2.75224494	3.05520556	3.48347627	2.58223623	2.0813013	1.30251594	3.74173145	2.59493424	1.97679571
1.52119365	0.89123326	0.60563787	0.79299441	0.58557308	1.10652925	0.98208362	0.84771711	1.77463689	0.92214203
2.6663146	2.94388376	2.97215872	2.65166448	2.97325197	2.48825059	2.51165537	2.78721345	2.49843508	3.40173968
1.98514919	2.8066112	2.57366682	2.00234972	2.38531734	2.38330962	1.97828868	2.24741332	2.03906294	1.84974209
1.51018792	2.08459601	1.55425561	1.09667888	2.07660046	2.05292962	1.60406182	1.52451733	1.68173237	1.48667988
2.46012403	3.24829783	3.24763031	3.23258071	3.06973957	2.9190139	2.10894185	3.26588726	2.66179323	2.95919262
0.94829914	0.99896357	1.01585926	0.87894663	0.87094333	1.06441011	0.62154564	1.28574159	1.25323324	0.88299513
3.08878294	3.08014713	2.24324604	2.72585877	3.07483341	3.10831108	2.9089772	2.82097913	3.47182784	2.86532548
3.0736032	3.78810835	3.68970391	2.91334154	3.81983218	3.28055914	3.06542581	3.10254913	3.60351329	3.08061848
2.49435136	3.38679739	2.47687681	2.04737945	2.76008663	3.26966024	2.79199935	2.6744889	3.53368731	2.30205279
2.42633874	2.88800894	2.26400808	1.70492593	2.15212195	3.05657695	2.67353083	2.2094927	3.50673667	2.04943895
4.67397959	4.50646176	4.04622307	4.21814078	4.38323372	4.57742857	5.02951404	3.49718282	5.19439578	4.1780714
4.26510386	4.833468	4.22034296	4.0078047	4.72169729	5.13760826	4.55221297	3.94626868	5.00075552	4.23022185
3.08836287	3.34569516	2.51486901	2.65440299	2.64874775	3.44152846	2.55930737	3.19048139	3.33157528	2.44544288
3.17488974	3.55030866	2.27985524	2.16495368	2.35806479	3.34834178	3.23032963	2.94536942	3.60714664	3.27684651
0.53768422	2.0941771	1.30860901	0.66039483	0.88438958	1.11637492	0.59326884	0.69399876	1.55250107	1.51646617
3.14744692	3.17992598	3.96200795	3.43495275	3.34445103	3.92243547	3.17100983	3.28279486	3.46836838	2.96068047
3.36567929	4.70615025	4.89128769	3.86825862	4.34027829	3.95690163	3.13851615	3.91401296	3.63594511	3.57847155

0.28308986	0.07841907	0.24698312	0.69905509	0.39096811	0.0440133	0.05874699	0.56331035	0.0561106	0.11996338
1.07904397	1.38181631	1.86389382	1.65580828	2.13727323	1.37262269	1.04015378	1.75227955	1.24551285	1.0624378
2.16742586	2.45601393	2.51413683	3.18209482	2.88725331	2.19174831	1.52863927	2.86348013	2.51732169	2.88685645
2.22154394	2.65868255	2.74369089	2.09707737	2.52705545	2.78137401	1.99498107	2.27738869	2.63104566	1.97094326
2.35386385	1.92028413	2.45208796	2.75172761	2.33613248	3.31146378	1.29828352	2.6211108	1.89394917	2.05724726
2.3906405	3.34553016	2.18793551	1.33074081	2.64942452	3.57275903	2.37979014	1.98246789	3.22487579	1.91431101
6.97273031	7.63757633	6.5357934	6.02015931	6.3459737	6.67032384	7.58885379	5.93708877	7.5627259	6.25668437
2.45804345	0.46987854	1.25170681	2.25679085	1.12197154	0.57579042	1.97376793	2.31079099	0.34176217	1.00633517
3.47093296	3.37043615	4.48829746	3.40088797	4.55647967	4.10372445	3.61068838	3.48712701	3.56618044	4.16469464
2.11789787	0.15783688	2.40407018	1.81171964	1.66825997	2.8282517	3.65461283	2.20223398	0.25694325	2.07327417
2.05478212	2.72309857	3.18809746	2.63982361	3.01394308	2.22115418	1.24711848	4.04397572	0.35044258	1.03209262
4.11918521	4.78723023	6.02531516	5.15962091	5.40931888	4.58114796	3.54761317	5.14377828	3.9760258	4.14745778
3.90157991	3.9880088	4.82798038	3.8615072	4.51358092	4.38455016	5.12151381	4.38584092	3.24421263	3.72873116
2.4999751	3.19921643	4.57668343	3.91690467	2.06195109	2.94470673	1.22815714	2.86189942	2.85512574	2.67735943
0.61276967	0.89891255	0.60442435	0.76896935	3.47761856	2.4561905	1.3228134	3.21268348	0.29915807	0.70868117
0.26126111	0.01513294	0.0649705	0.08812286	0.05416317	0.13587495	0.92171137	0.09947151	0	0.09056581
0.95184243	0.60986298	0.8968855	1.58018056	1.3218876	0.65563609	0.3446258	2.23446454	0.66037286	1.07524613
3.62783198	1.80074647	3.94970399	3.39874507	4.44596679	3.826937	3.48902216	2.70815065	2.61163181	2.86336515
4.18135448	4.36795043	3.9222529	3.06082137	3.29619159	3.59596677	4.25585614	1.26569754	4.37601021	3.79463258
3.6294936	2.79274181	3.54968632	2.51277662	2.24399066	2.78329447	3.89360087	2.06819881	3.13390955	3.98857521
6.51139503	0.0077701	4.52183776	1.9824432	0.02294391	6.53636124	7.93894156	0	0.79959876	0.21321361
4.13781876	0	1.5084439	0.66612934	0.01528716	0.85331802	4.80379344	0	0.01735479	1.00866826
3.42900684	2.02439068	2.51226331	3.26604953	2.35878263	2.53846053	3.14177339	3.35627166	3.244339	1.79089332
4.73445147	1.90760548	1.8938956	4.18085984	2.29695235	0.47666707	0.53629315	6.97528166	1.34863258	0.447833
0.10019363	1.42467924	1.35590501	1.84158826	1.28691005	0.92514438	1.55111223	2.06126038	0.27036076	0.02017412
3.04881677	3.57078634	3.32595891	3.9100969	3.55153728	3.61244879	3.57179833	3.06531999	3.22847828	3.14032825
0.95835053	0.72644455	1.65797306	1.70859967	2.45234762	2.81127669	1.03930823	2.25756233	0.59741855	1.43125432
2.05601514	1.6164889	2.70509489	3.42628197	1.84269831	3.53177974	1.9272217	2.79283574	1.36749686	2.13159694
2.19031308	0.92933817	2.08416489	2.19414009	2.27939512	1.5805645	1.85339127	2.88534728	0.86847606	1.36498376
2.87865869	3.08707248	2.99011908	2.79277495	3.31259042	2.88746032	2.56793	2.60453461	2.74409278	2.95941916
2.93219119	3.97874064	3.81892802	3.33674498	3.9310905	3.69044173	2.98738935	3.71083345	3.53760297	3.30668559
1.09012013	0.51470522	1.00514534	1.38891696	1.52039213	1.97355928	1.9373012	2.45794359	0.53611983	0.43589564
2.02892503	1.55012557	1.60810122	1.65623384	1.24343609	2.27664186	1.45080666	1.48078846	1.62313243	1.65261619
1.07622026	1.4187957	1.06775504	1.80705669	2.08664083	0.5949056	1.1783725	1.53429809	0.8568823	0.43400419
1.24624979	1.62757465	1.49716579	1.20239711	1.5373661	1.59688678	1.58814558	1.32879988	1.08367571	1.16855432
2.14875029	0.26616239	0.6265972	1.88880148	0.50202523	0.49298974	0.31482454	2.082825	0.16198673	1.02030353
4.68879761	4.9626888	4.92863432	4.68827397	5.25486428	4.42580474	4.47901778	4.65202547	4.20997143	5.30203937
1.91851699	2.14452119	1.92323175	2.05259746	1.60594675	2.25342962	2.08987948	1.91044969	2.55093723	2.35590128
2.72176169	1.5887926	1.42660948	2.97144123	0.91875809	1.03609631	0.21346204	2.61759311	0.8116502	0.68424117
3.80210473	4.16404733	3.78192712	3.89147286	4.72413666	2.76274216	2.36409868	3.17825117	4.41327956	3.08046416
3.90250999	5.50718793	4.02894568	3.00058623	3.69027911	3.71269734	3.16452824	2.46560597	5.02863553	4.12726956
1.13630997	0.58162205	0.975078	1.55697948	0.85310598	0.63787102	0.53745253	1.8582022	1.86599349	0.86844905
0.73055067	1.1958883	1.33239927	1.72867058	1.77743536	0.86905455	2.73442418	0.64594985	1.37974871	2.06661655
2.24759419	1.42249547	0.91928169	2.70103098	0.89998543	1.05375339	0.43355991	2.8261861	0.9447498	0.844709
1.93574265	3.1687989	1.55784946	1.48184036	3.01223082	2.29953445	1.91044923	1.6917103	2.11598889	1.81635859
1.71403996	3.32618476	2.54389072	0.95492193	3.30349824	3.12319147	1.65244732	1.2178442	1.89417383	1.62053967
3.29138376	2.20453038	3.84301818	3.90275507	4.30540488	2.95571781	3.8548127	3.6945091	2.29194036	3.28777145
1.70547203	2.08525064	1.7984826	1.8307745	1.80040367	1.80394135	1.46359329	1.81628562	1.77012992	1.5192134
3.7055482	4.67208613	2.43466445	3.40264422	2.58728619	2.36865894	1.68531283	3.20334935	2.23677872	2.85230074
2.1831999	2.52265186	2.70375141	1.57690252	2.0387576	1.83894862	1.88490283	1.72773289	2.54708394	1.83199198
2.07620463	1.69091199	1.92053332	1.87833858	1.90000078	2.45659489	1.22899632	2.31127631	1.93989957	1.64126375
2.67392402	2.45077566	3.25845659	2.18680587	2.84479456	2.71518962	2.74493045	2.35716136	2.23620995	2.03356164
4.89965886	6.11315696	6.13483011	4.78744933	5.84209952	5.89324716	5.02270338	5.25341199	5.95530527	5.15198213
3.2761394	4.58190394	4.40040562	3.74596776	3.99412081	4.10682186	3.0539857	3.64556965	3.45045394	3.3676999
2.85416055	2.95834422	2.71867131	2.46805923	2.91480672	2.42969204	2.00651493	2.80490281	2.38488434	2.53231295
2.58817893	3.82561709	3.75363204	2.43440734	3.74398594	3.66047516	2.07910941	3.41449845	3.27531661	3.63661619
1.11419716	0.63533502	1.66308563	2.13755123	0.66796887	1.200291	0.78615943	1.92458844	0.41748438	1.20693451
0.89753938	0.55297244	0.60431812	1.33919253	0.83711295	0.56197989	0.23573039	1.5422724	0.44802268	0.69624848
2.36305669	2.81624732	4.05374888	5.2776204	5.08443024	3.51578813	1.52451633	4.46594405	2.97380269	2.62818618

5.2751427	5.96801753	6.61794433	5.72849328	5.92443818	6.23921549	5.66439057	6.26990965	6.17156633	6.36971789
1.60247758	1.64948292	1.82863876	1.99393785	1.66573402	2.07864208	1.31676706	1.74170713	1.32723861	1.9241912
0.36335793	0.12052172	3.28322995	0.84739859	0.35519763	3.0020782	1.95377595	0.626532	0.16512286	1.73218742
0.00890257	0	1.12251368	0.08258768	0.0606578	0.12747031	0.01334811	0.05160714	0	0.02770927
1.95074792	2.12634739	1.30841927	1.05682148	3.44042442	1.72372953	1.76178472	1.36960283	2.82562045	1.67827214
4.08669493	4.65812933	3.83033491	3.40575082	4.39341346	4.92338756	4.56894897	3.91959365	4.72205228	4.22029057
1.26585567	1.207997	1.21461521	1.42826343	1.24931444	1.20821353	1.6881146	1.78447819	1.27400398	0.56319387
2.01662311	2.26039697	2.72884738	2.40627599	2.62809732	2.17371086	0.76105794	2.33563543	1.86337019	1.68159142
1.13706772	0.32973516	0.83556716	1.70464049	0.63516554	0.53468277	0.19735401	1.99254532	0.19169721	0.56617953
1.77841748	0.4762435	0.87777499	1.86308552	1.06188282	0.60477507	0.45142421	1.55799295	1.27131359	0.43825727
0.01759434	0.00675384	0.01132966	0.01675134	0.00890375	0.01314903	0	0.02478246	0.01514009	0.00553525
0.31459555	0.07281642	0.29040496	0.28435375	0.33608432	0.5703188	0.47500716	0.39581542	0.13586601	0.35994038
0.1431331	0.16306191	3.68316877	0.07669707	0.01885605	0.02228354	0.02797237	0.20591358	0.02140281	0.07464123
3.27547658	4.00411123	4.11061169	2.92743172	3.69860614	3.61166689	3.38579117	3.09419565	3.65467729	3.53833245
1.98528353	2.18224017	3.05968081	2.59395787	2.66885893	2.47958082	1.60047519	2.575348	1.6888882	2.04247389
2.82017466	3.3730096	2.889472	3.30158926	3.67161702	2.63776258	2.27955321	3.21076106	3.01672723	3.08723339
0.45897733	0.43619168	0.86339564	0.44393412	0.7341752	1.31077638	0.52287586	0.61339256	0.61450249	0.27393883
0.84043719	0.87681562	1.22185673	1.03141931	0.72930437	0.63493714	0.4275787	1.04391802	0.611541	0.74078358
3.8174784	3.15925643	2.95751012	3.04299097	2.9989585	3.09467135	4.9544706	2.82975486	4.45627515	3.83153523
0.92544922	0.1742666	0.36356183	0.61739739	0.26259484	0.15410801	0.09060946	0.19398002	0.33081686	0.17580903
0.80951662	0.34218891	0.2473489	1.21775689	1.31738961	0.42363753	0.38511493	0.90754078	0.17008863	0.38170307
3.52465146	2.27306828	2.65891817	3.43722822	2.65580631	2.12769155	2.30601277	4.03255944	2.34170371	2.05993769
2.95160748	3.85908338	3.04895725	4.09935712	4.01502113	3.74420277	2.34068908	3.86407436	2.9406993	3.12103418
3.82623422	4.28159642	3.90462684	4.23891162	4.32822751	3.87772338	3.16711368	4.39108454	3.59384423	4.16366641
3.91867746	4.15100582	3.66746204	3.26035803	3.22593903	3.89289987	3.38294798	3.6016353	4.06990967	4.05236462
2.17114602	2.51973305	2.14342704	2.6306257	2.5475301	2.13743594	1.13493857	2.38543153	1.55858238	2.06260811
3.40137456	4.38020916	2.80152881	3.36193824	3.0307569	4.43853448	4.46212573	2.99396413	3.97467139	2.38620679
2.06097095	2.14121585	1.76532226	2.29052543	2.16633559	1.07949192	1.14270526	2.27316719	1.27303707	0.88340995
2.80376856	3.08648925	2.9108552	2.65098346	2.6653837	2.51490715	2.87351261	3.15339813	3.13990216	2.56329109
1.04353411	0.27512782	0.36078925	0.82611087	0.66737451	0.36509672	0.50236074	0.41264867	0.25804294	0.13021414
0.06687269	0.04297941	3.88547233	0.36850428	0.11960707	0.72849158	0.98954053	0.28345895	0.09489132	1.76853728
0.19408653	0.0291289	0.10025664	0.56283761	0.28526082	0.0452157	0.02292563	0.53165349	0.10624694	0.04157988
0.94704165	1.61938351	1.63184654	1.01048055	1.24114895	1.87444683	1.1397381	1.24038743	1.58495947	1.13833417
0.11584049	0.07873663	0.07935773	0.19206912	0.22904468	0.24351708	0.04689495	0.33808366	0.21118329	0.24623536
0.65862798	6.21301134	0.54812956	1.86762089	2.41218955	0.27967503	0.08762427	1.71713752	0.9679507	0.37177174
3.2460034	4.04285479	4.29185814	3.89212586	4.15735244	4.12065705	3.7491314	4.42909537	3.70500557	3.82516789
2.75030089	3.36045785	2.40641781	2.77935944	3.10823478	2.42239857	2.27365341	2.94953468	2.99813089	2.40840913
2.02612666	2.92100512	3.16952735	2.17739361	3.20027742	3.19517529	2.12046766	2.54223056	2.53047935	3.23385152
1.77702726	1.16899403	2.81451759	3.11208813	1.75139467	2.00078646	2.43098867	2.74513239	1.48810095	1.92987823
1.10410657	1.31431006	1.34181713	1.08369265	1.163535	1.16182665	0.99792817	1.11496208	1.27606053	1.16201417
2.37112588	2.68836362	2.41137647	2.0702258	3.43370733	3.28762571	2.71007788	2.48273216	3.13867062	2.98077261
3.39963742	4.03927025	3.77699698	3.19460668	3.15964216	3.50007646	4.33701754	3.76678783	4.77134788	2.99634224
1.4845288	1.00150094	1.14381518	1.73611452	1.85993931	1.83913517	0.94076882	2.38629703	1.07527209	0.43450763
2.49221088	4.2848082	3.57365978	3.32023861	3.6297054	3.34251922	2.58270534	3.41476002	3.43860736	2.92876829
4.14868417	3.28858795	4.83206625	5.10565061	5.12699123	3.4821632	3.87010601	4.79187814	2.97703711	3.40979535
2.0334449	2.11759676	1.46516612	1.57882159	1.78905045	2.74158412	1.90143074	1.53040753	2.38634266	1.41510531
4.01532408	2.95412373	4.34849227	4.88349955	5.21712724	4.01533321	3.7760928	4.12437456	3.06367413	3.71324468
1.76866889	1.82030703	1.8621602	1.40331763	1.98989902	1.50551645	0.86636924	1.76213987	1.95126172	1.29302256
2.09599019	1.75921438	2.51939492	2.02639098	2.33886236	2.15940401	1.97177938	2.42152385	1.65646274	2.24695511
1.20634387	2.17246638	0.9176059	1.18488413	1.34150682	1.18669194	0.84267868	1.42796129	1.32380724	0.70586396
0.50982051	0.11189418	0.25223992	0.24691752	0.05644328	0.1050506	0.01364128	0.07328764	0.10101072	0.07656661
2.64289076	2.68594188	4.20809109	3.16576305	2.93970909	2.70409291	2.2383405	3.71489256	2.08781785	3.02546754
1.3037499	1.67439601	1.5613632	1.91430319	1.61984197	1.35572128	0.95707599	1.63145684	1.0392398	1.55992
2.56762657	2.75081662	3.42962296	2.75781904	3.46621541	3.03190981	2.5366997	2.71431022	2.61678159	3.33468001
1.0133669	0.92163887	1.12617271	0.52676428	1.79147154	1.23941715	0.7704646	0.66699144	1.12842988	1.32889118
2.32453216	0.41886605	1.51450251	2.83670412	3.53816596	2.84233979	1.70986947	1.79132172	0.89694676	2.08599187
3.29747611	3.97454506	3.99497587	3.32954726	3.52907205	3.45834129	3.12902315	3.37247992	3.7394912	3.59763674
1.46526408	0.65716998	1.24783159	2.00283996	1.70845068	0.73929179	0.72142037	1.72032602	0.63622636	1.0496154
0.7447367	0.11013681	1.55690484	0.36480283	1.3742602	0.77943967	0.35654785	0.4165897	0.29785114	0.18810119

3.40904044	3.25164748	3.60908445	3.29726245	3.43769236	3.85029962	3.46574074	3.14090094	2.84093568	4.2715752
1.31534746	0.8951901	2.78025921	1.45888858	2.96060012	0.4252288	2.75677247	1.16253594	0.09310114	4.82523495
2.97598522	3.46889181	3.94761077	3.01093114	3.43148858	3.96461078	3.62657205	4.00625028	4.06969723	3.98761985
6.02426286	1.11850451	2.75441256	4.6012015	0.13300425	0.56179593	4.92624565	2.05428686	4.11209615	6.9780072
3.62389912	3.95713085	2.59871233	4.03621959	4.39406126	3.90214071	2.54254956	3.98559344	5.2619788	2.00600376
1.11341209	0.22791865	0.65544851	1.15795449	0.54779223	0.28519992	0.36417204	1.46559085	0.01672417	0.60652168
1.49952739	1.74283137	2.82167292	1.62393381	1.60177331	1.74401613	1.44713811	1.31437079	1.50832612	1.39408651
3.16128237	3.5241707	3.07622956	2.22253295	3.26246197	3.67853657	2.51958285	3.60920991	3.49777473	2.92817061
4.26156735	4.48129617	4.83853279	4.54332013	4.72356988	4.64311249	4.59728821	4.3970858	4.760771	5.2875058
2.1970729	0.93459304	2.27767653	3.10583849	1.37392745	3.97221537	2.80615925	3.24241014	1.05035035	1.23723122
0.27184871	0.72640754	0.30421173	0.59664829	0.47425456	0.23038213	0.12295747	0.27398316	0.35152263	0.67748677
2.49318787	2.8919167	2.85295727	2.78809402	3.08374478	3.05769663	2.10449304	2.73433925	2.65131684	2.31407896
3.9945616	4.41115888	3.02340497	3.41828543	3.4294388	3.91213603	4.35542087	3.5232715	4.43867779	2.97701852
1.03025685	1.31650174	0.85372765	0.87932486	2.45739194	1.88847296	1.49886834	1.36320934	0.10648239	1.29567016
0	0.00761332	0.00767488	0.0226203	0.00753345	0.01776366	0.02672789	0.02791417	0	0.01863959
0.1088502	0.07654638	0.12638368	0.12637082	0.11818614	0.07836368	0.0153527	0.25943683	0.05065002	0.23017892
1.62794243	1.76157465	2.10994627	1.87438464	1.97149511	1.79324233	1.42789433	1.92214248	2.03300376	1.94576707
1.43518157	2.1199736	1.42000754	0.92852463	1.79973229	1.94032119	1.08644566	1.23960654	1.84434525	1.3142118
3.02158622	1.77127785	1.66181351	1.79361726	2.25781765	1.93570028	1.3037856	1.88595055	2.25019654	1.24377649
0.6452701	0.30704231	0.94573148	0.96121163	0.97335296	0.48886434	0.33541856	1.16042888	0.3757075	0.49051922
1.25365088	1.50777932	1.46961029	0.99992596	1.77923206	1.79229854	1.35491531	0.89942635	1.20229917	0.50610844
1.31430715	3.1033184	2.00984251	0.92556105	2.57515673	2.49569709	1.53866694	0.61236597	2.050944	1.34909338
3.36001077	4.93260618	5.13823677	6.01267967	4.74902496	5.10499281	3.22477356	5.19107671	3.96577485	5.03368959
3.80054922	1.70139561	3.51503147	3.00604914	3.98810306	4.61480295	4.21258001	1.70448503	2.79017749	4.02987967
1.05590579	2.02848642	1.03924394	0.71308571	0.67586568	1.73787539	1.23675109	1.42703942	2.27578826	1.21858609
2.6797298	2.87628482	2.53716086	2.26189656	3.42780775	3.20795135	2.82396791	2.99619789	2.24924083	3.24974599
4.78806745	5.68009977	6.00485434	5.28890677	6.11033664	6.7041507	5.75143513	3.87672034	5.88909956	6.32452899
4.14725682	0.39856735	5.99933087	1.78441237	0.48040797	5.53140194	5.74718134	0.62885749	0.96983397	3.69850862
3.58941909	4.46463965	3.18816212	3.61294966	3.89387908	3.83020888	2.23581173	5.35183768	4.89998959	4.51762873
2.58504103	3.28835869	2.41431951	2.49642756	2.35256667	3.6832384	2.17362761	3.5944055	2.45537538	2.13169793
4.50024627	4.6489627	4.4798877	4.60495589	4.16488638	4.26043373	4.31651424	4.44909487	4.42257739	4.27239397
0.10327949	0.08823381	0.32690205	0.37655863	0.61394508	0.28137695	0.39589029	0.89054093	0.2354078	0.33890048
2.66717585	3.62652579	4.00103111	2.61127257	3.90197848	4.06254789	2.52932456	3.88803251	3.32574236	3.43681369
2.73838116	3.45935414	3.59331444	3.03082324	3.20065828	3.83438557	3.45241386	3.52909263	3.375564	2.9650141
2.64021852	2.44168321	3.12106539	2.49341808	2.58091433	2.55176831	2.17849372	1.93048998	2.64844465	2.43270664
1.00979374	2.40965978	1.58887699	1.21520735	1.88328753	1.47637275	1.02146042	1.49210333	1.27993133	1.50009082
0.24947517	0.92309055	1.07040311	0.23079499	0.64378869	1.04866098	0.48622827	0.47576182	1.10879121	0.31582379
1.62371924	1.85796194	1.80948403	1.09283496	1.86289815	1.8238344	1.12412873	1.5811226	2.08945561	1.06901529
1.15477774	2.41859925	1.40072888	0.75049168	1.42865766	1.53574246	1.14019522	1.05980111	1.99727382	0.9899091
2.20333899	2.57696296	2.07980264	1.52588277	2.05930706	2.56611349	2.05099478	1.85322186	2.29228829	1.3155374
2.34225044	3.54751195	2.36316664	2.01523838	2.79912213	3.59289962	2.34683111	2.30409966	3.41398044	1.9374011
1.77397299	3.09282811	3.11844318	1.17756976	3.56637769	3.15195064	1.79851435	1.81857712	2.65490925	1.76276308
1.8733068	2.58612617	2.35298718	1.72065032	2.54123391	2.93531138	2.05871078	1.85349639	2.7955752	2.37277686
0.51894454	0.55876682	0.82124065	0.66778704	0.526802	0.46300327	0.27376073	1.02903257	0.20245351	0.77571045
0.90505825	1.50006121	3.01086899	2.01763853	3.01685517	2.36642195	2.17506343	2.71748507	0.89255355	1.9239812
2.55232664	4.43483074	3.89434143	2.82508388	4.19945766	3.44617564	2.69508589	2.93202925	3.79301471	2.56373426
2.04306672	1.6197334	3.51162235	1.66033948	2.47669241	2.73228189	2.76599063	1.60572115	0.71624181	3.65697183
0.88817161	1.15640171	1.83308247	1.93459825	2.33222724	0.78414357	1.19643107	1.99360342	0.73801539	3.06979353
2.02744669	2.92010674	2.52561497	2.01551841	2.77044631	2.47068026	2.33165831	2.09202966	2.57590147	1.88124451
2.96199576	3.27258179	2.16271832	2.60894696	2.87543159	3.076018	2.50043329	2.84191239	3.06791672	1.91642916
5.05403631	5.66177753	5.12979339	4.79143863	4.74828747	4.76917251	5.42161394	5.16313475	5.59446537	4.61509382
4.25608457	4.55584556	3.9028369	3.48912067	4.28591796	4.26010344	4.70378338	3.53320858	4.0611904	3.58092312
0.46140312	0.9869905	0.5767086	0.52919316	0.71031324	1.16394879	0.53548633	0.28893979	1.96009675	0.43004229
4.5206408	5.94521973	4.78231039	4.98746607	5.63821364	5.84382091	5.13652514	6.1686495	5.38647352	5.42594495
4.19793339	4.42349597	4.16585123	4.00457176	4.31628916	4.25544558	4.6106881	4.28184774	4.23660617	4.80632055
2.13423238	2.48391469	1.60228399	1.16327144	2.12029411	2.06304961	2.25836716	1.86140154	1.88821326	1.89300573
2.36894169	2.82469994	2.56279798	1.98151588	2.5501405	2.84774122	2.75793238	2.27874663	2.54705526	2.20912066
1.2668932	2.09992198	1.85851142	1.23425224	2.07880417	1.92338059	1.06660242	1.42268356	2.17516568	1.41658913
2.92133734	3.44683406	2.82330618	2.68567544	4.52023872	3.7604013	2.48635038	2.76392891	3.39312528	2.83757174

3.30713188	4.04324329	4.05764957	3.85385037	4.15260409	4.29860546	4.01978699	3.77071053	3.20036946	4.94948254
1.15277849	0.558017	2.02732313	1.87316343	1.04081816	0.85016699	0.4307499	2.20345259	0.45259477	1.03698717
7.77350032	7.81285767	6.70086466	7.15882108	6.75866259	6.9845974	7.88547112	6.85059263	8.06744649	7.08144007
0.03579784	0.08490899	0.5753622	0.90257686	0.52178116	0.35448272	0.06648072	0.34276343	0.03300859	0.48021742
1.48668043	1.09981965	1.89036007	3.21754741	1.42393984	1.94564439	0.41456115	2.62673198	0.66114896	2.96007851
0.561405	0.94668514	0.32160684	0.58508765	0.6277184	1.08288281	0.27513077	0.65815916	0.74222776	0.36713401
6.20567787	6.20617324	5.93597622	5.72290202	6.11510701	7.10339171	6.70644118	6.12726297	7.06133022	5.63213552
4.12445054	4.10254165	5.68139159	5.45992521	5.21444015	3.99674396	4.36306437	5.39633547	3.7241686	5.27143293
7.50916453	4.2402618	5.64831573	7.25770286	5.12507862	4.95058078	4.34544965	7.15119322	3.15739241	3.8075334
5.19795313	3.57047571	5.87474687	5.67082173	4.29957231	4.82682228	5.0350105	6.13206553	2.36473649	4.30640391
4.72047013	3.62314254	4.30335392	5.63781525	3.5377334	2.95271199	3.48130795	5.18849589	2.55546055	2.55131501
0.77891388	0.27606928	0.54390151	0.86684122	0.88653979	0.29340736	0.35825742	1.0023974	0.32803497	0.14684925
0.1415446	0.12294137	0.55158297	0.94397008	0.98927779	0.22847306	0.14532362	0.57248135	0.24376495	0.43458907
0.00595873	0.00759155	0.00955984	0.00849986	0.02241937	0.0088837	0.0022399	0.04496287	0.01277768	0.03693654
6.06112688	8.1072607	3.97237889	4.93847957	4.41521161	6.61626132	4.60270312	3.42945453	6.83331723	3.54983348
2.4984177	0.2057933	1.10014942	1.73784942	0.83087634	0.17840404	0.03433107	0.52007403	0.24347423	0.08787267
4.30593242	4.44069246	4.53113585	4.5759274	4.77054984	4.7718423	4.22370002	4.517085	4.53287306	4.34246047
3.22246728	3.04656554	3.4915942	3.0654812	2.89587698	2.83013584	2.64726396	3.20830219	3.07954468	2.60700701
0.15684349	0.1674961	0.35054808	0.13668139	0.35966954	0.27076929	0.06630892	0.51600994	0.2743525	0.22863051
3.82165775	3.1943286	3.48417349	3.27624706	2.65972501	3.22452543	3.92872488	3.04542786	3.88727628	3.1008619
1.24720289	2.06261206	2.51539829	2.27751429	3.50597484	2.21701955	1.42059758	3.18393167	1.88040807	2.28651339
0.47559068	0.41782109	0.84806363	0.85257117	0.45807387	0.31321788	0.34089012	0.92578116	0.18517333	0.41999252
1.49771077	1.86747041	2.09003811	1.88388933	1.79261236	2.70809708	1.13848542	2.03210626	1.73363586	1.52829563
0.73633787	0.7477083	1.31517473	2.55799878	0.70969567	1.21013837	0.5032485	1.78218781	0.59881781	1.54747501
3.89621525	4.33432663	4.23978636	4.07885896	4.47355322	3.74410112	3.36960166	4.00464271	3.99089768	3.48048061
3.95455347	3.66391041	3.96370164	3.60276007	3.73537643	3.95085379	4.58242955	2.1327994	4.31467344	5.0002269
1.49158214	1.80448633	1.12244126	1.24770177	2.46146319	1.7093329	1.33694625	1.70402085	1.42560847	1.92465825
1.903989	2.62270882	2.76546389	1.96775804	2.79468442	2.71047806	1.73461869	2.3168869	1.84066183	1.749946
0.25057262	0.91062138	0.49532639	0.63379812	1.028617	0.4286566	0.1566884	0.31961815	1.08230187	0.23566395
1.26266299	0.41317549	4.36960249	3.35949825	2.91670436	2.84497287	0.38193449	2.13368702	0.34475899	3.41610794
2.68481894	2.70924365	2.85805553	3.08238365	3.3703018	2.8888816	2.2842951	2.78300396	2.442654	3.17047028
1.39220841	1.98520351	1.38891791	1.01899882	1.58834699	1.89901352	1.45923429	1.13641822	1.8689094	1.24712074
2.442436	2.77345015	2.78153546	2.45821763	3.62248173	3.24014714	2.51240613	2.89959984	2.6194956	2.49644518
1.34886719	1.3531239	2.28844414	1.73667491	1.71738348	1.87215096	1.178106	1.61918745	1.3883459	1.4374671
3.04484186	2.46741383	2.4537516	3.46028285	2.64080248	2.90355864	2.47194053	3.3907978	2.46878363	3.00940915
1.49554058	2.33664466	4.25332264	4.97629068	3.4512425	3.62477718	2.14784985	4.28901588	1.74155814	3.56707853
2.81110283	2.07236659	2.40970426	2.39078779	2.04527059	2.47709364	2.10956202	2.57680974	2.36196383	2.25245621
3.51040641	3.48275015	3.58382791	2.72465201	3.15545673	3.50738853	3.14646183	3.09381089	3.5875829	3.43400161
0.55270527	1.05091491	0.65943663	0.79675882	0.6950529	0.49336553	0.183234	0.73806815	0.50395564	0.9145683
1.80546604	2.91533534	2.51854602	1.84300128	2.21157226	2.17918221	1.53010146	2.11869323	2.06602403	1.86559446
1.70953309	2.64766178	1.44710689	1.46015148	3.83211522	1.99810807	1.50076707	1.58190316	2.08868721	1.46003982
2.2187611	2.64728566	2.17549834	2.39059433	2.55555647	1.99063272	1.74823057	2.2082202	2.35495945	1.89401343
2.09404496	0.89999706	2.29734767	2.49919334	2.25575088	1.80619424	1.31241872	1.92595569	0.99625305	3.26547166
2.96499755	3.66560957	3.33940909	3.05360402	3.89439296	3.86633051	3.03581069	3.16055862	3.72700402	2.83482476
2.66685524	3.6444179	4.05249348	3.30396771	3.35425484	3.86689385	4.08744226	3.12495705	2.53247694	3.42111288
3.67738616	3.83933722	3.45264282	3.59713178	3.51326364	3.77943053	3.47751752	3.57025426	3.64199032	3.32258186
2.30698915	2.63838781	2.83549839	2.27012358	2.75994624	2.70572501	2.04589453	2.70216198	2.92206388	2.11477727
2.89344359	3.60656471	3.29514916	2.87305018	2.95491234	2.9457276	2.83995718	3.32179168	3.17567379	3.64662425
5.38633556	5.65433486	5.64408688	5.64215054	5.60875307	5.74485147	5.71500578	5.5818853	5.75086027	5.5584036
1.44419419	1.33473619	1.26852156	1.23244354	1.40038267	1.47701319	0.85686965	1.2818903	1.52330575	0.85808697
2.19962106	2.63356297	2.38857026	2.40765997	2.4450127	2.23294358	2.16784751	2.61486294	2.29310929	2.41948952
3.02179319	0.21658532	0.30503213	0.38889988	0.05279639	0.53149169	0.26266233	0.38516002	0.05983323	0.27850105
6.73980952	6.39956911	6.3432987	5.87914533	7.00372017	6.42569563	7.69395793	5.34146376	7.27977906	5.3079657
5.73046551	0.23828931	0.5083759	1.53691239	0.11923597	0.36368952	0.03653021	0.33483706	0.11855596	0.06600395
2.6152466	2.89701839	2.56225572	2.53825273	2.71142402	2.74934099	2.77730601	2.90094768	3.1336911	2.58743021
3.77639163	1.86425955	1.59207253	2.23431607	0.54624954	1.76761743	3.5190259	0.68186546	1.19019429	1.08181466
0.82201196	1.51366248	1.58518191	1.28794305	2.07059004	1.70516396	1.92157855	0.86507015	1.77429991	0.84219374
2.85798628	2.93296889	3.454176	3.22289778	4.1674274	3.51764907	3.45756073	2.85553211	2.81871678	2.21501108
2.28752008	0.44254252	0.85708432	1.26350581	0.91251417	1.27981747	1.1775316	0.6076875	1.14164595	0.22745435

1.99665729	4.01946387	4.82367156	3.14662649	5.68086409	5.00022661	4.94242726	2.92330841	3.99527188	2.87788173
6.96674396	6.37441164	5.26674518	6.27840414	5.83795885	6.60706082	6.0677458	5.45216821	6.79724741	5.74603677
2.63841492	1.30245315	3.6866733	1.13515555	2.2958483	2.19159409	2.25472613	2.64197877	0.99287745	0.54085434
2.33754712	2.321088	3.16424203	2.22557558	2.98534967	2.61183717	2.32806794	2.20582265	2.37566115	2.23754215
1.63003556	1.67744887	2.27550996	1.63646642	1.83546391	1.79327019	1.08782233	1.70965996	1.52181944	1.98482673
3.21437714	4.00044569	3.19933688	2.86270157	3.2494248	2.89326859	2.21243334	3.14355231	3.12285579	2.40083053
0.98167771	1.14082945	0.67502565	1.11618467	0.97806364	0.70502103	0.20736688	1.07212492	0.85341181	0.31060266
1.01009533	0.48553457	0.91162333	0.86970821	0.60767418	0.47419768	0.36742634	1.07199341	0.54268795	0.34744532
1.77070903	0.15154931	0.65952896	0.42081616	0.16690023	0.24894007	0.29488283	0.18437749	0.52481242	0.0231891
2.41004451	0.431757	0.33715576	0.61365176	0.77279753	0.7305196	0.32464585	0.26259305	0.57163563	0.053259
1.53563979	0.33087582	0.22249266	0.89383605	0.45177176	0.85983217	0.63381164	0.47780858	0.58482353	0.01515333
4.98139324	0.59370484	0.54583726	1.65120264	0.56518888	1.19974101	0.32134588	0.81514192	0.49780287	0.04268614
7.35091267	1.50498586	1.83425055	4.40125796	0.3294211	3.29704213	0.28935988	2.90360259	1.66554831	0.94614391
4.67926086	5.9789666	3.5313424	5.63360201	7.72123569	4.48104185	4.61776969	7.41119852	5.85977907	5.36578334
3.47058386	3.41143989	3.09092604	2.45899251	3.24373564	3.59471689	3.06379404	2.47579148	3.57936497	2.92414916
2.12410479	2.47498353	2.37426545	1.92133503	2.72063071	2.37267358	1.71365399	2.30830259	1.79657066	2.14968107
0.56767217	0.7255452	0.76389833	0.65741416	0.69522227	0.43295346	0.46639875	0.81546775	0.71719427	0.58886038
2.52638226	2.89280261	3.36985165	3.15045053	3.52870386	3.4401259	2.53185801	3.14847524	2.57735322	2.7534169
2.70165082	2.51832173	3.0717421	2.54667196	2.35134299	3.21298104	2.78390331	2.61034802	2.48128129	2.55544658
3.67788689	5.18365477	3.66671812	3.07373942	4.26503029	4.79090285	4.2159949	3.3095263	4.69251876	3.17899106
0.62108631	1.22793402	1.55577197	1.17243843	1.64748274	1.04337482	0.72533404	1.252175	0.89288767	0.77430121
3.3957169	3.62499765	3.51992624	3.24973686	3.93958355	5.36310754	3.59812002	2.83114667	3.79572202	6.36135168
2.16105406	2.20862397	3.70948586	4.15317337	2.54800747	3.14662964	1.89309244	3.97460901	1.7714267	2.94251294
5.15684662	3.84185947	3.49743198	3.44899485	4.16055549	3.97404627	3.51921081	3.3328902	4.0734462	5.13506276
2.31360028	3.31838663	1.87042235	2.47393083	3.06097062	1.90528857	1.36937224	3.06821408	1.45850422	1.3490498
1.40294649	0.01866249	0.10056048	0.22065902	0.06362752	0.02182482	0.02195958	0.1004563	0.06199407	0.07860652
2.00119052	1.14175786	1.90501212	2.40633849	1.22820534	1.01099963	0.93591964	2.09795919	0.47922315	1.15434117
2.15877519	1.86512724	2.55999878	2.04032392	2.17598007	2.10095523	2.22421352	2.44618414	1.64177546	1.61586712
0.23297825	0.36270321	0.65350812	0.08253233	1.10488026	2.53132863	0.41869332	0.56480153	0.06256191	0.25554742
0.09595452	0.02889562	0.12667806	0.02162996	0.02386807	0.03932431	0.05619575	0	1.06983227	0.13677096
1.72924205	1.3960863	2.42636964	2.63443681	1.93544262	1.69402943	1.53437955	2.91863131	1.19139593	1.90655794
2.13361506	1.30416888	0.89609473	1.00788239	1.51222565	1.17600353	0.66376472	1.36017195	1.02734569	0.25515578
3.13210692	1.56728434	1.57058436	1.75015508	1.94078564	1.81179908	1.10601049	1.86985973	1.76039641	0.68407587
1.31414844	2.16349169	1.71636796	1.62478159	2.14397419	1.53809106	1.56790212	1.56903175	2.01790745	1.64031693
2.44359854	1.92602547	2.50577593	2.8473248	2.32171596	1.98967544	1.48070678	3.53937743	1.54387801	3.84233945
1.14714646	1.63395246	2.73823613	1.83361612	2.10685884	2.38138266	1.73326162	1.94082322	1.51607817	1.91932934
0.7789663	0.3945507	1.27511203	2.20748656	0.40052877	0.92784667	0.21633553	2.50960491	0.13697601	1.30440977
0.13458441	0.06425035	0.07803067	0.03065591	0.06358917	0.0477811	0.09077647	0.22969136	0.18917295	0.04600159
0.13696453	0.04887506	0.12135701	0.11990711	0.10000888	0.12323594	0.05443968	0.17278666	0.10217747	0.07822012
0.04521146	0.0404106	0.01759829	0.01738049	0.03999136	0.04719832	0.02732664	0.02145717	0.14988613	0
1.50261869	0.54935874	1.05890141	1.8470927	1.26039784	0.86839698	0.49715669	2.00028932	0.90751312	2.04545888
1.01390407	0.72565729	1.24095961	1.44981851	0.41059446	0.71032746	0.15228417	1.5036654	0.58939011	0.65609548
0.06982901	1.15881475	0.05634935	0.85936549	5.72953217	0.69585118	0	1.2097777	3.51008569	1.08842357
0	0.00320483	0.34627258	0.16720011	0.02517617	0.23516744	0.13691526	0.17808956	0.02147896	0.09902934
2.52519014	1.38016909	2.09025364	3.29457156	1.02639672	1.61998736	0.8348447	3.2653967	0.80554699	1.027915
0.07769191	0.81409174	0.06272719	0.36696923	0.20678578	0.06312465	0.05872163	0.15566614	1.27135749	0.07610504
0.71964749	0.40479626	0.12307426	0.17876747	0.0139438	0.95773758	1.33037859	0.14890276	0.03148963	4.88091257
0.54645931	1.48965402	0.43723607	0.53219883	1.01923409	0.99569517	0.6714986	0.91115597	1.04300271	0.45791355
3.03516265	1.99245428	3.51875288	4.06079045	3.03545229	2.5567036	2.3514755	4.09665	1.61102239	2.68264163
0.51953837	0.84364421	0.68414748	0.27999054	0.59890708	0.90975798	0.6489648	0.40235015	1.00215784	0.54979118
2.50832649	2.33823599	2.27493305	2.2528904	2.49222707	2.35959293	2.02262011	2.09304466	2.33716851	2.77377133
1.48273573	2.57805462	1.4248782	2.93988461	1.71510838	0.69745946	1.79865962	1.4594845	3.47289481	1.08181144
1.32822366	2.126461	2.76670685	1.65013713	2.09456403	3.25859897	2.62110475	1.1188356	0.78210216	1.18583697
3.86312627	5.44174766	3.31143729	3.95642371	3.46739166	5.04448956	0.43100771	2.65268883	6.5140473	3.57403156
2.6660405	3.79279071	3.83148646	3.79384469	5.0397116	3.29068928	3.51365267	4.15827912	3.41689091	4.6012253
0.92120472	0.98605977	0.55217591	0.97273066	0.49678386	0.80288526	0.77940711	0.83093806	0.94076887	1.68157733
3.55293757	3.92121335	4.40688518	4.34689	3.95155436	3.8606901	4.61980835	3.37494726	2.83107091	2.49452183
2.96148091	1.96733044	2.11784933	1.84466636	2.36316277	2.55404302	2.39900098	1.88508077	2.28056368	1.20894205
4.53714802	5.18274112	4.15375265	4.15564181	5.35915165	5.52689385	5.19525481	4.29208464	4.83273006	4.1884836

1.23792229	3.19112214	1.7829908	1.57187299	2.20709383	1.80689582	1.58821973	1.57153373	2.08822595	1.46217422
1.07334433	0.70728788	0.62196357	0.73050383	0.63305908	1.16186955	0.29318712	0.62180756	2.06968002	0.75713234
4.28093845	3.66240838	5.02285167	6.48904684	3.10647818	4.68700197	1.70310287	5.94569978	2.25715962	4.29678174
4.13731456	2.3642981	4.6118871	5.20286599	3.24967838	3.63171032	4.79369136	3.47994984	2.57541799	4.19993363
3.13423697	3.94833272	4.38973938	4.70036358	4.46652746	4.12322653	3.35678845	5.10744657	3.21340711	4.01401748
1.3718236	0.8326586	1.61332701	2.63585339	1.40631491	0.87486985	0.29649559	3.17257751	0.81434687	0.51595001
2.55884222	1.98563009	2.60073248	2.45022336	3.02545491	2.90990395	2.10771413	3.32910617	2.14653195	1.88675683
0.31844278	0.3424975	0.90261801	0.96186596	0.53841893	0.48942557	0.51210232	1.17725847	0.26249281	1.81764824
0.53401039	0.03474081	0.27572605	0.08024121	2.50990015	0.02333365	1.07268504	0.94535316	0.04448063	5.23098288
4.40158013	4.72359312	3.99230031	4.15384444	4.27904131	4.3397813	4.25044512	4.26247491	4.42262113	3.78589786
1.23190285	1.31679888	1.6736565	1.90313756	1.69023056	1.71853465	1.22451237	1.62184874	1.28733113	1.60397059
2.44967183	2.7797473	3.03938506	2.2692844	2.95212992	2.21106597	1.93911988	2.31312109	2.2037289	2.52479937
0.3127527	3.64951019	0.42894828	0.81668361	0.29826783	0.20852091	0.12235273	0.88915493	2.38486251	0.21812452
1.3695716	2.66129562	2.69272924	1.17524754	2.91537167	2.93740716	1.6797618	1.49310349	2.22883485	1.75154789
0.54188623	0.65958237	1.51534173	2.12635208	0.76510774	1.47586804	0.23225805	1.94828844	0.85719463	1.27221844
4.37238803	4.50947432	4.91677247	4.93708201	3.6545704	4.25352898	3.58261618	5.1073002	3.61564103	4.29105896
2.0651852	2.9690823	2.46626284	1.93365664	2.7169315	2.37965625	1.68721832	2.14113197	2.49973942	1.91761917
5.29730874	4.91951993	5.06146058	5.45035436	4.95317241	5.01394571	5.89042577	4.97657558	5.21738156	5.730531
0.43854241	0.39507806	0.27310274	0.25230481	0.19674489	0.23004775	0.52674169	0.48526042	0.75368436	0.47207237
1.57106597	2.03831808	1.82097481	1.94205697	2.66062391	2.28168399	1.15739888	1.86646852	1.95247411	2.34153197
2.13232139	2.52112826	2.34978343	2.12981524	2.53539488	1.85398633	2.15320873	2.35617748	2.44911129	1.99412553
1.88724341	1.26583575	2.0145951	2.42166048	2.03857495	1.88852688	1.36252195	2.92864555	1.31097131	1.4218404
3.79961292	3.3701669	4.7349233	4.90690733	5.95062717	3.81369627	4.5923578	5.69336211	2.70250554	4.76329369
3.16525922	2.56403972	1.9278832	3.12156924	3.03603912	2.90369608	2.84871537	2.63030504	2.80014703	2.03853928
1.521247	1.49864265	2.3383765	2.08112939	2.20961388	2.13115793	1.58795088	2.36647785	1.25066614	2.0947384
6.80220033	7.13784139	7.48968709	6.72286135	7.34080641	7.31419085	6.56131243	6.9108371	7.71881641	7.1014039
2.67887795	5.23404746	0.69369791	2.34316602	1.70084669	0.59661203	1.41567939	1.67929803	2.52076337	1.46432909
5.56754868	5.34288376	4.35117078	5.19434595	5.25080248	4.77385397	6.35532182	5.13240141	5.90124329	5.10793617
3.14194946	4.20501225	3.09388747	2.89818255	2.79613456	3.65198974	3.5820478	3.41958293	4.2125644	3.13702703
4.68445713	5.7327759	4.7840517	4.23270265	4.60734258	5.29043332	5.13735692	6.40714355	5.63870305	5.96539538
2.52394246	3.20212448	2.8308484	3.10654063	2.76595917	2.68596923	2.31059717	3.10397715	2.47943247	1.71820903
2.30281063	1.64850475	2.60037908	2.80903403	2.29013051	1.75613987	2.2463149	2.92226844	1.501053	1.77235803
6.98655696	6.83293418	6.96270908	7.20670584	6.40797837	7.38258367	6.94344762	6.85293719	6.52777127	6.96548356
3.53474105	4.70619395	5.51577188	4.63222359	4.59561666	4.59518548	4.29998218	4.87488627	3.85256897	4.79684405
2.07221566	2.26253987	1.93710766	1.87412459	2.34546186	2.58037453	2.2760965	2.34569212	2.66195147	1.74504734
1.86074558	2.53383051	2.06754023	2.06465452	2.29555648	2.2261801	1.66352796	2.39912944	1.84677571	1.77378431
1.23086691	1.90502868	1.634335	1.34205509	2.37991717	2.08641264	1.01102843	1.60210019	2.0779832	1.42088384
3.51082011	3.90299145	3.71208103	3.25469491	4.19250228	4.08622298	3.522792	4.09669997	3.38083657	3.39326048
1.80381452	1.87479754	1.6668528	1.65511637	2.09536396	1.77836284	0.98746013	2.12998723	1.88265923	1.95516552
2.74429798	1.43775057	1.62750553	2.07972684	1.87695207	1.67439595	0.8979018	2.49049661	1.49390821	0.69043562
4.48817058	4.75449973	4.7511959	4.6095992	4.94735965	4.66971812	4.80752374	5.01380789	4.18514202	4.67182101
6.4013555	6.65038632	8.4221825	9.63345055	7.56779476	8.05403337	6.44763817	8.80086065	5.6467779	7.82078958
3.96050786	3.93362777	4.86560809	3.93208309	4.22676011	4.43769077	4.52067206	4.60282962	4.37122934	4.90608068
2.70551384	3.59171695	4.82123952	6.93146764	2.1984251	5.08822489	3.05069083	5.78248147	1.31412778	5.32860041
5.54103893	5.34273937	5.74965869	6.09602026	5.92961996	5.75816309	5.2269935	5.64646557	5.11871647	5.48385644
1.02335085	0.5183171	0.78188678	1.54938697	0.83541668	0.54375524	0.24385677	1.57533606	0.2498061	0.35341322
1.03326343	0.24612755	0.21216886	0.26442536	0.22428129	0.17333677	0.03233934	0.26462291	0.12926705	0.03371825
0.94600889	0.62198536	0.73247191	0.78316773	0.80459112	0.71061594	0.53877759	0.84484789	0.84019426	0.6349683
1.40996519	1.39613055	2.51482049	1.89726991	1.77585442	1.44269563	0.84133459	2.86263971	1.60702902	1.13744093
1.49031848	2.68972776	1.66298857	1.45625473	2.33526539	2.53539578	1.34017911	2.60781088	3.58946661	1.30342565
1.15772628	0.69754122	1.46605037	0.71422261	1.63514213	1.73133468	1.80912529	0.95407547	0.51942464	0.41896414
2.36562811	1.99571429	2.58633102	2.86709296	2.31035111	2.61165839	1.83519652	2.71318778	2.30232066	2.55748843
3.97298848	2.83923437	3.78317104	4.24961977	3.72907837	3.02482731	3.78187678	3.88944049	3.75966869	3.97362329
3.8937975	2.56800504	2.85259239	2.87199658	3.04362767	2.7266245	2.13323973	2.81482377	2.58958716	1.90935147
2.14370968	3.96303091	3.57032933	2.73860109	3.25733613	2.90439037	1.80817909	2.80556755	2.93235139	1.40431648
3.06081219	2.28548146	2.99347838	2.95807598	3.13051841	2.75344575	1.86748086	3.82382591	2.26638656	2.01885095
2.41058426	0.80879397	0.54146658	2.47998443	0.87900682	1.58430782	2.33580434	2.70579964	0.42368659	0.27420553
1.19413729	1.96155954	2.08607213	2.39218308	1.55784515	1.80135999	0.56416393	2.70446238	1.58244755	0.38668507
1.23862914	1.5900763	1.36542357	1.14269614	0.86650642	0.91713674	0.53978106	0.93375837	3.25508235	0.48782314

1.31998468	1.30332175	1.40353101	1.48768084	1.80571859	1.17202137	0.37813439	1.5592584	1.21568778	1.1061239
1.76037805	3.7865557	0.93850071	1.08276688	1.25779196	1.02181735	2.32511161	1.25733221	1.09574502	1.37651924
4.81308765	4.26325408	5.98494568	7.38447422	5.62550811	4.91440422	4.96598198	6.11693467	3.32666897	4.68263071
4.35025456	5.79786243	7.44177207	7.30470646	7.95816059	7.09853465	7.31144981	7.93931516	6.17864476	6.98371241
3.0115423	3.170899	3.23599471	3.42264292	3.43751447	3.02049048	3.38956707	2.43498023	2.68466056	2.27740702
3.71151193	3.37737052	4.01585329	4.2529513	2.98098437	3.36804223	1.98179383	3.7805522	3.24399511	2.68347783
2.63760127	5.16508284	3.53477094	2.6310038	3.69068451	3.61917692	2.94526901	2.72297286	3.57953046	3.04612218
2.67497403	3.38241439	2.59581045	1.93221457	3.27073011	3.11129972	1.92321416	2.39506836	3.2310444	2.25654432
1.42845249	3.04632742	5.30516922	5.33617693	5.21059653	3.78756357	3.38860169	5.99893887	2.53273477	4.21511056
2.93899633	3.05012484	3.19332455	3.21014884	3.85357054	3.32644896	2.761885	3.32500773	2.62516819	3.94469651
1.97263506	0.7424379	2.41190607	2.71540566	2.92459779	1.29263095	1.99311384	3.50462723	0.76214396	1.78573559
0.45770571	0.50227083	1.46053065	1.75688032	0.64858943	0.40421603	0.04968805	1.43425811	0.38484212	0.62091228
0.04286217	0.06770849	0.02768643	0.06081305	0.02717967	0.016141	0.07943555	0.07481659	0.03083869	0.05022704
0.36055971	0.04966628	0.0168811	0.0972597	0.00830928	0.12279895	0	0.11931555	0.17790826	0.02055107
1.88790516	2.01972858	2.61155625	1.91127953	1.93427207	2.29021352	1.89625143	1.98369454	1.93096925	2.12090521
2.71347992	2.62433527	3.12076482	2.02354068	2.7406617	2.85715521	2.31130481	1.94936234	3.10091892	2.61338588
1.60698516	1.93656129	2.21656661	2.01160563	2.00677167	1.705995	2.14628524	2.29968717	2.05655799	1.82749487
1.64573763	2.76444933	3.27775962	1.72349742	2.15281072	2.21591642	1.55884208	2.0083046	1.56715808	2.09746393
0.22961189	0.42043964	1.22572683	4.19314855	0.63448269	0.52176496	0.01430405	1.24744587	0.13103913	0.29709433
4.85393079	2.65615763	3.10545669	3.37809505	3.46206435	2.43599492	1.60495274	3.51975653	3.11899697	1.2066819
0.02146745	0.02730996	0.0138303	0	0.04035054	0.07851717	0	0.02522828	0	0.04994413
3.18667344	4.04566975	3.55220679	2.93195978	3.80847514	4.44941675	3.23551445	3.06600926	3.86377511	3.28908587
6.78088227	6.25835366	6.93002838	7.28391883	7.15814991	5.92090863	7.25697591	7.16203017	5.2692153	4.39444451
5.94904085	3.64233768	5.21976976	5.24716108	3.86839014	3.24299838	3.03839801	5.90882115	1.50394214	1.30278083
3.6426842	2.57587242	4.62943834	2.24393217	3.22625996	4.17508642	3.46065022	3.90091556	0.66722724	2.89465147
5.05655671	4.91002297	5.37382375	5.94084573	5.77530836	4.9991703	4.44585186	5.72675965	5.11011159	4.12598133
2.89799939	5.15135967	3.54153282	3.39499211	4.34086089	2.45690128	3.86928858	3.501822	4.58315279	0.94574087
3.65737708	6.51290732	2.19860334	3.60647316	5.1038904	3.93228927	0.96181276	2.31701159	5.25858109	3.13163798
4.29476869	0.37648744	2.98992951	3.69587138	4.58539274	5.01108299	5.20611017	2.67894651	3.43603013	3.34592933
1.85279573	3.46553342	3.00571747	3.12636722	4.24326871	3.67062222	1.8598017	3.73818118	3.82465042	1.71718104
1.11468586	1.39562447	1.60682726	2.63434769	1.95067241	0.75976593	0.25891249	2.31763599	0.91337829	0.86411751
1.57303411	0.25088288	2.47858781	1.58186179	3.07396296	0.43234821	2.8392012	1.30613158	0.24245987	0.22245055
1.11065073	0.99695181	2.51749495	1.4100497	1.12065713	1.55782645	1.25386728	0.8230007	2.6740014	1.23258146
0.26381733	0.08942321	0.35297491	1.2992502	0.1719039	0.29229495	0.05333939	1.63839686	0.11936636	0.17085338
0.42143326	0.39790387	0.03878488	0.15185831	0.15531584	0.03383939	0.25222758	0.21683361	0.46445795	0.37732288
3.5085097	3.09008181	4.65544138	5.09803045	1.99975872	3.45564091	2.55149218	3.91305545	3.00965299	3.55749374
2.82887094	1.68436828	4.30715929	4.27379075	4.13868111	3.0760897	1.91860202	3.29691639	2.04488946	3.33503752
0.11584751	0.06758693	0.04577027	0.0227817	0.13775114	0.95296924	0.07069529	0.45824856	0.50522187	0.12655161
0.19236649	0.53556663	0.06464109	1.86983675	0.22640627	1.40906134	3.95582587	0.14087185	3.04344553	0.06135496
2.44823804	3.00266135	3.29647744	2.9985625	2.55073244	2.6057767	2.34570727	3.06035363	2.42433807	3.23006347
2.58945143	2.96741638	3.02295403	2.82613071	3.46305838	2.94766914	2.18566676	3.00558247	2.58259853	2.63296142
2.49205552	2.74066138	3.02756975	3.56538842	3.58568643	3.39166216	2.81465338	4.3233342	3.69989677	3.38414255
2.56262916	3.06517405	3.15061143	3.1093631	3.10743097	3.25759274	2.43269352	3.08127089	3.06452238	2.76578523
2.97072114	5.11934274	3.86796809	2.91265223	4.33962822	4.3243366	3.89921899	5.65329622	4.34694697	3.78602195
2.1078353	2.74931108	3.00212274	1.83274986	2.80057158	2.83763937	2.17466463	2.30331715	2.57328861	1.84150963
3.36248086	2.96403434	3.56232644	3.80614999	3.10973949	3.19733086	2.50045183	3.48521089	3.06408304	2.68383812
3.44343916	1.73646591	3.39602143	3.14732596	3.26563708	2.30989938	2.76959978	4.18094784	2.43213882	2.91102776
2.90685823	3.75388414	3.80567873	3.37149562	3.95732293	4.02238364	2.17788195	3.44418686	2.89224613	2.94366969
2.50922783	2.77549794	3.29749063	3.30648257	3.45205629	3.4449118	2.27722368	2.90576929	3.22527563	3.74228945
3.25023713	3.15899403	2.62744123	3.65548079	3.35428117	2.51571183	2.90880915	3.42903957	2.69682577	2.05354432
0.47494246	0.23778565	0.89243588	1.28620729	0.60424351	0.72341871	0.12644465	0.96484306	0.2065382	0.65475578
4.7404339	5.0983917	4.60354103	5.90166742	5.9567827	5.67027254	4.74559013	4.70495098	5.42623336	5.17251176
3.53810431	7.97663532	4.29382044	5.40384977	7.42255004	7.14652241	3.38619619	4.4805075	6.91875161	5.94205723
2.70805859	3.84825084	2.45999457	1.83895686	2.73455249	2.82628781	2.60482668	1.91904871	3.47508492	1.76394337
2.88934618	5.40196675	2.81506977	3.47119514	3.63748316	4.05573917	2.17513862	2.97894479	4.30616962	2.95494485
2.91702551	3.10175925	2.76788086	3.98183854	3.40411508	1.83079316	1.63627671	4.0619411	3.10330586	2.36822754
4.30963859	4.59489042	4.20709859	4.13628404	4.67736127	4.56363121	4.15061842	4.45608706	4.44499319	4.35416194
2.15217097	1.37236166	1.69805166	2.08686067	1.53499047	1.33174949	1.56394223	1.66199697	0.76013803	0.71767554
3.2998778	3.26650294	4.07328149	2.98550875	4.65083202	4.06427369	3.63419946	2.37910161	2.80600555	2.76696111

0.19638671	0.28058712	0.08502468	1.18560674	2.70457698	0.60218448	0.18162979	0.70129377	0.8948301	0.54444961
2.42249543	2.70015033	1.38919618	1.60303062	2.10044924	2.20408886	1.12068576	1.99428112	1.93315345	1.77113066
4.68535609	5.64708759	2.93728878	2.86982308	4.09517739	4.70950283	3.77864103	3.374321	5.24102964	3.8087742
1.38734852	0.31374569	1.26943681	1.27809948	0.30362662	0.27894252	0.29604404	1.38077431	0.16685485	0.11605715
0.04939441	0.12600332	0.05628735	0.3983643	0.02097402	0.14645793	0.00418606	0.20198686	0.31927663	0.16249889
3.62103029	3.68328713	2.54713046	2.94887726	2.96138508	2.89183794	3.64461899	2.94643646	4.40622571	2.63777322
1.37634128	1.60820434	2.51657217	1.30596975	3.25860682	1.81221983	1.64377147	1.60411992	2.0945401	1.6282347
1.11683694	1.14068912	1.80702219	1.60629494	1.69256948	0.98515823	0.83041046	2.34830412	0.98120818	0.8128534
1.32591503	1.06111647	2.50114016	2.41881512	1.78726585	1.87257785	1.32399801	2.40590655	0.82175779	1.48002373
3.29411664	4.50330627	4.34185103	3.25404321	4.06863232	3.4680645	3.27452438	3.76544383	3.42225876	3.68613792
0.42101663	0.00980668	0.09283967	0.15760687	0.02891796	0.04535985	0.01537352	0.11629679	0.01467367	0.48204402
0.54586488	2.2178453	1.24683882	1.06115785	1.59457158	1.34610439	1.14973087	0.85992055	0.7303242	0.79689152
0.26813463	0.18896633	0.67251922	0.79804636	0.03669703	0.36049102	0.00552064	0.2537189	0.01574814	0.27496564
1.97250107	2.30444506	2.66790079	2.24678215	2.99345328	2.3762732	1.91154166	2.20730929	2.24844991	1.72919264
1.55964202	1.72083206	1.99725896	1.85345081	1.98725777	1.96601554	1.04727437	1.51661978	1.5666622	1.58572954
0.93841353	2.99447779	1.67910932	1.87054602	1.36100145	0.86400372	0.36685491	2.1013725	0.95681021	0.66242264
2.10384191	1.96390325	2.35352169	1.60303082	2.27800386	2.07345664	1.7686694	2.007183	2.17915599	1.46841363
3.95862297	3.6689099	3.39472342	4.88415626	2.98486493	2.61271201	1.13921339	3.62705295	3.29715527	3.15299166
5.31204405	3.87705078	4.96420064	4.8534866	7.73994877	4.22997085	4.56951924	6.06756211	3.81947611	6.67916625
0.33243578	0.33074218	0.14608657	0.32942801	0.60068547	0.42258255	0.55283607	1.05197442	0.51676877	0.35166183
0.41430917	0.64071924	1.73468777	0.3633143	0.4725135	0.82888104	0.72167161	1.04937273	1.12304376	0.30333691
0.02018896	0.01690833	0.01785125	0.01324292	0.07495101	0.02816836	0.00856119	0.01930834	0.01268987	0.04023799
0.87877189	4.4193316	2.29532538	1.68437457	2.27791476	0.47973025	0.61780845	1.40632371	4.3922724	0.55509851
5.15017775	4.96704242	6.28125352	6.42688275	5.38395253	5.226981	5.0593266	6.61327902	3.91870553	5.00971156
3.44279369	3.24688582	4.42397566	5.09275527	3.66296426	3.37857801	4.96326793	5.33866913	2.78236539	4.1410956
0.01681625	0.02140228	0.12485759	0.25511176	0.03165273	0.37892089	0	0.05212394	0	0.0175567
0.36821365	0.04576338	0.03473393	0.02580611	0.07834731	0.0135451	0	0.12333922	0	0.04222874
12.5842837	11.3536304	11.2112879	11.8897835	11.7687024	12.095775	12.5643761	11.071404	12.2863207	11.4953769
1.73444729	2.12576187	2.0824263	2.44860654	2.28773979	1.91653812	1.47043218	2.5797398	1.60016915	1.87706216
3.49032324	4.02751845	4.46336876	3.69567974	3.80460851	4.45044398	3.84076163	4.00667945	3.87566099	4.2159976
3.62735253	4.59809288	3.11768492	3.29116112	4.81658975	4.44062762	3.460583	3.79113308	4.02552154	3.44925999
2.85523216	3.47568876	3.69245079	3.13130996	3.7826561	3.68374569	3.17416486	3.53916997	3.58104235	3.41675905
5.49750582	4.82190186	5.25974967	4.56814861	4.83189445	5.24444543	4.70479112	4.21073152	5.3630358	5.73293867
1.95706554	2.05204322	1.77350187	1.88676904	2.71822929	3.29903035	1.80285859	1.77864939	1.91304334	2.19233478
2.40416589	3.26449458	3.40237123	2.71839762	2.73564527	3.03361962	3.11439296	2.69083955	3.87329255	3.18524092
4.82575893	6.51712354	5.47271535	5.40662742	7.80679259	5.60988398	5.13758402	6.80185807	5.27739932	5.19695586
4.09045752	5.16681976	4.16625329	3.90311629	5.13311852	4.68927296	4.56931784	4.49835947	5.41865959	3.89119281
1.54723422	2.05239341	1.31033257	1.30718876	1.60800286	1.34508721	1.22466688	1.2018151	2.14348717	1.04564807
4.21217162	4.83021937	3.23668617	4.0637918	4.12138683	4.39110429	4.62591419	3.0107904	4.72789059	3.30584243
4.97943635	0.1581314	0.43332306	4.44733883	4.17926792	4.09112533	4.45877476	3.45686816	0.33392479	5.35570366
2.95339781	4.03092184	2.56782918	2.35131364	3.12613475	4.31561804	3.40397893	2.83537812	3.47401978	1.91257157
1.6999366	2.05993575	1.22659671	2.02823817	3.02117291	1.79802046	0.79499289	2.6305028	2.47622995	1.8052139
2.46510946	3.31075272	1.44135804	2.11363534	2.74488397	2.38960599	2.54562305	2.37794848	3.64617076	1.76001937
2.12818313	3.20421148	2.42961993	2.20809724	2.71180534	2.68820125	2.23612822	2.3649624	2.80031772	2.65143214
3.23016298	3.79717181	3.28439535	3.03362375	3.34751767	3.54106933	3.16000802	3.52169579	3.44372617	3.7505041
1.52597873	0.32912402	0.63372407	1.69329719	0.643332	0.33280558	0.33465363	1.95014428	0.30282618	0.23722254
5.22112784	4.5722169	6.17271349	5.71608405	6.73799574	4.944384	7.74944162	3.80737027	6.77022632	3.7232778
6.37999054	4.92243641	6.72733226	6.31853998	7.18959608	5.74594746	6.19870669	7.02363075	5.21571906	5.34848957
2.82234048	1.99545305	3.0517337	4.05373024	1.91470147	2.4227982	1.81579128	3.39181153	1.78012696	2.15261943
0.73514445	0.1232338	0.1536375	0.35324282	0.38014344	0.19899415	0.06180383	0.57908388	0.15956257	0
3.33204541	2.27313773	2.69268919	3.15066826	3.88549093	2.51709504	2.03333312	3.46101286	2.58143289	1.70603165
2.57849356	3.07948833	3.14190943	2.94334378	3.62373956	3.41225084	2.09686622	2.66018651	2.30414045	3.20495074
2.45531905	2.1668332	3.09218299	3.65637198	3.54612865	3.02320212	2.92144291	3.72498002	1.83641575	2.30127118
2.66019085	4.13005596	2.31411098	3.66190539	3.58857015	1.98463516	2.57231891	3.36851538	2.20370909	1.10341839
1.16750873	1.80974995	1.41038349	1.3083493	1.62394598	1.30000085	0.73604289	0.65876506	2.3983537	1.10410916
2.70652909	1.92477356	2.97905513	3.2595683	2.67942204	2.17620614	1.11241041	4.20149791	1.75886942	1.76740517
3.03902049	4.66035602	4.28589654	3.41811811	3.72686876	4.08571632	4.50079755	3.20703456	4.35729667	2.58953292
3.13280311	2.04066867	3.05435788	4.11493096	3.52656472	3.70341409	2.65478617	4.05169888	1.72597462	2.31120156
1.89171682	3.18313071	4.72351449	4.03272575	3.93443712	3.21064323	3.15310895	4.31341211	3.13605438	3.33782392

0.18176184	0.02448386	0.56864139	0.42308713	0.6715195	0.3186177	0.09384253	0.18514403	0.05447373	0.16244709
3.74426238	4.03369561	4.05533499	3.58619414	3.65553825	3.44760796	3.71275185	3.7630255	4.01064626	3.70962103
5.13333855	2.85254873	2.96941775	3.77819635	3.287395	4.60598404	3.40333226	6.65351279	2.43240164	2.17649219
1.22589652	1.95317371	1.85498794	0.94818357	1.67892962	1.63764978	1.27121477	1.51781871	1.56922097	1.29474488
3.58216988	4.32051494	3.92325061	3.39238381	3.63221337	4.2770035	3.90759435	3.64835537	3.97575772	3.8867854
3.84237569	4.6142962	4.27607061	3.9035663	5.05725098	4.29607023	3.93362544	3.8084772	3.74951334	3.89784437
0.03618397	0	0	0	0.00576562	0.00681927	0.02048765	0.02139901	0	0.00715686
4.40671139	4.68858667	3.9974422	4.61417709	4.26531006	4.05601296	4.50706278	4.28226473	4.35790287	4.61646575
4.16409641	4.60057937	4.25785328	4.30446316	4.5672369	4.86357389	3.99556101	4.18349482	4.17216326	4.8369859
4.20391578	4.43783891	4.95408391	4.80221598	4.83278116	4.00884059	3.74751224	5.2813312	4.52849187	3.84797518
1.61543809	1.73919468	2.61951928	2.28395042	1.76552415	1.87511131	1.15494758	2.08214465	1.67226913	1.42994575
3.07029012	4.55624244	2.5936941	4.3250055	3.27531734	3.40868006	2.09966704	3.61720401	3.17611165	2.62093325
1.34495356	1.0498029	0.99251554	0.9196735	1.30571951	1.00725348	0.50615171	1.21533172	1.22450917	0.35594197
4.61552912	4.28811296	3.49469579	3.9156404	3.76724138	4.15114063	4.50580506	3.745347	4.47171482	4.0770468
2.92250167	3.14320931	3.35258782	3.80501764	3.03677672	2.88038292	2.71894681	3.63753117	2.58451826	3.98429541
4.30968607	3.91053198	3.73661811	4.16658646	4.02754454	3.46761286	3.9383427	4.00364354	4.3486004	4.29245214
1.10030144	0.36711385	1.09540131	1.12471333	0.85249933	1.24481402	0.60531165	1.59210478	0.48551778	0.5175815
1.74284353	2.09669079	1.80449795	1.42864707	1.91156689	1.89354339	1.46929532	1.59672027	1.95974914	1.30483058
1.60004577	1.68097759	1.46337202	1.30688326	2.19424302	1.83506378	1.16994354	1.66606168	1.71853654	1.53058942
0.07399409	0.02716483	0.0542559	0.08421232	0.01429387	1.36367979	0.64499516	0.07824715	0.0144338	0.21335205
0.00958359	0.0243086	2.1515733	0.09731587	0.00605146	0.85651022	0	0.02245392	0	0.02241819
0.89259825	0.01503833	2.40060013	0.48440997	0.02814384	0.48027218	0.01945709	0.14839144	0.22603401	0.08301604
0.85925841	0.67354838	2.45356711	1.40202864	1.34109906	0.64798724	1.1362356	2.67008712	1.65375233	1.04454325
0.31229018	0.25110601	1.43731128	0.1980371	0.23022123	2.53950062	0.10021065	0.86179164	0.17764703	2.74212848
0.05316544	0.09193284	0.07624502	0.08749979	0.08295645	0.07864053	0.01013013	0.15112153	0.01927199	0.13166138
0.09908928	0.06617003	0.06219236	0.03445259	0.16775113	0.06153914	0.11621342	0.51023718	0.46300407	0.3854506
3.62514397	4.48319678	3.80318231	4.09650453	3.21910554	2.90516833	3.42851497	3.77610849	3.87176793	3.71033441
4.00317291	3.91050555	5.09920677	5.37407658	4.3945432	3.4669936	3.63132174	5.0855202	3.38790971	3.54118081
5.35632289	5.07065241	4.64190259	4.88448574	4.31803324	4.35311701	5.75688195	5.01906277	5.81245855	4.65485986
0	0	0	0	0	0	0	0	0	0
3.26018783	3.4666248	2.99709886	2.92665042	3.94514127	3.16135974	3.43659403	3.13187441	3.67423738	3.36857218
2.28237896	0.09512076	0.09586687	1.03341111	0.09415213	0.58644697	4.30984368	0.21633614	0.28557646	1.30546083
1.9480665	2.71545556	3.33523554	2.40389853	3.6149447	2.45595055	2.64175249	2.79286763	1.75854876	1.33615256
3.01110885	5.45220504	3.35482737	2.76846666	3.0781556	3.55368199	2.69947849	3.44257425	2.78222944	2.49261907
3.77199179	2.85467769	2.7987477	2.84960156	2.72084962	3.32414925	3.06807937	2.69917546	2.60475151	2.74131027
1.78352299	2.3600747	1.53241078	1.2561757	1.94342016	2.14044966	1.29898063	1.37615875	1.07247042	1.40113222
5.47763264	5.47920178	4.39111818	3.82356765	3.56650068	4.89251636	4.18544301	3.71649738	5.70468214	3.24184323
2.00142002	1.86525582	2.23568174	2.30458528	2.36200618	2.10215546	2.35879108	2.80791667	1.74815221	1.85288876
1.94898758	2.53526854	2.3449152	2.16413941	2.09417038	2.3675309	1.50269051	2.46273731	1.53866891	2.35337198
2.55173612	3.79357196	3.66458988	2.91947613	3.69006972	3.54017571	2.26209306	2.94710766	3.16863335	3.84510393
0.35561171	0.09774155	2.7854798	0.25854494	0.24835143	0.244456	0.3929754	0.52823596	0.24349876	2.84694197
0.66533647	0.42790059	0.95432149	1.61302833	1.05707316	1.20377602	1.6323167	1.88982937	0.33803461	0.44444158
1.1882065	0.37172028	0.3646435	1.71983754	0.38321352	0.21650633	0.02702777	1.48831087	0.19104221	0.23205231
2.16328456	1.52201988	3.60445193	2.174682	1.74606166	2.68466397	0.91695919	2.78665733	1.88574893	1.23620018
0.4275926	1.10687018	0.63729928	0.59373163	1.33094309	0.85819955	0.17011558	0.48686236	0.49675412	0.69810923
3.3212885	4.4517053	1.41244965	3.44068926	4.74294072	3.81170196	2.19408616	2.37009874	3.2612431	3.90018824
0.29657882	0.06479555	0.01935581	0.13755592	0.10280851	0.04142613	0.0974727	0.15912848	0.01236325	0.0335397
1.1553125	0.00415845	0.00837209	0.2764615	0.00821784	0	0.00489747	0.00766805	0.00933241	0.00255645
0.29882952	0.09552563	0.14853544	0.10167464	0.27843679	0.31083607	0.12718537	0.17169607	0.33780483	0.1791133
2.75302541	2.28248573	1.76481749	2.04852904	2.04916488	2.1691065	2.80973053	2.13117923	1.99788066	1.9220196
2.60380975	5.33096451	1.80082214	2.66119083	1.75927586	1.39047753	1.23957493	3.85728039	1.67132908	1.04450573
0.95910792	0.85769605	0.76481062	0.90714893	1.3065834	1.28771322	0.98859864	1.20537415	1.08886497	0.9580364
1.28380781	1.87212401	1.26122788	1.19489796	1.43366967	1.21862873	1.22373902	1.04028814	1.78784679	0.82060286
0.46577075	0.24531415	0.72875563	2.49778463	0.38245756	0.43475785	0.16410029	2.11355898	0.19370098	0.48599737
9.64430578	10.4453126	9.56473572	9.54755214	9.38270672	9.81609895	10.9366939	9.32022828	10.2739012	10.7366825
2.91967506	0.97217842	0.710889	1.29545687	0.8140341	0.50575781	0.2592278	0.7968992	0.36068791	0.22065815
1.68201665	1.88755688	2.6866174	1.72296682	2.34129631	2.38471528	1.544932	2.19381064	2.01028051	1.75479428
3.05900649	3.83528498	3.13880517	2.40421491	3.0091398	3.46923592	3.80838941	2.84048797	4.64351363	2.79419426
4.51732274	4.89216159	5.70329965	4.41332224	5.18206599	5.76065753	5.81592944	4.9192781	5.06743778	5.72298223

2.89731286	4.07479803	3.76796702	2.81963779	3.52756359	3.7128849	3.00514324	3.16126828	3.59003133	3.31463074
2.77574059	2.0635687	3.31980399	4.71713462	3.82529866	3.28294336	4.00318037	4.92848379	1.59747625	1.71055344
3.48964398	3.14563202	3.61687086	3.61326234	3.09080806	2.93927939	3.72128686	3.98285889	2.64622583	3.30739943
0.08745224	0.00637076	0.00642229	0.05610308	0.01258035	0.01487298	0.00750192	0.10237069	0.04243369	0.02334784
0.69483231	1.22769843	1.6241551	0.66534765	1.9877683	1.27330979	0.91469148	1.11282208	0.35375164	0.8405679
4.99393472	5.19190029	3.69478763	5.64775035	4.89442566	4.16988671	4.49507902	5.49449098	3.71845333	5.23613758
0.92466457	1.96544515	1.40992183	1.62902689	2.55346392	2.0073209	1.2826286	1.89329499	1.64169424	1.94015136
0.06285052	0	0.04069702	0	0	1.58186486	1.73997671	0	0.76711936	0.57361801
1.96019438	0.82521072	1.42695863	2.08667409	1.59957842	0.85519703	1.00565749	2.77000252	1.27043975	1.01310653
4.59177081	7.07711668	2.95348984	5.86223507	8.26880698	2.80004428	3.73494664	7.59067962	5.73396241	0.3014964
0.09019999	0.20690148	1.09412433	0.35201074	5.14661697	0.10059279	0.00870957	0.42399565	4.15311478	0.02708251
4.1568691	5.90913449	3.9221422	3.72070496	3.39712938	5.46193267	3.9866906	2.56840335	5.7172186	3.50409055
2.24728853	3.29042416	3.79464963	2.47317533	6.01871722	3.59637909	2.69464699	2.98992579	3.12190314	3.88932713
3.82611376	4.16264764	4.64366725	3.9703669	3.94279924	3.95110989	4.47157708	4.68373439	4.27659823	4.03171278
2.89684868	2.96769201	3.22454818	3.19309215	3.51087231	3.12750419	2.16341245	3.51759836	3.02543971	2.49633911
1.01940557	1.25072471	0.76162325	0.97008033	0.96161235	1.10005276	1.10214169	0.86646072	1.36389971	0.92753981
2.21100305	3.39465015	2.65264377	2.23489788	2.82677353	3.15150566	2.68714063	2.25238233	3.12953771	2.31430375
3.2975321	1.16913124	1.64017403	4.76873312	4.64049111	3.44064762	1.74045477	1.87964561	3.91913323	1.42094872
3.69037801	3.63067403	4.43774935	4.44290092	4.45651743	4.3694774	4.06293575	4.42683595	3.32452961	3.85595763
3.97482259	4.46223631	3.36387774	3.24758971	3.80118432	3.88803773	3.43536586	3.27279	4.05556034	3.4691496
1.94141896	1.31478764	0.01317292	0.10397649	0.04475904	0.44026322	1.74302693	0.01206686	0.02921438	0
1.94910626	1.82829778	2.18986863	2.00168133	2.08199223	2.02227878	2.13563169	2.19726599	1.92422579	1.79464971
3.87484043	3.91648772	4.84307639	4.2376216	4.37884406	4.10053664	3.94643069	4.52129593	3.46976797	4.40075656
1.98722792	1.0500199	1.77214302	1.65347613	1.4902294	1.42122489	1.24043953	1.84531012	1.17113885	1.1891759
3.00623917	4.10931211	4.03324835	3.54916035	4.5784104	4.90262158	3.90638765	3.34215314	4.81030467	2.54269431
3.4777439	3.59017832	3.80727331	4.38895566	4.23104353	4.28780424	4.3261357	4.00009766	3.71030747	3.28027055
3.50765112	2.77902598	3.32780579	3.35598157	4.00677292	4.09190039	3.47013323	2.308722	3.61473405	3.4636707
3.00898641	3.66966926	3.36104708	3.46075353	5.19052968	4.88003554	4.05872344	3.34312195	5.6697311	2.18163925
1.58842623	2.41132105	1.48456587	1.06419579	2.88006346	2.78679002	1.96001812	1.04792788	2.58082272	0.67793947
2.63306357	3.52197333	0.56564472	0.27399245	0.0237945	0	0.16194073	0	0.35286251	0
2.46713256	3.13836853	2.31672197	2.13678878	2.538283	2.73368383	2.47588117	2.25822542	3.56218771	2.83276148
3.16787684	3.20716784	1.28948246	2.37422862	2.80026796	2.83554851	3.33364196	2.17356608	3.68946633	3.046278
2.23177509	2.52095489	2.64072149	2.2649962	2.30241759	3.03270364	2.43847427	2.90756519	2.25047242	3.19293566
2.08337849	1.9864587	2.29214321	1.65974556	3.20648125	3.09821345	1.69947059	1.90390328	2.12572172	1.65921092
3.04189549	2.99796376	2.33192136	2.46904326	2.38633131	2.03290909	2.64396542	2.74247623	2.71716472	2.47855549
0.00928589	0.00592546	0.08710363	0.11089611	0.00586326	0.02754106	0	0.08513529	0	0
1.62187962	2.01148042	1.48570873	0.90436232	1.40882479	1.94328807	1.9383715	1.27231964	1.57452175	1.76021952
2.08219857	3.61160806	3.35316835	2.34453492	2.3909766	2.80826352	1.99547139	2.1072016	2.35446383	2.45413889
3.52938896	4.14961737	1.56961824	2.22650397	2.73438979	2.89282646	3.14894809	2.33204374	3.87111336	1.91145875
0.61953202	1.09897638	0.43991786	0.93268665	1.06495417	0.32409077	0.05407043	0.65944706	0.84407263	0.28153119
3.42687137	0.39074322	4.44204355	2.31370192	4.51447512	4.30442418	4.7355981	4.23931837	1.02676778	1.22442179
0.26987335	0.04453687	0.16437223	0.11219207	0.07143825	0.0886835	0.01924684	0.39585613	0.05442804	0
1.60798904	2.10646123	1.21169173	1.58108047	1.78666239	2.40900675	1.54301535	1.95185474	2.74302023	1.21196808
0.9012577	0.89722782	2.79154766	1.5947639	0.70079532	1.63245927	0.58893183	1.1277199	0.31525728	1.09539165
0.07812199	0.01132555	0.84996005	0.10634812	0.02785563	0.05869102	0.01995269	0.28481593	0.06253176	0.22586912
1.10733048	1.41472437	1.75500001	1.25308248	3.07259834	1.81268092	1.25477712	1.51275831	1.32851823	1.26727492
4.157655	4.50422553	4.67830501	4.6371648	4.8404343	5.13979919	4.85213843	4.09119017	4.07759124	4.44803429
1.4299259	0.55257151	0.90713228	1.86146599	1.10733376	0.48568866	0.03642748	2.21002604	0.0854283	0.27109689
1.46335696	1.80129986	1.31682843	1.66857582	1.63411151	1.49298727	0.75370078	1.81922793	2.08841704	0.99569621
5.99670543	4.83397324	6.63490719	6.05557504	3.04947886	4.53843866	5.79803394	4.17399681	6.23493205	3.81122774
6.00085838	6.39679785	7.02855577	5.82145613	6.02627945	7.14272151	6.24144433	5.67581371	5.800718	6.3955414
1.23876473	3.57779961	0.93702644	0.93518318	2.08394385	0.87244597	0.21910619	1.57212819	2.98120257	0.90882323
1.08109946	0.68314519	3.30950378	1.9992426	1.95355011	2.56842574	0.66688483	2.29013214	1.14579906	1.38114023
1.1626776	0.87087657	0.90872367	0.99982787	1.36777383	1.00583913	0.79659099	0.93836234	1.17046077	0.88169819
4.56863368	4.08605958	5.50053277	5.52353536	4.29032878	4.1529818	3.47400272	5.22461635	3.23836102	3.40441388
1.66244678	2.08939505	1.66061218	1.22490427	1.32565726	1.96529338	1.44309952	1.71189186	2.24209899	1.25420803
3.04991942	3.492672	2.32212073	2.17823142	2.92695141	3.24532962	3.12863258	3.26794549	2.99397282	2.23600654
2.18680497	2.8259633	2.50658138	2.2390835	2.57962646	2.93835674	2.07042681	2.78155171	2.3328197	2.07389395
1.86179192	1.79806074	1.33703609	1.63065561	1.5705845	2.17174863	2.04905976	1.58177494	2.20913388	1.50867023

3.50729719	3.0433596	2.53505371	2.73746245	3.37097607	3.11255251	3.13266842	2.6927381	4.05831724	2.56182649
2.6496507	2.37508307	3.18487157	2.6535447	1.82931436	2.79891658	2.76316008	2.32566513	2.48281932	3.33266713
0.69854379	1.74481983	1.44855226	0.45650628	1.43573511	1.61032582	1.0713609	0.92721483	1.25365951	0.74530574
0.99819023	1.07436861	1.45061647	0.99281077	1.6300886	1.49656682	1.36709289	1.11107966	1.29700154	1.61832821
0.9688809	1.30265903	1.0103007	0.93901661	1.3759248	1.23159752	0.66060437	0.74750463	1.41279124	0.20655837
2.14858426	2.83875335	2.58052864	1.99897906	2.68618132	3.01037683	2.28523195	2.33754438	2.35908499	3.06396025
2.44437196	3.27400591	2.50125045	1.9042899	3.10869896	2.37770129	2.17251846	2.13855958	3.30787601	1.95978991
1.05867664	0.83301851	1.41404478	2.24013085	0.7969053	1.30032134	0.52131082	2.47407122	0.87680143	0.7972682
1.86374683	1.50157614	1.6411828	1.93618624	1.54836419	1.71177963	1.69800355	2.22284378	1.91033074	1.61698606
4.12672591	3.15406763	4.30617068	3.66558188	4.40749611	4.65370289	4.35336747	3.03750753	3.6648272	3.65378324
1.73707957	2.10755483	2.5525753	3.09257853	2.74931485	2.13352399	2.00284835	3.27593408	1.16088462	1.84495995
1.96660042	0.56926923	1.3310634	2.62820899	0.46375619	0.40174313	0.01398635	2.08640353	0.46585566	0.29507774
0.64846556	0.22117893	0.33202315	0.619804	0.12439847	0.12814675	0.05448226	0.45924775	0.123256	0.10758827
1.15146611	0.24266021	0.72499569	2.49328798	0.32475201	1.21721202	0.04380049	2.17460105	0.14137212	0.9624295
2.70823486	3.03232375	2.60869156	2.6952858	2.88338819	2.77479889	2.78735863	2.73363102	2.93632719	2.75839497
4.01606629	3.84869145	3.26766395	2.96532336	3.59764642	3.55060081	2.35983122	2.95748793	3.04573561	2.72025339
2.91456095	3.27145611	3.11016699	2.80978774	2.95240967	3.16024823	2.33059156	2.97884159	2.92399193	2.77724974
1.73560139	2.28539846	2.63150325	2.54562942	2.89945828	2.24754086	1.81198343	2.5127619	1.94655034	1.84573352
2.93724983	1.98461277	3.97243523	3.23273856	3.75473477	4.09341504	2.94806139	1.80492639	4.12601223	4.2434704
2.64304919	2.97566316	2.71165916	2.13035397	2.6313035	3.61644815	2.26168545	2.16771406	2.89932329	2.38229408
3.10319171	4.00964704	2.77158326	2.88873501	3.57890366	3.68904359	2.71843567	3.10812577	3.43429111	3.24516311
4.46918141	3.07508343	4.05401847	5.29979569	4.01558354	3.28305804	3.73366693	4.97225523	2.92658959	2.60133184
4.58934629	0.09995098	5.24264964	1.73074509	0.09893483	4.6732019	5.72986667	0.06590897	0.15554969	0.82166973
1.5663541	4.70692788	2.90190992	2.7802479	3.74437822	2.64369389	0.93450176	2.27627194	0.83069258	2.85915051
3.83629746	3.98933735	3.34258841	4.07512312	3.56807122	5.68141101	3.38326362	3.74041956	3.79406024	4.1434507
6.04736069	6.22203733	6.23933582	5.722025	6.25164434	6.14944116	6.67528985	5.71910387	5.98895187	6.03333837
0.91329555	0.58151893	2.01192738	1.32235173	0.90963696	0.52786247	0.19835293	2.92705127	0.21825847	0.41659023
0.83247848	0.45245835	1.5501361	0.61428348	0.50146694	0.39810166	0.15600359	0.72790063	0.43157839	0.06715557
1.56962271	1.86682576	1.89744826	1.97262765	1.82515297	2.14012495	1.1690459	2.07039495	1.58091072	1.91883297
3.76421385	4.73779963	3.94170428	3.72442863	4.44361811	4.60063957	3.40192589	3.44620141	4.35233242	4.26648946
2.84826405	3.37190987	3.79095	3.11933129	3.69896373	3.75955574	2.57490408	3.82964922	3.85790725	3.78555501
2.3281138	3.03708683	2.60233794	2.40796586	2.95818247	2.44038542	2.411305	2.59073111	2.63678664	2.51963379
2.14133298	1.17499219	1.10724953	1.68321944	1.54738099	1.15142356	0.92215639	1.99753887	1.59331251	0.51558073
4.71953279	3.88704326	3.32919833	4.1138144	4.27159588	3.94004764	3.39345844	4.32761676	4.45434041	2.18359778
3.44834177	2.77745591	2.63612637	3.43626739	3.34832952	2.84275192	1.59833075	4.07495888	2.58236608	1.45994216
4.60845275	3.15918182	2.75472518	3.86705535	3.45166642	2.95675031	2.64489229	4.30622769	4.30327464	2.0285309
2.1831192	1.94729496	1.8101767	2.44031114	2.3182669	1.96625375	1.02878203	3.11493059	1.85445289	1.07634305
0.9379357	1.04190479	0.88162389	0.81339063	0.97970363	0.82007936	0.66894609	0.53945921	0.90125119	0.81267131
2.78097348	3.92804714	2.3385667	1.85278912	2.64821456	3.22993534	2.99387246	2.11597458	3.95745676	2.25593101
0.9149408	2.0036254	1.35876059	0.80988662	1.34387603	1.3195356	1.37883576	1.2152254	1.73645115	0.77636034
1.16251644	2.60227282	1.37653993	0.59740469	1.37488356	1.8404908	1.08749864	0.65190627	2.24854636	0.45232411
0	0	0.47963997	0	0.02635212	0	0	0.04878642	0	0.03265504
4.4658195	4.84580448	6.1346564	4.90687865	4.52564727	4.91931104	5.05073175	5.02721821	5.10857179	5.46092572
0.20193844	0.16854617	0.25468313	0.09395928	1.06711213	0.10546235	0.10164082	0.40880924	0.21201267	0.09655869
3.97607354	4.04584877	4.63774934	4.21177266	4.03482325	5.31151255	4.14799505	4.34209884	4.07090862	4.13111089
1.74440586	1.42033151	1.87622488	1.88949186	2.07598024	1.90834263	1.2978118	2.03262512	1.45099351	1.22272112
3.28868808	2.9436573	4.14145416	4.2337329	3.22205152	3.45079556	3.69047747	4.51176313	3.3935758	3.65333359
4.54031831	3.04408	3.44719421	4.81787814	3.66058489	2.76416992	2.07144919	5.36867526	3.77217738	2.89974796
2.7184453	1.8436491	2.39517235	2.6471385	2.706818	2.00099608	1.7959855	3.71930133	2.03786901	2.2431651
4.45099727	0.70191126	5.13989089	3.82049322	0.74770572	4.47966934	4.29971423	0.19312945	3.04597796	6.26502364
3.6969595	2.82682298	3.49186497	4.08295779	4.40656544	4.66011779	6.15253712	4.88078003	3.56366853	4.99632318
0.15304536	0.19239836	0	0.11088514	0.02523456	0.02980956	0.02006454	0.64126054	0	0
0.7497378	0.79469955	2.06941326	2.03503096	0.98548974	1.1880932	0.55576831	2.31153625	1.24568894	1.14863722
1.04520399	0.62957925	1.15639394	1.83451663	1.14929636	0.82035822	0.66125696	1.64107979	0.35701149	3.64413343
1.48356544	1.41288546	2.16682849	0.78954673	1.39123761	2.00262133	1.87509692	0.89478151	1.46024204	2.75232504
0	0	0	0	0	0	0	0	0	0
1.34026854	1.67349562	1.56283614	0.83044107	1.62850524	1.57062626	1.33927614	1.0896482	1.8078214	1.80382809
1.13398715	1.51311915	1.01304826	0.93153264	0.99953715	1.47896514	0.56885621	0.60136136	1.68989352	0.78844258
0.07675355	0.27377132	2.44552227	0.85981461	0.92294807	0	0.43889887	0.06055356	2.08283942	1.0396029

0.07127161	0.11195931	1.02946633	0.33926257	0.22364099	0	0.14347971	0	1.59157161	0.22474841
3.83452777	4.22389473	4.01954556	3.5313913	3.9029566	4.04066926	4.06227876	4.32947192	4.18659057	4.21459608
4.37358165	4.96172659	4.45372336	4.44204281	4.72241459	4.77014919	4.23666404	4.28483881	4.31153646	4.86697956
2.52404043	3.6786654	2.01448204	1.26194447	1.54684168	2.65153612	2.2561853	1.34242299	0.09208523	3.08323858
1.53442874	2.03555709	2.01469756	1.63381476	1.83543201	1.57645903	0.62803864	1.33555535	2.05584614	0.74922294
2.12594348	2.99261404	2.89992891	2.1029476	2.45657025	2.96217082	2.15252479	2.34964108	2.12007957	3.99927595
0.07578999	0.02051911	0.06517201	0.09552141	0.0872786	3.72303827	0.15671479	0.16562401	0.1330555	0.30141292
0.21551629	0.2148389	0.53779042	0.75591485	0.67680072	0.14964484	0.08920838	0.90202035	0.05932449	0.12364252
3.56206996	4.21614276	4.39368545	3.02178713	4.18785564	4.70378764	3.19353969	3.34889093	4.17375531	3.37098298
3.59545726	3.77469494	3.81363452	4.24982368	4.38318726	3.95899519	3.6178873	4.59707469	3.49265952	3.81362641
2.24898677	1.35137276	2.2220696	2.76544016	1.71062033	1.60375167	0.84789654	3.08713238	1.27898678	1.45772065
1.4214417	1.04826841	2.4410287	2.77722398	3.40943976	1.51271035	1.12691833	3.21356791	0.82652706	1.45397062
1.04271529	0.66291442	1.74315309	2.07244476	1.4098749	1.22047265	1.02677925	2.21610835	0.64895627	1.18174554
2.22439497	1.74676193	2.2522402	2.80701701	2.18903789	1.944079	2.71114699	2.33106774	1.83092018	2.11052955
3.5686059	3.01627836	3.09869257	3.52080595	2.983346	3.43216166	3.45524507	3.469401	4.29292622	2.34112361
0.84260498	0.08986879	0.9016844	1.21694913	0.2102309	0.37960771	0.68322178	1.36858192	0.0810678	0.45821992
2.98745038	2.40627326	3.91733466	4.42887532	3.89995264	3.60997763	2.81065662	4.94798882	2.85211265	3.20066292
1.045706	1.61480967	1.71084088	0.79279785	1.65321261	1.92851597	1.37759122	0.93017527	1.71259964	0.90540906
3.78221407	3.41481378	4.39644093	4.28469342	5.17077591	4.00727395	4.1434885	4.35582796	3.35070082	3.59325771
5.35929626	6.03441237	5.66473294	5.47317672	5.39938349	6.39176839	5.60371498	5.13391756	5.42796764	5.33241088
3.57640047	3.58537568	2.68345498	2.52258217	2.87743119	3.4432847	3.78420568	2.83506049	3.80908354	3.56366578
0.01931784	0.03671515	0.15953948	0.63386119	0.01828126	0.14485202	0	0.14157114	0.01386707	2.3405134
3.03165966	2.0083351	1.95463918	2.58158444	2.02878484	1.96563786	2.20314376	2.2294101	2.45269448	3.14700494
2.48325611	2.48286121	3.06887697	2.29129037	2.14869561	2.64466576	3.2018637	2.37565503	3.12280125	2.43397912
2.84432378	3.38833233	3.32070657	2.99800043	3.12906854	3.7559468	3.31274872	2.89550593	3.51464786	3.05540092
4.62047988	4.34858722	3.9834689	4.1676578	3.51871429	4.39132257	4.73454708	4.17342587	5.0043087	3.97510126
3.05502953	2.46503358	3.97599896	2.31585948	3.04509976	3.25397726	2.49536647	2.80204707	2.3875523	3.03104286
3.02689878	3.25853732	3.03576708	3.09311261	3.22785639	2.91609133	2.94581207	3.05112228	2.98183464	2.98531808
2.68237717	1.431042	2.89806079	3.38811674	2.54945526	2.67474258	2.88070809	3.44229889	1.49328227	1.93025431
3.69709683	3.62989281	3.57690664	3.69292039	3.61702676	3.66748403	3.57983602	3.72982868	4.33144689	3.68706476
4.71824474	5.72457321	5.4665765	4.48412518	5.34178717	5.61413276	4.6689499	4.78146029	4.61492622	4.87313881
2.90179589	2.57384265	2.09017209	2.13905698	2.0128233	2.72533707	3.15566402	1.96404842	3.06614133	2.50552572
5.30586567	4.53930135	4.56204977	4.55424263	4.79690278	4.94891468	5.03046663	4.16731231	5.03429962	3.61509007
2.40910436	3.61623669	3.61894147	2.23110065	3.35509453	3.34568983	2.66253721	2.45496253	2.84605533	2.54402368
5.00538264	5.47130862	3.36996207	3.97641214	3.77919369	4.59625267	4.5514741	2.320133	5.09066719	3.83321188
2.06747317	1.98376684	1.73085306	1.45546266	1.96862196	1.85728892	1.83848015	1.63795889	1.85914211	1.38345808
2.74223222	3.53939315	3.04099599	2.75059447	3.67007526	3.31196258	2.19703046	2.80106866	3.08075914	3.39896956
2.51095827	3.48252061	3.37978668	2.14836654	2.79283602	3.39176848	2.72162596	2.52472471	2.82601874	2.13117097
4.36743592	4.39484596	4.5662633	3.41864738	4.34868243	3.35912696	3.22534977	3.5955601	4.11148416	3.8816408
2.54883873	3.23412145	2.74118579	1.78815136	3.14686699	3.7836434	3.13535504	1.99774796	3.50840927	2.15564419
3.71354795	3.34593776	3.30839873	4.05537991	4.11420729	3.34024825	4.49238682	3.29822642	3.3413627	3.62197738
1.82032427	0.88177713	1.35296134	2.6959082	1.69387261	2.42192089	0.96377664	1.54040828	0.986098	1.66952522
3.5320002	3.43379652	3.64685519	3.63150592	3.07174073	3.18039338	3.10467519	3.59961312	3.27922166	3.39561936
4.84300494	4.62761826	5.40272228	4.72900428	4.11485573	4.4492335	4.74337644	4.8415793	5.30614293	3.82602536
3.7374607	3.76113526	4.19937837	4.38519634	4.63793395	4.09019177	3.18311466	4.64494103	2.98174506	4.12162539
4.08671388	4.01579202	4.61924053	3.66235015	3.98508582	4.37672637	3.64264618	3.76172199	3.6279984	3.676137
2.34161031	0.93493773	1.22605543	2.30834055	0.84828028	1.2033829	1.97586316	2.3530207	3.61503155	1.504528
2.28582665	3.89876113	1.66056488	1.66566922	1.90215203	2.12588189	2.06598097	2.51882149	3.3511202	1.81113838
1.81081619	1.00868491	3.22017363	2.55155545	2.19229391	1.54418906	1.82233935	2.82969024	1.4017585	1.38480769
6.17050909	5.62679694	5.7328987	6.16833753	5.96622474	5.60191464	5.92539899	6.55499855	5.5733183	5.85293324
1.87399993	2.55128595	2.07994016	1.74704128	2.38614316	2.26158035	2.11075349	1.72169998	1.99105461	1.73087657
0.73262947	0.32869732	0.82947997	1.58164903	1.02476606	1.10073408	0.3038631	1.28720673	0.28287413	1.86765583
1.44077723	1.11974204	1.99640765	3.65475337	0.7843105	1.87903969	0.26322778	2.67310642	0.93179178	1.78187687
3.50646532	4.157377	3.45870799	3.70593253	3.95351946	3.99352805	3.6448168	4.0167392	4.47124522	3.34007829
6.14383263	7.19494261	5.58899054	6.33484369	6.70232552	6.00592659	4.9783428	6.18728131	6.58599107	6.73823725
0	0	0.02790548	0.03096977	0	0	0.00820815	0	0.0310824	0.0085612
6.02707181	6.24308005	6.15470162	6.0858187	6.13042402	5.861348	6.13868968	6.00067887	5.57382082	6.35329596
0.94066728	1.01658924	0.99210896	0.90697327	1.03572445	1.10042574	0.97262923	1.12936352	1.05163099	1.44699007
6.37015245	6.8068345	7.34463596	6.47248891	6.74966064	7.02348424	7.24924912	6.68040396	6.46982891	6.9584695

0.18136759	0.20819076	0.25303193	0.35409948	0.34347575	0.31655473	0.20272595	0.57262409	0.39408762	0.17474497
2.66376538	2.19288873	2.96999333	2.80323993	2.72415816	2.67724293	2.09875736	2.89889087	2.18886045	1.7772919
2.29751234	3.08542768	2.56663457	2.44947045	2.78751296	2.19267595	2.53485285	2.92726787	2.92927453	1.73102058
4.14633754	2.13219443	3.08229125	4.10839728	2.95066413	2.27009726	2.48498863	4.59161639	3.15256258	2.51234154
4.31654554	5.00359297	5.73141477	5.17995163	4.80031515	4.77486142	4.51373034	4.93863545	3.04433276	4.40748444
2.75443515	2.51219768	1.95196272	2.08663294	1.89240762	1.60022281	0.95678735	2.612405	1.94445502	1.09126716
0.36146955	0.24059671	0.12628078	0.82201007	0.44269879	0.45608677	0.23653153	0.56038125	0.49366011	0.15251491
6.60440351	6.18619116	6.28863937	6.13851033	6.18885849	6.66565817	7.40618461	5.46547879	7.03061432	6.25850241
3.19435239	0.63481615	1.332481	1.99855194	0.94994508	0.90541747	0.61729999	1.83814024	0.95133461	0.65206244
1.7513048	1.27121753	0.90748109	1.47551712	1.01140606	1.00561494	1.52104151	1.09577381	1.64646281	0.24414405
2.67538905	2.23016335	2.96284607	2.88756244	3.17905479	2.96051646	2.33137809	3.12461706	2.78223231	2.49351833
2.82223319	3.71751093	3.2266301	2.80844412	3.56577101	3.2499783	3.13626324	3.00693871	3.49412699	2.87515562
4.25471588	5.12938446	3.40115013	3.11342954	4.24337769	4.29832893	4.05555864	3.30078648	4.70243342	3.89115193
3.61685981	4.50826729	3.91313717	3.66964262	3.63096347	4.35717856	2.96078632	4.1482432	3.81412531	4.49431929
3.87182068	4.47304068	3.66445877	4.24302973	5.14025381	4.9875756	3.76169048	3.97481146	4.14571124	4.44713408
3.81793816	4.06609238	3.81038617	3.27797852	3.65334397	4.56903094	3.39300662	3.72837177	4.19256504	4.8659308
2.86599572	4.10965364	3.02105786	2.51982849	2.84653112	3.7318111	4.03112262	2.85986944	4.34496116	2.78143335
2.35439512	2.04645292	3.10970968	2.56806885	3.28649999	2.85760857	2.51285761	2.43719041	2.00072441	2.25470947
3.70209717	3.39076989	3.39143121	4.17466124	3.58971013	3.72303452	3.40333829	3.45257229	3.71705185	3.89004818
0.43969903	0.32172026	0.27029601	0.06380655	0.45105838	0.29916604	0.01280885	0.27458493	0.77806749	0.20018112
4.31177853	4.5882253	4.92690492	5.04049596	5.27868303	5.12089653	4.23832059	5.10749174	4.52865086	4.8062648
1.94267188	2.124212	2.62833003	2.07952559	2.62337255	2.57861182	1.98376589	2.39484231	2.30917791	2.26271716
2.96580816	3.58693293	3.91813199	3.40879282	3.9383341	3.52091148	3.51490798	3.85305773	3.79507273	3.55306277
2.82294334	3.17672441	3.29087871	3.55213529	3.85923126	3.2471606	3.31770975	3.8574181	3.11684522	3.13409917
3.12248348	3.48199892	2.7913134	2.43558402	3.18267992	3.00186967	3.24613552	2.8648246	3.62752642	2.5947611
0.00858855	0	0.00552434	0	0.01082462	0	0	0.02013187	0	0
4.58091482	4.98419584	4.7614028	4.27721121	4.58296369	4.61954207	4.10986214	4.23935982	5.23572924	4.24500219
0.72365915	0.03532234	2.57980807	0.73926559	0.12422905	1.77320595	1.14039937	0.30171933	0.22301799	3.24410149
1.42746533	0.24820965	0.92028053	0.40557003	1.25479807	1.38160634	0.61425251	0.95157267	0.82261173	0.701781
3.0739003	3.62132289	3.49995346	2.87001801	3.95534033	3.2370592	2.80994013	3.21273627	3.26888829	3.75292982
3.74977903	4.60845286	4.92586061	3.97883684	3.76816647	4.68838847	4.00759783	4.05416256	3.81898286	5.87188669
6.03851862	5.78857142	5.76992202	5.53359297	6.89513571	5.8178194	5.23883195	6.38322097	4.91964003	4.77738403
5.10158344	5.87810105	5.42552167	5.57347226	5.06878957	5.11568633	5.13900501	5.1955129	5.46390557	5.24914011
3.62758398	4.18216872	4.72574419	3.40078149	5.18433394	3.8397263	3.93298502	3.84843233	3.87171515	3.62980806
2.41394156	0.0499456	1.8824612	2.22179847	2.59234664	3.78784349	3.93569914	2.05079312	0.1448227	3.81037554
1.78443209	2.04844223	2.27990242	1.85198962	2.67106231	2.18610506	1.16488783	2.3768238	1.65310975	1.56695673
2.63120711	3.08270317	3.82182187	2.56492384	3.50913016	3.15280525	2.44367936	2.94179776	2.68572319	2.85622584
3.03838766	3.26992468	2.82216329	3.13608595	3.09177713	3.09398966	2.77076955	3.36482825	2.89395323	3.31130688
4.53737311	4.80374523	4.44125218	4.77190904	4.92013022	4.60540616	4.8798562	4.70299447	4.38871882	4.90116642
2.24257934	3.09110655	2.47839685	2.46546083	2.69992185	2.68592928	2.50981469	2.48180093	2.91473474	2.5088146
1.91681005	2.0988266	2.01635901	2.09602464	2.14286799	1.96953356	1.65245721	2.05471118	1.56445096	2.03067733
5.00714177	5.26479115	3.93349054	3.84085542	3.54297201	4.45406531	5.43989251	4.16803063	4.70198583	4.77070961
4.77823789	5.78674121	4.33801798	4.637895	4.96053911	5.32279605	5.2219209	5.05163539	5.66406961	4.81241047
3.02405812	4.45900944	1.24202138	6.44075121	0.22996588	0.23094049	0.13871772	0.01744763	4.12853598	0.01163656
0.0663744	0	0.02165881	0.06324477	0.02126156	0	0	0.03942095	0	0.02635801
5.52549342	0.34591039	7.26018089	0.74552345	0.42053584	4.28365402	7.31509144	0.40034514	1.35494057	5.17096419
5.77557577	6.55315747	5.74024957	5.9040127	6.04869747	6.08908164	5.0589926	4.9760915	5.40176883	5.57078456
1.15301954	1.78020761	1.33517008	1.03367197	1.46800012	1.41025516	0.90271921	1.59853288	1.29729667	2.17762498
2.35857667	3.06633995	2.28353198	2.55537849	2.46348989	2.53780473	2.40788027	2.50329926	2.62881055	2.05956668
0.87640271	0.12944077	0.22983511	0.38779594	0.26266971	0.90798505	0.16915867	0.92030298	0.26994865	0.23023446
1.4315503	1.68979884	2.4759242	1.59640786	2.59820562	2.46101238	1.37465018	1.67327635	1.78755876	1.62717995
1.93673405	1.90319699	2.15756356	1.69969665	2.33003453	2.26874182	1.79808682	2.14790902	2.02350328	1.99499084
1.11324808	1.4452205	1.26885722	1.05515569	1.58389896	1.6721758	1.10140875	1.24930747	1.25821191	0.99881646
1.03352217	1.22958123	1.26482812	1.08513963	1.25970743	1.05333261	0.6924671	1.36382289	0.98001665	1.03957831
2.60609783	2.38914373	2.15996084	2.57740594	2.71721472	2.39897803	2.524282	2.41947011	2.60117114	2.19776046
3.05007758	3.56237604	2.39120316	2.74255283	3.659906	3.16369714	3.27343792	3.73147467	3.61113144	2.82614811
2.69431691	3.08748195	3.06376778	2.69007376	3.13796795	2.51858456	2.62465257	3.09603846	2.8370936	2.63412683
3.08119618	2.41565819	2.67689696	3.54839215	1.52004404	1.11116347	0.51797523	3.05153859	1.69064179	1.75474499
3.32133792	3.58986436	3.75172981	2.83346848	3.27765036	3.20072328	2.81719788	2.95776995	3.3197086	3.04854328

3.65115966	4.38640345	3.79181527	3.27940268	3.53636729	3.55025769	3.29069286	3.13517878	3.76510078	3.52785407
2.48192349	2.50736341	4.18528089	4.66350725	3.73128959	2.86992173	3.73478467	4.80208069	2.64627482	3.86854659
0.22586613	0.47174458	0.40400711	0.10842946	0.15099213	0.49359443	0.15163985	0.1413435	0.25803931	0.12422339
2.69963662	6.36829048	2.69952171	4.01156174	2.75170297	4.40609366	0.53814237	4.01201046	5.51311516	1.21292715
2.53014469	4.82965273	4.12478142	3.40137427	3.09893878	2.90960469	1.81239457	2.76745445	3.86289051	1.91683739
1.98217345	1.38554601	2.89585338	2.67290615	2.77015522	2.69989852	2.42113797	3.42484023	1.77801037	2.40890096
2.34032552	2.34150241	2.71022328	2.05265442	2.51297185	2.47929279	1.52621575	2.18547673	1.83899734	3.68886538
3.64864579	0.89708341	0.46191139	2.21985268	0.99670056	3.07830092	3.41279719	5.07245113	0.39571401	0.63640747
3.55745915	3.00449964	3.14372382	2.79064703	2.27656444	2.82864505	2.75475364	3.31826511	3.14365242	2.46093307
2.18147927	2.26417901	1.89263636	2.14582663	2.1529625	1.45100531	2.42591736	2.2269582	2.02689716	2.38516165
5.54046615	5.82670721	5.77749826	5.19961928	5.67717381	6.41983886	6.18508615	5.30453549	5.76698686	4.99422575
3.58262357	3.82090657	4.04036909	3.7849313	3.49298251	4.01186925	3.74642077	3.83250953	3.68303082	4.21689717
0.86581324	0.44818373	0.79258968	1.98639634	0.2962192	0.8856083	0.63577964	1.87870375	0.35025769	0.46296488
3.23389552	3.84579507	3.31196394	3.02810327	3.55802273	3.72723624	3.29347658	3.26681099	3.62104192	3.30617139
2.65252734	3.05317144	2.39924783	2.65372482	2.8868407	2.4985877	2.51872518	2.49174112	2.86554191	2.57957402
2.30792719	3.24665786	2.4643052	2.33053923	2.71351141	2.51550484	2.53650797	2.37436281	3.0874829	2.16565532
5.7074052	4.60038392	5.65571192	5.20118967	5.58301058	5.45727372	5.08126079	5.00437834	5.09108576	3.54197724
2.82558262	3.96117075	4.07049528	3.40881562	4.0809788	3.43792973	2.96604446	4.00640439	3.9502767	2.94968936
4.76988035	4.70667495	4.84852695	5.0958811	4.45372312	3.85042454	4.19052404	4.19611142	4.27703468	4.67891651
2.69956845	1.86989919	1.47681202	1.45946318	1.71157422	1.68026479	0.63571843	2.05468421	1.51061589	1.02447023
0.19278456	0.3516855	0.52375929	0.99462842	0.52096358	0.4277622	0.17157623	0.85322224	0.24706531	0.44601777
3.28512062	3.44547857	3.41694539	3.75300036	3.88442993	3.00981842	3.36689144	3.49905728	3.71522815	2.76004158
3.14700937	2.87637749	3.6674057	3.53312864	4.56186074	3.65057323	3.39140859	3.60612756	3.12775725	2.38118073
0.97107524	0.36877968	0.55811833	1.3756297	0.72384196	0.59832039	0.2496989	2.13166717	0.47414703	0.47408165
3.06918085	1.90240436	3.13058371	3.64574215	2.61391716	2.78961237	1.48224972	4.02194275	1.54471717	4.66893372
1.13313108	1.38431804	1.43925913	1.712285	1.28927001	1.24051586	0.9534178	1.44088772	0.94012739	1.50577609
1.93964112	2.13155501	2.24784982	1.51859795	1.62914364	2.03725853	1.91569598	1.50034467	1.89044195	2.61783839
1.19493109	0.42228695	0.62934152	1.26817291	0.61234633	0.51730972	0.36303897	1.46667124	0.36876041	0.59682375
0.3164346	0.1201417	0.4942071	0.60696969	0.36657828	0.28721399	0.28883348	1.07468689	0.15138632	0.21863031
1.18738006	1.09363868	2.07819066	1.96121741	2.21393328	1.99546014	1.80879067	2.4618954	1.08995774	0.98877569
2.54796472	0.78832348	0.42039039	0.61582716	0.63542814	0.59991888	0.47204144	0.7070295	1.32704134	0.3887622
1.10310568	1.29634387	1.91031394	1.23003158	1.7333253	1.7850681	0.93773476	1.3326014	1.13970779	1.72661143
4.86685142	3.12706283	3.34816429	3.38300114	3.33284393	3.21787866	2.4500795	3.00983884	2.51349679	1.68167549
0.47174623	0.58674888	0.79634944	1.7055244	0.27391695	0.64793802	0.29652412	1.56893805	0.69031392	0.65390806
0	0.01635475	0.03278689	0	0.03218781	0	0	0	0	0
0.2278024	0.18431558	2.79044768	0.87209078	1.67593554	0.26809609	0.04559329	0.6072228	0.10236558	0.12932172
0.94128338	0.38850139	0.75959384	1.2762825	1.80320256	0.69619007	0.86764059	1.48188235	0.20910486	0.19818024
2.51098793	2.44037841	2.82554525	2.19694559	3.264825	1.81517103	1.68564066	2.26877528	1.65375233	1.04454325
0.26700507	0.10693713	0.73233739	0.31954169	0.09777238	0.23897169	0.23450349	0.42642861	0.14958357	0.68444603
2.04528427	1.8530524	2.70579624	2.7819143	2.03462063	1.98889351	1.77955729	3.61773212	2.29558341	1.86695667
0	0.06906964	0.18390347	0.19719364	0.08495566	0.02056007	0	0.15467767	0.11462026	0.04282788
0.43257844	0.16285553	0.22565578	0.27189546	0.18170384	0.10517325	0.1108961	0.10771006	0.15856947	0.05615114
0.29281898	0.28112619	0.86222991	0.46974756	0.59668944	0.50301113	0.31368966	0.62093862	0.48340396	0.40294892
1.83159321	0.84513957	0.95582249	0.90132752	1.47379461	0.96834229	0.92335474	0.64240495	0.80685304	0.31366467
2.59541925	1.38931657	3.0534445	3.13491707	2.65414304	2.91929011	3.19212099	3.68593609	2.09289378	2.55345461
0.52143304	0.46300264	0.61736034	0.51927501	0.63148604	0.62199422	0.24578517	0.52853179	0.73675238	0.24610454
0.39786388	0.74200036	0.71811862	0.56340536	1.18440412	0.96186776	1.35256232	0.40649234	0.5116573	0.61308622
3.42024097	4.49055027	6.80027765	5.18040655	5.19931438	5.5055302	5.73730338	4.4848183	4.88698708	6.74655863
2.22327761	3.30790218	2.64435027	4.34049742	5.46399219	2.49093001	0.91727892	4.32305076	5.18622884	1.47891004
0.80361887	0.773615	3.71424381	1.16307632	1.04222063	1.49802365	1.93958567	1.91085198	0.85747035	1.74462004
0.90457852	0.9011614	1.6965142	2.05235658	2.71068972	3.0351188	2.67390228	1.05391378	3.17238035	2.90301696
1.50530709	1.0245323	1.69250651	1.5883268	2.7205083	2.38210017	0.90475139	1.61513562	1.18028831	0.5799631
3.96192473	4.54949077	4.03275247	4.25847541	4.01842789	4.11280842	3.98017156	4.02360829	4.18309433	3.95770862
1.94570892	1.75857505	1.64154835	1.61518868	1.72004752	2.18712683	2.05622407	1.96123176	1.52290971	2.0333691
1.46845209	0.98220989	2.77470031	2.27230999	1.63029879	1.61357166	1.83259229	3.26330997	1.05125442	1.11697293
1.67295073	2.30342309	3.32799737	1.61634308	2.68920053	2.77693137	1.93321962	1.22521109	1.83132178	3.19283104
6.02143025	3.93370187	3.59744808	4.27991453	4.18243027	3.83793671	4.01684855	3.82273313	4.25428649	2.39301534
2.28922045	1.08272015	0.41336019	0.35010165	0.59686445	1.15677354	2.70466948	1.44128617	1.52184824	1.05415116
1.45714345	2.87904727	2.48400972	2.05005095	2.86710717	5.17327746	2.0351545	2.37887127	3.13854727	2.19878172

7.97291904	7.48299674	6.94919296	7.21623163	7.42919501	7.37566612	8.08675413	7.22034376	7.59554168	7.18324196
8.38009945	7.32863991	7.21375263	7.50781012	6.86639978	7.66074466	6.73963006	6.78011263	9.8022662	9.57550463
5.83628405	3.59083596	4.79549359	6.07013223	4.96956793	4.30748223	4.04002706	7.88823721	3.67531955	3.03071753
7.51134185	6.14795384	6.91126879	7.11673976	6.65042847	6.49301093	7.31117048	7.43671786	7.40156668	7.33846084
3.39807021	1.44340393	3.18511252	3.14682217	2.95882332	2.0560096	2.77503198	3.76523686	2.48367821	2.49399934
2.65092073	2.14435008	3.49657684	3.85979031	3.25566425	2.40591639	2.86725321	4.01881756	2.07639782	2.46397577
3.64987746	5.07854384	4.50507331	4.20019755	4.54823182	4.56643641	3.12200658	4.66878704	4.23425179	3.74501636
3.70942591	2.43850704	2.88538924	2.86789436	2.76858462	3.34419986	3.00164403	3.06788045	2.45132383	4.1940844
1.52232466	0.3695339	0.43356398	0.61553294	0.46265193	0.42954065	0.22891415	0.71546711	0.48102403	1.81195822
1.48124287	0.86474623	0.7797929	0.7140308	0.90300235	0.89356353	0.39481731	0.78248739	1.54762762	0.13484784
3.80939971	2.19494476	3.02913207	2.92419619	2.36389578	2.11757511	1.42682339	3.65179022	1.77269741	1.51009759
1.90391262	2.0553038	3.06310998	2.51789253	3.36107955	3.1230596	1.62123001	3.3403368	1.24804638	3.07143319
2.77307214	2.92826723	4.45688438	2.7221802	3.35139283	6.19091819	3.86131579	2.54098184	2.89620544	3.39748279
0.70171136	0.17291617	0.64478888	1.47459863	0.38800444	0.20680404	0.1380616	1.10402685	0.1463268	0.30726848
3.84998945	3.57084566	6.20704966	6.54324638	3.88156035	4.48042098	4.64593055	5.82669909	2.78978036	4.9854274
0.49357192	1.0415613	1.37270778	0.62861468	1.06611951	1.42067034	0.84029312	0.40618047	1.02651082	0.33546313
3.63085483	3.99702161	2.69484206	3.15075389	3.0790274	3.45340238	3.52749983	3.00920602	3.70757547	3.09156527
0.52071088	0.12743128	0.10541864	1.06176167	0.04267715	0.15612697	0.00894057	0.83138609	0.0338321	0.10810883
0.68651358	0.49265283	1.28539917	2.29080715	0.45539703	0.81041575	0.10882252	1.67823694	0.30247748	1.17751872
0.1723912	0.11844666	0.13593657	0.19998548	0.11520105	0.11121757	0.02879476	0.57920132	0.19774164	0.14173902
3.28212113	0.75824495	4.78042249	2.9966358	4.62746067	4.54798104	3.71019496	3.49754239	0.9706966	3.98843074
6.83451502	6.44290987	6.98818474	6.80444698	6.87516154	7.5221667	6.74801732	6.07977242	6.05262237	7.98328395
2.38282442	3.07767841	3.0422108	2.45832033	2.49964882	3.22254255	2.11072779	2.45937499	2.80852385	3.07473122
3.06825168	2.75387021	2.46118028	3.0524482	3.00457402	2.31029307	1.04545943	3.09647682	3.08116168	1.66798283
4.78701646	3.98965022	4.04280363	3.85865345	3.69790714	4.30772439	3.64791397	3.72525698	4.31486424	3.81548903
0.06652066	0.05062803	0.20601308	0.14988108	0.06813421	0.1158684	0.07470512	0.45193036	0.04206042	0.09354405
0.02010466	0	0.01295016	0.00640899	0.00848706	0.01003631	0.01009854	0.03916886	0.01921208	0.0105326
0	0.15525573	0.2877679	0.00887388	0.21850293	0.10430115	0.08189203	0.04868984	0.40104483	1.22358739
7.55706465	7.01114365	7.70409139	7.49187575	7.80425652	7.03328696	7.35224107	7.40217867	6.72820413	7.16665581
3.00078816	3.4406937	2.45281313	2.5612901	2.87798634	3.00044296	3.47159841	2.7893967	3.66624099	2.95192576
1.6553969	1.9218624	3.17868577	1.79689241	2.1124601	2.47383502	2.10190002	2.02920031	1.70180226	1.73687946
3.5843489	4.07869406	3.64966608	3.17908226	4.41017316	3.79911597	3.75683791	3.12407864	3.90156684	3.16871647
3.29367923	3.31034397	2.2795246	2.39951192	2.71031865	3.60223454	3.47926898	1.9653869	2.82752824	4.030844
3.15882925	3.75697121	2.84793134	3.18889857	3.75489179	3.69366969	3.21380988	3.71120997	3.69694053	2.96777804
1.31501185	1.40968965	1.18259989	0.97281267	1.88905976	1.74751364	0.69264777	1.27335459	1.10396581	1.79995507
3.22385858	2.65158978	2.96806184	2.14091422	1.70178447	5.93692329	3.6580301	2.14804511	3.72176971	2.2412125
3.90160474	4.12251321	4.11084972	3.18440561	3.72529961	4.17204652	3.73013523	2.98322666	4.7718558	3.43904681
2.75442343	3.91101911	3.324648	2.71475492	3.62383084	3.41270671	3.02160446	3.47146836	2.79002578	3.378743
0.02298314	0.01103229	0.01112139	0.04878007	0.02534492	0.02144873	0.03437663	0.17922198	0.08852142	0.00904415
4.72172335	5.01262966	5.05932189	4.64267497	4.96083598	5.32584639	4.98534569	4.51380993	4.79540691	5.04595458
2.80071048	3.33227322	3.6690684	2.84154913	3.63705008	3.36734639	2.91037734	3.14466445	2.96044743	4.20489132
3.35200656	2.59272701	3.46894119	2.50920719	2.53899098	3.79145712	4.33987931	2.44302947	2.82652881	2.53391468
5.7284785	5.00622554	6.10295852	6.50383622	5.33971644	5.06939909	5.11738322	6.86830608	3.91727116	4.79012724
1.84467429	0.71603835	3.3893158	1.49927692	1.22802797	1.04578676	0.7616118	2.05796792	0.87563008	2.46211432
5.48652904	5.43696503	4.08161237	4.41034033	4.27413641	4.4553251	4.79477983	4.19857103	5.7687278	4.35151063
5.5071616	6.68014892	4.93388598	4.46535434	4.98716257	5.33098529	5.37396225	4.26933442	5.96306023	5.99726434
2.55777233	4.24087415	0.87292005	4.20909735	0.87102158	2.59135113	0.18749407	1.7312072	8.0688666	0.27394979
3.19223317	3.05809353	2.12746209	3.06407288	2.81017324	2.83132343	1.66328511	2.54745631	4.09745749	2.30273119
1.07978348	2.41616868	1.83928373	1.13287161	1.79662868	1.46795108	1.27072962	1.01230809	1.73533153	0.91878279
5.45940849	5.83765497	5.23610469	5.22712698	4.64167253	6.11149077	6.48443706	5.3593091	5.93824564	6.91534468
2.74742905	4.35070648	0.11142754	0.75692347	0.11645426	3.95740981	3.67002142	0.0824588	0.06729872	0.18526916
1.84778469	2.92638639	1.46215134	4.12526379	2.40029515	4.17100726	2.54590309	2.3434147	0.67787201	1.66316283
2.32666339	0.37546474	1.14394911	1.78718696	0.54256593	0.41686544	0.69307453	1.68144493	0.38870259	0.23868215
5.67834605	5.75658621	5.53921954	4.74971901	4.81447909	5.59150551	5.69103434	5.08252244	5.58218143	5.11740953
1.21182353	2.37708682	1.66633673	1.06505056	1.36316675	2.11092964	2.10936357	1.31720752	2.10361303	1.82215825
8.6210008	7.16343091	6.85612464	9.02374174	7.86507129	8.3542289	9.15790524	7.88562143	5.33238433	8.47695479
0	0.0171567	0	0.53268895	0.13053315	0.03985603	0.35823331	0.17953506	0.14741554	0.04180567
1.69343376	2.16117678	1.13283542	1.22903062	1.11203984	1.57280725	1.84082192	2.08180362	2.34151115	1.19639146
2.50675173	2.96374676	2.70434824	2.57675683	3.17729713	3.28480015	2.37746772	2.78077278	2.67223719	2.34664613

3.90792382	4.03925097	3.44132702	3.46392273	3.9576698	3.6334324	4.58651032	3.53308234	4.21259531	3.3400972
3.88185027	4.43727821	3.57978953	3.36981416	3.81617487	3.44109251	3.56479731	2.90417177	3.97559662	3.37648405
3.93762096	4.04284935	3.15670937	3.39903742	3.73867828	3.60229264	3.47879504	2.92514774	4.63128577	3.16024684
4.6260017	5.19640224	5.06598858	4.76817681	4.67420425	4.55932722	4.93033554	4.87996648	5.04975186	4.78334029
2.91019761	3.11175355	3.64771085	2.48862977	2.87692185	3.4305903	3.21615642	3.22382921	3.09575902	4.03078067
2.09299047	3.12400585	2.34020723	1.91353045	3.05281492	2.65636901	2.03167406	2.4053996	2.64507893	2.32646136
0.24284577	0.52269287	0.11182629	0.3115861	0.18125774	0.24097828	0.2357166	0.21635158	0.45401737	0.21720223
0.39099265	0.51685705	0.27673367	0.23606525	0.48362908	0.62065813	0.23097647	0.48306167	0.84011126	0.2737948
1.8720023	1.63990831	1.31933505	1.615211	1.25928999	1.49582931	2.00131086	1.56172892	2.42262084	1.58095092
2.19183342	3.60073506	3.50985103	2.53260327	4.22925837	3.58757486	2.06537898	2.91275607	2.71481735	3.28888006
3.00284678	3.50472642	3.55672125	3.57689601	3.59043459	3.49429061	2.81689635	3.63567108	3.10515334	2.83563744
3.31879892	4.10329389	3.65667371	3.16989632	4.04791763	3.99889607	3.72204396	3.3280437	3.79076367	3.27582685
1.82934965	2.92652852	2.90017415	1.68222057	2.46068645	2.2195089	2.11237883	2.0617482	2.44927223	2.21784891
1.7879029	2.92195414	2.71433277	1.99766259	2.82502302	2.95491572	1.90715035	2.49719782	3.20855099	1.85060356
3.68245739	4.40305262	4.26791232	3.69740723	4.35160688	4.41679916	3.8571002	3.83175127	4.87253864	4.026051
3.4638078	3.89097616	3.33765198	3.4078028	3.60316287	3.66868402	3.45871744	3.7526495	3.32842731	3.31416861
3.12153726	3.67366241	4.87433584	3.41038143	4.03555964	4.40823106	2.96721527	3.3365351	3.33059178	3.60564848
1.71625289	2.32104833	2.69803672	1.67104486	1.84943852	2.60221561	1.92657312	2.62979282	2.52384107	1.60400921
2.61050765	3.27172868	3.59803247	2.50081208	3.43377201	4.00924726	2.70569627	2.84155074	3.03870235	3.60711439
4.11296498	4.52170619	3.61324897	3.69700685	3.52524535	3.5857076	4.04544066	3.95094546	3.68118077	3.91981657
1.49876201	1.9397631	1.46094652	1.15240812	1.87118534	1.21013555	1.42384444	1.49929146	1.93367622	1.17013542
0.22555706	0.22377411	0.29813473	0.28067635	0.22159204	0.40349466	0.09840665	0.23512572	0.2816137	0.16068401
0.45185952	0.03343027	0.55841875	0.18907283	0	2.10212473	2.97752734	0	0.84041809	2.3559825
4.7692254	4.93279033	4.8522837	4.98197032	4.77533819	5.27815314	4.96693692	4.95413415	4.91936621	5.0352245
[truncated: 731,551 more chars]
